# Inhibition of miR-29 has a significant lipid-lowering benefit through suppression of lipogenic programs in liver

**DOI:** 10.1038/srep12911

**Published:** 2015-08-06

**Authors:** C. Lisa Kurtz, Emily E. Fannin, Cynthia L. Toth, Daniel S. Pearson, Kasey C. Vickers, Praveen Sethupathy

**Affiliations:** 1 Department of Genetics, School of Medicine, University of North Carolina at Chapel Hill, Chapel Hill, NC, USA; 2 Department of Medicine, Division of Cardiovascular Medicine, Vanderbilt University, Nashville, TN, USA; 3 Children’s Hospital Boston, Harvard Medical School, Boston, MA, USA; 4 Lineberger Comprehensive Cancer Center, School of Medicine, University of North Carolina at Chapel Hill, NC, USA

## Abstract

MicroRNAs (miRNAs) are important regulators and potential therapeutic targets of metabolic disease. In this study we show by *in vivo* administration of locked nucleic acid (LNA) inhibitors that suppression of endogenous miR-29 lowers plasma cholesterol levels by ~40%, commensurate with the effect of statins, and reduces fatty acid content in the liver by ~20%. Whole transcriptome sequencing of the liver reveals 883 genes dysregulated (612 down, 271 up) by inhibition of miR-29. The set of 612 down-regulated genes are most significantly over-represented in lipid synthesis pathways. Among the up-regulated genes are the anti-lipogenic deacetylase sirtuin 1 (Sirt1) and the anti-lipogenic transcription factor aryl hydrocarbon receptor (Ahr), the latter of which we demonstrate is a direct target of miR-29. *In vitro* radiolabeled acetate incorporation assays confirm that pharmacologic inhibition of miR-29 significantly reduces *de novo* cholesterol and fatty acid synthesis. Our findings indicate that miR-29 controls hepatic lipogenic programs, likely in part through regulation of Ahr and Sirt1, and therefore may represent a candidate therapeutic target for metabolic disorders such as dyslipidemia.

The liver is the primary site of lipid synthesis and metabolism^1^,^2^. Proper hepatic control of lipid homeostasis is governed in large part by complex gene regulatory networks. In the last ten years, microRNAs (miRNAs) have emerged as important components of these networks^3^,^4^,^5^. The dysregulation of miRNA activity has been linked to various metabolic disorders of the liver such as hyperlipidemia^6^,^7^, steatosis^8^,^9^, insulin resistance^10^,^11^, and obesity^12^,^13^. The first miRNA that was shown to have an important role in lipid biology is miR-122^14^, one of the most abundant miRNAs in the mammalian liver^15^. *In vivo* studies in mice demonstrated that miR-122 is involved in the regulation of lipid synthesis^14^, catabolism^14^, and secretion^16^, and also has essential anti-inflammatory and anti-tumorigenic functions in the liver^17^. More recently, miR-122 was shown to control plasma cholesterol levels in humans as well^18^.

Since the discovery of miR-122, a growing number of miRNAs have been implicated in the control of lipid homeostasis. For example, Cheung *et al.* found that several miRNAs, including miR-27b, miR-34a, and miR-30, are more significantly altered in non-alcoholic steatohepatitis than miR-122^19^. Subsequent studies in mice demonstrated that miR-27b is a regulatory hub in hepatic lipid metabolic networks^6^, miR-34a contributes to hepatic steatosis via repression of sirtuin 1 (*Sirt1*)^20^, and miR-30 controls lipid synthesis and lipoprotein secretion^21^. *In vivo* studies in both mice and non-human primates showed that miR-33a and miR-33b, encoded within the genes *SREBF2* and *SREBF1*, respectively, are critical regulators of cholesterol balance^22^,^23^,^24^. Most recently, miR-223 was identified as a master coordinator of cholesterol uptake, efflux, and synthesis in the liver^25^. These and other related findings indicate that hepatic miRNAs are central to the governance of lipid homeostasis and are candidate therapeutic targets in metabolic disease.

We recently demonstrated that hepatic miR-29 is aberrantly elevated in two different models of metabolic dysfunction: high-fat diet-induced obese mice and Zucker Diabetic Fatty (*fa/fa*) rats^26^. Notably, miR-29 levels were restored in *fa/fa* rats upon treatment with Pioglitazone, which improves both insulin sensitivity and lipid profiles. We also showed *in vitro* that miR-29 fine-tunes the levels of key lipid metabolic genes^26^. To follow-up on these findings, we sought to investigate in mice the effects of loss of miR-29 function on circulating lipids using *in vivo* locked nucleic acid (LNA) technology.

## Results

### LNA administration *in vivo* strongly inhibits the miR-29 family in numerous tissues

**Figure 1:**
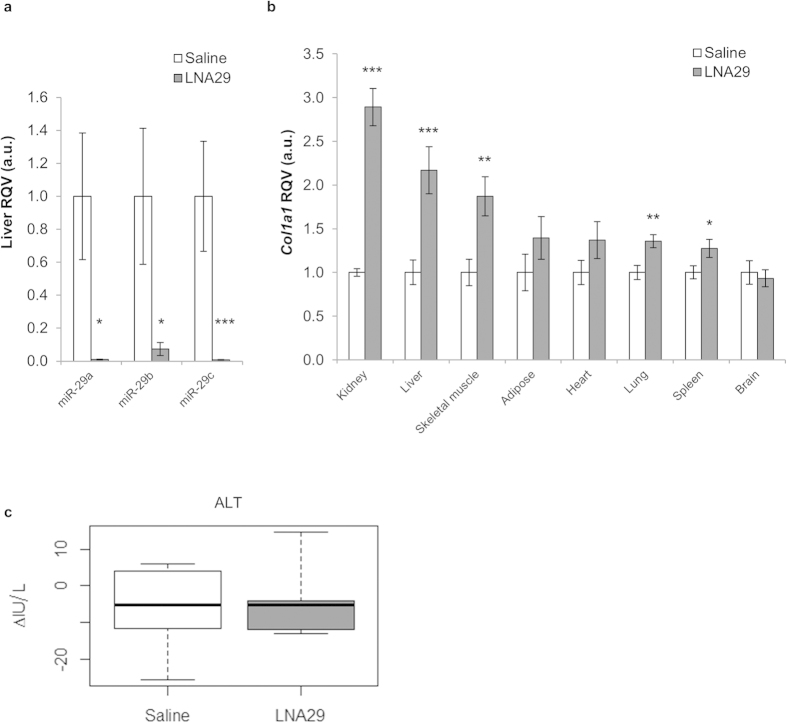
*In vivo* LNA29 administration effectively inhibits the miR-29 family in the liver. **(a,b)** RT-qPCR analysis of LNA29-treated (20 mg/kg) C57BL/6 J female mice (n = 14) with saline-treated age-, gender-, and strain-matched controls (n = 12) shows that endogenous expression of hepatic miR-29 is dramatically reduced (**a**); expression of *Col1a1*, a validated target of miR-29, is significantly elevated in several tissues including liver (**b**); *U6* and *Rps9* were used as expression normalizers for miRNA and gene analysis, respectively. (**c**) Plasma alanine transaminase (ALT) measurements (IU/L) are shown for LNA29-treated animals (n = 6) and saline treated controls (n = 5). *p < 0.05; **p < 0.01; ***p < 0.005; p-values were calculated by two-tailed unpaired Student’s t-test. Error bars represent standard error of the mean.

Two independent sets of 10-12 week-old C57BL/6 J female mice were injected with either saline (set 1, n = 6; set 2, n = 6) or locked nucleic acid inhibitors of miR-29abc (LNA29; set 1, n = 6; set 2, n = 8). As a control, another set of 10-12 week-old C57BL/6 J female mice were injected with either saline (n = 6) or a locked nucleic acid inhibitor of miR-27b (LNA-control, n = 5) (Methods). Seven days post injection the animals were sacrificed and tissue was collected. To determine the efficacy of endogenous miR-29 knock-down after treatment with LNA29, we examined the levels of miR-29 in liver. As expected, hepatic miR-29 expression was dramatically reduced in LNA29 treated animals (Fig. 1a), but not significantly altered in LNA-control treated animals (Supplementary Fig. S1). Furthermore, the levels of a validated miR-29 target gene, collagen type I alpha 1 (*Col1a1*)^27^, were increased significantly in multiple tissues including the kidney (2.9 fold), liver (2.2 fold), skeletal muscle (1.9 fold), lung (1.4 fold) and spleen (1.3 fold) (Fig. 1b). *Col1a1* levels were unchanged in brain, which is consistent with previous reports that tail-vein injected LNAs do not efficiently cross the blood-brain barrier. Another established miR-29 target gene, collagen type III alpha 1 (*Col3a1*)^27^, was also increased in the livers of LNA29-treated mice (Supplementary Fig. S1), but both *Col1a1* and *Col3a1* were not significantly altered in the livers of mice treated with LNA-control (Supplementary Fig. S1). Plasma ALT was measured to test for general liver damage and the levels were not significantly altered in LNA29-treated animals (Fig. 1c). Taken together, these data indicate that systemic administration of LNA29 leads to specific and potent suppression of miR-29 activity with no overt liver toxicity.

### Inhibition of miR-29 leads to a dramatic reduction in circulating cholesterol and triglycerides

**Figure 2:**
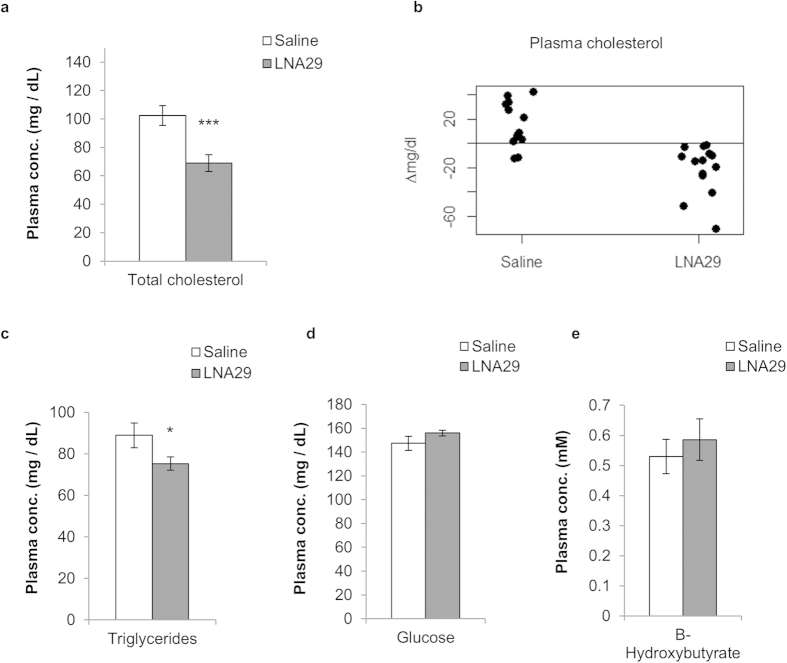
Inhibition of miR-29 leads to a significant reduction of plasma cholesterol and triglycerides. **(a,c–e)** Plasma was isolated from whole blood collected via submandibular bleed one week post-treatment with either LNA29 (n = 14) or saline (n = 12) and was analyzed for levels of total cholesterol (**a**), triglycerides (**c**), glucose (**d**), and β-hydroxybutyrate (**e**). **(b)** Changes in total plasma cholesterol (∆mg/dL) between pre- and post-dosing are shown for each mouse treated with either LNA29 (n = 14) or saline (n = 12). *p < 0.05; ***p < 0.005; p-values were calculated by two-tailed unpaired Student’s t-test. Error bars represent standard error of the mean.

Next, we assessed the effect of miR-29 inhibition on circulating lipid levels (Methods). We measured total cholesterol and triglycerides, as well as glucose and β-hydroxybutyrate (ketones), in plasma samples collected one week prior to dosing with LNA29 or saline and again six days post-dose. Pre-dose levels were comparable across all mice (Supplementary Fig. S2). Post-dose plasma cholesterol levels in LNA29-treated mice (n = 14) were reduced relative to saline-treated controls (n = 12) by ~40% (Fig. 2a), which matches what has been observed previously with the use of statin drugs. All 14 mice treated with LNA29 exhibited reduced plasma cholesterol compared to pre-dose levels, with an average reduction of 21.2 mg/dL (Fig. 2b). Plasma triglycerides were also significantly reduced in LNA29-treated animals compared to saline-treated controls (~15% decrease) (Fig. 2c). Glucose and β-hydroxybutyrate levels were not significantly altered in LNA29 treated mice (Fig. 2d,e). Neither plasma cholesterol nor triglycerides were altered in mice treated with LNA-control (n = 5) (Supplementary Fig. S3). Taken together, these data suggest a specific and strong effect of miR-29 inhibition on circulating lipids.

### Inhibition of miR-29 dysregulates more than 850 genes in mouse liver

**Figure 3:**
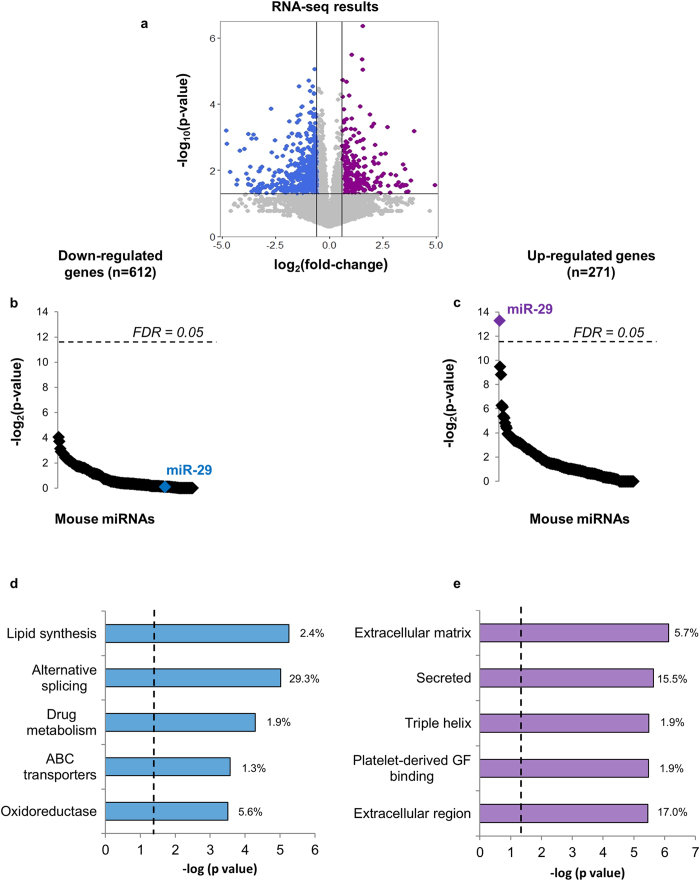
RNA-seq identifies lipid synthesis as the most enriched pathway among genes down-regulated in mouse liver upon inhibition of miR-29. (**a**) Deep sequencing of total RNA revealed 612 down-regulated genes (blue) and 271 up-regulated genes (purple) in the livers of LNA29-treated mice (n = 6) compared to saline-treated (n = 6) controls. (**b,c**) Results of miRNA target site enrichment analysis shown for down-regulated (**b**) and up-regulated genes (**c**). (**d,e**) Results of gene ontology (GO) term enrichment analysis using NIH David shown for down-regulated (**d**) and up-regulated genes (**e**). Vertical dashed lines represent corrected p < 0.05. RNA-seq p-values were calculated by one-tailed unpaired Student’s t-test. Percentages represent the number of genes from the gene set involved in the pathway divided by the total number of genes in the gene set.

The liver is the primary site of lipid synthesis. Therefore, in order to examine the mechanisms underlying the circulating lipid phenotype, we performed deep sequencing of messenger RNAs from the livers of both LNA29-treated (n = 6) and saline-treated mice (n = 6) (Methods). The mRNA expression levels of 883 genes were significantly altered (271 up, 612 down) in the livers of LNA29-treated mice compared to saline-treated controls (Fig. 3a, Supplementary Table S1; GEO accession number GSE63493). Suppression of miR-29 activity should lead to up-regulation of direct target genes. Accordingly, we found that only the up-regulated gene set was significantly enriched for predicted target sites of miR-29 and no other miRNA (Fig. 3b,c), which demonstrates the efficacy and specificity of LNA29. Interestingly, the 612 down-regulated genes were most significantly over-represented in lipid synthesis pathways (Fig. 3d, Supplementary Tables S2 and S3), whereas the up-regulated gene set was not enriched for any lipid-related pathway (Fig. 3e, Supplementary Tables S4 and S5M). These data indicate that inhibition of miR-29 leads to widespread repression of genes in pathways that govern both fatty acid and cholesterol synthesis.

### Inhibition of miR-29 represses fatty acid synthesis and reduces fatty acid content in the liver

**Figure 4:**
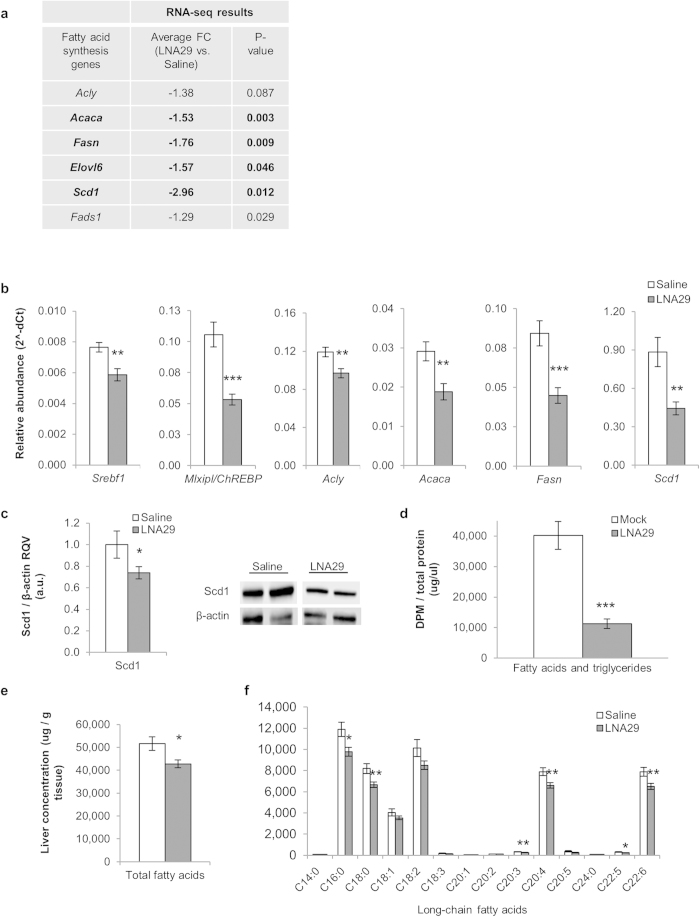
Inhibition of miR-29 represses the fatty acid synthesis pathway and reduces fatty acid content in the liver. (**a**) Expression fold-change (FC) by RNA-seq in LNA29-treated mice (n = 6) relative to saline-treated controls (n = 6) is shown for key genes in the hepatic fatty acid synthesis pathway. p-values were calculated by one-tailed unpaired Student’s t-test. (**b**) RT-qPCR validation of RNA-seq results shown for *Srebf1*, *Mlxipl/ChREBP*, *Acly*, *Acaca*, *Fasn* and *Scd1*. Relative abundance (2^-dCt) was compared between LNA29-treated (n = 14) and saline-treated (n = 12) mice. *Rsp9* was used as the normalizer. (**c**) Densitometry analysis shown for Scd1 protein in livers from LNA29-treated mice (n = 10) compared to saline-treated controls (n = 8). β-actin was used as the loading control. Immunoblot results also shown for two representative mice from each treatment group. (**d**) Quantification of radiolabeled acetate incorporation into fatty acids *in vitro* (Huh7 cells) after transfection with LNA29a (10 nM, n = 6) or transfection reagent alone (Mock, n = 6). (**e**) Measurement of total fatty acid levels in the livers of LNA29-treated animals (n = 14) compared to saline-treated controls (n = 12). (**f**) Levels of individual long-chain fatty acids in LNA29-treated relative to saline-treated mice. C14:0 (Myristic acid), C16:0 (Palmitic acid), C18:0 (Stearic acid), C18:1 (Oleic acid), C18:2 (Linoleic acid), C18:3 (Linolenic acid), C20:1 (Gondoic acid), C20:2 (Eicosadienoic acid), C20:3 (Homogamalinolenic acid), C20:4 (Arachidonic acid), C20:5 (Eicosapentaenoic acid), C24:0 (Lignoceric acid), C22:5 (Docosapentaenoic acid), C22:6 (Docosahexaenoic acid). *p < 0.05; **p < 0.01; ***p < 0.005; p-values were calculated by two-tailed unpaired Student’s t-test. Error bars represent standard error of the mean.

Several genes in the fatty acid synthesis pathway were significantly down-regulated by RNA-seq in the livers of LNA29-treated mice (Fig. 4a). We validated these findings using real-time quantitative PCR (RT-qPCR) for *Srebf1* (25% decrease), *Mlxipl*/*ChREBP* (50%), *Acly* (20%), *Acaca* (35%), *Fasn* (50%), and *Scd1* (50%) (Fig. 4b). Srebf1 and ChREBP are master transcriptional regulators of lipid synthesis^28^,^29^, and Acly, Acaca, Fasn, and Scd1 are key enzymes in fatty acid synthesis. We confirmed by immunoblot analysis that Scd1 protein was also significantly down-regulated (~25% loss) in LNA29-treated livers (Fig. 4c).

Based on these data, we hypothesized that inhibition of miR-29 leads to suppression of *de novo* fatty acid synthesis. To test this hypothesis we transfected human hepatoma (Huh7) cells with LNAs against miR-29a, which reduced fatty acid production by ~70% (Fig. 4d). LNAs against miR-29bc conferred a very similar effect (data not shown). Consistent with this finding, inhibition of miR-29 *in vivo* significantly reduced (~20% decrease) total fatty acid content in the livers of the LNA29-treated mice (n = 14) compared to saline-treated controls (n = 12) (Fig. 4e). Among the long-chain fatty acids, palmitic acid (C16:0), stearic acid (C18:0), homogamma-linolenic acid (GLA, C20:3), arachidonic acid (C20:4), docosapentaenoic (DPA, C22:5) and docosahexaenoic acid (DHA, C22:6) were each significantly reduced (Fig. 4f). Palmitic acid is the most common fatty acid in animals and is produced by enzymatic activity of Fasn, which we showed is significantly reduced in the livers of LNA29-treated mice (Fig. 4a,b).

### Inhibition of miR-29 represses the cholesterol synthesis pathway but slightly increases total hepatic cholesterol levels

**Figure 5:**
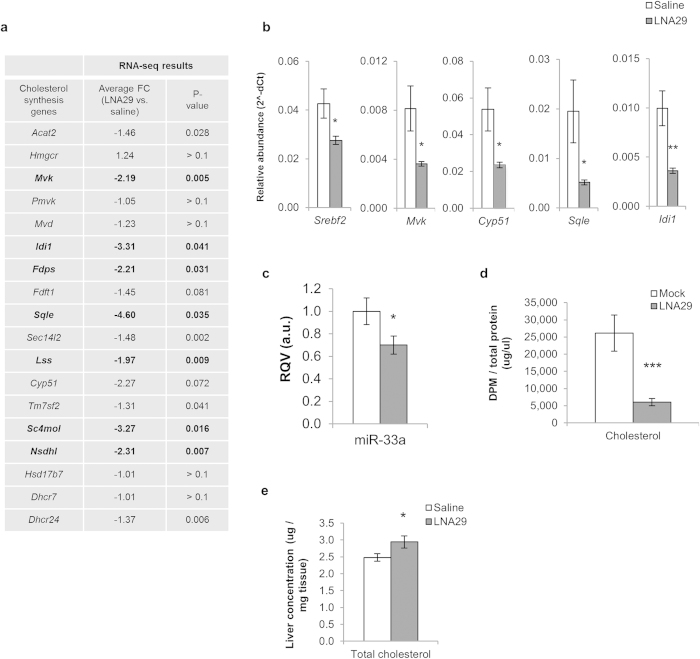
Inhibition of miR-29 represses the cholesterol synthesis pathway but slightly increases total hepatic cholesterol levels. (**a**) Expression fold-change (FC) by RNA-seq in LNA29-treated mice (n = 6) relative to saline-treated controls (n = 6) is shown for key genes in the hepatic cholesterol synthesis pathway. p-values were calculated by one-tailed unpaired Student’s t-test. (**b,c**) RT-qPCR results shown for *Srebf2*, *Mvk*, *Cyp51*, *Sqle* and *Idi1* (**b**) as well as for miR-33a (**c**), which is co-transcribed with *Srebf2*. (**d**) Quantification of radiolabeled acetate incorporation into cholesterol *in vitro* (Huh7 cells) after transfection with LNA29a (10 nM, n = 6) or transfection reagent alone (Mock, n = 6). (**e**) Measurement of total cholesterol levels in the livers of LNA29-treated mice (n = 14) compared to saline-treated controls (n = 12). *p < 0.05; **p < 0.01; ***p < 0.005; p-values were calculated by two-tailed unpaired Student’s t-test. Error bars represent standard error of the mean.

Numerous genes in the cholesterol synthesis pathway were significantly down-regulated in the livers of LNA29-treated mice (Fig. 5a). We validated these findings using RT-qPCR for *Srebf2* (35% decrease), *Mvk* (55%), *Cyp51* (55%), *Sqle* (75%), and *Idi1* (65%) (Fig. 5b). Srebf2, a master transcriptional regulator of cholesterol homeostasis^28^, is co-transcribed with miR-33a. We examined hepatic levels of miR-33a as a marker of transcription at the *Srebf2* locus and found that it was decreased by ~30% (Fig. 5c).

Interestingly, unlike most of the other enzymes in the cholesterol synthesis pathway, the rate-limiting enzyme, 3-Hydroxy-3-Methylglutaryl-CoA Reductase (*Hmgcr*), was not down-regulated by LNA29 (fold-change = 1.2) (Fig. 5a). Bioinformatic analysis revealed that in contrast to most other cholesterologenic genes, *Hmgcr* is predicted to be a direct target of miR-29. To test this prediction, we performed standard luciferase and site-directed mutagenesis assays with the 3′ UTR of HMGCR (Methods). Relative levels of luciferase activity were significantly decreased in the presence of miR-29a mimic for the plasmid with the *HMGCR* 3′ UTR, while a single point mutation in the miR-29 target site significantly (though not completely) rescued this effect (Supplementary Fig. S4). These results suggest that miR-29 inhibition *in vivo* likely alleviates miR-29-mediated direct repression of *Hmgcr*, thereby counteracting the overall suppressive effect of miR-29 inhibition on the cholesterol synthesis pathway.

Although *Hmgcr* is not significantly altered at the mRNA level in LNA29-treated livers, because most other genes in the cholesterol synthesis pathway are significantly down-regulated by miR-29 inhibition, we hypothesized that inhibition of miR-29 leads to an overall decrease in *de novo* cholesterol synthesis. To test this hypothesis, Huh7 cells were transfected as before with either LNA29a or LNA29bc. The knockdown of miR-29a significantly reduced acetate incorporation into cholesterol (Fig. 5d) and the results were very similar for LNA29bc (data not shown). However, notably, LNA29-treatment *in vivo* led to a modest but significant increase in total hepatic cholesterol levels (fold-change = 1.2) (Fig. 5e), suggesting that miR-29 may control not only cholesterol synthesis, but also other pathways that contribute to total intracellular cholesterol levels.

### LNA29 alleviates miR-29-mediated repression of anti-lipogenic factors Ahr, Foxo3 and Sirt1

**Figure 6:**
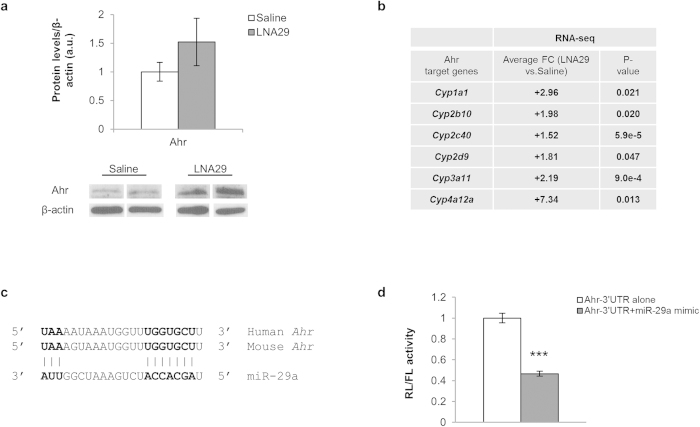
Inhibition of miR-29 alleviates miR-29-mediated repression of the anti-lipogenic transcription factor *Ahr*. (**a**) Densitometry analysis for hepatic Ahr protein from LNA29-treated mice (n = 5) compared to saline-treated controls (n = 3). β-actin was used as the loading control. Immunoblot results also shown for two representative mice from each treatment group. (**b**) Expression fold-change (FC) by RNA-seq in LNA29-treated mice (n = 6) relative to saline-treated controls (n = 6) is shown for genes known to be transcriptionally activated by Ahr. p-values were calculated by one-tailed unpaired Student’s t-test. **(c)** A diagram of the predicted base pairing between miR-29 and the *Ahr* 3′ UTR sequence of both mouse and human is shown. (**d**) Effects of miR-29a mimic (10 nM) in HEK293T cells on the activity of *Firefly* (FL) luciferase with or without the *Ahr* 3′ UTR normalized to *Renilla* luciferase (RL) are shown. ***p < 0.005; *p < 0.05; p-values were calculated by two-tailed unpaired Student’s t-test. Error bars represent standard error of the mean.

To explain the widespread down-regulation of genes in the hepatic fatty acid and cholesterol synthesis pathways mediated by LNA29-treatment, we hypothesized that inhibition of miR-29 promotes specific anti-lipogenic factors via loss of direct miR-29 targeting. Aryl hydrocarbon receptor (Ahr) has been implicated in the suppression of both fatty acid and cholesterol synthesis in the liver^30^,^31^. We found that Ahr was ~1.5-fold up-regulated in the mouse liver at both the mRNA and protein level by LNA29-treatment (Supplementary Table S1, Fig. 6a). Ahr is also widely appreciated as an activator of xenobiotic-metabolizing enzymes such as the cytochrome P450s^32^. RNA-seq data analysis revealed that several cytochrome P450 genes were indeed significantly up-regulated (ranging from ~2 to ~7-fold) in the livers of LNA29-treated mice, including *Cyp1a1*, *Cyp2b10*, *Cyp2c40*, *Cyp2d9*, *Cyp3a11* and *Cyp4a12a* (Fig. 6b), consistent with elevated Ahr transcriptional activity.

miR-29 is predicted to target the *Ahr* 3′ UTR at a site that is highly conserved between human and mouse (Fig. 6c). To determine if the site is functional, we performed standard 3′ UTR reporter gene assays (Methods). In the presence of the miR-29a mimic, relative levels of luciferase activity were reduced by more than 50% (Fig. 6d). Furthermore, a highly conservative single point mutation in the miR-29 target site slightly but significantly rescued this effect (Supplementary Fig. S5). These data support the model that inhibition of miR-29 *in vivo* leads to the loss of miR-29-mediated repression of *Ahr* and thereby promotes the anti-lipogenic activity of Ahr.

**Figure 7:**
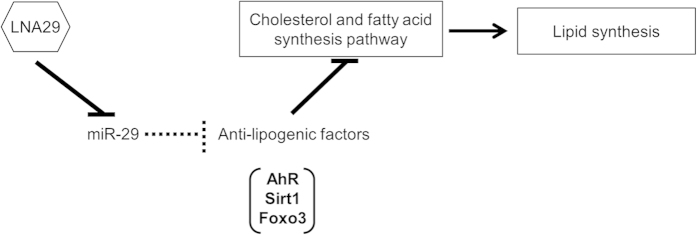
Schematic of the putative molecular mechanism underlying the effects of LNA29 treatment. In this model, under normal conditions, miR-29 may promote lipogenesis through suppression of *Ahr*, *Sirt1* and *Foxo3*. Upon LNA29 treatment, miR-29 activity is suppressed, and Ahr, Sirt1, and Foxo3 levels are elevated, leading to reduced cholesterol and fatty acid synthesis.

In addition to Ahr, the transcription factor forkhead box O3 (Foxo3) and the sirtuin family of NAD-dependent deacetylases have also been linked to the suppression of hepatic lipid synthesis^33^,^34^. Hepatic mRNA levels of a single sirtuin family member, sirtuin 1 (Sirt1), were increased in the LNA29-treated mice. Both Sirt1 and Foxo3 were previously validated as direct targets of miR-29 in other cell types^35^,^36^; therefore, we sought to evaluate whether Sirt1 and Foxo3 protein levels were altered in the livers of LNA29-treated mice. Immunoblot analysis revealed that the protein levels of both were increased (~1.6-fold) in the livers of LNA29-treated mice (Supplementary Fig. S6). Notably, Ahr and Sirt1 have been identified recently as transcriptional activators of Fgf21^37^,^38^,^39^, an emerging metabolic regulator with potential therapeutic value for dyslipidemia, obesity, type 2 diabetes and related diseases^40^,^41^. We demonstrated that *Fgf21* mRNA levels were increased in the livers of LNA29-treated mice (Supplementary Table S1). These data lend further support to the model that inhibition of miR-29 leads to the suppression of lipogenic programs likely in part through elevated expression and activity of the anti-lipogenic factors Ahr and Sirt1 (Fig. 7).

## Discussion

In this study, we used LNA technology to investigate the effects of loss of miR-29 function *in vivo* on lipid levels. We demonstrated that the knockdown of miR-29 in mice led to a significant reduction in plasma cholesterol and triglyceride levels, as well as reduced fatty acid content in the liver. RNA sequencing analysis revealed aberrant expression levels for over 850 genes, including 612 down-regulated genes that were most significantly over-represented in lipid synthesis pathways. Real-time quantitative PCR and immunoblot analyses confirmed altered cholesterologenic and lipogenic transcriptional profiles in the livers of mice treated with LNA29. We also demonstrated that loss of miR-29 function increased the mRNA and protein levels of the transcription factors Ahr and Foxo3, as well as the deacetylase Sirt1.

Our findings suggest that the loss of miR-29 leads to altered Ahr transcriptional activity. Previous studies have shown that miR-29 directly targets *Sirt1* and *Foxo3*^35^,^36^ and our study reveals that miR-29 likely also directly targets and represses *Ahr*. The activation of each of Ahr, Sirt1, and Foxo3 has been linked to the suppression of lipid synthesis^31^,^32^,^33^,^34^ (Fig. 7). Future work should investigate whether one or more of these factors are required/essential for miR-29-mediated control of lipid levels. It must be noted that activation of Sirt1 can also stimulate histone deacetylase 1 (HDAC1), a protein that in some contexts may promote cholesterol synthesis^42^. However, Sirt1 control of HDAC1 has been demonstrated primarily in cell types other than those within liver, most notably neurons^43^,^44^. In the fasted liver, Sirt1 control of HDAC1 has not been well investigated. Contrastingly, Sirt1-mediated repression of hepatic cholesterol and fatty acid synthesis has been reported in numerous studies^33^,^45^,^46^. The relevance of HDAC1 to the role of Sirt1 in miR-29-mediated regulation of lipid synthesis merits further detailed investigation.

Our study focused on direct (Ahr, Foxo3) and indirect (Sirt1) regulators of transcription as mediators of the effect of miR-29 on lipogenic programs in the liver. However, other mechanisms are also possible. One notable possibility involves insulin induced gene 1 and 2 (Insig1 and Insig2), which sequester Srebp1 and Srebp2 in the endoplasmic reticulum under high sterol conditions, thereby suppressing fatty acid and cholesterol synthesis. LNA29 treated mouse livers exhibited a significant increase in *Insig2* mRNA levels (Supplementary Table S1). It is possible that up-regulation of Insig2 contributes to decreased cholesterol synthesis in LNA29 treated mice.

We showed through *in vitro* acetate incorporation assays that inhibition of miR-29 dramatically reduced *de novo* synthesis of both fatty acids and cholesterol. In the miR-29 knockdown mice hepatic fatty acid levels were reduced, whereas cholesterol levels were slightly elevated. These findings raise the possibility that miR-29 regulates not only cholesterol synthesis, but also other important pathways that contribute to overall cholesterol homeostasis. Further detailed investigation is required to establish how miR-29 coordinates control of cholesterol synthesis, uptake, storage, metabolism, and/or efflux to fine-tune intracellular cholesterol levels, such as in the case of miR-223, which we recently demonstrated controls not only hepatic cholesterol synthesis, but high-density lipoprotein cholesterol uptake and cellular cholesterol efflux as well^25^.

Our findings indicate that miR-29 is a critical regulator of cholesterol and fatty acid synthesis. A recent study reported that inhibition of miR-29a leads to accumulation of fat in the liver^47^. However, a notable distinction between that study and ours is that we inhibited all three miR-29 family members as opposed to only miR-29a (Fig. 1). We also performed transcriptome-wide analysis of liver gene expression profiles to firmly establish the efficacy of miR-29abc inhibition (Fig. 3), in contrast to evaluating only a few known target genes^47^. These and other methodological differences between the studies merit further detailed investigation in order to improve our understanding of miR-29 functions in the liver and the dose-dependent nature of those functions.

miR-29 has been linked to several other biological processes, including cell proliferation^48^, interferon response^49^, extracellular matrix homeostasis^50^, and gluconeogenesis^51^. Decreased expression of miR-29 has been associated with multiple disease pathologies, including organ fibrosis and rhabdomyosarcoma^52^. It must be noted that it is not known whether the regulatory effects of miR-29 *in vivo* are greater on some pathways compared to others. To determine whether miR-29 represents a candidate therapeutic target for lipid metabolic disorders, future work must carefully assess the effects of long-term inhibition of miR-29 on not only hepatic and circulating lipid profiles, but also additional phenotypes such as hepatic proliferative capacity, inflammation, fibrosis, and insulin sensitivity.

## Methods

### Animal Studies

Eight week old C57BL/6 J female mice were purchased from Jackson Laboratories (Bar Harbor, ME) and maintained on a 12 hr light/dark cycle with access to a standard chow diet and H_2_O *ad libitum*. After a minimum of a 10 day period of acclimation, the animals were weighed and injected via tail vein with either RNase-free sterile saline (BioO Scientific; Austin, TX) or LNAs against mmu-miR-29a-3p (5′ ATTTCAGATGGTGCT 3′) and mmu-miR-29bc-3p (5′ ATTTCAAATGGTGCT 3′) or mmu-miR-27b-3p (5′ ACTTAGCCACTGTGA 3′) at 20mg/kg each (Exiqon; Woburn, MA). Blood was collected via submandibular bleed within one week before dosing with LNA and again six days post-dosing. Seven days after dosing with LNA, following an 18 hour fast, the animals were sacrificed by cervical dislocation without anesthesia and organs were collected. Organs were flash frozen in liquid nitrogen and kept at −80^o^C for further analysis. All animal work was performed in accordance with the Public Health Service Policy on Humane Care and Use of Laboratory Animals, and all studies were approved by the Institutional Animal Care and Use Committee (IACUC) at the University of North Carolina at Chapel Hill.

### RT-qPCR

Total RNA was isolated from tissues using the Total RNA Purification kit (Norgen; Ontario, Canada). One ug of total RNA was used for reverse transcription with the High Capacity RNA to cDNA kit (Life Technologies; Grand Island, NY) and 200 ng RNA was used for reverse transcription with the TaqMan microRNA Reverse Transcription kit (Life Technologies). Both miRNA and gene expression qPCR were performed using TaqMan assays with either TaqMan Universal PCR Master Mix (miRNA qPCR) or TaqMan Gene Expression Master Mix (Life Technologies) per the manufacturer’s protocol, on a BioRad CFX96 Touch Real Time PCR Detection System (Bio-Rad Laboratories, Inc.; Richmond, CA). Reactions were performed in triplicate using either *U6* (miRNA) or *Rps9* (gene expression) as the normalizer.

### Plasma metabolite analysis

Plasma was isolated by spinning whole blood for 10 min at 10000 g, 4 ^°^C. Plasma was used to measure total cholesterol, glucose, triglycerides, β-hydroxybutyrate and alanine aminotransferase (ALT) at the UNC Nutrition Obesity Research Center (NORC) Nutritional Biochemistry & Molecular Biology core facility.

### RNA deep sequencing

RNA was isolated as described previously from hepatic tissue from mice treated with LNA29 or saline. Libraries for RNA-seq were prepared using the Illumina TruSeq polyA + Sample Prep Kit. Single read sequencing (x100) was carried out on the Illumina HiSeq 2000 platform. Mapping of sequencing reads was performed by MapSplice2^53^ and quantification of gene expression by RSEM^54^. Differentially expressed genes in livers of LNA29-treated mice compared to saline-treated controls were identified by one-tailed, unpaired Student’s t-test. Genes were considered significantly up- or down-regulated if they had a fold change ≥+/−1.5 at a p-value ≤ 0.05. Gene ontology analysis was performed using both NIH DAVID and QIAGEN’s Ingenuity^®^ Pathway Analysis (IPA^®^, QIAGEN Redwood City; www.qiagen.com/ingenuity).

### Bioinformatics

3′ UTRs of significantly up- and down-regulated genes were analyzed for enrichment of predicted miRNA target sites as previously described^55^.

### Western blotting

Protein was isolated using RIPA buffer (Sigma-Aldrich; St. Louis, MO) and quantitated using the Pierce® Microplate BCA Protein Assay Kit – Reducing Agent compatible (Thermo Scientific Pierce; Rockford, IL). An Any kD^TM^ Mini-Protean TGX^TM^ precast gel (Bio-Rad Laboratories, Inc.; Richmond, CA) was run for 2 hours at 120 v, and protein was transferred to a nitrocellulose membrane using the Bio-Rad Midi Transfer Pack on the Bio-Rad Trans-Blot® Turbo Blotting System. Membranes were blocked in 5% non-fat dry milk in TBST for 1 hour at room temperature (RT) while shaking. Primary antibody incubation was done in 4% milk/TBST for 16 hours at 4 ^°^C with shaking as follows: Ahr—2.5 ug/ml (Abcam; Cambridge, MA), Foxo3, Scd1 and Sirt1—1 ug/ml (Abcam). Membranes were washed 3 times for 10 minutes with TBST at RT with shaking and then probed with secondary Ab in 1% milk (Goat anti-rabbit from Abcam: Ahr, Foxo3, Scd1; or anti-mouse – Cell Signaling; Danvers, MA: Sirt1) for 1 hour at RT with shaking. Finally, membranes were washed 3 times in TBST for 10 minutes at RT with shaking and signal was detected using the Amersham™ ECL™ Prime Western Blotting Detection Reagent (GE Healthcare Life Sciences; Piscataway, NJ). Membranes were exposed to film and images were analyzed using the Image Studio Lite software program (Licor, Lincoln, NE). The control antibody used was β-actin peroxidase (Sigma-Aldrich), diluted 1:40000 in 1% milk/TBST, probed for 30 minutes at room temperature while shaking and subject to ECL blotting detection reagent and film exposure. Protein concentration was expressed as fold increase relative to the control.

### Luciferase assay

HEK293T cells were maintained in 25 mM glucose DMEM (Sigma-Aldrich) supplemented with 10% FBS and 2 mM L-glutamine (Invitrogen; Grand Island, NY), and cultured in a humidified incubator at 37 ^°^C and 5% CO_2_. HEK293T cells were seeded into 24 well plates and allowed to grow overnight. Once the cells were approximately 70% confluent, they were transfected with 200 ng of pEZX-MT01 empty vector, vector containing the 3′UTR of *HMGCR or AHR* (GeneCopoeia, Rockville, MD) and 10 nM miRIDIAN microRNA hsa-miR-29a-3p mimic (Sequence: 5′ UAGCACCAUCUGAAAUCGGUUA 3′, Dharmacon; Lafayette, CO). Transfection was performed using Lipofectamine 2000 (Life Technologies). After 48 hours, the cells were lysed and luciferase activity was measured using the Luc-Pair luciferase assay kit (Agilent; Santa Clara, CA) on a GloMax® 96 Microplate Luminometer (Promega; Madison, WI). Site-directed mutagenesis was performed with the QuikChange II XL Site-Directed Mutagenesis Kit (Agilent).

### Acetate incorporation assay

Human hepatoma cells (Huh7) were seeded into 6-well plates at a density of 1 × 10^5^ cells/mL. After overnight growth, cells were transfected with either LNA29a, LNA29bc, or transfection reagent only (mock). Forty-eight hours after transfection, media was removed and replaced with DMEM low glucose lipoprotein deficient serum (LPDS) media. Seventeen hours later, low glucose media was removed and replaced with fresh serum-free low glucose media (cholesterol free) with 1uCi3H-acetic acid per well (0.5uCi/ml media) and incubated for 6 hours. Cells were washed twice with 1X Phosphate Buffered Saline. Two mL 3:2 hexane:isopropanol were added to each well; the cells were allowed to sit for 2 hours then placed in glass vials. Samples were dried down under nitrogen and 30 μl of cold 0.1 μg cholesterol:cholesteryloleate was added. Thirty μl of resuspended lipids were placed onto a plate and thin layer chromatography was performed (petroleum ether:diethyl ether:acetic acid). Spots were resolved with iodine, and cholesteryl ester, cholesterol and triglyceride spots were cut out and placed in 10 mL scintillation fluid. Total protein for each well was quantified using the BCA assay.

### Liver fatty acid analysis

Total lipids were extracted from 100 mg of liver tissue, first with methanol (MeOH)/chloroform (CHCl_3_), then with MeOH/CHCl_3_/H_2_0 (2:1:0.8) at the UNC Nutrition Obesity Research Center (NORC) Nutritional Biochemistry and Molecular Biology Core. Lipids were saponified and the fatty acids trans-methylated. The lower phase containing the fatty acid methyl esters was carefully transferred to a clean tube and evaporated to dryness under nitrogen. Fatty acid methyl esters were resuspended in 50 μl CHCl_3_:undecane (1:4) and analyzed using an Agilent 7890A GC system equipped with a capillary column coated with 70% cyanopropyl polysilphenylene-silozane (10 m × 100 μm ID-BPX70 0.2 um; SGE, Austin, TX). Data was analyzed with the Agilent GC Software ChemStation Rev. B03.02-SR2. Heptadecanoic acid (17:0) was added to the samples as an internal standard to correct for recovery. Individual fatty acids were identified by comparing their retention times with fatty acid standards (Nu Chek Prep, Elysian, MN) and quantified by comparing their peak areas with standards.

## 

## Additional Information

**Accession codes:** GEO accession number GSE63493; RNA-seq data from livers of LNA29 treated mice compared to saline-treated controls.

**How to cite this article**: Kurtz, C. L. *et al.* Inhibition of miR-29 has a significant lipid-lowering benefit through suppression of lipogenic programs in liver. *Sci. Rep.* **5**, 12911; doi: 10.1038/srep12911 (2015).

## Supplementary Material

Supplementary Information
