# Supplementary material for: Inhibition of miR-29 has a significant lipid-lowering benefit through suppression of lipogenic programs in liver
Source: Sci Rep. 2015 Aug 6;5:12911. doi: 10.1038/srep12911 (PMC4526858; doi:10.1038/srep12911)
Supplement: Supplementary Information [file srep12911-s1.pdf]

Inhibition of miR-29 has a significant lipid-lowering benefit through suppression of lipogenic programs in liver

C. Lisa Kurtz[1], Emily E. Fannin[1], Cynthia L. Toth[2], Daniel S. Pearson[3], Kasey C. Vickers[2], and Praveen Sethupathy[1,4]*

Supplementary Figure S1

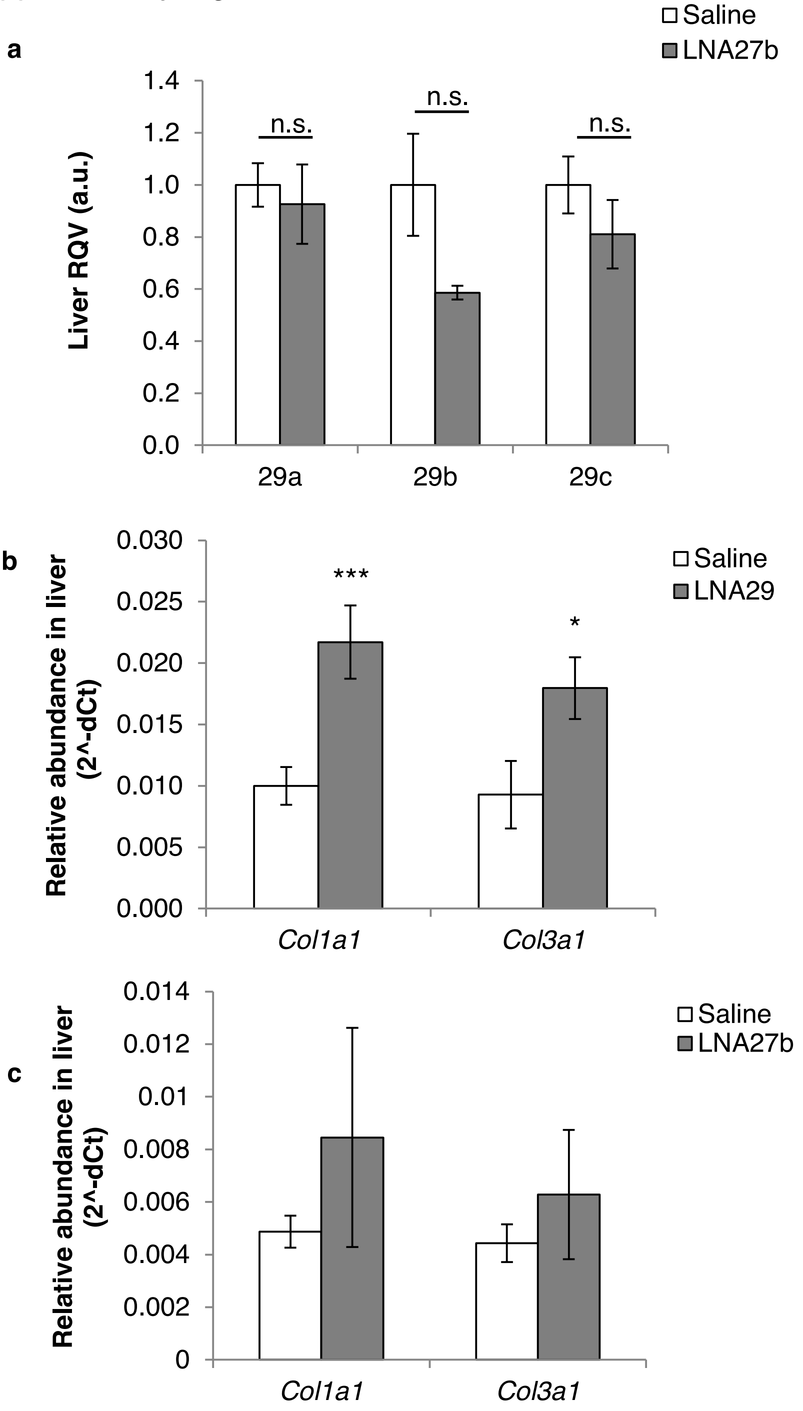

Supplementary Figure S1. (a) RT-qPCR analysis of LNA27b-treated (20 mg/kg) C57BL/6J female mice (n= 6) with saline-treated age-, gender-, and strain-matched controls (n=5) shows that endogenous expression of hepatic miR-29 is not significantly altered. **(b)** mRNA levels of *Col1a1* and *Col3a1*, validated miR-29 target genes, are significantly elevated in the liver of LNA29-treated mice. **(c)** mRNA levels of *Col1a1* and *Col3a1* are not significantly altered in the liver of LNA27b-treated mice. *U6* and *Rps9* were used as expression normalizers for miRNA and gene analysis, respectively. ***, $p<0.005$; *, $p<0.05$; p-values were calculated by two-tailed unpaired Student's t-test. Error bars represent standard error of the mean.

Supplementary Figure S2

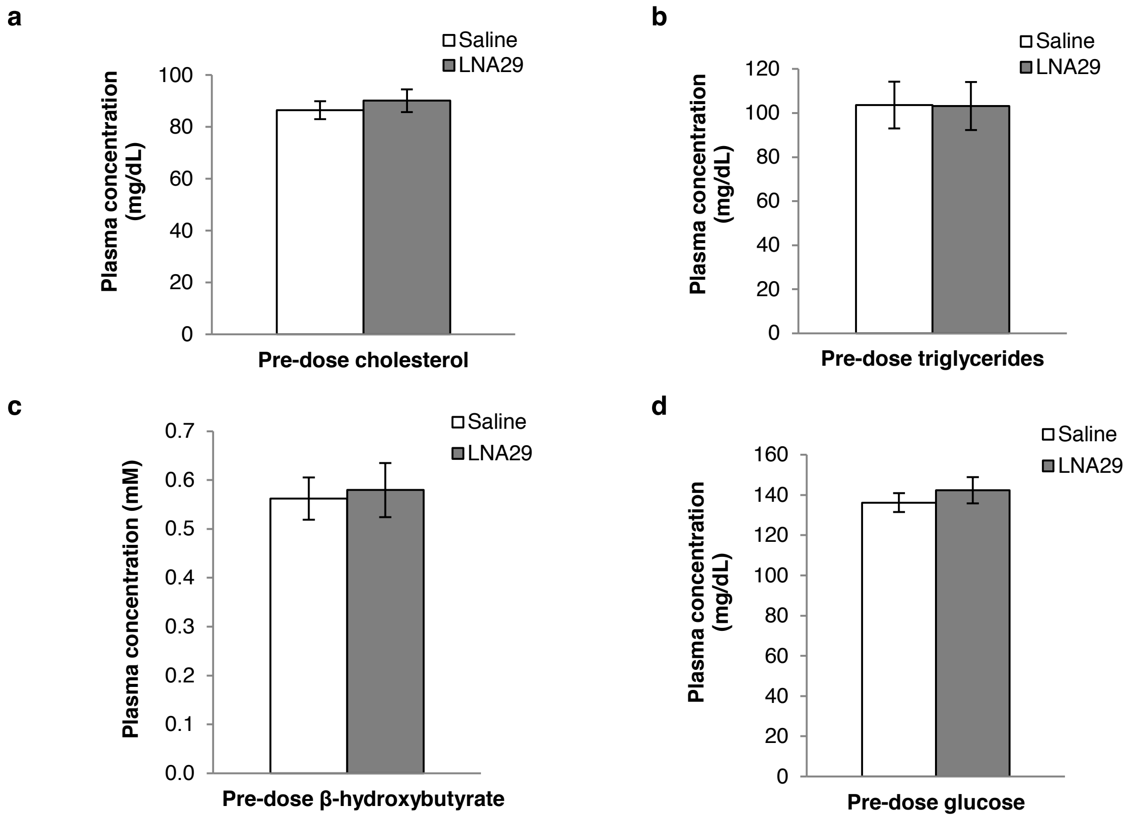

Supplementary Figure S2. Plasma was isolated from whole blood collected via submandibular bleed pre-treatment with either LNA29 (n=14) or saline (n=12) and was analyzed for levels of total cholesterol (a), triglycerides (b), glucose (c), and β-hydroxybutyrate (d).

Supplementary Figure S3

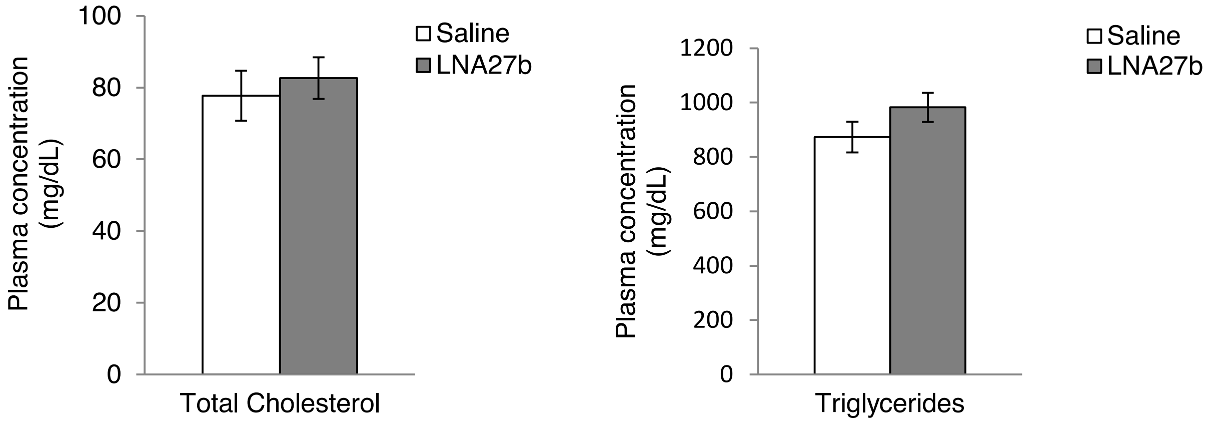

Supplementary Figure S3. Plasma was isolated from whole blood collected via submandibular bleed one week post-treatment with either LNA27b (n=5) or saline (n=6). Total cholesterol and triglycerides were not significantly altered in LNA-control treated mice.

Supplementary Figure S4

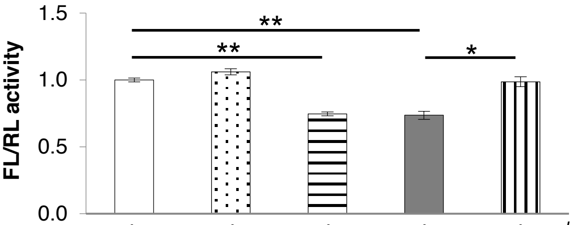

+	+	+	+	+	*HMGCR* 3' UTR
--	--	--	--	+	*HMGCR* 3'UTR with mutation in miR-29 target site
--	+	--	--	--	miR-375 mimic (negative control)
--	--	+	--	--	siRNA-*HMGCR* (positive control)
--	--	--	+	+	miR-29 mimic

```
5′ AUUAAUACUUCAUUUGGUGCUG   HMGCR
           ||||  |||||||
3′ AUUGGCUAAAGUCUACCACGAU   miR-29a
           ||||  ||||X||
5′ AUUAAUACUUCAUUUGGUCCUG   Mutation
```

Supplementary Figure S4. Effects of miR-29a mimic (10 nM) in HEK293T cells on the activity of *Firefly* (FL) luciferase containing either wild-type or mutated *HMGCR* 3' UTR normalized to *Renilla* luciferase (RL) are shown. Each condition had six replicates. The mutation was targeted to the predicted miR-29 target site. *, $p<0.05$; **, $p<0.01$; p-values were calculated by two-tailed unpaired Student's t-test. Error bars represent standard error of the mean.

Supplementary Figure S5

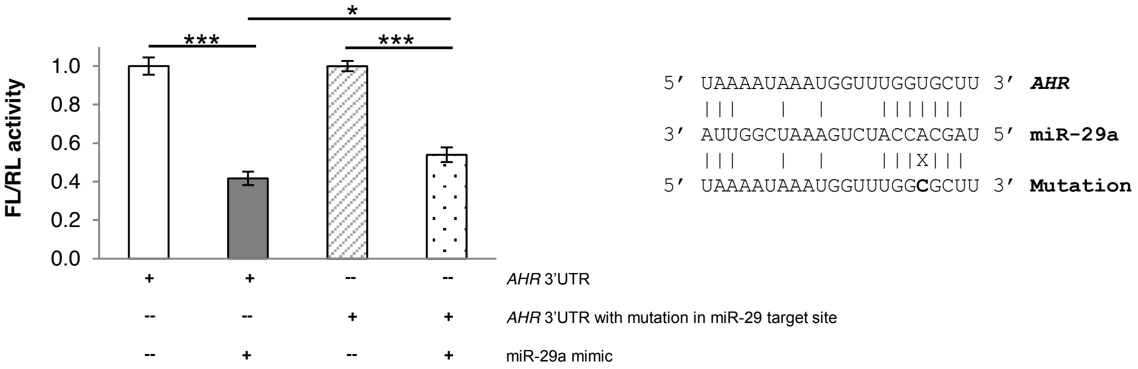

Supplementary Figure S5. Effects of miR-29a mimic (10 nM) in HEK293T cells on the activity of *Firefly* (FL) luciferase containing either wild-type or mutated *Ahr*3' UTR normalized to *Renilla* luciferase (RL) are shown. Each condition had six replicates. The mutation was targeted to the predicted miR-29 target site.

Supplementary Figure S6

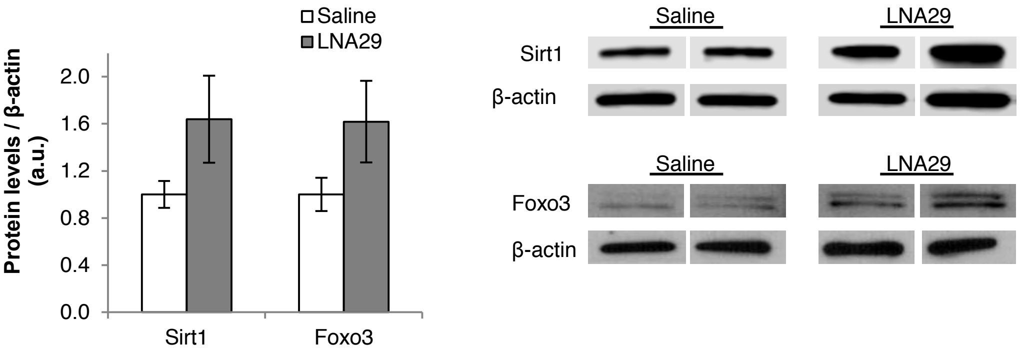

Supplementary Figure S6. Densitometry analyses shown for Sirt1 and Foxo3 in livers from LNA29-treated mice (n=5) compared to saline-treated controls (n=4). β-actin was used as a loading control. Immunoblot results are shown for two representative mice from each treatment group.

Supplementary Table S1. RNA-seq data from livers of LNA29-treated mice compared to saline-treated controls. mRNA fold-change was computed before and after discarding data points ± 1.5 times the standard deviation of the mean within each treatment category and p-values were calculated by one-tailed unpaired Student's t-test. (GEO accession number GSE63493).

Supplementary Table S2. Results of gene ontology (GO) term enrichment analysis using NIH David for down-regulated genes.

Supplementary Table S3. Results of GO term enrichment analysis using Ingenuity Pathway Analysis (IPA) for down-regulated genes.

Supplementary Table S4. Results of GO term enrichment analysis using NIH David for up-regulated genes.

Supplementary Table S5. Results of GO term enrichment analysis using IPA for up-regulated genes.

Supplemental Table S1.

	All data points included		*Data points beyond 1.5*stdev discarded*	
**Gene**	**FC (LNA)**	**P-value**	**FC (LNA)**	**P-value**
Ugt1a7c|394432	53.67886667	0.170446566	1	#DIV/0!
Mfrp|259172	30.9618	0.027620748	30.9618	0.027620748
Plin1|103968	25.99056667	0.170446566	1	#DIV/0!
LOC100302626|100302626	15.58051667	0.000647286	18.49662	1.44412E-06
Nrg1|211323	14.99348333	0.071828993	6.67142	0.056347996
Tff3|21786	14.55460109	0.097047679	9.159665022	0.086529212
Foxs1|14239	14.22866035	0.091083935	12.78472	0.003006972
Dsel|319901	13.96525	0.020563149	9.21114	0.013839142
Raet1a|19368	13.91424387	0.072428782	17.48572668	0.103075306
Zmynd12|332934	13.36296667	0.083414785	6.27946	0.148332518
Tagln3|56370	12.93766667	0.042712222	7.97804	0.066062971
Kirrel3|67703	12.77526667	0.10627979	4.37142	0.148332518
Igf2bp3|140488	12.21705	0.045223458	7.1133	0.055477299
Anln|68743	12.10743333	0.027701966	12.10743333	0.027701966
Serpina3b|271047	11.9414	0.09872422	4.57358	0.148332518
Slc7a14|241919	11.77293333	0.101207638	4.37142	0.148332518
Doc2b|13447	11.74098333	0.008946182	11.74098333	0.008946182
Tnf|21926	11.35551667	0.028231707	11.35551667	0.028231707
Styxl1|76571	11.18705	0.028845884	11.18705	0.028845884
Ncaph|215387	10.8206	0.006501621	10.8206	0.006501621
4921523A10Rik|110332	10.68586667	0.0267355	10.68586667	0.0267355
Zfp786|330301	10.66063333	0.091443403	4.57358	0.148332518
Megf6|230971	10.27116085	0.068154138	12.15304	0.030717181
Ybx2|53422	10.2673	0.170446566	1	#DIV/0!
S100a4|20198	10.2673	0.170446566	1	#DIV/0!
Klrc1|16641	10.2673	0.170446566	1	#DIV/0!
Dpysl5|65254	10.2673	0.170446566	1	#DIV/0!
Sftpd|20390	10.10061667	0.08801451	4.57358	0.148332518
Flrt2|399558	10.09441667	0.030807342	10.09441667	0.030807342
Gm9079|668272	10.09441667	0.030807342	10.09441667	0.030807342
Galnt9|231605	9.992783333	0.030268937	9.992783333	0.030268937
Col8a2|329941	9.7637	0.085905471	4.57358	0.148332518
Trim17|56631	9.638366667	0.170446566	1	#DIV/0!
Adra2a|11551	9.396590242	0.023044665	9.535655785	0.006330263
Grik4|110637	9.297916667	0.072956608	9.297916667	0.072956608
Gm10818|675921	9.0393	0.106322683	3.3	0.148332518
Mmp3|17392	9.0393	0.106322683	3.3	0.148332518
Tex19.1|73679	8.963416667	0.170446566	1	#DIV/0!
Sh2d1a|20400	8.898366667	0.072486194	8.898366667	0.072486194
Wdr65|68625	8.879098973	0.089653732	11.05418	0.127242563
4930583K01Rik|67745	8.872633333	0.028103571	8.872633333	0.028103571
Ccr2|12772	8.872633333	0.028103571	8.872633333	0.028103571
E030003E18Rik|320092	8.872633333	0.028103571	8.872633333	0.028103571
Fxyd7|57780	8.792424343	0.065568512	11.92074	0.087106547
Eme1|268465	8.742005732	0.014355636	28.56858333	0.014727512
Omp|18378	8.704166667	0.027773343	8.704166667	0.027773343
Hoxb8|15416	8.704166667	0.027773343	8.704166667	0.027773343
Fzd3|14365	8.377533333	0.077428357	4.57358	0.148332518
Rnu1b2|19845	8.377533333	0.077428357	4.57358	0.148332518

C4bp-ps1|12270	8.377533333	0.077428357	4.57358	0.148332518
Ptprn|19275	8.377533333	0.077428357	4.57358	0.148332518
Nsun7|70918	8.377533333	0.077428357	4.57358	0.148332518
Chst8|68947	8.377533333	0.077428357	4.57358	0.148332518
Ugt2b38|100559	8.371583333	0.087989736	3.8959	0.148332518
AB099516|554292	8.02055	0.074868905	4.64906	0.148332518
Snhg10|69434	7.977983333	0.075346019	4.57358	0.148332518
Sebox|18292	7.977983333	0.075346019	4.57358	0.148332518
Serp2|72661	7.87635	0.074873498	4.57358	0.148332518
Nanos1|332397	7.87635	0.074873498	4.57358	0.148332518
Bai2|230775	7.87635	0.074873498	4.57358	0.148332518
Galnt3|14425	7.87635	0.074873498	4.57358	0.148332518
Glt8d2|74782	7.87635	0.074873498	4.57358	0.148332518
Fsd2|244091	7.707883333	0.075998742	4.37142	0.148332518
Actl6b|83766	7.707883333	0.075998742	4.37142	0.148332518
Gpr179|217143	7.707883333	0.075998742	4.37142	0.148332518
Muc19|239611	7.707883333	0.075998742	4.37142	0.148332518
Tm4sf5|75604	7.707883333	0.075998742	4.37142	0.148332518
Gm17801|100272221	7.707883333	0.075998742	4.37142	0.148332518
Acot10|64833	7.68265	0.170446566	1	#DIV/0!
B3gat2|280645	7.68265	0.170446566	1	#DIV/0!
Cib3|234421	7.392247626	0.12124896	2.344248986	0.127168618
Raet1c|19370	7.391479404	0.094757776	6.37142	0.173296754
Cyp4a12a|277753	7.34014496	0.013267484	12.27266475	0.015996756
Ngp|18054	7.219879478	0.047297884	10.48472	0.021914722
1700018A04Rik|71307	7.122633333	0.170446566	1	#DIV/0!
4930444G20Rik|114671	7.122633333	0.170446566	1	#DIV/0!
Eomes|13813	7.122633333	0.170446566	1	#DIV/0!
Ush1c|72088	7.122633333	0.170446566	1	#DIV/0!
Gpr20|239530	7.122633333	0.170446566	1	#DIV/0!
Aplnr|23796	7.122633333	0.170446566	1	#DIV/0!
Spert|67926	7.122633333	0.170446566	1	#DIV/0!
Stag3|50878	7.122633333	0.170446566	1	#DIV/0!
Rimklb|108653	7.122633333	0.170446566	1	#DIV/0!
Il22ra2|237310	7.122633333	0.170446566	1	#DIV/0!
Nbea|26422	7.116433333	0.084914106	3.53972	0.148332518
A730069N07Rik|244425	6.955966667	0.072463803	6.955966667	0.072463803
Gpr171|229323	6.955966667	0.072463803	6.955966667	0.072463803
Mrgprf|211577	6.955966667	0.072463803	6.955966667	0.072463803
Sdk1|330222	6.955966667	0.072463803	6.955966667	0.072463803
Kif20a|19348	6.842012658	0.062528462	10.3	0.072944813
Gm2506|100039939	6.839616667	0.170446566	1	#DIV/0!
Mogat2|233549	6.815033333	0.087605735	3.3	0.148332518
Aldh1a2|19378	6.815033333	0.087605735	3.3	0.148332518
A930007I19Rik|77779	6.815033333	0.087605735	3.3	0.148332518
Olfr1396|258334	6.815033333	0.087605735	3.3	0.148332518
1700029J07Rik|69479	6.815033333	0.087605735	3.3	0.148332518
Trim10|19824	6.7875	0.072578363	6.7875	0.072578363
Capn9|73647	6.7875	0.072578363	6.7875	0.072578363
Tspan32|27027	6.7875	0.072578363	6.7875	0.072578363
Efs|13644	6.7875	0.072578363	6.7875	0.072578363
Bex2|12069	6.7875	0.072578363	6.7875	0.072578363
Bdkrb1|12061	6.7875	0.072578363	6.7875	0.072578363

Cox8b|12869	6.7875	0.072578363	6.7875	0.072578363
Faim2|72393	6.7875	0.072578363	6.7875	0.072578363
Cenpp|66336	6.7875	0.072578363	6.7875	0.072578363
Cyp26c1|546726	6.7875	0.072578363	6.7875	0.072578363
Akr1c18|105349	6.7875	0.072578363	6.7875	0.072578363
Ccdc40|207607	6.7875	0.072578363	6.7875	0.072578363
Sdcbp2|228765	6.785716667	0.170446566	1	#DIV/0!
Glipr1|73690	6.69678659	0.028417486	14.42104	0.023999739
Corin|53419	6.597876	0.000495812	27.49115	9.70684E-05
Pcdhga5|93713	6.52515	0.170446566	1	#DIV/0!
Klk1|16612	6.512125025	0.100764034	8.34716	0.173296754
Nfe2l3|18025	6.293632083	0.155787665	3.53972	0.173296754
H19|14955	6.235444534	0.148156953	1.505047411	0.16546411
Hist1h4f|319157	6.17925	0.170446566	1	#DIV/0!
Lamp3|239739	6.163947924	0.003140126	7.346325602	7.66539E-05
Greb1l|381157	6.12896	0.058687724	15.3224	0.055497328
Slc22a21|56517	6.106583333	0.074926697	3.64932	0.148332518
D630023F18Rik|98303	6.094416667	0.076298115	3.53972	0.148332518
Tmem130|243339	6.094416667	0.076298115	3.53972	0.148332518
Slc28a3|114304	6.094416667	0.076298115	3.53972	0.148332518
Mpp4|227157	6.094416667	0.076298115	3.53972	0.148332518
Fam101a|73121	6.094416667	0.076298115	3.53972	0.148332518
Fam163a|329274	6.094416667	0.076298115	3.53972	0.148332518
Liph|239759	6.094416667	0.076298115	3.53972	0.148332518
Xlr|22441	6.094416667	0.076298115	3.53972	0.148332518
D2Ertd750e|51944	6.087967684	0.058974011	15.3224	0.055497328
Zfp947|210853	6.009913333	0.058714121	15.02478333	0.054984817
Slc24a1|214111	5.92595	0.075125027	3.53972	0.148332518
Lif|16878	5.92595	0.075125027	3.53972	0.148332518
Tead4|21679	5.92595	0.075125027	3.53972	0.148332518
Fndc7|320181	5.92595	0.075125027	3.53972	0.148332518
Dlx4|13394	5.89465	0.078731019	3.3	0.148332518
Spink3|20730	5.89465	0.078731019	3.3	0.148332518
A830093I24Rik|207921	5.89465	0.078731019	3.3	0.148332518
C2cd4c|237397	5.89465	0.078731019	3.3	0.148332518
Mkrn3|22652	5.89465	0.078731019	3.3	0.148332518
Serpina3a|74069	5.89465	0.078731019	3.3	0.148332518
Mtnr1a|17773	5.89465	0.078731019	3.3	0.148332518
Hsd17b14|66065	5.89465	0.078731019	3.3	0.148332518
Gm6792|627821	5.89465	0.078731019	3.3	0.148332518
Il17d|239114	5.89465	0.078731019	3.3	0.148332518
Tgm4|331046	5.792683333	0.170446566	1	#DIV/0!
Pf4|56744	5.749159429	0.012325171	11.30235658	0.001592239
Tmem169|271711	5.726183333	0.077237977	3.3	0.148332518
1700007K13Rik|69327	5.726183333	0.077237977	3.3	0.148332518
Lctl|235435	5.726183333	0.077237977	3.3	0.148332518
Foxd3|15221	5.726183333	0.077237977	3.3	0.148332518
Map3k12|26404	5.566265559	0.200525554	-1.071949243	0.45778063
Gm1673|381633	5.469366267	0.01476794	13.76548333	0.01085985
Prrx1|18933	5.39955	0.170446566	1	#DIV/0!
Dpp6|13483	5.39955	0.170446566	1	#DIV/0!
Accn3|171209	5.39955	0.170446566	1	#DIV/0!
Clec4d|17474	5.39955	0.170446566	1	#DIV/0!

2810442I21Rik|72735	5.39955	0.170446566	1	#DIV/0!
Cd209d|170779	5.39955	0.170446566	1	#DIV/0!
Mmp12|17381	5.39955	0.170446566	1	#DIV/0!
Treh|58866	5.381069757	0.003234598	7.552823245	0.003997473
2310014L17Rik|381845	5.36478	0.201621969	1	#DIV/0!
Gm9897|208595	5.272103825	0.029153005	16.07991667	0.020760156
Gzma|14938	5.257386269	0.049053165	5.8273	0.045625723
Hey1|15213	5.222452	0.023625904	16.35616	0.013181761
Gm7030|630294	5.2044	0.170446566	1	#DIV/0!
Fez1|235180	5.0331	0.072795349	5.0331	0.072795349
Spire2|234857	5.0331	0.072795349	5.0331	0.072795349
Hsd3b5|15496	5.0331	0.072795349	5.0331	0.072795349
Gm17769|100045653	5.0331	0.072795349	5.0331	0.072795349
Klrd1|16643	5.0331	0.072795349	5.0331	0.072795349
Jph4|319984	5	0.170446566	1	#DIV/0!
Slc27a6|225579	5	0.170446566	1	#DIV/0!
1190002H23Rik|66214	5	0.170446566	1	#DIV/0!
Papolb|56522	5	0.170446566	1	#DIV/0!
Svep1|64817	4.951013333	0.049326105	12.37753333	0.039912242
Acot5|217698	4.939016667	0.170446566	1	#DIV/0!
Rin1|225870	4.923903572	0.006144895	5.858893822	0.000386475
Pigz|239827	4.922209688	0.133864225	4.57358	0.173296754
Loxhd1|240411	4.898366667	0.170446566	1	#DIV/0!
Sh3rf3|237353	4.898366667	0.170446566	1	#DIV/0!
F2rl2|14064	4.898366667	0.170446566	1	#DIV/0!
H2afy3|67552	4.898366667	0.170446566	1	#DIV/0!
Fscn2|238021	4.898366667	0.170446566	1	#DIV/0!
Prps1l1|75456	4.898366667	0.170446566	1	#DIV/0!
Ccl8|20307	4.898366667	0.170446566	1	#DIV/0!
Twist2|13345	4.898366667	0.170446566	1	#DIV/0!
Hormad2|75828	4.898366667	0.170446566	1	#DIV/0!
Snora78|100306952	4.898366667	0.170446566	1	#DIV/0!
Adra1d|11550	4.898366667	0.170446566	1	#DIV/0!
Aldoart2|79459	4.898366667	0.170446566	1	#DIV/0!
Oprd1|18386	4.898366667	0.170446566	1	#DIV/0!
Gngt1|14699	4.898366667	0.170446566	1	#DIV/0!
Myh4|17884	4.898366667	0.170446566	1	#DIV/0!
Tectb|21684	4.898366667	0.170446566	1	#DIV/0!
Epb4.2|13828	4.898366667	0.170446566	1	#DIV/0!
Sgol1|72415	4.898366667	0.170446566	1	#DIV/0!
1700009J07Rik|75188	4.898366667	0.170446566	1	#DIV/0!
Akap14|434756	4.898366667	0.170446566	1	#DIV/0!
Pcdh9|211712	4.898366667	0.170446566	1	#DIV/0!
Cttnbp2|30785	4.898366667	0.170446566	1	#DIV/0!
Klhl1|93688	4.898366667	0.170446566	1	#DIV/0!
Piwil2|57746	4.898366667	0.170446566	1	#DIV/0!
Cyp2t4|384724	4.898366667	0.170446566	1	#DIV/0!
Ephx3|71932	4.88682	0.073222368	7.1133	0.074643367
Gzmm|16904	4.848751515	0.055770721	10.245	0.023173348
Fam181b|58238	4.799191799	0.016375419	14.09134	0.004938992
Krt79|223917	4.630125042	0.022827453	16.10743333	0.011030825
Rfc4|106344	4.627851966	0.020671872	8.291153225	0.017580808
Gm6498|624367	4.545254967	0.062086415	11.21458333	0.048853

Arg2|11847	4.533372963	0.110976772	6.27946	0.173296754
Trps1|83925	4.451892337	0.023099972	4.451892337	0.023099972
Tyms-ps|22172	4.440437993	0.052949489	10.95596667	0.039448802
Palb2|233826	4.43194	0.153278681	3.53972	0.173296754
5730508B09Rik|70617	4.409484397	0.001711719	7.445940054	5.11865E-05
3930402G23Rik|665306	4.349974098	0.004134554	5.032002078	0.000556693
Serpina1e|20704	4.322861762	0.006285895	6.071916227	0.0064432
Erp27|69187	4.320739805	0.131503083	4.57358	0.173296754
Dnajc27|217378	4.315	0.055263978	10.7875	0.040042786
Rgs9bp|243923	4.312694524	0.054309398	10.85433333	0.039010666
Egfl8|81701	4.278790239	0.034525897	2.924157316	0.038263133
Mcoln2|68279	4.274346667	0.055187707	10.68586667	0.03955673
Serinc4|574418	4.261	0.170446566	1	#DIV/0!
Ptpla|30963	4.232574918	0.00039297	4.232574918	0.00039297
Tcea2|21400	4.217514552	0.065876108	2.788711944	0.129359652
Adam12|11489	4.20981702	0.030482292	4.20981702	0.030482292
Trpv4|63873	4.178378587	0.005437271	4.998059572	0.000103973
Card14|170720	4.1369	0.170446566	1	#DIV/0!
Eml5|319670	4.044816117	0.095580829	2.103182796	0.165860767
Gdf9|14566	4.041389572	0.103883388	9.04946	0.074324407
Gm10451|100041694	4.041238873	0.1140852	3.716963336	0.153115295
Apol7c|108956	4.040883333	0.170446566	1	#DIV/0!
Fam46b|100342	4.040246667	0.13217465	4.57358	0.173296754
Hist2h3b|319154	4.022016667	0.074325894	2.6	0.148332518
Sult2a4|434121	4.009433333	0.170446566	1	#DIV/0!
Hbq1b|544763	3.977983333	0.170446566	1	#DIV/0!
BC066135|271887	3.977983333	0.170446566	1	#DIV/0!
Kctd19|279499	3.977983333	0.170446566	1	#DIV/0!
Snora70|104368	3.977983333	0.170446566	1	#DIV/0!
Tnfaip6|21930	3.977983333	0.170446566	1	#DIV/0!
Serpinb10-ps|241197	3.977983333	0.170446566	1	#DIV/0!
Kif6|319991	3.977983333	0.170446566	1	#DIV/0!
BC055004|381680	3.977983333	0.170446566	1	#DIV/0!
Sdk2|237979	3.977983333	0.170446566	1	#DIV/0!
Plxnb3|140571	3.977983333	0.170446566	1	#DIV/0!
1700008O03Rik|69349	3.977983333	0.170446566	1	#DIV/0!
Qrich2|217341	3.977983333	0.170446566	1	#DIV/0!
Dppa3|73708	3.977983333	0.170446566	1	#DIV/0!
Btnl2|547431	3.977983333	0.170446566	1	#DIV/0!
Gcnt7|654821	3.977983333	0.170446566	1	#DIV/0!
Klra7|16638	3.977983333	0.170446566	1	#DIV/0!
B4galnt2|14422	3.977983333	0.170446566	1	#DIV/0!
Olfr315|258531	3.977983333	0.170446566	1	#DIV/0!
Sectm1b|58210	3.977983333	0.170446566	1	#DIV/0!
Smok2b|236574	3.977983333	0.170446566	1	#DIV/0!
Sim1|20464	3.977983333	0.170446566	1	#DIV/0!
Catsper3|76856	3.977983333	0.170446566	1	#DIV/0!
Gm11567|670533	3.977983333	0.170446566	1	#DIV/0!
Fam71d|70897	3.977983333	0.170446566	1	#DIV/0!
Mctp1|78771	3.977983333	0.170446566	1	#DIV/0!
Ube2t|67196	3.977983333	0.170446566	1	#DIV/0!
Olfr61|18362	3.977983333	0.170446566	1	#DIV/0!
Ccdc85a|216613	3.977983333	0.170446566	1	#DIV/0!

Zfp957|105590	3.977983333	0.170446566	1	#DIV/0!
Lrp8|16975	3.977983333	0.170446566	1	#DIV/0!
Gpr152|269053	3.977983333	0.170446566	1	#DIV/0!
Ydjc|69101	3.977983333	0.170446566	1	#DIV/0!
Zfp133-ps|668917	3.977983333	0.170446566	1	#DIV/0!
Gsdma|57911	3.977983333	0.170446566	1	#DIV/0!
Olfr982|258853	3.977983333	0.170446566	1	#DIV/0!
Gm5089|328479	3.977983333	0.170446566	1	#DIV/0!
Tslp|53603	3.977983333	0.170446566	1	#DIV/0!
4933402N03Rik|233918	3.977983333	0.170446566	1	#DIV/0!
Grik3|14807	3.977983333	0.170446566	1	#DIV/0!
Wnt7a|22421	3.977983333	0.170446566	1	#DIV/0!
LOC100499420|100499420	3.977983333	0.170446566	1	#DIV/0!
Frmd5|228564	3.977983333	0.170446566	1	#DIV/0!
Pgr|18667	3.977983333	0.170446566	1	#DIV/0!
Il1rl2|107527	3.977983333	0.170446566	1	#DIV/0!
Cxcl5|20311	3.977983333	0.170446566	1	#DIV/0!
Gad2|14417	3.977983333	0.170446566	1	#DIV/0!
Cr2|12902	3.977983333	0.170446566	1	#DIV/0!
Unc80|329178	3.977983333	0.170446566	1	#DIV/0!
Slc44a5|242259	3.977983333	0.170446566	1	#DIV/0!
Atp8a2|50769	3.977983333	0.170446566	1	#DIV/0!
BC048609|433215	3.977983333	0.170446566	1	#DIV/0!
Ppp1r1a|58200	3.977983333	0.170446566	1	#DIV/0!
Tnip3|414084	3.977983333	0.170446566	1	#DIV/0!
Pcdh10|18526	3.977983333	0.170446566	1	#DIV/0!
Gm4984|245347	3.977983333	0.170446566	1	#DIV/0!
9530059O14Rik|319626	3.977983333	0.170446566	1	#DIV/0!
Dmrtc1b|632708	3.977983333	0.170446566	1	#DIV/0!
BC061212|381724	3.977983333	0.170446566	1	#DIV/0!
1700001O22Rik|73598	3.977983333	0.170446566	1	#DIV/0!
Kcna7|16495	3.977983333	0.170446566	1	#DIV/0!
Gpr37l1|171469	3.977983333	0.170446566	1	#DIV/0!
Sostdc1|66042	3.977983333	0.170446566	1	#DIV/0!
Slc1a3|20512	3.977983333	0.170446566	1	#DIV/0!
Dnajb7|57755	3.977983333	0.170446566	1	#DIV/0!
Usp13|72607	3.977983333	0.170446566	1	#DIV/0!
Ppp1r42|69312	3.977983333	0.170446566	1	#DIV/0!
4930567H17Rik|619303	3.977983333	0.170446566	1	#DIV/0!
Cldn27|546519	3.977983333	0.170446566	1	#DIV/0!
Cmya5|76469	3.977983333	0.170446566	1	#DIV/0!
Gm15645|626055	3.977983333	0.170446566	1	#DIV/0!
Fancb|237211	3.977983333	0.170446566	1	#DIV/0!
9530077C05Rik|68283	3.977983333	0.170446566	1	#DIV/0!
Efhb|211482	3.977983333	0.170446566	1	#DIV/0!
D830046C22Rik|320197	3.977983333	0.170446566	1	#DIV/0!
Tbx10|109575	3.977983333	0.170446566	1	#DIV/0!
Timp4|110595	3.977983333	0.170446566	1	#DIV/0!
Fhl2|14200	3.977983333	0.170446566	1	#DIV/0!
Gpr119|236781	3.977983333	0.170446566	1	#DIV/0!
Vmn1r4|171194	3.977983333	0.170446566	1	#DIV/0!
Ifltd1|74071	3.977983333	0.170446566	1	#DIV/0!
Siah3|380918	3.977983333	0.170446566	1	#DIV/0!

Slfnl1|194219	3.977983333	0.170446566	1	#DIV/0!
Tmem89|69384	3.977983333	0.170446566	1	#DIV/0!
Lrrtm4|243499	3.977983333	0.170446566	1	#DIV/0!
Vmn1r53|113853	3.977983333	0.170446566	1	#DIV/0!
Cox4i2|84682	3.977983333	0.170446566	1	#DIV/0!
Hoxc4|15423	3.977983333	0.170446566	1	#DIV/0!
Dio2|13371	3.977983333	0.170446566	1	#DIV/0!
Dkkl1|50722	3.977983333	0.170446566	1	#DIV/0!
Aldh1l2|216188	3.977983333	0.170446566	1	#DIV/0!
Tmem8c|66139	3.977983333	0.170446566	1	#DIV/0!
Stk32a|269019	3.977983333	0.170446566	1	#DIV/0!
1700013N18Rik|73318	3.977983333	0.170446566	1	#DIV/0!
Kif5c|16574	3.977983333	0.170446566	1	#DIV/0!
A630055G03Rik|223970	3.977983333	0.170446566	1	#DIV/0!
Ccl7|20306	3.977983333	0.170446566	1	#DIV/0!
Pcdh20|219257	3.977983333	0.170446566	1	#DIV/0!
Htr1a|15550	3.977983333	0.170446566	1	#DIV/0!
Hist1h4n|319161	3.977983333	0.170446566	1	#DIV/0!
Taar5|215854	3.977983333	0.170446566	1	#DIV/0!
Kcnh2|16511	3.977983333	0.170446566	1	#DIV/0!
Omg|18377	3.977983333	0.170446566	1	#DIV/0!
4921511H03Rik|70920	3.977983333	0.170446566	1	#DIV/0!
Zic4|22774	3.977983333	0.170446566	1	#DIV/0!
Gm10857|100038358	3.977983333	0.170446566	1	#DIV/0!
Klf8|245671	3.977983333	0.170446566	1	#DIV/0!
Ncr1|17086	3.977983333	0.170446566	1	#DIV/0!
Ccr1|12768	3.977983333	0.170446566	1	#DIV/0!
Sult6b1|73671	3.977983333	0.170446566	1	#DIV/0!
Tbx1|21380	3.977983333	0.170446566	1	#DIV/0!
Adra2c|11553	3.977983333	0.170446566	1	#DIV/0!
Dnahc8|13417	3.977983333	0.170446566	1	#DIV/0!
Pdcd1lg2|58205	3.977983333	0.170446566	1	#DIV/0!
Dner|227325	3.977983333	0.170446566	1	#DIV/0!
Dusp27|240892	3.977983333	0.170446566	1	#DIV/0!
Bean1|65115	3.977983333	0.170446566	1	#DIV/0!
Galnt12|230145	3.977983333	0.170446566	1	#DIV/0!
Ankrd5|319196	3.977983333	0.170446566	1	#DIV/0!
Arid3c|550619	3.977983333	0.170446566	1	#DIV/0!
Ano9|71345	3.977983333	0.170446566	1	#DIV/0!
Rnft2|269695	3.977983333	0.170446566	1	#DIV/0!
Olfr855|258517	3.977983333	0.170446566	1	#DIV/0!
Ldlrad1|546840	3.977983333	0.170446566	1	#DIV/0!
Ltf|17002	3.977983333	0.170446566	1	#DIV/0!
Fbxo47|72973	3.977983333	0.170446566	1	#DIV/0!
Adcyap1|11516	3.977983333	0.170446566	1	#DIV/0!
Rhov|228543	3.977983333	0.170446566	1	#DIV/0!
AA388235|433100	3.977983333	0.170446566	1	#DIV/0!
Arrdc5|76920	3.977983333	0.170446566	1	#DIV/0!
Lin7b|22342	3.977983333	0.170446566	1	#DIV/0!
Ttc9|69480	3.977983333	0.170446566	1	#DIV/0!
Scn2a1|110876	3.977983333	0.170446566	1	#DIV/0!
Trim59|66949	3.977983333	0.170446566	1	#DIV/0!
Peg3as|100169889	3.977983333	0.170446566	1	#DIV/0!

Foxc2|14234	3.977983333	0.170446566	1	#DIV/0!
Nlrp9a|233001	3.977983333	0.170446566	1	#DIV/0!
Fsd1|240121	3.977983333	0.170446566	1	#DIV/0!
Reg4|67709	3.977983333	0.170446566	1	#DIV/0!
100043387|100043387	3.977983333	0.170446566	1	#DIV/0!
Nlrp4c|83564	3.977983333	0.170446566	1	#DIV/0!
Arsj|271970	3.977983333	0.170446566	1	#DIV/0!
Tmem202|73893	3.977983333	0.170446566	1	#DIV/0!
Tdh|58865	3.977983333	0.170446566	1	#DIV/0!
Hbq1a|216635	3.95793676	0.05601716	9.765483333	0.036994814
Dusp8|18218	3.953980146	0.104222197	1.994151827	0.194282453
Btg3|12228	3.942333205	0.000479978	5.122307458	0.000469775
H1fx|243529	3.933848241	0.037838335	2.753817983	0.054902851
Trem2|83433	3.906196823	0.010622187	3.906196823	0.010622187
Ly6c1|17067	3.901658182	0.224331247	1	#DIV/0!
Oscp1|230751	3.891007218	0.067938325	9.793016667	0.046113179
AA465934|613254	3.880067545	0.057767295	9.765483333	0.036994814
4930534B04Rik|75216	3.879358983	0.132757893	4.57358	0.173296754
Kif2c|73804	3.814045427	0.159242754	3.3	0.173296754
Olfr1467|258686	3.809516667	0.170446566	1	#DIV/0!
Lce1a1|67127	3.809516667	0.170446566	1	#DIV/0!
Vmn1r-ps79|100042966	3.809516667	0.170446566	1	#DIV/0!
D10Bwg1379e|215821	3.809516667	0.170446566	1	#DIV/0!
Ppp1r3d|228966	3.809516667	0.170446566	1	#DIV/0!
Ccl2|20296	3.809516667	0.170446566	1	#DIV/0!
Abcg4|192663	3.809516667	0.170446566	1	#DIV/0!
4930479M11Rik|74927	3.809516667	0.170446566	1	#DIV/0!
Olfr826|258671	3.809516667	0.170446566	1	#DIV/0!
Hcn4|330953	3.809516667	0.170446566	1	#DIV/0!
Hells|15201	3.809516667	0.170446566	1	#DIV/0!
Zfp287|170740	3.809516667	0.170446566	1	#DIV/0!
Frmpd4|333605	3.809516667	0.170446566	1	#DIV/0!
Jsrp1|71912	3.809516667	0.170446566	1	#DIV/0!
Serpina5|268591	3.809516667	0.170446566	1	#DIV/0!
Syngr4|58867	3.809516667	0.170446566	1	#DIV/0!
Ctsj|26898	3.809516667	0.170446566	1	#DIV/0!
4931417G12Rik|70979	3.809516667	0.170446566	1	#DIV/0!
Mypn|68802	3.809516667	0.170446566	1	#DIV/0!
Tnnc2|21925	3.809516667	0.170446566	1	#DIV/0!
Gm8579|667335	3.809516667	0.170446566	1	#DIV/0!
Cacna1f|54652	3.809516667	0.170446566	1	#DIV/0!
Vmn2r7|319217	3.809516667	0.170446566	1	#DIV/0!
Syce1|74075	3.809516667	0.170446566	1	#DIV/0!
Fbxo24|71176	3.809516667	0.170446566	1	#DIV/0!
Slc25a43|194744	3.809516667	0.170446566	1	#DIV/0!
2610017I09Rik|66297	3.809516667	0.170446566	1	#DIV/0!
Cenpf|108000	3.809516667	0.170446566	1	#DIV/0!
Aloxe3|23801	3.809516667	0.170446566	1	#DIV/0!
Pea15b|231332	3.809516667	0.170446566	1	#DIV/0!
Sgpp2|433323	3.809516667	0.170446566	1	#DIV/0!
Vmn2r96|433070	3.809516667	0.170446566	1	#DIV/0!
Lrrc18|67580	3.809516667	0.170446566	1	#DIV/0!
Gm8979|668108	3.809516667	0.170446566	1	#DIV/0!

Synb|239167	3.809516667	0.170446566	1	#DIV/0!
Mapk8ip2|60597	3.809516667	0.170446566	1	#DIV/0!
4932435O22Rik|319819	3.809516667	0.170446566	1	#DIV/0!
Slc34a1|20505	3.809516667	0.170446566	1	#DIV/0!
Slc6a20a|102680	3.809516667	0.170446566	1	#DIV/0!
Ntrk3|18213	3.809516667	0.170446566	1	#DIV/0!
Gpr34|23890	3.809516667	0.170446566	1	#DIV/0!
Cort|12854	3.809516667	0.170446566	1	#DIV/0!
Olfr1101|258584	3.809516667	0.170446566	1	#DIV/0!
Dlk1|13386	3.809516667	0.170446566	1	#DIV/0!
Gm7244|638580	3.809516667	0.170446566	1	#DIV/0!
Psca|72373	3.809516667	0.170446566	1	#DIV/0!
Car6|12353	3.809516667	0.170446566	1	#DIV/0!
Hsf5|327992	3.809516667	0.170446566	1	#DIV/0!
Gcm1|14531	3.809516667	0.170446566	1	#DIV/0!
Zfp473|243963	3.809516667	0.170446566	1	#DIV/0!
Ggt1|14598	3.809516667	0.170446566	1	#DIV/0!
Col22a1|69700	3.809516667	0.170446566	1	#DIV/0!
Spink4|20731	3.809516667	0.170446566	1	#DIV/0!
Tmem178|68027	3.809516667	0.170446566	1	#DIV/0!
Scn8a|20273	3.809516667	0.170446566	1	#DIV/0!
Gm13272|545648	3.809516667	0.170446566	1	#DIV/0!
Ccdc114|211535	3.74053955	0.133059501	3.042145947	0.16339816
Tesc|57816	3.739736748	0.081289166	2.163928773	0.137345333
Eln|13717	3.732224404	0.000206267	4.773052933	1.31312E-05
Stk30|26448	3.724305455	0.036369637	12.8023	0.012602066
Ppm1j|71887	3.723786667	0.0892605	8.21776	0.082854467
Nlrp10|244202	3.695626667	0.153034325	3.53972	0.173296754
Tigd3|332359	3.679888608	0.103722969	3.743778571	0.152352456
Usp50|75083	3.677031053	0.063669074	9.0724	0.039654813
1700101E01Rik|329375	3.62896	0.065032455	9.0724	0.039654813
Rad51|19361	3.608751748	0.064586353	8.903933333	0.039321972
9130206I24Rik|100040736	3.602540606	0.181963452	3.53972	0.173296754
Gpr126|215798	3.60237998	0.107316187	1.966139026	0.220917165
Slc10a6|75750	3.578797089	0.031720463	3.578797089	0.031720463
Trim16|94092	3.572586667	0.078605036	5.83972	0.070910138
Folr4|64931	3.56998223	0.075316679	3.56998223	0.075316679
Ccl19|24047	3.562291018	0.221815908	1	#DIV/0!
4930515G01Rik|67642	3.559346667	0.124033453	8.898366667	0.093248005
Ptplad2|66775	3.537752467	0.066745558	8.903933333	0.039321972
Gm10584|100043682	3.527786597	0.068048998	8.704166667	0.040875093
Tril|66873	3.469709374	0.017839765	8.405071429	0.000482755
Fam107a|268709	3.467252671	0.027858882	3.467252671	0.027858882
Hoxb6|15414	3.45838024	0.070329402	8.704166667	0.040875093
Col7a1|12836	3.453945497	0.16456407	5.67804	0.173296754
Zfp61|22719	3.403052924	0.028505725	4.565971429	0.012902258
A630033H20Rik|213438	3.358268	0.050822861	13.99278333	0.013272229
Apba2|11784	3.351013333	0.134555301	4.57358	0.173296754
BC096441|619441	3.349358879	0.057839439	3.349358879	0.057839439
Tubd1|56427	3.330075502	0.024834983	2.397835075	0.01236922
Klrb1c|17059	3.284361056	0.111964723	7.945	0.070678788
Wdr78|242584	3.26166479	0.138938281	4.37142	0.173296754
Tmem181d-ps|100040596	3.258824427	0.074217586	7.1151	0.045293991

Atg9b|213948	3.258726585	0.124863189	5.9435	0.070665653
C030039L03Rik|112415	3.258227066	0.160057873	6.27946	0.173296754
9430083A17Rik|77428	3.245143218	0.160934118	2.093696815	0.231256853
Mup13|100039089	3.233678952	0.188798813	-1.014766578	0.482895366
Slc8a2|110891	3.233465506	0.134933322	4.57358	0.173296754
Bcl2a1b|12045	3.214148479	0.062690338	4.610871595	0.030394878
Myo7b|17922	3.206109091	0.152221766	5.67804	0.173296754
Gm996|381353	3.192273658	0.135622345	4.57358	0.173296754
Pih1d2|72614	3.192273658	0.135622345	4.57358	0.173296754
Ubxn10|212190	3.192273658	0.135622345	4.57358	0.173296754
Fbn1|14118	3.191467001	0.007400191	3.041204771	0.005698293
Plxna3|18846	3.191193333	0.136762611	4.57358	0.173296754
Kcne4|57814	3.191193333	0.136762611	4.57358	0.173296754
Hmcn1|545370	3.187190303	0.08779567	10.95596667	0.039448802
Plod2|26432	3.185324	0.060376656	13.27218333	0.015336703
B430212C06Rik|338360	3.179370151	0.038929486	3.179370151	0.038929486
Rnf165|225743	3.17658871	0.039046076	3.17658871	0.039046076
Fgf12|14167	3.169668957	0.058078679	11.90945	0.015344684
Cx3cr1|13051	3.146303205	0.057393187	12.90393333	0.012958419
Oscar|232790	3.133335403	0.180252483	4.37142	0.173296754
Lrrc48|74665	3.129468247	0.138466691	4.57358	0.173296754
2610203C20Rik|100042464	3.129468247	0.138466691	4.57358	0.173296754
Ldoc1l|223732	3.123806667	0.140198227	4.37142	0.173296754
Ube2ql1|76980	3.12244934	0.00421758	5.925245764	0.000141973
Ogdhl|239017	3.116433333	0.170446566	1	#DIV/0!
Dlgap5|218977	3.116433333	0.170446566	1	#DIV/0!
Tcp10b|21462	3.116433333	0.170446566	1	#DIV/0!
Zfp184|193452	3.116433333	0.170446566	1	#DIV/0!
Atcay|16467	3.116433333	0.170446566	1	#DIV/0!
Clcnka|12733	3.116433333	0.170446566	1	#DIV/0!
Nap1l5|58243	3.116433333	0.170446566	1	#DIV/0!
Sun3|194974	3.116433333	0.170446566	1	#DIV/0!
Efcab6|77627	3.116433333	0.170446566	1	#DIV/0!
Efcab7|230500	3.116433333	0.170446566	1	#DIV/0!
Gapt|238875	3.116433333	0.170446566	1	#DIV/0!
Fbxo27|233040	3.116433333	0.170446566	1	#DIV/0!
Gm5477|432986	3.116433333	0.170446566	1	#DIV/0!
Cidea|12683	3.116433333	0.170446566	1	#DIV/0!
Zpbp|53604	3.116433333	0.170446566	1	#DIV/0!
Gm5124|331392	3.116433333	0.170446566	1	#DIV/0!
2310061N02Rik|69661	3.116433333	0.170446566	1	#DIV/0!
Gm14812|100038584	3.116433333	0.170446566	1	#DIV/0!
Megf11|214058	3.116433333	0.170446566	1	#DIV/0!
Tctex1d4|242646	3.116433333	0.170446566	1	#DIV/0!
Sstr3|20607	3.116433333	0.170446566	1	#DIV/0!
Pde6h|78600	3.116433333	0.170446566	1	#DIV/0!
Nr5a1|26423	3.116433333	0.170446566	1	#DIV/0!
Sertad4|214791	3.116433333	0.170446566	1	#DIV/0!
Ush2a|22283	3.116433333	0.170446566	1	#DIV/0!
4930407I10Rik|328573	3.116433333	0.170446566	1	#DIV/0!
1700001G17Rik|67503	3.116433333	0.170446566	1	#DIV/0!
Csf3|12985	3.116433333	0.170446566	1	#DIV/0!
Klk1b5|16622	3.116433333	0.170446566	1	#DIV/0!

4933400F21Rik|74403	3.116433333	0.170446566	1	#DIV/0!
Cplx4|225644	3.116433333	0.170446566	1	#DIV/0!
Tg|21819	3.116433333	0.170446566	1	#DIV/0!
Scn3a|20269	3.116433333	0.170446566	1	#DIV/0!
Gm1123|382097	3.116433333	0.170446566	1	#DIV/0!
Adh6a|69117	3.116433333	0.170446566	1	#DIV/0!
Prokr1|58182	3.116433333	0.170446566	1	#DIV/0!
Zfp365|216049	3.116433333	0.170446566	1	#DIV/0!
Ncrna00086|320237	3.116433333	0.170446566	1	#DIV/0!
AU018829|100041253	3.116433333	0.170446566	1	#DIV/0!
0610038B21Rik|70345	3.116433333	0.170446566	1	#DIV/0!
Asb17|66772	3.116433333	0.170446566	1	#DIV/0!
Rgs8|67792	3.116433333	0.170446566	1	#DIV/0!
Prh1|19131	3.116433333	0.170446566	1	#DIV/0!
Ifng|15978	3.116433333	0.170446566	1	#DIV/0!
Chi3l3|12655	3.116433333	0.170446566	1	#DIV/0!
Gm5460|432838	3.116433333	0.170446566	1	#DIV/0!
Cftr|12638	3.116433333	0.170446566	1	#DIV/0!
Gjb5|14622	3.116433333	0.170446566	1	#DIV/0!
Jph3|57340	3.116433333	0.170446566	1	#DIV/0!
Mrgprd|211578	3.116433333	0.170446566	1	#DIV/0!
Tuba3b|22147	3.116433333	0.170446566	1	#DIV/0!
F630043A04Rik|219114	3.116433333	0.170446566	1	#DIV/0!
Upk1b|22268	3.116433333	0.170446566	1	#DIV/0!
Ptger4|19219	3.116433333	0.170446566	1	#DIV/0!
Hsd3b4|15495	3.116433333	0.170446566	1	#DIV/0!
Taf9b|407786	3.116433333	0.170446566	1	#DIV/0!
Egr4|13656	3.116433333	0.170446566	1	#DIV/0!
Pdxk-ps|435518	3.116433333	0.170446566	1	#DIV/0!
9230110C19Rik|234912	3.116433333	0.170446566	1	#DIV/0!
Gpr52|620246	3.116433333	0.170446566	1	#DIV/0!
Slco5a1|240726	3.116433333	0.170446566	1	#DIV/0!
Veph1|72789	3.116433333	0.170446566	1	#DIV/0!
Nptx1|18164	3.116433333	0.170446566	1	#DIV/0!
Cav3|12391	3.116433333	0.170446566	1	#DIV/0!
Olfr1383|404337	3.116433333	0.170446566	1	#DIV/0!
Tc2n|74413	3.116433333	0.170446566	1	#DIV/0!
Celf5|319586	3.116433333	0.170446566	1	#DIV/0!
Wfdc6a|209351	3.116433333	0.170446566	1	#DIV/0!
Krtap17-1|77914	3.116433333	0.170446566	1	#DIV/0!
Adamts6|108154	3.116433333	0.170446566	1	#DIV/0!
Ptpn5|19259	3.116433333	0.170446566	1	#DIV/0!
Ska1|66468	3.116433333	0.170446566	1	#DIV/0!
Sh2d1b1|26904	3.116433333	0.170446566	1	#DIV/0!
Eid3|66341	3.116433333	0.170446566	1	#DIV/0!
Pcdha4-g|100384868	3.116433333	0.170446566	1	#DIV/0!
Gria4|14802	3.116433333	0.170446566	1	#DIV/0!
Otos|260301	3.116433333	0.170446566	1	#DIV/0!
Shc4|271849	3.116433333	0.170446566	1	#DIV/0!
Orc1|18392	3.116433333	0.170446566	1	#DIV/0!
A530088E08Rik|664702	3.116433333	0.170446566	1	#DIV/0!
Cd40|21939	3.114281061	0.061686419	3.114281061	0.061686419
Fam69b|56279	3.110410578	0.051552391	10.8206	0.011663991

Cml5|69049	3.110410578	0.051552391	10.8206	0.011663991
Amn|93835	3.109956831	0.041910654	3.886828467	0.024940117
Lyz1|17110	3.106509142	0.012594078	3.131760142	0.001449907
Lrrc15|74488	3.102382993	0.007713636	3.102382993	0.007713636
Fam83a|239463	3.101565146	0.020391283	3.691088418	0.002810987
Socs4|67296	3.096277143	0.068824264	18.06161667	0.013357098
A630001G21Rik|319997	3.082959213	0.194284497	-1.134788439	0.440829738
Zfp3|193043	3.078359612	0.025967824	3.078359612	0.025967824
Ranbp17|66011	3.07306	0.233338847	1	#DIV/0!
4930529C04Rik|619677	3.071681119	0.092905816	10.68586667	0.03955673
Gpr137b-ps|664862	3.062543314	0.016703881	5.365907656	0.00598711
Rg9mtd2|108943	3.061880171	0.004318888	2.571188616	0.00311099
Srl|106393	3.056113854	0.082256813	3.056113854	0.082256813
Ccdc28b|66264	3.052034456	0.00213483	2.592036127	0.000686924
Phlda3|27280	3.045634396	0.00114223	3.590575392	0.001265224
Serpina4-ps1|321018	3.035772862	0.058421123	3.035772862	0.058421123
Chrdl2|69121	3.026028196	0.014789388	3.026028196	0.014789388
Lysmd2|70082	3.001957091	0.158372339	2.4065875	0.148212279
Nrip1|268903	3.000609945	0.057647865	5.784507143	0.007629883
Dlec1|320256	2.973988596	0.117939438	1.674490167	0.251025789
Ahsg|11625	2.96829376	4.35996E-07	2.727464196	1.33065E-07
Defb1|13214	2.96477255	8.99996E-06	3.159219059	2.23542E-05
Cyp1a1|13076	2.962906367	0.020700109	6.241014184	0.00037111
Mfap2|17150	2.95313632	0.052099699	2.16180782	0.098348538
Ptar1|72351	2.937747381	0.131848884	6.91114	0.073393406
2010300C02Rik|72097	2.933210314	0.165946787	5.67804	0.173296754
Col3a1|12825	2.926735039	0.001165837	3.201547913	0.00184233
Mef2b|17259	2.916666667	0.170446566	1	#DIV/0!
4930422G04Rik|71643	2.916666667	0.170446566	1	#DIV/0!
Cxcl2|20310	2.916666667	0.170446566	1	#DIV/0!
Clec5a|23845	2.916666667	0.170446566	1	#DIV/0!
Gpr161|240888	2.916666667	0.170446566	1	#DIV/0!
Ifitm6|213002	2.916666667	0.170446566	1	#DIV/0!
Slmo1|225655	2.916666667	0.170446566	1	#DIV/0!
Dach1|13134	2.916666667	0.170446566	1	#DIV/0!
Hist3h2bb-ps|382522	2.916666667	0.170446566	1	#DIV/0!
Gja6|414089	2.916666667	0.170446566	1	#DIV/0!
Atf7ip2|75329	2.916666667	0.170446566	1	#DIV/0!
Idi2|320581	2.916666667	0.170446566	1	#DIV/0!
Actn3|11474	2.916666667	0.170446566	1	#DIV/0!
Prr7|432763	2.916666667	0.170446566	1	#DIV/0!
Gm12060|100134990	2.916666667	0.170446566	1	#DIV/0!
Slit2|20563	2.916666667	0.170446566	1	#DIV/0!
5730590G19Rik|77011	2.916666667	0.170446566	1	#DIV/0!
A2m|232345	2.916666667	0.170446566	1	#DIV/0!
Hrh1|15465	2.916666667	0.170446566	1	#DIV/0!
Gldn|235379	2.916666667	0.170446566	1	#DIV/0!
Gpr61|229714	2.916666667	0.170446566	1	#DIV/0!
Pbk|52033	2.916666667	0.170446566	1	#DIV/0!
Gm14351|668960	2.916666667	0.170446566	1	#DIV/0!
Kif18b|70218	2.916666667	0.170446566	1	#DIV/0!
Gm10416|667213	2.916666667	0.170446566	1	#DIV/0!
Wdr52|212517	2.916666667	0.170446566	1	#DIV/0!

D7Ertd443e|71007	2.916666667	0.170446566	1	#DIV/0!
9930111J21Rik1|667214	2.916666667	0.170446566	1	#DIV/0!
Calb1|12307	2.916666667	0.170446566	1	#DIV/0!
5830432E09Rik|67765	2.916666667	0.170446566	1	#DIV/0!
Smcp|17235	2.916666667	0.170446566	1	#DIV/0!
Apol8|239552	2.916666667	0.170446566	1	#DIV/0!
Adamtsl3|269959	2.916666667	0.170446566	1	#DIV/0!
Eddm3b|219026	2.916666667	0.170446566	1	#DIV/0!
Pou2f3|18988	2.916666667	0.170446566	1	#DIV/0!
Stc1|20855	2.916666667	0.170446566	1	#DIV/0!
H2-M5|240095	2.916666667	0.170446566	1	#DIV/0!
Syt4|20983	2.916666667	0.170446566	1	#DIV/0!
Wfdc13|408190	2.916666667	0.170446566	1	#DIV/0!
1700012A16Rik|71836	2.916666667	0.170446566	1	#DIV/0!
Itga2|16398	2.916666667	0.170446566	1	#DIV/0!
Unc5cl|76589	2.916666667	0.170446566	1	#DIV/0!
Gdf3|14562	2.916666667	0.170446566	1	#DIV/0!
Fam163b|109349	2.916666667	0.170446566	1	#DIV/0!
Lrfn1|80749	2.916666667	0.170446566	1	#DIV/0!
A230020J21Rik|100125339	2.916666667	0.170446566	1	#DIV/0!
Cacna1g|12291	2.916666667	0.170446566	1	#DIV/0!
Scara5|71145	2.916666667	0.170446566	1	#DIV/0!
Rab44|442827	2.916666667	0.170446566	1	#DIV/0!
9030418K01Rik|71532	2.916666667	0.170446566	1	#DIV/0!
Gjd3|353155	2.916666667	0.170446566	1	#DIV/0!
Ccdc109b|66815	2.916666667	0.170446566	1	#DIV/0!
Cldnd2|74276	2.916666667	0.170446566	1	#DIV/0!
Fam78b|226610	2.916666667	0.170446566	1	#DIV/0!
Ddx4|13206	2.916666667	0.170446566	1	#DIV/0!
Tmeff1|230157	2.916666667	0.170446566	1	#DIV/0!
Trim15|69097	2.916666667	0.170446566	1	#DIV/0!
Myoz1|59011	2.916666667	0.170446566	1	#DIV/0!
Galntl1|108760	2.916666667	0.170446566	1	#DIV/0!
Cacnb2|12296	2.916666667	0.170446566	1	#DIV/0!
Sfmbt2|353282	2.916666667	0.170446566	1	#DIV/0!
Gm16432|545391	2.916666667	0.170446566	1	#DIV/0!
Defb25|654459	2.916666667	0.170446566	1	#DIV/0!
Dsn1|66934	2.916666667	0.170446566	1	#DIV/0!
Kif15|209737	2.916666667	0.170446566	1	#DIV/0!
Gnrh1|14714	2.916666667	0.170446566	1	#DIV/0!
AU015228|99169	2.916666667	0.170446566	1	#DIV/0!
Pla2g2c|18781	2.916666667	0.170446566	1	#DIV/0!
Cdc7|12545	2.916666667	0.170446566	1	#DIV/0!
Stard6|170461	2.916666667	0.170446566	1	#DIV/0!
Utf1|22286	2.916666667	0.170446566	1	#DIV/0!
Rhag|19743	2.916666667	0.170446566	1	#DIV/0!
Gm11744|100038570	2.89224	0.155528256	3.67672	0.173296754
Lrrn3|16981	2.892152542	0.174223354	4.57358	0.173296754
Cyp21a1|13079	2.886793345	0.238328751	1	#DIV/0!
Mmp8|17394	2.886793345	0.238328751	1	#DIV/0!
Snora31|100303751	2.886793345	0.238328751	1	#DIV/0!
Sparc|20692	2.885448988	4.41793E-06	2.620785849	3.50602E-06
Stk36|269209	2.884148	0.136456126	6.87358	0.077234609

Aph1c|68318	2.880644935	0.035167643	3.954855645	0.028004916
E130317F20Rik|414101	2.87607034	0.076939028	2.87607034	0.076939028
Gm10684|100038468	2.85715	0.170446566	1	#DIV/0!
Nkain1|67149	2.855254079	0.052514697	5.812821429	0.021483219
Kcnab1|16497	2.849053333	0.240100451	1	#DIV/0!
Gm5936|546325	2.843088646	0.157812218	3.53972	0.173296754
Pde10a|23984	2.825459122	0.051299627	5.305119048	0.023535745
Il28ra|242700	2.824352419	0.054691734	4.961628017	0.029557463
Ephb2|13844	2.814005471	0.143554243	2.522267924	0.170579472
Atp4a|11944	2.807114454	0.102657133	9.765483333	0.036994814
Gm6578|625347	2.800028446	0.134958428	1.465737987	0.299106435
Gpr84|80910	2.798276035	0.110663236	3.275507143	0.137461249
Casp1|12362	2.788220077	0.023163397	2.788220077	0.023163397
Mt3|17751	2.787153169	0.161241959	3.53972	0.173296754
Tnfrsf22|79202	2.782386667	0.148760575	6.955966667	0.093223255
Kbtbd12|74589	2.782386667	0.148760575	6.955966667	0.093223255
Kcne3|57442	2.776091341	0.161058636	8.14716	0.070556641
Sytl2|83671	2.761524321	0.131665198	1.651708384	0.282826441
Lefty1|13590	2.757599408	0.108682563	4.84436092	0.075835431
5830433M19Rik|67770	2.756399218	0.02196407	2.759672917	0.020297306
2200002K05Rik|69137	2.750964273	0.149609968	6.7875	0.093349877
Ttc25|74407	2.750964273	0.149609968	6.7875	0.093349877
Slc7a15|328059	2.75024149	0.242980184	1	#DIV/0!
Gimap7|231932	2.739827501	0.225849817	1.282144901	0.403677687
Ntf3|18205	2.734465105	0.029969832	3.268667731	0.004514089
Slc30a3|22784	2.733228473	0.023936667	2.14232367	0.032594979
Lpl|16956	2.729160359	0.073642501	1.683789133	0.053204821
H2-Eb2|381091	2.726013333	0.167391575	3.3	0.173296754
Adcy1|432530	2.722279169	0.075215789	2.671746911	0.091718873
Ifi30|65972	2.720342849	0.001301376	3.2154996	0.001158145
Zfp82|330502	2.719985586	0.221490726	4.57358	0.173296754
Prx|19153	2.716398112	0.097732526	2.716398112	0.097732526
Taf7|24074	2.716266644	0.00011605	2.478310032	3.61116E-05
Carf|241066	2.715	0.152057578	6.7875	0.093349877
1700065D16Rik|73410	2.714286667	0.244880741	1	#DIV/0!
Adamts15|235130	2.713404498	0.018645576	2.285541019	0.038020753
Sypl2|17306	2.696841269	0.15332937	6.7875	0.093349877
Klhl35|72184	2.696132706	0.245863496	1	#DIV/0!
Gm8898|667962	2.690618563	0.056787405	3.5559811	0.04845082
Cacnb1|12295	2.679555487	0.091596511	3.729437629	0.072405744
Tspan2|70747	2.677213483	0.064172469	2.677213483	0.064172469
Zfp52|22710	2.675963394	0.062832648	5.447815476	0.024025977
Rn4.5s|19799	2.665493324	0.026808233	2.04960401	0.031553492
C1qtnf4|67445	2.660307122	0.032223216	4.057709175	0.016528429
2410066E13Rik|68235	2.655794567	0.199970755	3.53972	0.173296754
Fabp7|12140	2.649646748	0.013715606	2.155434307	0.01496086
Wisp2|22403	2.644901703	0.061070498	3.892293061	0.042641762
U05342|664779	2.641694657	0.129703502	14.40573333	0.037388319
Zfp57|22715	2.629614545	0.204910708	3.3	0.173296754
Ppic|19038	2.624083473	0.000730393	2.393000155	0.001017908
H2-Q2|15013	2.614588584	0.192793783	2.056679634	0.287773807
Tmem44|224090	2.61363711	0.004929071	3.907382947	0.000674506
Cdkl5|382253	2.612532873	0.037228385	6.325299372	0.000342126

1110038B12Rik|68763	2.608550791	0.031137125	2.434561288	0.00023327
Rgs20|58175	2.607539394	0.260997899	1	#DIV/0!
Ebf1|13591	2.607539394	0.260997899	1	#DIV/0!
C530028O21Rik|319352	2.603105145	0.007893786	4.147511331	0.00019383
Rab38|72433	2.59640355	0.064304296	4.599236461	0.030617495
Pira1|18722	2.587116252	0.263497363	-2.83174	0.173296754
Kcnj2|16518	2.5851103	0.128882411	2.8375	0.148931563
Tmsb15l|399591	2.575704309	0.162584865	5.83972	0.070910138
Jazf1|231986	2.569056268	0.132724472	2.889777527	0.16314115
Fabp5|16592	2.568420609	0.056266577	2.474011539	0.051193519
Cgref1|68567	2.560854185	0.083559063	1.618917549	0.126633563
Trpm5|56843	2.552982248	0.098723944	3.785941828	0.074465341
Kiss1|280287	2.55060734	0.142920574	2.486665807	0.141944192
Tmem181b-ps|547127	2.550544856	0.082579195	2.919312071	0.049069066
Kcnj15|16516	2.549113815	0.061040819	2.549113815	0.061040819
Gdf15|23886	2.534032693	0.049764334	1.610965677	0.124762153
Csl|71832	2.522788917	0.102748231	5.316067941	0.047644272
Hrk|12123	2.517914875	0.148064975	15.42055	0.041646676
Hist1h2ae|319166	2.517222222	0.187010324	10.57233333	0.093237772
Fxyd2|11936	2.515759198	0.119865831	16.8085	0.017611408
Mup11|100039028	2.504386307	0.017166586	3.994525553	8.30727E-05
1700012L04Rik|76383	2.503493125	0.189577433	1.101858099	0.45752294
Fam105a|223433	2.502444843	0.054944891	4.636983879	0.018121944
Ms4a7|109225	2.499942648	0.032945982	4.242399851	0.001695931
Fam132a|67389	2.499170484	0.095842674	4.981951187	0.044457051
Slc44a4|70129	2.493300172	0.073993218	3.669193055	0.050312798
Cyp4a12b|13118	2.487083939	0.115145375	2.487083939	0.115145375
Tes|21753	2.480663244	0.011677828	1.765747224	0.032827429
4632427E13Rik|666737	2.477653967	0.117495446	4.388884949	0.072624033
Clec9a|232414	2.476312705	0.115210454	2.476312705	0.115210454
Neurl1a|18011	2.475003873	0.120469272	1.458446522	0.255313708
Snord17|100313519	2.470058566	0.168893134	3.53972	0.173296754
4933408B17Rik|271508	2.470058566	0.168893134	3.53972	0.173296754
Zc3h6|78751	2.459397275	0.017606776	3.493962232	0.000354053
Kcnj9|16524	2.45895342	0.2149004	3.53972	0.173296754
Grrp1|72690	2.45071285	0.088983273	2.45071285	0.088983273
Wdyhv1|76773	2.45048315	0.030045549	2.458419579	0.006298747
Ier3|15937	2.449283445	0.168039384	3.856576765	0.15707777
Marco|17167	2.448560981	0.065451299	1.96459241	0.002722507
Fstl3|83554	2.447920618	0.153745599	2.790359268	0.128213591
Purg|75029	2.441730619	0.0206954	2.441730619	0.0206954
Slc4a11|269356	2.437766667	0.171835685	3.53972	0.173296754
Zbtb34|241311	2.43607593	0.00139803	3.11929472	0.000310128
Npas2|18143	2.429386104	0.006446645	2.839820784	0.005785473
Cldn7|53624	2.428781476	0.078067856	4.98608485	0.025165007
Amz1|231842	2.420937789	0.028185563	3.473544971	0.002086567
Nrp|654309	2.420696195	0.153312894	4.067889431	0.110381005
1700020L24Rik|66330	2.419481903	0.229625126	-1.010193328	0.495713255
Adcy3|104111	2.416598239	0.107172252	3.198618768	0.084668453
Lmod2|93677	2.412125424	0.210610801	4.37142	0.173296754
Mup10|100039008	2.406679378	0.199801202	5.488748047	0.132379071
Chga|12652	2.405786044	0.222351506	3.3	0.173296754
Gm10394|100039227	2.405666667	0.170446566	1	#DIV/0!

Bcl2a1a|12044	2.405666667	0.170446566	1	#DIV/0!
Duxbl|278672	2.405666667	0.170446566	1	#DIV/0!
Mettl7a2|393082	2.403699306	0.103703211	4.614924264	0.04929297
Kbtbd8|243574	2.401779261	0.17221504	3.53972	0.173296754
6330439K17Rik|241688	2.393088571	0.16512883	9.25162	0.073125351
2610524H06Rik|330173	2.391606674	0.156448133	2.365584001	0.182575382
Itk|16428	2.389093415	0.176790463	3.3	0.173296754
Il23a|83430	2.389093415	0.176790463	3.3	0.173296754
Myo1h|231646	2.384175508	0.148828838	2.189757812	0.087448342
Prkcc|18752	2.380221654	0.215488643	1.583400343	0.341557484
2610028H24Rik|76964	2.380013328	0.200710039	1	#DIV/0!
Tuba8|53857	2.373244561	0.006330817	2.01214985	0.004296846
Klhdc7b|546648	2.371216878	0.124900356	4.181386905	0.074733521
Atad5|237877	2.36928393	0.21925944	3.53972	0.173296754
Gdpd1|66569	2.368936556	0.01537315	1.995356884	0.033111036
Snrpn|20646	2.364964752	0.147948258	2.364964752	0.147948258
Etv1|14009	2.35786	0.179890944	3.3	0.173296754
B4galnt4|330671	2.35786	0.179890944	3.3	0.173296754
Cbr2|12409	2.35786	0.179890944	3.3	0.173296754
Eif4ebp3|108112	2.356717872	0.023755013	2.791353807	0.001791607
Abca14|67928	2.35452619	0.176928091	3.53972	0.173296754
Fbxo2|230904	2.351226258	0.062046805	1.982912292	0.105577688
Lingo4|320747	2.347597785	0.113520347	2.347597785	0.113520347
Hbegf|15200	2.343443295	0.035762657	2.343443295	0.035762657
Cenpw|66311	2.342471633	0.003615987	2.342471633	0.003615987
Fam54a|71804	2.342089928	0.181499927	3.3	0.173296754
Rnase10|75019	2.342089928	0.181499927	3.3	0.173296754
Vsig2|57276	2.341056373	0.085639457	2.341056373	0.085639457
Gpnmb|93695	2.339928941	0.013389833	2.339928941	0.013389833
1700102P08Rik|112418	2.338355483	0.158242336	2.576554452	0.166099216
A130049A11Rik|100125931	2.336128512	0.087195606	1.680992437	0.179410848
6720463M24Rik|77744	2.33049697	0.1434905	8.011083333	0.041592481
Sytl3|83672	2.33049697	0.1434905	8.011083333	0.041592481
Mex3a|72640	2.324930561	0.148713002	9.765483333	0.036994814
Tbx6|21389	2.324510461	0.120082481	2.324510461	0.120082481
Cdkn1a|12575	2.320331616	0.000574149	1.949242025	0.000531049
2810055G20Rik|77994	2.316591184	0.14872368	8.704166667	0.040875093
Il20rb|213208	2.313923531	0.07686541	1.636863269	0.146560101
Ranbp3l|223332	2.303770277	0.076673611	4.829592357	0.002861308
Gtf3c3|98488	2.303446842	0.083332353	6.555103707	0.001905055
Cend1|57754	2.303238698	0.039209414	3.074171835	0.024170728
Cd300lh|382551	2.30296	0.130123369	5.7574	0.045879649
Hsd17b1|15485	2.302807455	0.146948012	8.011083333	0.041592481
Elfn1|243312	2.301900721	0.172488393	2.185849682	0.229040358
Mmp2|17390	2.301516312	0.013846197	1.901963627	0.013607713
Gpcpd1|74182	2.300666261	0.007197504	2.088738822	0.016033855
Zfp28|22690	2.300061582	0.093168148	4.635274094	0.030909972
Ccne1|12447	2.294696164	0.063483626	1.731490523	0.12432269
Slc25a18|71803	2.291301818	0.1993985	4.57358	0.173296754
Galr2|14428	2.291301818	0.1993985	4.57358	0.173296754
Plch2|269615	2.291301818	0.1993985	4.57358	0.173296754
Il13ra2|16165	2.290473333	0.183544433	3.3	0.173296754
Gm3852|100042453	2.290473333	0.183544433	3.3	0.173296754

Ppfia2|327814	2.290473333	0.183544433	3.3	0.173296754
Gucy2c|14917	2.287314907	0.18338723	2.73911337	0.199024953
Ttll10|330010	2.279457836	0.292458552	1	#DIV/0!
Nfasc|269116	2.27529771	0.066768958	2.716264474	0.019677593
Mycn|18109	2.275153963	0.18524447	3.3	0.173296754
Ksr2|333050	2.275153963	0.18524447	3.3	0.173296754
Gpr25|383563	2.275153963	0.18524447	3.3	0.173296754
Senp8|71599	2.274579417	0.052543614	5.05030933	0.002852452
1700120K04Rik|68232	2.273524777	0.239636379	1.282144901	0.403677687
Rnf122|68867	2.266490667	0.030923588	2.142051757	0.021544319
Rims2|116838	2.264077995	0.202427117	4.57358	0.173296754
Gm5176|382421	2.264077995	0.202427117	4.57358	0.173296754
9230114K14Rik|414108	2.261164196	0.117924066	2.637930861	0.112832362
Cxcr6|80901	2.257673417	0.1981054	2.22660095	0.276424976
Kcnk1|16525	2.251261348	0.140423763	1.471957116	0.29708192
Fbp2|14120	2.244866574	0.205786281	4.37142	0.173296754
B3galtl|381694	2.242353424	0.047203915	2.242353424	0.047203915
Cxcr4|12767	2.242131018	0.047688181	1.682774246	0.067909936
Fgf21|56636	2.22043946	0.04010768	1.667096634	0.035509088
Dnmt3b|13436	2.218283759	0.004065788	2.39869549	0.001770362
Zfpm2|22762	2.216210946	0.219369102	-1.134788439	0.440829738
Prex2|109294	2.216160381	0.031667241	2.840056922	0.019698144
Bambi-ps1|81913	2.215651799	0.208060746	4.37142	0.173296754
Fam83c|71405	2.215269076	0.191416965	2.215269076	0.191416965
Zcchc3|67917	2.211327179	0.068548783	3.51551047	0.027762777
Plaur|18793	2.20964514	0.111383424	1.442741632	0.233355605
Mup12|100039054	2.206363097	0.103068227	4.678077427	0.028472374
Slc25a14|20523	2.203809886	0.043488831	2.211106646	0.011587813
Cd28|12487	2.2	0.290356397	1	#DIV/0!
Gab3|210710	2.199665627	0.203988044	1.678513561	0.28897435
Myo5a|17918	2.193968215	0.133069761	2.193968215	0.133069761
Chrna1|11435	2.193518709	0.22487356	-1.202217172	0.412618198
Plk1|18817	2.193265644	0.042079419	2.006262235	0.079761356
Gm684|270157	2.191152743	0.179439119	2.085614286	0.20345464
Rpph1|85029	2.190796733	0.191297962	2.254167548	0.083917159
Cyp3a11|13112	2.190212042	0.005157967	2.215739564	0.000901156
Sdsl|257635	2.189867706	0.000895364	1.781001307	0.001648827
Ctsk|13038	2.1886308	0.076316176	1.582914356	0.146353588
Gm1078|381835	2.188430076	0.270704681	1	#DIV/0!
Gm505|244666	2.181196811	0.163145159	2.536228187	0.180227025
Ammecr1|56068	2.179437443	0.158589641	1.337812817	0.344524566
Mmp7|17393	2.17570462	0.133472163	5.59481597	0.035771861
Arhgap15|76117	2.17332039	0.096493798	3.8645	0.03645035
Pycr1|209027	2.166478569	0.227538243	-1.217222222	0.407029201
Cdk1|12534	2.165065669	0.086906427	2.165065669	0.086906427
Nexn|68810	2.164040602	0.165239983	2.164040602	0.165239983
Tnfrsf23|79201	2.163673492	0.075533076	5.093762175	0.001129622
Ccdc38|237465	2.160493827	0.277241136	1	#DIV/0!
Nlrp1b|637515	2.15982	0.273363112	1	#DIV/0!
Ccnb2|12442	2.15982	0.273363112	1	#DIV/0!
Fstl1|14314	2.158412251	0.014354335	2.489197952	0.000942111
Kiss1r|114229	2.156574601	0.055970663	1.685628806	0.108641563
Adh7|11529	2.15563204	0.041322894	1.666914956	0.057967775

C1qtnf6|72709	2.153554844	0.008752276	2.133333625	0.006360016
Fst|14313	2.151105344	0.025004802	1.800066572	0.052063672
Pkp1|18772	2.145374479	0.274736836	1	#DIV/0!
Kif22|110033	2.141172487	0.121649122	2.141172487	0.121649122
Esm1|71690	2.139378613	0.195786709	1.135800217	0.439334996
Rnf222|320040	2.136554483	0.233161467	12.90835	0.093342877
Fcgbp|215384	2.135513426	0.295738231	-7.752983333	0.04363352
Scn4b|399548	2.13300482	0.059606122	2.701086705	0.00984032
Aatk|11302	2.129745309	0.008068342	1.596354982	0.013828644
Sh3kbp1|58194	2.129603398	0.087769011	3.289617313	0.04192803
Lepr|16847	2.128331766	0.039013422	3.035956208	0.013679005
Sult1e1|20860	2.127358373	0.17107164	3.71340082	0.075478342
Apoa4|11808	2.125675491	0.103542294	1.670077371	0.103075619
Hspa2|15512	2.123844273	0.026330353	1.716730735	0.032847113
Ncapg2|76044	2.12331939	0.220639957	4.57358	0.173296754
Car7|12354	2.119539183	0.054550945	3.71744624	0.00765789
6030429G01Rik|436022	2.118490596	0.223417656	8.898366667	0.093248005
Kbtbd3|69149	2.116930925	0.113248914	3.029013053	0.074474039
Snhg9|73474	2.114605088	0.179845494	2.522267924	0.170579472
2700046G09Rik|67188	2.114009353	0.003430975	2.467344101	0.001959975
BC088983|382010	2.11247683	0.007568708	2.855743599	8.71347E-05
Sag|20215	2.111635179	0.044670144	1.711331436	0.086461645
Ly9|17085	2.104024403	0.006610341	1.941218448	0.014717327
Nat1|17960	2.103318255	0.109114508	3.980420992	0.035292797
2310001K24Rik|69517	2.100965072	0.176137696	2.100965072	0.176137696
Srms|20811	2.099102744	0.196719596	1.885227492	0.252057711
Suv39h2|64707	2.0968603	0.156879055	2.055258092	0.209773586
Pxdn|69675	2.096142662	0.025354505	2.096142662	0.025354505
4930539E08Rik|207819	2.091802609	0.118760139	3.563647335	0.054359203
Gm3435|100041621	2.084896128	0.079441105	2.084896128	0.079441105
Fam161b|217705	2.08411204	0.184317777	2.540464286	0.169047969
Penk|18619	2.083733982	0.216684144	2.083733982	0.216684144
Igsf9|93842	2.077954955	0.153287471	1.880524014	0.189903834
Cx3cl1|20312	2.072067748	0.046512821	2.230650532	0.039428076
Tmem22|245020	2.072038788	0.294164169	1	#DIV/0!
Vgll3|73569	2.072038788	0.294164169	1	#DIV/0!
BC030867|217216	2.070856564	0.131677896	1.374335034	0.285401788
Dbf4|27214	2.065471429	0.218208113	6.91114	0.073393406
Ing4|28019	2.06198126	3.26359E-06	2.149322203	6.7935E-06
Layn|244864	2.061058945	0.128787189	4.360292172	0.036716707
Npb|208990	2.059326037	0.230012729	1.656626026	0.330587367
Tubb2a|22151	2.059242621	0.000272987	1.898604309	0.000121069
Mir715|751530	2.055241209	0.298133654	-2.8202	0.173296754
Tmem171|380863	2.048947682	0.238583655	6.67142	0.075644577
Syt5|53420	2.047420112	0.296653541	1	#DIV/0!
Tppp3|67971	2.046611324	0.152725	7.887068562	0.014687605
Rasgrf2|19418	2.043561543	0.109577893	2.043561543	0.109577893
Gm885|380732	2.040923904	0.148479768	8.185476568	0.010104572
Fhit|14198	2.040243189	0.022333956	2.625529333	0.001719268
A530064D06Rik|328830	2.039908403	0.202163233	5.0331	0.093589634
Avil|11567	2.039908403	0.202163233	5.0331	0.093589634
Itgae|16407	2.038743861	0.232333225	1.621721864	0.33676256
Prom2|192212	2.037492431	0.166085961	3.579326525	0.091893115

Myh7b|668940	2.035443888	0.173272009	1.273215927	0.369574783
Phf11|219131	2.031596688	0.219401834	3.072675697	0.141835809
Dmrt2|226049	2.027466274	0.167836552	3.60514881	0.091113317
Fam46c|74645	2.027073129	0.011325296	1.694444822	0.023649417
Rec8|56739	2.024150909	0.093601779	1.870833456	0.036245303
Zfp783|232785	2.023553939	0.225551501	6.955966667	0.093223255
Hcfc2|67933	2.022887974	0.154570388	1.230196262	0.352308462
Rps6ka3|110651	2.019317778	0.291320956	3.53972	0.173296754
Stk17b|98267	2.019103495	0.063565696	1.570647002	0.123664554
Zfp354c|30944	2.01324	0.20631898	5.0331	0.093589634
Fgfbp1|14181	2.01324	0.20631898	5.0331	0.093589634
Igfbp2|16008	2.011410149	0.00054602	2.038432205	0.000242094
Psmc3ip|19183	2.007461557	0.14951805	3.698758657	0.065622006
Card9|332579	2.006558598	0.113488165	2.223777971	0.072977175
Ltc4s|17001	2.00462337	0.130683874	1.328223571	0.285396401
5031414D18Rik|271221	2.003003439	0.089832033	1.595068474	0.181975171
Ly6a|110454	2.001552185	0.001163746	1.846822786	0.001629888
Ncam2|17968	1.994377827	0.157329603	1.994377827	0.157329603
Tfrc|22042	1.993922385	0.028426766	3.456360381	0.000034163
Epn3|71889	1.99310854	0.147972076	1.99310854	0.147972076
Slc37a1|224674	1.992835384	0.033178872	1.992835384	0.033178872
Dgkh|380921	1.991124477	0.164149999	1.991124477	0.164149999
Ap1m2|11768	1.990635056	0.235634918	1.656626026	0.330587367
Prom1|19126	1.989109759	0.249775977	-1.400394585	0.339887238
40977|216438	1.98906558	0.115531066	1.98906558	0.115531066
Myof|226101	1.988642512	0.120980629	1.954657622	0.07924522
AY512931|434310	1.98691199	0.173929923	3.519590809	0.093837856
Dnali1|75563	1.985301171	0.286197212	1	#DIV/0!
Steap1|70358	1.985301171	0.286197212	1	#DIV/0!
Ubd|24108	1.985301171	0.286197212	1	#DIV/0!
Lcn13|227627	1.985301171	0.286197212	1	#DIV/0!
Lta|16992	1.985301171	0.286197212	1	#DIV/0!
BC051142|407788	1.985301171	0.286197212	1	#DIV/0!
Lysmd4|75099	1.980849033	0.012742687	2.274520969	0.000380822
Mcts2|66405	1.980704072	0.012181779	2.49941426	0.003333406
Cyp2b10|13088	1.97760177	0.020226082	2.25797696	0.002420754
Pitx3|18742	1.974545455	0.231988449	6.7875	0.093349877
Spata6|67946	1.974026667	0.302735013	1	#DIV/0!
4632434I11Rik|74041	1.968463151	0.220449069	1.101858099	0.45752294
E130309D14Rik|432582	1.968454166	0.158798979	1.968454166	0.158798979
Rassf1|56289	1.967644374	0.006453285	2.09898358	0.000544821
Rbp1|19659	1.967350955	0.058643857	1.914304262	0.030184113
Tab3|66724	1.967215378	0.01890877	1.686124017	0.030737548
Smpd3|58994	1.961678463	0.077461557	2.61305197	0.044159231
Wdr96|70806	1.96083565	0.220136118	1.138603377	0.444164115
Brip1|237911	1.960420074	0.168104463	1.960420074	0.168104463
4930538K18Rik|75180	1.960302262	0.178886823	3.472454672	0.096192094
BC060267|212516	1.959346667	0.289264215	1	#DIV/0!
Spink5|72432	1.959346667	0.289264215	1	#DIV/0!
Dyx1c1|67685	1.959346667	0.289264215	1	#DIV/0!
Baalc|118452	1.959346667	0.289264215	1	#DIV/0!
Gm8580|667338	1.959346667	0.289264215	1	#DIV/0!
Ccl11|20292	1.959346667	0.289264215	1	#DIV/0!

Dnase1|13419	1.959346667	0.289264215	1	#DIV/0!
Fut7|14347	1.958999665	0.246712071	3.3	0.173296754
Trim36|28105	1.958999665	0.246712071	3.3	0.173296754
Zfp658|210104	1.953071829	0.175935369	1.150887417	0.405805975
3110007F17Rik|73061	1.951085134	0.235807493	6.7875	0.093349877
Ints10|70885	1.950490713	0.096660172	1.950490713	0.096660172
Dzip3|224170	1.9483722	0.155525754	2.019641377	0.144590241
Muc4|140474	1.946241971	0.290847976	1	#DIV/0!
Itm2a|16431	1.946241971	0.290847976	1	#DIV/0!
Gm13275|545652	1.946241971	0.290847976	1	#DIV/0!
Pdzd4|245469	1.944437603	0.179571152	1.944437603	0.179571152
Gpr137b|83924	1.942829125	0.209940218	1.023981074	0.486887059
Slc9a5|277973	1.94175242	0.070019944	2.305650535	0.015335482
Creb3l4|78284	1.938031143	0.188170772	3.905680448	0.086032803
Ier5l|72500	1.937245201	0.118341906	3.268101674	0.041409704
Jmjd5|77035	1.93668277	0.013257348	2.266295749	0.007863698
Hspb1|15507	1.93544959	0.028378217	2.184202953	0.024927151
9130017N09Rik|78906	1.933503171	0.27032728	-1.809413362	0.226028814
Vpreb1|22362	1.933268044	0.247043309	1.656626026	0.330587367
A330035P11Rik|319727	1.928883645	0.007868311	1.656124792	0.003106526
Marveld3|73608	1.927876176	0.095452585	2.262623317	0.044965343
Eid1|58521	1.925679661	0.019519075	1.925679661	0.019519075
Ugt1a6a|94284	1.923739013	0.210679737	-1.025878318	0.453827192
Gadd45b|17873	1.92305936	0.002641501	2.065628744	0.00053833
Itga10|213119	1.922457896	0.110645728	1.391465011	0.233856321
Trove2|20822	1.922422757	0.112451801	1.922422757	0.112451801
Tal1|21349	1.922113627	0.166705323	5.06877381	0.032065754
Capn3|12335	1.916248261	0.133938064	2.870671244	0.07220323
Il1a|16175	1.916180134	0.108370733	1.820487034	0.111036953
Apol7b|278679	1.916037164	0.25914363	1	#DIV/0!
Fam198b|68659	1.912613584	0.304682484	-4.016833333	0.093227909
Txk|22165	1.912227017	0.208392139	7.842616667	0.040301615
Cidec|14311	1.910994211	0.141016799	2.047605433	0.053903212
Runx1|12394	1.905478734	0.032548491	2.567618806	0.000690233
Hspa1b|15511	1.904566786	0.091060947	2.269347998	0.074324716
Sh3tc2|225608	1.903617393	0.074881546	3.390054844	0.001362515
Glt25d2|269132	1.902621674	0.077536435	2.257293357	0.01892472
Lysmd1|217779	1.901497049	5.40958E-05	2.02109471	8.67866E-07
Fosb|14282	1.900778191	0.14426159	1.394162681	0.290810988
Ddit4|74747	1.899115409	0.053630163	1.523151917	0.099826314
Tmem54|66260	1.895670226	0.313548122	1	#DIV/0!
Cnksr1|194231	1.894918071	0.241377687	-1.174476973	0.420024314
Zfp846|244721	1.892908801	0.178794118	1.892908801	0.178794118
Tspan13|66109	1.892362268	0.114526768	1.126562206	0.376045493
Fanca|14087	1.892337384	0.150358689	1.892337384	0.150358689
Col8a1|12837	1.890470678	0.231884769	-1.162638987	0.41190955
Ddah2|51793	1.890232859	0.013703879	1.675390751	0.024971637
Dcdc2c|68511	1.888041275	0.096070096	1.888041275	0.096070096
5730528L13Rik|66665	1.887852815	0.266293326	-1.426573059	0.352378067
5430405H02Rik|74487	1.887684593	0.061937661	1.217042356	0.195139733
Igfbp1|16006	1.886098867	0.130745046	1.925998776	0.103697721
Siglech|233274	1.885254977	0.12510092	2.764166053	0.064647616
Tbx21|57765	1.883833924	0.120575076	1.595428175	0.189013465

Klf7|93691	1.882202326	0.14337754	3.192546191	0.056981832
Myo9a|270163	1.880144888	0.089675214	2.247580254	0.022733228
Cyp2d12|380997	1.873223624	0.085914719	1.955564114	0.064015483
Uchl1|22223	1.86793095	0.265184327	1.282144901	0.403677687
Cacna2d1|12293	1.867489037	0.239825307	3.320684524	0.155017867
1700084E18Rik|67350	1.867032963	0.128763403	1.867032963	0.128763403
Tmsb15b1|666244	1.866970666	0.260477861	3.53972	0.173296754
A530016L24Rik|319942	1.862740498	0.197405026	3.497505952	0.095363911
Ehhadh|74147	1.861911261	0.05347085	2.178873156	0.040674574
B430010I23Rik|78849	1.860139241	0.30187567	1	#DIV/0!
Dusp18|75219	1.851312328	0.254394939	6.955966667	0.093223255
Kcna6|16494	1.851312328	0.254394939	6.955966667	0.093223255
4932412H11Rik|242838	1.850062673	0.083122889	1.525256151	0.168033467
Zfp97|22759	1.849137617	0.115846707	2.684457243	0.036792832
Spsb4|211949	1.845932937	0.16121437	-1.103316447	0.330148753
Tmprss7|208171	1.843836	0.322338549	1	#DIV/0!
Mcm6|17219	1.843785888	0.008928312	1.700296016	0.019195677
Jhdm1d|338523	1.840651077	0.004745942	2.187984517	0.000968336
Skp2|27401	1.839848893	0.2147503	1.510407341	0.324601155
Tet3|194388	1.83638884	0.023662505	2.006477848	0.010864762
Dclre1c|227525	1.833769191	0.042059703	2.683141734	0.001654843
Lig4|319583	1.832544396	0.10940121	2.681593502	0.012429051
Rufy4|435626	1.832047627	0.135357138	1.832047627	0.135357138
Cd63|12512	1.830288376	0.027903065	1.886615748	0.036277974
Ecm2|407800	1.829758324	0.133563277	1.269051753	0.292224207
Clec3b|21922	1.828548408	0.258785711	7.1133	0.074643367
Nid2|18074	1.827234954	0.090477552	1.455004478	0.186254313
Med23|70208	1.82661756	0.028617177	2.519072422	0.002459056
Ttc32|75516	1.826507951	0.005107483	1.938736061	0.00292119
Cldn15|60363	1.819787048	0.215306112	3.618330576	0.10102568
Gm14207|100043609	1.819718321	0.142478868	1.269700358	0.309313476
Parp16|214424	1.816580892	0.011615038	1.569294186	0.009061054
Snhg6|73824	1.814799478	0.01040376	1.408684724	0.022457994
Rcan3|53902	1.814753172	0.162299917	1.18458007	0.365432505
Zfp273|212569	1.814209841	0.246917908	3.223135008	0.156688344
4931428F04Rik|74356	1.813231469	0.162219399	2.429793257	0.112989565
Prss57|73106	1.81110758	0.324261626	1	#DIV/0!
Zfp605|675812	1.809934083	0.183687906	1.809934083	0.183687906
Arl6|56297	1.807544058	0.154461078	1.807544058	0.154461078
Cyp2d9|13105	1.806977275	0.047225227	1.650674848	0.055279471
Tnfrsf18|21936	1.806636712	0.200313113	1.18929412	0.403510819
Psrc1|56742	1.806475366	0.261786747	6.7875	0.093349877
Ccdc19|71870	1.806475366	0.261786747	6.7875	0.093349877
Fam64a|109212	1.806475366	0.261786747	6.7875	0.093349877
Spnb4|80297	1.806475366	0.261786747	6.7875	0.093349877
Hspb11|72938	1.805956502	0.211816892	1.83139214	0.184075273
Edn1|13614	1.805903505	0.088316047	1.182982474	0.302553007
Col6a1|12833	1.80427886	0.001882042	1.802201697	0.000717476
Pik3cb|74769	1.800031909	0.006084966	1.54867738	0.010268405
Ovol1|18426	1.799334436	0.185076784	1.240252903	0.371501171
Zfp821|75871	1.798982278	0.126234781	1.222081006	0.270544159
Itgax|16411	1.798884543	0.225862309	1.812305732	0.263474441
Epc2|227867	1.797572484	0.011232787	1.414889956	0.037499412

Kcnj5|16521	1.795327656	0.316423918	-3.967316667	0.09324139
Lrrc8c|100604	1.793547681	0.06385905	1.183135955	0.208922724
Mansc1|67729	1.792748372	0.077417935	2.812322306	0.002762728
Ppp1r2-ps3|546723	1.792222246	0.217668718	1.154511099	0.426935518
Ephb3|13845	1.791502058	0.11700592	1.791502058	0.11700592
Chka|12660	1.790557051	0.064567217	1.429717899	0.020162251
Ctnnbip1|67087	1.788661109	0.005185969	1.81772115	0.000586067
Pkia|18767	1.787999197	0.274366994	1.264185714	0.40907038
Sult1d1|53315	1.787167947	0.008315686	1.912031992	8.36347E-05
A330023F24Rik|320977	1.786004684	0.071770901	2.921065285	0.004705172
Ccna2|12428	1.785497074	0.157127453	2.176844107	0.127815782
Stx1a|20907	1.784800511	0.172700259	1.703271258	0.125853994
Msc|17681	1.784682105	0.250591835	-1.066288814	0.470534859
4930544O15Rik|75173	1.783573733	0.261874253	-1.075198276	0.470223483
Mpzl1|68481	1.78306785	0.006096781	2.201680587	4.36302E-05
Efemp1|216616	1.780261558	0.076158226	2.130996759	0.009842697
Itga8|241226	1.779802277	0.182514258	-1.089058757	0.37677266
Pkib|18768	1.779258376	0.069126935	1.63781005	0.02260723
Slc25a35|71998	1.77760003	0.12834542	1.77760003	0.12834542
Galnt11|231050	1.777344437	0.034871659	1.484838724	0.072119937
Efna2|13637	1.777182487	0.272033416	1.621721864	0.33676256
Ear2|13587	1.775864738	0.131925283	1.775864738	0.131925283
Oxct1|67041	1.774319356	0.100657983	1.367954468	0.210139847
Fut2|14344	1.772921212	0.26552988	3.53972	0.173296754
B930059L03Rik|319786	1.772921212	0.26552988	3.53972	0.173296754
Pdgfd|71785	1.772921212	0.26552988	3.53972	0.173296754
Stat4|20849	1.772921212	0.26552988	3.53972	0.173296754
Gm17821|100316870	1.772290507	0.153902129	1.173255876	0.351135792
Ric3|320360	1.77193439	0.273114192	1.633421429	0.334663434
Hsd17b13|243168	1.770711074	0.001423228	1.58321934	0.001835554
Agap2|216439	1.76854733	0.046418039	1.476148479	0.087568903
Hic2|58180	1.768246968	0.01706697	2.436033584	0.000120424
Slc5a3|53881	1.767457338	0.105301734	2.197988533	0.025066395
Gata3|14462	1.766820878	0.228575886	10.8206	0.011663991
2410006H16Rik|69221	1.765657507	0.077881156	1.562107498	0.015306015
Pcdhga1|93709	1.763475608	0.326873436	1	#DIV/0!
Col5a1|12831	1.762894969	0.008834558	1.934678399	0.000133289
Btg2|12227	1.762318164	0.144433555	2.017209207	0.009939599
2610035D17Rik|72386	1.760992284	0.074786241	3.148286877	0.000611846
Lmod3|320502	1.760917905	0.144112669	1.912093547	0.123041915
Slc12a8|171286	1.757751544	0.171115474	1.146381847	0.387961247
Nudt21|68219	1.757170488	0.015460604	1.735276099	0.00141261
H2-Ob|15002	1.756074819	0.213104934	1.672705826	0.268647481
Angptl7|654812	1.753784568	0.161531694	2.794105545	0.068038367
Cenph|26886	1.751856465	0.269870644	3.53972	0.173296754
2610020H08Rik|434234	1.74864438	0.150037751	1.74864438	0.150037751
Fbxl16|214931	1.748510801	0.167672588	3.685770001	0.008344521
Cd209b|69165	1.747952614	0.318935971	3.3	0.173296754
Zfp451|98403	1.747783013	0.062198589	1.747783013	0.062198589
Zfp959|224893	1.742218307	0.047531538	1.568345932	0.087102845
Rasal1|19415	1.741806123	0.210784685	-1.022983438	0.482344907
Emcn|59308	1.74138881	0.033108366	1.575025606	0.064344019
Ush1g|16470	1.741055127	0.166435849	-1.050596242	0.448492769

Kifc1|100042970	1.740651071	0.169005504	2.841706696	0.068706115
Prf1|18646	1.739962815	0.223787884	1.161797284	0.423418494
Nhlrc1|105193	1.739883241	0.092224062	2.407124419	0.032735318
Gins2|272551	1.739656996	0.197232994	2.794941181	0.102756446
Abhd10|213012	1.738235227	0.013335056	1.575073231	0.029166502
Ptpdc1|218232	1.737793143	0.127591068	2.452384709	0.058602847
Krt23|94179	1.737114812	0.131235988	1.737114812	0.131235988
Lzic|69151	1.737090033	0.002955914	1.976369559	0.00077934
Col4a2|12827	1.736979094	2.13639E-05	1.821740748	2.16022E-05
Fam186b|545136	1.736676989	0.334571441	1	#DIV/0!
Cks2|66197	1.735700168	0.10561465	1.763947743	0.037253545
C1galt1c1|59048	1.735590156	0.000279736	1.907986483	6.42354E-05
Cdyl2|75796	1.733716803	0.15360861	2.483012638	0.020371565
Ramp3|56089	1.732294275	0.181885827	1.843195698	0.110966406
Tnni2|21953	1.731704079	0.251125963	1.731704079	0.251125963
Nfe2|18022	1.730130982	0.290244161	1.195314368	0.428478238
Rarres1|109222	1.730100691	0.026798247	2.380697793	3.11289E-05
Dll1|13388	1.729578131	0.010637865	1.729578131	0.010637865
Cyb5r1|72017	1.729551784	0.003858571	1.435813019	0.000958772
Dbndd2|52840	1.726938418	0.011919613	1.529651904	0.025648072
Lrdd|57913	1.726065198	0.163097076	2.35528214	0.062278499
2310004N24Rik|69535	1.725196086	0.100782864	1.108633165	0.287286593
Cd300lf|246746	1.724785363	0.00056329	1.928470282	7.94367E-05
Col1a1|12842	1.721825668	0.045244534	1.299759363	0.152217594
Sash3|74131	1.721021135	0.068593456	1.301826224	0.053890885
Smarca5-ps|545700	1.719804593	0.009727439	1.719804593	0.009727439
Cenpk|60411	1.71937522	0.291968926	1.178571429	0.43416
Shisa4|77552	1.71937522	0.291968926	1.178571429	0.43416
Kcnab2|16498	1.718469773	0.121030551	1.329766625	0.251485966
Accn5|58170	1.716352782	0.002090329	1.439185911	0.003522238
Ckap2|80986	1.714807273	0.277582289	3.3	0.173296754
Dpysl3|22240	1.714807273	0.277582289	3.3	0.173296754
Dnmt3a|13435	1.71383065	0.001354634	1.785844441	0.000201942
Tmem80|71448	1.713764996	0.022986222	1.562325511	0.046984063
D630032N06Rik|654810	1.713717165	0.147843662	4.055582598	0.000652268
Fam83g|69640	1.711561302	0.018996389	1.711561302	0.018996389
Ccdc106|232821	1.711147256	0.058010461	2.149159003	0.023422073
Pcdhb16|93887	1.710854104	0.254378817	1.710854104	0.254378817
Bfsp2|107993	1.710854104	0.254378817	1.710854104	0.254378817
4930579G22Rik|69034	1.710536334	0.267667343	-1.207318182	0.410710794
Adam8|11501	1.710536334	0.267667343	-1.207318182	0.410710794
Vangl2|93840	1.710384512	0.290156517	3.53972	0.173296754
Serpinb6b|20708	1.710214518	0.11028437	3.298156346	0.000197535
Fbxl22|74165	1.710162403	0.16328143	1.183427785	0.358411948
Ccdc138|76138	1.709700266	0.028334413	2.236112773	0.001490233
Slc9a7|236727	1.709491575	0.231419597	2.115242794	0.199265129
Exd1|241624	1.709462987	0.227036993	1.072515922	0.460982508
Tmem29|382245	1.708618854	0.141213054	1.43902314	0.178969994
Cybrd1|73649	1.707492233	0.244198189	1.022371059	0.488874049
Wdr86|269633	1.706194715	0.22539027	1.706194715	0.22539027
Btbd11|74007	1.705274093	0.184911255	1.705274093	0.184911255
Srrm4|68955	1.704100317	0.059954125	1.952395527	0.044373009
Gcnt1|14537	1.703625617	0.255866169	1.703625617	0.255866169

Smok3a|545814	1.703625617	0.255866169	1.703625617	0.255866169
Dnahc1|110084	1.703430269	0.278007657	3.53972	0.173296754
Wt1|22431	1.703430269	0.278007657	3.53972	0.173296754
Pi15|94227	1.703430269	0.278007657	3.53972	0.173296754
Grap2|17444	1.7	0.339818682	1	#DIV/0!
Gnpda2|67980	1.699387637	0.126068259	1.428674132	0.208987186
Ankrd9|74251	1.699176302	0.093758255	1.95642891	0.074601532
Mbip|217588	1.69899193	0.161947626	1.218406139	0.342702781
Hs3st1|15476	1.695730498	0.34062582	1	#DIV/0!
Prr18|320111	1.694433	0.282056419	3.3	0.173296754
Mbd4|17193	1.694012395	0.212697946	1.821115418	0.200948525
Vcam1|22329	1.693944121	0.023511608	1.488706307	0.042004168
Pms2|18861	1.6935719	0.052516991	1.440002085	0.105258322
Pigf|18701	1.692938638	0.156907336	1.085655529	0.377576123
Tmem206|66950	1.692498016	0.085943141	1.311504657	0.167144999
Rad54b|623474	1.690479956	0.174340794	2.97593794	0.049958104
Slc7a10|53896	1.690137306	0.203690447	1.386923112	0.166845771
Gm13315|625342	1.68976391	0.258744163	1.68976391	0.258744163
C5ar1|12273	1.687817161	0.081243525	1.290873716	0.135833757
Airn|104103	1.68717619	0.247310324	2.628480853	0.116118185
Nanp|67311	1.686355938	0.02405028	1.507505494	0.047992762
Atp1a3|232975	1.685024599	0.083236902	1.685024599	0.083236902
Wdr54|75659	1.684117565	0.165443657	1.684117565	0.165443657
Dip2a|64451	1.682825195	0.066415187	1.682825195	0.066415187
Insig2|72999	1.682129121	0.019839264	1.540656675	0.041351494
Elovl7|74559	1.6819329	0.079951444	1.6819329	0.079951444
A330069E16Rik|606735	1.680274376	0.171521126	1.118080797	0.394830313
Syt6|54524	1.675322584	0.20855111	1.542129164	0.275963748
Cd48|12506	1.673950796	0.168670083	3.685421333	0.005826323
Tgif1|21815	1.672893046	0.016903457	1.842750691	0.0031074
E130012A19Rik|103551	1.672072696	0.013036737	1.549040332	0.002105175
Tmem146|106757	1.671238691	0.165596091	2.147528101	0.112038532
Slc8a3|110893	1.670918023	0.154260638	1.670918023	0.154260638
Atad2b|320817	1.669917167	0.049286721	1.914344735	0.033015476
Tnnc1|21924	1.669137207	0.168789764	1.801115536	0.140617952
Erp29|67397	1.668462013	0.003901476	1.536803774	0.005588999
Krr1|52705	1.667167849	0.026937062	1.716725195	0.005058466
Usp11|236733	1.666712393	0.094188072	1.435338308	0.181910264
Rab36|76877	1.666496373	0.045681388	2.044672393	0.016290145
Dync2li1|213575	1.66518265	0.234308448	1.02476681	0.485703619
Neu3|50877	1.663177059	0.036498129	2.062716556	0.008987374
Wdhd1|218973	1.662844164	0.285085214	1.446634395	0.37940868
Sh3pxd2b|268396	1.662281413	0.10592893	2.164525682	0.049243938
Pdgfra|18595	1.661502139	0.053168492	1.924325303	0.00574447
Epcam|17075	1.658917542	0.08667398	2.359416025	0.003884268
Hbb-b1|15129	1.658808621	0.171135035	1.05586455	0.427938081
Pim2|18715	1.658251384	0.019006746	1.780601518	0.009386387
Raet1e|379043	1.65803139	0.16978345	1.965652706	0.113942275
Ng23|78376	1.657400179	0.279682712	10.68586667	0.03955673
Cyth3|19159	1.65722947	0.002042493	1.561350672	0.003935428
2810030E01Rik|72668	1.656308014	0.234924922	4.996574711	0.015247526
Rab30|75985	1.655995849	0.006425989	1.655995849	0.006425989
Pygm|19309	1.655458762	0.19544163	2.511424773	0.098401738

Plp1|18823	1.65496306	0.293997646	6.7875	0.093349877
2810021B07Rik|66308	1.654770698	0.013962365	1.562543792	0.005172639
2410137F16Rik|76798	1.65311698	0.049677617	1.823515109	0.000534107
Wipi1|52639	1.649950383	0.027026089	2.127702102	0.000140678
Egr3|13655	1.646006803	0.290703755	3.3	0.173296754
B3galt6|117592	1.643935222	0.017913926	1.669474221	0.00601375
4933421E11Rik|321000	1.643668916	0.139795695	2.811726142	0.016368122
Zfp449|78619	1.642434197	0.304308741	1.264185714	0.40907038
Zfp40|22700	1.641908034	0.186520531	2.562985067	0.078656243
Armcx5|494468	1.641726912	0.133000759	1.956941884	0.041573613
S100a9|20202	1.639865273	0.205082172	2.25638843	0.131729559
Pdgfb|18591	1.639785943	0.117553031	1.297355082	0.244620616
Dnalc1|105000	1.638835762	0.286085794	-1.591089844	0.221834324
Wwtr1|97064	1.637902241	0.023253166	1.801856214	0.00559497
4933430H15Rik|74485	1.637634894	0.354082855	-5.925	0.173296754
40969|72925	1.63757504	0.317334291	-2.305926269	0.163045149
AU018091|245128	1.635608	0.307092358	3.3	0.173296754
Ccdc39|51938	1.635249119	0.330786619	1	#DIV/0!
Dpysl2|12934	1.635219745	0.093746883	1.635219745	0.093746883
Chic2|74277	1.634456015	0.054750552	1.376056289	0.105309207
Lepre1|56401	1.634419673	0.001742034	1.634419673	0.001742034
Pot1a|101185	1.634302801	0.017722571	1.416974326	0.034758524
Alg11|207958	1.631288237	0.099144374	1.776843013	0.069860994
Tdg|21665	1.631250763	0.0003055	1.851294875	9.40261E-06
Plagl1|22634	1.630344615	0.280842618	17.66206667	0.013575129
Csad|246277	1.629664286	0.046941103	1.85112938	0.007702907
5330426P16Rik|68190	1.629620238	0.083456989	1.256503542	0.135659571
Chsy3|78923	1.627568608	0.090868264	1.125571103	0.331498439
Ampd3|11717	1.625667229	0.11320683	2.690317436	0.001913164
Brsk1|381979	1.622981059	0.281827988	2.416498236	0.177579722
Yy2|100073351	1.622191011	0.338059472	1	#DIV/0!
Ccdc136|232664	1.62201305	0.299045994	3.53972	0.173296754
Top2a|21973	1.62201305	0.299045994	3.53972	0.173296754
Spef1|70997	1.621861073	0.079702089	1.156735878	0.254705091
Nid1|18073	1.62092155	0.025165198	2.032004612	0.001891444
Cd93|17064	1.620481641	0.013355103	1.834817538	0.005442019
Ripply1|622473	1.620006918	0.109418172	2.482791777	0.00478219
Ms4a6b|69774	1.619123801	0.215488456	1.034552071	0.474591385
B930041F14Rik|230991	1.61804507	0.129623833	1.92939465	0.035965247
Sowahc|268301	1.617891915	0.103755829	1.617891915	0.103755829
Dnm1|13429	1.617232416	0.168687387	1.368976929	0.2586574
Ccnb1|268697	1.615943179	0.303199473	6.7875	0.093349877
Dusp5|240672	1.614646679	0.282028388	3.280483654	0.135584145
Klhl23|277396	1.614003622	0.119958136	1.924575533	0.027838627
Neil2|382913	1.613822526	0.204946328	1.150188264	0.403957413
N28178|230085	1.612968336	0.286060305	1.656626026	0.330587367
C1qtnf3|81799	1.612271091	0.329399618	1	#DIV/0!
Enkur|71233	1.612271091	0.329399618	1	#DIV/0!
Pate2|330921	1.612271091	0.329399618	1	#DIV/0!
Naa11|97243	1.612271091	0.329399618	1	#DIV/0!
AI854703|243373	1.612271091	0.329399618	1	#DIV/0!
6330403A02Rik|381310	1.612271091	0.329399618	1	#DIV/0!
4930539J05Rik|319587	1.612271091	0.329399618	1	#DIV/0!

Ddx25|30959	1.612271091	0.329399618	1	#DIV/0!
Cryba4|12959	1.612271091	0.329399618	1	#DIV/0!
Dnajb13|69387	1.612271091	0.329399618	1	#DIV/0!
Sfrp4|20379	1.612271091	0.329399618	1	#DIV/0!
Zfp353|234203	1.612271091	0.329399618	1	#DIV/0!
Bst1|12182	1.612271091	0.329399618	1	#DIV/0!
Stc2|20856	1.612271091	0.329399618	1	#DIV/0!
Bicd1|12121	1.612271091	0.329399618	1	#DIV/0!
Celf3|78784	1.612271091	0.329399618	1	#DIV/0!
D630042P16Rik|243612	1.612271091	0.329399618	1	#DIV/0!
E230008N13Rik|381522	1.612271091	0.329399618	1	#DIV/0!
4930486L24Rik|214639	1.610299403	0.24916252	3.286172748	0.091708258
Ablim2|231148	1.609464281	0.064952026	1.863579639	0.008588502
Dcakd|68087	1.609446259	0.000143569	1.476339825	3.01731E-05
Gulp1|70676	1.609028219	0.242591433	2.86110119	0.109735813
Nrg4|83961	1.608617475	0.012856647	1.608617475	0.012856647
Atr|245000	1.608331881	0.165536819	1.159746679	0.360352085
Myadm|50918	1.608086824	0.023359229	1.455713508	0.049074279
Pus7l|78895	1.607172561	0.066179081	1.752046554	0.017436041
Gm14430|627914	1.606469787	0.273142306	4.265682105	0.084695435
Sesn3|75747	1.606390303	0.186819853	1.938834203	0.145831587
Opn3|13603	1.605987021	0.201435558	2.26552607	0.114826986
Ctsf|56464	1.605984493	0.000355363	1.426465138	0.000260911
D030056L22Rik|225995	1.605594909	0.094715339	2.689412067	0.000348772
P2ry14|140795	1.605590687	0.175073558	1.605590687	0.175073558
Gm4841|225594	1.605445423	0.119479619	1.276859448	0.248987319
1700021C14Rik|76927	1.605164826	0.161675246	1.605164826	0.161675246
Nasp|50927	1.604128761	0.003901397	1.71822512	0.00298969
Lama2|16773	1.603533792	0.171647025	1.603533792	0.171647025
Gm3230|100041244	1.603410186	0.1908988	1.603410186	0.1908988
Eif3j|78655	1.602958309	0.006995331	1.839362787	0.001126859
Dusp22|105352	1.602626874	0.002739512	1.518785601	0.005855771
Retnlg|245195	1.60073399	0.252065169	3.286172748	0.091708258
Zfp472|224691	1.599960359	0.232411893	1.599960359	0.232411893
Gas2l3|237436	1.599272212	0.057190988	1.184722635	0.161224317
Smoc2|64074	1.599246733	0.040753925	1.95887898	0.009648713
Ccbp2|59289	1.599205728	0.127719482	1.599205728	0.127719482
1810011H11Rik|69069	1.599096849	0.260571911	-1.058006311	0.468991309
Id4|15904	1.598845597	0.162066484	1.598845597	0.162066484
Spa17|20686	1.598651823	0.247926028	-1.037466066	0.477413445
D16Ertd472e|67102	1.598422055	0.007726001	1.598422055	0.007726001
Tmem194b|227094	1.598214999	0.284206304	5.191177856	0.074944339
Nedd1|17997	1.596600817	0.115474362	1.264206874	0.243064343
Hist2h3c2-ps|97114	1.594619687	0.218656718	2.925783386	0.067209103
Fcrla|98752	1.594344022	0.12711092	2.618395089	0.003831982
Gabpb1|14391	1.592773053	0.00614252	1.439893325	0.012161006
Fam134b|66270	1.592536899	0.067561217	1.471544529	0.066667248
6530418L21Rik|109050	1.592473714	0.224462127	2.95373508	0.071577527
Cyp2c67|545288	1.591918179	0.007090146	1.601646286	0.001884363
Lancl1|14768	1.591471844	0.002388913	1.665682536	0.001298225
D430020J02Rik|319545	1.591193333	0.333510192	1	#DIV/0!
Bcl2l15|229672	1.591193333	0.333510192	1	#DIV/0!
Pif1|208084	1.591193333	0.333510192	1	#DIV/0!

Srpk3|56504	1.591193333	0.333510192	1	#DIV/0!
4932418E24Rik|329366	1.591193333	0.333510192	1	#DIV/0!
Tmem45a|56277	1.591193333	0.333510192	1	#DIV/0!
Chl1|12661	1.591193333	0.333510192	1	#DIV/0!
Sprr2b|20756	1.591193333	0.333510192	1	#DIV/0!
Gpm6b|14758	1.591193333	0.333510192	1	#DIV/0!
Ckmt1|12716	1.591193333	0.333510192	1	#DIV/0!
Gng8|14709	1.591193333	0.333510192	1	#DIV/0!
Slc23a3|22626	1.591193333	0.333510192	1	#DIV/0!
Dcx|13193	1.591193333	0.333510192	1	#DIV/0!
Tll1|21892	1.591193333	0.333510192	1	#DIV/0!
Zcchc5|213436	1.591193333	0.333510192	1	#DIV/0!
Nup210|54563	1.590926934	0.005163037	1.914753749	0.000133265
Ppm1h|319468	1.590665639	0.057227654	2.148649147	0.003543931
Fam72a|108900	1.590446028	0.31730025	1.170129778	0.437059136
Jag2|16450	1.589790083	0.290294141	1.621721864	0.33676256
Cerk|223753	1.588946837	0.044856175	1.3860525	0.08897764
Gprin3|243385	1.587447821	0.201069479	2.277317959	0.10591002
Snupn|66069	1.587309293	0.023059387	1.747241268	0.004350286
Tmem116|77462	1.586550029	0.14557918	1.156464707	0.325969498
B3galnt1|26879	1.585600248	0.247540931	2.840724964	0.108233825
Morc4|75746	1.58488368	0.06677429	1.155106376	0.251654237
Klhdc3|71765	1.581859358	0.014515732	1.407203286	0.016919528
Rps6ka5|73086	1.581674262	0.293285717	1.561221429	0.345871498
Mier3|218613	1.581373308	0.113823035	1.894277172	0.017593478
Hapln1|12950	1.580550957	0.335626927	1	#DIV/0!
Ak8|68870	1.580550957	0.335626927	1	#DIV/0!
2010015L04Rik|544678	1.580550957	0.335626927	1	#DIV/0!
Glb1l2|244757	1.580550957	0.335626927	1	#DIV/0!
Nudt11|58242	1.580550957	0.335626927	1	#DIV/0!
Cxcr2|12765	1.580550957	0.335626927	1	#DIV/0!
Fsd1l|319636	1.580550957	0.335626927	1	#DIV/0!
Pcdhb14|93885	1.580550957	0.335626927	1	#DIV/0!
Hes7|84653	1.580550957	0.335626927	1	#DIV/0!
Fasl|14103	1.580550957	0.335626927	1	#DIV/0!
Optc|269120	1.580550957	0.335626927	1	#DIV/0!
Slc4a10|94229	1.580550957	0.335626927	1	#DIV/0!
P2rx6|18440	1.580550957	0.335626927	1	#DIV/0!
Ly86|17084	1.580239076	0.08482527	1.861862536	0.01425558
Gjc3|118446	1.579920553	0.07632588	1.312006845	0.154570709
2410022L05Rik|66423	1.57788623	0.120627201	1.57788623	0.120627201
Gabrr2|14409	1.577176088	0.30820807	3.53972	0.173296754
Dnd1|213236	1.575613358	0.198628813	2.516108079	0.07074333
Hoxa5|15402	1.575490549	0.269689825	1.492617986	0.321647633
Fdxacb1|382137	1.574961386	0.078665184	1.42116787	0.147901136
Sestd1|228071	1.57430781	0.252213203	1.192147174	0.369163595
Gpx7|67305	1.573831363	0.082496685	1.43010164	0.150824899
1700030K09Rik|72254	1.572678536	0.080601178	1.572678536	0.080601178
Itga11|319480	1.572421054	0.300720352	3.327242993	0.08214779
Dtna|13527	1.572179196	0.301935514	3.422055173	0.136421656
Gt(ROSA)26Sor|14910	1.571768582	0.147996493	2.132183595	0.06274425
Lyz2|17105	1.571066609	0.020978591	1.360990167	0.014562262
Calr4|108802	1.570778182	0.352322708	1	#DIV/0!

Cox6b2|333182	1.570358496	0.23182545	-1.088200321	0.423395033
9130019P16Rik|100042056	1.570256579	0.337701205	1	#DIV/0!
Foxq1|15220	1.570175412	0.041192701	2.145683924	0.001199667
1600002K03Rik|69770	1.56985325	0.007767184	1.644830435	0.001568458
Slc7a4|224022	1.56965119	0.249730179	-1.092720178	0.438525627
Hmgb2|97165	1.568599541	0.03865003	1.4512837	0.004966728
Tmem88b|320587	1.567918006	0.142613859	1.877702539	0.038067883
H2-Bl|14963	1.566738995	0.281967429	1.093820762	0.456057209
Tctn1|654470	1.565884542	0.141154224	2.849911855	0.002551325
Gfpt2|14584	1.565732637	0.177085292	1.565732637	0.177085292
Rfc5|72151	1.56369916	0.026287288	1.56369916	0.026287288
Tomm20|67952	1.562911843	0.005730443	1.465093774	0.013306094
Frat2|212398	1.562817387	0.015008263	1.938019809	5.19821E-05
Btbd10|68815	1.562718128	0.110105921	1.554503445	0.01407623
Vash1|238328	1.562314689	0.179042935	2.502290241	0.045014704
Gjc2|118454	1.562108025	0.14243572	1.562108025	0.14243572
Il7r|16197	1.561689375	0.319149176	1.264185714	0.40907038
Arhgef10|234094	1.561174097	0.081170525	2.355225044	0.000294116
Rasl11a|68895	1.560918253	0.263471521	-1.021117677	0.48843767
Nbl1|17965	1.560616914	0.224123569	1.560616914	0.224123569
0610010O12Rik|66060	1.559585688	0.013424795	1.411261452	0.028579683
Dnajb5|56323	1.559465084	0.070041396	1.329946116	0.142902753
Prkra|23992	1.559429544	0.084471454	1.538733998	0.055104923
Tbc1d9|71310	1.558043053	0.169975312	1.558043053	0.169975312
Syn3|27204	1.558033426	0.060014449	1.733225681	0.020325823
Ly6c2|100041546	1.556760423	0.302448095	-1.413112558	0.336046301
Fabp4|11770	1.555888027	0.130996482	1.633621918	0.088940405
Trim30b|244183	1.55578618	0.298658649	1.550039004	0.34809122
Uck2|80914	1.555534351	0.013009059	1.402923563	0.018157915
Lgi3|213469	1.555052675	0.342162404	1.170129778	0.437059136
Mitf|17342	1.554501312	0.154793217	2.659005118	0.006003288
Rprd1a|225283	1.553921026	0.019442125	1.553921026	0.019442125
Pacrgl|66768	1.553752432	0.151815086	1.559325676	0.129875028
Snx8|231834	1.553603244	0.003666477	1.581292096	0.001339715
Fam73a|215708	1.553110455	0.009704595	1.669422325	0.001839827
Msh5|17687	1.552285189	0.300408339	1.202941586	0.406502778
Col9a3|12841	1.552115173	0.355818567	1	#DIV/0!
Grip2|243547	1.552115173	0.355818567	1	#DIV/0!
Spns1|73658	1.552114521	1.86231E-05	1.50462401	2.42115E-05
Unc45b|217012	1.551522209	0.110816912	1.295191071	0.207300744
D3Ertd254e|241944	1.550321623	0.205456718	-1.012658604	0.48573066
Mitd1|69028	1.550054037	0.181041043	1.702241461	0.128772699
Thbd|21824	1.549981151	0.15441603	1.179650758	0.32896355
Trmt11|73681	1.549749327	0.158044517	2.30907801	0.040354098
Pla2g7|27226	1.549663498	0.055437868	1.484157313	0.020365534
Treml2|328833	1.54965616	0.202792098	1.309465955	0.298915572
Dnaic1|68922	1.549216748	0.208533331	1.409166295	0.255948929
Gfod1|328232	1.548744518	0.237273037	1.548744518	0.237273037
Camk4|12326	1.548400408	0.238457863	1.897134943	0.18332558
Pdk4|27273	1.548297846	0.082605725	1.597363838	0.045847794
Cnnm1|83674	1.548130419	0.300774108	1.561221429	0.345871498
Tmem200b|623230	1.547672227	0.159807021	1.31376077	0.214507599
Fmo3|14262	1.546794514	0.042880032	1.963178797	0.001247135

Mycl1|16918	1.544664124	0.063517674	1.739653145	0.042698852
Dmrta2|242620	1.543991786	0.340461672	1	#DIV/0!
Mybpc2|233199	1.543991786	0.340461672	1	#DIV/0!
Ptprr|19279	1.543991786	0.340461672	1	#DIV/0!
Tex12|66654	1.543991786	0.340461672	1	#DIV/0!
Lhfpl4|269788	1.543991786	0.340461672	1	#DIV/0!
Crlf1|12931	1.543991786	0.340461672	1	#DIV/0!
AI429214|621080	1.543659029	0.274025351	-1.545699182	0.181043012
Myc|17869	1.543225787	0.101162912	1.089161531	0.354665473
Loxl4|67573	1.542782138	0.146056754	1.127108982	0.33185127
Lnx1|16924	1.540895169	0.280208374	-1.187493756	0.402928474
Col1a2|12843	1.540750877	0.015148384	1.852572646	0.000966699
Wfdc2|67701	1.538068644	0.20152326	-1.168801684	0.274855216
Xrn1|24127	1.537985247	0.196644878	-1.083893283	0.420822078
Trim13|66597	1.53797707	0.183943505	1.299597075	0.283667164
Figf|14205	1.537025115	0.231473699	2.088968806	0.109749661
Styx|56291	1.536301193	0.219565828	2.801802244	0.050413261
Fpgt|75540	1.536143441	0.218346592	2.598637941	0.067424281
Hcst|23900	1.535983687	0.266660878	1.256853002	0.375302811
Fmn2|54418	1.535813957	0.214574236	2.097063616	0.12559501
2210408I21Rik|72371	1.535762912	0.271142875	3.152792568	0.097328085
Pnrc1|108767	1.53510786	0.041387844	1.555842865	0.007378161
Lrrn4cl|68852	1.534816085	0.206154791	1.534816085	0.206154791
Ppid|67738	1.534522964	0.041166701	1.534522964	0.041166701
Mmp11|17385	1.534353078	0.093865424	1.67257936	0.058867608
Phf10|72057	1.533265584	0.019953762	1.72476554	0.007948434
Mup20|381530	1.533140166	0.003629128	1.793632035	6.05279E-05
Gm14403|433520	1.531839274	0.062308366	1.695764071	0.003324117
Apex2|77622	1.53089955	0.025376986	1.734764466	0.010107784
Gm14434|668039	1.530409833	0.292898403	3.711837887	0.101383067
Rwdd2a|69519	1.529982304	0.320965612	-1.76025563	0.245710688
Chadl|214685	1.52959715	0.244522287	1.576626233	0.241936065
Anxa13|69787	1.529512029	0.246558279	2.176557753	0.150790365
Fyttd1|69823	1.529481084	0.031438407	1.803747552	0.006922127
Gm561|228715	1.529241847	0.042860945	1.368290734	0.086429621
Haghl|68977	1.529007951	0.11325651	1.529007951	0.11325651
Med31|67279	1.528499771	0.118717161	1.277679232	0.219991269
Them4|75778	1.527974844	0.004694177	1.478063041	0.001694963
Zfp366|238803	1.526082857	0.345733557	3.53972	0.173296754
Agxt2l1|71760	1.525569137	0.0080857	1.389069131	0.008470735
Blmh|104184	1.525383658	0.000624579	1.525383658	0.000624579
Ptprm|19274	1.524458804	0.188661168	1.160908025	0.373083355
Gm4013|100042757	1.524198416	0.090880098	1.44444941	0.094530773
Cyp2c40|13099	1.524037714	5.89652E-05	1.514136635	7.71541E-06
Phospho1|237928	1.523977009	0.028281498	1.523977009	0.028281498
Gm5607|434280	1.523806667	0.344811039	1	#DIV/0!
Gpr143|18241	1.523806667	0.344811039	1	#DIV/0!
Eda2r|245527	1.523806667	0.344811039	1	#DIV/0!
Spata17|74717	1.523806667	0.344811039	1	#DIV/0!
Cyb5r2|320635	1.523806667	0.344811039	1	#DIV/0!
AU023871|106722	1.523806667	0.344811039	1	#DIV/0!
Il1rn|16181	1.523665053	0.031750869	1.347817839	0.056407876
Hspa1l|15482	1.523593207	0.180133251	2.074413169	0.084458387

Ccno|218630	1.523122753	0.177202593	1.815633751	0.068671382
Rnf144a|108089	1.521787154	0.067416119	1.717408719	0.011454019
Serpinh1|12406	1.521617511	0.012522605	1.371995266	0.014588717
Lamtor3|56692	1.521566859	0.008030339	1.521566859	0.008030339
Fbxo44|230903	1.520979276	0.063378479	1.565434627	0.037322805
Clec4n|56620	1.520585967	0.251566621	1.341255403	0.276262558
Leprotl1|68192	1.520308446	0.001878333	1.336276236	0.003597429
Rab3b|69908	1.520171418	0.223219206	2.451183368	0.081306495
Rasl2-9-ps|19428	1.519895066	0.208242547	1.056067682	0.451113008
Slc43a2|215113	1.518996735	0.026644094	1.275738218	0.047380587
Dnajc24|99349	1.518634373	0.143762708	1.85808255	0.084413074
Abcb1b|18669	1.517868413	0.127813212	1.963768857	0.042895707
Hist1h2ac|319164	1.517325281	0.294290837	-1.327863533	0.3395387
Cyp26b1|232174	1.517253848	0.175138758	1.311476574	0.266994516
Egfl6|54156	1.517219203	0.298445829	-1.134788439	0.440829738
Otud1|71198	1.516809503	0.184149991	1.516809503	0.184149991
Kctd5|69259	1.516270061	0.003559442	1.595094914	0.000489951
Galntl2|78754	1.515658791	0.362921753	-4.6202	0.07056113
Loxl2|94352	1.515001621	0.11542724	1.659504888	0.053043363
6330503K22Rik|101565	1.514113161	0.246076273	4.287574707	0.004643506
Pcdhb9|93880	1.513614992	0.347048781	1	#DIV/0!
Espnl|227357	1.513614992	0.347048781	1	#DIV/0!
Wnt6|22420	1.513614992	0.347048781	1	#DIV/0!
Cacna1h|58226	1.513614992	0.347048781	1	#DIV/0!
Cdkl3|213084	1.513614992	0.347048781	1	#DIV/0!
Muc2|17831	1.513614992	0.347048781	1	#DIV/0!
Foxred2|239554	1.513614992	0.347048781	1	#DIV/0!
Hmga2|15364	1.513614992	0.347048781	1	#DIV/0!
Arl4a|11861	1.512815055	0.018808521	1.435935565	0.016907704
4933433P14Rik|66787	1.510478533	0.056422827	1.201561744	0.186737957
Elf4|56501	1.509758557	0.084037864	1.784058531	0.040996457
Eef1e1|66143	1.509091414	0.049851427	1.902142762	0.00389414
4921531C22Rik|66730	1.508119084	0.143125479	1.508119084	0.143125479
Tdp1|104884	1.506845115	0.191157632	1.062828363	0.4340114
Basp1|70350	1.505555795	0.229516995	2.722960505	0.053310408
Yod1|226418	1.505360233	0.079234556	1.677589338	0.029385856
Ostc|66357	1.505213455	0.003698621	1.505213455	0.003698621
Rps27a|78294	1.503780395	0.05899527	1.246739733	0.077578772
AI506816|433855	1.503576894	0.018465531	1.228203987	0.01899898
Mbnl3|171170	1.503284651	0.266908369	-1.364833499	0.27514407
Cth|107869	1.502899451	0.077606159	1.502899451	0.077606159
Uap1l1|227620	1.501499268	0.118694157	1.231810917	0.093864715
Rabl2|68708	1.500779567	0.16057069	1.814235327	0.10326542
Gnb4|14696	1.500275723	0.245041763	1.810320672	0.174098776
Usp2|53376	1.500018051	0.114804354	1.500018051	0.114804354
Cdca4|71963	1.499212742	0.079290751	1.388359563	0.13154936
Id1|15901	1.49918516	0.221235865	1.098701917	0.364479087
Utp23|78581	1.498266283	0.022851095	1.323865223	0.044744872
Gpr153|100129	1.49812176	0.126542799	1.897636837	0.012509157
Metrnl|210029	1.497167297	0.058621107	1.497167297	0.058621107
Abcg3|27405	1.496594879	0.250812516	2.09719198	0.085248798
Caskin1|268932	1.496485249	0.296372051	1.869568631	0.262507494
Itga5|16402	1.496091338	0.000841445	1.444909909	9.08194E-05

Cd86|12524	1.495086867	0.249474109	1.495086867	0.249474109
Col4a1|12826	1.494456392	0.01019788	1.378848238	0.022095056
Pam16|66449	1.494285688	0.090368043	1.723161268	0.015507514
Pdxp|57028	1.49369221	0.302924202	-1.120799574	0.446529338
Cxx1c|72865	1.493638138	0.240689816	-1.048079823	0.460385891
Syt9|60510	1.49363479	0.284847167	2.497345645	0.15023545
Cntnap1|53321	1.49318664	0.218886533	1.075820605	0.440481019
Rab34|19376	1.492712295	0.169996807	1.06103035	0.409963228
Akr1b3|11677	1.49139661	0.032425612	1.702621254	0.011095391
Slc25a33|70556	1.49068832	0.076461858	1.27179778	0.156051525
Zfp109|56869	1.49057391	0.298051838	3.884819044	0.079108848
Tmem189|407243	1.489694628	0.024470261	1.375578277	0.051076718
Dcaf4|73828	1.488135949	0.116163928	1.263372542	0.231804503
Gata5|14464	1.487381248	0.304720339	-1.125555129	0.444583663
Zfp688|69234	1.486767757	0.074543312	1.269328572	0.158402985
Gm16515|24083	1.486691234	0.098782751	1.766817784	0.048429334
Cyb561|13056	1.486682606	0.01289864	1.486682606	0.01289864
Gm10052|654467	1.486498468	0.054425788	1.708514668	0.024044223
Zyg11a|230590	1.485971928	0.334366802	3.53972	0.173296754
Glp2r|93896	1.485802747	0.307469052	-1.202217172	0.412618198
Gm6225|633947	1.485365753	0.295536465	3.142379154	0.110829606
Gal3st1|53897	1.485353516	0.181174725	1.935212217	0.03877158
Dleu2|668253	1.483734761	0.085025644	1.274874458	0.174638979
Dis3|72662	1.483440762	0.071698186	1.2935024	0.146717988
Cd300lb|217304	1.483338548	0.250884917	1.397926113	0.268339602
Asf1a|66403	1.483189256	0.178755872	1.304274245	0.20307533
Fam189a2|381217	1.482617595	0.078993409	1.393575225	0.028167327
Tigd2|68140	1.482576269	0.080882018	1.380783218	0.142321095
Ccdc148|227933	1.482247167	0.169329044	1.193865056	0.329205452
1110020A21Rik|68531	1.48143262	0.220529882	2.067446514	0.111647371
Tmem39a|67846	1.480191197	0.030323204	1.886250268	0.000644759
5430402O13Rik|73183	1.479301419	0.204870007	1.11607949	0.40463379
2610039C10Rik|66578	1.478748007	0.255313892	2.049454485	0.157687848
Ctdspl2|329506	1.478354143	0.187782579	1.14140149	0.375388333
Gpr157|269604	1.478028226	0.012069183	1.478028226	0.012069183
Anxa1|16952	1.477864408	0.229834851	2.082032377	0.118941435
Celf2|14007	1.477777878	0.123766061	1.402830161	0.140365493
Bok|51800	1.477398605	0.00866254	1.361571201	0.018187599
Galc|14420	1.476931759	0.021432313	1.634307207	0.0095011
Rad51c|114714	1.476797103	0.180016042	1.917674808	0.018159798
Cyp2c69|100043108	1.476643144	0.042819821	1.476643144	0.042819821
Ppp1r14a|68458	1.476234581	0.202429315	1.476234581	0.202429315
Snx24|69226	1.47569025	0.113471698	1.074861404	0.388119972
Zfp651|270210	1.475125874	0.092836532	1.475125874	0.092836532
Gtpbp2|56055	1.474356772	0.012217025	1.433855061	0.002944848
Wdr47|99512	1.473239224	0.267382334	1.247693513	0.359706472
Unc13b|22249	1.470855997	0.023081946	1.674915903	0.005375902
Trim45|229644	1.470472773	0.197742655	1.470472773	0.197742655
Tmtc1|387314	1.468820233	0.13853875	1.473527165	0.038804557
Tomt|791260	1.468576966	0.225550785	-1.178587428	0.333808582
Gem|14579	1.468368912	0.228583045	1.672315717	0.160612305
Cav1|12389	1.468218653	0.288998444	-1.123819285	0.432906884
Ctla2a|13024	1.467784128	0.13787279	2.203102937	0.00440941

Prr15|78004	1.467486826	0.312698756	-1.217222222	0.407029201
Rnmt|67897	1.467441968	0.010653239	1.594639417	0.004902311
Cep70|68121	1.467274298	0.303617064	1.741557483	0.264325109
Plxna1|18844	1.46564466	0.064454262	1.15061706	0.236867964
Lsm5|66373	1.465086614	0.276650904	1.817915524	0.21549686
Mlf1|17349	1.464174545	0.330521691	5.0331	0.093589634
A830080D01Rik|382252	1.464174545	0.330521691	5.0331	0.093589634
Rassf10|78748	1.464174545	0.330521691	5.0331	0.093589634
Kdm2b|30841	1.463624351	0.160700159	1.452099642	0.11742642
Cbr3|109857	1.463490639	0.084966591	1.770455624	0.027365596
Syt12|171180	1.462378844	0.220447848	-1.142453521	0.367631576
Gm9992|667055	1.462120402	0.252754229	1.009326982	0.493355312
Slc26a11|268512	1.461948145	0.005808637	1.371075532	0.012994408
Ahi1|52906	1.461445604	0.324601227	1.550039004	0.34809122
Adnp2|240442	1.46139495	0.219793073	2.342400888	0.05690876
Chrm1|12669	1.461294043	0.16165591	1.6257827	0.098804297
Zmat1|215693	1.460974562	0.168629251	-1.043883908	0.44080055
Rbbp8|225182	1.460620565	0.145477328	1.479873563	0.050321416
Eml6|237711	1.460606061	0.371637124	1	#DIV/0!
Atp11b|76295	1.459736247	0.064850385	1.661121937	0.013621546
Tmem55b|219024	1.459719215	0.007043215	1.551012435	0.004807465
Fam187b|76415	1.459251864	0.048677622	1.378918222	0.085859652
Slc35e3|215436	1.458149029	0.092891839	1.614380504	0.009062465
Tat|234724	1.458120092	0.07800485	1.710750107	0.031974476
Nap1l1|53605	1.457400894	0.002920931	1.457400894	0.002920931
Slco1a1|28248	1.457149104	0.041225585	1.626926634	0.020332895
Eri1|67276	1.455321313	0.189934072	1.891633522	0.095431409
Pgam5|72542	1.455195459	0.084607691	1.360478228	0.011097706
Gm15772|100034726	1.45508339	0.397040671	-159.72342	0.070951641
Fam164c|72350	1.454635324	0.054712534	1.565094754	0.028377718
Jph1|57339	1.454545455	0.372796286	1	#DIV/0!
4930453N24Rik|67609	1.453967228	0.052609639	1.679115368	0.018253246
Nlrp12|378425	1.453309796	0.03670937	1.304634286	0.070894495
Gm10639|100042314	1.452857727	0.27526477	1.452857727	0.27526477
Tfeb|21425	1.45278101	0.009083848	1.380682422	0.020407911
Ccdc122|108811	1.45235913	0.201357566	1.126176735	0.391450626
Cpeb3|208922	1.452306823	0.222505383	1.407813988	0.218020386
2810454H06Rik|72813	1.452171165	0.372720229	-2.76078	0.173296754
Nip7|66164	1.450196588	0.005789742	1.54401215	0.003464869
Col4a3|12828	1.450055071	0.338996507	10.68586667	0.03955673
Adra1a|11549	1.448068237	0.104114654	2.03128055	0.001335851
D230037D09Rik|320351	1.447897694	0.088418718	1.930340432	0.004248331
Ggt5|23887	1.447659507	0.025715275	1.315368297	0.047765982
Tmem198|319998	1.447445361	0.343047623	-1.924451597	0.222249861
Samsn1|67742	1.447377722	0.300100475	-1.186624586	0.403199787
Hccs|15159	1.447235225	0.118265324	1.382152726	0.165346169
A830082N09Rik|414093	1.446778134	0.335849673	5.0331	0.093589634
Nek2|18005	1.445879939	0.272876361	-1.131666396	0.407857015
Scamp5|56807	1.445458226	0.015854416	1.549177813	0.010346847
4931440P22Rik|71004	1.443330144	0.258568166	1.443330144	0.258568166
Mark1|226778	1.44319648	0.292197929	1.380313321	0.29986082
Pigc|67292	1.44306489	0.037172174	1.44306489	0.037172174
Tusc5|237858	1.44203427	0.161159893	1.189560462	0.313745804

Rab6b|270192	1.441643016	0.144010578	1.197318563	0.288572468
Sspn|16651	1.441451757	0.22189476	1.875628403	0.128510793
Ift80|68259	1.441285413	0.304948702	1.441285413	0.304948702
Dpy19l1|244745	1.441280805	0.012885667	1.331853063	0.022070456
Enox2|209224	1.441050153	0.112757746	1.578445871	0.064241961
Thsd7a|330267	1.440855769	0.277153022	1.440855769	0.277153022
Tmem164|209497	1.439204059	0.104135116	1.720025429	0.04301679
Cpxm1|56264	1.438958506	0.077866574	1.096291686	0.300850527
Tmem50b|77975	1.437994786	0.133275232	1.437994786	0.133275232
Gm4535|100043585	1.437978445	0.198843461	1.713296487	0.07598982
Parg|26430	1.437884884	0.007658356	1.437884884	0.007658356
Aifm2|71361	1.437573464	5.00433E-05	1.437573464	5.00433E-05
P2ry12|70839	1.43726345	0.376580875	1	#DIV/0!
Gm6377|622976	1.43726345	0.376580875	1	#DIV/0!
Fgfbp3|72514	1.436533446	0.237920079	2.159930886	0.091964252
Mdga1|74762	1.43640584	0.333714983	1.255130842	0.411829119
Pak6|214230	1.435511399	0.145314012	1.285884312	0.229163778
Tollip|54473	1.435381699	0.000518031	1.451707806	7.60791E-05
Tspyl2|52808	1.434994058	0.011105822	1.434994058	0.011105822
Nfxl1|100978	1.434677285	0.151005267	1.67894942	0.041930173
Ercc8|71991	1.434281115	0.182052227	1.200815148	0.306148939
E130308A19Rik|230259	1.434125302	0.196182397	1.200686053	0.318876424
Msh2|17685	1.433837131	0.03926054	1.403177762	0.00819562
Cep57l1|103268	1.433801186	0.230330496	1.433801186	0.230330496
Cyp4f18|72054	1.432388113	0.223314613	-1.04543465	0.459223657
1700040L02Rik|73287	1.431859551	0.144352513	2.062429783	0.009656562
Apbb1ip|54519	1.431638614	0.149190799	2.133336226	0.012898604
Far1|67420	1.430582801	0.377132364	-2.8	0.173296754
Pros1|19128	1.429847337	0.067297671	1.429847337	0.067297671
4833442J19Rik|320204	1.429686467	0.054978668	1.284954306	0.063605765
Ccl25|20300	1.428859426	0.204287231	1.428859426	0.204287231
Dcun1d3|233805	1.428476905	0.007934991	1.428476905	0.007934991
Trim72|434246	1.428291419	0.303595886	2.385256317	0.155903388
Prps1l3|328099	1.427923604	0.077184489	1.575328796	0.052359391
Tmem120b|330189	1.427468184	0.248842224	-1.324980782	0.166775619
Cpz|242939	1.426810116	0.281612218	1.426810116	0.281612218
Morf4l1|21761	1.426642794	0.000803631	1.300191428	0.001265608
Fbxo10|269529	1.426265734	0.179612997	1.101534391	0.384044447
Cd3g|12502	1.426091385	0.185710805	1.701700637	0.056908728
Cspg4|121021	1.42603798	0.270682115	1.42603798	0.270682115
Tmem216|68642	1.425649041	0.129003991	1.425649041	0.129003991
Olfr1033|258571	1.425324895	0.243160342	-1.235682044	0.284787689
Cdca5|67849	1.424979394	0.378608862	1	#DIV/0!
Manba|110173	1.424694653	0.105698136	1.286728984	0.181311222
Cry1|12952	1.424548817	0.039626761	1.310347507	0.001941683
Wdr92|103784	1.424266957	0.043448151	1.424266957	0.043448151
Nat14|269854	1.424114757	0.151589424	1.650753753	0.099230678
Pfn2|18645	1.424047249	0.1781429	1.699516571	0.048298843
Npat|244879	1.42400877	0.081804854	1.900174054	0.002051717
Glt28d2|320302	1.423893733	0.151376284	1.705952512	0.020989191
Polr2h|245841	1.423216272	0.080014808	1.661197783	0.010589413
Cln6|76524	1.422685465	0.147508512	1.137626726	0.121554816
Mettl4|76781	1.422576061	0.277399557	-1.092741834	0.436908946

Whsc1|107823	1.422350706	0.076418429	1.589388028	0.045391128
BC100451|58251	1.422228	0.35122214	3.53972	0.173296754
Mtf2|17765	1.422147473	0.298482829	-1.260274249	0.343823515
Vav1|22324	1.421968815	0.147811996	1.421968815	0.147811996
Fam35a|75698	1.421939759	0.01705109	1.54165182	0.008193389
4930579K19Rik|75881	1.421921731	0.307271044	2.901746366	0.107384197
Plekhg1|213783	1.421166703	0.067528335	1.408662508	0.019979567
Mfsd7c|217721	1.420652013	0.049519676	1.757833209	0.001699707
Yrdc|230734	1.4206216	0.001218221	1.4206216	0.001218221
Carns1|107239	1.420558972	0.232722014	1.032411096	0.471270506
Polr2d|69241	1.420223621	0.036072785	1.331962237	0.020237041
Ccl27b|100040048	1.420092323	0.321518831	8.404084902	0.000634102
Tek|21687	1.419753073	0.151971233	1.365267355	0.166163244
Siva1|30954	1.419503692	0.034726264	1.299475984	0.070500943
H2-M3|14991	1.418900874	0.114061243	1.939007863	0.004869833
Tmc7|209760	1.418188539	0.167304048	1.655232943	0.067647171
Plk2|20620	1.418074066	0.038447271	1.302695499	0.078096678
Ctsl|13039	1.417230989	0.024522743	1.548842919	0.011426539
Rcn1|19672	1.416584869	0.025599819	1.169398589	0.076128199
Lrat|79235	1.416518739	0.168374716	1.667761447	0.049128382
41162|103080	1.416464953	0.163193599	1.584200382	0.068347162
Kdm6b|216850	1.416437965	0.029242679	1.266481161	0.042606312
Itgb2|16414	1.416009848	0.113356762	1.201110115	0.234899837
Col4a5|12830	1.415746909	0.260529353	1.415746909	0.260529353
Klhl9|242521	1.415568679	0.031405274	1.595111493	0.00850716
Cd83|12522	1.415454893	0.271041297	1.196041987	0.373753569
Setdb2|239122	1.414400783	0.313738203	-1.596986964	0.106972279
C2cd4d|271944	1.413799646	0.209716476	1.735794837	0.023828646
Pafah1b3|18476	1.413378372	0.216116258	-1.032875478	0.453316681
Phf13|230936	1.413210152	0.176110567	1.82096403	0.071668902
Sp5|64406	1.413115412	0.276999541	-1.452450598	0.137779374
Mogat1|68393	1.413023282	0.299755667	1.413023282	0.299755667
Smc5|226026	1.412423922	0.260619969	-1.002172774	0.498351266
Fancm|104806	1.411850242	0.281237636	-1.003996774	0.497448057
Ttc8|76260	1.411798147	0.261293896	2.004778333	0.135859139
Lbr|98386	1.41162008	0.187632119	1.889024934	0.066820898
2610301B20Rik|67157	1.411037523	0.219253234	1.072975131	0.433157647
Col5a2|12832	1.410495482	0.236545323	1.421702807	0.194334091
Insl6|27356	1.410196042	0.324347148	1.09482839	0.450334516
2310008H04Rik|224008	1.409812937	0.243399914	2.442770362	0.038925762
Grap|71520	1.409284483	0.0378249	1.141154007	0.115295609
Mageh1|75625	1.409096735	0.207231055	1.552504139	0.180598188
Tuft1|22156	1.409090056	0.177980305	1.589685246	0.098142136
Nccrp1|233038	1.40824647	0.307271775	2.160783016	0.178229908
Arc|11838	1.408048675	0.382468346	1	#DIV/0!
Rel|19696	1.408048675	0.382468346	1	#DIV/0!
1110034G24Rik|73747	1.40788285	0.043273091	1.516755155	0.010380729
Dctpp1|66422	1.407862899	0.055590452	1.379545932	0.014239642
Zbtb16|235320	1.407385018	0.154961926	1.150203817	0.320095041
Slc15a2|57738	1.407275347	0.197590539	1.407275347	0.197590539
Ntpcr|66566	1.406961559	0.046263349	1.136851567	0.170467988
Arl3|56350	1.406753351	0.042861338	1.334996708	0.030518999
Inpp5d|16331	1.406502749	0.104577901	1.706698025	0.03065706

Rltpr|234695	1.406228221	0.305356037	1.406228221	0.305356037
Tlr13|279572	1.405995325	0.276471615	1.441053198	0.29170982
Ncrna00085|75202	1.405526824	0.213261271	1.405526824	0.213261271
Gtf2a2|235459	1.40535501	0.034237626	1.286955417	0.070836622
Hist3h2a|319162	1.404600058	0.090026539	1.528523259	0.068273553
Enho|69638	1.404264327	0.041237745	1.152162089	0.155358038
Park2|50873	1.403712234	0.068235728	1.224194344	0.135634807
LOC319574|319574	1.403651984	0.298859436	1.267785937	0.377660013
Prkcq|18761	1.403377248	0.250440253	1.176928072	0.367056618
Col6a5|665033	1.403376716	0.358032949	3.3	0.173296754
Atp1b2|11932	1.401737131	0.168950262	1.738173047	0.059269753
Pcdhgb4|93701	1.401654008	0.387686767	1	#DIV/0!
Tmem79|71913	1.400949732	0.198290108	1.357658581	0.184495712
Cela2a|13706	1.400610955	0.285581	-1.36168866	0.078568306
Rbak|57782	1.400476448	0.225012303	1.734326902	0.123566277
Ppp1r3c|53412	1.400350331	0.043449129	1.343486997	0.000111711
Lrrc42|77809	1.400043293	0.014561619	1.498343912	0.008131867
Wdr91|101240	1.399883378	0.018947685	1.655766915	0.000085238
Psg23|56868	1.399745384	0.203319559	2.238544129	0.012421612
C030048B08Rik|269623	1.399400124	0.15300152	-1.04882757	0.385253224
Sox9|20682	1.399244899	0.066058938	1.549182811	0.037780502
Senp1|223870	1.398862163	0.171347591	-1.022317423	0.468778024
Jam2|67374	1.398526315	0.282053085	-1.118818431	0.415602248
Wdr89|72338	1.398046966	0.142280761	1.09887391	0.32063493
Abhd13|68904	1.397813297	0.073306137	1.455740935	0.002756559
Slamf7|75345	1.397339487	0.320838157	-1.071785009	0.465764816
Armcx2|67416	1.397323052	0.261380443	2.060191377	0.007945367
Il18r1|16182	1.397200771	0.02669261	1.448055668	0.005802332
Zdhhc1|70796	1.397033813	0.08969211	1.070907198	0.342167162
Wdsub1|72137	1.396732471	0.049148588	1.707646131	0.003496516
BC002230|217827	1.396486181	0.00795864	1.286019507	0.013531857
Cox7a1|12865	1.396447222	0.286365303	1.232044444	0.361067665
Stam2|56324	1.396372793	0.060984059	1.396372793	0.060984059
Csnk1g3|70425	1.396179709	0.061370183	1.503879065	0.028496796
Rpa3|68240	1.395835851	0.203729515	2.153265814	0.018641756
Impdh1|23917	1.395309044	0.111442794	1.24223798	0.21303372
Cdh3|12560	1.394942433	0.297823171	-1.090252881	0.446329168
Apoa1|11806	1.394648937	0.037961239	1.225104043	0.052879531
B9d2|232987	1.394146868	0.094119076	1.558774841	0.025809428
Cdkn2d|12581	1.394088241	0.230342023	2.03521418	0.07417965
Cdk2|12566	1.39382183	0.077326498	1.25865515	0.154654788
Nupr1|56312	1.393189784	0.161323783	1.393189784	0.161323783
Fam58b|69109	1.392999326	0.091927457	1.530547141	0.037502711
Nol8|70930	1.392850817	0.150070229	1.392850817	0.150070229
Rnf138|56515	1.392824627	0.093352185	1.629300964	0.034660211
Capn12|60594	1.392682411	0.205687103	1.622825377	0.064377159
Chchd2|14004	1.39256154	0.025297188	1.677036142	0.000484158
Rcc1|100088	1.392304812	0.047292497	1.262994302	0.096261802
Nupl1|71844	1.391259921	0.2569377	-1.006771463	0.494369797
Cln8|26889	1.391179193	0.020667258	1.596892232	0.000653363
Bak1|12018	1.390933519	0.015999639	1.613392352	5.77095E-05
Wbscr25|71304	1.390836727	0.302276732	-1.099650326	0.442407865
Lsm1|67207	1.390692335	0.110075999	1.368596306	0.092681323

Slc38a9|268706	1.38997561	0.109474772	1.38997561	0.109474772
Rab39|270160	1.389457302	0.347964429	1.195314368	0.428478238
C8b|110382	1.389212747	0.010236132	1.475877407	0.000912007
Ypel2|77864	1.388365271	0.003676366	1.390637432	0.000841385
Adat2|66757	1.388238576	0.181345074	1.38984008	0.068969996
Lor|16939	1.387953754	0.283363163	-1.301284902	0.17489906
1700048O20Rik|69430	1.387820893	0.27741802	1.291520216	0.23909431
Fam118b|109229	1.387655785	0.174046963	1.103801761	0.369355192
Snrpf|69878	1.38765416	0.07917831	1.561901914	0.011338939
Zfp870|240066	1.38759085	0.14107951	1.586080276	0.044078895
Pdgfc|54635	1.387509488	0.253488483	1.846260801	0.142154439
Cyp39a1|56050	1.387463386	0.071748376	1.581985742	0.003009222
Slc7a5|20539	1.38745561	0.144422018	1.586985531	0.046471325
Nxn|18230	1.387373962	0.152935837	1.21367985	0.114308126
Hes1|15205	1.386884442	0.157067296	1.39969936	0.132215489
Nrm|106582	1.386773538	0.225385307	1.843380978	0.105461215
Mcts1|68995	1.386593619	0.033463805	1.487106863	0.022000739
Trmt61b|68789	1.386447987	0.19095783	1.58547861	0.094374134
Cntf|12803	1.386338358	0.352653129	-1.660939759	0.284397279
Zfp51|22709	1.385267798	0.313249393	1.26304666	0.371654839
Dll4|54485	1.384873276	0.225223922	1.070811292	0.430119125
Gna15|14676	1.384514192	0.334955267	1.641393273	0.301430455
Snapc1|75627	1.384440112	0.042704895	1.511700334	0.007923519
Gm15421|100042049	1.38407586	0.032547422	1.572779774	0.005193794
Ppp6c|67857	1.383094267	0.080272598	1.383094267	0.080272598
Zfp760|240034	1.382785229	0.299954047	4.115709757	0.002664025
Gm11437|628813	1.382645201	0.22068191	2.235204596	0.018149289
Tax1bp3|76281	1.382167234	0.027392619	1.265864139	0.056824502
Cbl|12402	1.38201033	0.317611868	-1.173406417	0.410469846
Agps|228061	1.381888331	0.133080263	1.719949628	0.036532663
2210020M01Rik|66528	1.381734986	0.349738086	1.633421429	0.334663434
C1qtnf5|235312	1.381590675	0.185860896	1.261694588	0.131634801
Tead1|21676	1.381298575	0.057169881	1.74054975	0.000290679
2700081O15Rik|108899	1.381222255	0.13626359	1.652318357	0.058424842
Rras2|66922	1.381136188	0.114030988	1.192572255	0.233371982
Lins|72635	1.380992818	0.030886011	1.302096666	0.064079561
Gm10345|100041269	1.380414419	0.144303921	1.115748346	0.317043865
Stmn1|16765	1.38039923	0.108781721	1.180896052	0.227095328
BC022687|217887	1.380106635	0.246798527	1.163916234	0.367030839
Ppp1r32|67752	1.379892085	0.321041159	2.780872515	0.111971221
Kdm5b|75605	1.378887892	0.05133566	1.378887892	0.05133566
1110002L01Rik|100043040	1.378760724	0.182458454	1.371440301	0.075250003
Strn3|94186	1.378259969	0.141972046	1.445370985	0.073289199
Rpl22l1|68028	1.377927016	0.140130954	1.104216321	0.312483984
Bub1b|12236	1.377619555	0.34084237	2.365584001	0.182575382
Dtx3|80904	1.377163739	0.053001586	1.490307489	0.034335657
Frat1|14296	1.376902785	0.043677314	1.376902785	0.043677314
Lpin2|64898	1.376372632	0.042781561	1.376372632	0.042781561
Rce1|19671	1.375437773	0.050157057	1.226954506	0.094901196
Ces1a|244595	1.375387163	0.339208367	-1.465641937	0.303997643
Oip5|70645	1.374683544	0.34444809	2.424107143	0.19229339
Fastkd2|75619	1.374478055	0.113839805	1.548876571	0.031160822
Fam195b|192173	1.373792622	0.061269609	1.221511895	0.122357919

Mug2|17837	1.373675111	0.062349509	1.473536144	0.046423225
Bola2|66162	1.373632874	0.128365393	1.171196441	0.263718737
Rpp38|227522	1.373495246	0.296209009	1.970377453	0.137455265
Sirt1|93759	1.37304183	0.208868785	2.181260198	0.010270298
Gm6194|620966	1.372782482	0.212912525	1.045797334	0.44794724
1110006O24Rik|66123	1.372646503	0.169786739	1.706462061	0.069085724
Cep72|74470	1.37227945	0.345541793	2.458544324	0.189381453
Amica1|270152	1.372261549	0.260361294	2.242442651	0.060952075
Arhgef4|226970	1.3719189	0.388976427	-2.76078	0.173296754
Gm11968|100042173	1.37055569	0.089817377	1.047981285	0.348758311
Ppif|105675	1.370498904	0.001087256	1.296603083	0.001487398
2610002J02Rik|67513	1.370081687	0.003162522	1.50955002	0.000148601
Setd7|73251	1.369667668	0.103516386	1.78930741	0.005806303
Hspg2|15530	1.369091069	0.115236576	1.237320369	0.106688412
Sgk1|20393	1.368740089	0.116506252	1.198916739	0.232339128
Pigp|56176	1.36856045	0.047074824	1.211036601	0.076176391
Prkg1|19091	1.367654286	0.375827874	4.57358	0.173296754
Zfp655|72611	1.366713295	0.108992665	1.552703244	0.019793067
Rabggtb|19352	1.36551309	0.006003011	1.36551309	0.006003011
Gar1|68147	1.364703102	0.088266085	1.214645462	0.179582409
Brd9|105246	1.363442044	0.138481541	1.173116554	0.275151951
Lsm3|67678	1.363085051	0.187959287	2.194928092	0.003381248
Spon1|233744	1.362859078	0.303925207	1.861784853	0.19447366
3110009E18Rik|73103	1.362236821	0.315219873	-1.114180589	0.43641851
Wscd1|216881	1.36208651	0.204346412	-1.113823797	0.340409323
Cds1|74596	1.361541512	0.362279629	8.011083333	0.041592481
Haus2|66296	1.361361311	0.1729494	2.045272545	0.003789986
Slc4a1|20533	1.361316811	0.339122912	1.263632904	0.384713168
Ssh3|245857	1.361238838	0.016160074	1.1672619	0.018511932
Gm13306|100039863	1.360369942	0.334686754	6.352862915	0.000716936
Twist1|22160	1.36026632	0.278835097	2.098213132	0.105693718
Ppp3cc|19057	1.359744087	0.194921436	1.7782093	0.065357324
Acer3|66190	1.35938856	0.118933104	1.536204102	0.02980746
Arl5c|217151	1.359254928	0.293639921	2.046415927	0.139427098
Nup133|234865	1.358906392	0.082597932	1.744125147	0.002709953
Med21|108098	1.358438724	0.127486467	1.358438724	0.127486467
Cyp4a14|13119	1.358373333	0.124091569	1.358373333	0.124091569
Slmo2|66390	1.357959876	0.064869786	1.361990676	0.012190592
Ccnj|240665	1.357841631	0.200888629	1.042322362	0.443035771
Rasgrp4|233046	1.357802696	0.274515725	1.208097625	0.315824161
Slc17a1|20504	1.357398964	0.035868766	1.257061371	0.073352575
Mex3d|237400	1.357115058	0.16746054	1.626939405	0.081329265
Wdr35|74682	1.356671943	0.143662469	1.159173624	0.288210463
Ppp1r8|100336	1.356576985	0.051705162	1.356576985	0.051705162
Ube2a|22209	1.355976249	0.057032568	1.355976249	0.057032568
Zmynd19|67187	1.355929409	0.178663829	1.607443583	0.099332768
Mmp17|23948	1.355878163	0.230302419	1.355878163	0.230302419
Plcb2|18796	1.355235885	0.246094923	1.355235885	0.246094923
Haus5|71909	1.354945519	0.284068213	1.470776545	0.201520183
Ap4b1|67489	1.353971813	0.09236122	1.342937918	0.04318066
Nsd1|18193	1.353744226	0.002038404	1.498701285	1.80703E-05
Atp6ap2|70495	1.353452434	0.090501124	1.312645316	0.055555904
Clasp2|76499	1.353302104	0.018118375	1.353302104	0.018118375

Rps8|20116	1.352460032	0.062510097	1.192936907	0.117890047
Lsm10|116748	1.352383842	0.158727739	1.109804991	0.338591136
Ccl24|56221	1.35237494	0.099669424	1.740396588	0.008892536
Epm2a|13853	1.352001307	0.353769561	-1.653158046	0.25567537
Rrm2|20135	1.351958619	0.126271718	1.579091685	0.054205982
Cmtm4|97487	1.351697007	0.08964865	1.150543132	0.173589202
Arfip1|99889	1.351467142	0.051793937	1.295390943	0.026251792
Rpl35a|57808	1.350614716	0.106042108	1.142648344	0.221787363
Tmem2|83921	1.350035066	0.079452328	1.47433744	0.007579514
Cryl1|68631	1.34970719	0.121235003	1.223046899	0.208527504
Cyp4a10|13117	1.349671228	0.150322252	1.202338051	0.232975001
Zxdb|668166	1.349602436	0.259379272	-1.230810399	0.281407107
Taf1b|21340	1.349564812	0.226767642	-1.113027338	0.363607062
Nup37|69736	1.349388556	0.268683165	1.918049114	0.117829423
Ctla2b|13025	1.349297006	0.250863234	1.343300747	0.287173677
Igf1|16000	1.34837827	0.011158889	1.256447305	0.015297132
Ablim3|319713	1.348361356	0.04200757	1.435966228	0.029287904
Ier5|15939	1.348054487	0.181787059	1.078706257	0.389961804
Klhl6|239743	1.347885869	0.226454843	1.610533264	0.079583931
D0H4S114|27528	1.34755902	0.113501965	1.697005168	0.009287699
Sike1|66641	1.347100087	0.067181105	1.184095221	0.127903943
Snrpg|68011	1.346234924	0.110903104	1.346234924	0.110903104
Fmr1nb|207854	1.346211852	0.404078801	3.53972	0.173296754
5830415F09Rik|74753	1.346102186	0.20350651	-1.01949463	0.475762024
Map1lc3a|66734	1.345464526	0.03361466	1.230986165	0.062323825
Dgkd|227333	1.345287995	0.027830615	1.345287995	0.027830615
Acot1|26897	1.345216581	0.050063144	1.451758519	0.029862112
Lamc1|226519	1.343987228	0.043203993	1.343987228	0.043203993
Sema7a|20361	1.342639675	0.362599296	1.282144901	0.403677687
Msl3l2|73390	1.34257337	0.272332789	1.597511955	0.146144483
Serpine2|20720	1.34206431	0.147519292	1.671389326	0.035744342
Gpc1|14733	1.341878663	0.196080381	1.785269264	0.049676939
Il17rb|50905	1.341766899	0.076140971	1.240977736	0.140021744
Armc2|213402	1.341387134	0.286454329	1.341387134	0.286454329
E130112N10Rik|78668	1.341040048	0.321737386	1.341040048	0.321737386
Klhdc2|69554	1.340956481	0.104326306	1.340956481	0.104326306
Gm826|329554	1.340547393	0.239556218	1.61395093	0.156256445
1810063B07Rik|67509	1.34001544	0.069206153	1.168478163	0.123569811
Tcea1|21399	1.339968571	0.040213209	1.188625758	0.041440837
H2afx|15270	1.339826968	0.114004269	1.339826968	0.114004269
B9d1|27078	1.339802596	0.306365222	2.156277011	0.125131424
Zswim1|71971	1.339625858	0.147814668	1.808545507	0.003710229
Arhgap5|11855	1.339556029	0.31732865	2.936487147	0.047820025
BC067068|216292	1.33940019	0.27372647	1.870879121	0.128139235
1700086O06Rik|73516	1.339247466	0.18679342	1.72502717	0.055167574
Kdelc1|72050	1.338917752	0.093180005	1.068052166	0.343075477
Ap4s1|11782	1.338642836	0.09243582	1.2679656	0.043226124
4930506M07Rik|71653	1.338635436	0.012281539	1.240710275	0.022410113
Trim47|217333	1.338250726	0.1815359	-1.068079385	0.337023301
Helb|117599	1.338030552	0.131218934	1.338030552	0.131218934
Cdc37l1|67072	1.337972465	0.056676698	1.693733558	8.33092E-05
0610009B22Rik|66050	1.337883213	0.063180472	1.523116098	0.015226865
Rps9|76846	1.337873195	0.12253146	1.113944867	0.26419511

Hspbap1|66667	1.337854815	0.144333551	1.025787862	0.451695278
Cyp2c68|433247	1.33751581	0.008408766	1.383368421	0.001548213
Fmo4|226564	1.33717119	0.165993534	1.600935374	0.011715063
Zbtb6|241322	1.336857469	0.138675646	1.336857469	0.138675646
Zw10|26951	1.336116258	0.121783719	1.116771799	0.262279617
Arf2|11841	1.336086201	0.078185902	1.195474776	0.044513196
Procr|19124	1.335826314	0.20166961	1.335826314	0.20166961
Zfp738|408068	1.3354431	0.223458335	1.548137552	0.091222488
Exosc8|69639	1.334946265	0.105226714	1.216425959	0.187864301
Accs|329470	1.334775682	0.197431426	1.531053017	0.084592589
Aimp1|13722	1.334624786	0.036461564	1.334624786	0.036461564
Gabarapl2|93739	1.334236654	7.15116E-05	1.350810571	0.000023648
1500011B03Rik|66236	1.334172098	0.167320985	1.348772634	0.124779713
Fkbp10|14230	1.334016502	0.212519748	1.334016502	0.212519748
Sox7|20680	1.333997301	0.08599546	1.452565238	0.031803714
Eps15|13858	1.333908471	0.055124034	1.420383388	0.03966891
H2-Ab1|14961	1.333889699	0.172403395	1.022444016	0.438759934
Pank2|74450	1.333738129	0.058511229	1.647611219	0.000241989
Prkch|18755	1.333354483	0.221979179	1.033479292	0.459778453
Glrx5|73046	1.333243688	0.01763666	1.42610756	0.00012261
Ccdc162|75973	1.333097717	0.136939679	1.34094776	0.17750103
Ints8|72656	1.332954834	0.263341591	1.009863622	0.49115838
Rab12|19328	1.332450609	0.071585078	1.395094898	0.022748765
Snx10|71982	1.332419741	0.116271159	1.518214503	0.015702194
Mrpl23|19935	1.332336435	0.163798245	1.242137076	0.100200188
Cabyr|71132	1.33208454	0.187142612	1.33208454	0.187142612
Tmem48|72787	1.331939504	0.302373151	1.331939504	0.302373151
Fam18a|383103	1.331768658	0.289287336	2.400344436	0.044420576
Loh12cr1|67774	1.331323908	0.096227085	1.561815385	0.022192538
Tmem41a|66664	1.331304236	0.064076371	1.331304236	0.064076371
Peg10|170676	1.330249549	0.234777422	1.116683499	0.378914291
Tmem87b|72477	1.330228958	0.084285913	1.724318323	0.000254854
Cdc73|214498	1.329716659	0.115059444	1.551359636	0.038907117
Rrs1|59014	1.329664745	0.04256029	1.199986926	0.073230531
Ubn1|170644	1.329561277	0.009707352	1.245522671	0.013061471
Patz1|56218	1.329225233	0.155972096	1.528816781	0.085972628
Urb1|207932	1.328915205	0.272972101	-1.167589566	0.294506566
Sdad1|231452	1.32887523	0.100736997	1.32887523	0.100736997
Pkdcc|106522	1.328624036	0.013066653	1.5073956	0.000530167
Tsga10|211484	1.328364436	0.379094624	1.621721864	0.33676256
Isyna1|71780	1.328276577	0.091439756	1.721312433	0.001368978
Pdss1|56075	1.328049201	0.307259316	-1.412454118	0.223939638
Lass6|241447	1.328024932	0.263080526	1.059609134	0.450477882
Ptgfr|19220	1.327846269	0.36327268	1.016451995	0.491391961
Susd2|71733	1.327810305	0.225188551	1.987120046	0.017774769
Slc30a4|22785	1.327628073	0.243004911	1.327628073	0.243004911
Clpx|270166	1.327513056	0.076178613	1.236125909	0.138300966
Rnf215|71673	1.327127914	0.022161217	1.482897728	0.001393208
Ubac1|98766	1.326069157	0.043848475	1.207077288	0.083514526
Bcl10|12042	1.32598021	0.047204038	1.226498156	0.096638185
Ube2u|381534	1.325855274	0.212593412	1.474594225	0.096296417
Mipol1|73490	1.325633367	0.349009283	-1.605913973	0.215079121
Nsl1|381318	1.325600743	0.300561326	2.030178782	0.124330777

Kdm1a|99982	1.325298047	0.029425946	1.340322029	0.007981237
Slc2a6|227659	1.325273224	0.326824593	1.325273224	0.326824593
Esrp1|207920	1.325150865	0.366877839	-1.417117702	0.354892428
2010111I01Rik|72061	1.324763504	0.092464269	1.138181617	0.182795256
Fert2|14158	1.324474003	0.266031123	2.092427303	0.050130648
Phf5a|68479	1.324278003	0.03819952	1.114579811	0.118531704
Nnmt|18113	1.324146344	0.101303156	1.047024691	0.381540861
Lsm14a|67070	1.323658714	0.105727747	1.519166059	0.038802023
Rpl9|20005	1.323515939	0.10576529	1.147859693	0.221911867
Tinagl1|94242	1.3234238	0.020527821	1.373069792	0.013876022
Pigw|70325	1.323230314	0.172085626	1.323230314	0.172085626
Nova2|384569	1.322981809	0.246825965	1.199536828	0.164929423
Cdyl|12593	1.321977596	0.308733017	1.654810424	0.176968877
Gm6222|621381	1.321725939	0.088922914	1.352105305	0.021117256
Rbm45|241490	1.321582888	0.15563099	1.142853546	0.201810487
Med7|66213	1.32150484	0.11282826	1.716838796	0.004487871
Gtpbp10|207704	1.32145793	0.141956782	1.32145793	0.141956782
Serpina3k|20714	1.32125328	0.155726265	1.805170594	0.005075358
Birc5|11799	1.321204839	0.199866337	-1.054521738	0.421071723
Rasa4|54153	1.320993595	0.262140185	-1.001173652	0.49887128
Nudcd1|67429	1.320643163	0.24029007	1.320643163	0.24029007
Arl2bp|107566	1.319983224	0.086201963	1.077139749	0.31529962
AI597468|103266	1.319876118	0.262515653	1.193406236	0.338996245
Sdr9c7|70061	1.319534038	0.027517549	1.243826162	0.057345173
Nfil3|18030	1.319513436	0.056461348	1.22276576	0.111325988
Bnip3|12176	1.319475579	0.017156073	1.417204844	0.000901685
Mat1a|11720	1.319336024	0.091816062	1.410549083	0.069501786
Nt5dc2|70021	1.318831704	0.167091749	1.230282473	0.14303055
Cd2|12481	1.318559409	0.280251274	1.286487709	0.303166733
Fam76b|72826	1.318395245	0.239972111	1.153688785	0.368542004
Snrnp48|67797	1.318238672	0.079464728	1.447831937	0.017808006
Plscr1|22038	1.317953812	0.158691399	1.165868882	0.274566156
Rasl10b|276952	1.317796154	0.22594826	2.007147433	0.011562315
Lmbrd2|320506	1.317025714	0.413546139	1	#DIV/0!
Dusp1|19252	1.316840875	0.116430618	1.759838203	0.000892811
Orc2|18393	1.316289001	0.137734192	1.316289001	0.137734192
Mical3|194401	1.315952104	0.121809512	1.315952104	0.121809512
Snrpe|20643	1.315892847	0.096202725	1.180664841	0.1871235
Surf6|20935	1.315817875	0.07180487	1.315817875	0.07180487
Usp42|76800	1.315767833	0.302423474	1.51733978	0.154110183
AU019823|270156	1.314945574	0.09904878	1.177226094	0.201026674
Hsbp1l1|66255	1.314884361	0.145716093	1.445638676	0.05068447
Scube1|64706	1.314429009	0.34207906	-1.412498427	0.282553547
Cps1|227231	1.313938251	0.224406693	1.627366723	0.110611638
Ubxn7|224111	1.313740591	0.092348204	1.389015362	0.003732769
Mbnl2|105559	1.313307469	0.132632986	1.410105756	0.039955139
Col16a1|107581	1.312807881	0.101413835	1.498142595	0.005545439
Slc22a3|20519	1.312772605	0.213019308	-1.08143516	0.376620663
Rlf|109263	1.312748899	0.194550914	1.851970532	0.020295597
Eif2b4|13667	1.312612405	0.01220745	1.36576626	0.005983813
Slc25a22|68267	1.312034606	0.06187691	1.312034606	0.06187691
H3f3a|15078	1.311988626	0.00073643	1.31261444	0.000255742
Gstp2|14869	1.311383903	0.259325007	1.992257008	0.047176275

Il11ra1|16157	1.311289683	0.062890641	1.151451901	0.09325275
Map3k5|26408	1.311227608	0.036637781	1.40852425	0.003965919
Nop58|55989	1.311024479	0.166070322	1.19470213	0.26038195
Ythdf2|213541	1.310672715	0.005857494	1.399373327	0.000990158
Dsc2|13506	1.309823883	0.126301383	1.286913217	0.049619188
Xrcc2|57434	1.309774895	0.311106927	-1.096428588	0.417307835
Npm3-ps1|108176	1.309760356	0.118310478	1.485768698	0.053007316
Sarm1|237868	1.309578571	0.303473865	1.309578571	0.303473865
Msln|56047	1.309578571	0.303473865	1.309578571	0.303473865
Celf4|108013	1.309477819	0.388102049	2.327685805	0.199681503
R74862|97423	1.30933296	0.105839337	1.496359664	0.037534667
Jrk|16469	1.309165646	0.204488037	1.095259498	0.370001762
Suv420h2|232811	1.309091816	0.006007346	1.223903313	0.002321666
Pi4k2b|67073	1.309041294	0.187253353	1.516633707	0.107921726
Jund|16478	1.308766777	0.130441884	1.015336965	0.463975821
Ccng2|12452	1.308322813	0.169466721	1.099848205	0.34898321
Sclt1|67161	1.308170015	0.248322748	1.499685166	0.139667529
Slc36a4|234967	1.308068514	0.369438342	1.487826752	0.329104443
Rps14|20044	1.307978789	0.155089801	1.066101339	0.355642106
Ugt1a9|394434	1.307610884	0.146922805	1.009297939	0.480239337
Wnt5a|22418	1.307572893	0.237606545	-1.006281101	0.491913716
Sms|20603	1.307393534	0.129802487	1.307393534	0.129802487
Atg16l2|73683	1.307390964	0.181569272	1.693303974	0.031489145
Mapre1|13589	1.30661037	0.017557078	1.388840008	0.007744322
Dtwd2|68857	1.306479311	0.249161664	1.056538931	0.444698072
Kpna3|16648	1.306285693	0.136538455	1.14498485	0.273063259
Pcgf2|22658	1.306182186	0.067437402	1.162751209	0.12896825
Tmem229b|268567	1.306152991	0.259888614	1.761317976	0.105510624
Hdac2|15182	1.305885187	0.022629627	1.305885187	0.022629627
Lrrk2|66725	1.305735556	0.386700135	9.793016667	0.046113179
Ttpa|50500	1.305565496	0.001004642	1.346746191	0.000715902
Gm10012|100043160	1.30517788	0.100299915	1.30517788	0.100299915
Pitpnm1|18739	1.305034219	0.134400637	1.154045034	0.250983365
Nkg7|72310	1.304936512	0.245681173	-1.020905539	0.474007924
Twistnb|28071	1.304799939	0.168501732	-1.045688966	0.404587289
Hspa12b|72630	1.304487883	0.267306189	-1.041970602	0.456531367
Arntl|11865	1.304312481	0.113017713	1.468429271	0.00222741
Ralb|64143	1.303803705	0.093573919	1.138954727	0.191933326
Rps28|54127	1.303757119	0.133450255	1.11930878	0.282724936
Zfp773|76373	1.303751035	0.30049496	1.303751035	0.30049496
Phyhipl|70911	1.303687472	0.407978939	1	#DIV/0!
1810041L15Rik|72301	1.303687472	0.407978939	1	#DIV/0!
Ddx43|100048658	1.303687472	0.407978939	1	#DIV/0!
Rtkn|20166	1.303034302	0.128451058	1.303034302	0.128451058
Il4i1|14204	1.302437028	0.346195753	2.653032424	0.103806698
Csrp3|13009	1.302313659	0.125577048	1.376148653	0.108315994
Hap1|15114	1.302226876	0.129178064	1.302226876	0.129178064
Mcl1|17210	1.301780064	0.026407441	1.201322094	0.042046542
Mgat5|107895	1.301425722	0.336198347	1.959518702	0.182816276
1110017F19Rik|68528	1.301388731	0.329558967	1.225643903	0.36366942
Krtcap3|69815	1.301122059	0.29147	1.382105023	0.227181499
Gm13253|664903	1.300718773	0.171014209	1.208828458	0.146615196
Gspt2|14853	1.300691639	0.317752496	1.300691639	0.317752496

Acsl4|50790	1.300679663	0.104056308	1.18128699	0.194254506
Atp7a|11977	1.300564975	0.126540605	1.73915065	0.001533265
Gpr162|14788	1.300485388	0.279143885	1.363385568	0.207085746
Lrp6|16974	1.300442754	0.039087206	1.481612964	0.001245551
Rpl10a|19896	1.300433953	0.097604545	1.17625131	0.195908269
Zfp945|240041	1.300226889	0.108575121	1.428428166	0.03494535
Nob1|67619	1.300128819	0.063138616	1.381837151	0.029800875
Bax|12028	1.299808592	0.084768013	1.299808592	0.084768013
D1Ertd622e|52392	1.299771578	0.205782693	1.817992916	0.018256542
Gmps|229363	1.299729997	0.017385593	1.371347744	0.00413943
Trp53inp1|60599	1.299601442	0.056903687	1.597075844	0.000163372
Wbscr27|79565	1.299473027	0.011518323	1.245244279	0.002024828
Nuak1|77976	1.299449497	0.270676545	1.299449497	0.270676545
Tbxa2r|21390	1.299076529	0.215684564	1.698501371	0.058561073
Sytl4|27359	1.299002048	0.353766015	2.384759398	0.152225637
Ctse|13034	1.298609387	0.032812268	1.189019495	0.051362471
Egfr|13649	1.298530526	0.075916422	1.285730312	0.046835409
Pla2g12a|66350	1.298159264	0.024405851	1.298159264	0.024405851
Lmnb1|16906	1.298086872	0.146644951	1.298086872	0.146644951
Lrp12|239393	1.298057362	0.301449041	1.151040157	0.376503807
Dram1|71712	1.297796403	0.359714671	2.714109596	0.129040948
Hilpda|69573	1.29744457	0.204667867	1.160950271	0.174992222
Zfp322a|218100	1.297327839	0.198392599	1.050009659	0.423312355
Dph5|69740	1.297278659	0.127592199	1.477920335	0.054843354
Slamf9|98365	1.29683233	0.325374571	1.29683233	0.325374571
Tmem161b|72745	1.296741569	0.314291111	-1.31535848	0.209942938
Polr2l|66491	1.296596074	0.087304017	1.169188178	0.179001915
Rps24|20088	1.29653728	0.212179659	1.001531293	0.497303756
Hoxa4|15401	1.29651352	0.28569814	1.543052249	0.147966979
Kcnk6|52150	1.296226506	0.264717584	-1.025748713	0.472639823
Asb2|65256	1.296031159	0.235161176	1.825883773	0.006194697
Qtrt1|60507	1.295998223	0.133538899	1.46676914	0.064572029
Dcaf12|68970	1.295846812	0.046005225	1.295846812	0.046005225
5930416I19Rik|72440	1.295674305	0.061647724	1.173078654	0.125941386
Ankrd33b|67434	1.2954062	0.123262911	1.450924916	0.02628547
Ttc23|67009	1.295403056	0.013146872	1.286446918	0.004625176
Ptbp2|56195	1.295147939	0.33462305	1.295147939	0.33462305
Rnf214|235315	1.295014824	0.038458357	1.295014824	0.038458357
Wfdc15b|192201	1.294634254	0.390120286	1.633421429	0.334663434
Scml4|268297	1.294625137	0.361359982	2.596338175	0.142234751
Cpeb2|231207	1.2945435	0.246237371	1.708312514	0.055688574
Sla2|77799	1.2944655	0.354060314	1.716508186	0.234913993
Slc17a8|216227	1.294452852	0.240537901	1.778380364	0.058923634
Utp11l|67205	1.29432847	0.131592169	1.568334176	0.019766562
Rpl37a|19981	1.294285854	0.21008009	1.151651667	0.197164371
Hhat|226861	1.294158081	0.318403107	1.601299082	0.165097903
Cd9|12527	1.293793098	0.185616347	1.701754199	0.020512222
Cnnm2|94219	1.293585153	0.117618409	1.128403071	0.247791745
Sco1|52892	1.293493014	0.058613217	1.250239357	0.097986723
Ddx52|78394	1.292670181	0.137622113	1.292670181	0.137622113
Myom1|17929	1.292436155	0.17926307	-1.024729294	0.453497657
Dmxl2|235380	1.292268761	0.228126255	1.729034679	0.056108548
Slc19a1|20509	1.291957851	0.018687497	1.291957851	0.018687497

Tnpo1|238799	1.291598191	0.241062655	1.481447118	0.098077756
Hmox1|15368	1.291425314	0.180548714	1.29744383	0.134372834
Rhbdf2|217344	1.291278176	0.030420868	1.11096233	0.089140707
Uqcrh|66576	1.291176578	0.121881842	1.103156411	0.262495948
Ip6k2|76500	1.291071243	0.036904147	1.379628034	0.007912898
Rad1|19355	1.29101872	0.184849115	1.280311884	0.156845426
Llgl1|16897	1.290795171	0.162641948	1.045200301	0.38588014
Amy1|11722	1.29054254	0.028500358	1.174319335	0.04607237
Rnf219|72486	1.290027758	0.305396813	1.670431061	0.148097804
Smc2|14211	1.289931855	0.359863659	-1.185670432	0.415689945
Pigv|230801	1.28953103	0.112908708	1.510808273	0.02269094
Zcrb1|67197	1.289439653	0.11019962	1.289439653	0.11019962
Psmd9|67151	1.289403346	0.024280808	1.289403346	0.024280808
Oraov1|72284	1.288904346	0.072343868	1.331785367	0.02039887
Mudeng|74385	1.288825606	0.299189023	1.809905415	0.136149862
Ddx3x|13205	1.288814754	0.127557692	1.474223726	0.015287814
Terf2|21750	1.288565419	0.046015673	1.184602268	0.103145943
Rab21|216344	1.288440697	0.06520757	1.080797491	0.243316103
Plin4|57435	1.288330584	0.01832248	1.201051933	0.026623928
Sh3gl1|20405	1.287897598	0.004639181	1.294457707	0.001541191
Grhl1|195733	1.287650334	0.355901861	-1.123925492	0.440140822
Tuba1a|22142	1.28757012	0.237870216	1.633372865	0.099194172
Hsp90aa1|15519	1.287556334	0.060118096	1.261890023	0.057279333
Ryk|20187	1.28719371	0.007751035	1.153108268	0.00433372
Gtf2h1|14884	1.287007318	0.049142878	1.537809388	0.00013957
F3|14066	1.286804593	0.282694682	1.081664273	0.423683423
4931406P16Rik|233103	1.286632905	0.139930454	1.702802514	0.003574371
Sertad1|55942	1.2861694	0.213578138	-1.11879517	0.225970574
Kctd3|226823	1.28612668	0.118528782	1.28612668	0.118528782
Fkbp5|14229	1.285516276	0.140557687	1.285516276	0.140557687
Rpp40|208366	1.285442964	0.099928066	1.341285012	0.090211124
Pigx|72084	1.285412196	0.097177165	1.285412196	0.097177165
St6galnac2|20446	1.285392311	0.333613732	1.426600316	0.296746291
Rplp2|67186	1.285391623	0.215661171	1.102929402	0.268775755
Taf13|99730	1.285321685	0.044537329	1.263008791	0.037927078
Gm4794|215895	1.285239263	0.355460197	1.612368991	0.26211305
Usp27x|54651	1.285109295	0.179754251	1.480663267	0.094752878
Mrpl40|18100	1.284947273	0.005787461	1.326518909	0.004684033
Ehd2|259300	1.284730092	0.142790782	1.284730092	0.142790782
Armc8|74125	1.284642098	0.074773353	1.443424593	0.018226996
Cd34|12490	1.283925672	0.333547786	1.333920548	0.271050335
Cyp17a1|13074	1.28381985	0.208748069	1.00340356	0.493521247
Bace1|23821	1.283636434	0.174587316	1.560680393	0.050145655
Dynlt1b|21648	1.283328158	0.201745811	1.32141306	0.099853631
Apoo|68316	1.283266495	0.223300697	1.046745975	0.439090039
Calhm2|72691	1.283060828	0.291239856	-1.279452049	0.246895161
Uchl3|50933	1.28290389	0.143070555	1.634817265	0.010106274
4732471D19Rik|319719	1.2828841	0.291350207	-1.179361859	0.292541716
Tbc1d8|54610	1.282752601	0.127095558	1.028932166	0.422228209
2310008H09Rik|66356	1.282580049	0.000312447	1.22891572	0.000141346
Tmem203|227615	1.282518519	0.008016988	1.194436129	0.008124054
Tbl2|27368	1.282470152	0.012009457	1.22323835	0.011160277
Leprel4|66180	1.282436085	0.267349622	1.966835104	0.031157641

Epsti1|108670	1.282377439	0.176279145	1.714252037	0.013654318
Wdr77|70465	1.282239797	0.032011077	1.406717806	0.003532002
Fam180a|208164	1.28220422	0.318987859	1.078297343	0.440841645
Esf1|66580	1.282058498	0.214010811	1.881937229	0.006225763
Ihh|16147	1.281817362	0.265211809	1.281817362	0.265211809
Fbxo17|50760	1.281711601	0.294668054	1.525330155	0.155435693
Atp6v1g1|66290	1.281638801	0.046739449	1.336700026	0.010991427
Camsap1|227634	1.281605029	0.101044509	1.390314674	0.01553566
Cpt1b|12895	1.281357931	0.084285966	1.077126492	0.295076115
Lat|16797	1.280907509	0.293708485	1.635050281	0.17266186
Cyp4a31|666168	1.280414533	0.187291619	1.13295043	0.314773395
LOC106740|106740	1.280373388	0.356012866	1.964827528	0.202463383
Map4k4|26921	1.279967804	0.164945931	1.668794333	0.005033898
Med12l|329650	1.279625103	0.360844391	-1.070040211	0.449808949
Rnpc3|67225	1.279454384	0.23514239	1.902110763	0.020343554
Rerg|232441	1.279357869	0.227683703	1.19422052	0.135741673
Uaca|72565	1.279206963	0.131266153	1.131173416	0.266153431
E2f8|108961	1.27918947	0.112022676	1.13122635	0.233843583
Hic1|15248	1.279014449	0.286870241	1.26911991	0.284628489
Hsf2|15500	1.278664328	0.110048366	1.165705918	0.214030584
Mri1|67873	1.278598594	0.077827304	1.278598594	0.077827304
Zfp830|66983	1.2785636	0.021564867	1.326944029	0.003337026
Ccdc115|69668	1.277762219	0.021169963	1.282214351	0.009238137
Gmnn|57441	1.277592662	0.148825831	1.277592662	0.148825831
Epdr1|105298	1.277499476	0.361701497	3.041166332	0.075689291
Nudt16|75686	1.277348645	0.02906797	1.389585957	0.005472633
Idh3a|67834	1.277152445	0.137906311	1.595860269	0.008170057
Usp33|170822	1.277005632	0.098900606	1.496812282	0.01036499
Tmod1|21916	1.276805907	0.186579066	1.119481874	0.324328707
Rusc1|72296	1.276792943	0.103542422	1.424079618	0.014363489
Yy1|22632	1.276533327	0.280469378	-1.079012725	0.414661706
Anapc10|68999	1.276048879	0.26388262	1.069175846	0.434337871
D330050I16Rik|414115	1.275998931	0.254775449	-1.047990959	0.434345656
Ccdc58|381045	1.275701924	0.039959212	1.36709544	0.00662747
Pgm1|66681	1.275641193	0.2877669	1.629854466	0.160052382
Rgs2|19735	1.275481234	0.125348805	1.299530416	0.074435692
Unc5b|107449	1.275357982	0.078074705	1.182278703	0.155432277
Trappc6a|67091	1.27507642	0.066613585	1.27507642	0.066613585
Gtf2h4|14885	1.275012314	0.039735641	1.380561161	0.012466469
Rpl13a|22121	1.274978556	0.205055614	1.012224795	0.476494282
Herpud1|64209	1.274931541	0.141020456	1.51074395	0.031972921
Plac8|231507	1.274885839	0.128290791	1.190158425	0.219210404
Srgn|19073	1.274750768	0.077960605	1.370066849	0.02729245
Dnajb1|81489	1.27465715	0.089117427	1.429574569	0.003107344
Micall2|231830	1.274366232	0.196411751	1.351895526	0.127286446
Cct8|12469	1.274141294	0.012628308	1.203573327	0.020060727
Cdc25a|12530	1.274133149	0.276954553	1.274133149	0.276954553
Cited4|56222	1.273832238	0.263159957	1.520254552	0.097756019
E330009J07Rik|243780	1.273324632	0.230992257	1.273324632	0.230992257
Ces1b|382044	1.27321326	0.142699229	-1.035427045	0.306942854
Ranbp9|56705	1.273134466	0.181628215	1.737184613	0.003753421
Rbm8a|60365	1.272714209	0.027396108	1.327332708	0.013860822
Rps12|20042	1.272298041	0.055208805	1.190204517	0.112441828

Sac3d1|66406	1.272204804	0.050710962	1.273049243	0.004934954
Arl16|70317	1.271741806	0.163820442	1.271741806	0.163820442
Sema3c|20348	1.271603616	0.414863585	-2.8	0.173296754
Rsl24d1|225215	1.271552861	0.056203784	1.172432061	0.113875697
Gstp1|14870	1.271354677	0.228660311	1.355342765	0.077752591
Lpin1|14245	1.270998847	0.257276139	1.193736368	0.256131255
Tomm34|67145	1.270969341	0.035584918	1.270969341	0.035584918
AI428936|233066	1.270648223	0.329591654	1.073204424	0.451406879
Rnf41|67588	1.270556237	0.092269985	1.132771389	0.190853416
Gm6484|624219	1.270555665	0.334717364	-1.62315939	0.043210847
Rbfox3|52897	1.270541601	0.273567053	1.020787432	0.460858458
Htra3|78558	1.270530537	0.198508936	1.262785654	0.111614711
St8sia1|20449	1.27049673	0.388481082	1.264185714	0.40907038
Dapk2|13143	1.270280682	0.104529301	1.40962116	0.01558745
Lingo1|235402	1.270173511	0.400672151	4.577104461	0.132622695
Sox12|20667	1.269739036	0.05088207	1.176140252	0.104006185
Plac9|211623	1.269345693	0.288131551	-1.178804432	0.255346841
Slc35f5|74150	1.269206089	0.059843105	1.244280518	0.026484847
2610002I17Rik|72341	1.269177094	0.259471335	1.100873314	0.354544757
Cldn1|12737	1.269144181	0.166765479	1.269144181	0.166765479
4932415G12Rik|67723	1.268730175	0.295597274	-1.022205596	0.480336809
1700094D03Rik|73545	1.268725017	0.319169413	-1.327083648	0.152383082
Tbc1d7|67046	1.268681199	0.082939107	1.51518745	0.004501555
Pum2|80913	1.268673299	0.04099643	1.346041229	0.003764333
Zfp369|170936	1.268640853	0.371855314	1.268640853	0.371855314
Bin1|30948	1.268566771	0.134254746	1.272308802	0.058355935
Adam33|110751	1.268414714	0.339416302	-1.252399805	0.336793742
Thg1l|66628	1.268237874	0.285371268	1.061665172	0.437870818
Bri3bp|76809	1.268192004	0.027186612	1.200025049	0.056952373
Rinl|320435	1.268127923	0.269499734	1.017289461	0.482093556
Akirin2|433693	1.268046746	0.024591756	1.199060471	0.051661147
Eif2b3|108067	1.267869318	0.215380452	-1.107908897	0.292979294
Hist2h2be|319190	1.267358307	0.241153855	1.039861831	0.45142649
Ccdc163|68394	1.267336621	0.259944077	2.020904544	0.013016348
Fam50b|108161	1.267208703	0.414822897	-7.703466667	0.044331703
Fgr|14191	1.267081816	0.220423381	1.515765937	0.032032161
Gm12166|100039624	1.266880331	0.288651989	1.062154148	0.43852334
Mgat2|217664	1.266838914	0.157549265	1.267734096	0.09378979
Ms4a4d|66607	1.266628587	0.18636661	1.266628587	0.18636661
Rnaseh2b|67153	1.266607527	0.21800673	1.294282978	0.150916351
Dnajc15|66148	1.266382767	0.1014847	1.266382767	0.1014847
Cage1|71213	1.265975449	0.35084738	1.265975449	0.35084738
Tas1r1|110326	1.265975449	0.35084738	1.265975449	0.35084738
Zfp560|434377	1.265937252	0.241100924	1.265937252	0.241100924
Lpar6|67168	1.265895964	0.107486549	1.444578996	0.026465671
Polb|18970	1.265750612	0.132003817	1.349907414	0.101097691
Slc13a5|237831	1.265463244	0.288160701	1.516168308	0.128180681
Sprn|212518	1.265445051	0.231422777	1.404412836	0.14034196
Rad9b|231724	1.265173454	0.230759763	1.582310324	0.085033782
Arhgap11a|228482	1.264880129	0.252657212	1.21513463	0.265243956
Nfkbil1|18038	1.264835183	0.280522112	-1.128067647	0.329804968
Mcart1|230125	1.264724312	0.076477187	1.280731601	0.04373857
1110007C09Rik|68480	1.264183719	0.073051068	1.177094144	0.141117734

Zfp511|69752	1.263987633	0.132664449	1.354639138	0.075626285
Rpl18|19899	1.263958369	0.197175567	1.189959809	0.178058253
Akap2|11641	1.263641191	0.037171126	1.263641191	0.037171126
Tmem139|109218	1.263565863	0.396012066	1.59986242	0.353323128
6330409N04Rik|66674	1.263464609	0.14546113	1.335235565	0.029657642
Hprt|15452	1.263439124	0.059066386	1.468669787	0.003271988
Plin5|66968	1.263306838	0.060810534	1.202470841	0.11658769
Prune2|353211	1.26328075	0.374045171	1.26328075	0.374045171
Zfp618|72701	1.26328075	0.374045171	1.26328075	0.374045171
Rgs11|50782	1.263086079	0.40305271	1	#DIV/0!
F630028O10Rik|100038363	1.263086079	0.40305271	1	#DIV/0!
Oaz3|53814	1.263086079	0.40305271	1	#DIV/0!
Fam19a1|320265	1.263086079	0.40305271	1	#DIV/0!
Gm3285|100041351	1.263086079	0.40305271	1	#DIV/0!
1810033B17Rik|69189	1.263086079	0.40305271	1	#DIV/0!
Mboat1|218121	1.263086079	0.40305271	1	#DIV/0!
A030009H04Rik|80515	1.263014841	0.386157635	-1.363516108	0.370712423
Tgfbr2|21813	1.263010095	0.235555801	1.049345712	0.438140878
Bbip1|66360	1.262942118	0.086148389	1.388422766	0.011122374
Nup205|70699	1.262829896	0.289338624	2.00366706	0.034966747
Lcmt2|329504	1.262493736	0.082168963	1.381068546	0.012593806
Dnase2a|13423	1.262439179	0.15054663	1.282431634	0.096690824
B020018G12Rik|545388	1.262403994	0.126280298	1.395635595	0.032391857
Gm6762|627480	1.262074581	0.289043436	1.48831481	0.203074199
2210021J22Rik|72355	1.261775966	0.098648886	1.181586369	0.123474688
Flcn|216805	1.261703028	0.046866711	1.165503781	0.090995254
Pus7|78697	1.261135241	0.048214783	1.173962495	0.097721933
Mppe1|225651	1.26109192	0.147892885	-1.003909312	0.489677631
Filip1|70598	1.260297682	0.409958295	-3.967316667	0.09324139
4430402I18Rik|381218	1.259914203	0.293987773	1.067027507	0.435388417
Fh1|14194	1.259536076	0.081016275	1.279323087	0.037091131
5730455P16Rik|70591	1.259258205	0.152520768	1.557678019	0.009908643
2510009E07Rik|72190	1.259257782	0.109646755	1.219995357	0.071253745
Ptges3|56351	1.259186443	0.110545389	1.188040503	0.115549505
Vasp|22323	1.259099106	0.024659663	1.184577555	0.046266529
Tmco7|272538	1.259095266	0.129478084	1.156654866	0.23820214
Mtfmt|69606	1.258997758	0.144824639	1.432583494	0.015564548
1700020I14Rik|66602	1.258930963	0.074674412	1.240577443	0.032674346
Npm2|328440	1.258566919	0.409043451	4.37142	0.173296754
Birc2|11797	1.258154825	0.084132852	1.111986026	0.169876097
Fyn|14360	1.258091137	0.088065632	1.187295912	0.15513608
Soat1|20652	1.257942179	0.181218505	1.257942179	0.181218505
Anxa5|11747	1.257872577	0.071473909	1.182498728	0.051393185
Man1c1|230815	1.257746997	0.03011664	1.245480488	0.018686807
Ass1|11898	1.257648142	0.046960211	1.156243996	0.086263546
Mtrr|210009	1.257595185	0.047351307	1.141108333	0.065586948
Zfp14|243906	1.257328325	0.392927559	-2.303330578	0.124495049
Slc37a4|14385	1.25711406	0.052276391	1.366109503	0.003272164
Gm5643|434858	1.257079443	0.11878695	1.027076331	0.423133771
Gpsm2|76123	1.25701364	0.191037724	1.221727887	0.106256821
Vamp3|22319	1.256795201	0.120740057	1.224527394	0.167401304
Ei24|13663	1.256664413	0.007119934	1.313484772	0.000946715
Map3k6|53608	1.256521841	0.156217178	1.387298659	0.058918305

4930427A07Rik|104732	1.256355292	0.402929688	1.724477316	0.34172029
8430408G22Rik|213393	1.256057907	0.205935064	1.256057907	0.205935064
Prkcz|18762	1.255916852	0.121272106	1.357759039	0.076614303
Galnt6|207839	1.25584532	0.307006359	2.273540547	0.013912409
Hist1h1d|14957	1.255620669	0.367182345	1.255620669	0.367182345
Elovl5|68801	1.255141233	0.223741957	1.145228295	0.281429585
Rps3a|20091	1.254539351	0.077434059	1.16570862	0.155344244
Alpl|11647	1.254472025	0.05848423	1.207932714	0.032156749
Foxm1|14235	1.254095304	0.365601524	2.159052936	0.162310568
Arhgap28|268970	1.254079719	0.378023984	1.369634198	0.361484993
Enpp6|320981	1.253923287	0.353046538	1.763945274	0.212162417
Cdc42ep5|58804	1.253911075	0.216771717	1.253911075	0.216771717
Prdm11|100042784	1.253880866	0.412051413	4.57358	0.173296754
Lsm11|72290	1.253820553	0.195974841	1.577867739	0.031393608
Msrb3|320183	1.253803037	0.249934998	1.280838855	0.215819773
Notch4|18132	1.253634321	0.229035794	1.701802558	0.025275758
Htatip2|53415	1.253614895	0.043687215	1.285795333	0.020108346
Gm6623|625785	1.25343601	0.214359498	1.437029975	0.071543027
Mfge8|17304	1.253034615	0.183052456	1.038463906	0.416027824
Irf4|16364	1.25300195	0.378270519	1.25300195	0.378270519
Hoxa1|15394	1.25300195	0.378270519	1.25300195	0.378270519
Gm7102|633057	1.25300195	0.378270519	1.25300195	0.378270519
Rac3|170758	1.2526589	0.303165447	1.495353096	0.150432088
Atp6v1e1|11973	1.252582855	0.01833258	1.199511278	0.039290397
Cltb|74325	1.252319965	0.060007903	1.16513953	0.122591397
Rps15a|267019	1.252200044	0.161667347	1.094117045	0.327499085
Itgb1bp1|16413	1.252004941	0.23753116	-1.140354543	0.103716268
B3gnt2|53625	1.251757477	0.184998633	1.251757477	0.184998633
F11r|16456	1.251566486	0.060334122	1.450305124	0.00232154
1600012F09Rik|67008	1.251513905	0.206006366	1.251513905	0.206006366
2810008M24Rik|75616	1.251338955	0.272283928	1.687520235	0.08316994
Pcmt1|18537	1.250660701	0.037377605	1.279601932	0.000668061
Zfand5|22682	1.25065477	0.145315409	1.25065477	0.145315409
Actr6|67019	1.250643246	0.275495842	1.547516592	0.146288143
1700040I03Rik|73327	1.250381284	0.096696328	1.174717661	0.178572879
Mesdc1|80889	1.250297827	0.199827889	1.111327604	0.282592645
Ddx10|77591	1.250231061	0.16750185	1.577719994	0.009288739
Got1|14718	1.250176388	0.156124231	1.084187569	0.329089499
Ppm1d|53892	1.249594015	0.233238958	1.249594015	0.233238958
Dpep1|13479	1.249430222	0.335030745	2.132131673	0.027709587
Atf4|11911	1.249319687	0.026679668	1.216877423	0.05290656
Espn|56226	1.249112559	0.285526175	1.580556004	0.145538939
Spata1|70951	1.248799418	0.368642454	-1.529725169	0.230937166
Arg1|11846	1.24859995	0.175867707	1.40968992	0.091419364
Orc4|26428	1.248578044	0.268066965	1.433338981	0.18757837
Obfc1|108689	1.2485057	0.25443861	1.008738461	0.48873456
Nop16|28126	1.248200753	0.030209259	1.107953769	0.098595922
Gm14085|381417	1.248142057	0.405483337	3.15	0.173296754
Kpna1|16646	1.247938474	0.174066291	1.2886688	0.109198174
Mbtd1|103537	1.247899593	0.100333637	1.182911649	0.110651864
Fam49b|223601	1.247853207	0.289680781	1.123595736	0.37538781
Arf5|11844	1.247560096	0.14151771	-1.020373449	0.406118137
Ssr2|66256	1.247528543	0.015222938	1.249551884	0.005506476

Ncoa4|27057	1.247361263	0.198703724	1.645952665	0.015356014
Hsph1|15505	1.247344359	0.144752508	1.083095397	0.311967479
Scpep1|74617	1.247092563	0.007136894	1.202599171	0.015850126
A130010J15Rik|319266	1.247076886	0.18673329	1.440639246	0.082795565
Mrpl47|74600	1.247074245	0.066162826	1.368588795	0.002331176
Lgmn|19141	1.24674991	0.02860161	1.209646999	0.055601554
Pdlim4|30794	1.246678697	0.195383777	1.410231791	0.063767778
C6|12274	1.246655011	0.114607779	1.514180507	0.003908266
Necab2|117148	1.246573333	0.408482923	1	#DIV/0!
Pcdhb10|93881	1.246573333	0.408482923	1	#DIV/0!
Rab37|58222	1.246573333	0.408482923	1	#DIV/0!
Rnf180|71816	1.246573333	0.408482923	1	#DIV/0!
Pde3a|54611	1.246573333	0.408482923	1	#DIV/0!
Ttll7|70892	1.246573333	0.408482923	1	#DIV/0!
Ets2|23872	1.246274143	0.041952771	1.103412587	0.143590551
Hsp90ab1|15516	1.246110697	0.001941279	1.290250042	0.000226811
Itgb3|16416	1.246062697	0.105389941	1.312560339	0.007627088
Mrc2|17534	1.246037234	0.166565294	1.268222522	0.099386432
Dnalc4|54152	1.246007038	0.123331004	1.342379964	0.055421474
Fras1|231470	1.245628048	0.362016075	-1.621128921	0.098811758
Rftn1|76438	1.245570675	0.261270473	1.245570675	0.261270473
Rbm14|56275	1.245407074	0.156555328	1.119340728	0.295366458
Ddhd1|114874	1.245112447	0.147503479	1.416535489	0.022191437
Tdo2|56720	1.245040772	0.150505153	1.245040772	0.150505153
AI646023|192734	1.244761628	0.414449858	-4.016833333	0.093227909
Rbpms|19663	1.244664298	0.067992499	1.149352622	0.138941827
Ece2|107522	1.244430793	0.092293835	1.317501065	0.048618966
Akap5|238276	1.244374964	0.286120969	1.08257927	0.355311505
Cdk12|69131	1.244302288	0.098104268	1.365723386	0.014726678
2010320M18Rik|72093	1.244038056	0.195640567	1.063103343	0.380283445
Entpd2|12496	1.243939742	0.152866812	-1.004356106	0.486840102
Rpsa|16785	1.243759669	0.192426332	1.038157498	0.422828288
Il3ra|16188	1.243376492	0.27050209	1.11039165	0.288954643
Zfp775|243372	1.243269453	0.172906434	1.091226163	0.339696916
2310004I24Rik|66358	1.243251776	0.108160366	1.178309684	0.179266755
Zfp420|233058	1.242959609	0.380557468	1.22753158	0.397601243
Spryd3|223918	1.242861034	0.071339165	1.283270161	0.014371509
Hmgcr|15357	1.242631847	0.173487284	1.474083607	0.044176204
Ccdc69|52570	1.242537098	0.390935557	1.282144901	0.403677687
Wdr8|59002	1.242064718	0.093662581	1.147602125	0.180131365
S100g|12309	1.241893769	0.430664868	1	#DIV/0!
Tfb1m|224481	1.241553543	0.206741765	1.407356185	0.071388916
Fbxo38|107035	1.241519306	0.147019088	1.515290966	0.014623796
Pddc1|213350	1.241465508	0.120873858	1.023202097	0.431346556
Zfp120|104348	1.241273462	0.085182587	1.475761286	0.002898373
Dock10|210293	1.241240871	0.372441017	1.241240871	0.372441017
Prpf38a|230596	1.24092439	0.113106845	1.377671402	0.041871153
Sphk1|20698	1.240897654	0.305868421	1.299550627	0.265924373
Gna13|14674	1.240881237	0.147814094	1.405592619	0.056240282
Prkab2|108097	1.240865927	0.291336101	1.952643798	0.023205611
Ptpro|19277	1.240839005	0.40189544	-2.344287879	0.152822898
Rnf125|67664	1.240832974	0.337806013	1.240832974	0.337806013
F2r|14062	1.240579697	0.201173573	1.240579697	0.201173573

Serinc3|26943	1.240514247	0.115683263	1.506231624	0.003051223
Atad2|70472	1.240430697	0.319958488	1.383574291	0.223908429
Hemk1|69536	1.240328209	0.117787139	1.208223178	0.080568946
Atp2b1|67972	1.240148169	0.149601916	1.548601222	0.013256181
Serf1|20365	1.240074976	0.26515782	1.288796475	0.125091498
Kti12|100087	1.239997736	0.154886344	-1.020553863	0.442523895
Lrrfip2|71268	1.239934113	0.077620323	1.239934113	0.077620323
Cebpb|12608	1.239930067	0.133921517	-1.001290618	0.495814938
Hpx|15458	1.239850576	0.2491069	-1.1796517	0.162538551
Fam173b|68073	1.239533665	0.097496476	1.239533665	0.097496476
Rp2h|19889	1.239376818	0.276903859	1.922631262	0.021868703
Ttc21b|73668	1.239043673	0.30518822	1.880884332	0.057149499
Abt1|30946	1.239022222	0.045749774	1.077474791	0.161505301
Mlkl|74568	1.238628015	0.234934434	-1.006828369	0.488202072
Katna1|23924	1.238485674	0.094074124	1.116575241	0.195085296
Fnbp1|14269	1.238256252	0.158885368	1.089101893	0.323905019
Adcy2|210044	1.238235878	0.411260687	1	#DIV/0!
Grin2d|14814	1.238235878	0.411260687	1	#DIV/0!
Ttc16|338348	1.238235878	0.411260687	1	#DIV/0!
Lin9|72568	1.238235878	0.411260687	1	#DIV/0!
A3galt2|215493	1.238235878	0.411260687	1	#DIV/0!
Miox|56727	1.238235878	0.411260687	1	#DIV/0!
Gm11545|217122	1.238235878	0.411260687	1	#DIV/0!
Nqo1|18104	1.238166404	0.15630925	1.387140999	0.03288644
Tmem161a|234371	1.238011246	0.127613508	1.380760632	0.016080294
Gm962|381201	1.23791331	0.085568877	1.23791331	0.085568877
Thap3|69876	1.237861217	0.17271786	1.064346857	0.367063539
Rgag4|331474	1.237635384	0.386738804	-1.409431999	0.337288407
Rpusd1|106707	1.237456401	0.252169026	1.237456401	0.252169026
Fscn1|14086	1.237411461	0.330687796	-1.480136198	0.005923895
Lilrb4|14728	1.23725439	0.343179331	-1.251620294	0.315335931
B630005N14Rik|101148	1.237254375	0.14394426	1.237254375	0.14394426
Lsr|54135	1.236827424	0.009498502	1.236827424	0.009498502
Rps19|20085	1.236774843	0.256785912	1.134042877	0.268571781
Lemd2|224640	1.236741979	0.045071236	1.135490069	0.084622376
Irak3|73914	1.236514044	0.316082925	1.547447019	0.193296358
Fau|14109	1.23622059	0.263686985	1.090094471	0.335596859
1110003E01Rik|68552	1.235532542	0.031726946	1.300515378	0.008165501
Mamstr|74490	1.23528202	0.398935525	1.099838978	0.465920044
Capn11|268958	1.235231971	0.422929276	2.052716667	0.237969445
1700019E19Rik|76411	1.235196609	0.241388355	1.500814969	0.100169928
X99384|27355	1.234877593	0.156852273	1.061072597	0.348257354
Lage3|66192	1.234828092	0.115849451	1.163748035	0.203349517
Mff|75734	1.234742285	0.044849578	1.344011381	0.007319448
Dpp7|83768	1.234646134	0.109152768	1.11696512	0.22566249
Impa2|114663	1.234456827	0.171682944	1.202079658	0.180871164
Mocs3|69372	1.234425711	0.116863874	1.043457074	0.366377313
Msx1|17701	1.234312236	0.39403971	1.264185714	0.40907038
Mirg|100040724	1.234312236	0.39403971	1.264185714	0.40907038
4632415K11Rik|74347	1.234034772	0.155703554	1.404054934	0.013626303
Rabgef1|56715	1.23395276	0.040016718	1.087301534	0.142319067
Gnl3|30877	1.233677788	0.109632465	1.152167687	0.195638328
Fam98c|73833	1.233620521	0.182129733	1.101064747	0.322030222

2010321M09Rik|69882	1.233580196	0.175454851	1.393972045	0.037326022
Rbm28|68272	1.23354566	0.12778455	1.260173755	0.059256766
Prex1|277360	1.233525712	0.225452897	-1.125805269	0.244250795
Fancl|67030	1.233418743	0.205421442	-1.095917294	0.254884599
Ibtk|108837	1.23291294	0.076178754	1.066913833	0.280652191
Igfbp3|16009	1.232788994	0.153639318	1.2261499	0.123248394
Gadd45g|23882	1.232584383	0.297312029	1.295629251	0.179000707
Dio3os|353504	1.232245712	0.282123983	-1.1974058	0.26524066
Nudt3|56409	1.232111955	0.055152869	1.067050675	0.189016213
Echdc1|52665	1.232060043	0.317267575	1.232060043	0.317267575
Gdf10|14560	1.231997689	0.204259157	1.570547274	0.005860611
Ptpn21|24000	1.231984987	0.099496666	1.145529594	0.19350821
2410042D21Rik|72425	1.231693457	0.198628886	1.241548415	0.124116808
Mcm8|66634	1.231647033	0.366652203	1.231647033	0.366652203
Prcp|72461	1.23144564	0.178267047	-1.055332712	0.34992086
Mat2b|108645	1.231247046	0.145534672	1.529975925	0.009679331
Traf4|22032	1.231153573	0.104846584	1.231153573	0.104846584
Pip5k1a|18720	1.230932779	0.153828425	1.350459262	0.054751798
Alyref2|56009	1.230795405	0.176772112	1.230795405	0.176772112
Dtx4|207521	1.230774135	0.040519546	1.331288545	0.00054998
Usp31|76179	1.230761948	0.218120052	1.446948387	0.094643831
Rnf2|19821	1.230739885	0.265308785	-1.180872703	0.24063583
Ncdn|26562	1.230656012	0.036712869	1.162558006	0.075841324
Osbpl11|106326	1.230328219	0.103929419	1.333236496	0.027399194
Hsd17b10|15108	1.230283309	0.104767804	1.230283309	0.104767804
Morc3|338467	1.230148994	0.287379469	1.869255342	0.015116773
Zfp551|619331	1.230118415	0.395639307	1.255130842	0.411829119
Tmem138|72982	1.230014725	0.258912482	1.230014725	0.258912482
Sesn1|140742	1.229918514	0.224305831	1.362934823	0.154458023
Frrs1|20321	1.229653605	0.172402559	1.055095008	0.364180435
Mmrn1|70945	1.229530649	0.411656108	-1.314264015	0.384854801
Prim1|19075	1.229344402	0.370227346	2.267741467	0.119830742
Ldlrap1|100017	1.229231479	0.010978828	1.229231479	0.010978828
Csrp2|13008	1.229192797	0.148344278	1.068437748	0.325430518
Brca2|12190	1.229153608	0.259760891	1.530077395	0.102087806
Srsf10|14105	1.229151345	0.210813326	1.37424992	0.130333991
Immp1l|66541	1.228939161	0.231684056	1.038634072	0.441853674
Lrig3|320398	1.228896773	0.250492493	1.876614129	0.000506883
Cacnb4|12298	1.228816837	0.311523089	-1.048703811	0.454518603
Gpatch4|66614	1.228700552	0.063402456	1.288688701	0.031833136
Emg1|14791	1.228594983	0.046968706	1.305733111	0.009288872
Htra4|330723	1.228592204	0.334285843	1.403419362	0.270636016
Cyp3a16|13114	1.22841091	0.298705225	1.404923822	0.133637385
Rsl1d1|66409	1.228304574	0.040559833	1.095246192	0.152734283
Wipf2|68524	1.228276885	0.072742842	1.223533725	0.0160278
Por|18984	1.228261819	0.034157372	1.322688349	0.000431858
Abtb2|99382	1.22807992	0.23011168	1.002386288	0.495660621
Urb2|382038	1.227773732	0.056479599	1.321131159	0.018541856
Timm10|30059	1.227735464	0.122475485	1.227735464	0.122475485
Tanc1|66860	1.227482228	0.239349087	1.078586848	0.388272661
Hnrpdl|50926	1.2274221	0.118558213	1.201486912	0.079503794
Fkbp11|66120	1.227420443	0.26349274	1.517009631	0.111992211
9430023L20Rik|68118	1.227328564	0.073237389	1.227328564	0.073237389

Senp3|80886	1.227280733	0.061242565	1.28982787	0.003994328
Phf15|76901	1.227262079	0.123855474	1.491304909	0.004981094
Mfsd9|211798	1.227244101	0.372801358	-1.235101385	0.378186279
Igdcc4|56741	1.227196255	0.4106547	5.0331	0.093589634
Arhgap31|12549	1.227002882	0.283387019	1.774962896	0.03080661
Ggct|110175	1.226932924	0.163370616	-1.034775426	0.380435168
Slc35b3|108652	1.226803175	0.199086139	1.309589049	0.139673427
Tm2d3|68634	1.226802954	0.121552753	1.086676084	0.260570414
Tmem62|96957	1.226651695	0.137561976	1.320108364	0.087898623
Erh|13877	1.226207892	0.096462532	1.226207892	0.096462532
Zdhhc20|75965	1.226099523	0.238978831	1.649856489	0.029066617
Gm166|233899	1.225829035	0.255207474	1.767507984	0.013097747
Ap3s1|11777	1.225654866	0.104802974	1.361081863	0.029193174
Cldn14|56173	1.225454923	0.082077549	1.104361271	0.166963426
Siah1a|20437	1.225334079	0.192894886	1.049738552	0.390733749
Ppcs|106564	1.225275301	0.038496468	1.225275301	0.038496468
Heatr5b|320473	1.225273836	0.201989106	1.101711718	0.335409391
Rad54l2|81000	1.225221525	0.125446882	1.469017182	0.008375983
Cd6|12511	1.225166728	0.310573615	1.034981666	0.463856679
Pik3ca|18706	1.224965937	0.014079392	1.229282631	0.000763032
Zfp217|228913	1.22493675	0.233281067	1.066747265	0.407858138
Chst1|76969	1.224832554	0.360278229	1.224832554	0.360278229
Aebp2|11569	1.224721093	0.117798886	1.125605875	0.231083232
Agpat4|68262	1.224713133	0.179780382	-1.054399735	0.351223124
Slc1a4|55963	1.224584895	0.323848084	1.023382853	0.477402703
Rps23|66475	1.224432493	0.147697546	1.067776661	0.323625221
Ubr1|22222	1.224307344	0.20060009	1.488657482	0.039932439
C430048L16Rik|77604	1.224209164	0.342197393	1.024722699	0.479238561
Acacb|100705	1.22414651	0.116085172	1.24586343	0.035107462
Pppde1|78825	1.224133947	0.152863512	1.180556878	0.175244834
Zbtb42|382639	1.224133296	0.197507692	1.21468438	0.176657077
Ctnnd2|18163	1.224065209	0.39822924	1.264185714	0.40907038
Tspyl3|241732	1.224065209	0.39822924	1.264185714	0.40907038
Tmem70|70397	1.223906618	0.031548628	1.153898197	0.064370614
BC057022|433940	1.223840315	0.261059887	1.790179213	0.017708926
Mrps35|232536	1.223797644	0.080579886	1.141490415	0.162385187
Kctd13|233877	1.223474737	0.242190196	1.191118521	0.222580784
2310033P09Rik|67862	1.223456692	0.059523554	1.187933467	0.033806245
Fry|320365	1.223301199	0.287697336	1.570111689	0.117818801
Ankrd39|109346	1.222995156	0.146702101	1.222995156	0.146702101
Stap2|106766	1.222989385	0.109756283	1.467842619	0.003036261
Imp3|102462	1.222889581	0.176474659	1.054925205	0.378217226
Zbtb39|320080	1.222867378	0.138266347	1.163844634	0.11031339
Foxo3|56484	1.222750572	0.212624438	1.367486049	0.087305968
Zfp119a|104349	1.222594795	0.323834553	2.110464409	0.025267897
Riok1|71340	1.222371614	0.252252544	1.222371614	0.252252544
Slc4a1ap|20534	1.222281205	0.075816306	1.222281205	0.075816306
Atp6ap1|54411	1.222275752	0.030489512	1.14058563	0.044001078
Tor1b|30934	1.222185996	0.036363437	1.222185996	0.036363437
Senp6|215351	1.222162235	0.062010593	1.222162235	0.062010593
Tmem82|213989	1.222087297	0.109740843	1.192628325	0.059658886
Zfat|380993	1.221917967	0.246739421	1.157219087	0.212369489
H2-K2|630499	1.221884726	0.196531475	1.028852622	0.435680161

Mir703|735265	1.221620793	0.249937625	1.492063102	0.096949868
Msh6|17688	1.221583838	0.253548661	1.247362255	0.180324279
Fam60a|56306	1.221380988	0.355974744	-1.082536534	0.446768122
Tmem101|76547	1.221369904	0.041545061	1.143871078	0.084479339
Lancl2|71835	1.221331155	0.307658866	1.707075356	0.077162098
Tbc1d10b|68449	1.221279323	0.038453969	1.320303044	0.001663199
Mill2|243864	1.221277501	0.366813906	1.221277501	0.366813906
Snrpc|20630	1.221276309	0.01904377	1.268208573	0.007049078
Ints7|77065	1.221195115	0.261510741	1.049150826	0.440056545
Wdr34|71820	1.221022234	0.126529733	1.466843883	0.010393333
Ccdc9|243846	1.221013924	0.126993174	1.190588787	0.05720766
Cyp4a32|100040843	1.220900976	0.2512344	1.220900976	0.2512344
Eif4a1|13681	1.220752202	0.064357375	1.220752202	0.064357375
Hr|15460	1.220349321	0.272361237	1.220349321	0.272361237
Pir|69656	1.220175317	0.108180863	1.178828208	0.10953782
Zfp54|22712	1.220169015	0.354070268	1.031087153	0.476163505
Mir101a|387143	1.220147699	0.414699976	-1.700679995	0.182430754
Nup54|269113	1.220121696	0.225843689	1.220121696	0.225843689
Nudt18|213484	1.219936945	0.127823872	1.244199496	0.077210581
C80913|19777	1.219808045	0.002170678	1.29638768	0.000106286
Capn10|23830	1.219800468	0.02562144	1.219800468	0.02562144
Ms4a4b|60361	1.219703061	0.349045854	1.21984852	0.298035344
Fam189a1|70638	1.219459773	0.421562816	-4.016833333	0.093227909
Rbm4b|66704	1.219412875	0.138251397	1.19359346	0.123297909
Rps26|27370	1.219392274	0.155705464	1.067288404	0.335450497
Nfatc4|73181	1.219256692	0.24680322	1.568297552	0.011185266
Gm12657|667250	1.219188565	0.061418196	1.395952792	0.000776904
Zfp593|68040	1.219082432	0.280870789	1.219082432	0.280870789
Wnt4|22417	1.218916258	0.358995321	1.855601949	0.158101376
Ncapd2|68298	1.218806405	0.143699993	1.227483564	0.069520466
Tonsl|72749	1.218657482	0.365930983	1.24776777	0.281526487
Prmt5|27374	1.21852229	0.068524919	1.275514756	0.036284937
Ntrk1|18211	1.21841936	0.261540215	1.082269984	0.405036955
Meaf6|70088	1.218306603	0.202157695	1.572921704	0.00779145
Fut1|14343	1.218278833	0.345837949	-1.364963679	0.153711317
Rorc|19885	1.21824097	0.093511638	1.248377877	0.035596696
Ppan|235036	1.218227696	0.140737955	1.058988616	0.314536405
B430306N03Rik|320148	1.218116075	0.24322812	1.071271905	0.395656174
Slc6a8|102857	1.218012172	0.187324111	1.069728542	0.368315554
Hspa4l|18415	1.217952961	0.194474804	1.087096733	0.342011386
Ppp2r4|110854	1.217413959	0.065254886	1.217413959	0.065254886
Tmem151a|381199	1.217226937	0.374931673	1.900066507	0.186724779
Rpl36al|66483	1.217056067	0.185554802	1.059621383	0.379232034
Tmem86a|67893	1.217014308	0.111501285	1.36652892	0.02202378
Mrpl32|75398	1.21696544	0.072299303	1.194028758	0.030560736
Rplp1|56040	1.216828996	0.276880575	-1.229673216	0.109549678
Emp2|13731	1.216706356	0.171466925	1.26720676	0.062264322
Ehmt1|77683	1.216589868	0.139645261	1.250246185	0.054454764
Pole4|66979	1.21657568	0.127247055	1.21657568	0.127247055
3230401D17Rik|66680	1.216372144	0.066495337	1.330667802	0.011858921
Sgtb|218544	1.216123063	0.395496619	-1.469007782	0.317043871
Exoc2|66482	1.21590654	0.241764514	1.36295706	0.158471198
Cd52|23833	1.215733924	0.270609002	-1.075728431	0.369702517

Maz|17188	1.215705273	0.182903889	1.042820045	0.399842434
Itga2b|16399	1.215639669	0.248078628	-1.039986342	0.4209101
Dcaf6|74106	1.21556059	0.070929697	1.151840613	0.024765814
Ldhb|16832	1.215443089	0.21593327	1.375322291	0.117722364
Socs5|56468	1.215292573	0.332760111	-1.2439695	0.251072561
Ercc3|13872	1.215270107	0.168675721	1.060216175	0.35972882
Hexb|15212	1.215207907	0.167086144	1.080830955	0.333214699
Hmg1l1|434174	1.215174919	0.054310279	1.231350838	0.014696177
Lnx2|140887	1.215132185	0.055219732	1.134177864	0.112842627
Ccdc15|245902	1.215093219	0.369918663	1.215093219	0.369918663
Rmi1|74386	1.214931473	0.165982937	1.29686741	0.053963965
Arl6ip5|65106	1.214587594	0.029928944	1.325090821	0.001678539
2010305A19Rik|69893	1.214502923	0.183716138	1.214502923	0.183716138
4930503L19Rik|269033	1.214285714	0.437695173	1	#DIV/0!
Hsp90b1|22027	1.214086846	0.098471021	1.464001479	0.000316062
Sowaha|237761	1.213982889	0.359535795	1.213982889	0.359535795
Dcaf13|223499	1.213794402	0.114156815	1.132919578	0.067905449
Hsf2bp|74377	1.213489281	0.397604444	1.213489281	0.397604444
Akt3|23797	1.213391538	0.300645306	1.213391538	0.300645306
Wdr5|140858	1.212847801	0.022809698	1.110171267	0.062008719
Egr1|13653	1.21275754	0.275946386	-1.004869606	0.493665107
G2e3|217558	1.212692587	0.397277013	2.038087685	0.217686927
Cbx6|494448	1.212522313	0.172613505	1.077185499	0.342367122
C9|12279	1.212504951	0.03619967	1.147549997	0.072300248
Pak1ip1|68083	1.212227738	0.08149234	1.151419817	0.149183094
Rabl3|67657	1.212131053	0.096962528	1.120618349	0.191337618
Klf11|194655	1.212088388	0.153568311	1.212088388	0.153568311
Nek8|140859	1.211889992	0.216754559	1.169471473	0.225124294
Sppl3|74585	1.211769415	0.115968694	-1.00738884	0.454195937
Sumo3|20610	1.211736644	0.015288003	1.197621346	0.006711117
Rpl36a|19982	1.211704749	0.240450782	1.219310287	0.095552329
2700023E23Rik|70036	1.211589354	0.308915153	-1.063058053	0.432999923
Smpd2|20598	1.211538652	0.08081441	1.158107135	0.148806185
Gdap2|14547	1.211156132	0.188751787	1.393722689	0.066530657
1700066M21Rik|73467	1.211134013	0.207403777	1.195210317	0.18155282
Actb|11461	1.211098723	0.106557078	1.107242748	0.219962028
Zfp943|74670	1.210626726	0.263377687	1.445183986	0.049821949
Clstn3|232370	1.210535039	0.242936261	1.436942731	0.103257913
Atp6v1d|73834	1.210461459	0.074349751	1.191172625	0.049277362
D19Ertd737e|76539	1.210459177	0.218895745	-1.11396221	0.177677732
Ndufa5|68202	1.210282872	0.168860665	-1.01035378	0.47385928
Gmppb|331026	1.210272872	0.177578345	1.402972765	0.046901843
Rb1cc1|12421	1.210137145	0.211775057	1.564820883	0.002878966
Ubtd2|327900	1.210045778	0.31641289	1.01372833	0.484857886
Rps19-ps3|277692	1.210038729	0.234005319	1.014405771	0.474497752
Alkbh1|211064	1.209988703	0.079754261	1.209988703	0.079754261
Lig3|16882	1.209914002	0.129580326	1.021956149	0.434574179
Rassf3|192678	1.209807779	0.093872877	1.365258927	0.00660231
Dennd4a|102442	1.209717713	0.13174231	1.122536606	0.245041714
AI846148|68229	1.209648089	0.149117285	-1.024334607	0.410635991
Sema3f|20350	1.209645114	0.163991615	1.240613111	0.105563532
Tbc1d24|224617	1.209437161	0.156063882	1.088904102	0.298137
Rars|104458	1.209184825	0.039237853	1.209184825	0.039237853

Cox16|66272	1.209166105	0.047364503	1.252384296	0.03578061
Rpl23|65019	1.209091197	0.059260191	1.055711755	0.226243436
Mbnl1|56758	1.209062742	0.099255625	1.035208184	0.364253165
C87436|232196	1.208974936	0.156257044	1.208974936	0.156257044
Acnat2|209186	1.208937175	0.086021286	1.397314341	9.35491E-05
Ndufb2|68198	1.208810097	0.149805897	1.211262476	0.044233311
Rraga|68441	1.208644956	0.035796778	1.14168336	0.06957952
Tusc1|69136	1.208428786	0.245313656	-1.133315258	0.227358228
Pwp1|103136	1.208145092	0.219649947	1.07720324	0.380544671
Hexa|15211	1.208100452	0.11185486	1.185954062	0.081844244
Gtf2e2|68153	1.207970654	0.185971054	1.207970654	0.185971054
Cadm4|260299	1.207888403	0.212697154	1.394415538	0.088912466
Vmp1|75909	1.207763089	0.029477123	1.3205935	0.001252066
Cdk9|107951	1.20758929	0.105575617	1.133327007	0.192751379
Rpp30|54364	1.207534354	0.303639046	-1.082050796	0.402450513
Dnajc8|68598	1.207514541	0.07216957	1.207514541	0.07216957
Pnpt1|71701	1.207484695	0.292254089	1.398476606	0.140044676
Ptp4a1|19243	1.207367791	0.101840315	1.302262904	0.023847873
Cycs|13063	1.207327662	0.114747765	1.206814847	0.072314688
Dnajc12|30045	1.207291211	0.156098215	1.230425194	0.064494886
Gdf2|12165	1.207163569	0.04518972	1.207163569	0.04518972
Atxn7l3b|382423	1.207025322	0.077835857	1.128042181	0.158393048
Fam109a|231717	1.207004785	0.077862596	1.207004785	0.077862596
Myeov2|66915	1.206953168	0.115424321	1.002294145	0.489072967
Tpst1|22021	1.206689836	0.101275124	1.206689836	0.101275124
Plin2|11520	1.206651132	0.172792209	1.132656653	0.18179932
Epc1|13831	1.20651264	0.088155595	1.20651264	0.088155595
Tbp|21374	1.206417075	0.218260609	-1.076961018	0.336859917
Cep76|225659	1.206406781	0.274539269	1.206406781	0.274539269
Ifi27l2b|217845	1.20616648	0.139626225	1.20616648	0.139626225
Cbx3|12417	1.206160673	0.050672719	1.121358515	0.092773642
Pnpla2|66853	1.206087986	0.101992776	1.205974754	0.091655745
AI118078|244886	1.206071834	0.353373551	-1.089387769	0.368162488
Pigt|78928	1.205916485	0.116739046	1.205916485	0.116739046
Traf2|22030	1.205586525	0.184146245	1.034644572	0.411206201
Isg20l2|229504	1.205207844	0.034135785	1.145766983	0.068900383
Bcl7c|12055	1.205169597	0.253631486	1.150555105	0.273172947
Galntl4|233733	1.205168906	0.323025022	1.151423698	0.364555122
Fas|14102	1.205125328	0.205513249	1.234212381	0.08633485
Lpar2|53978	1.205027253	0.366790988	1.011787147	0.488717394
Rsad2|58185	1.205026846	0.248825624	1.205026846	0.248825624
Srsf9|108014	1.204818704	0.057623965	1.187078428	0.089412725
Cgrrf1|68755	1.204805718	0.152863271	1.351528304	0.050917617
Caln1|140904	1.204745767	0.251989632	1.025029239	0.462587125
Rbmx|19655	1.204586129	0.127051165	1.450082392	0.004087244
Adcyap1r1|11517	1.204478181	0.304277174	-1.108642241	0.360777983
Nhlrc2|66866	1.204390851	0.211618134	1.236610819	0.110804248
Sub1|20024	1.20406521	0.02677179	1.249211082	0.016206716
Atp6v0b|114143	1.204058263	0.071588268	1.045315192	0.272809987
Map3k1|26401	1.203879029	0.304802463	1.203879029	0.304802463
Ssb|20823	1.203859607	0.119317592	1.325265531	0.011852544
Tbca|21371	1.203772103	0.083877787	1.157259408	0.051166201
Il1r1|16177	1.203532267	0.285526137	1.625351555	0.051660231

Abca9|217262	1.203451049	0.400383065	-1.465157012	0.318275402
Zfp212|232784	1.203326186	0.196477661	-1.05320905	0.369376489
Pcyox1l|240334	1.203275735	0.297315693	1.464432736	0.157229704
Tubb4b|227613	1.20326031	0.09541606	1.249998749	0.066739964
Prr3|75210	1.203234545	0.10521897	1.345110587	0.015449999
Fam55d|244853	1.20297971	0.410025603	3.907410225	0.07895149
A930001N09Rik|77128	1.202947258	0.188461278	1.202947258	0.188461278
Fam131c|277743	1.202876294	0.166839671	-1.035344677	0.370047836
Supt3h|109115	1.202543032	0.30584607	1.404496125	0.105889684
Eif3e|16341	1.20250339	0.048370906	1.133764222	0.099079925
Zfp46|22704	1.202492688	0.1904286	1.36085529	0.031532007
1110008J03Rik|100764	1.20233137	0.226250907	1.391460715	0.038042068
Cdh1|12550	1.202302375	0.228296479	1.658995467	0.001533459
Eif1a|13664	1.201983138	0.175654831	1.164982979	0.136784416
Prc1|233406	1.201879057	0.402331357	1.201879057	0.402331357
Angel2|52477	1.201849045	0.146808636	1.201849045	0.146808636
Tnik|665113	1.20182586	0.402886026	-1.817607799	0.201128651
Bub1|12235	1.201808426	0.407356298	1.195314368	0.428478238
Ung|22256	1.201738149	0.148287427	1.125158908	0.156053134
BC017612|170748	1.201339293	0.122974199	1.270428935	0.066414035
Aprt|11821	1.201136963	0.143208496	1.282159722	0.071656455
Rhoc|11853	1.200936164	0.190966824	1.130184508	0.129627884
Rab3a|19339	1.20056306	0.215120297	1.008657805	0.479843297
Adal|75894	1.200429288	0.275086499	1.200429288	0.275086499
Aldh1a7|26358	1.200381016	0.163188879	1.3312342	0.033164078
Rhpn2|52428	1.200356161	0.247065549	1.445671173	0.086059242
Atp5g3|228033	1.200337553	0.034935489	1.278472176	0.006889345
Elk4|13714	1.200290297	0.093478885	1.200290297	0.093478885
Rnf217|268291	1.200252269	0.148770211	1.081256698	0.293991877
Usp20|74270	1.200126733	0.127761096	1.316907833	0.047693291
Taf4b|72504	1.199791099	0.409022405	-1.779313652	0.246948927
Scoc|56367	1.199671215	0.311132582	-1.105928806	0.372767289
Igfbp7|29817	1.199666768	0.074129367	1.047259131	0.289556773
Lrrc33|224109	1.199548933	0.162176997	1.164611424	0.117746234
Ppp4r2|232314	1.199233125	0.225779995	1.482602423	0.032323029
Rpl5|100503670	1.199203411	0.127442416	1.097490523	0.257025788
Cttnbp2nl|80281	1.199033407	0.299967895	1.36845016	0.080890681
2410131K14Rik|76792	1.198909508	0.128223941	1.43802279	0.004509138
Rgs4|19736	1.198849966	0.381552677	2.362070998	0.081612439
Nhsl1|215819	1.198783402	0.302415532	1.94151797	0.003806704
Urm1|68205	1.198773706	0.109645812	1.29820696	0.021464417
Rpp21|67676	1.198650606	0.218514984	1.113503406	0.333023893
Fcna|14133	1.198638424	0.022402952	1.198638424	0.022402952
Golga3|269682	1.198427154	0.160789766	1.332803251	0.063888444
Arf6|11845	1.198369464	0.051636551	1.060885182	0.192306322
Clip4|78785	1.198262043	0.421473229	5.0331	0.093589634
A330049M08Rik|230822	1.198232707	0.340513677	1.198232707	0.340513677
2010107E04Rik|70257	1.198223618	0.149023143	-1.01431139	0.439150644
Ndufa2|17991	1.19817101	0.231428028	-1.004388913	0.490555209
4933422H20Rik|432613	1.198079092	0.269258913	1.198079092	0.269258913
1500001M20Rik|68971	1.1977416	0.307225455	1.311591353	0.252290429
Ciapin1|109006	1.19760548	0.13365959	1.081100189	0.280193889
A930012L18Rik|626275	1.197581142	0.414865642	-1.144896552	0.447167923

Nt5c2|76952	1.197349698	0.056159662	1.2531896	0.03260017
Adck5|268822	1.19733673	0.11420523	1.086786755	0.236283094
1700030C10Rik|69513	1.197311244	0.308937072	1.118292142	0.366836352
Ube2n|93765	1.197019914	0.010469022	1.258124177	0.001185855
Fam167a|219148	1.196989281	0.385538989	-1.430229289	0.272060568
Cul5|75717	1.196627283	0.206229415	1.042358633	0.413598366
Kdm4a|230674	1.196596298	0.13371573	1.269169605	0.070488433
Srsf1|110809	1.196588212	0.154987392	1.196588212	0.154987392
Spon2|100689	1.196521386	0.148946396	1.279642532	0.072478305
A330040F15Rik|74333	1.196392551	0.409868573	-2.085282363	0.154585183
Ubxn11|67586	1.196114513	0.408588343	1.255130842	0.411829119
Gm5177|382450	1.196114513	0.408588343	1.255130842	0.411829119
Zbtb26|320633	1.196095735	0.347713778	1.196095735	0.347713778
Crtap|56693	1.195873106	0.251573839	1.189897061	0.170505164
Rad17|19356	1.195392228	0.108378805	1.308350147	0.031745984
Set|56086	1.195141948	0.078331291	1.195141948	0.078331291
Brix1|67832	1.195073551	0.144056221	1.094058539	0.281278594
Zfp953|629016	1.194925475	0.350575594	1.194925475	0.350575594
Zfp595|218314	1.194893788	0.332864902	-1.115591365	0.388242975
Hhex|15242	1.194817449	0.345516673	-1.220966275	0.294587217
Rpl36|54217	1.194752412	0.288390236	-1.087306462	0.359604351
Fam129b|227737	1.194735039	0.229323065	1.104424121	0.188072485
Minos1|433771	1.194633084	0.111575945	1.101817472	0.226918441
Chchd5|66170	1.194595968	0.1718355	1.039792696	0.385371515
Cdnf|227526	1.194514176	0.312662866	1.425582359	0.122378952
Elp3|74195	1.194377236	0.197961332	1.024827812	0.437539522
Slc25a47|104910	1.194277659	0.089584539	1.160803018	0.024781354
Wbp4|22380	1.194180863	0.080313158	1.144030651	0.078840225
Ash2l|23808	1.194132641	0.09449624	1.344454211	0.003687045
Pcgf6|71041	1.194058222	0.210689033	1.549287471	0.009597096
Gng2|14702	1.194045772	0.325259431	1.194045772	0.325259431
Bace2|56175	1.193853742	0.234939392	1.131428727	0.263119595
Pdzd11|72621	1.193700315	0.13749935	1.2973946	0.035853414
Zfp784|654801	1.193692723	0.240622841	1.193692723	0.240622841
Plk3|12795	1.193660747	0.276040595	1.496094741	0.08595487
Kctd15|233107	1.193304817	0.11538777	1.308938565	0.003682011
Acyp2|75572	1.192880955	0.381909529	2.002052472	0.143054761
Dnajb6|23950	1.192575292	0.163315026	1.192575292	0.163315026
Notch1|18128	1.192178841	0.204526713	1.242874239	0.081760346
Slc16a1|20501	1.192111378	0.138820962	1.259494424	0.079877551
Med10|28077	1.192039334	0.06392835	1.062842693	0.223686304
Casp8ap2|26885	1.192027102	0.367141822	1.192027102	0.367141822
2900010M23Rik|67267	1.191991657	0.232002327	-1.113798351	0.232539297
Haus7|73738	1.191980545	0.164021667	1.068950604	0.333718238
Tcp11l2|216198	1.191834663	0.297781605	1.033976928	0.46188077
Agpat9|231510	1.19178197	0.230371423	1.029759924	0.445341223
Emp1|13730	1.191765312	0.411605861	1.195314368	0.428478238
Sip1|66603	1.191731238	0.224077839	1.051044548	0.400977396
Gm7609|665378	1.191713865	0.382737633	2.026293467	0.137926329
Txnl4a|27366	1.191697352	0.0080141	1.166882657	0.018176507
Ube2j2|140499	1.191615618	0.137213072	1.11268629	0.2509439
Enpp2|18606	1.191603984	0.241248511	1.533131171	0.012353345
Mid2|23947	1.191563265	0.403080743	1.107925644	0.449595369

Fastkd5|380601	1.191379809	0.265824187	1.588963846	0.027067119
Gfod2|70575	1.191378291	0.109284037	1.11464397	0.205381025
Itm2c|64294	1.191353175	0.017446253	1.094831122	0.045898006
4930403N07Rik|73936	1.191304111	0.385337562	1.756516064	0.211013396
Arfgef1|211673	1.191246853	0.198725936	1.191246853	0.198725936
Rngtt|24018	1.190970099	0.325769611	1.701260412	0.073527569
Olfr856-ps1|624341	1.190957377	0.312019686	1.327142176	0.241774988
2010003K11Rik|69861	1.190921907	0.260831005	-1.026489848	0.452187571
Parvg|64099	1.190870883	0.275565362	-1.177224379	0.20226527
Nxf1|53319	1.190852094	0.120688677	-1.002322033	0.486921321
Atpbd4|66632	1.190756079	0.193990013	1.105299143	0.307249167
Cebpg|12611	1.190678152	0.029701919	1.297732223	3.08148E-05
Fam174b|100038347	1.190668143	0.083163459	1.086895647	0.168298902
Traf3ip1|74019	1.190384287	0.227919107	-1.108591751	0.23405828
Hs2st1|23908	1.190338262	0.261311854	1.190338262	0.261311854
Clcn5|12728	1.190228635	0.411221383	1.264185714	0.40907038
Shb|230126	1.19019032	0.120919	1.218275735	0.0309584
Tcf19|106795	1.190063589	0.348033089	1.09611263	0.411842249
Churc1|211151	1.189882171	0.099222574	1.110783753	0.198953027
Vegfc|22341	1.189860108	0.358561392	-1.475437307	0.145118594
Mir17hg|75957	1.189796811	0.41215364	1.170129778	0.437059136
Ndufs4|17993	1.189776462	0.146396476	1.073521368	0.304173356
Cwc25|67480	1.189698517	0.229370054	1.389479884	0.082696418
Brp44|70456	1.189676085	0.060577665	1.098807642	0.11599498
Utp20|70683	1.189652908	0.213528301	1.189652908	0.213528301
Ube2m|22192	1.189601641	0.18323741	-1.058433372	0.280374823
Ttc33|67515	1.189556759	0.145856011	1.113405964	0.220699428
Asl|109900	1.189523377	0.158812481	1.244418331	0.114895709
Rundc3a|51799	1.189449842	0.153978428	1.001123074	0.496645045
2210018M11Rik|233545	1.189389724	0.071654834	1.331812435	0.003004808
Uba6|231380	1.189388449	0.265955447	-1.054544838	0.391969266
Gpr172b|52710	1.189279301	0.162867122	1.292988426	0.056524944
Enah|13800	1.189265729	0.39067696	1.813056099	0.208718294
A130040M12Rik|319269	1.188854525	0.349115773	1.188854525	0.349115773
8430406I07Rik|74528	1.188754374	0.212490672	1.348059503	0.041629778
Hmgxb3|106894	1.188743906	0.148411303	1.032614088	0.405850968
Kif5b|16573	1.18872823	0.202731054	1.574661687	0.000190327
Hspa13|110920	1.188663295	0.285098774	1.673874909	0.017754176
D14Abb1e|218850	1.188528235	0.151670978	1.188528235	0.151670978
Vwf|22371	1.188478474	0.213665755	1.188478474	0.213665755
Lrig2|269473	1.18830243	0.152882767	1.18830243	0.152882767
Ripply3|170765	1.188233327	0.364215164	-1.041454457	0.465340323
Rbm15|229700	1.188130461	0.275529005	1.178028245	0.174616752
Gsr|14782	1.188080976	0.023626163	1.188080976	0.023626163
Ndufb4|68194	1.18804327	0.101475674	1.093528307	0.211030248
Adamts9|101401	1.187941488	0.371555221	1.187941488	0.371555221
Atp6v1c1|66335	1.187904533	0.060390879	1.187904533	0.060390879
Zbtb7c|207259	1.187903501	0.319335388	-1.122202563	0.351384897
Klhl32|212390	1.187845914	0.141598703	1.187845914	0.141598703
2310016M24Rik|66379	1.187672461	0.123255856	1.311092427	0.031242196
Thoc1|225160	1.187594352	0.157582788	1.099179124	0.287000079
Gm5617|434402	1.187584006	0.143715189	1.187584006	0.143715189
Rbm15b|109095	1.187475436	0.276875681	1.187475436	0.276875681

Naa20|67877	1.187445701	0.217621184	1.502521696	0.011016545
Dcp2|70640	1.187442211	0.264224965	1.272143107	0.196385977
Seh1l|72124	1.187413593	0.100448887	1.179076716	0.027097347
3110082I17Rik|73212	1.187287881	0.181056859	1.15392178	0.181137836
Rogdi|66049	1.187208226	0.192140523	1.09060313	0.321198079
Ccdc90b|66365	1.186945301	0.275749849	1.033806533	0.448706248
H2-Aa|14960	1.186833055	0.284562124	-1.100396526	0.301317955
Chpf|74241	1.186828145	0.264244926	1.269509455	0.010289553
Fhod1|234686	1.186756378	0.158807499	1.079169361	0.302466805
Bcl2l1|12048	1.186650512	0.106155825	1.075753125	0.222026173
Oas1a|246730	1.186613973	0.292830975	1.36306092	0.135808472
Tmem18|211986	1.186448892	0.168550321	1.077647024	0.324864857
2310045N01Rik|72368	1.186199354	0.121184763	1.110839467	0.229369167
Nutf2|68051	1.186189265	0.103737334	1.085276183	0.215301595
Wrb|71446	1.186075549	0.326673277	1.411703611	0.110933742
Tmem135|72759	1.185877315	0.192830624	1.359527408	0.055208905
Rnd1|223881	1.185736452	0.219112887	1.237078879	0.057917735
Pdlim1|54132	1.185518768	0.059088253	1.115549005	0.12070504
Mcm10|70024	1.185474679	0.1282708	1.290872472	0.022024612
Vps29|56433	1.185368896	0.134827072	1.108000671	0.240871219
Bcam|57278	1.185315606	0.260087534	1.001423074	0.497604965
Ubfd1|28018	1.185223698	0.055905111	1.332697915	0.001306258
Ren2|19702	1.185215559	0.416643489	1.150701957	0.450338304
Sntg2|268534	1.18519724	0.307123503	1.759680614	0.031487133
Bbs2|67378	1.185094329	0.302790958	1.471419608	0.131407284
Acadsb|66885	1.184876155	0.042081542	1.112706689	0.072645506
Atp6v0d2|242341	1.184813694	0.309312904	1.443441222	0.077261806
Krcc1|57896	1.184605122	0.205931123	1.070251453	0.368021463
Pid1|98496	1.184600574	0.124173314	1.184600574	0.124173314
Ilvbl|216136	1.184509362	0.145683713	1.184509362	0.145683713
Rpl19|19921	1.184444815	0.275072297	1.111013268	0.286493796
Irak4|266632	1.184379498	0.145094979	1.16297901	0.136196633
Acsm2|233799	1.184335482	0.402500128	2.35484547	0.137660468
Obsl1|98733	1.184186147	0.366134053	1.184186147	0.366134053
Nudcd2|52653	1.184089988	0.183789494	1.060917145	0.351985846
Fbl|14113	1.184058642	0.174538082	1.054122836	0.362603177
Ikzf4|22781	1.184042317	0.409955072	2.495036093	0.151126021
Zfp71-rs1|235907	1.183995776	0.33171996	-1.00805062	0.490755365
Slc13a4|243755	1.183942021	0.414754484	1.178571429	0.43416
Fam113b|239647	1.183926407	0.108344545	1.281412186	0.037561713
Tubb5|22154	1.183876029	0.155599576	1.174779625	0.052605617
Gm3833|100042415	1.183621596	0.272413012	-1.213802317	0.046437947
Rpl18a|76808	1.183611693	0.272242149	-1.060702867	0.381338137
Mki67|17345	1.183602513	0.39345127	1.622532447	0.256539303
Igsf8|140559	1.183554344	0.254751364	-1.020617752	0.459478067
Dem1|73172	1.183486929	0.297433516	1.190219708	0.261201203
Srf|20807	1.18344531	0.03469193	1.153304376	0.066867415
Rps16|20055	1.183427207	0.227615003	-1.000721854	0.498286826
2610204G22Rik|70448	1.183274414	0.394032592	1.820157617	0.207329861
Btf3|218490	1.183176124	0.107731072	1.104678285	0.214085604
Dgat1|13350	1.183075409	0.053897636	1.183075409	0.053897636
Erich1|234086	1.182921768	0.328699917	1.458816847	0.182476319
Det1|76375	1.182855961	0.272892765	1.182855961	0.272892765

Paics|67054	1.182559649	0.011342728	1.099314219	0.018546965
Nsun2|28114	1.182380805	0.091534912	1.14085252	0.014663493
Fam117b|72750	1.182360677	0.22805723	1.182360677	0.22805723
Pdia5|72599	1.182336497	0.095125102	1.166751348	0.035355607
Gsg1l|269994	1.182191812	0.405185128	-1.615913663	0.244974828
Timm8b|30057	1.182185374	0.100656718	1.283399414	0.027673453
Kcng4|66733	1.182120928	0.428022407	1	#DIV/0!
Cdkn2a|12578	1.182120928	0.428022407	1	#DIV/0!
Pabpc1l|381404	1.182120928	0.428022407	1	#DIV/0!
Rpl3l|66211	1.182120928	0.428022407	1	#DIV/0!
Fkbp6|94244	1.182120928	0.428022407	1	#DIV/0!
Zfp488|382867	1.182120928	0.428022407	1	#DIV/0!
Lrrn1|16979	1.182120928	0.428022407	1	#DIV/0!
C330021F23Rik|546049	1.181984224	0.155126367	-1.030133618	0.371268938
1700003E16Rik|71837	1.181968062	0.333234585	1.024008621	0.463101133
Jag1|16449	1.181787905	0.323717692	1.731609106	0.043086369
Eed|13626	1.181746347	0.254552197	1.181746347	0.254552197
Mapre3|100732	1.181167838	0.155959883	1.232668873	0.07498346
Setd4|224440	1.181128908	0.284839359	1.326352492	0.192598073
Ccnjl|380694	1.181022306	0.421598419	-1.877217172	0.253663907
6330416G13Rik|230279	1.180845923	0.228969909	1.082198442	0.357818347
Alyref|21681	1.180581461	0.138319128	1.388734669	0.00429947
Phc2|54383	1.180382464	0.123118052	1.069142047	0.263936749
Sec11a|56529	1.1801724	0.115483524	1.194403328	0.051022677
1110049F12Rik|66193	1.180125607	0.041006441	1.137098338	0.082478172
Cyp2c70|226105	1.179949138	0.274058295	-1.052616825	0.401456027
Zhx2|387609	1.179903065	0.130447223	1.179903065	0.130447223
2610101N10Rik|67958	1.179770568	0.197113395	1.332629002	0.071716999
Hspa5|14828	1.179683555	0.270902605	1.555132527	0.016082492
Ms4a6c|73656	1.179388151	0.310135851	1.60489489	0.05706931
Alkbh2|231642	1.179348104	0.337738901	1.006571262	0.48987436
Pld6|194908	1.179217389	0.325013888	-1.160691022	0.286672468
Mical2|320878	1.178942528	0.108035441	1.178942528	0.108035441
AI450353|103729	1.178847292	0.356542829	1.178847292	0.356542829
Capn6|12338	1.17882635	0.356990855	1.618103207	0.16108132
Gm13889|620695	1.178735394	0.303817351	-1.047355103	0.437351724
Bcl11b|58208	1.178601821	0.421093154	-2.237469697	0.16135399
Oas1g|23960	1.178588813	0.3168744	1.178588813	0.3168744
Gpr35|64095	1.17832672	0.381964275	1.17832672	0.381964275
Rnf121|75212	1.178211521	0.182524359	1.173770498	0.033554069
1810063B05Rik|67892	1.178080228	0.266745678	1.335352514	0.155912123
Rpf1|70285	1.177910813	0.174682659	1.305222883	0.030465437
St8sia4|20452	1.177668806	0.346301598	-1.010311365	0.489160098
Rpl35|66489	1.177549134	0.281395759	1.091016771	0.292208773
Unc13a|382018	1.177437791	0.252864442	1.43037554	0.059997818
Lrmp|16970	1.177309326	0.316962807	1.74132668	0.009645367
Tbcb|66411	1.177227995	0.180464273	1.111550191	0.287098411
Psmb4|19172	1.177139695	0.03627186	1.140903951	0.016161582
Dek|110052	1.176886591	0.282063396	1.458104715	0.085903225
Nop56|67134	1.176811712	0.094710155	1.144288381	0.036876904
Emd|13726	1.176773451	0.111051359	1.016314838	0.419685429
Cpd|12874	1.176639727	0.229909533	1.176639727	0.229909533
Mrpl20|66448	1.176514981	0.099706353	1.176514981	0.099706353

P4ha1|18451	1.17623276	0.107510883	1.257447347	0.047051917
Rps10|67097	1.176135935	0.21073722	-1.086884309	0.215598996
Map3k10|269881	1.175795575	0.129420757	1.295547235	0.031189978
Psmg4|69666	1.175678528	0.152661987	1.173268841	0.034725836
Mob4|19070	1.175630651	0.130155114	1.361878018	0.00691945
Gm7104|633093	1.175608	0.443532116	1	#DIV/0!
Klhl11|217194	1.175608	0.443532116	1	#DIV/0!
A930011G23Rik|319818	1.175608	0.443532116	1	#DIV/0!
Creld1|171508	1.175520891	0.085091343	1.345716106	0.00141864
Csrnp2|207785	1.175344978	0.276893178	-1.157808361	0.212410392
Tpt1|22070	1.174915961	0.105933334	1.099880683	0.211341263
Npm1|18148	1.174864101	0.077729521	1.11519827	0.151440312
Dirc2|224132	1.174863208	0.031044038	1.120012933	0.05860479
Ndufv2|72900	1.174839374	0.066811137	1.099017018	0.133347006
Siah1b|20438	1.174522209	0.387266496	-1.446519002	0.218759954
Ercc4|50505	1.174106336	0.102826322	1.088869819	0.208861405
Klhdc10|76788	1.174073736	0.050090265	1.174073736	0.050090265
Bach1|12013	1.173920135	0.295534689	1.173920135	0.295534689
Ccdc56|52469	1.173704883	0.069066431	1.173704883	0.069066431
Rps5|20103	1.173376813	0.247802341	1.005389211	0.489408577
Sf3b3|101943	1.173103228	0.20005747	-1.051350049	0.35319757
Psmg2|107047	1.173091231	0.204494644	1.173091231	0.204494644
Lfng|16848	1.172978248	0.154526348	1.228326543	0.063942705
Cdca3|14793	1.172877731	0.307214136	1.278539544	0.222389143
Ikzf2|22779	1.172875241	0.363328849	2.108741018	0.024168518
Polr1e|64424	1.172818575	0.303353189	1.172818575	0.303353189
Uqcrfs1|66694	1.172801164	0.038766618	1.28220905	0.001092411
Fgfr1|14182	1.172491711	0.216061367	1.172491711	0.216061367
Rph3al|380714	1.172290899	0.412053421	1.084493596	0.441089956
Coq7|12850	1.17213954	0.194816347	1.413498452	0.021166167
Qsox1|104009	1.17209353	0.08011243	1.17209353	0.08011243
Endog|13804	1.172086789	0.201382163	-1.058084442	0.323968064
Tmem39b|230770	1.172069524	0.193405627	1.301174193	0.041399441
Pdcd2l|68079	1.171851759	0.191834205	1.067386321	0.34391782
Comtd1|69156	1.171773922	0.3046897	1.260360927	0.170359931
Msh3|17686	1.171600353	0.224001484	1.380935606	0.046562033
Gm16378|100043225	1.171596188	0.107208361	1.143068534	0.074952438
Heatr1|217995	1.171502394	0.109185084	1.171502394	0.109185084
Kctd20|66989	1.1714123	0.228351574	1.062734717	0.378672248
Ggnbp1|70772	1.171348433	0.252211962	1.042320495	0.310031783
Pde4c|110385	1.171343106	0.201806268	-1.062686715	0.303409685
Rd3|74023	1.171243716	0.249042127	-1.124031316	0.205364319
Gpld1|14756	1.171222743	0.018197915	1.171222743	0.018197915
Msr1|20288	1.171206812	0.278716692	1.033012044	0.447284978
H2afv|77605	1.171038658	0.138076886	1.062322652	0.295419274
Gm6402|623169	1.170960359	0.297026403	1.248691848	0.146319832
Stxbp5|78808	1.170885014	0.262306772	1.170885014	0.262306772
Iah1|67732	1.170856119	0.108478344	1.012548835	0.436104511
Wibg|78428	1.170782396	0.173097958	1.068927224	0.332787071
Camk2b|12323	1.170778171	0.31311342	1.029345324	0.462381482
Invs|16348	1.170739539	0.344282585	1.649026172	0.095718159
Ing1|26356	1.170529275	0.231631973	1.33209139	0.09675943
Vps25|28084	1.17049228	0.084596206	1.260726721	0.018971319

Gucy1a3|60596	1.17047396	0.325979548	1.193308042	0.288094862
Zfp30|22693	1.170350203	0.320980288	1.170350203	0.320980288
Arhgef3|71704	1.17033403	0.232016782	1.284164276	0.082355092
Acer2|230379	1.17027186	0.116068507	1.192137216	0.110344119
Amigo1|229715	1.170131172	0.146482867	1.17086529	0.049633448
Ost4|67695	1.169935176	0.105730979	1.022329602	0.391399232
Snx20|71607	1.16987537	0.397590327	-1.343191281	0.318876392
Dhodh|56749	1.169815679	0.208175743	1.348312993	0.051155338
Btf3l4|70533	1.169789813	0.050383888	1.169789813	0.050383888
Tm4sf4|229302	1.169721133	0.058945068	1.111758546	0.120425985
Mal2|105853	1.169646665	0.18028781	1.29502799	0.069205364
Tomm6|66119	1.169608169	0.155016303	1.058134851	0.326115604
Tmem120a|215210	1.169570319	0.125270212	1.204709348	0.100947304
Dnaja4|58233	1.169464851	0.322417492	1.169464851	0.322417492
Rchy1|68098	1.169427658	0.098516415	1.062334923	0.18463296
Plaa|18786	1.169391076	0.125122892	1.266203196	0.019254721
Cast|12380	1.169369473	0.204289185	1.161486469	0.203564326
Mrfap1|67568	1.169247831	0.141385828	1.253906061	0.047044679
Gnl1|14670	1.169034398	0.156771758	1.057822486	0.328670865
Arhgef2|16800	1.169010956	0.222329297	1.169010956	0.222329297
Dpt|56429	1.168935665	0.284421422	-1.142763393	0.270404893
Ccdc73|211936	1.16879777	0.418648431	1.002806537	0.498817668
Ccdc21|70012	1.168170258	0.324435737	-1.141247725	0.301490639
Mfsd8|72175	1.167941678	0.303162384	1.37456662	0.163370081
Sbds|66711	1.167753025	0.029382574	1.071585446	0.094296807
Cab39l|69008	1.167697955	0.167389574	1.317694259	0.019909718
Inhba|16323	1.167544183	0.322468047	1.388818085	0.116154653
Mapk11|19094	1.167455853	0.289251654	1.167455853	0.289251654
Sec23a|20334	1.167436829	0.265962309	1.183101409	0.215076752
Hmgb3|15354	1.16740487	0.170131822	1.413013934	0.003839748
Alkbh6|233065	1.167197437	0.08982849	1.185310004	0.031165971
Ppil3|70225	1.167194556	0.210475405	1.035943258	0.416553152
Kctd7|212919	1.166926821	0.387623365	-1.088974331	0.434485817
Ndufb6|230075	1.16680291	0.017887747	1.110766516	0.025493214
Cdk4|12567	1.166701409	0.194285655	-1.04796512	0.344111912
Mki67ip|67949	1.166683132	0.17408844	1.069567924	0.321268149
Pacrg|69310	1.166666667	0.433741809	1	#DIV/0!
Baiap2l2|207495	1.166666667	0.433741809	1	#DIV/0!
Bmp10|12154	1.166666667	0.433741809	1	#DIV/0!
Chrdl1|83453	1.166666667	0.433741809	1	#DIV/0!
Gm13238|236069	1.166666667	0.433741809	1	#DIV/0!
Ttc22|230576	1.166666667	0.433741809	1	#DIV/0!
Zscan10|332221	1.166666667	0.433741809	1	#DIV/0!
Chgb|12653	1.166666667	0.433741809	1	#DIV/0!
Mmp27|234911	1.166666667	0.433741809	1	#DIV/0!
Dhh|13363	1.166666667	0.433741809	1	#DIV/0!
D830030K20Rik|320333	1.166666667	0.433741809	1	#DIV/0!
Cxxc4|319478	1.166666667	0.433741809	1	#DIV/0!
5430405G05Rik|108832	1.166666667	0.433741809	1	#DIV/0!
Rnaseh2a|69724	1.166653034	0.160111596	1.195747965	0.101706556
Osgin1|71839	1.16660773	0.296477255	-1.036254343	0.445502778
Arpc1a|56443	1.166321998	0.014073803	1.134148248	0.030524625
Wnt5b|22419	1.166311979	0.27185553	1.561395254	0.009759317

Rassf4|213391	1.165965297	0.298663824	-1.188779916	0.208442043
Mms19|72199	1.165895297	0.158131813	1.165895297	0.158131813
Nsmaf|18201	1.165758579	0.279015627	1.208642219	0.202194994
Smcr7l|239555	1.16572152	0.126726692	1.16572152	0.126726692
Ogfod1|270086	1.165707974	0.246182461	1.165707974	0.246182461
Cox4i1|12857	1.165702504	0.191239571	1.048661701	0.379010864
B230120H23Rik|65964	1.165668099	0.203389292	1.158128358	0.113607191
Rsbn1|229675	1.165620097	0.426363226	2.791782706	0.160879221
Ostb|330962	1.165607184	0.42418314	-2.583325975	0.0615742
Asb8|78541	1.165604581	0.047166197	1.219838404	0.010565331
Fam115a|77574	1.165278719	0.388616192	1.625890204	0.215871312
Dcaf12l1|245404	1.165151326	0.369954098	-1.416853238	0.186792482
Rgl2|19732	1.165104123	0.070782252	1.209133685	0.007229236
C030046I01Rik|109284	1.165006301	0.068804386	1.0845802	0.128166682
Cdk7|12572	1.164875459	0.101935362	1.011544208	0.427996311
Grn|14824	1.164765129	0.062194536	1.24194931	0.012229376
Angpt2|11601	1.164692899	0.393499011	1.164692899	0.393499011
Chfr|231600	1.164470396	0.226213388	1.083080115	0.353731045
Wdr43|72515	1.164405924	0.019743762	1.109789778	0.031234034
Wtip|101543	1.164404941	0.352076436	1.150012803	0.295984863
Psmb7|19177	1.164348704	0.08163882	1.08772598	0.167684911
Agfg1|15463	1.164322887	0.263260067	1.240147341	0.127425522
Zfp26|22688	1.164279988	0.292748059	1.164279988	0.292748059
Jun|16476	1.164131667	0.254323394	1.164131667	0.254323394
Rab27a|11891	1.164090272	0.264119268	1.256422555	0.197077496
Nat8|68396	1.16401302	0.385167083	1.587020173	0.217829834
Gm5148|381438	1.163854664	0.180585177	-1.045202742	0.3255113
Nlrc4|268973	1.16372579	0.422523456	3.168797619	0.09792829
Swap70|20947	1.163702847	0.280855531	1.278592757	0.167460027
Psmg3|66506	1.163669249	0.195591639	1.257030467	0.086325629
Naca|17938	1.163640791	0.120103657	1.017576566	0.427177022
AI462493|107197	1.163559204	0.230163474	1.019022719	0.457423985
Dnaja1|15502	1.163535621	0.157059608	1.332777518	0.007498539
Narf|67608	1.163408155	0.20905417	1.44220022	0.008961513
Nol10|217431	1.163387431	0.099181254	1.163387431	0.099181254
Pabpn1|54196	1.16333846	0.161996896	1.16333846	0.161996896
G3bp1|27041	1.1633089	0.049287991	1.224948705	0.014284447
4933432B09Rik|75729	1.163217594	0.392816686	1.163217594	0.392816686
Gm5595|434179	1.163196616	0.390918424	-1.05368395	0.461995549
Ccdc8|434130	1.163186547	0.41543557	2.766348671	0.092098057
Atp6v1b2|11966	1.163110264	0.118727428	1.114058547	0.139578995
Cd74|16149	1.163023583	0.327431044	-1.093577488	0.274410385
Senp7|66315	1.162996105	0.27425017	1.419020146	0.068828883
Ube2s|77891	1.162939481	0.26677358	1.162939481	0.26677358
Scn1b|20266	1.162810817	0.223426378	1.380876061	0.02753551
Dcun1d5|76863	1.162803367	0.156541447	1.162803367	0.156541447
Dpf3|70127	1.162690923	0.295279255	-1.035283093	0.407842961
0610009D07Rik|66055	1.162425057	0.151763494	1.172315132	0.099143558
Ncl|17975	1.162234905	0.193762659	1.038265138	0.397955814
1600002H07Rik|72016	1.162219803	0.147470103	1.162219803	0.147470103
Adck4|76889	1.162192222	0.270609737	-1.066939332	0.327854648
Tnfrsf10b|21933	1.162185708	0.365667533	1.162185708	0.365667533
E030024N20Rik|595139	1.162143576	0.145923907	1.150733776	0.083601265

Spata24|71242	1.162077256	0.271313675	1.009113988	0.482271509
Rpl27|19942	1.16196737	0.194773839	-1.049931577	0.334810077
Erp44|76299	1.161751203	0.027216292	1.171610047	0.007587586
Cct5|12465	1.161645811	0.086294276	1.208354185	0.00556123
Lrch4|231798	1.161566992	0.410569179	-1.394599698	0.322100701
Cpm|70574	1.161323527	0.256909404	1.071979224	0.346473023
Cbfa2t2|12396	1.16130561	0.275215492	1.317867311	0.152013569
Rps3|27050	1.161283059	0.239764654	1.004836475	0.488985137
Snhg7|72091	1.161280123	0.393876865	-1.503998666	0.17396124
S100a13|20196	1.161095588	0.281465999	-1.028224717	0.4500283
Zfp566|72556	1.16108863	0.390553888	1.16108863	0.390553888
Adamts13|279028	1.161087794	0.215393751	1.455447902	0.007272478
Aldoa|11674	1.160848782	0.093102509	1.109875596	0.175892906
D18Ertd653e|52662	1.160774387	0.285201452	-1.005633676	0.49097026
Shfm1|20422	1.160688232	0.202178811	-1.048296868	0.355674858
Rpl13|270106	1.160592669	0.271582858	-1.159189434	0.14998973
Atp5l|27425	1.160436789	0.24368019	-1.103942301	0.242373302
Lmod1|93689	1.160402055	0.386720902	1.439603776	0.177448313
Zbtb46|72147	1.160305793	0.375924678	-1.153811245	0.383911645
Snx25|102141	1.160003706	0.263464496	-1.025366411	0.445767895
Jmy|57748	1.159937187	0.375209935	1.054640109	0.440921962
Tigit|100043314	1.159865072	0.427102472	1.099838978	0.465920044
Cog6|67542	1.15986367	0.300462768	1.315429802	0.143214961
Prdm16|70673	1.159776501	0.400376053	1.159776501	0.400376053
Snhg8|69895	1.159746502	0.267669868	-1.017098338	0.466398702
Tex9|21778	1.159733333	0.42415282	1.178571429	0.43416
Dtymk|21915	1.159629695	0.117022409	1.091985778	0.220797368
Slc2a3|20527	1.159487881	0.383230263	-1.2570926	0.316762244
E130201H02Rik|78552	1.159472653	0.341311184	-1.030077964	0.462411479
Snrnp40|66585	1.159433418	0.260569003	1.203209039	0.197828705
Cd300a|217303	1.159361331	0.207364167	1.044708098	0.395475592
Mfap1a|67532	1.159227979	0.247401194	1.150932581	0.21751729
Kin|16588	1.15921373	0.099930596	1.202714858	0.039777699
Pglyrp2|57757	1.158971254	0.025995486	1.103180202	0.044345243
Ndufaf2|75597	1.158897299	0.132607238	1.338834807	0.005113125
Slc2a10|170441	1.158863651	0.436660012	1	#DIV/0!
Hipk4|233020	1.158863651	0.436660012	1	#DIV/0!
Synpo2|118449	1.158863651	0.436660012	1	#DIV/0!
Nkain2|432450	1.158863651	0.436660012	1	#DIV/0!
Lpar4|78134	1.158863651	0.436660012	1	#DIV/0!
4932441J04Rik|319216	1.158863651	0.436660012	1	#DIV/0!
C330027C09Rik|224171	1.158863651	0.436660012	1	#DIV/0!
9530026P05Rik|330385	1.158863651	0.436660012	1	#DIV/0!
Mrpl54|66047	1.158764347	0.105379825	1.094898494	0.206662337
Gtpbp4|69237	1.158742929	0.177955077	1.074655078	0.321482798
Id2|15902	1.158422734	0.255485487	1.355247924	0.082215697
Smap1|98366	1.158205772	0.197489994	1.419911954	0.006595859
St8sia3|20451	1.158067954	0.397489773	-1.431454432	0.242113817
Neurl2|415115	1.158045109	0.353358389	-1.060786023	0.442233181
2310037I24Rik|69612	1.158034297	0.242912253	1.241087173	0.026669592
Bsdc1|100383	1.157688612	0.060077883	1.060041575	0.208077039
Depdc7|211896	1.157570045	0.290247252	1.091007119	0.382737645
Prrt4|101359	1.157231515	0.445548341	1	#DIV/0!

Comp|12845	1.157231515	0.445548341	1	#DIV/0!
Sema4f|20355	1.157231515	0.445548341	1	#DIV/0!
Nfkb2|18034	1.156492186	0.27321381	1.049475562	0.344348599
Fxr1|14359	1.156478678	0.149642465	1.266586697	0.042114686
Zbtb37|240869	1.15646318	0.413262928	1.15646318	0.413262928
Sptlc2|20773	1.156366172	0.136803192	1.156366172	0.136803192
Taf9|108143	1.156349987	0.156995475	1.259026828	0.056900966
S1pr2|14739	1.156140399	0.18042108	-1.024986793	0.410261811
Sema4d|20354	1.156082168	0.36873617	-1.188755473	0.334695565
Mak16|67920	1.156070102	0.196802718	1.156070102	0.196802718
Arhgap25|232201	1.1558242	0.356740394	-1.245578467	0.212444371
Gm10136|672214	1.15577187	0.320520934	1.105995862	0.288734726
Nsmce1|67711	1.155681751	0.124442583	1.072529095	0.169192818
Psmb5|19173	1.155672019	0.023221132	1.166185418	0.008130138
Ctnnb1|12387	1.155620734	0.213534459	1.300019028	0.073073705
Cd3e|12501	1.155611435	0.362793964	1.040642049	0.4569728
Atp5g2|67942	1.155593618	0.204099209	1.013339023	0.457785882
Pcdhgb8|93705	1.155503877	0.054290458	1.18096429	0.011554789
9530068E07Rik|213673	1.1554161	0.048928813	1.1554161	0.048928813
Siah2|20439	1.154983427	0.202483011	1.309049058	0.049261957
Mrgpre|244238	1.15490362	0.269846423	1.205414388	0.079817368
Zfp108|54678	1.154856193	0.380059341	-1.231043035	0.322061312
Zfp954|232853	1.154842106	0.244402994	1.085031702	0.273145666
Pgls|66171	1.154821193	0.233026807	1.045123782	0.401365571
Jdp2|81703	1.154787722	0.201918613	1.235510412	0.105485418
Lpcat3|14792	1.15478236	0.113662177	1.098560146	0.20592672
Skp1a|21402	1.154680651	0.091834578	1.15013522	0.045301882
Rhob|11852	1.154665872	0.145183055	1.066110938	0.294161365
Clec12a|232413	1.154616706	0.371418162	1.177925184	0.351610501
2310061C15Rik|66531	1.154493819	0.245812092	1.016275506	0.46574485
Rnd3|74194	1.154484219	0.252854126	1.474771095	0.01565874
Magix|54634	1.154480294	0.167928755	1.154480294	0.167928755
Ttc1|66827	1.154440193	0.14431398	1.220945005	0.066081082
Fam108b|226016	1.154355974	0.258856484	1.298445479	0.083462925
Gpx4|625249	1.154113326	0.294541533	-1.094639131	0.289155216
1600027N09Rik|73247	1.154040115	0.319636713	1.154040115	0.319636713
Trim28|21849	1.153950477	0.093025743	1.083236638	0.191763239
Atg2b|76559	1.153937051	0.073648238	1.104631096	0.146358617
Lrpprc|72416	1.153858453	0.151294172	1.349662266	0.004039182
H2afj|232440	1.153724189	0.270759701	-1.124178559	0.246690797
Usmg5|66477	1.153600181	0.181695062	1.153600181	0.181695062
Gm8801|667766	1.153553769	0.376751597	1.153553769	0.376751597
Prpf18|67229	1.15348798	0.107726971	1.053673082	0.224191991
Insc|233752	1.153110454	0.302208661	-1.077509962	0.360753886
Pigyl|66268	1.153105171	0.191516606	-1.046125705	0.30708359
Heph|15203	1.153024677	0.422186822	-1.663186782	0.253509159
Cdkn1b|12576	1.152897888	0.225860238	1.311565417	0.070227029
Ift74|67694	1.152871266	0.330895973	1.290577351	0.242177014
Ak4|11639	1.152830256	0.204293294	1.152830256	0.204293294
Smndc1|76479	1.152735978	0.286424268	1.064160638	0.409600658
Tiprl|226591	1.152683772	0.146654626	1.140238104	0.119680473
Aass|30956	1.152570097	0.309987163	1.390726403	0.130387816
Mef2d|17261	1.152495196	0.304971442	1.174122496	0.276934983

Tbc1d8b|245638	1.152375961	0.377418904	1.521317836	0.149729106
Vamp8|22320	1.152347821	0.22647601	1.029645966	0.431791392
Dock4|238130	1.15229137	0.311044225	1.300049053	0.111134036
Dhps|330817	1.152291368	0.132844251	1.00235009	0.489134741
Lcor|212391	1.152137571	0.425454842	-1.614048851	0.284375762
Efna5|13640	1.152063638	0.205023312	1.079351604	0.33334727
1700030J22Rik|69528	1.152006208	0.388445953	1.152006208	0.388445953
Napsa|16541	1.151815193	0.411039032	1.916350438	0.182743304
Gaa|14387	1.151801279	0.055390277	1.151801279	0.055390277
Mfap3|216760	1.151748925	0.122951198	1.252457366	0.025391592
2210010C17Rik|70080	1.151526102	0.380952436	1.650881959	0.154686998
Sos2|20663	1.151369088	0.359803619	1.233218732	0.303312009
Rnf168|70238	1.15135242	0.207714434	1.154611902	0.121747615
2810403D21Rik|69964	1.151218104	0.400387548	1.151218104	0.400387548
Adh6-ps1|639769	1.151162081	0.305397146	-1.162378606	0.239477405
Gcc1|74375	1.151037315	0.182391239	1.034015682	0.383151451
G6pc|14377	1.151007545	0.274037951	1.064312321	0.394797327
Cnot8|69125	1.150936069	0.083361293	1.068445197	0.159274431
LOC100504473|100504473	1.150894388	0.430487538	2.576568707	0.106702947
Pparg|19016	1.15088923	0.293701391	1.15088923	0.293701391
Cryzl1|66609	1.150805703	0.215852189	1.272482765	0.053996072
Esam|69524	1.150711804	0.175655103	1.059971999	0.328439467
Etohi1|626848	1.150623729	0.416440009	-1.407238997	0.319263545
Zfp580|68992	1.150535616	0.296061387	1.616834636	0.004819577
Srpk1|20815	1.150382119	0.156933755	1.345290454	0.006590245
Prdx4|53381	1.150273689	0.093439211	1.150273689	0.093439211
E330033B04Rik|319722	1.150229946	0.34780849	1.020818976	0.476145749
Atic|108147	1.150214756	0.019100404	1.115890919	0.03911489
Tcf7l2|21416	1.150184632	0.233505876	1.476805727	0.001312228
Gtf2h3|209357	1.149901225	0.166920515	1.053200566	0.338156921
Cnr2|12802	1.149808175	0.381493918	1.644864333	0.154547274
Ckap4|216197	1.149756151	0.241031984	1.267699622	0.036938109
Slc30a9|109108	1.149586158	0.037097662	1.215659659	0.003033565
1700047I17Rik2|100101807	1.149492227	0.305742056	-1.205843392	0.123638566
Chst15|77590	1.149486002	0.178056572	1.2903516	0.030521837
Vamp4|53330	1.149475472	0.345007029	1.2819547	0.264378606
Atp1a2|98660	1.149367457	0.354669091	1.191949507	0.273889597
Xpnpep1|170750	1.149317698	0.074792067	1.070477321	0.145582351
Exosc9|50911	1.149308754	0.078341409	1.149308754	0.078341409
Anp32b|67628	1.149143936	0.160820272	1.149143936	0.160820272
Sp2|78912	1.148946012	0.252633255	1.294856266	0.114937715
Rbm39|170791	1.148863664	0.2566526	1.112561476	0.196730077
Wdr82|77305	1.148688329	0.168119267	1.175179258	0.031167568
Slc29a3|71279	1.148686174	0.211083582	-1.04225567	0.378859139
Zfp367|238673	1.148586721	0.380241335	2.023635437	0.032752292
Cd27|21940	1.148374263	0.342977156	-1.26938726	0.189216339
Eml4|78798	1.148320134	0.223104508	1.148320134	0.223104508
Zfp280c|208968	1.148136411	0.316432902	1.309933983	0.148653935
Morn1|76866	1.148074205	0.201780852	1.148074205	0.201780852
Nudt4|71207	1.147880325	0.149922018	-1.003016843	0.487691114
Rps18|20084	1.147796218	0.247494157	-1.103808775	0.221336896
Metap1|75624	1.147680411	0.128042182	1.147680411	0.128042182
Manf|74840	1.147546987	0.300922162	1.345909771	0.059520841

Fbxl4|269514	1.147545327	0.231743279	-1.082771844	0.258045092
Tank|21353	1.14749013	0.309248619	-1.193182823	0.185740966
Smad7|17131	1.147475348	0.282627374	1.30046826	0.093570855
Rabac1|14470	1.147469858	0.228167097	-1.077817808	0.266112061
Pitpna|18738	1.147278383	0.037906978	1.238365146	0.001224966
Ticam2|225471	1.147262611	0.409276791	1.147262611	0.409276791
Rtcd1|66368	1.147247867	0.271168657	-1.001060614	0.497937235
D930014E17Rik|57373	1.147173058	0.240458063	1.1329362	0.219793462
1810008A18Rik|108707	1.147095631	0.045152134	1.181709484	0.022220303
Ppp3cb|19056	1.147094067	0.243247833	1.370295576	0.023640302
Ube2g2|22213	1.146991867	0.128812193	1.218949174	0.058184933
Rpl12|269261	1.146780407	0.193425371	1.241267495	0.066158631
Synrg|217030	1.146747203	0.103256654	1.058061795	0.212095793
Renbp|19703	1.146700664	0.392245954	-1.008682268	0.490291882
St14|19143	1.146683414	0.354306866	1.146683414	0.354306866
Txnip|56338	1.146658803	0.320872325	1.40486523	0.027058171
Fis1|66437	1.146652363	0.253451855	1.115498683	0.231963044
B3gat3|72727	1.146626863	0.114697486	1.068034916	0.239492441
Pef1|67898	1.146612669	0.072909497	1.146612669	0.072909497
Usp14|59025	1.146325399	0.194757852	1.280972096	0.050088798
Trim12c|319236	1.146198681	0.363380635	1.831209364	0.027928897
Rpl14|67115	1.145951889	0.336231052	-1.151888494	0.270239397
Pik3c2a|18704	1.145707543	0.330411407	1.282224412	0.159462641
Cisd1|52637	1.145553251	0.070653377	1.076357976	0.142242834
Sft2d3|67158	1.145461014	0.318802304	1.440978688	0.090072852
Gpaa1|14731	1.145384349	0.181629971	1.145384349	0.181629971
Lbh|77889	1.145240221	0.252759473	1.119377337	0.171593383
Chtf8|214987	1.145085352	0.138428473	1.145085352	0.138428473
Zmynd15|574428	1.14505623	0.267237934	-1.031001786	0.426185194
Abhd4|105501	1.144981009	0.031513073	1.144981009	0.031513073
0610007L01Rik|71667	1.144961907	0.055709044	1.20764869	0.011276811
Fam120b|67544	1.144839514	0.087984046	1.221247667	0.019095162
Capn1|12333	1.144815551	0.075825706	1.194300156	0.037333779
Dhx35|71715	1.144810795	0.35470205	1.609090033	0.071056997
Ccne2|12448	1.144753102	0.422299942	2.126739986	0.17308943
D430019H16Rik|268595	1.144436299	0.365012227	1.780720553	0.042091528
Samm50|68653	1.144365379	0.108445708	1.16789019	0.09471146
Mpv17l2|234384	1.144167044	0.177634255	-1.009504641	0.466316061
Ddx28|71986	1.14406509	0.191303223	1.14406509	0.191303223
2310030G06Rik|66952	1.144052767	0.151660953	1.144052767	0.151660953
Ccl21c|65956	1.144025586	0.391061623	1.138958497	0.361880689
Gm10591|100504239	1.144025586	0.391061623	1.138958497	0.361880689
Arpc3|56378	1.143942789	0.12561684	1.30128801	0.005237241
Foxred1|235169	1.143745245	0.168433428	1.232432059	0.04603072
Naa50|72117	1.143641405	0.128330051	1.143641405	0.128330051
Slc39a1|30791	1.143638147	0.113894953	1.112655793	0.118062444
Cacna1d|12289	1.143563005	0.249994897	1.143563005	0.249994897
Fam83d|71878	1.14348201	0.450025457	1	#DIV/0!
Tekt4|71840	1.14348201	0.450025457	1	#DIV/0!
C1ql1|23829	1.14348201	0.450025457	1	#DIV/0!
Spag5|54141	1.14348201	0.450025457	1	#DIV/0!
Nudt17|78373	1.14348201	0.450025457	1	#DIV/0!
5430427O19Rik|71398	1.14348201	0.450025457	1	#DIV/0!

Zfp872|619310	1.14348201	0.450025457	1	#DIV/0!
BC024582|414068	1.14348201	0.450025457	1	#DIV/0!
Ndufa6|67130	1.143476879	0.135858292	1.001551438	0.492395302
Hras1|15461	1.143414104	0.298434618	1.128748388	0.217907704
Hint1|15254	1.143400985	0.211927471	-1.062040118	0.286834604
Paqr3|231474	1.143379295	0.308831503	-1.000843117	0.498757991
Mtl5|17771	1.143260807	0.413969596	1.881281608	0.182743554
Exoc5|105504	1.143223752	0.285667422	1.143223752	0.285667422
H2-Q7|15018	1.143156813	0.096735321	1.23580479	0.010156905
Nrbp1|192292	1.143123872	0.140544215	1.054536436	0.296559001
Cno|117197	1.143069842	0.330839592	1.143069842	0.330839592
Lass5|71949	1.143066848	0.267008706	1.435849664	0.013010336
Slc17a9|228993	1.143040749	0.245983383	1.361964435	0.023816933
Med6|69792	1.142997705	0.33935336	1.062377199	0.428313441
Rbbp7|245688	1.142791516	0.153899709	1.068154596	0.286925005
Cmtm3|68119	1.142752314	0.207281152	1.212386229	0.140037366
Klhdc5|232539	1.142610491	0.262588306	-1.010800741	0.475512676
BC039771|408057	1.142564442	0.42572511	1.047677258	0.477214915
Ofd1|237222	1.142365734	0.304038421	1.399286551	0.083838302
Arl6ip6|65103	1.142355558	0.304081113	1.142355558	0.304081113
Tcta|102791	1.142322477	0.217434885	-1.057014325	0.321258859
Nek3|23954	1.142290982	0.345202243	1.191288201	0.291516057
Cd3eap|70333	1.142021653	0.134701682	1.058106697	0.282115022
Nkiras1|69721	1.141973969	0.148527595	1.141973969	0.148527595
Ufd1l|22230	1.141856707	0.079100598	1.141856707	0.079100598
H2-Eb1|14969	1.141849202	0.358026813	-1.124787212	0.225315482
D030028A08Rik|319371	1.141637662	0.358091205	1.696863396	0.043701708
Polr2k|17749	1.141545389	0.367610833	1.141545389	0.367610833
Pgk1|18655	1.141534555	0.046666514	1.141534555	0.046666514
Pbx3|18516	1.141414281	0.343193161	1.142644292	0.231256067
Bst2|69550	1.141382965	0.294360265	-1.062380995	0.369845513
Pelo|105083	1.14137083	0.242249534	-1.080163319	0.284066555
Ywhae|22627	1.141296555	0.066203511	1.137384019	0.025665964
Sass6|72776	1.141255913	0.415858729	2.253962154	0.101904273
Nemf|66244	1.141255484	0.315243511	1.509224741	0.04398915
Ctgf|14219	1.141077995	0.29004417	1.39387828	0.057603552
Mrp63|67840	1.141075686	0.199948929	1.137015464	0.126475126
Ppia|268373	1.140993754	0.221290958	1.129926286	0.185050493
Supt4h1|20922	1.140837164	0.155773604	1.140837164	0.155773604
Chd1|12648	1.140725233	0.301850813	1.360284418	0.10977089
Itga9|104099	1.140667979	0.254910015	-1.047557847	0.325834542
Eef1b2|55949	1.140552289	0.193650158	1.055271417	0.354762848
Tars|110960	1.140511011	0.020965159	1.105248733	0.041607716
Fbxw9|68628	1.140463	0.132033653	1.140463	0.132033653
Enox|70252	1.140405521	0.372629303	1.140405521	0.372629303
Ocln|18260	1.140356193	0.153074661	1.187029263	0.098600762
Trmt1|212528	1.140300376	0.141910198	1.170676567	0.036936761
Ilkap|67444	1.13983299	0.268555941	-1.030048906	0.426798453
Rpl27a|26451	1.139773225	0.259110267	-1.001282127	0.497078481
4921530D09Rik|77058	1.139624595	0.434856741	-1.591003234	0.290870527
Dnajb9|27362	1.139597204	0.228418344	1.205750357	0.103702524
Gtpbp6|107999	1.139578929	0.164063219	1.031325287	0.36226935
Clec7a|56644	1.139478886	0.348724389	1.339368729	0.153620058

6330512M04Rik|320802	1.139394636	0.398028385	1.632142574	0.188564188
Sugt1|67955	1.139247739	0.202636829	1.028694215	0.407181186
Snrpd3|67332	1.139178828	0.149313849	1.223422969	0.031109717
Tardbp|230908	1.138889692	0.119038136	1.138889692	0.119038136
Hivep2|15273	1.138783108	0.346606718	1.380764204	0.179114075
App|11820	1.138733101	0.267870133	-1.119243958	0.211914655
Nle1|217011	1.138688088	0.292228058	-1.004682176	0.491789277
Schip1|30953	1.138631011	0.431458821	1.366291578	0.378497556
Utp18|217109	1.138627625	0.299988487	1.006445389	0.488353325
Polr1c|20016	1.138580528	0.101815657	1.172917677	0.068703043
Ptcd3|69956	1.138316728	0.130718032	1.138316728	0.130718032
Zfp750|319530	1.137970336	0.206327181	1.258602275	0.028519318
Tmem231|234740	1.137816447	0.434532488	-3.010586893	0.023511985
Myom3|242702	1.137688449	0.42284563	-1.576144205	0.250276096
Lpcat4|99010	1.137676271	0.281705407	1.255866405	0.135973942
Slx4|52864	1.137594533	0.187585739	1.134455174	0.043340438
Trmt61a|328162	1.137281611	0.179109476	1.326545761	0.010906753
Zbtb32|58206	1.137165207	0.389523198	1.355646318	0.233074117
Prkaa1|105787	1.137118469	0.328321559	1.311379102	0.193097228
Manbal|69161	1.137107743	0.249398231	1.137107743	0.249398231
Chchd1|66121	1.137095002	0.289441908	1.027307261	0.450521303
Aldh1a3|56847	1.137082	0.437865718	-1.085993843	0.458354237
Ahctf1|226747	1.137044954	0.204775504	1.267302189	0.054047309
2010109K11Rik|72123	1.136970067	0.348497052	1.136970067	0.348497052
Rbm17|76938	1.136969944	0.13061847	1.225274654	0.032078063
Braf|109880	1.136965852	0.30561685	1.136965852	0.30561685
Pea15a|18611	1.136835854	0.233647935	1.213269552	0.157376594
Rbbp4|19646	1.136757484	0.018254595	1.064453107	0.038657409
Ap2s1|232910	1.136639579	0.25279171	-1.094515811	0.231073154
Zfp513|101023	1.136471074	0.169839542	1.248833635	0.008378559
Baat1|231841	1.136018631	0.194612994	1.136018631	0.194612994
Tmed9|67511	1.135612491	0.196331105	1.274107861	0.0333333
Rpl34|68436	1.13560286	0.277583029	1.00784323	0.484854092
Ndufb3|66495	1.135488615	0.067447637	1.187771983	0.023654003
4833420G17Rik|67392	1.135469748	0.152051878	1.223613218	0.024275235
Trub2|227682	1.135396426	0.215113652	1.025552301	0.427403852
Pop4|66161	1.135094551	0.229193166	1.220289739	0.13915485
Gpsm1|67839	1.135033291	0.382420295	1.135033291	0.382420295
Serf2|378702	1.134925083	0.193981058	-1.054821246	0.238697633
Tsku|244152	1.134830149	0.269854534	1.018719169	0.463106146
Nox4|50490	1.134803062	0.399742787	1.352691087	0.256582045
Exosc2|227715	1.134761929	0.145002443	1.286434362	0.005283689
Iws1|73473	1.134750869	0.332461286	1.292600194	0.16535684
Galnt10|171212	1.134723437	0.328721029	-1.207999951	0.163658864
Fmnl1|57778	1.134721658	0.362315921	-1.188351331	0.273069579
Coro1a|12721	1.13459522	0.330498831	-1.236858558	0.126946476
Ccni|12453	1.134571622	0.284148776	1.005553628	0.489638542
Fxyd3|17178	1.134500912	0.414778359	-1.093903908	0.433766023
Gm10319|381806	1.134429553	0.395996908	1.134429553	0.395996908
2810001G20Rik|66456	1.134400018	0.349700474	1.134400018	0.349700474
Bbs10|71769	1.13434921	0.387329329	1.13434921	0.387329329
Mphosph10|67973	1.134020669	0.126801315	1.077765006	0.23371093
Dstn|56431	1.133718428	0.128124527	1.091833631	0.139251205

Eif1ax|66235	1.133711491	0.263197957	1.173678184	0.050474629
Rybp|56353	1.13363513	0.305672015	1.376731671	0.080456687
Kbtbd2|210973	1.13357922	0.293202097	1.13357922	0.293202097
Pkn2|109333	1.133550085	0.344608774	1.026555923	0.449012605
Rab17|19329	1.133489459	0.259939142	1.082164271	0.201085125
Timm22|56322	1.133300377	0.177573046	1.062268697	0.314469406
Ptrh2|217057	1.133238285	0.274434874	1.133238285	0.274434874
Ddx31|227674	1.133229372	0.281462625	1.043134144	0.426151837
Aimp2|231872	1.133154626	0.186129161	1.04150057	0.362058559
Lmbrd1|68421	1.132933647	0.135524893	1.201228215	0.010309917
Ascc1|69090	1.132924717	0.331237825	1.130699502	0.304390108
Aamp|227290	1.132771557	0.073514479	1.030448708	0.228217483
Mapk8ip1|19099	1.132758356	0.259390071	1.190673277	0.138375942
Usp37|319651	1.1326776	0.258876597	1.1326776	0.258876597
Gcc2|70297	1.132589541	0.33928367	1.675189478	0.007733013
Pop1|67724	1.132580746	0.302789179	1.053684205	0.4024549
Scyl2|213326	1.132336793	0.352586588	1.724929901	0.013543739
Eif1|20918	1.132261383	0.136836261	1.11872472	0.059133129
Mycbp|56309	1.132126037	0.280565058	-1.00377231	0.491303669
Arl8a|68724	1.13211927	0.157929076	1.13211927	0.157929076
Paip1|218693	1.132008919	0.271469799	-1.05069694	0.356014088
Ndufs3|68349	1.131913732	0.152764156	1.067929014	0.277898424
Xpo7|65246	1.131889737	0.189046524	1.332823383	0.002526265
Isy1|57905	1.131619868	0.113087708	1.157318321	0.091754731
2610034B18Rik|70420	1.131538246	0.192368774	1.025532747	0.409937697
Apex1|11792	1.131510177	0.180613253	1.105652687	0.138621041
Dhx9|13211	1.13146264	0.315904289	-1.04510231	0.42503072
Pik3ip1|216505	1.131360416	0.408329283	1.820585212	0.140133306
Ddhd2|72108	1.131356235	0.247580194	1.220033334	0.027624213
Fxr2|23879	1.131339461	0.127742847	1.179887352	0.064675842
Slc43a3|58207	1.131272662	0.279551748	1.043583551	0.41791025
Shoc2|56392	1.131255939	0.161072331	1.212455762	0.06366507
1700020O03Rik|70373	1.13100131	0.320573485	1.276307096	0.15333604
Plcg2|234779	1.130967943	0.41268354	1.762413835	0.177476566
Senp2|75826	1.130916548	0.183214106	1.216935388	0.079847042
Slc25a15|18408	1.130800647	0.197282054	1.130800647	0.197282054
9530008L14Rik|109254	1.130793228	0.033980373	1.171260869	0.006057155
Rasgrp1|19419	1.130785223	0.417799498	1.382707879	0.287824254
Sec22a|317717	1.130732397	0.25026095	1.083161244	0.245731811
Pkn3|263803	1.130710176	0.352001452	1.279311313	0.210354229
Taf10|24075	1.130635234	0.191416885	1.130635234	0.191416885
Psmc2|19181	1.130450208	0.154541414	1.297282532	0.004599688
2900064A13Rik|73024	1.130446857	0.054632528	1.171857246	0.023568047
Agxt|11611	1.130439167	0.353129068	-1.206270439	0.163383557
Tom1|21968	1.130408406	0.249964031	-1.09464345	0.166571757
Gin1|252876	1.130404299	0.35308288	-1.075445234	0.409636013
Isg20|57444	1.130366171	0.3109783	-1.011507207	0.464811901
Rab33b|19338	1.130356415	0.255918756	1.063371648	0.3450662
Trpt1|107328	1.130321287	0.270879082	1.24207204	0.059926649
Psma2|19166	1.13019906	0.152305726	-1.004910803	0.477307752
Orai1|109305	1.130172643	0.302862329	1.050256033	0.352598907
Kat5|81601	1.130167353	0.218732237	1.130167353	0.218732237
Chmp7|105513	1.13014507	0.103526023	1.247546134	0.002643431

Slc27a4|26569	1.1301229	0.095052187	1.208403487	0.012889648
Gm16379|100040259	1.130115549	0.160693977	1.038876534	0.344439687
Lyrm7|75530	1.129924834	0.289062559	-1.06113203	0.344642346
Snap23|20619	1.129699072	0.188044944	1.298697276	0.01679915
Haus4|219072	1.129630135	0.191230672	-1.022077352	0.417750454
Rsph3b|100037282	1.129586656	0.288868752	1.084236263	0.298555753
Ero1lb|67475	1.129551913	0.213788692	1.129551913	0.213788692
Spast|50850	1.129519797	0.25558026	1.129519797	0.25558026
Srp54b|665155	1.12920609	0.205148667	1.091824391	0.181486743
Mapk8|26419	1.129164172	0.32023691	-1.015122073	0.464977129
Jmjd6|107817	1.129133132	0.146184829	1.048595646	0.30016699
Mrpl24|67707	1.12911917	0.152902014	1.041519365	0.321832629
Atp6v0e|11974	1.128981398	0.168659169	1.128981398	0.168659169
Micalcl|100504195	1.128943925	0.39412171	1.445613809	0.232648402
Atp6v0d1|11972	1.128878522	0.107804293	1.01941659	0.394057832
Chd2|244059	1.128526764	0.316926909	1.011497587	0.482760409
Rpl8|26961	1.128514613	0.293768941	-1.035323551	0.426428457
Anxa3|11745	1.128476551	0.343033323	1.579355375	0.040664712
Eif4h|22384	1.128287379	0.052040467	1.180940503	0.004210742
Abca6|76184	1.128277791	0.181956387	1.126500485	0.081656236
Mafk|17135	1.128194351	0.176481673	1.227377517	0.051038731
Rab2a|59021	1.128152921	0.036828917	1.095477126	0.075885566
Pwp2|110816	1.12797424	0.246740558	1.01823287	0.41130735
Rap1b|215449	1.127959354	0.298410619	1.127959354	0.298410619
Acot12|74156	1.127953019	0.219238971	1.21794313	0.112743624
Ttc35|66736	1.127934514	0.29276179	1.02079961	0.460012465
Naa40|70999	1.127885547	0.283724049	1.303634778	0.09678833
Impdh2|23918	1.127871259	0.275267934	-1.133178768	0.11956072
Trim3|55992	1.127778135	0.217424499	1.315658644	0.017158331
AF251705|140497	1.127732188	0.373226109	-1.163697053	0.327287398
Atp8b2|54667	1.127725377	0.37282551	1.470824381	0.155181122
Vdac3|22335	1.127694038	0.097062363	1.127694038	0.097062363
Cxcl16|66102	1.127637845	0.306057524	1.275544137	0.165614541
Rspry1|67610	1.12760233	0.332233783	1.128965973	0.305372746
Tbpl1|237336	1.12758037	0.224108367	1.12758037	0.224108367
Agmo|319660	1.127550712	0.298993849	1.127550712	0.298993849
Iqcc|230767	1.127346943	0.350169816	1.127346943	0.350169816
Slc15a4|100561	1.1273229	0.141578019	1.002403681	0.483833238
Sepw1|20364	1.127322799	0.341468119	1.052445057	0.39527639
Ndnl2|66647	1.127263403	0.25989727	1.042447354	0.413616691
Dhx33|216877	1.127229807	0.214505687	1.006956549	0.474781968
Psmd4|19185	1.127202226	0.19467804	1.183007454	0.117194886
Lphn3|319387	1.127181484	0.439276142	1.264185714	0.40907038
Samd14|217125	1.127145741	0.35555491	1.115705188	0.351482659
Fbxo31|76454	1.127107811	0.167819826	1.044712415	0.33307028
Hs1bp3|58240	1.127106239	0.217125382	1.127106239	0.217125382
Usf2|22282	1.127055432	0.130235103	1.042053208	0.284019516
Acot2|171210	1.126977944	0.281024259	-1.130994779	0.167623956
Frmd6|319710	1.126928465	0.38559891	1.600234848	0.120697043
Fbxw2|30050	1.126906121	0.034477489	1.138397123	0.002141452
Rab31|106572	1.126900757	0.366180208	1.126900757	0.366180208
Tnks|21951	1.126850605	0.357204412	1.171926685	0.292126327
Smchd1|74355	1.126769704	0.37758527	1.134260911	0.351395001

Arpc1b|11867	1.126707304	0.241534803	1.018499786	0.452457497
Vasn|246154	1.126673505	0.205314171	1.091373736	0.166918566
Gde1|56209	1.126670784	0.131354162	1.051449647	0.272289563
Mafg|17134	1.126648583	0.27035923	-1.097138172	0.263977721
Ngrn|83485	1.126616806	0.18617372	-1.023915403	0.40554771
Cnot6l|231464	1.126612428	0.323081628	1.120394382	0.318559183
Fndc3b|72007	1.126558909	0.404232826	1.887919501	0.0780449
Mthfr|17769	1.126554434	0.310055044	1.126554434	0.310055044
Ppp2r2d|52432	1.126516068	0.221966551	1.013670023	0.457115156
Ube2e2|218793	1.126511744	0.23747899	1.126511744	0.23747899
Hddc2|69692	1.126476661	0.377366391	1.347415124	0.180170065
Psmd13|23997	1.12637904	0.192778476	1.12637904	0.192778476
Hspe1|15528	1.126344568	0.084196427	1.064477405	0.173311522
Cox7a2l|20463	1.126277324	0.236436385	-1.012711504	0.454636332
Atp5o|28080	1.125600851	0.255991862	1.031968981	0.429962476
Tuba4a|22145	1.125555766	0.250311023	1.086542331	0.277659836
Guca1b|107477	1.125447215	0.423080006	1.73392566	0.215742312
Cdk10|234854	1.125330762	0.262296254	1.009005369	0.478988323
Eapp|66266	1.125256015	0.299702658	1.357535852	0.058964696
Pikfyve|18711	1.125224923	0.317701436	1.125224923	0.317701436
Hpcal1|53602	1.125151473	0.196158081	1.125151473	0.196158081
Ccdc28a|215814	1.125099099	0.247734087	1.368087338	0.011691535
Cyr61|16007	1.125090783	0.339952673	-1.293227369	0.014941387
St6galnac3|20447	1.125067139	0.415426784	1.125067139	0.415426784
Crip2|68337	1.125028469	0.154752341	1.125028469	0.154752341
Blm|12144	1.124832446	0.400669909	1.639626697	0.153900282
Atl2|56298	1.124580009	0.27288748	1.119462569	0.19600951
Brp44l|55951	1.124530528	0.202003683	1.01652205	0.438332657
Arl15|218639	1.124450833	0.363870125	1.002809207	0.496605638
Gm5424|432466	1.124447973	0.096886941	1.07300761	0.191253667
Dym|69190	1.124347814	0.207910665	1.049631278	0.364917036
Kit|16590	1.124345268	0.360944178	1.709276021	0.006639052
2310039H08Rik|67101	1.124190911	0.239666556	1.244364941	0.047910727
Atg4d|235040	1.12410999	0.208590939	1.039565974	0.385988293
Nvl|67459	1.124105852	0.251885653	1.345124441	0.035405022
Sema4c|20353	1.124099816	0.282873478	1.18252117	0.044674017
D17Wsu92e|224647	1.124090428	0.040170743	1.168831064	0.009108722
Fam105b|432940	1.124023293	0.225538847	1.046719931	0.376481402
Prdx3|11757	1.124020898	0.210385835	1.277339128	0.023374027
Ubl4|27643	1.123954699	0.123292611	1.0117767	0.437667716
Actr10|56444	1.123887562	0.04024881	1.151052394	0.020933159
Arhgap10|78514	1.123872558	0.31764132	1.262632556	0.145567062
Htatsf1|72459	1.123871506	0.285949529	1.063082908	0.365691742
Avpr1a|54140	1.12385988	0.188098404	1.029312907	0.386303504
Tox|252838	1.123785428	0.367111013	1.390575844	0.181635837
Usp38|74841	1.123701284	0.28556311	-1.089221265	0.31042962
Gars|353172	1.123695004	0.170394627	1.053596116	0.322761316
Mfn1|67414	1.123681762	0.103600004	1.234251986	0.007203512
Gtf2b|229906	1.12359977	0.326557667	-1.032320139	0.440583913
Vps8|209018	1.123504327	0.287838089	1.067782402	0.287196272
Hsd17b11|114664	1.123476634	0.169064229	1.078662149	0.268540644
Snx21|101113	1.12346875	0.266558556	1.295743301	0.062954804
Hspd1|15510	1.123444355	0.194088438	1.132050637	0.125824777

Cox11|69802	1.123345746	0.326099419	1.240406723	0.223942332
Cep97|74201	1.123339303	0.315051786	1.206438429	0.159910425
Osgep|66246	1.123262551	0.325887262	-1.175021267	0.140960969
Smagp|207818	1.123228758	0.203025227	1.150306933	0.095710491
Naga|17939	1.123208852	0.265073791	1.097664113	0.220069494
Hadhb|231086	1.123201248	0.127485916	1.085374575	0.146086021
Rps20|67427	1.122856963	0.317413827	-1.200904043	0.097621772
Tspyl4|72480	1.122784242	0.402918408	1.122784242	0.402918408
Fbxo32|67731	1.122735618	0.398775667	1.122735618	0.398775667
Sfpq|71514	1.122590233	0.269387203	1.00453315	0.489546964
Wdr12|57750	1.122583714	0.191797913	1.222334826	0.059108249
Aacs|78894	1.122459437	0.25218221	1.122459437	0.25218221
Csgalnact2|78752	1.122371844	0.410497785	1.331675987	0.247895672
Mttp|17777	1.122317225	0.186310296	1.122317225	0.186310296
Gorab|98376	1.122236704	0.399060604	-1.155352767	0.38056666
Rpgrip1|77945	1.122115712	0.266602609	1.028975688	0.438539065
Ebna1bp2|69072	1.122070197	0.174429979	1.122070197	0.174429979
Pacs2|217893	1.121768422	0.310047451	1.121768422	0.310047451
Ccl27a|20301	1.121754161	0.377451264	1.416634455	0.185718155
Hoxb7|15415	1.121607891	0.424993464	1.577793729	0.260321642
Ahcy|269378	1.121397458	0.183165645	1.044443693	0.352226223
Msmp|100039672	1.121348203	0.380079534	1.043357472	0.439073508
Cct7|12468	1.121274422	0.130750211	1.121274422	0.130750211
Parn|74108	1.121206693	0.173185496	1.289083596	0.005132453
Azi2|27215	1.12116008	0.167419147	1.188227319	0.080933827
Psma1|26440	1.121069361	0.211779132	1.079963544	0.302402263
Arpc4|68089	1.121023205	0.270804065	-1.015083124	0.461426035
Grb10|14783	1.120843348	0.392875511	1.88970882	0.038412476
1110065P20Rik|68920	1.120840377	0.240374976	1.052724207	0.372818074
Plg|18815	1.120805499	0.234588837	-1.091186899	0.046241758
Rps15|20054	1.120568444	0.356954548	-1.132435868	0.316393068
Abcc1|17250	1.120528292	0.411915146	-1.062160652	0.450449683
2010016I18Rik|69206	1.120434388	0.439605144	2.617043494	0.129315134
Prdx2|21672	1.120279702	0.234090047	-1.001946053	0.493348415
Med4|67381	1.120246197	0.250474216	1.00677349	0.481655694
Kif3b|16569	1.120094525	0.253232674	1.342688548	0.028954104
Dip2b|239667	1.12007934	0.307225196	1.022259975	0.462722336
Prickle1|106042	1.120045863	0.328401967	1.342555914	0.117521122
Myct1|68632	1.120010943	0.364673381	1.314094304	0.222526232
Adam15|11490	1.119764232	0.319376267	-1.102153985	0.262501137
Timm17a|21854	1.11974161	0.262932221	1.214579022	0.152632781
Fam81a|76886	1.119738351	0.369593556	1.452863561	0.131267206
Nagk|56174	1.119716107	0.195629531	1.133005654	0.122695438
4930412F15Rik|242408	1.119592667	0.250954854	-1.091553481	0.189306101
G0s2|14373	1.119586107	0.34865589	1.119586107	0.34865589
Pnpla3|116939	1.119507957	0.445737271	3.579326525	0.091893115
Nol11|68979	1.11948959	0.297627243	1.331299583	0.062192149
Mtmr2|77116	1.119379175	0.338608391	1.011851938	0.477567804
Tbl1xr1|81004	1.11937854	0.138251627	1.096170878	0.090496865
Cd276|102657	1.119294762	0.408518471	2.286181103	0.004495831
Ncapd3|78658	1.119264098	0.367736782	-1.349397496	0.074693571
Zbtb33|56805	1.119165167	0.399645383	2.103906183	0.00438707
Ctns|83429	1.119137347	0.204610943	1.036972844	0.385283508

Rpl22|19934	1.119110865	0.247978939	-1.015411375	0.447896178
Slc2a1|20525	1.119082622	0.30390884	1.035869276	0.395328644
Rps27|57294	1.119060447	0.311040551	-1.052897893	0.392509035
Bad|12015	1.118918752	0.27940989	-1.029676406	0.422627462
Rragc|54170	1.118734581	0.112511213	1.017378603	0.401600656
Ddx21|56200	1.118574801	0.226267122	1.025400072	0.420565479
Bcl2l14|66813	1.118513314	0.422201218	-1.338962617	0.313846823
Orc6|56452	1.118404163	0.333340935	-1.07228766	0.382604006
Gm608|207806	1.118383699	0.28878112	1.1708916	0.22842387
Pgp|67078	1.118243454	0.233964448	1.039661254	0.398641504
Rnf34|80751	1.118207547	0.373267431	-1.143480411	0.335881397
Tspo|12257	1.118050089	0.183212981	1.052335486	0.332973026
Gm5077|317677	1.118049184	0.423957991	-1.618448589	0.16183379
Stard3nl|76205	1.117825313	0.301538995	1.225361494	0.157183738
Frg1|14300	1.117787005	0.238682914	1.019263421	0.446321757
Psen2|19165	1.117738721	0.113849607	1.055192856	0.237408741
Snx17|266781	1.117726314	0.140644404	1.194380791	0.036431885
Ube2b|22210	1.117652196	0.129614987	1.087462109	0.078167177
Mug1|17836	1.117620966	0.280179348	1.117620966	0.280179348
1110002N22Rik|68550	1.117508429	0.284603896	1.117508429	0.284603896
Gart|14450	1.117459361	0.089957597	1.157538003	0.037711576
Fam59a|381126	1.117364344	0.262585933	1.150251563	0.08024772
Tspan5|56224	1.11728169	0.394744239	1.11728169	0.394744239
Lias|79464	1.116998424	0.190651982	-1.023243939	0.404806671
B4galt4|56375	1.116657996	0.436295632	2.111254404	0.170969182
Alas2|11656	1.116627271	0.347518366	-1.07671342	0.387910117
Mysm1|320713	1.116442483	0.251041472	1.116442483	0.251041472
Hspa8|15481	1.116285431	0.263206719	1.116285431	0.263206719
6330549D23Rik|229613	1.116273293	0.378167839	-1.154205059	0.329859314
Tuba1b|22143	1.116217616	0.286363576	1.025391405	0.404966378
Fam40a|229707	1.116149228	0.155408477	1.116149228	0.155408477
Bbs1|52028	1.116062518	0.351482078	1.36362387	0.139292471
Ccdc25|67179	1.116031925	0.205630213	1.281554432	0.020208186
Slco1a4|28250	1.115989677	0.270258396	1.149381171	0.111316054
Psph|100678	1.115858018	0.342847513	1.058102776	0.406316515
2410075B13Rik|223648	1.115650859	0.387097272	-1.155675184	0.34804895
Dis3l2|208718	1.115648282	0.186599413	-1.020370945	0.412379223
Dvl3|13544	1.115572925	0.323440355	1.115572925	0.323440355
Bik|12124	1.115465594	0.315349184	1.250122381	0.13206427
Egfl7|353156	1.115354783	0.210849052	1.024275631	0.418163534
H2-D1|14964	1.115279008	0.08370237	1.14373648	0.052251163
Fam195a|68241	1.11523235	0.174862143	1.169869475	0.086805188
Arhgap18|73910	1.115104739	0.359943233	1.115104739	0.359943233
Ifi27l1|52668	1.115032652	0.309497583	-1.035059837	0.42948865
Zfp13|22654	1.114999905	0.321225769	1.354218962	0.069816053
Ddx39|68278	1.114837619	0.154939717	1.044991965	0.314803552
Stxbp3a|20912	1.114780926	0.328626588	1.251508524	0.153969018
Tex10|269536	1.114774771	0.376983748	1.35050973	0.210628965
Dynlt1f|100040531	1.11472948	0.420408084	-1.317860292	0.311196402
Nkiras2|71966	1.114539216	0.208182502	1.114539216	0.208182502
Pex19|19298	1.114454758	0.179664141	1.114454758	0.179664141
Wdr41|218460	1.114451773	0.209259398	1.114451773	0.209259398
Fermt2|218952	1.114343801	0.255466338	1.21905046	0.082421872

Vkorc1|27973	1.114326089	0.296069268	-1.038099467	0.409538045
Gp9|54368	1.114277986	0.453634384	-1.631371973	0.295028973
Rps6|20104	1.114173639	0.266657404	1.009026986	0.477752604
Ppp2cb|19053	1.114171567	0.175371322	1.114171567	0.175371322
Tmem160|69094	1.114163259	0.348082978	-1.11937974	0.301128474
Rhog|56212	1.114042397	0.248051346	-1.072988166	0.262373883
Tsnax|53424	1.1140324	0.150401646	1.252835875	0.005500249
Srp14|20813	1.114021567	0.219682017	1.207379773	0.089248871
A730017L22Rik|613258	1.114015152	0.22223204	1.300130135	0.016412587
Cdipt|52858	1.113942117	0.085624631	1.113942117	0.085624631
C3ar1|12267	1.113937709	0.404661566	1.113937709	0.404661566
Tmem179b|67706	1.113601304	0.230955892	1.217084403	0.087042649
2700078E11Rik|78832	1.113550619	0.064076085	1.113550619	0.064076085
Rnf14|56736	1.113370898	0.210497712	1.256332714	0.010000732
Hnrnpab|15384	1.113297047	0.141508401	-1.007634329	0.448625931
Rnf38|73469	1.113286736	0.312408653	1.339708392	0.059091975
Hopx|74318	1.113259339	0.244710339	1.294826331	0.021627083
Abhd8|64296	1.113216387	0.29153867	1.00422148	0.491139787
Dmwd|13401	1.113165944	0.143968259	1.051572965	0.281114075
Tlr4|21898	1.113146667	0.329843654	1.113146667	0.329843654
Dynlrb1|67068	1.113136559	0.17596249	1.113136559	0.17596249
Ubqln1|56085	1.113101122	0.120848356	1.123398994	0.038140885
Sox18|20672	1.113020661	0.320090204	1.113020661	0.320090204
4732416N19Rik|320737	1.112908246	0.459065259	1	#DIV/0!
Serbp1|66870	1.112797178	0.04949704	1.112797178	0.04949704
Riok2|67045	1.112738314	0.119439081	1.003316652	0.466348262
C030044B11Rik|68128	1.112689096	0.331480769	-1.089911959	0.323698684
Mfsd3|69572	1.11266982	0.231144357	1.11266982	0.231144357
Phax|56698	1.112601183	0.25025659	1.111266186	0.197866713
Zfp930|234358	1.112529386	0.396557643	1.566439308	0.128662737
Tmem176b|65963	1.112472816	0.307782354	-1.075231844	0.301676224
Mtap7|17761	1.112444187	0.329940315	1.302889086	0.136418739
Rps21|66481	1.112415744	0.334878377	-1.222150605	0.071495105
Snhg12|100039864	1.112406147	0.363619154	-1.096180135	0.374759536
Rpl24|68193	1.112328832	0.33814535	1.122425971	0.275730149
Ptges2|96979	1.112320864	0.199455088	1.270154138	0.012067359
D17H6S53E|114585	1.112295769	0.284332904	1.235719106	0.13357643
Ndufs5|595136	1.112154691	0.2457132	1.040997218	0.318005002
Mxd4|17122	1.112091232	0.139435787	1.009465181	0.44757809
Mrpl33|66845	1.112003984	0.318195203	1.279192616	0.137570605
Fbxl18|231863	1.111967297	0.37903123	1.047810246	0.426725503
Timm8a1|30058	1.111870144	0.244679668	-1.066381531	0.268663788
Parvb|170736	1.111793898	0.282570035	1.013564124	0.470315749
Cdc20|107995	1.111781863	0.424618059	1.697935956	0.194810627
Sae1|56459	1.111719836	0.272720078	-1.104182711	0.17204154
5033406O09Rik|77675	1.111698799	0.315417191	1.043782112	0.407899506
Fkbp7|14231	1.11169118	0.420611024	1.11169118	0.420611024
Hspb8|80888	1.111642831	0.208319557	1.006822391	0.470038964
Armc10|67211	1.111547605	0.300094164	1.296857892	0.077816923
Gimap6|231931	1.111532038	0.198229461	1.182020416	0.07446177
Mrps33|14548	1.111467408	0.255757878	1.027543816	0.431644547
41157|18951	1.111460752	0.354699178	1.198983984	0.145100637
Ifrd1|15982	1.111440442	0.389704514	-1.163753566	0.337600415

Lnpep|240028	1.111372744	0.443757077	1.756849872	0.229137739
Rc3h2|319817	1.111325098	0.318931643	1.439146289	0.004440595
Sec23ip|207352	1.111280243	0.265215155	1.322451204	0.034145373
Ercc2|13871	1.111189483	0.196088162	1.094532336	0.174687484
Ywhaz|22631	1.111151948	0.102851489	1.06999992	0.194230109
Spc25|66442	1.111050032	0.431829872	1.381064559	0.3272513
Csf2rb2|12984	1.111038738	0.369577768	1.438080927	0.109069492
Nudt16l1|66911	1.111026427	0.204231553	1.03250345	0.389789436
Mrpl2|27398	1.110941254	0.280369017	1.110941254	0.280369017
Chd1l|68058	1.110875851	0.319016132	1.051836516	0.414061508
Hbp1|73389	1.110737222	0.15434808	1.031372773	0.338773118
Fkbp1a|14225	1.110722167	0.083424829	1.110697497	0.118416443
Rarg|19411	1.11069834	0.300871492	1.268064432	0.11336814
Yap1|22601	1.110650847	0.184868977	-1.024541349	0.376288184
Gata4|14463	1.110523932	0.254498514	1.328072806	0.006060889
Cox17|12856	1.110459779	0.269773809	-1.006798861	0.481555411
Diablo|66593	1.110442724	0.115482643	1.088168549	0.110231179
Upf3a|67031	1.11032848	0.301243485	1.226192218	0.167765602
Lonrf3|74365	1.110280287	0.308837084	-1.021394559	0.450211712
Eif2s3x|26905	1.110244226	0.161439981	1.110244226	0.161439981
Cog3|338337	1.110218602	0.295369454	1.22738887	0.115495149
Gpbp1l1|77110	1.110212835	0.247124148	1.110212835	0.247124148
5430435G22Rik|226421	1.11020524	0.363989091	1.409851382	0.111472716
Avpi1|69534	1.110168754	0.251435241	-1.009356237	0.468817417
C330019G07Rik|215476	1.11011682	0.305173358	1.442592532	0.003741134
Rps17|20068	1.110093438	0.315532532	1.107498923	0.222618544
Gata6|14465	1.110035802	0.328466995	-1.06662476	0.371760016
Iqcg|69707	1.110004634	0.272364279	1.00083027	0.497648125
Tab2|68652	1.109934233	0.338260967	1.230233836	0.190205395
Timm23|53600	1.109927272	0.088113515	1.058428815	0.181229465
Cct6a|12466	1.109564903	0.213667858	1.150569345	0.094470799
Tmsb10|19240	1.109420466	0.349264865	-1.261972821	0.052146408
Max|17187	1.10937131	0.14978965	1.099703302	0.077897626
Cntrob|216846	1.10934553	0.323450634	1.11142978	0.2970781
D030016E14Rik|320714	1.109282887	0.308752847	1.22959112	0.132381047
Ctsc|13032	1.109164749	0.147231891	1.191452472	0.023970835
Zc4h2|245522	1.10915968	0.445880011	-1.85002843	0.229359943
Mrpl17|27397	1.108999261	0.145266361	1.042031315	0.297809142
Rab11a|53869	1.108981417	0.159976124	1.108981417	0.159976124
Mcee|73724	1.108833856	0.157155781	1.168112336	0.07026117
Snrpd2|107686	1.10866929	0.305191602	1.14196801	0.200276239
Zfp809|235047	1.108584877	0.355771444	-1.158297685	0.209459607
Wipf1|215280	1.108406059	0.392973227	1.609669151	0.068368552
Pdhx|27402	1.1083711	0.203229039	1.194534415	0.072212822
2810025M15Rik|69953	1.108352721	0.33881455	-1.063705182	0.39047937
Gm16516|24082	1.10834004	0.425707244	1.89636615	0.122370382
Mbd5|109241	1.108314616	0.388015045	1.767515641	0.018534399
Hdac4|208727	1.108309651	0.428018349	1.9456525	0.12235555
Tacc2|57752	1.108279457	0.312163099	1.208697567	0.201849316
Jph2|59091	1.10822303	0.461153577	1	#DIV/0!
1700003F12Rik|75480	1.10822303	0.461153577	1	#DIV/0!
Ak7|78801	1.10822303	0.461153577	1	#DIV/0!
Adam22|11496	1.10822303	0.461153577	1	#DIV/0!

4930579F01Rik|67741	1.10822303	0.461153577	1	#DIV/0!
Nup62cl|279706	1.10822303	0.461153577	1	#DIV/0!
Tor1a|30931	1.108127369	0.148245383	1.041320862	0.307922352
2610029G23Rik|67683	1.108050836	0.34711603	1.431286558	0.031502504
Tnpo2|212999	1.10796638	0.09879867	1.052159048	0.204989245
Phtf1|18685	1.107955115	0.39889533	-1.271399476	0.234119093
Lmo4|16911	1.107900834	0.339495744	1.107900834	0.339495744
Pfdn5|56612	1.10788102	0.32869675	-1.038714855	0.430136312
Mfng|17305	1.107870774	0.381319473	1.107870774	0.381319473
Ankrd27|245886	1.107756515	0.277280937	1.107756515	0.277280937
Etfb|110826	1.107735636	0.322015826	-1.048044021	0.404180253
Ier2|15936	1.10772134	0.376586887	1.008115766	0.487473412
Slc45a3|212980	1.107654073	0.279148285	1.349513887	0.013853443
Arpp19|59046	1.107618873	0.296635882	-1.020910637	0.451275312
Mob3b|214944	1.107554557	0.214659366	1.175473745	0.116752555
Zfand6|65098	1.107505517	0.195827636	1.131366599	0.078886707
Sfxn3|94280	1.107462234	0.396054633	-1.345178719	0.088164404
Ept1|28042	1.1073488	0.268430509	1.315642198	0.000983409
Itfg3|106581	1.107306423	0.203173814	1.056411244	0.330919451
Tssc4|56844	1.107279678	0.30481233	1.107279678	0.30481233
Pigl|327942	1.10725823	0.296301642	1.10725823	0.296301642
Plekhh2|213556	1.107145396	0.438749971	-1.500772018	0.285957398
Lin52|217708	1.106912279	0.38589597	-1.080458945	0.397336681
Eif2s2|67204	1.106901677	0.096986609	1.074039599	0.179824239
Spin1|20729	1.106854273	0.239222077	1.192590642	0.115839589
C85492|215494	1.106822623	0.326436825	1.233523556	0.191066768
Rsl1|380855	1.106698059	0.42549747	1.552196866	0.235488401
Hars|15115	1.106636045	0.126911343	1.160735706	0.048299305
Psmd2|21762	1.106604503	0.032057165	1.106604503	0.032057165
Rpl37|67281	1.106561698	0.331117759	-1.196366831	0.10823891
Fam164a|67306	1.106459271	0.430093501	1.886640467	0.145598876
1700017B05Rik|74211	1.106430539	0.324872161	1.273415389	0.138546103
Tsr1|104662	1.106318966	0.318723165	1.106318966	0.318723165
Lag3|16768	1.10631389	0.382416157	1.10631389	0.382416157
Shprh|268281	1.106300208	0.370954237	1.402628545	0.123690386
Hist1h4h|69386	1.106171388	0.450112259	1.752000096	0.288047588
Stip1|20867	1.106131443	0.260001346	1.11268058	0.190229647
Rab3gap1|226407	1.106096381	0.24110096	1.106096381	0.24110096
Stx12|100226	1.106085038	0.314866774	1.106085038	0.314866774
Rbm27|225432	1.105960569	0.348135152	1.484468311	0.03014541
Mcm9|71567	1.105938731	0.410279998	1.042230547	0.462527448
Foxj2|60611	1.105915149	0.340138681	1.326689381	0.109021461
Creb3l1|26427	1.105826575	0.309263476	1.105826575	0.309263476
Abca7|27403	1.105760304	0.269152982	1.191276434	0.155566237
Bnip1|224630	1.105746757	0.216809934	1.11743185	0.137796962
Toe1|68276	1.10566176	0.346153789	1.490338632	0.025188178
Smad9|55994	1.105659015	0.389788719	1.519434448	0.099604753
Il17ra|16172	1.105644173	0.262628708	1.023495213	0.44045272
Lmtk3|381983	1.105575539	0.443294088	2.332659533	0.138606261
Ppp2r5c|26931	1.105400713	0.229366459	1.105400713	0.229366459
Atp1b1|11931	1.10528435	0.264034929	-1.050301723	0.356697458
Tmtc3|237500	1.105273198	0.378175086	1.344634259	0.190342406
Rpl17|319195	1.105212803	0.339760914	-1.213809916	0.10125559

Gemin5|216766	1.10499632	0.283018791	1.042077962	0.386522265
Kazald1|107250	1.104964044	0.412757383	1.221074732	0.329994789
Prorsd1|67939	1.104830815	0.278352573	1.104830815	0.278352573
Zcchc9|69085	1.104734249	0.219245134	1.104734249	0.219245134
Gm14005|100043424	1.104730097	0.420988366	1.104730097	0.420988366
Lhfp|108927	1.104707917	0.3451109	-1.010256819	0.475426806
Rpl15|66480	1.104701041	0.232518581	1.025068367	0.422380345
Ccdc101|75565	1.104697783	0.347351122	1.104697783	0.347351122
B3gntl1|210004	1.104632801	0.387361841	-1.336166911	0.14787827
Prmt10|102182	1.104506325	0.356867498	-1.069488431	0.397215205
Higd2a|67044	1.104454529	0.204101451	1.252594547	0.019630776
Mpnd|68047	1.104448947	0.281678605	-1.034063051	0.394441764
Cyp4f16|70101	1.104388978	0.343751079	-1.102378366	0.302677481
Ephx2|13850	1.10434538	0.203932119	1.021347798	0.415362395
Bet1l|54399	1.104318464	0.182970276	1.104318464	0.182970276
Elp2|58523	1.104273246	0.183980061	1.104273246	0.183980061
Uhmk1|16589	1.104242325	0.442297595	-1.016937286	0.491400683
D14Ertd449e|66039	1.104178616	0.204019839	1.224258065	0.014247022
Sdc4|20971	1.10388338	0.207575352	1.10388338	0.207575352
Rad51l1|19363	1.103865246	0.354098368	-1.097037384	0.341035662
Gng10|14700	1.103710801	0.405191532	1.474043782	0.1833363
Ppp3r1|19058	1.103681981	0.104908413	1.103681981	0.104908413
Rnf114|81018	1.103635494	0.145343177	1.001651995	0.490551332
Snora43|100306955	1.103507938	0.454669634	3.976520578	0.089749558
Pecam1|18613	1.103405377	0.147688096	1.156663963	0.065876235
Nsun5|100609	1.103304311	0.147732533	1.103840105	0.180193753
Clybl|69634	1.103289491	0.200312541	1.190288046	0.058819294
Pcgf5|76073	1.103232663	0.383032061	1.080553958	0.378077203
Amdhd2|245847	1.103170968	0.31167376	1.058144125	0.323790353
Kctd11|216858	1.103146432	0.394476925	-1.008899635	0.48514357
Psma6|26443	1.102993652	0.148059389	1.044874308	0.291050116
Rrm2b|382985	1.102808145	0.436357954	1.111313646	0.42052129
Rffl|67338	1.102737926	0.239353533	1.102737926	0.239353533
Rnf145|74315	1.102712375	0.326977668	1.381560987	0.008031962
Thrb|21834	1.102706034	0.330490784	1.447941129	0.011300893
D11Wsu99e|28081	1.102686668	0.248163419	1.008432938	0.473306343
Tmbim1|69660	1.102617442	0.103072648	1.102617442	0.103072648
Zbtb20|56490	1.102515896	0.39987432	1.121626463	0.352992644
Ncoa5|228869	1.102504067	0.174258205	1.043438007	0.323236153
Rad18|58186	1.10242024	0.425220975	1.005423262	0.496162914
Osbpl1a|64291	1.102396854	0.259269947	1.102396854	0.259269947
Mad2l1bp|66591	1.102389372	0.312271614	1.102389372	0.312271614
Bhmt2|64918	1.102236286	0.245212385	1.216522994	0.023994075
Lyrm1|73919	1.102181846	0.36840482	-1.083996121	0.357661446
Atp2a3|53313	1.102176128	0.350408673	-1.026731708	0.459346407
Tdrd7|100121	1.102038238	0.259739314	1.115477991	0.146100629
Srcin1|56013	1.101852091	0.398088262	1.31590084	0.196662591
Rps27l|67941	1.101777591	0.284126015	1.101777591	0.284126015
Wdr45|54636	1.10162642	0.143499985	1.152013627	0.049997403
Shroom2|110380	1.101561924	0.335200396	1.200616964	0.088502721
Gpn2|100210	1.101463525	0.223506337	1.219785165	0.030405443
Irf2|16363	1.101441558	0.23921039	1.211268409	0.06407405
Gipc1|67903	1.101430406	0.224632709	1.137278522	0.105467466

Rfwd2|26374	1.101213529	0.273018408	1.103215119	0.163024006
Oaz1|18245	1.101159311	0.280990155	1.07284229	0.264012062
Fam71e1|75538	1.101022257	0.347089668	1.101022257	0.347089668
Rpl11|67025	1.100840892	0.351992704	-1.075389298	0.375063628
Slc20a1|20515	1.100835527	0.224616823	1.142650777	0.083887681
Msrb2|76467	1.100631976	0.218996276	1.154113457	0.123338481
Sp3|20687	1.100627308	0.344304551	-1.05793388	0.399046982
Ndufa4l2|407790	1.100611451	0.426442481	-1.072120647	0.423102978
Pop5|117109	1.100583748	0.318434503	-1.011709287	0.473824517
Zfp622|52521	1.100484083	0.194686375	1.100484083	0.194686375
Rmnd5a|68477	1.100326817	0.236868444	1.295949109	0.002311151
Zmym5|219105	1.100208827	0.278939161	1.112814767	0.168590973
Setdb1|84505	1.100198468	0.309057068	1.361263979	0.030758345
Smyd5|232187	1.100143779	0.134708014	1.072867818	0.042728779
Exosc6|72544	1.100135003	0.34474886	1.100135003	0.34474886
Ifi205|226695	1.09998942	0.446630311	1.09998942	0.446630311
Zfp428|232969	1.099908918	0.385805846	-1.174224288	0.282660173
Utp15|105372	1.099842052	0.321219358	1.058811756	0.304960224
Pdzk1ip1|67182	1.099841188	0.341650858	1.099841188	0.341650858
Spint2|20733	1.099797553	0.231200916	-1.042593418	0.337333608
Mtmr1|53332	1.099790483	0.334100003	1.226614915	0.192132605
Mif4gd|69674	1.099748217	0.251831934	1.002566823	0.491499777
Rrp15|67223	1.099741817	0.290817921	-1.087712155	0.264919104
Ndrg1|17988	1.09966836	0.309974328	1.1928478	0.195447882
Gpr30|76854	1.099662837	0.436994614	1.099662837	0.436994614
Mtdh|67154	1.09962478	0.36018058	1.09962478	0.36018058
Ndufb5|66046	1.099498517	0.221664002	-1.045441822	0.300370355
Tmem69|230657	1.099456461	0.195475365	1.019666599	0.406341625
Odc1|18263	1.099259225	0.183126375	1.099259225	0.183126375
Kbtbd7|211255	1.099111359	0.351499176	1.333578223	0.10174052
Ogfr|72075	1.099043727	0.250306956	1.150951601	0.163779882
Dip2c|208440	1.098969187	0.356105883	1.098969187	0.356105883
Pdgfrl|68797	1.098964022	0.434375662	1.162218389	0.407080954
Bag3|29810	1.098686929	0.254855416	1.038907711	0.392479289
D4Bwg0951e|52829	1.098649549	0.121120235	1.005221335	0.460829289
Rab9|56382	1.09853215	0.16328893	1.09853215	0.16328893
Selk|80795	1.09845338	0.217527483	-1.032632913	0.359510377
Ahsa1|217737	1.098444677	0.137184245	1.09608058	0.164717935
Rpl34-ps1|619547	1.098442499	0.428043681	-1.339851816	0.160150656
Stat5b|20851	1.098391503	0.267269319	1.255299663	0.023815405
Psmb6|19175	1.098306983	0.22561424	1.038500292	0.379614887
Cyb561d2|56368	1.098282814	0.183984418	-1.017648284	0.408067406
Keg1|64697	1.098269554	0.287278911	1.098269554	0.287278911
Idh2|269951	1.098198704	0.133156997	1.009831228	0.412668566
Slc35a2|22232	1.098162305	0.201199679	-1.04182226	0.256887945
Srpx2|68792	1.098147993	0.386944891	1.169916978	0.248348775
Poc1a|70235	1.098090041	0.384273805	1.338528924	0.174277393
Ndufa3|66091	1.098072102	0.276611335	1.014717837	0.462192565
Lyar|17089	1.097912233	0.288548839	1.202704789	0.101736007
Cyba|13057	1.097827575	0.325436758	-1.071915322	0.325307737
Med29|67224	1.097811367	0.296922359	1.157527537	0.208140377
Glt25d1|234407	1.097801579	0.257119919	1.097801579	0.257119919
Zc3h15|69082	1.097687649	0.309909161	1.091031779	0.253393438

Klhl21|242785	1.097639016	0.31839421	1.287234306	0.068942412
Zdhhc12|66220	1.097617392	0.196853045	1.06880236	0.16337232
S100a10|20194	1.097562521	0.303456578	1.097562521	0.303456578
Sat1|20229	1.097545188	0.216853549	1.07983925	0.151149922
Rpl28|19943	1.097463906	0.347517863	-1.106302754	0.281117063
Hltf|20585	1.097429091	0.349552428	1.12050628	0.228296663
Hnrnpl|15388	1.097268432	0.211221084	-1.038190085	0.304500587
Fam160a1|229488	1.097231473	0.343569824	1.17198671	0.267361261
Irf2bp2|270110	1.09690605	0.221946359	1.168983812	0.076438275
Foxp1|108655	1.096902468	0.28977675	1.096902468	0.28977675
Rpl26|19941	1.096847187	0.296701674	1.029052211	0.437358277
Runx1t1|12395	1.096807203	0.452865627	-2.497414141	0.097650894
Trim33|94093	1.096772475	0.411921141	1.586979468	0.118518349
Prpsap1|67763	1.09669427	0.179476635	1.149240001	0.04320726
Ngly1|59007	1.096493888	0.354408888	-1.02006055	0.465179321
Smo|319757	1.096322757	0.277703331	-1.004372737	0.48612605
Calr3|73316	1.096182829	0.382139373	-1.197886736	0.201896338
Nmb|68039	1.095997167	0.45353772	1.195314368	0.428478238
Rab7l1|226422	1.095925482	0.267738784	1.224088392	0.0615747
Taf1c|21341	1.09590417	0.348580503	1.09590417	0.348580503
Pck1|18534	1.095871157	0.154745267	1.029428474	0.333965255
Ube2v2|70620	1.095806412	0.368439016	1.23012265	0.126649667
Mbd2|17191	1.095522428	0.270472559	1.20368302	0.099989684
Atg4a|666468	1.095431575	0.395412342	1.095431575	0.395412342
Gss|14854	1.095399591	0.254379992	1.022531042	0.429598782
Gpatch3|242691	1.09538786	0.33959274	1.09538786	0.33959274
Kcmf1|74287	1.095292084	0.171038006	1.033932038	0.342469783
Clpp|53895	1.095245458	0.261988598	1.151025718	0.165626989
Cul3|26554	1.095239407	0.291061521	1.19904249	0.140460435
Synj1|104015	1.095220172	0.349138125	-1.01683975	0.459077209
Mta2|23942	1.095212225	0.147742448	1.050313975	0.275945749
Exosc4|109075	1.095151005	0.210353183	1.026242361	0.393830438
Hint2|68917	1.095148608	0.302271916	-1.03363461	0.408800587
Grm8|14823	1.095055814	0.465706597	1	#DIV/0!
Hist3h2ba|78303	1.095055814	0.465706597	1	#DIV/0!
Dcn|13179	1.095004333	0.314227215	1.125095677	0.188459893
Ncoa3|17979	1.094910829	0.307368128	-1.022401996	0.445455754
Atxn3|110616	1.0949006	0.311691716	-1.001780194	0.49611231
Pigm|67556	1.094864622	0.390685979	1.289684368	0.236098186
1810022K09Rik|69126	1.094793135	0.376976602	1.311598355	0.095080912
Zbtb24|268294	1.09478433	0.248333718	1.177887818	0.111685232
Mrpl9|78523	1.094772342	0.129683132	1.062020555	0.228460554
Ybey|216119	1.094761038	0.328235204	1.194342346	0.208204152
Slc39a9|328133	1.094712159	0.320824718	1.410275386	0.001788397
Bop1|12181	1.094576476	0.230986758	1.094576476	0.230986758
Fcgr2b|14130	1.094535836	0.324657802	1.022808319	0.454810755
Nat2|17961	1.094496255	0.350811059	1.230081055	0.203391653
Adamtsl2|77794	1.094461526	0.325905706	1.26015174	0.109648391
Pcna|18538	1.094411825	0.192080869	1.238843314	0.001174608
Pfkfb1|18639	1.094170092	0.335743927	1.22730503	0.128036507
Sycp3|20962	1.094068721	0.367885571	1.307764588	0.064719865
Btg1|12226	1.094007317	0.263083665	1.174547111	0.107449447
Yipf2|74766	1.093905776	0.318477089	-1.027222525	0.438588121

Nceh1|320024	1.093758454	0.370198281	1.18724982	0.284306191
Cbfb|12400	1.093678919	0.370990009	1.482770955	0.035554301
Bag5|70369	1.093456856	0.279283006	1.138106744	0.105369104
Uqcrb|67530	1.093452193	0.253178446	1.025073031	0.421089576
Gemin7|69731	1.093392287	0.284294304	-1.023612573	0.425132522
Cep63|28135	1.093300822	0.269499015	-1.00891431	0.470572641
Gnb1l|13972	1.093269048	0.317683584	-1.015206686	0.462629997
E2f1|13555	1.093256312	0.308247422	1.251617925	0.078314821
Prhoxnb|231903	1.093244698	0.339331913	1.403246991	0.012677972
Rpia|19895	1.093141028	0.376574801	1.093141028	0.376574801
Ddx42|72047	1.093128347	0.263673661	1.087348434	0.141603135
Tspan12|269831	1.093093278	0.320769091	1.117573978	0.253526188
Chmp4b|75608	1.093069229	0.140499065	1.008161367	0.445781133
Rpl7l1|66229	1.093037296	0.190675431	-1.025964142	0.352798147
Gm6900|628596	1.09302935	0.289516648	1.319034153	0.013944165
Smyd1|12180	1.092956764	0.348767726	-1.056415523	0.397502117
Zbtb40|230848	1.092867149	0.351550544	1.211350948	0.222166299
Tspan3|56434	1.092782742	0.333959528	1.092782742	0.333959528
Kynu|70789	1.092759295	0.243544565	1.163766057	0.02394275
Nmd3|97112	1.092704578	0.327279249	-1.169729457	0.081133644
Plin3|66905	1.092689927	0.178863929	1.092689927	0.178863929
Abcb7|11306	1.092563821	0.346424794	1.310531343	0.083086586
Ube2f|67921	1.092500248	0.209126638	1.013987153	0.436078895
Nudt15|214254	1.092384738	0.444308127	1.799787385	0.205334449
Gas5|14455	1.092370894	0.302998371	1.132966742	0.153965801
Ythdc2|240255	1.092301287	0.391662196	1.581170321	0.027592626
Cdk2ap1|13445	1.092275735	0.194230503	1.163341032	0.062706872
Krt10|16661	1.092220426	0.399241219	1.45260338	0.12408355
Usf1|22278	1.092207626	0.323323429	-1.05040351	0.378187146
Mxd3|17121	1.092196453	0.419432893	1.092196453	0.419432893
Capza1|12340	1.092194534	0.301040867	-1.016460804	0.45722112
Kif24|109242	1.09217231	0.334577555	-1.044324615	0.408087654
Polr3gl|69870	1.092134011	0.215600773	1.165773157	0.082361784
Vps37b|330192	1.092131663	0.192744084	1.092131663	0.192744084
Blcap|53619	1.092102584	0.255941621	1.092102584	0.255941621
Prmt1|15469	1.092050349	0.263531596	-1.004190525	0.484251024
Irgq|210146	1.092031253	0.322645539	1.163015354	0.133600894
Ddb1|13194	1.091953949	0.195567079	1.036792034	0.354674931
Ppargc1b|170826	1.091858916	0.397147493	-1.094904744	0.397484019
Adra2b|11552	1.091836215	0.243125228	1.024962849	0.41512615
Tpm1|22003	1.091802691	0.239676029	1.064836261	0.174222602
Tlk2|24086	1.091744525	0.30195655	1.091744525	0.30195655
Slc12a3|20497	1.091667256	0.465581866	-2.76078	0.173296754
Alg9|102580	1.091584977	0.273141434	1.155982611	0.156058519
Fam3c|27999	1.09148403	0.195725452	1.09148403	0.195725452
2310030N02Rik|76947	1.091474977	0.351748381	-1.014866464	0.472540488
Prkar1a|19084	1.091351278	0.096150869	1.10407485	0.02946061
Srgap2|14270	1.091061109	0.332076103	1.183610772	0.042014407
Tns1|21961	1.090864537	0.328369796	1.077425487	0.324624723
Phyh|16922	1.090863153	0.316494013	1.090863153	0.316494013
Mmrn2|105450	1.0907973	0.296925061	1.0907973	0.296925061
Phf17|269424	1.090759689	0.360203153	1.457026596	0.020905107
Atp6v0c|11984	1.090594174	0.077943265	1.029317314	0.268754034

Hoxb4|15412	1.090431888	0.355456695	1.218927361	0.151802023
Nras|18176	1.090328927	0.217790158	1.069121679	0.290437407
Ctsz|64138	1.090308772	0.324966037	-1.027375703	0.439375979
Ncbp1|433702	1.090249821	0.306648343	-1.026298038	0.420550798
1700012B15Rik|74173	1.090152123	0.357460475	1.221623761	0.212833569
Nrf1|18181	1.090082815	0.208845654	1.119768223	0.098576514
Tmed1|17083	1.090043833	0.327054351	-1.022827062	0.45098957
Mrpl55|67212	1.089692534	0.322493165	1.225564136	0.132835163
Klhl36|234796	1.089688728	0.378098017	1.204890511	0.270860903
Wdr83|67836	1.089677819	0.253631909	-1.060541212	0.254263001
Ddr2|18214	1.08961365	0.429089839	1.630074019	0.158168861
Mcm3|17215	1.089581305	0.368946301	1.004879473	0.492461106
Cblc|80794	1.089524255	0.365009999	1.135691867	0.257372146
Osbp|76303	1.089499134	0.324505178	1.132007604	0.219152203
Rnf113a2|66381	1.089442827	0.283376739	-1.006015037	0.482072889
Gramd4|223752	1.089402417	0.392707335	1.179769075	0.273507839
Adcy9|11515	1.089358926	0.386152354	1.308617865	0.18798668
Il21r|60504	1.089345025	0.425367039	1.444684304	0.229385645
Btrc|12234	1.08932917	0.107356971	1.169704611	0.006335788
Kctd10|330171	1.089286801	0.359402413	-1.046791017	0.422289807
Ckap5|75786	1.089238517	0.338630811	1.003212454	0.493546911
Banf1|23825	1.08918415	0.204643201	1.135329011	0.109641888
Tinf2|28113	1.089100306	0.301263921	-1.004497421	0.488509826
Pkmyt1|268930	1.089054974	0.365231051	1.458088699	0.001787659
Lrrc58|320184	1.08902468	0.342765035	1.15682691	0.163887159
Tsga14|83922	1.089021044	0.421494393	1.046616044	0.457406848
Fam160b2|239170	1.088991948	0.202199688	1.094816415	0.079255318
Inpp5a|212111	1.088988846	0.24376681	1.16815238	0.101338891
Tmem100|67888	1.088965036	0.44805352	-1.659929646	0.223458614
Slc27a2|26458	1.088930672	0.318026979	1.088930672	0.318026979
AA543186|100272219	1.088846188	0.438993673	-1.305757248	0.32891176
Mlxip|208104	1.088836827	0.345625853	-1.020757357	0.430487587
Mrps18b|66973	1.088756968	0.221142031	1.130828588	0.139349485
Ifnar1|15975	1.088723311	0.13102851	1.117679583	0.08635332
Ing2|69260	1.08861323	0.284785894	1.012927891	0.462801905
Zdhhc21|68268	1.088612555	0.394460412	1.379758158	0.147306926
H2-T23|15040	1.088606831	0.11206042	1.053421919	0.215555661
Entpd7|93685	1.088562239	0.398282965	1.269579058	0.253504379
Cyld|74256	1.088535658	0.31599356	1.081886449	0.314690046
Rhod|11854	1.088506389	0.349515882	-1.100594752	0.271472446
Tfpi|21788	1.088489503	0.427415995	1.088489503	0.427415995
Tnks2|74493	1.088382009	0.278063369	1.183123161	0.083093383
3110043O21Rik|73205	1.088325633	0.348784137	-1.069840691	0.359163744
Cdc42ep4|56699	1.088282912	0.190778983	1.143457455	0.067680361
Foxa3|15377	1.088237091	0.146902994	1.0361787	0.300157856
Wsb2|59043	1.088199223	0.244824513	1.022994392	0.419027123
0610012G03Rik|106264	1.088185557	0.220253272	1.100983238	0.111268008
Cd207|246278	1.088067147	0.406653871	1.213186314	0.275999454
Srcrb4d|109267	1.088020375	0.420701448	-1.329379391	0.21094128
N4bp3|212706	1.08801525	0.39447829	-1.195011459	0.237959169
BC024659|108934	1.087917344	0.325763177	1.087917344	0.325763177
Capzb|12345	1.08787271	0.288289968	-1.001967154	0.494393898
Myl6|17904	1.087779611	0.306376304	-1.02903158	0.419201553

Selm|114679	1.087751551	0.414790913	-1.267420178	0.242194199
Pan2|103135	1.087742095	0.273068779	1.18488642	0.053824829
Fermt1|241639	1.087740168	0.377860053	1.213706717	0.224082538
3110003A17Rik|73112	1.08771878	0.225757772	1.170634754	0.066268703
Zwint|52696	1.087676627	0.218276997	1.227244513	0.00788407
Aldh16a1|69748	1.087665599	0.244483114	1.087665599	0.244483114
Zfp110|65020	1.087596924	0.29670025	1.087596924	0.29670025
Cxcl13|55985	1.087589551	0.4218921	-1.194198578	0.343543363
Gm2002|100038993	1.087550437	0.449613211	1.899849747	0.087408186
Anp32a|11737	1.087542917	0.251211884	1.087542917	0.251211884
Suz12|52615	1.087527007	0.348128338	1.070208491	0.316495787
Fxyd1|56188	1.08751198	0.375295144	1.060021079	0.365348006
Slc25a39|68066	1.087439679	0.138122008	1.06069043	0.229787144
Fam160a2|74349	1.087416084	0.266290302	1.27105821	0.012066784
Ttc7b|104718	1.087396907	0.298618742	1.013841221	0.465900838
Snx27|76742	1.087321874	0.330163774	1.087321874	0.330163774
Atrip|235610	1.087298744	0.32511655	1.171300699	0.212923415
Irak2|108960	1.087165638	0.214343717	1.019631853	0.41596332
Tmem30a|69981	1.0871216	0.319286813	1.114436818	0.139999244
Kcnk3|16527	1.087103107	0.417280107	1.376563963	0.229468232
Tap2|21355	1.087050966	0.196499732	1.087050966	0.196499732
Papln|170721	1.087002673	0.425945843	-1.056558218	0.452059344
Cwf19l2|244672	1.08697524	0.427449613	1.393805995	0.144944726
Il11ra2|16158	1.086859353	0.450218696	1.907399471	0.088433925
Traip|22036	1.086835718	0.454656906	-1.823402228	0.222971531
Psmb2|26445	1.086811087	0.281963062	1.154549443	0.152870768
Ctsd|13033	1.08667127	0.272644435	1.007008677	0.478284738
Polr2e|66420	1.086658486	0.307695769	1.013558807	0.468375825
Pes1|64934	1.086284969	0.250446638	1.086284969	0.250446638
Mrps23|64656	1.086259448	0.255142135	1.086259448	0.255142135
Tspan14|52588	1.086189097	0.203473069	1.155909119	0.039261769
Rab11b|19326	1.086174502	0.071228718	1.118874841	0.017130541
Gsg2|14841	1.086094977	0.458740931	3.157199253	0.095887618
Notum|77583	1.086084272	0.144193482	1.133862899	0.037374297
St3gal5|20454	1.086062938	0.199441412	1.142989079	0.068787804
Lhx6|16874	1.08605613	0.358705718	1.08605613	0.358705718
Ccl3|20302	1.086052424	0.453058154	1.086052424	0.453058154
Gm10941|100169878	1.086052424	0.453058154	1.086052424	0.453058154
2010107H07Rik|66487	1.085994761	0.359095345	-1.006513589	0.489306191
Calr|12317	1.085974964	0.344761552	1.168234706	0.224391832
Fosl2|14284	1.085954689	0.345694509	-1.023317827	0.451081186
Mbd1|17190	1.08589517	0.327248393	1.08589517	0.327248393
Tmem102|380705	1.08585406	0.266551761	1.08585406	0.266551761
Slc27a1|26457	1.085795934	0.272659386	1.011155929	0.465981528
Tmed2|56334	1.085730134	0.104897799	1.036517873	0.219835051
Tada3|101206	1.085723638	0.309110671	-1.014759494	0.461728763
Tmem181a|77106	1.085682428	0.435321923	-1.174056028	0.311131545
Pde8b|218461	1.085654528	0.450540352	1.723251843	0.240544606
Ndufa4|17992	1.085651223	0.310088146	1.006482231	0.484589132
Cd44|12505	1.085646073	0.363669017	1.183582807	0.158371288
Pigu|228812	1.085635707	0.270478436	1.012444533	0.461681864
Tgoln1|22134	1.085531922	0.197789536	1.14532469	0.079176596
Tpd52|21985	1.085529851	0.318283059	1.204624358	0.139890756

Ptp4a3|19245	1.085520925	0.238527633	1.192395865	0.029426637
Mpg|268395	1.085511097	0.254992111	1.076578682	0.18950189
Ppp1r3b|244416	1.085398176	0.347278004	1.094576619	0.251030605
Qpct|70536	1.08534743	0.363457003	1.289135554	0.122936663
Zc3h7a|106205	1.085345566	0.375526169	1.085345566	0.375526169
Wsb1|78889	1.085230277	0.406001244	-1.200579048	0.273208862
Rbm4|19653	1.085184794	0.248265017	-1.004482069	0.480638904
Mir142|387160	1.085050047	0.445324408	1.085050047	0.445324408
Atxn10|54138	1.085045678	0.222419446	1.046191092	0.236635886
Pmm1|29858	1.084998216	0.314870878	1.095011281	0.150703152
Hspa9|15526	1.084923675	0.200321554	1.034969455	0.35085359
Vrk3|101568	1.08485355	0.131685967	1.05188388	0.235664943
9130023H24Rik|100043133	1.084767529	0.377566001	1.037724398	0.434758035
Csnk1d|104318	1.084754463	0.241725671	1.179691532	0.054781414
Samd4|74480	1.084739529	0.399972499	1.078573781	0.385758633
Surf2|20931	1.084722231	0.277617136	1.093263201	0.160072283
Ppp1r3g|76487	1.084670708	0.452651245	2.702090216	0.056083401
4930471M23Rik|74919	1.084489324	0.209867592	1.216460083	0.004581126
Tmem55a|72519	1.084486822	0.42382563	1.017674719	0.480732338
Gngt2|14710	1.084475181	0.39463207	-1.064565628	0.390917777
Gnb1|14688	1.084474879	0.149308179	-1.005900891	0.453161967
Ppfia4|68507	1.084459095	0.414566471	1.698385249	0.046333718
Trmt2a|15547	1.084398484	0.311053032	1.084398484	0.311053032
Ngdn|68966	1.08437247	0.28496557	1.005988434	0.482541992
Adpgk|72141	1.084310036	0.265381248	1.010866184	0.464436803
Timp2|21858	1.084270906	0.26251665	1.177624122	0.049774041
4930420K17Rik|652925	1.08423125	0.334989081	-1.048063772	0.387656015
Wisp1|22402	1.084225513	0.374281698	-1.009969652	0.472508444
Ptpmt1|66461	1.084197259	0.237832515	1.084197259	0.237832515
Npepps|19155	1.08408593	0.177640496	1.142970378	0.056371584
Cct2|12461	1.08406535	0.241771064	-1.041189466	0.319297217
A1bg|117586	1.083954317	0.438087623	1.052660912	0.455601444
Exoc6b|75914	1.083938007	0.38416721	-1.231381619	0.170111998
Pde4a|18577	1.083934419	0.402530841	1.08663238	0.387351778
Leprel2|14789	1.083906069	0.370686617	-1.073685355	0.378509196
Psma5|26442	1.083818748	0.253229805	1.083818748	0.253229805
Sgpp1|81535	1.083721434	0.233803306	1.172280207	0.054813247
Slx1b|75764	1.083663638	0.360668237	-1.112280597	0.256147644
Rab6a|19346	1.083614791	0.145186933	1.033526124	0.295629995
Tmco6|71983	1.083493557	0.33564474	1.094866639	0.251148579
Evl|14026	1.083423248	0.368642543	1.17975158	0.264148137
Fam103a1|67148	1.08342132	0.271860838	1.179821162	0.050816024
2810432D09Rik|69961	1.083333523	0.343363097	-1.020021886	0.460082287
Nap1l4|17955	1.083298478	0.179459987	1.022283467	0.375278988
Zfp266|77519	1.083228442	0.347272053	1.216532498	0.171594885
Sdcbp|53378	1.083176945	0.308834546	1.195996425	0.125807373
Atp2a2|11938	1.083095934	0.224848899	1.16862045	0.04821131
Kctd9|105440	1.083095438	0.382275291	1.197735644	0.241461546
Cdkl2|53886	1.083077333	0.425307361	1.517040546	0.162582138
Mmp14|17387	1.082777658	0.2079883	-1.03811656	0.172791667
Zfp143|20841	1.082683399	0.417146364	1.289258227	0.09376157
Ube2e3|22193	1.082581855	0.351303117	1.161381654	0.23641785
Rcc2|108911	1.082577553	0.198446305	1.095992003	0.059341719

Spire1|68166	1.082429567	0.447505891	1.489598533	0.284410012
Srsf6|67996	1.082416638	0.305194903	1.317288776	0.004109346
Bend3|331623	1.082353964	0.413988119	1.374312253	0.200079127
Cdhr5|72040	1.082339859	0.336278383	-1.05777321	0.356830266
Emilin2|246707	1.082331257	0.402020139	-1.285390659	0.183850289
Wbscr16|94254	1.082318617	0.207931933	1.082318617	0.207931933
Lpxn|107321	1.082201952	0.439612797	-1.101525314	0.422065555
1810014F10Rik|69064	1.082130632	0.375274903	-1.010453591	0.472171512
Lsm7|66094	1.082031101	0.321452155	1.096413136	0.236142392
Clk2|12748	1.081986784	0.374762805	1.318037196	0.104062637
2700089E24Rik|381820	1.08198324	0.194715279	1.207575562	0.00049618
Aoah|27052	1.081879604	0.380756744	1.375486132	0.03974296
1190005I06Rik|68918	1.081878878	0.40777115	-1.181571594	0.212233997
Rp9|55934	1.081855922	0.352222056	-1.039126579	0.423844511
Chchd4|72170	1.081848011	0.210229102	1.048815418	0.316553147
Fmnl2|71409	1.081809561	0.430638943	1.270173871	0.279896128
Lcmt1|30949	1.081795411	0.334961605	-1.025694167	0.44261247
Nptn|20320	1.081770048	0.387315917	1.050661248	0.401864866
Csf2rb|12983	1.081631575	0.377562304	1.022330389	0.458177503
Phlpp2|244650	1.081622698	0.367768338	1.43447218	0.019596858
Hsdl2|72479	1.081603924	0.276573024	1.16828882	0.116775394
Rbks|71336	1.081598082	0.331135661	1.197201625	0.116764256
Ccdc22|54638	1.081575524	0.385296572	1.076916656	0.338502628
Ficd|231630	1.081526956	0.374846562	1.396454163	0.037050858
Flt3l|14256	1.081481232	0.338591597	1.266148809	0.0628531
Gosr1|53334	1.081321437	0.348373764	1.339481242	0.042143678
Arglu1|234023	1.081287652	0.238134179	1.128606277	0.150389507
Tlr2|24088	1.081248721	0.427231261	1.081248721	0.427231261
Zfp874b|408067	1.081171331	0.390361048	-1.124593423	0.321563902
Dnajb14|70604	1.081003896	0.414144845	1.081003896	0.414144845
Slc25a46|67453	1.081002427	0.286637373	1.185677105	0.094054647
Cox5a|12858	1.080987013	0.292671304	1.059135589	0.256173936
Rpl39|67248	1.080974267	0.350800431	-1.193406731	0.062575198
Snrnp27|66618	1.080957935	0.33337295	1.080957935	0.33337295
Nup50|18141	1.080955672	0.265120201	1.01837451	0.440351917
Tmtc2|278279	1.080884295	0.345160219	1.152925267	0.074394016
Cct4|12464	1.080821938	0.293463781	1.080821938	0.293463781
Zbtb43|71834	1.080807875	0.376517069	1.188250654	0.234802805
Usp15|14479	1.080657313	0.345061565	1.08972224	0.287838963
Pex16|18633	1.080595138	0.257018469	1.041003311	0.374438122
Rhbdl3|246104	1.080586766	0.455695994	2.154086947	0.161214996
Rpn1|103963	1.080583616	0.2711452	1.142301461	0.139716485
Mns1|17427	1.080506809	0.448789265	1.080506809	0.448789265
Psma4|26441	1.080329678	0.212158544	1.028884475	0.378028074
Rbm7|67010	1.080303003	0.394997739	-1.083496636	0.392427122
Wdr3|269470	1.080261854	0.383836619	1.202829521	0.258199947
Cisd3|217149	1.079858626	0.317830877	-1.103237296	0.214585606
Pfdn2|18637	1.079783117	0.277608089	1.079783117	0.277608089
Arsg|74008	1.079659206	0.345283947	1.250889625	0.094726816
Chd4|107932	1.079635185	0.106518557	1.044094053	0.212046539
Asrgl1|66514	1.079613625	0.251067471	1.092454441	0.123033563
Tmem223|66836	1.079577236	0.355670522	-1.011428175	0.476943787
Chmp1b|67064	1.07953913	0.354417073	1.228816534	0.153085127

Stat5a|20850	1.079511593	0.334002161	1.19935139	0.153181279
Ints2|70422	1.079497959	0.454785861	-1.04057646	0.478336464
Mrps17|66258	1.079496699	0.287896692	1.251797918	0.027601354
Wbp5|22381	1.079471424	0.29646182	-1.072115314	0.267867183
Dpp3|75221	1.079413636	0.209523747	1.199687181	0.010229384
Reep4|72549	1.079410784	0.344581431	1.226795671	0.131001432
Clec1b|56760	1.079322173	0.33777992	-1.070659112	0.304860204
Yipf5|67180	1.079311358	0.319696199	1.13091405	0.138504833
Maged2|80884	1.079206123	0.37418233	1.183998368	0.219676856
Dusp10|63953	1.079201876	0.385976835	1.183086642	0.257890909
Reep5|13476	1.079189999	0.39004607	-1.158904486	0.24403699
Ces2d-ps|667754	1.079186869	0.475508772	1	#DIV/0!
Arhgef10l|72754	1.079151769	0.229557083	1.079151769	0.229557083
Mettl10|72096	1.079133816	0.455798105	-1.820480784	0.194152814
Atp5k|11958	1.079097892	0.398907418	1.017452917	0.461526873
Ints5|109077	1.079097418	0.275476601	-1.010299919	0.457251033
Adss|11566	1.079085581	0.297900557	1.053992875	0.270482456
8430419L09Rik|74525	1.079077339	0.163752046	1.032737082	0.320181029
Repin1|58887	1.0789916	0.223630608	1.043067338	0.338672934
H2afy2|404634	1.078990681	0.363661943	1.078990681	0.363661943
Psap|19156	1.078982057	0.075109179	1.120915897	0.001977687
2310003F16Rik|67693	1.07892984	0.340134695	-1.026159219	0.442103503
Cblb|208650	1.078925146	0.352329398	1.078925146	0.352329398
Gm10653|677044	1.078889156	0.330292325	-1.145673729	0.093184406
Hnrnpa0|77134	1.078885718	0.391546838	-1.059662605	0.41965308
Pign|27392	1.078873356	0.40726447	1.349526088	0.177192659
Cntln|338349	1.078855992	0.465525078	1.656626026	0.330587367
Itsn2|20403	1.078838715	0.423751379	1.845151606	0.007065639
Ube2i|22196	1.078834428	0.211412677	1.085186595	0.115404787
Hiatl1|66631	1.078737171	0.384720609	1.078737171	0.384720609
Zfp454|237758	1.078505994	0.471601984	-3.925	0.173296754
Trmt112|67674	1.078433799	0.275830614	1.139457389	0.164170586
Csrp1|13007	1.078426249	0.293636347	1.267396484	0.007077966
Zfp251|71591	1.078322658	0.418428655	-1.40270781	0.118445469
Il10rb|16155	1.078200107	0.368547829	1.241204298	0.165668796
Sf3a3|75062	1.078144048	0.293075751	1.154697293	0.129080959
Zmat3|22401	1.078123853	0.316825305	1.160344126	0.184126886
Adamts14|237360	1.078102686	0.357388849	-1.074428568	0.339345207
Gltscr2|68077	1.078044457	0.343750418	-1.04006449	0.409609262
Cabp4|73660	1.078041678	0.438923006	1.078041678	0.438923006
Dync2h1|110350	1.078038272	0.436729072	1.078038272	0.436729072
Tbc1d17|233204	1.077986698	0.215742728	1.017008298	0.418920328
Tnfaip8|106869	1.077920293	0.456191443	-1.870393594	0.174549207
Baz1b|22385	1.07791856	0.330002991	1.337842201	0.005320764
Slc25a3|18674	1.077845968	0.087240142	1.03406787	0.167986606
Tpra1|24100	1.077823079	0.324329209	1.190798038	0.139907344
Chic1|12212	1.077733774	0.321129268	1.077733774	0.321129268
Slbp|20492	1.077682923	0.402082691	1.22645061	0.230784511
Cbwd1|226043	1.077535846	0.417524446	1.345184653	0.215474656
Tnfrsf12a|27279	1.07752616	0.371306002	1.07752616	0.371306002
Alb|11657	1.077519741	0.291317498	-1.017123268	0.40057876
4933426M11Rik|217684	1.077433207	0.38502662	1.312026584	0.121762252
Tmem11|216821	1.077398615	0.166675163	1.077398615	0.166675163

Dtd1|66044	1.07735312	0.340733421	1.07735312	0.340733421
Fam162a|70186	1.07731277	0.276551543	-1.067562025	0.235721956
Bambi|68010	1.077279985	0.404865931	1.077279985	0.404865931
2310036O22Rik|68544	1.077269218	0.30332975	-1.108752923	0.105884014
Bet1|12068	1.077262553	0.344538691	1.108174468	0.243396073
Cox6b1|110323	1.077261655	0.359173132	1.068069645	0.346903726
Rpl6|19988	1.077246533	0.319656676	-1.003938184	0.490002355
Gm5431|432555	1.077222838	0.464138274	-2.542818182	0.142006587
Unc119b|106840	1.077210809	0.187961651	1.12150259	0.092025596
Id3|15903	1.077210676	0.360609504	-1.001275695	0.496498582
Rnaseh2c|68209	1.077205715	0.380443384	-1.22922713	0.124878812
Inpp4a|269180	1.077095485	0.259233229	1.226051294	0.016421595
Adk|11534	1.077084843	0.321636576	-1.034765672	0.400881108
C130022K22Rik|232236	1.077041687	0.327936111	1.129267451	0.240971088
Golga7|57437	1.076955687	0.292878123	1.258287549	0.01691387
Dtnb|13528	1.076901833	0.286096435	1.076901833	0.286096435
Ramp2|54409	1.076761469	0.330942472	1.076761469	0.330942472
Unkl|74154	1.076704922	0.367045163	1.283748479	0.091230653
Ankrd54|223690	1.076698625	0.352475507	1.337832237	0.031523653
Ccdc134|76457	1.076611681	0.368010229	1.083921128	0.301400819
Eif6|16418	1.076566601	0.286669599	1.174665295	0.093928658
Prr24|66300	1.076491942	0.353983905	1.076491942	0.353983905
Hspa1a|193740	1.076442159	0.43657184	1.095759061	0.422706344
1500003O03Rik|56398	1.076425973	0.311319748	1.161846264	0.13388833
Mrpl51|66493	1.076346602	0.320633588	1.134507344	0.215975417
Klk1b4|18048	1.0763041	0.449350488	1.305619716	0.263214304
Plp2|18824	1.076280113	0.414674847	-1.171043662	0.314061076
Rdh1|107605	1.076280104	0.459574804	1.121266245	0.449231917
Haus3|231123	1.076254903	0.440680469	-1.709418759	0.079627308
Atp6v1h|108664	1.076211613	0.372632561	1.177851539	0.252027092
Cyp2c38|13097	1.07618707	0.25079926	-1.055482724	0.221589241
Cbx8|30951	1.076166869	0.324941296	-1.048229113	0.325719443
Metap2|56307	1.076061505	0.355587906	1.214011343	0.161569102
Fnip2|329679	1.076016323	0.344854706	-1.008264827	0.481009094
0910001L09Rik|66096	1.075959605	0.389239228	-1.148679386	0.246680711
Tmed5|73130	1.075944012	0.293605148	-1.008181878	0.470768883
6430527G18Rik|238330	1.075928514	0.36510178	-1.032015324	0.434440173
Nipbl|71175	1.075899918	0.319076358	1.075899918	0.319076358
Pon2|330260	1.075884537	0.202976285	1.075884537	0.202976285
Zfp768|233890	1.075881983	0.170002497	1.173848562	0.005632875
Ip6k1|27399	1.075878705	0.12212803	1.093770098	0.091053222
Gfer|11692	1.075757281	0.29684678	-1.027892036	0.382173758
Uqcr10|66152	1.075746723	0.368194183	-1.052285532	0.40384736
Nfatc2ip|18020	1.075706994	0.392229019	1.306890655	0.145008195
1810055G02Rik|72056	1.075691347	0.394326584	-1.03854622	0.423372397
Slc25a12|78830	1.075536452	0.322915212	1.288123509	0.019379013
Vapa|30960	1.075459618	0.250797338	1.082236757	0.043429535
Laptm4a|17775	1.075450387	0.112991574	1.052277961	0.1977666
Psma7|26444	1.075418987	0.308882603	1.016628267	0.456590027
Nr1i2|18171	1.075407826	0.319276779	-1.115435386	0.138165337
Cd24a|12484	1.075392897	0.431797081	1.218269551	0.311411014
Lats1|16798	1.075365448	0.368774021	1.377231438	0.03358538
Slain2|75991	1.075234941	0.342669275	1.071575684	0.296671979

Relb|19698	1.075113042	0.383085937	-1.224928329	0.127998099
Eif1ad|69860	1.075084755	0.265911283	1.039974966	0.325917088
Arhgap19|71085	1.075033367	0.400491934	-1.003839435	0.493252077
Dynll2|68097	1.07501103	0.288253349	1.018551907	0.441765061
Ndufa1|54405	1.07491647	0.35537482	-1.048474192	0.396366999
Asb1|65247	1.074794241	0.323025995	-1.109454048	0.157641219
Smarcd2|83796	1.074710478	0.311931901	-1.020289483	0.438728682
Ddx1|104721	1.074679831	0.260592628	1.157352619	0.047019296
Atp5e|67126	1.074625581	0.398807337	-1.008130747	0.483645016
Dpm3|68563	1.074530506	0.378403206	1.058201637	0.374948926
Nomo1|211548	1.074510555	0.204353071	1.137142251	0.03341458
Bri3|55950	1.074509411	0.31266635	-1.102535119	0.167290486
Taf1|270627	1.074500014	0.373322462	1.01560491	0.474713828
Vsig10|231668	1.074457937	0.411091259	1.074457937	0.411091259
Nlrx1|270151	1.074369344	0.249474467	1.044502132	0.287060696
Cct3|12462	1.074173837	0.250024348	1.082743904	0.15405835
Gm12034|382492	1.074164494	0.363219469	1.074164494	0.363219469
Top3a|21975	1.074140479	0.367290467	1.007545074	0.480285591
Thada|240174	1.074042229	0.264405777	1.035533663	0.33871155
Hmbox1|219150	1.073912168	0.352373737	-1.032346773	0.422085096
Pim3|223775	1.073759594	0.295473947	1.073759594	0.295473947
Cecr2|330409	1.073647156	0.42610232	-1.17704945	0.337175598
Vbp1|22327	1.073593638	0.435744055	-1.113814351	0.361019963
Zfp874a|238692	1.073523936	0.39566485	1.068178108	0.389186211
Dusp23|68440	1.073522842	0.372904839	1.073522842	0.372904839
Ndfip1|65113	1.073420394	0.303961916	1.014492668	0.459542108
Ttc39c|72747	1.073335508	0.250611124	1.082197512	0.12085714
Dlgap4|228836	1.073271495	0.325899251	-1.059915449	0.29038307
Mpv17l|93734	1.073264894	0.254984686	1.005616611	0.476321528
A630066F11Rik|320642	1.073188549	0.454012781	1.186385662	0.388215906
Pbrm1|66923	1.073106343	0.391659985	1.27544016	0.16600237
Cox6a1|12861	1.07300453	0.355760637	-1.147218024	0.173901958
Wwp2|66894	1.072990361	0.27315042	1.101244847	0.22701014
Nfu1|56748	1.072934271	0.173638054	1.043242039	0.129985445
Cox7c|12867	1.072883333	0.282432086	1.017084334	0.445177814
Pard6a|56513	1.072805346	0.397481964	1.235839211	0.228932075
Ap1b1|11764	1.07278517	0.190081897	1.106215641	0.117748172
Bhmt|12116	1.072646553	0.412857783	1.053291853	0.423912674
Ndufc2|68197	1.072645293	0.191500142	1.072645293	0.191500142
Git2|26431	1.072596568	0.22903132	1.072596568	0.22903132
Prdx5|54683	1.072581395	0.161400372	1.000601619	0.495449939
Mavs|228607	1.072501247	0.257409292	1.072501247	0.257409292
Iqck|434232	1.072370472	0.469052113	3.133345238	0.159901737
Cog8|97484	1.072368797	0.326564401	-1.00145676	0.49629199
Ywhab|54401	1.072366785	0.138661793	1.049509414	0.079218733
Fpr2|14289	1.072349951	0.462126442	-1.052954382	0.472033354
Msl1|74026	1.07231195	0.241835127	1.123664061	0.110662472
Prtg|235472	1.072299933	0.465352052	1.255130842	0.411829119
Phldb1|102693	1.072236466	0.294598592	1.134411809	0.176445413
C030034I22Rik|77533	1.072217916	0.443561798	1.467459434	0.238551673
2400003C14Rik|71955	1.072194358	0.338952107	1.300481328	0.019737943
Dnase1l1|69537	1.072140657	0.417117348	1.507573837	0.069599033
Ttc27|74196	1.072107517	0.376330389	-1.012829012	0.476509671

1200011M11Rik|74133	1.072096056	0.400885798	1.072096056	0.400885798
Tulp3|22158	1.072087433	0.418613003	-1.197394785	0.280029512
Ccrl1|252837	1.072074399	0.388030421	1.072074399	0.388030421
Pld3|18807	1.071961675	0.29428336	-1.031300007	0.366799283
Trmt6|66926	1.071897045	0.365901459	-1.045871648	0.396284018
Polr3f|70408	1.071803333	0.411668651	-1.170104951	0.287301646
Ppp1r14b|18938	1.071770937	0.327808891	-1.033488571	0.390288144
Unc119|22248	1.071753335	0.360552304	-1.040693039	0.415460291
Fnta|14272	1.071742493	0.268199551	1.079520122	0.156494912
Uvrag|78610	1.071724666	0.352337402	-1.159257617	0.111149574
Nup85|445007	1.071632414	0.334717961	1.150343341	0.182575324
Kcnn2|140492	1.071595298	0.351995927	1.090837859	0.2269716
Nucks1|98415	1.071593439	0.266986019	1.208101713	0.024893192
Crlf2|57914	1.071510551	0.404116461	1.215247129	0.263910778
Prmt3|71974	1.071503588	0.354599063	1.323687082	0.019797953
Dvl2|13543	1.071462688	0.273558109	1.205897188	0.033026109
Mest|17294	1.071424571	0.403632338	-1.071309181	0.391616968
Qser1|99003	1.071314321	0.327715512	1.231836451	0.029067673
Rfxap|170767	1.071263213	0.321773119	1.038605695	0.383558439
Rab24|19336	1.071259303	0.325029217	1.001843538	0.494901893
Ddx5|13207	1.071210077	0.29426807	1.158652805	0.114206209
Gpx1|14775	1.071184106	0.407577806	-1.03625752	0.411537361
Pfkm|18642	1.071016185	0.284263997	1.015008564	0.448252078
Plekhg3|263406	1.070878281	0.309393507	1.070878281	0.309393507
Syncrip|56403	1.070864285	0.267088779	1.070864285	0.267088779
Tep1|21745	1.070812839	0.372267078	1.276762686	0.079323297
Rpl10|110954	1.070785032	0.37996076	-1.220769596	0.08999936
Fam13b|225358	1.070758151	0.40079841	-1.108693708	0.345558774
Fem1a|14154	1.070722567	0.283780636	1.110395739	0.104607817
Cercam|99151	1.070617504	0.433378911	-1.568173682	0.04804627
Atp5h|71679	1.070336556	0.334838573	-1.024253904	0.436158679
Slc1a2|20511	1.070310126	0.312504741	1.231572348	0.017977479
Anks3|72615	1.069996408	0.349442319	1.201979566	0.13325854
Paf1|54624	1.069962197	0.356799309	1.069962197	0.356799309
Pde1a|18573	1.069949569	0.367551466	1.142101223	0.17620642
Snrpd1|20641	1.069942528	0.358973564	1.297735258	0.054074977
Polr1d|20018	1.069764695	0.322690283	-1.009049774	0.474673462
Dlgap1|224997	1.069596606	0.449020026	1.293675631	0.313422222
1500010J02Rik|68964	1.06954805	0.367236017	-1.168707916	0.146035975
Terf1|21749	1.069491126	0.350906836	1.069491126	0.350906836
Metrn|70083	1.069443278	0.431329155	-1.226545361	0.250316678
Psmb3|26446	1.069398564	0.325968484	1.269258731	0.021289661
Sun2|223697	1.069316765	0.251447012	1.129976556	0.085855417
Pqlc2|212555	1.069294876	0.293282885	1.128338699	0.175152118
Tcf7l1|21415	1.069276421	0.200609912	1.116662111	0.064753203
Scrn3|74616	1.069220787	0.326739958	1.180742164	0.116932854
Nr2f1|13865	1.069209405	0.450242292	1.069209405	0.450242292
Tyw3|209584	1.069124404	0.42641708	-1.500880644	0.023347513
Elovl3|12686	1.069096597	0.390983879	1.380466307	0.017876168
Athl1|212974	1.069007682	0.279258891	1.217134334	0.016928835
1600021P15Rik|239796	1.068973047	0.304925885	1.068973047	0.304925885
Retn|57264	1.068923993	0.479640709	1	#DIV/0!
Dhx8|217207	1.06888427	0.2729544	1.130747005	0.136276334

Trafd1|231712	1.068777804	0.101955861	1.046464026	0.186981684
Clec10a|17312	1.068695162	0.454103605	1.702630489	0.197137012
Axin2|12006	1.068647342	0.437674088	-1.090462378	0.388573598
Fat4|329628	1.068601664	0.455768934	1.629101651	0.234637347
Ccng1|12450	1.068567198	0.366157544	1.06105261	0.226331012
Dnajc7|56354	1.068542336	0.3024558	1.068542336	0.3024558
Acp5|11433	1.068519171	0.354951114	-1.064269767	0.335234121
Sf3b5|66125	1.068512488	0.278118319	-1.001388811	0.49448098
Sds|231691	1.068496117	0.308185578	1.068496117	0.308185578
Cep110|26920	1.068427355	0.386074602	1.091652726	0.276810762
Prdx1|18477	1.068358867	0.293148014	1.006110119	0.479473404
Ppp1r10|52040	1.06830505	0.414416416	1.218964279	0.276630546
Eef1d|66656	1.068206256	0.38984667	-1.092226262	0.342080875
1110005A03Rik|74319	1.068170569	0.401624284	1.068170569	0.401624284
Zfyve1|217695	1.067988949	0.271551072	1.141263817	0.100898626
Tnfsf12|21944	1.067953283	0.304302032	1.23195814	0.014716657
U2af2|22185	1.067929967	0.161975981	-1.010832525	0.398138698
4833422C13Rik|373852	1.067924953	0.450940537	1.067924953	0.450940537
Atg16l1|77040	1.067762742	0.301564514	1.165549449	0.04670278
Rnasek|52898	1.067724892	0.389012776	1.118211505	0.278309334
Cox7b|66142	1.06772174	0.288556542	-1.069869249	0.207226186
Dffa|13347	1.067674063	0.325678593	1.044126263	0.328885707
Zfp462|242466	1.067670981	0.468077156	-3.088025253	0.059099193
Hoxb3|15410	1.067652568	0.433534054	1.657108054	0.063626753
Stradb|227154	1.06761617	0.241001055	1.06761617	0.241001055
Hist1h4i|319158	1.067578359	0.445357257	1.067578359	0.445357257
Ndufc1|66377	1.067576247	0.316394455	1.054920426	0.311369071
Nlgn2|216856	1.067575131	0.337700573	1.066711763	0.289823703
ORF19|68767	1.067561377	0.295435379	1.067561377	0.295435379
Armc6|76813	1.067496932	0.390446549	1.198407267	0.192566292
Smg7|226517	1.067450674	0.319507303	1.188665904	0.004538998
Pick1|18693	1.067411064	0.312848775	1.007692271	0.476210302
Acaa1a|113868	1.067396806	0.330643955	1.192527906	0.08809275
Mif|17319	1.067352342	0.341265428	1.033983598	0.401110804
Pdcd6|18570	1.067313138	0.252460104	1.010080518	0.452884778
Ankmy2|217473	1.067257094	0.356717828	-1.050881107	0.376772163
Arpc5|67771	1.067256034	0.305489131	-1.000236795	0.499236737
Tmem126a|66271	1.067122051	0.341784222	-1.022788254	0.435282031
Crls1|66586	1.067106293	0.289196861	1.032296671	0.396634997
Sdhb|67680	1.066967488	0.306155044	-1.078664478	0.212633125
Ppap2c|50784	1.066957399	0.188395341	1.013651147	0.403316056
Gp1bb|14724	1.066948925	0.47340076	1	#DIV/0!
Accn2|11419	1.066872736	0.322250453	1.14476459	0.146060978
Dcaf17|75763	1.066870492	0.398066891	1.294769433	0.127075398
Ccdc120|54648	1.066774137	0.457188577	-1.166136669	0.383663267
Cmpk1|66588	1.066771447	0.329229833	1.066771447	0.329229833
Tmem59|56374	1.066749685	0.099229959	1.035983698	0.202135686
Rock2|19878	1.066725026	0.40194844	-1.276154027	0.091243995
Ifi35|70110	1.066679089	0.373879242	1.142512617	0.253863697
2310046O06Rik|78323	1.066674239	0.349040039	-1.076816673	0.254875839
Clip3|76686	1.066656947	0.448754466	1.505525964	0.225818971
Ndufa11|69875	1.066596686	0.370562332	1.219800452	0.138960825
Med30|69790	1.066485981	0.427064246	-1.180938971	0.31352457

Actl6a|56456	1.066390357	0.344521254	1.177547285	0.100974622
Rai12|54351	1.066304861	0.319483727	1.125879	0.190178917
Rnf6|74132	1.066270865	0.392253745	1.406663044	0.032136233
Hint3|66847	1.066243551	0.369760561	-1.161889435	0.154122528
Tmem41b|233724	1.066149966	0.311211826	1.000644007	0.497860073
Fcrls|80891	1.066057669	0.479449757	1	#DIV/0!
Hpca|15444	1.065986332	0.469828932	1.050723353	0.475649888
Lin7c|22343	1.06597756	0.405609623	1.466316549	0.033957232
Fbxo3|57443	1.065948753	0.312110822	1.065948753	0.312110822
Ndufb11|104130	1.065838811	0.357656454	-1.056065545	0.359156333
BC056474|414077	1.065718257	0.353370662	1.069785827	0.313336932
Acbd3|170760	1.065661052	0.431372816	1.539346854	0.067540821
Dbil5|13168	1.065632789	0.465486568	-1.762234392	0.245786637
Zfyve20|78287	1.065617898	0.397944258	1.194802343	0.248526173
Pkp2|67451	1.065541946	0.361613481	1.150513598	0.11352343
Aasdhppt|67618	1.065526441	0.353960268	-1.148165598	0.100463928
Mrps25|64658	1.065492083	0.310685832	-1.083917898	0.139647901
Morf4l2|56397	1.065485162	0.342874924	1.065485162	0.342874924
Mllt6|246198	1.06545758	0.35860115	1.215875327	0.101642506
Skap2|54353	1.065444759	0.30333961	1.065444759	0.30333961
Nfia|18027	1.065435081	0.33692024	1.065435081	0.33692024
Tpmt|22017	1.065434004	0.202949106	1.122786787	0.0247495
Mrps9|69527	1.065410299	0.280586677	1.148551039	0.079707656
Ptpn9|56294	1.065363442	0.389653469	1.371053843	0.044870288
Mettl21a|67099	1.065218179	0.350604678	1.297523825	0.011987453
Gpr132|56696	1.064979022	0.452242803	-1.247905598	0.351791864
Usp10|22224	1.064939893	0.333907933	1.103252947	0.181862053
Spg21|27965	1.064864439	0.345207964	1.167150849	0.161097895
Ccdc12|72654	1.064837754	0.358069321	-1.064861912	0.327543388
Dapk1|69635	1.064831719	0.372607647	1.335709418	0.026176055
Zfp91|109910	1.064789829	0.364059889	1.11500455	0.282465548
Smurf2|66313	1.064732737	0.388254338	1.309797591	0.044373658
Ugt2a3|72094	1.06471723	0.247199967	1.026681091	0.388840763
Slc31a2|20530	1.06470649	0.3040727	1.006284888	0.479468497
Bre|107976	1.064667889	0.29890082	1.015058129	0.451127688
Guk1|14923	1.064659256	0.295429945	1.00135206	0.495143498
9930104L06Rik|194268	1.06457793	0.393181087	-1.056535514	0.39348814
Dennd5b|320560	1.064506488	0.373548799	1.144389634	0.062273048
Apip|56369	1.064461699	0.3705515	-1.030032425	0.431737585
Klf10|21847	1.064446922	0.442488119	1.472701876	0.189675533
Mtch2|56428	1.06434301	0.174669434	1.028963109	0.319378412
Tomm7|66169	1.064340907	0.328788163	1.064340907	0.328788163
Zfp74|72723	1.064334303	0.353084457	1.073192885	0.256077182
Spcs1|69019	1.064315625	0.264445959	1.100463334	0.173862127
Tmem144|70652	1.064279543	0.46181974	1.064279543	0.46181974
Mettl8|228019	1.064186663	0.362063824	1.102264258	0.223857243
Hal|15109	1.06414678	0.291498787	1.06414678	0.291498787
Ociad1|68095	1.064055109	0.289588897	1.019040907	0.414200459
Dact1|59036	1.063991573	0.471955306	-3.346162822	0.024440623
Ubxn6|66530	1.063930698	0.352211589	-1.043615491	0.384647415
Grcc10|14790	1.063916958	0.374632244	-1.184257422	0.095358596
Ppm1b|19043	1.063907188	0.283196734	1.063907188	0.283196734
Hira|15260	1.063898021	0.244755362	1.10565916	0.127648309

Tubgcp4|51885	1.06389234	0.389297367	-1.063417477	0.36976439
Ccdc32|269336	1.063889745	0.390531317	-1.066441555	0.364416521
Ptpn1|19246	1.063671167	0.219627056	-1.034088966	0.237607195
Ssbp1|381760	1.063663051	0.351130585	1.161878515	0.178345294
Arl13b|68146	1.063592745	0.44121243	1.063592745	0.44121243
D19Bwg1357e|52874	1.063590734	0.329105273	-1.016685563	0.435504096
Vrk2|69922	1.063559598	0.299354778	1.204451565	0.016559101
Eltd1|170757	1.063515924	0.371150775	-1.137158113	0.199256695
Lman2l|214895	1.063507647	0.372234991	-1.069306499	0.342034605
Snx1|56440	1.063469663	0.322376085	1.143647307	0.157681553
Zrsr2|22184	1.063428369	0.380825857	-1.051498355	0.385833727
Cdk13|69562	1.063356031	0.367588965	-1.141938471	0.176059505
Pxmp3|19302	1.063323283	0.319909719	1.063323283	0.319909719
Sec22b|20333	1.063290029	0.353807576	1.15161148	0.173430527
Znhit6|229937	1.063257253	0.370831181	-1.04764137	0.381833243
Taf12|66464	1.063251213	0.310492839	1.000483639	0.498412203
Trnau1ap|71787	1.063201359	0.357038511	1.141747887	0.227455965
Anapc16|52717	1.063166906	0.334680382	-1.017491072	0.449457293
Zfp961|234413	1.063067195	0.420666857	-1.10656096	0.373733202
Wdr5b|69544	1.063051434	0.444752659	1.063051434	0.444752659
Mmadhc|109129	1.062921767	0.281562765	-1.012886932	0.4392171
Zfp869|66869	1.062899851	0.365922208	1.027662069	0.425864985
Haus8|76478	1.062836481	0.415617477	-1.336264302	0.055491208
Vps54|245944	1.062784363	0.366968389	-1.088389646	0.255562167
Arhgap9|216445	1.062558448	0.400789567	1.062558448	0.400789567
Fgfr1op|75296	1.062404177	0.400867971	1.192265255	0.245748225
Zmynd11|66505	1.062400134	0.373042737	1.226715328	0.104308409
Papd7|210106	1.062261467	0.409506487	-1.060003618	0.388086728
Rpl41|67945	1.062212403	0.415925813	1.001445342	0.496694815
Fcgr4|246256	1.06216848	0.373558804	1.21275425	0.014430299
Cnot6|104625	1.062146932	0.419759056	1.321723183	0.163338592
Lrrc27|76612	1.062114754	0.456521627	1.062114754	0.456521627
Commd6|66200	1.0621042	0.36058224	1.177348092	0.160770526
6430548M08Rik|234797	1.06198824	0.343691458	-1.004759129	0.486739687
Fgg|99571	1.061958809	0.307285601	-1.005555273	0.478886743
Tie1|21846	1.061926995	0.303427537	1.092759378	0.143386339
Tsc22d3|14605	1.061879741	0.354530879	1.259634224	0.050758508
Clip1|56430	1.061672378	0.417575685	-1.006967121	0.48536121
Chchd7|66433	1.061630912	0.382805943	1.061768432	0.376582716
Ndufaf3|66706	1.061501929	0.359192866	1.161863335	0.147967335
Drg1|13494	1.061480552	0.279961117	1.101833543	0.104049225
Rassf6|73246	1.061287912	0.415008046	-1.306489637	0.077879287
Gm5887|545893	1.061258918	0.467754835	-1.945954545	0.198949878
Sorl1|20660	1.061230764	0.449104921	1.061230764	0.449104921
Chst14|72136	1.061168955	0.410066562	1.175910256	0.284712305
Cyp8b1|13124	1.061077899	0.40175141	1.230282131	0.191059992
Srd5a3|57357	1.060963816	0.300548873	1.052714104	0.293984939
D7Ertd143e|100303645	1.060942851	0.416989789	1.352486425	0.103314539
Nhp2l1|20826	1.060882242	0.36788271	-1.011145766	0.474028376
Psmg1|56088	1.060700924	0.285322946	1.118459667	0.121906933
Scnm1|69269	1.060692052	0.406558945	1.200138972	0.244117575
41163|52398	1.060654347	0.376707425	1.060654347	0.376707425
Traf5|22033	1.06060285	0.40597247	-1.068255074	0.380822465

Hdgf|15191	1.060409853	0.105078052	1.090061254	0.027570989
Mst1|15235	1.060408412	0.225771515	-1.034700031	0.231512126
Mrpl43|94067	1.060406058	0.279713436	1.016663994	0.396811795
Psmc1|19179	1.060405412	0.25228939	1.082924681	0.101350772
Cisd2|67006	1.060295209	0.346008445	-1.000551398	0.498564601
Ensa|56205	1.06005706	0.337480454	-1.046193237	0.337300532
Rpl38|67671	1.05997361	0.401507809	-1.248344409	0.080789936
Cyp2d34|223706	1.059930483	0.47540746	-1.099033333	0.463232905
Il2ra|16184	1.059622413	0.467687086	-1.62524033	0.275850303
Csrnp1|215418	1.059539642	0.391788253	-1.054393853	0.386585531
Pfdn1|67199	1.059516148	0.40160973	1.013264364	0.452990117
Fam166a|68222	1.05947916	0.478923159	1	#DIV/0!
Top3b|21976	1.059474761	0.326653973	1.141342375	0.11437905
Glrx3|30926	1.059363246	0.365969646	1.14825885	0.214913099
Shf|435684	1.059351973	0.41570784	-1.14737226	0.284177497
Zfp704|170753	1.059339976	0.438094709	-1.224252246	0.285027752
Dohh|102115	1.059335289	0.372621223	-1.029791301	0.427882262
Slc12a2|20496	1.05932004	0.380595111	1.048314342	0.38013064
D930048N14Rik|97775	1.059295821	0.454884171	1.125489435	0.378698794
Lyrm4|380840	1.059277399	0.356571822	1.284917835	0.000161061
Klc2|16594	1.059238113	0.339550724	1.109650389	0.244765013
Ddx51|69663	1.059129974	0.381945787	1.171391329	0.171979959
Slco1b2|28253	1.05904367	0.395558561	1.195109105	0.133255001
Phb|18673	1.059028812	0.323900792	-1.091297145	0.155831488
Dync1h1|13424	1.058950039	0.336894776	-1.000558179	0.497710236
Cwc15|66070	1.058942358	0.250562367	1.131579613	0.036496497
Fbxo42|213499	1.05884909	0.339977938	-1.113203228	0.10220569
Rpl32|19951	1.058748381	0.390421627	-1.068699882	0.366050093
Apol6|71939	1.058729856	0.478854904	1	#DIV/0!
Kcnj11|16514	1.058729856	0.478854904	1	#DIV/0!
Acta1|11459	1.058729856	0.478854904	1	#DIV/0!
F13a1|74145	1.058729856	0.478854904	1	#DIV/0!
Lrrc2|74249	1.058729856	0.478854904	1	#DIV/0!
Armc3|70882	1.058729856	0.478854904	1	#DIV/0!
Gm5150|381484	1.058729856	0.478854904	1	#DIV/0!
Rims3|242662	1.058729856	0.478854904	1	#DIV/0!
Stox1|216021	1.058729856	0.478854904	1	#DIV/0!
Nkx2-6|18092	1.058729856	0.478854904	1	#DIV/0!
Ghrl|58991	1.058729856	0.478854904	1	#DIV/0!
Usp43|216835	1.058729856	0.478854904	1	#DIV/0!
Ak5|229949	1.058729856	0.478854904	1	#DIV/0!
Aldh3a1|11670	1.058729856	0.478854904	1	#DIV/0!
Cacna1s|12292	1.058729856	0.478854904	1	#DIV/0!
Trim65|338364	1.058423947	0.421086614	1.453769808	0.056552051
Atp8b1|54670	1.058380731	0.396407374	1.058380731	0.396407374
Ppa2|74776	1.058299105	0.285857517	1.002939378	0.487561901
0610007P14Rik|58520	1.058209106	0.362706569	1.276770016	0.022842332
Ptrf|19285	1.058198035	0.392866342	1.158117148	0.254129171
Atf3|11910	1.058165537	0.46253975	-1.029676965	0.483450161
Myl12a|67268	1.058065321	0.288220299	-1.013768038	0.432828271
Rps6kb1|72508	1.058057545	0.399212653	1.060284419	0.373134301
Maml1|103806	1.057934045	0.366321974	1.057934045	0.366321974
Fam13a|58909	1.057876436	0.422891222	1.46147508	0.063799628

Pdss2|71365	1.057845368	0.420827417	1.039772076	0.422159465
Mdn1|100019	1.057765338	0.409671829	-1.039454683	0.441557174
Zfp146|26465	1.057720221	0.440498622	-1.215734453	0.298671328
Gnptg|214505	1.057696785	0.291619	1.098884679	0.180783873
0610012H03Rik|74088	1.057663015	0.385101751	1.199079195	0.0997253
Ufc1|66155	1.057583571	0.337800893	1.057583571	0.337800893
Pold1|18971	1.057552945	0.43591897	1.057552945	0.43591897
Stoml1|69106	1.057519855	0.331712918	1.052452876	0.323201503
H2afz|51788	1.057435215	0.399282746	-1.217195804	0.062835776
Grb2|14784	1.057411683	0.143966452	1.123651951	0.002662088
Etf1|225363	1.057376134	0.195968547	1.094166286	0.06964865
Ccdc75|53951	1.057334465	0.32868158	-1.013591577	0.454674002
Hmgb1|15289	1.057281416	0.163296869	1.057281416	0.163296869
Stub1|56424	1.057225172	0.307840998	-1.001389446	0.494798598
Ube2z|268470	1.057147361	0.23550374	-1.001715018	0.486854431
Pus1|56361	1.057052819	0.305824804	1.014454118	0.447547795
Zfand3|21769	1.057013815	0.169289036	1.09786476	0.038873544
Sytl1|269589	1.057008938	0.405874933	-1.0523086	0.416545329
Atxn7l3|217218	1.056980517	0.341356971	1.241799427	0.01454146
Slc44a2|68682	1.05697597	0.370936943	1.272184208	0.032883567
Gan|209239	1.056971339	0.468215699	1.056971339	0.468215699
1810032O08Rik|66293	1.056919574	0.432307537	1.271188813	0.241143804
Pdia6|71853	1.056873342	0.375914883	-1.048986148	0.385658661
Rps6kc1|320119	1.056821821	0.398290387	1.216661393	0.171029761
Gmeb1|56809	1.056732805	0.354771964	1.056732805	0.354771964
Nipal1|70701	1.056680988	0.395791298	1.092333876	0.303761993
Dusp4|319520	1.056633307	0.465710977	1.491127627	0.297671603
Sulf2|72043	1.05661879	0.414261866	-1.090709326	0.298004476
Slc4a8|59033	1.056479601	0.441174132	1.29730939	0.257014712
H2-T9|15051	1.056441511	0.351788118	-1.023405692	0.383534905
Cog1|16834	1.05631956	0.276480225	1.033282048	0.307798649
Orc3|50793	1.05629673	0.373331783	1.17502575	0.16149541
Hyal1|15586	1.056281392	0.386019857	1.056281392	0.386019857
Ncoa2|17978	1.056264256	0.448799233	1.36150933	0.18556115
Heatr8|381538	1.056247287	0.468449533	-1.859926552	0.19322266
Daxx|13163	1.056241143	0.36219867	-1.038830327	0.395324135
Tmem192|73067	1.05618417	0.362623524	1.1349573	0.192985846
Rapgef3|223864	1.056180164	0.412268648	-1.120911463	0.304782248
Dock9|105445	1.056096469	0.409686024	1.21010663	0.217083334
Zfp553|233887	1.056088978	0.36369151	-1.03117987	0.411032954
5730403B10Rik|66626	1.056043689	0.334255797	1.18653355	0.031838819
Npc2|67963	1.056005199	0.362852177	-1.151448248	0.033017163
Arpc5l|74192	1.056001449	0.337126182	-1.035334287	0.373486448
Lpcat2|270084	1.0559651	0.462768579	-1.588592905	0.170297701
Ccdc6|76551	1.055855807	0.264567401	-1.006476187	0.458983856
Mnat1|17420	1.055853066	0.397638688	1.190221947	0.204912242
Sdf2l1|64136	1.05579392	0.437091092	1.631022496	0.02547318
Sarnp|66118	1.055776259	0.410279069	1.055776259	0.410279069
Ccdc53|67282	1.055764203	0.378744029	1.055764203	0.378744029
Evi5l|213027	1.055721735	0.433811988	1.196270936	0.309304052
Zdhhc17|320150	1.055592328	0.433758312	1.259524294	0.251194636
Gtf2e1|74197	1.055504352	0.445281464	-1.220515186	0.307894961
Snrpb|20638	1.055450387	0.348976602	1.055450387	0.348976602

Rnf8|58230	1.05543866	0.373628012	-1.139953572	0.152899179
Adm|11535	1.055429784	0.382864623	1.055429784	0.382864623
Ubxn2b|68053	1.055310054	0.462871313	1.055310054	0.462871313
0610030E20Rik|68364	1.055291958	0.37581673	-1.068883125	0.320215297
AU022252|230696	1.055251323	0.142228768	1.024163074	0.288076573
Klc3|232943	1.055249462	0.462827911	-1.971155704	0.078069994
Rbpj|19664	1.055238358	0.384330486	1.137936389	0.23177822
Mettl11a|66617	1.055197001	0.387789507	1.23856589	0.074638499
Stac3|237611	1.054789847	0.473261891	1.255130842	0.411829119
Fdx1|14148	1.054570543	0.383918556	-1.023086523	0.447319703
Fuz|70300	1.054569799	0.381832624	1.126595861	0.220114731
Psmc5|19184	1.054550354	0.322320108	1.054550354	0.322320108
Epha2|13836	1.054534012	0.387177822	1.155490765	0.225924522
Atf1|11908	1.054474617	0.421695207	1.29516672	0.145792618
1110002B05Rik|104725	1.054439532	0.328407021	-1.004029037	0.485581938
Slc24a6|170756	1.054436138	0.366907589	-1.013798039	0.463465038
Mtg1|212508	1.054355522	0.360704549	-1.039094657	0.388116973
Ltv1|353258	1.054293048	0.40245642	1.178454097	0.189369603
Med26|70625	1.054261937	0.404174407	1.054261937	0.404174407
Ptplad1|57874	1.05419143	0.307122569	1.102582419	0.186117209
Bpnt1|23827	1.054152771	0.343071173	1.239947757	0.007367987
Taf15|70439	1.054082867	0.388307124	1.054082867	0.388307124
Tpr|108989	1.054002163	0.363978957	1.137486043	0.202510764
Ndufb10|68342	1.054000207	0.365424154	-1.052335881	0.348618012
Fbxw11|103583	1.053938911	0.41555243	-1.095935977	0.322132398
3110056O03Rik|73218	1.053841547	0.333448729	1.011665297	0.450328559
Phka1|18679	1.053730951	0.426119916	1.426562935	0.077802347
Rrp9|27966	1.053580842	0.315700639	1.18763534	0.02825545
Fat1|14107	1.053547345	0.418576869	1.200154724	0.246572953
Tm2d1|94043	1.053514047	0.446706256	1.462838761	0.147161923
Mrps10|64657	1.05350729	0.346735911	1.05350729	0.346735911
Pbx1|18514	1.053495253	0.391013301	1.141547082	0.199471078
Arfgap1|228998	1.053438619	0.195058601	1.017622871	0.369338402
C330018D20Rik|77422	1.053432831	0.437744571	1.212223225	0.263692457
Ptpn2|19255	1.053400502	0.360094101	1.164913408	0.070737093
Rab4a|19341	1.053381344	0.360291327	1.173653139	0.101529508
Pcdhga4|93712	1.053296632	0.482972668	1	#DIV/0!
Trib2|217410	1.053251363	0.454843574	-1.097085571	0.418501383
Pxmp4|59038	1.053250394	0.289953867	1.077439868	0.233917904
Mapk1|26413	1.053226371	0.372419638	1.055912235	0.278849401
Abtb1|80283	1.053220393	0.314567429	1.00735939	0.473252148
Gpc6|23888	1.053171252	0.437766867	1.255132388	0.200746292
Nme7|171567	1.052981404	0.407692315	-1.095244964	0.328265048
Ifngr2|15980	1.052911431	0.351256881	1.115769048	0.200939449
Fus|233908	1.052897257	0.379073497	1.035376639	0.369441752
BC031181|407819	1.052863579	0.301919477	1.052863579	0.301919477
Hnrnpk|15387	1.052736968	0.33245029	1.096110131	0.224675498
Exoc3l|277978	1.052717403	0.408299688	1.00080909	0.498612146
Atp1b3|11933	1.052647013	0.346204489	1.109819467	0.223637775
Tchp|77832	1.052643159	0.376408708	1.093851463	0.301268008
Mlx|21428	1.052570957	0.352436763	-1.110288084	0.134312545
Grhpr|76238	1.052498791	0.358852954	1.162424387	0.118067019
Ppp1r15b|108954	1.052490792	0.367024915	1.052490792	0.367024915

Gm6525|624713	1.052371771	0.474664292	1.178571429	0.43416
Fam25c|69134	1.052350366	0.433828434	-1.211731089	0.230657108
Cmc1|67899	1.052342599	0.39129722	-1.040931791	0.405424457
Col5a3|53867	1.052324368	0.430584742	1.026415415	0.448582492
Calm2|12314	1.052260528	0.334441434	1.052260528	0.334441434
Zdhhc7|102193	1.052229302	0.335252984	1.118382411	0.177794887
Rps25|75617	1.052217526	0.37208387	-1.117856819	0.174115652
Zfp161|22666	1.052216219	0.427504721	1.052216219	0.427504721
A430005L14Rik|97159	1.052198305	0.356068463	1.094722383	0.264329093
Ahcyl1|229709	1.05219701	0.393109732	1.284398922	0.05711628
Zfp933|242747	1.052148515	0.439907673	1.075201585	0.410595076
Prelid2|77619	1.052023731	0.448290896	1.351739801	0.230183384
Tgds|76355	1.052018645	0.40824618	1.209453859	0.120982041
Vrk1|22367	1.051998157	0.40604068	1.044335979	0.404210433
Tnfrsf26|244237	1.051934246	0.461822982	1.051934246	0.461822982
Rnf11|29864	1.051853837	0.387152024	-1.040933679	0.409250969
Vhl|22346	1.051839811	0.363287675	-1.066271931	0.269874871
Gpihbp1|68453	1.051803328	0.387009795	1.055907388	0.342788987
Vdac2|22334	1.051738988	0.340374077	1.027741442	0.364495376
Zfp238|30928	1.051657002	0.402253658	1.12248861	0.213860626
Pard6g|93737	1.051619067	0.471081746	1.051619067	0.471081746
Pycr2|69051	1.051585999	0.41098804	1.051585999	0.41098804
Zfp84|74352	1.051538293	0.439949275	-1.145865814	0.275944095
Thap6|381650	1.051529207	0.421239571	1.051529207	0.421239571
Med13|327987	1.051453867	0.394767155	1.249881944	0.044954876
Isca1|69046	1.051448919	0.333889368	1.131767448	0.111120227
Gfm1|28030	1.051395379	0.395568946	1.200198858	0.147337514
Mxd1|17119	1.051221916	0.401341275	1.166897915	0.18880935
P4ha2|18452	1.051103207	0.409083043	1.051103207	0.409083043
Ndufa13|67184	1.051020749	0.412123052	-1.079675453	0.363800207
Spryd4|66701	1.050984071	0.374652112	-1.150622904	0.07312724
Gm13611|622534	1.050937446	0.435198347	-1.205252929	0.239862322
Zfml|18139	1.050908421	0.425589977	-1.271318125	0.132886456
Rpl29|19944	1.050904067	0.418335496	-1.121929649	0.299145944
Hgd|15233	1.050898606	0.324403834	1.050898606	0.324403834
Angptl4|57875	1.050791998	0.316493024	1.114327556	0.142884614
Afap1|70292	1.05075476	0.468084928	-1.281066738	0.335869801
Impa1|55980	1.05069354	0.354983967	1.232534106	0.015739895
Mapk13|26415	1.050648653	0.466112809	-1.521596721	0.24318857
Rab5b|19344	1.050582585	0.246537068	1.14268588	0.008292959
Tbc1d30|74694	1.050573476	0.421334937	-1.020792744	0.462789114
Zfyve19|72008	1.050565573	0.34452806	-1.077473814	0.191564914
Larp7|28036	1.050365002	0.396882201	1.050365002	0.396882201
Gpr68|238377	1.050347384	0.463103365	1.050347384	0.463103365
Arfip2|76932	1.050309658	0.372793055	-1.038609017	0.383755999
Agap1|347722	1.050308884	0.35020742	1.221796888	0.015424929
Hinfp|102423	1.050216166	0.455824589	1.606793227	0.110951371
Cnbp|12785	1.050214831	0.336926772	1.1266631	0.142339072
Slamf8|74748	1.050097764	0.47184832	1.998609651	0.185167526
AW209491|105351	1.050072992	0.403336525	-1.085627557	0.290154971
H2-Ke2|14976	1.049969696	0.410756068	1.049969696	0.410756068
Ccdc93|70829	1.049968769	0.378465908	1.121582077	0.22584182
2310061J03Rik|66391	1.049929987	0.411920597	-1.080121227	0.358838888

Arl11|219144	1.049875847	0.466464889	-1.358507869	0.317300187
Gm6654|626175	1.049758437	0.383513041	1.053253589	0.33131161
Saal1|78935	1.049510018	0.433515613	1.028064249	0.446028134
1110051M20Rik|228356	1.04949852	0.445597683	1.144845445	0.317801845
Mtx1|17827	1.049439524	0.388997208	-1.052293392	0.376845898
Timm13|30055	1.049409263	0.409479658	1.081196085	0.328703497
Chad|12643	1.049166111	0.473925396	-1.968383838	0.195840227
Gle1|74412	1.04914493	0.35415994	-1.089945394	0.198574647
Fkbp4|14228	1.049094199	0.387781195	-1.018278947	0.432173602
Mybbp1a|18432	1.049088365	0.321459753	1.040721473	0.304066022
Maml3|433586	1.049014894	0.425040784	-1.125693186	0.294355732
Adrb3|11556	1.049014799	0.445469304	1.282111314	0.250134728
Lap3|66988	1.048942832	0.259212245	-1.001359708	0.491020158
Rnf13|24017	1.048901111	0.418110256	1.100960142	0.316979315
Rpp14|67053	1.048873624	0.345306767	1.188630477	0.045998453
Gja4|14612	1.048867905	0.338286219	1.047897675	0.260439467
Ankrd28|105522	1.048849552	0.429474563	1.190686238	0.233636388
Mrpl28|68611	1.048802053	0.419507117	1.24724495	0.150854463
Nhsl2|100042480	1.048774515	0.433945025	1.294371581	0.169548496
Med9|192191	1.048725176	0.319860441	1.098937773	0.068168361
Chst13|71797	1.048724181	0.416593356	-1.083875058	0.358269833
Tha1|71776	1.048703447	0.38910602	1.153909744	0.19722371
Clu|12759	1.048694672	0.351778886	-1.028920092	0.400361555
Cenpa|12615	1.048600629	0.45737984	-1.038179648	0.464023008
4921513D23Rik|223989	1.048578873	0.338352657	1.049205688	0.283587022
Alkbh5|268420	1.048523621	0.253503721	1.067128033	0.108232403
Pstpip1|19200	1.048453505	0.462874026	1.442046654	0.256672771
Ctbs|74245	1.048434167	0.409152071	1.351611768	0.016571407
Ube4b|63958	1.048423477	0.30191571	-1.02273674	0.350244946
Dennd1b|329260	1.048350453	0.454392735	-1.554566441	0.08793987
4930579G24Rik|75939	1.048313939	0.454472086	1.704781267	0.057886952
Mosc2|67247	1.048291154	0.179885708	1.013235232	0.37116271
Znf512b|269401	1.048179542	0.420780298	1.048179542	0.420780298
Commd9|76501	1.048175919	0.433777705	1.002490621	0.495946851
Syt15|319508	1.048131501	0.438133211	1.436941739	0.096659449
Bod1|69556	1.048096944	0.346377242	-1.048356764	0.286775016
1110038F14Rik|117171	1.04809223	0.399428148	-1.10528118	0.23639053
1810027O10Rik|69186	1.048034504	0.428453382	-1.18026315	0.211498899
Myo1b|17912	1.048027123	0.401452713	1.190930429	0.159238374
Etaa1|68145	1.047994359	0.462833131	1.577597564	0.188040321
Add1|11518	1.047978867	0.3052414	1.047978867	0.3052414
H1f0|14958	1.047971982	0.329916143	1.158308393	0.056955807
Lrp5|16973	1.047950329	0.345435372	1.047950329	0.345435372
Cp|12870	1.047874439	0.391552834	-1.040284849	0.407555048
Cbx1|12412	1.047860548	0.38677149	1.030023551	0.40251499
Ano10|102566	1.047823409	0.383216244	-1.134427424	0.151053648
Akt2|11652	1.047818718	0.302575667	1.042397474	0.245369093
1500012F01Rik|68949	1.047800144	0.416341455	-1.072173275	0.376702929
Crybg3|224273	1.047705428	0.422058867	1.047705428	0.422058867
Lsm2|27756	1.047697451	0.384509641	1.047697451	0.384509641
Traf3|22031	1.047688303	0.442980204	1.337615115	0.173458508
Prep|19072	1.047662402	0.362692535	1.095907433	0.087861098
1810013L24Rik|69053	1.047650814	0.390189967	1.198928748	0.090436735

Pgm3|109785	1.047622538	0.451485184	1.047622538	0.451485184
Mepce|231803	1.047617733	0.383236107	-1.141634357	0.093579333
Itpa|16434	1.047562359	0.298312879	-1.01717157	0.399913451
Rabep1|54189	1.047549017	0.388131319	1.084751981	0.258238711
Ppp1r11|76497	1.04751619	0.340413538	1.04751619	0.340413538
Nt5c3|107569	1.047486301	0.363342403	1.19181163	0.058366165
Gatad2a|234366	1.047335774	0.205548757	1.080582554	0.07871334
Zfp827|622675	1.047269823	0.466562225	1.047269823	0.466562225
Lgi4|243914	1.04726355	0.467083044	1.607982192	0.216077246
Stoml2|66592	1.04719803	0.331448507	-1.037153313	0.286696563
Tmub1|64295	1.047161684	0.421760135	1.047161684	0.421760135
Anxa4|11746	1.047085668	0.375994126	1.001450647	0.494743433
Zfp316|54201	1.047029791	0.396332895	1.047029791	0.396332895
Ctu2|66965	1.047011634	0.4216477	1.188870898	0.187724679
Sqstm1|18412	1.04695866	0.330163097	1.04695866	0.330163097
Snrpa1|68981	1.046927123	0.34380414	-1.008886957	0.468712409
Ankrd13c|433667	1.046912746	0.435923492	1.046912746	0.435923492
Mrpl11|66419	1.04683162	0.351955262	-1.029351122	0.383774767
Gfra1|14585	1.046830408	0.426842745	1.041888343	0.438885553
Osbpl7|71240	1.046787784	0.412389278	1.168536054	0.191177688
Pafah1b1|18472	1.04676513	0.353303579	1.04676513	0.353303579
Gsto1|14873	1.04671636	0.213208499	1.04671636	0.213208499
Ciao1|26371	1.046640892	0.352488115	-1.103561845	0.095162994
Ppp4c|56420	1.046625704	0.412626578	-1.069604691	0.37055003
Rtn4rl1|237847	1.046591779	0.335481157	1.046591779	0.335481157
Doc2a|13446	1.046524843	0.437948668	1.046524843	0.437948668
Tbx3|21386	1.046523573	0.4364452	1.046523573	0.4364452
Golga2|99412	1.046505478	0.329261324	1.046505478	0.329261324
Smarcd1|83797	1.04643864	0.411394424	1.184035717	0.144675885
Terf2ip|57321	1.046327049	0.351836512	1.129500918	0.098773012
Pbxip1|229534	1.046215897	0.303452471	-1.021080398	0.372510481
Appl2|216190	1.046178332	0.411279191	-1.021756262	0.450105054
Nab2|17937	1.04617662	0.356232168	-1.018456152	0.434340812
A230046K03Rik|319277	1.046125631	0.427722957	1.046125631	0.427722957
Clec2d|93694	1.046119972	0.407324672	-1.092433992	0.299045091
Adrbk2|320129	1.045954062	0.458537381	1.437876758	0.204721188
Tubb4a|22153	1.045883462	0.476788533	-2.597803724	0.094986309
Psmc6|67089	1.045857736	0.4053912	-1.04416738	0.410584722
Hmox2|15369	1.045832514	0.375952087	1.045832514	0.375952087
Mrps24|64660	1.045778112	0.377004326	-1.121189137	0.133604322
Nenf|66208	1.045745837	0.441433771	-1.034329296	0.450275874
Zfp637|232337	1.045736321	0.357941536	1.045736321	0.357941536
Acap3|140500	1.045727372	0.430694447	1.379233784	0.065282343
Scamp1|107767	1.04571433	0.440350723	1.104600296	0.347368017
H2-Q8|15019	1.045680531	0.393982094	1.140135801	0.218747916
Rps4x|20102	1.04552847	0.405131177	-1.057160725	0.3801631
Gramd3|107022	1.04538473	0.371678501	-1.039308899	0.37722193
M6pr|17113	1.045348458	0.321066021	-1.015769012	0.425329241
Nop10|66181	1.045208127	0.350126417	-1.089188119	0.126902733
Mdh1|17449	1.045194302	0.288387106	1.018569141	0.350783567
2810004N23Rik|66523	1.045184664	0.275925891	-1.00134802	0.491279079
Serpina3h|546546	1.045182045	0.4734906	1.676433559	0.250918887
Leprot|230514	1.045106158	0.381574103	1.007194569	0.467251711

Map3k9|338372	1.044927387	0.474759578	1.044927387	0.474759578
Ahsa2|268390	1.044904693	0.414731871	1.301924614	0.062338759
Taf4a|228980	1.044874614	0.445351505	1.368443511	0.131551041
Fam19a2|268354	1.044843054	0.460079045	1.044843054	0.460079045
Irx3|16373	1.044829265	0.473844241	-1.050159484	0.473962888
Tmem57|66146	1.044810113	0.349781705	-1.016146022	0.437618098
Ruvbl1|56505	1.044791388	0.36219195	1.090607435	0.257993588
Pigh|110417	1.044688494	0.453113978	-1.307552335	0.200902064
Jmjd4|194952	1.044672852	0.410276633	1.15087092	0.240069466
Mpzl2|14012	1.04466721	0.412536628	1.206374629	0.145607222
Spata22|380709	1.044655819	0.472348623	1.570378755	0.26713503
2300009A05Rik|69478	1.04465235	0.416838891	-1.202058116	0.133463688
Pbx2|18515	1.044333167	0.345977161	-1.089029634	0.118312548
Rnaset2b|68195	1.044196974	0.416851186	-1.094216763	0.317317503
Tmem87a|211499	1.044180319	0.439313104	1.044180319	0.439313104
Pygo2|68911	1.044060614	0.363572616	1.189592877	0.046383864
Smarcc1|20588	1.044041386	0.365438782	1.166122146	0.038495838
1600029D21Rik|76509	1.043910882	0.427844739	1.233868492	0.170387604
Ankrd46|68839	1.043909915	0.412813771	1.280903219	0.051146032
Nup155|170762	1.043875721	0.404953588	1.193060863	0.128363409
Fam86|70511	1.043855548	0.428549753	-1.091150262	0.357342623
Parva|57342	1.043843344	0.275545139	-1.000014169	0.499911077
Fmo5|14263	1.043705453	0.3715978	1.152396721	0.0973439
Ssbp2|66970	1.043691466	0.467163718	1.460557681	0.253663621
Eif1b|68969	1.043683365	0.399979041	1.115752399	0.253601916
Fbxo8|50753	1.043649422	0.354593323	-1.018687929	0.428096778
Hexdc|238023	1.04348564	0.417609677	-1.074922681	0.337619517
Rpl7a|27176	1.043371777	0.378146972	1.043371777	0.378146972
Rusc2|100213	1.043366074	0.41036704	1.315747863	0.015621796
Acadvl|11370	1.0433487	0.339538645	1.076300285	0.245776823
Itpr1|16438	1.043277851	0.435483923	1.446264703	0.017267386
Iffo1|320678	1.043181029	0.421441344	-1.106751946	0.253130682
Kdm5a|214899	1.04307399	0.411696518	1.151709386	0.232142595
Ldha|16828	1.043034795	0.311241637	1.043034795	0.311241637
Spnb1|20741	1.042825451	0.438819204	1.023844787	0.458512866
Tpm4|326618	1.042811319	0.387128454	-1.039795664	0.379130786
Plekhg5|269608	1.042808789	0.434868425	-1.136813849	0.246283824
Setd8|67956	1.042761216	0.335538092	-1.010102966	0.457432144
Pdia4|12304	1.042759187	0.404629827	1.107860355	0.178010739
Nsmce4a|67872	1.042683449	0.379675387	1.052895245	0.269463596
Cygb|114886	1.042649758	0.391456043	-1.074278843	0.149683471
Ubl5|66177	1.042517285	0.40556746	1.047294182	0.363680359
Nudc|18221	1.042514897	0.377735391	1.093377184	0.254264687
Eif4e2|26987	1.042428881	0.168062973	1.038555402	0.118559341
Ttyh3|78339	1.04233425	0.402067124	-1.046371859	0.380409109
Usp6nl|98910	1.042127469	0.457977596	1.498920319	0.113663649
Socs7|192157	1.042109239	0.416286242	1.186218321	0.17683478
Zfp326|54367	1.042098303	0.410397285	-1.157415284	0.16052878
Ap2m1|11773	1.042075856	0.304438608	-1.017541985	0.378845932
Tm6sf1|107769	1.042061804	0.471485723	2.472755473	0.008963823
Pmpcb|73078	1.042056085	0.289117385	1.042056085	0.289117385
Rasgef1b|320292	1.042054199	0.415492731	1.159220039	0.223985943
Zfp932|69504	1.042036582	0.458964909	1.384357896	0.211368203

Gpx3|14778	1.042036111	0.426871401	-1.075375196	0.3743024
Armcx3|71703	1.041784249	0.418963809	1.179736968	0.197951383
Tmem64|100201	1.041709693	0.420982848	1.149218016	0.167805259
Trim27|19720	1.04153422	0.409184376	-1.095316825	0.264875932
Clns1a|12729	1.041526075	0.354802517	1.042346024	0.332459728
Cnot7|18983	1.041516234	0.440816261	1.076555219	0.380842365
BC016495|225994	1.041504523	0.411015425	1.147754257	0.15792041
Nck1|17973	1.041438576	0.451620836	-1.112527997	0.362499838
Ap3s2|11778	1.041329441	0.3262756	1.041329441	0.3262756
Rnf139|75841	1.04125162	0.435524391	-1.244976859	0.154567868
Prkrip1|66801	1.041228212	0.387701975	1.033158905	0.397350654
Heatr3|234549	1.041134807	0.386745485	1.111076625	0.212495632
Pkig|18769	1.041121795	0.362490215	-1.016795055	0.441055946
Vegfb|22340	1.041104475	0.441094293	-1.331364444	0.048911348
Arnt|11863	1.04109199	0.420432797	1.326427468	0.019855119
Dennd5a|19347	1.041064271	0.427961045	1.191811057	0.211419293
Map1lc3b|67443	1.041043672	0.302135661	1.021515493	0.399469715
Itga1|109700	1.040977649	0.447483451	1.128811049	0.284499114
D130043K22Rik|210108	1.040797457	0.46879859	1.102644747	0.417911906
Sucla2|20916	1.04073793	0.361207587	1.038333359	0.249344188
Zfp740|68744	1.040718533	0.410240903	1.067860591	0.299667405
Itsn1|16443	1.040711687	0.379470511	1.069927812	0.245870608
D19Ertd386e|52013	1.040691163	0.408215423	-1.131117315	0.078688399
Rcbtb1|71330	1.040682857	0.361648098	1.186322308	0.02374231
Gchfr|320415	1.040670437	0.432801096	-1.150149504	0.23937959
Rab2b|76338	1.040610708	0.463383537	-1.245433755	0.315882053
4933439C10Rik|74476	1.040588313	0.447069642	-1.151284521	0.268479841
Api5|11800	1.040584024	0.34566109	1.017410292	0.402510509
Camk2g|12325	1.040564127	0.440350022	1.424159469	0.040416191
Rasd1|19416	1.040516294	0.448108028	1.315125143	0.165317412
Srm|20810	1.040432866	0.404800345	-1.060343997	0.352043218
Ptprd|19266	1.040298374	0.403673045	1.129170445	0.203239233
Junb|16477	1.040233867	0.439452258	1.040233867	0.439452258
Dusp3|72349	1.040216437	0.339350187	1.068873369	0.204790453
Gltp|56356	1.04012456	0.412895899	-1.031084569	0.402724905
Rhobtb1|69288	1.040086241	0.406731354	-1.085713843	0.274881236
Rnf144b|218215	1.04007826	0.427147666	1.384261311	0.002340552
Rbfa|68731	1.040076154	0.40335319	-1.055589019	0.361031504
Amdhd1|71761	1.040066353	0.349884092	1.105386268	0.15078022
Trmt12|68260	1.039986625	0.411781141	1.168116648	0.172689812
1110059G10Rik|66202	1.039986142	0.447693577	-1.023695845	0.466642994
Ormdl1|227102	1.039949475	0.411007192	1.130331081	0.248705106
Zfp423|94187	1.039833438	0.477381501	-2.121330272	0.121092804
Psmd8|57296	1.039801903	0.382643961	1.091781581	0.251272411
Efcab4a|213573	1.03979129	0.432807326	-1.068090611	0.378477084
Trp53inp2|68728	1.039762776	0.323719171	1.117191385	0.035158092
Esrra|26379	1.039747424	0.397520233	1.039747424	0.397520233
Chmp6|208092	1.039523368	0.381291095	-1.002319867	0.49324376
Hspbp1|66245	1.039497276	0.426386259	1.037086949	0.421089195
Chpf2|100910	1.039453758	0.402610588	-1.146039259	0.10940207
Leng1|69757	1.039393188	0.40346209	1.078795837	0.325082768
Myl12b|67938	1.039347027	0.294038939	1.027914583	0.236502233
Otud4|73945	1.039242916	0.435253304	1.039242916	0.435253304

Myo1e|71602	1.039214869	0.376204226	1.046548787	0.277137188
Acaa1b|235674	1.039115686	0.410060203	1.039115686	0.410060203
Serpina3m|20717	1.039110218	0.42489637	1.313210514	0.033940662
2210015D19Rik|76508	1.039059379	0.421361747	1.091425041	0.332315242
Msi2|76626	1.039015915	0.425936687	1.197217957	0.171845936
Prpf19|28000	1.038955151	0.248180015	1.038955151	0.248180015
Mphosph9|269702	1.038944094	0.459841762	1.583041265	0.074068933
Mnt|17428	1.038901869	0.441417824	1.062538005	0.38548346
Tcp1|21454	1.038874116	0.262354346	1.081102511	0.072562986
C230081A13Rik|244895	1.038801134	0.430746758	-1.210886773	0.101154457
Cdc42se1|57912	1.038787486	0.314090138	1.038787486	0.314090138
Rnf40|233900	1.038776718	0.35849918	1.038776718	0.35849918
BC002163|170658	1.038702161	0.418497781	1.055690883	0.344353784
Spcs2|66624	1.038655727	0.400362262	1.038655727	0.400362262
Mfhas1|52065	1.038634588	0.394213144	-1.056735572	0.30456528
Tmem141|51875	1.038624344	0.44704636	-1.079535461	0.314750811
Fam174a|67698	1.038615875	0.447452296	1.405734351	0.082701323
Hectd1|207304	1.038589451	0.417817738	1.278511524	0.045120293
Upf1|19704	1.038571555	0.293939408	-1.003260556	0.478260303
2310003C23Rik|76425	1.038562985	0.374743903	1.117125484	0.128013223
Rpusd3|101122	1.038550652	0.432762468	1.209319602	0.183421732
Clec4a1|269799	1.038537309	0.450295223	1.038537309	0.450295223
Tradd|71609	1.038439764	0.388450109	-1.067704656	0.256008793
Ube2d2|56550	1.038307688	0.364040314	-1.015817483	0.420939383
Grasp|56149	1.038307146	0.421836239	1.208702004	0.115613461
Rapgef4|56508	1.038254046	0.446043621	1.224019235	0.2362299
Atp6v1f|66144	1.038179674	0.40642296	-1.049714869	0.37621942
Lsm4|50783	1.038107358	0.422354234	1.038107358	0.422354234
Rilpl2|80291	1.03808435	0.391830741	-1.015003179	0.458334131
Aif1l|108897	1.038041005	0.480181115	-2.514459712	0.094684578
Csnk2b|13001	1.037959701	0.397120586	1.037959701	0.397120586
Zfp703|353310	1.037854839	0.407586192	1.215030887	0.067692377
Sec24d|69608	1.037853391	0.441912304	-1.145084853	0.282190578
Ccpg1|72278	1.037852176	0.385633739	1.137687179	0.122654832
Nosip|66394	1.037833695	0.377521907	-1.093306552	0.176891134
Polr2f|69833	1.037821613	0.407763573	-1.065080722	0.331979824
Ddx6|13209	1.037810978	0.373358506	1.116767575	0.150044394
Atf6b|12915	1.037807175	0.373413293	-1.022903402	0.373099052
Efnb1|13641	1.037723987	0.40271795	-1.009559498	0.475381101
Mcart6|67062	1.037609224	0.459717202	1.235327852	0.250688934
Tprgl|67808	1.037602379	0.251281335	1.056798261	0.167958696
Sirt4|75387	1.037477259	0.429821661	-1.083582085	0.322965312
Trp53|22059	1.037277498	0.370684518	1.037277498	0.370684518
Ndufv1|17995	1.037160924	0.344585752	1.078277338	0.200502989
Fev|260298	1.037151403	0.481934061	1.170129778	0.437059136
Zfp771|244216	1.037033799	0.442643106	-1.176420531	0.217838669
Slc6a6|21366	1.036946045	0.410064073	-1.171971761	0.030065014
Hif1an|319594	1.036908688	0.39574058	1.109020924	0.207105065
Rtf1|76246	1.036884072	0.400678094	1.061607156	0.28178943
Map4k1|26411	1.036756663	0.466315338	-1.44863836	0.140681049
2900092E17Rik|67278	1.036748069	0.430614025	-1.11045224	0.285517018
Zfp113|56314	1.036727857	0.450492395	-1.05698373	0.424140714
Gpr180|58245	1.036672736	0.377717723	1.102960845	0.196367065

Vps28|66914	1.03664452	0.373844311	1.081216135	0.076204017
Pgap3|320655	1.036581761	0.432180953	1.036581761	0.432180953
Ap1s1|11769	1.036390851	0.378970526	1.084611559	0.240828038
Cd320|54219	1.036390423	0.441945932	1.017455104	0.445881987
Ndufb9|66218	1.036389332	0.41612047	-1.059672507	0.36156747
Rtn2|20167	1.036388056	0.474974131	1.411630972	0.230431711
Ddit3|13198	1.036328546	0.451015349	-1.039503374	0.425199032
Mtfp1|67900	1.036311471	0.379256957	1.089997325	0.237512804
St5|76954	1.036188438	0.384088745	-1.016411979	0.444418113
Dctn3|53598	1.036184775	0.337881678	1.036184775	0.337881678
Mfsd5|106073	1.036177618	0.342658987	1.081227904	0.173616135
Ywhaq|22630	1.036137499	0.35109594	-1.007280623	0.4686964
Rbm3|19652	1.036092207	0.425606633	-1.110391281	0.252632246
Tsc22d2|72033	1.03601744	0.446071885	-1.116043316	0.338053941
Mogs|57377	1.035994309	0.366368766	-1.002701672	0.490037185
Bckdhb|12040	1.035866023	0.379029422	1.099758085	0.202439823
Adat1|30947	1.035830054	0.41759194	-1.0913058	0.219303429
Tgfbr3|21814	1.035803691	0.441309601	1.417712684	0.005556594
Rab32|67844	1.035787555	0.412003628	1.035787555	0.412003628
Prdm4|72843	1.035739798	0.452317248	1.035739798	0.452317248
Flywch2|76917	1.035707672	0.461699524	1.151176346	0.342582252
Creb3|12913	1.035676868	0.365062845	-1.015859629	0.39224967
Scaf11|72193	1.035542388	0.375376577	1.09580224	0.182024785
Fpgs|14287	1.035533978	0.313680692	1.035533978	0.313680692
F9|14071	1.035434098	0.340967794	-1.019752337	0.394598589
Pdcd11|18572	1.035417946	0.397881915	1.117724638	0.167989424
Khsrp|16549	1.03534671	0.319586362	-1.051503699	0.164037123
Dpagt1|13478	1.035278514	0.377855	1.115991036	0.098225013
Marveld1|277010	1.035204851	0.416669229	-1.054432311	0.371372599
Tatdn1|69694	1.03517402	0.435455158	1.100189768	0.307252477
Slc16a10|72472	1.035115635	0.362918284	1.035115635	0.362918284
Ly6d|17068	1.035112766	0.466921851	-1.059234226	0.437750051
Cebpd|12609	1.035072685	0.412376255	-1.001545342	0.494573113
Copb1|70349	1.035062062	0.428012008	1.094163527	0.199711866
Kbtbd4|67136	1.035018355	0.394061625	-1.022676486	0.43166056
Raf1|110157	1.034986035	0.340136026	1.098615665	0.092916058
Med20|56771	1.034980995	0.419105678	1.171814496	0.139729053
Cox4nb|18117	1.034969951	0.345574768	1.003327014	0.480879551
Entpd1|12495	1.034956081	0.415063414	1.251409356	0.01962271
Klhl5|71778	1.034934276	0.426618924	1.126376341	0.246136082
Clic1|114584	1.034924036	0.393212606	1.112146658	0.194970811
Asgr1|11889	1.03473217	0.30221865	-1.006159361	0.455610188
Npdc1|18146	1.034676098	0.451280329	1.192022443	0.231823885
As3mt|57344	1.034609817	0.399398974	1.136197928	0.139517103
Jtb|23922	1.034560203	0.420731279	1.040315899	0.395545044
Vamp5|53620	1.034486848	0.407583777	-1.053478745	0.230931982
Eny2|223527	1.034456438	0.358000262	-1.061369933	0.217666382
Vkorc1l1|69568	1.034418517	0.447915315	1.034418517	0.447915315
Scyl1|78891	1.034300916	0.381830654	1.034300916	0.381830654
Stard7|99138	1.03426648	0.347325339	1.087410945	0.153023594
Darc|13349	1.03425913	0.475022835	-1.208652636	0.361353141
Xpo6|74204	1.034141916	0.342663868	1.073473537	0.189265331
Tshz2|228911	1.034088455	0.441694752	1.366000979	0.022912932

Znhit2-ps|29805	1.034044569	0.423712034	1.034044569	0.423712034
Map4k5|399510	1.033931005	0.440677813	1.033931005	0.440677813
Get4|67604	1.03390446	0.39037791	1.112982287	0.171691727
Irs1|16367	1.033883562	0.455483575	1.033883562	0.455483575
Irf8|15900	1.033847549	0.404824894	1.033847549	0.404824894
Caml|12328	1.033723119	0.304901009	-1.034442946	0.260643142
Rilpl1|75695	1.033690519	0.428851408	1.032821678	0.423811641
Smcr8|237782	1.033681734	0.406967697	1.12519811	0.191007203
Gpt2|108682	1.033607405	0.410962032	1.211316911	0.044825041
Mapkapk2|17164	1.033463284	0.331098421	-1.025999253	0.294684101
Pde2a|207728	1.033365011	0.387439306	1.105246855	0.181940333
Cul2|71745	1.033180987	0.419673583	-1.094038574	0.240965787
Pcdhga8|93716	1.033166051	0.488590015	-1.2762	0.173296754
Nom1|433864	1.033129914	0.417337619	1.248934385	0.013130731
Vps39|269338	1.033063599	0.425667899	1.137026941	0.229520246
Tnfrsf1b|21938	1.033007379	0.396025881	-1.046817649	0.338343432
Rps11|27207	1.032975481	0.427589867	1.030688185	0.440251234
Pnpla8|67452	1.032969853	0.426905907	1.126675062	0.255158148
Abcb6|74104	1.032922357	0.349315108	1.08426284	0.134578579
Actr3|74117	1.032891906	0.376946624	1.093226983	0.166212865
Nme1|18102	1.032856738	0.415340399	-1.059497747	0.321967056
Nrtn|18188	1.032842049	0.473454408	-1.331147262	0.28901939
Rbm6|19654	1.032728399	0.429099706	-1.101213717	0.265808223
Mxra8|74761	1.032717737	0.41919838	-1.049495907	0.3670167
Ccdc72|66167	1.032603332	0.359558608	1.032603332	0.359558608
Sart1|20227	1.032599905	0.36426803	-1.033521102	0.312373465
Mapk7|23939	1.032568222	0.432338558	1.036574575	0.427425685
Xrn2|24128	1.032562676	0.421619046	1.227595028	0.062285366
Tmem147|69804	1.032406509	0.395647116	1.012935034	0.441169749
Gm10845|100038734	1.032388599	0.472591351	1.150923	0.355490412
Stk38l|232533	1.032233558	0.453261447	1.032233558	0.453261447
Tmem176a|66058	1.032224274	0.426741409	-1.12356619	0.156874689
Slc7a2|11988	1.032207886	0.443890203	1.205385445	0.186762235
Stk4|58231	1.032148689	0.449223488	-1.008036019	0.480123257
Rbmxl1|19656	1.032053382	0.367478764	-1.088930452	0.039159547
Ascc2|75452	1.03204562	0.417752914	1.134169576	0.191496489
Slc33a1|11416	1.032009403	0.421609444	1.03739481	0.380197245
Gne|50798	1.031997401	0.42758426	1.031997401	0.42758426
Ppip5k2|227399	1.031883589	0.459590845	1.332356762	0.046705623
Ranbp6|240614	1.031870883	0.459109333	1.207769874	0.232487438
Rbbp6|19647	1.031771721	0.425053834	1.178732661	0.107866629
Il34|76527	1.031640103	0.437860722	1.129641595	0.250743494
Sema6b|20359	1.031629776	0.448900322	-1.180638529	0.195339204
Ccnf|12449	1.031626443	0.448449599	-1.017159558	0.46895777
Eef1g|67160	1.031552779	0.393806346	1.031552779	0.393806346
Supv3l1|338359	1.03154544	0.412040922	1.03154544	0.412040922
Cib1|23991	1.031466745	0.403577523	-1.0245188	0.425941549
9130011E15Rik|71617	1.03140539	0.457017805	-1.357366023	0.04944249
Gm4980|245190	1.031309202	0.410958798	1.031309202	0.410958798
1700021F05Rik|67851	1.031241934	0.410701956	1.113953368	0.179101446
Zfand2a|100494	1.031222663	0.466278931	1.272892174	0.26462553
Fgfr1op2|67529	1.030997305	0.39753553	1.107668883	0.146780797
Srd5a1|78925	1.030988231	0.422652986	1.122096218	0.230451784

Fam167b|230766	1.030956762	0.441893877	1.030956762	0.441893877
Snhg3|399101	1.030914058	0.451708131	1.058444277	0.35374077
Spg20|229285	1.030870905	0.398004914	1.115046654	0.152445761
Tmem93|66048	1.030789781	0.41174691	1.040585666	0.336052617
Ctnnd1|12388	1.030763592	0.285887926	1.006539734	0.449926783
Ank|11732	1.030659975	0.456069988	1.191633692	0.269568438
Cobl|12808	1.030607321	0.448182822	1.120472552	0.308769326
Nt5c|50773	1.03056609	0.447097481	-1.115650863	0.306912105
Eif3f|66085	1.03054027	0.392959494	-1.049126367	0.274189505
Cul1|26965	1.030403248	0.407541861	-1.028140048	0.409900173
Rnf7|19823	1.030383565	0.389720505	1.100174412	0.170539175
Ubxn1|225896	1.030378523	0.432843544	-1.06321177	0.364318072
3200002M19Rik|75430	1.030362211	0.434112666	1.030362211	0.434112666
Adrb2|11555	1.030282449	0.476599463	1.600726015	0.175319646
Nrxn2|18190	1.03021243	0.445502735	-1.079038181	0.348885187
Rbm22|66810	1.030203079	0.447030745	-1.12262979	0.289438401
Akap11|219181	1.030174637	0.408576164	-1.050689886	0.318613681
6720456B07Rik|101314	1.030164874	0.39574189	1.042928472	0.326940898
Actr5|109275	1.030123986	0.415358396	1.055331038	0.310450453
Smek1|68734	1.030097816	0.397964029	1.030097816	0.397964029
Cpne5|240058	1.03008618	0.47668013	2.00598855	0.039286495
Fam116a|211922	1.030024585	0.422871558	-1.033838059	0.395079482
Usp4|22258	1.030018706	0.308527363	1.021634451	0.315413265
Gpr107|277463	1.029932848	0.416137971	1.029932848	0.416137971
Serpina1a|20700	1.029907427	0.419093664	-1.159676216	0.030401264
Phf8|320595	1.029717629	0.432273902	1.178872575	0.121717459
Dbt|13171	1.029691484	0.443398516	1.236393591	0.079097585
Cfi|12630	1.029616528	0.33814443	1.029616528	0.33814443
Eef1a1|13627	1.029600776	0.402134168	-1.117327514	0.073450869
Psmd6|66413	1.029562918	0.384102118	1.073234194	0.246013182
Mtmr6|219135	1.029527696	0.461469678	1.489391889	0.036091845
Cript|56724	1.029495329	0.425245941	1.113545953	0.245873927
Prkag1|19082	1.029442294	0.386623818	1.029442294	0.386623818
Ankle2|71782	1.029348716	0.413243071	-1.060165036	0.281219082
Tpd52l2|66314	1.0293067	0.377046479	1.0293067	0.377046479
Ino80e|233875	1.029301919	0.411751219	1.187539781	0.052959055
Cdk19|78334	1.02927731	0.456874793	-1.160263518	0.277293121
Rasip1|69903	1.029194124	0.461175342	-1.262724048	0.013668692
Rpap3|71919	1.029142823	0.415179047	1.121337205	0.13930065
Mmachc|67096	1.029120457	0.434009335	1.235345917	0.068593948
Lipo1|381236	1.029021087	0.484084416	-1.695167316	0.261082002
2310057M21Rik|68277	1.029001405	0.469202185	1.029001405	0.469202185
Romo1|67067	1.028987085	0.448867087	-1.164967398	0.189974924
Cdcp1|109332	1.028944232	0.457582733	-1.293880375	0.125263539
Casd1|213819	1.028927415	0.456748475	1.421498738	0.029170958
Mylip|218203	1.028912736	0.39525935	1.086670304	0.219487009
Aste1|66595	1.028793623	0.456111039	1.201731026	0.238090158
Zfp68|24135	1.028764685	0.444281771	1.014316953	0.461649126
Rfx3|19726	1.028752559	0.481836072	1.679540169	0.22578993
Pdcl3|68833	1.028734802	0.442318847	-1.179691266	0.034953893
Ppp3ca|19055	1.028604321	0.450866311	-1.068305486	0.38072702
Cars|27267	1.028504577	0.4414467	1.014208715	0.464169855
Rangap1|19387	1.028360684	0.340393488	1.065903662	0.169378802

Slc35c1|228368	1.028356169	0.390276325	1.154371678	0.025638566
Fam192a|102122	1.028266381	0.422717003	1.028266381	0.422717003
Pcsk7|18554	1.028240114	0.389809818	-1.081015315	0.166641379
Il22ra1|230828	1.028227981	0.485054732	-1.010293975	0.492306781
Serpinf1|20317	1.028198478	0.422506787	-1.158403919	0.049226507
Crp|12944	1.028181588	0.375958302	1.099452374	0.08912434
Ppdpf|66496	1.02814979	0.431286107	1.026787035	0.418212079
Mbl2|17195	1.02810957	0.449735033	-1.09427341	0.230313477
Ube2g1|67128	1.028082261	0.427584742	1.028082261	0.427584742
Ces2a|102022	1.028058614	0.409359576	-1.121813039	0.046444606
Rod1|230257	1.027931464	0.451659415	-1.092973543	0.350176581
Aldh1a1|11668	1.027884152	0.452349291	1.356847753	0.031483136
Dhrs7b|216820	1.027777271	0.412788072	-1.048355679	0.340225268
Ncstn|59287	1.027565119	0.331337146	1.027565119	0.331337146
Lmna|16905	1.027516354	0.420764347	-1.130843412	0.122011052
Hcn2|15166	1.027459242	0.479915962	1.027459242	0.479915962
Nol12|97961	1.027455095	0.455367376	1.101137926	0.328771078
Anxa2|12306	1.027448377	0.432731018	1.112361258	0.232361464
Ampd2|109674	1.027447251	0.39052368	-1.01506447	0.410163235
Dnajc3|100037258	1.027436302	0.418514117	1.110795888	0.202483993
Polr3e|26939	1.027368025	0.405003282	1.108584752	0.151194293
Sdf2|20316	1.027305553	0.434762477	-1.086114382	0.249034462
1300014I06Rik|66895	1.027283641	0.359430092	1.058164366	0.225880692
Cbfa2t3|12398	1.027281579	0.456134857	1.243589177	0.152647833
Capza2|12343	1.027205294	0.44899673	1.344170465	0.010846873
Jarid2|16468	1.027193415	0.452511121	1.317936737	0.065044269
Sdhaf1|68332	1.027177927	0.433969936	1.096025905	0.280186184
Vta1|66201	1.027140196	0.411270737	1.027140196	0.411270737
Pex26|74043	1.027139484	0.395063269	-1.019502188	0.420147273
Ptprj|19271	1.027118661	0.441694876	1.268077114	0.042420158
1810026J23Rik|69773	1.027072493	0.430025016	1.097930629	0.259209456
Ctps|51797	1.027011557	0.46959924	1.397315162	0.131700896
Nop2|110109	1.026979301	0.370803856	1.03067097	0.322206954
Uqcr11|66594	1.026888115	0.453523508	-1.270734237	0.074218233
Mrpl13|68537	1.026765837	0.426569435	1.086815043	0.276552792
Podxl|27205	1.026736805	0.453941965	1.101496197	0.270321032
Cyp3a59|100041449	1.026732748	0.457965217	1.026732748	0.457965217
Nhp2|52530	1.026654299	0.429135892	-1.005106377	0.479506975
Tysnd1|71767	1.026617538	0.397513726	1.030799063	0.3280641
Cetn2|26370	1.02658845	0.428088028	1.109831971	0.234753367
Xcl1|16963	1.026549761	0.482029978	1.026549761	0.482029978
Abcb4|18670	1.026545057	0.402710264	1.026545057	0.402710264
4831426I19Rik|212073	1.026439208	0.46272492	1.174969383	0.256208666
Rhobtb3|73296	1.026418243	0.465630572	1.026418243	0.465630572
Scmh1|29871	1.026340167	0.451075528	-1.247519042	0.070485961
F10|14058	1.026286911	0.448165162	-1.137540204	0.211762776
Zhx1|22770	1.026228915	0.450577799	1.182731745	0.193474816
Zfp219|69890	1.026206134	0.427468395	1.030602537	0.382258976
Rps13|68052	1.026113032	0.451388692	-1.134710078	0.24593566
2310003L22Rik|69487	1.025991078	0.465854513	1.025991078	0.465854513
BC031781|208768	1.025878751	0.457516344	1.249458946	0.130750674
Plod3|26433	1.025818805	0.413831578	-1.121966371	0.053814029
Mark2|13728	1.025796107	0.41536501	1.025796107	0.41536501

Cab39|12283	1.025755367	0.4170841	1.114884066	0.156079365
Pgpep1l|78444	1.025656778	0.488586072	-1.049506076	0.483220987
Lrrc47|72946	1.025652888	0.45418957	1.351512247	0.016450764
Trrap|100683	1.025650422	0.434022196	1.025650422	0.434022196
Psmc4|23996	1.025544766	0.420414258	1.068493459	0.303486292
Rin2|74030	1.025522329	0.448758989	1.290711993	0.022697839
Acmsd|266645	1.025476277	0.442212391	-1.071686064	0.259784688
Acpl2|235534	1.025385387	0.464953706	1.025385387	0.464953706
Adam11|11488	1.025372984	0.472300637	1.2475301	0.28268363
BC048546|232400	1.025328615	0.46042135	-1.339354127	0.025133932
S100a1|20193	1.025323963	0.443785676	-1.114210375	0.227869492
Syngr2|20973	1.025322916	0.365733503	-1.024683388	0.331575816
E430018J23Rik|101604	1.025215439	0.452785481	-1.075967883	0.352140925
Supt16h|114741	1.025186582	0.460057808	1.002864943	0.494222506
Rplp0|11837	1.025067961	0.447575506	-1.079922347	0.339688142
Rsc1a1|69994	1.025062158	0.40565155	1.025062158	0.40565155
Eif4e|13684	1.025060433	0.427067969	1.058350467	0.34554544
Fam173a|214917	1.02501217	0.446063491	-1.09092278	0.306000804
Chkb|12651	1.025012054	0.383861512	-1.06407761	0.18412167
B3gnt3|72297	1.024921279	0.463125444	1.024921279	0.463125444
Alms1|236266	1.024912651	0.487792798	-1.299585882	0.391329351
Cda|72269	1.024905127	0.453618961	1.008583393	0.477762899
Lipt2|67164	1.024862193	0.472871887	-1.291346296	0.220673447
A630072M18Rik|320770	1.02480799	0.448218691	-1.109479346	0.268175408
Fmo2|55990	1.024774999	0.473981824	1.44506833	0.122042068
Aff4|93736	1.024749711	0.462613433	1.024749711	0.462613433
Zbtb25|109929	1.024735984	0.474485889	1.603829401	0.060200606
Grsf1|231413	1.024683795	0.451705463	1.148654681	0.244614302
Zfp438|240186	1.024622101	0.470948377	1.024622101	0.470948377
Bmyc|107771	1.024497892	0.466536913	1.44501659	0.022827161
Grb14|50915	1.024390624	0.416536791	-1.020555721	0.42881639
Ube2c|68612	1.024372414	0.488649967	-1.05694243	0.473102772
Serinc2|230779	1.024263727	0.451427081	1.024263727	0.451427081
Clta|12757	1.024229338	0.426480979	-1.135381832	0.072112466
Bmp2|12156	1.024221563	0.454612094	-1.087781337	0.238566232
Nin|18080	1.024142384	0.4720461	-1.175097476	0.325257269
Plod1|18822	1.024116546	0.414335746	1.107592139	0.097202694
Zfp708|432769	1.023990083	0.48471126	1.527564287	0.274467785
Gsk3a|606496	1.023971404	0.386529411	1.023971404	0.386529411
Spag7|216873	1.023941841	0.42283118	-1.129988121	0.061712705
Mettl2|52686	1.023894272	0.415049281	1.072854396	0.256580831
9830147E19Rik|208111	1.023883784	0.472240089	1.023883784	0.472240089
Ermp1|226090	1.023842801	0.447473292	1.029590551	0.420092929
Snx6|72183	1.023842756	0.449397002	1.135330299	0.209941862
Crbn|58799	1.023830965	0.454543227	-1.120777103	0.222742975
Pcnp|76302	1.023768287	0.405148375	-1.034323725	0.354324249
Bche|12038	1.023726344	0.424017264	1.067472837	0.220800524
Med27|68975	1.023678874	0.450007591	1.105555817	0.30588405
Luzp1|269593	1.02360694	0.45572889	1.185811875	0.187371048
Arid2|77044	1.02359037	0.458147741	1.326126286	0.040042429
Tmem140|68487	1.023529927	0.469245698	1.012939495	0.471320388
Uqcrq|22272	1.02337713	0.452964181	-1.237854081	0.053547474
Gnb2l1|14694	1.023354119	0.426775628	1.023354119	0.426775628

Trib1|211770	1.023330154	0.443553077	1.179106552	0.077566212
Ppp1r15a|17872	1.02325773	0.437312844	1.000508263	0.497893636
Hamp|84506	1.023244527	0.476963022	-1.333516211	0.221135604
Lipa|16889	1.023216117	0.405612867	1.086039876	0.150143015
Sec24b|99683	1.023087619	0.473554107	1.111801578	0.335791309
Glrx|93692	1.023077909	0.438058989	-1.057328772	0.348351692
Rab11fip3|215445	1.023029859	0.460150166	1.023029859	0.460150166
2610001J05Rik|66520	1.022951498	0.43503403	1.022951498	0.43503403
Fn3k|63828	1.022638367	0.469844052	1.068946061	0.40251434
Dedd2|67379	1.022582546	0.432655461	-1.035081725	0.399452527
Psma3|19167	1.022580247	0.413287183	-1.098121056	0.083314268
Cdc42bpa|226751	1.022395629	0.414728865	1.002447438	0.482668097
Myo10|17909	1.022351802	0.460278439	1.105003269	0.280170237
Mrpl18|67681	1.022348518	0.409639047	1.088850201	0.134513797
Exosc3|66362	1.022252373	0.445282008	-1.072820957	0.300919017
Ugt2b35|243085	1.022238478	0.464788677	-1.164802976	0.25074828
Ror1|26563	1.022104456	0.483295006	-1.331816821	0.309415265
Hnrnpu|51810	1.022102468	0.409435287	-1.021534826	0.3482989
Rab35|77407	1.022032937	0.390690691	1.022032937	0.390690691
Imp4|27993	1.022017127	0.423874105	1.022017127	0.423874105
Ap2a2|11772	1.02199817	0.353506723	1.093926214	0.013241627
Tgfb2|21808	1.021958947	0.487184813	-1.702123386	0.179137021
Cybasc3|225912	1.021934806	0.425781282	-1.072641455	0.204621359
Sun1|77053	1.021909547	0.465899659	1.200603794	0.230356273
Eif5|217869	1.021875882	0.44286005	1.136573325	0.166939183
Ezh1|14055	1.021802172	0.436344476	-1.126872319	0.142198258
Alkbh7|66400	1.021797523	0.460227557	-1.232248624	0.120479004
Dpf2|19708	1.02177473	0.379149618	-1.0655613	0.066887553
Pole3|59001	1.02171032	0.453349593	-1.102302273	0.281302821
Brd3|67382	1.021657128	0.439519703	1.210481188	0.011723616
Tsen54|76265	1.02160481	0.460806564	-1.107416171	0.317163248
Zfp282|101095	1.021515478	0.459667477	1.021515478	0.459667477
Brd7|26992	1.021428926	0.45767172	1.118835343	0.295603185
Zfp938|237411	1.021425643	0.471413757	-1.099563419	0.367788642
Ubap1|67123	1.021386111	0.40332838	-1.025046568	0.382460943
Nampt|59027	1.021375434	0.46628075	1.342864046	0.082594405
Hnrnpr|74326	1.021288179	0.472099377	-1.241572109	0.150612984
Sephs2|20768	1.021179893	0.43147102	-1.041038659	0.358821267
Slc41a3|71699	1.02115634	0.444685608	1.13335405	0.175272059
Tbc1d22b|381085	1.021144743	0.45656251	1.275439558	0.048270455
Lmo2|16909	1.021103116	0.474812018	1.021103116	0.474812018
Pam|18484	1.02097122	0.468890532	-1.319594205	0.097399461
Iqgap1|29875	1.020935158	0.471526163	-1.052679202	0.347222446
Fkbp2|14227	1.020848205	0.463099172	-1.106340355	0.325398101
Cnn2|12798	1.020839456	0.468233279	-1.211463914	0.180096292
Abce1|24015	1.020768371	0.442002152	1.195949076	0.041391212
Prpf38b|66921	1.020746233	0.452068124	1.174093179	0.113499438
Tyms|22171	1.020727143	0.461915378	1.042123663	0.391973405
Hk1|15275	1.020713199	0.427807633	1.020713199	0.427807633
Zfp36|22695	1.020709177	0.459163186	1.05080433	0.374021392
Dhx36|72162	1.02063731	0.4704461	1.102720993	0.363886327
2310079F23Rik|78890	1.020586833	0.452930318	1.020586833	0.452930318
Gxylt1|223827	1.020427309	0.468464613	1.402618159	0.026306629

Pdik1l|230809	1.020396464	0.466214876	-1.124213882	0.310026513
Cmtm7|102545	1.020318804	0.471143609	-1.341707325	0.083580085
Tox4|268741	1.019897709	0.449087785	1.131720381	0.187358236
Hps3|12807	1.019838605	0.474874217	-1.106239759	0.366712542
Ttc3|22129	1.019835194	0.462939174	1.019835194	0.462939174
Copz1|56447	1.019831029	0.403109721	-1.023088064	0.376603091
Qrich1|69232	1.019812352	0.447380629	1.098516246	0.243567544
C030006K11Rik|223665	1.019806807	0.435983646	1.072298255	0.279319749
Wdr33|74320	1.019768205	0.44590691	1.185876964	0.067334924
Slc10a3|214601	1.019675783	0.473442595	-1.200886068	0.253629413
Sec63|140740	1.019645679	0.426586803	1.064652863	0.138044538
Rpl23a|268449	1.019506263	0.45503279	-1.088038629	0.300641492
Pten|19211	1.019438863	0.430668459	1.019438863	0.430668459
Tmem208|66320	1.019416564	0.453743919	-1.061027791	0.349542777
Pigq|14755	1.019320681	0.453638949	1.12758686	0.180948995
Srp19|66384	1.019237379	0.432248372	1.019237379	0.432248372
Dpm1|13480	1.01920118	0.4551124	-1.038329081	0.377162248
Itgb1|16412	1.019161853	0.428677624	-1.048235239	0.309659194
Oxnad1|218885	1.019041574	0.457972605	-1.071441947	0.335540729
B4galt5|56336	1.0190057	0.473902264	1.312523339	0.119019068
AI314976|106821	1.018846508	0.46143972	-1.057648839	0.361056367
Sox5|20678	1.018818535	0.478816783	-1.175737928	0.290714612
Eif3k|73830	1.018714026	0.435686937	-1.019142552	0.438232636
Swi5|72931	1.018671267	0.409764973	1.058129666	0.233586443
Bdh2|69772	1.018586698	0.479154571	-1.187593547	0.223173542
Ccndbp1|17151	1.018579902	0.409552294	1.018579902	0.409552294
Fam18b|67510	1.018475829	0.476621925	1.018475829	0.476621925
Dus4l|71916	1.018444149	0.477056822	-1.395534711	0.103264352
Scamp2|24044	1.018434393	0.436757568	1.024226022	0.397211672
Xbp1|22433	1.018423263	0.443745796	1.170521628	0.072275645
Ssrp1|20833	1.018368715	0.436116157	-1.120584944	0.078125447
Ttc5|219022	1.018358686	0.465540555	-1.053453626	0.402567768
Sesn2|230784	1.018356401	0.475755006	-1.20679763	0.254229228
Arvcf|11877	1.018355439	0.459472479	1.131433899	0.200226204
Wapal|218914	1.018329034	0.469246113	1.189577669	0.219717309
Sec61b|66212	1.01821988	0.454612919	1.036206423	0.396889079
Ighmbp2|20589	1.018208116	0.446535569	1.113188276	0.18853813
Sumo2|170930	1.018184357	0.458918765	1.018184357	0.458918765
Gm14288|13999	1.018093582	0.467529208	-1.11475572	0.308282912
Aagab|66939	1.018078504	0.395409654	-1.011061091	0.433947659
Snx29|74478	1.018047782	0.486072294	-1.353359962	0.293706138
Sap30l|50724	1.017992347	0.464201951	-1.132876152	0.235210056
Nsa2|59050	1.017991562	0.420950494	1.017991562	0.420950494
5530601H04Rik|71445	1.017970554	0.48167536	-1.173877573	0.318484866
9130011J15Rik|66818	1.017960308	0.430158761	1.084977563	0.189553095
Nedd8|18002	1.017889368	0.4395906	1.105654644	0.157879435
Ccrl2|54199	1.017886021	0.485389987	1.330957295	0.296902167
Slc45a4|106068	1.017881361	0.474863693	-1.137839492	0.294786809
Icosl|50723	1.017771752	0.476131604	1.017771752	0.476131604
Ppp2ca|19052	1.017703547	0.457657667	-1.18857335	0.078882086
Mlycd|56690	1.017664553	0.461456636	-1.054319121	0.391367461
Thoc2|331401	1.017643681	0.479141697	1.254441175	0.252899506
Pmf1|67037	1.017628239	0.455687555	1.017628239	0.455687555

1810031K17Rik|69171	1.017598902	0.405266858	-1.013869284	0.365124449
Hip1|215114	1.017536269	0.470958152	1.141808939	0.297953629
Tbcel|272589	1.017533044	0.477890412	1.53438233	0.014192859
Dbr1|83703	1.017472419	0.449988491	1.190053278	0.045697376
Sult1a1|20887	1.017459148	0.459892068	-1.178355344	0.128912129
Sparcl1|13602	1.017452044	0.460676677	1.017452044	0.460676677
Ndufs7|75406	1.017450057	0.458489881	-1.088851664	0.291670128
Rfwd3|234736	1.017411869	0.463362113	1.017290958	0.440911306
Wdr26|226757	1.017404794	0.456018144	1.064381032	0.297754925
Per1|18626	1.017364409	0.460473661	1.11914588	0.257209194
Bcas2|68183	1.017309338	0.425055176	1.075656703	0.19249145
Gpr98|110789	1.01729724	0.489150407	-2.005626993	0.107561566
Def8|23854	1.017262937	0.454115621	1.102938068	0.225626541
Mta3|116871	1.017198773	0.448455351	1.017198773	0.448455351
2210403K04Rik|100042498	1.017095286	0.466192904	-1.231070288	0.091865017
Thumpd2|72167	1.017032677	0.485421598	1.017032677	0.485421598
Gpbp1|73274	1.017018816	0.44351683	1.017018816	0.44351683
Abca8b|27404	1.016893973	0.478017775	1.278105321	0.191324937
Fbxo7|69754	1.01681976	0.452820296	1.026158507	0.410479072
Riok3|66878	1.016807236	0.40887415	-1.033530626	0.290268618
Timm50|66525	1.016785152	0.450553932	-1.074575327	0.272053359
Rangrf|57785	1.016767317	0.48638934	1.016767317	0.48638934
Atp5g1|11951	1.016749935	0.467282182	-1.142083193	0.221420126
Zhx3|320799	1.016645312	0.467311168	1.016645312	0.467311168
Ptdss2|27388	1.016619613	0.463717238	1.016619613	0.463717238
Vps53|68299	1.016601495	0.419466599	1.112329568	0.041571044
Serpina1c|20702	1.016575234	0.447024874	-1.083926298	0.135734178
Creld2|76737	1.016543769	0.480040558	1.292744859	0.023855555
Crocc|230872	1.016522477	0.479166825	-1.419839673	0.056296284
Drap1|66556	1.016471085	0.465039173	1.013783548	0.462715375
Tmem167|66074	1.016387275	0.424574735	1.080646333	0.104963624
Fam26e|103511	1.016364976	0.491924933	1.195314368	0.428478238
Ftcd|14317	1.016339044	0.440299634	1.016339044	0.440299634
Mta1|116870	1.016329764	0.405344205	1.016329764	0.405344205
Cntn2|21367	1.016328212	0.482802951	1.212398432	0.277251959
Rsrc2|208606	1.016292175	0.476707333	-1.13150072	0.334256829
Cox7a2|12866	1.016275022	0.461397655	-1.200845736	0.055650028
Wdr85|67228	1.016123337	0.481498626	1.606754981	0.011207523
1110021L09Rik|76306	1.01598842	0.487222569	1.121204434	0.362056735
5430407P10Rik|227545	1.015965821	0.460074611	1.120143421	0.185017161
Samd4b|233033	1.015934841	0.40902992	1.015934841	0.40902992
Mettl5|75422	1.015930888	0.464428723	1.015930888	0.464428723
Chek1|12649	1.01589854	0.491594667	1.784660662	0.257682973
Tsfm|66399	1.01589764	0.467664154	-1.167208333	0.125686256
Ccdc99|70385	1.015877143	0.493760401	3.53972	0.173296754
Plekha8|231999	1.015866009	0.461030925	-1.053352238	0.378373784
2810002N01Rik|68020	1.015854098	0.439463805	-1.03131394	0.383980574
Lrrc14|223664	1.015790613	0.452483106	-1.062335569	0.313130201
Zufsp|72580	1.015753544	0.4905525	-2.550426666	0.006602581
Pomp|66537	1.015698261	0.450686776	1.112774252	0.100285588
Srxn1|76650	1.015695598	0.455292599	1.158599983	0.060506765
2810459M11Rik|72792	1.015690411	0.403885517	1.015690411	0.403885517
BC031353|235493	1.0156799	0.465169545	-1.057439171	0.259823114

Hook2|170833	1.015579804	0.425031271	-1.040875473	0.261419831
Pias4|59004	1.015526652	0.457918167	1.082355893	0.28553276
Slc41a1|98396	1.015482232	0.473961852	1.290790607	0.101719432
P2ry1|18441	1.015470422	0.465403525	1.111213594	0.278694233
Ppt2|54397	1.015439538	0.421812357	1.052936682	0.244595992
Zfp235|56525	1.015422801	0.48099469	-1.26825072	0.202888759
Ecd|70601	1.015325418	0.454360067	-1.048006622	0.353348062
Ppp1r12c|232807	1.015225325	0.449542454	1.070595631	0.27556142
Tuba1c|22146	1.015210745	0.46420639	-1.085274339	0.271429978
Rcl1|59028	1.015201024	0.450033613	-1.056652389	0.312570377
Ndufa8|68375	1.015071658	0.448415767	1.015071658	0.448415767
Egflam|268780	1.0150645	0.489557842	-1.378503281	0.308946836
Mt2|17750	1.015032873	0.478746885	1.015032873	0.478746885
Apold1|381823	1.014985278	0.488817955	1.53255533	0.221283047
Uxt|22294	1.014967062	0.479122771	-1.177019735	0.218296501
Usp49|224836	1.014959775	0.490109336	-1.6339871	0.21713774
Slc3a1|20532	1.014879378	0.479397398	-1.034542899	0.444623618
Hscb|100900	1.01475245	0.484857208	-1.509174339	0.02864394
Rsu1|20163	1.014705922	0.467889647	1.142682325	0.21439893
Il18bp|16068	1.014661098	0.474422488	-1.140135803	0.270896604
Eif3l|223691	1.014623706	0.439063726	1.043211037	0.334500695
Tppp|72948	1.0145651	0.482429555	1.503591803	0.054055554
Bpgm|12183	1.014494967	0.457944359	-1.063394866	0.319993699
Acbd6|72482	1.014488554	0.463656538	-1.077703294	0.28907919
Tmem204|407831	1.014420875	0.461040802	1.055867884	0.366814458
Rab3gap2|98732	1.01440573	0.468139497	-1.093923929	0.272878777
Yars|107271	1.014302292	0.465563205	1.014302292	0.465563205
Kif21a|16564	1.014283979	0.482296794	1.298591092	0.186324138
Hspa14|50497	1.01424738	0.471135661	-1.23082755	0.082667166
Fam176a|232146	1.014218469	0.451729651	1.066207706	0.285370982
Shkbp1|192192	1.014216672	0.457367757	1.081248777	0.261824899
C1qa|12259	1.014098047	0.473583641	-1.016424092	0.461627355
Trim39|79263	1.014045486	0.462811698	1.014045486	0.462811698
Tifa|211550	1.013999606	0.454698707	-1.076824849	0.231745062
Wdr31|71354	1.013892893	0.494840415	1	#DIV/0!
Dab1|13131	1.013892893	0.494840415	1	#DIV/0!
Mcrs1|51812	1.013818826	0.471866441	1.013818826	0.471866441
Becn1|56208	1.013806188	0.454329509	1.051824862	0.347545197
Orai3|269999	1.013792366	0.446273895	1.013792366	0.446273895
Efhd1|98363	1.013766529	0.492401929	-1.762234392	0.245786637
Uhrf2|109113	1.013732938	0.467155592	1.062327114	0.281253371
Fbxo11|225055	1.013703741	0.486825169	1.149827282	0.365148416
Npm3|18150	1.013671641	0.470487541	1.105381786	0.276848154
Ran|19384	1.013661321	0.417574738	-1.007446223	0.457141065
1810030N24Rik|66291	1.013649402	0.468738871	-1.110428411	0.247540441
Rab3ip|216363	1.013596032	0.428765789	1.013596032	0.428765789
Pja2|224938	1.013576914	0.481011029	1.013330528	0.477715336
Nsf|18195	1.013508231	0.469742536	1.013508231	0.469742536
Nat10|98956	1.013436515	0.462680693	-1.010225419	0.465657688
Ppp1r16b|228852	1.0131421	0.480165548	1.222828644	0.203939333
Prdm10|382066	1.013141542	0.490901655	1.429213653	0.290484699
Zfp810|235050	1.012999361	0.486879728	-1.088445091	0.416098649
Napb|17957	1.012962752	0.489183329	-1.409240487	0.238529812

Tmem149|101883	1.012959265	0.486489745	1.320645605	0.162041805
Zfp941|407812	1.012958975	0.492276972	1.012958975	0.492276972
Bms1|213895	1.012957917	0.464759982	1.142226063	0.111555106
Ppp1cb|19046	1.012930887	0.470275523	1.233033611	0.058866791
Eif4a2|13682	1.012925838	0.47599065	1.012925838	0.47599065
Pink1|68943	1.012902193	0.451775745	1.012902193	0.451775745
Tapt1|231225	1.012870909	0.47722768	1.139429594	0.250254174
2410003K15Rik|75593	1.012866435	0.467501801	-1.01973858	0.429998776
Apoc4|11425	1.012865934	0.477650112	1.003886348	0.491031811
Slc18a1|110877	1.012849949	0.479960032	1.015325378	0.46027234
Wdfy2|268752	1.012832873	0.493825867	3.836820084	0.036982216
Ddost|13200	1.012823935	0.439174417	1.066808366	0.197034363
Zfp60|22718	1.012811726	0.485973474	1.212197348	0.265160197
C030046E11Rik|226089	1.01271148	0.486730672	1.407737373	0.153717648
Nedd4l|83814	1.012701593	0.459022762	1.012701593	0.459022762
Hs3st3b1|54710	1.012616131	0.469885548	-1.066413967	0.339113184
Pfkfb4|270198	1.012606423	0.493667059	-1.501130924	0.326323118
Mpeg1|17476	1.012599161	0.480704388	-1.153900573	0.287899276
1810043H04Rik|208501	1.012506912	0.473697281	1.022943194	0.429615548
Traf6|22034	1.012398629	0.48153359	1.012398629	0.48153359
Sla|20491	1.012358845	0.489009811	-1.048930827	0.456221966
Gpank1|81845	1.012358384	0.478332199	-1.298839288	0.047756426
Slc11a2|18174	1.012331183	0.473009294	1.094895559	0.249298255
Foxo1|56458	1.012262427	0.47259142	1.012262427	0.47259142
Ndufb7|66916	1.01225036	0.473311266	-1.12546316	0.226549497
Peo1|226153	1.012230121	0.435644881	-1.020705259	0.389671031
Zzz3|108946	1.012199577	0.458669698	-1.133034319	0.014089444
Slfn2|20556	1.012178053	0.475893488	-1.164089988	0.16351753
Zfp639|67778	1.012091911	0.48139506	1.160151717	0.213298931
Camk2d|108058	1.012076782	0.483610288	1.012076782	0.483610288
Bsg|12215	1.012072513	0.460265217	-1.148245608	0.02878425
Eme2|193838	1.011894034	0.471348507	-1.044747357	0.396097109
Col4a3bp|68018	1.011885399	0.464676174	1.076656763	0.294082613
2310022B05Rik|69551	1.011762221	0.463681059	-1.071905982	0.275668129
Entpd4|67464	1.011730061	0.444314998	-1.081476218	0.119211247
Sirt5|68346	1.011594143	0.477416316	1.133442596	0.268941542
Snrpb2|20639	1.011492103	0.461825092	-1.054478841	0.308270271
Plrg1|53317	1.01139229	0.473410391	-1.123311866	0.20377911
Smox|228608	1.011314226	0.48133497	1.011314226	0.48133497
Nop14|75416	1.011267989	0.428231434	1.051645243	0.164846457
Qpctl|67369	1.011231165	0.458724957	1.049260635	0.26067872
Amfr|23802	1.011218611	0.465335942	1.090739598	0.210640884
Tmed7|66676	1.011206028	0.479501347	1.10071771	0.340037586
Utrn|22288	1.01120308	0.486710845	1.478400963	0.080852457
Dpm2|13481	1.011172267	0.46684672	1.011172267	0.46684672
Isoc2a|664994	1.011164798	0.465132126	-1.053142578	0.341345581
Filip1l|78749	1.011090083	0.486170289	1.312315586	0.16488001
Atrnl1|226255	1.011077575	0.484090908	1.388274764	0.058800688
Exoc8|102058	1.01101019	0.470405438	1.039151021	0.366666103
Mrps28|66230	1.010910359	0.477115687	1.009275755	0.476162226
Kif26b|269152	1.010899474	0.494239965	1.797535714	0.255240005
Klc1|16593	1.010874319	0.472352192	-1.081689731	0.276191058
Baiap2|108100	1.010799243	0.445295679	-1.039395095	0.272971972

Gatsl3|71962	1.010727941	0.48437803	1.010727941	0.48437803
Cox5b|12859	1.010641575	0.47108057	1.014431184	0.439272412
Prox2|73422	1.010570435	0.486698505	1.211027289	0.278458986
Rnf149|67702	1.010569644	0.460621666	1.0796703	0.189972933
Nhlrc3|212114	1.010531918	0.49003898	1.081633	0.414668405
Cirh1a|21771	1.010510507	0.474169996	1.102321458	0.271930433
Phf21a|192285	1.010467214	0.463258449	-1.035432679	0.383385723
Baiap2l1|66898	1.010401571	0.469984537	1.010401571	0.469984537
Dysf|26903	1.010394323	0.477991306	-1.10214317	0.297370164
Ss18|268996	1.010252597	0.472392944	1.065393688	0.300413033
Otud6b|72201	1.010248513	0.48558237	1.269619466	0.162446502
Josd2|66124	1.010232129	0.477758675	1.010232129	0.477758675
Polrmt|216151	1.010160432	0.441430593	-1.011950801	0.412696895
Tmem109|68539	1.010143333	0.469380308	-1.075703224	0.243116456
Dhdds|67422	1.01010982	0.465826763	1.158076712	0.041364505
Slc48a1|67739	1.010108576	0.462268763	-1.034429237	0.378765363
Rhoa|11848	1.010099234	0.445515404	-1.015024855	0.392693111
Zfp568|243905	1.010085238	0.486081761	1.114392739	0.22592233
Rrp36|224823	1.009975139	0.462427478	-1.057398141	0.278879441
Fbln1|14114	1.009930522	0.491309782	1.163097969	0.384883335
Mark3|17169	1.009901306	0.462433949	-1.070757491	0.203172992
Dnajb2|56812	1.009894617	0.465525793	-1.078469556	0.201101993
Prr15l|217138	1.009880813	0.493232699	1.315818981	0.34111768
Gpd1l|333433	1.009865576	0.473552106	-1.035893179	0.412718223
1700029F09Rik|75623	1.009799331	0.48951019	-1.225168737	0.301004293
Igfbp4|16010	1.009769549	0.456474502	-1.095271088	0.086612097
Zfp277|246196	1.009739165	0.488317103	-1.259744876	0.230311121
Ddx39b|53817	1.00971216	0.443246302	-1.070498887	0.068269535
Ypel3|66090	1.009707092	0.471588945	1.026139925	0.382229005
Klhl22|224023	1.009693589	0.476995871	1.123309736	0.228356622
Tyrobp|22177	1.009571811	0.481317024	-1.049086943	0.39283632
Cd14|12475	1.009536128	0.48560033	1.139764975	0.277333574
Plekha3|83435	1.009480396	0.482469199	1.169613391	0.214214508
Ccbl2|229905	1.009455942	0.471968443	1.045227856	0.311362517
Tmem177|66343	1.009446463	0.478775152	-1.08864154	0.291356643
Slc16a9|66859	1.009431647	0.488533495	-1.056979812	0.435814355
Adamts1|11504	1.009349241	0.489431619	1.009349241	0.489431619
Plxnb1|235611	1.009286665	0.467410047	1.002913202	0.485380904
Ppil2|66053	1.009187671	0.47628159	1.009187671	0.47628159
Ubtf|21429	1.009144338	0.421322914	1.041824555	0.124994903
Chac2|68044	1.00914126	0.479294682	-1.06377086	0.369019704
Eif4ebp1|13685	1.009124922	0.475181805	-1.096766448	0.188517669
Thumpd1|233802	1.009121754	0.477831872	-1.053944123	0.372540545
Hdac1|433759	1.009096219	0.47979786	1.009096219	0.47979786
Rap1a|109905	1.009054131	0.482868244	-1.106301304	0.317242309
Ogfod2|66627	1.009015352	0.479254475	-1.108059907	0.259122166
Zbtb41|226470	1.008993822	0.489232376	1.298605593	0.198849459
Erlin2|244373	1.008989616	0.47639675	-1.074464468	0.314985732
Ttf1|22130	1.008861099	0.485771342	1.168371381	0.212600019
AW549877|106064	1.008853695	0.487950103	1.292226372	0.14475601
Slc25a11|67863	1.00884277	0.45086951	-1.023056841	0.375647259
Exosc10|50912	1.008788448	0.471761477	1.081178004	0.256833572
Pdcl|67466	1.008683029	0.489807825	1.008683029	0.489807825

Elf2|69257	1.008650672	0.489008338	1.242678911	0.239658601
Polr2g|67710	1.008578796	0.476957769	1.008578796	0.476957769
Tspan7|21912	1.008562086	0.475193824	-1.004290905	0.481802375
Trip12|14897	1.008478536	0.475899972	1.176375203	0.069359948
Clmn|94040	1.008475816	0.457647967	1.008475816	0.457647967
Snx11|74479	1.008449751	0.476876996	1.007469565	0.464483512
Rfc2|19718	1.008404997	0.486167362	1.047094493	0.358000453
Eef2|13629	1.008381897	0.466393133	-1.044720571	0.324593304
Ppm1m|67905	1.008314127	0.478234244	-1.085658124	0.256036895
Vps26a|30930	1.008265955	0.47689476	-1.103638549	0.195861698
Rnf39|386454	1.008154457	0.488221345	1.354759509	0.098654852
Peg13|353342	1.008141043	0.476986055	1.099184134	0.236420155
Ephx1|13849	1.008103223	0.47918222	1.144806128	0.123220217
Klra2|16633	1.008040439	0.489940803	1.008040439	0.489940803
Fam108c|70178	1.008022931	0.479154367	1.122779514	0.192817381
Atad1|67979	1.008012863	0.486943338	1.107432441	0.20123543
Tsta3|22122	1.008004202	0.478720972	1.133633782	0.144729449
Eif3d|55944	1.00792612	0.477892299	1.00792612	0.477892299
Ncaph2|52683	1.007891672	0.469185441	-1.047321399	0.319509977
Skiv2l|108077	1.007885873	0.46335611	1.007885873	0.46335611
Chd6|71389	1.007877478	0.487617381	1.332045002	0.078712095
Rilp|280408	1.007838306	0.483401833	-1.230558896	0.069995126
Coq4|227683	1.007734183	0.48411179	-1.216810605	0.090970757
Gdi2|14569	1.007728169	0.464337354	-1.037769489	0.254999179
Exosc7|66446	1.007632921	0.469568901	-1.034233587	0.365418974
Tbc1d22a|223754	1.0075924	0.480058568	1.0075924	0.480058568
Cybb|13058	1.007535173	0.483911274	-1.075467199	0.34045947
Ptpn4|19258	1.007493161	0.496031655	1.784660662	0.257682973
Dazap1|70248	1.007389668	0.473096074	1.139869316	0.046369124
C2|12263	1.007361898	0.482510261	-1.16975141	0.027615773
5930434B04Rik|381356	1.007360463	0.472176438	1.063448543	0.236221414
Col6a2|12834	1.007346654	0.491129678	-1.181238318	0.14805545
1110067D22Rik|216551	1.007314594	0.480110336	1.050329843	0.373109246
Gadd45a|13197	1.007312829	0.492362937	1.007312829	0.492362937
Acap2|78618	1.007216713	0.492700572	-1.02027764	0.466355918
Ube2h|22214	1.00719926	0.469337624	1.054014334	0.274142698
Mrpl19|56284	1.007153399	0.486303431	1.257187039	0.078168702
Thap7|69009	1.007145738	0.484060335	-1.231140124	0.035607578
Eif2a|229317	1.007074653	0.462795576	-1.089931276	0.018332413
Snhg1|83673	1.00702157	0.490444801	1.165787395	0.271958275
Utp3|65961	1.006997727	0.476566258	1.006997727	0.476566258
Ppargc1a|19017	1.006967909	0.493334816	-1.136378123	0.352202139
Grpel1|17713	1.006961816	0.466073069	1.016967326	0.395598594
Hspb2|69253	1.006957827	0.496085219	-1.23872035	0.390455289
Prpf4b|19134	1.006935593	0.492008468	1.113924706	0.330611429
Prpf6|68879	1.006903895	0.470325997	-1.016501278	0.404745967
Ngfrap1|12070	1.006873833	0.484676305	-1.097672598	0.297193069
Gm12216|622459	1.006828386	0.496118085	1.481669861	0.318085772
Cdk2ap2|52004	1.006820387	0.488414342	1.021763717	0.445113121
Ldlrad3|241576	1.006758773	0.487724332	1.208203403	0.136593573
Thy1|21838	1.006751641	0.485230883	1.006751641	0.485230883
Usp7|252870	1.006744766	0.478358802	-1.158985578	0.024414827
Phospho2|73373	1.006700616	0.484943515	-1.125912132	0.221687169

Anp32e|66471	1.006682319	0.481902113	1.174427709	0.080695173
Ipmk|69718	1.00665063	0.47633859	-1.058759089	0.295105461
Igf2r|16004	1.006539595	0.485514991	-1.082674244	0.315005643
Gtf3c4|269252	1.006537729	0.487462722	1.177379746	0.179289713
Manea|242362	1.006448761	0.4909229	-1.228168133	0.209728986
Aspdh|68352	1.006328382	0.483775543	1.022176745	0.431558928
Gys2|232493	1.006226809	0.482434227	-1.00421624	0.486352538
Ldb1|16825	1.006162487	0.477710855	1.006162487	0.477710855
Ugt3a2|223337	1.006155083	0.47598166	-1.06955871	0.210341758
Tada2a|217031	1.006145572	0.490779914	1.00341008	0.491260233
Hyal2|15587	1.006145218	0.474449328	1.038872812	0.348742827
Pex5|19305	1.006136258	0.48133664	1.08976541	0.211519859
Dio3|107585	1.006124474	0.496334442	-1.659963261	0.249112461
Klf16|118445	1.006116118	0.487196522	1.007942077	0.480130229
Nploc4|217365	1.005982734	0.473245259	-1.043774864	0.306801684
Ogdh|18293	1.0059289	0.448515321	1.0059289	0.448515321
Rnf4|19822	1.005923815	0.485360733	1.105539749	0.226799787
Rad9|19367	1.005895776	0.490407449	1.005895776	0.490407449
Llph|66225	1.005886683	0.484243257	-1.076096406	0.308496462
Ostf1|20409	1.005846721	0.474774543	1.005846721	0.474774543
1810009A15Rik|66276	1.005830337	0.486707767	1.005830337	0.486707767
Obfc2a|109019	1.005828343	0.494224615	1.279370062	0.244543196
Sf3b2|319322	1.005747085	0.4746881	1.120913263	0.025981127
Ttl|69737	1.00569798	0.489401515	1.154376795	0.183667691
Man1a2|17156	1.005658298	0.471674978	1.035372099	0.338411794
Xpot|73192	1.005650129	0.487447702	1.004001773	0.489587366
Urod|22275	1.005513578	0.487510767	1.038802498	0.382325508
Klf6|23849	1.005504735	0.490274068	1.273308795	0.109814205
Hba-a2|110257	1.00547755	0.494091586	-1.249343404	0.279007734
Atox1|11927	1.005465796	0.488826582	1.070440347	0.325800728
Eif3b|27979	1.005376178	0.480330044	1.005376178	0.480330044
Psmd1|70247	1.005313196	0.480790467	-1.057534781	0.297859782
Csnk1e|27373	1.00520531	0.490496394	1.024990117	0.433234388
Nuak2|74137	1.005168273	0.487911815	1.005168273	0.487911815
5730469M10Rik|70564	1.004839999	0.48447497	-1.077865113	0.25142213
Slfn5|327978	1.004792071	0.489701331	1.113623021	0.278942225
Atp5j|11957	1.004780567	0.477378959	1.065420055	0.185539963
Dynlt3|67117	1.004740358	0.488603796	-1.073658835	0.338586925
Crb3|224912	1.004714101	0.482442579	-1.074277647	0.173089033
Znhit1|70103	1.004712859	0.489784008	1.030880594	0.42270676
Pcdh1|75599	1.004637443	0.483468813	-1.056850013	0.289938321
Tmem88|67020	1.004591809	0.495079743	-1.196105042	0.281050278
Naa35|78689	1.004546678	0.488398295	-1.05206281	0.380351132
Tmem191c|224019	1.004405143	0.492673712	-1.089616673	0.328225283
4930402H24Rik|228602	1.004334708	0.495724882	1.168685064	0.299246196
Ift20|55978	1.004205003	0.487551558	1.042652002	0.38290975
Lmln|239833	1.004202025	0.49769512	-2.043390563	0.184333064
Itgav|16410	1.00419945	0.49181907	-1.058149377	0.393463274
Wdr7|104082	1.004154987	0.493588465	1.156355835	0.290973033
Pik3r2|18709	1.004108931	0.485817931	1.144929488	0.054629037
Dnaja2|56445	1.00402132	0.49045019	1.008552025	0.476826723
Cfl1|12631	1.003964003	0.488444617	-1.077629425	0.283584716
Trpc6|22068	1.003935941	0.492888974	-1.040964609	0.406375976

Vopp1|232023	1.003932589	0.494889984	-1.045570401	0.435469066
Zwilch|68014	1.003868108	0.497937416	-2.600054995	0.091584858
A230051G13Rik|216792	1.003852714	0.489890846	-1.104710598	0.229263892
Fgb|110135	1.003813801	0.490125876	-1.100968559	0.207602062
Stab2|192188	1.003692711	0.482015302	1.003692711	0.482015302
Dsp|109620	1.003678009	0.495377636	1.279889215	0.197220744
Rpf2|67239	1.00367057	0.494788079	1.00367057	0.494788079
Coq10b|67876	1.003594846	0.491346623	-1.111217844	0.241943655
Mrpl22|216767	1.003553514	0.489411877	-1.036284858	0.320272242
Pa2g4|18813	1.003511108	0.486403337	1.120811197	0.081240179
Hbs1l|56422	1.003504094	0.486975404	1.127802426	0.081276901
Pfn1|18643	1.003416433	0.49233217	-1.222214513	0.0679824
Aldoart1|353204	1.003409033	0.498458011	3.852678571	0.080253065
Hsd17b4|15488	1.003397964	0.491587831	1.101102743	0.238240504
Mfsd11|69900	1.003371038	0.491535162	1.009590598	0.463236464
Fam168a|319604	1.003337529	0.493150938	1.086985827	0.267090714
Cd180|17079	1.003321238	0.497927592	-1.561907297	0.215130811
1190005F20Rik|98685	1.00328404	0.488820718	1.074234768	0.23636237
Ly6e|17069	1.003279096	0.49452265	-1.261097323	0.047122837
Canx|12330	1.003251182	0.487079051	1.051828653	0.308444253
Rft1|328370	1.003226696	0.490970287	-1.022330423	0.382447916
Nr1h4|20186	1.003155114	0.490016611	1.003155114	0.490016611
Surf1|20930	1.003140028	0.492649463	-1.084150404	0.17605069
Egln2|112406	1.003128442	0.491377735	-1.115623155	0.168288562
Eif3h|68135	1.003125668	0.484407813	-1.062584528	0.103084129
E4f1|13560	1.003086605	0.495150928	1.139375826	0.278596551
Pydc4|623121	1.003004119	0.498283018	1.652436434	0.267702974
2310047B19Rik|66962	1.002992663	0.493562506	1.0099554	0.470905862
Nudt1|17766	1.002991899	0.493469077	1.066887807	0.362277422
Ube2d3|66105	1.002980529	0.492265376	-1.096148081	0.261498596
Lyrm5|67636	1.002920344	0.492631118	-1.191914103	0.073391888
Tfec|21426	1.002890194	0.495809217	-1.081692648	0.375873312
Tsn|22099	1.002831188	0.492131078	1.095439383	0.248348788
Dck|13178	1.002802671	0.497847697	-1.476381935	0.2357447
Rbm18|67889	1.002778589	0.495099373	1.182427459	0.2021954
Mocs2|17434	1.002738357	0.487612442	-1.047902701	0.287441014
Dnase2b|56629	1.002693002	0.493323511	1.002693002	0.493323511
Vwa3a|233813	1.002688635	0.498820915	-3.967316667	0.09324139
Il7|16196	1.002688635	0.498820915	-3.967316667	0.09324139
Catsperg1|320225	1.002688635	0.498820915	-3.967316667	0.09324139
Tnfsf9|21950	1.002688635	0.498820915	-3.967316667	0.09324139
Sh2d4b|328381	1.002688635	0.498820915	-3.967316667	0.09324139
Ubash3a|328795	1.002688635	0.498820915	-3.967316667	0.09324139
Ubac2|68889	1.002654316	0.488860272	1.07385624	0.18039722
Wbscr22|66138	1.002641708	0.492473709	-1.168260072	0.056594893
Fam73b|108958	1.002571295	0.4942189	-1.065283644	0.30333315
Gm5088|328451	1.002565553	0.490694642	1.031995641	0.360985824
Erg|13876	1.002516996	0.497827603	1.075569681	0.439969022
Mzt1|76789	1.002465381	0.496257341	-1.144127225	0.309914371
Magt1|67075	1.002366879	0.489778409	1.057494066	0.242018294
Dnajb11|67838	1.002341737	0.493217774	1.111265577	0.174459237
Psmc3|19182	1.002303136	0.492582727	-1.042346759	0.376155689
Timm9|30056	1.002207325	0.493569499	1.101662481	0.208700293

Cmas|12764	1.002163527	0.48673654	-1.015421972	0.411873777
Sik1|17691	1.002160479	0.496570861	1.002160479	0.496570861
Mpzl3|319742	1.00213223	0.499069061	1.195314368	0.428478238
Gabpa|14390	1.002116394	0.49629809	1.345439486	0.01320468
Rnf130|59044	1.001823932	0.4924701	1.03634055	0.36342197
Smcr7|237781	1.001814272	0.494891624	1.001814272	0.494891624
B3gnt1|108902	1.001791893	0.495413283	1.060966299	0.360606941
Nphp1|53885	1.001773603	0.497044137	1.176825882	0.234316061
Elf1|13709	1.001628828	0.497723265	1.308109443	0.094720912
Zfp385b|241494	1.001483439	0.497280143	1.023778709	0.44606459
Cant1|76025	1.001471443	0.493940061	-1.018474685	0.379959568
Gcn1l1|231659	1.001465607	0.489445466	-1.026606334	0.313394799
Faim|23873	1.001399122	0.498031611	-1.143192731	0.297713682
Skil|20482	1.001336604	0.498704636	-1.098162335	0.382873896
Dcaf10|242418	1.001329415	0.497064733	-1.136957601	0.207253081
Eml2|72205	1.00131708	0.497169194	1.111296841	0.250788047
2310035K24Rik|69596	1.001315849	0.496253031	1.001315849	0.496253031
Msl2|77853	1.0013147	0.497950636	1.0013147	0.497950636
Ccdc117|104479	1.001272655	0.496648339	1.018822685	0.407038404
Igbp1|18518	1.00126816	0.495230551	-1.05528137	0.288650125
Tmem219|68742	1.001219863	0.497219703	-1.002145969	0.493266267
Rfc1|19687	1.001187046	0.497304539	1.009562201	0.47151272
Wbp7|75410	1.001182454	0.49708758	1.134326727	0.168474055
Ebp|13595	1.001170599	0.49498382	-1.107876208	0.073639452
Atp1a1|11928	1.000825116	0.495409301	1.057735946	0.115461362
Vat1|26949	1.000801008	0.496887478	1.053879172	0.302892666
Gpn1|74254	1.000767821	0.498173037	1.104407576	0.269372846
Nkrf|77286	1.000597505	0.499569044	1.000597505	0.499569044
Mkl2|239719	1.00053492	0.499335841	1.00053492	0.499335841
Hibch|227095	1.000505463	0.498671211	1.077505787	0.31378782
Rbms1|56878	1.000499782	0.499587368	1.467420972	0.214228303
Ppig|228005	1.00031052	0.498434902	-1.017696387	0.369424557
Sel1l|20338	1.000281742	0.499333782	1.003178045	0.492538965
Myg1|60315	1.000274793	0.499244102	1.096256272	0.246349852
Ndufb8|67264	1.000194634	0.499352391	-1.067309241	0.269759327
Hmgn1|15312	1.000192624	0.499067924	-1.042153066	0.295256168
Gadd45gip1|102060	1.000191723	0.499684132	-1.175405635	0.239654177
Slc35e1|270066	1.000189697	0.499444107	1.079821199	0.256953964
Zc3h3|223642	1.00009819	0.499752297	-1.09225947	0.258129378
Eif3g|53356	1.000077139	0.499770529	1.143987014	0.122302503
C1qbp|12261	1.000058707	0.499725948	-1.102626369	0.063848346
Gramd1a|52857	1.000010923	0.499973153	-1.065639018	0.291855615
Pabpc6|67543	-1	#DIV/0!	1	#DIV/0!
Olfr516|258720	-1	#DIV/0!	1	#DIV/0!
Olfr651|258809	-1	#DIV/0!	1	#DIV/0!
Hrnr|68723	-1	#DIV/0!	1	#DIV/0!
1700069L16Rik|381707	-1	#DIV/0!	1	#DIV/0!
Nlrp4b|210045	-1	#DIV/0!	1	#DIV/0!
Mir1a-2|723959	-1	#DIV/0!	1	#DIV/0!
Olfr544|257926	-1	#DIV/0!	1	#DIV/0!
Olfr1040|404323	-1	#DIV/0!	1	#DIV/0!
Cdkn3|72391	-1	#DIV/0!	1	#DIV/0!
AA467197|433470	-1	#DIV/0!	1	#DIV/0!

Olfr550|259108	-1	#DIV/0!	1	#DIV/0!
Taar8c|494546	-1	#DIV/0!	1	#DIV/0!
Olfr592|404317	-1	#DIV/0!	1	#DIV/0!
Cdc25c|12532	-1	#DIV/0!	1	#DIV/0!
Svs4|20941	-1	#DIV/0!	1	#DIV/0!
Nps|100043254	-1	#DIV/0!	1	#DIV/0!
Trpv5|194352	-1	#DIV/0!	1	#DIV/0!
Rbm46|633285	-1	#DIV/0!	1	#DIV/0!
Col19a1|12823	-1	#DIV/0!	1	#DIV/0!
4930483J18Rik|67638	-1	#DIV/0!	1	#DIV/0!
1190003K10Rik|68880	-1	#DIV/0!	1	#DIV/0!
Vmn2r55|100042499	-1	#DIV/0!	1	#DIV/0!
Rnf182|328234	-1	#DIV/0!	1	#DIV/0!
Gm14379|100270707	-1	#DIV/0!	1	#DIV/0!
Cdh22|104010	-1	#DIV/0!	1	#DIV/0!
Gm2030|100039065	-1	#DIV/0!	1	#DIV/0!
Olfr1359|258067	-1	#DIV/0!	1	#DIV/0!
Vmn1r230|171231	-1	#DIV/0!	1	#DIV/0!
Olfr1426|258805	-1	#DIV/0!	1	#DIV/0!
Mir871|100124478	-1	#DIV/0!	1	#DIV/0!
1700015G11Rik|75529	-1	#DIV/0!	1	#DIV/0!
Hist1h3f|260423	-1	#DIV/0!	1	#DIV/0!
Nrip2|60345	-1	#DIV/0!	1	#DIV/0!
1700013H16Rik|75514	-1	#DIV/0!	1	#DIV/0!
Kndc1|76484	-1	#DIV/0!	1	#DIV/0!
Olfr1173|404329	-1	#DIV/0!	1	#DIV/0!
Tmc2|192140	-1	#DIV/0!	1	#DIV/0!
Krtdap|64661	-1	#DIV/0!	1	#DIV/0!
Bhlhe22|59058	-1	#DIV/0!	1	#DIV/0!
Pcdhb3|93874	-1	#DIV/0!	1	#DIV/0!
Rbfox1|268859	-1	#DIV/0!	1	#DIV/0!
Snord88a|100217433	-1	#DIV/0!	1	#DIV/0!
Mir1937b-5|100499525	-1	#DIV/0!	1	#DIV/0!
Cmtm2a|73381	-1	#DIV/0!	1	#DIV/0!
Olfr1273-ps|258977	-1	#DIV/0!	1	#DIV/0!
Stk33|117229	-1	#DIV/0!	1	#DIV/0!
Spem1|74288	-1	#DIV/0!	1	#DIV/0!
Olfr461|258380	-1	#DIV/0!	1	#DIV/0!
Crisp4|78081	-1	#DIV/0!	1	#DIV/0!
Hist1h2aa|319163	-1	#DIV/0!	1	#DIV/0!
Creg2|263764	-1	#DIV/0!	1	#DIV/0!
Olfr62|18363	-1	#DIV/0!	1	#DIV/0!
Snord99|100217437	-1	#DIV/0!	1	#DIV/0!
Ube1y1|22202	-1	#DIV/0!	1	#DIV/0!
Tfap2a|21418	-1	#DIV/0!	1	#DIV/0!
Mir743b|100124496	-1	#DIV/0!	1	#DIV/0!
Gykl1|14625	-1	#DIV/0!	1	#DIV/0!
Spag11a|78128	-1	#DIV/0!	1	#DIV/0!
Cst6|73720	-1	#DIV/0!	1	#DIV/0!
Gm11818|208820	-1	#DIV/0!	1	#DIV/0!
Cdh10|320873	-1	#DIV/0!	1	#DIV/0!
Olfr507|258738	-1	#DIV/0!	1	#DIV/0!
Olfr1402|258272	-1	#DIV/0!	1	#DIV/0!

Zdhhc11|71164	-1	#DIV/0!	1	#DIV/0!
Gm5886|545886	-1	#DIV/0!	1	#DIV/0!
Clec2l|665180	-1	#DIV/0!	1	#DIV/0!
Mir450-1|723869	-1	#DIV/0!	1	#DIV/0!
4933427D06Rik|232217	-1	#DIV/0!	1	#DIV/0!
Lce1e|68694	-1	#DIV/0!	1	#DIV/0!
Prl8a9|67310	-1	#DIV/0!	1	#DIV/0!
Vmn2r110|224582	-1	#DIV/0!	1	#DIV/0!
Ccdc153|270150	-1	#DIV/0!	1	#DIV/0!
Mir1965|100316758	-1	#DIV/0!	1	#DIV/0!
Tnfsf15|326623	-1	#DIV/0!	1	#DIV/0!
Cts6|58518	-1	#DIV/0!	1	#DIV/0!
Mir3102|100526508	-1	#DIV/0!	1	#DIV/0!
Tas2r121|387349	-1	#DIV/0!	1	#DIV/0!
Abpd|494519	-1	#DIV/0!	1	#DIV/0!
Olfr887|258415	-1	#DIV/0!	1	#DIV/0!
Smarca1|93761	-1	#DIV/0!	1	#DIV/0!
Olfr47|18346	-1	#DIV/0!	1	#DIV/0!
2410137M14Rik|76797	-1	#DIV/0!	1	#DIV/0!
Olfr681|404318	-1	#DIV/0!	1	#DIV/0!
Bnipl|171388	-1	#DIV/0!	1	#DIV/0!
Mir704|735289	-1	#DIV/0!	1	#DIV/0!
Gm15319|100040599	-1	#DIV/0!	1	#DIV/0!
Olfr1038-ps|259015	-1	#DIV/0!	1	#DIV/0!
Mir448|723914	-1	#DIV/0!	1	#DIV/0!
Gja8|14616	-1	#DIV/0!	1	#DIV/0!
Wdr20b|70948	-1	#DIV/0!	1	#DIV/0!
Gm15284|100038927	-1	#DIV/0!	1	#DIV/0!
Gm15698|217066	-1	#DIV/0!	1	#DIV/0!
Krtap1-5|69664	-1	#DIV/0!	1	#DIV/0!
Mc3r|17201	-1	#DIV/0!	1	#DIV/0!
Prkg2|19092	-1	#DIV/0!	1	#DIV/0!
Gabra5|110886	-1	#DIV/0!	1	#DIV/0!
Cwh43|231293	-1	#DIV/0!	1	#DIV/0!
Krt73|223915	-1	#DIV/0!	1	#DIV/0!
Gm4489|100043513	-1	#DIV/0!	1	#DIV/0!
Nat3|17962	-1	#DIV/0!	1	#DIV/0!
Slc18a2|214084	-1	#DIV/0!	1	#DIV/0!
Mboat4|234155	-1	#DIV/0!	1	#DIV/0!
Mir683-2|100124649	-1	#DIV/0!	1	#DIV/0!
Serpine3|319433	-1	#DIV/0!	1	#DIV/0!
Nkx2-4|228731	-1	#DIV/0!	1	#DIV/0!
Tdpoz3|399674	-1	#DIV/0!	1	#DIV/0!
Olfr366|236509	-1	#DIV/0!	1	#DIV/0!
Crh|12918	-1	#DIV/0!	1	#DIV/0!
Mir222|723828	-1	#DIV/0!	1	#DIV/0!
1700011F03Rik|74221	-1	#DIV/0!	1	#DIV/0!
Rimbp2|231760	-1	#DIV/0!	1	#DIV/0!
Mir1249|100526461	-1	#DIV/0!	1	#DIV/0!
Mir465b-1|100124473	-1	#DIV/0!	1	#DIV/0!
Mir701|735278	-1	#DIV/0!	1	#DIV/0!
4933406J08Rik|74401	-1	#DIV/0!	1	#DIV/0!
Mir1964|100316711	-1	#DIV/0!	1	#DIV/0!

Olfr1255|258979	-1	#DIV/0!	1	#DIV/0!
Gm806|328250	-1	#DIV/0!	1	#DIV/0!
Rhox2f|434764	-1	#DIV/0!	1	#DIV/0!
Olfr800|258541	-1	#DIV/0!	1	#DIV/0!
Olfr1097|258840	-1	#DIV/0!	1	#DIV/0!
Zfp663|381405	-1	#DIV/0!	1	#DIV/0!
Mir3093|100526540	-1	#DIV/0!	1	#DIV/0!
Lcn3|16820	-1	#DIV/0!	1	#DIV/0!
2310043J07Rik|69665	-1	#DIV/0!	1	#DIV/0!
Olfr1156|258814	-1	#DIV/0!	1	#DIV/0!
Olfr1148|258220	-1	#DIV/0!	1	#DIV/0!
Gdf5|14563	-1	#DIV/0!	1	#DIV/0!
Khdc1a|368204	-1	#DIV/0!	1	#DIV/0!
Tmem207|100043057	-1	#DIV/0!	1	#DIV/0!
A730037C10Rik|320604	-1	#DIV/0!	1	#DIV/0!
Gpr101|245424	-1	#DIV/0!	1	#DIV/0!
Vmn1r5|171192	-1	#DIV/0!	1	#DIV/0!
Stmn2|20257	-1	#DIV/0!	1	#DIV/0!
Olfr50|18350	-1	#DIV/0!	1	#DIV/0!
Esco2|71988	-1	#DIV/0!	1	#DIV/0!
Tnfrsf13b|57916	-1	#DIV/0!	1	#DIV/0!
Pbp2|76400	-1	#DIV/0!	1	#DIV/0!
Defb43|654458	-1	#DIV/0!	1	#DIV/0!
Gm94|225443	-1	#DIV/0!	1	#DIV/0!
Ano4|320091	-1	#DIV/0!	1	#DIV/0!
Mirlet7g|387249	-1	#DIV/0!	1	#DIV/0!
Sprr2d|20758	-1	#DIV/0!	1	#DIV/0!
Iqcf3|68265	-1	#DIV/0!	1	#DIV/0!
2310057J18Rik|67719	-1	#DIV/0!	1	#DIV/0!
Krtap13|16699	-1	#DIV/0!	1	#DIV/0!
4932441B19Rik|238683	-1	#DIV/0!	1	#DIV/0!
Vmn2r53|637908	-1	#DIV/0!	1	#DIV/0!
Mir500|723974	-1	#DIV/0!	1	#DIV/0!
4930449I24Rik|67410	-1	#DIV/0!	1	#DIV/0!
Snora34|100217417	-1	#DIV/0!	1	#DIV/0!
Grin1|14810	-1	#DIV/0!	1	#DIV/0!
Oprm1|18390	-1	#DIV/0!	1	#DIV/0!
Tas2r107|387342	-1	#DIV/0!	1	#DIV/0!
Slc9a3|105243	-1	#DIV/0!	1	#DIV/0!
Ccdc65|105833	-1	#DIV/0!	1	#DIV/0!
Vmn2r37|22305	-1	#DIV/0!	1	#DIV/0!
Hdx|245596	-1	#DIV/0!	1	#DIV/0!
Mir183|387178	-1	#DIV/0!	1	#DIV/0!
Vgf|381677	-1	#DIV/0!	1	#DIV/0!
Vmn2r115|638102	-1	#DIV/0!	1	#DIV/0!
Krtap5-3|77215	-1	#DIV/0!	1	#DIV/0!
Olfr978|259109	-1	#DIV/0!	1	#DIV/0!
Olfr645|258247	-1	#DIV/0!	1	#DIV/0!
Insm1|53626	-1	#DIV/0!	1	#DIV/0!
1700008F21Rik|75453	-1	#DIV/0!	1	#DIV/0!
1700011E24Rik|75467	-1	#DIV/0!	1	#DIV/0!
Adcy8|11514	-1	#DIV/0!	1	#DIV/0!
Atp1b4|67821	-1	#DIV/0!	1	#DIV/0!

Krtap4-9|665998	-1	#DIV/0!	1	#DIV/0!
Chst9|71367	-1	#DIV/0!	1	#DIV/0!
Fanci|208836	-1	#DIV/0!	1	#DIV/0!
Olfr397|258343	-1	#DIV/0!	1	#DIV/0!
Oxct2a|64059	-1	#DIV/0!	1	#DIV/0!
Olfr485|258041	-1	#DIV/0!	1	#DIV/0!
Otoa|246190	-1	#DIV/0!	1	#DIV/0!
Olfr1537-ps1|257959	-1	#DIV/0!	1	#DIV/0!
Themis|210757	-1	#DIV/0!	1	#DIV/0!
Olfr398|258705	-1	#DIV/0!	1	#DIV/0!
Olfr1167|258291	-1	#DIV/0!	1	#DIV/0!
Lce1f|67828	-1	#DIV/0!	1	#DIV/0!
Kir3dl2|245615	-1	#DIV/0!	1	#DIV/0!
Snord45c|100217425	-1	#DIV/0!	1	#DIV/0!
Olfr1346|258918	-1	#DIV/0!	1	#DIV/0!
Hfm1|330149	-1	#DIV/0!	1	#DIV/0!
Lhfpl5|328789	-1	#DIV/0!	1	#DIV/0!
Slc26a5|80979	-1	#DIV/0!	1	#DIV/0!
Msgn1|56184	-1	#DIV/0!	1	#DIV/0!
Olfr926|258811	-1	#DIV/0!	1	#DIV/0!
Vmn2r62|546964	-1	#DIV/0!	1	#DIV/0!
Ascl4|67341	-1	#DIV/0!	1	#DIV/0!
Olfr205|257881	-1	#DIV/0!	1	#DIV/0!
Usp26|83563	-1	#DIV/0!	1	#DIV/0!
Olfr883|258414	-1	#DIV/0!	1	#DIV/0!
Olfr154|27216	-1	#DIV/0!	1	#DIV/0!
Lman1l|235416	-1	#DIV/0!	1	#DIV/0!
Gm5458|432825	-1	#DIV/0!	1	#DIV/0!
1700015E13Rik|76925	-1	#DIV/0!	1	#DIV/0!
C86695|97476	-1	#DIV/0!	1	#DIV/0!
Prb1|381833	-1	#DIV/0!	1	#DIV/0!
Ddx3y|26900	-1	#DIV/0!	1	#DIV/0!
Igfn1|226438	-1	#DIV/0!	1	#DIV/0!
Arxes2|76976	-1	#DIV/0!	1	#DIV/0!
Olfr556|258749	-1	#DIV/0!	1	#DIV/0!
Speer4e|624245	-1	#DIV/0!	1	#DIV/0!
Ostn|239790	-1	#DIV/0!	1	#DIV/0!
Prl7a2|19114	-1	#DIV/0!	1	#DIV/0!
Sh3gl2|20404	-1	#DIV/0!	1	#DIV/0!
Hoxa11as|15397	-1	#DIV/0!	1	#DIV/0!
Cntnap4|170571	-1	#DIV/0!	1	#DIV/0!
Hoxd10|15430	-1	#DIV/0!	1	#DIV/0!
Muc5b|74180	-1	#DIV/0!	1	#DIV/0!
Mir668|751523	-1	#DIV/0!	1	#DIV/0!
Il4|16189	-1	#DIV/0!	1	#DIV/0!
Olfr491|258731	-1	#DIV/0!	1	#DIV/0!
Hemt1|15202	-1	#DIV/0!	1	#DIV/0!
Allc|94041	-1	#DIV/0!	1	#DIV/0!
Pcdhb2|93873	-1	#DIV/0!	1	#DIV/0!
Slurp1|57277	-1	#DIV/0!	1	#DIV/0!
Bcl2l10|12049	-1	#DIV/0!	1	#DIV/0!
Ifna7|15970	-1	#DIV/0!	1	#DIV/0!
Olfr239|100038860	-1	#DIV/0!	1	#DIV/0!

Mir9-3|723968	-1	#DIV/0!	1	#DIV/0!
Cbln4|228942	-1	#DIV/0!	1	#DIV/0!
Olfr196|258775	-1	#DIV/0!	1	#DIV/0!
Olfr1250|258967	-1	#DIV/0!	1	#DIV/0!
Slc45a2|22293	-1	#DIV/0!	1	#DIV/0!
Rhox10|434769	-1	#DIV/0!	1	#DIV/0!
Klk1b11|16613	-1	#DIV/0!	1	#DIV/0!
Heatr7b2|223825	-1	#DIV/0!	1	#DIV/0!
Arsi|545260	-1	#DIV/0!	1	#DIV/0!
Sgcg|24053	-1	#DIV/0!	1	#DIV/0!
Gm8709|667572	-1	#DIV/0!	1	#DIV/0!
Olfr1083-ps|404326	-1	#DIV/0!	1	#DIV/0!
Dnajc6|72685	-1	#DIV/0!	1	#DIV/0!
Gsx2|14843	-1	#DIV/0!	1	#DIV/0!
Olfr1280|258910	-1	#DIV/0!	1	#DIV/0!
Eno4|226265	-1	#DIV/0!	1	#DIV/0!
Olfr1342|258708	-1	#DIV/0!	1	#DIV/0!
Mir200b|387243	-1	#DIV/0!	1	#DIV/0!
Olfr1201|258897	-1	#DIV/0!	1	#DIV/0!
Kcnip2|80906	-1	#DIV/0!	1	#DIV/0!
Gm6531|624855	-1	#DIV/0!	1	#DIV/0!
Skint7|328505	-1	#DIV/0!	1	#DIV/0!
Rspo4|228770	-1	#DIV/0!	1	#DIV/0!
Cthrc1|68588	-1	#DIV/0!	1	#DIV/0!
Vmn1r209|432736	-1	#DIV/0!	1	#DIV/0!
Mir7b|723883	-1	#DIV/0!	1	#DIV/0!
4930433N12Rik|114673	-1	#DIV/0!	1	#DIV/0!
Slc19a3|80721	-1	#DIV/0!	1	#DIV/0!
Gm5478|432987	-1	#DIV/0!	1	#DIV/0!
Casc5|76464	-1	#DIV/0!	1	#DIV/0!
Snord88c|100217434	-1	#DIV/0!	1	#DIV/0!
Klk11|56538	-1	#DIV/0!	1	#DIV/0!
4930584F24Rik|75942	-1	#DIV/0!	1	#DIV/0!
Trp63|22061	-1	#DIV/0!	1	#DIV/0!
Dgat2l6|668257	-1	#DIV/0!	1	#DIV/0!
Crx|12951	-1	#DIV/0!	1	#DIV/0!
Mir193b|100124432	-1	#DIV/0!	1	#DIV/0!
Vmn1r193|171259	-1	#DIV/0!	1	#DIV/0!
Tbr1|21375	-1	#DIV/0!	1	#DIV/0!
Prss3|22073	-1	#DIV/0!	1	#DIV/0!
Cpa2|232680	-1	#DIV/0!	1	#DIV/0!
Il12b|16160	-1	#DIV/0!	1	#DIV/0!
Mir710|735270	-1	#DIV/0!	1	#DIV/0!
Gm606|239789	-1	#DIV/0!	1	#DIV/0!
Fbxw22|382156	-1	#DIV/0!	1	#DIV/0!
Mir184|387179	-1	#DIV/0!	1	#DIV/0!
E530001F21Rik|279610	-1	#DIV/0!	1	#DIV/0!
Vmn1r21|171201	-1	#DIV/0!	1	#DIV/0!
Csmd1|94109	-1	#DIV/0!	1	#DIV/0!
2610028E06Rik|72395	-1	#DIV/0!	1	#DIV/0!
Mir3473|100499528	-1	#DIV/0!	1	#DIV/0!
Hoxa13|15398	-1	#DIV/0!	1	#DIV/0!
Vsig1|78789	-1	#DIV/0!	1	#DIV/0!

Sox11|20666	-1	#DIV/0!	1	#DIV/0!
Ptchd3|74675	-1	#DIV/0!	1	#DIV/0!
Gm11213|670833	-1	#DIV/0!	1	#DIV/0!
Nppa|230899	-1	#DIV/0!	1	#DIV/0!
Wdr16|71860	-1	#DIV/0!	1	#DIV/0!
Olfr667|259062	-1	#DIV/0!	1	#DIV/0!
Arhgap20|244867	-1	#DIV/0!	1	#DIV/0!
Trim9|94090	-1	#DIV/0!	1	#DIV/0!
Olfr729|258275	-1	#DIV/0!	1	#DIV/0!
Asb5|76294	-1	#DIV/0!	1	#DIV/0!
Bhlhe23|140489	-1	#DIV/0!	1	#DIV/0!
Tmem213|77522	-1	#DIV/0!	1	#DIV/0!
Olfr1440|258679	-1	#DIV/0!	1	#DIV/0!
Mir713|751548	-1	#DIV/0!	1	#DIV/0!
Gm5464|432870	-1	#DIV/0!	1	#DIV/0!
Mir196a-2|723958	-1	#DIV/0!	1	#DIV/0!
Gm11937|100041488	-1	#DIV/0!	1	#DIV/0!
Bmp8a|12163	-1	#DIV/0!	1	#DIV/0!
Capn13|381122	-1	#DIV/0!	1	#DIV/0!
Rnase9|328401	-1	#DIV/0!	1	#DIV/0!
Gm13629|100038743	-1	#DIV/0!	1	#DIV/0!
B830017H08Rik|433004	-1	#DIV/0!	1	#DIV/0!
E330017A01Rik|224247	-1	#DIV/0!	1	#DIV/0!
Olfr479|257891	-1	#DIV/0!	1	#DIV/0!
Il17a|16171	-1	#DIV/0!	1	#DIV/0!
Cd209e|170780	-1	#DIV/0!	1	#DIV/0!
Gm11569|670472	-1	#DIV/0!	1	#DIV/0!
Gng3|14704	-1	#DIV/0!	1	#DIV/0!
4931431F19Rik|70980	-1	#DIV/0!	1	#DIV/0!
4930542N07Rik|75185	-1	#DIV/0!	1	#DIV/0!
Mir711|751536	-1	#DIV/0!	1	#DIV/0!
Oog1|193322	-1	#DIV/0!	1	#DIV/0!
B130006D01Rik|320616	-1	#DIV/0!	1	#DIV/0!
Pnoc|18155	-1	#DIV/0!	1	#DIV/0!
Fam135b|70363	-1	#DIV/0!	1	#DIV/0!
1700061G19Rik|78625	-1	#DIV/0!	1	#DIV/0!
9230107M04Rik|77684	-1	#DIV/0!	1	#DIV/0!
Stfa2|20862	-1	#DIV/0!	1	#DIV/0!
Gm12886|666921	-1	#DIV/0!	1	#DIV/0!
Tdgf1|21667	-1	#DIV/0!	1	#DIV/0!
Gsdmcl-ps|74460	-1	#DIV/0!	1	#DIV/0!
Rims1|116837	-1	#DIV/0!	1	#DIV/0!
Grm7|108073	-1	#DIV/0!	1	#DIV/0!
Mc4r|17202	-1	#DIV/0!	1	#DIV/0!
Gdpd4|233537	-1	#DIV/0!	1	#DIV/0!
Rhox4e|194856	-1	#DIV/0!	1	#DIV/0!
Gm11529|100038600	-1	#DIV/0!	1	#DIV/0!
Olfr814|259165	-1	#DIV/0!	1	#DIV/0!
Klra14|654449	-1	#DIV/0!	1	#DIV/0!
A330076H08Rik|320026	-1	#DIV/0!	1	#DIV/0!
Krt33a|71888	-1	#DIV/0!	1	#DIV/0!
Dear1|654362	-1	#DIV/0!	1	#DIV/0!
Atoh1|11921	-1	#DIV/0!	1	#DIV/0!

Olfr467|257919	-1	#DIV/0!	1	#DIV/0!
Serpinb13|241196	-1	#DIV/0!	1	#DIV/0!
Fgf22|67112	-1	#DIV/0!	1	#DIV/0!
Defb3|27358	-1	#DIV/0!	1	#DIV/0!
1700067P10Rik|68224	-1	#DIV/0!	1	#DIV/0!
Abcb5|77706	-1	#DIV/0!	1	#DIV/0!
Olfr166|259071	-1	#DIV/0!	1	#DIV/0!
Cdh17|12557	-1	#DIV/0!	1	#DIV/0!
1700003G18Rik|69350	-1	#DIV/0!	1	#DIV/0!
Prok2|50501	-1	#DIV/0!	1	#DIV/0!
Fgf7|14178	-1	#DIV/0!	1	#DIV/0!
Olfr904|258797	-1	#DIV/0!	1	#DIV/0!
Vpreb2|22363	-1	#DIV/0!	1	#DIV/0!
Vmn1r88|100312474	-1	#DIV/0!	1	#DIV/0!
9130209A04Rik|77668	-1	#DIV/0!	1	#DIV/0!
Piwil1|57749	-1	#DIV/0!	1	#DIV/0!
4930549C01Rik|67663	-1	#DIV/0!	1	#DIV/0!
Olfr384|193053	-1	#DIV/0!	1	#DIV/0!
Olah|99035	-1	#DIV/0!	1	#DIV/0!
Pou6f2|218030	-1	#DIV/0!	1	#DIV/0!
Crisp3|11572	-1	#DIV/0!	1	#DIV/0!
Mir329|723842	-1	#DIV/0!	1	#DIV/0!
Olfr263|18341	-1	#DIV/0!	1	#DIV/0!
Epha8|13842	-1	#DIV/0!	1	#DIV/0!
Olfr282|258449	-1	#DIV/0!	1	#DIV/0!
Mir125a|387235	-1	#DIV/0!	1	#DIV/0!
Gm13040|100040854	-1	#DIV/0!	1	#DIV/0!
Lrrc26|227618	-1	#DIV/0!	1	#DIV/0!
Pld5|319455	-1	#DIV/0!	1	#DIV/0!
Mc1r|17199	-1	#DIV/0!	1	#DIV/0!
Fgf6|14177	-1	#DIV/0!	1	#DIV/0!
A930003O13Rik|330052	-1	#DIV/0!	1	#DIV/0!
Gm13102|100041077	-1	#DIV/0!	1	#DIV/0!
Mir207|387203	-1	#DIV/0!	1	#DIV/0!
Il1f8|69677	-1	#DIV/0!	1	#DIV/0!
Efcab8|329541	-1	#DIV/0!	1	#DIV/0!
Mir1966|100316712	-1	#DIV/0!	1	#DIV/0!
Tmem90a|627191	-1	#DIV/0!	1	#DIV/0!
Olfr70|56014	-1	#DIV/0!	1	#DIV/0!
Klk1b27|16619	-1	#DIV/0!	1	#DIV/0!
Olfr403|404316	-1	#DIV/0!	1	#DIV/0!
Kpna7|381686	-1	#DIV/0!	1	#DIV/0!
Chrnb4|108015	-1	#DIV/0!	1	#DIV/0!
Mir1306|100316814	-1	#DIV/0!	1	#DIV/0!
Slfn10-ps|237887	-1	#DIV/0!	1	#DIV/0!
Gap43|14432	-1	#DIV/0!	1	#DIV/0!
Olfr441|258649	-1	#DIV/0!	1	#DIV/0!
Npy|109648	-1	#DIV/0!	1	#DIV/0!
Gm4884|233164	-1	#DIV/0!	1	#DIV/0!
Hmgb4|69317	-1	#DIV/0!	1	#DIV/0!
L1td1|381591	-1	#DIV/0!	1	#DIV/0!
Gja3|14611	-1	#DIV/0!	1	#DIV/0!
Sntn|218739	-1	#DIV/0!	1	#DIV/0!

Gm7056|631105	-1	#DIV/0!	1	#DIV/0!
Gm1527|385263	-1	#DIV/0!	1	#DIV/0!
Abca13|268379	-1	#DIV/0!	1	#DIV/0!
Olfr487|258042	-1	#DIV/0!	1	#DIV/0!
Pou3f4|18994	-1	#DIV/0!	1	#DIV/0!
Cd200r3|74603	-1	#DIV/0!	1	#DIV/0!
Abcc8|20927	-1	#DIV/0!	1	#DIV/0!
Ibsp|15891	-1	#DIV/0!	1	#DIV/0!
Hs3st5|319415	-1	#DIV/0!	1	#DIV/0!
Spam1|20690	-1	#DIV/0!	1	#DIV/0!
Mir153|387171	-1	#DIV/0!	1	#DIV/0!
Gm853|332942	-1	#DIV/0!	1	#DIV/0!
Nmbr|18101	-1	#DIV/0!	1	#DIV/0!
Defb50|387334	-1	#DIV/0!	1	#DIV/0!
Sult1c1|20888	-1	#DIV/0!	1	#DIV/0!
Kcng3|225030	-1	#DIV/0!	1	#DIV/0!
Gm12|192852	-1	#DIV/0!	1	#DIV/0!
Spink8|78709	-1	#DIV/0!	1	#DIV/0!
Gm5414|406223	-1	#DIV/0!	1	#DIV/0!
Thbs4|21828	-1	#DIV/0!	1	#DIV/0!
Vmn1r45|22297	-1	#DIV/0!	1	#DIV/0!
Krt42|68239	-1	#DIV/0!	1	#DIV/0!
Nap1l2|17954	-1	#DIV/0!	1	#DIV/0!
1700006E09Rik|75437	-1	#DIV/0!	1	#DIV/0!
Gm14347|627264	-1	#DIV/0!	1	#DIV/0!
Pcdha6|12937	-1	#DIV/0!	1	#DIV/0!
AY761184|382000	-1	#DIV/0!	1	#DIV/0!
Fhl4|14202	-1	#DIV/0!	1	#DIV/0!
Mir19b-2|387195	-1	#DIV/0!	1	#DIV/0!
Olfr11|218066	-1	#DIV/0!	1	#DIV/0!
Rsph6a|83434	-1	#DIV/0!	1	#DIV/0!
BC018473|193217	-1	#DIV/0!	1	#DIV/0!
Olfr828|258598	-1	#DIV/0!	1	#DIV/0!
BC080695|329986	-1	#DIV/0!	1	#DIV/0!
Tmprss15|19146	-1	#DIV/0!	1	#DIV/0!
Rad51ap2|209550	-1	#DIV/0!	1	#DIV/0!
Kcnmb1|16533	-1	#DIV/0!	1	#DIV/0!
Tacr3|21338	-1	#DIV/0!	1	#DIV/0!
Tmprss11a|194597	-1	#DIV/0!	1	#DIV/0!
Olfr644|259125	-1	#DIV/0!	1	#DIV/0!
Magea6|17142	-1	#DIV/0!	1	#DIV/0!
Olfr345|258947	-1	#DIV/0!	1	#DIV/0!
Mir9-2|723967	-1	#DIV/0!	1	#DIV/0!
Olfr1459|258684	-1	#DIV/0!	1	#DIV/0!
Gm239|237558	-1	#DIV/0!	1	#DIV/0!
Mospd4|72076	-1	#DIV/0!	1	#DIV/0!
Hist1h2bm|319186	-1	#DIV/0!	1	#DIV/0!
Olfr284|258278	-1	#DIV/0!	1	#DIV/0!
Olfr469|258418	-1	#DIV/0!	1	#DIV/0!
Mir138-1|387156	-1	#DIV/0!	1	#DIV/0!
Abra|223513	-1	#DIV/0!	1	#DIV/0!
Wnt3|22415	-1	#DIV/0!	1	#DIV/0!
Cdh7|241201	-1	#DIV/0!	1	#DIV/0!

Olfr1121|258345	-1	#DIV/0!	1	#DIV/0!
Igfbpl1|75426	-1	#DIV/0!	1	#DIV/0!
Olfr1487|258629	-1	#DIV/0!	1	#DIV/0!
Cdrt4|66338	-1	#DIV/0!	1	#DIV/0!
Gm16390|100040937	-1	#DIV/0!	1	#DIV/0!
Krt26|320864	-1	#DIV/0!	1	#DIV/0!
Olfr767|258315	-1	#DIV/0!	1	#DIV/0!
Dmrtb1|56296	-1	#DIV/0!	1	#DIV/0!
1700074P13Rik|73481	-1	#DIV/0!	1	#DIV/0!
Mir1947|100316698	-1	#DIV/0!	1	#DIV/0!
Slc22a14|382113	-1	#DIV/0!	1	#DIV/0!
Slitrk1|76965	-1	#DIV/0!	1	#DIV/0!
Hpse2|545291	-1	#DIV/0!	1	#DIV/0!
Fabp6|16204	-1	#DIV/0!	1	#DIV/0!
Otop3|69602	-1	#DIV/0!	1	#DIV/0!
Trex2|24102	-1	#DIV/0!	1	#DIV/0!
Adam3|11497	-1	#DIV/0!	1	#DIV/0!
Olfr406-ps|258181	-1	#DIV/0!	1	#DIV/0!
Mir467d|100124447	-1	#DIV/0!	1	#DIV/0!
Mrgprx1|404242	-1	#DIV/0!	1	#DIV/0!
Mir1893|100316773	-1	#DIV/0!	1	#DIV/0!
Acpt|546967	-1	#DIV/0!	1	#DIV/0!
1700019O17Rik|71863	-1	#DIV/0!	1	#DIV/0!
Vmn2r59|628444	-1	#DIV/0!	1	#DIV/0!
Gabrg3|14407	-1	#DIV/0!	1	#DIV/0!
Mageb5|74271	-1	#DIV/0!	1	#DIV/0!
Lce1h|67718	-1	#DIV/0!	1	#DIV/0!
Rfx8|619289	-1	#DIV/0!	1	#DIV/0!
Olfr235|258681	-1	#DIV/0!	1	#DIV/0!
Vmn1r52|113849	-1	#DIV/0!	1	#DIV/0!
Prss52|73382	-1	#DIV/0!	1	#DIV/0!
Krt20|66809	-1	#DIV/0!	1	#DIV/0!
4933405O20Rik|243996	-1	#DIV/0!	1	#DIV/0!
Prss58|232717	-1	#DIV/0!	1	#DIV/0!
1110032F04Rik|68725	-1	#DIV/0!	1	#DIV/0!
Bglap2|12097	-1	#DIV/0!	1	#DIV/0!
Mrgprx2|243978	-1	#DIV/0!	1	#DIV/0!
Gad1|14415	-1	#DIV/0!	1	#DIV/0!
CK137956|635169	-1	#DIV/0!	1	#DIV/0!
Arxes1|76219	-1	#DIV/0!	1	#DIV/0!
Pappa2|23850	-1	#DIV/0!	1	#DIV/0!
Neurog2|11924	-1	#DIV/0!	1	#DIV/0!
Vmn1r155|667530	-1	#DIV/0!	1	#DIV/0!
Tas2r110|387344	-1	#DIV/0!	1	#DIV/0!
Apol11a|626615	-1	#DIV/0!	1	#DIV/0!
Barhl2|104382	-1	#DIV/0!	1	#DIV/0!
Olfr803|258547	-1	#DIV/0!	1	#DIV/0!
Vmn2r98|224552	-1	#DIV/0!	1	#DIV/0!
2210010C04Rik|67373	-1	#DIV/0!	1	#DIV/0!
Vmn1r86|100312473	-1	#DIV/0!	1	#DIV/0!
Pou3f1|18991	-1	#DIV/0!	1	#DIV/0!
Olfr78|170639	-1	#DIV/0!	1	#DIV/0!
Umod|22242	-1	#DIV/0!	1	#DIV/0!

Mir1224|100316739	-1	#DIV/0!	1	#DIV/0!
6430531B16Rik|381933	-1	#DIV/0!	1	#DIV/0!
Nrsn1|22360	-1	#DIV/0!	1	#DIV/0!
Krt39|237934	-1	#DIV/0!	1	#DIV/0!
Rbmxl2|76572	-1	#DIV/0!	1	#DIV/0!
Olfr124|259064	-1	#DIV/0!	1	#DIV/0!
4933407L21Rik|71141	-1	#DIV/0!	1	#DIV/0!
A730017C20Rik|225583	-1	#DIV/0!	1	#DIV/0!
Col11a1|12814	-1	#DIV/0!	1	#DIV/0!
Map6d1|208158	-1	#DIV/0!	1	#DIV/0!
Mir1969|100316777	-1	#DIV/0!	1	#DIV/0!
Mir1199|100316677	-1	#DIV/0!	1	#DIV/0!
Stx19|68159	-1	#DIV/0!	1	#DIV/0!
Dpcr1|268949	-1	#DIV/0!	1	#DIV/0!
Sh2d1b2|545378	-1	#DIV/0!	1	#DIV/0!
Vmn1r64|404285	-1	#DIV/0!	1	#DIV/0!
Ccdc147|381229	-1	#DIV/0!	1	#DIV/0!
Hist1h1a|80838	-1	#DIV/0!	1	#DIV/0!
Slc7a3|11989	-1	#DIV/0!	1	#DIV/0!
Ttll6|237930	-1	#DIV/0!	1	#DIV/0!
Drd2|13489	-1	#DIV/0!	1	#DIV/0!
Vmn1r34|546901	-1	#DIV/0!	1	#DIV/0!
2610002D18Rik|69885	-1	#DIV/0!	1	#DIV/0!
Mir3067|100526532	-1	#DIV/0!	1	#DIV/0!
Gsc2|195333	-1	#DIV/0!	1	#DIV/0!
1700057G04Rik|78459	-1	#DIV/0!	1	#DIV/0!
Vmn1r122|435951	-1	#DIV/0!	1	#DIV/0!
Prss16|54373	-1	#DIV/0!	1	#DIV/0!
Vax2|24113	-1	#DIV/0!	1	#DIV/0!
Rhox1|385343	-1	#DIV/0!	1	#DIV/0!
Teddm1|240819	-1	#DIV/0!	1	#DIV/0!
Olfr732|258659	-1	#DIV/0!	1	#DIV/0!
Sez6l|56747	-1	#DIV/0!	1	#DIV/0!
Rprl3|19785	-1	#DIV/0!	1	#DIV/0!
Mirlet7f-1|387252	-1	#DIV/0!	1	#DIV/0!
Olfr342|258950	-1	#DIV/0!	1	#DIV/0!
Galntl5|67909	-1	#DIV/0!	1	#DIV/0!
Mip|17339	-1	#DIV/0!	1	#DIV/0!
Opn1mw|14539	-1	#DIV/0!	1	#DIV/0!
Myot|58916	-1	#DIV/0!	1	#DIV/0!
Olfr1347|258383	-1	#DIV/0!	1	#DIV/0!
Vmn2r70|670940	-1	#DIV/0!	1	#DIV/0!
Pdc|20028	-1	#DIV/0!	1	#DIV/0!
Olfr492|258490	-1	#DIV/0!	1	#DIV/0!
Dupd1|435391	-1	#DIV/0!	1	#DIV/0!
Defb40|360217	-1	#DIV/0!	1	#DIV/0!
Cpb1|76703	-1	#DIV/0!	1	#DIV/0!
Olfr545|258837	-1	#DIV/0!	1	#DIV/0!
Efcab10|75040	-1	#DIV/0!	1	#DIV/0!
S100a2|628324	-1	#DIV/0!	1	#DIV/0!
Obox5|252829	-1	#DIV/0!	1	#DIV/0!
Olfr1137|258101	-1	#DIV/0!	1	#DIV/0!
Tas2r123|353167	-1	#DIV/0!	1	#DIV/0!

Gm221|237250	-1	#DIV/0!	1	#DIV/0!
C030030A07Rik|654818	-1	#DIV/0!	1	#DIV/0!
Olfr872|258553	-1	#DIV/0!	1	#DIV/0!
Olfr1451|258700	-1	#DIV/0!	1	#DIV/0!
4921539E11Rik|70941	-1	#DIV/0!	1	#DIV/0!
Rhox2c|100039948	-1	#DIV/0!	1	#DIV/0!
2700086A05Rik|72628	-1	#DIV/0!	1	#DIV/0!
Vmn2r87|625131	-1	#DIV/0!	1	#DIV/0!
Hand1|15110	-1	#DIV/0!	1	#DIV/0!
Gm10220|434689	-1	#DIV/0!	1	#DIV/0!
Zfy2|22768	-1	#DIV/0!	1	#DIV/0!
Prl5a1|28078	-1	#DIV/0!	1	#DIV/0!
Gm16532|100042450	-1	#DIV/0!	1	#DIV/0!
Kcnip1|70357	-1	#DIV/0!	1	#DIV/0!
Alpk2|225638	-1	#DIV/0!	1	#DIV/0!
2310007L24Rik|75573	-1	#DIV/0!	1	#DIV/0!
Krtap2-4|71453	-1	#DIV/0!	1	#DIV/0!
Mir3068|100526546	-1	#DIV/0!	1	#DIV/0!
Olfr460|258381	-1	#DIV/0!	1	#DIV/0!
Magea10|236852	-1	#DIV/0!	1	#DIV/0!
Olfr704|257902	-1	#DIV/0!	1	#DIV/0!
Olfr53|258962	-1	#DIV/0!	1	#DIV/0!
Ptger3|19218	-1	#DIV/0!	1	#DIV/0!
Olfr46|18345	-1	#DIV/0!	1	#DIV/0!
Olfr1213|258900	-1	#DIV/0!	1	#DIV/0!
4933436C20Rik|71296	-1	#DIV/0!	1	#DIV/0!
C2cd4b|75697	-1	#DIV/0!	1	#DIV/0!
Slc22a6|18399	-1	#DIV/0!	1	#DIV/0!
Olfr385|259025	-1	#DIV/0!	1	#DIV/0!
Olfr173|259002	-1	#DIV/0!	1	#DIV/0!
Tdrd1|83561	-1	#DIV/0!	1	#DIV/0!
Snora20|100303746	-1	#DIV/0!	1	#DIV/0!
Gpr111|435529	-1	#DIV/0!	1	#DIV/0!
Mir320|723838	-1	#DIV/0!	1	#DIV/0!
Prr9|109314	-1	#DIV/0!	1	#DIV/0!
Gm5136|368203	-1	#DIV/0!	1	#DIV/0!
1700024G13Rik|67085	-1	#DIV/0!	1	#DIV/0!
4930503H13Rik|74941	-1	#DIV/0!	1	#DIV/0!
Agtr2|11609	-1	#DIV/0!	1	#DIV/0!
Gm13124|627085	-1	#DIV/0!	1	#DIV/0!
Sdr16c6|242286	-1	#DIV/0!	1	#DIV/0!
Clca2|80797	-1	#DIV/0!	1	#DIV/0!
Ppef2|19023	-1	#DIV/0!	1	#DIV/0!
Galr1|14427	-1	#DIV/0!	1	#DIV/0!
Vmn2r41|100042848	-1	#DIV/0!	1	#DIV/0!
Cnksr2|245684	-1	#DIV/0!	1	#DIV/0!
Pax2|18504	-1	#DIV/0!	1	#DIV/0!
AA387883|100043450	-1	#DIV/0!	1	#DIV/0!
Olfr1307|257956	-1	#DIV/0!	1	#DIV/0!
Ndst3|83398	-1	#DIV/0!	1	#DIV/0!
Vmn2r38|434110	-1	#DIV/0!	1	#DIV/0!
Gm5726|435946	-1	#DIV/0!	1	#DIV/0!
Bsx|244813	-1	#DIV/0!	1	#DIV/0!

Aanat|11298	-1	#DIV/0!	1	#DIV/0!
Gm8884|667933	-1	#DIV/0!	1	#DIV/0!
1810030J14Rik|66289	-1	#DIV/0!	1	#DIV/0!
Sohlh2|74434	-1	#DIV/0!	1	#DIV/0!
Mbd3l2|234988	-1	#DIV/0!	1	#DIV/0!
AA545190|57866	-1	#DIV/0!	1	#DIV/0!
Crmp1|12933	-1	#DIV/0!	1	#DIV/0!
Gm14525|100039120	-1	#DIV/0!	1	#DIV/0!
Cacng4|54377	-1	#DIV/0!	1	#DIV/0!
Mir31|723895	-1	#DIV/0!	1	#DIV/0!
Vmn1r14|113864	-1	#DIV/0!	1	#DIV/0!
Vmn1r148|81011	-1	#DIV/0!	1	#DIV/0!
Usp9y|107868	-1	#DIV/0!	1	#DIV/0!
Cnga3|12790	-1	#DIV/0!	1	#DIV/0!
Olfr679|259046	-1	#DIV/0!	1	#DIV/0!
Rxfp4|242093	-1	#DIV/0!	1	#DIV/0!
Accn4|241118	-1	#DIV/0!	1	#DIV/0!
Npy2r|18167	-1	#DIV/0!	1	#DIV/0!
Pax6os1|402728	-1	#DIV/0!	1	#DIV/0!
Il6|16193	-1	#DIV/0!	1	#DIV/0!
Dlx3|13393	-1	#DIV/0!	1	#DIV/0!
Olfr1415|258228	-1	#DIV/0!	1	#DIV/0!
Snord83b|100302601	-1	#DIV/0!	1	#DIV/0!
Gm11435|629303	-1	#DIV/0!	1	#DIV/0!
Tnni1|21952	-1	#DIV/0!	1	#DIV/0!
Gm5935|546282	-1	#DIV/0!	1	#DIV/0!
4930547N16Rik|75317	-1	#DIV/0!	1	#DIV/0!
Hist1h3e|319151	-1	#DIV/0!	1	#DIV/0!
Olfr1328|258394	-1	#DIV/0!	1	#DIV/0!
Gm16523|100042584	-1	#DIV/0!	1	#DIV/0!
Vstm2l|277432	-1	#DIV/0!	1	#DIV/0!
Olfr444|258650	-1	#DIV/0!	1	#DIV/0!
Ms4a5|269063	-1	#DIV/0!	1	#DIV/0!
Gm5635|434729	-1	#DIV/0!	1	#DIV/0!
Ntf5|78405	-1	#DIV/0!	1	#DIV/0!
Trat1|77647	-1	#DIV/0!	1	#DIV/0!
Gm10375|100042342	-1	#DIV/0!	1	#DIV/0!
Adam7|11500	-1	#DIV/0!	1	#DIV/0!
Gas2l2|237891	-1	#DIV/0!	1	#DIV/0!
1700128F08Rik|76669	-1	#DIV/0!	1	#DIV/0!
Daf2|13137	-1	#DIV/0!	1	#DIV/0!
Olfr112|258096	-1	#DIV/0!	1	#DIV/0!
Snord15a|449630	-1	#DIV/0!	1	#DIV/0!
Nlrp9b|243874	-1	#DIV/0!	1	#DIV/0!
Iqsec3|243621	-1	#DIV/0!	1	#DIV/0!
Dbc1|56710	-1	#DIV/0!	1	#DIV/0!
Olfr328|258495	-1	#DIV/0!	1	#DIV/0!
Vmn1r124|670764	-1	#DIV/0!	1	#DIV/0!
Arhgef38|77669	-1	#DIV/0!	1	#DIV/0!
Clcn1|12723	-1	#DIV/0!	1	#DIV/0!
Maneal|215090	-1	#DIV/0!	1	#DIV/0!
Gm9112|668339	-1	#DIV/0!	1	#DIV/0!
Olfr294|257904	-1	#DIV/0!	1	#DIV/0!

Olfr971|258607	-1	#DIV/0!	1	#DIV/0!
Olfr1082|404473	-1	#DIV/0!	1	#DIV/0!
Mir3107|100526510	-1	#DIV/0!	1	#DIV/0!
Psg17|26437	-1	#DIV/0!	1	#DIV/0!
Hist2h2ab|621893	-1	#DIV/0!	1	#DIV/0!
Nkx2-9|18094	-1	#DIV/0!	1	#DIV/0!
Ppyr1|19065	-1	#DIV/0!	1	#DIV/0!
Vmn2r31|100042591	-1	#DIV/0!	1	#DIV/0!
Vmn1r42|113848	-1	#DIV/0!	1	#DIV/0!
Gm10731|100039043	-1	#DIV/0!	1	#DIV/0!
Obox3|246791	-1	#DIV/0!	1	#DIV/0!
Il23r|209590	-1	#DIV/0!	1	#DIV/0!
Klhl31|244923	-1	#DIV/0!	1	#DIV/0!
C330005M16Rik|101744	-1	#DIV/0!	1	#DIV/0!
Olfr148|258498	-1	#DIV/0!	1	#DIV/0!
Mir3085|100526549	-1	#DIV/0!	1	#DIV/0!
A430089I19Rik|331195	-1	#DIV/0!	1	#DIV/0!
Olfr367-ps|545417	-1	#DIV/0!	1	#DIV/0!
Rho|212541	-1	#DIV/0!	1	#DIV/0!
Tekt1|21689	-1	#DIV/0!	1	#DIV/0!
Olfr652|259050	-1	#DIV/0!	1	#DIV/0!
Ampd1|229665	-1	#DIV/0!	1	#DIV/0!
Gm15299|100041811	-1	#DIV/0!	1	#DIV/0!
Gm128|229588	-1	#DIV/0!	1	#DIV/0!
1500009L16Rik|69784	-1	#DIV/0!	1	#DIV/0!
Olfr135|258329	-1	#DIV/0!	1	#DIV/0!
Gabra1|14394	-1	#DIV/0!	1	#DIV/0!
Olfr52|18352	-1	#DIV/0!	1	#DIV/0!
Olfr693|258445	-1	#DIV/0!	1	#DIV/0!
Mir411|723936	-1	#DIV/0!	1	#DIV/0!
Vmn2r6|667069	-1	#DIV/0!	1	#DIV/0!
Phactr3|74189	-1	#DIV/0!	1	#DIV/0!
Tcfl5|277353	-1	#DIV/0!	1	#DIV/0!
1700020A23Rik|75656	-1	#DIV/0!	1	#DIV/0!
Omt2a|18379	-1	#DIV/0!	1	#DIV/0!
Defb19|246700	-1	#DIV/0!	1	#DIV/0!
Mir302b|723948	-1	#DIV/0!	1	#DIV/0!
Mir148b|724064	-1	#DIV/0!	1	#DIV/0!
2900092C05Rik|73090	-1	#DIV/0!	1	#DIV/0!
Olfr176|258995	-1	#DIV/0!	1	#DIV/0!
Igll1|16136	-1	#DIV/0!	1	#DIV/0!
Ascl1|17172	-1	#DIV/0!	1	#DIV/0!
Olfr900|258874	-1	#DIV/0!	1	#DIV/0!
Pcp4|18546	-1	#DIV/0!	1	#DIV/0!
Oxt|18429	-1	#DIV/0!	1	#DIV/0!
Gm648|270599	-1	#DIV/0!	1	#DIV/0!
Olfr165|258458	-1	#DIV/0!	1	#DIV/0!
Olfr998|258428	-1	#DIV/0!	1	#DIV/0!
Gm5156|381936	-1	#DIV/0!	1	#DIV/0!
Ifna4|15967	-1	#DIV/0!	1	#DIV/0!
Olfr1110|258765	-1	#DIV/0!	1	#DIV/0!
Usp17l5|13532	-1	#DIV/0!	1	#DIV/0!
Cpvl|71287	-1	#DIV/0!	1	#DIV/0!

Gm13057|100040861	-1	#DIV/0!	1	#DIV/0!
Neurod4|11923	-1	#DIV/0!	1	#DIV/0!
Mir876|100124458	-1	#DIV/0!	1	#DIV/0!
Astl|215095	-1	#DIV/0!	1	#DIV/0!
Vmn1r213|171249	-1	#DIV/0!	1	#DIV/0!
Krtap26-1|69533	-1	#DIV/0!	1	#DIV/0!
Rhox4f|636177	-1	#DIV/0!	1	#DIV/0!
Scrt2|545474	-1	#DIV/0!	1	#DIV/0!
Trim29|72169	-1	#DIV/0!	1	#DIV/0!
A230057D06Rik|319893	-1	#DIV/0!	1	#DIV/0!
Moxd2|194357	-1	#DIV/0!	1	#DIV/0!
Ear7|53873	-1	#DIV/0!	1	#DIV/0!
Olfr1130|258835	-1	#DIV/0!	1	#DIV/0!
Ear11|93726	-1	#DIV/0!	1	#DIV/0!
Mir143|387161	-1	#DIV/0!	1	#DIV/0!
Reg3b|18489	-1	#DIV/0!	1	#DIV/0!
Olfr1013|258757	-1	#DIV/0!	1	#DIV/0!
Gm16386|100042679	-1	#DIV/0!	1	#DIV/0!
BC052688|432812	-1	#DIV/0!	1	#DIV/0!
Snord70|100217459	-1	#DIV/0!	1	#DIV/0!
Xkr5|319581	-1	#DIV/0!	1	#DIV/0!
1700092M07Rik|74307	-1	#DIV/0!	1	#DIV/0!
Cpa4|71791	-1	#DIV/0!	1	#DIV/0!
Hist1h3a|360198	-1	#DIV/0!	1	#DIV/0!
Opcml|330908	-1	#DIV/0!	1	#DIV/0!
Spaca7|78634	-1	#DIV/0!	1	#DIV/0!
Olfr1510|258423	-1	#DIV/0!	1	#DIV/0!
Bcan|12032	-1	#DIV/0!	1	#DIV/0!
1700020C07Rik|75642	-1	#DIV/0!	1	#DIV/0!
Dpp10|269109	-1	#DIV/0!	1	#DIV/0!
Olfr389|259011	-1	#DIV/0!	1	#DIV/0!
Gjb4|14621	-1	#DIV/0!	1	#DIV/0!
Ccdc150|78016	-1	#DIV/0!	1	#DIV/0!
5930412G12Rik|319616	-1	#DIV/0!	1	#DIV/0!
Tlx2|21909	-1	#DIV/0!	1	#DIV/0!
Mir1196|100316771	-1	#DIV/0!	1	#DIV/0!
Vmn2r19|232358	-1	#DIV/0!	1	#DIV/0!
Pgbd5|209966	-1	#DIV/0!	1	#DIV/0!
Glb1l3|70893	-1	#DIV/0!	1	#DIV/0!
Adamts17|233332	-1	#DIV/0!	1	#DIV/0!
Vmn1r58|81014	-1	#DIV/0!	1	#DIV/0!
Sel1l2|228684	-1	#DIV/0!	1	#DIV/0!
Scn5a|20271	-1	#DIV/0!	1	#DIV/0!
Ankrd36|76389	-1	#DIV/0!	1	#DIV/0!
Spint4|78239	-1	#DIV/0!	1	#DIV/0!
Olfr195|259000	-1	#DIV/0!	1	#DIV/0!
Mir375|723900	-1	#DIV/0!	1	#DIV/0!
Ube2dnl|237009	-1	#DIV/0!	1	#DIV/0!
Mir126|387145	-1	#DIV/0!	1	#DIV/0!
Mir122a|387231	-1	#DIV/0!	1	#DIV/0!
Olfr618|259049	-1	#DIV/0!	1	#DIV/0!
Olfr671|257910	-1	#DIV/0!	1	#DIV/0!
Cryba1|12957	-1	#DIV/0!	1	#DIV/0!

4930547C10Rik|68274	-1	#DIV/0!	1	#DIV/0!
Mir2136|100316725	-1	#DIV/0!	1	#DIV/0!
Hoxb13|15408	-1	#DIV/0!	1	#DIV/0!
Taar7a|215856	-1	#DIV/0!	1	#DIV/0!
Cma2|545055	-1	#DIV/0!	1	#DIV/0!
Sftpb|20388	-1	#DIV/0!	1	#DIV/0!
Has1|15116	-1	#DIV/0!	1	#DIV/0!
Gm4832|225058	-1	#DIV/0!	1	#DIV/0!
2610018G03Rik|70415	-1	#DIV/0!	1	#DIV/0!
Gm17359|100233207	-1	#DIV/0!	1	#DIV/0!
Mir1933|100316753	-1	#DIV/0!	1	#DIV/0!
Mir467a-2|100526522	-1	#DIV/0!	1	#DIV/0!
A730020M07Rik|100503044	-1	#DIV/0!	1	#DIV/0!
Slitrk4|245446	-1	#DIV/0!	1	#DIV/0!
Prok1|246691	-1	#DIV/0!	1	#DIV/0!
1700008P20Rik|69301	-1	#DIV/0!	1	#DIV/0!
Olfr399|259006	-1	#DIV/0!	1	#DIV/0!
Vmn2r85|623734	-1	#DIV/0!	1	#DIV/0!
Gm12769|100043860	-1	#DIV/0!	1	#DIV/0!
Btn1a1|12231	-1	#DIV/0!	1	#DIV/0!
Ivl|16447	-1	#DIV/0!	1	#DIV/0!
Olfr146|258742	-1	#DIV/0!	1	#DIV/0!
Astn1|11899	-1	#DIV/0!	1	#DIV/0!
Nlrp4e|446099	-1	#DIV/0!	1	#DIV/0!
Vmn1r1|625823	-1	#DIV/0!	1	#DIV/0!
Olfr101|258831	-1	#DIV/0!	1	#DIV/0!
Ces2b|234669	-1	#DIV/0!	1	#DIV/0!
Rhox4d|664610	-1	#DIV/0!	1	#DIV/0!
1700019A02Rik|69397	-1	#DIV/0!	1	#DIV/0!
H2-T3|15043	-1	#DIV/0!	1	#DIV/0!
Vmn1r135|667404	-1	#DIV/0!	1	#DIV/0!
Mir3077|100526476	-1	#DIV/0!	1	#DIV/0!
Fcrl5|329693	-1	#DIV/0!	1	#DIV/0!
Olfr1036|258245	-1	#DIV/0!	1	#DIV/0!
Krt34|16672	-1	#DIV/0!	1	#DIV/0!
L3mbtl4|320858	-1	#DIV/0!	1	#DIV/0!
BB031773|100473	-1	#DIV/0!	1	#DIV/0!
Kcnu1|16532	-1	#DIV/0!	1	#DIV/0!
Rprml|104582	-1	#DIV/0!	1	#DIV/0!
Olfr1489|258628	-1	#DIV/0!	1	#DIV/0!
Gm5531|433365	-1	#DIV/0!	1	#DIV/0!
Rab9b|319642	-1	#DIV/0!	1	#DIV/0!
Gm12597|242517	-1	#DIV/0!	1	#DIV/0!
Fam154a|75811	-1	#DIV/0!	1	#DIV/0!
Mirlet7c-2|723966	-1	#DIV/0!	1	#DIV/0!
Olfr924|404322	-1	#DIV/0!	1	#DIV/0!
Mir1961|100316841	-1	#DIV/0!	1	#DIV/0!
Mir20a|387139	-1	#DIV/0!	1	#DIV/0!
Lmx1a|110648	-1	#DIV/0!	1	#DIV/0!
Greb1|268527	-1	#DIV/0!	1	#DIV/0!
Slc22a19|207151	-1	#DIV/0!	1	#DIV/0!
Ccdc79|320022	-1	#DIV/0!	1	#DIV/0!
Olfr1112|258655	-1	#DIV/0!	1	#DIV/0!

Pde6b|18587	-1	#DIV/0!	1	#DIV/0!
Rab33a|19337	-1	#DIV/0!	1	#DIV/0!
2610034M16Rik|69239	-1	#DIV/0!	1	#DIV/0!
Zscan4c|245109	-1	#DIV/0!	1	#DIV/0!
Aym1|503692	-1	#DIV/0!	1	#DIV/0!
Olfr1277|258391	-1	#DIV/0!	1	#DIV/0!
Mir539|723917	-1	#DIV/0!	1	#DIV/0!
Dnmt3l|54427	-1	#DIV/0!	1	#DIV/0!
Lrrtm1|74342	-1	#DIV/0!	1	#DIV/0!
Syt16|238266	-1	#DIV/0!	1	#DIV/0!
Spink10|328971	-1	#DIV/0!	1	#DIV/0!
Ptn|19242	-1	#DIV/0!	1	#DIV/0!
Tm4sf20|66261	-1	#DIV/0!	1	#DIV/0!
Mir15b|387175	-1	#DIV/0!	1	#DIV/0!
Foxr1|382074	-1	#DIV/0!	1	#DIV/0!
Clec18a|353287	-1	#DIV/0!	1	#DIV/0!
Tdrd12|71981	-1	#DIV/0!	1	#DIV/0!
Gm15070|100038527	-1	#DIV/0!	1	#DIV/0!
Lgi1|56839	-1	#DIV/0!	1	#DIV/0!
Kcnj6|16522	-1	#DIV/0!	1	#DIV/0!
Mir503|723879	-1	#DIV/0!	1	#DIV/0!
Gabrr1|14408	-1	#DIV/0!	1	#DIV/0!
Gabrr3|328699	-1	#DIV/0!	1	#DIV/0!
Magea2|17138	-1	#DIV/0!	1	#DIV/0!
Olfr891|258471	-1	#DIV/0!	1	#DIV/0!
Hmx1|15371	-1	#DIV/0!	1	#DIV/0!
B020004J07Rik|545662	-1	#DIV/0!	1	#DIV/0!
Dscc1|72107	-1	#DIV/0!	1	#DIV/0!
Ccdc108|241116	-1	#DIV/0!	1	#DIV/0!
Mir669b|735255	-1	#DIV/0!	1	#DIV/0!
Mir344b|100526558	-1	#DIV/0!	1	#DIV/0!
Pappa|18491	-1	#DIV/0!	1	#DIV/0!
Slc34a3|142681	-1	#DIV/0!	1	#DIV/0!
Olfr402|258703	-1	#DIV/0!	1	#DIV/0!
Nos1|18125	-1	#DIV/0!	1	#DIV/0!
Pmp2|18857	-1	#DIV/0!	1	#DIV/0!
Olfr884|257996	-1	#DIV/0!	1	#DIV/0!
Trpc5|22067	-1	#DIV/0!	1	#DIV/0!
Gm4461|100043474	-1	#DIV/0!	1	#DIV/0!
4930488L21Rik|75809	-1	#DIV/0!	1	#DIV/0!
Clec4e|56619	-1	#DIV/0!	1	#DIV/0!
3110082D06Rik|627626	-1	#DIV/0!	1	#DIV/0!
Ly6f|17071	-1	#DIV/0!	1	#DIV/0!
Gm4307|100043239	-1	#DIV/0!	1	#DIV/0!
Ceacam14|67084	-1	#DIV/0!	1	#DIV/0!
Mir882|100124461	-1	#DIV/0!	1	#DIV/0!
Gm2825|100040533	-1	#DIV/0!	1	#DIV/0!
Syt2|20980	-1	#DIV/0!	1	#DIV/0!
1700049E15Rik|100039727	-1	#DIV/0!	1	#DIV/0!
Scarna6|100217415	-1	#DIV/0!	1	#DIV/0!
Prame|75829	-1	#DIV/0!	1	#DIV/0!
Mir25|723926	-1	#DIV/0!	1	#DIV/0!
Slx|664829	-1	#DIV/0!	1	#DIV/0!

Rbm44|329207	-1	#DIV/0!	1	#DIV/0!
Olfr187|258319	-1	#DIV/0!	1	#DIV/0!
Mir27a|387220	-1	#DIV/0!	1	#DIV/0!
Mir3065|100526518	-1	#DIV/0!	1	#DIV/0!
Olfr868|258552	-1	#DIV/0!	1	#DIV/0!
Taar7b|209517	-1	#DIV/0!	1	#DIV/0!
Krt31|16660	-1	#DIV/0!	1	#DIV/0!
Ear1|13586	-1	#DIV/0!	1	#DIV/0!
Vmn1r207-ps|432735	-1	#DIV/0!	1	#DIV/0!
Six3|20473	-1	#DIV/0!	1	#DIV/0!
Mir878|100124479	-1	#DIV/0!	1	#DIV/0!
Prl8a2|13529	-1	#DIV/0!	1	#DIV/0!
Mir467b|735257	-1	#DIV/0!	1	#DIV/0!
Bves|23828	-1	#DIV/0!	1	#DIV/0!
Cyp4a29-ps|230639	-1	#DIV/0!	1	#DIV/0!
D5Ertd577e|320549	-1	#DIV/0!	1	#DIV/0!
Gm5622|434459	-1	#DIV/0!	1	#DIV/0!
Olfr623|259126	-1	#DIV/0!	1	#DIV/0!
Defa26|626708	-1	#DIV/0!	1	#DIV/0!
Speer5-ps1|70365	-1	#DIV/0!	1	#DIV/0!
Olfr1356|258305	-1	#DIV/0!	1	#DIV/0!
Gramd2|546134	-1	#DIV/0!	1	#DIV/0!
Tnfsf4|22164	-1	#DIV/0!	1	#DIV/0!
Sprr1a|20753	-1	#DIV/0!	1	#DIV/0!
Olfr64|18366	-1	#DIV/0!	1	#DIV/0!
Scrn1|69938	-1	#DIV/0!	1	#DIV/0!
2300005B03Rik|69462	-1	#DIV/0!	1	#DIV/0!
Olfr1053|257974	-1	#DIV/0!	1	#DIV/0!
Vmn1r158|100043067	-1	#DIV/0!	1	#DIV/0!
Snora26|100313943	-1	#DIV/0!	1	#DIV/0!
Krt2|16681	-1	#DIV/0!	1	#DIV/0!
Ccna1|12427	-1	#DIV/0!	1	#DIV/0!
Gm5894|545947	-1	#DIV/0!	1	#DIV/0!
Olfr1129|258111	-1	#DIV/0!	1	#DIV/0!
Gng4|14706	-1	#DIV/0!	1	#DIV/0!
Mir182|387177	-1	#DIV/0!	1	#DIV/0!
Olfr846|258279	-1	#DIV/0!	1	#DIV/0!
Eya4|14051	-1	#DIV/0!	1	#DIV/0!
Taar4|209513	-1	#DIV/0!	1	#DIV/0!
Gm597|210962	-1	#DIV/0!	1	#DIV/0!
Olfr517|258136	-1	#DIV/0!	1	#DIV/0!
Tktl2|74419	-1	#DIV/0!	1	#DIV/0!
Sv2c|75209	-1	#DIV/0!	1	#DIV/0!
Olfr710|258594	-1	#DIV/0!	1	#DIV/0!
Cryga|12964	-1	#DIV/0!	1	#DIV/0!
Vmn1r212|171275	-1	#DIV/0!	1	#DIV/0!
Defb48|432867	-1	#DIV/0!	1	#DIV/0!
Olfr646|259058	-1	#DIV/0!	1	#DIV/0!
Iqcf4|67320	-1	#DIV/0!	1	#DIV/0!
Slc24a2|76376	-1	#DIV/0!	1	#DIV/0!
Gm5166|382233	-1	#DIV/0!	1	#DIV/0!
Tsks|22116	-1	#DIV/0!	1	#DIV/0!
Dmrt3|240590	-1	#DIV/0!	1	#DIV/0!

Olfr117|258263	-1	#DIV/0!	1	#DIV/0!
Foxe1|110805	-1	#DIV/0!	1	#DIV/0!
Olfr1252|404331	-1	#DIV/0!	1	#DIV/0!
Mir1264|100526460	-1	#DIV/0!	1	#DIV/0!
Vmn1r66|171264	-1	#DIV/0!	1	#DIV/0!
Upk2|22269	-1	#DIV/0!	1	#DIV/0!
Enthd1|383075	-1	#DIV/0!	1	#DIV/0!
Olfr1474|258123	-1	#DIV/0!	1	#DIV/0!
Mir17|723905	-1	#DIV/0!	1	#DIV/0!
Grm6|108072	-1	#DIV/0!	1	#DIV/0!
4931417E11Rik|66740	-1	#DIV/0!	1	#DIV/0!
Olfr576|258248	-1	#DIV/0!	1	#DIV/0!
Mir346|723847	-1	#DIV/0!	1	#DIV/0!
S100a7a|381493	-1	#DIV/0!	1	#DIV/0!
Olfr149|235256	-1	#DIV/0!	1	#DIV/0!
Slc7a9|30962	-1	#DIV/0!	1	#DIV/0!
Gm10406|100038847	-1	#DIV/0!	1	#DIV/0!
Dub1a|381944	-1	#DIV/0!	1	#DIV/0!
Six3os1|100043902	-1	#DIV/0!	1	#DIV/0!
Grm1|14816	-1	#DIV/0!	1	#DIV/0!
Cep55|74107	-1	#DIV/0!	1	#DIV/0!
Nlrp5|23968	-1	#DIV/0!	1	#DIV/0!
Depdc1b|218581	-1	#DIV/0!	1	#DIV/0!
Klk5|68668	-1	#DIV/0!	1	#DIV/0!
Atp4b|11945	-1	#DIV/0!	1	#DIV/0!
1700113H08Rik|76640	-1	#DIV/0!	1	#DIV/0!
Olfr799|258929	-1	#DIV/0!	1	#DIV/0!
Rhox3-ps|546295	-1	#DIV/0!	1	#DIV/0!
1700009N14Rik|75471	-1	#DIV/0!	1	#DIV/0!
Snord89|100217461	-1	#DIV/0!	1	#DIV/0!
Mir3103|100526559	-1	#DIV/0!	1	#DIV/0!
Msx2|17702	-1	#DIV/0!	1	#DIV/0!
Clps|109791	-1	#DIV/0!	1	#DIV/0!
Th|21823	-1	#DIV/0!	1	#DIV/0!
Olfr790|258935	-1	#DIV/0!	1	#DIV/0!
Olfr596|100041187	-1	#DIV/0!	1	#DIV/0!
Fbxw18|546161	-1	#DIV/0!	1	#DIV/0!
Fbxw15|382105	-1	#DIV/0!	1	#DIV/0!
Cdk15|271697	-1	#DIV/0!	1	#DIV/0!
Olfr1305|258396	-1	#DIV/0!	1	#DIV/0!
Snora62|104433	-1	#DIV/0!	1	#DIV/0!
Doxl2|243376	-1	#DIV/0!	1	#DIV/0!
Gm13871|667780	-1	#DIV/0!	1	#DIV/0!
Sox2ot|320478	-1	#DIV/0!	1	#DIV/0!
Olfr1023|258580	-1	#DIV/0!	1	#DIV/0!
Mir485|723875	-1	#DIV/0!	1	#DIV/0!
Tshb|22094	-1	#DIV/0!	1	#DIV/0!
Gm5878|545861	-1	#DIV/0!	1	#DIV/0!
Gm9733|751864	-1	#DIV/0!	1	#DIV/0!
Gm10471|100039045	-1	#DIV/0!	1	#DIV/0!
Olfr476|258926	-1	#DIV/0!	1	#DIV/0!
Celf6|76183	-1	#DIV/0!	1	#DIV/0!
Lhfpl1|237091	-1	#DIV/0!	1	#DIV/0!

Gpr26|233919	-1	#DIV/0!	1	#DIV/0!
Cyp27b1|13115	-1	#DIV/0!	1	#DIV/0!
Ccdc36|434438	-1	#DIV/0!	1	#DIV/0!
Kcnc2|268345	-1	#DIV/0!	1	#DIV/0!
Pabpc5|93728	-1	#DIV/0!	1	#DIV/0!
Olfr675|258147	-1	#DIV/0!	1	#DIV/0!
Nhs|195727	-1	#DIV/0!	1	#DIV/0!
1700049L16Rik|108950	-1	#DIV/0!	1	#DIV/0!
1700029F12Rik|66479	-1	#DIV/0!	1	#DIV/0!
Amy2a5|109959	-1	#DIV/0!	1	#DIV/0!
Olfr44|18343	-1	#DIV/0!	1	#DIV/0!
Gm5941|546347	-1	#DIV/0!	1	#DIV/0!
Elavl4|15572	-1	#DIV/0!	1	#DIV/0!
6330407A03Rik|70720	-1	#DIV/0!	1	#DIV/0!
Mir24-2|723960	-1	#DIV/0!	1	#DIV/0!
Zfy1|22767	-1	#DIV/0!	1	#DIV/0!
Slc35f4|75288	-1	#DIV/0!	1	#DIV/0!
Olfr568|259095	-1	#DIV/0!	1	#DIV/0!
Gm6034|547347	-1	#DIV/0!	1	#DIV/0!
Gm5938|546335	-1	#DIV/0!	1	#DIV/0!
Krtap4-6|68768	-1	#DIV/0!	1	#DIV/0!
Olfr160|80706	-1	#DIV/0!	1	#DIV/0!
Pou3f3|18993	-1	#DIV/0!	1	#DIV/0!
Rbp2|19660	-1	#DIV/0!	1	#DIV/0!
Gm9268|668612	-1	#DIV/0!	1	#DIV/0!
Olfr600|259048	-1	#DIV/0!	1	#DIV/0!
Zim3|116811	-1	#DIV/0!	1	#DIV/0!
St8sia5|225742	-1	#DIV/0!	1	#DIV/0!
Npw|381073	-1	#DIV/0!	1	#DIV/0!
Gm15217|100041724	-1	#DIV/0!	1	#DIV/0!
Pom121l2|195236	-1	#DIV/0!	1	#DIV/0!
Olfr109|258832	-1	#DIV/0!	1	#DIV/0!
Gm9524|671232	-1	#DIV/0!	1	#DIV/0!
Obp1a|18249	-1	#DIV/0!	1	#DIV/0!
Olfr1265|258340	-1	#DIV/0!	1	#DIV/0!
Olfr1094|258362	-1	#DIV/0!	1	#DIV/0!
Olfr975|258825	-1	#DIV/0!	1	#DIV/0!
Mir181a-1|735252	-1	#DIV/0!	1	#DIV/0!
E330013P04Rik|107376	-1	#DIV/0!	1	#DIV/0!
Birc7|329581	-1	#DIV/0!	1	#DIV/0!
Olfr1278|258389	-1	#DIV/0!	1	#DIV/0!
Olfr1320|236784	-1	#DIV/0!	1	#DIV/0!
Sult2b1|54200	-1	#DIV/0!	1	#DIV/0!
Speer8-ps1|74062	-1	#DIV/0!	1	#DIV/0!
Acvr1c|269275	-1	#DIV/0!	1	#DIV/0!
Cd70|21948	-1	#DIV/0!	1	#DIV/0!
Taar9|503558	-1	#DIV/0!	1	#DIV/0!
Il31ra|218624	-1	#DIV/0!	1	#DIV/0!
Csn1s1|12990	-1	#DIV/0!	1	#DIV/0!
4921528O07Rik|78258	-1	#DIV/0!	1	#DIV/0!
Syngr3|20974	-1	#DIV/0!	1	#DIV/0!
Mmp21|214766	-1	#DIV/0!	1	#DIV/0!
Mir7-2|723884	-1	#DIV/0!	1	#DIV/0!

Ms4a3|170813	-1	#DIV/0!	1	#DIV/0!
Olfr1394|258273	-1	#DIV/0!	1	#DIV/0!
Olfr1309|258439	-1	#DIV/0!	1	#DIV/0!
Slco6c1|74441	-1	#DIV/0!	1	#DIV/0!
Clca4|229927	-1	#DIV/0!	1	#DIV/0!
Vmn1r227|171229	-1	#DIV/0!	1	#DIV/0!
Olfr262|258683	-1	#DIV/0!	1	#DIV/0!
Mir186|387181	-1	#DIV/0!	1	#DIV/0!
Mgat4c|67569	-1	#DIV/0!	1	#DIV/0!
C86187|97402	-1	#DIV/0!	1	#DIV/0!
Olfr792|258150	-1	#DIV/0!	1	#DIV/0!
Mir326|723840	-1	#DIV/0!	1	#DIV/0!
Olfr33|18332	-1	#DIV/0!	1	#DIV/0!
Olfr913|258225	-1	#DIV/0!	1	#DIV/0!
Skint5|242627	-1	#DIV/0!	1	#DIV/0!
Fgf5|14176	-1	#DIV/0!	1	#DIV/0!
Klk1b21|16616	-1	#DIV/0!	1	#DIV/0!
Olfr26|18324	-1	#DIV/0!	1	#DIV/0!
Gpr158|241263	-1	#DIV/0!	1	#DIV/0!
Olfr547|259083	-1	#DIV/0!	1	#DIV/0!
Ifna14|404549	-1	#DIV/0!	1	#DIV/0!
Crygf|12969	-1	#DIV/0!	1	#DIV/0!
Fpr3|14294	-1	#DIV/0!	1	#DIV/0!
Olfr1208|258774	-1	#DIV/0!	1	#DIV/0!
Itgb1bp3|69564	-1	#DIV/0!	1	#DIV/0!
Vmn2r92|627111	-1	#DIV/0!	1	#DIV/0!
Lhx1|16869	-1	#DIV/0!	1	#DIV/0!
Nppc|18159	-1	#DIV/0!	1	#DIV/0!
Olfr303|258612	-1	#DIV/0!	1	#DIV/0!
Tgm5|74176	-1	#DIV/0!	1	#DIV/0!
Cyp2j11|100066	-1	#DIV/0!	1	#DIV/0!
Mtag2|50994	-1	#DIV/0!	1	#DIV/0!
1700019M22Rik|69423	-1	#DIV/0!	1	#DIV/0!
Ppp1r14d|72112	-1	#DIV/0!	1	#DIV/0!
Ferd3l|114712	-1	#DIV/0!	1	#DIV/0!
Ceacam20|71601	-1	#DIV/0!	1	#DIV/0!
Nlrp4f|97895	-1	#DIV/0!	1	#DIV/0!
9330158H04Rik|319472	-1	#DIV/0!	1	#DIV/0!
Fam184b|58227	-1	#DIV/0!	1	#DIV/0!
Tmem28|620592	-1	#DIV/0!	1	#DIV/0!
Gm2663|100040208	-1	#DIV/0!	1	#DIV/0!
Trim66|330627	-1	#DIV/0!	1	#DIV/0!
Olfr980|259110	-1	#DIV/0!	1	#DIV/0!
Slc14a2|27411	-1	#DIV/0!	1	#DIV/0!
Olfr1104|258763	-1	#DIV/0!	1	#DIV/0!
Cxcr1|227288	-1	#DIV/0!	1	#DIV/0!
Reg1|19692	-1	#DIV/0!	1	#DIV/0!
4931428L18Rik|70988	-1	#DIV/0!	1	#DIV/0!
Fgf17|14171	-1	#DIV/0!	1	#DIV/0!
Cypt14|664704	-1	#DIV/0!	1	#DIV/0!
Mir3083|100526534	-1	#DIV/0!	1	#DIV/0!
Vmn1r176|546943	-1	#DIV/0!	1	#DIV/0!
Mir26a-1|387218	-1	#DIV/0!	1	#DIV/0!

D6Ertd474e|52285	-1	#DIV/0!	1	#DIV/0!
Vmn2r52|384534	-1	#DIV/0!	1	#DIV/0!
Olfr113|258286	-1	#DIV/0!	1	#DIV/0!
Actl7a|11470	-1	#DIV/0!	1	#DIV/0!
Mpped1|223726	-1	#DIV/0!	1	#DIV/0!
Krtap6-2|16701	-1	#DIV/0!	1	#DIV/0!
Dcdc2b|667452	-1	#DIV/0!	1	#DIV/0!
Smok2a|27263	-1	#DIV/0!	1	#DIV/0!
S100a3|20197	-1	#DIV/0!	1	#DIV/0!
Mir1967|100316819	-1	#DIV/0!	1	#DIV/0!
Dpy19l2|320752	-1	#DIV/0!	1	#DIV/0!
Ang2|11731	-1	#DIV/0!	1	#DIV/0!
Abhd16b|241850	-1	#DIV/0!	1	#DIV/0!
Abpa|11354	-1	#DIV/0!	1	#DIV/0!
Olfr1079|258402	-1	#DIV/0!	1	#DIV/0!
Reg3d|30053	-1	#DIV/0!	1	#DIV/0!
Gm5065|272350	-1	#DIV/0!	1	#DIV/0!
4933406I18Rik|71045	-1	#DIV/0!	1	#DIV/0!
Olfr1032|258572	-1	#DIV/0!	1	#DIV/0!
Arntl2|272322	-1	#DIV/0!	1	#DIV/0!
Vmn2r13|231589	-1	#DIV/0!	1	#DIV/0!
AA619741|241051	-1	#DIV/0!	1	#DIV/0!
Pcdhb19|93890	-1	#DIV/0!	1	#DIV/0!
Krtap5-2|71623	-1	#DIV/0!	1	#DIV/0!
Olfr361|258365	-1	#DIV/0!	1	#DIV/0!
Il1f5|54450	-1	#DIV/0!	1	#DIV/0!
Scel|64929	-1	#DIV/0!	1	#DIV/0!
Hist1h3i|319153	-1	#DIV/0!	1	#DIV/0!
Mir676|751534	-1	#DIV/0!	1	#DIV/0!
Mir30b|387226	-1	#DIV/0!	1	#DIV/0!
1700007B14Rik|71831	-1	#DIV/0!	1	#DIV/0!
Zfp458|238690	-1	#DIV/0!	1	#DIV/0!
Tmprss3|140765	-1	#DIV/0!	1	#DIV/0!
Ankrd45|73844	-1	#DIV/0!	1	#DIV/0!
Vmn1r75|171241	-1	#DIV/0!	1	#DIV/0!
Cypt4|235067	-1	#DIV/0!	1	#DIV/0!
Nkx2-2|18088	-1	#DIV/0!	1	#DIV/0!
Mir374|751546	-1	#DIV/0!	1	#DIV/0!
Lrr1|69706	-1	#DIV/0!	1	#DIV/0!
Olfr122|258285	-1	#DIV/0!	1	#DIV/0!
Mir759|791080	-1	#DIV/0!	1	#DIV/0!
Rp1l1|271209	-1	#DIV/0!	1	#DIV/0!
Ptcra|19208	-1	#DIV/0!	1	#DIV/0!
Cypt1|66742	-1	#DIV/0!	1	#DIV/0!
Egam-1c|100047130	-1	#DIV/0!	1	#DIV/0!
Mov10l1|83456	-1	#DIV/0!	1	#DIV/0!
Spock1|20745	-1	#DIV/0!	1	#DIV/0!
Bex6|328660	-1	#DIV/0!	1	#DIV/0!
Tas2r113|387345	-1	#DIV/0!	1	#DIV/0!
Fam155a|270028	-1	#DIV/0!	1	#DIV/0!
C130080G10Rik|100303644	-1	#DIV/0!	1	#DIV/0!
Fbxl13|320118	-1	#DIV/0!	1	#DIV/0!
D030025P21Rik|100303738	-1	#DIV/0!	1	#DIV/0!

Vmn2r9|435864	-1	#DIV/0!	1	#DIV/0!
Olfr527|257939	-1	#DIV/0!	1	#DIV/0!
9430076C15Rik|320189	-1	#DIV/0!	1	#DIV/0!
Olfr642|258326	-1	#DIV/0!	1	#DIV/0!
Mir216b|735308	-1	#DIV/0!	1	#DIV/0!
Gm4846|226601	-1	#DIV/0!	1	#DIV/0!
Olfr784|258724	-1	#DIV/0!	1	#DIV/0!
Rlbp1|19771	-1	#DIV/0!	1	#DIV/0!
Mir30e|723836	-1	#DIV/0!	1	#DIV/0!
H2afb1|68231	-1	#DIV/0!	1	#DIV/0!
Foxr2|436240	-1	#DIV/0!	1	#DIV/0!
Gucy1a2|234889	-1	#DIV/0!	1	#DIV/0!
Tmem121|69195	-1	#DIV/0!	1	#DIV/0!
9530053A07Rik|319482	-1	#DIV/0!	1	#DIV/0!
Pcdha8|353235	-1	#DIV/0!	1	#DIV/0!
Rgs6|50779	-1	#DIV/0!	1	#DIV/0!
Krt4|16682	-1	#DIV/0!	1	#DIV/0!
St18|240690	-1	#DIV/0!	1	#DIV/0!
Zkscan2|210162	-1	#DIV/0!	1	#DIV/0!
Vmn1r171|81012	-1	#DIV/0!	1	#DIV/0!
Ppp1r1c|75276	-1	#DIV/0!	1	#DIV/0!
Atoh7|53404	-1	#DIV/0!	1	#DIV/0!
Olfr1157|258846	-1	#DIV/0!	1	#DIV/0!
Erv3|71995	-1	#DIV/0!	1	#DIV/0!
Lhx5|16873	-1	#DIV/0!	1	#DIV/0!
Mnx1|15285	-1	#DIV/0!	1	#DIV/0!
Ifnb1|15977	-1	#DIV/0!	1	#DIV/0!
Gm4975|244495	-1	#DIV/0!	1	#DIV/0!
Piwil4|330890	-1	#DIV/0!	1	#DIV/0!
Pth2|114640	-1	#DIV/0!	1	#DIV/0!
Olfr1331|258159	-1	#DIV/0!	1	#DIV/0!
Mir718|751533	-1	#DIV/0!	1	#DIV/0!
Cox8c|75483	-1	#DIV/0!	1	#DIV/0!
D930020B18Rik|216393	-1	#DIV/0!	1	#DIV/0!
Dhrs7c|68460	-1	#DIV/0!	1	#DIV/0!
Cdh23|22295	-1	#DIV/0!	1	#DIV/0!
Mir1932|100316690	-1	#DIV/0!	1	#DIV/0!
Gm11595|100040276	-1	#DIV/0!	1	#DIV/0!
Scml2|107815	-1	#DIV/0!	1	#DIV/0!
Cstl1|228756	-1	#DIV/0!	1	#DIV/0!
Klk4|56640	-1	#DIV/0!	1	#DIV/0!
Olfr1230|258785	-1	#DIV/0!	1	#DIV/0!
Mir669c|735273	-1	#DIV/0!	1	#DIV/0!
E030002O03Rik|244180	-1	#DIV/0!	1	#DIV/0!
Gm5712|435755	-1	#DIV/0!	1	#DIV/0!
Gm4792|215472	-1	#DIV/0!	1	#DIV/0!
Vmn2r107|22312	-1	#DIV/0!	1	#DIV/0!
Mir1937a|100316692	-1	#DIV/0!	1	#DIV/0!
Tnn|329278	-1	#DIV/0!	1	#DIV/0!
Mir24-1|387142	-1	#DIV/0!	1	#DIV/0!
Adamts18|208936	-1	#DIV/0!	1	#DIV/0!
Sycp2|320558	-1	#DIV/0!	1	#DIV/0!
Vmn1r46|113856	-1	#DIV/0!	1	#DIV/0!

Vmn1r85|252909	-1	#DIV/0!	1	#DIV/0!
E330014E10Rik|665943	-1	#DIV/0!	1	#DIV/0!
1700034E13Rik|78414	-1	#DIV/0!	1	#DIV/0!
Lipi|320355	-1	#DIV/0!	1	#DIV/0!
Rps6ka6|67071	-1	#DIV/0!	1	#DIV/0!
Vmn1r47|113846	-1	#DIV/0!	1	#DIV/0!
Olfr513|258718	-1	#DIV/0!	1	#DIV/0!
Slitrk5|75409	-1	#DIV/0!	1	#DIV/0!
Myh1|17879	-1	#DIV/0!	1	#DIV/0!
Oxtr|18430	-1	#DIV/0!	1	#DIV/0!
4930550C14Rik|75311	-1	#DIV/0!	1	#DIV/0!
C030023E24Rik|320247	-1	#DIV/0!	1	#DIV/0!
4930451I11Rik|78118	-1	#DIV/0!	1	#DIV/0!
Slc22a22|210463	-1	#DIV/0!	1	#DIV/0!
Izumo2|75510	-1	#DIV/0!	1	#DIV/0!
Islr2|320563	-1	#DIV/0!	1	#DIV/0!
Kdm4d|244694	-1	#DIV/0!	1	#DIV/0!
1700090G07Rik|78469	-1	#DIV/0!	1	#DIV/0!
Sp9|381373	-1	#DIV/0!	1	#DIV/0!
Taar3|493809	-1	#DIV/0!	1	#DIV/0!
Gm1631|381371	-1	#DIV/0!	1	#DIV/0!
Cyp4x1|81906	-1	#DIV/0!	1	#DIV/0!
Gm5168|382275	-1	#DIV/0!	1	#DIV/0!
Pax7|18509	-1	#DIV/0!	1	#DIV/0!
Fap|14089	-1	#DIV/0!	1	#DIV/0!
Gm5334|384639	-1	#DIV/0!	1	#DIV/0!
Gm5485|433023	-1	#DIV/0!	1	#DIV/0!
Nrsn2|228777	-1	#DIV/0!	1	#DIV/0!
Olfr1153|258633	-1	#DIV/0!	1	#DIV/0!
Mir1186|100316666	-1	#DIV/0!	1	#DIV/0!
1700001K19Rik|66323	-1	#DIV/0!	1	#DIV/0!
Olfr829|259070	-1	#DIV/0!	1	#DIV/0!
Gpr141|353346	-1	#DIV/0!	1	#DIV/0!
Uts2r|217369	-1	#DIV/0!	1	#DIV/0!
Sprr2h|20762	-1	#DIV/0!	1	#DIV/0!
5830405N20Rik|67596	-1	#DIV/0!	1	#DIV/0!
Mir1900|100316681	-1	#DIV/0!	1	#DIV/0!
Vmn2r68-ps|620697	-1	#DIV/0!	1	#DIV/0!
Mir741|100049545	-1	#DIV/0!	1	#DIV/0!
Ccdc144b|241943	-1	#DIV/0!	1	#DIV/0!
Arl14|71619	-1	#DIV/0!	1	#DIV/0!
Mir1931|100316689	-1	#DIV/0!	1	#DIV/0!
Olfr1181|258060	-1	#DIV/0!	1	#DIV/0!
Zp3r|22789	-1	#DIV/0!	1	#DIV/0!
Mybph|53311	-1	#DIV/0!	1	#DIV/0!
Slc16a14|71781	-1	#DIV/0!	1	#DIV/0!
Mir3109|100526560	-1	#DIV/0!	1	#DIV/0!
1700080O16Rik|74279	-1	#DIV/0!	1	#DIV/0!
Tnp1|21958	-1	#DIV/0!	1	#DIV/0!
Gm11128|100504710	-1	#DIV/0!	1	#DIV/0!
Pcdha1|116731	-1	#DIV/0!	1	#DIV/0!
Gm44|212952	-1	#DIV/0!	1	#DIV/0!
Olfr1337|258306	-1	#DIV/0!	1	#DIV/0!

Skint4|320640	-1	#DIV/0!	1	#DIV/0!
A230073K19Rik|613263	-1	#DIV/0!	1	#DIV/0!
Vmn2r2|100125586	-1	#DIV/0!	1	#DIV/0!
Olfr1324|258289	-1	#DIV/0!	1	#DIV/0!
Gm10267|100042855	-1	#DIV/0!	1	#DIV/0!
4932425I24Rik|320214	-1	#DIV/0!	1	#DIV/0!
Pdia2|69191	-1	#DIV/0!	1	#DIV/0!
Hsfy2|71066	-1	#DIV/0!	1	#DIV/0!
Olfr770|258862	-1	#DIV/0!	1	#DIV/0!
Kcnd3|56543	-1	#DIV/0!	1	#DIV/0!
Olfr1469|258690	-1	#DIV/0!	1	#DIV/0!
Slc5a9|230612	-1	#DIV/0!	1	#DIV/0!
Olfr1499|258792	-1	#DIV/0!	1	#DIV/0!
Magea1|17137	-1	#DIV/0!	1	#DIV/0!
Vmn2r34|100042636	-1	#DIV/0!	1	#DIV/0!
Gm6927|628893	-1	#DIV/0!	1	#DIV/0!
Rhox3e|100135657	-1	#DIV/0!	1	#DIV/0!
Pcdhb6|93877	-1	#DIV/0!	1	#DIV/0!
Olfr1512|258424	-1	#DIV/0!	1	#DIV/0!
Nphs1|54631	-1	#DIV/0!	1	#DIV/0!
Krt83|406219	-1	#DIV/0!	1	#DIV/0!
Olfr1368|258527	-1	#DIV/0!	1	#DIV/0!
Vmn2r-ps54|75835	-1	#DIV/0!	1	#DIV/0!
Adam18|13524	-1	#DIV/0!	1	#DIV/0!
Olfr951|258046	-1	#DIV/0!	1	#DIV/0!
B3gat1|76898	-1	#DIV/0!	1	#DIV/0!
Olfr1178|258203	-1	#DIV/0!	1	#DIV/0!
Oas1e|231699	-1	#DIV/0!	1	#DIV/0!
Aicda|11628	-1	#DIV/0!	1	#DIV/0!
Krt85|53622	-1	#DIV/0!	1	#DIV/0!
Mir103-1|723824	-1	#DIV/0!	1	#DIV/0!
Fam46d|213449	-1	#DIV/0!	1	#DIV/0!
Mir452|723919	-1	#DIV/0!	1	#DIV/0!
Six1|20471	-1	#DIV/0!	1	#DIV/0!
Olfr765|544748	-1	#DIV/0!	1	#DIV/0!
Olfr313|258529	-1	#DIV/0!	1	#DIV/0!
4933417G07Rik|71182	-1	#DIV/0!	1	#DIV/0!
1700021F07Rik|72221	-1	#DIV/0!	1	#DIV/0!
Gm14459|546263	-1	#DIV/0!	1	#DIV/0!
Elovl4|83603	-1	#DIV/0!	1	#DIV/0!
Skint8|639774	-1	#DIV/0!	1	#DIV/0!
Olfr601|258311	-1	#DIV/0!	1	#DIV/0!
Lcn10|332578	-1	#DIV/0!	1	#DIV/0!
Pglyrp3|242100	-1	#DIV/0!	1	#DIV/0!
Mir30d|387228	-1	#DIV/0!	1	#DIV/0!
Kctd16|383348	-1	#DIV/0!	1	#DIV/0!
Kap|16483	-1	#DIV/0!	1	#DIV/0!
Vmn2r58|628422	-1	#DIV/0!	1	#DIV/0!
Clrn3|212070	-1	#DIV/0!	1	#DIV/0!
Zdhhc22|238331	-1	#DIV/0!	1	#DIV/0!
2900052N01Rik|73040	-1	#DIV/0!	1	#DIV/0!
Slc45a1|242773	-1	#DIV/0!	1	#DIV/0!
Txndc3|73412	-1	#DIV/0!	1	#DIV/0!

Iqub|214704	-1	#DIV/0!	1	#DIV/0!
1700007G11Rik|75784	-1	#DIV/0!	1	#DIV/0!
Olfr1377|258913	-1	#DIV/0!	1	#DIV/0!
Nlrp14|76858	-1	#DIV/0!	1	#DIV/0!
Hoxd9|15438	-1	#DIV/0!	1	#DIV/0!
Pitx2|18741	-1	#DIV/0!	1	#DIV/0!
Ifitm7|74482	-1	#DIV/0!	1	#DIV/0!
Mir149|387167	-1	#DIV/0!	1	#DIV/0!
Pira6|18729	-1	#DIV/0!	1	#DIV/0!
1700026D11Rik|70371	-1	#DIV/0!	1	#DIV/0!
BC055111|242602	-1	#DIV/0!	1	#DIV/0!
Ldhc|16833	-1	#DIV/0!	1	#DIV/0!
4930435E12Rik|74663	-1	#DIV/0!	1	#DIV/0!
1700106J16Rik|74297	-1	#DIV/0!	1	#DIV/0!
4921515J06Rik|66715	-1	#DIV/0!	1	#DIV/0!
B930007M17Rik|381476	-1	#DIV/0!	1	#DIV/0!
1700065I17Rik|67343	-1	#DIV/0!	1	#DIV/0!
Panx3|208098	-1	#DIV/0!	1	#DIV/0!
Olfr299|257929	-1	#DIV/0!	1	#DIV/0!
AA792892|100554	-1	#DIV/0!	1	#DIV/0!
Mir195|387190	-1	#DIV/0!	1	#DIV/0!
Tgm6|241636	-1	#DIV/0!	1	#DIV/0!
Mrap2|244958	-1	#DIV/0!	1	#DIV/0!
Camk1g|215303	-1	#DIV/0!	1	#DIV/0!
9830107B12Rik|328829	-1	#DIV/0!	1	#DIV/0!
Gm6696|626682	-1	#DIV/0!	1	#DIV/0!
Slc5a5|114479	-1	#DIV/0!	1	#DIV/0!
Megf10|70417	-1	#DIV/0!	1	#DIV/0!
Olfr25|18323	-1	#DIV/0!	1	#DIV/0!
Olfr206|258993	-1	#DIV/0!	1	#DIV/0!
Nox3|224480	-1	#DIV/0!	1	#DIV/0!
Crygd|12967	-1	#DIV/0!	1	#DIV/0!
Gfra3|14587	-1	#DIV/0!	1	#DIV/0!
Hoxc10|209448	-1	#DIV/0!	1	#DIV/0!
Gm15144|78092	-1	#DIV/0!	1	#DIV/0!
Pin1-ps1|241593	-1	#DIV/0!	1	#DIV/0!
Mir100|723892	-1	#DIV/0!	1	#DIV/0!
Gm3604|100041979	-1	#DIV/0!	1	#DIV/0!
Gm101|226356	-1	#DIV/0!	1	#DIV/0!
Gm4907|236749	-1	#DIV/0!	1	#DIV/0!
Tas2r103|667992	-1	#DIV/0!	1	#DIV/0!
Cxcl17|232983	-1	#DIV/0!	1	#DIV/0!
Pramel4|347710	-1	#DIV/0!	1	#DIV/0!
Snord52|100217427	-1	#DIV/0!	1	#DIV/0!
Olfr1176|258767	-1	#DIV/0!	1	#DIV/0!
Vmn1r83|171243	-1	#DIV/0!	1	#DIV/0!
Mir465|723888	-1	#DIV/0!	1	#DIV/0!
Lrrc55|241528	-1	#DIV/0!	1	#DIV/0!
A930038C07Rik|68169	-1	#DIV/0!	1	#DIV/0!
Tcp11|21463	-1	#DIV/0!	1	#DIV/0!
Olfr1317|258440	-1	#DIV/0!	1	#DIV/0!
Gsx1|14842	-1	#DIV/0!	1	#DIV/0!
Wdr95|381693	-1	#DIV/0!	1	#DIV/0!

Vmn2r28|665255	-1	#DIV/0!	1	#DIV/0!
Rln1|19773	-1	#DIV/0!	1	#DIV/0!
Olfr373|258532	-1	#DIV/0!	1	#DIV/0!
Amy2a3|100043686	-1	#DIV/0!	1	#DIV/0!
Gif|14603	-1	#DIV/0!	1	#DIV/0!
Ctxn2|381418	-1	#DIV/0!	1	#DIV/0!
5830411N06Rik|244234	-1	#DIV/0!	1	#DIV/0!
Olfr362|259053	-1	#DIV/0!	1	#DIV/0!
Adam6a|238406	-1	#DIV/0!	1	#DIV/0!
Fmo6|226565	-1	#DIV/0!	1	#DIV/0!
Vmn1r81|171244	-1	#DIV/0!	1	#DIV/0!
Phox2b|18935	-1	#DIV/0!	1	#DIV/0!
Olfr211|258914	-1	#DIV/0!	1	#DIV/0!
Vmn1r196|100312484	-1	#DIV/0!	1	#DIV/0!
1810010D01Rik|100040049	-1	#DIV/0!	1	#DIV/0!
4933401B06Rik|71011	-1	#DIV/0!	1	#DIV/0!
Rmst|110333	-1	#DIV/0!	1	#DIV/0!
Olfr283|259038	-1	#DIV/0!	1	#DIV/0!
Guca2a|14915	-1	#DIV/0!	1	#DIV/0!
St6galnac5|26938	-1	#DIV/0!	1	#DIV/0!
Gprin2|432839	-1	#DIV/0!	1	#DIV/0!
Catsper4|329954	-1	#DIV/0!	1	#DIV/0!
Olfr1182|258167	-1	#DIV/0!	1	#DIV/0!
Xylt1|233781	-1	#DIV/0!	1	#DIV/0!
Psg25|114868	-1	#DIV/0!	1	#DIV/0!
Tfap2c|21420	-1	#DIV/0!	1	#DIV/0!
AI836003|239650	-1	#DIV/0!	1	#DIV/0!
Olfr497|258733	-1	#DIV/0!	1	#DIV/0!
Fbxw26|382109	-1	#DIV/0!	1	#DIV/0!
Hoxd3|15434	-1	#DIV/0!	1	#DIV/0!
Zbbx|213234	-1	#DIV/0!	1	#DIV/0!
Stfa3|20863	-1	#DIV/0!	1	#DIV/0!
Gm11190|791423	-1	#DIV/0!	1	#DIV/0!
Vmn1r113|436135	-1	#DIV/0!	1	#DIV/0!
Lrrc3b|218763	-1	#DIV/0!	1	#DIV/0!
Ceacam15|101434	-1	#DIV/0!	1	#DIV/0!
Rnf133|386611	-1	#DIV/0!	1	#DIV/0!
Mir3058|100526463	-1	#DIV/0!	1	#DIV/0!
Tekt3|71062	-1	#DIV/0!	1	#DIV/0!
Bpifc|270757	-1	#DIV/0!	1	#DIV/0!
Slc1a1|20510	-1	#DIV/0!	1	#DIV/0!
Olfr171|258960	-1	#DIV/0!	1	#DIV/0!
Wfdc15a|68221	-1	#DIV/0!	1	#DIV/0!
Ssx9|382206	-1	#DIV/0!	1	#DIV/0!
Synpr|72003	-1	#DIV/0!	1	#DIV/0!
Clec4b2|381809	-1	#DIV/0!	1	#DIV/0!
Olfr943|258323	-1	#DIV/0!	1	#DIV/0!
4930442L01Rik|67583	-1	#DIV/0!	1	#DIV/0!
Olfr609|259086	-1	#DIV/0!	1	#DIV/0!
Vmn2r95|328759	-1	#DIV/0!	1	#DIV/0!
Taar8b|382348	-1	#DIV/0!	1	#DIV/0!
Olfr248|258709	-1	#DIV/0!	1	#DIV/0!
Olfr606|259098	-1	#DIV/0!	1	#DIV/0!

Vmn1r32|171188	-1	#DIV/0!	1	#DIV/0!
Olfr23|18321	-1	#DIV/0!	1	#DIV/0!
Pnmal2|434128	-1	#DIV/0!	1	#DIV/0!
Diap3|56419	-1	#DIV/0!	1	#DIV/0!
Aqp2|11827	-1	#DIV/0!	1	#DIV/0!
Olfr520|259066	-1	#DIV/0!	1	#DIV/0!
BC048502|223927	-1	#DIV/0!	1	#DIV/0!
Dcc|13176	-1	#DIV/0!	1	#DIV/0!
Prr23a|623166	-1	#DIV/0!	1	#DIV/0!
Olfr890|258474	-1	#DIV/0!	1	#DIV/0!
Pnliprp2|18947	-1	#DIV/0!	1	#DIV/0!
Crispld1|83691	-1	#DIV/0!	1	#DIV/0!
Mir3059|100526516	-1	#DIV/0!	1	#DIV/0!
BC051019|57355	-1	#DIV/0!	1	#DIV/0!
Olfr1325|258393	-1	#DIV/0!	1	#DIV/0!
Havcr1|171283	-1	#DIV/0!	1	#DIV/0!
Sema3a|20346	-1	#DIV/0!	1	#DIV/0!
4930592I03Rik|75853	-1	#DIV/0!	1	#DIV/0!
Mir344d-3|100526543	-1	#DIV/0!	1	#DIV/0!
Gm5134|333669	-1	#DIV/0!	1	#DIV/0!
Lce3f|69520	-1	#DIV/0!	1	#DIV/0!
Grpr|14829	-1	#DIV/0!	1	#DIV/0!
Krt12|268482	-1	#DIV/0!	1	#DIV/0!
Olfr923|258812	-1	#DIV/0!	1	#DIV/0!
Gpr83|14608	-1	#DIV/0!	1	#DIV/0!
Fpr-rs3|14290	-1	#DIV/0!	1	#DIV/0!
Snord12|100217443	-1	#DIV/0!	1	#DIV/0!
Gm4906|236663	-1	#DIV/0!	1	#DIV/0!
Kcnj3|16519	-1	#DIV/0!	1	#DIV/0!
Amy2b|545562	-1	#DIV/0!	1	#DIV/0!
Lep|16846	-1	#DIV/0!	1	#DIV/0!
Olfr1258|258980	-1	#DIV/0!	1	#DIV/0!
Olfr228|258400	-1	#DIV/0!	1	#DIV/0!
Olfr126|258892	-1	#DIV/0!	1	#DIV/0!
Rhox4a|664609	-1	#DIV/0!	1	#DIV/0!
Cyp11b1|110115	-1	#DIV/0!	1	#DIV/0!
Kcns1|16538	-1	#DIV/0!	1	#DIV/0!
Ctsll3|70202	-1	#DIV/0!	1	#DIV/0!
Pcdhgb5|93702	-1	#DIV/0!	1	#DIV/0!
Apln|30878	-1	#DIV/0!	1	#DIV/0!
Pkhd1l1|192190	-1	#DIV/0!	1	#DIV/0!
Kbtbd5|72330	-1	#DIV/0!	1	#DIV/0!
Gm889|380755	-1	#DIV/0!	1	#DIV/0!
Areg|11839	-1	#DIV/0!	1	#DIV/0!
Olfr449|259067	-1	#DIV/0!	1	#DIV/0!
Olfr1123|258347	-1	#DIV/0!	1	#DIV/0!
Aknad1|329738	-1	#DIV/0!	1	#DIV/0!
2310034C09Rik|117172	-1	#DIV/0!	1	#DIV/0!
L3mbtl1|241764	-1	#DIV/0!	1	#DIV/0!
Ano7|404545	-1	#DIV/0!	1	#DIV/0!
Mir543|723881	-1	#DIV/0!	1	#DIV/0!
Cdr1|631990	-1	#DIV/0!	1	#DIV/0!
Atp6v1b1|110935	-1	#DIV/0!	1	#DIV/0!

Phex|18675	-1	#DIV/0!	1	#DIV/0!
Sprr2g|20761	-1	#DIV/0!	1	#DIV/0!
Vmn2r35|625353	-1	#DIV/0!	1	#DIV/0!
Ankrd60|70065	-1	#DIV/0!	1	#DIV/0!
Olfr1247|258968	-1	#DIV/0!	1	#DIV/0!
Gm13939|100038454	-1	#DIV/0!	1	#DIV/0!
Mcpt1|17224	-1	#DIV/0!	1	#DIV/0!
Cmtm2b|75502	-1	#DIV/0!	1	#DIV/0!
Olfr136|258803	-1	#DIV/0!	1	#DIV/0!
Oosp1|170834	-1	#DIV/0!	1	#DIV/0!
Mir135b|723818	-1	#DIV/0!	1	#DIV/0!
Tsix|22097	-1	#DIV/0!	1	#DIV/0!
Mir137|387155	-1	#DIV/0!	1	#DIV/0!
Gfral|404194	-1	#DIV/0!	1	#DIV/0!
Mir872|100124456	-1	#DIV/0!	1	#DIV/0!
Bpifb1|228801	-1	#DIV/0!	1	#DIV/0!
Pkd2l1|329064	-1	#DIV/0!	1	#DIV/0!
Ppp1r17|19051	-1	#DIV/0!	1	#DIV/0!
Defa3|13237	-1	#DIV/0!	1	#DIV/0!
Spats1|71020	-1	#DIV/0!	1	#DIV/0!
Olfr8|18372	-1	#DIV/0!	1	#DIV/0!
Gm5494|433107	-1	#DIV/0!	1	#DIV/0!
Rasgef1a|70727	-1	#DIV/0!	1	#DIV/0!
Palm2|242481	-1	#DIV/0!	1	#DIV/0!
Gm16442|620401	-1	#DIV/0!	1	#DIV/0!
Olfr116|258625	-1	#DIV/0!	1	#DIV/0!
Rbp3|19661	-1	#DIV/0!	1	#DIV/0!
Olfr700|258593	-1	#DIV/0!	1	#DIV/0!
Gm17495|100126778	-1	#DIV/0!	1	#DIV/0!
Cadm2|239857	-1	#DIV/0!	1	#DIV/0!
Lbx1|16814	-1	#DIV/0!	1	#DIV/0!
Tas2r118|387347	-1	#DIV/0!	1	#DIV/0!
Slc22a2|20518	-1	#DIV/0!	1	#DIV/0!
Il18rap|16174	-1	#DIV/0!	1	#DIV/0!
Naaladl1|381204	-1	#DIV/0!	1	#DIV/0!
Bpifb3|378700	-1	#DIV/0!	1	#DIV/0!
Vmn2r113|434701	-1	#DIV/0!	1	#DIV/0!
Olfr98|258503	-1	#DIV/0!	1	#DIV/0!
Olfr1126|258834	-1	#DIV/0!	1	#DIV/0!
Olfr881|258413	-1	#DIV/0!	1	#DIV/0!
Olfr649|259057	-1	#DIV/0!	1	#DIV/0!
1700061J05Rik|73376	-1	#DIV/0!	1	#DIV/0!
G6b|114763	-1	#DIV/0!	1	#DIV/0!
Prss37|67690	-1	#DIV/0!	1	#DIV/0!
Olfr490|258491	-1	#DIV/0!	1	#DIV/0!
Boll|75388	-1	#DIV/0!	1	#DIV/0!
Vmn1r200|171246	-1	#DIV/0!	1	#DIV/0!
Tmem74|239408	-1	#DIV/0!	1	#DIV/0!
Mir379|723858	-1	#DIV/0!	1	#DIV/0!
Snora28|100316932	-1	#DIV/0!	1	#DIV/0!
4930404A10Rik|74847	-1	#DIV/0!	1	#DIV/0!
Cbln2|12405	-1	#DIV/0!	1	#DIV/0!
Mir1946a|100316697	-1	#DIV/0!	1	#DIV/0!

Htr5a|15563	-1	#DIV/0!	1	#DIV/0!
Gm1141|382221	-1	#DIV/0!	1	#DIV/0!
Cacng3|54376	-1	#DIV/0!	1	#DIV/0!
Smpx|66106	-1	#DIV/0!	1	#DIV/0!
Slc5a2|246787	-1	#DIV/0!	1	#DIV/0!
Kcna3|16491	-1	#DIV/0!	1	#DIV/0!
Gm765|330390	-1	#DIV/0!	1	#DIV/0!
Gm13305|100042555	-1	#DIV/0!	1	#DIV/0!
Olfr731|258360	-1	#DIV/0!	1	#DIV/0!
Fbxo43|78803	-1	#DIV/0!	1	#DIV/0!
Olfr539|258963	-1	#DIV/0!	1	#DIV/0!
Wnt1|22408	-1	#DIV/0!	1	#DIV/0!
Sim2|20465	-1	#DIV/0!	1	#DIV/0!
Etv2|14008	-1	#DIV/0!	1	#DIV/0!
Hist1h2af|319173	-1	#DIV/0!	1	#DIV/0!
Crybb2|12961	-1	#DIV/0!	1	#DIV/0!
Olfr12|257890	-1	#DIV/0!	1	#DIV/0!
Gm6455|623849	-1	#DIV/0!	1	#DIV/0!
Nhlh2|18072	-1	#DIV/0!	1	#DIV/0!
Lypd6b|71897	-1	#DIV/0!	1	#DIV/0!
Gm9767|100040851	-1	#DIV/0!	1	#DIV/0!
Chit1|71884	-1	#DIV/0!	1	#DIV/0!
Hoxd1|15429	-1	#DIV/0!	1	#DIV/0!
Svs3b|329557	-1	#DIV/0!	1	#DIV/0!
Tex24|541463	-1	#DIV/0!	1	#DIV/0!
Kcnb2|98741	-1	#DIV/0!	1	#DIV/0!
Zpbp2|69376	-1	#DIV/0!	1	#DIV/0!
Olfr60|18361	-1	#DIV/0!	1	#DIV/0!
Zscan5b|170734	-1	#DIV/0!	1	#DIV/0!
Asb11|68854	-1	#DIV/0!	1	#DIV/0!
Olfr569|259092	-1	#DIV/0!	1	#DIV/0!
Higd1b|75689	-1	#DIV/0!	1	#DIV/0!
Olfr309|258196	-1	#DIV/0!	1	#DIV/0!
Mir702|735283	-1	#DIV/0!	1	#DIV/0!
Tulp2|56734	-1	#DIV/0!	1	#DIV/0!
Olfr229|258606	-1	#DIV/0!	1	#DIV/0!
Pramef12|77632	-1	#DIV/0!	1	#DIV/0!
Olfr934|258434	-1	#DIV/0!	1	#DIV/0!
Olfr357|258616	-1	#DIV/0!	1	#DIV/0!
Ssxb3|278174	-1	#DIV/0!	1	#DIV/0!
Defb35|246084	-1	#DIV/0!	1	#DIV/0!
Igbp1b|50540	-1	#DIV/0!	1	#DIV/0!
Mir684-2|735261	-1	#DIV/0!	1	#DIV/0!
Gm10696|100043188	-1	#DIV/0!	1	#DIV/0!
Gria2|14800	-1	#DIV/0!	1	#DIV/0!
Aipl1|114230	-1	#DIV/0!	1	#DIV/0!
5430421N21Rik|100126226	-1	#DIV/0!	1	#DIV/0!
Slc6a20b|22599	-1	#DIV/0!	1	#DIV/0!
Foxe3|30923	-1	#DIV/0!	1	#DIV/0!
Slc38a8|234788	-1	#DIV/0!	1	#DIV/0!
D130040H23Rik|211135	-1	#DIV/0!	1	#DIV/0!
Eif4e1b|218268	-1	#DIV/0!	1	#DIV/0!
Olfr552|259106	-1	#DIV/0!	1	#DIV/0!

Klra15|27423	-1	#DIV/0!	1	#DIV/0!
Gm5114|330513	-1	#DIV/0!	1	#DIV/0!
Syt13|80976	-1	#DIV/0!	1	#DIV/0!
Snord34|27210	-1	#DIV/0!	1	#DIV/0!
1700025F22Rik|69416	-1	#DIV/0!	1	#DIV/0!
Pira11|18724	-1	#DIV/0!	1	#DIV/0!
Fer1l4|74562	-1	#DIV/0!	1	#DIV/0!
Speer6-ps1|73266	-1	#DIV/0!	1	#DIV/0!
Mir467f|100316749	-1	#DIV/0!	1	#DIV/0!
F630042J09Rik|100038627	-1	#DIV/0!	1	#DIV/0!
Atp6v1g3|338375	-1	#DIV/0!	1	#DIV/0!
Cabp7|192650	-1	#DIV/0!	1	#DIV/0!
Krtap4-16|435285	-1	#DIV/0!	1	#DIV/0!
Krt15|16665	-1	#DIV/0!	1	#DIV/0!
Krtap13-1|268905	-1	#DIV/0!	1	#DIV/0!
Snord67|100217458	-1	#DIV/0!	1	#DIV/0!
Galp|232836	-1	#DIV/0!	1	#DIV/0!
Mir762|791073	-1	#DIV/0!	1	#DIV/0!
Gm15056|100041654	-1	#DIV/0!	1	#DIV/0!
Cd40lg|21947	-1	#DIV/0!	1	#DIV/0!
Olfr1061|259022	-1	#DIV/0!	1	#DIV/0!
Foxd4|14237	-1	#DIV/0!	1	#DIV/0!
Gm6994|629678	-1	#DIV/0!	1	#DIV/0!
Vmn1r24|171191	-1	#DIV/0!	1	#DIV/0!
Ntng1|80883	-1	#DIV/0!	1	#DIV/0!
Adam2|11495	-1	#DIV/0!	1	#DIV/0!
Ankrd22|52024	-1	#DIV/0!	1	#DIV/0!
Htr2b|15559	-1	#DIV/0!	1	#DIV/0!
Spata18|73472	-1	#DIV/0!	1	#DIV/0!
Fam24a|68223	-1	#DIV/0!	1	#DIV/0!
Grm2|108068	-1	#DIV/0!	1	#DIV/0!
Olfr348|258946	-1	#DIV/0!	1	#DIV/0!
2410012M07Rik|71979	-1	#DIV/0!	1	#DIV/0!
Fnd3c2|331491	-1	#DIV/0!	1	#DIV/0!
Atp6ap1l|435376	-1	#DIV/0!	1	#DIV/0!
Olfr43|258706	-1	#DIV/0!	1	#DIV/0!
Anxa10|26359	-1	#DIV/0!	1	#DIV/0!
Vmn1r123|384695	-1	#DIV/0!	1	#DIV/0!
Mcpt2|17225	-1	#DIV/0!	1	#DIV/0!
Olfr123|258623	-1	#DIV/0!	1	#DIV/0!
E230025N22Rik|240216	-1	#DIV/0!	1	#DIV/0!
Vmn2r80|624765	-1	#DIV/0!	1	#DIV/0!
4930465K10Rik|67640	-1	#DIV/0!	1	#DIV/0!
6330403K07Rik|103712	-1	#DIV/0!	1	#DIV/0!
Olfr1044|259013	-1	#DIV/0!	1	#DIV/0!
4932411E22Rik|214604	-1	#DIV/0!	1	#DIV/0!
Olfr1200|257887	-1	#DIV/0!	1	#DIV/0!
Zcchc16|619287	-1	#DIV/0!	1	#DIV/0!
Hspb3|56534	-1	#DIV/0!	1	#DIV/0!
Vmn1r217|171273	-1	#DIV/0!	1	#DIV/0!
Olfr788|258544	-1	#DIV/0!	1	#DIV/0!
Pkd1l3|244646	-1	#DIV/0!	1	#DIV/0!
Rnase1|19752	-1	#DIV/0!	1	#DIV/0!

Gm1993|100038977	-1	#DIV/0!	1	#DIV/0!
Pnlip|69060	-1	#DIV/0!	1	#DIV/0!
Dctd|320685	-1	#DIV/0!	1	#DIV/0!
Slco6b1|67854	-1	#DIV/0!	1	#DIV/0!
Prl4a1|19110	-1	#DIV/0!	1	#DIV/0!
Olfr1340|258301	-1	#DIV/0!	1	#DIV/0!
Gm17727|100312986	-1	#DIV/0!	1	#DIV/0!
Olfr1198|404330	-1	#DIV/0!	1	#DIV/0!
Mmp13|17386	-1	#DIV/0!	1	#DIV/0!
Olfr390|258344	-1	#DIV/0!	1	#DIV/0!
8030411F24Rik|78609	-1	#DIV/0!	1	#DIV/0!
Mslnl|328783	-1	#DIV/0!	1	#DIV/0!
Ttc24|214191	-1	#DIV/0!	1	#DIV/0!
Slc5a8|216225	-1	#DIV/0!	1	#DIV/0!
Vmn2r83|625029	-1	#DIV/0!	1	#DIV/0!
Olfr1018|258579	-1	#DIV/0!	1	#DIV/0!
H28|15061	-1	#DIV/0!	1	#DIV/0!
Mum1l1|245631	-1	#DIV/0!	1	#DIV/0!
Otol1|229389	-1	#DIV/0!	1	#DIV/0!
Trim71|636931	-1	#DIV/0!	1	#DIV/0!
Olfr1392|258462	-1	#DIV/0!	1	#DIV/0!
4933403O08Rik|71030	-1	#DIV/0!	1	#DIV/0!
Vmn2r114|666002	-1	#DIV/0!	1	#DIV/0!
Ppp2r2c|269643	-1	#DIV/0!	1	#DIV/0!
Olfr703|258589	-1	#DIV/0!	1	#DIV/0!
9430021M05Rik|77288	-1	#DIV/0!	1	#DIV/0!
4930507D05Rik|74706	-1	#DIV/0!	1	#DIV/0!
Adam25|23793	-1	#DIV/0!	1	#DIV/0!
Gm4894|235327	-1	#DIV/0!	1	#DIV/0!
Speer2|224318	-1	#DIV/0!	1	#DIV/0!
Skint11|230623	-1	#DIV/0!	1	#DIV/0!
Pla2g10|26565	-1	#DIV/0!	1	#DIV/0!
Prss42|235628	-1	#DIV/0!	1	#DIV/0!
4930415F15Rik|73862	-1	#DIV/0!	1	#DIV/0!
Pabpn1l|382035	-1	#DIV/0!	1	#DIV/0!
Slc36a3|215332	-1	#DIV/0!	1	#DIV/0!
Vmn1r72|252905	-1	#DIV/0!	1	#DIV/0!
Olfr304|258089	-1	#DIV/0!	1	#DIV/0!
Efcab3|70894	-1	#DIV/0!	1	#DIV/0!
Olfr701|66786	-1	#DIV/0!	1	#DIV/0!
Olfr339|258951	-1	#DIV/0!	1	#DIV/0!
Pcdhb11|93882	-1	#DIV/0!	1	#DIV/0!
BC100530|100034684	-1	#DIV/0!	1	#DIV/0!
Cntnap3|238680	-1	#DIV/0!	1	#DIV/0!
Dlg2|23859	-1	#DIV/0!	1	#DIV/0!
Olfr921|258778	-1	#DIV/0!	1	#DIV/0!
Olfr192|404309	-1	#DIV/0!	1	#DIV/0!
Fam150a|620393	-1	#DIV/0!	1	#DIV/0!
Gm10389|100038608	-1	#DIV/0!	1	#DIV/0!
Acer1|171168	-1	#DIV/0!	1	#DIV/0!
Sbpl|638345	-1	#DIV/0!	1	#DIV/0!
Trim52|212085	-1	#DIV/0!	1	#DIV/0!
Olfr697|258592	-1	#DIV/0!	1	#DIV/0!

Opn5|353344	-1	#DIV/0!	1	#DIV/0!
Pcdhga6|93714	-1	#DIV/0!	1	#DIV/0!
Amelx|11704	-1	#DIV/0!	1	#DIV/0!
Tdpoz5|399676	-1	#DIV/0!	1	#DIV/0!
Fstl4|320027	-1	#DIV/0!	1	#DIV/0!
Gm15104|333588	-1	#DIV/0!	1	#DIV/0!
Vmn2r12|627569	-1	#DIV/0!	1	#DIV/0!
Htr5b|15564	-1	#DIV/0!	1	#DIV/0!
Vmn1r41|113857	-1	#DIV/0!	1	#DIV/0!
Gm4850|226957	-1	#DIV/0!	1	#DIV/0!
Vmn2r51|100042921	-1	#DIV/0!	1	#DIV/0!
Olfr1006|258563	-1	#DIV/0!	1	#DIV/0!
Vmn2r81|216144	-1	#DIV/0!	1	#DIV/0!
Ysk4|22625	-1	#DIV/0!	1	#DIV/0!
Lcn12|77701	-1	#DIV/0!	1	#DIV/0!
Gm5627|434510	-1	#DIV/0!	1	#DIV/0!
Olfr459|258569	-1	#DIV/0!	1	#DIV/0!
Foxg1|15228	-1	#DIV/0!	1	#DIV/0!
Gm5476|432985	-1	#DIV/0!	1	#DIV/0!
Pramef6|195555	-1	#DIV/0!	1	#DIV/0!
Defa5|13239	-1	#DIV/0!	1	#DIV/0!
Olfr635|259122	-1	#DIV/0!	1	#DIV/0!
Bai3|210933	-1	#DIV/0!	1	#DIV/0!
Mir362|723851	-1	#DIV/0!	1	#DIV/0!
4930588N13Rik|75860	-1	#DIV/0!	1	#DIV/0!
Ccnb1ip1|239083	-1	#DIV/0!	1	#DIV/0!
Slco1a5|108096	-1	#DIV/0!	1	#DIV/0!
Olfr1087|258843	-1	#DIV/0!	1	#DIV/0!
Vsx1|114889	-1	#DIV/0!	1	#DIV/0!
Vmn1r173|545934	-1	#DIV/0!	1	#DIV/0!
Obox6|252830	-1	#DIV/0!	1	#DIV/0!
Olfr1302|258891	-1	#DIV/0!	1	#DIV/0!
Olfr1427|258674	-1	#DIV/0!	1	#DIV/0!
Defb42|619548	-1	#DIV/0!	1	#DIV/0!
Oog2|381570	-1	#DIV/0!	1	#DIV/0!
Hsd3b1|15492	-1	#DIV/0!	1	#DIV/0!
Prl3a1|67000	-1	#DIV/0!	1	#DIV/0!
Hist1h1b|56702	-1	#DIV/0!	1	#DIV/0!
Mir3100|100526504	-1	#DIV/0!	1	#DIV/0!
Fam71b|432552	-1	#DIV/0!	1	#DIV/0!
Rimbp3|239731	-1	#DIV/0!	1	#DIV/0!
Olfr1314|258442	-1	#DIV/0!	1	#DIV/0!
Slc5a12|241612	-1	#DIV/0!	1	#DIV/0!
9430041J12Rik|77323	-1	#DIV/0!	1	#DIV/0!
Vmn2r93|627132	-1	#DIV/0!	1	#DIV/0!
Tbx22|245572	-1	#DIV/0!	1	#DIV/0!
Vmn1r100|100043536	-1	#DIV/0!	1	#DIV/0!
Susd5|382111	-1	#DIV/0!	1	#DIV/0!
Krtap24-1|239932	-1	#DIV/0!	1	#DIV/0!
Vwc2|319922	-1	#DIV/0!	1	#DIV/0!
Gm5082|328231	-1	#DIV/0!	1	#DIV/0!
Gm1045|381651	-1	#DIV/0!	1	#DIV/0!
Gm9047|668210	-1	#DIV/0!	1	#DIV/0!

Vmn1r237|171234	-1	#DIV/0!	1	#DIV/0!
Zfp36l3|333473	-1	#DIV/0!	1	#DIV/0!
BC117090|100038854	-1	#DIV/0!	1	#DIV/0!
Vmn1r20|434017	-1	#DIV/0!	1	#DIV/0!
Olfr1276|258390	-1	#DIV/0!	1	#DIV/0!
Olfr849|258520	-1	#DIV/0!	1	#DIV/0!
Cts8|56094	-1	#DIV/0!	1	#DIV/0!
Gm3646|100042065	-1	#DIV/0!	1	#DIV/0!
Mir29c|387224	-1	#DIV/0!	1	#DIV/0!
Wnt10b|22410	-1	#DIV/0!	1	#DIV/0!
Vmn2r32|22311	-1	#DIV/0!	1	#DIV/0!
Krt72-ps|105866	-1	#DIV/0!	1	#DIV/0!
Zfp804a|241514	-1	#DIV/0!	1	#DIV/0!
Serpinb2|18788	-1	#DIV/0!	1	#DIV/0!
Cntn4|269784	-1	#DIV/0!	1	#DIV/0!
Glod5|69824	-1	#DIV/0!	1	#DIV/0!
Defb34|360211	-1	#DIV/0!	1	#DIV/0!
Fam164b|75122	-1	#DIV/0!	1	#DIV/0!
Cysltr2|70086	-1	#DIV/0!	1	#DIV/0!
Gprin1|26913	-1	#DIV/0!	1	#DIV/0!
Olfr905|258800	-1	#DIV/0!	1	#DIV/0!
Gm5346|384813	-1	#DIV/0!	1	#DIV/0!
Dmrtc1a|70887	-1	#DIV/0!	1	#DIV/0!
Nkx2-2as|100313531	-1	#DIV/0!	1	#DIV/0!
Olfr273|258821	-1	#DIV/0!	1	#DIV/0!
Snord33|27208	-1	#DIV/0!	1	#DIV/0!
Rex2|19715	-1	#DIV/0!	1	#DIV/0!
Bsph1|330470	-1	#DIV/0!	1	#DIV/0!
Fezf2|54713	-1	#DIV/0!	1	#DIV/0!
Ly6g6d|114654	-1	#DIV/0!	1	#DIV/0!
Hist1h4d|319156	-1	#DIV/0!	1	#DIV/0!
D4Ertd617e|100041290	-1	#DIV/0!	1	#DIV/0!
Clca6|99663	-1	#DIV/0!	1	#DIV/0!
Nkx2-3|18089	-1	#DIV/0!	1	#DIV/0!
Olfr213|258020	-1	#DIV/0!	1	#DIV/0!
Chn1|108699	-1	#DIV/0!	1	#DIV/0!
Ifna6|15969	-1	#DIV/0!	1	#DIV/0!
4921530L21Rik|66732	-1	#DIV/0!	1	#DIV/0!
Matn3|17182	-1	#DIV/0!	1	#DIV/0!
Apobec4|71281	-1	#DIV/0!	1	#DIV/0!
Bpifb2|66557	-1	#DIV/0!	1	#DIV/0!
Gm15085|100039934	-1	#DIV/0!	1	#DIV/0!
Scgb1a1|22287	-1	#DIV/0!	1	#DIV/0!
Crabp2|12904	-1	#DIV/0!	1	#DIV/0!
Mir296|723906	-1	#DIV/0!	1	#DIV/0!
Tomm20l|75266	-1	#DIV/0!	1	#DIV/0!
Dsg4|16769	-1	#DIV/0!	1	#DIV/0!
Clec3a|403395	-1	#DIV/0!	1	#DIV/0!
Psd2|74002	-1	#DIV/0!	1	#DIV/0!
Vmn2r63|435975	-1	#DIV/0!	1	#DIV/0!
Vmn1r48|113845	-1	#DIV/0!	1	#DIV/0!
Btnl5|81497	-1	#DIV/0!	1	#DIV/0!
Scrt1|170729	-1	#DIV/0!	1	#DIV/0!

Iltifb|116849	-1	#DIV/0!	1	#DIV/0!
Vmn1r57|665150	-1	#DIV/0!	1	#DIV/0!
Olfr862|258555	-1	#DIV/0!	1	#DIV/0!
Prl6a1|19111	-1	#DIV/0!	1	#DIV/0!
Tgm3|21818	-1	#DIV/0!	1	#DIV/0!
Nxf7|170722	-1	#DIV/0!	1	#DIV/0!
Syce1l|668110	-1	#DIV/0!	1	#DIV/0!
Olfr45|18344	-1	#DIV/0!	1	#DIV/0!
4933429O19Rik|66770	-1	#DIV/0!	1	#DIV/0!
Ccr8|12776	-1	#DIV/0!	1	#DIV/0!
Klrc3|58179	-1	#DIV/0!	1	#DIV/0!
Ebf2|13592	-1	#DIV/0!	1	#DIV/0!
Ttc23l|75777	-1	#DIV/0!	1	#DIV/0!
Olfr655|258817	-1	#DIV/0!	1	#DIV/0!
Adamts16|271127	-1	#DIV/0!	1	#DIV/0!
Zscan18|232875	-1	#DIV/0!	1	#DIV/0!
Taar2|209512	-1	#DIV/0!	1	#DIV/0!
Gm4937|238829	-1	#DIV/0!	1	#DIV/0!
Olfr1336|258917	-1	#DIV/0!	1	#DIV/0!
4930526D03Rik|277496	-1	#DIV/0!	1	#DIV/0!
Pate4|56872	-1	#DIV/0!	1	#DIV/0!
Mir496|751524	-1	#DIV/0!	1	#DIV/0!
Cdh9|12565	-1	#DIV/0!	1	#DIV/0!
Tmem151b|210573	-1	#DIV/0!	1	#DIV/0!
Olfr970|258604	-1	#DIV/0!	1	#DIV/0!
Olfr137|258481	-1	#DIV/0!	1	#DIV/0!
Vmn1r157|667551	-1	#DIV/0!	1	#DIV/0!
Gpr45|93690	-1	#DIV/0!	1	#DIV/0!
Olfr948|257912	-1	#DIV/0!	1	#DIV/0!
Foxl2os|768252	-1	#DIV/0!	1	#DIV/0!
Kcnk9|223604	-1	#DIV/0!	1	#DIV/0!
Vmn1r203|171270	-1	#DIV/0!	1	#DIV/0!
4930428D18Rik|619294	-1	#DIV/0!	1	#DIV/0!
5730416F02Rik|70550	-1	#DIV/0!	1	#DIV/0!
Zfp42|22702	-1	#DIV/0!	1	#DIV/0!
Ang3|11730	-1	#DIV/0!	1	#DIV/0!
Bpifa5|67135	-1	#DIV/0!	1	#DIV/0!
Efcab1|66793	-1	#DIV/0!	1	#DIV/0!
Gm13271|435791	-1	#DIV/0!	1	#DIV/0!
1700020D05Rik|75555	-1	#DIV/0!	1	#DIV/0!
Gm6938|629024	-1	#DIV/0!	1	#DIV/0!
Tnni3k|435766	-1	#DIV/0!	1	#DIV/0!
Edn2|13615	-1	#DIV/0!	1	#DIV/0!
Olfr1|258923	-1	#DIV/0!	1	#DIV/0!
Gm10494|100038718	-1	#DIV/0!	1	#DIV/0!
Mir21|387140	-1	#DIV/0!	1	#DIV/0!
Cldn23|71908	-1	#DIV/0!	1	#DIV/0!
Olfr1341|258852	-1	#DIV/0!	1	#DIV/0!
Mir3074-1|100526474	-1	#DIV/0!	1	#DIV/0!
Defb11|246081	-1	#DIV/0!	1	#DIV/0!
Pcdhgb1|93699	-1	#DIV/0!	1	#DIV/0!
Olfr1109|258762	-1	#DIV/0!	1	#DIV/0!
Mir99a|387229	-1	#DIV/0!	1	#DIV/0!

Qrfpr|229214	-1	#DIV/0!	1	#DIV/0!
Olfr96|258507	-1	#DIV/0!	1	#DIV/0!
Pcdhga3|93711	-1	#DIV/0!	1	#DIV/0!
Lipn|70166	-1	#DIV/0!	1	#DIV/0!
Vmn2r10|22307	-1	#DIV/0!	1	#DIV/0!
Gm10272|16697	-1	#DIV/0!	1	#DIV/0!
Tmem174|68344	-1	#DIV/0!	1	#DIV/0!
Snord64|100217429	-1	#DIV/0!	1	#DIV/0!
Olfr1263|258790	-1	#DIV/0!	1	#DIV/0!
Vmn2r5|667060	-1	#DIV/0!	1	#DIV/0!
Tchhl1|71325	-1	#DIV/0!	1	#DIV/0!
Zfp936|668620	-1	#DIV/0!	1	#DIV/0!
1810009J06Rik|73626	-1	#DIV/0!	1	#DIV/0!
Olfr1299|258886	-1	#DIV/0!	1	#DIV/0!
Olfr564|258356	-1	#DIV/0!	1	#DIV/0!
Mir376b|723934	-1	#DIV/0!	1	#DIV/0!
4933402P03Rik|108803	-1	#DIV/0!	1	#DIV/0!
Mir697|735277	-1	#DIV/0!	1	#DIV/0!
Gm16897|320400	-1	#DIV/0!	1	#DIV/0!
Olfr961|258497	-1	#DIV/0!	1	#DIV/0!
1700025C18Rik|72211	-1	#DIV/0!	1	#DIV/0!
Hist1h3h|319152	-1	#DIV/0!	1	#DIV/0!
Olfr1166|258644	-1	#DIV/0!	1	#DIV/0!
Gm4349|100043305	-1	#DIV/0!	1	#DIV/0!
Zfp819|74400	-1	#DIV/0!	1	#DIV/0!
Olfr922|258777	-1	#DIV/0!	1	#DIV/0!
Snord35a|27211	-1	#DIV/0!	1	#DIV/0!
Vmn1r112|100043569	-1	#DIV/0!	1	#DIV/0!
Mir193|387188	-1	#DIV/0!	1	#DIV/0!
Olfr164|258443	-1	#DIV/0!	1	#DIV/0!
Btnl6|624681	-1	#DIV/0!	1	#DIV/0!
Olfr418-ps1|258645	-1	#DIV/0!	1	#DIV/0!
Zan|22635	-1	#DIV/0!	1	#DIV/0!
Adam39|546055	-1	#DIV/0!	1	#DIV/0!
Klhl10|66720	-1	#DIV/0!	1	#DIV/0!
Siglec5|233186	-1	#DIV/0!	1	#DIV/0!
Olfr312|258065	-1	#DIV/0!	1	#DIV/0!
Olfr958|258327	-1	#DIV/0!	1	#DIV/0!
Olfr473|258771	-1	#DIV/0!	1	#DIV/0!
Atp2a1|11937	-1	#DIV/0!	1	#DIV/0!
Rptn|20129	-1	#DIV/0!	1	#DIV/0!
3110039M20Rik|67293	-1	#DIV/0!	1	#DIV/0!
Rxfp2|140498	-1	#DIV/0!	1	#DIV/0!
Crygb|12965	-1	#DIV/0!	1	#DIV/0!
Mir484|723916	-1	#DIV/0!	1	#DIV/0!
Rag1|19373	-1	#DIV/0!	1	#DIV/0!
Olfr987|257951	-1	#DIV/0!	1	#DIV/0!
Mir680-1|735268	-1	#DIV/0!	1	#DIV/0!
Gm14374|434725	-1	#DIV/0!	1	#DIV/0!
Mir1956|100316706	-1	#DIV/0!	1	#DIV/0!
Olfr771|258540	-1	#DIV/0!	1	#DIV/0!
Nptxr|73340	-1	#DIV/0!	1	#DIV/0!
Serpinb6c|97848	-1	#DIV/0!	1	#DIV/0!

Mboat2|67216	-1	#DIV/0!	1	#DIV/0!
Gm572|230909	-1	#DIV/0!	1	#DIV/0!
Fam75d3|72219	-1	#DIV/0!	1	#DIV/0!
Olfr870|57251	-1	#DIV/0!	1	#DIV/0!
Olfr725|258314	-1	#DIV/0!	1	#DIV/0!
Olfr1158|258639	-1	#DIV/0!	1	#DIV/0!
BC061237|385138	-1	#DIV/0!	1	#DIV/0!
Foxi3|232077	-1	#DIV/0!	1	#DIV/0!
Crybb1|12960	-1	#DIV/0!	1	#DIV/0!
Mir547|723918	-1	#DIV/0!	1	#DIV/0!
Krtap4-2|68673	-1	#DIV/0!	1	#DIV/0!
Mir742|100049548	-1	#DIV/0!	1	#DIV/0!
Mir690|751543	-1	#DIV/0!	1	#DIV/0!
Olfr434|258366	-1	#DIV/0!	1	#DIV/0!
Txndc8|67402	-1	#DIV/0!	1	#DIV/0!
Mtap9|213582	-1	#DIV/0!	1	#DIV/0!
Tmigd1|66601	-1	#DIV/0!	1	#DIV/0!
Gpr123|52389	-1	#DIV/0!	1	#DIV/0!
AU021034|219170	-1	#DIV/0!	1	#DIV/0!
Tmem30c|71027	-1	#DIV/0!	1	#DIV/0!
Gm10248|791307	-1	#DIV/0!	1	#DIV/0!
Npas4|225872	-1	#DIV/0!	1	#DIV/0!
Zkscan16|100041581	-1	#DIV/0!	1	#DIV/0!
Foxl1|14241	-1	#DIV/0!	1	#DIV/0!
Defb13|246083	-1	#DIV/0!	1	#DIV/0!
Olfr130|258480	-1	#DIV/0!	1	#DIV/0!
Mc5r|17203	-1	#DIV/0!	1	#DIV/0!
H2-M10.2|333715	-1	#DIV/0!	1	#DIV/0!
Dlgap2|244310	-1	#DIV/0!	1	#DIV/0!
Mrgprg|381974	-1	#DIV/0!	1	#DIV/0!
Ceacam19|319930	-1	#DIV/0!	1	#DIV/0!
Mir217|387213	-1	#DIV/0!	1	#DIV/0!
Tubb2a-ps2|627110	-1	#DIV/0!	1	#DIV/0!
2310046A06Rik|69642	-1	#DIV/0!	1	#DIV/0!
Arhgdig|14570	-1	#DIV/0!	1	#DIV/0!
Mylk2|228785	-1	#DIV/0!	1	#DIV/0!
Cypt2-ps|245566	-1	#DIV/0!	1	#DIV/0!
Fscb|623046	-1	#DIV/0!	1	#DIV/0!
Olfr938|258430	-1	#DIV/0!	1	#DIV/0!
1700001C02Rik|75434	-1	#DIV/0!	1	#DIV/0!
Defb15|246082	-1	#DIV/0!	1	#DIV/0!
Oprl1|18389	-1	#DIV/0!	1	#DIV/0!
Olfr71|56015	-1	#DIV/0!	1	#DIV/0!
LOC243676|243676	-1	#DIV/0!	1	#DIV/0!
Tff1|21784	-1	#DIV/0!	1	#DIV/0!
Cyp4f40|631304	-1	#DIV/0!	1	#DIV/0!
Gm13088|277668	-1	#DIV/0!	1	#DIV/0!
Olfr984|258601	-1	#DIV/0!	1	#DIV/0!
Olfr1323|258385	-1	#DIV/0!	1	#DIV/0!
Grik2|14806	-1	#DIV/0!	1	#DIV/0!
5330439B14Rik|321015	-1	#DIV/0!	1	#DIV/0!
Hist1h3c|319148	-1	#DIV/0!	1	#DIV/0!
Sprr3|20766	-1	#DIV/0!	1	#DIV/0!

Gm4297|100043216	-1	#DIV/0!	1	#DIV/0!
Mir1247|100526515	-1	#DIV/0!	1	#DIV/0!
Klrb1a|17057	-1	#DIV/0!	1	#DIV/0!
Mir194-2|723957	-1	#DIV/0!	1	#DIV/0!
A930003A15Rik|68162	-1	#DIV/0!	1	#DIV/0!
Zp4-ps|664793	-1	#DIV/0!	1	#DIV/0!
Olfr643|259081	-1	#DIV/0!	1	#DIV/0!
Vgll2|215031	-1	#DIV/0!	1	#DIV/0!
Myh13|544791	-1	#DIV/0!	1	#DIV/0!
D030018L15Rik|402773	-1	#DIV/0!	1	#DIV/0!
Olfr837|258558	-1	#DIV/0!	1	#DIV/0!
Gpr150|238725	-1	#DIV/0!	1	#DIV/0!
Gm4871|231885	-1	#DIV/0!	1	#DIV/0!
Olfr3|18328	-1	#DIV/0!	1	#DIV/0!
Vsx2|12677	-1	#DIV/0!	1	#DIV/0!
Mir669k|100316663	-1	#DIV/0!	1	#DIV/0!
Sh3gl3|20408	-1	#DIV/0!	1	#DIV/0!
4930503E14Rik|74954	-1	#DIV/0!	1	#DIV/0!
Fam71e2|243822	-1	#DIV/0!	1	#DIV/0!
Hyal6|74409	-1	#DIV/0!	1	#DIV/0!
Olfr66|18367	-1	#DIV/0!	1	#DIV/0!
Slc6a1|232333	-1	#DIV/0!	1	#DIV/0!
4930594C11Rik|77633	-1	#DIV/0!	1	#DIV/0!
Mir669a-2|735258	-1	#DIV/0!	1	#DIV/0!
Vmn2r27|232367	-1	#DIV/0!	1	#DIV/0!
Aqp6|11831	-1	#DIV/0!	1	#DIV/0!
Mir592|735266	-1	#DIV/0!	1	#DIV/0!
Elmod1|270162	-1	#DIV/0!	1	#DIV/0!
Gm16405|100040867	-1	#DIV/0!	1	#DIV/0!
Slc26a7|208890	-1	#DIV/0!	1	#DIV/0!
Fam124b|241128	-1	#DIV/0!	1	#DIV/0!
Tas2r131|387356	-1	#DIV/0!	1	#DIV/0!
4933425L06Rik|66763	-1	#DIV/0!	1	#DIV/0!
Hoxc5|15424	-1	#DIV/0!	1	#DIV/0!
Gm833|330004	-1	#DIV/0!	1	#DIV/0!
Arnt2|11864	-1	#DIV/0!	1	#DIV/0!
1700022I11Rik|67317	-1	#DIV/0!	1	#DIV/0!
Gm9159|668415	-1	#DIV/0!	1	#DIV/0!
Olfr1423|258675	-1	#DIV/0!	1	#DIV/0!
Slit3|20564	-1	#DIV/0!	1	#DIV/0!
Olfr458|258436	-1	#DIV/0!	1	#DIV/0!
Prdm8|77630	-1	#DIV/0!	1	#DIV/0!
9230110F15Rik|77080	-1	#DIV/0!	1	#DIV/0!
Olfr503|259143	-1	#DIV/0!	1	#DIV/0!
1110017D15Rik|73721	-1	#DIV/0!	1	#DIV/0!
Mir205|387201	-1	#DIV/0!	1	#DIV/0!
Snora69|104369	-1	#DIV/0!	1	#DIV/0!
Gm5538|433597	-1	#DIV/0!	1	#DIV/0!
A530098C11Rik|433294	-1	#DIV/0!	1	#DIV/0!
Mirlet7a-2|723965	-1	#DIV/0!	1	#DIV/0!
Etd|69501	-1	#DIV/0!	1	#DIV/0!
Mir883b|100124493	-1	#DIV/0!	1	#DIV/0!
Tex28|385380	-1	#DIV/0!	1	#DIV/0!

Olfr533|258056	-1	#DIV/0!	1	#DIV/0!
Gm3428|100041605	-1	#DIV/0!	1	#DIV/0!
Sec14l5|665119	-1	#DIV/0!	1	#DIV/0!
Adprhl1|234072	-1	#DIV/0!	1	#DIV/0!
Ppp1r36|210762	-1	#DIV/0!	1	#DIV/0!
6030422M02Rik|240697	-1	#DIV/0!	1	#DIV/0!
6720489N17Rik|211378	-1	#DIV/0!	1	#DIV/0!
Cldn16|114141	-1	#DIV/0!	1	#DIV/0!
Slc9a4|110895	-1	#DIV/0!	1	#DIV/0!
Unc5c|22253	-1	#DIV/0!	1	#DIV/0!
Tsnaxip1|72236	-1	#DIV/0!	1	#DIV/0!
6430411K18Rik|76880	-1	#DIV/0!	1	#DIV/0!
4930524B15Rik|67592	-1	#DIV/0!	1	#DIV/0!
Mir673|751547	-1	#DIV/0!	1	#DIV/0!
Gm12776|100043868	-1	#DIV/0!	1	#DIV/0!
Olfr1020|258573	-1	#DIV/0!	1	#DIV/0!
Mir147|387165	-1	#DIV/0!	1	#DIV/0!
4930432K09Rik|73779	-1	#DIV/0!	1	#DIV/0!
Ereg|13874	-1	#DIV/0!	1	#DIV/0!
Olfr49|18348	-1	#DIV/0!	1	#DIV/0!
Cndp1|338403	-1	#DIV/0!	1	#DIV/0!
Klra10|16628	-1	#DIV/0!	1	#DIV/0!
Mir449b|100190765	-1	#DIV/0!	1	#DIV/0!
Vmn1r170|546944	-1	#DIV/0!	1	#DIV/0!
4930474N05Rik|218921	-1	#DIV/0!	1	#DIV/0!
H60b|667281	-1	#DIV/0!	1	#DIV/0!
Gdf7|238057	-1	#DIV/0!	1	#DIV/0!
Olfr1275|257980	-1	#DIV/0!	1	#DIV/0!
Olfr522|258954	-1	#DIV/0!	1	#DIV/0!
Vmn1r195|171257	-1	#DIV/0!	1	#DIV/0!
Zp3|22788	-1	#DIV/0!	1	#DIV/0!
Otop2|237987	-1	#DIV/0!	1	#DIV/0!
Mir707|735269	-1	#DIV/0!	1	#DIV/0!
Tmem132d|243274	-1	#DIV/0!	1	#DIV/0!
Glipr1l1|69286	-1	#DIV/0!	1	#DIV/0!
Vmn1r169|100043103	-1	#DIV/0!	1	#DIV/0!
Olfr918|258372	-1	#DIV/0!	1	#DIV/0!
Lcn4|16821	-1	#DIV/0!	1	#DIV/0!
Cypt12|75439	-1	#DIV/0!	1	#DIV/0!
Gm17830|100415784	-1	#DIV/0!	1	#DIV/0!
Olfr69|18370	-1	#DIV/0!	1	#DIV/0!
Mir568|100124467	-1	#DIV/0!	1	#DIV/0!
Camp|12796	-1	#DIV/0!	1	#DIV/0!
Olfr815|258665	-1	#DIV/0!	1	#DIV/0!
Olfr535|258956	-1	#DIV/0!	1	#DIV/0!
1700045I19Rik|74264	-1	#DIV/0!	1	#DIV/0!
Mrgpra2b|235712	-1	#DIV/0!	1	#DIV/0!
Slc6a11|243616	-1	#DIV/0!	1	#DIV/0!
Tmod2|50876	-1	#DIV/0!	1	#DIV/0!
Olfr73|117004	-1	#DIV/0!	1	#DIV/0!
Sox1|20664	-1	#DIV/0!	1	#DIV/0!
Olfr1161|258845	-1	#DIV/0!	1	#DIV/0!
Olfr954|258328	-1	#DIV/0!	1	#DIV/0!

Gm10439|382243	-1	#DIV/0!	1	#DIV/0!
Serpinb3c|381286	-1	#DIV/0!	1	#DIV/0!
4922502D21Rik|381816	-1	#DIV/0!	1	#DIV/0!
Trpv6|64177	-1	#DIV/0!	1	#DIV/0!
Gpr22|73010	-1	#DIV/0!	1	#DIV/0!
Serpinb9g|93806	-1	#DIV/0!	1	#DIV/0!
Vmn1r117|667262	-1	#DIV/0!	1	#DIV/0!
Pou4f3|18998	-1	#DIV/0!	1	#DIV/0!
Olfr727|258316	-1	#DIV/0!	1	#DIV/0!
Bc1-ps1|12031	-1	#DIV/0!	1	#DIV/0!
Lrrc10|237560	-1	#DIV/0!	1	#DIV/0!
Gm12888|545677	-1	#DIV/0!	1	#DIV/0!
Gm7271|639545	-1	#DIV/0!	1	#DIV/0!
Olfr512|258719	-1	#DIV/0!	1	#DIV/0!
1700028P14Rik|67483	-1	#DIV/0!	1	#DIV/0!
Dlx6as2|13397	-1	#DIV/0!	1	#DIV/0!
Shcbp1|20419	-1	#DIV/0!	1	#DIV/0!
Olfr1411|258483	-1	#DIV/0!	1	#DIV/0!
Galnt5|241391	-1	#DIV/0!	1	#DIV/0!
Odf4|252868	-1	#DIV/0!	1	#DIV/0!
Vmn1r234|171232	-1	#DIV/0!	1	#DIV/0!
Olfr175-ps1|259004	-1	#DIV/0!	1	#DIV/0!
E330021D16Rik|100502936	-1	#DIV/0!	1	#DIV/0!
Krt13|16663	-1	#DIV/0!	1	#DIV/0!
Olfr597|258135	-1	#DIV/0!	1	#DIV/0!
Vax2os1|574519	-1	#DIV/0!	1	#DIV/0!
Vmn1r197|171278	-1	#DIV/0!	1	#DIV/0!
Stmn3|20262	-1	#DIV/0!	1	#DIV/0!
Ifna11|15964	-1	#DIV/0!	1	#DIV/0!
F2rl3|14065	-1	#DIV/0!	1	#DIV/0!
Abpg|110187	-1	#DIV/0!	1	#DIV/0!
Plb1|665270	-1	#DIV/0!	1	#DIV/0!
Mir1896|100316752	-1	#DIV/0!	1	#DIV/0!
Mir32|723837	-1	#DIV/0!	1	#DIV/0!
Gm15091|382244	-1	#DIV/0!	1	#DIV/0!
Olfr1122|259033	-1	#DIV/0!	1	#DIV/0!
Olfr1012|258561	-1	#DIV/0!	1	#DIV/0!
Pvalb|19293	-1	#DIV/0!	1	#DIV/0!
Mir3081|100526478	-1	#DIV/0!	1	#DIV/0!
Alx4|11695	-1	#DIV/0!	1	#DIV/0!
Ms4a15|545279	-1	#DIV/0!	1	#DIV/0!
Olfr381|259024	-1	#DIV/0!	1	#DIV/0!
Col17a1|12821	-1	#DIV/0!	1	#DIV/0!
Zar1|317755	-1	#DIV/0!	1	#DIV/0!
Gm2012|100039030	-1	#DIV/0!	1	#DIV/0!
1700001L19Rik|69315	-1	#DIV/0!	1	#DIV/0!
Gm12830|433746	-1	#DIV/0!	1	#DIV/0!
Tmed11|67366	-1	#DIV/0!	1	#DIV/0!
Olfr808|258930	-1	#DIV/0!	1	#DIV/0!
Mir767|100316827	-1	#DIV/0!	1	#DIV/0!
Vstm2a|211739	-1	#DIV/0!	1	#DIV/0!
4930433I11Rik|243944	-1	#DIV/0!	1	#DIV/0!
Rprl2|19784	-1	#DIV/0!	1	#DIV/0!

Cdh19|227485	-1	#DIV/0!	1	#DIV/0!
Gm5128|331529	-1	#DIV/0!	1	#DIV/0!
Gm14920|100039319	-1	#DIV/0!	1	#DIV/0!
Fam5c|215378	-1	#DIV/0!	1	#DIV/0!
Gm266|212539	-1	#DIV/0!	1	#DIV/0!
Fpr-rs6|321020	-1	#DIV/0!	1	#DIV/0!
Olfr212|258019	-1	#DIV/0!	1	#DIV/0!
4930550L24Rik|75352	-1	#DIV/0!	1	#DIV/0!
Dlx6|13396	-1	#DIV/0!	1	#DIV/0!
Ms4a10|69826	-1	#DIV/0!	1	#DIV/0!
Mir203|387199	-1	#DIV/0!	1	#DIV/0!
Gm14725|628053	-1	#DIV/0!	1	#DIV/0!
Dok5|76829	-1	#DIV/0!	1	#DIV/0!
Mir1912|100526529	-1	#DIV/0!	1	#DIV/0!
Fhl5|57756	-1	#DIV/0!	1	#DIV/0!
Olfr1048|259016	-1	#DIV/0!	1	#DIV/0!
Mir3095|100526502	-1	#DIV/0!	1	#DIV/0!
Vmn1r54|113851	-1	#DIV/0!	1	#DIV/0!
Wnt8b|22423	-1	#DIV/0!	1	#DIV/0!
Cntnap5b|241175	-1	#DIV/0!	1	#DIV/0!
Kank4|242553	-1	#DIV/0!	1	#DIV/0!
Olfr472|258770	-1	#DIV/0!	1	#DIV/0!
Rfpl3s|75258	-1	#DIV/0!	1	#DIV/0!
1700023A16Rik|69371	-1	#DIV/0!	1	#DIV/0!
Vmn1r68|628580	-1	#DIV/0!	1	#DIV/0!
Olfr169|258158	-1	#DIV/0!	1	#DIV/0!
Krtap22-2|68740	-1	#DIV/0!	1	#DIV/0!
Hs6st3|50787	-1	#DIV/0!	1	#DIV/0!
Cfc1|12627	-1	#DIV/0!	1	#DIV/0!
Olfr488|258727	-1	#DIV/0!	1	#DIV/0!
Olfr1437|258117	-1	#DIV/0!	1	#DIV/0!
Rgs22|626596	-1	#DIV/0!	1	#DIV/0!
Cacna1b|12287	-1	#DIV/0!	1	#DIV/0!
Olfr97|258505	-1	#DIV/0!	1	#DIV/0!
Stac|20840	-1	#DIV/0!	1	#DIV/0!
Mir202|387198	-1	#DIV/0!	1	#DIV/0!
Emx2os|329078	-1	#DIV/0!	1	#DIV/0!
Vmn2r112|628185	-1	#DIV/0!	1	#DIV/0!
Tmem210|78217	-1	#DIV/0!	1	#DIV/0!
Olfr1205|258898	-1	#DIV/0!	1	#DIV/0!
Adamts20|223838	-1	#DIV/0!	1	#DIV/0!
Sln|66402	-1	#DIV/0!	1	#DIV/0!
Katnal2|71206	-1	#DIV/0!	1	#DIV/0!
Klk1b16|16615	-1	#DIV/0!	1	#DIV/0!
Gm5415|408191	-1	#DIV/0!	1	#DIV/0!
Mafa|378435	-1	#DIV/0!	1	#DIV/0!
Gm14151|433486	-1	#DIV/0!	1	#DIV/0!
Tas2r139|353148	-1	#DIV/0!	1	#DIV/0!
Wfdc8|277343	-1	#DIV/0!	1	#DIV/0!
Mir432|100316735	-1	#DIV/0!	1	#DIV/0!
Dazl|13164	-1	#DIV/0!	1	#DIV/0!
Gm6880|628456	-1	#DIV/0!	1	#DIV/0!
Asb9|69299	-1	#DIV/0!	1	#DIV/0!

Sva|20939	-1	#DIV/0!	1	#DIV/0!
Olfr1131|258652	-1	#DIV/0!	1	#DIV/0!
Olfr153|110511	-1	#DIV/0!	1	#DIV/0!
Rhox2e|100040016	-1	#DIV/0!	1	#DIV/0!
E330012B07Rik|100039641	-1	#DIV/0!	1	#DIV/0!
Lpar3|65086	-1	#DIV/0!	1	#DIV/0!
9430031J16Rik|241134	-1	#DIV/0!	1	#DIV/0!
Fbxw19|235612	-1	#DIV/0!	1	#DIV/0!
Spdyb|74673	-1	#DIV/0!	1	#DIV/0!
Dnaic2|432611	-1	#DIV/0!	1	#DIV/0!
Hcrtr2|387285	-1	#DIV/0!	1	#DIV/0!
Olfr1031|257916	-1	#DIV/0!	1	#DIV/0!
Gm13011|242711	-1	#DIV/0!	1	#DIV/0!
Mir615|751557	-1	#DIV/0!	1	#DIV/0!
Olfr688|259161	-1	#DIV/0!	1	#DIV/0!
Olfr279|258502	-1	#DIV/0!	1	#DIV/0!
Vmn1r77|628946	-1	#DIV/0!	1	#DIV/0!
Calcoco2|76815	-1	#DIV/0!	1	#DIV/0!
Olfr24|18322	-1	#DIV/0!	1	#DIV/0!
Olfr106-ps|257925	-1	#DIV/0!	1	#DIV/0!
Pcdhgc5|93708	-1	#DIV/0!	1	#DIV/0!
Mir3060|100526466	-1	#DIV/0!	1	#DIV/0!
Vmn1r121|667240	-1	#DIV/0!	1	#DIV/0!
Olfr1351|259042	-1	#DIV/0!	1	#DIV/0!
Nefm|18040	-1	#DIV/0!	1	#DIV/0!
Pnliprp1|18946	-1	#DIV/0!	1	#DIV/0!
Tacr2|21337	-1	#DIV/0!	1	#DIV/0!
Gm7337|654494	-1	#DIV/0!	1	#DIV/0!
AI593442|330941	-1	#DIV/0!	1	#DIV/0!
Svs3a|64335	-1	#DIV/0!	1	#DIV/0!
Otogl|628870	-1	#DIV/0!	1	#DIV/0!
4833423E24Rik|228151	-1	#DIV/0!	1	#DIV/0!
Cyp24a1|13081	-1	#DIV/0!	1	#DIV/0!
Hdgfl1|15192	-1	#DIV/0!	1	#DIV/0!
Snord1b|100216531	-1	#DIV/0!	1	#DIV/0!
Mir218-1|723822	-1	#DIV/0!	1	#DIV/0!
Dsc1|13505	-1	#DIV/0!	1	#DIV/0!
Vmn2r102|224572	-1	#DIV/0!	1	#DIV/0!
Gm14743|236874	-1	#DIV/0!	1	#DIV/0!
Tcl1b1|27379	-1	#DIV/0!	1	#DIV/0!
Olfr417|258238	-1	#DIV/0!	1	#DIV/0!
Magea4|17140	-1	#DIV/0!	1	#DIV/0!
Car12|76459	-1	#DIV/0!	1	#DIV/0!
Gm5|194292	-1	#DIV/0!	1	#DIV/0!
Fgf20|80857	-1	#DIV/0!	1	#DIV/0!
Gm6890|628518	-1	#DIV/0!	1	#DIV/0!
Olfr1330|258331	-1	#DIV/0!	1	#DIV/0!
Vmn1r9|171203	-1	#DIV/0!	1	#DIV/0!
Rasgrf1|19417	-1	#DIV/0!	1	#DIV/0!
Olfr1154|258641	-1	#DIV/0!	1	#DIV/0!
4930428E23Rik|434800	-1	#DIV/0!	1	#DIV/0!
Slc26a3|13487	-1	#DIV/0!	1	#DIV/0!
Olfr1000|257899	-1	#DIV/0!	1	#DIV/0!

Fam159a|545667	-1	#DIV/0!	1	#DIV/0!
Mir130b|723816	-1	#DIV/0!	1	#DIV/0!
Igfl3|232925	-1	#DIV/0!	1	#DIV/0!
Tph2|216343	-1	#DIV/0!	1	#DIV/0!
Il1f6|54448	-1	#DIV/0!	1	#DIV/0!
Mir804|100049547	-1	#DIV/0!	1	#DIV/0!
Gm14139|100271882	-1	#DIV/0!	1	#DIV/0!
Mir381|723935	-1	#DIV/0!	1	#DIV/0!
Defb44-ps|654454	-1	#DIV/0!	1	#DIV/0!
Ifnz|319146	-1	#DIV/0!	1	#DIV/0!
Vmn1r36|171184	-1	#DIV/0!	1	#DIV/0!
Inpp4b|234515	-1	#DIV/0!	1	#DIV/0!
Vmn2r22|546913	-1	#DIV/0!	1	#DIV/0!
Tpsg1|26945	-1	#DIV/0!	1	#DIV/0!
3425401B19Rik|100504518	-1	#DIV/0!	1	#DIV/0!
Mir2861|100499514	-1	#DIV/0!	1	#DIV/0!
Vmn2r88|669149	-1	#DIV/0!	1	#DIV/0!
Dbx1|13172	-1	#DIV/0!	1	#DIV/0!
Vmn2r94|665227	-1	#DIV/0!	1	#DIV/0!
Mir1934|100316840	-1	#DIV/0!	1	#DIV/0!
Gata1|14460	-1	#DIV/0!	1	#DIV/0!
Olfr1066|257880	-1	#DIV/0!	1	#DIV/0!
Vmn2r118|383258	-1	#DIV/0!	1	#DIV/0!
Prlhr|226278	-1	#DIV/0!	1	#DIV/0!
Abpb|233099	-1	#DIV/0!	1	#DIV/0!
4930512B01Rik|74724	-1	#DIV/0!	1	#DIV/0!
Olfr1151|258631	-1	#DIV/0!	1	#DIV/0!
Armc4|74934	-1	#DIV/0!	1	#DIV/0!
H2-M9|14997	-1	#DIV/0!	1	#DIV/0!
Mir1a-1|387136	-1	#DIV/0!	1	#DIV/0!
Olfr209|404311	-1	#DIV/0!	1	#DIV/0!
Ear10|93725	-1	#DIV/0!	1	#DIV/0!
Prdm13|230025	-1	#DIV/0!	1	#DIV/0!
D330045A20Rik|102871	-1	#DIV/0!	1	#DIV/0!
Tspan6|56496	-1	#DIV/0!	1	#DIV/0!
Ptgdr|19214	-1	#DIV/0!	1	#DIV/0!
Tex13a|67944	-1	#DIV/0!	1	#DIV/0!
Scn1a|20265	-1	#DIV/0!	1	#DIV/0!
Col28a1|213945	-1	#DIV/0!	1	#DIV/0!
Ccdc11|74453	-1	#DIV/0!	1	#DIV/0!
Gm14548|100038909	-1	#DIV/0!	1	#DIV/0!
Zp1|22786	-1	#DIV/0!	1	#DIV/0!
Gng13|64337	-1	#DIV/0!	1	#DIV/0!
Gm5592|434172	-1	#DIV/0!	1	#DIV/0!
Spock3|72902	-1	#DIV/0!	1	#DIV/0!
AY512915|414067	-1	#DIV/0!	1	#DIV/0!
Olfr1308|258258	-1	#DIV/0!	1	#DIV/0!
Rnf148|71300	-1	#DIV/0!	1	#DIV/0!
1700110M21Rik|76627	-1	#DIV/0!	1	#DIV/0!
Shisa7|232813	-1	#DIV/0!	1	#DIV/0!
Sox8|20681	-1	#DIV/0!	1	#DIV/0!
Svs1|243377	-1	#DIV/0!	1	#DIV/0!
Tpo|22018	-1	#DIV/0!	1	#DIV/0!

Syt10|54526	-1	#DIV/0!	1	#DIV/0!
Pcdhac1|353236	-1	#DIV/0!	1	#DIV/0!
Mir365-2|723853	-1	#DIV/0!	1	#DIV/0!
Nt5c1a|230718	-1	#DIV/0!	1	#DIV/0!
Dlx1|13390	-1	#DIV/0!	1	#DIV/0!
Clec12b|71183	-1	#DIV/0!	1	#DIV/0!
Vmn1r215|171253	-1	#DIV/0!	1	#DIV/0!
Timp1|21857	-1	#DIV/0!	1	#DIV/0!
Kcnmb4|58802	-1	#DIV/0!	1	#DIV/0!
Ccdc27|381580	-1	#DIV/0!	1	#DIV/0!
4933400C05Rik|328019	-1	#DIV/0!	1	#DIV/0!
Il22|50929	-1	#DIV/0!	1	#DIV/0!
Kcne1l|66240	-1	#DIV/0!	1	#DIV/0!
Nlrp4a|243880	-1	#DIV/0!	1	#DIV/0!
Slc17a6|140919	-1	#DIV/0!	1	#DIV/0!
Mir134|387152	-1	#DIV/0!	1	#DIV/0!
Olfr809|258321	-1	#DIV/0!	1	#DIV/0!
Olfr659|259052	-1	#DIV/0!	1	#DIV/0!
Mei1|74369	-1	#DIV/0!	1	#DIV/0!
Mir297a-3|100124482	-1	#DIV/0!	1	#DIV/0!
Kir3dl1|245616	-1	#DIV/0!	1	#DIV/0!
4930558C23Rik|67654	-1	#DIV/0!	1	#DIV/0!
4932411N23Rik|237029	-1	#DIV/0!	1	#DIV/0!
1700026D08Rik|75556	-1	#DIV/0!	1	#DIV/0!
1700042G07Rik|67323	-1	#DIV/0!	1	#DIV/0!
Elfn2|207393	-1	#DIV/0!	1	#DIV/0!
Olfr370|258267	-1	#DIV/0!	1	#DIV/0!
Olfr821|258772	-1	#DIV/0!	1	#DIV/0!
V1rd18|404288	-1	#DIV/0!	1	#DIV/0!
Olfr914|258782	-1	#DIV/0!	1	#DIV/0!
Defa24|503491	-1	#DIV/0!	1	#DIV/0!
Olfr1215|258451	-1	#DIV/0!	1	#DIV/0!
Mir133b|723817	-1	#DIV/0!	1	#DIV/0!
Gm5126|331480	-1	#DIV/0!	1	#DIV/0!
Cdsn|386463	-1	#DIV/0!	1	#DIV/0!
Cxcl3|330122	-1	#DIV/0!	1	#DIV/0!
Vmn2r76|675969	-1	#DIV/0!	1	#DIV/0!
Gm136|214568	-1	#DIV/0!	1	#DIV/0!
Olfr1188|258921	-1	#DIV/0!	1	#DIV/0!
Olfr1009|258565	-1	#DIV/0!	1	#DIV/0!
Ccdc116|76872	-1	#DIV/0!	1	#DIV/0!
Olfr139|259005	-1	#DIV/0!	1	#DIV/0!
Myog|17928	-1	#DIV/0!	1	#DIV/0!
Ccdc154|207209	-1	#DIV/0!	1	#DIV/0!
Musk|18198	-1	#DIV/0!	1	#DIV/0!
Slc46a2|30936	-1	#DIV/0!	1	#DIV/0!
Gm10280|791378	-1	#DIV/0!	1	#DIV/0!
Pou4f2|18997	-1	#DIV/0!	1	#DIV/0!
Zc3h12b|547176	-1	#DIV/0!	1	#DIV/0!
Vmn1r104|667135	-1	#DIV/0!	1	#DIV/0!
5033404E19Rik|114668	-1	#DIV/0!	1	#DIV/0!
Olfr641|259075	-1	#DIV/0!	1	#DIV/0!
Krtap16-5|77918	-1	#DIV/0!	1	#DIV/0!

Psg-ps1|232919	-1	#DIV/0!	1	#DIV/0!
4930515G16Rik|75070	-1	#DIV/0!	1	#DIV/0!
Olfr1461|258299	-1	#DIV/0!	1	#DIV/0!
Wap|22373	-1	#DIV/0!	1	#DIV/0!
Zfp300|245368	-1	#DIV/0!	1	#DIV/0!
Tulp1|22157	-1	#DIV/0!	1	#DIV/0!
Hhla1|654498	-1	#DIV/0!	1	#DIV/0!
Tktl1|83553	-1	#DIV/0!	1	#DIV/0!
Olfr889|258475	-1	#DIV/0!	1	#DIV/0!
Tssk3|58864	-1	#DIV/0!	1	#DIV/0!
Taar7d|435206	-1	#DIV/0!	1	#DIV/0!
Tas2r136|353165	-1	#DIV/0!	1	#DIV/0!
Olfr613|259104	-1	#DIV/0!	1	#DIV/0!
Krtap4-1|665891	-1	#DIV/0!	1	#DIV/0!
Dapl1|76747	-1	#DIV/0!	1	#DIV/0!
Psg26|574429	-1	#DIV/0!	1	#DIV/0!
Plag1|56711	-1	#DIV/0!	1	#DIV/0!
Fam170a|225497	-1	#DIV/0!	1	#DIV/0!
Snora7a|100217451	-1	#DIV/0!	1	#DIV/0!
Snord116|64243	-1	#DIV/0!	1	#DIV/0!
Mrgprb4|233230	-1	#DIV/0!	1	#DIV/0!
Triml2|622117	-1	#DIV/0!	1	#DIV/0!
Snord47|100217446	-1	#DIV/0!	1	#DIV/0!
Ptprh|545902	-1	#DIV/0!	1	#DIV/0!
Tepp|73407	-1	#DIV/0!	1	#DIV/0!
Olfr749|56858	-1	#DIV/0!	1	#DIV/0!
Ccdc18|73254	-1	#DIV/0!	1	#DIV/0!
Fgf3|14174	-1	#DIV/0!	1	#DIV/0!
Olfr1412|258274	-1	#DIV/0!	1	#DIV/0!
Mir1b|100316783	-1	#DIV/0!	1	#DIV/0!
Gm5347|384814	-1	#DIV/0!	1	#DIV/0!
2810433D01Rik|77132	-1	#DIV/0!	1	#DIV/0!
Olfr1290|257662	-1	#DIV/0!	1	#DIV/0!
Snord65|100217444	-1	#DIV/0!	1	#DIV/0!
Klra13-ps|16631	-1	#DIV/0!	1	#DIV/0!
Mir683-1|751559	-1	#DIV/0!	1	#DIV/0!
1500015O10Rik|78896	-1	#DIV/0!	1	#DIV/0!
1700031M16Rik|73302	-1	#DIV/0!	1	#DIV/0!
Mak|17152	-1	#DIV/0!	1	#DIV/0!
Vmn1r11|113860	-1	#DIV/0!	1	#DIV/0!
Snora61|100217440	-1	#DIV/0!	1	#DIV/0!
Nr2e1|21907	-1	#DIV/0!	1	#DIV/0!
Ccl26|541307	-1	#DIV/0!	1	#DIV/0!
Neurod2|18013	-1	#DIV/0!	1	#DIV/0!
Prl3d1|18775	-1	#DIV/0!	1	#DIV/0!
Tecrl|243078	-1	#DIV/0!	1	#DIV/0!
Adamts19|240322	-1	#DIV/0!	1	#DIV/0!
Kcnh8|211468	-1	#DIV/0!	1	#DIV/0!
1700034J05Rik|73344	-1	#DIV/0!	1	#DIV/0!
Gm15881|100271928	-1	#DIV/0!	1	#DIV/0!
Olfr295|258850	-1	#DIV/0!	1	#DIV/0!
Styk1|243659	-1	#DIV/0!	1	#DIV/0!
Mir706|735290	-1	#DIV/0!	1	#DIV/0!

Lce3b|66344	-1	#DIV/0!	1	#DIV/0!
Gzmd|14941	-1	#DIV/0!	1	#DIV/0!
Klra17|170733	-1	#DIV/0!	1	#DIV/0!
4930579C15Rik|67753	-1	#DIV/0!	1	#DIV/0!
Tmco2|69469	-1	#DIV/0!	1	#DIV/0!
Crygs|12970	-1	#DIV/0!	1	#DIV/0!
Vmn1r22|171196	-1	#DIV/0!	1	#DIV/0!
Olfr1105|258085	-1	#DIV/0!	1	#DIV/0!
Fam187a|66784	-1	#DIV/0!	1	#DIV/0!
Tdpoz1|207213	-1	#DIV/0!	1	#DIV/0!
Olfr1143|258290	-1	#DIV/0!	1	#DIV/0!
Olfr1056|259020	-1	#DIV/0!	1	#DIV/0!
Olfr694|258444	-1	#DIV/0!	1	#DIV/0!
Olfr1513|258008	-1	#DIV/0!	1	#DIV/0!
Cel|12613	-1	#DIV/0!	1	#DIV/0!
Vash2|226841	-1	#DIV/0!	1	#DIV/0!
Snord42a|100529076	-1	#DIV/0!	1	#DIV/0!
G6pc2|14378	-1	#DIV/0!	1	#DIV/0!
Sycp1|20957	-1	#DIV/0!	1	#DIV/0!
Krtap16-3|71369	-1	#DIV/0!	1	#DIV/0!
Gm5771|436523	-1	#DIV/0!	1	#DIV/0!
Mir1945|100316833	-1	#DIV/0!	1	#DIV/0!
Mirlet7b|387245	-1	#DIV/0!	1	#DIV/0!
Olfr18|18315	-1	#DIV/0!	1	#DIV/0!
Olfr1507|57269	-1	#DIV/0!	1	#DIV/0!
Olfr311|258530	-1	#DIV/0!	1	#DIV/0!
4930555G01Rik|108978	-1	#DIV/0!	1	#DIV/0!
Svs6|20945	-1	#DIV/0!	1	#DIV/0!
Trhde|237553	-1	#DIV/0!	1	#DIV/0!
Duox1|99439	-1	#DIV/0!	1	#DIV/0!
Olfr504|258163	-1	#DIV/0!	1	#DIV/0!
Vmn1r219|171272	-1	#DIV/0!	1	#DIV/0!
6330527O06Rik|76161	-1	#DIV/0!	1	#DIV/0!
Zfp711|245595	-1	#DIV/0!	1	#DIV/0!
Tdrd5|214575	-1	#DIV/0!	1	#DIV/0!
Olfr478|258729	-1	#DIV/0!	1	#DIV/0!
Olfr427|259162	-1	#DIV/0!	1	#DIV/0!
T|20997	-1	#DIV/0!	1	#DIV/0!
Gabra4|14397	-1	#DIV/0!	1	#DIV/0!
1700016M24Rik|69439	-1	#DIV/0!	1	#DIV/0!
Trim50|215061	-1	#DIV/0!	1	#DIV/0!
Olfr161|258859	-1	#DIV/0!	1	#DIV/0!
Nxph4|104080	-1	#DIV/0!	1	#DIV/0!
Kcnq3|110862	-1	#DIV/0!	1	#DIV/0!
Lcn5|13863	-1	#DIV/0!	1	#DIV/0!
Eya1|14048	-1	#DIV/0!	1	#DIV/0!
Satl1|73809	-1	#DIV/0!	1	#DIV/0!
Ctrl|109660	-1	#DIV/0!	1	#DIV/0!
Krt28|70843	-1	#DIV/0!	1	#DIV/0!
Olfr825|258672	-1	#DIV/0!	1	#DIV/0!
Mir124a-1|387233	-1	#DIV/0!	1	#DIV/0!
Tmem45b|235135	-1	#DIV/0!	1	#DIV/0!
Vmn1r174|404291	-1	#DIV/0!	1	#DIV/0!

Kprp|433619	-1	#DIV/0!	1	#DIV/0!
Dusp21|73547	-1	#DIV/0!	1	#DIV/0!
Krtap16-1|170651	-1	#DIV/0!	1	#DIV/0!
Opalin|226115	-1	#DIV/0!	1	#DIV/0!
Gm362|236774	-1	#DIV/0!	1	#DIV/0!
Mir672|751535	-1	#DIV/0!	1	#DIV/0!
Slfn4|20558	-1	#DIV/0!	1	#DIV/0!
4930430D24Rik|194735	-1	#DIV/0!	1	#DIV/0!
Speer9-ps1|73949	-1	#DIV/0!	1	#DIV/0!
Olfr571|259089	-1	#DIV/0!	1	#DIV/0!
Gpr142|217302	-1	#DIV/0!	1	#DIV/0!
Gm14461|329436	-1	#DIV/0!	1	#DIV/0!
Vsig8|240916	-1	#DIV/0!	1	#DIV/0!
Mir201|387197	-1	#DIV/0!	1	#DIV/0!
Stra8|20899	-1	#DIV/0!	1	#DIV/0!
Klrg1|50928	-1	#DIV/0!	1	#DIV/0!
4931429L15Rik|74361	-1	#DIV/0!	1	#DIV/0!
Mir721|723975	-1	#DIV/0!	1	#DIV/0!
Zdhhc19|245308	-1	#DIV/0!	1	#DIV/0!
Pla2g5|18784	-1	#DIV/0!	1	#DIV/0!
Sall4|99377	-1	#DIV/0!	1	#DIV/0!
Tas2r144|387515	-1	#DIV/0!	1	#DIV/0!
Olfr352|258942	-1	#DIV/0!	1	#DIV/0!
Olfr1371|276865	-1	#DIV/0!	1	#DIV/0!
Alox12b|11686	-1	#DIV/0!	1	#DIV/0!
Olfr1045|259019	-1	#DIV/0!	1	#DIV/0!
Hormad1|67981	-1	#DIV/0!	1	#DIV/0!
4930417O13Rik|73870	-1	#DIV/0!	1	#DIV/0!
Olfr1357|257883	-1	#DIV/0!	1	#DIV/0!
Chrng|11449	-1	#DIV/0!	1	#DIV/0!
Gm10789|100038709	-1	#DIV/0!	1	#DIV/0!
Ctrb1|66473	-1	#DIV/0!	1	#DIV/0!
Ear6|93719	-1	#DIV/0!	1	#DIV/0!
Snord15b|449631	-1	#DIV/0!	1	#DIV/0!
Cryge|12968	-1	#DIV/0!	1	#DIV/0!
Gm4745|194588	-1	#DIV/0!	1	#DIV/0!
Mir470|723873	-1	#DIV/0!	1	#DIV/0!
Mir1894|100316810	-1	#DIV/0!	1	#DIV/0!
Irx4|50916	-1	#DIV/0!	1	#DIV/0!
Cldn13|57255	-1	#DIV/0!	1	#DIV/0!
Mcpt-ps1|17223	-1	#DIV/0!	1	#DIV/0!
Ucma|68527	-1	#DIV/0!	1	#DIV/0!
Olfr1253|258370	-1	#DIV/0!	1	#DIV/0!
Nxph1|18231	-1	#DIV/0!	1	#DIV/0!
Gdpd2|71584	-1	#DIV/0!	1	#DIV/0!
1700028M03Rik|69460	-1	#DIV/0!	1	#DIV/0!
Olfr56|18356	-1	#DIV/0!	1	#DIV/0!
Npbwr1|226304	-1	#DIV/0!	1	#DIV/0!
Olfr638|259124	-1	#DIV/0!	1	#DIV/0!
Lrp2|14725	-1	#DIV/0!	1	#DIV/0!
Phxr4|18689	-1	#DIV/0!	1	#DIV/0!
Mir453|100124484	-1	#DIV/0!	1	#DIV/0!
Gm6026|547210	-1	#DIV/0!	1	#DIV/0!

Olfr653|57250	-1	#DIV/0!	1	#DIV/0!
Reg3g|19695	-1	#DIV/0!	1	#DIV/0!
BB014433|434285	-1	#DIV/0!	1	#DIV/0!
Zswim2|71861	-1	#DIV/0!	1	#DIV/0!
Tmem200a|77220	-1	#DIV/0!	1	#DIV/0!
Olfr358|227789	-1	#DIV/0!	1	#DIV/0!
Gm4814|100502942	-1	#DIV/0!	1	#DIV/0!
9430070O13Rik|77352	-1	#DIV/0!	1	#DIV/0!
Vmn2r33|624512	-1	#DIV/0!	1	#DIV/0!
Olfr1495|258341	-1	#DIV/0!	1	#DIV/0!
Defb20|319579	-1	#DIV/0!	1	#DIV/0!
Odz4|23966	-1	#DIV/0!	1	#DIV/0!
Klk1b26|16618	-1	#DIV/0!	1	#DIV/0!
4930470P17Rik|67637	-1	#DIV/0!	1	#DIV/0!
1810065E05Rik|69864	-1	#DIV/0!	1	#DIV/0!
Chi3l4|104183	-1	#DIV/0!	1	#DIV/0!
Gm12887|666927	-1	#DIV/0!	1	#DIV/0!
Prnd|26434	-1	#DIV/0!	1	#DIV/0!
Mir23a|387216	-1	#DIV/0!	1	#DIV/0!
1700018B24Rik|66332	-1	#DIV/0!	1	#DIV/0!
Vmn1r179|404286	-1	#DIV/0!	1	#DIV/0!
Arl13a|74448	-1	#DIV/0!	1	#DIV/0!
Snord95|100216540	-1	#DIV/0!	1	#DIV/0!
Mageb4|434903	-1	#DIV/0!	1	#DIV/0!
Slc25a41|103775	-1	#DIV/0!	1	#DIV/0!
Mir598|100124452	-1	#DIV/0!	1	#DIV/0!
Olfr435|258647	-1	#DIV/0!	1	#DIV/0!
Ttc21a|74052	-1	#DIV/0!	1	#DIV/0!
Cldn18|56492	-1	#DIV/0!	1	#DIV/0!
Rhox4b|57737	-1	#DIV/0!	1	#DIV/0!
Olfr1111|258586	-1	#DIV/0!	1	#DIV/0!
Krtap31-2|432602	-1	#DIV/0!	1	#DIV/0!
Iqch|78250	-1	#DIV/0!	1	#DIV/0!
Mir98|723947	-1	#DIV/0!	1	#DIV/0!
4930426L09Rik|74629	-1	#DIV/0!	1	#DIV/0!
Prl2c5|107849	-1	#DIV/0!	1	#DIV/0!
Olfr1333|258265	-1	#DIV/0!	1	#DIV/0!
Akp3|11648	-1	#DIV/0!	1	#DIV/0!
4933417A18Rik|66761	-1	#DIV/0!	1	#DIV/0!
Spaca1|67652	-1	#DIV/0!	1	#DIV/0!
Olfr1028|257936	-1	#DIV/0!	1	#DIV/0!
Mir873|100124457	-1	#DIV/0!	1	#DIV/0!
Mir501|751560	-1	#DIV/0!	1	#DIV/0!
Krt27|16675	-1	#DIV/0!	1	#DIV/0!
Asz1|74068	-1	#DIV/0!	1	#DIV/0!
Olfr1294|258887	-1	#DIV/0!	1	#DIV/0!
Defb5|81007	-1	#DIV/0!	1	#DIV/0!
Sele|20339	-1	#DIV/0!	1	#DIV/0!
Spata16|70862	-1	#DIV/0!	1	#DIV/0!
Bglap|12096	-1	#DIV/0!	1	#DIV/0!
Wnt8a|20890	-1	#DIV/0!	1	#DIV/0!
Zfp457|431706	-1	#DIV/0!	1	#DIV/0!
1700029P11Rik|66346	-1	#DIV/0!	1	#DIV/0!

Olfr1089|257933	-1	#DIV/0!	1	#DIV/0!
Gm6406|623186	-1	#DIV/0!	1	#DIV/0!
Olfr1297|258890	-1	#DIV/0!	1	#DIV/0!
Olfr1446|258699	-1	#DIV/0!	1	#DIV/0!
Oca2|18431	-1	#DIV/0!	1	#DIV/0!
Kcns2|16539	-1	#DIV/0!	1	#DIV/0!
Gdpd3|68616	-1	#DIV/0!	1	#DIV/0!
Tbx15|21384	-1	#DIV/0!	1	#DIV/0!
Olfr807|258931	-1	#DIV/0!	1	#DIV/0!
Hpdl|242642	-1	#DIV/0!	1	#DIV/0!
Olfr816|258667	-1	#DIV/0!	1	#DIV/0!
Fosl1|14283	-1	#DIV/0!	1	#DIV/0!
Snord68|100302565	-1	#DIV/0!	1	#DIV/0!
Olfr145|258310	-1	#DIV/0!	1	#DIV/0!
Lipm|78753	-1	#DIV/0!	1	#DIV/0!
Vwa5b2|328643	-1	#DIV/0!	1	#DIV/0!
Mir671|735264	-1	#DIV/0!	1	#DIV/0!
AI747448|99709	-1	#DIV/0!	1	#DIV/0!
Olfr860|258521	-1	#DIV/0!	1	#DIV/0!
Snord53|100217456	-1	#DIV/0!	1	#DIV/0!
Olfr690|56860	-1	#DIV/0!	1	#DIV/0!
Gm15107|434864	-1	#DIV/0!	1	#DIV/0!
Cpa3|12873	-1	#DIV/0!	1	#DIV/0!
Vmn1r16|171202	-1	#DIV/0!	1	#DIV/0!
Fgf14|14169	-1	#DIV/0!	1	#DIV/0!
Fcrlb|435653	-1	#DIV/0!	1	#DIV/0!
Snora21|100302498	-1	#DIV/0!	1	#DIV/0!
Samd7|75953	-1	#DIV/0!	1	#DIV/0!
Vmn1r225|171228	-1	#DIV/0!	1	#DIV/0!
Tmprss11f|243083	-1	#DIV/0!	1	#DIV/0!
Olfr624|258189	-1	#DIV/0!	1	#DIV/0!
Olfr178|258999	-1	#DIV/0!	1	#DIV/0!
Drd3|13490	-1	#DIV/0!	1	#DIV/0!
Hist1h2bl|319185	-1	#DIV/0!	1	#DIV/0!
Muc13|17063	-1	#DIV/0!	1	#DIV/0!
Ccnb3|209091	-1	#DIV/0!	1	#DIV/0!
Cdhr4|69398	-1	#DIV/0!	1	#DIV/0!
Slc32a1|22348	-1	#DIV/0!	1	#DIV/0!
Ccdc87|399599	-1	#DIV/0!	1	#DIV/0!
Prrxl1|107751	-1	#DIV/0!	1	#DIV/0!
Lyg1|69541	-1	#DIV/0!	1	#DIV/0!
Vmn1r44|113854	-1	#DIV/0!	1	#DIV/0!
Pla2g2e|26970	-1	#DIV/0!	1	#DIV/0!
Gm9871|207157	-1	#DIV/0!	1	#DIV/0!
Olfr1043|258570	-1	#DIV/0!	1	#DIV/0!
Gm5084|100503759	-1	#DIV/0!	1	#DIV/0!
Vmn1r76|171239	-1	#DIV/0!	1	#DIV/0!
1700031F05Rik|73300	-1	#DIV/0!	1	#DIV/0!
Mir3078|100526520	-1	#DIV/0!	1	#DIV/0!
Mir423|751519	-1	#DIV/0!	1	#DIV/0!
Chodl|246048	-1	#DIV/0!	1	#DIV/0!
Mir212|387208	-1	#DIV/0!	1	#DIV/0!
Sprr4|229562	-1	#DIV/0!	1	#DIV/0!

1700018A14Rik|69391	-1	#DIV/0!	1	#DIV/0!
Riiad1|66353	-1	#DIV/0!	1	#DIV/0!
Gm4710|100043885	-1	#DIV/0!	1	#DIV/0!
Olfr1338|258259	-1	#DIV/0!	1	#DIV/0!
Gm6588|625464	-1	#DIV/0!	1	#DIV/0!
A630010A05Rik|545280	-1	#DIV/0!	1	#DIV/0!
Tas2r120|387348	-1	#DIV/0!	1	#DIV/0!
Scarna13|100306943	-1	#DIV/0!	1	#DIV/0!
Mir684-1|735274	-1	#DIV/0!	1	#DIV/0!
Mirlet7f-2|387253	-1	#DIV/0!	1	#DIV/0!
Pdyn|18610	-1	#DIV/0!	1	#DIV/0!
Olfr197|258477	-1	#DIV/0!	1	#DIV/0!
Gm7714|665615	-1	#DIV/0!	1	#DIV/0!
Tex21|80384	-1	#DIV/0!	1	#DIV/0!
Tas2r138|387513	-1	#DIV/0!	1	#DIV/0!
Cbln1|12404	-1	#DIV/0!	1	#DIV/0!
Best2|212989	-1	#DIV/0!	1	#DIV/0!
Olfr902|258798	-1	#DIV/0!	1	#DIV/0!
Olfr1128|258346	-1	#DIV/0!	1	#DIV/0!
E430016F16Rik|414121	-1	#DIV/0!	1	#DIV/0!
Lhcgr|16867	-1	#DIV/0!	1	#DIV/0!
Olfr31|18330	-1	#DIV/0!	1	#DIV/0!
Hoxc6|15425	-1	#DIV/0!	1	#DIV/0!
Speer4d|360220	-1	#DIV/0!	1	#DIV/0!
Snord55|100216533	-1	#DIV/0!	1	#DIV/0!
Nppb|18158	-1	#DIV/0!	1	#DIV/0!
S100a5|20199	-1	#DIV/0!	1	#DIV/0!
Clrn1|229320	-1	#DIV/0!	1	#DIV/0!
Hoxd4|15436	-1	#DIV/0!	1	#DIV/0!
Vmn1r221|100312485	-1	#DIV/0!	1	#DIV/0!
Gm13083|279185	-1	#DIV/0!	1	#DIV/0!
Rhox2g|434766	-1	#DIV/0!	1	#DIV/0!
Olfr1199|258450	-1	#DIV/0!	1	#DIV/0!
Mir3066|100526470	-1	#DIV/0!	1	#DIV/0!
Olfr1234|258975	-1	#DIV/0!	1	#DIV/0!
Skor2|664805	-1	#DIV/0!	1	#DIV/0!
Krtap1-3|435273	-1	#DIV/0!	1	#DIV/0!
Il28b|338374	-1	#DIV/0!	1	#DIV/0!
1700034I23Rik|73297	-1	#DIV/0!	1	#DIV/0!
Lrguk|74354	-1	#DIV/0!	1	#DIV/0!
Olfr746|258295	-1	#DIV/0!	1	#DIV/0!
C130071C03Rik|320203	-1	#DIV/0!	1	#DIV/0!
Mis18bp1|217653	-1	#DIV/0!	1	#DIV/0!
Morc1|17450	-1	#DIV/0!	1	#DIV/0!
Alk|11682	-1	#DIV/0!	1	#DIV/0!
Klf17|75753	-1	#DIV/0!	1	#DIV/0!
Snord82|80828	-1	#DIV/0!	1	#DIV/0!
Mir124a-2|723950	-1	#DIV/0!	1	#DIV/0!
Clca3|23844	-1	#DIV/0!	1	#DIV/0!
Olfr51|18351	-1	#DIV/0!	1	#DIV/0!
Bnc2|242509	-1	#DIV/0!	1	#DIV/0!
Cib4|73259	-1	#DIV/0!	1	#DIV/0!
Snord37|100217454	-1	#DIV/0!	1	#DIV/0!

Olfr633|258351	-1	#DIV/0!	1	#DIV/0!
Tmco5|67356	-1	#DIV/0!	1	#DIV/0!
Col9a2|12840	-1	#DIV/0!	1	#DIV/0!
4933434I20Rik|67555	-1	#DIV/0!	1	#DIV/0!
Mmel1|27390	-1	#DIV/0!	1	#DIV/0!
Gzmf|14943	-1	#DIV/0!	1	#DIV/0!
Mbd3l1|73503	-1	#DIV/0!	1	#DIV/0!
Nhedc1|74446	-1	#DIV/0!	1	#DIV/0!
Mir669a-3|735272	-1	#DIV/0!	1	#DIV/0!
Serpinb3b|383548	-1	#DIV/0!	1	#DIV/0!
Ccdc105|70976	-1	#DIV/0!	1	#DIV/0!
Tas2r125|387352	-1	#DIV/0!	1	#DIV/0!
Ccdc33|382077	-1	#DIV/0!	1	#DIV/0!
Bmpr1b|12167	-1	#DIV/0!	1	#DIV/0!
Zscan30|328918	-1	#DIV/0!	1	#DIV/0!
Ntsr1|18216	-1	#DIV/0!	1	#DIV/0!
H2-M10.3|110696	-1	#DIV/0!	1	#DIV/0!
Ccdc60|269693	-1	#DIV/0!	1	#DIV/0!
Gm2897|100040671	-1	#DIV/0!	1	#DIV/0!
Rhox6|19202	-1	#DIV/0!	1	#DIV/0!
Hist1h2ai|319191	-1	#DIV/0!	1	#DIV/0!
Olfr59|18359	-1	#DIV/0!	1	#DIV/0!
Mir337|723843	-1	#DIV/0!	1	#DIV/0!
Cyp2ab1|224044	-1	#DIV/0!	1	#DIV/0!
A230065H16Rik|380787	-1	#DIV/0!	1	#DIV/0!
Lce1b|68720	-1	#DIV/0!	1	#DIV/0!
Tas2r108|57253	-1	#DIV/0!	1	#DIV/0!
Olfr1233|258974	-1	#DIV/0!	1	#DIV/0!
Tcam1|75870	-1	#DIV/0!	1	#DIV/0!
Lilra6|18726	-1	#DIV/0!	1	#DIV/0!
Myl1|17901	-1	#DIV/0!	1	#DIV/0!
Gpx5|14780	-1	#DIV/0!	1	#DIV/0!
Cts3|117066	-1	#DIV/0!	1	#DIV/0!
Vmn1r70|171262	-1	#DIV/0!	1	#DIV/0!
Sorcs1|58178	-1	#DIV/0!	1	#DIV/0!
Mir1937b-2|100499520	-1	#DIV/0!	1	#DIV/0!
Olfr976|258364	-1	#DIV/0!	1	#DIV/0!
Hist1h2bj|319183	-1	#DIV/0!	1	#DIV/0!
Mir1948|100316699	-1	#DIV/0!	1	#DIV/0!
Best1|24115	-1	#DIV/0!	1	#DIV/0!
Mir669e|100316806	-1	#DIV/0!	1	#DIV/0!
4931406H21Rik|77592	-1	#DIV/0!	1	#DIV/0!
Serpinb9c|20707	-1	#DIV/0!	1	#DIV/0!
Krt5|110308	-1	#DIV/0!	1	#DIV/0!
Ankmy1|241158	-1	#DIV/0!	1	#DIV/0!
Olfr1164|258634	-1	#DIV/0!	1	#DIV/0!
Serpini2|67931	-1	#DIV/0!	1	#DIV/0!
Vmn1r198|171254	-1	#DIV/0!	1	#DIV/0!
Aadacl3|230883	-1	#DIV/0!	1	#DIV/0!
Mepe|94111	-1	#DIV/0!	1	#DIV/0!
Olfr391-ps|258236	-1	#DIV/0!	1	#DIV/0!
Vmn2r49|625605	-1	#DIV/0!	1	#DIV/0!
Grp|225642	-1	#DIV/0!	1	#DIV/0!

Olfr314|257917	-1	#DIV/0!	1	#DIV/0!
Olfr1505|258151	-1	#DIV/0!	1	#DIV/0!
Vmn2r99|665376	-1	#DIV/0!	1	#DIV/0!
Mir1298|100526462	-1	#DIV/0!	1	#DIV/0!
Olfr1335|435804	-1	#DIV/0!	1	#DIV/0!
Prap1|22264	-1	#DIV/0!	1	#DIV/0!
Ncapg|54392	-1	#DIV/0!	1	#DIV/0!
Gpr87|84111	-1	#DIV/0!	1	#DIV/0!
Ffar1|233081	-1	#DIV/0!	1	#DIV/0!
Vmn2r60|637898	-1	#DIV/0!	1	#DIV/0!
Mir30c-2|723964	-1	#DIV/0!	1	#DIV/0!
Olfr380|259027	-1	#DIV/0!	1	#DIV/0!
C87499|381590	-1	#DIV/0!	1	#DIV/0!
Olfr551|258750	-1	#DIV/0!	1	#DIV/0!
Rln3|212108	-1	#DIV/0!	1	#DIV/0!
Gtf2a1l|71828	-1	#DIV/0!	1	#DIV/0!
Ccin|442829	-1	#DIV/0!	1	#DIV/0!
Olfr10|18307	-1	#DIV/0!	1	#DIV/0!
1700016K19Rik|74230	-1	#DIV/0!	1	#DIV/0!
Olfr670|384703	-1	#DIV/0!	1	#DIV/0!
Trpc7|26946	-1	#DIV/0!	1	#DIV/0!
Olfr125|258287	-1	#DIV/0!	1	#DIV/0!
Gm2694|100040294	-1	#DIV/0!	1	#DIV/0!
Fstl5|213262	-1	#DIV/0!	1	#DIV/0!
Gm41|245502	-1	#DIV/0!	1	#DIV/0!
6030468B19Rik|77727	-1	#DIV/0!	1	#DIV/0!
D130009I18Rik|320249	-1	#DIV/0!	1	#DIV/0!
Svs2|53878	-1	#DIV/0!	1	#DIV/0!
Mir488|735253	-1	#DIV/0!	1	#DIV/0!
4921509C19Rik|381393	-1	#DIV/0!	1	#DIV/0!
Vmn1r168|100043101	-1	#DIV/0!	1	#DIV/0!
Casp14|12365	-1	#DIV/0!	1	#DIV/0!
4930451C15Rik|74685	-1	#DIV/0!	1	#DIV/0!
9430007A20Rik|381572	-1	#DIV/0!	1	#DIV/0!
Cntn1|12805	-1	#DIV/0!	1	#DIV/0!
4931429I11Rik|70989	-1	#DIV/0!	1	#DIV/0!
Oas1d|100535	-1	#DIV/0!	1	#DIV/0!
Cd226|225825	-1	#DIV/0!	1	#DIV/0!
Oprk1|18387	-1	#DIV/0!	1	#DIV/0!
Olfr640|258819	-1	#DIV/0!	1	#DIV/0!
Spaca4|69363	-1	#DIV/0!	1	#DIV/0!
Cldn24|100039801	-1	#DIV/0!	1	#DIV/0!
Lrrc10b|278795	-1	#DIV/0!	1	#DIV/0!
Neil3|234258	-1	#DIV/0!	1	#DIV/0!
Zdhhc23|332175	-1	#DIV/0!	1	#DIV/0!
Isl1|16392	-1	#DIV/0!	1	#DIV/0!
Olfr318|258494	-1	#DIV/0!	1	#DIV/0!
Fam23a|625286	-1	#DIV/0!	1	#DIV/0!
Gm8439|667063	-1	#DIV/0!	1	#DIV/0!
Tcl1b3|27378	-1	#DIV/0!	1	#DIV/0!
Dmc1|13404	-1	#DIV/0!	1	#DIV/0!
Gm904|380845	-1	#DIV/0!	1	#DIV/0!
Olfr761|258094	-1	#DIV/0!	1	#DIV/0!

Olfr39|258822	-1	#DIV/0!	1	#DIV/0!
Mir3106|100526464	-1	#DIV/0!	1	#DIV/0!
4930504O13Rik|403200	-1	#DIV/0!	1	#DIV/0!
Rhox2d|434760	-1	#DIV/0!	1	#DIV/0!
H2-M2|14990	-1	#DIV/0!	1	#DIV/0!
Otud7a|170711	-1	#DIV/0!	1	#DIV/0!
Speer1-ps1|70896	-1	#DIV/0!	1	#DIV/0!
Gdf11|14561	-1	#DIV/0!	1	#DIV/0!
Fshb|14308	-1	#DIV/0!	1	#DIV/0!
Kcnk16|74571	-1	#DIV/0!	1	#DIV/0!
Olfr619|259080	-1	#DIV/0!	1	#DIV/0!
Olfr147|258869	-1	#DIV/0!	1	#DIV/0!
Gucy1b2|239134	-1	#DIV/0!	1	#DIV/0!
Elavl3|15571	-1	#DIV/0!	1	#DIV/0!
Ott|18422	-1	#DIV/0!	1	#DIV/0!
Mir505|751545	-1	#DIV/0!	1	#DIV/0!
Il9|16198	-1	#DIV/0!	1	#DIV/0!
Olfr705|259034	-1	#DIV/0!	1	#DIV/0!
Olfr457|258989	-1	#DIV/0!	1	#DIV/0!
Olfr1189|258768	-1	#DIV/0!	1	#DIV/0!
Hbb-bh1|15132	-1	#DIV/0!	1	#DIV/0!
Vmn1r73|171237	-1	#DIV/0!	1	#DIV/0!
Tacstd2|56753	-1	#DIV/0!	1	#DIV/0!
Zic1|22771	-1	#DIV/0!	1	#DIV/0!
Olfr1391|258460	-1	#DIV/0!	1	#DIV/0!
Nmu|56183	-1	#DIV/0!	1	#DIV/0!
Olig3|94222	-1	#DIV/0!	1	#DIV/0!
Defb9|246079	-1	#DIV/0!	1	#DIV/0!
Olfr464|258407	-1	#DIV/0!	1	#DIV/0!
Il17b|56069	-1	#DIV/0!	1	#DIV/0!
Nr2e3|23958	-1	#DIV/0!	1	#DIV/0!
Olfr714|259035	-1	#DIV/0!	1	#DIV/0!
Prl2c1|666317	-1	#DIV/0!	1	#DIV/0!
Fbxw20|434440	-1	#DIV/0!	1	#DIV/0!
4921517D21Rik|67722	-1	#DIV/0!	1	#DIV/0!
Gfra4|14588	-1	#DIV/0!	1	#DIV/0!
Olfr773|257664	-1	#DIV/0!	1	#DIV/0!
Snord61|353374	-1	#DIV/0!	1	#DIV/0!
1700024P04Rik|69382	-1	#DIV/0!	1	#DIV/0!
Grid2ip|170935	-1	#DIV/0!	1	#DIV/0!
Ucn3|83428	-1	#DIV/0!	1	#DIV/0!
Znrf4|20834	-1	#DIV/0!	1	#DIV/0!
Noxa1|241275	-1	#DIV/0!	1	#DIV/0!
Mir1953|100316704	-1	#DIV/0!	1	#DIV/0!
Ccdc54|69339	-1	#DIV/0!	1	#DIV/0!
Slfn14-ps|237890	-1	#DIV/0!	1	#DIV/0!
Gm364|245423	-1	#DIV/0!	1	#DIV/0!
Gh|14599	-1	#DIV/0!	1	#DIV/0!
Mmp10|17384	-1	#DIV/0!	1	#DIV/0!
Olfr733|258657	-1	#DIV/0!	1	#DIV/0!
Rnase11|497113	-1	#DIV/0!	1	#DIV/0!
Olfr330|258879	-1	#DIV/0!	1	#DIV/0!
Mir463|723887	-1	#DIV/0!	1	#DIV/0!

Spn-ps|20738	-1	#DIV/0!	1	#DIV/0!
Olfr495|258361	-1	#DIV/0!	1	#DIV/0!
Rhpn1|14787	-1	#DIV/0!	1	#DIV/0!
Olfr995|258426	-1	#DIV/0!	1	#DIV/0!
Olfr421|258715	-1	#DIV/0!	1	#DIV/0!
Olfr157|100040268	-1	#DIV/0!	1	#DIV/0!
Defb4|56519	-1	#DIV/0!	1	#DIV/0!
Mir369|723933	-1	#DIV/0!	1	#DIV/0!
Glra3|110304	-1	#DIV/0!	1	#DIV/0!
2010317E24Rik|72080	-1	#DIV/0!	1	#DIV/0!
Adam28|13522	-1	#DIV/0!	1	#DIV/0!
Olfr663|257914	-1	#DIV/0!	1	#DIV/0!
Gm6904|628693	-1	#DIV/0!	1	#DIV/0!
Olfr183|258478	-1	#DIV/0!	1	#DIV/0!
Gm2049|100039109	-1	#DIV/0!	1	#DIV/0!
Gm4541|100043595	-1	#DIV/0!	1	#DIV/0!
Fam131b|76156	-1	#DIV/0!	1	#DIV/0!
Slc35d3|76157	-1	#DIV/0!	1	#DIV/0!
Hist1h2br|665622	-1	#DIV/0!	1	#DIV/0!
Vmn2r25|545874	-1	#DIV/0!	1	#DIV/0!
Cacng5|140723	-1	#DIV/0!	1	#DIV/0!
Olfr57|18357	-1	#DIV/0!	1	#DIV/0!
Ccdc81|70884	-1	#DIV/0!	1	#DIV/0!
Sprr1b|20754	-1	#DIV/0!	1	#DIV/0!
Col25a1|77018	-1	#DIV/0!	1	#DIV/0!
Vmn1r132|100043604	-1	#DIV/0!	1	#DIV/0!
Tarm1|245126	-1	#DIV/0!	1	#DIV/0!
Ceacam-ps1|100038912	-1	#DIV/0!	1	#DIV/0!
Morn3|74890	-1	#DIV/0!	1	#DIV/0!
Mir1929|100316688	-1	#DIV/0!	1	#DIV/0!
Hapln2|73940	-1	#DIV/0!	1	#DIV/0!
Vmn1r74|171240	-1	#DIV/0!	1	#DIV/0!
Pom121l12|432536	-1	#DIV/0!	1	#DIV/0!
C130026L21Rik|330164	-1	#DIV/0!	1	#DIV/0!
41166|74222	-1	#DIV/0!	1	#DIV/0!
Mir26b|387219	-1	#DIV/0!	1	#DIV/0!
Usp51|635253	-1	#DIV/0!	1	#DIV/0!
Hspb9|75482	-1	#DIV/0!	1	#DIV/0!
Olfr1410|258484	-1	#DIV/0!	1	#DIV/0!
Dusp13|27389	-1	#DIV/0!	1	#DIV/0!
Vmn2r111|210876	-1	#DIV/0!	1	#DIV/0!
Lgals12|56072	-1	#DIV/0!	1	#DIV/0!
Vmn1r-ps103|171245	-1	#DIV/0!	1	#DIV/0!
2900041M22Rik|78403	-1	#DIV/0!	1	#DIV/0!
Vmn2r121|100038941	-1	#DIV/0!	1	#DIV/0!
Wbp2nl|74716	-1	#DIV/0!	1	#DIV/0!
Mrgpra9|668725	-1	#DIV/0!	1	#DIV/0!
2810429I04Rik|76937	-1	#DIV/0!	1	#DIV/0!
Pyy|217212	-1	#DIV/0!	1	#DIV/0!
Mir449c|735309	-1	#DIV/0!	1	#DIV/0!
Prpmp5|381832	-1	#DIV/0!	1	#DIV/0!
Sfta2|433102	-1	#DIV/0!	1	#DIV/0!
Olfr591|258139	-1	#DIV/0!	1	#DIV/0!

Gm4177|100043025	-1	#DIV/0!	1	#DIV/0!
Mc2r|17200	-1	#DIV/0!	1	#DIV/0!
Gm14484|547160	-1	#DIV/0!	1	#DIV/0!
Mir744|791070	-1	#DIV/0!	1	#DIV/0!
Olfr822|258666	-1	#DIV/0!	1	#DIV/0!
Pcdh19|279653	-1	#DIV/0!	1	#DIV/0!
Cldn19|242653	-1	#DIV/0!	1	#DIV/0!
Fam71f1|330277	-1	#DIV/0!	1	#DIV/0!
Olfr498|258304	-1	#DIV/0!	1	#DIV/0!
Zic5|65100	-1	#DIV/0!	1	#DIV/0!
4930430A15Rik|67575	-1	#DIV/0!	1	#DIV/0!
Olfr672|258755	-1	#DIV/0!	1	#DIV/0!
Mir465c-1|100124441	-1	#DIV/0!	1	#DIV/0!
Bpifa6|545477	-1	#DIV/0!	1	#DIV/0!
Il13|16163	-1	#DIV/0!	1	#DIV/0!
Gm16387|100038914	-1	#DIV/0!	1	#DIV/0!
Tmem72|319776	-1	#DIV/0!	1	#DIV/0!
St8sia6|241230	-1	#DIV/0!	1	#DIV/0!
Mir763|791076	-1	#DIV/0!	1	#DIV/0!
Gm4922|237300	-1	#DIV/0!	1	#DIV/0!
Olfr218|258880	-1	#DIV/0!	1	#DIV/0!
Ern2|26918	-1	#DIV/0!	1	#DIV/0!
Luzp2|233271	-1	#DIV/0!	1	#DIV/0!
Mir132|387150	-1	#DIV/0!	1	#DIV/0!
Myo3b|329421	-1	#DIV/0!	1	#DIV/0!
Fgf23|64654	-1	#DIV/0!	1	#DIV/0!
Fbxo41|330369	-1	#DIV/0!	1	#DIV/0!
Mir219-1|723823	-1	#DIV/0!	1	#DIV/0!
Mir551b|791072	-1	#DIV/0!	1	#DIV/0!
Vmn2r26|56552	-1	#DIV/0!	1	#DIV/0!
Mir669g|100316802	-1	#DIV/0!	1	#DIV/0!
Chrna9|231252	-1	#DIV/0!	1	#DIV/0!
Terc|21748	-1	#DIV/0!	1	#DIV/0!
Olfr1418|258227	-1	#DIV/0!	1	#DIV/0!
Chrm4|12672	-1	#DIV/0!	1	#DIV/0!
Vmn1r159|670857	-1	#DIV/0!	1	#DIV/0!
Mageb16|71967	-1	#DIV/0!	1	#DIV/0!
Hist1h2ba|319177	-1	#DIV/0!	1	#DIV/0!
Ppp2r2b|72930	-1	#DIV/0!	1	#DIV/0!
Kcnmb3|100502876	-1	#DIV/0!	1	#DIV/0!
Dydc1|69496	-1	#DIV/0!	1	#DIV/0!
Mtnr1b|244701	-1	#DIV/0!	1	#DIV/0!
Mir1187|100316803	-1	#DIV/0!	1	#DIV/0!
Rex2|100043034	-1	#DIV/0!	1	#DIV/0!
Adam24|13526	-1	#DIV/0!	1	#DIV/0!
Gm5615|434396	-1	#DIV/0!	1	#DIV/0!
Hoxc8|15426	-1	#DIV/0!	1	#DIV/0!
Gpr21|338346	-1	#DIV/0!	1	#DIV/0!
Sorcs3|66673	-1	#DIV/0!	1	#DIV/0!
Fam71a|619288	-1	#DIV/0!	1	#DIV/0!
Olfr5|18349	-1	#DIV/0!	1	#DIV/0!
Olfr1449|258300	-1	#DIV/0!	1	#DIV/0!
Rimkla|194237	-1	#DIV/0!	1	#DIV/0!

Vmn1r194|626299	-1	#DIV/0!	1	#DIV/0!
Olfr1136|258653	-1	#DIV/0!	1	#DIV/0!
Snord73a|19870	-1	#DIV/0!	1	#DIV/0!
Cldn20|621628	-1	#DIV/0!	1	#DIV/0!
Gm13298|545611	-1	#DIV/0!	1	#DIV/0!
Hcrt|15171	-1	#DIV/0!	1	#DIV/0!
Adora2b|11541	-1	#DIV/0!	1	#DIV/0!
Olfr1472|258685	-1	#DIV/0!	1	#DIV/0!
Zscan4e|665848	-1	#DIV/0!	1	#DIV/0!
Olfr267|258922	-1	#DIV/0!	1	#DIV/0!
Olfr678|258753	-1	#DIV/0!	1	#DIV/0!
Hdhd1a|67365	-1	#DIV/0!	1	#DIV/0!
Olfr65|18365	-1	#DIV/0!	1	#DIV/0!
Klk9|101533	-1	#DIV/0!	1	#DIV/0!
Wdr17|244484	-1	#DIV/0!	1	#DIV/0!
9530036O11Rik|654796	-1	#DIV/0!	1	#DIV/0!
Gm773|331416	-1	#DIV/0!	1	#DIV/0!
Olfr150|258602	-1	#DIV/0!	1	#DIV/0!
2210415F13Rik|70163	-1	#DIV/0!	1	#DIV/0!
Mageb16-ps1|100039436	-1	#DIV/0!	1	#DIV/0!
4932411G14Rik|238663	-1	#DIV/0!	1	#DIV/0!
1700017N19Rik|66605	-1	#DIV/0!	1	#DIV/0!
Vmn2r74|546980	-1	#DIV/0!	1	#DIV/0!
Olfr360|258615	-1	#DIV/0!	1	#DIV/0!
Mir128-1|387147	-1	#DIV/0!	1	#DIV/0!
Rergl|632971	-1	#DIV/0!	1	#DIV/0!
Krtap4-7|76444	-1	#DIV/0!	1	#DIV/0!
Mir324|723896	-1	#DIV/0!	1	#DIV/0!
Gm10636|100038607	-1	#DIV/0!	1	#DIV/0!
Grip1|74053	-1	#DIV/0!	1	#DIV/0!
Mir367|723911	-1	#DIV/0!	1	#DIV/0!
Gm5416|408196	-1	#DIV/0!	1	#DIV/0!
Gm766|330440	-1	#DIV/0!	1	#DIV/0!
Jakmip2|76217	-1	#DIV/0!	1	#DIV/0!
Gpr50|14765	-1	#DIV/0!	1	#DIV/0!
Zcchc13|75064	-1	#DIV/0!	1	#DIV/0!
Cct8l1|242891	-1	#DIV/0!	1	#DIV/0!
Gsdmc|83492	-1	#DIV/0!	1	#DIV/0!
Nup210l|77595	-1	#DIV/0!	1	#DIV/0!
4931406B18Rik|74054	-1	#DIV/0!	1	#DIV/0!
Mrgpra6|381886	-1	#DIV/0!	1	#DIV/0!
Pthlh|19227	-1	#DIV/0!	1	#DIV/0!
Hist1h2bh|319182	-1	#DIV/0!	1	#DIV/0!
Olfr121|258622	-1	#DIV/0!	1	#DIV/0!
1700014N06Rik|69389	-1	#DIV/0!	1	#DIV/0!
Hpcal4|170638	-1	#DIV/0!	1	#DIV/0!
Cst12|69362	-1	#DIV/0!	1	#DIV/0!
Phkg1|18682	-1	#DIV/0!	1	#DIV/0!
Insl5|23919	-1	#DIV/0!	1	#DIV/0!
Olfr1339|258851	-1	#DIV/0!	1	#DIV/0!
2010109I03Rik|67038	-1	#DIV/0!	1	#DIV/0!
Agbl2|271813	-1	#DIV/0!	1	#DIV/0!
Olfr429|258717	-1	#DIV/0!	1	#DIV/0!

Olfr1436|258682	-1	#DIV/0!	1	#DIV/0!
6230409E13Rik|76132	-1	#DIV/0!	1	#DIV/0!
Gm6289|622139	-1	#DIV/0!	1	#DIV/0!
Rxfp1|381489	-1	#DIV/0!	1	#DIV/0!
Dync1i1|13426	-1	#DIV/0!	1	#DIV/0!
Nrk|27206	-1	#DIV/0!	1	#DIV/0!
Efhc2|74405	-1	#DIV/0!	1	#DIV/0!
4932414N04Rik|75721	-1	#DIV/0!	1	#DIV/0!
Disc1|244667	-1	#DIV/0!	1	#DIV/0!
1700001K23Rik|69319	-1	#DIV/0!	1	#DIV/0!
Ipw|16353	-1	#DIV/0!	1	#DIV/0!
Acsm4|233801	-1	#DIV/0!	1	#DIV/0!
Mir879|100124492	-1	#DIV/0!	1	#DIV/0!
Gm11149|100036537	-1	#DIV/0!	1	#DIV/0!
Cntn6|53870	-1	#DIV/0!	1	#DIV/0!
Mir3086|100526480	-1	#DIV/0!	1	#DIV/0!
Olfr835|257872	-1	#DIV/0!	1	#DIV/0!
Gm12171|675689	-1	#DIV/0!	1	#DIV/0!
Defb36|266620	-1	#DIV/0!	1	#DIV/0!
Plcxd3|239318	-1	#DIV/0!	1	#DIV/0!
A530053G22Rik|208079	-1	#DIV/0!	1	#DIV/0!
Lrrc34|71827	-1	#DIV/0!	1	#DIV/0!
Gm7173|636104	-1	#DIV/0!	1	#DIV/0!
Pgr15l|245526	-1	#DIV/0!	1	#DIV/0!
Pcdhb21|93892	-1	#DIV/0!	1	#DIV/0!
Siglec15|620235	-1	#DIV/0!	1	#DIV/0!
Olfr810|258543	-1	#DIV/0!	1	#DIV/0!
Olfr414|258756	-1	#DIV/0!	1	#DIV/0!
9230104L09Rik|77705	-1	#DIV/0!	1	#DIV/0!
Vmn1r178|232959	-1	#DIV/0!	1	#DIV/0!
Slco1a6|28254	-1	#DIV/0!	1	#DIV/0!
BC049635|277773	-1	#DIV/0!	1	#DIV/0!
Crxos1|546024	-1	#DIV/0!	1	#DIV/0!
Olfr470|258417	-1	#DIV/0!	1	#DIV/0!
C030019I05Rik|320116	-1	#DIV/0!	1	#DIV/0!
Csta|209294	-1	#DIV/0!	1	#DIV/0!
Fam5b|240843	-1	#DIV/0!	1	#DIV/0!
Amtn|71421	-1	#DIV/0!	1	#DIV/0!
Lce1m|66203	-1	#DIV/0!	1	#DIV/0!
H2-M10.4|224753	-1	#DIV/0!	1	#DIV/0!
Dlx1as|111970	-1	#DIV/0!	1	#DIV/0!
Ubqlnl|244179	-1	#DIV/0!	1	#DIV/0!
Prss22|70835	-1	#DIV/0!	1	#DIV/0!
Vmn1r39|546903	-1	#DIV/0!	1	#DIV/0!
Dcaf12l2|245403	-1	#DIV/0!	1	#DIV/0!
Prop1|19127	-1	#DIV/0!	1	#DIV/0!
Olfr1118|404328	-1	#DIV/0!	1	#DIV/0!
Gm15292|100041787	-1	#DIV/0!	1	#DIV/0!
Chrnd|11447	-1	#DIV/0!	1	#DIV/0!
4930443G12Rik|74686	-1	#DIV/0!	1	#DIV/0!
Olfr599|258726	-1	#DIV/0!	1	#DIV/0!
Rnu11|353373	-1	#DIV/0!	1	#DIV/0!
Cilp2|68709	-1	#DIV/0!	1	#DIV/0!

Rhcg|56315	-1	#DIV/0!	1	#DIV/0!
Gm1965|434065	-1	#DIV/0!	1	#DIV/0!
Vmn1r40|113855	-1	#DIV/0!	1	#DIV/0!
2010106E10Rik|67715	-1	#DIV/0!	1	#DIV/0!
1700109H08Rik|77036	-1	#DIV/0!	1	#DIV/0!
Gm9|194854	-1	#DIV/0!	1	#DIV/0!
Olfr566|258168	-1	#DIV/0!	1	#DIV/0!
Fabp9|21884	-1	#DIV/0!	1	#DIV/0!
Nxf3|245610	-1	#DIV/0!	1	#DIV/0!
Otx2os1|606497	-1	#DIV/0!	1	#DIV/0!
Rhox3c|100135654	-1	#DIV/0!	1	#DIV/0!
Gm4216|100043083	-1	#DIV/0!	1	#DIV/0!
Oog3|100012	-1	#DIV/0!	1	#DIV/0!
Capns2|69543	-1	#DIV/0!	1	#DIV/0!
Vmn1r235|171233	-1	#DIV/0!	1	#DIV/0!
Pdzd3|170761	-1	#DIV/0!	1	#DIV/0!
Skint10|230613	-1	#DIV/0!	1	#DIV/0!
Cacng2|12300	-1	#DIV/0!	1	#DIV/0!
Olfr677|258355	-1	#DIV/0!	1	#DIV/0!
Stmn1-rs1|111186	-1	#DIV/0!	1	#DIV/0!
Gm5797|545013	-1	#DIV/0!	1	#DIV/0!
Defb22|442835	-1	#DIV/0!	1	#DIV/0!
Olfr1350|258384	-1	#DIV/0!	1	#DIV/0!
1700096J18Rik|67927	-1	#DIV/0!	1	#DIV/0!
Ocm|18261	-1	#DIV/0!	1	#DIV/0!
Tspan10|208634	-1	#DIV/0!	1	#DIV/0!
Slc18a3|20508	-1	#DIV/0!	1	#DIV/0!
Gipc2|54120	-1	#DIV/0!	1	#DIV/0!
Mir497|751537	-1	#DIV/0!	1	#DIV/0!
Rhox2h|622301	-1	#DIV/0!	1	#DIV/0!
Bpifb4|381399	-1	#DIV/0!	1	#DIV/0!
Olfr1212|258241	-1	#DIV/0!	1	#DIV/0!
Mstn|17700	-1	#DIV/0!	1	#DIV/0!
Skint6|230622	-1	#DIV/0!	1	#DIV/0!
Tlx1|21908	-1	#DIV/0!	1	#DIV/0!
Lcn14|383678	-1	#DIV/0!	1	#DIV/0!
Olfr639|259088	-1	#DIV/0!	1	#DIV/0!
Snord42b|100529077	-1	#DIV/0!	1	#DIV/0!
Figla|26910	-1	#DIV/0!	1	#DIV/0!
Vmn1r18|171199	-1	#DIV/0!	1	#DIV/0!
Mir694|751558	-1	#DIV/0!	1	#DIV/0!
Gpr113|381628	-1	#DIV/0!	1	#DIV/0!
Krtap6-1|16700	-1	#DIV/0!	1	#DIV/0!
Dleu7|239133	-1	#DIV/0!	1	#DIV/0!
Olfr1160|258643	-1	#DIV/0!	1	#DIV/0!
Spinlw1|75526	-1	#DIV/0!	1	#DIV/0!
Mir681|751538	-1	#DIV/0!	1	#DIV/0!
Sdr16c5|242285	-1	#DIV/0!	1	#DIV/0!
4930467E23Rik|626415	-1	#DIV/0!	1	#DIV/0!
Gm11487|433719	-1	#DIV/0!	1	#DIV/0!
Olfr1313|258257	-1	#DIV/0!	1	#DIV/0!
Olfr1022|258582	-1	#DIV/0!	1	#DIV/0!
Trim38|214158	-1	#DIV/0!	1	#DIV/0!

1700017D01Rik|69369	-1	#DIV/0!	1	#DIV/0!
Mrgprh|80978	-1	#DIV/0!	1	#DIV/0!
Olfr1002|258566	-1	#DIV/0!	1	#DIV/0!
Vipr2|22355	-1	#DIV/0!	1	#DIV/0!
Gm4763|210155	-1	#DIV/0!	1	#DIV/0!
Enpp7|238011	-1	#DIV/0!	1	#DIV/0!
Olfr722|258487	-1	#DIV/0!	1	#DIV/0!
Zscan4b|665780	-1	#DIV/0!	1	#DIV/0!
Bsnd|140475	-1	#DIV/0!	1	#DIV/0!
Foxn4|116810	-1	#DIV/0!	1	#DIV/0!
4933406K04Rik|71033	-1	#DIV/0!	1	#DIV/0!
Vmn1r214|171248	-1	#DIV/0!	1	#DIV/0!
Pla2g2d|18782	-1	#DIV/0!	1	#DIV/0!
Cdcp2|242603	-1	#DIV/0!	1	#DIV/0!
Gm581|210541	-1	#DIV/0!	1	#DIV/0!
BC030500|234290	-1	#DIV/0!	1	#DIV/0!
Il17c|234836	-1	#DIV/0!	1	#DIV/0!
Cyp2c66|69888	-1	#DIV/0!	1	#DIV/0!
Vmn2r67|620672	-1	#DIV/0!	1	#DIV/0!
Smr2|20600	-1	#DIV/0!	1	#DIV/0!
Vmn1r184|100312477	-1	#DIV/0!	1	#DIV/0!
Il1rapl1|331461	-1	#DIV/0!	1	#DIV/0!
Mir758|791071	-1	#DIV/0!	1	#DIV/0!
Mir345|723946	-1	#DIV/0!	1	#DIV/0!
Mycbpap|104601	-1	#DIV/0!	1	#DIV/0!
Ifi202b|26388	-1	#DIV/0!	1	#DIV/0!
Dppa4|73693	-1	#DIV/0!	1	#DIV/0!
Tmem95|432576	-1	#DIV/0!	1	#DIV/0!
Tas2r122|630845	-1	#DIV/0!	1	#DIV/0!
Olfr372|404315	-1	#DIV/0!	1	#DIV/0!
Txndc6|623534	-1	#DIV/0!	1	#DIV/0!
Vmn2r108|627805	-1	#DIV/0!	1	#DIV/0!
Cox7b2|78174	-1	#DIV/0!	1	#DIV/0!
Gm6760|627470	-1	#DIV/0!	1	#DIV/0!
Olfr1124|259030	-1	#DIV/0!	1	#DIV/0!
Gm5916|546123	-1	#DIV/0!	1	#DIV/0!
Hbb-bh2|436003	-1	#DIV/0!	1	#DIV/0!
Gm6772|627607	-1	#DIV/0!	1	#DIV/0!
Nr0b1|11614	-1	#DIV/0!	1	#DIV/0!
Mir653|100124468	-1	#DIV/0!	1	#DIV/0!
Olfr371|258858	-1	#DIV/0!	1	#DIV/0!
Mir3075|100526533	-1	#DIV/0!	1	#DIV/0!
Chia|81600	-1	#DIV/0!	1	#DIV/0!
Fezf1|73191	-1	#DIV/0!	1	#DIV/0!
9230115E21Rik|408028	-1	#DIV/0!	1	#DIV/0!
Gm7616|665389	-1	#DIV/0!	1	#DIV/0!
Atp1a4|27222	-1	#DIV/0!	1	#DIV/0!
Gm14483|100042922	-1	#DIV/0!	1	#DIV/0!
Gm6642|625995	-1	#DIV/0!	1	#DIV/0!
Cyp2j12|242546	-1	#DIV/0!	1	#DIV/0!
Skor1|207667	-1	#DIV/0!	1	#DIV/0!
Myh15|667772	-1	#DIV/0!	1	#DIV/0!
Snord8|100217445	-1	#DIV/0!	1	#DIV/0!

Capn8|170725	-1	#DIV/0!	1	#DIV/0!
Hist1h2ao|665433	-1	#DIV/0!	1	#DIV/0!
1700129C05Rik|67932	-1	#DIV/0!	1	#DIV/0!
Fam3b|52793	-1	#DIV/0!	1	#DIV/0!
Tppp2|219038	-1	#DIV/0!	1	#DIV/0!
Dmbx1|140477	-1	#DIV/0!	1	#DIV/0!
Mir678|751554	-1	#DIV/0!	1	#DIV/0!
Gm5885|545884	-1	#DIV/0!	1	#DIV/0!
Slc9a2|226999	-1	#DIV/0!	1	#DIV/0!
Dlx5|13395	-1	#DIV/0!	1	#DIV/0!
C1ql3|227580	-1	#DIV/0!	1	#DIV/0!
Snord111|100217465	-1	#DIV/0!	1	#DIV/0!
Gm14354|74851	-1	#DIV/0!	1	#DIV/0!
Gm6086|619597	-1	#DIV/0!	1	#DIV/0!
Rhox3a|382209	-1	#DIV/0!	1	#DIV/0!
Mir1899|100316772	-1	#DIV/0!	1	#DIV/0!
1700054O13Rik|67334	-1	#DIV/0!	1	#DIV/0!
Agbl4|78933	-1	#DIV/0!	1	#DIV/0!
Prima1|170952	-1	#DIV/0!	1	#DIV/0!
Mir92-2|723942	-1	#DIV/0!	1	#DIV/0!
Olfr1428|258673	-1	#DIV/0!	1	#DIV/0!
Klk7|23993	-1	#DIV/0!	1	#DIV/0!
Gm13280|230398	-1	#DIV/0!	1	#DIV/0!
Mir20b|723923	-1	#DIV/0!	1	#DIV/0!
Lyzl6|69444	-1	#DIV/0!	1	#DIV/0!
4933409G03Rik|227998	-1	#DIV/0!	1	#DIV/0!
Vmn1r79|100042437	-1	#DIV/0!	1	#DIV/0!
Ssxb1|67985	-1	#DIV/0!	1	#DIV/0!
Odf3l2|382384	-1	#DIV/0!	1	#DIV/0!
Olfr133|258828	-1	#DIV/0!	1	#DIV/0!
Olfr1024|257900	-1	#DIV/0!	1	#DIV/0!
1700001P01Rik|72215	-1	#DIV/0!	1	#DIV/0!
Paqr5|74090	-1	#DIV/0!	1	#DIV/0!
Cyp2b23|243881	-1	#DIV/0!	1	#DIV/0!
Chrna3|110834	-1	#DIV/0!	1	#DIV/0!
Gcg|14526	-1	#DIV/0!	1	#DIV/0!
Gm5934|546272	-1	#DIV/0!	1	#DIV/0!
Olfr1303|258397	-1	#DIV/0!	1	#DIV/0!
Gpr15|71223	-1	#DIV/0!	1	#DIV/0!
Tal2|21350	-1	#DIV/0!	1	#DIV/0!
Tctex1d1|67344	-1	#DIV/0!	1	#DIV/0!
Olfr191|258035	-1	#DIV/0!	1	#DIV/0!
Olfr598|257975	-1	#DIV/0!	1	#DIV/0!
Yipf7|75581	-1	#DIV/0!	1	#DIV/0!
Slc35f3|210027	-1	#DIV/0!	1	#DIV/0!
Msx3|17703	-1	#DIV/0!	1	#DIV/0!
Olfr378|259026	-1	#DIV/0!	1	#DIV/0!
Myb|17863	-1	#DIV/0!	1	#DIV/0!
Npc1l1|237636	-1	#DIV/0!	1	#DIV/0!
Olfr1180|258920	-1	#DIV/0!	1	#DIV/0!
Klk1b3|18050	-1	#DIV/0!	1	#DIV/0!
Rhox3f|621852	-1	#DIV/0!	1	#DIV/0!
Colq|382864	-1	#DIV/0!	1	#DIV/0!

Nphs1as|445267	-1	#DIV/0!	1	#DIV/0!
Olfr802|258934	-1	#DIV/0!	1	#DIV/0!
Mir29b-2|723963	-1	#DIV/0!	1	#DIV/0!
Olfr494|258732	-1	#DIV/0!	1	#DIV/0!
Fam123c|211383	-1	#DIV/0!	1	#DIV/0!
Zpld1|239852	-1	#DIV/0!	1	#DIV/0!
Spag16|66722	-1	#DIV/0!	1	#DIV/0!
Tex101|56746	-1	#DIV/0!	1	#DIV/0!
Frem2|242022	-1	#DIV/0!	1	#DIV/0!
Olfr994|258425	-1	#DIV/0!	1	#DIV/0!
Olfr1098|258842	-1	#DIV/0!	1	#DIV/0!
Olfr1026|258577	-1	#DIV/0!	1	#DIV/0!
Ceacam9|26368	-1	#DIV/0!	1	#DIV/0!
Gm5087|328354	-1	#DIV/0!	1	#DIV/0!
Slc47a2|380701	-1	#DIV/0!	1	#DIV/0!
Gm3286|100041354	-1	#DIV/0!	1	#DIV/0!
Amy2a4|100043684	-1	#DIV/0!	1	#DIV/0!
Rgs13|246709	-1	#DIV/0!	1	#DIV/0!
Ctsq|104002	-1	#DIV/0!	1	#DIV/0!
Neurog1|18014	-1	#DIV/0!	1	#DIV/0!
Olfr291|258410	-1	#DIV/0!	1	#DIV/0!
D630042F21Rik|330428	-1	#DIV/0!	1	#DIV/0!
Mir3097|100526557	-1	#DIV/0!	1	#DIV/0!
Vmn2r54|666085	-1	#DIV/0!	1	#DIV/0!
Mir300|723833	-1	#DIV/0!	1	#DIV/0!
Foxd1|15229	-1	#DIV/0!	1	#DIV/0!
Defa20|68009	-1	#DIV/0!	1	#DIV/0!
Olfr720|258387	-1	#DIV/0!	1	#DIV/0!
Vax1|22326	-1	#DIV/0!	1	#DIV/0!
Pnmt|18948	-1	#DIV/0!	1	#DIV/0!
Trim67|330863	-1	#DIV/0!	1	#DIV/0!
Tub|22141	-1	#DIV/0!	1	#DIV/0!
Pgk2|18663	-1	#DIV/0!	1	#DIV/0!
Prss29|114662	-1	#DIV/0!	1	#DIV/0!
Vmn2r-ps11|319210	-1	#DIV/0!	1	#DIV/0!
Olfr1163|258638	-1	#DIV/0!	1	#DIV/0!
Olfr1057|404325	-1	#DIV/0!	1	#DIV/0!
Speer3|71026	-1	#DIV/0!	1	#DIV/0!
Tubb3|22152	-1	#DIV/0!	1	#DIV/0!
Snord11|100217421	-1	#DIV/0!	1	#DIV/0!
Vmn1r25|113865	-1	#DIV/0!	1	#DIV/0!
Vmn1r149|100043051	-1	#DIV/0!	1	#DIV/0!
Olfr194|433031	-1	#DIV/0!	1	#DIV/0!
4932415M13Rik|211496	-1	#DIV/0!	1	#DIV/0!
Gpbar1|227289	-1	#DIV/0!	1	#DIV/0!
Vmn1r7|434016	-1	#DIV/0!	1	#DIV/0!
Myo1a|432516	-1	#DIV/0!	1	#DIV/0!
Vmn1r204|632793	-1	#DIV/0!	1	#DIV/0!
Lce1c|73719	-1	#DIV/0!	1	#DIV/0!
Vmn2r120|224916	-1	#DIV/0!	1	#DIV/0!
Cpne6|12891	-1	#DIV/0!	1	#DIV/0!
Rtp1|239766	-1	#DIV/0!	1	#DIV/0!
Zic3|22773	-1	#DIV/0!	1	#DIV/0!

Olfr103|258830	-1	#DIV/0!	1	#DIV/0!
BC051070|229688	-1	#DIV/0!	1	#DIV/0!
Olfr1497|258736	-1	#DIV/0!	1	#DIV/0!
Mir363|723852	-1	#DIV/0!	1	#DIV/0!
Vmn1r15|113863	-1	#DIV/0!	1	#DIV/0!
Vmn1r201|171255	-1	#DIV/0!	1	#DIV/0!
Olfr95|258506	-1	#DIV/0!	1	#DIV/0!
Il31|76399	-1	#DIV/0!	1	#DIV/0!
Cyp2w1|545817	-1	#DIV/0!	1	#DIV/0!
Pde11a|241489	-1	#DIV/0!	1	#DIV/0!
Olfr937|258431	-1	#DIV/0!	1	#DIV/0!
Mir196a-1|387191	-1	#DIV/0!	1	#DIV/0!
Gm5591|434171	-1	#DIV/0!	1	#DIV/0!
Klk13|626834	-1	#DIV/0!	1	#DIV/0!
1700016C15Rik|69428	-1	#DIV/0!	1	#DIV/0!
Arx|11878	-1	#DIV/0!	1	#DIV/0!
Slc22a16|70840	-1	#DIV/0!	1	#DIV/0!
Vmn1r233|171236	-1	#DIV/0!	1	#DIV/0!
Ntn5|243967	-1	#DIV/0!	1	#DIV/0!
BC049730|232972	-1	#DIV/0!	1	#DIV/0!
Cacna2d2|56808	-1	#DIV/0!	1	#DIV/0!
Tlx3|27140	-1	#DIV/0!	1	#DIV/0!
Mir374c|100526513	-1	#DIV/0!	1	#DIV/0!
Olfr1270|258987	-1	#DIV/0!	1	#DIV/0!
Olfr963|258087	-1	#DIV/0!	1	#DIV/0!
Olfr1222|258177	-1	#DIV/0!	1	#DIV/0!
4931440L10Rik|71001	-1	#DIV/0!	1	#DIV/0!
Try5|103964	-1	#DIV/0!	1	#DIV/0!
Olfr220|546747	-1	#DIV/0!	1	#DIV/0!
Defb41|77673	-1	#DIV/0!	1	#DIV/0!
Olfr572|259093	-1	#DIV/0!	1	#DIV/0!
Mir875|100124469	-1	#DIV/0!	1	#DIV/0!
Pou5f1|18999	-1	#DIV/0!	1	#DIV/0!
Lpcat2b|70902	-1	#DIV/0!	1	#DIV/0!
4933421I07Rik|71162	-1	#DIV/0!	1	#DIV/0!
5430402E10Rik|71351	-1	#DIV/0!	1	#DIV/0!
Ccdc103|73293	-1	#DIV/0!	1	#DIV/0!
Tbc1d21|74286	-1	#DIV/0!	1	#DIV/0!
Spata21|329972	-1	#DIV/0!	1	#DIV/0!
4930519F16Rik|75106	-1	#DIV/0!	1	#DIV/0!
Pramel5|384077	-1	#DIV/0!	1	#DIV/0!
Mir412|723913	-1	#DIV/0!	1	#DIV/0!
Lingo3|237403	-1	#DIV/0!	1	#DIV/0!
Olfr167|258937	-1	#DIV/0!	1	#DIV/0!
Mir3471-1|100499521	-1	#DIV/0!	1	#DIV/0!
Ces2f|71903	-1	#DIV/0!	1	#DIV/0!
Ido1|15930	-1	#DIV/0!	1	#DIV/0!
1700015F17Rik|381716	-1	#DIV/0!	1	#DIV/0!
Mir154|387172	-1	#DIV/0!	1	#DIV/0!
Adm2|223780	-1	#DIV/0!	1	#DIV/0!
Mir330|724063	-1	#DIV/0!	1	#DIV/0!
Padi3|18601	-1	#DIV/0!	1	#DIV/0!
Pcdha12|192164	-1	#DIV/0!	1	#DIV/0!

Mir705|735267	-1	#DIV/0!	1	#DIV/0!
Olfr1465|258121	-1	#DIV/0!	1	#DIV/0!
Mir3072|100499517	-1	#DIV/0!	1	#DIV/0!
Ifnab|15974	-1	#DIV/0!	1	#DIV/0!
Myoc|17926	-1	#DIV/0!	1	#DIV/0!
Mir27b|387221	-1	#DIV/0!	1	#DIV/0!
Htr1b|15551	-1	#DIV/0!	1	#DIV/0!
Skint1|639781	-1	#DIV/0!	1	#DIV/0!
Dmrtc2|71241	-1	#DIV/0!	1	#DIV/0!
Mir1968|100316713	-1	#DIV/0!	1	#DIV/0!
Tmprss5|80893	-1	#DIV/0!	1	#DIV/0!
Mir344e|100526541	-1	#DIV/0!	1	#DIV/0!
Cts8-ps|116911	-1	#DIV/0!	1	#DIV/0!
Olfr452|258207	-1	#DIV/0!	1	#DIV/0!
Tmem90b|433485	-1	#DIV/0!	1	#DIV/0!
4933406F09Rik|68308	-1	#DIV/0!	1	#DIV/0!
Dyrk4|101320	-1	#DIV/0!	1	#DIV/0!
BC089491|280621	-1	#DIV/0!	1	#DIV/0!
Prss40|21756	-1	#DIV/0!	1	#DIV/0!
Mir667|751552	-1	#DIV/0!	1	#DIV/0!
Olfr419|258710	-1	#DIV/0!	1	#DIV/0!
Mir682|751556	-1	#DIV/0!	1	#DIV/0!
Arhgap36|75404	-1	#DIV/0!	1	#DIV/0!
Mei4|75033	-1	#DIV/0!	1	#DIV/0!
Olfr1286|277562	-1	#DIV/0!	1	#DIV/0!
Olfr947-ps1|257924	-1	#DIV/0!	1	#DIV/0!
Mir302a|723920	-1	#DIV/0!	1	#DIV/0!
Rs1|20147	-1	#DIV/0!	1	#DIV/0!
Mab21l3|242125	-1	#DIV/0!	1	#DIV/0!
Alox8|11688	-1	#DIV/0!	1	#DIV/0!
Sox30|214105	-1	#DIV/0!	1	#DIV/0!
Ptprv|13924	-1	#DIV/0!	1	#DIV/0!
Vmn2r48|625580	-1	#DIV/0!	1	#DIV/0!
Gm12298|100038666	-1	#DIV/0!	1	#DIV/0!
Mir380|723859	-1	#DIV/0!	1	#DIV/0!
1700008A04Rik|69351	-1	#DIV/0!	1	#DIV/0!
Vmn2r16|384220	-1	#DIV/0!	1	#DIV/0!
Gstt4|75886	-1	#DIV/0!	1	#DIV/0!
Olfr908|258872	-1	#DIV/0!	1	#DIV/0!
Pcdha2|353234	-1	#DIV/0!	1	#DIV/0!
2010001E11Rik|72045	-1	#DIV/0!	1	#DIV/0!
Patl2|67578	-1	#DIV/0!	1	#DIV/0!
Prss33|353130	-1	#DIV/0!	1	#DIV/0!
Chat|12647	-1	#DIV/0!	1	#DIV/0!
Strc|140476	-1	#DIV/0!	1	#DIV/0!
Pcdha10|12943	-1	#DIV/0!	1	#DIV/0!
Olfr559|259116	-1	#DIV/0!	1	#DIV/0!
Crygc|12966	-1	#DIV/0!	1	#DIV/0!
Cdh18|320865	-1	#DIV/0!	1	#DIV/0!
Scnn1g|20278	-1	#DIV/0!	1	#DIV/0!
Gm8267|666744	-1	#DIV/0!	1	#DIV/0!
Nanos3|244551	-1	#DIV/0!	1	#DIV/0!
Kcnk12|210741	-1	#DIV/0!	1	#DIV/0!

Cpa6|329093	-1	#DIV/0!	1	#DIV/0!
Fmo9|240894	-1	#DIV/0!	1	#DIV/0!
Vmn2r-ps129|665976	-1	#DIV/0!	1	#DIV/0!
Rfpl4b|215919	-1	#DIV/0!	1	#DIV/0!
1700012B09Rik|69325	-1	#DIV/0!	1	#DIV/0!
1700123L14Rik|78482	-1	#DIV/0!	1	#DIV/0!
Fam92b|436062	-1	#DIV/0!	1	#DIV/0!
Ins2|16334	-1	#DIV/0!	1	#DIV/0!
Nek11|208583	-1	#DIV/0!	1	#DIV/0!
Gm6985|629581	-1	#DIV/0!	1	#DIV/0!
4930458L03Rik|78124	-1	#DIV/0!	1	#DIV/0!
Olfr748|258113	-1	#DIV/0!	1	#DIV/0!
Olfr781|258723	-1	#DIV/0!	1	#DIV/0!
Olfr481|258927	-1	#DIV/0!	1	#DIV/0!
Fabp12|75497	-1	#DIV/0!	1	#DIV/0!
Psg21|72242	-1	#DIV/0!	1	#DIV/0!
Mir23b|387217	-1	#DIV/0!	1	#DIV/0!
Muc20|224116	-1	#DIV/0!	1	#DIV/0!
Snora35|100303736	-1	#DIV/0!	1	#DIV/0!
Mybphl|68753	-1	#DIV/0!	1	#DIV/0!
Fam43b|625638	-1	#DIV/0!	1	#DIV/0!
Vmn1r31|626828	-1	#DIV/0!	1	#DIV/0!
Ly6h|23934	-1	#DIV/0!	1	#DIV/0!
Olfr201|258996	-1	#DIV/0!	1	#DIV/0!
Kdm5d|20592	-1	#DIV/0!	1	#DIV/0!
Adamts8|30806	-1	#DIV/0!	1	#DIV/0!
Grm5|108071	-1	#DIV/0!	1	#DIV/0!
Vmn1r23|171197	-1	#DIV/0!	1	#DIV/0!
Fam122c|73866	-1	#DIV/0!	1	#DIV/0!
Fam115e|403088	-1	#DIV/0!	1	#DIV/0!
Olfr1179|258919	-1	#DIV/0!	1	#DIV/0!
Dsg1a|13510	-1	#DIV/0!	1	#DIV/0!
4930571K23Rik|75861	-1	#DIV/0!	1	#DIV/0!
Olfr1134|259032	-1	#DIV/0!	1	#DIV/0!
Otx2|18424	-1	#DIV/0!	1	#DIV/0!
Olfr1373|211472	-1	#DIV/0!	1	#DIV/0!
Sowahd|245381	-1	#DIV/0!	1	#DIV/0!
Htr2a|15558	-1	#DIV/0!	1	#DIV/0!
Klra21|93968	-1	#DIV/0!	1	#DIV/0!
Chrne|11448	-1	#DIV/0!	1	#DIV/0!
Lyzl4|69032	-1	#DIV/0!	1	#DIV/0!
Mir680-2|751551	-1	#DIV/0!	1	#DIV/0!
Rhox9|104384	-1	#DIV/0!	1	#DIV/0!
Tnfsf8|21949	-1	#DIV/0!	1	#DIV/0!
Tex19.2|70956	-1	#DIV/0!	1	#DIV/0!
3830403N18Rik|70691	-1	#DIV/0!	1	#DIV/0!
Tll2|24087	-1	#DIV/0!	1	#DIV/0!
Myocd|214384	-1	#DIV/0!	1	#DIV/0!
Vmn2r21|546912	-1	#DIV/0!	1	#DIV/0!
Trpd52l3|66745	-1	#DIV/0!	1	#DIV/0!
Krtap20-2|622935	-1	#DIV/0!	1	#DIV/0!
Olfr118|404308	-1	#DIV/0!	1	#DIV/0!
Csn2|12991	-1	#DIV/0!	1	#DIV/0!

St6galnac1|20445	-1	#DIV/0!	1	#DIV/0!
Sstr4|20608	-1	#DIV/0!	1	#DIV/0!
Trpa1|277328	-1	#DIV/0!	1	#DIV/0!
Kera|16545	-1	#DIV/0!	1	#DIV/0!
Olfr1256|258985	-1	#DIV/0!	1	#DIV/0!
Kis2|751866	-1	#DIV/0!	1	#DIV/0!
2310002L13Rik|75577	-1	#DIV/0!	1	#DIV/0!
Mir1190|100316805	-1	#DIV/0!	1	#DIV/0!
Cpxcr1|382239	-1	#DIV/0!	1	#DIV/0!
Il24|93672	-1	#DIV/0!	1	#DIV/0!
Sbp|20234	-1	#DIV/0!	1	#DIV/0!
Tnfsf11|21943	-1	#DIV/0!	1	#DIV/0!
Olfr132|257889	-1	#DIV/0!	1	#DIV/0!
4930522H14Rik|67646	-1	#DIV/0!	1	#DIV/0!
Speer4b|73526	-1	#DIV/0!	1	#DIV/0!
4932438H23Rik|74387	-1	#DIV/0!	1	#DIV/0!
Shbg|20415	-1	#DIV/0!	1	#DIV/0!
Snora41|100217464	-1	#DIV/0!	1	#DIV/0!
Cacng8|81905	-1	#DIV/0!	1	#DIV/0!
Prl2c2|18811	-1	#DIV/0!	1	#DIV/0!
Olfr1310|258441	-1	#DIV/0!	1	#DIV/0!
Olfr1259|258338	-1	#DIV/0!	1	#DIV/0!
Gm6121|619991	-1	#DIV/0!	1	#DIV/0!
Tcp10c|100041352	-1	#DIV/0!	1	#DIV/0!
Gm2863|100040606	-1	#DIV/0!	1	#DIV/0!
V1rg10|171266	-1	#DIV/0!	1	#DIV/0!
Opn4|30044	-1	#DIV/0!	1	#DIV/0!
Npy5r|18168	-1	#DIV/0!	1	#DIV/0!
Olfr796|258933	-1	#DIV/0!	1	#DIV/0!
Ubl4b|67591	-1	#DIV/0!	1	#DIV/0!
Lrrtm3|216028	-1	#DIV/0!	1	#DIV/0!
Zfp534|100043100	-1	#DIV/0!	1	#DIV/0!
Dao|13142	-1	#DIV/0!	1	#DIV/0!
6530411M01Rik|67791	-1	#DIV/0!	1	#DIV/0!
Gm17660|100271704	-1	#DIV/0!	1	#DIV/0!
Olfr768|258863	-1	#DIV/0!	1	#DIV/0!
Krt86|16679	-1	#DIV/0!	1	#DIV/0!
Mir764|791081	-1	#DIV/0!	1	#DIV/0!
Snord22|100127111	-1	#DIV/0!	1	#DIV/0!
Ccdc13|434446	-1	#DIV/0!	1	#DIV/0!
Defb2|13215	-1	#DIV/0!	1	#DIV/0!
Elf5|13711	-1	#DIV/0!	1	#DIV/0!
Padi1|18599	-1	#DIV/0!	1	#DIV/0!
Lce1l|73730	-1	#DIV/0!	1	#DIV/0!
Klf14|619665	-1	#DIV/0!	1	#DIV/0!
Mageb18|215641	-1	#DIV/0!	1	#DIV/0!
Crtac1|72832	-1	#DIV/0!	1	#DIV/0!
Lce1i|76585	-1	#DIV/0!	1	#DIV/0!
Rhox2b|100039913	-1	#DIV/0!	1	#DIV/0!
Ccdc42|276920	-1	#DIV/0!	1	#DIV/0!
4921504E06Rik|70909	-1	#DIV/0!	1	#DIV/0!
Dnajb8|56691	-1	#DIV/0!	1	#DIV/0!
Mir669i|100316668	-1	#DIV/0!	1	#DIV/0!

1700020N01Rik|67692	-1	#DIV/0!	1	#DIV/0!
Gm4301|100043224	-1	#DIV/0!	1	#DIV/0!
Il1f10|215274	-1	#DIV/0!	1	#DIV/0!
Epha6|13840	-1	#DIV/0!	1	#DIV/0!
Olfr1209|258453	-1	#DIV/0!	1	#DIV/0!
Csn1s2b|12992	-1	#DIV/0!	1	#DIV/0!
Mirlet7d|387247	-1	#DIV/0!	1	#DIV/0!
Olfr1441|258678	-1	#DIV/0!	1	#DIV/0!
Atp12a|192113	-1	#DIV/0!	1	#DIV/0!
Snord49b|100217426	-1	#DIV/0!	1	#DIV/0!
Usp44|327799	-1	#DIV/0!	1	#DIV/0!
Otor|57329	-1	#DIV/0!	1	#DIV/0!
Sox2|20674	-1	#DIV/0!	1	#DIV/0!
Tacr1|21336	-1	#DIV/0!	1	#DIV/0!
Chrm3|12671	-1	#DIV/0!	1	#DIV/0!
Olfr1385|258027	-1	#DIV/0!	1	#DIV/0!
1700111N16Rik|74305	-1	#DIV/0!	1	#DIV/0!
Gm5647|434881	-1	#DIV/0!	1	#DIV/0!
Mir141|387159	-1	#DIV/0!	1	#DIV/0!
Mir542|723901	-1	#DIV/0!	1	#DIV/0!
Slc16a4|229699	-1	#DIV/0!	1	#DIV/0!
Gm10436|100039315	-1	#DIV/0!	1	#DIV/0!
Vmn1r93|404290	-1	#DIV/0!	1	#DIV/0!
1700073E17Rik|381827	-1	#DIV/0!	1	#DIV/0!
Klk14|317653	-1	#DIV/0!	1	#DIV/0!
Spata4|69281	-1	#DIV/0!	1	#DIV/0!
Cnfn|72383	-1	#DIV/0!	1	#DIV/0!
Gm2022|100039052	-1	#DIV/0!	1	#DIV/0!
Mep1a|17287	-1	#DIV/0!	1	#DIV/0!
Tmem132b|208151	-1	#DIV/0!	1	#DIV/0!
Trim30e-ps1|625321	-1	#DIV/0!	1	#DIV/0!
Mir33|723897	-1	#DIV/0!	1	#DIV/0!
Mir344-2|100124439	-1	#DIV/0!	1	#DIV/0!
Gm7444|665005	-1	#DIV/0!	1	#DIV/0!
Snord2|100216532	-1	#DIV/0!	1	#DIV/0!
Rdh12|77974	-1	#DIV/0!	1	#DIV/0!
Olfr1352|259074	-1	#DIV/0!	1	#DIV/0!
Gm10466|100038617	-1	#DIV/0!	1	#DIV/0!
Hoxd11|15431	-1	#DIV/0!	1	#DIV/0!
Hoxc12|15421	-1	#DIV/0!	1	#DIV/0!
Snora5c|100217467	-1	#DIV/0!	1	#DIV/0!
Kcnmb2|72413	-1	#DIV/0!	1	#DIV/0!
Ndst4|64580	-1	#DIV/0!	1	#DIV/0!
Vit|74199	-1	#DIV/0!	1	#DIV/0!
Olfr917|258183	-1	#DIV/0!	1	#DIV/0!
Lim2|233187	-1	#DIV/0!	1	#DIV/0!
Bhlha9|320522	-1	#DIV/0!	1	#DIV/0!
Prl7c1|67505	-1	#DIV/0!	1	#DIV/0!
Zcchc18|66995	-1	#DIV/0!	1	#DIV/0!
Olfr1281|257979	-1	#DIV/0!	1	#DIV/0!
Ptpn20|19256	-1	#DIV/0!	1	#DIV/0!
Olfr1102|228228	-1	#DIV/0!	1	#DIV/0!
Olfm4|380924	-1	#DIV/0!	1	#DIV/0!

Ripply2|382089	-1	#DIV/0!	1	#DIV/0!
Olfr782|257985	-1	#DIV/0!	1	#DIV/0!
Slc9a10|208169	-1	#DIV/0!	1	#DIV/0!
1700023E05Rik|71868	-1	#DIV/0!	1	#DIV/0!
Mir3112|100526514	-1	#DIV/0!	1	#DIV/0!
Gm3434|100041618	-1	#DIV/0!	1	#DIV/0!
Clca5|229933	-1	#DIV/0!	1	#DIV/0!
Krtap8-2|16704	-1	#DIV/0!	1	#DIV/0!
Vmn1r115|667273	-1	#DIV/0!	1	#DIV/0!
BC048679|210321	-1	#DIV/0!	1	#DIV/0!
Olfr1386|257888	-1	#DIV/0!	1	#DIV/0!
Btnl3|100038862	-1	#DIV/0!	1	#DIV/0!
Ptchd1|211612	-1	#DIV/0!	1	#DIV/0!
Gk2|14626	-1	#DIV/0!	1	#DIV/0!
Olfr706|258350	-1	#DIV/0!	1	#DIV/0!
Snord57|100217428	-1	#DIV/0!	1	#DIV/0!
Ankrd7|75196	-1	#DIV/0!	1	#DIV/0!
Gm9731|732482	-1	#DIV/0!	1	#DIV/0!
C130026I21Rik|620078	-1	#DIV/0!	1	#DIV/0!
Sox14|20669	-1	#DIV/0!	1	#DIV/0!
Scarna2|100217438	-1	#DIV/0!	1	#DIV/0!
Poln|272158	-1	#DIV/0!	1	#DIV/0!
Cyp11a1|13070	-1	#DIV/0!	1	#DIV/0!
Ndp|17986	-1	#DIV/0!	1	#DIV/0!
Gm3020|100040880	-1	#DIV/0!	1	#DIV/0!
Zic2|22772	-1	#DIV/0!	1	#DIV/0!
Ctla4|12477	-1	#DIV/0!	1	#DIV/0!
Rpl10l|238217	-1	#DIV/0!	1	#DIV/0!
Cpne9|211232	-1	#DIV/0!	1	#DIV/0!
Gm10536|100038410	-1	#DIV/0!	1	#DIV/0!
H2-M10.6|399549	-1	#DIV/0!	1	#DIV/0!
Mrgprb5|404239	-1	#DIV/0!	1	#DIV/0!
Nav3|260315	-1	#DIV/0!	1	#DIV/0!
Psors1c2|57390	-1	#DIV/0!	1	#DIV/0!
Mir196b|723820	-1	#DIV/0!	1	#DIV/0!
Gm4312|100043247	-1	#DIV/0!	1	#DIV/0!
Lhfpl3|269629	-1	#DIV/0!	1	#DIV/0!
4930567H12Rik|75930	-1	#DIV/0!	1	#DIV/0!
Vmn1r78|171242	-1	#DIV/0!	1	#DIV/0!
Dgkk|331374	-1	#DIV/0!	1	#DIV/0!
4930548H24Rik|67656	-1	#DIV/0!	1	#DIV/0!
Grem1|23892	-1	#DIV/0!	1	#DIV/0!
Olfr1419|257938	-1	#DIV/0!	1	#DIV/0!
Olfr331|258179	-1	#DIV/0!	1	#DIV/0!
Olfr376|258924	-1	#DIV/0!	1	#DIV/0!
Nrg3|18183	-1	#DIV/0!	1	#DIV/0!
Cd109|235505	-1	#DIV/0!	1	#DIV/0!
Mir770|791079	-1	#DIV/0!	1	#DIV/0!
Gm13109|329984	-1	#DIV/0!	1	#DIV/0!
Mir1952|100316816	-1	#DIV/0!	1	#DIV/0!
Olfr1406|258758	-1	#DIV/0!	1	#DIV/0!
Fgf2|14173	-1	#DIV/0!	1	#DIV/0!
Pcsk2|18549	-1	#DIV/0!	1	#DIV/0!

Vmn1r223|100036518	-1	#DIV/0!	1	#DIV/0!
Olfr1272|258982	-1	#DIV/0!	1	#DIV/0!
1700018C11Rik|75524	-1	#DIV/0!	1	#DIV/0!
Cyp2j13|230459	-1	#DIV/0!	1	#DIV/0!
Gm156|232415	-1	#DIV/0!	1	#DIV/0!
Zp2|22787	-1	#DIV/0!	1	#DIV/0!
Mir1938|100316693	-1	#DIV/0!	1	#DIV/0!
Vmn2r116|619697	-1	#DIV/0!	1	#DIV/0!
Gm5129|332993	-1	#DIV/0!	1	#DIV/0!
Atp2c2|69047	-1	#DIV/0!	1	#DIV/0!
Vmn1r229|171224	-1	#DIV/0!	1	#DIV/0!
Olfr77|258336	-1	#DIV/0!	1	#DIV/0!
Gm9999|629141	-1	#DIV/0!	1	#DIV/0!
Olfr1100|258587	-1	#DIV/0!	1	#DIV/0!
Olfr888|258416	-1	#DIV/0!	1	#DIV/0!
Klk1b1|16623	-1	#DIV/0!	1	#DIV/0!
Has2|15117	-1	#DIV/0!	1	#DIV/0!
Olfr1264|258206	-1	#DIV/0!	1	#DIV/0!
Olfr715|258776	-1	#DIV/0!	1	#DIV/0!
Defb29|75400	-1	#DIV/0!	1	#DIV/0!
Syt14|329324	-1	#DIV/0!	1	#DIV/0!
Defa-ps1|727720	-1	#DIV/0!	1	#DIV/0!
Olfr686|259072	-1	#DIV/0!	1	#DIV/0!
Olfr901|258028	-1	#DIV/0!	1	#DIV/0!
Plac1|56096	-1	#DIV/0!	1	#DIV/0!
1700042B14Rik|73347	-1	#DIV/0!	1	#DIV/0!
Adcy10|271639	-1	#DIV/0!	1	#DIV/0!
Olfr1306|258023	-1	#DIV/0!	1	#DIV/0!
Mir139|387157	-1	#DIV/0!	1	#DIV/0!
Lemd1|213409	-1	#DIV/0!	1	#DIV/0!
1600029O15Rik|665268	-1	#DIV/0!	1	#DIV/0!
Nell2|54003	-1	#DIV/0!	1	#DIV/0!
Olfr288|545140	-1	#DIV/0!	1	#DIV/0!
Epx|13861	-1	#DIV/0!	1	#DIV/0!
Olfr1162|258105	-1	#DIV/0!	1	#DIV/0!
Bcat1|12035	-1	#DIV/0!	1	#DIV/0!
Psg18|26438	-1	#DIV/0!	1	#DIV/0!
Vmn1r69|252904	-1	#DIV/0!	1	#DIV/0!
Gm15114|100034729	-1	#DIV/0!	1	#DIV/0!
Dppa2|73703	-1	#DIV/0!	1	#DIV/0!
Olfr466|258816	-1	#DIV/0!	1	#DIV/0!
Hist1h3b|319150	-1	#DIV/0!	1	#DIV/0!
Foxb2|14240	-1	#DIV/0!	1	#DIV/0!
Olfr736|258660	-1	#DIV/0!	1	#DIV/0!
Olfr215|258438	-1	#DIV/0!	1	#DIV/0!
Ccdc135|330830	-1	#DIV/0!	1	#DIV/0!
Uts2|24111	-1	#DIV/0!	1	#DIV/0!
Hyal5|74468	-1	#DIV/0!	1	#DIV/0!
Hoxc9|15427	-1	#DIV/0!	1	#DIV/0!
Prl3c1|27372	-1	#DIV/0!	1	#DIV/0!
Tex11|83558	-1	#DIV/0!	1	#DIV/0!
Vmn1r12|626397	-1	#DIV/0!	1	#DIV/0!
Gm525|217071	-1	#DIV/0!	1	#DIV/0!

Slco1c1|58807	-1	#DIV/0!	1	#DIV/0!
Fscn3|56223	-1	#DIV/0!	1	#DIV/0!
Ssxb5|387586	-1	#DIV/0!	1	#DIV/0!
Tmem92|544806	-1	#DIV/0!	1	#DIV/0!
Olfr410|258702	-1	#DIV/0!	1	#DIV/0!
Mir1898|100316684	-1	#DIV/0!	1	#DIV/0!
Gm4971|244061	-1	#DIV/0!	1	#DIV/0!
Lin28a|83557	-1	#DIV/0!	1	#DIV/0!
Pkd1l2|76645	-1	#DIV/0!	1	#DIV/0!
Olfr1490|258098	-1	#DIV/0!	1	#DIV/0!
Olfr502|258734	-1	#DIV/0!	1	#DIV/0!
Trim75|333307	-1	#DIV/0!	1	#DIV/0!
Krtap10-4|100191037	-1	#DIV/0!	1	#DIV/0!
Drd4|13491	-1	#DIV/0!	1	#DIV/0!
Trim43b|666747	-1	#DIV/0!	1	#DIV/0!
Defa-ps13|654456	-1	#DIV/0!	1	#DIV/0!
Olfr29-ps1|29848	-1	#DIV/0!	1	#DIV/0!
Olfr1168|258524	-1	#DIV/0!	1	#DIV/0!
Il1r2|16178	-1	#DIV/0!	1	#DIV/0!
Magea5|17141	-1	#DIV/0!	1	#DIV/0!
Rtdr1|71236	-1	#DIV/0!	1	#DIV/0!
Olfr1378|258912	-1	#DIV/0!	1	#DIV/0!
9230113P08Rik|77908	-1	#DIV/0!	1	#DIV/0!
Lrrc36|270091	-1	#DIV/0!	1	#DIV/0!
S100b|20203	-1	#DIV/0!	1	#DIV/0!
Gkn1|66283	-1	#DIV/0!	1	#DIV/0!
Gsdmc3|270328	-1	#DIV/0!	1	#DIV/0!
Olfr127|258374	-1	#DIV/0!	1	#DIV/0!
Mir761|791075	-1	#DIV/0!	1	#DIV/0!
Tex13|83555	-1	#DIV/0!	1	#DIV/0!
Tmem196|217951	-1	#DIV/0!	1	#DIV/0!
Olfr110|258325	-1	#DIV/0!	1	#DIV/0!
Oas1f|243262	-1	#DIV/0!	1	#DIV/0!
Olfr893|258333	-1	#DIV/0!	1	#DIV/0!
Olfr735|257909	-1	#DIV/0!	1	#DIV/0!
Gm14124|100216455	-1	#DIV/0!	1	#DIV/0!
Slc10a4|231290	-1	#DIV/0!	1	#DIV/0!
Trim61|260296	-1	#DIV/0!	1	#DIV/0!
Mtap7d2|78283	-1	#DIV/0!	1	#DIV/0!
Try4|22074	-1	#DIV/0!	1	#DIV/0!
E230019M04Rik|331537	-1	#DIV/0!	1	#DIV/0!
Hoxc13|15422	-1	#DIV/0!	1	#DIV/0!
2610109H07Rik|70433	-1	#DIV/0!	1	#DIV/0!
2410018L13Rik|69732	-1	#DIV/0!	1	#DIV/0!
Gm12633|619842	-1	#DIV/0!	1	#DIV/0!
Dsg1b|225256	-1	#DIV/0!	1	#DIV/0!
Crisp1|11571	-1	#DIV/0!	1	#DIV/0!
Alx1|216285	-1	#DIV/0!	1	#DIV/0!
Lce6a|78382	-1	#DIV/0!	1	#DIV/0!
Six4|20474	-1	#DIV/0!	1	#DIV/0!
Scd4|329065	-1	#DIV/0!	1	#DIV/0!
Plcz1|114875	-1	#DIV/0!	1	#DIV/0!
Serpinb9b|20706	-1	#DIV/0!	1	#DIV/0!

Mir666|751521	-1	#DIV/0!	1	#DIV/0!
Olfr1232|258320	-1	#DIV/0!	1	#DIV/0!
Mir1186b|100499527	-1	#DIV/0!	1	#DIV/0!
Kcnj1|56379	-1	#DIV/0!	1	#DIV/0!
Sncb|104069	-1	#DIV/0!	1	#DIV/0!
Gm5820|545253	-1	#DIV/0!	1	#DIV/0!
Gm17252|100312987	-1	#DIV/0!	1	#DIV/0!
Mir509|100124449	-1	#DIV/0!	1	#DIV/0!
Olfr1093|258363	-1	#DIV/0!	1	#DIV/0!
Vmn1r160|620758	-1	#DIV/0!	1	#DIV/0!
Olfr58|18358	-1	#DIV/0!	1	#DIV/0!
Mir208b|100124433	-1	#DIV/0!	1	#DIV/0!
Psg29|114872	-1	#DIV/0!	1	#DIV/0!
BB019430|103505	-1	#DIV/0!	1	#DIV/0!
Olfr965|258165	-1	#DIV/0!	1	#DIV/0!
Mir504|100124476	-1	#DIV/0!	1	#DIV/0!
Mir599|100316736	-1	#DIV/0!	1	#DIV/0!
Fbxo48|319701	-1	#DIV/0!	1	#DIV/0!
Kcna10|242151	-1	#DIV/0!	1	#DIV/0!
Glycam1|14663	-1	#DIV/0!	1	#DIV/0!
Vcan|13003	-1	#DIV/0!	1	#DIV/0!
Ubqln3|244178	-1	#DIV/0!	1	#DIV/0!
Spink7|408198	-1	#DIV/0!	1	#DIV/0!
Oc90|18256	-1	#DIV/0!	1	#DIV/0!
Wnt16|93735	-1	#DIV/0!	1	#DIV/0!
Ceacam12|67315	-1	#DIV/0!	1	#DIV/0!
Lrp1b|94217	-1	#DIV/0!	1	#DIV/0!
Sprr2i|20763	-1	#DIV/0!	1	#DIV/0!
Cym|229697	-1	#DIV/0!	1	#DIV/0!
Gm1679|381667	-1	#DIV/0!	1	#DIV/0!
Magea3|17139	-1	#DIV/0!	1	#DIV/0!
Olfr1186|258523	-1	#DIV/0!	1	#DIV/0!
Lcn6|620709	-1	#DIV/0!	1	#DIV/0!
Gm884|380730	-1	#DIV/0!	1	#DIV/0!
Capsl|75568	-1	#DIV/0!	1	#DIV/0!
Snord87|266793	-1	#DIV/0!	1	#DIV/0!
Kif4-ps|74947	-1	#DIV/0!	1	#DIV/0!
Gpr75|237716	-1	#DIV/0!	1	#DIV/0!
Tcte1|21645	-1	#DIV/0!	1	#DIV/0!
Abhd12b|328121	-1	#DIV/0!	1	#DIV/0!
Clec4b1|69810	-1	#DIV/0!	1	#DIV/0!
Lrrc30|240131	-1	#DIV/0!	1	#DIV/0!
Gm10486|100039550	-1	#DIV/0!	1	#DIV/0!
Gm16119|100271841	-1	#DIV/0!	1	#DIV/0!
Irx5|54352	-1	#DIV/0!	1	#DIV/0!
Odz2|23964	-1	#DIV/0!	1	#DIV/0!
Zfyve28|231125	-1	#DIV/0!	1	#DIV/0!
4933400A11Rik|66747	-1	#DIV/0!	1	#DIV/0!
Obox1|71468	-1	#DIV/0!	1	#DIV/0!
Pramel1|83491	-1	#DIV/0!	1	#DIV/0!
Gm8817|667794	-1	#DIV/0!	1	#DIV/0!
Olfr656|259078	-1	#DIV/0!	1	#DIV/0!
Cmtm5|67272	-1	#DIV/0!	1	#DIV/0!

Accsl|381411	-1	#DIV/0!	1	#DIV/0!
Gm13078|277666	-1	#DIV/0!	1	#DIV/0!
Mir338|723844	-1	#DIV/0!	1	#DIV/0!
Olfr538|258201	-1	#DIV/0!	1	#DIV/0!
Padi6|242726	-1	#DIV/0!	1	#DIV/0!
Gal|14419	-1	#DIV/0!	1	#DIV/0!
1700109F18Rik|73429	-1	#DIV/0!	1	#DIV/0!
Gpr63|81006	-1	#DIV/0!	1	#DIV/0!
Krtap14|23927	-1	#DIV/0!	1	#DIV/0!
Cldn8|54420	-1	#DIV/0!	1	#DIV/0!
Tmc5|74424	-1	#DIV/0!	1	#DIV/0!
Frmd3|242506	-1	#DIV/0!	1	#DIV/0!
Slc38a5|209837	-1	#DIV/0!	1	#DIV/0!
Vmn1r80|171238	-1	#DIV/0!	1	#DIV/0!
Gm20580|100126777	-1	#DIV/0!	1	#DIV/0!
Slitrk2|245450	-1	#DIV/0!	1	#DIV/0!
Wif1|24117	-1	#DIV/0!	1	#DIV/0!
4922505E12Rik|433386	-1	#DIV/0!	1	#DIV/0!
Olfr168|258354	-1	#DIV/0!	1	#DIV/0!
Olfr583|258752	-1	#DIV/0!	1	#DIV/0!
Gm7257|639025	-1	#DIV/0!	1	#DIV/0!
Vmn2r65|100009609	-1	#DIV/0!	1	#DIV/0!
Iqcf1|74267	-1	#DIV/0!	1	#DIV/0!
Olfr317|257931	-1	#DIV/0!	1	#DIV/0!
Sycn|68416	-1	#DIV/0!	1	#DIV/0!
4930448K20Rik|74662	-1	#DIV/0!	1	#DIV/0!
Pbsn|54192	-1	#DIV/0!	1	#DIV/0!
Gm11961|327860	-1	#DIV/0!	1	#DIV/0!
Olfr270|258600	-1	#DIV/0!	1	#DIV/0!
Mir199b|387239	-1	#DIV/0!	1	#DIV/0!
Gm1587|380920	-1	#DIV/0!	1	#DIV/0!
Mir223|723814	-1	#DIV/0!	1	#DIV/0!
Olfr1480|404339	-1	#DIV/0!	1	#DIV/0!
Olfr668|259061	-1	#DIV/0!	1	#DIV/0!
Olfr1216|258895	-1	#DIV/0!	1	#DIV/0!
Olfr75-ps1|258186	-1	#DIV/0!	1	#DIV/0!
Myt1l|17933	-1	#DIV/0!	1	#DIV/0!
Ccr1l1|12770	-1	#DIV/0!	1	#DIV/0!
Nlrp2|232827	-1	#DIV/0!	1	#DIV/0!
Lgr6|329252	-1	#DIV/0!	1	#DIV/0!
4833403I15Rik|74574	-1	#DIV/0!	1	#DIV/0!
Olfr180|258178	-1	#DIV/0!	1	#DIV/0!
Olfr1322|257978	-1	#DIV/0!	1	#DIV/0!
Pdcd1|18566	-1	#DIV/0!	1	#DIV/0!
Defa21|66298	-1	#DIV/0!	1	#DIV/0!
Btn3a3|632126	-1	#DIV/0!	1	#DIV/0!
Snord19|100217423	-1	#DIV/0!	1	#DIV/0!
Ropn1|76378	-1	#DIV/0!	1	#DIV/0!
Mir19a|723891	-1	#DIV/0!	1	#DIV/0!
4930578I06Rik|67750	-1	#DIV/0!	1	#DIV/0!
Mir1906-1|100316809	-1	#DIV/0!	1	#DIV/0!
Olfr1042|257941	-1	#DIV/0!	1	#DIV/0!
Mir3089|100526499	-1	#DIV/0!	1	#DIV/0!

Pcdhb1|93872	-1	#DIV/0!	1	#DIV/0!
Wnt3a|22416	-1	#DIV/0!	1	#DIV/0!
Tm4sf19|277203	-1	#DIV/0!	1	#DIV/0!
Ang4|219033	-1	#DIV/0!	1	#DIV/0!
D630041G03Rik|320749	-1	#DIV/0!	1	#DIV/0!
2310079G19Rik|69699	-1	#DIV/0!	1	#DIV/0!
1700123I01Rik|622554	-1	#DIV/0!	1	#DIV/0!
Neurod6|11922	-1	#DIV/0!	1	#DIV/0!
Chrna5|110835	-1	#DIV/0!	1	#DIV/0!
Mir720|735260	-1	#DIV/0!	1	#DIV/0!
Krtap3-2|66708	-1	#DIV/0!	1	#DIV/0!
Olfr134|258829	-1	#DIV/0!	1	#DIV/0!
Olfr945|258499	-1	#DIV/0!	1	#DIV/0!
Olfr493|258307	-1	#DIV/0!	1	#DIV/0!
Nol4|319211	-1	#DIV/0!	1	#DIV/0!
Olfr19|18316	-1	#DIV/0!	1	#DIV/0!
Gpr173|70771	-1	#DIV/0!	1	#DIV/0!
Olfr1370|258528	-1	#DIV/0!	1	#DIV/0!
Calca|12310	-1	#DIV/0!	1	#DIV/0!
Il19|329244	-1	#DIV/0!	1	#DIV/0!
Isx|71597	-1	#DIV/0!	1	#DIV/0!
Gm4598|100043706	-1	#DIV/0!	1	#DIV/0!
Gp6|243816	-1	#DIV/0!	1	#DIV/0!
Olfr120|258624	-1	#DIV/0!	1	#DIV/0!
Olfr622|259087	-1	#DIV/0!	1	#DIV/0!
Fcrl6|677296	-1	#DIV/0!	1	#DIV/0!
Kntc1|208628	-1	#DIV/0!	1	#DIV/0!
Olfr1245|258784	-1	#DIV/0!	1	#DIV/0!
Mir700|735285	-1	#DIV/0!	1	#DIV/0!
Olfr1431|258409	-1	#DIV/0!	1	#DIV/0!
Adad1|21744	-1	#DIV/0!	1	#DIV/0!
Fam26d|270711	-1	#DIV/0!	1	#DIV/0!
Rhox11|194738	-1	#DIV/0!	1	#DIV/0!
Olfr1211|258025	-1	#DIV/0!	1	#DIV/0!
Tas2r109|387343	-1	#DIV/0!	1	#DIV/0!
Mir698|735263	-1	#DIV/0!	1	#DIV/0!
Olfr1477|258691	-1	#DIV/0!	1	#DIV/0!
Mlc1|170790	-1	#DIV/0!	1	#DIV/0!
Xkr4|497097	-1	#DIV/0!	1	#DIV/0!
Olfr20|258925	-1	#DIV/0!	1	#DIV/0!
Mir434|723867	-1	#DIV/0!	1	#DIV/0!
Luzp4|434865	-1	#DIV/0!	1	#DIV/0!
Dnajc5b|66326	-1	#DIV/0!	1	#DIV/0!
Nespas|56802	-1	#DIV/0!	1	#DIV/0!
Otx1|18423	-1	#DIV/0!	1	#DIV/0!
Snap25|20614	-1	#DIV/0!	1	#DIV/0!
Olfr412|258153	-1	#DIV/0!	1	#DIV/0!
Pla2g2a|18780	-1	#DIV/0!	1	#DIV/0!
Mir1951|100316703	-1	#DIV/0!	1	#DIV/0!
Olfr969|258823	-1	#DIV/0!	1	#DIV/0!
Zscan4f|665902	-1	#DIV/0!	1	#DIV/0!
Vmn2r69|330581	-1	#DIV/0!	1	#DIV/0!
Olfr455|546896	-1	#DIV/0!	1	#DIV/0!

Nlrp1c-ps|627984	-1	#DIV/0!	1	#DIV/0!
Olfr577|259113	-1	#DIV/0!	1	#DIV/0!
Pex5l|58869	-1	#DIV/0!	1	#DIV/0!
Tcerg1l|70571	-1	#DIV/0!	1	#DIV/0!
Mir2137|100316779	-1	#DIV/0!	1	#DIV/0!
Cldn11|18417	-1	#DIV/0!	1	#DIV/0!
Cpne4|74020	-1	#DIV/0!	1	#DIV/0!
Gm4133|100042964	-1	#DIV/0!	1	#DIV/0!
Mir215|387211	-1	#DIV/0!	1	#DIV/0!
Cacna2d4|319734	-1	#DIV/0!	1	#DIV/0!
Csn1s2a|12993	-1	#DIV/0!	1	#DIV/0!
Lgals7|16858	-1	#DIV/0!	1	#DIV/0!
Gm5142|380907	-1	#DIV/0!	1	#DIV/0!
Olfr824|258669	-1	#DIV/0!	1	#DIV/0!
41164|71089	-1	#DIV/0!	1	#DIV/0!
Olfr698|258595	-1	#DIV/0!	1	#DIV/0!
Olfr1052|259012	-1	#DIV/0!	1	#DIV/0!
Gm20516|100126768	-1	#DIV/0!	1	#DIV/0!
Vmn2r24|243628	-1	#DIV/0!	1	#DIV/0!
2810408A11Rik|70419	-1	#DIV/0!	1	#DIV/0!
Try10|436522	-1	#DIV/0!	1	#DIV/0!
BC048562|434439	-1	#DIV/0!	1	#DIV/0!
Olfr138|170648	-1	#DIV/0!	1	#DIV/0!
Olfr654|258377	-1	#DIV/0!	1	#DIV/0!
Npas3|27386	-1	#DIV/0!	1	#DIV/0!
Tmprss13|214531	-1	#DIV/0!	1	#DIV/0!
Gml|625599	-1	#DIV/0!	1	#DIV/0!
Olfr1120|259031	-1	#DIV/0!	1	#DIV/0!
Bpifa1|18843	-1	#DIV/0!	1	#DIV/0!
Ccdc42b|546886	-1	#DIV/0!	1	#DIV/0!
Wfdc10|629756	-1	#DIV/0!	1	#DIV/0!
Tas2r114|387346	-1	#DIV/0!	1	#DIV/0!
Olfr1138|258632	-1	#DIV/0!	1	#DIV/0!
Brsk2|75770	-1	#DIV/0!	1	#DIV/0!
Fam194a|545527	-1	#DIV/0!	1	#DIV/0!
Ina|226180	-1	#DIV/0!	1	#DIV/0!
Shisa3|330096	-1	#DIV/0!	1	#DIV/0!
Ucp1|22227	-1	#DIV/0!	1	#DIV/0!
Defb7|246080	-1	#DIV/0!	1	#DIV/0!
Snora44|100217418	-1	#DIV/0!	1	#DIV/0!
Snord1c|100216536	-1	#DIV/0!	1	#DIV/0!
Moxd1|59012	-1	#DIV/0!	1	#DIV/0!
Olfr804|258068	-1	#DIV/0!	1	#DIV/0!
Gm15386|654464	-1	#DIV/0!	1	#DIV/0!
T2|21331	-1	#DIV/0!	1	#DIV/0!
Snord96a|100216534	-1	#DIV/0!	1	#DIV/0!
Epo|13856	-1	#DIV/0!	1	#DIV/0!
Snph|241727	-1	#DIV/0!	1	#DIV/0!
Klra18|93970	-1	#DIV/0!	1	#DIV/0!
Fam150b|100294583	-1	#DIV/0!	1	#DIV/0!
Cyp2j7-ps|546837	-1	#DIV/0!	1	#DIV/0!
Rorb|225998	-1	#DIV/0!	1	#DIV/0!
Il1rapl2|60367	-1	#DIV/0!	1	#DIV/0!

Il20ra|237313	-1	#DIV/0!	1	#DIV/0!
Ermn|77767	-1	#DIV/0!	1	#DIV/0!
H2-M10.1|14985	-1	#DIV/0!	1	#DIV/0!
Gm10104|100041688	-1	#DIV/0!	1	#DIV/0!
Prm3|19120	-1	#DIV/0!	1	#DIV/0!
Klk6|19144	-1	#DIV/0!	1	#DIV/0!
Il25|140806	-1	#DIV/0!	1	#DIV/0!
Gm10512|100038394	-1	#DIV/0!	1	#DIV/0!
Col6a4|68553	-1	#DIV/0!	1	#DIV/0!
Cldn9|56863	-1	#DIV/0!	1	#DIV/0!
Pnma5|385377	-1	#DIV/0!	1	#DIV/0!
Grxcr1|433899	-1	#DIV/0!	1	#DIV/0!
Mir708|735284	-1	#DIV/0!	1	#DIV/0!
Pou5f2|75507	-1	#DIV/0!	1	#DIV/0!
Olfr734|258658	-1	#DIV/0!	1	#DIV/0!
Vmn1r19|171200	-1	#DIV/0!	1	#DIV/0!
Ypel4|241525	-1	#DIV/0!	1	#DIV/0!
Ambn|11698	-1	#DIV/0!	1	#DIV/0!
C87977|97187	-1	#DIV/0!	1	#DIV/0!
Mybpc1|109272	-1	#DIV/0!	1	#DIV/0!
Vmn1r116|667268	-1	#DIV/0!	1	#DIV/0!
Mir760|791077	-1	#DIV/0!	1	#DIV/0!
Gabrp|216643	-1	#DIV/0!	1	#DIV/0!
Olfr332|257932	-1	#DIV/0!	1	#DIV/0!
Gm614|245536	-1	#DIV/0!	1	#DIV/0!
Mir471|723939	-1	#DIV/0!	1	#DIV/0!
Sp8|320145	-1	#DIV/0!	1	#DIV/0!
Olfr1261|258466	-1	#DIV/0!	1	#DIV/0!
Gpr139|209776	-1	#DIV/0!	1	#DIV/0!
Bpifb9b|433492	-1	#DIV/0!	1	#DIV/0!
Glra4|14657	-1	#DIV/0!	1	#DIV/0!
Cd163l1|244233	-1	#DIV/0!	1	#DIV/0!
Gm20581|100126779	-1	#DIV/0!	1	#DIV/0!
Olfr612|545985	-1	#DIV/0!	1	#DIV/0!
Vmn1r224|665525	-1	#DIV/0!	1	#DIV/0!
Olfr1243|258971	-1	#DIV/0!	1	#DIV/0!
Vmn1r8|171205	-1	#DIV/0!	1	#DIV/0!
Irx6|64379	-1	#DIV/0!	1	#DIV/0!
Glyatl3|435528	-1	#DIV/0!	1	#DIV/0!
Mir468|723871	-1	#DIV/0!	1	#DIV/0!
Mir200c|723944	-1	#DIV/0!	1	#DIV/0!
Scarna17|100217466	-1	#DIV/0!	1	#DIV/0!
Olfr1183|258522	-1	#DIV/0!	1	#DIV/0!
5330434G04Rik|100043213	-1	#DIV/0!	1	#DIV/0!
Prss28|114661	-1	#DIV/0!	1	#DIV/0!
1700108J01Rik|68245	-1	#DIV/0!	1	#DIV/0!
1700018B08Rik|76405	-1	#DIV/0!	1	#DIV/0!
Kcnh1|16510	-1	#DIV/0!	1	#DIV/0!
Olfr319|258493	-1	#DIV/0!	1	#DIV/0!
Olfr1219|258901	-1	#DIV/0!	1	#DIV/0!
Olfr242|406175	-1	#DIV/0!	1	#DIV/0!
Actrt2|73353	-1	#DIV/0!	1	#DIV/0!
Olfr859|258519	-1	#DIV/0!	1	#DIV/0!

Mir693|751553	-1	#DIV/0!	1	#DIV/0!
Emx1|13796	-1	#DIV/0!	1	#DIV/0!
A330021E22Rik|207686	-1	#DIV/0!	1	#DIV/0!
Rhd|19746	-1	#DIV/0!	1	#DIV/0!
Cdh15|12555	-1	#DIV/0!	1	#DIV/0!
Vmn1r188|252912	-1	#DIV/0!	1	#DIV/0!
Olfr482|258728	-1	#DIV/0!	1	#DIV/0!
B3gnt5|108105	-1	#DIV/0!	1	#DIV/0!
Slc6a3|13162	-1	#DIV/0!	1	#DIV/0!
Gm10649|100039211	-1	#DIV/0!	1	#DIV/0!
Kcnk15|241769	-1	#DIV/0!	1	#DIV/0!
Slc39a12|277468	-1	#DIV/0!	1	#DIV/0!
Ccdc63|330188	-1	#DIV/0!	1	#DIV/0!
Olfr1312|258359	-1	#DIV/0!	1	#DIV/0!
C2cd4a|244911	-1	#DIV/0!	1	#DIV/0!
Olfr691|259063	-1	#DIV/0!	1	#DIV/0!
Sprr2a1|20755	-1	#DIV/0!	1	#DIV/0!
Defa17|23855	-1	#DIV/0!	1	#DIV/0!
Gnrhr|14715	-1	#DIV/0!	1	#DIV/0!
4930447C04Rik|75801	-1	#DIV/0!	1	#DIV/0!
Krt74|406222	-1	#DIV/0!	1	#DIV/0!
Awat1|245533	-1	#DIV/0!	1	#DIV/0!
LOC100302567|100302567	-1	#DIV/0!	1	#DIV/0!
Mir486|723876	-1	#DIV/0!	1	#DIV/0!
Mir103-2|723825	-1	#DIV/0!	1	#DIV/0!
3110099E03Rik|100043232	-1	#DIV/0!	1	#DIV/0!
Mir1905|100316808	-1	#DIV/0!	1	#DIV/0!
Mir466i|100316665	-1	#DIV/0!	1	#DIV/0!
Olfr186|258318	-1	#DIV/0!	1	#DIV/0!
Gm13547|433416	-1	#DIV/0!	1	#DIV/0!
Olfr1387|258465	-1	#DIV/0!	1	#DIV/0!
Cldn22|75677	-1	#DIV/0!	1	#DIV/0!
Hist1h2bf|319180	-1	#DIV/0!	1	#DIV/0!
Fbxw24|382106	-1	#DIV/0!	1	#DIV/0!
Mir493|100124466	-1	#DIV/0!	1	#DIV/0!
Cer1|12622	-1	#DIV/0!	1	#DIV/0!
Olfr202|258997	-1	#DIV/0!	1	#DIV/0!
Avpr2|12000	-1	#DIV/0!	1	#DIV/0!
Slc6a14|56774	-1	#DIV/0!	1	#DIV/0!
Itgb6|16420	-1	#DIV/0!	1	#DIV/0!
Ret|19713	-1	#DIV/0!	1	#DIV/0!
Krtap8-1|16703	-1	#DIV/0!	1	#DIV/0!
Gm10510|100038481	-1	#DIV/0!	1	#DIV/0!
Magee2|272790	-1	#DIV/0!	1	#DIV/0!
Calhm1|546729	-1	#DIV/0!	1	#DIV/0!
Kel|23925	-1	#DIV/0!	1	#DIV/0!
Lelp1|69332	-1	#DIV/0!	1	#DIV/0!
Olfr1471|258231	-1	#DIV/0!	1	#DIV/0!
Skint9|329918	-1	#DIV/0!	1	#DIV/0!
Olfr867|257898	-1	#DIV/0!	1	#DIV/0!
Tnmd|64103	-1	#DIV/0!	1	#DIV/0!
Rspo2|239405	-1	#DIV/0!	1	#DIV/0!
Gm11541|432589	-1	#DIV/0!	1	#DIV/0!

Fam162b|77296	-1	#DIV/0!	1	#DIV/0!
Mir301b|791069	-1	#DIV/0!	1	#DIV/0!
Gm14345|630022	-1	#DIV/0!	1	#DIV/0!
Fam190a|232035	-1	#DIV/0!	1	#DIV/0!
Stfa1|20861	-1	#DIV/0!	1	#DIV/0!
Xlr5c|27084	-1	#DIV/0!	1	#DIV/0!
4930595M18Rik|245492	-1	#DIV/0!	1	#DIV/0!
Olfr894|258868	-1	#DIV/0!	1	#DIV/0!
Trhr2|170732	-1	#DIV/0!	1	#DIV/0!
Baiap3|545192	-1	#DIV/0!	1	#DIV/0!
Snora3|100302499	-1	#DIV/0!	1	#DIV/0!
Slc7a12|140918	-1	#DIV/0!	1	#DIV/0!
Drd1a|13488	-1	#DIV/0!	1	#DIV/0!
Plac8l1|69401	-1	#DIV/0!	1	#DIV/0!
Olfr17|18314	-1	#DIV/0!	1	#DIV/0!
Vmn2r-ps60|628490	-1	#DIV/0!	1	#DIV/0!
Snora65|104367	-1	#DIV/0!	1	#DIV/0!
Myt1|17932	-1	#DIV/0!	1	#DIV/0!
Tyr|22173	-1	#DIV/0!	1	#DIV/0!
Cplx3|235415	-1	#DIV/0!	1	#DIV/0!
Vmn1r238|100312476	-1	#DIV/0!	1	#DIV/0!
Rag2|19374	-1	#DIV/0!	1	#DIV/0!
Ppp1r2-ps9|67395	-1	#DIV/0!	1	#DIV/0!
Gast|14459	-1	#DIV/0!	1	#DIV/0!
Olfr1214|258899	-1	#DIV/0!	1	#DIV/0!
Grin2c|14813	-1	#DIV/0!	1	#DIV/0!
Cpa1|109697	-1	#DIV/0!	1	#DIV/0!
Taar6|215855	-1	#DIV/0!	1	#DIV/0!
Gm4981|245263	-1	#DIV/0!	1	#DIV/0!
Vmn1r55|384522	-1	#DIV/0!	1	#DIV/0!
Olfr272|258836	-1	#DIV/0!	1	#DIV/0!
Olfr1116-ps|257875	-1	#DIV/0!	1	#DIV/0!
Olfr368|258371	-1	#DIV/0!	1	#DIV/0!
Olfr105-ps|257893	-1	#DIV/0!	1	#DIV/0!
40972|381270	-1	#DIV/0!	1	#DIV/0!
Capza3|12344	-1	#DIV/0!	1	#DIV/0!
Olfr453|258016	-1	#DIV/0!	1	#DIV/0!
Ttc29|73301	-1	#DIV/0!	1	#DIV/0!
Srrm3|58212	-1	#DIV/0!	1	#DIV/0!
Gm3696|100042149	-1	#DIV/0!	1	#DIV/0!
Mir464|723943	-1	#DIV/0!	1	#DIV/0!
Olfr128|383243	-1	#DIV/0!	1	#DIV/0!
1700022A21Rik|72252	-1	#DIV/0!	1	#DIV/0!
Olfr777|258537	-1	#DIV/0!	1	#DIV/0!
Gm1332|383766	-1	#DIV/0!	1	#DIV/0!
1700003M02Rik|69329	-1	#DIV/0!	1	#DIV/0!
Gsdma2|76758	-1	#DIV/0!	1	#DIV/0!
Olfr1051|404324	-1	#DIV/0!	1	#DIV/0!
Mog|17441	-1	#DIV/0!	1	#DIV/0!
Nnat|18111	-1	#DIV/0!	1	#DIV/0!
Olfr843|258560	-1	#DIV/0!	1	#DIV/0!
Fa2h|338521	-1	#DIV/0!	1	#DIV/0!
Lyzl1|67328	-1	#DIV/0!	1	#DIV/0!

Scarna3b|100217447	-1	#DIV/0!	1	#DIV/0!
Hbb-y|15135	-1	#DIV/0!	1	#DIV/0!
2410004A20Rik|66991	-1	#DIV/0!	1	#DIV/0!
Olfr1170|258525	-1	#DIV/0!	1	#DIV/0!
Vmn1r37|171183	-1	#DIV/0!	1	#DIV/0!
Tssk5|73542	-1	#DIV/0!	1	#DIV/0!
Rp1|19888	-1	#DIV/0!	1	#DIV/0!
Vmn2r91|665210	-1	#DIV/0!	1	#DIV/0!
Myl10|59310	-1	#DIV/0!	1	#DIV/0!
Vmn1r56|81015	-1	#DIV/0!	1	#DIV/0!
Alppl2|11650	-1	#DIV/0!	1	#DIV/0!
Olfr275|258857	-1	#DIV/0!	1	#DIV/0!
Sstr1|20605	-1	#DIV/0!	1	#DIV/0!
Lrrc69|73314	-1	#DIV/0!	1	#DIV/0!
Olfr1248|258787	-1	#DIV/0!	1	#DIV/0!
Csn3|12994	-1	#DIV/0!	1	#DIV/0!
Galnt13|271786	-1	#DIV/0!	1	#DIV/0!
4930578C19Rik|75905	-1	#DIV/0!	1	#DIV/0!
Matn1|17180	-1	#DIV/0!	1	#DIV/0!
Hist2h2bb|319189	-1	#DIV/0!	1	#DIV/0!
Gpr33|14762	-1	#DIV/0!	1	#DIV/0!
Tdpoz2|399673	-1	#DIV/0!	1	#DIV/0!
Klra19|93971	-1	#DIV/0!	1	#DIV/0!
Nog|18121	-1	#DIV/0!	1	#DIV/0!
Olfr605|258156	-1	#DIV/0!	1	#DIV/0!
2610100L16Rik|70441	-1	#DIV/0!	1	#DIV/0!
Olfr1311|258271	-1	#DIV/0!	1	#DIV/0!
Mixl1|27217	-1	#DIV/0!	1	#DIV/0!
1700093K21Rik|67358	-1	#DIV/0!	1	#DIV/0!
Nkx3-2|12020	-1	#DIV/0!	1	#DIV/0!
Olfr985|258854	-1	#DIV/0!	1	#DIV/0!
Olfr108|258457	-1	#DIV/0!	1	#DIV/0!
Gm1110|382064	-1	#DIV/0!	1	#DIV/0!
Olfr310|258222	-1	#DIV/0!	1	#DIV/0!
Zfp600|667666	-1	#DIV/0!	1	#DIV/0!
Gm11565|670550	-1	#DIV/0!	1	#DIV/0!
Gm5634|434726	-1	#DIV/0!	1	#DIV/0!
Zcchc12|72693	-1	#DIV/0!	1	#DIV/0!
Madcam1|17123	-1	#DIV/0!	1	#DIV/0!
Olfr1484|258288	-1	#DIV/0!	1	#DIV/0!
Spaca3|75622	-1	#DIV/0!	1	#DIV/0!
Krt71|56735	-1	#DIV/0!	1	#DIV/0!
Sval2|84543	-1	#DIV/0!	1	#DIV/0!
Pnck|93843	-1	#DIV/0!	1	#DIV/0!
1700001F09Rik|71826	-1	#DIV/0!	1	#DIV/0!
Pcdhac2|353237	-1	#DIV/0!	1	#DIV/0!
Olfr301|257958	-1	#DIV/0!	1	#DIV/0!
Lipk|240633	-1	#DIV/0!	1	#DIV/0!
H2-Ea-ps|14968	-1	#DIV/0!	1	#DIV/0!
Ssxb8|631002	-1	#DIV/0!	1	#DIV/0!
4930590J08Rik|381798	-1	#DIV/0!	1	#DIV/0!
Plcd4|18802	-1	#DIV/0!	1	#DIV/0!
4930468A15Rik|74987	-1	#DIV/0!	1	#DIV/0!

Cylc1|67407	-1	#DIV/0!	1	#DIV/0!
Tnfrsf8|21941	-1	#DIV/0!	1	#DIV/0!
Mir93|723885	-1	#DIV/0!	1	#DIV/0!
Vmn1r199|171247	-1	#DIV/0!	1	#DIV/0!
Banf2|403171	-1	#DIV/0!	1	#DIV/0!
Ky|16716	-1	#DIV/0!	1	#DIV/0!
4933413G19Rik|71149	-1	#DIV/0!	1	#DIV/0!
Mir188|387183	-1	#DIV/0!	1	#DIV/0!
Olfr1223|258894	-1	#DIV/0!	1	#DIV/0!
Olfr1254|258468	-1	#DIV/0!	1	#DIV/0!
Dgki|320127	-1	#DIV/0!	1	#DIV/0!
Ap3b2|11775	-1	#DIV/0!	1	#DIV/0!
Gadl1|73748	-1	#DIV/0!	1	#DIV/0!
Adam29|244486	-1	#DIV/0!	1	#DIV/0!
Pou4f1|18996	-1	#DIV/0!	1	#DIV/0!
Defb39|360214	-1	#DIV/0!	1	#DIV/0!
Olfr1019|259017	-1	#DIV/0!	1	#DIV/0!
H1foo|171506	-1	#DIV/0!	1	#DIV/0!
Asb18|208372	-1	#DIV/0!	1	#DIV/0!
Olfr1404|258881	-1	#DIV/0!	1	#DIV/0!
Olfr1457|258568	-1	#DIV/0!	1	#DIV/0!
Rbmy1a1|19657	-1	#DIV/0!	1	#DIV/0!
Vmn1r180|232962	-1	#DIV/0!	1	#DIV/0!
Htr3a|15561	-1	#DIV/0!	1	#DIV/0!
Olfr960|258276	-1	#DIV/0!	1	#DIV/0!
Olfr536|258513	-1	#DIV/0!	1	#DIV/0!
Pebp4|73523	-1	#DIV/0!	1	#DIV/0!
Bnc1|12173	-1	#DIV/0!	1	#DIV/0!
Spdya|70891	-1	#DIV/0!	1	#DIV/0!
4930511I11Rik|67645	-1	#DIV/0!	1	#DIV/0!
Helt|234219	-1	#DIV/0!	1	#DIV/0!
Olfr1226|258969	-1	#DIV/0!	1	#DIV/0!
Enam|13801	-1	#DIV/0!	1	#DIV/0!
Kcna1|16485	-1	#DIV/0!	1	#DIV/0!
Art2b|11872	-1	#DIV/0!	1	#DIV/0!
Ifna9|15972	-1	#DIV/0!	1	#DIV/0!
Mir669h|100316831	-1	#DIV/0!	1	#DIV/0!
Ccl1|20290	-1	#DIV/0!	1	#DIV/0!
Gm8773|667705	-1	#DIV/0!	1	#DIV/0!
Snora2b|100217416	-1	#DIV/0!	1	#DIV/0!
Ephb1|270190	-1	#DIV/0!	1	#DIV/0!
Mir218-2|723924	-1	#DIV/0!	1	#DIV/0!
Olfr866|258551	-1	#DIV/0!	1	#DIV/0!
Mir382|723912	-1	#DIV/0!	1	#DIV/0!
Mir450b|751532	-1	#DIV/0!	1	#DIV/0!
Mir92b|100124470	-1	#DIV/0!	1	#DIV/0!
Gm5127|331493	-1	#DIV/0!	1	#DIV/0!
Npffr1|237362	-1	#DIV/0!	1	#DIV/0!
4930503B20Rik|75015	-1	#DIV/0!	1	#DIV/0!
Olfr1133|258348	-1	#DIV/0!	1	#DIV/0!
Vmn1r67|171263	-1	#DIV/0!	1	#DIV/0!
Rdh8|235033	-1	#DIV/0!	1	#DIV/0!
Cntnap5a|636808	-1	#DIV/0!	1	#DIV/0!

Olfr1077-ps1|625853	-1	#DIV/0!	1	#DIV/0!
Olfr827|258297	-1	#DIV/0!	1	#DIV/0!
Olfr224|258198	-1	#DIV/0!	1	#DIV/0!
Btbd17|72014	-1	#DIV/0!	1	#DIV/0!
4930431A04Rik|100043861	-1	#DIV/0!	1	#DIV/0!
Olfr1448|258696	-1	#DIV/0!	1	#DIV/0!
Gm5169|382277	-1	#DIV/0!	1	#DIV/0!
4930544D05Rik|668433	-1	#DIV/0!	1	#DIV/0!
Nkx6-1|18096	-1	#DIV/0!	1	#DIV/0!
Hba-x|15126	-1	#DIV/0!	1	#DIV/0!
Grin3a|242443	-1	#DIV/0!	1	#DIV/0!
Nrip3|78593	-1	#DIV/0!	1	#DIV/0!
Muc15|269328	-1	#DIV/0!	1	#DIV/0!
Vmn1r89|171260	-1	#DIV/0!	1	#DIV/0!
Nr4a3|18124	-1	#DIV/0!	1	#DIV/0!
1700108F19Rik|73272	-1	#DIV/0!	1	#DIV/0!
Amac1|56293	-1	#DIV/0!	1	#DIV/0!
Gm14744|628923	-1	#DIV/0!	1	#DIV/0!
Crisp2|22024	-1	#DIV/0!	1	#DIV/0!
Oxgr1|239283	-1	#DIV/0!	1	#DIV/0!
Scgn|214189	-1	#DIV/0!	1	#DIV/0!
Rit2|19762	-1	#DIV/0!	1	#DIV/0!
Gm6040|574083	-1	#DIV/0!	1	#DIV/0!
Prg3|53856	-1	#DIV/0!	1	#DIV/0!
Tsga8|100502723	-1	#DIV/0!	1	#DIV/0!
Dhrs2|71412	-1	#DIV/0!	1	#DIV/0!
Ctsr|56835	-1	#DIV/0!	1	#DIV/0!
Zfp382|233060	-1	#DIV/0!	1	#DIV/0!
Bglap-rs1|12095	-1	#DIV/0!	1	#DIV/0!
Olfr1165-ps|258642	-1	#DIV/0!	1	#DIV/0!
Hes2|15206	-1	#DIV/0!	1	#DIV/0!
Mir146|387164	-1	#DIV/0!	1	#DIV/0!
Olfr63|258939	-1	#DIV/0!	1	#DIV/0!
Olfr876|258883	-1	#DIV/0!	1	#DIV/0!
Lmo1|109594	-1	#DIV/0!	1	#DIV/0!
Il2|16183	-1	#DIV/0!	1	#DIV/0!
Mir302d|723928	-1	#DIV/0!	1	#DIV/0!
Gm4302|100043227	-1	#DIV/0!	1	#DIV/0!
Olfr155|29845	-1	#DIV/0!	1	#DIV/0!
Slc2a7|435818	-1	#DIV/0!	1	#DIV/0!
Olfr67|18368	-1	#DIV/0!	1	#DIV/0!
Cdhr1|170677	-1	#DIV/0!	1	#DIV/0!
Olfr325|258261	-1	#DIV/0!	1	#DIV/0!
Msx1as|93895	-1	#DIV/0!	1	#DIV/0!
Gm6614|625716	-1	#DIV/0!	1	#DIV/0!
Ctag2|70062	-1	#DIV/0!	1	#DIV/0!
Tpbpb|116913	-1	#DIV/0!	1	#DIV/0!
Defb14|244332	-1	#DIV/0!	1	#DIV/0!
Olfr27|258826	-1	#DIV/0!	1	#DIV/0!
Vstm2b|58188	-1	#DIV/0!	1	#DIV/0!
Gjd4|225152	-1	#DIV/0!	1	#DIV/0!
Speer4f|70935	-1	#DIV/0!	1	#DIV/0!
Efcab5|319634	-1	#DIV/0!	1	#DIV/0!

Mir1936|100316812	-1	#DIV/0!	1	#DIV/0!
Ppp3r2|19059	-1	#DIV/0!	1	#DIV/0!
Olfr1242|258970	-1	#DIV/0!	1	#DIV/0!
Olfr532|259028	-1	#DIV/0!	1	#DIV/0!
1700011I03Rik|75444	-1	#DIV/0!	1	#DIV/0!
1700010M22Rik|66328	-1	#DIV/0!	1	#DIV/0!
Cyp2c53-ps|638988	-1	#DIV/0!	1	#DIV/0!
Zdhhc25|70073	-1	#DIV/0!	1	#DIV/0!
Prdm12|381359	-1	#DIV/0!	1	#DIV/0!
Olfr898|258871	-1	#DIV/0!	1	#DIV/0!
Them5|66198	-1	#DIV/0!	1	#DIV/0!
Defa22|382059	-1	#DIV/0!	1	#DIV/0!
Wnt10a|22409	-1	#DIV/0!	1	#DIV/0!
Krtap15|26560	-1	#DIV/0!	1	#DIV/0!
Olfr68|18369	-1	#DIV/0!	1	#DIV/0!
Vmn2r84|625068	-1	#DIV/0!	1	#DIV/0!
Avp|11998	-1	#DIV/0!	1	#DIV/0!
Atp13a5|268878	-1	#DIV/0!	1	#DIV/0!
Acsl6|216739	-1	#DIV/0!	1	#DIV/0!
Olfr1445|258694	-1	#DIV/0!	1	#DIV/0!
Krtap27-1|239933	-1	#DIV/0!	1	#DIV/0!
Mir1192|100316672	-1	#DIV/0!	1	#DIV/0!
Stk31|77485	-1	#DIV/0!	1	#DIV/0!
Lhx9|16876	-1	#DIV/0!	1	#DIV/0!
Gm11564|670496	-1	#DIV/0!	1	#DIV/0!
4930429F11Rik|74622	-1	#DIV/0!	1	#DIV/0!
Olfr869|258550	-1	#DIV/0!	1	#DIV/0!
Cpa5|74649	-1	#DIV/0!	1	#DIV/0!
Odz1|23963	-1	#DIV/0!	1	#DIV/0!
Tex22|75671	-1	#DIV/0!	1	#DIV/0!
Adam34|252866	-1	#DIV/0!	1	#DIV/0!
9530003J23Rik|77397	-1	#DIV/0!	1	#DIV/0!
Cuzd1|16433	-1	#DIV/0!	1	#DIV/0!
4930562C15Rik|78809	-1	#DIV/0!	1	#DIV/0!
Asb12|70392	-1	#DIV/0!	1	#DIV/0!
7530420F21Rik|320019	-1	#DIV/0!	1	#DIV/0!
Kcnj14|211480	-1	#DIV/0!	1	#DIV/0!
Tpbg|21983	-1	#DIV/0!	1	#DIV/0!
Olfr689|258745	-1	#DIV/0!	1	#DIV/0!
Scrg1|20284	-1	#DIV/0!	1	#DIV/0!
Scin|20259	-1	#DIV/0!	1	#DIV/0!
Olfr486|258489	-1	#DIV/0!	1	#DIV/0!
Rnu73b|19871	-1	#DIV/0!	1	#DIV/0!
Olfr555|259107	-1	#DIV/0!	1	#DIV/0!
Ptchd2|242748	-1	#DIV/0!	1	#DIV/0!
Fam159b|77803	-1	#DIV/0!	1	#DIV/0!
Olfr347|258945	-1	#DIV/0!	1	#DIV/0!
Olfr740|258661	-1	#DIV/0!	1	#DIV/0!
Gm13276|545649	-1	#DIV/0!	1	#DIV/0!
Olfr392|259008	-1	#DIV/0!	1	#DIV/0!
Mir370|723854	-1	#DIV/0!	1	#DIV/0!
Dusp9|75590	-1	#DIV/0!	1	#DIV/0!
Olfr286|629524	-1	#DIV/0!	1	#DIV/0!

4921511M17Rik|667256	-1	#DIV/0!	1	#DIV/0!
Olfr1046|258575	-1	#DIV/0!	1	#DIV/0!
Smr3a|20599	-1	#DIV/0!	1	#DIV/0!
Foxo6|329934	-1	#DIV/0!	1	#DIV/0!
Olfr775|258538	-1	#DIV/0!	1	#DIV/0!
Dmrt1|50796	-1	#DIV/0!	1	#DIV/0!
Dnahc6|330355	-1	#DIV/0!	1	#DIV/0!
Mir99b|387230	-1	#DIV/0!	1	#DIV/0!
6430704M03Rik|230235	-1	#DIV/0!	1	#DIV/0!
Calb2|12308	-1	#DIV/0!	1	#DIV/0!
Mir1904|100316683	-1	#DIV/0!	1	#DIV/0!
A630095E13Rik|235973	-1	#DIV/0!	1	#DIV/0!
Triml1|244448	-1	#DIV/0!	1	#DIV/0!
Sytl5|236643	-1	#DIV/0!	1	#DIV/0!
Nctc1|330677	-1	#DIV/0!	1	#DIV/0!
Vmn1r27|171206	-1	#DIV/0!	1	#DIV/0!
Olfr834|258074	-1	#DIV/0!	1	#DIV/0!
Gm949|381142	-1	#DIV/0!	1	#DIV/0!
Mir877|100124459	-1	#DIV/0!	1	#DIV/0!
Tmem27|57394	-1	#DIV/0!	1	#DIV/0!
Vmn1r95|100312475	-1	#DIV/0!	1	#DIV/0!
Htr7|15566	-1	#DIV/0!	1	#DIV/0!
Cela3b|67868	-1	#DIV/0!	1	#DIV/0!
Cdhr3|68764	-1	#DIV/0!	1	#DIV/0!
Hist1h2bn|319187	-1	#DIV/0!	1	#DIV/0!
1700001J11Rik|72224	-1	#DIV/0!	1	#DIV/0!
Olfr519|277935	-1	#DIV/0!	1	#DIV/0!
Tmco5b|75275	-1	#DIV/0!	1	#DIV/0!
Mir3074-2|100526528	-1	#DIV/0!	1	#DIV/0!
Ptf1a|19213	-1	#DIV/0!	1	#DIV/0!
Samd12|320679	-1	#DIV/0!	1	#DIV/0!
Vmn2r86|625109	-1	#DIV/0!	1	#DIV/0!
Olfr1010|258255	-1	#DIV/0!	1	#DIV/0!
Gm14851|634825	-1	#DIV/0!	1	#DIV/0!
Snora16a|100310813	-1	#DIV/0!	1	#DIV/0!
Defb37|353320	-1	#DIV/0!	1	#DIV/0!
4930528A17Rik|67735	-1	#DIV/0!	1	#DIV/0!
Lypd4|232973	-1	#DIV/0!	1	#DIV/0!
9030224M15Rik|327747	-1	#DIV/0!	1	#DIV/0!
Serpinb9d|20726	-1	#DIV/0!	1	#DIV/0!
Olfr1049|259018	-1	#DIV/0!	1	#DIV/0!
Uts2d|224065	-1	#DIV/0!	1	#DIV/0!
Ssxb10|385312	-1	#DIV/0!	1	#DIV/0!
Krt36|16673	-1	#DIV/0!	1	#DIV/0!
Chrm5|213788	-1	#DIV/0!	1	#DIV/0!
Dnajb3|15504	-1	#DIV/0!	1	#DIV/0!
Mageb1|17145	-1	#DIV/0!	1	#DIV/0!
Bpifa2e|19194	-1	#DIV/0!	1	#DIV/0!
Tdrd6|210510	-1	#DIV/0!	1	#DIV/0!
Klk1b9|13648	-1	#DIV/0!	1	#DIV/0!
Opn1sw|12057	-1	#DIV/0!	1	#DIV/0!
Prss45|260408	-1	#DIV/0!	1	#DIV/0!
Igf2as|111975	-1	#DIV/0!	1	#DIV/0!

Krt33b|16671	-1	#DIV/0!	1	#DIV/0!
Tmem179|104885	-1	#DIV/0!	1	#DIV/0!
Olfr107|258504	-1	#DIV/0!	1	#DIV/0!
Olfr13|18310	-1	#DIV/0!	1	#DIV/0!
Gm14346|668958	-1	#DIV/0!	1	#DIV/0!
Edn3|13616	-1	#DIV/0!	1	#DIV/0!
Vmn2r36|100042653	-1	#DIV/0!	1	#DIV/0!
Olfr986|258608	-1	#DIV/0!	1	#DIV/0!
Mmp16|17389	-1	#DIV/0!	1	#DIV/0!
Mir145|387163	-1	#DIV/0!	1	#DIV/0!
Mir709|735271	-1	#DIV/0!	1	#DIV/0!
Dkk4|234130	-1	#DIV/0!	1	#DIV/0!
Spink13|100038417	-1	#DIV/0!	1	#DIV/0!
Msmb|17695	-1	#DIV/0!	1	#DIV/0!
Slc5a4a|64452	-1	#DIV/0!	1	#DIV/0!
Asphd1|233879	-1	#DIV/0!	1	#DIV/0!
Vmn1r189|252906	-1	#DIV/0!	1	#DIV/0!
Kcne1|16509	-1	#DIV/0!	1	#DIV/0!
Mir3061|100526467	-1	#DIV/0!	1	#DIV/0!
Olfr1360|258536	-1	#DIV/0!	1	#DIV/0!
Olfr1085|258583	-1	#DIV/0!	1	#DIV/0!
Defa-rs7|13226	-1	#DIV/0!	1	#DIV/0!
1700007I08Rik|70362	-1	#DIV/0!	1	#DIV/0!
Vmn2r20|667180	-1	#DIV/0!	1	#DIV/0!
Lrrc6|54562	-1	#DIV/0!	1	#DIV/0!
Olfr437|258293	-1	#DIV/0!	1	#DIV/0!
Kctd4|67516	-1	#DIV/0!	1	#DIV/0!
Klhl34|245683	-1	#DIV/0!	1	#DIV/0!
Bpifb6|228796	-1	#DIV/0!	1	#DIV/0!
Alpi|76768	-1	#DIV/0!	1	#DIV/0!
Clgn|12745	-1	#DIV/0!	1	#DIV/0!
Gm10863|100041655	-1	#DIV/0!	1	#DIV/0!
Gm13278|545651	-1	#DIV/0!	1	#DIV/0!
Olfr1197|433449	-1	#DIV/0!	1	#DIV/0!
Gm10391|100039293	-1	#DIV/0!	1	#DIV/0!
Olfr456|259144	-1	#DIV/0!	1	#DIV/0!
Slfn3|20557	-1	#DIV/0!	1	#DIV/0!
Dbx2|223843	-1	#DIV/0!	1	#DIV/0!
Dach2|93837	-1	#DIV/0!	1	#DIV/0!
Obox2|246792	-1	#DIV/0!	1	#DIV/0!
4732456N10Rik|239673	-1	#DIV/0!	1	#DIV/0!
Vmn2r97|627367	-1	#DIV/0!	1	#DIV/0!
Foxn1|15218	-1	#DIV/0!	1	#DIV/0!
Epyc|13516	-1	#DIV/0!	1	#DIV/0!
Gdap1l1|228858	-1	#DIV/0!	1	#DIV/0!
Nlrp9c|330490	-1	#DIV/0!	1	#DIV/0!
Olfr801|258282	-1	#DIV/0!	1	#DIV/0!
Olfr585|259091	-1	#DIV/0!	1	#DIV/0!
Olfr1298|258888	-1	#DIV/0!	1	#DIV/0!
Ccr4|12773	-1	#DIV/0!	1	#DIV/0!
Sstr5|20609	-1	#DIV/0!	1	#DIV/0!
Olfr231|404222	-1	#DIV/0!	1	#DIV/0!
Mir297b|735281	-1	#DIV/0!	1	#DIV/0!

Vmn1r51|22296	-1	#DIV/0!	1	#DIV/0!
Fbxw14|50757	-1	#DIV/0!	1	#DIV/0!
Trpc4|22066	-1	#DIV/0!	1	#DIV/0!
Nrl|18185	-1	#DIV/0!	1	#DIV/0!
Olfr1086|258585	-1	#DIV/0!	1	#DIV/0!
Mir489|723877	-1	#DIV/0!	1	#DIV/0!
Aurkc|20871	-1	#DIV/0!	1	#DIV/0!
Ugt8a|22239	-1	#DIV/0!	1	#DIV/0!
Foxf2|14238	-1	#DIV/0!	1	#DIV/0!
Olfr1062|259082	-1	#DIV/0!	1	#DIV/0!
Fbxo16|50759	-1	#DIV/0!	1	#DIV/0!
Lingo2|242384	-1	#DIV/0!	1	#DIV/0!
Olfr857|257963	-1	#DIV/0!	1	#DIV/0!
Dclk1|13175	-1	#DIV/0!	1	#DIV/0!
Hrh3|99296	-1	#DIV/0!	1	#DIV/0!
Lancl3|236285	-1	#DIV/0!	1	#DIV/0!
Gm16287|100038595	-1	#DIV/0!	1	#DIV/0!
Lypd5|76942	-1	#DIV/0!	1	#DIV/0!
Esx1|13984	-1	#DIV/0!	1	#DIV/0!
Olfr907|258801	-1	#DIV/0!	1	#DIV/0!
A630089N07Rik|320586	-1	#DIV/0!	1	#DIV/0!
Mir450-2|723938	-1	#DIV/0!	1	#DIV/0!
Vmn1r226|171225	-1	#DIV/0!	1	#DIV/0!
Mir377|723857	-1	#DIV/0!	1	#DIV/0!
Ms4a2|14126	-1	#DIV/0!	1	#DIV/0!
4930563D23Rik|75328	-1	#DIV/0!	1	#DIV/0!
Olfr1058|258386	-1	#DIV/0!	1	#DIV/0!
Mir30a|387225	-1	#DIV/0!	1	#DIV/0!
Olfr554|258322	-1	#DIV/0!	1	#DIV/0!
Gm11202|790912	-1	#DIV/0!	1	#DIV/0!
Lypd6|320343	-1	#DIV/0!	1	#DIV/0!
Vmn2r46|100042894	-1	#DIV/0!	1	#DIV/0!
Mesp1|17292	-1	#DIV/0!	1	#DIV/0!
Olfr6|233670	-1	#DIV/0!	1	#DIV/0!
Olfr955|258242	-1	#DIV/0!	1	#DIV/0!
Cacna1e|12290	-1	#DIV/0!	1	#DIV/0!
Csf2|12981	-1	#DIV/0!	1	#DIV/0!
Sgcz|244431	-1	#DIV/0!	1	#DIV/0!
Mir335|723930	-1	#DIV/0!	1	#DIV/0!
Ddi1|71829	-1	#DIV/0!	1	#DIV/0!
Olfr395|259007	-1	#DIV/0!	1	#DIV/0!
Ttk|22137	-1	#DIV/0!	1	#DIV/0!
Olfr631|258961	-1	#DIV/0!	1	#DIV/0!
Vmn2r43|381838	-1	#DIV/0!	1	#DIV/0!
Hoxb9|15417	-1	#DIV/0!	1	#DIV/0!
Olfr329-ps|259148	-1	#DIV/0!	1	#DIV/0!
Nxf2|83454	-1	#DIV/0!	1	#DIV/0!
Pou1f1|18736	-1	#DIV/0!	1	#DIV/0!
Hoxa9|15405	-1	#DIV/0!	1	#DIV/0!
2610021K21Rik|78767	-1	#DIV/0!	1	#DIV/0!
Olfr290|258411	-1	#DIV/0!	1	#DIV/0!
Vmn2r50|434117	-1	#DIV/0!	1	#DIV/0!
Mir677|751518	-1	#DIV/0!	1	#DIV/0!

Pth|19226	-1	#DIV/0!	1	#DIV/0!
Cdh29|320169	-1	#DIV/0!	1	#DIV/0!
AU019990|767814	-1	#DIV/0!	1	#DIV/0!
Gtsf1|74174	-1	#DIV/0!	1	#DIV/0!
Cst11|78240	-1	#DIV/0!	1	#DIV/0!
BC048507|408058	-1	#DIV/0!	1	#DIV/0!
Mir483|723874	-1	#DIV/0!	1	#DIV/0!
Vmn1r120|435953	-1	#DIV/0!	1	#DIV/0!
Tas2r106|387341	-1	#DIV/0!	1	#DIV/0!
Ctrc|76701	-1	#DIV/0!	1	#DIV/0!
Wfdc11|629761	-1	#DIV/0!	1	#DIV/0!
Krt81|64818	-1	#DIV/0!	1	#DIV/0!
Gmcl1l|71847	-1	#DIV/0!	1	#DIV/0!
Mir449a|723868	-1	#DIV/0!	1	#DIV/0!
Gm17455|100302688	-1	#DIV/0!	1	#DIV/0!
Tph1|21990	-1	#DIV/0!	1	#DIV/0!
Gm15023|100040635	-1	#DIV/0!	1	#DIV/0!
Prss38|216797	-1	#DIV/0!	1	#DIV/0!
Rtp2|224055	-1	#DIV/0!	1	#DIV/0!
Olfr1462|258688	-1	#DIV/0!	1	#DIV/0!
Sval1|71578	-1	#DIV/0!	1	#DIV/0!
Defb21|403172	-1	#DIV/0!	1	#DIV/0!
Serpina1f|68348	-1	#DIV/0!	1	#DIV/0!
Col2a1|12824	-1	#DIV/0!	1	#DIV/0!
Spint3|629747	-1	#DIV/0!	1	#DIV/0!
Vmn1r10|113858	-1	#DIV/0!	1	#DIV/0!
Gm6084|619517	-1	#DIV/0!	1	#DIV/0!
Gprc6a|210198	-1	#DIV/0!	1	#DIV/0!
BC037032|414066	-1	#DIV/0!	1	#DIV/0!
Gm6300|622229	-1	#DIV/0!	1	#DIV/0!
Olfr1408|258759	-1	#DIV/0!	1	#DIV/0!
Mrgprb2|243979	-1	#DIV/0!	1	#DIV/0!
Gm9513|671003	-1	#DIV/0!	1	#DIV/0!
Vmn2r100|627537	-1	#DIV/0!	1	#DIV/0!
Pirt|193003	-1	#DIV/0!	1	#DIV/0!
Kcnf1|382571	-1	#DIV/0!	1	#DIV/0!
Lypd2|68311	-1	#DIV/0!	1	#DIV/0!
Olfr506|258215	-1	#DIV/0!	1	#DIV/0!
Olfr739|258663	-1	#DIV/0!	1	#DIV/0!
Adam1b|280667	-1	#DIV/0!	1	#DIV/0!
Kcnh6|192775	-1	#DIV/0!	1	#DIV/0!
Tubb1|545486	-1	#DIV/0!	1	#DIV/0!
Gm5132|333452	-1	#DIV/0!	1	#DIV/0!
Cyp11b2|13072	-1	#DIV/0!	1	#DIV/0!
Speer7-ps1|75858	-1	#DIV/0!	1	#DIV/0!
Mir224|723894	-1	#DIV/0!	1	#DIV/0!
Gm732|213450	-1	#DIV/0!	1	#DIV/0!
Olfr906|258799	-1	#DIV/0!	1	#DIV/0!
Vmn1r165|100043667	-1	#DIV/0!	1	#DIV/0!
Olfr340|258953	-1	#DIV/0!	1	#DIV/0!
1700018F24Rik|69396	-1	#DIV/0!	1	#DIV/0!
P2rx1|18436	-1	#DIV/0!	1	#DIV/0!
Tmprss11bnl|319875	-1	#DIV/0!	1	#DIV/0!

1700034F02Rik|73324	-1	#DIV/0!	1	#DIV/0!
Rtkn2|170799	-1	#DIV/0!	1	#DIV/0!
Olfr584|259056	-1	#DIV/0!	1	#DIV/0!
V1ra8|113850	-1	#DIV/0!	1	#DIV/0!
Il12a|16159	-1	#DIV/0!	1	#DIV/0!
Pcdha5|12941	-1	#DIV/0!	1	#DIV/0!
Mir200a|387242	-1	#DIV/0!	1	#DIV/0!
Nipal4|214112	-1	#DIV/0!	1	#DIV/0!
Hoxd12|15432	-1	#DIV/0!	1	#DIV/0!
Fgf15|14170	-1	#DIV/0!	1	#DIV/0!
Gzmg|14944	-1	#DIV/0!	1	#DIV/0!
Tmem145|330485	-1	#DIV/0!	1	#DIV/0!
Gm14850|100041895	-1	#DIV/0!	1	#DIV/0!
Scgb3a1|68662	-1	#DIV/0!	1	#DIV/0!
Klhl14|225266	-1	#DIV/0!	1	#DIV/0!
Defb47|654465	-1	#DIV/0!	1	#DIV/0!
Vmn1r63|81017	-1	#DIV/0!	1	#DIV/0!
Gm15097|434869	-1	#DIV/0!	1	#DIV/0!
Gm10474|100038638	-1	#DIV/0!	1	#DIV/0!
1700084C01Rik|78465	-1	#DIV/0!	1	#DIV/0!
2810032G03Rik|72669	-1	#DIV/0!	1	#DIV/0!
Best3|382427	-1	#DIV/0!	1	#DIV/0!
Dscam|13508	-1	#DIV/0!	1	#DIV/0!
Gm5868|545758	-1	#DIV/0!	1	#DIV/0!
Gm17019|66773	-1	#DIV/0!	1	#DIV/0!
Snord66|100217430	-1	#DIV/0!	1	#DIV/0!
Ugt2a2|552899	-1	#DIV/0!	1	#DIV/0!
Prss44|73336	-1	#DIV/0!	1	#DIV/0!
Als2cr11|73463	-1	#DIV/0!	1	#DIV/0!
Cylc2|74914	-1	#DIV/0!	1	#DIV/0!
Snord71|100217432	-1	#DIV/0!	1	#DIV/0!
Vwa5b1|75718	-1	#DIV/0!	1	#DIV/0!
Krt16|16666	-1	#DIV/0!	1	#DIV/0!
Spink2|69982	-1	#DIV/0!	1	#DIV/0!
Sval3|387564	-1	#DIV/0!	1	#DIV/0!
BC049715|320135	-1	#DIV/0!	1	#DIV/0!
Serpinb3d|394252	-1	#DIV/0!	1	#DIV/0!
Cdh8|12564	-1	#DIV/0!	1	#DIV/0!
Gzmc|14940	-1	#DIV/0!	1	#DIV/0!
Mael|98558	-1	#DIV/0!	1	#DIV/0!
Duoxa2|66811	-1	#DIV/0!	1	#DIV/0!
2700046A07Rik|78449	-1	#DIV/0!	1	#DIV/0!
Dbpht2|386753	-1	#DIV/0!	1	#DIV/0!
Olfr74|117005	-1	#DIV/0!	1	#DIV/0!
5330413P13Rik|414081	-1	#DIV/0!	1	#DIV/0!
1700026J04Rik|71864	-1	#DIV/0!	1	#DIV/0!
Crabp1|12903	-1	#DIV/0!	1	#DIV/0!
Klk15|317652	-1	#DIV/0!	1	#DIV/0!
Slc7a13|74087	-1	#DIV/0!	1	#DIV/0!
4930529F22Rik|384806	-1	#DIV/0!	1	#DIV/0!
Klri1|503550	-1	#DIV/0!	1	#DIV/0!
Fshr|14309	-1	#DIV/0!	1	#DIV/0!
Olfr728|258039	-1	#DIV/0!	1	#DIV/0!

Hs6st2|50786	-1	#DIV/0!	1	#DIV/0!
Sohlh1|227631	-1	#DIV/0!	1	#DIV/0!
Grm4|268934	-1	#DIV/0!	1	#DIV/0!
Tas2r102|387339	-1	#DIV/0!	1	#DIV/0!
Gm4934|238662	-1	#DIV/0!	1	#DIV/0!
Gm609|208166	-1	#DIV/0!	1	#DIV/0!
Olfr1414|259041	-1	#DIV/0!	1	#DIV/0!
Olfr787|258069	-1	#DIV/0!	1	#DIV/0!
Mir129-1|387237	-1	#DIV/0!	1	#DIV/0!
Olfr1251|259145	-1	#DIV/0!	1	#DIV/0!
Folh1|53320	-1	#DIV/0!	1	#DIV/0!
Olfr355|258618	-1	#DIV/0!	1	#DIV/0!
Cysltr1|58861	-1	#DIV/0!	1	#DIV/0!
Zfp286|192651	-1	#DIV/0!	1	#DIV/0!
Mir1897|100316679	-1	#DIV/0!	1	#DIV/0!
Snord16a|100217453	-1	#DIV/0!	1	#DIV/0!
Sh3rf2|269016	-1	#DIV/0!	1	#DIV/0!
Olfr1287|257935	-1	#DIV/0!	1	#DIV/0!
Apobec2|11811	-1	#DIV/0!	1	#DIV/0!
Mir881|100124460	-1	#DIV/0!	1	#DIV/0!
Olfr320|216783	-1	#DIV/0!	1	#DIV/0!
Scarna9|100216535	-1	#DIV/0!	1	#DIV/0!
Olfr1344|257882	-1	#DIV/0!	1	#DIV/0!
Vmn1r110|100043561	-1	#DIV/0!	1	#DIV/0!
Krt24|75706	-1	#DIV/0!	1	#DIV/0!
Mir1251|100526517	-1	#DIV/0!	1	#DIV/0!
Olfr1015|258564	-1	#DIV/0!	1	#DIV/0!
Vwc2l|320460	-1	#DIV/0!	1	#DIV/0!
Gpr165|76206	-1	#DIV/0!	1	#DIV/0!
Vmn1r91|667067	-1	#DIV/0!	1	#DIV/0!
E030025P04Rik|268498	-1	#DIV/0!	1	#DIV/0!
Mir290|100049710	-1	#DIV/0!	1	#DIV/0!
Epha10|230735	-1	#DIV/0!	1	#DIV/0!
Olfr1221|258904	-1	#DIV/0!	1	#DIV/0!
Olfr1321|236785	-1	#DIV/0!	1	#DIV/0!
Mapk10|26414	-1	#DIV/0!	1	#DIV/0!
Zmat4|320158	-1	#DIV/0!	1	#DIV/0!
4933416C03Rik|619332	-1	#DIV/0!	1	#DIV/0!
Gm4214|100043079	-1	#DIV/0!	1	#DIV/0!
Gm3867|100042485	-1	#DIV/0!	1	#DIV/0!
Olfr102|258218	-1	#DIV/0!	1	#DIV/0!
Vmn2r8|627479	-1	#DIV/0!	1	#DIV/0!
Lrriq4|68307	-1	#DIV/0!	1	#DIV/0!
Lct|226413	-1	#DIV/0!	1	#DIV/0!
Snora15|100113364	-1	#DIV/0!	1	#DIV/0!
Olfr179|404346	-1	#DIV/0!	1	#DIV/0!
Lce1g|66195	-1	#DIV/0!	1	#DIV/0!
Trpm3|226025	-1	#DIV/0!	1	#DIV/0!
Grid2|14804	-1	#DIV/0!	1	#DIV/0!
D730001G18Rik|78725	-1	#DIV/0!	1	#DIV/0!
2210409E12Rik|72381	-1	#DIV/0!	1	#DIV/0!
Olfr1065|258403	-1	#DIV/0!	1	#DIV/0!
Snora36b|100217449	-1	#DIV/0!	1	#DIV/0!

Gm7849|665927	-1	#DIV/0!	1	#DIV/0!
Olfr1196|258456	-1	#DIV/0!	1	#DIV/0!
Tas2r117|353166	-1	#DIV/0!	1	#DIV/0!
C330024D21Rik|320479	-1	#DIV/0!	1	#DIV/0!
Olfr543|257947	-1	#DIV/0!	1	#DIV/0!
Mir2145-2|100316733	-1	#DIV/0!	1	#DIV/0!
Gm5800|545047	-1	#DIV/0!	1	#DIV/0!
AI414108|102623	-1	#DIV/0!	1	#DIV/0!
1700011A15Rik|66322	-1	#DIV/0!	1	#DIV/0!
Nalcn|338370	-1	#DIV/0!	1	#DIV/0!
Scnn1b|20277	-1	#DIV/0!	1	#DIV/0!
Olfr111|545205	-1	#DIV/0!	1	#DIV/0!
Olfr899|258472	-1	#DIV/0!	1	#DIV/0!
Anks1b|77531	-1	#DIV/0!	1	#DIV/0!
4921510H08Rik|66716	-1	#DIV/0!	1	#DIV/0!
Gm10754|100038699	-1	#DIV/0!	1	#DIV/0!
1700010B08Rik|75485	-1	#DIV/0!	1	#DIV/0!
Nphs2|170484	-1	#DIV/0!	1	#DIV/0!
Vmn2r72-ps|244114	-1	#DIV/0!	1	#DIV/0!
Myf6|17878	-1	#DIV/0!	1	#DIV/0!
Chrna6|11440	-1	#DIV/0!	1	#DIV/0!
Gm5039|266459	-1	#DIV/0!	1	#DIV/0!
Prss43|272643	-1	#DIV/0!	1	#DIV/0!
Mir343|100124438	-1	#DIV/0!	1	#DIV/0!
Slc15a1|56643	-1	#DIV/0!	1	#DIV/0!
Morc2b|240069	-1	#DIV/0!	1	#DIV/0!
Rhox4g|664608	-1	#DIV/0!	1	#DIV/0!
1700125D06Rik|68233	-1	#DIV/0!	1	#DIV/0!
Ccdc110|212392	-1	#DIV/0!	1	#DIV/0!
Olfr1099|258764	-1	#DIV/0!	1	#DIV/0!
Vmn1r222|171274	-1	#DIV/0!	1	#DIV/0!
Snora52|100217419	-1	#DIV/0!	1	#DIV/0!
Krtap16-2|170657	-1	#DIV/0!	1	#DIV/0!
Lhx8|16875	-1	#DIV/0!	1	#DIV/0!
Olfr104-ps|257948	-1	#DIV/0!	1	#DIV/0!
En2|13799	-1	#DIV/0!	1	#DIV/0!
Hus1b|210554	-1	#DIV/0!	1	#DIV/0!
Lpo|76113	-1	#DIV/0!	1	#DIV/0!
Olfr1424|258676	-1	#DIV/0!	1	#DIV/0!
Mir3064|100526469	-1	#DIV/0!	1	#DIV/0!
Icam5|15898	-1	#DIV/0!	1	#DIV/0!
Ovch2|244199	-1	#DIV/0!	1	#DIV/0!
Pof1b|69693	-1	#DIV/0!	1	#DIV/0!
Snord69|100217431	-1	#DIV/0!	1	#DIV/0!
Tas2r115|353325	-1	#DIV/0!	1	#DIV/0!
Pglyrp4|384997	-1	#DIV/0!	1	#DIV/0!
Olfr1500|258097	-1	#DIV/0!	1	#DIV/0!
Negr1|320840	-1	#DIV/0!	1	#DIV/0!
Gm13125|627009	-1	#DIV/0!	1	#DIV/0!
Mir669j|100316770	-1	#DIV/0!	1	#DIV/0!
Olfr716|258597	-1	#DIV/0!	1	#DIV/0!
Tas2r143|387514	-1	#DIV/0!	1	#DIV/0!
Gpr151|240239	-1	#DIV/0!	1	#DIV/0!

Olfr190|258392	-1	#DIV/0!	1	#DIV/0!
Scg5|20394	-1	#DIV/0!	1	#DIV/0!
Spata20|217116	-1	#DIV/0!	1	#DIV/0!
1700027A23Rik|100503311	-1	#DIV/0!	1	#DIV/0!
Ppp1r2-ps7|76705	-1	#DIV/0!	1	#DIV/0!
Kcnk7|16530	-1	#DIV/0!	1	#DIV/0!
Ccdc70|67929	-1	#DIV/0!	1	#DIV/0!
Iqcj|208426	-1	#DIV/0!	1	#DIV/0!
Hist1h3g|97908	-1	#DIV/0!	1	#DIV/0!
Mir7-1|723902	-1	#DIV/0!	1	#DIV/0!
Olfr661|258743	-1	#DIV/0!	1	#DIV/0!
Stra6|20897	-1	#DIV/0!	1	#DIV/0!
Gabra6|14399	-1	#DIV/0!	1	#DIV/0!
Slc17a7|72961	-1	#DIV/0!	1	#DIV/0!
Gpc5|103978	-1	#DIV/0!	1	#DIV/0!
Olfr293|257906	-1	#DIV/0!	1	#DIV/0!
Gm8096|666422	-1	#DIV/0!	1	#DIV/0!
4933430I17Rik|214106	-1	#DIV/0!	1	#DIV/0!
Dcst1|77772	-1	#DIV/0!	1	#DIV/0!
Tinag|26944	-1	#DIV/0!	1	#DIV/0!
Evx1|14028	-1	#DIV/0!	1	#DIV/0!
Ankk1|244859	-1	#DIV/0!	1	#DIV/0!
Unc5d|210801	-1	#DIV/0!	1	#DIV/0!
Mir1903|100316680	-1	#DIV/0!	1	#DIV/0!
Olfr1106|258747	-1	#DIV/0!	1	#DIV/0!
Mir883a|100124462	-1	#DIV/0!	1	#DIV/0!
Troap|78733	-1	#DIV/0!	1	#DIV/0!
Krtap9-5|435286	-1	#DIV/0!	1	#DIV/0!
Ano3|228432	-1	#DIV/0!	1	#DIV/0!
Mir541|723941	-1	#DIV/0!	1	#DIV/0!
A730018C14Rik|100504733	-1	#DIV/0!	1	#DIV/0!
Col10a1|12813	-1	#DIV/0!	1	#DIV/0!
1700010D01Rik|76386	-1	#DIV/0!	1	#DIV/0!
Resp18|19711	-1	#DIV/0!	1	#DIV/0!
Mir298|723832	-1	#DIV/0!	1	#DIV/0!
Mro|71263	-1	#DIV/0!	1	#DIV/0!
Lhx4|16872	-1	#DIV/0!	1	#DIV/0!
Vmn1r185|171265	-1	#DIV/0!	1	#DIV/0!
Myf5|17877	-1	#DIV/0!	1	#DIV/0!
Vmn2r61|637873	-1	#DIV/0!	1	#DIV/0!
Defb30|73670	-1	#DIV/0!	1	#DIV/0!
1700003H04Rik|384775	-1	#DIV/0!	1	#DIV/0!
Hist1h4m|100041230	-1	#DIV/0!	1	#DIV/0!
Skint3|195564	-1	#DIV/0!	1	#DIV/0!
Ifne|230405	-1	#DIV/0!	1	#DIV/0!
Speer4a|75657	-1	#DIV/0!	1	#DIV/0!
4932414J04Rik|237694	-1	#DIV/0!	1	#DIV/0!
Olfr48|18347	-1	#DIV/0!	1	#DIV/0!
Ankrd34a|545554	-1	#DIV/0!	1	#DIV/0!
Gm4847|226604	-1	#DIV/0!	1	#DIV/0!
Olfr119|258095	-1	#DIV/0!	1	#DIV/0!
Spink12|78242	-1	#DIV/0!	1	#DIV/0!
Spata19|75469	-1	#DIV/0!	1	#DIV/0!

Spata9|75571	-1	#DIV/0!	1	#DIV/0!
Taar7f|435207	-1	#DIV/0!	1	#DIV/0!
Olfr393|259010	-1	#DIV/0!	1	#DIV/0!
Samd5|320825	-1	#DIV/0!	1	#DIV/0!
Olfr1454|258687	-1	#DIV/0!	1	#DIV/0!
Adad2|75773	-1	#DIV/0!	1	#DIV/0!
1700030F18Rik|72277	-1	#DIV/0!	1	#DIV/0!
Olfr193|257972	-1	#DIV/0!	1	#DIV/0!
Pcdhga11|93723	-1	#DIV/0!	1	#DIV/0!
Btbd16|330660	-1	#DIV/0!	1	#DIV/0!
Fthl17|83457	-1	#DIV/0!	1	#DIV/0!
Olfr685|258160	-1	#DIV/0!	1	#DIV/0!
Osbpl6|99031	-1	#DIV/0!	1	#DIV/0!
Tmem132c|208213	-1	#DIV/0!	1	#DIV/0!
Olfr1141|258630	-1	#DIV/0!	1	#DIV/0!
Mir383|723860	-1	#DIV/0!	1	#DIV/0!
Tubal3|238463	-1	#DIV/0!	1	#DIV/0!
Olfr745|258296	-1	#DIV/0!	1	#DIV/0!
Mir190b|100124481	-1	#DIV/0!	1	#DIV/0!
Olfr1008|258866	-1	#DIV/0!	1	#DIV/0!
Rnf207|433809	-1	#DIV/0!	1	#DIV/0!
Mirlet7a-1|387244	-1	#DIV/0!	1	#DIV/0!
Gkn3|68888	-1	#DIV/0!	1	#DIV/0!
Olfr94|258219	-1	#DIV/0!	1	#DIV/0!
2410017I17Rik|675325	-1	#DIV/0!	1	#DIV/0!
Hist1h2ab|319172	-1	#DIV/0!	1	#DIV/0!
Psg22|243862	-1	#DIV/0!	1	#DIV/0!
Olfr895|258875	-1	#DIV/0!	1	#DIV/0!
1700013G24Rik|69380	-1	#DIV/0!	1	#DIV/0!
Ldhal6b|106557	-1	#DIV/0!	1	#DIV/0!
5031410I06Rik|381622	-1	#DIV/0!	1	#DIV/0!
Galntl6|270049	-1	#DIV/0!	1	#DIV/0!
Olfr292|258613	-1	#DIV/0!	1	#DIV/0!
Olfr818|258773	-1	#DIV/0!	1	#DIV/0!
Gm1661|381544	-1	#DIV/0!	1	#DIV/0!
Vgll1|170828	-1	#DIV/0!	1	#DIV/0!
Mir138-2|723956	-1	#DIV/0!	1	#DIV/0!
Serpina3j|238395	-1	#DIV/0!	1	#DIV/0!
Myh8|17885	-1	#DIV/0!	1	#DIV/0!
Olfr1218|258815	-1	#DIV/0!	1	#DIV/0!
Gm13128|626995	-1	#DIV/0!	1	#DIV/0!
Pax8|18510	-1	#DIV/0!	1	#DIV/0!
Slc6a19|74338	-1	#DIV/0!	1	#DIV/0!
2900079G21Rik|620760	-1	#DIV/0!	1	#DIV/0!
Gm17677|100312949	-1	#DIV/0!	1	#DIV/0!
Gm17365|100312956	-1	#DIV/0!	1	#DIV/0!
2210407C18Rik|78354	-1	#DIV/0!	1	#DIV/0!
Olfr401|258701	-1	#DIV/0!	1	#DIV/0!
Cnga1|12788	-1	#DIV/0!	1	#DIV/0!
Zfp474|66758	-1	#DIV/0!	1	#DIV/0!
Mir540|723880	-1	#DIV/0!	1	#DIV/0!
Gm4861|229862	-1	#DIV/0!	1	#DIV/0!
Mgat5b|268510	-1	#DIV/0!	1	#DIV/0!

Mir467a-7|100526553	-1	#DIV/0!	1	#DIV/0!
Olfr1288|258395	-1	#DIV/0!	1	#DIV/0!
Lefty2|320202	-1	#DIV/0!	1	#DIV/0!
Snord49a|100217455	-1	#DIV/0!	1	#DIV/0!
Vmn1r218|171256	-1	#DIV/0!	1	#DIV/0!
Olfr1257|258984	-1	#DIV/0!	1	#DIV/0!
Spata3|70060	-1	#DIV/0!	1	#DIV/0!
Nefl|18039	-1	#DIV/0!	1	#DIV/0!
Olfr711|259037	-1	#DIV/0!	1	#DIV/0!
Gm12603|100040617	-1	#DIV/0!	1	#DIV/0!
Mir341|723846	-1	#DIV/0!	1	#DIV/0!
Lrrc52|240899	-1	#DIV/0!	1	#DIV/0!
1700052I22Rik|67340	-1	#DIV/0!	1	#DIV/0!
E130304I02Rik|78547	-1	#DIV/0!	1	#DIV/0!
1700011L22Rik|67687	-1	#DIV/0!	1	#DIV/0!
Krt76|77055	-1	#DIV/0!	1	#DIV/0!
Hist1h2bg|319181	-1	#DIV/0!	1	#DIV/0!
Vmn2r1|56544	-1	#DIV/0!	1	#DIV/0!
Olfr871|258905	-1	#DIV/0!	1	#DIV/0!
Gje1|76743	-1	#DIV/0!	1	#DIV/0!
Dmrtc1c1|71083	-1	#DIV/0!	1	#DIV/0!
Calm5|494124	-1	#DIV/0!	1	#DIV/0!
AY761185|503556	-1	#DIV/0!	1	#DIV/0!
Csrnp3|77771	-1	#DIV/0!	1	#DIV/0!
Uty|22290	-1	#DIV/0!	1	#DIV/0!
Tgm7|640543	-1	#DIV/0!	1	#DIV/0!
Vmn1r210|171269	-1	#DIV/0!	1	#DIV/0!
D430041D05Rik|241589	-1	#DIV/0!	1	#DIV/0!
Vmn2r79|621430	-1	#DIV/0!	1	#DIV/0!
Mep1b|17288	-1	#DIV/0!	1	#DIV/0!
2310002L09Rik|71886	-1	#DIV/0!	1	#DIV/0!
Neu4|241159	-1	#DIV/0!	1	#DIV/0!
Pnma2|239157	-1	#DIV/0!	1	#DIV/0!
Coro6|216961	-1	#DIV/0!	1	#DIV/0!
Cyp2c65|72303	-1	#DIV/0!	1	#DIV/0!
Cdx2|12591	-1	#DIV/0!	1	#DIV/0!
Krt1|16678	-1	#DIV/0!	1	#DIV/0!
Mmp1b|83996	-1	#DIV/0!	1	#DIV/0!
1700071K01Rik|237880	-1	#DIV/0!	1	#DIV/0!
Mir10a|723893	-1	#DIV/0!	1	#DIV/0!
Kcng1|241794	-1	#DIV/0!	1	#DIV/0!
Lao1|100470	-1	#DIV/0!	1	#DIV/0!
5530400C23Rik|232426	-1	#DIV/0!	1	#DIV/0!
Gpr1|241070	-1	#DIV/0!	1	#DIV/0!
Gm10334|100040233	-1	#DIV/0!	1	#DIV/0!
Cldn26|74720	-1	#DIV/0!	1	#DIV/0!
Cetn4|207175	-1	#DIV/0!	1	#DIV/0!
Olfr560|259117	-1	#DIV/0!	1	#DIV/0!
Ppef1|237178	-1	#DIV/0!	1	#DIV/0!
Camkv|235604	-1	#DIV/0!	1	#DIV/0!
Sprr2e|20759	-1	#DIV/0!	1	#DIV/0!
Olfr952|235248	-1	#DIV/0!	1	#DIV/0!
Ttc18|76670	-1	#DIV/0!	1	#DIV/0!

Gm749|328788	-1	#DIV/0!	1	#DIV/0!
Ly6k|76486	-1	#DIV/0!	1	#DIV/0!
Tfap2d|226896	-1	#DIV/0!	1	#DIV/0!
Slc22a8|19879	-1	#DIV/0!	1	#DIV/0!
Mir124a-3|723951	-1	#DIV/0!	1	#DIV/0!
2200002J24Rik|69147	-1	#DIV/0!	1	#DIV/0!
Olfr750|404319	-1	#DIV/0!	1	#DIV/0!
Olfr141|257913	-1	#DIV/0!	1	#DIV/0!
A330050F15Rik|320722	-1	#DIV/0!	1	#DIV/0!
Eps8l3|99662	-1	#DIV/0!	1	#DIV/0!
Olfr936|628171	-1	#DIV/0!	1	#DIV/0!
Tmem117|320709	-1	#DIV/0!	1	#DIV/0!
Cetn1|26369	-1	#DIV/0!	1	#DIV/0!
Odam|69592	-1	#DIV/0!	1	#DIV/0!
Cypt15|78631	-1	#DIV/0!	1	#DIV/0!
Btnl7|195349	-1	#DIV/0!	1	#DIV/0!
Olfr806|258546	-1	#DIV/0!	1	#DIV/0!
Olfr957|258740	-1	#DIV/0!	1	#DIV/0!
Dcpp1|13184	-1	#DIV/0!	1	#DIV/0!
1700007K09Rik|69318	-1	#DIV/0!	1	#DIV/0!
Olfr9|18373	-1	#DIV/0!	1	#DIV/0!
Trdn|76757	-1	#DIV/0!	1	#DIV/0!
Olfr1054|259021	-1	#DIV/0!	1	#DIV/0!
Olfr1279|258388	-1	#DIV/0!	1	#DIV/0!
Gm11596|670464	-1	#DIV/0!	1	#DIV/0!
Gm5577|434064	-1	#DIV/0!	1	#DIV/0!
Olfr1152|258103	-1	#DIV/0!	1	#DIV/0!
Olfr204|258994	-1	#DIV/0!	1	#DIV/0!
Olfr1395|258877	-1	#DIV/0!	1	#DIV/0!
Tmc1|13409	-1	#DIV/0!	1	#DIV/0!
Rnf17|30054	-1	#DIV/0!	1	#DIV/0!
Mir344f|100526527	-1	#DIV/0!	1	#DIV/0!
Plekhg4|102075	-1	#DIV/0!	1	#DIV/0!
Slxl1|75140	-1	#DIV/0!	1	#DIV/0!
Tcl1b5|27382	-1	#DIV/0!	1	#DIV/0!
Snord85|100217460	-1	#DIV/0!	1	#DIV/0!
Pcsk1n|30052	-1	#DIV/0!	1	#DIV/0!
Xirp2|241431	-1	#DIV/0!	1	#DIV/0!
Vrtn|432677	-1	#DIV/0!	1	#DIV/0!
Gm839|330379	-1	#DIV/0!	1	#DIV/0!
Rnase12|497106	-1	#DIV/0!	1	#DIV/0!
7420461P10Rik|100038657	-1	#DIV/0!	1	#DIV/0!
4930412O13Rik|381347	-1	#DIV/0!	1	#DIV/0!
40978|632687	-1	#DIV/0!	1	#DIV/0!
Hephl1|244698	-1	#DIV/0!	1	#DIV/0!
Insrr|23920	-1	#DIV/0!	1	#DIV/0!
Vmn1r216|171279	-1	#DIV/0!	1	#DIV/0!
Odf3l1|382075	-1	#DIV/0!	1	#DIV/0!
Vmn1r103|667129	-1	#DIV/0!	1	#DIV/0!
Tuba3a|22144	-1	#DIV/0!	1	#DIV/0!
Mir136|387154	-1	#DIV/0!	1	#DIV/0!
Gm8720|667599	-1	#DIV/0!	1	#DIV/0!
Dynlrb2|75465	-1	#DIV/0!	1	#DIV/0!

A330050B17Rik|654424	-1	#DIV/0!	1	#DIV/0!
Cst10|58214	-1	#DIV/0!	1	#DIV/0!
Gsdmc2|331063	-1	#DIV/0!	1	#DIV/0!
Svs5|20944	-1	#DIV/0!	1	#DIV/0!
Odf1|18285	-1	#DIV/0!	1	#DIV/0!
Vmn2r4|637053	-1	#DIV/0!	1	#DIV/0!
2300003K06Rik|666021	-1	#DIV/0!	1	#DIV/0!
Nox1|237038	-1	#DIV/0!	1	#DIV/0!
Serpinb7|116872	-1	#DIV/0!	1	#DIV/0!
Vmn2r75|546981	-1	#DIV/0!	1	#DIV/0!
Slc26a9|320718	-1	#DIV/0!	1	#DIV/0!
Prl|19109	-1	#DIV/0!	1	#DIV/0!
Olfr1535|404335	-1	#DIV/0!	1	#DIV/0!
Prss41|71003	-1	#DIV/0!	1	#DIV/0!
Gm13285|545654	-1	#DIV/0!	1	#DIV/0!
Trim58|216781	-1	#DIV/0!	1	#DIV/0!
Lrrn2|16980	-1	#DIV/0!	1	#DIV/0!
Olfr911-ps1|258873	-1	#DIV/0!	1	#DIV/0!
Mir3092|100526525	-1	#DIV/0!	1	#DIV/0!
Vmn1r231|171230	-1	#DIV/0!	1	#DIV/0!
Mir469|723872	-1	#DIV/0!	1	#DIV/0!
Vmn2r66|233437	-1	#DIV/0!	1	#DIV/0!
Pcdhga12|93724	-1	#DIV/0!	1	#DIV/0!
Hrasls5|66727	-1	#DIV/0!	1	#DIV/0!
Ceacam11|66996	-1	#DIV/0!	1	#DIV/0!
Olfr823|258668	-1	#DIV/0!	1	#DIV/0!
Spinkl|77424	-1	#DIV/0!	1	#DIV/0!
Tas2r134|387511	-1	#DIV/0!	1	#DIV/0!
Rnu12|104307	-1	#DIV/0!	1	#DIV/0!
Olfr1466|258689	-1	#DIV/0!	1	#DIV/0!
Mir216a|387212	-1	#DIV/0!	1	#DIV/0!
Trp73|22062	-1	#DIV/0!	1	#DIV/0!
Bpifb5|228802	-1	#DIV/0!	1	#DIV/0!
Catsperg2|76718	-1	#DIV/0!	1	#DIV/0!
Tceal7|100040972	-1	#DIV/0!	1	#DIV/0!
Ddn|13199	-1	#DIV/0!	1	#DIV/0!
Mos|17451	-1	#DIV/0!	1	#DIV/0!
Olfr1390|259068	-1	#DIV/0!	1	#DIV/0!
4921501E09Rik|74042	-1	#DIV/0!	1	#DIV/0!
Olfr1039|257950	-1	#DIV/0!	1	#DIV/0!
Gjd2|14617	-1	#DIV/0!	1	#DIV/0!
Cdh20|23836	-1	#DIV/0!	1	#DIV/0!
Ctf2|244218	-1	#DIV/0!	1	#DIV/0!
Mirlet7c-1|387246	-1	#DIV/0!	1	#DIV/0!
1700072E05Rik|73495	-1	#DIV/0!	1	#DIV/0!
Mir880|100124480	-1	#DIV/0!	1	#DIV/0!
Aox4|71872	-1	#DIV/0!	1	#DIV/0!
2310050C09Rik|66533	-1	#DIV/0!	1	#DIV/0!
2610305D13Rik|112422	-1	#DIV/0!	1	#DIV/0!
Krtap16-4|170654	-1	#DIV/0!	1	#DIV/0!
Mmp1a|83995	-1	#DIV/0!	1	#DIV/0!
Pla2g1b|18778	-1	#DIV/0!	1	#DIV/0!
Mir1197|100316751	-1	#DIV/0!	1	#DIV/0!

4921511C20Rik|245598	-1	#DIV/0!	1	#DIV/0!
Spag11b|546038	-1	#DIV/0!	1	#DIV/0!
Csnka2ip|224291	-1	#DIV/0!	1	#DIV/0!
9130404H23Rik|74556	-1	#DIV/0!	1	#DIV/0!
Prss35|244954	-1	#DIV/0!	1	#DIV/0!
Gm906|380882	-1	#DIV/0!	1	#DIV/0!
Htr1f|15557	-1	#DIV/0!	1	#DIV/0!
H2-M10.5|224761	-1	#DIV/0!	1	#DIV/0!
BC021785|215928	-1	#DIV/0!	1	#DIV/0!
Ifna12|242519	-1	#DIV/0!	1	#DIV/0!
Cntn3|18488	-1	#DIV/0!	1	#DIV/0!
Mir466g|100124495	-1	#DIV/0!	1	#DIV/0!
Gm9961|791359	-1	#DIV/0!	1	#DIV/0!
Il10|16153	-1	#DIV/0!	1	#DIV/0!
Mab21l1|17116	-1	#DIV/0!	1	#DIV/0!
Kcna4|16492	-1	#DIV/0!	1	#DIV/0!
Olfr629|258818	-1	#DIV/0!	1	#DIV/0!
Olfr774|258232	-1	#DIV/0!	1	#DIV/0!
Foxl2|26927	-1	#DIV/0!	1	#DIV/0!
Tmem217|71138	-1	#DIV/0!	1	#DIV/0!
Vmn1r129|621510	-1	#DIV/0!	1	#DIV/0!
Mir199a-1|387194	-1	#DIV/0!	1	#DIV/0!
Fbxw28|668758	-1	#DIV/0!	1	#DIV/0!
Vmn1r33|171187	-1	#DIV/0!	1	#DIV/0!
Slco4c1|227394	-1	#DIV/0!	1	#DIV/0!
Fxyd4|108017	-1	#DIV/0!	1	#DIV/0!
Il3|16187	-1	#DIV/0!	1	#DIV/0!
Mesp2|17293	-1	#DIV/0!	1	#DIV/0!
Olfr38|258988	-1	#DIV/0!	1	#DIV/0!
Pth2r|213527	-1	#DIV/0!	1	#DIV/0!
Olfr1140|258635	-1	#DIV/0!	1	#DIV/0!
Mir410|723863	-1	#DIV/0!	1	#DIV/0!
Gm5294|384244	-1	#DIV/0!	1	#DIV/0!
Ttc34|242800	-1	#DIV/0!	1	#DIV/0!
Olfr845|258249	-1	#DIV/0!	1	#DIV/0!
Pcdha7|12939	-1	#DIV/0!	1	#DIV/0!
Sec1|56546	-1	#DIV/0!	1	#DIV/0!
Olfr32|18331	-1	#DIV/0!	1	#DIV/0!
Fam183b|75429	-1	#DIV/0!	1	#DIV/0!
Snord100|100529075	-1	#DIV/0!	1	#DIV/0!
Olfr1348|258915	-1	#DIV/0!	1	#DIV/0!
Khdc1b|98582	-1	#DIV/0!	1	#DIV/0!
Olfr658|259051	-1	#DIV/0!	1	#DIV/0!
4931423N10Rik|70981	-1	#DIV/0!	1	#DIV/0!
9630028H03Rik|320684	-1	#DIV/0!	1	#DIV/0!
Reg3a|19694	-1	#DIV/0!	1	#DIV/0!
Vmn1r59|404284	-1	#DIV/0!	1	#DIV/0!
Olfr156|29846	-1	#DIV/0!	1	#DIV/0!
1700109G14Rik|67355	-1	#DIV/0!	1	#DIV/0!
Sun5|76407	-1	#DIV/0!	1	#DIV/0!
Bmp15|12155	-1	#DIV/0!	1	#DIV/0!
Mir208a|387204	-1	#DIV/0!	1	#DIV/0!
Gm12789|381536	-1	#DIV/0!	1	#DIV/0!

Vmn1r28|171198	-1	#DIV/0!	1	#DIV/0!
Olfr874|258882	-1	#DIV/0!	1	#DIV/0!
Slc5a4b|64454	-1	#DIV/0!	1	#DIV/0!
Mir351|723910	-1	#DIV/0!	1	#DIV/0!
Lin28b|380669	-1	#DIV/0!	1	#DIV/0!
Btn2a2|238555	-1	#DIV/0!	1	#DIV/0!
Slc6a15|103098	-1	#DIV/0!	1	#DIV/0!
Poteg|70952	-1	#DIV/0!	1	#DIV/0!
Tfap2b|21419	-1	#DIV/0!	1	#DIV/0!
Gpr6|140741	-1	#DIV/0!	1	#DIV/0!
Fam70a|245386	-1	#DIV/0!	1	#DIV/0!
Fut9|14348	-1	#DIV/0!	1	#DIV/0!
Mir3094|100526501	-1	#DIV/0!	1	#DIV/0!
F730043M19Rik|320046	-1	#DIV/0!	1	#DIV/0!
Tnr|21960	-1	#DIV/0!	1	#DIV/0!
Gcnt3|72077	-1	#DIV/0!	1	#DIV/0!
Hist1h2an|319170	-1	#DIV/0!	1	#DIV/0!
Cacng6|54378	-1	#DIV/0!	1	#DIV/0!
Fbxw13|211305	-1	#DIV/0!	1	#DIV/0!
Ankfn1|382543	-1	#DIV/0!	1	#DIV/0!
Gm16381|100042786	-1	#DIV/0!	1	#DIV/0!
Gm17384|100322896	-1	#DIV/0!	1	#DIV/0!
C530008M17Rik|320827	-1	#DIV/0!	1	#DIV/0!
Olfr608|258751	-1	#DIV/0!	1	#DIV/0!
Foxc1|17300	-1	#DIV/0!	1	#DIV/0!
Gpa33|59290	-1	#DIV/0!	1	#DIV/0!
Nkx3-1|18095	-1	#DIV/0!	1	#DIV/0!
Olfr1496|258991	-1	#DIV/0!	1	#DIV/0!
Psapl1|76943	-1	#DIV/0!	1	#DIV/0!
Xlr5b|627081	-1	#DIV/0!	1	#DIV/0!
Olfr1225|258893	-1	#DIV/0!	1	#DIV/0!
Gm5111|330305	-1	#DIV/0!	1	#DIV/0!
Cngb1|333329	-1	#DIV/0!	1	#DIV/0!
Spag6|50525	-1	#DIV/0!	1	#DIV/0!
Mir22|387141	-1	#DIV/0!	1	#DIV/0!
4930404H21Rik|73808	-1	#DIV/0!	1	#DIV/0!
Rgs7|24012	-1	#DIV/0!	1	#DIV/0!
Pde6a|225600	-1	#DIV/0!	1	#DIV/0!
Mir29b-1|387223	-1	#DIV/0!	1	#DIV/0!
Gm13101|626922	-1	#DIV/0!	1	#DIV/0!
Snord4a|100216539	-1	#DIV/0!	1	#DIV/0!
Pabpc2|18459	-1	#DIV/0!	1	#DIV/0!
4921517D22Rik|70900	-1	#DIV/0!	1	#DIV/0!
Mcpt9|17232	-1	#DIV/0!	1	#DIV/0!
Itln1|16429	-1	#DIV/0!	1	#DIV/0!
Dnahc9|237806	-1	#DIV/0!	1	#DIV/0!
Hmx3|15373	-1	#DIV/0!	1	#DIV/0!
Krtap7-1|71363	-1	#DIV/0!	1	#DIV/0!
Fate1|77905	-1	#DIV/0!	1	#DIV/0!
Ms4a13|73466	-1	#DIV/0!	1	#DIV/0!
Asb16|217217	-1	#DIV/0!	1	#DIV/0!
Gm5483|433016	-1	#DIV/0!	1	#DIV/0!
Mir211|387207	-1	#DIV/0!	1	#DIV/0!

Abo|80908	-1	#DIV/0!	1	#DIV/0!
Gm438|329993	-1	#DIV/0!	1	#DIV/0!
Ear4|53877	-1	#DIV/0!	1	#DIV/0!
Calcr|12311	-1	#DIV/0!	1	#DIV/0!
Tcl1b2|27381	-1	#DIV/0!	1	#DIV/0!
Dytn|241073	-1	#DIV/0!	1	#DIV/0!
Mir691|751561	-1	#DIV/0!	1	#DIV/0!
Krtap1-4|629873	-1	#DIV/0!	1	#DIV/0!
Olfr930|258269	-1	#DIV/0!	1	#DIV/0!
Lyg2|332427	-1	#DIV/0!	1	#DIV/0!
Spink6|433180	-1	#DIV/0!	1	#DIV/0!
Olfr1316|258737	-1	#DIV/0!	1	#DIV/0!
Olfr968|258605	-1	#DIV/0!	1	#DIV/0!
Lce1a2|73722	-1	#DIV/0!	1	#DIV/0!
Scd3|30049	-1	#DIV/0!	1	#DIV/0!
Hs3st2|195646	-1	#DIV/0!	1	#DIV/0!
Lrrc43|381741	-1	#DIV/0!	1	#DIV/0!
Nanog|71950	-1	#DIV/0!	1	#DIV/0!
Olfr847|258518	-1	#DIV/0!	1	#DIV/0!
Olfr307|258610	-1	#DIV/0!	1	#DIV/0!
Oog4|242737	-1	#DIV/0!	1	#DIV/0!
Tmem190|78052	-1	#DIV/0!	1	#DIV/0!
Olfr462|258406	-1	#DIV/0!	1	#DIV/0!
Lrfn5|238205	-1	#DIV/0!	1	#DIV/0!
Vmn2r117|619788	-1	#DIV/0!	1	#DIV/0!
Scarna10|100217413	-1	#DIV/0!	1	#DIV/0!
4933412E24Rik|71088	-1	#DIV/0!	1	#DIV/0!
Olfr1271|258789	-1	#DIV/0!	1	#DIV/0!
Cass4|320664	-1	#DIV/0!	1	#DIV/0!
1700086L19Rik|74284	-1	#DIV/0!	1	#DIV/0!
Olfr1433|258680	-1	#DIV/0!	1	#DIV/0!
Snord90|100217435	-1	#DIV/0!	1	#DIV/0!
Taar8a|215859	-1	#DIV/0!	1	#DIV/0!
Olfr172|259003	-1	#DIV/0!	1	#DIV/0!
Prss32|69814	-1	#DIV/0!	1	#DIV/0!
Sptlc3|228677	-1	#DIV/0!	1	#DIV/0!
Mir1962|100316818	-1	#DIV/0!	1	#DIV/0!
Gm13178|546849	-1	#DIV/0!	1	#DIV/0!
Cubn|65969	-1	#DIV/0!	1	#DIV/0!
Olfr981|258283	-1	#DIV/0!	1	#DIV/0!
Gsdmc4|74548	-1	#DIV/0!	1	#DIV/0!
Nms|433292	-1	#DIV/0!	1	#DIV/0!
Mir1892|100316682	-1	#DIV/0!	1	#DIV/0!
Lcn8|78076	-1	#DIV/0!	1	#DIV/0!
Glp1r|14652	-1	#DIV/0!	1	#DIV/0!
Gm7073|631784	-1	#DIV/0!	1	#DIV/0!
Olfr702|258590	-1	#DIV/0!	1	#DIV/0!
Hist1h2ak|319169	-1	#DIV/0!	1	#DIV/0!
Sprr2k|20765	-1	#DIV/0!	1	#DIV/0!
Nkx2-5|18091	-1	#DIV/0!	1	#DIV/0!
Pramel3|83565	-1	#DIV/0!	1	#DIV/0!
Olfr1095|258725	-1	#DIV/0!	1	#DIV/0!
Adam6b|238405	-1	#DIV/0!	1	#DIV/0!

Gm6320|622408	-1	#DIV/0!	1	#DIV/0!
Sis|69983	-1	#DIV/0!	1	#DIV/0!
Krt75|109052	-1	#DIV/0!	1	#DIV/0!
Klra9|16640	-1	#DIV/0!	1	#DIV/0!
Hrh4|225192	-1	#DIV/0!	1	#DIV/0!
Amph|218038	-1	#DIV/0!	1	#DIV/0!
Mir133a-2|723954	-1	#DIV/0!	1	#DIV/0!
Olfr665|258810	-1	#DIV/0!	1	#DIV/0!
Pdha2|18598	-1	#DIV/0!	1	#DIV/0!
Gm11166|625850	-1	#DIV/0!	1	#DIV/0!
Olfr266|258482	-1	#DIV/0!	1	#DIV/0!
Olig2|50913	-1	#DIV/0!	1	#DIV/0!
Mir582|100124489	-1	#DIV/0!	1	#DIV/0!
Olfr259|258766	-1	#DIV/0!	1	#DIV/0!
Gkn2|66284	-1	#DIV/0!	1	#DIV/0!
Il17f|257630	-1	#DIV/0!	1	#DIV/0!
Mchr1|207911	-1	#DIV/0!	1	#DIV/0!
Vmn2r15|211223	-1	#DIV/0!	1	#DIV/0!
Olfr531|258955	-1	#DIV/0!	1	#DIV/0!
Mir376a|723855	-1	#DIV/0!	1	#DIV/0!
Il21|60505	-1	#DIV/0!	1	#DIV/0!
Lhb|16866	-1	#DIV/0!	1	#DIV/0!
Npy6r|18169	-1	#DIV/0!	1	#DIV/0!
D030047H15Rik|100037396	-1	#DIV/0!	1	#DIV/0!
Gm694|277744	-1	#DIV/0!	1	#DIV/0!
BC053393|407814	-1	#DIV/0!	1	#DIV/0!
4930471G03Rik|74966	-1	#DIV/0!	1	#DIV/0!
9330159F19Rik|212448	-1	#DIV/0!	1	#DIV/0!
4933436I01Rik|66780	-1	#DIV/0!	1	#DIV/0!
Prss21|57256	-1	#DIV/0!	1	#DIV/0!
Olfr707|194433	-1	#DIV/0!	1	#DIV/0!
Defa-rs1|13218	-1	#DIV/0!	1	#DIV/0!
Rhox2a|75199	-1	#DIV/0!	1	#DIV/0!
4930415L06Rik|245511	-1	#DIV/0!	1	#DIV/0!
Arhgef33|381112	-1	#DIV/0!	1	#DIV/0!
Lrriq1|74978	-1	#DIV/0!	1	#DIV/0!
Mir1193|100316673	-1	#DIV/0!	1	#DIV/0!
Rab39b|67790	-1	#DIV/0!	1	#DIV/0!
Tbx18|76365	-1	#DIV/0!	1	#DIV/0!
Tspyl5|239364	-1	#DIV/0!	1	#DIV/0!
Fam196b|574403	-1	#DIV/0!	1	#DIV/0!
4930568D16Rik|75859	-1	#DIV/0!	1	#DIV/0!
Rgag1|209540	-1	#DIV/0!	1	#DIV/0!
Abca16|233810	-1	#DIV/0!	1	#DIV/0!
Vmn2r78|637896	-1	#DIV/0!	1	#DIV/0!
Olfr1014|258562	-1	#DIV/0!	1	#DIV/0!
Mir1982|100316778	-1	#DIV/0!	1	#DIV/0!
Fgf16|80903	-1	#DIV/0!	1	#DIV/0!
Tex15|104271	-1	#DIV/0!	1	#DIV/0!
Ccdc74a|72315	-1	#DIV/0!	1	#DIV/0!
Retnlb|57263	-1	#DIV/0!	1	#DIV/0!
Olfr1353|259044	-1	#DIV/0!	1	#DIV/0!
5430419D17Rik|71395	-1	#DIV/0!	1	#DIV/0!

Agr3|403205	-1	#DIV/0!	1	#DIV/0!
Chrnb3|108043	-1	#DIV/0!	1	#DIV/0!
Hcn1|15165	-1	#DIV/0!	1	#DIV/0!
AK129341|234915	-1	#DIV/0!	1	#DIV/0!
Hmx2|15372	-1	#DIV/0!	1	#DIV/0!
Mir4660|100526482	-1	#DIV/0!	1	#DIV/0!
1700028B04Rik|70001	-1	#DIV/0!	1	#DIV/0!
2610318N02Rik|70458	-1	#DIV/0!	1	#DIV/0!
Olfr510|258308	-1	#DIV/0!	1	#DIV/0!
Mir10b|387144	-1	#DIV/0!	1	#DIV/0!
Olfr1380|404336	-1	#DIV/0!	1	#DIV/0!
Mir181d|100049549	-1	#DIV/0!	1	#DIV/0!
Spaca5|278203	-1	#DIV/0!	1	#DIV/0!
AF357355|100303646	-1	#DIV/0!	1	#DIV/0!
Vmn2r101|627576	-1	#DIV/0!	1	#DIV/0!
Olfr549|259105	-1	#DIV/0!	1	#DIV/0!
BB287469|544881	-1	#DIV/0!	1	#DIV/0!
Olfr1420|258405	-1	#DIV/0!	1	#DIV/0!
Gm5796|545007	-1	#DIV/0!	1	#DIV/0!
Krtap16-7|170656	-1	#DIV/0!	1	#DIV/0!
Gm20408|100126765	-1	#DIV/0!	1	#DIV/0!
LOC547349|547349	-1	#DIV/0!	1	#DIV/0!
Bpifa3|73388	-1	#DIV/0!	1	#DIV/0!
Tmed6|66269	-1	#DIV/0!	1	#DIV/0!
Vmn2r105|627743	-1	#DIV/0!	1	#DIV/0!
Nmur2|216749	-1	#DIV/0!	1	#DIV/0!
Mir15a|387174	-1	#DIV/0!	1	#DIV/0!
Olfr1502|258793	-1	#DIV/0!	1	#DIV/0!
Gm1140|382217	-1	#DIV/0!	1	#DIV/0!
1700125H20Rik|73634	-1	#DIV/0!	1	#DIV/0!
Sirpb1a|320832	-1	#DIV/0!	1	#DIV/0!
Gm10922|668964	-1	#DIV/0!	1	#DIV/0!
Btg4|56057	-1	#DIV/0!	1	#DIV/0!
Nkx6-3|74561	-1	#DIV/0!	1	#DIV/0!
Serpinb5|20724	-1	#DIV/0!	1	#DIV/0!
Mir654|100124453	-1	#DIV/0!	1	#DIV/0!
Insm2|56856	-1	#DIV/0!	1	#DIV/0!
Gpr82|319200	-1	#DIV/0!	1	#DIV/0!
Vmn2r30|22306	-1	#DIV/0!	1	#DIV/0!
Cldn17|239931	-1	#DIV/0!	1	#DIV/0!
Olfr853|258908	-1	#DIV/0!	1	#DIV/0!
Mir34b|723849	-1	#DIV/0!	1	#DIV/0!
Mir344g|100526507	-1	#DIV/0!	1	#DIV/0!
Vmn1r13|113862	-1	#DIV/0!	1	#DIV/0!
Rtbdn|234542	-1	#DIV/0!	1	#DIV/0!
Fam69c|240479	-1	#DIV/0!	1	#DIV/0!
Olfr1318|258022	-1	#DIV/0!	1	#DIV/0!
Impg1|63859	-1	#DIV/0!	1	#DIV/0!
Naip7|53880	-1	#DIV/0!	1	#DIV/0!
1700008P02Rik|69347	-1	#DIV/0!	1	#DIV/0!
Prl7b1|75596	-1	#DIV/0!	1	#DIV/0!
Gpx2-ps1|14777	-1	#DIV/0!	1	#DIV/0!
Gm8348|666890	-1	#DIV/0!	1	#DIV/0!

Olfr99|258508	-1	#DIV/0!	1	#DIV/0!
Pnmal1|71691	-1	#DIV/0!	1	#DIV/0!
Olfr726|258313	-1	#DIV/0!	1	#DIV/0!
BC027072|225004	-1	#DIV/0!	1	#DIV/0!
Gsdmcl1|74236	-1	#DIV/0!	1	#DIV/0!
Arl9|384185	-1	#DIV/0!	1	#DIV/0!
Vwa2|240675	-1	#DIV/0!	1	#DIV/0!
Dscaml1|114873	-1	#DIV/0!	1	#DIV/0!
Ptgs2|19225	-1	#DIV/0!	1	#DIV/0!
Vmn1r6|171193	-1	#DIV/0!	1	#DIV/0!
4833427G06Rik|235345	-1	#DIV/0!	1	#DIV/0!
Mir669p-2|100526538	-1	#DIV/0!	1	#DIV/0!
Smok4a|272667	-1	#DIV/0!	1	#DIV/0!
Agtr1b|11608	-1	#DIV/0!	1	#DIV/0!
Atp6v1e2|74915	-1	#DIV/0!	1	#DIV/0!
Ldlrad2|435811	-1	#DIV/0!	1	#DIV/0!
Pcdha11|12942	-1	#DIV/0!	1	#DIV/0!
Scn9a|20274	-1	#DIV/0!	1	#DIV/0!
Gm9839|408192	-1	#DIV/0!	1	#DIV/0!
Gm13023|194227	-1	#DIV/0!	1	#DIV/0!
Olfr281|258277	-1	#DIV/0!	1	#DIV/0!
Olfr1239|258972	-1	#DIV/0!	1	#DIV/0!
Dnajc5g|231098	-1	#DIV/0!	1	#DIV/0!
Vmn1r127|621561	-1	#DIV/0!	1	#DIV/0!
Mir187|387182	-1	#DIV/0!	1	#DIV/0!
Serpinb9f|20709	-1	#DIV/0!	1	#DIV/0!
Vmn2r11|384219	-1	#DIV/0!	1	#DIV/0!
Rab26|328778	-1	#DIV/0!	1	#DIV/0!
Igdcc3|19289	-1	#DIV/0!	1	#DIV/0!
Dkk1|13380	-1	#DIV/0!	1	#DIV/0!
Tsga13|116732	-1	#DIV/0!	1	#DIV/0!
Tac1|21333	-1	#DIV/0!	1	#DIV/0!
Mettl11b|240879	-1	#DIV/0!	1	#DIV/0!
Olfr615|259084	-1	#DIV/0!	1	#DIV/0!
Hes5|15208	-1	#DIV/0!	1	#DIV/0!
H1fnt|70069	-1	#DIV/0!	1	#DIV/0!
Olfr1362|258739	-1	#DIV/0!	1	#DIV/0!
Mir669m-2|100316834	-1	#DIV/0!	1	#DIV/0!
Arpp21|74100	-1	#DIV/0!	1	#DIV/0!
Mirlet7i|387251	-1	#DIV/0!	1	#DIV/0!
Gm595|209005	-1	#DIV/0!	1	#DIV/0!
Olfr1289|258399	-1	#DIV/0!	1	#DIV/0!
Vat1l|270097	-1	#DIV/0!	1	#DIV/0!
Glrb|14658	-1	#DIV/0!	1	#DIV/0!
En1|13798	-1	#DIV/0!	1	#DIV/0!
Agrp|11604	-1	#DIV/0!	1	#DIV/0!
Vmn2r42|22310	-1	#DIV/0!	1	#DIV/0!
Cck|12424	-1	#DIV/0!	1	#DIV/0!
Mir695|735287	-1	#DIV/0!	1	#DIV/0!
Gip|14607	-1	#DIV/0!	1	#DIV/0!
Mir9-1|387133	-1	#DIV/0!	1	#DIV/0!
Prm2|19119	-1	#DIV/0!	1	#DIV/0!
Tmprss11e|243084	-1	#DIV/0!	1	#DIV/0!

Vmn2r14|231591	-1	#DIV/0!	1	#DIV/0!
Sult2a6|629219	-1	#DIV/0!	1	#DIV/0!
F730016J06Rik|320194	-1	#DIV/0!	1	#DIV/0!
Olfr648|258746	-1	#DIV/0!	1	#DIV/0!
1700016H13Rik|74218	-1	#DIV/0!	1	#DIV/0!
Mir331|723908	-1	#DIV/0!	1	#DIV/0!
Tac2|21334	-1	#DIV/0!	1	#DIV/0!
Olfr851|258907	-1	#DIV/0!	1	#DIV/0!
1700123K08Rik|76658	-1	#DIV/0!	1	#DIV/0!
Fat2|245827	-1	#DIV/0!	1	#DIV/0!
Tac4|93670	-1	#DIV/0!	1	#DIV/0!
Lce1d|69611	-1	#DIV/0!	1	#DIV/0!
Gm1568|380768	-1	#DIV/0!	1	#DIV/0!
Ppapdc1a|381925	-1	#DIV/0!	1	#DIV/0!
Trhr|22045	-1	#DIV/0!	1	#DIV/0!
Gm10081|384732	-1	#DIV/0!	1	#DIV/0!
Prmt8|381813	-1	#DIV/0!	1	#DIV/0!
Lalba|16770	-1	#DIV/0!	1	#DIV/0!
Olfr344|258621	-1	#DIV/0!	1	#DIV/0!
Tas2r135|387512	-1	#DIV/0!	1	#DIV/0!
Hoxa6|15403	-1	#DIV/0!	1	#DIV/0!
Olfr582|259055	-1	#DIV/0!	1	#DIV/0!
Olfr586|259115	-1	#DIV/0!	1	#DIV/0!
Nell1|338352	-1	#DIV/0!	1	#DIV/0!
Duoxa1|213696	-1	#DIV/0!	1	#DIV/0!
Olfr553|233578	-1	#DIV/0!	1	#DIV/0!
Frmpd3|245643	-1	#DIV/0!	1	#DIV/0!
1700020N18Rik|67086	-1	#DIV/0!	1	#DIV/0!
Pla2g4e|329502	-1	#DIV/0!	1	#DIV/0!
Prdm14|383491	-1	#DIV/0!	1	#DIV/0!
Vmn1r17|171189	-1	#DIV/0!	1	#DIV/0!
EU599041|100170401	-1	#DIV/0!	1	#DIV/0!
Gm13084|381569	-1	#DIV/0!	1	#DIV/0!
Agr2|23795	-1	#DIV/0!	1	#DIV/0!
Olfr993|258427	-1	#DIV/0!	1	#DIV/0!
Olfr811|258545	-1	#DIV/0!	1	#DIV/0!
Gm1968|328657	-1	#DIV/0!	1	#DIV/0!
Serpinb3a|20248	-1	#DIV/0!	1	#DIV/0!
Mrgpra1|233221	-1	#DIV/0!	1	#DIV/0!
Olfr223|258421	-1	#DIV/0!	1	#DIV/0!
Zik1|22775	-1	#DIV/0!	1	#DIV/0!
Actl7b|11471	-1	#DIV/0!	1	#DIV/0!
Hsf3|245525	-1	#DIV/0!	1	#DIV/0!
Olfr1229|257921	-1	#DIV/0!	1	#DIV/0!
Cacna2d3|12294	-1	#DIV/0!	1	#DIV/0!
Olfr738|258662	-1	#DIV/0!	1	#DIV/0!
Snora75|100303740	-1	#DIV/0!	1	#DIV/0!
4933406M09Rik|240755	-1	#DIV/0!	1	#DIV/0!
Cdx1|12590	-1	#DIV/0!	1	#DIV/0!
Olfr420|258302	-1	#DIV/0!	1	#DIV/0!
2310002J15Rik|67859	-1	#DIV/0!	1	#DIV/0!
Gm9758|381714	-1	#DIV/0!	1	#DIV/0!
Slc30a2|230810	-1	#DIV/0!	1	#DIV/0!

Emid2|140709	-1	#DIV/0!	1	#DIV/0!
Mir421|100124494	-1	#DIV/0!	1	#DIV/0!
Htr2c|15560	-1	#DIV/0!	1	#DIV/0!
Lrrc38|242735	-1	#DIV/0!	1	#DIV/0!
Olfr1047|259014	-1	#DIV/0!	1	#DIV/0!
St6gal2|240119	-1	#DIV/0!	1	#DIV/0!
Krt9|107656	-1	#DIV/0!	1	#DIV/0!
Ifna5|15968	-1	#DIV/0!	1	#DIV/0!
Dtl|76843	-1	#DIV/0!	1	#DIV/0!
1700006A11Rik|71824	-1	#DIV/0!	1	#DIV/0!
Gm428|242502	-1	#DIV/0!	1	#DIV/0!
Zfp536|243937	-1	#DIV/0!	1	#DIV/0!
Nek10|238944	-1	#DIV/0!	1	#DIV/0!
Trim54|58522	-1	#DIV/0!	1	#DIV/0!
Mir1963|100316710	-1	#DIV/0!	1	#DIV/0!
Gm5468|432939	-1	#DIV/0!	1	#DIV/0!
Gm2016|100039042	-1	#DIV/0!	1	#DIV/0!
Hsd11b2|15484	-1	#DIV/0!	1	#DIV/0!
Ubtfl1|546118	-1	#DIV/0!	1	#DIV/0!
Olfr1367|258526	-1	#DIV/0!	1	#DIV/0!
Gm7904|666043	-1	#DIV/0!	1	#DIV/0!
4930596D02Rik|239036	-1	#DIV/0!	1	#DIV/0!
Olfr424|258716	-1	#DIV/0!	1	#DIV/0!
Tshr|22095	-1	#DIV/0!	1	#DIV/0!
Lrrtm2|107065	-1	#DIV/0!	1	#DIV/0!
Defa25|13236	-1	#DIV/0!	1	#DIV/0!
Olfr836|258557	-1	#DIV/0!	1	#DIV/0!
Cntnap5c|620292	-1	#DIV/0!	1	#DIV/0!
Artn|11876	-1	#DIV/0!	1	#DIV/0!
Dspp|666279	-1	#DIV/0!	1	#DIV/0!
Dsc3|13507	-1	#DIV/0!	1	#DIV/0!
Trh|22044	-1	#DIV/0!	1	#DIV/0!
Ugt2a1|94215	-1	#DIV/0!	1	#DIV/0!
Fcer1a|14125	-1	#DIV/0!	1	#DIV/0!
Rab42-ps|242681	-1	#DIV/0!	1	#DIV/0!
Olfr620|258808	-1	#DIV/0!	1	#DIV/0!
Pcdhb12|93883	-1	#DIV/0!	1	#DIV/0!
AU022751|102991	-1	#DIV/0!	1	#DIV/0!
Fzd10|93897	-1	#DIV/0!	1	#DIV/0!
Olfr832|258075	-1	#DIV/0!	1	#DIV/0!
Syt17|110058	-1	#DIV/0!	1	#DIV/0!
Jakmip3|74004	-1	#DIV/0!	1	#DIV/0!
Krtap5-1|50774	-1	#DIV/0!	1	#DIV/0!
Igf2bp1|140486	-1	#DIV/0!	1	#DIV/0!
Mir688|751542	-1	#DIV/0!	1	#DIV/0!
Pcdhb8|93879	-1	#DIV/0!	1	#DIV/0!
Cdh11|12552	-1	#DIV/0!	1	#DIV/0!
Tmsb15a|78478	-1	#DIV/0!	1	#DIV/0!
Tmem182|381339	-1	#DIV/0!	1	#DIV/0!
Lce3c|94060	-1	#DIV/0!	1	#DIV/0!
Olfr983|258824	-1	#DIV/0!	1	#DIV/0!
Olfr346|258940	-1	#DIV/0!	1	#DIV/0!
Mir490|735279	-1	#DIV/0!	1	#DIV/0!

Olfr1084|258235	-1	#DIV/0!	1	#DIV/0!
Serpinb1b|282663	-1	#DIV/0!	1	#DIV/0!
4930525F21Rik|75823	-1	#DIV/0!	1	#DIV/0!
Omt2b|382088	-1	#DIV/0!	1	#DIV/0!
Mir219-2|723904	-1	#DIV/0!	1	#DIV/0!
Gm7550|665239	-1	#DIV/0!	1	#DIV/0!
Tmprss11c|435845	-1	#DIV/0!	1	#DIV/0!
Krtap4-8|665992	-1	#DIV/0!	1	#DIV/0!
1700008I05Rik|71841	-1	#DIV/0!	1	#DIV/0!
Tmprss12|75002	-1	#DIV/0!	1	#DIV/0!
Sct|20287	-1	#DIV/0!	1	#DIV/0!
4933425O20Rik|66766	-1	#DIV/0!	1	#DIV/0!
Pcsk1|18548	-1	#DIV/0!	1	#DIV/0!
Gpha2|170458	-1	#DIV/0!	1	#DIV/0!
D730048I06Rik|68171	-1	#DIV/0!	1	#DIV/0!
Olfr669|259045	-1	#DIV/0!	1	#DIV/0!
Mir1960|100316709	-1	#DIV/0!	1	#DIV/0!
Six6|20476	-1	#DIV/0!	1	#DIV/0!
Vmn1r192|252907	-1	#DIV/0!	1	#DIV/0!
Acsbg2|328845	-1	#DIV/0!	1	#DIV/0!
Zscan4d|545913	-1	#DIV/0!	1	#DIV/0!
Gdap1|14545	-1	#DIV/0!	1	#DIV/0!
Pmch|110312	-1	#DIV/0!	1	#DIV/0!
Vmn2r89|22301	-1	#DIV/0!	1	#DIV/0!
6820408C15Rik|228778	-1	#DIV/0!	1	#DIV/0!
Sphkap|77629	-1	#DIV/0!	1	#DIV/0!
Nxph2|18232	-1	#DIV/0!	1	#DIV/0!
Vmn2r104|22313	-1	#DIV/0!	1	#DIV/0!
Vmn1r181|404289	-1	#DIV/0!	1	#DIV/0!
Mir125b-2|723952	-1	#DIV/0!	1	#DIV/0!
Tpbpa|21984	-1	#DIV/0!	1	#DIV/0!
Klk1b22|13646	-1	#DIV/0!	1	#DIV/0!
Tas2r129|387354	-1	#DIV/0!	1	#DIV/0!
Rhox3b|100135653	-1	#DIV/0!	1	#DIV/0!
Vmn1r211|171277	-1	#DIV/0!	1	#DIV/0!
Tas2r119|57254	-1	#DIV/0!	1	#DIV/0!
Lmx1b|16917	-1	#DIV/0!	1	#DIV/0!
Gpr85|64450	-1	#DIV/0!	1	#DIV/0!
4932429P05Rik|245509	-1	#DIV/0!	1	#DIV/0!
Gm7168|635895	-1	#DIV/0!	1	#DIV/0!
Dmrtc1c2|668357	-1	#DIV/0!	1	#DIV/0!
Taf7l|74469	-1	#DIV/0!	1	#DIV/0!
Frem3|333315	-1	#DIV/0!	1	#DIV/0!
Olfr356|258617	-1	#DIV/0!	1	#DIV/0!
Rhox3g-ps|546294	-1	#DIV/0!	1	#DIV/0!
Nkain3|269513	-1	#DIV/0!	1	#DIV/0!
Gucy2f|245650	-1	#DIV/0!	1	#DIV/0!
Lypd3|72434	-1	#DIV/0!	1	#DIV/0!
Tas2r124|387351	-1	#DIV/0!	1	#DIV/0!
Olfr374|258335	-1	#DIV/0!	1	#DIV/0!
Phox2a|11859	-1	#DIV/0!	1	#DIV/0!
Gm4371|100043335	-1	#DIV/0!	1	#DIV/0!
Pcdha9|192161	-1	#DIV/0!	1	#DIV/0!

1700084J12Rik|73486	-1	#DIV/0!	1	#DIV/0!
Slc13a1|55961	-1	#DIV/0!	1	#DIV/0!
Krt6b|16688	-1	#DIV/0!	1	#DIV/0!
Gm1604b|381059	-1	#DIV/0!	1	#DIV/0!
4832428D23Rik|403183	-1	#DIV/0!	1	#DIV/0!
Olfr603|259073	-1	#DIV/0!	1	#DIV/0!
Olfr480|56861	-1	#DIV/0!	1	#DIV/0!
Rhox12|382282	-1	#DIV/0!	1	#DIV/0!
Upk3a|22270	-1	#DIV/0!	1	#DIV/0!
Gm14316|641368	-1	#DIV/0!	1	#DIV/0!
Olfr1491|258342	-1	#DIV/0!	1	#DIV/0!
Tprg|71338	-1	#DIV/0!	1	#DIV/0!
Magea8|17144	-1	#DIV/0!	1	#DIV/0!
Gm381|214308	-1	#DIV/0!	1	#DIV/0!
Atp8b3|67331	-1	#DIV/0!	1	#DIV/0!
Kcnj4|16520	-1	#DIV/0!	1	#DIV/0!
Vmn1r60|636697	-1	#DIV/0!	1	#DIV/0!
Snora81|100217420	-1	#DIV/0!	1	#DIV/0!
Snord23|100217441	-1	#DIV/0!	1	#DIV/0!
Gucy2d|14918	-1	#DIV/0!	1	#DIV/0!
Mir129-2|723953	-1	#DIV/0!	1	#DIV/0!
Defb12|77674	-1	#DIV/0!	1	#DIV/0!
Olfr589|259054	-1	#DIV/0!	1	#DIV/0!
Klk1b24|16617	-1	#DIV/0!	1	#DIV/0!
Vip|22353	-1	#DIV/0!	1	#DIV/0!
Mir743|100049546	-1	#DIV/0!	1	#DIV/0!
Mir344c|100526506	-1	#DIV/0!	1	#DIV/0!
H2-M11|224754	-1	#DIV/0!	1	#DIV/0!
Olfr152|258640	-1	#DIV/0!	1	#DIV/0!
Olfr1249|257984	-1	#DIV/0!	1	#DIV/0!
Hhatl|74770	-1	#DIV/0!	1	#DIV/0!
Ghrh|14601	-1	#DIV/0!	1	#DIV/0!
Vmn2r3|637004	-1	#DIV/0!	1	#DIV/0!
Kncn|654462	-1	#DIV/0!	1	#DIV/0!
2310042E22Rik|66561	-1	#DIV/0!	1	#DIV/0!
Olfr1354|259163	-1	#DIV/0!	1	#DIV/0!
Pfpl|56093	-1	#DIV/0!	1	#DIV/0!
Fgf8|14179	-1	#DIV/0!	1	#DIV/0!
Htr6|15565	-1	#DIV/0!	1	#DIV/0!
Gm436|230890	-1	#DIV/0!	1	#DIV/0!
Mir133a-1|387151	-1	#DIV/0!	1	#DIV/0!
Gm4858|229571	-1	#DIV/0!	1	#DIV/0!
Olfr447|258990	-1	#DIV/0!	1	#DIV/0!
1700034O15Rik|76606	-1	#DIV/0!	1	#DIV/0!
Gm97|225923	-1	#DIV/0!	1	#DIV/0!
Olfr2|18317	-1	#DIV/0!	1	#DIV/0!
Klri2|320407	-1	#DIV/0!	1	#DIV/0!
Irx2|16372	-1	#DIV/0!	1	#DIV/0!
1700041C23Rik|67319	-1	#DIV/0!	1	#DIV/0!
Serpinb11|66957	-1	#DIV/0!	1	#DIV/0!
Clec2e|232409	-1	#DIV/0!	1	#DIV/0!
9630013A20Rik|319903	-1	#DIV/0!	1	#DIV/0!
Pla2g4d|78390	-1	#DIV/0!	1	#DIV/0!

Prph2|19133	-1	#DIV/0!	1	#DIV/0!
Epgn|71920	-1	#DIV/0!	1	#DIV/0!
Psg16|26436	-1	#DIV/0!	1	#DIV/0!
Clvs2|215890	-1	#DIV/0!	1	#DIV/0!
Krtap9-1|16705	-1	#DIV/0!	1	#DIV/0!
4933415F23Rik|66755	-1	#DIV/0!	1	#DIV/0!
Hyal4|77042	-1	#DIV/0!	1	#DIV/0!
Otop1|21906	-1	#DIV/0!	1	#DIV/0!
Klra22|93969	-1	#DIV/0!	1	#DIV/0!
4930583H14Rik|67749	-1	#DIV/0!	1	#DIV/0!
Lgsn|266744	-1	#DIV/0!	1	#DIV/0!
Dppa1|347708	-1	#DIV/0!	1	#DIV/0!
Olfr1240|258804	-1	#DIV/0!	1	#DIV/0!
Kcnh5|238271	-1	#DIV/0!	1	#DIV/0!
Mir350|723921	-1	#DIV/0!	1	#DIV/0!
Mir181c|723819	-1	#DIV/0!	1	#DIV/0!
Trnp1|69539	-1	#DIV/0!	1	#DIV/0!
Ascl2|17173	-1	#DIV/0!	1	#DIV/0!
Pdzrn4|239618	-1	#DIV/0!	1	#DIV/0!
Olfr483|258730	-1	#DIV/0!	1	#DIV/0!
Zfp92|22754	-1	#DIV/0!	1	#DIV/0!
Mir107|723826	-1	#DIV/0!	1	#DIV/0!
Olfr915|258781	-1	#DIV/0!	1	#DIV/0!
Nhlh1|18071	-1	#DIV/0!	1	#DIV/0!
Olfr794|258375	-1	#DIV/0!	1	#DIV/0!
Txndc2|213272	-1	#DIV/0!	1	#DIV/0!
Olfr338|258949	-1	#DIV/0!	1	#DIV/0!
Neurod1|18012	-1	#DIV/0!	1	#DIV/0!
Nebl|74103	-1	#DIV/0!	1	#DIV/0!
Psg28|114871	-1	#DIV/0!	1	#DIV/0!
Olfr131|258867	-1	#DIV/0!	1	#DIV/0!
Fcnb|14134	-1	#DIV/0!	1	#DIV/0!
Tmprss11d|231382	-1	#DIV/0!	1	#DIV/0!
Gm10318|622629	-1	#DIV/0!	1	#DIV/0!
Gabrg2|14406	-1	#DIV/0!	1	#DIV/0!
Iqcf5|75470	-1	#DIV/0!	1	#DIV/0!
Gm11756|623281	-1	#DIV/0!	1	#DIV/0!
Skint2|329919	-1	#DIV/0!	1	#DIV/0!
Defb10|246085	-1	#DIV/0!	1	#DIV/0!
BC051665|218275	-1	#DIV/0!	1	#DIV/0!
Srgap1|117600	-1	#DIV/0!	1	#DIV/0!
1700013D24Rik|76921	-1	#DIV/0!	1	#DIV/0!
Sec14l3|380683	-1	#DIV/0!	1	#DIV/0!
Olfr747|258264	-1	#DIV/0!	1	#DIV/0!
1700054K19Rik|67336	-1	#DIV/0!	1	#DIV/0!
Olfr616|259103	-1	#DIV/0!	1	#DIV/0!
Olfr1195|258748	-1	#DIV/0!	1	#DIV/0!
Olfr1231|258446	-1	#DIV/0!	1	#DIV/0!
Tlr8|170744	-1	#DIV/0!	1	#DIV/0!
Gsdma3|450219	-1	#DIV/0!	1	#DIV/0!
Mir340|723845	-1	#DIV/0!	1	#DIV/0!
Rgs7bp|52882	-1	#DIV/0!	1	#DIV/0!
2010107G12Rik|243753	-1	#DIV/0!	1	#DIV/0!

Grxcr2|332309	-1	#DIV/0!	1	#DIV/0!
Sgcd|24052	-1	#DIV/0!	1	#DIV/0!
Cga|12640	-1	#DIV/0!	1	#DIV/0!
2900092D14Rik|73100	-1	#DIV/0!	1	#DIV/0!
Olfr521|258353	-1	#DIV/0!	1	#DIV/0!
Gbx1|231044	-1	#DIV/0!	1	#DIV/0!
Gpr176|381413	-1	#DIV/0!	1	#DIV/0!
Cts7|56092	-1	#DIV/0!	1	#DIV/0!
Olfr448|258270	-1	#DIV/0!	1	#DIV/0!
Rhox13|73614	-1	#DIV/0!	1	#DIV/0!
Krtap11-1|16693	-1	#DIV/0!	1	#DIV/0!
Mir344|723931	-1	#DIV/0!	1	#DIV/0!
Ifna13|230396	-1	#DIV/0!	1	#DIV/0!
Pcdhgb7|93704	-1	#DIV/0!	1	#DIV/0!
Olfr354|258941	-1	#DIV/0!	1	#DIV/0!
Gphb5|217674	-1	#DIV/0!	1	#DIV/0!
Fam19a4|320701	-1	#DIV/0!	1	#DIV/0!
Mir185|387180	-1	#DIV/0!	1	#DIV/0!
Gm8300|666806	-1	#DIV/0!	1	#DIV/0!
Slc26a4|23985	-1	#DIV/0!	1	#DIV/0!
Slc25a31|73333	-1	#DIV/0!	1	#DIV/0!
Olfr1238|258786	-1	#DIV/0!	1	#DIV/0!
Krtap5-5|114666	-1	#DIV/0!	1	#DIV/0!
Scube3|268935	-1	#DIV/0!	1	#DIV/0!
Fbxo40|207215	-1	#DIV/0!	1	#DIV/0!
Olfr90|258469	-1	#DIV/0!	1	#DIV/0!
Osta|106407	-1	#DIV/0!	1	#DIV/0!
Pramel7|347712	-1	#DIV/0!	1	#DIV/0!
Serpinb9e|20710	-1	#DIV/0!	1	#DIV/0!
Olfr692|258352	-1	#DIV/0!	1	#DIV/0!
Dlx2|13392	-1	#DIV/0!	1	#DIV/0!
Fut4-ps1|14346	-1	#DIV/0!	1	#DIV/0!
Olfr394|259009	-1	#DIV/0!	1	#DIV/0!
Ghsr|208188	-1	#DIV/0!	1	#DIV/0!
Mir34c|723932	-1	#DIV/0!	1	#DIV/0!
Hepacam2|101202	-1	#DIV/0!	1	#DIV/0!
Gm14092|627302	-1	#DIV/0!	1	#DIV/0!
Olfr791|258932	-1	#DIV/0!	1	#DIV/0!
Srd5a2|94224	-1	#DIV/0!	1	#DIV/0!
Ccdc113|244608	-1	#DIV/0!	1	#DIV/0!
Htr3b|57014	-1	#DIV/0!	1	#DIV/0!
9230102K24Rik|109335	-1	#DIV/0!	1	#DIV/0!
Mir3101|100526505	-1	#DIV/0!	1	#DIV/0!
Gm4776|212225	-1	#DIV/0!	1	#DIV/0!
Trap1a|22037	-1	#DIV/0!	1	#DIV/0!
Mir495|751522	-1	#DIV/0!	1	#DIV/0!
AI504432|229694	-1	#DIV/0!	1	#DIV/0!
Mlana|77836	-1	#DIV/0!	1	#DIV/0!
Mir204|387200	-1	#DIV/0!	1	#DIV/0!
Adam26b|382007	-1	#DIV/0!	1	#DIV/0!
Olfr1113|404327	-1	#DIV/0!	1	#DIV/0!
Olfr741|258233	-1	#DIV/0!	1	#DIV/0!
Gm15308|100041890	-1	#DIV/0!	1	#DIV/0!

4933407P14Rik|237958	-1	#DIV/0!	1	#DIV/0!
Gm7157|635396	-1	#DIV/0!	1	#DIV/0!
Prl8a8|74188	-1	#DIV/0!	1	#DIV/0!
Defb26|654457	-1	#DIV/0!	1	#DIV/0!
Wfdc5|209232	-1	#DIV/0!	1	#DIV/0!
Mir1188|100316669	-1	#DIV/0!	1	#DIV/0!
Ttc9b|73032	-1	#DIV/0!	1	#DIV/0!
Olfr786|258542	-1	#DIV/0!	1	#DIV/0!
Otp|18420	-1	#DIV/0!	1	#DIV/0!
Kif27|75050	-1	#DIV/0!	1	#DIV/0!
Dmp1|13406	-1	#DIV/0!	1	#DIV/0!
Bmp3|110075	-1	#DIV/0!	1	#DIV/0!
2300002M23Rik|69542	-1	#DIV/0!	1	#DIV/0!
Abpe|381970	-1	#DIV/0!	1	#DIV/0!
Cartpt|27220	-1	#DIV/0!	1	#DIV/0!
Krtap12-1|16694	-1	#DIV/0!	1	#DIV/0!
Gabra2|14395	-1	#DIV/0!	1	#DIV/0!
Gzme|14942	-1	#DIV/0!	1	#DIV/0!
Ucn2|171530	-1	#DIV/0!	1	#DIV/0!
B3galt5|93961	-1	#DIV/0!	1	#DIV/0!
Pramef17|626943	-1	#DIV/0!	1	#DIV/0!
Slc22a13|102570	-1	#DIV/0!	1	#DIV/0!
Olfr724|258485	-1	#DIV/0!	1	#DIV/0!
Acrv1|11451	-1	#DIV/0!	1	#DIV/0!
Ppp1r27|68701	-1	#DIV/0!	1	#DIV/0!
Vmn1r172|81010	-1	#DIV/0!	1	#DIV/0!
Olfr477|258928	-1	#DIV/0!	1	#DIV/0!
Gpr62|436090	-1	#DIV/0!	1	#DIV/0!
Mir3475|100499512	-1	#DIV/0!	1	#DIV/0!
Gm6878|628416	-1	#DIV/0!	1	#DIV/0!
1700044K03Rik|67321	-1	#DIV/0!	1	#DIV/0!
Gm6042|574415	-1	#DIV/0!	1	#DIV/0!
Olfr181|259001	-1	#DIV/0!	1	#DIV/0!
Mmp20|30800	-1	#DIV/0!	1	#DIV/0!
Vmn1r175|622222	-1	#DIV/0!	1	#DIV/0!
Vmn2r44|434113	-1	#DIV/0!	1	#DIV/0!
Cd200r2|271375	-1	#DIV/0!	1	#DIV/0!
Ccdc146|75172	-1	#DIV/0!	1	#DIV/0!
Prokr2|246313	-1	#DIV/0!	1	#DIV/0!
Olfr805|258548	-1	#DIV/0!	1	#DIV/0!
Wbscr28|76629	-1	#DIV/0!	1	#DIV/0!
Atp2b3|320707	-1	#DIV/0!	1	#DIV/0!
Vmn2r71|233445	-1	#DIV/0!	1	#DIV/0!
Glipr1l2|67537	-1	#DIV/0!	1	#DIV/0!
Fbll1|237730	-1	#DIV/0!	1	#DIV/0!
Pinc|723792	-1	#DIV/0!	1	#DIV/0!
Gabrb2|14401	-1	#DIV/0!	1	#DIV/0!
Snord58b|100217457	-1	#DIV/0!	1	#DIV/0!
1700058G18Rik|67342	-1	#DIV/0!	1	#DIV/0!
Art5|11875	-1	#DIV/0!	1	#DIV/0!
Krt6a|16687	-1	#DIV/0!	1	#DIV/0!
Prss39|21755	-1	#DIV/0!	1	#DIV/0!
Ces5a|67935	-1	#DIV/0!	1	#DIV/0!

Izumo1|73456	-1	#DIV/0!	1	#DIV/0!
Fpr-rs4|14291	-1	#DIV/0!	1	#DIV/0!
Cabp5|29865	-1	#DIV/0!	1	#DIV/0!
Myo3a|667663	-1	#DIV/0!	1	#DIV/0!
Gm813|328695	-1	#DIV/0!	1	#DIV/0!
Olfr820|258670	-1	#DIV/0!	1	#DIV/0!
Defb38|360212	-1	#DIV/0!	1	#DIV/0!
Mir365-1|723899	-1	#DIV/0!	1	#DIV/0!
Gm9125|668359	-1	#DIV/0!	1	#DIV/0!
Lcn9|77704	-1	#DIV/0!	1	#DIV/0!
Olfr813|258252	-1	#DIV/0!	1	#DIV/0!
Mir135a-2|723955	-1	#DIV/0!	1	#DIV/0!
Npffr2|104443	-1	#DIV/0!	1	#DIV/0!
Vax2os2|545859	-1	#DIV/0!	1	#DIV/0!
Pcdha4|12936	-1	#DIV/0!	1	#DIV/0!
Vmn1r206|171250	-1	#DIV/0!	1	#DIV/0!
Noto|384452	-1	#DIV/0!	1	#DIV/0!
Casr|12374	-1	#DIV/0!	1	#DIV/0!
Has2as|594843	-1	#DIV/0!	1	#DIV/0!
5031426D15Rik|68144	-1	#DIV/0!	1	#DIV/0!
Vmn2r45|100042810	-1	#DIV/0!	1	#DIV/0!
Fbxw16|320083	-1	#DIV/0!	1	#DIV/0!
Ankrd29|225187	-1	#DIV/0!	1	#DIV/0!
Defb6|116746	-1	#DIV/0!	1	#DIV/0!
Olfr1389|259069	-1	#DIV/0!	1	#DIV/0!
Hes3|15207	-1	#DIV/0!	1	#DIV/0!
Acan|11595	-1	#DIV/0!	1	#DIV/0!
9530002B09Rik|77432	-1	#DIV/0!	1	#DIV/0!
Klk12|69511	-1	#DIV/0!	1	#DIV/0!
Arr3|170735	-1	#DIV/0!	1	#DIV/0!
Gm11563|100040248	-1	#DIV/0!	1	#DIV/0!
Gm12169|210535	-1	#DIV/0!	1	#DIV/0!
Tsx|22127	-1	#DIV/0!	1	#DIV/0!
Lipf|67717	-1	#DIV/0!	1	#DIV/0!
Ror2|26564	-1	#DIV/0!	1	#DIV/0!
Sox21|223227	-1	#DIV/0!	1	#DIV/0!
Olfr308|258614	-1	#DIV/0!	1	#DIV/0!
Mir3091|100526556	-1	#DIV/0!	1	#DIV/0!
Krtap10-10|544710	-1	#DIV/0!	1	#DIV/0!
Pnma3|245468	-1	#DIV/0!	1	#DIV/0!
Gm17689|100312948	-1	#DIV/0!	1	#DIV/0!
Klra3|16634	-1	#DIV/0!	1	#DIV/0!
Hist4h4|320332	-1	#DIV/0!	1	#DIV/0!
Vmn1r236|171235	-1	#DIV/0!	1	#DIV/0!
Gp2|67133	-1	#DIV/0!	1	#DIV/0!
Olfr1532-ps1|258173	-1	#DIV/0!	1	#DIV/0!
Eras|353283	-1	#DIV/0!	1	#DIV/0!
Smgc|223809	-1	#DIV/0!	1	#DIV/0!
Glra1|14654	-1	#DIV/0!	1	#DIV/0!
Dusp15|252864	-1	#DIV/0!	1	#DIV/0!
Olfr1301|258889	-1	#DIV/0!	1	#DIV/0!
Trim55|381485	-1	#DIV/0!	1	#DIV/0!
Prl3b1|18776	-1	#DIV/0!	1	#DIV/0!

2010005H15Rik|76770	-1	#DIV/0!	1	#DIV/0!
Olfr1511|258268	-1	#DIV/0!	1	#DIV/0!
Vmn1r126|100043013	-1	#DIV/0!	1	#DIV/0!
Defb23|629114	-1	#DIV/0!	1	#DIV/0!
Tnfsf18|240873	-1	#DIV/0!	1	#DIV/0!
Prss1|114228	-1	#DIV/0!	1	#DIV/0!
Gm6460|623898	-1	#DIV/0!	1	#DIV/0!
Tmem63c|217733	-1	#DIV/0!	1	#DIV/0!
C87414|381654	-1	#DIV/0!	1	#DIV/0!
Mir16-2|723949	-1	#DIV/0!	1	#DIV/0!
Olfr214|258754	-1	#DIV/0!	1	#DIV/0!
Olfr730|258486	-1	#DIV/0!	1	#DIV/0!
Igsf9b|235086	-1	#DIV/0!	1	#DIV/0!
4931409K22Rik|231045	-1	#DIV/0!	1	#DIV/0!
2310005G13Rik|69457	-1	#DIV/0!	1	#DIV/0!
Vmn2r90|626942	-1	#DIV/0!	1	#DIV/0!
Tas2r130|387355	-1	#DIV/0!	1	#DIV/0!
Krtap5-4|50775	-1	#DIV/0!	1	#DIV/0!
1700121N20Rik|76639	-1	#DIV/0!	1	#DIV/0!
Atp6v1c2|68775	-1	#DIV/0!	1	#DIV/0!
Olfr632|259123	-1	#DIV/0!	1	#DIV/0!
Gm5105|329763	-1	#DIV/0!	1	#DIV/0!
2310033E01Rik|641361	-1	#DIV/0!	1	#DIV/0!
Sez6|20370	-1	#DIV/0!	1	#DIV/0!
Myod1|17927	-1	#DIV/0!	1	#DIV/0!
4933405L10Rik|71046	-1	#DIV/0!	1	#DIV/0!
Iqcf6|100041096	-1	#DIV/0!	1	#DIV/0!
Tdrd9|74691	-1	#DIV/0!	1	#DIV/0!
Slc12a1|20495	-1	#DIV/0!	1	#DIV/0!
Olfr885|257885	-1	#DIV/0!	1	#DIV/0!
Olfr382|258435	-1	#DIV/0!	1	#DIV/0!
Olfr933|258433	-1	#DIV/0!	1	#DIV/0!
1110032A04Rik|66183	-1	#DIV/0!	1	#DIV/0!
Olfr143|258802	-1	#DIV/0!	1	#DIV/0!
1700012B07Rik|69324	-1	#DIV/0!	1	#DIV/0!
Stfa2l1|268885	-1	#DIV/0!	1	#DIV/0!
Mir717|751531	-1	#DIV/0!	1	#DIV/0!
Snord1a|100216538	-1	#DIV/0!	1	#DIV/0!
Prl7d1|18814	-1	#DIV/0!	1	#DIV/0!
1700029H14Rik|66501	-1	#DIV/0!	1	#DIV/0!
4930521A18Rik|74708	-1	#DIV/0!	1	#DIV/0!
Mir687|751541	-1	#DIV/0!	1	#DIV/0!
Gprc5d|93746	-1	#DIV/0!	1	#DIV/0!
Prss48|368202	-1	#DIV/0!	1	#DIV/0!
Itgad|381924	-1	#DIV/0!	1	#DIV/0!
1700016D06Rik|76413	-1	#DIV/0!	1	#DIV/0!
4921524L21Rik|70901	-1	#DIV/0!	1	#DIV/0!
Kcnd2|16508	-1	#DIV/0!	1	#DIV/0!
Fbxo15|50764	-1	#DIV/0!	1	#DIV/0!
Rax|19434	-1	#DIV/0!	1	#DIV/0!
Kctd8|243043	-1	#DIV/0!	1	#DIV/0!
Olfr935|258741	-1	#DIV/0!	1	#DIV/0!
Gm10400|100093700	-1	#DIV/0!	1	#DIV/0!

Olfr159|29849	-1	#DIV/0!	1	#DIV/0!
Mir802|791074	-1	#DIV/0!	1	#DIV/0!
Olfr305|258609	-1	#DIV/0!	1	#DIV/0!
Olfr142|406186	-1	#DIV/0!	1	#DIV/0!
Olfr1135|258654	-1	#DIV/0!	1	#DIV/0!
Mobp|17433	-1	#DIV/0!	1	#DIV/0!
Sfrp2|20319	-1	#DIV/0!	1	#DIV/0!
BC049352|408059	-1	#DIV/0!	1	#DIV/0!
Prl3d3|215029	-1	#DIV/0!	1	#DIV/0!
Gm13749|433315	-1	#DIV/0!	1	#DIV/0!
Zfp345|545471	-1	#DIV/0!	1	#DIV/0!
S100z|268686	-1	#DIV/0!	1	#DIV/0!
Mir125b-1|387236	-1	#DIV/0!	1	#DIV/0!
Srsf12|272009	-1	#DIV/0!	1	#DIV/0!
Mir1942|100316774	-1	#DIV/0!	1	#DIV/0!
Olfr558|259097	-1	#DIV/0!	1	#DIV/0!
Olfr350|258620	-1	#DIV/0!	1	#DIV/0!
Hoxa11|15396	-1	#DIV/0!	1	#DIV/0!
Mir679|751539	-1	#DIV/0!	1	#DIV/0!
Olfr1204|258455	-1	#DIV/0!	1	#DIV/0!
Pax6|18508	-1	#DIV/0!	1	#DIV/0!
Vmn2r73|620928	-1	#DIV/0!	1	#DIV/0!
Rasgef1c|74563	-1	#DIV/0!	1	#DIV/0!
Pip5kl1|227733	-1	#DIV/0!	1	#DIV/0!
Ccdc129|232016	-1	#DIV/0!	1	#DIV/0!
Vmn1r137|100043614	-1	#DIV/0!	1	#DIV/0!
Olfr617|258838	-1	#DIV/0!	1	#DIV/0!
Pax4|18506	-1	#DIV/0!	1	#DIV/0!
Apol10b|328561	-1	#DIV/0!	1	#DIV/0!
Kremen2|73016	-1	#DIV/0!	1	#DIV/0!
Tdpoz4|399675	-1	#DIV/0!	1	#DIV/0!
Gm1973|100038846	-1	#DIV/0!	1	#DIV/0!
Prss34|328780	-1	#DIV/0!	1	#DIV/0!
1700012A03Rik|76382	-1	#DIV/0!	1	#DIV/0!
Olfr1413|259039	-1	#DIV/0!	1	#DIV/0!
Snora33|100529074	-1	#DIV/0!	1	#DIV/0!
Ccdc155|384619	-1	#DIV/0!	1	#DIV/0!
Mir3110|100526512	-1	#DIV/0!	1	#DIV/0!
Wisp3|327743	-1	#DIV/0!	1	#DIV/0!
Slc5a7|63993	-1	#DIV/0!	1	#DIV/0!
Glt6d1|71103	-1	#DIV/0!	1	#DIV/0!
P2rx2|231602	-1	#DIV/0!	1	#DIV/0!
Spesp1|66712	-1	#DIV/0!	1	#DIV/0!
Add2|11519	-1	#DIV/0!	1	#DIV/0!
Olfr988|258166	-1	#DIV/0!	1	#DIV/0!
Ifnk|387510	-1	#DIV/0!	1	#DIV/0!
Mir1902|100316678	-1	#DIV/0!	1	#DIV/0!
Oas1h|246729	-1	#DIV/0!	1	#DIV/0!
Olfr557|258358	-1	#DIV/0!	1	#DIV/0!
Col9a1|12839	-1	#DIV/0!	1	#DIV/0!
Mir34a|723848	-1	#DIV/0!	1	#DIV/0!
Olfr877|258412	-1	#DIV/0!	1	#DIV/0!
Kcnn3|140493	-1	#DIV/0!	1	#DIV/0!

Mill1|266815	-1	#DIV/0!	1	#DIV/0!
Mir344d-2|100526531	-1	#DIV/0!	1	#DIV/0!
Olfr1080|258404	-1	#DIV/0!	1	#DIV/0!
Mir499|735275	-1	#DIV/0!	1	#DIV/0!
Myl7|17898	-1	#DIV/0!	1	#DIV/0!
Vmn1r35|171185	-1	#DIV/0!	1	#DIV/0!
Nme5|75533	-1	#DIV/0!	1	#DIV/0!
Olfr979|259112	-1	#DIV/0!	1	#DIV/0!
Lrrc23|16977	-1	#DIV/0!	1	#DIV/0!
Htr4|15562	-1	#DIV/0!	1	#DIV/0!
Fam59b|242915	-1	#DIV/0!	1	#DIV/0!
Xlr5a|574438	-1	#DIV/0!	1	#DIV/0!
Olfr1246|258788	-1	#DIV/0!	1	#DIV/0!
Golga7b|71146	-1	#DIV/0!	1	#DIV/0!
Olfr628|259159	-1	#DIV/0!	1	#DIV/0!
Wfdc9|629754	-1	#DIV/0!	1	#DIV/0!
Agbl1|244071	-1	#DIV/0!	1	#DIV/0!
Frmpd1|666060	-1	#DIV/0!	1	#DIV/0!
Olfr257|404314	-1	#DIV/0!	1	#DIV/0!
Olfr744|257884	-1	#DIV/0!	1	#DIV/0!
Hist2h2ac|319176	-1	#DIV/0!	1	#DIV/0!
4933402J07Rik|330820	-1	#DIV/0!	1	#DIV/0!
Myh2|17882	-1	#DIV/0!	1	#DIV/0!
Gm6592|625480	-1	#DIV/0!	1	#DIV/0!
1700013F07Rik|75504	-1	#DIV/0!	1	#DIV/0!
Pitx1|18740	-1	#DIV/0!	1	#DIV/0!
Serpinb6e|435350	-1	#DIV/0!	1	#DIV/0!
Gm11938|100041412	-1	#DIV/0!	1	#DIV/0!
Pou3f2|18992	-1	#DIV/0!	1	#DIV/0!
Gm20594|100463512	-1	#DIV/0!	1	#DIV/0!
Olfr298|257905	-1	#DIV/0!	1	#DIV/0!
Mir874|100124491	-1	#DIV/0!	1	#DIV/0!
Cst7|13011	-1	#DIV/0!	1	#DIV/0!
Defb46|574081	-1	#DIV/0!	1	#DIV/0!
Pip|18716	-1	#DIV/0!	1	#DIV/0!
Nmur1|14767	-1	#DIV/0!	1	#DIV/0!
Gm8660|667485	-1	#DIV/0!	1	#DIV/0!
Vmn1r26|171190	-1	#DIV/0!	1	#DIV/0!
Snora74a|436583	-1	#DIV/0!	1	#DIV/0!
Vmn2r106|224576	-1	#DIV/0!	1	#DIV/0!
Snora47|100217450	-1	#DIV/0!	1	#DIV/0!
Gapdhs|14447	-1	#DIV/0!	1	#DIV/0!
Gm10324|628709	-1	#DIV/0!	1	#DIV/0!
4930544G11Rik|67653	-1	#DIV/0!	1	#DIV/0!
Mir1943|100316695	-1	#DIV/0!	1	#DIV/0!
Vmn1r177|384572	-1	#DIV/0!	1	#DIV/0!
Olfr1283|228443	-1	#DIV/0!	1	#DIV/0!
Vmn1r183|209824	-1	#DIV/0!	1	#DIV/0!
Mir128-2|723815	-1	#DIV/0!	1	#DIV/0!
Gm10421|100038689	-1	#DIV/0!	1	#DIV/0!
Kcnv1|67498	-1	#DIV/0!	1	#DIV/0!
Ctnna3|216033	-1	#DIV/0!	1	#DIV/0!
Mir692-1|751529	-1	#DIV/0!	1	#DIV/0!

Pcdhb13|93884	-1	#DIV/0!	1	#DIV/0!
Bhlhe41|79362	-1	#DIV/0!	1	#DIV/0!
Gm5891|545929	-1	#DIV/0!	1	#DIV/0!
1700011F14Rik|75645	-1	#DIV/0!	1	#DIV/0!
4930417G10Rik|74855	-1	#DIV/0!	1	#DIV/0!
Zfp735|76390	-1	#DIV/0!	1	#DIV/0!
Olfr578|259119	-1	#DIV/0!	1	#DIV/0!
Mir532|751544	-1	#DIV/0!	1	#DIV/0!
Gpr37|14763	-1	#DIV/0!	1	#DIV/0!
Olfr1202|258454	-1	#DIV/0!	1	#DIV/0!
Scgb3a2|117158	-1	#DIV/0!	1	#DIV/0!
Ica1l|70375	-1	#DIV/0!	1	#DIV/0!
Olfr1274-ps|258330	-1	#DIV/0!	1	#DIV/0!
Cntnap2|66797	-1	#DIV/0!	1	#DIV/0!
Hsd17b3|15487	-1	#DIV/0!	1	#DIV/0!
Plac1l|225922	-1	#DIV/0!	1	#DIV/0!
Tmem233|545798	-1	#DIV/0!	1	#DIV/0!
Gdnf|14573	-1	#DIV/0!	1	#DIV/0!
Vmn1r125|667215	-1	#DIV/0!	1	#DIV/0!
Gm13103|194225	-1	#DIV/0!	1	#DIV/0!
1700060C20Rik|73399	-1	#DIV/0!	1	#DIV/0!
Rad21l|668929	-1	#DIV/0!	1	#DIV/0!
Mir192|387187	-1	#DIV/0!	1	#DIV/0!
Irgc1|210145	-1	#DIV/0!	1	#DIV/0!
Ptprz1|19283	-1	#DIV/0!	1	#DIV/0!
Olfr611|258722	-1	#DIV/0!	1	#DIV/0!
Ang5|503844	-1	#DIV/0!	1	#DIV/0!
A630073D07Rik|381819	-1	#DIV/0!	1	#DIV/0!
Gm9962|791383	-1	#DIV/0!	1	#DIV/0!
Tmem225|75667	-1	#DIV/0!	1	#DIV/0!
Rcvrn|19674	-1	#DIV/0!	1	#DIV/0!
Cryba2|12958	-1	#DIV/0!	1	#DIV/0!
Trim43a|547109	-1	#DIV/0!	1	#DIV/0!
Ssxb9|387131	-1	#DIV/0!	1	#DIV/0!
Mir18|387135	-1	#DIV/0!	1	#DIV/0!
1110059M19Rik|68800	-1	#DIV/0!	1	#DIV/0!
Trcg1|541610	-1	#DIV/0!	1	#DIV/0!
Gcm2|107889	-1	#DIV/0!	1	#DIV/0!
Krt82|114566	-1	#DIV/0!	1	#DIV/0!
BC061194|381350	-1	#DIV/0!	1	#DIV/0!
Ooep|67968	-1	#DIV/0!	1	#DIV/0!
Klrc2|16642	-1	#DIV/0!	1	#DIV/0!
Mir1907|100316685	-1	#DIV/0!	1	#DIV/0!
Adam5|11499	-1	#DIV/0!	1	#DIV/0!
Mir150|387168	-1	#DIV/0!	1	#DIV/0!
Prl2b1|66392	-1	#DIV/0!	1	#DIV/0!
Mir181b-2|723903	-1	#DIV/0!	1	#DIV/0!
Mir3087|100526555	-1	#DIV/0!	1	#DIV/0!
AF366264|231201	-1	#DIV/0!	1	#DIV/0!
Lzts1|211134	-1	#DIV/0!	1	#DIV/0!
Olfr1381|258461	-1	#DIV/0!	1	#DIV/0!
Fbxw21|320082	-1	#DIV/0!	1	#DIV/0!
Defa-ps12|654452	-1	#DIV/0!	1	#DIV/0!

Mir194-1|387189	-1	#DIV/0!	1	#DIV/0!
Spef2|320277	-1	#DIV/0!	1	#DIV/0!
Brs3|12209	-1	#DIV/0!	1	#DIV/0!
Dsg3|13512	-1	#DIV/0!	1	#DIV/0!
Vmn1r187|100039499	-1	#DIV/0!	1	#DIV/0!
Palm3|74337	-1	#DIV/0!	1	#DIV/0!
Gm6583|625424	-1	#DIV/0!	1	#DIV/0!
Prl2c3|18812	-1	#DIV/0!	1	#DIV/0!
4932443I19Rik|403185	-1	#DIV/0!	1	#DIV/0!
Ucn|22226	-1	#DIV/0!	1	#DIV/0!
4930529M08Rik|78774	-1	#DIV/0!	1	#DIV/0!
Gm15127|434866	-1	#DIV/0!	1	#DIV/0!
Mir487b|723940	-1	#DIV/0!	1	#DIV/0!
Dbh|13166	-1	#DIV/0!	1	#DIV/0!
Cplx1|12889	-1	#DIV/0!	1	#DIV/0!
Vmn1r29|113859	-1	#DIV/0!	1	#DIV/0!
Calm4|80796	-1	#DIV/0!	1	#DIV/0!
Xlr4c|72891	-1	#DIV/0!	1	#DIV/0!
Ly6g6f|433099	-1	#DIV/0!	1	#DIV/0!
Kcnk18|332396	-1	#DIV/0!	1	#DIV/0!
Olfr812|258791	-1	#DIV/0!	1	#DIV/0!
Mir494|723878	-1	#DIV/0!	1	#DIV/0!
Gm20410|100126773	-1	#DIV/0!	1	#DIV/0!
Klra12|16630	-1	#DIV/0!	1	#DIV/0!
Olfr1206|258896	-1	#DIV/0!	1	#DIV/0!
Zfp616|327963	-1	#DIV/0!	1	#DIV/0!
Calcb|116903	-1	#DIV/0!	1	#DIV/0!
Snord91a|100217469	-1	#DIV/0!	1	#DIV/0!
Hapln3|67666	-1	#DIV/0!	1	#DIV/0!
Catsper1|225865	-1	#DIV/0!	1	#DIV/0!
Svop|68666	-1	#DIV/0!	1	#DIV/0!
Trim40|195359	-1	#DIV/0!	1	#DIV/0!
Il20|58181	-1	#DIV/0!	1	#DIV/0!
Mrgprb1|233231	-1	#DIV/0!	1	#DIV/0!
Tmprss11g|320454	-1	#DIV/0!	1	#DIV/0!
Gm10697|100042761	-1	#DIV/0!	1	#DIV/0!
Klf5|12224	-1	#DIV/0!	1	#DIV/0!
Mir1930|100316811	-1	#DIV/0!	1	#DIV/0!
Cd300c|387565	-1	#DIV/0!	1	#DIV/0!
Kcnip4|80334	-1	#DIV/0!	1	#DIV/0!
Slitrk3|386750	-1	#DIV/0!	1	#DIV/0!
Rfpl4|192658	-1	#DIV/0!	1	#DIV/0!
Mir322|723907	-1	#DIV/0!	1	#DIV/0!
Hecw1|94253	-1	#DIV/0!	1	#DIV/0!
Gpr149|229357	-1	#DIV/0!	1	#DIV/0!
Olfr1447|258698	-1	#DIV/0!	1	#DIV/0!
Xpnpep2|170745	-1	#DIV/0!	1	#DIV/0!
Gucy2e|14919	-1	#DIV/0!	1	#DIV/0!
Gm6812|627927	-1	#DIV/0!	1	#DIV/0!
Olfr1366|258280	-1	#DIV/0!	1	#DIV/0!
Zfp239|22685	-1	#DIV/0!	1	#DIV/0!
Olfr1282|258909	-1	#DIV/0!	1	#DIV/0!
Prlh|623503	-1	#DIV/0!	1	#DIV/0!

Hils1|54388	-1	#DIV/0!	1	#DIV/0!
Gm382|211208	-1	#DIV/0!	1	#DIV/0!
Olfr508|258769	-1	#DIV/0!	1	#DIV/0!
Pcdhga10|93722	-1	#DIV/0!	1	#DIV/0!
Npvf|60531	-1	#DIV/0!	1	#DIV/0!
Krt78|332131	-1	#DIV/0!	1	#DIV/0!
Sema3e|20349	-1	#DIV/0!	1	#DIV/0!
Gm106|226866	-1	#DIV/0!	1	#DIV/0!
Mir181a-2|387176	-1	#DIV/0!	1	#DIV/0!
Calml3|70405	-1	#DIV/0!	1	#DIV/0!
Otud6a|408193	-1	#DIV/0!	1	#DIV/0!
Tbata|65971	-1	#DIV/0!	1	#DIV/0!
Olfr713|259036	-1	#DIV/0!	1	#DIV/0!
Olfr115|257908	-1	#DIV/0!	1	#DIV/0!
Sgol2|68549	-1	#DIV/0!	1	#DIV/0!
Pramel6|347711	-1	#DIV/0!	1	#DIV/0!
Vmn1r232|171227	-1	#DIV/0!	1	#DIV/0!
Olfr570|259114	-1	#DIV/0!	1	#DIV/0!
Slitrk6|239250	-1	#DIV/0!	1	#DIV/0!
Olfr1501|258626	-1	#DIV/0!	1	#DIV/0!
4922501L14Rik|209601	-1	#DIV/0!	1	#DIV/0!
Cadps|27062	-1	#DIV/0!	1	#DIV/0!
Olfr944|258500	-1	#DIV/0!	1	#DIV/0!
Olfr1220|258902	-1	#DIV/0!	1	#DIV/0!
Gm6164|620574	-1	#DIV/0!	1	#DIV/0!
C77370|245555	-1	#DIV/0!	1	#DIV/0!
Mir467a-3|100526484	-1	#DIV/0!	1	#DIV/0!
Olfr222|257962	-1	#DIV/0!	1	#DIV/0!
Zg16|69036	-1	#DIV/0!	1	#DIV/0!
Mrgprb3|404238	-1	#DIV/0!	1	#DIV/0!
Slc6a18|22598	-1	#DIV/0!	1	#DIV/0!
Slc5a10|109342	-1	#DIV/0!	1	#DIV/0!
Rbpjl|19668	-1	#DIV/0!	1	#DIV/0!
Klra8|16639	-1	#DIV/0!	1	#DIV/0!
Atp13a4|224079	-1	#DIV/0!	1	#DIV/0!
Gzmk|14945	-1	#DIV/0!	1	#DIV/0!
Alx3|11694	-1	#DIV/0!	1	#DIV/0!
Snord110|100217452	-1	#DIV/0!	1	#DIV/0!
Ptx4|68509	-1	#DIV/0!	1	#DIV/0!
Fignl1|60530	-1	#DIV/0!	1	#DIV/0!
Gm7134|634340	-1	#DIV/0!	1	#DIV/0!
Krt25|70810	-1	#DIV/0!	1	#DIV/0!
Olfr1450|258368	-1	#DIV/0!	1	#DIV/0!
Olfr1076|258401	-1	#DIV/0!	1	#DIV/0!
Evx2|14029	-1	#DIV/0!	1	#DIV/0!
Khdrbs2|170771	-1	#DIV/0!	1	#DIV/0!
Gm13177|435815	-1	#DIV/0!	1	#DIV/0!
1700080E11Rik|73532	-1	#DIV/0!	1	#DIV/0!
Lrfn2|70530	-1	#DIV/0!	1	#DIV/0!
Rxfp3|239336	-1	#DIV/0!	1	#DIV/0!
Plscr5|331000	-1	#DIV/0!	1	#DIV/0!
Kirrel2|243911	-1	#DIV/0!	1	#DIV/0!
Mdh1b|76668	-1	#DIV/0!	1	#DIV/0!

Ins1|16333	-1	#DIV/0!	1	#DIV/0!
Olfr1016|257915	-1	#DIV/0!	1	#DIV/0!
1600015I10Rik|69761	-1	#DIV/0!	1	#DIV/0!
Apol10a|245282	-1	#DIV/0!	1	#DIV/0!
4933402E13Rik|74437	-1	#DIV/0!	1	#DIV/0!
Hist1h2bk|319184	-1	#DIV/0!	1	#DIV/0!
Mir674|732489	-1	#DIV/0!	1	#DIV/0!
1700019N19Rik|67507	-1	#DIV/0!	1	#DIV/0!
Defb18|654460	-1	#DIV/0!	1	#DIV/0!
Olfr411|258704	-1	#DIV/0!	1	#DIV/0!
Olfr878|258794	-1	#DIV/0!	1	#DIV/0!
Olfr91|258470	-1	#DIV/0!	1	#DIV/0!
Gm11468|670775	-1	#DIV/0!	1	#DIV/0!
4930474M22Rik|74917	-1	#DIV/0!	1	#DIV/0!
Klhl33|546611	-1	#DIV/0!	1	#DIV/0!
4930511M11Rik|75010	-1	#DIV/0!	1	#DIV/0!
Mir130a|387149	-1	#DIV/0!	1	#DIV/0!
Ip6k3|271424	-1	#DIV/0!	1	#DIV/0!
Tcl1b4|27380	-1	#DIV/0!	1	#DIV/0!
Cst8|13012	-1	#DIV/0!	1	#DIV/0!
Zscan4a|434555	-1	#DIV/0!	1	#DIV/0!
Olfr243|436002	-1	#DIV/0!	1	#DIV/0!
Gm6607|625603	-1	#DIV/0!	1	#DIV/0!
4930469G21Rik|74933	-1	#DIV/0!	1	#DIV/0!
Acbd7|78245	-1	#DIV/0!	1	#DIV/0!
4930524E20Rik|75097	-1	#DIV/0!	1	#DIV/0!
Pmfbp1|56523	-1	#DIV/0!	1	#DIV/0!
Olfr30|18329	-1	#DIV/0!	1	#DIV/0!
Olfr1300-ps1|258199	-1	#DIV/0!	1	#DIV/0!
Pldi|73616	-1	#DIV/0!	1	#DIV/0!
Gm829|329839	-1	#DIV/0!	1	#DIV/0!
Olfr1029|258154	-1	#DIV/0!	1	#DIV/0!
Olfr1349|269862	-1	#DIV/0!	1	#DIV/0!
Il5|16191	-1	#DIV/0!	1	#DIV/0!
Slc6a5|104245	-1	#DIV/0!	1	#DIV/0!
Prm1|19118	-1	#DIV/0!	1	#DIV/0!
AI314831|329480	-1	#DIV/0!	1	#DIV/0!
Stoml3|229277	-1	#DIV/0!	1	#DIV/0!
Olfr875|258744	-1	#DIV/0!	1	#DIV/0!
Fam170b|105511	-1	#DIV/0!	1	#DIV/0!
Olfr996|258429	-1	#DIV/0!	1	#DIV/0!
Bai1|107831	-1	#DIV/0!	1	#DIV/0!
Sbk2|381836	-1	#DIV/0!	1	#DIV/0!
Gm815|329047	-1	#DIV/0!	1	#DIV/0!
Fam179a|320159	-1	#DIV/0!	1	#DIV/0!
Olfr514|258721	-1	#DIV/0!	1	#DIV/0!
Stmn4|56471	-1	#DIV/0!	1	#DIV/0!
Gm10665|100043058	-1	#DIV/0!	1	#DIV/0!
Gabrb1|14400	-1	#DIV/0!	1	#DIV/0!
Ofcc1|218165	-1	#DIV/0!	1	#DIV/0!
Prss2|22072	-1	#DIV/0!	1	#DIV/0!
Syt8|55925	-1	#DIV/0!	1	#DIV/0!
Olfr450|258437	-1	#DIV/0!	1	#DIV/0!

Nrn1l|234700	-1	#DIV/0!	1	#DIV/0!
4931407G18Rik|70977	-1	#DIV/0!	1	#DIV/0!
Olfr723|259147	-1	#DIV/0!	1	#DIV/0!
Abcc12|244562	-1	#DIV/0!	1	#DIV/0!
Gipr|381853	-1	#DIV/0!	1	#DIV/0!
Wfdc6b|433502	-1	#DIV/0!	1	#DIV/0!
Olfr1417|258938	-1	#DIV/0!	1	#DIV/0!
Rrh|20132	-1	#DIV/0!	1	#DIV/0!
9130204L05Rik|229550	-1	#DIV/0!	1	#DIV/0!
Aadacl2|639634	-1	#DIV/0!	1	#DIV/0!
Pfn3|75477	-1	#DIV/0!	1	#DIV/0!
Dcpp2|630537	-1	#DIV/0!	1	#DIV/0!
Vmn2r47|100042891	-1	#DIV/0!	1	#DIV/0!
Olfr780|258281	-1	#DIV/0!	1	#DIV/0!
6030498E09Rik|77883	-1	#DIV/0!	1	#DIV/0!
Ccdc121|403180	-1	#DIV/0!	1	#DIV/0!
Kcnh7|170738	-1	#DIV/0!	1	#DIV/0!
Olfr1055|259023	-1	#DIV/0!	1	#DIV/0!
Mir3099|100499513	-1	#DIV/0!	1	#DIV/0!
Catsperb|271036	-1	#DIV/0!	1	#DIV/0!
Magel2|27385	-1	#DIV/0!	1	#DIV/0!
Vmn2r109|627814	-1	#DIV/0!	1	#DIV/0!
Tcl1|21432	-1	#DIV/0!	1	#DIV/0!
Mir376c|723856	-1	#DIV/0!	1	#DIV/0!
Fgf4|14175	-1	#DIV/0!	1	#DIV/0!
Mir1944|100316696	-1	#DIV/0!	1	#DIV/0!
Olfr743|219019	-1	#DIV/0!	1	#DIV/0!
Emx2|13797	-1	#DIV/0!	1	#DIV/0!
Ssxb2|387132	-1	#DIV/0!	1	#DIV/0!
Muc5ac|17833	-1	#DIV/0!	1	#DIV/0!
Efhc1|71877	-1	#DIV/0!	1	#DIV/0!
Olfr170|258959	-1	#DIV/0!	1	#DIV/0!
Mir1971|100316715	-1	#DIV/0!	1	#DIV/0!
Lce3a|545548	-1	#DIV/0!	1	#DIV/0!
Olfr76|258677	-1	#DIV/0!	1	#DIV/0!
Phlda2|22113	-1	#DIV/0!	1	#DIV/0!
Shisa6|380702	-1	#DIV/0!	1	#DIV/0!
Phgr1|53906	-1	#DIV/0!	1	#DIV/0!
Hoxa7|15404	-1	#DIV/0!	1	#DIV/0!
Antxrl|239029	-1	#DIV/0!	1	#DIV/0!
BC051628|332713	-1	#DIV/0!	1	#DIV/0!
Adam26a|13525	-1	#DIV/0!	1	#DIV/0!
Olfr769|257667	-1	#DIV/0!	1	#DIV/0!
Mir299|723927	-1	#DIV/0!	1	#DIV/0!
Vmn1r82|171268	-1	#DIV/0!	1	#DIV/0!
Emilin3|280635	-1	#DIV/0!	1	#DIV/0!
Nepn|66650	-1	#DIV/0!	1	#DIV/0!
Olfr776|404321	-1	#DIV/0!	1	#DIV/0!
Tgif2lx2|100039551	-1	#DIV/0!	1	#DIV/0!
Hoxd13|15433	-1	#DIV/0!	1	#DIV/0!
Spatc1|74281	-1	#DIV/0!	1	#DIV/0!
B3gnt4|231727	-1	#DIV/0!	1	#DIV/0!
Mir429|723865	-1	#DIV/0!	1	#DIV/0!

Olfr1115|258294	-1	#DIV/0!	1	#DIV/0!
Vsnl1|26950	-1	#DIV/0!	1	#DIV/0!
Olfr1228|258973	-1	#DIV/0!	1	#DIV/0!
Olfr1504|258627	-1	#DIV/0!	1	#DIV/0!
Ninj2|29862	-1	#DIV/0!	1	#DIV/0!
Rgs18|64214	-1	#DIV/0!	1	#DIV/0!
Gm8677|667512	-1	#DIV/0!	1	#DIV/0!
Mir344d-1|100526544	-1	#DIV/0!	1	#DIV/0!
Gnat3|242851	-1	#DIV/0!	1	#DIV/0!
Olfr575|259118	-1	#DIV/0!	1	#DIV/0!
Ptprn2|19276	-1	#DIV/0!	1	#DIV/0!
Prl7a1|19113	-1	#DIV/0!	1	#DIV/0!
Vmn2r18|632671	-1	#DIV/0!	1	#DIV/0!
Sv2a|64051	-1	#DIV/0!	1	#DIV/0!
Hoxa10|15395	-1	#DIV/0!	1	#DIV/0!
Olfr1193|329460	-1	#DIV/0!	1	#DIV/0!
Nanos2|378430	-1	#DIV/0!	1	#DIV/0!
4930402F06Rik|74854	-1	#DIV/0!	1	#DIV/0!
Ryr2|20191	-1	#DIV/0!	1	#DIV/0!
Olfr287|634104	-1	#DIV/0!	1	#DIV/0!
Arhgap40|545481	-1	#DIV/0!	1	#DIV/0!
P2ry10|78826	-1	#DIV/0!	1	#DIV/0!
Snord72|100302529	-1	#DIV/0!	1	#DIV/0!
Ear5|54159	-1	#DIV/0!	1	#DIV/0!
Olfr341|258952	-1	#DIV/0!	1	#DIV/0!
Vmn2r56|629079	-1	#DIV/0!	1	#DIV/0!
Mir16-1|387134	-1	#DIV/0!	1	#DIV/0!
H2-M1|224756	-1	#DIV/0!	1	#DIV/0!
Vmn1r151|435947	-1	#DIV/0!	1	#DIV/0!
Snord92|100217462	-1	#DIV/0!	1	#DIV/0!
Kcnc1|16502	-1	#DIV/0!	1	#DIV/0!
Iqca|74918	-1	#DIV/0!	1	#DIV/0!
Olfr919|258432	-1	#DIV/0!	1	#DIV/0!
Ceacam5|73250	-1	#DIV/0!	1	#DIV/0!
Olfr972|258603	-1	#DIV/0!	1	#DIV/0!
Psmb11|73902	-1	#DIV/0!	1	#DIV/0!
Cxcl15|20309	-1	#DIV/0!	1	#DIV/0!
Olfr530|258512	-1	#DIV/0!	1	#DIV/0!
Krtap16-8|68484	-1	#DIV/0!	1	#DIV/0!
Olfr1269|258339	-1	#DIV/0!	1	#DIV/0!
Gm4736|114600	-1	#DIV/0!	1	#DIV/0!
Olfr15|18312	-1	#DIV/0!	1	#DIV/0!
Mir221|723827	-1	#DIV/0!	1	#DIV/0!
Dppa5a|434423	-1	#DIV/0!	1	#DIV/0!
Dmkn|73712	-1	#DIV/0!	1	#DIV/0!
Gm11554|670482	-1	#DIV/0!	1	#DIV/0!
Dfnb59|381375	-1	#DIV/0!	1	#DIV/0!
Gm11426|791422	-1	#DIV/0!	1	#DIV/0!
Theg|21830	-1	#DIV/0!	1	#DIV/0!
Gm590|235634	-1	#DIV/0!	1	#DIV/0!
Krt32|16670	-1	#DIV/0!	1	#DIV/0!
Gm10823|100038623	-1	#DIV/0!	1	#DIV/0!
Olfr683|259047	-1	#DIV/0!	1	#DIV/0!

Mcpt8|17231	-1	#DIV/0!	1	#DIV/0!
Zar1l|545824	-1	#DIV/0!	1	#DIV/0!
Shc3|20418	-1	#DIV/0!	1	#DIV/0!
Olfr676|259099	-1	#DIV/0!	1	#DIV/0!
Sgca|20391	-1	#DIV/0!	1	#DIV/0!
Neurog3|11925	-1	#DIV/0!	1	#DIV/0!
Vmn1r143|667469	-1	#DIV/0!	1	#DIV/0!
Ccl12|20293	-1	#DIV/0!	1	#DIV/0!
Olfr54|18354	-1	#DIV/0!	1	#DIV/0!
3632451O06Rik|67419	-1	#DIV/0!	1	#DIV/0!
Enox1|239188	-1	#DIV/0!	1	#DIV/0!
Zdbf2|73884	-1	#DIV/0!	1	#DIV/0!
Mir3474|100499529	-1	#DIV/0!	1	#DIV/0!
Chrna7|11441	-1	#DIV/0!	1	#DIV/0!
Tnp2|21959	-1	#DIV/0!	1	#DIV/0!
Mir574|100124451	-1	#DIV/0!	1	#DIV/0!
Tas2r137|574417	-1	#DIV/0!	1	#DIV/0!
Gm9705|677156	-1	#DIV/0!	1	#DIV/0!
Mir297-1|723831	-1	#DIV/0!	1	#DIV/0!
Prss54|70993	-1	#DIV/0!	1	#DIV/0!
Olfr523|258511	-1	#DIV/0!	1	#DIV/0!
Tex16|83556	-1	#DIV/0!	1	#DIV/0!
Mir140|387158	-1	#DIV/0!	1	#DIV/0!
Defb45|433490	-1	#DIV/0!	1	#DIV/0!
Scarna3a|100217414	-1	#DIV/0!	1	#DIV/0!
Dub2a|384701	-1	#DIV/0!	1	#DIV/0!
Olfr873|258554	-1	#DIV/0!	1	#DIV/0!
Rprm|67874	-1	#DIV/0!	1	#DIV/0!
Olfr316|258064	-1	#DIV/0!	1	#DIV/0!
Snx31|66696	-1	#DIV/0!	1	#DIV/0!
Krtap3-1|69473	-1	#DIV/0!	1	#DIV/0!
Dcpp3|620253	-1	#DIV/0!	1	#DIV/0!
BC016579|212998	-1	#DIV/0!	1	#DIV/0!
Ttc39d|67737	-1	#DIV/0!	1	#DIV/0!
Fam40b|320609	-1	#DIV/0!	1	#DIV/0!
Olfr430|258713	-1	#DIV/0!	1	#DIV/0!
Mir146b|751550	-1	#DIV/0!	1	#DIV/0!
Irg1|16365	-1	#DIV/0!	1	#DIV/0!
Mir148a|387166	-1	#DIV/0!	1	#DIV/0!
Lass3|545975	-1	#DIV/0!	1	#DIV/0!
Olfr140|57272	-1	#DIV/0!	1	#DIV/0!
4930557A04Rik|385317	-1	#DIV/0!	1	#DIV/0!
Mir30c-1|387227	-1	#DIV/0!	1	#DIV/0!
Gm15293|100041759	-1	#DIV/0!	1	#DIV/0!
Sox15|20670	-1	#DIV/0!	1	#DIV/0!
Diras1|208666	-1	#DIV/0!	1	#DIV/0!
Pgf|18654	-1	#DIV/0!	1	#DIV/0!
Ccr6|12458	-1	#DIV/0!	1	#DIV/0!
Guca2b|14916	-1	#DIV/0!	1	#DIV/0!
Shox2|20429	-1	#DIV/0!	1	#DIV/0!
Vmn1r2|100312470	-1	#DIV/0!	1	#DIV/0!
Olfr151|406176	-1	#DIV/0!	1	#DIV/0!
1700022P22Rik|75558	-1	#DIV/0!	1	#DIV/0!

Vmn2r40|100042781	-1	#DIV/0!	1	#DIV/0!
Gsc|14836	-1	#DIV/0!	1	#DIV/0!
BC030870|407795	-1	#DIV/0!	1	#DIV/0!
C1ql2|226359	-1	#DIV/0!	1	#DIV/0!
Mir101b|724062	-1	#DIV/0!	1	#DIV/0!
Sry|21674	-1	#DIV/0!	1	#DIV/0!
Mtap7d3|320923	-1	#DIV/0!	1	#DIV/0!
2810405F15Rik|69974	-1	#DIV/0!	1	#DIV/0!
Ecel1|13599	-1	#DIV/0!	1	#DIV/0!
Chst5|56773	-1	#DIV/0!	1	#DIV/0!
Mir152|387170	-1	#DIV/0!	1	#DIV/0!
Gm10229|100040201	-1	#DIV/0!	1	#DIV/0!
Myo15|17910	-1	#DIV/0!	1	#DIV/0!
Lrrc7|242274	-1	#DIV/0!	1	#DIV/0!
Psg19|26439	-1	#DIV/0!	1	#DIV/0!
Vmn1r119|384696	-1	#DIV/0!	1	#DIV/0!
Ch25h|12642	-1	#DIV/0!	1	#DIV/0!
Npsr1|319239	-1	#DIV/0!	1	#DIV/0!
Clcnkb|56365	-1	#DIV/0!	1	#DIV/0!
Mrgpra4|235854	-1	#DIV/0!	1	#DIV/0!
Clec4a4|474145	-1	#DIV/0!	1	#DIV/0!
Scn2b|72821	-1	#DIV/0!	1	#DIV/0!
Olfr974|259111	-1	#DIV/0!	1	#DIV/0!
Mir26a-2|723962	-1	#DIV/0!	1	#DIV/0!
Foxi2|270004	-1	#DIV/0!	1	#DIV/0!
Psg20|434540	-1	#DIV/0!	1	#DIV/0!
Mir465c-2|100124464	-1	#DIV/0!	1	#DIV/0!
1700066O22Rik|74916	-1	#DIV/0!	1	#DIV/0!
AW551984|244810	-1	#DIV/0!	1	#DIV/0!
Mir302c|723835	-1	#DIV/0!	1	#DIV/0!
Spink14|433178	-1	#DIV/0!	1	#DIV/0!
Lrrc9|78257	-1	#DIV/0!	1	#DIV/0!
Bet3l|692132	-1	#DIV/0!	1	#DIV/0!
Hist1h2bq|665596	-1	#DIV/0!	1	#DIV/0!
a|50518	-1	#DIV/0!	1	#DIV/0!
Prg2|19074	-1	#DIV/0!	1	#DIV/0!
Sv2b|64176	-1	#DIV/0!	1	#DIV/0!
Olfr509|258369	-1	#DIV/0!	1	#DIV/0!
Gtsf1l|68236	-1	#DIV/0!	1	#DIV/0!
Olfr1453|258695	-1	#DIV/0!	1	#DIV/0!
Psg27|545925	-1	#DIV/0!	1	#DIV/0!
Olfr959|258501	-1	#DIV/0!	1	#DIV/0!
Olfr1463|258120	-1	#DIV/0!	1	#DIV/0!
Olfr772|257666	-1	#DIV/0!	1	#DIV/0!
Abph|57426	-1	#DIV/0!	1	#DIV/0!
Gm757|329360	-1	#DIV/0!	1	#DIV/0!
Krtap31-1|70831	-1	#DIV/0!	1	#DIV/0!
Mrgpra2a|668727	-1	#DIV/0!	1	#DIV/0!
Dlx6as1|320038	-1	#DIV/0!	1	#DIV/0!
Uncx|22255	-1	#DIV/0!	1	#DIV/0!
Htr1d|15552	-1	#DIV/0!	1	#DIV/0!
Mrgpra3|233222	-1	#DIV/0!	1	#DIV/0!
Crhbp|12919	-1	#DIV/0!	1	#DIV/0!

4930505A04Rik|75087	-1	#DIV/0!	1	#DIV/0!
Olfr1145|258317	-1	#DIV/0!	1	#DIV/0!
Ctxn3|629147	-1	#DIV/0!	1	#DIV/0!
Olfr1262|258976	-1	#DIV/0!	1	#DIV/0!
Khdc1c|433278	-1	#DIV/0!	1	#DIV/0!
Cypt3|69361	-1	#DIV/0!	1	#DIV/0!
Pip5k1b|18719	-1	#DIV/0!	1	#DIV/0!
Hoxc11|109663	-1	#DIV/0!	1	#DIV/0!
Olfr1107|258841	-1	#DIV/0!	1	#DIV/0!
Adam21|56622	-1	#DIV/0!	1	#DIV/0!
Vmn2r82|624845	-1	#DIV/0!	1	#DIV/0!
Prp2|83380	-1	#DIV/0!	1	#DIV/0!
Ccl20|20297	-1	#DIV/0!	1	#DIV/0!
Gm12695|620779	-1	#DIV/0!	1	#DIV/0!
Actrt1|73360	-1	#DIV/0!	1	#DIV/0!
Ly6g5c|114652	-1	#DIV/0!	1	#DIV/0!
Mir451|723870	-1	#DIV/0!	1	#DIV/0!
Mir665|751555	-1	#DIV/0!	1	#DIV/0!
Mir1955|100316756	-1	#DIV/0!	1	#DIV/0!
Ankar|319695	-1	#DIV/0!	1	#DIV/0!
Lgi2|246316	-1	#DIV/0!	1	#DIV/0!
Mir96|723886	-1	#DIV/0!	1	#DIV/0!
Ribc2|67747	-1	#DIV/0!	1	#DIV/0!
Vmn1r202|171258	-1	#DIV/0!	1	#DIV/0!
Olfr225|257886	-1	#DIV/0!	1	#DIV/0!
Clec2g|70809	-1	#DIV/0!	1	#DIV/0!
Gm13277|545650	-1	#DIV/0!	1	#DIV/0!
Tbpl2|227606	-1	#DIV/0!	1	#DIV/0!
U90926|57425	-1	#DIV/0!	1	#DIV/0!
Ajap1|230959	-1	#DIV/0!	1	#DIV/0!
Cnga2|12789	-1	#DIV/0!	1	#DIV/0!
Sox3|20675	-1	#DIV/0!	1	#DIV/0!
Olfr1329|258214	-1	#DIV/0!	1	#DIV/0!
Mcoln3|171166	-1	#DIV/0!	1	#DIV/0!
Prl2a1|56635	-1	#DIV/0!	1	#DIV/0!
Cntn5|244682	-1	#DIV/0!	1	#DIV/0!
Krt77|406220	-1	#DIV/0!	1	#DIV/0!
Vmn1r167|622032	-1	#DIV/0!	1	#DIV/0!
Olfr1388|258459	-1	#DIV/0!	1	#DIV/0!
Aqp12|208760	-1	#DIV/0!	1	#DIV/0!
Il28a|330496	-1	#DIV/0!	1	#DIV/0!
Olfr1508|57270	-1	#DIV/0!	1	#DIV/0!
7420426K07Rik|546157	-1	#DIV/0!	1	#DIV/0!
Olfr198|258036	-1	#DIV/0!	1	#DIV/0!
Pnpla5|75772	-1	#DIV/0!	1	#DIV/0!
Krt14|16664	-1	#DIV/0!	1	#DIV/0!
BC048943|217874	-1	#DIV/0!	1	#DIV/0!
Eif2s3y|26908	-1	#DIV/0!	1	#DIV/0!
Rfx6|320995	-1	#DIV/0!	1	#DIV/0!
Mirlet7e|387248	-1	#DIV/0!	1	#DIV/0!
4933411G11Rik|330228	-1	#DIV/0!	1	#DIV/0!
2410004P03Rik|73667	-1	#DIV/0!	1	#DIV/0!
Tram1l1|229801	-1	#DIV/0!	1	#DIV/0!

Ndc80|67052	-1	#DIV/0!	1	#DIV/0!
Gm15315|100041952	-1	#DIV/0!	1	#DIV/0!
Mir323|723839	-1	#DIV/0!	1	#DIV/0!
Mrgprb8|404240	-1	#DIV/0!	1	#DIV/0!
Hist2h4|97122	-1	#DIV/0!	1	#DIV/0!
Gm14511|434727	-1	#DIV/0!	1	#DIV/0!
Rhox4c|434759	-1	#DIV/0!	1	#DIV/0!
Olfr1494|258992	-1	#DIV/0!	1	#DIV/0!
Tas2r140|387616	-1	#DIV/0!	1	#DIV/0!
Klra23|79410	-1	#DIV/0!	1	#DIV/0!
Krt84|16680	-1	#DIV/0!	1	#DIV/0!
Tas2r104|387340	-1	#DIV/0!	1	#DIV/0!
Olfr541|258964	-1	#DIV/0!	1	#DIV/0!
Ros1|19886	-1	#DIV/0!	1	#DIV/0!
Pdcl2|79455	-1	#DIV/0!	1	#DIV/0!
Ifna1|15962	-1	#DIV/0!	1	#DIV/0!
Spo11|26972	-1	#DIV/0!	1	#DIV/0!
Olfr351|258944	-1	#DIV/0!	1	#DIV/0!
Rhox3h|434758	-1	#DIV/0!	1	#DIV/0!
Fam83b|208994	-1	#DIV/0!	1	#DIV/0!
Tas2r116|112408	-1	#DIV/0!	1	#DIV/0!
Olfr324|257892	-1	#DIV/0!	1	#DIV/0!
1700012P22Rik|69364	-1	#DIV/0!	1	#DIV/0!
Krt40|406221	-1	#DIV/0!	1	#DIV/0!
Fam83e|73813	-1	#DIV/0!	1	#DIV/0!
Fam178b|381337	-1	#DIV/0!	1	#DIV/0!
Rnf112|22671	-1	#DIV/0!	1	#DIV/0!
Otof|83762	-1	#DIV/0!	1	#DIV/0!
Xlr4a|434794	-1	#DIV/0!	1	#DIV/0!
5330437I02Rik|319888	-1	#DIV/0!	1	#DIV/0!
Pet2|18630	-1	#DIV/0!	1	#DIV/0!
Vmn2r17|384221	-1	#DIV/0!	1	#DIV/0!
Rbp7|63954	-1	#DIV/0!	1	#DIV/0!
Ffar3|233080	-1	#DIV/0!	1	#DIV/0!
Bpi|329547	-1	#DIV/0!	1	#DIV/0!
Olfr432|258711	-1	#DIV/0!	1	#DIV/0!
Olfr524|258055	-1	#DIV/0!	1	#DIV/0!
Nhedc2|97086	-1	#DIV/0!	1	#DIV/0!
Vmn2r23|435916	-1	#DIV/0!	1	#DIV/0!
Mir491|735282	-1	#DIV/0!	1	#DIV/0!
Olfr1090|258844	-1	#DIV/0!	1	#DIV/0!
Gm15093|100039890	-1	#DIV/0!	1	#DIV/0!
Olfr742|258422	-1	#DIV/0!	1	#DIV/0!
Ifna2|15965	-1	#DIV/0!	1	#DIV/0!
Pcdha3|192163	-1	#DIV/0!	1	#DIV/0!
C130079G13Rik|229333	-1	#DIV/0!	1	#DIV/0!
Olfr446|258292	-1	#DIV/0!	1	#DIV/0!
Epha5|13839	-1	#DIV/0!	1	#DIV/0!
Edil3|13612	-1	#DIV/0!	1	#DIV/0!
Pcdhgc4|93707	-1	#DIV/0!	1	#DIV/0!
Snord104|100216537	-1	#DIV/0!	1	#DIV/0!
Gm4340|100043292	-1	#DIV/0!	1	#DIV/0!
Mir3070a|100526472	-1	#DIV/0!	1	#DIV/0!

Hesx1|15209	-1	#DIV/0!	1	#DIV/0!
Tmem211|333048	-1	#DIV/0!	1	#DIV/0!
Ankrd33|208258	-1	#DIV/0!	1	#DIV/0!
Lonrf2|381338	-1	#DIV/0!	1	#DIV/0!
Depdc1a|76131	-1	#DIV/0!	1	#DIV/0!
Vmn1r84|171267	-1	#DIV/0!	1	#DIV/0!
Ceacam13|69785	-1	#DIV/0!	1	#DIV/0!
Mettl7a3|668178	-1	#DIV/0!	1	#DIV/0!
4930502E18Rik|75013	-1	#DIV/0!	1	#DIV/0!
Vmn1r30|171195	-1	#DIV/0!	1	#DIV/0!
Vmn1r62|81016	-1	#DIV/0!	1	#DIV/0!
Mir339|723898	-1	#DIV/0!	1	#DIV/0!
Mir409|723862	-1	#DIV/0!	1	#DIV/0!
Krt35|53617	-1	#DIV/0!	1	#DIV/0!
Lmo3|109593	-1	#DIV/0!	1	#DIV/0!
Olfr433|258712	-1	#DIV/0!	1	#DIV/0!
Cst13|69294	-1	#DIV/0!	1	#DIV/0!
Prss51|74215	-1	#DIV/0!	1	#DIV/0!
Abpz|233090	-1	#DIV/0!	1	#DIV/0!
Vmn1r38|171186	-1	#DIV/0!	1	#DIV/0!
Nuf2|66977	-1	#DIV/0!	1	#DIV/0!
Vmn1r50|113852	-1	#DIV/0!	1	#DIV/0!
Izumo3|69314	-1	#DIV/0!	1	#DIV/0!
A830018L16Rik|320492	-1	#DIV/0!	1	#DIV/0!
Ctsm|64139	-1	#DIV/0!	1	#DIV/0!
Rpp25|102614	-1	#DIV/0!	1	#DIV/0!
Vmn2r39|545909	-1	#DIV/0!	1	#DIV/0!
Mir3057|100526465	-1	#DIV/0!	1	#DIV/0!
Gm1337|383787	-1	#DIV/0!	1	#DIV/0!
Olfr1509|57271	-1	#DIV/0!	1	#DIV/0!
Slc35f1|215085	-1	#DIV/0!	1	#DIV/0!
Olfr221|258420	-1	#DIV/0!	1	#DIV/0!
Gm3086|100040999	-1	#DIV/0!	1	#DIV/0!
B3galt2|26878	-1	#DIV/0!	1	#DIV/0!
2310050B05Rik|69678	-1	#DIV/0!	1	#DIV/0!
Mir680-3|751520	-1	#DIV/0!	1	#DIV/0!
Sprr2f|20760	-1	#DIV/0!	1	#DIV/0!
Olfr819|100043200	-1	#DIV/0!	1	#DIV/0!
Tbx5|21388	-1	#DIV/0!	1	#DIV/0!
Olfr561|259096	-1	#DIV/0!	1	#DIV/0!
Gm1995|100038982	-1	#DIV/0!	1	#DIV/0!
Gpr31c|436440	-1	#DIV/0!	1	#DIV/0!
Mir384|723861	-1	#DIV/0!	1	#DIV/0!
Gm14501|547154	-1	#DIV/0!	1	#DIV/0!
Mtap6|17760	-1	#DIV/0!	1	#DIV/0!
Mir3070b|100526519	-1	#DIV/0!	1	#DIV/0!
Xkr7|228787	-1	#DIV/0!	1	#DIV/0!
Olfr484|258492	-1	#DIV/0!	1	#DIV/0!
Gm9696|676914	-1	#DIV/0!	1	#DIV/0!
Meox2|17286	-1	#DIV/0!	1	#DIV/0!
Cnr1|12801	-1	#DIV/0!	1	#DIV/0!
Wdr64|75820	-1	#DIV/0!	1	#DIV/0!
Mir466|723922	-1	#DIV/0!	1	#DIV/0!

Gm9376|668814	-1	#DIV/0!	1	#DIV/0!
3110047P20Rik|319807	-1	#DIV/0!	1	#DIV/0!
Gpr128|239853	-1	#DIV/0!	1	#DIV/0!
4921533I20Rik|664619	-1	#DIV/0!	1	#DIV/0!
Vmn1r71|252910	-1	#DIV/0!	1	#DIV/0!
Kif2b|73470	-1	#DIV/0!	1	#DIV/0!
Hydin|244653	-1	#DIV/0!	1	#DIV/0!
Scn11a|24046	-1	#DIV/0!	1	#DIV/0!
Gm8787|667736	-1	#DIV/0!	1	#DIV/0!
Mir455|735262	-1	#DIV/0!	1	#DIV/0!
Dydc2|71200	-1	#DIV/0!	1	#DIV/0!
AF357359|100303647	-1	#DIV/0!	1	#DIV/0!
Gm14458|100042782	-1	#DIV/0!	1	#DIV/0!
Olfr1260|258983	-1	#DIV/0!	1	#DIV/0!
Olfr297|258611	-1	#DIV/0!	1	#DIV/0!
Olfr1475|258298	-1	#DIV/0!	1	#DIV/0!
3830431G21Rik|217682	-1	#DIV/0!	1	#DIV/0!
Prrx2|20204	-1	#DIV/0!	1	#DIV/0!
Wdr69|71227	-1	#DIV/0!	1	#DIV/0!
Olfr830|258559	-1	#DIV/0!	1	#DIV/0!
Art1|11870	-1	#DIV/0!	1	#DIV/0!
Sprr2j-ps|20764	-1	#DIV/0!	1	#DIV/0!
1700018L24Rik|75528	-1	#DIV/0!	1	#DIV/0!
Olfr1361|258534	-1	#DIV/0!	1	#DIV/0!
Olfr55|100038859	-1	#DIV/0!	1	#DIV/0!
Gm1322|383709	-1	#DIV/0!	1	#DIV/0!
Gm4566|100043644	-1	#DIV/0!	1	#DIV/0!
Actbl2|238880	-1	#DIV/0!	1	#DIV/0!
Gm20554|328287	-1	#DIV/0!	1	#DIV/0!
Mir210|387206	-1	#DIV/0!	1	#DIV/0!
Cnih3|72978	-1	#DIV/0!	1	#DIV/0!
C130060K24Rik|243407	-1	#DIV/0!	1	#DIV/0!
Gm3750|100042254	-1	#DIV/0!	1	#DIV/0!
Vmn1r191|632534	-1	#DIV/0!	1	#DIV/0!
Zfp352|236537	-1	#DIV/0!	1	#DIV/0!
Olfr850|258516	-1	#DIV/0!	1	#DIV/0!
Olfr1444|258697	-1	#DIV/0!	1	#DIV/0!
Wfdc16|277345	-1	#DIV/0!	1	#DIV/0!
Olfr916|258780	-1	#DIV/0!	1	#DIV/0!
4930480E11Rik|74910	-1	#DIV/0!	1	#DIV/0!
Slc22a20|381203	-1	#DIV/0!	1	#DIV/0!
Ccdc83|75338	-1	#DIV/0!	1	#DIV/0!
Klk1b7-ps|16604	-1	#DIV/0!	1	#DIV/0!
Odf3|69287	-1	#DIV/0!	1	#DIV/0!
Olfr1425|258155	-1	#DIV/0!	1	#DIV/0!
Gm5662|435337	-1	#DIV/0!	1	#DIV/0!
Gm12238|100303747	-1	#DIV/0!	1	#DIV/0!
Olfr16|18313	-1	#DIV/0!	1	#DIV/0!
Prss55|71037	-1	#DIV/0!	1	#DIV/0!
Spink11|433181	-1	#DIV/0!	1	#DIV/0!
Snora23|100379145	-1	#DIV/0!	1	#DIV/0!
Clrn2|624224	-1	#DIV/0!	1	#DIV/0!
Mir29a|387222	-1	#DIV/0!	1	#DIV/0!

Mir669p-1|100526552	-1	#DIV/0!	1	#DIV/0!
Dgkb|217480	-1	#DIV/0!	1	#DIV/0!
Gm11559|100415785	-1	#DIV/0!	1	#DIV/0!
Iapp|15874	-1	#DIV/0!	1	#DIV/0!
Zfp964|636741	-1	#DIV/0!	1	#DIV/0!
Vmn1r142|667464	-1	#DIV/0!	1	#DIV/0!
Aire|11634	-1	#DIV/0!	1	#DIV/0!
Olfr1037|259151	-1	#DIV/0!	1	#DIV/0!
Olfr666|259100	-1	#DIV/0!	1	#DIV/0!
5730422E09Rik|433966	-1	#DIV/0!	1	#DIV/0!
Vmn2r103|627636	-1	#DIV/0!	1	#DIV/0!
Olfr593|258378	-1	#DIV/0!	1	#DIV/0!
Rbm11|224344	-1	#DIV/0!	1	#DIV/0!
6330407J23Rik|67412	-1	#DIV/0!	1	#DIV/0!
Olfr1382|257971	-1	#DIV/0!	1	#DIV/0!
Gm4759|209380	-1	#DIV/0!	1	#DIV/0!
4921528I01Rik|70950	-1	#DIV/0!	1	#DIV/0!
Gzmn|245839	-1	#DIV/0!	1	#DIV/0!
Gm10024|100009614	-1	#DIV/0!	1	#DIV/0!
AF529169|209743	-1	#DIV/0!	1	#DIV/0!
Efcab4b|381812	-1	#DIV/0!	1	#DIV/0!
Cdk5r2|12570	-1	#DIV/0!	1	#DIV/0!
Ldoc1|434784	-1	#DIV/0!	1	#DIV/0!
Scarna8|100217448	-1	#DIV/0!	1	#DIV/0!
Mir1901|100316686	-1	#DIV/0!	1	#DIV/0!
Klra6|16637	-1	#DIV/0!	1	#DIV/0!
Pnma1|70481	-1	#DIV/0!	1	#DIV/0!
Cdh24|239096	-1	#DIV/0!	1	#DIV/0!
Mir431|723866	-1	#DIV/0!	1	#DIV/0!
Prl3d2|215028	-1	#DIV/0!	1	#DIV/0!
Olfr129|258324	-1	#DIV/0!	1	#DIV/0!
Nts|67405	-1	#DIV/0!	1	#DIV/0!
Gbx2|14472	-1	#DIV/0!	1	#DIV/0!
Vmn1r3|100312471	-1	#DIV/0!	1	#DIV/0!
Hist1h2ag|319167	-1	#DIV/0!	1	#DIV/0!
Itgb8|320910	-1	#DIV/0!	1	#DIV/0!
Olfr1384|258464	-1	#DIV/0!	1	#DIV/0!
Sycp1-ps1|20958	-1	#DIV/0!	1	#DIV/0!
Snord38a|100217424	-1	#DIV/0!	1	#DIV/0!
Gm5072|278167	-1	#DIV/0!	1	#DIV/0!
Taar7e|276742	-1	#DIV/0!	1	#DIV/0!
Prol1|17830	-1	#DIV/0!	1	#DIV/0!
Cyp2j8|665095	-1	#DIV/0!	1	#DIV/0!
Kcne2|246133	-1	#DIV/0!	1	#DIV/0!
Prl8a6|19112	-1	#DIV/0!	1	#DIV/0!
Olfr199|404310	-1	#DIV/0!	1	#DIV/0!
Gm5567|434008	-1	#DIV/0!	1	#DIV/0!
Avpr1b|26361	-1	#DIV/0!	1	#DIV/0!
Gm12253|624860	-1	#DIV/0!	1	#DIV/0!
Tas2r105|57252	-1	#DIV/0!	1	#DIV/0!
Olfr630|259102	-1	#DIV/0!	1	#DIV/0!
Vmn1r128|667199	-1	#DIV/0!	1	#DIV/0!
Foxi1|14233	-1	#DIV/0!	1	#DIV/0!

Mir669a-1|735256	-1	#DIV/0!	1	#DIV/0!
Fbxo39|628100	-1	#DIV/0!	1	#DIV/0!
Olfr695|258591	-1	#DIV/0!	1	#DIV/0!
Olfr93|258051	-1	#DIV/0!	1	#DIV/0!
Acoxl|74121	-1	#DIV/0!	1	#DIV/0!
Chrm2|243764	-1	#DIV/0!	1	#DIV/0!
Hoxd8|15437	-1	#DIV/0!	1	#DIV/0!
Crhr1|12921	-1	#DIV/0!	1	#DIV/0!
Olfr1364|258533	-1	#DIV/0!	1	#DIV/0!
Dll3|13389	-1	#DIV/0!	1	#DIV/0!
9930023K05Rik|226245	-1	#DIV/0!	1	#DIV/0!
Slco6d1|70866	-1	#DIV/0!	1	#DIV/0!
Olfr237-ps1|258648	-1	#DIV/0!	1	#DIV/0!
Tspy-ps|22109	-1	#DIV/0!	1	#DIV/0!
Mir3073|100526547	-1	#DIV/0!	1	#DIV/0!
Car4|12351	-1	#DIV/0!	1	#DIV/0!
Olfr1184|258820	-1	#DIV/0!	1	#DIV/0!
Fgf10|14165	-1	#DIV/0!	1	#DIV/0!
A4gnt|333424	-1	#DIV/0!	1	#DIV/0!
Prss46|74306	-1	#DIV/0!	1	#DIV/0!
Olfr525|258958	-1	#DIV/0!	1	#DIV/0!
Vmn1r208|171252	-1	#DIV/0!	1	#DIV/0!
Olfr657|258309	-1	#DIV/0!	1	#DIV/0!
Gm13119|433779	-1	#DIV/0!	1	#DIV/0!
Ceacam16|330483	-1	#DIV/0!	1	#DIV/0!
Mir1983|100316716	-1	#DIV/0!	1	#DIV/0!
Mir3108|100526511	-1	#DIV/0!	1	#DIV/0!
Hsh2d|209488	-1	#DIV/0!	1	#DIV/0!
Tmem212|208613	-1	#DIV/0!	1	#DIV/0!
Lrrc66|231296	-1	#DIV/0!	1	#DIV/0!
Chrna10|504186	-1	#DIV/0!	1	#DIV/0!
Rpe65|19892	-1	#DIV/0!	1	#DIV/0!
Ctsg|13035	-1	#DIV/0!	1	#DIV/0!
Ccdc88a|108686	-1	#DIV/0!	1	#DIV/0!
Pga5|58803	-1	#DIV/0!	1	#DIV/0!
Frmd7|385354	-1	#DIV/0!	1	#DIV/0!
Grik1|14805	-1	#DIV/0!	1	#DIV/0!
Ceacam10|26366	-1	#DIV/0!	1	#DIV/0!
Mycs|17870	-1	#DIV/0!	1	#DIV/0!
Mir190|387185	-1	#DIV/0!	1	#DIV/0!
Myo16|244281	-1	#DIV/0!	1	#DIV/0!
Olfr247|258266	-1	#DIV/0!	1	#DIV/0!
Olfr1284|258379	-1	#DIV/0!	1	#DIV/0!
Snora19|100217439	-1	#DIV/0!	1	#DIV/0!
Gm12794|332923	-1	#DIV/0!	1	#DIV/0!
Tspan11|68498	-1	#DIV/0!	1	#DIV/0!
Olfr1355|257734	-1	#DIV/0!	1	#DIV/0!
Wee2|381759	-1	#DIV/0!	1	#DIV/0!
Olfr594|258246	-1	#DIV/0!	1	#DIV/0!
Olfr365|258656	-1	#DIV/0!	1	#DIV/0!
Gm6537|624918	-1	#DIV/0!	1	#DIV/0!
AU015836|385493	-1	#DIV/0!	1	#DIV/0!
E230016K23Rik|414102	-1	#DIV/0!	1	#DIV/0!

Zfp804b|207618	-1	#DIV/0!	1	#DIV/0!
Snord35b|27212	-1	#DIV/0!	1	#DIV/0!
Prss27|213171	-1	#DIV/0!	1	#DIV/0!
Sall3|20689	-1	#DIV/0!	1	#DIV/0!
Olfr1295|258398	-1	#DIV/0!	1	#DIV/0!
Olfr474|258488	-1	#DIV/0!	1	#DIV/0!
Fibcd1|98970	-1	#DIV/0!	1	#DIV/0!
Gm8882|667929	-1	#DIV/0!	1	#DIV/0!
Olfr463|258408	-1	#DIV/0!	1	#DIV/0!
Gm10921|668963	-1	#DIV/0!	1	#DIV/0!
Olfr323|258373	-1	#DIV/0!	1	#DIV/0!
Vmn1r87|171261	-1	#DIV/0!	1	#DIV/0!
Vmn1r228|171226	-1	#DIV/0!	1	#DIV/0!
Rnf183|76072	-1	#DIV/0!	1	#DIV/0!
Popdc3|78977	-1	#DIV/0!	1	#DIV/0!
Mir328|723841	-1	#DIV/0!	1	#DIV/0!
Olfr1155|258636	-1	#DIV/0!	1	#DIV/0!
Mir511|100124488	-1	#DIV/0!	1	#DIV/0!
Vmn1r114|100042996	-1	#DIV/0!	1	#DIV/0!
E130114P18Rik|319865	-1	#DIV/0!	1	#DIV/0!
Grin2a|14811	-1	#DIV/0!	1	#DIV/0!
Gm2176|100039348	-1	#DIV/0!	1	#DIV/0!
Vmn1r220|171271	-1	#DIV/0!	1	#DIV/0!
Gcet2|14525	-1	#DIV/0!	1	#DIV/0!
Lcn11|227630	-1	#DIV/0!	1	#DIV/0!
Fndc3c1|333564	-1	#DIV/0!	1	#DIV/0!
Pcdh11x|245578	-1	#DIV/0!	1	#DIV/0!
Reg2|19693	-1	#DIV/0!	1	#DIV/0!
Actl9|69481	-1	#DIV/0!	1	#DIV/0!
Olfr1132|258833	-1	#DIV/0!	1	#DIV/0!
Mir106b|723925	-1	#DIV/0!	1	#DIV/0!
Tigd4|403175	-1	#DIV/0!	1	#DIV/0!
Ang6|630952	-1	#DIV/0!	1	#DIV/0!
Olfr203|258479	-1	#DIV/0!	1	#DIV/0!
Coch|12810	-1	#DIV/0!	1	#DIV/0!
6030405A18Rik|329641	-1	#DIV/0!	1	#DIV/0!
Rassf9|237504	-1	#DIV/0!	1	#DIV/0!
8430431K14Rik|78103	-1	#DIV/0!	1	#DIV/0!
Rhox7|547168	-1	#DIV/0!	1	#DIV/0!
2310057N15Rik|69696	-1	#DIV/0!	1	#DIV/0!
1700108M19Rik|71156	-1	#DIV/0!	1	#DIV/0!
Adam30|71078	-1	#DIV/0!	1	#DIV/0!
Scg3|20255	-1	#DIV/0!	1	#DIV/0!
Slc6a2|20538	-1	#DIV/0!	1	#DIV/0!
Olfr1416|259040	-1	#DIV/0!	1	#DIV/0!
Gm3238|100041261	-1	#DIV/0!	1	#DIV/0!
Ankrd6|140577	-1	#DIV/0!	1	#DIV/0!
Mir301|723834	-1	#DIV/0!	1	#DIV/0!
Mir3088|100526498	-1	#DIV/0!	1	#DIV/0!
Klk1b8|16624	-1	#DIV/0!	1	#DIV/0!
Cyp19a1|13075	-1	#DIV/0!	1	#DIV/0!
Snap91|20616	-1	#DIV/0!	1	#DIV/0!
Vmn1r107|673977	-1	#DIV/0!	1	#DIV/0!

Bpifb9a|71425	-1	#DIV/0!	1	#DIV/0!
Shroom4|208431	-1	#DIV/0!	1	#DIV/0!
Lhx3|16871	-1	#DIV/0!	1	#DIV/0!
Vmn1r43|113847	-1	#DIV/0!	1	#DIV/0!
Nlgn1|192167	-1	#DIV/0!	1	#DIV/0!
Ralyl|76897	-1	#DIV/0!	1	#DIV/0!
Mt4|17752	-1	#DIV/0!	1	#DIV/0!
Olfr967|258086	-1	#DIV/0!	1	#DIV/0!
Crct1|74175	-1	#DIV/0!	1	#DIV/0!
LOC100043315|100043315	-1	#DIV/0!	1	#DIV/0!
4933402N22Rik|545732	-1	#DIV/0!	1	#DIV/0!
Olfr1217|258903	-1	#DIV/0!	1	#DIV/0!
Bmx|12169	-1	#DIV/0!	1	#DIV/0!
Hist1h3d|319149	-1	#DIV/0!	1	#DIV/0!
3830417A13Rik|70696	-1	#DIV/0!	1	#DIV/0!
Mir1981|100316821	-1	#DIV/0!	1	#DIV/0!
Olfr992|258865	-1	#DIV/0!	1	#DIV/0!
Cyp2b19|13090	-1	#DIV/0!	1	#DIV/0!
Olfr798|258549	-1	#DIV/0!	1	#DIV/0!
Cdh12|215654	-1	#DIV/0!	1	#DIV/0!
Vmn1r205|171251	-1	#DIV/0!	1	#DIV/0!
Slc16a8|57274	-1	#DIV/0!	1	#DIV/0!
Klra1|16627	-1	#DIV/0!	1	#DIV/0!
Vmn1r49|24112	-1	#DIV/0!	1	#DIV/0!
Olfr854|258515	-1	#DIV/0!	1	#DIV/0!
Olfr1241|258447	-1	#DIV/0!	1	#DIV/0!
Ftmt|67634	-1	#DIV/0!	1	#DIV/0!
Snord45b|100217468	-1	#DIV/0!	1	#DIV/0!
Spz1|79401	-1	#DIV/0!	1	#DIV/0!
Nptx2|53324	-1	#DIV/0!	1	#DIV/0!
Gm5382|385328	-1	#DIV/0!	1	#DIV/0!
Mir105|100124463	-1	#DIV/0!	1	#DIV/0!
Foxb1|64290	-1	#DIV/0!	1	#DIV/0!
Serpina9|71907	-1	#DIV/0!	1	#DIV/0!
C230004F18Rik|331424	-1	#DIV/0!	1	#DIV/0!
Klrb1f|232408	-1	#DIV/0!	1	#DIV/0!
Vmn1r65|81013	-1	#DIV/0!	1	#DIV/0!
Taar1|111174	-1	#DIV/0!	1	#DIV/0!
Dok6|623279	-1	#DIV/0!	1	#DIV/0!
AY026312|170939	-1	#DIV/0!	1	#DIV/0!
Snora68|104370	-1	#DIV/0!	1	#DIV/0!
Art2a-ps|11871	-1	#DIV/0!	1	#DIV/0!
Gm7534|665186	-1	#DIV/0!	1	#DIV/0!
Krtap9-3|75586	-1	#DIV/0!	1	#DIV/0!
Mir18b|100124431	-1	#DIV/0!	1	#DIV/0!
Otog|18419	-1	#DIV/0!	1	#DIV/0!
Bcl2a1c|12046	-1	#DIV/0!	1	#DIV/0!
Asphd2|72898	-1	#DIV/0!	1	#DIV/0!
4933433C11Rik|74472	-1	#DIV/0!	1	#DIV/0!
Defb8|244334	-1	#DIV/0!	1	#DIV/0!
Defb28|545475	-1	#DIV/0!	1	#DIV/0!
Serpinb6d|238568	-1	#DIV/0!	1	#DIV/0!
Elavl2|15569	-1	#DIV/0!	1	#DIV/0!

Cxx1b|553127	-1.000066526	0.499844477	-1.128214457	0.151627474
Zfp157|72154	-1.00010845	0.499852834	-1.134896732	0.320615039
Ipo11|76582	-1.000218376	0.499711918	-1.000218376	0.499711918
AW555464|217882	-1.000221163	0.499200363	-1.056670294	0.308015224
Vim|22352	-1.000253609	0.499539219	1.114859963	0.289526788
Ssna1|68475	-1.000297168	0.499426798	-1.000297168	0.499426798
Edem3|66967	-1.000298463	0.49959974	1.138846281	0.207946877
Chsy1|269941	-1.000407005	0.499309283	1.116992918	0.301937037
Tsc22d4|78829	-1.00046643	0.498644758	1.081896954	0.250196848
Rbp4|19662	-1.000487208	0.498831325	-1.214913736	0.056408459
Vps36|70160	-1.000505612	0.498913955	-1.222319584	0.086775891
Tgfa|21802	-1.000651406	0.499218477	1.188127614	0.312285685
Vcp|269523	-1.000657968	0.496564281	-1.046059295	0.24929852
Coro1c|23790	-1.000673177	0.49552627	1.030092969	0.306180462
Azgp1|12007	-1.000715132	0.496657422	-1.067191149	0.128027901
Pkhd1|241035	-1.000720585	0.4990249	1.168372411	0.306219536
2700062C07Rik|68046	-1.000730398	0.49836713	-1.122927186	0.242128499
Spr|20751	-1.000789767	0.498031101	-1.111618108	0.234221442
Nprl3|17168	-1.00081732	0.498039169	-1.081086797	0.307660993
G630090E17Rik|100041085	-1.000848682	0.497554482	-1.076853876	0.294164184
Myo1f|17916	-1.000858536	0.498901485	-1.44377103	0.0628428
Rexo2|104444	-1.000860816	0.497253829	-1.000860816	0.497253829
Gm5803|545091	-1.000875788	0.498842804	-1.000875788	0.498842804
Mcfd2|193813	-1.000897099	0.497748501	1.103535465	0.217070057
Mxi1|17859	-1.000953992	0.497366197	1.030226397	0.405419999
Mef2c|17260	-1.000991693	0.499409403	-1.700998606	0.240444782
Aqp1|11826	-1.001016315	0.496913996	-1.146561929	0.109328035
Mettl13|71449	-1.00107397	0.497972294	-1.106306348	0.30027142
Tmem9|66241	-1.001166809	0.494794028	-1.050003087	0.273428207
Cebpz|12607	-1.001296526	0.495553921	-1.129222044	0.112964064
Zfp619|70227	-1.001316765	0.499463222	-2.76078	0.173296754
Npc1|18145	-1.001408403	0.49721009	-1.001408403	0.49721009
Vnn3|26464	-1.001493301	0.495142284	-1.150893096	0.065862662
41154|18000	-1.001523609	0.496098885	1.087309968	0.292225983
Smc1b|140557	-1.001550199	0.499319463	-3.98415	0.093264411
Itgb1bp2|26549	-1.001550199	0.499319463	-3.98415	0.093264411
Hmmr|15366	-1.001550199	0.499319463	-3.98415	0.093264411
Ovgp1|12659	-1.001550199	0.499319463	-3.98415	0.093264411
Cd244|18106	-1.001550199	0.499319463	-3.98415	0.093264411
Zmynd8|228880	-1.001571959	0.494895222	1.106010217	0.122645768
Pex11c|69129	-1.001580811	0.495429204	-1.001580811	0.495429204
Rpl4|67891	-1.001636007	0.496220254	-1.097416854	0.295104997
Wdfy3|72145	-1.001763286	0.496938169	-1.001763286	0.496938169
Slc40a1|53945	-1.001786444	0.493545573	1.057242873	0.192784325
Cdc42|12540	-1.001792823	0.493988496	1.091988655	0.176689411
Phldb3|232970	-1.001883504	0.496270761	1.109868345	0.179629958
Ddc|13195	-1.001913376	0.493945276	-1.001913376	0.493945276
Mgat4b|103534	-1.002025274	0.493559484	1.048539685	0.359065879
Adipor1|72674	-1.002038738	0.492541066	-1.053712573	0.30690125
Usp36|72344	-1.002054574	0.49567669	1.143261409	0.209866405
Aftph|216549	-1.002068454	0.492971397	1.074737636	0.249622716
Zswim7|69747	-1.002192369	0.495347912	-1.028114871	0.427728672
Slc25a1|13358	-1.00221639	0.489721149	-1.110155581	0.033247589

Cuta|67675	-1.002326469	0.494703293	1.00391699	0.487509962
Mad1l1|17120	-1.002371579	0.495135588	1.076894819	0.347563246
Nsfl1c|386649	-1.002373625	0.488776394	1.038344168	0.319081041
Ripk1|19766	-1.00244911	0.492847186	1.057787546	0.345515154
4632428N05Rik|74048	-1.002487382	0.495430622	-1.267420751	0.096080638
Smad1|17125	-1.002502256	0.492555814	1.068511153	0.309371298
Erap1|80898	-1.002514882	0.495889479	-1.009787561	0.482809225
Sf3b1|81898	-1.002563151	0.495341042	1.296008839	0.047219017
Oxa1l|69089	-1.002588451	0.49078603	-1.002588451	0.49078603
Eif4enif1|74203	-1.00261785	0.494402485	1.133094396	0.228528125
Tcf21|21412	-1.00262964	0.496322614	-1.201928702	0.25622666
Plek|56193	-1.002650222	0.498290264	1.861388373	0.151782945
Homer2|26557	-1.002685029	0.492999749	-1.088412459	0.287685599
Spsb1|74646	-1.002685861	0.496313678	1.163540529	0.266966271
Mllt11|56772	-1.002721251	0.496954398	1.20185781	0.314002842
Sertad2|58172	-1.002775507	0.494977328	-1.184834716	0.185679231
H13|14950	-1.002868591	0.492642546	-1.000184767	0.499374015
Tomm40|53333	-1.002887264	0.491960764	-1.002887264	0.491960764
E2f2|242705	-1.002944512	0.498061741	-1.603370829	0.237225529
Lsm6|78651	-1.00295136	0.493311924	-1.204997494	0.10910073
Nudt19|110959	-1.003096579	0.488712668	-1.042049081	0.312645531
Cnksr3|215748	-1.003106969	0.493053537	-1.119046605	0.214081735
Hps4|192232	-1.003204235	0.491341586	1.174815916	0.079219119
Fam165b|68936	-1.003266709	0.493480639	-1.003266709	0.493480639
Chmp2a|68953	-1.003293234	0.492541881	-1.097096348	0.301487875
Higd1a|56295	-1.003348362	0.486688198	-1.05860195	0.279427651
Ccdc71|72454	-1.003361573	0.475710699	1.03266187	0.250879292
Mkl1|223701	-1.003371889	0.492384661	1.027191099	0.395537335
Rpain|69723	-1.003379083	0.490231742	-1.177183732	0.055692167
Arrb1|109689	-1.003397991	0.48800402	-1.124907627	0.086258535
Rps29|20090	-1.003399329	0.497263414	1.513614703	0.19563049
Slc22a17|59049	-1.003462187	0.495572469	-1.268226212	0.204154455
Cyth2|19158	-1.003495377	0.489287569	1.077218322	0.270965662
Safb|224903	-1.003621243	0.487976637	-1.003621243	0.487976637
Eprs|107508	-1.003641391	0.478151067	-1.03412978	0.308108135
Vtn|22370	-1.003660733	0.493592133	-1.0690491	0.324556222
Krtcap2|66059	-1.003711782	0.491370532	-1.099900381	0.288742468
Slc25a40|319653	-1.003738992	0.497696609	1.24673798	0.369951756
Tceal8|66684	-1.003775473	0.49155846	-1.232973839	0.046113672
Mob2|101513	-1.003822088	0.492819913	-1.29925595	0.036873389
H2-K1|14972	-1.003844639	0.484501754	-1.103875929	0.104065938
2310022A10Rik|66367	-1.003905446	0.487147125	-1.003905446	0.487147125
Smad5|17129	-1.003927879	0.486638693	-1.071785495	0.269489861
BC048355|381101	-1.003933192	0.489366134	1.069850374	0.309782722
Kctd17|72844	-1.003956751	0.492881503	-1.099582027	0.326710785
Srgap3|259302	-1.004020067	0.494916438	-1.191354084	0.297349342
Tnfaip1|21927	-1.00402644	0.482543242	1.037718634	0.338024654
Suclg1|56451	-1.004030922	0.479769542	-1.060505455	0.190681382
Smarcad1|13990	-1.004054115	0.493562307	1.188259586	0.227318789
Farp1|223254	-1.004193616	0.490301945	1.10283179	0.276904897
Ptma|19231	-1.004213097	0.482604835	1.012370736	0.432767501
Cntfr|12804	-1.004238295	0.492006941	-1.004238295	0.492006941
Gm9199|668489	-1.004241094	0.497125346	1.4358054	0.292430042

Rbm38|56190	-1.004265215	0.495014538	-1.166964687	0.301079282
Zbtb11|271377	-1.004282359	0.495841806	-1.608909321	0.087823726
Itfg2|101142	-1.004407687	0.477330411	1.023929788	0.381683476
Zbtb7a|16969	-1.004443028	0.488164356	1.004097473	0.481982178
Gm3258|100041294	-1.004447061	0.488028887	-1.031139544	0.425632727
Creb1|12912	-1.004455057	0.494373861	1.157521577	0.298848024
Stk40|74178	-1.004468872	0.474491835	-1.049539916	0.219111944
Zfp408|381410	-1.004621975	0.486821125	-1.002030616	0.492902673
Rrp8|101867	-1.004693984	0.482513265	1.001765374	0.492004283
Prcc|94315	-1.004704021	0.488132509	-1.105368538	0.25196378
Abca5|217265	-1.004712664	0.495080511	-1.004712664	0.495080511
Rasl11b|68939	-1.004743649	0.491080912	-1.004743649	0.491080912
Mrpl15|27395	-1.004767813	0.487610051	-1.080190937	0.195314422
Ccdc107|622404	-1.004768798	0.491166436	1.010749385	0.474893515
Abcd4|19300	-1.004952128	0.489533369	-1.004952128	0.489533369
Eaf1|74427	-1.00496096	0.493846542	1.44431709	0.072330542
Cad|69719	-1.005105505	0.487347598	-1.074996929	0.333799137
Efr3a|76740	-1.005119537	0.483366453	-1.084411046	0.237197706
Agpat3|28169	-1.005157137	0.464446231	-1.005157137	0.464446231
Ddx46|212880	-1.005165165	0.492405886	1.23191265	0.185198703
Mamld1|333639	-1.005226199	0.496586031	-1.547243792	0.231027677
Klf2|16598	-1.005244234	0.490153425	1.177703973	0.17129259
Calcoco1|67488	-1.005303857	0.474131461	-1.040449627	0.158845321
Pnpla7|241274	-1.005330369	0.483721783	1.013464733	0.444272944
Fcgrt|14132	-1.005346174	0.483919699	1.011763648	0.454540964
2510002D24Rik|72307	-1.005479453	0.486636032	-1.005479453	0.486636032
A430105I19Rik|214239	-1.005481691	0.493422939	-1.20292438	0.246808271
Gm11127|100529082	-1.005529388	0.489703091	-1.04273492	0.404958942
Shc1|20416	-1.005534575	0.472573697	1.069618892	0.059445749
Reps1|19707	-1.005547823	0.489534985	1.067531967	0.387604509
Pogk|71592	-1.005634368	0.492541136	1.409000647	0.072485385
Hmgn2|15331	-1.005679914	0.487595996	1.088243088	0.304004914
Fuk|234730	-1.005711618	0.488224239	-1.005711618	0.488224239
AI316807|102032	-1.005731138	0.474432162	1.044680598	0.303045346
2310001A20Rik|71881	-1.00580067	0.481027988	-1.104701764	0.092653545
Jmjd7|433466	-1.005888354	0.494346762	-1.312887705	0.264402531
Smtnl2|276829	-1.005955983	0.496961328	-1.893566965	0.212929597
Tmx1|72736	-1.005963394	0.485332464	1.04400405	0.402957044
Stard5|170460	-1.00597889	0.48020652	1.142560385	0.064095887
Tap1|21354	-1.006063574	0.488058838	-1.054915067	0.363877177
Zmat2|66492	-1.00610091	0.469402439	-1.044076894	0.294106453
5430417L22Rik|100043272	-1.006186049	0.486170912	1.045297317	0.360618876
Plcg1|18803	-1.006225527	0.480129382	-1.005824745	0.468351556
Nudt2|66401	-1.00642425	0.488160665	-1.00642425	0.488160665
40973|69104	-1.006461732	0.4739953	-1.051847788	0.297880094
Dtnbp1|94245	-1.006494891	0.485420699	-1.096734356	0.305637498
Afg3l1|114896	-1.006508274	0.464773486	-1.058749659	0.154225715
Ppfia1|233977	-1.006521577	0.489945557	1.228435476	0.164804769
D10Wsu102e|28109	-1.006563821	0.483748351	-1.094917726	0.28628946
Net1|56349	-1.006586728	0.485200625	-1.107878909	0.280690559
Trpc4ap|56407	-1.006600065	0.475542596	-1.077544409	0.221611757
Mpp6|56524	-1.006602048	0.468697618	-1.108283012	0.029277064
Arih1|23806	-1.006613601	0.483881108	1.116175241	0.217006068

Crcp|12909	-1.006657366	0.483102258	1.072279555	0.313805924
Lsg1|224092	-1.006674345	0.479298229	-1.061287148	0.317531671
Ndufs2|226646	-1.006706705	0.44252716	1.015392967	0.368504584
Uevld|54122	-1.006744638	0.494372074	1.44731878	0.21808598
Chuk|12675	-1.006773476	0.487171165	1.132163939	0.268015594
Rmnd1|66084	-1.006829668	0.485964202	-1.006829668	0.485964202
Syn1|20964	-1.006832031	0.49397609	-1.006832031	0.49397609
Slc25a28|246696	-1.006845945	0.478599374	-1.006845945	0.478599374
Slc16a6|104681	-1.006872247	0.492581533	1.512617567	0.090170125
Casc3|192160	-1.0068954	0.48090795	1.159144594	0.104856387
Apol7e|666348	-1.006925777	0.496790846	-1.7804	0.173296754
Dync1i2|13427	-1.006979379	0.48703788	-1.101585788	0.335281409
4833439L19Rik|97820	-1.007010077	0.478501694	1.058973948	0.315474941
Pglyrp1|21946	-1.007207676	0.49720062	-1.262599539	0.402575049
Smarca5|93762	-1.007319086	0.483527972	1.112453029	0.214658655
Btbd7|238386	-1.00734753	0.494312112	1.364669794	0.28997094
Gm11428|100034251	-1.007443553	0.489029797	1.095504804	0.364125115
Gm15455|433287	-1.007478691	0.485500489	-1.007478691	0.485500489
Inppl1|16332	-1.007530072	0.463035614	-1.05319658	0.25219222
Mthfsd|234814	-1.007540877	0.478627143	-1.180489471	0.063525568
Calu|12321	-1.007684971	0.472476015	-1.007684971	0.472476015
Gpr116|224792	-1.007687937	0.481311207	1.229078454	0.007552908
Trim8|93679	-1.007695664	0.45573622	1.077459466	0.053787025
Ap4m1|11781	-1.007757152	0.490833234	1.211464932	0.190855277
Pphln1|223828	-1.007791342	0.481531491	1.192296116	0.090112107
Zfp563|240068	-1.007817873	0.490577822	-1.007817873	0.490577822
Mier2|70427	-1.007829878	0.478413542	-1.05639247	0.325327492
Ap2b1|71770	-1.007845083	0.467824705	1.113627837	0.051363693
Bnip2|12175	-1.007864706	0.481148443	-1.007864706	0.481148443
Ciita|12265	-1.007881731	0.495970361	2.293962892	0.168028304
Cd68|12514	-1.007937151	0.483828659	-1.009897125	0.466951887
Pdp1|381511	-1.007989058	0.49674815	-2.8	0.173296754
Pcdhb18|93889	-1.007989058	0.49674815	-2.8	0.173296754
Ythdf1|228994	-1.008058645	0.476089848	-1.008058645	0.476089848
Perp|64058	-1.008072664	0.465497034	-1.008072664	0.465497034
Mkks|59030	-1.008120597	0.467784228	1.104520192	0.101116017
Ifngr1|15979	-1.008212568	0.481892955	-1.153291744	0.081645806
Alg2|56737	-1.008218195	0.483620784	-1.008218195	0.483620784
9330111N05Rik|319983	-1.008223578	0.482325407	-1.008223578	0.482325407
Azi1|12009	-1.008247043	0.483468703	-1.008247043	0.483468703
Fbxl6|30840	-1.008313642	0.469174908	-1.008313642	0.469174908
Nolc1|70769	-1.008340387	0.467169367	1.053450771	0.287983637
Herpud2|80517	-1.008351306	0.486209054	-1.078222689	0.359186483
Rap2a|76108	-1.00835702	0.487076361	-1.169661775	0.272546932
Ube2q1|70093	-1.008395697	0.455080367	-1.008395697	0.455080367
Zbtb22|81630	-1.008407524	0.469649596	1.068294638	0.243410145
Dhcr7|13360	-1.008432459	0.471882753	1.068039852	0.272432954
Retsat|67442	-1.008517458	0.479734011	1.08407342	0.312904356
Maea|59003	-1.008524772	0.458604409	-1.008524772	0.458604409
Zfp330|30932	-1.00862235	0.475352722	1.021831495	0.386637023
Zfp707|69020	-1.008671119	0.468501731	-1.136824935	0.073691269
Psmd5|66998	-1.008684934	0.481515897	1.030733926	0.414965323
Pafah1b2|18475	-1.0086924	0.47277594	-1.064079474	0.307209424

Cpsf4|54188	-1.008771274	0.469599481	1.110474353	0.135973626
Slc7a6os|66432	-1.008821414	0.473937387	1.158475362	0.063107374
Brms1|107392	-1.008990809	0.477691375	-1.008990809	0.477691375
Polr2c|20021	-1.008994147	0.478031762	-1.008994147	0.478031762
Lig1|16881	-1.009064273	0.488751035	1.177050028	0.313142235
Rrp1b|72462	-1.009113516	0.485730659	-1.282282662	0.075238759
Sgk2|27219	-1.009141614	0.466415221	-1.071629243	0.253371483
Cadm1|54725	-1.009157193	0.474035898	-1.189033424	0.035811804
Rictor|78757	-1.009213523	0.489751268	-1.009213523	0.489751268
Mrpl41|107733	-1.009237732	0.479859282	1.077729428	0.32932327
Cish|12700	-1.009260825	0.467720744	1.005981904	0.474526321
Rrn3|106298	-1.009269206	0.475023161	1.100342016	0.223156962
Sema5a|20356	-1.009316207	0.493175485	-1.009316207	0.493175485
Pnp|18950	-1.009414856	0.464214707	1.106152126	0.123145585
Sertad3|170742	-1.009444426	0.474972364	1.103209936	0.219458042
Chrna2|110902	-1.009476801	0.473963745	1.093625875	0.198134428
Golph3|66629	-1.00949447	0.484225661	1.10317045	0.32813769
Ninj1|18081	-1.009526838	0.480259799	-1.04013316	0.384804368
Polr2b|231329	-1.009533628	0.477952414	-1.225124952	0.04629953
Gmds|218138	-1.009567421	0.488835746	1.103543446	0.380204109
Car5b|56078	-1.00959325	0.486194264	-1.00959325	0.486194264
Pygb|110078	-1.009624394	0.482247518	1.176728191	0.174616499
Hs3st3a1|15478	-1.009740329	0.49161455	-1.426193587	0.17974999
Elmo1|140580	-1.009752622	0.487916055	1.46414219	0.037355499
Mastl|67121	-1.009766255	0.495725951	-4.016833333	0.093227909
2310007B03Rik|71874	-1.009766255	0.495725951	-4.016833333	0.093227909
Lekr1|624866	-1.009766255	0.495725951	-4.016833333	0.093227909
Vdr|22337	-1.009766255	0.495725951	-4.016833333	0.093227909
Fam184a|75906	-1.009766255	0.495725951	-4.016833333	0.093227909
Wfdc3|71856	-1.009766255	0.495725951	-4.016833333	0.093227909
Ces3b|13909	-1.009840001	0.473003427	-1.018160818	0.425719581
Sat2|69215	-1.00988868	0.473805042	-1.00988868	0.473805042
Zdhhc14|224454	-1.010057265	0.480791161	-1.270652504	0.080180145
Sdhd|66925	-1.010094555	0.447414048	1.02754111	0.358182858
Eri3|140546	-1.010181752	0.472900441	-1.128917929	0.16505362
Camk2n2|73047	-1.010189176	0.480524848	1.112712849	0.301726765
Sec11c|66286	-1.010200629	0.48111833	-1.181417689	0.190224716
Thap4|67026	-1.010231995	0.467207911	-1.010231995	0.467207911
Zmym3|56364	-1.010283182	0.480503606	-1.123950544	0.293048896
0610007P22Rik|68327	-1.010295566	0.472479474	-1.010295566	0.472479474
Ivns1abp|117198	-1.010362206	0.479872257	1.156125847	0.198975504
Tmsb4x|19241	-1.01036414	0.485960721	-1.362116403	0.042575778
Gnb5|14697	-1.010429306	0.491804739	-1.448134364	0.244553918
Trim34a|94094	-1.010432839	0.4863821	-1.278957027	0.185288458
Rab13|68328	-1.010540789	0.486354442	-1.292437486	0.171597439
Rnasel|24014	-1.01054626	0.485389094	-1.185906281	0.281553993
Mrpl48|52443	-1.010564348	0.477733777	1.101927616	0.29892755
Usp5|22225	-1.010578121	0.451356726	-1.010578121	0.451356726
Tbc1d15|66687	-1.010607846	0.479433835	-1.029562495	0.424942879
Kat2b|18519	-1.010662626	0.482854791	-1.010662626	0.482854791
Mtmr11|194126	-1.010702147	0.490340785	-1.702306718	0.071330792
Arfrp1|76688	-1.010749603	0.465990041	-1.010749603	0.465990041
Gfpt1|14583	-1.010754202	0.486867317	1.108217012	0.350640912

Snx3|54198	-1.010837684	0.424786927	1.022789696	0.331579577
Reps2|194590	-1.010860351	0.490728802	1.372462499	0.184389685
Nfatc3|18021	-1.010942744	0.479416686	1.031701873	0.424961117
41159|235072	-1.010977346	0.482275906	1.18415345	0.162441895
Alg1|208211	-1.010995208	0.433315221	-1.053503304	0.186001788
Taldo1|21351	-1.011085288	0.4631845	1.11610248	0.139808752
Klf3|16599	-1.011091687	0.477146299	-1.173993203	0.158472359
Aven|74268	-1.011092413	0.475603361	-1.045606609	0.411736893
D16H22S680E|27883	-1.011137632	0.471746208	1.098621312	0.207998184
Dffb|13368	-1.011137852	0.48564645	-1.011137852	0.48564645
Txnl1|53382	-1.011168442	0.464829822	-1.040378178	0.328966397
Slc35b1|110172	-1.011261066	0.476969655	1.122473778	0.259672076
Zfp41|22701	-1.011298127	0.491012579	-1.012545531	0.488901211
Irf2bp1|272359	-1.011366562	0.465917502	-1.011366562	0.465917502
Slc17a2|218103	-1.011426119	0.466629606	-1.119183992	0.158174973
Plcl2|224860	-1.011431245	0.466587483	1.0640953	0.301651934
Rab40c|224624	-1.011460157	0.463890815	1.131556953	0.101257686
Zfp644|52397	-1.011473142	0.486345522	-1.181606569	0.285317452
Srfbp1|67222	-1.011543527	0.466398561	-1.011543527	0.466398561
Aspscr1|68938	-1.011551306	0.471621369	1.046041781	0.390580522
Stx17|67727	-1.011574175	0.469635313	-1.011574175	0.469635313
Gm4902|236451	-1.011630684	0.486758837	-1.011630684	0.486758837
Mt1|17748	-1.011645435	0.485953782	1.02881185	0.460829401
Hsd17b7|15490	-1.011653459	0.484431635	-1.011653459	0.484431635
Tspan4|64540	-1.011670224	0.468998748	1.110014188	0.186350872
Nol9|74035	-1.011755953	0.404023893	-1.040787845	0.173111105
Fkbp9|27055	-1.01175796	0.468687234	-1.04495142	0.390633806
Nfx1|74164	-1.011807748	0.463324185	-1.000492883	0.498017431
Mrpl21|353242	-1.011888964	0.462785883	-1.072674757	0.29403014
Eml3|225898	-1.011893878	0.453283248	-1.03250026	0.302971693
Ggnbp2|217039	-1.011941954	0.472172675	-1.102164039	0.263336831
Zfp275|27081	-1.012013328	0.483943853	-1.190344854	0.236617109
Insl3|16336	-1.012078886	0.49302935	-2.019042817	0.167534604
Tfap4|83383	-1.012247587	0.469525516	-1.005965192	0.48175261
Tmem9b|56786	-1.012306623	0.47802948	-1.221233077	0.124904917
Gstk1|76263	-1.012316897	0.415854235	1.025141178	0.298354048
C1d|57316	-1.012324729	0.455088926	-1.012324729	0.455088926
Spata13|219140	-1.012332674	0.477892227	1.124481784	0.181258025
Psmd11|69077	-1.012351845	0.446164684	1.026670238	0.383245877
Rps7|20115	-1.012406672	0.468583534	-1.204245291	0.06613148
Impg2|224224	-1.012461286	0.488263003	-1.375225352	0.225993519
Cxxc1|74322	-1.012476915	0.446998324	1.04495688	0.302164719
Acadl|11363	-1.012530841	0.4618299	1.057571486	0.330368047
Psmd10|53380	-1.012561445	0.470279478	1.097547789	0.274645469
Il17rd|171463	-1.012577145	0.490620378	1.641166232	0.156742294
Abhd1|57742	-1.012614634	0.48851706	-1.012614634	0.48851706
Pcbp1|23983	-1.012618765	0.460840027	-1.012618765	0.460840027
Zfp575|101544	-1.012753364	0.480269527	-1.080854246	0.363312849
Lrrk1|233328	-1.01283419	0.47284884	1.076668999	0.335451393
U2af1|108121	-1.012934981	0.476337352	-1.012934981	0.476337352
Ccdc123|72140	-1.012947772	0.471625414	-1.012947772	0.471625414
Zfp759|268670	-1.012963073	0.487154047	-1.205257318	0.300335651
Brd2|14312	-1.012970508	0.444140308	-1.067772423	0.206431016

Gdi1|14567	-1.013046894	0.436835483	-1.00343735	0.477931405
Osbpl8|237542	-1.013172667	0.476762823	1.225888836	0.061643041
Hunk|26559	-1.013255419	0.482969702	1.425147735	0.063021599
Map2k2|26396	-1.013258331	0.47057996	-1.112106215	0.277814314
Sepp1|20363	-1.013279145	0.447406578	1.028593921	0.391823304
Rdh14|105014	-1.013387644	0.474967253	-1.277318642	0.072642727
Tmcc1|330401	-1.013389702	0.457655461	1.044081579	0.35869808
Ilf3|16201	-1.013447516	0.454782438	1.078536861	0.206380588
Fkbpl|56299	-1.013453705	0.481449649	-1.219779263	0.241490525
Sil1|81500	-1.013454137	0.474672688	-1.013454137	0.474672688
Adcy4|104110	-1.013459292	0.470017816	-1.244664208	0.04676644
Brwd3|382236	-1.01348546	0.484017236	1.460486548	0.05979764
Ftsj1|54632	-1.01350826	0.436708369	-1.047238516	0.300044646
Pde9a|18585	-1.013670537	0.460152573	-1.111878863	0.189571792
1200014J11Rik|66874	-1.013680696	0.468398414	1.128684786	0.185540899
Rac1|19353	-1.013689095	0.404354604	-1.041771921	0.222798497
2310046K01Rik|69698	-1.013693919	0.490677745	-1.722583671	0.178732435
Stk35|67333	-1.013997551	0.467479977	1.026059939	0.443086131
4921524J17Rik|66714	-1.014007703	0.482327315	-1.501518454	0.0324447
Ces1d|104158	-1.014091084	0.464194493	-1.211696577	0.055225605
Atp2c1|235574	-1.014113794	0.474304689	1.120152458	0.297132694
Phb2|12034	-1.014150996	0.428298976	-1.042224913	0.307636692
Lsm12|268490	-1.014215253	0.462801055	-1.014215253	0.462801055
Etnk2|214253	-1.014315724	0.43392061	-1.038118393	0.305639334
Pabpc4|230721	-1.014349977	0.428497864	1.031909685	0.323921038
Aim1|11630	-1.014358561	0.474340988	-1.099105496	0.332593106
Gpr56|14766	-1.014397923	0.468268044	-1.248523124	0.045127837
1810019J16Rik|69073	-1.014401365	0.45099923	-1.014401365	0.45099923
2610027L16Rik|67842	-1.014448559	0.427770249	1.003030951	0.479657866
Chchd3|66075	-1.014448899	0.441521888	1.043097236	0.321267852
Ntng2|171171	-1.014462835	0.493334813	-1.722963636	0.273335094
Chek2|50883	-1.014496383	0.489544714	-2.056027442	0.059523874
Dbn1|56320	-1.014515795	0.48263683	-1.132796966	0.350466069
Trit1|66966	-1.014528272	0.466730083	-1.033315103	0.375452876
Tor2a|30933	-1.014534734	0.449044015	-1.081206809	0.236113906
Cebpe|110794	-1.014615898	0.476374771	-1.002946627	0.494693598
Arl10|56795	-1.014633093	0.487425768	1.049882659	0.437044848
Psmd3|22123	-1.014641834	0.453589576	-1.060533174	0.32912327
Myh10|77579	-1.014670007	0.481765953	1.233396622	0.24458571
Psmb1|19170	-1.014818899	0.453358142	-1.180058068	0.028582581
41161|53860	-1.014897399	0.431860503	-1.004765894	0.470435298
4632415L05Rik|70808	-1.014923466	0.469686531	-1.014923466	0.469686531
Amd1|11702	-1.014927773	0.468462	1.069462241	0.36554785
Pex1|71382	-1.014983445	0.468209965	1.045967747	0.411916032
Dhx57|106794	-1.015005079	0.476381426	-1.183973157	0.189943619
Gck|103988	-1.015029915	0.484475162	-1.241606208	0.288608638
Rbx1|56438	-1.015167791	0.429248349	-1.02287842	0.362436391
Gabarap|56486	-1.015255484	0.461977821	-1.117320345	0.232308309
Ovca2|246257	-1.015280094	0.435037295	-1.088207904	0.088682864
Akirin1|68050	-1.015288116	0.452967877	-1.010297535	0.461362748
Cd59b|333883	-1.015396651	0.478602573	1.139310071	0.330291212
Enpp3|209558	-1.015418678	0.464785205	-1.151501774	0.174579021
Ssu72|68991	-1.015485635	0.421048851	-1.065404745	0.163043956

Gm16517|110012	-1.015543835	0.470016735	-1.015543835	0.470016735
Plbd2|71772	-1.01554994	0.457085642	-1.099280814	0.250377964
Tfam|21780	-1.01555586	0.456650648	1.064209729	0.325747969
Slc27a5|26459	-1.015764689	0.418349162	-1.067371902	0.143102448
Atp13a1|170759	-1.015770134	0.424763259	-1.015770134	0.424763259
Ccdc142|243510	-1.015912875	0.434845931	-1.080259792	0.160962123
Aip|11632	-1.015915485	0.455687456	-1.207549184	0.024152885
Maf1|68877	-1.015937632	0.460300874	-1.015937632	0.460300874
Ctnna1|12385	-1.015977726	0.451456098	1.125353416	0.141448221
Npy1r|18166	-1.015986716	0.492263219	-2.098176768	0.227724758
Ilf2|67781	-1.016083643	0.458375643	-1.1990108	0.056818032
Fam3a|66294	-1.016120499	0.44848018	-1.060825354	0.318729957
Mtpap|67440	-1.016169221	0.469422954	1.063331713	0.382603853
Bysl|53414	-1.016223913	0.443461886	1.063139893	0.234752158
Timm44|21856	-1.016289743	0.434522443	1.021156029	0.415553008
Puf60|67959	-1.016364662	0.418355411	-1.048402773	0.282531247
Arf3|11842	-1.016373691	0.466113632	1.027721028	0.412778226
Snw1|66354	-1.016385322	0.428304239	-1.072512607	0.202700872
Crem|12916	-1.016387365	0.473953622	-1.340753796	0.076745199
Pld2|18806	-1.016416704	0.474890679	-1.210839036	0.188944119
Efha1|68514	-1.016454764	0.417737305	-1.11238006	0.037518934
Galnt14|71685	-1.016474095	0.491610936	-1.750086636	0.276292757
Ptgds|19215	-1.016475262	0.484900046	-1.016475262	0.484900046
Ndufa7|66416	-1.01648804	0.45573259	-1.200379663	0.052900578
Oplah|75475	-1.016506243	0.399882909	-1.021915469	0.293075777
Dhx37|208144	-1.016535922	0.464015783	-1.116006394	0.275979529
Cdc37|12539	-1.016548695	0.467918356	-1.014912962	0.46137081
Tmem222|52174	-1.016556404	0.463250024	-1.09915089	0.306207986
Gnpat|14712	-1.016585078	0.427223074	-1.072437747	0.202405174
Miip|28010	-1.016652706	0.45880521	1.068694842	0.33785042
Itprip|414801	-1.016835285	0.475761984	-1.440364137	0.022395959
Uqcc|56046	-1.016880499	0.407937	-1.061501212	0.186030105
Wdr74|107071	-1.016913477	0.457019588	-1.016913477	0.457019588
Osbpl9|100273	-1.016997357	0.423528894	-1.048544534	0.297918709
Sema4b|20352	-1.017073415	0.453493895	-1.017073415	0.453493895
Cchcr1|240084	-1.017076273	0.479141015	1.281738778	0.187172756
Aga|11593	-1.017077579	0.447887608	1.127312955	0.130688684
Yipf4|67864	-1.017085053	0.460939017	1.096654103	0.282371617
Lrrc8a|241296	-1.01713364	0.447985291	-1.115855365	0.163502237
Ankfy1|11736	-1.017182518	0.466655192	1.242442593	0.078035127
Adam23|23792	-1.017222936	0.462914574	1.056033686	0.389614636
Tmub2|72053	-1.017250255	0.416641964	1.005783185	0.473625778
C1qc|12262	-1.017252378	0.45699747	-1.144003493	0.158812618
Sars|20226	-1.017460537	0.442874267	-1.077895645	0.26988268
Ctr9|22083	-1.017508647	0.449024999	1.065135546	0.288477475
Il1rap|16180	-1.017586978	0.460606223	-1.017586978	0.460606223
Casp9|12371	-1.017592248	0.456729312	-1.017592248	0.456729312
Bcl7b|12054	-1.017599161	0.440710257	-1.096295309	0.163045245
Dot1l|208266	-1.017613234	0.476640291	1.056557752	0.404781714
Mrpl27|94064	-1.017616763	0.430658997	-1.054513251	0.308433611
Fahd1|68636	-1.017786516	0.456822252	-1.076796583	0.156163184
Aplp1|11803	-1.017790247	0.490679887	-1.238100091	0.415076597
Trim24|21848	-1.017859442	0.461590776	-1.13896577	0.18631951

Snurf|84704	-1.017972742	0.487159005	-1.017972742	0.487159005
Psd4|215632	-1.017984988	0.464195902	-1.033978063	0.399096328
Prss8|76560	-1.018055057	0.483379467	-1.436967838	0.191906697
Rgs1|50778	-1.018099729	0.486600934	-1.018099729	0.486600934
S100a16|67860	-1.018122074	0.470598031	-1.118246988	0.23887655
Napepld|242864	-1.018192992	0.490400583	-1.571790977	0.158746995
Arhgef26|622434	-1.018232715	0.459367007	-1.232870483	0.080369388
Fars2|69955	-1.018264583	0.461443518	1.047541375	0.355101916
Mdp1|67881	-1.018294484	0.460185619	-1.133194777	0.20025742
Acp1|11431	-1.018326131	0.413009277	-1.010126093	0.422332551
Nif3l1|65102	-1.018377676	0.449455532	-1.134807836	0.143696638
Mtmr4|170749	-1.01841746	0.453358192	-1.01841746	0.453358192
Uhrf1bp1l|75089	-1.018511651	0.457099995	1.054722811	0.372393401
Degs1|13244	-1.018517325	0.433321781	1.062715508	0.245723487
Polr2j|20022	-1.018544277	0.462435805	1.128120726	0.239339535
Zswim5|74464	-1.018854065	0.484475875	1.911973184	0.00861303
Elof1|66126	-1.018957795	0.467432613	-1.006310242	0.484193251
Slc4a2|20535	-1.019032791	0.317550544	-1.019032791	0.317550544
Cyhr1|54151	-1.019039236	0.440804527	-1.099182908	0.215886249
Ufm1|67890	-1.019100852	0.433652981	1.057110159	0.287657899
Tmed3|66111	-1.019116794	0.467280211	1.156097836	0.241217187
Nt5dc3|103466	-1.019120915	0.461049725	-1.019120915	0.461049725
Arrdc2|70807	-1.019138834	0.475144162	-1.019138834	0.475144162
Scp2|20280	-1.019155825	0.432652535	-1.154471539	0.054275603
Slc35b2|73836	-1.019174686	0.406965635	-1.019174686	0.406965635
Lcorl|209707	-1.019209648	0.490846068	-1.162032469	0.433589133
Brcc3|210766	-1.019292389	0.46885037	-1.232332043	0.163227775
C230096C10Rik|230866	-1.019304824	0.456971917	-1.16418641	0.156888728
Ulk3|71742	-1.01936618	0.462430927	-1.274968752	0.072231961
Dst|13518	-1.019423187	0.425181402	1.106807972	0.067949743
Ppp2r1b|73699	-1.019471027	0.458592069	-1.038299953	0.369585078
Fastk|66587	-1.019533641	0.398032218	-1.019533641	0.398032218
Serinc5|218442	-1.019582587	0.482127474	1.479551381	0.137271807
Zfp329|67230	-1.019615262	0.479803571	-1.219391763	0.275616332
Ikzf5|67143	-1.019642888	0.476541547	-1.019642888	0.476541547
Arhgef25|52666	-1.019673461	0.475833314	1.1731677	0.268103729
F2|14061	-1.01968402	0.430066877	-1.157615591	0.015133252
1500011H22Rik|68948	-1.01969598	0.475443605	1.012060357	0.475150693
C130036L24Rik|319336	-1.019839084	0.480998591	-1.41624733	0.190406543
Cdk6|12571	-1.019849315	0.478774524	1.19550285	0.324427888
Ctdsp2|52468	-1.019860158	0.401033091	-1.082078996	0.103393156
Galk1|14635	-1.019893628	0.460426658	-1.08875349	0.258909151
Spnb3|20743	-1.019948706	0.415716107	-1.019948706	0.415716107
Smarcb1|20587	-1.019995159	0.437402138	-1.168620049	0.066757765
Fam89b|17826	-1.020091228	0.438274556	-1.020091228	0.438274556
Top1|21969	-1.020171427	0.46966798	1.358948711	0.028971237
Tgs1|116940	-1.020268447	0.458131851	1.131362384	0.218893067
Trip6|22051	-1.020340923	0.43035838	1.050861065	0.301028654
Spred1|114715	-1.02034746	0.484976865	-1.365186658	0.303152818
Pitpnm2|19679	-1.020508612	0.432976129	-1.180191325	0.02300071
Txndc12|66073	-1.020545261	0.436688825	-1.025843726	0.394070862
Clec4a2|26888	-1.020608257	0.483173358	-1.398609928	0.259634157
Sh3bgrl3|73723	-1.020716537	0.463491792	-1.068223706	0.33636104

Dnajc17|69408	-1.020753602	0.462162691	-1.020753602	0.462162691
Use1|67023	-1.020754669	0.458151751	-1.166265815	0.196446585
Mosc1|66112	-1.020785253	0.419903587	-1.083048125	0.181760837
4930455F23Rik|74895	-1.020811367	0.459409764	-1.009066516	0.472224094
Wdr1|22388	-1.020812538	0.39709317	-1.020812538	0.39709317
Eppk1|223650	-1.020826318	0.452657155	-1.082501256	0.27403136
BC003965|214489	-1.020874349	0.441300987	-1.099425612	0.223704719
Tshz1|110796	-1.020897108	0.465423657	1.274984938	0.101135534
Pank1|75735	-1.020953357	0.463290262	1.103517952	0.33263809
B230312A22Rik|230088	-1.021020913	0.441339228	-1.015044179	0.424927294
Tmco1|68944	-1.021042238	0.45088885	1.180066089	0.107823898
Raly|19383	-1.021053564	0.451687489	-1.146177349	0.192716249
Polm|54125	-1.021078539	0.46045302	-1.109392497	0.265797587
Nek5|330721	-1.021082443	0.487944114	1.952283016	0.177025661
Ak2|11637	-1.02109502	0.374968646	-1.02109502	0.374968646
Vps18|228545	-1.021113069	0.401464174	-1.021113069	0.401464174
Gm14326|665211	-1.021141524	0.475223023	-1.254907086	0.255777601
Sra1|24068	-1.021155494	0.438961841	-1.021155494	0.438961841
Mrpl50|28028	-1.02120169	0.413748741	-1.058473058	0.279443365
Ddx50|94213	-1.021204765	0.397311581	1.01561529	0.384727216
Znrf2|387524	-1.021239472	0.472413125	1.289029077	0.133323618
Pdrg1|68559	-1.021496025	0.449095217	1.070419158	0.320098528
Xab2|67439	-1.021658756	0.425740919	1.009679122	0.461047905
Cpsf2|51786	-1.021720175	0.46005523	1.091874929	0.321182031
Sbno2|216161	-1.021756769	0.443648649	-1.078685103	0.320852258
Mamdc4|381352	-1.021810856	0.483479225	1.804995212	0.044653072
Donson|60364	-1.021835953	0.484930508	-1.021835953	0.484930508
Tax1bp1|52440	-1.021836694	0.454134384	1.205625463	0.102565219
Ugt2b5|22238	-1.021872986	0.426191673	-1.021872986	0.426191673
Lrrc57|66606	-1.021887358	0.462426658	1.134536912	0.093054222
Thoc3|73666	-1.021919936	0.420351783	-1.167550495	0.014052069
Nde1|67203	-1.021939035	0.456470232	-1.167041574	0.201959982
Mapre2|212307	-1.02194946	0.44649216	-1.203935982	0.098254726
Tmem33|67878	-1.021968372	0.442481353	1.069114581	0.31888893
Suv420h1|225888	-1.021988831	0.449054011	-1.11623551	0.261093463
Stk38|106504	-1.022083975	0.4180268	1.027799983	0.397736677
Actr8|56249	-1.022137184	0.434144277	-1.078186767	0.280910752
Rg9mtd1|52575	-1.022253282	0.439131135	-1.022253282	0.439131135
Maff|17133	-1.022273748	0.475096288	-1.022273748	0.475096288
Zfp653|319601	-1.022334371	0.456695926	-1.11653216	0.282746087
Mrpl37|56280	-1.022399457	0.399382629	-1.123287753	0.053243855
Tpst2|22022	-1.022453467	0.424169088	1.05374961	0.28482476
Ticam1|106759	-1.022571162	0.437240479	1.07748035	0.270405278
Pemt|18618	-1.022668429	0.454661337	-1.019999524	0.44439405
Vps37c|107305	-1.022785211	0.415150467	-1.005549551	0.473427529
1500032L24Rik|69029	-1.02282793	0.44721519	1.078691312	0.321574019
Cdk14|18647	-1.022858744	0.486127657	-1.222242267	0.387350903
Pfkp|56421	-1.022869784	0.472128391	-1.223250592	0.218417019
Rab4b|19342	-1.022896665	0.451796533	-1.010003115	0.465380264
Lrrc16a|68732	-1.022897172	0.481270207	1.579749624	0.018881243
Med11|66172	-1.022907689	0.463294202	1.120899178	0.288313183
Tox2|269389	-1.022920113	0.485844779	-1.155542448	0.390624835
Cfhr2|545366	-1.022941961	0.445385343	-1.061679415	0.330639894

Zfp53|24132	-1.022950658	0.475869636	-1.409805934	0.153102385
Rcor2|104383	-1.022972993	0.486864591	1.943674352	0.178354219
Faf1|14084	-1.022994951	0.424349683	-1.08232729	0.240619774
Slc30a1|22782	-1.022995755	0.446248897	1.089175495	0.289301889
Tm9sf3|107358	-1.023025486	0.424199587	-1.023025486	0.424199587
Zfp747|269997	-1.023112924	0.45194652	-1.023112924	0.45194652
Zfp335|329559	-1.023173256	0.456689334	-1.263450293	0.092015465
Tex264|21767	-1.023192874	0.43379411	-1.096881309	0.252565807
Steap3|68428	-1.023216552	0.429820245	-1.023216552	0.429820245
Gsn|227753	-1.023256848	0.466796614	1.221628356	0.192114475
Gm6724|626954	-1.023262755	0.476265398	-1.412183243	0.165951165
Brf2|66653	-1.023284331	0.460873044	1.092655014	0.338072811
Ptbp1|19205	-1.023298521	0.420500896	1.099291151	0.158193275
Rhot2|214952	-1.023304346	0.424662487	-1.181643916	0.021229183
Sar1a|20224	-1.023336644	0.375846168	-1.011937145	0.441106539
Pgd|110208	-1.023337115	0.410220299	-1.023337115	0.410220299
Bhlhe40|20893	-1.023359766	0.460703157	-1.068900017	0.345772027
Eif3a|13669	-1.023404091	0.443520072	1.106994531	0.216850191
Ghitm|66092	-1.023455871	0.4282928	-1.023455871	0.4282928
1200016B10Rik|66875	-1.023551389	0.466444411	1.170628217	0.210472654
Cog7|233824	-1.02370752	0.37824227	-1.070837648	0.147167555
Cinp|67236	-1.023760101	0.427829415	-1.023760101	0.427829415
Aph1a|226548	-1.023764647	0.31786268	-1.001368187	0.488637262
Fam168b|214469	-1.023860337	0.460340969	1.300733209	0.042922351
Chchd10|103172	-1.023940611	0.440888105	1.057030912	0.350873067
Gm98|225908	-1.023941101	0.440853007	1.086256157	0.067162919
Agap3|213990	-1.023976169	0.374721412	-1.023976169	0.374721412
Cklf|75458	-1.024059082	0.481921016	1.242318118	0.311822675
Lars2|102436	-1.024114226	0.468992557	-1.093691478	0.338781811
Rarres2|71660	-1.024222344	0.460412156	-1.254729475	0.121785472
Scarb2|12492	-1.024225849	0.355870037	1.024349864	0.310122594
Dusp11|72102	-1.02433203	0.43115359	1.017105831	0.429232476
Pfas|237823	-1.024372552	0.458593044	1.241307487	0.142184586
Wdr4|57773	-1.024381547	0.400364101	-1.136770683	0.047349848
Echs1|93747	-1.024393699	0.271643018	-1.053363228	0.059945523
Gabarapl1|57436	-1.024395513	0.433631697	-1.218154809	0.026686731
Polr2m|28015	-1.024431358	0.389462762	-1.024431358	0.389462762
Ccdc124|234388	-1.02444311	0.458482157	-1.02444311	0.458482157
Rnaseh1|19819	-1.024458845	0.454910831	-1.060177424	0.24777572
6530401N04Rik|328092	-1.024497271	0.420151383	1.050830284	0.307391746
Lima1|65970	-1.024576411	0.434219892	1.101068268	0.179385293
Wipi2|74781	-1.024647217	0.379378233	-1.087452362	0.09488452
Fetub|59083	-1.024749394	0.449997632	-1.2814143	0.04744876
Tmem66|67887	-1.024815705	0.380788613	-1.01767564	0.345376272
Rufy2|70432	-1.024819518	0.467318133	1.171045855	0.298148569
Snx32|225861	-1.024833567	0.467205913	-1.161807328	0.248149814
Spsb3|79043	-1.02488946	0.431944771	-1.21188211	0.034328386
Asxl2|75302	-1.024971472	0.470211959	-1.024971472	0.470211959
Zap70|22637	-1.025045435	0.449224075	1.08193598	0.320242993
P2ry2|18442	-1.025076866	0.436479599	-1.088548039	0.289374406
Tubgcp6|328580	-1.025153257	0.437197261	-1.025153257	0.437197261
Ddt|13202	-1.025184272	0.443924324	-1.025184272	0.443924324
Akap13|75547	-1.025204072	0.463262035	1.229518774	0.161757762

Hnrnpa1|15382	-1.025274729	0.453503481	-1.321820741	0.039671829
Snapc2|102209	-1.025275224	0.438271553	-1.214871793	0.076189296
Gimap3|83408	-1.025483879	0.446254034	-1.073467369	0.256997134
Fam108a|216169	-1.025485887	0.430785293	-1.11657214	0.180384626
Ssr4|20832	-1.025528467	0.42503235	-1.192910663	0.044178416
Dyrk2|69181	-1.025744412	0.46045835	-1.025744412	0.46045835
Mvp|78388	-1.025757795	0.434932873	-1.107330259	0.260059729
Zranb1|360216	-1.025782912	0.435764941	-1.025782912	0.435764941
Cox19|68033	-1.025795169	0.440474847	1.061208403	0.349880012
Mkrn1|54484	-1.025820305	0.421133637	-1.115916614	0.133706944
Sap18|20220	-1.025852973	0.359795874	-1.067299992	0.170666192
Camk1|52163	-1.025856211	0.464082787	1.165518735	0.132035249
Xcr1|23832	-1.025862323	0.476875322	1.156502255	0.360111999
AI182371|98870	-1.025874005	0.45105146	-1.230382019	0.090995833
1110001A16Rik|68554	-1.025894119	0.408263708	-1.025894119	0.408263708
Trpc2|22064	-1.025946932	0.46429671	1.088914164	0.391809288
Casc4|319996	-1.026005674	0.468578961	-1.235303892	0.17649846
1810035L17Rik|380773	-1.026013479	0.422517651	-1.117786707	0.177697011
Ado|211488	-1.02605601	0.431388696	-1.086087267	0.287851015
Dcaf8|98193	-1.026139944	0.339175093	-1.00385023	0.476307197
Rpl3|27367	-1.026156655	0.398757622	-1.072524577	0.236945873
Xpr1|19775	-1.026169173	0.371309195	-1.127800883	0.013166808
Cdc27|217232	-1.026204382	0.44369607	-1.065503369	0.287157246
Smc4|70099	-1.026239144	0.477461788	1.372784993	0.244803193
Ndufaf4|68493	-1.026321452	0.423279994	-1.191035221	0.052199678
D17Wsu104e|28106	-1.026437497	0.441355972	-1.159821313	0.177979161
2310011J03Rik|66374	-1.026541176	0.434817732	-1.026541176	0.434817732
Ndrg3|29812	-1.026566503	0.397207721	-1.141852473	0.034289169
Ube2r2|67615	-1.026643987	0.418676382	-1.108901871	0.19818703
Tmbim4|68212	-1.02666324	0.39794231	1.04758481	0.285357953
Napg|108123	-1.02668644	0.438121195	1.055255559	0.365525882
4933409K07Rik|108816	-1.026719153	0.42747174	1.008409074	0.468922121
Slc39a11|69806	-1.026723203	0.454761319	1.066316319	0.387335525
Yipf3|28064	-1.026751057	0.413251007	-1.08168925	0.265198557
Mup2|17841	-1.026785767	0.487407397	1.389511645	0.345619417
Gspt1|14852	-1.026869703	0.414840272	-1.08802091	0.234258572
Eno1|13806	-1.027020217	0.424252597	1.028587052	0.395536234
Brca1|12189	-1.027042766	0.478344744	-1.395031364	0.267461448
Etfa|110842	-1.027077689	0.359973787	-1.062448047	0.20138294
Cep135|381644	-1.027095911	0.466334622	-1.121934465	0.348318824
Szt2|230676	-1.027100903	0.396530208	-1.037808597	0.306505645
Nup93|71805	-1.027179304	0.474419942	-1.620813531	0.047724049
Trim32|69807	-1.027185058	0.455365855	1.087624303	0.326449396
BC029722|613262	-1.027273295	0.418470156	-1.191576493	0.035126964
Fxc1|14356	-1.027421424	0.440497474	-1.032057058	0.422982447
Chordc1|66917	-1.027439432	0.42197946	-1.202888052	0.037427894
Fyb|23880	-1.027485813	0.438572325	-1.186059528	0.109370386
Strap|20901	-1.027498173	0.395372108	-1.027498173	0.395372108
Art4|109978	-1.027507105	0.457168927	-1.027507105	0.457168927
Nol6|230082	-1.02750789	0.36215875	1.008789691	0.454209061
Gm4787|214321	-1.027531427	0.489365619	3.3	0.173296754
Rtn3|20168	-1.027540144	0.392518941	-1.093399221	0.138350188
Tmem60|212090	-1.027627723	0.463768634	1.344520376	0.114338795

Daglb|231871	-1.027695165	0.436708814	-1.119693372	0.233090168
BC003266|80284	-1.027707912	0.407066028	1.10378487	0.139181588
Commd10|69456	-1.027756646	0.443558579	-1.195748993	0.13387159
Parl|381038	-1.027927955	0.426313642	-1.102394752	0.240779941
Lrsam1|227738	-1.027936709	0.445056682	-1.117729945	0.136759456
Mrpl10|107732	-1.027989394	0.382526196	1.047448558	0.234370294
Abcf1|224742	-1.028017707	0.358178866	1.017442879	0.400646284
Faah|14073	-1.028051532	0.236528035	-1.048984204	0.103224902
Nhej1|75570	-1.028106701	0.458239311	-1.223238202	0.216726869
Ugt2b36|231396	-1.028111988	0.398657958	1.032640664	0.376711409
Ubr5|70790	-1.028117747	0.453934588	1.187328402	0.178261958
0610007C21Rik|381629	-1.028119236	0.388334074	-1.103350028	0.078072435
Fzr1|56371	-1.028162442	0.414517172	-1.012182784	0.451598235
Lztr1|66863	-1.028172112	0.38624504	1.075430607	0.17914454
Cd163|93671	-1.028246195	0.444287845	-1.028246195	0.444287845
Prkcd|18753	-1.028319242	0.460574887	1.220118524	0.200448835
Hnrnpd|11991	-1.028353123	0.440492037	-1.027084359	0.412390044
Ech1|51798	-1.028360426	0.406985632	-1.028360426	0.406985632
Taz|66826	-1.028377606	0.439139882	-1.028377606	0.439139882
Bmp5|12160	-1.028391642	0.480059299	-1.195149504	0.36511007
Fggy|75578	-1.028543283	0.394110153	-1.087106477	0.189793574
Ptprs|19280	-1.028565448	0.456158438	-1.24885965	0.166571798
Fbxo18|50755	-1.028640152	0.423073501	-1.028640152	0.423073501
Rheb|19744	-1.028656633	0.439703183	1.063103913	0.373736081
Nans|94181	-1.02868178	0.447681659	-1.089530479	0.294539627
D630045J12Rik|330286	-1.028682357	0.443392598	-1.280994931	0.063760424
Zc3hc1|232679	-1.028686121	0.45047247	-1.028686121	0.45047247
Gatad1|67210	-1.028773581	0.377358114	1.08349187	0.081720442
Poldip3|73826	-1.028824953	0.375089552	-1.069927419	0.218837808
Kctd6|71393	-1.028925821	0.453294754	-1.333452714	0.073264846
Uap1|107652	-1.028945215	0.440586087	1.221911503	0.061336936
Fem1b|14155	-1.028969611	0.433589253	-1.149930553	0.191036166
Smarce1|57376	-1.028992349	0.430957801	-1.028992349	0.430957801
Sbno1|243272	-1.02906294	0.445088415	1.019899706	0.441550414
Alg8|381903	-1.029078225	0.468824571	-1.029078225	0.468824571
Park7|57320	-1.02909878	0.359270242	-1.077336795	0.143848136
Ccdc94|72886	-1.029110535	0.429680879	-1.041278477	0.37815575
Sumf1|58911	-1.029228495	0.41425627	1.04589258	0.362378386
Ppil6|73075	-1.029241268	0.483386241	-1.130783055	0.437947892
1110059E24Rik|66206	-1.029287774	0.417675944	-1.095469934	0.245498602
2510049J12Rik|70291	-1.029295826	0.413483732	1.118981281	0.136594799
E2f4|104394	-1.029308916	0.342014788	-1.008195411	0.434487176
Sash1|70097	-1.029328763	0.462819226	1.244504393	0.20660387
Fam32a|67922	-1.029470634	0.409754259	-1.085689165	0.268740152
Ctbp1|13016	-1.029483917	0.302446317	-1.029483917	0.302446317
Pomgnt1|68273	-1.02949225	0.43880995	-1.146732357	0.232547775
Glrx2|69367	-1.029574906	0.401881266	1.026509516	0.406180332
Scaf4|224432	-1.029715605	0.429875486	-1.13378879	0.219569212
Acin1|56215	-1.029744837	0.395349896	-1.091649242	0.190132445
Brwd1|93871	-1.029888329	0.42625158	-1.029888329	0.42625158
Txndc15|69672	-1.029950833	0.371364524	-1.029950833	0.371364524
Fbxo22|71999	-1.030048752	0.396163778	1.008577723	0.470238807
Ctif|269037	-1.030193732	0.443896172	1.078367033	0.362778109

Atp5c1|11949	-1.030248735	0.327055518	-1.109562491	0.025998168
Pik3r4|75669	-1.030288213	0.431369499	1.037188021	0.420672721
E130309D02Rik|231868	-1.030306016	0.403184013	1.01281308	0.45881409
Pvrl2|19294	-1.030307917	0.421499026	-1.134524073	0.185654757
Ssbp3|72475	-1.030376763	0.318346291	-1.024366853	0.268818982
Sgms1|208449	-1.030467653	0.409408464	1.128102721	0.102162544
Atp9a|11981	-1.030483296	0.346841074	-1.028826453	0.368773186
Mrps12|24030	-1.030498331	0.399508758	1.039067994	0.351812829
Tagln2|21346	-1.030500348	0.42379454	-1.046631773	0.330190776
Hexim1|192231	-1.030529299	0.358499796	-1.030529299	0.358499796
Socs3|12702	-1.030531467	0.455867643	-1.20227483	0.253910544
Pabpc1|18458	-1.030532193	0.397750459	-1.106681752	0.144849513
Atp5j2|57423	-1.030538464	0.440188403	-1.286345012	0.063577906
Dynlt1e|100040631	-1.030601934	0.477194093	1.400578006	0.274137403
Sp1|20683	-1.030657002	0.447616876	1.242219336	0.125344351
Pik3c2g|18705	-1.030711041	0.46456532	1.133099751	0.366688948
Polr3a|218832	-1.030722447	0.435467184	-1.030722447	0.435467184
Leo1|235497	-1.030806111	0.405081321	-1.028971963	0.370067864
Rc3h1|381305	-1.030881404	0.449871788	1.077239373	0.269580866
Elmod3|232089	-1.03096652	0.396227618	-1.03096652	0.396227618
Rnf103|22644	-1.030968052	0.420135681	1.14416333	0.130789751
Eif5b|226982	-1.031032049	0.410989411	1.025118939	0.425806112
Cox6c|12864	-1.031130309	0.408549394	-1.187884432	0.06058346
Afg3l2|69597	-1.031169104	0.35622769	1.010872446	0.444337535
Eftud2|20624	-1.031177591	0.384866983	1.034662185	0.358486083
Fam98b|68215	-1.031216094	0.461881709	-1.246642251	0.253488914
Commd5|66398	-1.031218842	0.41316252	-1.095535064	0.249663049
Qsox2|227638	-1.031276623	0.452185157	-1.364543504	0.05206433
Ccdc24|381546	-1.031328291	0.459286888	-1.289318515	0.185086587
H2-Gs10|436493	-1.031333957	0.432746462	1.203989993	0.070746718
Fam110a|73847	-1.031349502	0.423840782	-1.031349502	0.423840782
Ykt6|56418	-1.031359076	0.33556237	-1.082131655	0.08813628
Apon|28194	-1.031364675	0.389592114	1.016339329	0.417391142
BC028528|229600	-1.031366117	0.476289634	1.449077745	0.242466668
Rai2|24004	-1.031375268	0.439670832	1.224741387	0.086925157
Cirbp|12696	-1.031401097	0.433516308	-1.176491471	0.163331047
Rsrc1|66880	-1.031442245	0.432209599	1.049647617	0.388907555
Sys1|66460	-1.031569527	0.391742709	-1.031569527	0.391742709
Eif3i|54709	-1.031575349	0.415966893	-1.031575349	0.415966893
Spp2|75396	-1.031612347	0.433273062	-1.123395659	0.273910397
Sdr39u1|654795	-1.031801041	0.354902086	-1.079467697	0.177100703
Nfyc|18046	-1.031901842	0.412357395	-1.102758483	0.251607027
Wdr60|217935	-1.031907616	0.442645419	-1.178585953	0.166676266
Trim62|67525	-1.031991158	0.471110775	1.647507276	0.067905027
Gtf3c1|233863	-1.032044628	0.346646588	1.058714064	0.166115797
Rpl21|19933	-1.032064914	0.451798475	-1.069889491	0.365045843
F12|58992	-1.032131782	0.396781746	-1.006764939	0.47918088
Rnh1|107702	-1.032174387	0.356985522	-1.061352127	0.168422558
Tsen2|381802	-1.032224669	0.417190906	1.146142825	0.083681058
Mmd2|75104	-1.032237118	0.469221699	-1.403255836	0.126603282
Poc5|67463	-1.032246504	0.426070804	-1.007575961	0.472432146
Cldn12|64945	-1.032257102	0.417174761	1.094891158	0.207796711
Pex13|72129	-1.032259302	0.414934411	-1.159523332	0.108303212

Clptm1|56457	-1.032323919	0.365870315	-1.139689895	0.050532825
Foxo4|54601	-1.03240499	0.402711091	-1.215079983	0.008440883
Zfp59|22717	-1.032477491	0.480042661	-2.250103451	0.074196186
Cdc42bpg|240505	-1.032509265	0.445301103	1.174417627	0.185318067
Mrto4|69902	-1.032572222	0.430210759	-1.032572222	0.430210759
Ankrd52|237615	-1.03261091	0.465604385	1.562110885	0.033367542
Chaf1b|110749	-1.032661041	0.44891603	-1.032661041	0.44891603
Vps4a|116733	-1.032723668	0.376977369	-1.091639474	0.192937718
Ermap|27028	-1.032844168	0.485591506	1.255130842	0.411829119
Klf13|50794	-1.032859046	0.42398048	-1.138467993	0.179139912
1810046J19Rik|103742	-1.032892715	0.405805449	-1.147862947	0.106093579
Slc2a5|56485	-1.032934804	0.448378275	1.292580073	0.082029291
Cpsf3l|71957	-1.032984801	0.436483008	-1.231232188	0.089548723
Gm13476|433424	-1.032990279	0.462579257	-1.207069162	0.2705832
Colec11|71693	-1.032994872	0.393430053	-1.032994872	0.393430053
Gm9104|668319	-1.03303445	0.448070492	-1.239578883	0.173425022
4932416H05Rik|654409	-1.033123609	0.465780041	1.556713611	0.045907999
Ctu1|233189	-1.033203099	0.417250736	1.072339796	0.278751227
Pgam2|56012	-1.03320711	0.483068245	-1.792828283	0.26550972
Ganab|14376	-1.033221528	0.320110122	-1.086388723	0.080077226
Arfgap3|66251	-1.033263689	0.42813902	1.118263353	0.209054156
Eps15l1|13859	-1.033267321	0.410061891	-1.125113056	0.198375394
Tada1|27878	-1.033333418	0.442222917	-1.033333418	0.442222917
Ralgds|19730	-1.033392714	0.445217465	-1.377807895	0.034211987
Wfs1|22393	-1.033413977	0.439276279	1.092577171	0.311099274
Commd2|52245	-1.033503895	0.417330114	1.148638288	0.135265782
Setx|269254	-1.033523246	0.468384732	1.303430422	0.259962272
Slc9a8|77031	-1.033529089	0.39978766	1.014008426	0.459523439
Lgals8|56048	-1.033565559	0.425793148	-1.002537207	0.491658606
Slc4a7|218756	-1.033571227	0.465943898	-1.166232585	0.259833607
Snta1|20648	-1.0335746	0.428820906	-1.0335746	0.428820906
Adi1|104923	-1.033645329	0.35255887	-1.140667146	0.027887787
Pold3|67967	-1.033667993	0.439052563	1.080021774	0.362658251
Ganc|76051	-1.033675934	0.441509817	1.056830472	0.399728134
Tmco4|77056	-1.033705157	0.418961688	-1.108919143	0.252408475
Incenp|16319	-1.033713232	0.468435205	-1.033713232	0.468435205
Mypop|232934	-1.033758733	0.463320737	1.107699885	0.398730526
Myst2|217127	-1.033794745	0.431934927	-1.302390314	0.032843744
Mapk6|50772	-1.033812425	0.44705298	-1.049545603	0.417135053
Mettl6|67011	-1.033888399	0.426487848	-1.033888399	0.426487848
Acad11|102632	-1.033891495	0.41317733	-1.033891495	0.41317733
Dap3|65111	-1.033896789	0.409130334	-1.186933598	0.02263735
Zfp85-rs1|22746	-1.033937977	0.480432216	1.730652546	0.230959098
F13b|14060	-1.034076081	0.443451151	-1.208418346	0.201310775
Rnf220|66743	-1.034108278	0.21959699	-1.034108278	0.21959699
Usp25|30940	-1.034116576	0.415117012	1.164523337	0.07064634
Eif5a|276770	-1.034159799	0.30389502	-1.065187654	0.075384489
Gtdc1|227835	-1.034186212	0.443347142	1.174399993	0.190838535
Elp4|77766	-1.034213303	0.43767221	-1.321693468	0.03219599
Ccnd3|12445	-1.034218769	0.377547236	-1.030791341	0.369794461
Apod|11815	-1.034233278	0.487300913	-5.725	0.078582895
Zfp296|63872	-1.034235253	0.464940268	-1.034235253	0.464940268
Tcap|21393	-1.034308472	0.487027443	-7.367	0.093227434

AY074887|246735	-1.034308472	0.487027443	-7.367	0.093227434
Ptgfrn|19221	-1.034312041	0.451578133	1.316171336	0.11029822
Foxn2|14236	-1.034326427	0.442879074	-1.237336904	0.152935146
Hebp1|15199	-1.034379683	0.328400884	-1.083544499	0.107833309
Lars|107045	-1.034406911	0.397045414	1.12577663	0.092401522
Gimap8|243374	-1.034433325	0.458377504	-1.235279674	0.208963534
Ogt|108155	-1.034441599	0.453239692	1.188772601	0.256218211
Tom1l2|216810	-1.034449532	0.418432258	-1.115336269	0.239891468
Tfb2m|15278	-1.034458973	0.434926695	-1.128865505	0.26886835
Pstk|214580	-1.034737319	0.462920059	-1.29543107	0.249631897
Apoc3|11814	-1.034754216	0.428841403	-1.157674172	0.17296076
Mccc1|72039	-1.034851648	0.400776372	-1.050196917	0.339770676
Psenen|66340	-1.03488043	0.447053634	-1.292621154	0.108612396
Psmd7|17463	-1.034943902	0.304686423	-1.034943902	0.304686423
Iscu|66383	-1.034948799	0.319983738	1.000260325	0.497895465
Tor3a|30935	-1.034950919	0.432620558	-1.034950919	0.432620558
Apc|11789	-1.035008765	0.460245324	1.276178094	0.210184642
Tmed8|382620	-1.035060775	0.468381487	-1.211325422	0.34829553
Mrm1|217038	-1.035087849	0.417243761	-1.21754985	0.072435025
Fundc2|67391	-1.035105566	0.45791422	-1.551751573	0.034183947
8430410K20Rik|78100	-1.035187235	0.454097342	1.125182087	0.323673503
Ankrd13a|68420	-1.035273956	0.378678053	1.098406393	0.111014056
Trp53bp2|209456	-1.035276121	0.455890083	1.027548831	0.463378488
Zfp689|71131	-1.035370773	0.445896519	-1.035370773	0.445896519
Tbk1|56480	-1.035384446	0.451547775	-1.029884636	0.451352422
A430033K04Rik|243308	-1.035447471	0.418565115	-1.229518772	0.074110668
Sft2d2|108735	-1.035470263	0.422855157	1.111896671	0.222775044
Cwc22|80744	-1.035472729	0.424886923	-1.035472729	0.424886923
Icmt|57295	-1.03547643	0.259772313	-1.03547643	0.259772313
Zfp295|114565	-1.035540152	0.402798438	-1.035540152	0.402798438
Decr1|67460	-1.035558256	0.373061318	-1.054143583	0.255723909
Slc38a4|69354	-1.035635855	0.338177799	-1.138759352	0.023537177
Cltc|67300	-1.035638004	0.415708622	1.139251041	0.167059407
4933407C03Rik|74440	-1.035653751	0.281270142	1.001910439	0.485512211
Ambra1|228361	-1.035713756	0.368410767	1.018970959	0.42616929
Mettl7a1|70152	-1.035742798	0.35363857	-1.036979049	0.307501213
Limd1|29806	-1.035803417	0.336408051	-1.09436102	0.114333754
Nr2f6|13864	-1.035873312	0.421988222	-1.132282189	0.221550938
Scarf1|380713	-1.035985302	0.44731296	-1.363296478	0.026239805
Nup62|18226	-1.036086556	0.409587972	-1.221911587	0.055659001
4933439F18Rik|66771	-1.036105589	0.388003176	-1.036105589	0.388003176
Kif1c|16562	-1.036146274	0.319394415	1.052836039	0.157144819
Poglut1|224143	-1.036155015	0.427318329	-1.036155015	0.427318329
Vps35|65114	-1.036163072	0.437824421	-1.043938526	0.413294483
Cbln3|56410	-1.036239186	0.443913214	-1.363979174	0.067129836
Cln3|12752	-1.0362963	0.378231945	-1.0362963	0.378231945
Plekhm2|69582	-1.036306308	0.40183178	-1.123885716	0.2038162
Nudt6|229228	-1.036345043	0.383973053	-1.040319102	0.340911986
Cnn3|71994	-1.03641205	0.341528198	-1.03641205	0.341528198
Mrpl42|67270	-1.036419635	0.359498839	-1.014906918	0.429294093
Zbtb17|22642	-1.036437722	0.372431331	1.08004259	0.195348218
Bnip3l|12177	-1.036453925	0.341692582	-1.137272746	0.045250219
Arsa|11883	-1.036468482	0.3778258	1.022311367	0.417201841

Gm6682|626534	-1.036491776	0.387085323	-1.197605076	0.02781523
Peli1|67245	-1.036508458	0.431407594	1.219850531	0.094917449
Fkbp3|30795	-1.036582015	0.39611416	1.03564612	0.387502056
Tmem183a|57439	-1.036690919	0.247984155	-1.036690919	0.247984155
Comt|12846	-1.036708037	0.401799511	-1.140628954	0.162740308
Marcks|17118	-1.036766031	0.444926446	-1.197589024	0.196774485
Ndufs6|407785	-1.036839926	0.400898565	-1.206522426	0.054410513
Arf4|11843	-1.036851678	0.361358359	-1.049612364	0.196681216
Iars|105148	-1.036875774	0.343159244	-1.08790974	0.150680466
Arhgef1|16801	-1.036876445	0.306342747	-1.036876445	0.306342747
Hps1|192236	-1.036906007	0.426266873	1.193526634	0.128247301
Slc25a5|11740	-1.03691393	0.292636525	1.005143826	0.462630445
Ubqln2|54609	-1.036986216	0.448406393	1.022295262	0.463761125
Snrk|20623	-1.036990922	0.403145433	1.142565455	0.098157898
Mxra7|67622	-1.036992129	0.38936925	-1.149063317	0.088643283
Clk1|12747	-1.037037428	0.451789178	-1.204073741	0.280828994
Polr3h|78929	-1.037092749	0.420385249	-1.271166688	0.051037576
Cyp4b1|13120	-1.03711031	0.385950816	-1.03711031	0.385950816
Ankhd1|108857	-1.037185656	0.399198134	-1.117112384	0.194565128
1110001J03Rik|66117	-1.037202605	0.422123274	-1.166624908	0.193550373
Casp7|12369	-1.037205762	0.443703473	-1.417754852	0.032484723
D8Ertd738e|101966	-1.037233243	0.425050731	-1.190617627	0.159391741
2410016O06Rik|71952	-1.037307419	0.430232396	-1.107499404	0.3146139
Bmp7|12162	-1.037353642	0.470195195	-1.876265586	0.068096032
Magoh|17149	-1.037372577	0.419986077	-1.057611548	0.350327696
Zfp668|244219	-1.037410572	0.402883467	-1.134276556	0.153399738
Ebpl|68177	-1.037439678	0.413593076	-1.267815788	0.015064959
Fam114a2|67726	-1.037471131	0.395987573	1.02230569	0.438999651
Hmgcl|15356	-1.037501298	0.330611344	-1.088219176	0.127929087
Clcf1|56708	-1.037557173	0.471992273	1.398956514	0.272131559
Ddx27|228889	-1.037561498	0.35255061	-1.037561498	0.35255061
2400001E08Rik|66508	-1.037648848	0.365283792	-1.169471143	0.033674974
Zcchc10|67966	-1.03764978	0.450187151	1.160138968	0.262560957
1110058L19Rik|68002	-1.037654607	0.41411375	1.058830116	0.348425241
Sp110|109032	-1.037673023	0.445687811	-1.384045367	0.013343461
Ube2l3|22195	-1.037684968	0.299682347	-1.037684968	0.299682347
Pla1a|85031	-1.037773017	0.328493214	-1.037773017	0.328493214
Abhd14a|68644	-1.037818941	0.416953075	-1.037818941	0.416953075
Eea1|216238	-1.037922431	0.468226042	1.145932609	0.347187261
Ttpal|76080	-1.03792416	0.445615467	-1.180174307	0.248186107
Zcchc24|71918	-1.038021267	0.315039031	1.052311856	0.174445364
Fga|14161	-1.038052227	0.365031053	-1.167198914	0.035643814
Hkdc1|216019	-1.038054744	0.480549365	1.823071741	0.250512967
Tirap|117149	-1.03805535	0.442969448	1.321557414	0.04112792
Fam126b|213056	-1.038096616	0.460593433	-1.11789748	0.365747286
Fam45a|67894	-1.038128648	0.445937842	-1.070535449	0.383025103
Dzip1l|72507	-1.038169681	0.478232479	1.673633937	0.250929993
Dnajc11|230935	-1.038187687	0.39543169	-1.216365293	0.038316959
Tfpi2|21789	-1.038208739	0.445409534	-1.227423014	0.227941586
Nrarp|67122	-1.038270982	0.466337622	-1.038270982	0.466337622
Vps33b|233405	-1.038274957	0.368413504	-1.136484538	0.068992616
Trim37|68729	-1.038286042	0.426323073	-1.000915722	0.497771365
Ubap2l|74383	-1.038323709	0.287008623	-1.079850831	0.113552953

Prkaa2|108079	-1.038421364	0.4098991	1.033900435	0.386802608
Lamtor2|83409	-1.038442003	0.381711809	-1.102363468	0.223949081
Unc79|217843	-1.03852125	0.463098288	1.244508221	0.301900802
Rrm1|20133	-1.038567649	0.451206525	-1.474855864	0.06451733
Nln|75805	-1.038569043	0.415416595	1.01973138	0.446601934
Lipe|16890	-1.03860856	0.370734388	-1.09657145	0.216489109
Napa|108124	-1.038621435	0.39131723	-1.110888558	0.226815272
Slc25a34|384071	-1.038676322	0.368593092	-1.038676322	0.368593092
Dolpp1|57170	-1.038709306	0.373166229	-1.013220457	0.458232349
Fam117a|215512	-1.038853463	0.438331983	-1.140303702	0.290715307
Gmeb2|229004	-1.038892653	0.383836128	-1.038892653	0.383836128
Sdf4|20318	-1.038977493	0.300239486	-1.093399003	0.053713827
Ly96|17087	-1.03898119	0.450570681	-1.115171141	0.328474869
Zfp629|320683	-1.039097057	0.405007207	-1.136225212	0.209958313
Ptov1|84113	-1.039117054	0.423940116	-1.196804023	0.162926182
Thrap3|230753	-1.039179809	0.296357611	-1.039179809	0.296357611
Rps2|16898	-1.039227669	0.393258552	-1.235094298	0.012223563
Mrpl36|94066	-1.039248525	0.392539261	-1.144618245	0.095120678
Serpina10|217847	-1.039261661	0.374004161	-1.039261661	0.374004161
Eif4b|75705	-1.039301057	0.307316586	-1.039301057	0.307316586
Arhgap23|58996	-1.039317065	0.434293442	-1.174680479	0.252053449
Ahcyl2|74340	-1.03938013	0.410414866	-1.03938013	0.410414866
Cd2bp2|70233	-1.039393538	0.306058566	1.046975189	0.178072623
Nmral1|67824	-1.039394809	0.453899848	-1.318926359	0.198017136
Bloc1s2|73689	-1.039459521	0.424894384	-1.248228201	0.069351873
A430078G23Rik|319493	-1.039552837	0.472758298	1.329963963	0.327491449
Zfp787|67109	-1.03960619	0.371481879	1.039941229	0.35081562
S100a11|20195	-1.039635506	0.446098127	-1.523432092	0.001883954
Vps52|224705	-1.0397033	0.276550711	-1.076619398	0.126727631
Chpt1|212862	-1.03970669	0.400999236	-1.037644834	0.364675583
Sugp1|70616	-1.039775503	0.36706737	1.007051714	0.471122659
Uspl1|231915	-1.039814547	0.412375984	1.073153496	0.332670653
Zc3h7b|20286	-1.039821414	0.439656285	1.333046446	0.032025201
Cdk17|237459	-1.039897017	0.447782609	1.097793275	0.352681209
Fcf1|73736	-1.039965758	0.427595697	1.073738045	0.353464256
Tbrg4|21379	-1.040006762	0.324094358	-1.067106925	0.23537367
Omd|27047	-1.040022904	0.476207007	-1.457040245	0.28385737
Gja1|14609	-1.040082897	0.448924725	1.233826507	0.206653853
Disp2|214240	-1.040200234	0.471460787	1.144485906	0.409239478
Zfp36l1|12192	-1.04021046	0.444349493	-1.007857149	0.48754048
Nek6|59126	-1.040214254	0.248708075	-1.040214254	0.248708075
Epm2aip1|77781	-1.040320621	0.453710911	1.451510389	0.064572625
Serpinb6a|20719	-1.040365985	0.418305362	-1.155037377	0.228879479
Gm1987|100504362	-1.040425288	0.475116807	-1.053622661	0.465711755
Cops8|108679	-1.04051349	0.358451996	1.078261386	0.19113517
Polg2|50776	-1.040666688	0.458055008	-1.360316213	0.142470119
Pus3|67049	-1.040734906	0.462634528	1.025461064	0.473225512
Snap47|67826	-1.040803076	0.32380671	-1.092057894	0.131681287
Plekho2|102595	-1.040835504	0.393999984	-1.040835504	0.393999984
Xpo5|72322	-1.040837778	0.403055148	-1.154487876	0.176589969
0610010F05Rik|71675	-1.040840624	0.455531133	1.150179439	0.322701986
Dock7|67299	-1.040867001	0.460767293	-1.040867001	0.460767293
Fbxo33|70611	-1.040874679	0.42227191	-1.040874679	0.42227191

Lenep|57275	-1.040912642	0.465331461	-1.240958209	0.305043794
Fchsd2|207278	-1.04094762	0.433028516	-1.04094762	0.433028516
Cfb|14962	-1.041004499	0.367096634	-1.126682583	0.13352085
Ccdc86|108673	-1.041113956	0.42546583	1.087198162	0.31782694
Actg1|11465	-1.041138202	0.396727865	1.023726907	0.441636312
Hsdl1|72552	-1.041148429	0.335663859	-1.041148429	0.335663859
Papd5|214627	-1.041152272	0.444165424	1.127730382	0.279052594
Tprkb|69786	-1.041181604	0.338040921	-1.085258484	0.122265135
Tmem127|69470	-1.04119755	0.393115921	-1.04119755	0.393115921
Ccs|12460	-1.041298599	0.36449537	1.039110213	0.348818395
Etohd2|13996	-1.041327021	0.399263124	-1.120442118	0.2224415
Lamb3|16780	-1.04134686	0.440000917	1.170730015	0.2529146
Cotl1|72042	-1.041359471	0.397640079	-1.135131899	0.207778677
Kif14|381293	-1.041422578	0.481834526	-3.967316667	0.09324139
Arhgap8|73167	-1.041422578	0.481834526	-3.967316667	0.09324139
Lix1|66643	-1.041422578	0.481834526	-3.967316667	0.09324139
Gm1082|381868	-1.041422578	0.481834526	-3.967316667	0.09324139
Dock11|75974	-1.041422578	0.481834526	-3.967316667	0.09324139
Slc22a12|20521	-1.041422578	0.481834526	-3.967316667	0.09324139
Klhl8|246293	-1.041493961	0.452704406	1.243488774	0.243276926
Blvra|109778	-1.04156648	0.41840677	-1.04156648	0.41840677
Ltbp2|16997	-1.041602765	0.478288598	-2.217419325	0.174481199
Leng9|243813	-1.041618803	0.435245681	1.319071832	0.028616611
Sdr42e1|74032	-1.041666827	0.356367568	-1.178518643	0.032394987
Ankrd40|71452	-1.041682282	0.293495963	1.039058354	0.260300308
Bud31|231889	-1.041755171	0.412321077	-1.041755171	0.412321077
Serpina11|380780	-1.041789734	0.3943962	-1.041789734	0.3943962
Pex14|56273	-1.041816845	0.318980418	-1.079203587	0.201780762
Tcf3|21423	-1.041852297	0.382382866	-1.22501588	0.019782339
Zdhhc4|72881	-1.041852685	0.350925846	-1.041852685	0.350925846
Hnrnpc|15381	-1.04195009	0.26378533	-1.123294539	0.003828545
Dnajc25|72429	-1.042099551	0.325640774	-1.042099551	0.325640774
BC004004|80748	-1.042205338	0.382543998	-1.042205338	0.382543998
2210013O21Rik|70123	-1.042333886	0.470783876	-1.614328214	0.191328352
Gstm7|68312	-1.042354974	0.358943476	-1.042354974	0.358943476
Erc1|111173	-1.042376324	0.361674878	1.093195174	0.145125453
Ulk2|29869	-1.04241284	0.429110458	1.126479115	0.284774195
Ralgapa1|56784	-1.042416958	0.448904252	1.145154724	0.303751972
Dnajc10|66861	-1.042482967	0.421912727	-1.178668562	0.218197199
Eif4a3|192170	-1.042524618	0.367000013	1.020298791	0.433518908
Lmcd1|30937	-1.042577311	0.45578029	-1.042577311	0.45578029
2900053A13Rik|554362	-1.04258317	0.436091727	-1.065232008	0.331849242
Txnrd1|50493	-1.042652599	0.323326723	-1.089929489	0.158798898
Gga2|74105	-1.042703136	0.398968759	-1.042703136	0.398968759
Hnrnpa2b1|53379	-1.042767914	0.370461819	1.019041413	0.440515839
Ankrd44|329154	-1.04277258	0.459858691	-1.418708542	0.148421125
Dusp6|67603	-1.04277301	0.44015313	1.201675253	0.210137355
Efhd2|27984	-1.042824379	0.327380633	1.016588073	0.415072381
Denr|68184	-1.042877302	0.398961304	1.051699555	0.374349183
Casp2|12366	-1.042889219	0.456352429	-1.23208918	0.31153902
Txn2|56551	-1.042952643	0.345955256	-1.095965356	0.200032966
8430410A17Rik|232210	-1.04296611	0.429210576	1.09660591	0.317560653
Aurkaip1|66077	-1.04301115	0.40537177	-1.043439182	0.403692047

Podn|242608	-1.043199459	0.415690057	-1.192520097	0.172596056
Tfe3|209446	-1.04320464	0.350464806	-1.022257176	0.404923174
Tacc1|320165	-1.043281496	0.37320371	-1.011256102	0.446624904
St6galnac6|50935	-1.043319856	0.200653504	-1.015218948	0.371337147
Adrm1|56436	-1.043367171	0.381105873	1.054213028	0.305812043
Sdhaf2|66072	-1.043412887	0.357405215	1.015708695	0.445555194
Plekhb2|226971	-1.04342648	0.415505277	-1.04342648	0.415505277
Cideb|12684	-1.043436092	0.338616606	-1.017420561	0.417185836
Ttc30b|72421	-1.04352939	0.458995145	-1.455419802	0.175078962
Thra|21833	-1.043536316	0.38508804	-1.043536316	0.38508804
BC026590|230234	-1.043622989	0.437062294	1.126595614	0.3263594
Grk5|14773	-1.043643881	0.419402071	1.208054304	0.120368248
Zfp609|214812	-1.043777107	0.388877807	1.090106494	0.194181632
Yif1a|68090	-1.043808069	0.33492302	-1.153648029	0.050965915
Naip2|17948	-1.043818649	0.414609218	-1.128964641	0.265040131
Fam151b|73942	-1.043860533	0.438849515	1.355302925	0.026369906
Tbrg1|21376	-1.043862979	0.408492818	-1.04316444	0.38642065
Gng12|14701	-1.043943235	0.35759638	1.003855823	0.486770694
Mdm2|17246	-1.044002944	0.390288544	-1.098778546	0.251338745
Agpat6|102247	-1.04402651	0.304164376	-1.096497424	0.126624185
Fam19a5|106014	-1.044173626	0.462609445	-1.479388449	0.203729018
Rwdd2b|53858	-1.044212747	0.429107579	-1.044212747	0.429107579
1700049G17Rik|73430	-1.04421307	0.480549249	2.037346527	0.18884387
Cpsf3|54451	-1.044301778	0.319651402	1.053526273	0.245474719
Syp|20977	-1.044474946	0.37714983	1.118075452	0.143416338
H2-DMb1|14999	-1.044622084	0.457245839	-1.69582472	0.0580612
Fam100a|207740	-1.044715268	0.389235668	-1.139543705	0.199027079
Pola1|18968	-1.044721325	0.4649635	-1.044721325	0.4649635
Gm7854|665934	-1.044733585	0.466850921	-1.044733585	0.466850921
H3f3b|15081	-1.044751161	0.328797893	-1.044751161	0.328797893
Steap2|74051	-1.04476489	0.459874784	1.40945721	0.18164137
Ring1|19763	-1.044784906	0.366746118	1.02668589	0.408004788
Slc25a20|57279	-1.044791132	0.326424907	-1.077330229	0.18038604
Rbpms2|71973	-1.044805924	0.212286546	-1.031195168	0.246851245
AI413582|106672	-1.044826842	0.434349269	1.101310605	0.360696559
Mlf2|30853	-1.044845155	0.37641904	-1.119211942	0.214522614
Inpp1|16329	-1.044955552	0.429548955	-1.245913359	0.172004165
Gyk|14933	-1.04497592	0.40444011	1.134938103	0.212571231
Map4k2|26412	-1.04505249	0.441787021	1.092617532	0.373989064
Cops6|26893	-1.045165809	0.372653785	-1.221775869	0.028861145
Ttc13|234875	-1.045167331	0.357923247	-1.08221565	0.26659977
Mfsd10|68294	-1.045240649	0.401126935	1.072261217	0.331285837
Megf8|269878	-1.045410753	0.373564657	1.038057369	0.372489125
Ppp5c|19060	-1.045411051	0.335161504	-1.096538137	0.178766486
Gab1|14388	-1.045458611	0.420738682	1.229991132	0.111463955
Calm3|12315	-1.045608305	0.209543312	1.018318956	0.299339315
Zer1|227693	-1.045632945	0.305797439	1.002028537	0.489774199
Phactr2|215789	-1.045647734	0.412176848	1.222263887	0.049550994
Tmprss2|50528	-1.045651863	0.435037008	1.310819349	0.094815049
Mcam|84004	-1.04565989	0.424169154	-1.18271621	0.153656451
Tcp11l1|320554	-1.045755036	0.4496925	-1.045755036	0.4496925
Cops5|26754	-1.045766424	0.382723957	-1.150152007	0.16230844
0610037P05Rik|66086	-1.045770708	0.421200837	-1.045770708	0.421200837

C1qtnf1|56745	-1.045805722	0.344442073	-1.039642453	0.179911495
Cd59a|12509	-1.04581047	0.283666021	-1.072371542	0.115267818
Lrrfip1|16978	-1.045815665	0.439592667	1.360682264	0.07307331
Ifitm5|73835	-1.045841336	0.479934407	-3.98415	0.093264411
Mtus1|102103	-1.045856758	0.378414222	1.058839689	0.317525162
A2ld1|223267	-1.045877261	0.385476536	1.060220263	0.331326281
Fam96b|68523	-1.045883217	0.371946525	-1.129811909	0.183898296
Unc5a|107448	-1.045890437	0.428066081	-1.12345979	0.335499458
Kdsr|70750	-1.04589867	0.399335049	-1.047594289	0.372579764
Creb3l3|208677	-1.045997091	0.329656905	-1.045997091	0.329656905
ORF61|216157	-1.046057639	0.325817371	-1.103295053	0.138484414
Yipf6|77929	-1.046078417	0.369653337	-1.008206906	0.470231869
2810046L04Rik|212127	-1.046108925	0.444387725	-1.046108925	0.444387725
Clcn6|26372	-1.0462166	0.470733845	-1.756698102	0.200157308
Sft2d1|106489	-1.046287256	0.368840569	-1.046287256	0.368840569
Rfxank|19727	-1.046341655	0.373858935	1.001890849	0.494721432
Csnk2a2|13000	-1.046345628	0.358975779	-1.046345628	0.358975779
Haus1|225745	-1.046536771	0.426263182	-1.192898952	0.243143137
Foxp4|74123	-1.04654601	0.386411794	-1.144352999	0.193927284
Gtf2f2|68705	-1.046567506	0.4103588	-1.201775021	0.162858441
Zfp445|235682	-1.04664561	0.419313181	-1.010627155	0.464344824
Ppp4r1|70351	-1.046706004	0.382989826	1.049959037	0.36418197
Arih2|23807	-1.046732074	0.308300705	-1.046732074	0.308300705
Mtmr3|74302	-1.046732348	0.381990577	1.130018363	0.143351882
Hnf4a|15378	-1.046778663	0.296145918	-1.150493517	0.016797398
Acox2|93732	-1.046805989	0.379386285	1.104932607	0.196537701
Cdc16|69957	-1.046808854	0.374910814	-1.046808854	0.374910814
Hnrnpm|76936	-1.046833745	0.337724081	1.080163167	0.166094159
Glyat|107146	-1.04688363	0.306756435	1.012722489	0.43045322
Mmd|67468	-1.046950868	0.379543824	-1.163772813	0.128372184
Psme3|19192	-1.047219885	0.197248689	-1.072940919	0.103449622
5430416O09Rik|71406	-1.047247928	0.472067513	-1.290366661	0.346379627
Adprhl2|100206	-1.047252762	0.376172608	-1.2481755	0.017137048
Lsm14b|241846	-1.047350933	0.361664358	-1.047350933	0.361664358
Ccdc130|67736	-1.047364851	0.399143322	-1.133702779	0.232440768
Tceal1|237052	-1.047371519	0.475140161	1.973183884	0.200748856
Gon4l|76022	-1.047417406	0.399995646	-1.295465739	0.034405818
Hacl1|56794	-1.047425952	0.379828397	-1.047425952	0.379828397
Grhl3|230824	-1.047431906	0.483116311	1	#DIV/0!
Nat8l|269642	-1.047431906	0.483116311	1	#DIV/0!
Olfm2|244723	-1.047431906	0.483116311	1	#DIV/0!
Cpt1a|12894	-1.047466189	0.407491775	-1.143233074	0.23627006
Nat9|66176	-1.047546843	0.406634626	-1.047546843	0.406634626
Mrpl49|18120	-1.047564927	0.327279802	-1.11597021	0.132803081
Ehbp1l1|114601	-1.047595717	0.400569248	1.161743696	0.144950715
Surf4|20932	-1.047684572	0.24720069	-1.023090192	0.329260363
Litaf|56722	-1.047793202	0.311244274	-1.047793202	0.311244274
C1qtnf9|239126	-1.047871029	0.45896922	-1.973070551	0.009943005
Rexo1|66932	-1.047881383	0.285519329	-1.023041216	0.395325894
Morc2a|74522	-1.04796189	0.378748294	-1.234266915	0.022322643
Plekha1|101476	-1.048096768	0.447709279	1.309678177	0.1730362
Kif3a|16568	-1.048133049	0.359190301	-1.043053296	0.341387487
Cenpb|12616	-1.048145351	0.313689926	-1.048145351	0.313689926

Tomm22|223696	-1.048162922	0.341700073	-1.048162922	0.341700073
Trabd|67976	-1.048197447	0.375988565	1.133092505	0.109546831
Slc17a4|319848	-1.048332685	0.391715994	1.180881357	0.052468563
6330408A02Rik|321008	-1.048338722	0.401102457	1.157744603	0.179182403
Tbc1d13|70296	-1.048442759	0.380007104	-1.017558629	0.430828496
Yme1l1|27377	-1.048496971	0.358138174	-1.09421783	0.250172772
Rps19bp1|66538	-1.048519858	0.402030649	-1.126428134	0.281680269
Abcc3|76408	-1.048567589	0.323030442	-1.13264995	0.075553075
Tmem119|231633	-1.048586525	0.464839336	1.75158346	0.127450781
Proc|19123	-1.048653288	0.395123703	-1.180270419	0.161967213
Grk6|26385	-1.048662333	0.329306906	1.030054882	0.363443069
Sgk3|170755	-1.048666233	0.426107516	1.124339648	0.226860117
Abhd15|67477	-1.048709405	0.370375923	-1.246409766	0.013226298
Kdelr2|66913	-1.048748102	0.334653763	-1.107299304	0.184878781
9030025P20Rik|100041574	-1.048809655	0.433268285	1.076505449	0.403841095
Itih2|16425	-1.048846134	0.34924424	1.002094055	0.493417534
Senp5|320213	-1.048888224	0.421483247	-1.224420913	0.195582912
Hsf4|26386	-1.048909189	0.403361364	-1.318174502	0.024380414
2410001C21Rik|66404	-1.048928464	0.322075261	-1.048928464	0.322075261
Msra|110265	-1.049006345	0.249462107	-1.049006345	0.249462107
Tusc2|80385	-1.049037737	0.227654931	-1.016563184	0.391453228
Dhx16|69192	-1.049142075	0.344696131	-1.198629618	0.032105189
Pttg1|30939	-1.049167847	0.355595133	-1.024122262	0.407365614
D19Wsu162e|226178	-1.049269415	0.317174844	-1.133567675	0.066016421
Cd5l|11801	-1.049270421	0.380267649	-1.191259713	0.085897617
Ppp1r1b|19049	-1.049351709	0.417034336	1.065280707	0.380111061
0610011F06Rik|68347	-1.049416882	0.373704315	-1.141533027	0.18652911
Cnih|12793	-1.049429949	0.377783148	-1.049429949	0.377783148
Hist1h1c|50708	-1.049475442	0.407959299	-1.193916256	0.191285336
Pja1|18744	-1.049506205	0.348456724	1.03644098	0.349883876
2610301G19Rik|219158	-1.049506526	0.349107103	-1.049506526	0.349107103
Ercc6|319955	-1.049513747	0.42791712	-1.049513747	0.42791712
Pgrmc2|70804	-1.049600186	0.333837438	-1.049600186	0.333837438
Sin3b|20467	-1.049624467	0.350007335	-1.12571554	0.170155975
Plau|18792	-1.049696284	0.470380931	-1.927200277	0.17672832
Bcmo1|63857	-1.049697332	0.372013753	-1.163683061	0.129791303
Csnk2a1|12995	-1.049715541	0.374862923	1.065705785	0.300429287
Dnajc21|78244	-1.049784216	0.397064415	-1.046958268	0.361295294
Lrrc25|211228	-1.049804534	0.444274362	1.324137311	0.127740125
Atp5a1|11946	-1.049958439	0.295095692	-1.098776629	0.133080825
Edc4|234699	-1.049962229	0.169322031	-1.049962229	0.169322031
2700060E02Rik|68045	-1.049994454	0.357033826	1.007944727	0.475434439
Vprbp|321006	-1.050022106	0.339073934	-1.040946233	0.311321331
Med22|20933	-1.050105604	0.29977736	-1.070503604	0.167117531
Mea1|17256	-1.05012375	0.34472675	1.028158676	0.399033077
Cyp2j5|13109	-1.050175049	0.274378421	-1.028529525	0.283140225
Asnsd1|70396	-1.050198529	0.325262693	-1.047513866	0.268233603
Galk2|69976	-1.050280526	0.368025185	-1.025436661	0.401491637
Itpkc|233011	-1.050303531	0.383064633	-1.166659129	0.163460691
Chd9|109151	-1.050307613	0.419875496	1.290337295	0.043688026
Kptn|70394	-1.050323476	0.415073148	-1.279039508	0.089628848
Taok2|381921	-1.050510615	0.341726475	-1.050510615	0.341726475
Arhgef17|207212	-1.050573941	0.418090763	1.031408935	0.452434412

Unc50|67387	-1.050640102	0.400493854	1.023972523	0.429926236
Aen|68048	-1.050829153	0.306564363	-1.081018591	0.232368596
Taf11|68776	-1.050833827	0.374341353	-1.261680857	0.023505352
Ccdc164|381738	-1.05091797	0.44123352	-1.051461491	0.402990633
Klhl4|237010	-1.050931864	0.471072676	-1.809413362	0.226028814
Sfxn5|94282	-1.05097524	0.275000296	1.001479659	0.491840729
Mospd3|68929	-1.051033155	0.424836832	-1.041035799	0.429093058
Rars2|109093	-1.051045429	0.415609187	-1.260555318	0.13036389
Casp6|12368	-1.051149515	0.40831673	-1.051149515	0.40831673
Trio|223435	-1.051154097	0.449246803	1.49349412	0.089919524
Mier1|71148	-1.051160922	0.374781586	-1.010469689	0.454854183
Grwd1|101612	-1.0512403	0.380671504	1.065437173	0.326385335
Atp5d|66043	-1.051291316	0.370738658	-1.236161909	0.049034081
Ctdspl|69274	-1.051329134	0.421110922	1.067777013	0.401514927
Tmem68|72098	-1.051355205	0.42268925	1.111985762	0.33489327
Dscr3|13185	-1.051388092	0.346616587	-1.147386108	0.115148639
Angptl6|70726	-1.051398972	0.386127133	1.054378878	0.37225659
Cdh2|12558	-1.051402298	0.339194552	1.038903743	0.34317536
Apof|103161	-1.051436088	0.203007067	-1.051436088	0.203007067
Tapbp|21356	-1.051549182	0.306229824	-1.143927017	0.031727128
Kcnq1|16535	-1.051591711	0.451797074	-1.874576599	0.007051356
Pah|18478	-1.051596207	0.385828722	1.051764205	0.379393778
Mrps14|64659	-1.051599894	0.303234915	-1.051599894	0.303234915
Dbnl|13169	-1.051667258	0.255696831	-1.051667258	0.255696831
N4bp2l1|100637	-1.051703238	0.3716129	1.014300537	0.461882748
6720456H20Rik|218989	-1.051716511	0.39424696	-1.051716511	0.39424696
Osgin2|209212	-1.051745845	0.456211913	1.129754906	0.387322132
2510039O18Rik|77034	-1.051796443	0.303277137	-1.068461903	0.18566813
Gins3|78833	-1.051803454	0.470788619	-1.123868494	0.441486971
Asna1|56495	-1.051827523	0.378237035	-1.051827523	0.378237035
Esyt2|52635	-1.051930684	0.390431336	1.102085037	0.201946696
Xrcc5|22596	-1.051979331	0.297565576	-1.001251989	0.49450511
Erbb2ip|59079	-1.051984699	0.432591706	1.338556787	0.098610275
Yipf1|230584	-1.051998133	0.383854574	-1.202728962	0.101457076
2210012G02Rik|66526	-1.052116043	0.373196903	-1.068290707	0.275785091
Kpnb1|16211	-1.052123427	0.310168927	-1.102170003	0.162695008
Mkln1|27418	-1.052150755	0.384365921	1.027695865	0.438628181
Sri|109552	-1.052162413	0.330405157	-1.052162413	0.330405157
Hyou1|12282	-1.052208674	0.422310438	1.089065999	0.239463879
Islr|26968	-1.052283778	0.415873125	-1.038934284	0.40106454
Kpna4|16649	-1.052309139	0.395275457	1.203908201	0.058340452
Tmem5|216395	-1.052311819	0.415361513	-1.052311819	0.415361513
C1rl|232371	-1.052320508	0.310683062	-1.027750213	0.356336736
Rhbdf1|13650	-1.052397015	0.380055893	1.02190793	0.44990977
Plekho1|67220	-1.052429054	0.421007203	-1.133559974	0.223190582
Ttc17|74569	-1.05257098	0.345917996	-1.236561271	0.006781385
Nxt1|56488	-1.052774713	0.393343491	-1.012269361	0.460332679
Zbtb10|229055	-1.052803934	0.437885514	-1.218686057	0.252133213
Tmem37|170706	-1.052814829	0.404585581	-1.024194035	0.448860506
Zfp111|56707	-1.05284656	0.391721098	1.064201399	0.290613952
Brd1|223770	-1.052940184	0.421042231	1.025487958	0.461825812
Ddrgk1|77006	-1.05294113	0.380240753	-1.138198541	0.232354656
Tmem184c|234463	-1.053034731	0.437461522	-1.106345348	0.370532155

Nup98|269966	-1.053068565	0.374000776	-1.206037789	0.066693882
Smurf1|75788	-1.053104862	0.416850048	1.069437698	0.380870024
Clint1|216705	-1.053176768	0.407314114	1.101649302	0.313857981
Zcchc8|70650	-1.053220779	0.312018882	1.066827684	0.200965787
Wwox|80707	-1.053261621	0.397140949	-1.138096808	0.252460259
Med17|234959	-1.053267785	0.416626011	-1.053267785	0.416626011
Srsf2|20382	-1.053303477	0.350845634	1.018624988	0.439796836
Nipal3|74552	-1.05335474	0.423229778	1.294701911	0.09740734
Cerkl|228094	-1.053406502	0.454666428	-1.053406502	0.454666428
Gfm2|320806	-1.053410291	0.289231315	1.00421057	0.478634529
Gm6813|627939	-1.053433226	0.403327647	-1.190675385	0.207595511
Dub1|13531	-1.053527317	0.470697005	1.481669861	0.318085772
Usp35|244144	-1.053651236	0.431515671	-1.412295159	0.081534115
Fam125a|73711	-1.053732336	0.363011271	-1.009089272	0.471264925
Gm2a|14667	-1.053758784	0.353077893	-1.053758784	0.353077893
Mphosph8|75339	-1.053866453	0.450076515	1.255420717	0.295406679
Tspan33|232670	-1.053882996	0.394362397	-1.053882996	0.394362397
Pgm2|72157	-1.05388505	0.251609118	-1.144522849	0.022273229
Man2a1|17158	-1.053886917	0.259280425	-1.025469741	0.263445085
Larp4|207214	-1.05391149	0.402601257	1.070327449	0.258829034
Tmed4|103694	-1.053925528	0.32821288	-1.056122479	0.324121953
Ccdc85b|240514	-1.05393033	0.42623138	-1.05393033	0.42623138
D10Wsu52e|28088	-1.053957575	0.269914455	-1.078145881	0.204121228
1500011K16Rik|67885	-1.0539637	0.318279795	-1.109295963	0.164819465
Slc35a1|24060	-1.054017456	0.372804477	-1.260609471	0.043183685
Trp53rk|76367	-1.054098993	0.422982459	-1.030173418	0.453400109
Krt18|16668	-1.054107743	0.367530743	-1.14675816	0.191152444
1190003J15Rik|76974	-1.054132139	0.365601233	-1.133100994	0.216476555
Polr2i|69920	-1.054163548	0.383960738	-1.178726756	0.119187471
Cdkal1|68916	-1.054206678	0.418127996	-1.252738032	0.181111049
Pmvk|68603	-1.054233822	0.370296031	-1.080058343	0.27712045
Atg4c|242557	-1.054242939	0.386044684	-1.095347546	0.223929289
Mup5|17844	-1.054253062	0.413732448	-1.033103187	0.392919644
Mrpl12|56282	-1.054338763	0.359899523	-1.160323919	0.141669506
Lsmd1|78304	-1.054405806	0.397102806	1.037639693	0.419253673
Ankrd35|213121	-1.054420727	0.476263483	-4.016833333	0.093227909
Rab40b|217371	-1.054420727	0.476263483	-4.016833333	0.093227909
Kpna6|16650	-1.054436362	0.361099075	-1.007272413	0.468593941
Dcps|69305	-1.054524158	0.364362406	-1.054524158	0.364362406
Prpf40a|56194	-1.05454556	0.434407542	1.21889119	0.240414953
Ltn1|78913	-1.054571528	0.378897665	1.062287971	0.346101589
Grb7|14786	-1.05462454	0.267252889	-1.05462454	0.267252889
Kdm5c|20591	-1.054631208	0.278115031	-1.054631208	0.278115031
Wdr46|57315	-1.054633662	0.35001008	1.074184011	0.27054121
Pzp|11287	-1.054664789	0.399484024	-1.366574851	0.01637783
Txn1|22166	-1.05467129	0.303444657	1.046630886	0.295941127
Tcf20|21411	-1.054701758	0.372050878	-1.007711374	0.474551575
Ptms|69202	-1.05472435	0.425921413	-1.157422394	0.20840897
Hcfc1r1|353502	-1.05473787	0.374419894	1.040442222	0.401935378
Uckl1|68556	-1.054742083	0.38509755	-1.127699034	0.215523779
Mal|17153	-1.05483	0.478341436	-2.8202	0.173296754
Snip1|76793	-1.054872749	0.3980532	1.030939519	0.439131775
Zfp691|195522	-1.054903229	0.376655925	-1.054903229	0.376655925

Bcl2l2|12050	-1.054925785	0.431326584	1.192196411	0.26333273
Aktip|14339	-1.055028211	0.406054624	-1.154580917	0.210612364
Gm5766|436332	-1.05505964	0.408870112	-1.05505964	0.408870112
5830416P10Rik|381232	-1.055094871	0.479210987	3.3	0.173296754
Tomm5|68512	-1.055103785	0.380056615	-1.055103785	0.380056615
Cox10|70383	-1.0551098	0.327255799	-1.138517089	0.088562089
Mrap|77037	-1.055119651	0.327975571	-1.110969824	0.20230112
Serpinc1|11905	-1.055128723	0.194195132	-1.096281627	0.043087258
Mgea5|76055	-1.055176333	0.396613278	1.053717095	0.38602698
Tnfaip8l2|69769	-1.055199081	0.425071413	1.08940732	0.36637518
Srpr|67398	-1.055218531	0.233317861	-1.007435361	0.450845864
Letm2|270035	-1.055243192	0.409938642	1.079052352	0.351259307
Pon1|18979	-1.055251296	0.32345142	1.012062298	0.456435385
Efr3b|668212	-1.055332313	0.47449419	1.150701957	0.450338304
Zfr|22763	-1.055390638	0.427532048	1.069192251	0.321784499
AW146154|101835	-1.055458816	0.446427105	-1.380110041	0.219843227
Tmem168|101118	-1.055487982	0.41662487	-1.303488639	0.11473491
St3gal4|20443	-1.055561453	0.272760925	-1.021727153	0.355795171
Scamp4|56214	-1.055583189	0.282576133	-1.070927415	0.175405399
Fam175a|70681	-1.055761876	0.428899488	-1.196302832	0.262683467
Ddx55|67848	-1.05576635	0.366151258	-1.07308869	0.269629398
Chst2|54371	-1.055804671	0.399676989	-1.11962433	0.207534161
Txndc11|106200	-1.055834923	0.393229927	-1.211840334	0.153095059
Icam4|78369	-1.055895156	0.480218211	1	#DIV/0!
Anxa11|11744	-1.055923349	0.303798841	1.005681015	0.475365629
Nrbf2|641340	-1.055926906	0.392911692	1.079927493	0.303193823
Tcte2|21646	-1.056007366	0.469443787	1.488232276	0.316376312
Pcbp2|18521	-1.056020171	0.280446937	1.016311763	0.30908079
Rbm26|74213	-1.056122887	0.446202226	1.395613884	0.136648334
Birc6|12211	-1.056131346	0.366472782	1.027724442	0.344710228
Bbox1|170442	-1.056280315	0.338540693	1.006213749	0.480010448
1810013D10Rik|66278	-1.056304571	0.360409019	-1.129523953	0.225041423
Abcf3|27406	-1.056330688	0.22323203	-1.056234472	0.153190563
Kras|16653	-1.056335916	0.352916402	1.048766015	0.345252305
Krba1|77827	-1.056337242	0.380502481	-1.056337242	0.380502481
C2cd2l|71764	-1.056372867	0.333923631	-1.072534692	0.198103366
Cd72|12517	-1.056501795	0.429791809	-1.229911766	0.220908441
Syvn1|74126	-1.056518339	0.392366016	-1.003283811	0.489952724
Dad1|13135	-1.056554555	0.355714259	1.046526315	0.36029988
Psen1|19164	-1.05659054	0.222070784	-1.104242853	0.072136553
Cul4a|99375	-1.056597178	0.384673451	1.157946379	0.166620669
Ngf|18049	-1.056619551	0.399722682	-1.056619551	0.399722682
Adarb2|94191	-1.056664549	0.47661978	1.428581492	0.383239861
Map3k7|26409	-1.056678679	0.373092414	-1.184935134	0.142991706
Tmem150a|232086	-1.056699515	0.305063062	-1.079139213	0.209184769
Fam199x|245622	-1.05670915	0.377831797	-1.09033212	0.276006234
Fam20a|208659	-1.056731807	0.354651661	-1.147758306	0.175003242
Naa16|66897	-1.056745632	0.412271738	-1.448718417	0.007327985
Deptor|97998	-1.056773398	0.388879829	1.035827728	0.350642887
Mob1b|68473	-1.056779997	0.43439072	1.125605746	0.270595644
Abhd5|67469	-1.05685726	0.281837939	-1.123215493	0.068524655
Smarcal1|54380	-1.056884855	0.403936999	1.037265385	0.376463016
Ilk|16202	-1.056895186	0.320645511	1.08232083	0.171145812

Ftsj3|56095	-1.056974367	0.314119572	1.017145827	0.426408249
Klraq1|73825	-1.056974707	0.369811057	-1.005580152	0.48765328
Gm8615|667410	-1.056982799	0.380330064	-1.097226642	0.268324326
AI317395|215929	-1.056994726	0.383607422	1.15035317	0.184949447
Prkcsh|19089	-1.057041727	0.337143856	-1.057041727	0.337143856
Endou|19011	-1.057057135	0.477310394	-8.121	0.046661899
Tmem159|233806	-1.057098552	0.360723975	-1.076739949	0.286473052
D4Ertd22e|213491	-1.05720539	0.277582415	-1.071948963	0.162054735
Map2k7|26400	-1.057223356	0.330153563	1.082901739	0.210533051
Tnfrsf11a|21934	-1.057225662	0.457811156	-1.761738237	0.129185532
Spry1|24063	-1.057258484	0.413396541	-1.206464284	0.240746615
Dpy30|66310	-1.05734868	0.371497074	1.05756507	0.354963042
Ubxn4|67812	-1.057403294	0.258649067	-1.007569725	0.461842415
Enpp4|224794	-1.057407304	0.44825232	1.186259146	0.298925108
Pex6|224824	-1.057523795	0.35411076	-1.205935454	0.042857708
Adrbk1|110355	-1.057528219	0.218308305	-1.096679019	0.101318511
Gm10767|100038538	-1.057607814	0.39370366	1.211785302	0.084079804
Slc7a1|11987	-1.057622589	0.458973409	1.890534483	0.069915791
Gdpd5|233552	-1.057685162	0.443507123	-1.200569815	0.317403676
Lhpp|76429	-1.057708372	0.324124219	1.028570746	0.378379421
Lpar5|381810	-1.057714028	0.467373956	-1.821090369	0.224432855
Lrrc16b|268747	-1.057714028	0.467373956	-1.821090369	0.224432855
1810074P20Rik|67490	-1.057741415	0.37827903	1.151649028	0.156252362
Tceb3|27224	-1.057749885	0.31658121	-1.118722347	0.155926487
Mcm4|17217	-1.057755473	0.441379359	1.16121803	0.355039293
Atg13|51897	-1.057784086	0.315103203	-1.122486653	0.140753288
Katnal1|231912	-1.057796941	0.427294884	-1.181662258	0.311445896
Meis2|17536	-1.057856076	0.42060554	-1.329690786	0.125440281
Arid3b|56380	-1.057894276	0.435231155	-1.443970963	0.106640904
Rnf170|77733	-1.057898698	0.43236928	-1.034950273	0.451469674
Ppp2r5a|226849	-1.057969992	0.339475277	1.039638735	0.364161668
Cflar|12633	-1.05802986	0.242382159	1.039999284	0.206315784
Afm|280662	-1.058041037	0.374448398	-1.160578517	0.168970102
Ranbp1|19385	-1.058078191	0.353085891	-1.129490655	0.219793147
Gm4952|240549	-1.05812561	0.375094354	-1.05812561	0.375094354
Rpl31|114641	-1.058209087	0.411982159	-1.280712635	0.14487834
Ankzf1|52231	-1.058232854	0.339231945	-1.058232854	0.339231945
Mll1|214162	-1.058249267	0.400748755	1.078908606	0.254331472
Rala|56044	-1.058261117	0.363618849	-1.074149395	0.304186859
Rer1|67830	-1.058315168	0.148654654	-1.081758289	0.084613657
Arap1|69710	-1.05834277	0.302271698	-1.123035074	0.118554223
Zscan29|99334	-1.058358128	0.377667765	-1.183434844	0.171759542
Pttg1ip|108705	-1.058628104	0.349991742	1.035034025	0.398307039
Errfi1|74155	-1.05871109	0.350141375	1.11785144	0.148562124
Cdk11b|12537	-1.0587124	0.252611239	-1.117411979	0.058903408
Slc39a13|68427	-1.058716562	0.283609919	-1.123406815	0.112706153
Ahr|11622	-1.058754013	0.437643653	1.474601542	0.06721011
Ubxn2a|217379	-1.058820606	0.346818665	1.019709759	0.44469135
Atg10|66795	-1.058866135	0.420785444	-1.058866135	0.420785444
Bptf|207165	-1.058957903	0.419781974	-1.099530844	0.337007635
Uox|22262	-1.058984766	0.266215468	-1.084458912	0.151583927
Erf|13875	-1.059036277	0.30277817	-1.059036277	0.30277817
BC020535|228788	-1.059045478	0.406468283	1.041875297	0.42965397

Mrpl45|67036	-1.059091077	0.324892468	-1.20670922	0.046274423
Ik|24010	-1.059127076	0.32161168	-1.022497465	0.363484746
Tspan31|67125	-1.059142302	0.270732774	-1.123529054	0.094036092
Patl1|225929	-1.05914967	0.381044747	-1.188602591	0.125777445
Taok1|216965	-1.059162085	0.401882981	1.116149603	0.224402439
Als2cl|235633	-1.059196307	0.33868448	1.040177558	0.364237761
2700049A03Rik|76967	-1.059244155	0.46086859	1.185286783	0.400930459
Dstyk|213452	-1.05935437	0.368280384	1.153980378	0.08859516
Hand2|15111	-1.059358916	0.293928836	-1.059358916	0.293928836
Iars2|381314	-1.059492048	0.239139392	-1.158039297	0.016352731
Rnps1|19826	-1.059502483	0.30476788	-1.059502483	0.30476788
Il16|16170	-1.059513231	0.396902484	-1.120635701	0.310465202
Fam96a|68250	-1.059539587	0.236840719	-1.029116004	0.361797504
Fth1|14319	-1.059540012	0.29839925	-1.144034878	0.045444812
Ccdc50|67501	-1.059567938	0.390961526	1.199876333	0.113001539
Brap|72399	-1.05957712	0.292263955	-1.017030627	0.438335764
Ppp6r3|52036	-1.059634853	0.280783641	-1.059634853	0.280783641
Zfp167|382118	-1.059653338	0.469589216	1.216561098	0.370645645
Tfcp2|21422	-1.059656272	0.386509888	1.207749976	0.060082406
Letmd1|68614	-1.059661437	0.331332249	1.095706898	0.172064076
Tnfsf13b|24099	-1.059698432	0.468900983	-2.349388889	0.152090257
Klhdc4|234825	-1.05976361	0.379463858	-1.152785952	0.238761521
Cog2|76332	-1.059791078	0.359279099	1.118732692	0.195948506
Setd3|52690	-1.059906065	0.200656361	-1.117146658	0.026662636
Vps4b|20479	-1.059907979	0.389745302	-1.021564235	0.446876183
Bccip|66165	-1.060063381	0.249320005	-1.115886682	0.092570295
Gm13251|433791	-1.060091765	0.478912219	-2.76078	0.173296754
Crip3|114570	-1.060108391	0.468545088	1.859246314	0.224452502
1110038D17Rik|68778	-1.060124933	0.314362853	1.081596209	0.167607088
Lrrc20|216011	-1.060144427	0.430498083	-1.365029376	0.165279158
Cdo1|12583	-1.060172391	0.236554953	-1.107184142	0.104606008
Ubb|22187	-1.060271617	0.350792165	-1.118513878	0.248662911
Pard6b|58220	-1.060277195	0.420409689	-1.008977297	0.485398802
Hsf1|15499	-1.060323943	0.272933053	-1.043109217	0.305594034
2810408M09Rik|381406	-1.0603355	0.322492223	-1.112678022	0.200704982
Ap2a1|11771	-1.060336762	0.30154962	-1.063916692	0.230533076
Ptprk|19272	-1.060369935	0.369629731	-1.060369935	0.369629731
C2cd3|277939	-1.060373845	0.397170796	1.210130945	0.139077005
Clcc1|229725	-1.06040547	0.252029788	-1.06040547	0.252029788
Mlph|171531	-1.060406035	0.415652102	1.086420167	0.363787417
Fhl3|14201	-1.060417705	0.457101344	1.391127896	0.283048041
Gyg|27357	-1.060431027	0.421169853	-1.480378671	0.050405516
Gm527|217648	-1.060476119	0.465840342	-1.825845924	0.2234056
Slc39a7|14977	-1.060516489	0.307661418	1.016025677	0.437605532
Rrp1|18114	-1.060538787	0.328322582	-1.012047266	0.464075149
2700050L05Rik|214764	-1.060542108	0.369931421	-1.278956084	0.056597356
Lztfl1|93730	-1.060545472	0.414563886	-1.060545472	0.414563886
Plekhm1|353047	-1.060563816	0.315608941	-1.212773676	0.020167887
Jagn1|67767	-1.060650181	0.286024358	1.001256516	0.494384066
Agxt2l2|72947	-1.060674742	0.274401713	-1.060674742	0.274401713
Tfdp1|21781	-1.060749602	0.413922107	-1.060749602	0.413922107
Gm5506|433182	-1.060755215	0.291229391	-1.039204357	0.315925838
Bivm|246229	-1.06082568	0.450247667	1.421271659	0.206216878

LOC100048884|100048884	-1.060837091	0.449059083	1.318251823	0.274662359
Ankrd13b|268445	-1.060864749	0.421255401	-1.36420822	0.115172095
Otub1|107260	-1.060901265	0.371636934	-1.060901265	0.371636934
Prpf8|192159	-1.060905767	0.138909123	-1.039031722	0.239078902
Tpp1|12751	-1.06092756	0.257095014	-1.145679097	0.020847763
Elmod2|244548	-1.06097693	0.415542672	-1.311046678	0.145426956
Pqbp1|54633	-1.060996331	0.270817876	-1.036959261	0.361758231
Xkr6|219149	-1.061009994	0.462433669	1.400397676	0.317642215
Axin1|12005	-1.061033851	0.194602585	-1.039334884	0.231585818
Vipr1|22354	-1.061096435	0.251939736	-1.017701094	0.419637419
Phf2|18676	-1.061107712	0.403870287	-1.005802143	0.489184589
Mrps6|121022	-1.061129468	0.391566384	-1.061129468	0.391566384
Adssl1|11565	-1.061130096	0.317698335	-1.061130096	0.317698335
Nlrp3|216799	-1.061134297	0.45695867	1.507589768	0.222833067
Lrrc3|237387	-1.061190601	0.191141009	-1.061190601	0.191141009
Flt3|14255	-1.061229715	0.466849126	-1.827587288	0.237280066
Dda1|66498	-1.061234741	0.194969446	-1.047155409	0.18031207
Crip1|12925	-1.061261667	0.447823571	-1.611788948	0.128549377
Uggt1|320011	-1.061285263	0.334871764	-1.061791392	0.298702989
Cecr5|214932	-1.061364156	0.248793769	-1.017247954	0.420130376
Mapk9|26420	-1.061392444	0.354129233	-1.257005456	0.050307203
Eif2c4|76850	-1.061405001	0.443394456	-1.362311823	0.246752633
Cggbp1|106143	-1.061428561	0.327342658	-1.061428561	0.327342658
Taf8|63856	-1.061433303	0.394893716	1.046417991	0.421895932
Stxbp2|20911	-1.061492739	0.278144334	-1.121647516	0.127389748
Sel1l3|231238	-1.061503144	0.386134981	-1.225698191	0.145449973
Pla2g16|225845	-1.061527772	0.358979748	1.048650362	0.372959989
Ccdc80|67896	-1.061551008	0.271722164	-1.139521914	0.063566993
Zfp414|328801	-1.0615571	0.37770558	-1.0615571	0.37770558
Fam100b|319370	-1.061604293	0.325384543	-1.058810981	0.355344198
Clp1|98985	-1.061667259	0.406892168	-1.061667259	0.406892168
Xrcc1|22594	-1.06169478	0.326674673	-1.06169478	0.326674673
Pitpnb|56305	-1.061709373	0.260667872	-1.061709373	0.260667872
5430411K18Rik|100502841	-1.061809943	0.434707409	-1.061809943	0.434707409
Mpp1|17524	-1.062001368	0.347606689	1.151920947	0.015984049
Fam120c|207375	-1.062060519	0.422132948	-1.30236472	0.194481755
Cplx2|12890	-1.062075914	0.312542954	-1.056600192	0.276764565
Tcf25|66855	-1.062140464	0.133162335	-1.044241672	0.137738637
Lama1|16772	-1.062148213	0.446476716	-1.27160003	0.276447384
Dgcr6|13353	-1.062228177	0.383415559	1.108977104	0.249650028
Cttn|13043	-1.062245267	0.320393939	-1.061690428	0.314700885
Sh3bp5l|79566	-1.062311276	0.299470001	-1.062311276	0.299470001
Hpn|15451	-1.062318287	0.243790967	-1.062318287	0.243790967
Las1l|76130	-1.062329013	0.257946314	-1.111597723	0.132721039
Pdcd4|18569	-1.062379255	0.204602823	1.011130631	0.423168904
Slc29a1|63959	-1.062398251	0.305705808	-1.062398251	0.305705808
Ide|15925	-1.062451721	0.285949439	-1.062451721	0.285949439
Sfi1|78887	-1.062537804	0.347463042	1.034909003	0.391846455
Tmem38a|74166	-1.062587094	0.386205127	-1.164133345	0.220875401
Gm6907|628705	-1.062598948	0.45497877	1.087130534	0.426277191
Golt1a|68338	-1.062601457	0.327859462	-1.116661215	0.225343989
Arf1|11840	-1.062615567	0.253861925	-1.007405643	0.462420649
Scrn2|217140	-1.062741316	0.3730716	-1.200480545	0.155543982

Ablim1|226251	-1.062743628	0.331456688	1.011851584	0.462487372
Gcnt4|218476	-1.062756634	0.435192947	-1.062756634	0.435192947
Vps37a|52348	-1.062766945	0.434730863	1.106206276	0.349721482
Zfp207|22680	-1.062882187	0.391778954	-1.0361044	0.394455542
Ggps1|14593	-1.062920087	0.426251015	-1.234559858	0.235208288
Scrib|105782	-1.06294829	0.23900056	-1.123200008	0.049355127
Reep3|28193	-1.063078051	0.344126724	-1.165799011	0.150641952
Tk2|57813	-1.063120818	0.276137715	-1.145733619	0.029308682
Mrps18c|68735	-1.063133549	0.36240913	-1.122342192	0.258385137
Cwc27|67285	-1.06313845	0.345086921	-1.213774687	0.047699943
Slmap|83997	-1.063163395	0.420821162	1.368276479	0.050807732
Edf1|59022	-1.063173982	0.367691468	-1.177594569	0.184888364
Habp4|56541	-1.063206887	0.258560033	-1.063206887	0.258560033
Flnb|286940	-1.063224794	0.230347741	-1.049239403	0.230106648
Rprd2|75137	-1.063285071	0.36549758	-1.038420802	0.354550603
Mrps26|99045	-1.063291901	0.362372126	1.012755195	0.469701857
Gas2|14453	-1.063361268	0.384011667	-1.063361268	0.384011667
Rhbdl2|230726	-1.06336538	0.38573672	-1.06336538	0.38573672
Dctn4|67665	-1.063377547	0.325720492	-1.081829116	0.2342399
Kif9|16578	-1.063469835	0.457069171	1.310268853	0.335902723
Nadsyn1|78914	-1.063491653	0.341514201	1.00659246	0.482822163
Tars2|71807	-1.063636979	0.328671818	1.024392126	0.415300335
Lta4h|16993	-1.063677291	0.343284256	1.09823069	0.195460637
Sdhc|66052	-1.063682826	0.153695037	-1.05696063	0.112014916
Arfgap2|77038	-1.06368566	0.160027951	-1.110566583	0.028381468
Cables2|252966	-1.063711576	0.355718327	-1.173067816	0.166937726
5133401N09Rik|75731	-1.063732054	0.362813862	-1.223439731	0.086732358
Gnl3l|237107	-1.063759476	0.39768614	1.086283068	0.287713458
Sec24a|77371	-1.063780728	0.392565707	1.232292523	0.083888827
Slu7|193116	-1.063808356	0.292536211	-1.020820289	0.430562588
Brms1l|52592	-1.063820762	0.430869188	1.4277651	0.085984859
Uba52|22186	-1.063867268	0.391291851	-1.221150439	0.185193243
BC053749|333193	-1.063907682	0.407512191	-1.248671116	0.204297273
Pepd|18624	-1.063975668	0.306954603	-1.063975668	0.306954603
Stxbp1|20910	-1.063997838	0.402604158	1.046495844	0.423655999
Dld|13382	-1.064049682	0.268158433	-1.104735464	0.163424368
Glce|93683	-1.064059356	0.398051413	1.10774016	0.317941828
Homer1|26556	-1.064162277	0.434760774	1.121579257	0.364669254
Cd38|12494	-1.064172777	0.224707936	-1.116394228	0.083521642
Zeb1|21417	-1.064190954	0.425208418	1.077864705	0.367603618
Ctf1|13019	-1.064209838	0.326565004	-1.090596458	0.203256832
Sorbs2|234214	-1.064234045	0.390147211	-1.064234045	0.390147211
Itga3|16400	-1.06427016	0.351816441	-1.06427016	0.351816441
Rab22a|19334	-1.06430954	0.304908981	-1.06430954	0.304908981
Adarb1|110532	-1.064323845	0.389313011	-1.185672052	0.195968316
Ccl6|20305	-1.064407301	0.359553814	1.064707307	0.29576868
Gnat1|14685	-1.064411458	0.407530987	-1.276189942	0.174278896
Cc2d1b|319965	-1.064492928	0.331934633	-1.018794248	0.451843984
Tbc1d2b|67016	-1.06453399	0.308145989	-1.06453399	0.308145989
Colec12|140792	-1.064593452	0.359654638	-1.119915903	0.205267336
Glyctk|235582	-1.064613708	0.091904485	-1.064613708	0.091904485
Rras|20130	-1.064628316	0.177111969	-1.097822998	0.079604431
Gnl2|230737	-1.064653665	0.334066647	-1.134371729	0.206424258

Maml2|270118	-1.064712418	0.42719873	-1.064712418	0.42719873
Btbd6|399566	-1.064726777	0.290722716	-1.135233639	0.101486242
Trim25|217069	-1.064766342	0.342651676	1.117870005	0.1586771
Gemin4|276919	-1.064767404	0.35298285	-1.13731407	0.214851347
Fbxo6|50762	-1.064775961	0.313813723	1.017222238	0.441069311
Dctn6|22428	-1.064904927	0.259604671	-1.011085967	0.452029495
Diexf|215193	-1.06496139	0.310458224	-1.06496139	0.310458224
Erlec1|66753	-1.065002406	0.364932411	1.043125384	0.403716627
Palm|18483	-1.065069586	0.439502421	1.098774323	0.380227946
Zrsr1|22183	-1.065101056	0.374666653	-1.118286952	0.294443011
2410015M20Rik|224904	-1.065222578	0.305015811	-1.111948112	0.104170848
Tmem38b|52076	-1.065290682	0.257376817	-1.144063832	0.0225482
Zfand2b|68818	-1.065335061	0.369126232	-1.199517245	0.162195824
Pigk|329777	-1.065348414	0.253816158	-1.007542667	0.464998159
Polr1a|20019	-1.065435124	0.300791338	1.001345663	0.495031816
Eps8l2|98845	-1.065445725	0.285018197	-1.016678133	0.416077453
Cpne8|66871	-1.065501737	0.417538656	-1.026943819	0.463499936
Uso1|56041	-1.065545256	0.328537704	1.020802608	0.431711007
Dnttip1|76233	-1.065616752	0.336143183	1.093128882	0.229492606
Ocrl|320634	-1.065637753	0.424779751	1.076478314	0.406737669
Arrb2|216869	-1.065663511	0.331517374	-1.013648248	0.465008522
Hax1|23897	-1.065740304	0.280164598	1.01009756	0.45545491
Cstb|13014	-1.065771362	0.343045432	-1.211373781	0.06780594
Gcap14|72972	-1.065820199	0.296074193	-1.112723189	0.105042085
Cep164|214552	-1.065821978	0.379300541	1.111968169	0.242868777
Sox13|20668	-1.065850689	0.328710368	-1.065850689	0.328710368
Stat3|20848	-1.065859175	0.243830074	-1.065859175	0.243830074
Ubc|22190	-1.06588766	0.37346771	-1.06588766	0.37346771
Mx2|17858	-1.065904698	0.417976796	-1.065904698	0.417976796
Tmem111|66087	-1.065962426	0.217479916	-1.051631603	0.172296827
Mrpl14|68463	-1.065963243	0.387906109	-1.134944235	0.194052354
Ptgs1|19224	-1.0659948	0.31715071	-1.0659948	0.31715071
Sypl|19027	-1.066073654	0.284554132	1.069106121	0.174033872
Tmem218|66279	-1.06607667	0.403189027	-1.067085755	0.315870351
Zc3h8|57432	-1.066101653	0.438196294	-1.717616038	0.070672082
Tle1|21885	-1.066135114	0.321464776	-1.066135114	0.321464776
Anapc1|17222	-1.066155837	0.301892611	-1.066155837	0.301892611
Anks1|224650	-1.066248774	0.361111924	-1.066248774	0.361111924
Slco2a1|24059	-1.066332138	0.350138981	-1.073627614	0.285930564
Ulk1|22241	-1.066373991	0.325541423	-1.02893772	0.358100269
Recql5|170472	-1.0663961	0.342977587	-1.076965941	0.281336103
C130074G19Rik|226777	-1.066404264	0.199644718	-1.01964486	0.381675526
Rit1|19769	-1.066410194	0.333165757	-1.066410194	0.333165757
4831440E17Rik|320965	-1.066429736	0.445738176	-1.497742744	0.208788028
Srp9|27058	-1.066456355	0.294644164	-1.171455803	0.052831759
Myzap|102371	-1.066473724	0.45442757	-1.795267073	0.153502328
Etfdh|66841	-1.066505499	0.37260353	1.177150542	0.127843479
Serp1|28146	-1.066512716	0.348409929	-1.054011368	0.306202838
Zcchc17|619605	-1.066520059	0.327374448	-1.077894976	0.231203248
Esd|13885	-1.066575488	0.308353657	1.024672062	0.408711619
Dctn2|69654	-1.066579403	0.257162393	-1.098233894	0.191677431
Gcfc1|67367	-1.066655109	0.394331551	-1.216035127	0.216241936
Phf1|21652	-1.06665828	0.337782795	-1.282382071	0.012907382

Cdk5rap2|214444	-1.066704289	0.394253874	-1.037120726	0.41234903
Tec|21682	-1.066823865	0.330462422	-1.140142901	0.17462796
Trappc4|60409	-1.066835913	0.280583989	-1.134851992	0.125959215
Usp30|100756	-1.066844576	0.341166299	-1.283420665	0.020188741
E2f5|13559	-1.066875663	0.354531093	-1.066875663	0.354531093
AK010878|100233175	-1.066939802	0.294382995	-1.084970149	0.124909965
Rdh5|19682	-1.066943208	0.388494258	-1.294163732	0.087036484
Gltpd2|216871	-1.066978047	0.343674688	-1.163197614	0.177498193
Phpt1|75454	-1.066983048	0.352512781	-1.150048915	0.079990222
Ptpn12|19248	-1.066984041	0.444965098	-1.114804833	0.410602489
Arl6ip4|65105	-1.066988768	0.368413094	-1.177671308	0.206367418
G6pc3|68401	-1.066992557	0.30698694	-1.140011148	0.131272767
Mdfi|17240	-1.067021148	0.449237688	-1.067021148	0.449237688
Rnf126|70294	-1.067021419	0.313228713	1.009426328	0.467645758
Aadat|23923	-1.067121633	0.284566771	-1.1286167	0.128247726
C1ra|50909	-1.067172953	0.126807778	-1.067172953	0.126807778
Ndfip2|76273	-1.067173659	0.195212142	-1.067173659	0.195212142
2510003E04Rik|72320	-1.067203849	0.350506777	-1.067203849	0.350506777
Dexi|58239	-1.067216869	0.347264209	-1.191868779	0.129969119
Shank3|58234	-1.067223585	0.35235453	-1.203878591	0.119616676
Trnt1|70047	-1.067243958	0.404884913	-1.474987689	0.029298839
Kpna2|16647	-1.067253775	0.3659178	1.044752452	0.39152566
Mgat1|17308	-1.067274112	0.25979643	-1.05355955	0.255087258
Pear1|73182	-1.067275531	0.217956496	-1.062618306	0.197782023
Sergef|27414	-1.067342623	0.408294157	-1.05406157	0.399138385
Mapkapk5|17165	-1.067456433	0.301726179	1.086314329	0.155076331
Peli2|93834	-1.067510369	0.378955933	1.178377493	0.166289612
Fam84b|399603	-1.067521407	0.419001574	1.018395077	0.470346219
Nicn1|66257	-1.067640008	0.362341912	1.059788196	0.336717716
Dis3l|213550	-1.067687015	0.33848629	-1.177522296	0.14098774
E430025E21Rik|223593	-1.067692967	0.345156217	1.022727635	0.443853053
Lcat|16816	-1.067731732	0.288741662	-1.220356203	0.015478075
Yif1b|77254	-1.06783555	0.351631165	-1.090270117	0.254630146
Prrc2a|53761	-1.067837487	0.168169049	-1.067837487	0.168169049
Acadm|11364	-1.067851068	0.34015707	-1.067851068	0.34015707
Auh|11992	-1.067913336	0.24609568	-1.131513262	0.063889935
Eif2b1|209354	-1.06791361	0.313324232	-1.131699269	0.192359887
Phf19|74016	-1.067920409	0.451621845	-1.263536548	0.350430504
Ift52|245866	-1.067959236	0.310111043	-1.178146012	0.078156591
Btd|26363	-1.067969159	0.158820317	-1.153013849	0.00315476
Klhl7|52323	-1.067982568	0.403694455	-1.275225326	0.181465346
Sav1|64010	-1.068008135	0.408175508	1.265938244	0.161687291
Pm20d2|242377	-1.068053842	0.452423697	-1.101640999	0.415350534
Cops2|12848	-1.068080415	0.301003848	-1.221521954	0.035299663
Phf14|75725	-1.068207518	0.294703339	1.073317453	0.211022633
Anks6|75691	-1.068214984	0.454887509	-1.145341761	0.404553878
H2-Q10|15007	-1.068238273	0.297059728	-1.239960635	0.001986039
Tmem98|103743	-1.068264741	0.354807678	-1.21620946	0.109761258
Rfesd|218341	-1.068280592	0.417150295	-1.141106368	0.286854611
Git1|216963	-1.068337003	0.280929466	-1.003942467	0.481839198
Arl1|104303	-1.068379187	0.42934881	1.041242192	0.448606294
Zmym4|67785	-1.06840208	0.430474139	-1.06840208	0.430474139
Cdk16|18555	-1.068414346	0.282327441	-1.068414346	0.282327441

Aplp2|11804	-1.068463245	0.242717653	-1.14133838	0.060006368
Ppp4r1l-ps|100043911	-1.068485592	0.381133627	1.179142718	0.181794119
Apbb2|11787	-1.068512324	0.357002758	1.002204883	0.49538661
Zfp87|170763	-1.068571756	0.430785193	-1.068571756	0.430785193
Casp8|12370	-1.068582439	0.300527637	1.016742911	0.439016456
Pih1d1|68845	-1.068713085	0.361616999	-1.064738297	0.336015195
Fam69a|67266	-1.068736586	0.332516193	-1.162936262	0.128793144
Ifnar2|15976	-1.068751302	0.248386311	-1.137354591	0.053224116
Prkce|18754	-1.068835874	0.336038952	-1.068835874	0.336038952
Gp49a|14727	-1.068847892	0.421152553	-1.068847892	0.421152553
Mrps31|57312	-1.069194395	0.248413947	-1.031122433	0.254727723
Nup153|218210	-1.069197052	0.413519851	1.239258237	0.155178153
Rhbdd3|279766	-1.069206341	0.369456299	-1.3800991	0.004067878
Hus1|15574	-1.069284111	0.444229451	-1.277145337	0.289011056
Aptx|66408	-1.069408376	0.342235565	-1.069408376	0.342235565
2210404J11Rik|381062	-1.069515738	0.412985012	1.137710023	0.33080503
Eef2k|13631	-1.069630125	0.407147632	1.17689821	0.242881669
Aurka|20878	-1.069661502	0.418195082	-1.001078265	0.498569733
Cd3d|12500	-1.069723633	0.449672574	-1.069723633	0.449672574
Heyl|56198	-1.069736491	0.449166887	1.2299902	0.328866652
Usp1|230484	-1.069763463	0.41515579	-1.281592792	0.154280835
Mul1|68350	-1.06978764	0.214870311	-1.040563876	0.324283771
Plekhb1|27276	-1.069820167	0.346279771	-1.047965007	0.376325206
Hif1a|15251	-1.069883409	0.387251889	-1.214342149	0.213626066
Snx5|69178	-1.069946369	0.287375503	1.058512864	0.280521367
Pld1|18805	-1.070016823	0.454135391	-1.023441799	0.485499707
Ipo13|230673	-1.070076037	0.299359327	-1.031431748	0.411197242
Idua|15932	-1.070135813	0.293055399	-1.168638716	0.081334528
P2rx7|18439	-1.070146537	0.361090496	1.026684817	0.445234834
Oat|18242	-1.070150561	0.408753381	-1.070150561	0.408753381
Ipo4|75751	-1.070211095	0.243684562	-1.128333958	0.085720583
Arhgap24|231532	-1.070242319	0.375988608	-1.207147199	0.195178618
Thop1|50492	-1.070307035	0.336435059	-1.056934421	0.235382307
Sh3bp5|24056	-1.070315124	0.389550941	1.217292428	0.154308124
Ipo9|226432	-1.070343877	0.260972246	1.067081456	0.097512646
Slc34a2|20531	-1.070479304	0.437276061	-1.258499518	0.282973751
Dolk|227697	-1.070492589	0.321629264	-1.070492589	0.321629264
Nrbp2|223649	-1.070656884	0.092031283	-1.07090979	0.058886369
Pfkl|18641	-1.070671919	0.338508403	-1.045341054	0.353295735
Capg|12332	-1.070702973	0.432314237	-1.158008145	0.261588295
Gimap1|16205	-1.070727323	0.356390946	-1.070727323	0.356390946
Ppp1r35|69871	-1.070736722	0.407035879	-1.070736722	0.407035879
Gfap|14580	-1.070767805	0.464464284	-1.966634453	0.201402532
Mrps22|64655	-1.070768162	0.308407956	-1.157235043	0.10691948
4933424B01Rik|71177	-1.070861612	0.357859898	1.06726755	0.305824504
0610011L14Rik|68295	-1.070908569	0.258487582	-1.020705782	0.422321341
Men1|17283	-1.07105803	0.259868678	-1.135661727	0.115372313
Nadk|192185	-1.071060925	0.26384902	1.059573717	0.204320471
D230025D16Rik|234678	-1.071110928	0.326942765	-1.053466004	0.267358367
Nfkbia|18035	-1.071142096	0.330845616	1.067908331	0.250667111
Fancf|100040608	-1.071236149	0.421826896	-1.071236149	0.421826896
Ctsa|19025	-1.071422396	0.231979228	-1.116232623	0.132160756
Tmem50a|71817	-1.071448513	0.365667219	-1.173702477	0.104569132

H47|109815	-1.071519007	0.290247638	1.012210634	0.454701924
Sart3|53890	-1.071663365	0.335340436	-1.099379195	0.2278106
Ubr3|68795	-1.07167738	0.36027137	-1.012054653	0.469240704
Xpc|22591	-1.071694229	0.355797179	-1.148989055	0.191743763
Rnmtl1|67390	-1.071735546	0.344399322	-1.038174189	0.396506134
Fmn1|14260	-1.071806008	0.438059497	1.23113892	0.326990727
Mrpl30|107734	-1.071936211	0.32686119	1.000159748	0.49956405
Rdbp|27632	-1.071954157	0.319182732	-1.002544677	0.491629201
Whsc2|24116	-1.071975869	0.383127873	-1.174626247	0.237686673
Slc38a6|625098	-1.072056396	0.346659963	-1.072056396	0.346659963
Armc5|233912	-1.072086785	0.283362971	-1.162605277	0.09044619
Pi4kb|107650	-1.072114623	0.301518441	-1.165496969	0.077669614
Pvrl1|58235	-1.072200418	0.088887211	-1.053595828	0.043130415
9430016H08Rik|68115	-1.072336184	0.261073188	-1.136322486	0.098060083
Tmem17|103765	-1.072359947	0.443023209	1.108702251	0.410869475
Ckb|12709	-1.072392384	0.423943646	-1.378368507	0.122809445
Dos|100503659	-1.072411819	0.371843224	-1.072411819	0.371843224
Ncbp2|68092	-1.072420064	0.304302146	-1.126241492	0.194179212
Hnrnpf|98758	-1.072472858	0.30427419	1.082512591	0.218278421
Kcnc3|16504	-1.072474512	0.259229504	-1.114857114	0.162160689
D1Bwg0212e|52846	-1.072514072	0.344590618	1.037711029	0.408803004
Ccdc55|237859	-1.072759062	0.330176404	-1.1622734	0.180908939
Zmat5|67178	-1.072775999	0.364320957	1.117751187	0.181283466
Pgpep1|66522	-1.072776129	0.187323926	1.021786559	0.329770698
Ltbr|17000	-1.072798046	0.29639878	-1.15879397	0.092083382
Lzts2|226154	-1.072816865	0.311144282	1.003604736	0.488785148
Zyg11b|414872	-1.072820575	0.311126664	-1.081790852	0.134942509
Nt5m|103850	-1.072820846	0.397216453	-1.244295568	0.218869107
Unc45a|101869	-1.073139172	0.289433561	-1.164131581	0.103651189
Fbxl20|72194	-1.073266827	0.249300998	-1.002667691	0.487816217
Gnb2|14693	-1.07331654	0.33340704	-1.032138516	0.405175639
Slc22a7|108114	-1.07338744	0.346392806	-1.125549797	0.210983225
9130401M01Rik|75758	-1.073409661	0.334265887	-1.294959474	0.028656031
Elovl2|54326	-1.073541075	0.355866638	1.047039465	0.398193915
Xylt2|217119	-1.073600011	0.225787006	-1.002931668	0.483386471
Ears2|67417	-1.073605286	0.357305007	-1.073605286	0.357305007
9430038I01Rik|77252	-1.073702973	0.397798807	-1.167552185	0.277547313
Nfib|18028	-1.073712417	0.268924038	1.010957719	0.451832202
Fam160b1|226252	-1.073780173	0.339296542	-1.073780173	0.339296542
Ppp2r5b|225849	-1.073852642	0.314550585	-1.132170018	0.153941477
Rwdd1|66521	-1.073886819	0.346638406	-1.073886819	0.346638406
Cdk5rap3|80280	-1.073890587	0.361971363	-1.005689177	0.488881543
Pmm2|54128	-1.073898202	0.25418568	-1.121159668	0.158658297
Ncln|103425	-1.073939278	0.27034551	-1.073939278	0.27034551
Mrps16|66242	-1.073942214	0.320167585	-1.073942214	0.320167585
Ctdsp1|227292	-1.073969233	0.279795655	-1.168500107	0.081222754
Anxa6|11749	-1.074007974	0.256837282	-1.148099419	0.094556374
Prkci|18759	-1.074023322	0.3942362	-1.105472011	0.334718389
Zmpste24|230709	-1.074137135	0.209113599	1.019212974	0.39313549
Hnrnpa3|229279	-1.074152428	0.214212912	-1.113504105	0.117771148
Maged1|94275	-1.074169127	0.227778592	-1.074169127	0.227778592
Mettl7b|71664	-1.074198026	0.361252558	-1.091766801	0.258144319
P2rx4|18438	-1.074259586	0.309116744	-1.225797926	0.019986468

Pigb|55981	-1.074302537	0.41254541	-1.14405374	0.33413305
Sf3b4|107701	-1.074309649	0.304693647	-1.074309649	0.304693647
Fbxo36|66153	-1.074397168	0.330464673	-1.303557346	0.017972039
Nipa2|93790	-1.074487533	0.404424868	-1.037170215	0.432670478
Ehd3|57440	-1.074504888	0.381948357	-1.011636037	0.47153785
Cpb2|56373	-1.07451869	0.260605779	-1.128039516	0.134894151
Gnmt|14711	-1.074661962	0.367884039	-1.390850566	0.019416365
Tmem110|69179	-1.074664568	0.333715984	-1.017151457	0.460054107
A530032D15Rik|381287	-1.07470019	0.467233076	-3.88109794	0.096090833
Tmod3|50875	-1.074863521	0.343036741	-1.074863521	0.343036741
Ppat|231327	-1.074871466	0.3506876	1.108137789	0.146520116
Pik3cd|18707	-1.075020049	0.354236505	-1.267554856	0.063734645
4931408A02Rik|70967	-1.075021836	0.444292296	1.047065153	0.467580986
Slc2a8|56017	-1.075137572	0.294275193	-1.046374989	0.378357553
Kdm4b|193796	-1.075262239	0.21326589	-1.10628405	0.144542057
Tpi1|21991	-1.075262335	0.235811913	-1.189216772	0.0307713
Npnt|114249	-1.075308235	0.451688564	1.018517157	0.482800486
Dpp4|13482	-1.075332099	0.255499484	-1.016257403	0.436098262
Tshz3|243931	-1.075374215	0.463807336	1.002806537	0.498817668
Fam53c|66306	-1.075432107	0.417350468	1.073112027	0.391637025
0610010K14Rik|104457	-1.075495003	0.304588651	-1.021223416	0.419761835
Alg3|208624	-1.075566887	0.311527532	-1.075566887	0.311527532
Pdzk1|59020	-1.07563969	0.242171782	-1.07563969	0.242171782
Sh3d19|27059	-1.07565622	0.335061246	1.14706076	0.08959885
Rassf7|66985	-1.075685473	0.242989291	-1.138115961	0.085436491
Sfxn1|14057	-1.07582686	0.061171461	-1.063104089	0.052183607
Srp68|217337	-1.075856505	0.251587007	1.057124888	0.20527949
Obfc2b|69917	-1.075926987	0.264948379	-1.075926987	0.264948379
Dhx30|72831	-1.075957797	0.29741249	1.006147296	0.48073829
Nudt5|53893	-1.075978101	0.377403257	-1.298705158	0.101586647
Fads6|328035	-1.075993101	0.3618539	1.070032899	0.354661388
Gpatch1|67471	-1.076064451	0.28358863	-1.076064451	0.28358863
Noc3l|57753	-1.076155021	0.267952247	-1.011280754	0.460291722
Tceb2|67673	-1.076174741	0.356889651	-1.076174741	0.356889651
Ikbkap|230233	-1.076222306	0.313333397	-1.197000597	0.06901806
Pex11a|18631	-1.076230541	0.39120832	1.251277539	0.136283106
Mettl1|17299	-1.076235652	0.365885386	-1.002284015	0.495765625
Arl5a|75423	-1.076305337	0.200528437	-1.039578723	0.326086671
BC027231|212547	-1.076306935	0.379254508	-1.243793833	0.118377047
Fam120a|218236	-1.076329545	0.352727332	1.004034222	0.490032508
Rnf185|193670	-1.076365238	0.212364119	-1.076365238	0.212364119
Arcn1|213827	-1.076394853	0.3514437	1.060013328	0.363400894
Tgtp2|100039796	-1.076556606	0.396678631	-1.346630785	0.071744788
Keap1|50868	-1.076581079	0.133830308	-1.110677681	0.063068398
Bcas3|192197	-1.076649425	0.228852009	-1.076649425	0.228852009
Ctss|13040	-1.076670508	0.327782812	-1.036772897	0.420916658
Nbeal2|235627	-1.076676305	0.400581657	1.136814873	0.241989793
1520402A15Rik|68075	-1.076716048	0.469823981	-2.8202	0.173296754
Fam54b|76824	-1.076735089	0.131643089	-1.076735089	0.131643089
B4galt2|53418	-1.076794679	0.464802687	1.061455911	0.478986093
Enoph1|67870	-1.076840469	0.410256856	-1.18256212	0.284769934
0610037L13Rik|74098	-1.077004642	0.324728725	-1.077004642	0.324728725
Tmco3|234076	-1.077055877	0.406887396	1.038649784	0.450730076

BC021614|225884	-1.077106617	0.404846728	-1.035823911	0.444066115
Lifr|16880	-1.077126748	0.296219978	-1.026801906	0.424957986
Anapc11|66156	-1.077195473	0.381214496	-1.327114127	0.086303052
Slc20a2|20516	-1.077218796	0.32433539	1.010511553	0.473778155
Plvap|84094	-1.077235529	0.207167539	-1.066267157	0.168339533
Lxn|17035	-1.077293126	0.458081413	1.00700148	0.495959403
Pitrm1|69617	-1.077324157	0.354069346	1.031601207	0.436775995
Ube4a|140630	-1.077383216	0.314720787	1.050729494	0.322355172
Man2a2|140481	-1.077433392	0.384582934	1.029061385	0.405851387
Dcp1b|319618	-1.077445128	0.410495508	-1.285653294	0.140427904
Chmp5|76959	-1.077524658	0.315810284	-1.072020196	0.318834605
Rad23a|19358	-1.077557104	0.280822296	1.000330678	0.498874317
Csk|12988	-1.07760483	0.163289078	-1.13626853	0.03069316
Iigp1|60440	-1.077715858	0.261237535	1.049233047	0.300063291
Lrfn3|233067	-1.077719107	0.334286738	1.008665765	0.478617363
Klf15|66277	-1.07773313	0.255938258	-1.07773313	0.255938258
Ppp4r4|74521	-1.077772954	0.439686431	1.325653603	0.28688103
Epb4.1l1|13821	-1.07778595	0.386083206	-1.07778595	0.386083206
Sap30|60406	-1.07781735	0.391181185	1.061267229	0.401904008
Mre11a|17535	-1.077871286	0.368115783	1.047285771	0.416506358
Snapc4|227644	-1.077919369	0.273193189	-1.015583958	0.449569079
Zfp27|22689	-1.077921014	0.378078112	-1.194699618	0.216298054
Kdelr1|68137	-1.077922546	0.212187487	-1.039991179	0.336849749
Bin3|57784	-1.077945402	0.260107415	-1.222399128	0.019846921
L3mbtl2|214669	-1.078140631	0.318912601	-1.297473476	0.01850696
Josd1|74158	-1.078246342	0.235421696	-1.027570672	0.391279987
Stx5a|56389	-1.078265	0.327788746	-1.165716105	0.193984335
Hip1r|29816	-1.078278069	0.344741354	-1.289362392	0.025224556
Pak4|70584	-1.078299624	0.252316051	-1.227544102	0.00400343
Slc25a25|227731	-1.078304181	0.382859089	-1.199885076	0.221136987
Pnpo|103711	-1.078306433	0.171828065	-1.032314309	0.329247283
Mreg|381269	-1.078342442	0.339108866	-1.036555666	0.425878506
Ugcg|22234	-1.078360574	0.367109003	1.070851617	0.367722559
Impact|16210	-1.078363515	0.399032861	-1.231133776	0.258234939
Rgp1|242406	-1.078472323	0.243933935	1.042987823	0.29870294
4930481A15Rik|74931	-1.078490223	0.295212611	-1.092924296	0.284806255
BC017643|217370	-1.078606412	0.344386887	-1.199053714	0.126982793
Nfrkb|235134	-1.078638007	0.349609236	1.050926761	0.370546784
Vps26b|69091	-1.078649318	0.337370725	-1.069081771	0.283194656
Sox17|20671	-1.078827434	0.453959937	-2.134697578	0.13622783
Acsf3|257633	-1.078899217	0.208213134	-1.146673231	0.033644698
Slc31a1|20529	-1.078908128	0.2336845	-1.078908128	0.2336845
Rbbp5|213464	-1.078921341	0.385493784	1.066334202	0.403206455
Usp24|329908	-1.078995881	0.11970591	-1.078995881	0.11970591
Dock1|330662	-1.079016041	0.405760476	1.017465012	0.461313034
Aif1|11629	-1.079030184	0.427897771	-1.709579765	0.042093515
D5Ertd579e|320661	-1.079068685	0.358907459	-1.079068685	0.358907459
Ier3ip1|66191	-1.079104909	0.30542353	-1.003893265	0.489528117
Mtap|66902	-1.079229919	0.298619447	1.086487072	0.214236755
Kng2|385643	-1.079235988	0.37117417	-1.456790262	0.006226903
Hnrnph1|59013	-1.079238591	0.390485402	1.042526509	0.435265857
Dnlz|52838	-1.079295648	0.239694476	1.000661652	0.496539698
C630004H02Rik|217310	-1.079307596	0.422876931	-1.59995082	0.071262155

Cndp2|66054	-1.079374281	0.237648623	-1.090657694	0.085855277
Tmem106b|71900	-1.079382048	0.342729046	1.164703589	0.083959654
Rapgefl1|268480	-1.079383975	0.433925966	-1.925811615	0.040327091
Tnfrsf1a|21937	-1.079401717	0.221411073	-1.197211657	0.022909174
Spcs3|76687	-1.079460063	0.30236896	-1.193969217	0.098087223
Plekhf1|72287	-1.079542942	0.408734213	-1.443427494	0.104461532
Proca1|216974	-1.079554237	0.419390298	1.281115753	0.211157723
Tspan8|216350	-1.079558923	0.434510776	1.061079825	0.455192656
Hist1h2ah|319168	-1.079572729	0.410650695	1.088250046	0.407134578
Prkaca|18747	-1.07958816	0.193909297	-1.084802338	0.134883582
Pllp|67801	-1.079704481	0.191521059	-1.01895868	0.39545319
Herc1|235439	-1.079861873	0.394998328	1.037204428	0.426966906
Sec61a1|53421	-1.079863312	0.303173596	-1.16946608	0.150683586
Opa3|403187	-1.079917809	0.335618791	1.033108785	0.422719031
Rtn4ip1|170728	-1.079922956	0.290674537	1.103758939	0.08237023
Apoa5|66113	-1.080011613	0.312203401	-1.080011613	0.312203401
Rcn3|52377	-1.080015788	0.402014545	-1.161703935	0.23093227
Arhgdia|192662	-1.080021199	0.23516021	-1.04477927	0.344327317
Ddx49|234374	-1.080053083	0.271884167	1.002110803	0.492404854
Sfr1|67788	-1.08014532	0.337791765	1.011133891	0.476167842
Trappc5|66682	-1.080164276	0.251999206	-1.130596339	0.158880924
Plekha5|109135	-1.080197781	0.383299681	1.244735028	0.128307553
Opa1|74143	-1.080234836	0.342362791	1.028689475	0.388749496
Trmu|72026	-1.080249697	0.382670114	-1.296296254	0.14841193
Gtf2i|14886	-1.080312466	0.144394281	-1.093468761	0.01669769
Ctxn1|330695	-1.080334666	0.452982175	-1.057297582	0.464839941
Arap3|106952	-1.080345476	0.242923374	1.032996933	0.338589743
Catsper2|212670	-1.080388057	0.381686995	1.06082889	0.394139241
Zbtb2|381990	-1.080509791	0.334915806	1.119586293	0.212997045
Snx13|217463	-1.08059149	0.348631714	-1.032676481	0.419079888
Nubp2|26426	-1.080625296	0.153939083	-1.080625296	0.153939083
Atp5b|11947	-1.08067395	0.112192705	-1.157007959	0.005354957
Ptplb|70757	-1.080793063	0.296360519	-1.186865353	0.108126063
Ptprg|19270	-1.080816625	0.358222633	1.049784662	0.405482057
Fam136a|66488	-1.08086386	0.343627805	1.159439515	0.129015551
Cnnm3|94218	-1.080872645	0.262321885	-1.157360878	0.115246202
Ccdc23|69216	-1.08102341	0.337473835	-1.238564395	0.102528924
Ccdc59|52713	-1.081056815	0.285304708	1.008253918	0.470186275
Ube2o|217342	-1.08110676	0.298481672	-1.08110676	0.298481672
Akr1b7|11997	-1.081175266	0.435998827	-1.088491797	0.418275187
Zfp839|72805	-1.081216156	0.227337291	-1.01654246	0.429601837
Slc12a4|20498	-1.08125218	0.286868238	-1.25731316	0.023593623
Fam134c|67998	-1.081286116	0.131636089	-1.081286116	0.131636089
Mpdu1|24070	-1.081428571	0.279221797	-1.081428571	0.279221797
Gtf2ird1|57080	-1.081437968	0.310089511	-1.081437968	0.310089511
Il4ra|16190	-1.081448358	0.175413825	-1.137583358	0.036734623
Snrnp70|20637	-1.081475774	0.307741207	-1.081475774	0.307741207
Kidins220|77480	-1.081486917	0.3189613	-1.081486917	0.3189613
Actr2|66713	-1.081545369	0.291437858	1.029242676	0.393703466
Fto|26383	-1.081561723	0.282041891	-1.081561723	0.282041891
Tm4sf1|17112	-1.081649464	0.354522266	-1.190489237	0.185110031
Adprh|11544	-1.081729581	0.319476212	1.114023431	0.189244749
Chrac1|93696	-1.081782625	0.234389774	-1.081782625	0.234389774

Bloc1s3|232946	-1.081803438	0.360362573	-1.081803438	0.360362573
Scarb1|20778	-1.081808186	0.214546862	-1.041424329	0.279948927
Adora1|11539	-1.081836873	0.305501929	-1.072675431	0.297341105
Tfpt|69714	-1.081945515	0.311775	-1.281023153	0.026840731
Zfp764|233893	-1.082071253	0.394007973	-1.104343674	0.274690718
Trim2|80890	-1.082087525	0.414736511	1.178691013	0.322955699
Pole|18973	-1.08223087	0.416755144	1.112824819	0.394912474
Epha1|13835	-1.08227193	0.244225207	-1.218651524	0.027796728
Deb1|26901	-1.082313163	0.332191534	-1.082313163	0.332191534
Ppm1g|14208	-1.082328293	0.238223294	-1.13046363	0.150180518
2310035C23Rik|227446	-1.082369743	0.368581718	-1.207726632	0.226733592
2810021J22Rik|69944	-1.082411263	0.42965291	-1.082411263	0.42965291
Rhou|69581	-1.082433266	0.307155779	-1.178649234	0.151526004
Cdc42ep2|104252	-1.082436828	0.372110266	-1.284467102	0.140215507
BC003331|226499	-1.082464202	0.27608055	-1.02779826	0.420897708
Tnip2|231130	-1.082465771	0.399206314	1.063616692	0.42563155
Atg5|11793	-1.082482458	0.174453175	-1.082482458	0.174453175
Fibp|58249	-1.082516644	0.316739913	-1.028296085	0.394953781
Apobr|171504	-1.082557087	0.319235835	1.101074049	0.231085602
Dqx1|93838	-1.082624009	0.226431739	-1.170327863	0.044310974
Uba7|74153	-1.082724286	0.31452788	-1.017259873	0.460368584
Ccdc104|216618	-1.082725521	0.357076464	1.056403885	0.373360914
Uba5|66663	-1.082736398	0.33501745	-1.166413282	0.198004502
Srek1|218543	-1.082747524	0.287008161	-1.082747524	0.287008161
Kcnj8|16523	-1.082767932	0.403148988	1.060238463	0.433359256
Sec13|110379	-1.082820985	0.241021835	1.045809896	0.2897558
Pura|19290	-1.08290501	0.332704286	-1.196813996	0.170012668
Mir1895|100316832	-1.083012072	0.449045492	1.745770261	0.163534591
Trim7|94089	-1.083048609	0.335654064	-1.172513873	0.18806829
Nme6|54369	-1.083057891	0.368766594	1.19183052	0.180471439
Mrpl52|68836	-1.083060948	0.383160493	-1.348574254	0.105058156
Whamm|434204	-1.083178198	0.230677995	-1.222741663	0.012977541
Srprb|20818	-1.083258719	0.327028332	1.036037467	0.411261452
Acot9|56360	-1.083278059	0.450777208	1.601926307	0.243606372
Adh5|11532	-1.083285215	0.179102723	-1.133204803	0.061065396
1300001I01Rik|74148	-1.083332645	0.052145545	-1.089407727	0.012780007
Stbd1|52331	-1.083382194	0.220469989	-1.1470298	0.069924
Gtf2h5|66467	-1.083545079	0.266314463	-1.219045199	0.03035603
Bola3|78653	-1.083549292	0.18007787	-1.124453118	0.101892674
Fam83f|213956	-1.083588804	0.435909268	-1.083588804	0.435909268
Rbm41|237073	-1.083652867	0.450503588	-1.197779736	0.394899452
Gm1821|218963	-1.083686007	0.292803196	-1.175748048	0.102917726
Itih1|16424	-1.083877361	0.263695414	-1.097978922	0.185184352
Camta1|100072	-1.083886854	0.392381992	-1.358832471	0.134854897
Pdhb|68263	-1.083933845	0.291113754	1.104164994	0.094555023
Vamp2|22318	-1.083967595	0.26489602	-1.11456947	0.111143879
Fcho2|218503	-1.084115578	0.423320278	1.338966517	0.197701065
Preb|50907	-1.084174556	0.078885169	-1.084174556	0.078885169
Zfp503|218820	-1.084195784	0.447004179	-1.991464949	0.134641892
Ddx47|67755	-1.084257279	0.135073636	-1.112401701	0.080222298
Rasa2|114713	-1.084294821	0.407017783	-1.084294821	0.407017783
Gch1|14528	-1.084381598	0.232795913	-1.020434301	0.421792368
Csrp2bp|228714	-1.084454511	0.263565095	-1.084454511	0.263565095

Sephs1|109079	-1.084456441	0.158849342	-1.152504107	0.020460655
Phrf1|101471	-1.08449141	0.265414822	1.011030541	0.452081628
Tmem56|99887	-1.084505063	0.373005409	1.138731381	0.237367924
Rmnd5b|66089	-1.084569879	0.252222632	-1.169944839	0.089828203
BC048403|270802	-1.08469983	0.436099976	1.489283152	0.177167238
Parp9|80285	-1.08473127	0.273677356	-1.024956451	0.428776385
Dapk3|13144	-1.084756603	0.306511754	-1.169305231	0.148947195
Glyr1|74022	-1.084812155	0.236614072	-1.007997556	0.451422224
Ewsr1|14030	-1.084825067	0.132905033	-1.11891253	0.072092657
Prkacb|18749	-1.084827427	0.325659974	-1.169841329	0.207834444
Dazap2|23994	-1.084872373	0.293396031	1.018240577	0.443481693
2900005J15Rik|67261	-1.08490679	0.343720207	-1.044721593	0.419680809
Ephb4|13846	-1.084923381	0.199467793	-1.011793913	0.430796068
Phlda1|21664	-1.084929023	0.343225075	-1.084929023	0.343225075
Minpp1|17330	-1.084930368	0.151880156	-1.084930368	0.151880156
Ctnnal1|54366	-1.084939991	0.421916064	-1.084939991	0.421916064
Prrc1|73137	-1.084962347	0.165702656	-1.022271256	0.356396171
Tlr1|21897	-1.084984844	0.446254984	-1.575659799	0.257144871
Cxcl11|56066	-1.08508646	0.45846401	2.218072465	0.163883125
Cd99l2|171486	-1.08518152	0.310417939	-1.178829942	0.135829469
Gstm4|14865	-1.085223229	0.229685383	-1.149009685	0.08536234
F630110N24Rik|73822	-1.085230986	0.438052971	-1.085230986	0.438052971
Ypel5|383295	-1.085261955	0.270315683	-1.033142026	0.337832858
Mapk1ip1l|218975	-1.085344796	0.394474521	1.118338927	0.279018681
Lrrc32|434215	-1.085347249	0.265143261	-1.009398839	0.46707395
Cyc1|66445	-1.085364414	0.114646803	-1.085364414	0.114646803
Asb6|72323	-1.085408166	0.247556868	-1.085408166	0.247556868
Cep152|99100	-1.085470851	0.373694903	1.102314841	0.281562964
Pias1|56469	-1.085505731	0.29643824	1.081212403	0.24670558
Svip|75744	-1.085550301	0.369110802	-1.318367062	0.104776133
Txndc17|52700	-1.085559295	0.289331669	-1.274241694	0.028370514
Pi4ka|224020	-1.085584619	0.227862709	-1.085584619	0.227862709
Fbxo25|66822	-1.085587301	0.37376688	-1.000880293	0.498735624
Arl8b|67166	-1.085649848	0.22037192	-1.085649848	0.22037192
Arpc2|76709	-1.085687163	0.030023326	-1.0693836	0.06199197
Hnrnpul2|68693	-1.085744366	0.229727102	1.049813221	0.224967262
Fhl1|14199	-1.085833606	0.416157335	1.472526368	0.083750399
Exoc3|211446	-1.085844808	0.279477235	-1.184860556	0.06742039
Prpf31|68988	-1.085953064	0.323633728	1.015764287	0.460092106
Znrf1|170737	-1.085984176	0.314761665	-1.33209959	0.012117663
Uxs1|67883	-1.085989979	0.38148257	-1.148189209	0.254108995
Tle4|21888	-1.086012985	0.370118307	1.228425618	0.123050274
Paip2b|232164	-1.086033414	0.3405637	-1.204415702	0.146123041
Gkap1|56278	-1.086036979	0.35346107	-1.086036979	0.35346107
Nsmce2|68501	-1.086049779	0.30282502	-1.22111177	0.08231741
Prune|229589	-1.086097578	0.300651802	-1.055194194	0.316081133
Tmf1|232286	-1.086129615	0.399407985	-1.182763222	0.261217555
Ptpn3|545622	-1.086165521	0.321003893	-1.086165521	0.321003893
Kars|85305	-1.086240018	0.186258742	-1.0386039	0.334742608
Zfp444|72667	-1.086248428	0.290327814	-1.283897058	0.019025622
Cyth1|19157	-1.086397257	0.28153937	1.060536992	0.290366742
Araf|11836	-1.086413363	0.15407751	-1.190514464	0.003472365
Adsl|11564	-1.086447753	0.144013907	-1.083746999	0.094645845

Tasp1|75812	-1.086497609	0.391835722	-1.372109021	0.133507331
Pycard|66824	-1.086497672	0.438748222	1.015457402	0.488093642
Ergic2|67456	-1.086642169	0.282590793	-1.006920402	0.478572678
Gmpr2|105446	-1.08664651	0.193983541	-1.025669252	0.380648132
Fam158a|85308	-1.086649258	0.345193916	-1.292063638	0.072430481
Zglp1|100009600	-1.086832941	0.468696171	1	#DIV/0!
Guf1|231279	-1.086834329	0.33273743	-1.011086151	0.476773286
Smpd1|20597	-1.086929253	0.161551797	-1.135236161	0.069127567
Stx2|13852	-1.087028033	0.306254724	1.029320423	0.417300116
D6Wsu163e|28040	-1.087068119	0.369897258	-1.087068119	0.369897258
C030016D13Rik|107372	-1.087118788	0.417726844	-1.75881774	0.042552402
Apbb3|225372	-1.08723485	0.221131115	-1.217420482	0.020338848
A930005H10Rik|68161	-1.087283917	0.372983639	-1.156891514	0.259513243
Sorbs1|20411	-1.087324734	0.411348615	1.24859331	0.244048466
0610031J06Rik|56700	-1.087359687	0.284193158	-1.15169209	0.184808728
Akt1|11651	-1.087386414	0.11910144	-1.161445778	0.010528794
Smek2|104570	-1.087455141	0.387618942	1.09625975	0.276140048
Cd302|66205	-1.087458633	0.192977743	-1.087458633	0.192977743
Fbxo9|71538	-1.087496579	0.259798827	-1.011520861	0.462215561
Neurl4|216860	-1.087506081	0.308489579	1.057230911	0.305971931
Anubl1|67492	-1.087669351	0.392120403	-1.087669351	0.392120403
Kng1|16644	-1.087710007	0.35613218	-1.448464099	0.010150592
Baat|12012	-1.08773898	0.142946955	-1.043137013	0.27352011
Sh2d7|244885	-1.087857581	0.461706469	2.424107143	0.19229339
Ifitm1|68713	-1.087860872	0.328998266	1.047047472	0.368143437
Dclre1b|140917	-1.08797613	0.38946964	-1.375978809	0.128248225
Mapk3|26417	-1.088015332	0.183264806	-1.147285563	0.068520395
Mettl14|210529	-1.088051716	0.342169159	-1.061562583	0.395401803
Zc3h10|103284	-1.088260227	0.297562999	1.01436562	0.455399455
Dcun1d1|114893	-1.088264751	0.1854887	-1.147554818	0.050856396
Eif2b5|224045	-1.08827428	0.252473617	-1.151905788	0.144222976
Arrdc4|66412	-1.088282409	0.373861955	1.091434061	0.359547135
Cars2|71941	-1.088309572	0.351945996	1.018479715	0.464409947
Mrps34|79044	-1.088365087	0.259180613	-1.176158447	0.070208407
5033414D02Rik|67759	-1.08837642	0.414859335	-1.303598878	0.183686181
Mterfd1|66410	-1.088440133	0.329825119	-1.088440133	0.329825119
Txlna|109658	-1.088456983	0.274569023	1.016169639	0.435606004
Arhgef18|102098	-1.08852837	0.256231137	-1.101348962	0.154583241
Deaf1|54006	-1.088529414	0.234222519	-1.043177809	0.298771594
Myo1c|17913	-1.088679507	0.133195249	-1.097094337	0.082760577
St13|70356	-1.088739326	0.273745727	1.068015592	0.269695574
Phf23|78246	-1.088820264	0.159699474	-1.04234464	0.301056981
Ammecr1l|225339	-1.088846825	0.410267738	1.409293009	0.125331171
Ezr|22350	-1.088862113	0.411103017	-1.086553536	0.406596766
Top2b|21974	-1.088928325	0.290480654	-1.088928325	0.290480654
Krt19|16669	-1.088939484	0.409147811	1.172402411	0.330887778
Psmf1|228769	-1.088946745	0.21813742	-1.155742565	0.094111877
Lrrc8d|231549	-1.088958774	0.192645442	-1.061081291	0.281435854
Itga7|16404	-1.089090883	0.346045465	-1.089090883	0.346045465
Ugt2b37|112417	-1.089124367	0.411000428	-1.089124367	0.411000428
Mrvi1|17540	-1.089138603	0.384003852	1.108520218	0.352721789
Zfp949|71640	-1.089178709	0.285290701	-1.199662836	0.104221464
Gnaq|14682	-1.08919228	0.36802057	1.121331855	0.283883151

Ttll13|269954	-1.089264068	0.421949335	-1.296188149	0.254837692
Med8|80509	-1.089318344	0.269933967	-1.266139371	0.02693089
Foxk2|68837	-1.089370861	0.280450645	-1.184313804	0.125682973
Ubr7|66622	-1.089500106	0.348455593	1.031431811	0.443408333
Tsr2|69499	-1.089507469	0.296264587	-1.237901671	0.057907477
Kif13a|16553	-1.089553489	0.386292461	-1.274025049	0.094083993
4933434E20Rik|99650	-1.089610951	0.195319734	-1.101060201	0.110197872
Flad1|319945	-1.08968682	0.195893948	-1.044311046	0.327110052
Prnp|19122	-1.089775284	0.145367693	-1.158140676	0.018066723
Pan3|72587	-1.089786662	0.347975159	1.195173259	0.091609929
Ppm1a|19042	-1.089800984	0.24092335	-1.147483142	0.125139412
Rlim|19820	-1.089805126	0.270820239	-1.293171996	0.005419948
Sec61a2|57743	-1.089925943	0.369317701	-1.375636541	0.063260593
Zdhhc6|66980	-1.089940985	0.193122717	-1.141797297	0.082018614
Fip1l1|66899	-1.089961075	0.231657182	-1.028485256	0.401885995
Pcyt2|68671	-1.089965654	0.218776286	-1.161239562	0.087131817
Slc17a5|235504	-1.089991928	0.315921951	-1.334791133	0.025223938
Zfp952|240067	-1.090004078	0.366422267	1.09799833	0.285488131
Dusp2|13537	-1.090035563	0.421260295	-1.264329518	0.278376604
Stx4a|20909	-1.090041055	0.245478977	-1.090041055	0.245478977
Pex12|103737	-1.090052302	0.196317024	-1.229122973	0.003532992
Timp3|21859	-1.090103027	0.426073286	1.24926645	0.319369515
Pdcd2|18567	-1.090129217	0.328177002	1.009095856	0.479439197
Mfsd2a|76574	-1.090134125	0.340591478	-1.393339197	0.024190147
Slc25a44|229517	-1.09019543	0.176713069	-1.029088172	0.352201506
Fgd6|13998	-1.090238078	0.354661995	1.065709437	0.381643965
Man1a|17155	-1.090239815	0.364546343	-1.040284729	0.414451972
Adcy5|224129	-1.090319811	0.430873993	1.40854175	0.225429072
Copg|54161	-1.090364984	0.211682862	-1.090364984	0.211682862
Prps1|19139	-1.090468792	0.301465203	-1.090468792	0.301465203
Plk1s1|228730	-1.090500362	0.308541112	-1.010395723	0.452474194
Itih5|209378	-1.090501135	0.40866678	1.278418122	0.201976736
Grina|66168	-1.090566971	0.213503514	-1.090566971	0.213503514
Blvrb|233016	-1.090624578	0.321453424	1.067289331	0.309925482
Maf|17132	-1.090657102	0.401361348	1.081763805	0.332319548
Ptp4a2|19244	-1.090729689	0.100361951	-1.090729689	0.100361951
Efna1|13636	-1.0907425	0.338451601	1.028663841	0.444338704
Cyb5d2|192986	-1.090747453	0.113024193	-1.046843254	0.230237941
Mios|252875	-1.090761054	0.303504729	1.041323016	0.351744471
Hpse|15442	-1.090801166	0.370426379	-1.111146496	0.310200675
Ncam1|17967	-1.090818036	0.43733707	-1.218643176	0.374216389
Ndufs1|227197	-1.090825462	0.322257042	1.046442224	0.386316599
Phkg2|68961	-1.090852782	0.245850838	-1.15437017	0.097217564
Inca1|103844	-1.090887806	0.312683962	-1.022931483	0.433013789
Diap1|13367	-1.090907937	0.095460299	-1.132408949	0.019733066
Asap2|211914	-1.090917998	0.387131857	-1.090917998	0.387131857
Wdtc1|230796	-1.090955629	0.27354174	-1.183402027	0.123059562
Nek7|59125	-1.091311821	0.324560562	1.073614437	0.295146981
Hnrnph3|432467	-1.091352483	0.304469863	-1.350454006	0.006371872
Pde6d|18582	-1.091401132	0.242529477	-1.091401132	0.242529477
Tgfbi|21810	-1.091468714	0.255966211	-1.091468714	0.255966211
Bfar|67118	-1.091517694	0.254146186	-1.091517694	0.254146186
Fam65a|75687	-1.091571979	0.289502304	1.019913181	0.440516738

Pgs1|74451	-1.09157901	0.267743205	1.005881582	0.480328196
Lrriq3|74435	-1.091676	0.453698385	1.540949445	0.312650402
Helz|78455	-1.091695322	0.310826864	-1.022110922	0.408815873
Ctdnep1|67181	-1.091739445	0.184446818	-1.166313603	0.02009718
Stk32c|57740	-1.091805697	0.422108171	1.146619207	0.389158388
Nedd4|17999	-1.091847408	0.333272491	1.035651705	0.426767181
1700029I15Rik|75641	-1.091860638	0.435262381	-1.564840708	0.22849052
Arid5a|214855	-1.091893686	0.376311977	-1.168348157	0.25708342
Fance|72775	-1.092121124	0.30245738	-1.310338678	0.035863898
40970|224703	-1.092174619	0.172340818	-1.206987947	0.009919059
Mrs2|380836	-1.092202268	0.382825392	1.139326156	0.306545422
Mutyh|70603	-1.092281082	0.451060722	-2.164615657	0.174610843
Dph3|105638	-1.092297434	0.278649367	-1.287977997	0.018859941
Rtp3|235636	-1.092298715	0.242255328	-1.164742631	0.119638022
Derl1|67819	-1.092321422	0.135632562	-1.092321422	0.135632562
6030458C11Rik|77877	-1.092380008	0.277403277	1.004441874	0.485292988
Nr0b2|23957	-1.092439127	0.402071815	-1.413674854	0.16683703
Plxdc2|67448	-1.092484625	0.448641026	1.051959991	0.465263252
Ptch1|19206	-1.092505206	0.413907719	1.349235054	0.157020309
Naa10|56292	-1.092564676	0.335215813	-1.09119905	0.296794938
Ssbp4|76900	-1.092575818	0.351036559	-1.092575818	0.351036559
Slc22a18|18400	-1.092599543	0.151508686	-1.212692008	0.000232194
Smc6|67241	-1.092614991	0.162937275	-1.045131683	0.302177065
Pde4b|18578	-1.092701854	0.396137047	-1.091561373	0.371180448
Sigirr|24058	-1.092709901	0.373368946	-1.39762286	0.0705658
Dnaja3|83945	-1.092728148	0.17861014	1.027409622	0.290953015
Bbc3|170770	-1.092739075	0.291854932	-1.012173022	0.467814973
Mast4|328329	-1.092750314	0.386673247	-1.092750314	0.386673247
Ate1|11907	-1.092752562	0.396571961	-1.092752562	0.396571961
Rps4y2|66184	-1.092803527	0.343419835	1.159519574	0.195392738
Klf9|16601	-1.092813363	0.39753943	1.017886364	0.476625803
Arid1b|239985	-1.092887202	0.340058702	-1.034522724	0.393997248
AI987944|233168	-1.092920389	0.324478663	-1.092920389	0.324478663
Gm4987|245405	-1.092945806	0.419069587	1.317275296	0.237108226
Ddx24|27225	-1.092981118	0.113617039	-1.057089759	0.212158436
Numa1|101706	-1.093018386	0.200085688	-1.037072916	0.358833529
Tlr6|21899	-1.093037576	0.466232868	1	#DIV/0!
Elk3|13713	-1.093066077	0.296927485	1.008265031	0.478739678
Rragd|52187	-1.093157669	0.416297446	1.122900305	0.399786921
Hspa4|15525	-1.093213963	0.067061634	-1.158779103	0.002172534
Zfp106|20402	-1.09322275	0.225402532	-1.102670673	0.167466162
Oxsr1|108737	-1.093235154	0.357755761	1.12974844	0.232433695
Fxn|14297	-1.093327001	0.315121223	-1.250054722	0.095676225
Ppp6r1|243819	-1.093415546	0.101781818	-1.045834557	0.210588551
Apoc2|11813	-1.093520149	0.356657993	-1.294247406	0.136654619
Uqcrc2|67003	-1.093520638	0.147583241	-1.136321372	0.037834612
Fam129c|100037278	-1.093559414	0.448099225	-1.953378788	0.19862072
Fam19a3|329731	-1.093559414	0.448099225	-1.953378788	0.19862072
Cyp2c39|13098	-1.093618681	0.366085708	-1.093618681	0.366085708
Ppap2a|19012	-1.093635246	0.310559804	1.039179028	0.379751779
Ccnt2|72949	-1.093659537	0.372083159	1.151862293	0.243502198
2700097O09Rik|72658	-1.09373591	0.399386337	1.316926827	0.18668664
Sntg1|71096	-1.093752051	0.46721525	1	#DIV/0!

Rbm42|68035	-1.093861916	0.272604782	-1.206893242	0.044871553
Fez2|225020	-1.093927712	0.268039657	-1.286860296	0.019410829
Prrg3|208748	-1.093935354	0.408056982	-1.219666534	0.282936538
Etv6|14011	-1.093993122	0.26975359	-1.093993122	0.26975359
Nme3|79059	-1.094010756	0.276029694	-1.209731597	0.091826101
Mto1|68291	-1.094104833	0.336253603	-1.187554832	0.203311463
Taf6|21343	-1.094132494	0.187331637	-1.160131463	0.046057726
Brf1|72308	-1.094170541	0.286601785	-1.01496218	0.462810068
Snrnp35|76167	-1.094216575	0.302020317	1.021471451	0.44472463
Pak1|18479	-1.094223845	0.418354594	1.202353288	0.341082538
Nod1|107607	-1.09422856	0.33412252	-1.166502479	0.253280883
Tex261|21766	-1.094299877	0.142575705	-1.130031446	0.087330335
Copb2|50797	-1.094326265	0.174642402	-1.072372264	0.107801989
Agpat5|52123	-1.094345117	0.350625017	1.094083496	0.315819953
Gusb|110006	-1.094376279	0.263894052	-1.000975501	0.496886449
Nt5c3l|68106	-1.094378839	0.407859894	-1.094378839	0.407859894
Tead2|21677	-1.094401743	0.400902727	-1.094401743	0.400902727
Havcr2|171285	-1.094441607	0.427574072	1.088090611	0.427947013
Nprl2|56032	-1.094451706	0.301309018	1.117850748	0.164904585
Dom3z|112403	-1.094466548	0.318387425	-1.135770598	0.231026046
Dact3|629378	-1.09454477	0.356133728	1.090563272	0.290638326
Map2k1|26395	-1.094577571	0.339561722	1.021059813	0.45712538
Eif4g2|13690	-1.094628184	0.363292411	1.222068462	0.149354767
Tm9sf2|68059	-1.094642953	0.309836756	-1.215274758	0.144805459
1810020D17Rik|66273	-1.094810808	0.316048489	-1.094810808	0.316048489
Cdkn2aipnl|52626	-1.094965793	0.18739887	-1.179919482	0.033578918
Mlh3|217716	-1.094966534	0.321097237	1.149212272	0.139764886
Sh3rf1|59009	-1.094970556	0.41239332	-1.471459302	0.180932952
Usp9x|22284	-1.094999789	0.279973705	-1.201781251	0.116578121
Tnfrsf14|230979	-1.095014221	0.438357603	1.3199245	0.248658046
Qars|97541	-1.09502312	0.22557467	-1.09502312	0.22557467
Pts|19286	-1.095099863	0.20643971	-1.152384871	0.110469051
Gm14431|100303732	-1.095119951	0.451092207	-1.5378	0.167706141
Mrpl44|69163	-1.095183705	0.251372462	1.056528209	0.296444885
Fzd7|14369	-1.095345343	0.401297582	-1.451036311	0.14915249
Dhx29|218629	-1.095352184	0.37455238	1.135506144	0.296673174
Med19|381379	-1.095353952	0.282866141	-1.200605881	0.084323837
Zfp706|68036	-1.095364525	0.233239986	-1.256878651	0.017232289
Atp5f1|11950	-1.095431774	0.151904542	-1.178293856	0.009847096
Mfn2|170731	-1.095452047	0.303067506	1.123690837	0.157774541
Bcap31|27061	-1.095473958	0.030301007	-1.095473958	0.030301007
Pik3c3|225326	-1.095516647	0.237966874	-1.166825275	0.121836287
Plxna2|18845	-1.095528255	0.294621854	1.093925272	0.234044125
Rtp4|67775	-1.095646092	0.29163955	-1.175156899	0.157368486
Wdr44|72404	-1.095664796	0.451439637	-3.187161616	0.058355663
1810030O07Rik|69155	-1.095842768	0.333765015	-1.095842768	0.333765015
Vps13b|666173	-1.095844255	0.3919689	-1.314530709	0.222424674
Chmp3|66700	-1.095856699	0.295268814	-1.22472254	0.107935295
Adck3|67426	-1.095890085	0.170653974	-1.124135688	0.052554514
Fam50a|108160	-1.09594734	0.266816547	-1.174172527	0.122401503
Fam169a|320557	-1.095948477	0.453714834	-1.122857175	0.457104283
Ddx59|67997	-1.095960879	0.391967399	-1.095960879	0.391967399
Gatsl2|80909	-1.095981981	0.433934989	1.340895194	0.306899364

Rasgrp3|240168	-1.095988197	0.408421993	-1.07973984	0.42022956
Gm14420|628308	-1.096041291	0.26687518	-1.207481971	0.089674629
Itm2b|16432	-1.096098365	0.204179502	-1.128306437	0.082449503
Alg12|223774	-1.096147652	0.252421458	-1.096147652	0.252421458
Fbxw4|30838	-1.096202125	0.316105494	-1.342833692	0.041460051
Mzt2|72083	-1.096222448	0.252524184	-1.189565626	0.065529628
Cyp2d10|13101	-1.096235908	0.272721815	1.014482163	0.452954725
Wdr55|67936	-1.096390704	0.193608488	-1.076320553	0.180733138
Asgr2|11890	-1.096418096	0.291215596	-1.26558216	0.038846794
Gm4498|100043523	-1.096483333	0.170446566	1	#DIV/0!
Gm4141|100042976	-1.096483333	0.170446566	1	#DIV/0!
Gm4201|100043061	-1.096483333	0.170446566	1	#DIV/0!
Eif2s1|13665	-1.096509493	0.213106712	-1.186805366	0.054960954
Ppp2r1a|51792	-1.096513784	0.153696683	-1.154741739	0.035306027
Bmpr1a|12166	-1.096939075	0.389310242	-1.284003586	0.243202006
Mat2a|232087	-1.097064007	0.222713375	-1.084423654	0.174601027
Dnajc1|13418	-1.097125691	0.122950736	-1.163399748	0.015334217
Derl2|116891	-1.097160927	0.212259187	1.023860869	0.401627513
Commd7|99311	-1.097172173	0.246077623	1.001637467	0.493887675
Apba3|57267	-1.097396382	0.21663313	-1.097396382	0.21663313
Heatr5a|320487	-1.09740753	0.386915637	1.18198059	0.265400664
Tob2|57259	-1.097415327	0.357977996	-1.089451947	0.330037771
Rab5a|271457	-1.097550873	0.366770761	-1.257788738	0.21642058
C1qb|12260	-1.097571124	0.277097143	-1.20325055	0.119614719
Gemin6|67242	-1.097654385	0.412419184	-1.502208192	0.173804243
Tmcc2|68875	-1.097735474	0.363287203	1.049585241	0.428311361
Gtl3|14894	-1.097745924	0.267521458	-1.073298647	0.253464393
Atpif1|11983	-1.097786477	0.261808114	-1.018114279	0.445606348
Larp1|73158	-1.097842079	0.264968861	-1.17610584	0.149394356
Fam172a|68675	-1.097857199	0.418442077	-1.441216015	0.231761713
Yeats4|64050	-1.097877292	0.202348019	-1.034394589	0.367118686
1110054O05Rik|66209	-1.097889663	0.373681073	-1.510959448	0.053398815
Snx7|76561	-1.097927409	0.365030803	1.156522204	0.242736194
Grpel2|17714	-1.097929482	0.27921545	1.000723699	0.497823851
Ywhah|22629	-1.097940728	0.23080176	-1.028218209	0.403630952
Klkb1|16621	-1.097953588	0.232061415	-1.181249252	0.064494684
Gm2382|100039707	-1.098032046	0.146613358	-1.098032046	0.146613358
Amn1|232566	-1.09805755	0.414593618	1.228753995	0.315887073
Dynll1|56455	-1.098075588	0.280377922	-1.187476545	0.153121671
Bend7|209645	-1.098142162	0.353285021	1.090084437	0.341738948
B3gnt8|232984	-1.098181762	0.302373269	1.027416127	0.430915042
Ostm1|14628	-1.098184221	0.349375886	-1.098184221	0.349375886
Rnf167|70510	-1.098198278	0.277279036	-1.062105816	0.32861517
4932438A13Rik|229227	-1.098288834	0.38794194	1.044262091	0.443762944
Gpt|76282	-1.098380078	0.156009678	-1.145885642	0.079964729
Ushbp1|234395	-1.09838497	0.280361585	-1.173360784	0.119211936
Vps72|21427	-1.098389299	0.257069147	-1.212688837	0.07512037
Ecm1|13601	-1.098480497	0.17165278	-1.098480497	0.17165278
Mad2l2|71890	-1.098499773	0.212990624	-1.056119893	0.323850461
Mtpn|14489	-1.098515761	0.373389413	-1.039741235	0.42939764
Med18|67219	-1.098823745	0.356126905	1.048806003	0.411701733
Mob3a|208228	-1.098824805	0.35303444	-1.178343657	0.175991344
Tmem97|69071	-1.09891053	0.120563994	-1.163165101	0.005711297

Rai1|19377	-1.098918057	0.316482079	-1.289235729	0.067946587
Tns4|217169	-1.098923521	0.43049474	-1.643237139	0.199632415
Mtap7d1|245877	-1.098926735	0.088323672	-1.143410629	0.01640494
Mgat4a|269181	-1.098969044	0.426471358	1.351831964	0.273587034
Mrpl4|66163	-1.099019777	0.234589028	-1.172189147	0.094134357
Cfhr1|50702	-1.09902659	0.257513167	-1.196382976	0.107083494
Calcrl|54598	-1.099068589	0.41818106	-1.245882633	0.301680706
Cdc42se2|72729	-1.099074618	0.360376425	-1.108587709	0.341003647
E2f3|13557	-1.099134962	0.328650767	-1.099134962	0.328650767
A530013C23Rik|329562	-1.09916836	0.419169186	-2.044082876	0.020830994
Celsr1|12614	-1.099218024	0.322548806	1.162411082	0.119504353
Exosc5|27998	-1.099221159	0.299569077	-1.204985244	0.159474574
Slc9a3r2|65962	-1.099240799	0.281456898	-1.016939086	0.455715997
D6Wsu116e|28006	-1.099296716	0.235947152	-1.034262687	0.397509465
Gtpbp1|14904	-1.099328317	0.148553946	-1.099328317	0.148553946
Casp4|12363	-1.099362454	0.442759694	1.566000326	0.260764541
1810006K21Rik|69038	-1.099523228	0.328563388	1.020093653	0.462077049
Fam110b|242297	-1.09956913	0.441456198	-1.49318803	0.267883497
Pcolce|18542	-1.099635802	0.326143748	-1.305019121	0.072639224
Coq5|52064	-1.0996663	0.2328977	-1.0996663	0.2328977
Arl4d|80981	-1.09968912	0.328539987	-1.000630659	0.498859427
Syf2|68592	-1.099741906	0.141452459	-1.035898847	0.297797751
Ptdss1|19210	-1.099799279	0.15384786	-1.227207225	0.001349191
Icam1|15894	-1.099800115	0.233365406	-1.153431546	0.139567223
Sec62|69276	-1.099853367	0.157853157	-1.050400385	0.292669348
Zcchc2|227449	-1.099920532	0.375167959	-1.099920532	0.375167959
Txnl4b|234723	-1.099971207	0.256074996	-1.099971207	0.256074996
Slc22a5|20520	-1.099991492	0.225287065	-1.191199645	0.075512253
Gprc5c|70355	-1.099998958	0.192562044	-1.104419399	0.087029359
Ttyh1|57776	-1.10009172	0.409053658	-1.817530336	0.049125667
Apeh|235606	-1.10020687	0.188304726	-1.107976281	0.082586144
Aadac|67758	-1.100248404	0.131563864	-1.161307126	0.033200492
Vdac1|22333	-1.100251963	0.053976575	-1.17213277	0.000148122
2900097C17Rik|347740	-1.10026854	0.278951888	1.077606493	0.280742692
Tbc1d10a|103724	-1.100293936	0.239734498	1.052250026	0.305680401
Nostrin|329416	-1.100301045	0.293173125	-1.100054449	0.256198342
Mn1|433938	-1.100352448	0.356338071	-1.070002872	0.346281755
Cyp2a12|13085	-1.100514541	0.150661124	-1.054725511	0.270683477
Mars|216443	-1.10056644	0.16859738	-1.109892727	0.041699506
Mrrf|67871	-1.100581696	0.270427993	-1.100581696	0.270427993
Tmem20|240660	-1.100594113	0.331047683	1.009439171	0.474659656
Me1|17436	-1.10072901	0.207995587	-1.021274821	0.41111862
Enc1|13803	-1.100751063	0.377884206	-1.368164566	0.153304211
Bscl2|14705	-1.100783307	0.247938823	1.006865157	0.474449073
Nrgn|64011	-1.100884618	0.404309918	1.358528265	0.105649276
Uqcrc1|22273	-1.10093307	0.145199166	-1.10093307	0.145199166
Ddx19b|234733	-1.101004708	0.251973983	-1.101004708	0.251973983
Ogfrl1|70155	-1.10105329	0.382714276	1.143647253	0.323108016
Sgsm3|105835	-1.101091127	0.376012217	-1.233073143	0.200340315
Dus1l|68730	-1.101098307	0.232659049	-1.101098307	0.232659049
Abcd3|19299	-1.101151731	0.276446009	1.013229138	0.461466177
Klhl2|77113	-1.101160497	0.385995726	1.006319009	0.492418007
Rdh7|54150	-1.101191234	0.180880097	-1.164238843	0.078451095

Gltpd1|79554	-1.101252437	0.259019104	-1.033582594	0.334010421
Sod1|20655	-1.101383581	0.309673394	-1.225850014	0.153808647
Zfp825|235956	-1.101489748	0.218904881	-1.083575725	0.174479123
Fam36a|66359	-1.101597875	0.225100859	-1.16822288	0.101454452
9030625A04Rik|210808	-1.101600697	0.290727091	-1.216698121	0.135290528
Atg12|67526	-1.101647686	0.266682077	-1.224798926	0.034242197
Chid1|68038	-1.101667201	0.277345623	-1.202089599	0.137037482
Lyrm2|108755	-1.101977205	0.335510837	-1.449644014	0.01926469
Zfp35|22694	-1.101990623	0.28548669	-1.101990623	0.28548669
Poc1b|382406	-1.102074205	0.352725153	-1.241526785	0.217321483
Vma21|67048	-1.102088635	0.311479973	1.029313328	0.435070312
Dkc1|245474	-1.10214163	0.275990476	-1.10214163	0.275990476
Furin|18550	-1.10217291	0.130139312	-1.05680156	0.248550696
Slc39a14|213053	-1.102193098	0.267397729	1.051950033	0.350165117
Gtf3c2|71752	-1.102345359	0.247792838	-1.028095592	0.41641755
2010106G01Rik|66552	-1.102353182	0.312166476	1.137281848	0.163220536
Sgms2|74442	-1.102433335	0.356461262	-1.007670111	0.482920115
Tmem167b|67495	-1.102474084	0.33482457	-1.318577013	0.090123169
Mospd2|76763	-1.10253996	0.3975264	1.20033393	0.282772298
Ranbp2|19386	-1.102545158	0.413939963	-1.050107426	0.457137635
Mecr|26922	-1.102579282	0.247261065	-1.04452362	0.378778702
Cxcl1|14825	-1.102622038	0.446297183	-1.101104161	0.457196108
Carkd|69225	-1.102627731	0.208932944	-1.102627731	0.208932944
Anapc13|69010	-1.102634943	0.299798084	-1.091913658	0.257614372
Snai1|20613	-1.102654683	0.334136459	1.038188306	0.431029199
Atg9a|245860	-1.102746724	0.079922312	-1.016775197	0.326826317
Tst|22117	-1.102749648	0.274377194	-1.174418293	0.180474034
1110004F10Rik|56372	-1.10275926	0.167667386	-1.142288518	0.101001429
Gm8909|667977	-1.102768552	0.46446883	1	#DIV/0!
Chm|12662	-1.102771761	0.422709513	1.103344271	0.405332569
2310044H10Rik|69683	-1.102773128	0.287103952	-1.217162224	0.132785671
Ints4|101861	-1.102838343	0.283277585	-1.102838343	0.283277585
Fbxo30|71865	-1.102876109	0.363737751	1.034403878	0.441605177
D15Ertd621e|210998	-1.102983893	0.351261989	-1.096541384	0.270325645
Tssk2|22115	-1.103023756	0.461369474	1	#DIV/0!
Gfi1|14581	-1.103023756	0.461369474	1	#DIV/0!
Lgals2|107753	-1.103023756	0.461369474	1	#DIV/0!
Kcna5|16493	-1.103023756	0.461369474	1	#DIV/0!
Spdef|30051	-1.103023756	0.461369474	1	#DIV/0!
Rep15|66532	-1.103023756	0.461369474	1	#DIV/0!
Shc2|216148	-1.103023756	0.461369474	1	#DIV/0!
Scn3b|235281	-1.103023756	0.461369474	1	#DIV/0!
Ppifos|68173	-1.103023756	0.461369474	1	#DIV/0!
Mcm5|17218	-1.103070001	0.387421293	-1.103070001	0.387421293
Lrp10|65107	-1.103110074	0.177904179	-1.104550433	0.100595026
Msto1|229524	-1.103153221	0.269733208	-1.016903943	0.440069246
Cdc123|98828	-1.103190691	0.177465854	-1.047076174	0.318546057
Star|20845	-1.103300768	0.420451904	-2.289166424	0.002629101
Mrps36|66128	-1.103420828	0.278812929	-1.251351611	0.068561902
Zfp2|22678	-1.103655491	0.405852188	1.066898461	0.39959947
Wdr61|66317	-1.10367932	0.193392821	-1.038088117	0.361140061
Narg2|93697	-1.103704938	0.3592823	-1.143588669	0.235583368
Akap8l|54194	-1.103710289	0.2327165	-1.200505159	0.08252173

Larp1b|214048	-1.103789078	0.265322724	-1.19830045	0.049758081
2410089E03Rik|73692	-1.103816842	0.384328365	-1.288870008	0.195036129
Slc7a8|50934	-1.103883549	0.325651608	-1.029730539	0.411159615
Oas3|246727	-1.103925293	0.376935558	1.313055525	0.100568822
Rnft1|76892	-1.103968797	0.363168299	1.004523099	0.493447535
Ube2e1|22194	-1.103974735	0.385750729	-1.124134544	0.286606652
Mapk15|332110	-1.104093635	0.366729629	-1.004854459	0.493919215
Decr2|26378	-1.104257461	0.106444605	-1.062660509	0.207754675
Mctp2|244049	-1.104259806	0.375854756	-1.258515643	0.213439808
BC005624|227707	-1.10434101	0.190250846	-1.040583766	0.352877792
Acvr1|11477	-1.104360669	0.367391779	-1.238729384	0.253968022
Snx12|55988	-1.104367903	0.214074258	-1.104367903	0.214074258
Cul4b|72584	-1.104464669	0.336484258	1.059159237	0.392053935
Tnfaip8l1|66443	-1.104491668	0.256570045	-1.160858976	0.116662473
Nudt22|68323	-1.104634938	0.267346407	1.076267132	0.270383554
D430042O09Rik|233865	-1.104758527	0.213522093	1.055613915	0.207711461
Ripk2|192656	-1.104837218	0.377851859	-1.104837218	0.377851859
Atp6v1a|11964	-1.104901096	0.320603666	-1.104901096	0.320603666
Bub3|12237	-1.105047754	0.163039266	-1.134244205	0.122084009
Mettl16|67493	-1.105062806	0.216112306	-1.0214576	0.423262307
Engase|217364	-1.105071324	0.205303164	1.045855876	0.255150662
Abhd16a|193742	-1.105077884	0.069264145	-1.14462122	0.02426691
Ppp2r3a|235542	-1.105312383	0.349126836	1.00604124	0.490193812
Otud7b|229603	-1.105336207	0.262684519	-1.055049825	0.37032642
1110034B05Rik|68736	-1.105336319	0.40462589	-1.571445828	0.131377168
Mrps21|66292	-1.105458688	0.315893584	-1.065682065	0.305904921
Mrps27|218506	-1.105497001	0.258821134	-1.014346607	0.457136914
Klc4|74764	-1.105501532	0.161467622	-1.105501532	0.161467622
Grem2|23893	-1.105535734	0.356637826	-1.539438376	0.015224935
Pdzd8|107368	-1.105551509	0.340292329	1.105256187	0.286431049
9030425E11Rik|71566	-1.105600254	0.395377397	-1.742553972	0.031364529
9830001H06Rik|320706	-1.105610997	0.206384115	1.052837484	0.197073969
Gm11194|790911	-1.105686963	0.457785507	1.150701957	0.450338304
Rnf150|330812	-1.105747413	0.374879679	-1.105747413	0.374879679
Smg5|229512	-1.105873632	0.127251442	-1.065159991	0.12833148
Amigo2|105827	-1.105884813	0.294747666	-1.105884813	0.294747666
Rpa1|68275	-1.10590874	0.286301387	-1.343094339	0.02901624
Tomm70a|28185	-1.105979143	0.235818102	-1.310097651	0.004820899
Dnajc18|76594	-1.106000054	0.264590499	1.025683887	0.409639255
Psmd12|66997	-1.106080034	0.200526535	1.0315115	0.336522403
Tm2d2|69742	-1.106116043	0.152906918	-1.106116043	0.152906918
Umps|22247	-1.106140553	0.313553942	-1.054286169	0.40818156
Micall1|27008	-1.10614916	0.299717196	-1.303280931	0.046789255
Fut11|73068	-1.106152098	0.371944579	1.27033446	0.165570967
Lats2|50523	-1.106241909	0.412433195	-1.224583761	0.321752687
Abi2|329165	-1.106254396	0.417444862	-1.488990659	0.229586367
Fut10|171167	-1.106285461	0.428894684	1.24799293	0.361390656
Prickle3|54630	-1.106377716	0.264331835	-1.025635244	0.432369589
Gpm6a|234267	-1.106459524	0.44336767	-2.077814532	0.188577653
Vps11|71732	-1.106565678	0.183677708	-1.220868873	0.009204323
Prosc|114863	-1.106589394	0.119038228	-1.106589394	0.119038228
Serpina1b|20701	-1.106733169	0.231788677	-1.283232024	0.00350329
Ctsh|13036	-1.106816279	0.17954709	-1.236767915	0.013765771

F7|14068	-1.106816875	0.270252511	-1.212573064	0.127323376
Rnf20|109331	-1.106818375	0.319123041	1.0566967	0.377594974
Slc35c2|228875	-1.10688986	0.198925512	-1.267328383	0.009418539
Steap4|117167	-1.106910185	0.243640155	-1.106910185	0.243640155
Gpkow|209416	-1.106925437	0.35905561	-1.303324909	0.183512114
Pin1|23988	-1.10693089	0.197459294	-1.10693089	0.197459294
Pitpnc1|71795	-1.107076922	0.300460864	-1.074255893	0.322731779
Prpf40b|54614	-1.107092652	0.295100025	1.01364511	0.464405059
Gpr137|107173	-1.107107918	0.148261832	-1.04809064	0.293655633
Sin3a|20466	-1.107214122	0.246446463	-1.120640828	0.190601548
Jkamp|104771	-1.107235419	0.378000846	-1.107235419	0.378000846
Gpr89|67549	-1.107272036	0.266251488	-1.032918496	0.422682831
Cfp|18636	-1.107300686	0.258091511	-1.107300686	0.258091511
Rbm25|67039	-1.107319164	0.266620241	-1.016879207	0.422309473
Pknox1|18771	-1.10732262	0.194311877	-1.209891951	0.01321719
Tpd52l1|21987	-1.107373541	0.309134556	-1.054219697	0.313839484
Atg2a|329015	-1.107374067	0.086865592	-1.05926074	0.178906283
Dmbt1|12945	-1.107378048	0.470748597	-2.8	0.173296754
Pkm2|18746	-1.107397363	0.243135191	-1.076815804	0.223835871
Dbi|13167	-1.107454923	0.297714051	-1.107454923	0.297714051
Gosr2|56494	-1.107456763	0.105487075	-1.107456763	0.105487075
Mcc|328949	-1.107480957	0.312281131	-1.060445768	0.35990186
Map3k3|26406	-1.107502781	0.383514862	-1.107502781	0.383514862
1700021K19Rik|100502698	-1.107534302	0.199906029	-1.107534302	0.199906029
Ccdc88c|68339	-1.107556171	0.410420126	1.182933057	0.361044289
Znrf3|407821	-1.107598225	0.371133271	1.327627504	0.019691073
Appl1|72993	-1.10760624	0.365805139	-1.352733886	0.157412574
Pik3r1|18708	-1.107616598	0.361848974	1.227408811	0.196252074
Stau2|29819	-1.107649615	0.416124651	-1.305073909	0.276082037
Mtf1|17764	-1.107665513	0.16580976	-1.164693388	0.063834855
Fam176b|230752	-1.107673756	0.34825319	-1.302318508	0.161749213
Triap1|69076	-1.10777347	0.298053504	1.12281646	0.188878842
Tnpo3|320938	-1.107796142	0.2302513	-1.011081117	0.458647667
2010012O05Rik|66439	-1.107865732	0.271234374	-1.32900111	0.028348941
Abhd12|76192	-1.107878299	0.238817133	-1.139229924	0.093550531
Kremen1|84035	-1.107886268	0.272406618	-1.314043932	0.041210102
Mtmr14|97287	-1.108093191	0.244686526	1.064190107	0.233008862
Wdfy1|69368	-1.108182408	0.284307578	-1.011131229	0.474936062
Cnst|226744	-1.10850571	0.402873721	1.107748108	0.34120505
Stk39|53416	-1.108511966	0.403686133	-1.308146657	0.221157384
Lamp2|16784	-1.108514137	0.254952514	-1.116290072	0.237503357
C4bp|12269	-1.108687609	0.285459238	-1.013152094	0.470998302
Sec24c|218811	-1.108822247	0.1613695	-1.042212369	0.317766367
Ethe1|66071	-1.108854061	0.238512756	-1.194587602	0.118033265
Cnpy2|56530	-1.108890829	0.15150897	-1.087780132	0.215965812
Pnkp|59047	-1.108910646	0.269207718	-1.01403469	0.465415578
4930581F22Rik|78934	-1.108912523	0.286586363	-1.202065393	0.144395775
Ecsit|26940	-1.108986423	0.23551382	-1.279785428	0.026548699
Bag4|67384	-1.109052459	0.359361326	-1.321982116	0.173590732
Mtmr9|210376	-1.109072841	0.41917065	1.426576913	0.212510287
B4galt3|57370	-1.109133845	0.295403435	-1.374274907	0.02522531
Ublcp1|79560	-1.109174389	0.252471782	-1.219016307	0.057269627
Wac|225131	-1.109225176	0.212540733	-1.016966578	0.436624521

Pomt2|217734	-1.109426507	0.148851262	-1.03128175	0.32448956
Gpn3|68080	-1.109476205	0.2906711	-1.396740837	0.003428925
Grtp1|66790	-1.109479087	0.292046003	-1.109479087	0.292046003
Casp3|12367	-1.109481631	0.391004591	1.078699749	0.408394536
Paqr7|71904	-1.109483797	0.149790131	-1.109483797	0.149790131
Slc25a26|67582	-1.109500149	0.253102327	-1.218836565	0.059915749
2610029I01Rik|77032	-1.109526163	0.241361317	1.006549053	0.473037804
Znrd1|66136	-1.109553749	0.259323106	-1.220822609	0.107437584
Stag2|20843	-1.109690622	0.279106597	1.017580695	0.453168163
Cbx2|12416	-1.109794753	0.310998075	-1.328047986	0.000985848
Malt1|240354	-1.109807339	0.394994659	-1.409805383	0.206748338
Tln1|21894	-1.109886466	0.153501567	-1.162921892	0.07896819
Supt6h|20926	-1.109961251	0.110472982	-1.109961251	0.110472982
Rsf1|233532	-1.109978151	0.314415047	-1.009785066	0.480843622
Psmb8|16913	-1.110111384	0.265063331	-1.082189411	0.250887285
Oit3|18302	-1.110138532	0.222630333	-1.137315261	0.109420123
Psmb10|19171	-1.110198108	0.357442218	-1.268507924	0.124862363
Serpinf2|18816	-1.110262982	0.208909297	-1.120031215	0.13952764
Pdia3|14827	-1.110264041	0.245897066	-1.110264041	0.245897066
Th1l|57314	-1.110274736	0.195010903	-1.029394601	0.389916804
Rpgr|19893	-1.110369511	0.447767173	-1.036968712	0.482217965
Afap1l1|106877	-1.11044809	0.256023113	-1.213878333	0.078479375
1110008P14Rik|73737	-1.110491985	0.308443478	-1.097788722	0.30183335
Ctsb|13030	-1.110693559	0.12203297	-1.138985864	0.025549629
Akt1s1|67605	-1.110706307	0.211416865	-1.041167034	0.368196131
Gramd1c|207798	-1.110768456	0.252297127	-1.017287572	0.449591755
Isca2|74316	-1.110771938	0.2016744	-1.032839144	0.380733558
Gm3336|100041434	-1.110840338	0.40839292	-1.294709025	0.200701304
Stt3b|68292	-1.110879597	0.205646563	-1.182300617	0.080239932
Ube2l6|56791	-1.110890582	0.046744228	-1.110890582	0.046744228
Bag2|213539	-1.110910985	0.297642976	-1.294749682	0.07317934
Poll|56626	-1.110939614	0.256689948	-1.013232892	0.464697767
Hdac5|15184	-1.110948608	0.195119984	-1.109660573	0.10187663
Zfp94|22756	-1.11098598	0.398440205	-1.411145229	0.219727047
Thpo|21832	-1.111041211	0.234662526	-1.342109453	0.000715648
Cog4|102339	-1.111298475	0.159255882	-1.114142822	0.085190994
Ubl3|24109	-1.111317483	0.228705355	-1.002153367	0.49195363
Tbc1d25|209815	-1.111359305	0.342856213	-1.394703273	0.067880075
Pex11b|18632	-1.111367437	0.113417543	-1.138411559	0.080908498
Lbp|16803	-1.111497231	0.154745119	-1.158872301	0.092000771
Lcp1|18826	-1.111499801	0.264986523	-1.111499801	0.264986523
Akr7a5|110198	-1.111551277	0.313148704	-1.319809258	0.0766729
Mrps2|118451	-1.111595606	0.313129146	-1.219801433	0.166078118
Rnf141|67150	-1.111606482	0.427468748	1.59679169	0.175708399
Dctn5|59288	-1.111636596	0.276113839	-1.082574284	0.259386008
Dync1li1|235661	-1.111705663	0.221688397	-1.111705663	0.221688397
Rpe|66646	-1.111729495	0.333219985	1.015627999	0.475144227
Ddx54|71990	-1.111889008	0.225825863	-1.120661677	0.144997835
Ptk2b|19229	-1.112037451	0.153831	1.001957967	0.489030649
Trappc2l|59005	-1.11204765	0.299307973	1.152740182	0.07098105
Cs|12974	-1.112079557	0.085055309	-1.135916065	0.060885929
Tstd2|272027	-1.112083631	0.369736722	1.090153211	0.391516785
Zswim4|212168	-1.112113203	0.213922314	-1.187946364	0.082497338

Rad51l3|19364	-1.112229317	0.224296694	-1.20880169	0.08706057
Atp6v0a1|11975	-1.112347847	0.126595337	1.000294618	0.49790148
Ndufab1|70316	-1.112387674	0.296898894	1.011340967	0.475766955
Mllt4|17356	-1.112394862	0.399792432	1.08902241	0.37673646
Mut|17850	-1.112410977	0.326648946	1.051242886	0.406809493
AI314180|230249	-1.112520703	0.142970971	-1.036876802	0.306013003
Fbxl19|233902	-1.112523182	0.157628457	-1.049900071	0.300280968
Rab1|19324	-1.11255315	0.267344944	-1.019535657	0.450066644
Prmt2|15468	-1.112578498	0.411759383	-1.630036358	0.163641542
Sh3tc1|231147	-1.112626488	0.295505242	-1.411927822	0.004393944
Atg4b|66615	-1.112652312	0.173229209	-1.112652312	0.173229209
Mtx2|53375	-1.112654646	0.255638483	-1.036232662	0.414098127
Tm7sf3|67623	-1.112678056	0.09520585	-1.112678056	0.09520585
Hadha|97212	-1.112690885	0.105316282	-1.112690885	0.105316282
Dlat|235339	-1.11271352	0.301790856	1.115875043	0.253480367
Pop7|74097	-1.112887052	0.263120415	-1.050579951	0.385369415
Sccpdh|109232	-1.112892251	0.388346995	1.294136637	0.148074256
Lilra5|232801	-1.112912637	0.375775578	-1.468814834	0.113950942
Rbm47|245945	-1.112989136	0.342181553	-1.232569317	0.235020501
Erlin1|226144	-1.113044392	0.307975405	-1.369798337	0.019122428
Rab8b|235442	-1.11311066	0.385629168	-1.347192347	0.23014393
Tbccd1|70573	-1.113123403	0.164006586	-1.113123403	0.164006586
Selenbp2|20342	-1.113132509	0.398073734	-1.568755008	0.136152677
Ccz1|231874	-1.113222161	0.334243251	-1.113222161	0.334243251
Stx18|71116	-1.113253848	0.202446647	-1.113253848	0.202446647
Ntrk2|18212	-1.11330059	0.419060125	1.324251398	0.298690411
Shroom3|27428	-1.113328958	0.216339753	-1.243776443	0.02870958
Zfp523|224656	-1.113382568	0.243249368	-1.113382568	0.243249368
Dnajc19|67713	-1.113389438	0.246895029	-1.113389438	0.246895029
Ap1ar|211556	-1.113389825	0.336832916	1.220222648	0.111556157
Elovl1|54325	-1.113600083	0.248989958	1.014066956	0.451084106
Zfp868|234362	-1.113631599	0.392222239	1.035863469	0.467742906
H2-Q6|110557	-1.113641368	0.299483957	1.006481709	0.486812659
Padi2|18600	-1.113644249	0.41421642	-1.147248567	0.382504897
Kcnj16|16517	-1.113654411	0.260440914	-1.347945206	0.013470644
Cacng1|12299	-1.113734611	0.436962391	1.820526892	0.173977924
Pcbd1|13180	-1.113779743	0.084236672	-1.173031938	0.005246178
Igf2bp2|319765	-1.113791759	0.315391909	-1.113791759	0.315391909
Slc12a9|83704	-1.113813944	0.251503077	-1.197014984	0.119176624
Lin37|75660	-1.113819901	0.284448195	-1.025554266	0.443911024
2700007P21Rik|212772	-1.11402108	0.360463761	-1.303566538	0.207081988
Ccdc125|76041	-1.114068266	0.251775512	-1.114068266	0.251775512
Pigo|56703	-1.114084326	0.210959201	-1.011105711	0.456397786
Phactr4|100169	-1.114102693	0.242100754	-1.080484105	0.278101146
Limk2|16886	-1.114141476	0.348548255	1.121097904	0.298552698
Srp54a|24067	-1.114197098	0.299096474	-1.043895774	0.422644677
St7|64213	-1.114246149	0.26215878	-1.265757551	0.062537619
Apoh|11818	-1.114311589	0.187835869	-1.224776373	0.002410226
Asah1|11886	-1.114337549	0.370209515	1.13988931	0.325746117
G3bp2|23881	-1.114410485	0.346718534	-1.036635755	0.43037128
Rxrg|20183	-1.114463113	0.322222584	-1.114463113	0.322222584
Bdh1|71911	-1.114466795	0.121856519	-1.070628542	0.221379428
Cope|59042	-1.114489245	0.220190321	1.00291048	0.487391157

Hgs|15239	-1.114619621	0.050311372	-1.08555292	0.101316807
Rhot1|59040	-1.114642578	0.194173113	-1.114642578	0.194173113
Cd80|12519	-1.114716062	0.451648716	3.286172748	0.091708258
Slc25a4|11739	-1.114934452	0.298510357	-1.037580914	0.428452184
Tmem30b|238257	-1.114991661	0.316589944	-1.29693586	0.12384731
Slc10a2|20494	-1.115015901	0.406700742	1.332386316	0.238370282
Mrpl16|94063	-1.115063344	0.151861617	-1.115063344	0.151861617
Alg5|66248	-1.1151115	0.090404329	-1.020333699	0.353729502
Mrps5|77721	-1.115155545	0.138991242	-1.048509849	0.28141885
Mrpl38|60441	-1.115261996	0.172978171	-1.192499245	0.038564276
Tlcd1|68385	-1.115285344	0.273465587	-1.115285344	0.273465587
Mau2|74549	-1.115311263	0.222977449	-1.115311263	0.222977449
Ifrd2|15983	-1.115382933	0.012720444	-1.082348911	0.015203205
Fam161a|73873	-1.115391161	0.385784906	-1.251049021	0.273154605
2610008E11Rik|72128	-1.115401256	0.401707912	1.245554265	0.298694359
Mrps18a|68565	-1.115418561	0.204338764	-1.115418561	0.204338764
3110002H16Rik|76482	-1.115439708	0.286812322	-1.235432729	0.10539683
Mov10|17454	-1.115603731	0.162532682	-1.097975059	0.136369799
Nt5dc1|319638	-1.115668705	0.367181478	1.315267625	0.111740721
Glod4|67201	-1.115733126	0.20631701	-1.1829533	0.117927209
Rpl30|19946	-1.115755573	0.089832785	-1.178669013	0.005031281
Mrpl1|94061	-1.115781648	0.285074747	1.051697781	0.34523326
Nags|217214	-1.115818028	0.162200317	-1.158265225	0.111904133
Bud13|215051	-1.115900442	0.245960695	-1.018648745	0.4498611
Tarbp2|21357	-1.115907818	0.197892521	-1.115907818	0.197892521
Ccdc97|52132	-1.115909391	0.163917273	-1.134904503	0.056330258
Mbd3|17192	-1.115941609	0.300888108	-1.273782517	0.124328081
Asxl1|228790	-1.115973691	0.272283313	-1.052404479	0.390071533
Serping1|12258	-1.11598166	0.172971012	-1.11598166	0.172971012
Lmbr1|56873	-1.11599785	0.363954796	-1.614474685	0.036164142
Prrg2|65116	-1.116016476	0.203253447	-1.196268756	0.093329136
Zfp498|666311	-1.116026554	0.376892358	-1.116026554	0.376892358
Gjb1|14618	-1.116159675	0.192876088	-1.214142051	0.053490258
Cul9|78309	-1.116269814	0.244397263	-1.116269814	0.244397263
Fbxw10|213980	-1.116286781	0.456834809	1	#DIV/0!
Scn4a|110880	-1.116286781	0.456834809	1	#DIV/0!
Kdelr3|105785	-1.116286781	0.456834809	1	#DIV/0!
Gpx2|14776	-1.116286781	0.456834809	1	#DIV/0!
Tmem215|320500	-1.116286781	0.456834809	1	#DIV/0!
D730005E14Rik|109361	-1.116286781	0.456834809	1	#DIV/0!
Ipo5|70572	-1.116352905	0.113440971	-1.137254054	0.03443302
Tanc2|77097	-1.11637934	0.401078244	-1.751491996	0.071732125
Cstf3|228410	-1.116394418	0.239501145	-1.116394418	0.239501145
Cd7|12516	-1.116403127	0.408171134	1.066433433	0.442314567
Spop|20747	-1.116480845	0.263241317	-1.048162464	0.291238775
Mis12|67139	-1.116492241	0.300746551	-1.116492241	0.300746551
Tmed10|68581	-1.116514726	0.029414511	-1.084849828	0.060758923
Tmem53|68777	-1.116669671	0.236531566	-1.200727338	0.031896508
Wars|22375	-1.116680568	0.248533635	-1.036490445	0.406752853
Rrp12|107094	-1.116695724	0.240177318	-1.238456333	0.077375668
Unc93b1|54445	-1.116704051	0.067622153	-1.142326697	0.049100808
Amotl1|75723	-1.116773324	0.210810335	-1.189698426	0.091179678
Rpl7|19989	-1.116806893	0.146927635	-1.061679211	0.269194786

Xpo1|103573	-1.116809298	0.232445782	-1.21967188	0.095284204
Cmtm8|70031	-1.116820538	0.067983532	-1.206962169	0.002891802
Mtch1|56462	-1.116860131	0.171861672	-1.212566718	0.036345097
Serpinb8|20725	-1.116917567	0.303460264	-1.274795404	0.131000826
C230052I12Rik|101831	-1.116966399	0.335109119	1.212485726	0.136502161
Trappc3|27096	-1.117048922	0.09968561	-1.235171833	0.001010217
Ppara|19013	-1.117083043	0.118529808	-1.043256599	0.253788758
Ehmt2|110147	-1.117140599	0.160616946	-1.165329868	0.050774779
2210411K11Rik|664968	-1.117170628	0.338622162	1.200558067	0.186209174
Dync1li2|234663	-1.117171038	0.267328946	1.017793448	0.450167145
Wdr45l|66840	-1.117206418	0.166442447	-1.117206418	0.166442447
Ddx19a|13680	-1.11729046	0.226448969	-1.208777952	0.105662939
Glipr2|384009	-1.117371647	0.417791125	-1.117371647	0.417791125
Heatr2|433956	-1.11749856	0.201493337	-1.11749856	0.201493337
Pcid2|234069	-1.117555517	0.167241688	-1.071279278	0.1709662
Ssr3|67437	-1.117629705	0.174645291	-1.030146857	0.373303343
Limk1|16885	-1.117732191	0.332530455	1.119414868	0.252462643
Igf1r|16001	-1.117804586	0.39161909	-1.399518427	0.224715379
Prodh2|56189	-1.117814045	0.246277712	-1.295586087	0.016988836
Aup1|11993	-1.117817817	0.203622552	-1.194657072	0.101576833
Strn4|97387	-1.1178207	0.243843398	-1.1178207	0.243843398
Lrrc41|230654	-1.117824975	0.160226114	-1.049613078	0.308138464
Gm13498|227885	-1.11786922	0.314499667	-1.005713667	0.490369901
Cfd|11537	-1.117929232	0.468955426	-2.8202	0.173296754
Fcer1g|14127	-1.117991094	0.288461544	-1.117991094	0.288461544
Sgta|52551	-1.118009885	0.119913874	-1.228849365	0.008690011
Rela|19697	-1.118086247	0.10966327	-1.143377558	0.027771886
Hdlbp|110611	-1.118235667	0.089789327	-1.070887144	0.181321
Ndrg2|29811	-1.118271659	0.176611506	-1.216310549	0.039577327
Amh|11705	-1.118272848	0.435132612	1.018192343	0.490530101
Fam92a|68099	-1.118294675	0.334533804	-1.212920139	0.231654465
Acsm5|272428	-1.118298836	0.165275317	-1.27966975	0.003392185
Lman2|66890	-1.118306618	0.030506095	-1.118306618	0.030506095
Pak2|224105	-1.11851635	0.281369299	-1.361810609	0.020341498
Ppp2r3c|59032	-1.118541792	0.225910242	1.050650263	0.320624319
Tsg101|22088	-1.118561213	0.200605902	-1.118561213	0.200605902
Jub|16475	-1.118576707	0.287276714	1.03316087	0.419042905
Ppl|19041	-1.118617222	0.359243842	1.055781144	0.3932856
Cd33|12489	-1.11867306	0.454690003	-1.305244483	0.378399987
Rhbdl1|214951	-1.118721254	0.355378637	1.031820344	0.438482947
3830406C13Rik|218734	-1.118853994	0.211437447	-1.002842533	0.487877475
Hpgd|15446	-1.118908696	0.268491953	-1.014936594	0.462832328
Fam38b|667742	-1.11891447	0.38785793	-1.00734791	0.492397667
Fah|14085	-1.118936212	0.154440105	-1.176278521	0.08116936
Akr1b8|14187	-1.119009192	0.322970217	-1.019592182	0.470233855
Snd1|56463	-1.119031485	0.218956001	-1.053345304	0.354927216
2510006D16Rik|76799	-1.119072948	0.084587625	-1.119072948	0.084587625
D930015E06Rik|229473	-1.119134376	0.126086274	-1.121073066	0.074362361
Oma1|67013	-1.119195609	0.286087705	-1.314624356	0.058635642
Ccdc127|67433	-1.119227608	0.12944888	-1.119227608	0.12944888
Sbf1|77980	-1.119330403	0.219861591	-1.054079518	0.360933687
Slc37a2|56857	-1.119375667	0.365359336	-1.375865193	0.125047615
Dhrs4|28200	-1.119426077	0.128437571	-1.119426077	0.128437571

Poldip2|67811	-1.119436999	0.128424945	-1.119436999	0.128424945
Fam53a|74504	-1.119521464	0.259950442	-1.261296473	0.083540126
Pfdn4|109054	-1.11955125	0.34614374	-1.367468815	0.134236468
Cand1|71902	-1.119586195	0.228944363	-1.228020726	0.086781707
Ythdc1|231386	-1.119649355	0.338660292	1.094450359	0.344233609
2810410L24Rik|100042332	-1.119701766	0.390881117	-1.918229861	0.013472978
Fam53b|77938	-1.119723365	0.233029282	-1.250126249	0.06092429
Stx16|228960	-1.119730534	0.300169456	1.033049304	0.397883278
Vill|22351	-1.119795517	0.386642708	-1.336006132	0.198731765
Spata2|263876	-1.119854733	0.252588263	1.085249797	0.207846437
Sumo1|22218	-1.119894918	0.259321875	-1.119894918	0.259321875
Tbx2|21385	-1.119925586	0.351854249	1.030591469	0.454699156
Usp16|74112	-1.119939465	0.319491984	-1.227663966	0.190252185
Ext1|14042	-1.120046461	0.177881134	-1.204330413	0.063679525
1300018J18Rik|223776	-1.120048575	0.216562971	-1.190017106	0.130241671
Pogz|229584	-1.120158085	0.293238205	-1.006957994	0.483310387
Psme2|19188	-1.12020238	0.362284952	1.011679186	0.4769713
1500002O20Rik|71997	-1.120228264	0.211901355	-1.120228264	0.211901355
Prpf4|70052	-1.120251262	0.252366725	-1.205348506	0.125696471
Ces1e|13897	-1.120275462	0.288347483	-1.297704987	0.017099651
Klf4|16600	-1.120288085	0.386150145	-1.411513943	0.208960691
Ccnd2|12444	-1.120344064	0.390155491	-1.127248857	0.380974524
Ube2j1|56228	-1.120379165	0.196764032	-1.208304354	0.079416978
Cyp4f15|106648	-1.120479694	0.236705849	-1.285102028	0.024358684
Dph2|67728	-1.120631325	0.328907642	1.173755311	0.219962008
Pi16|74116	-1.120639499	0.441669825	-1.581645013	0.312620442
Aebp1|11568	-1.120644876	0.411079166	-1.120644876	0.411079166
Bap1|104416	-1.120674402	0.161499799	1.008235911	0.460262594
Gys1|14936	-1.120687059	0.319624276	-1.278205848	0.113120004
Rrnad1|229503	-1.120750454	0.246144524	-1.042965897	0.394929665
Sfrp5|54612	-1.120830446	0.377816357	-1.20151509	0.275095866
Anxa7|11750	-1.120844763	0.21028128	-1.228298293	0.066689978
Arhgef16|230972	-1.120864797	0.375133176	1.137470391	0.352317177
Llgl2|217325	-1.12087657	0.089632227	-1.168293475	0.038567949
Kctd2|70382	-1.120924891	0.171750789	-1.041153185	0.347279844
Stam|20844	-1.120958159	0.366540253	-1.407412725	0.159644715
Pum1|80912	-1.120959134	0.221670436	-1.004999612	0.482024177
Celsr2|53883	-1.121040454	0.403316093	-2.018776862	0.000360338
Pde4d|238871	-1.121050579	0.350047045	1.056857202	0.425085697
Arhgap42|71544	-1.121151144	0.356623951	-1.096466413	0.369899751
Calm1|12313	-1.121171814	0.034529558	-1.07357186	0.056897095
Ppm1k|243382	-1.121259441	0.328189506	-1.053640658	0.406822385
Des|13346	-1.121293433	0.375013175	1.193024035	0.277546976
Mesdc2|67943	-1.121298227	0.242040948	-1.225246557	0.076592633
Slc35d2|70484	-1.121331725	0.201538662	-1.106050964	0.144065498
Acot13|66834	-1.121338002	0.290033446	-1.321450426	0.065652141
Tspan9|109246	-1.121386156	0.210894407	-1.057194397	0.299313326
Arhgef19|213649	-1.12144935	0.014744689	-1.080901424	0.015488771
Rian|75745	-1.12145379	0.432737273	-1.945954545	0.198949878
B3galnt2|97884	-1.121515267	0.250855677	-1.121515267	0.250855677
H2-T10|15024	-1.121736851	0.36387237	-1.11845961	0.334609079
Wfikkn1|215001	-1.121743596	0.424947084	1.32508503	0.336299047
Dpcd|226162	-1.121822737	0.266126926	-1.121822737	0.266126926

Picalm|233489	-1.121846955	0.312568141	-1.121846955	0.312568141
Chmp1a|234852	-1.121880716	0.054149382	-1.121880716	0.054149382
Dpyd|99586	-1.121957115	0.359951851	-1.018005216	0.467709354
1300002K09Rik|74152	-1.121972681	0.113285918	-1.22449057	0.011678065
Pelp1|75273	-1.122099274	0.129305365	-1.183560076	0.035784467
Yes1|22612	-1.12225743	0.373952467	1.34243224	0.14304793
Ccdc77|67200	-1.122340225	0.364982218	-1.04443916	0.442129618
Amacr|17117	-1.122346032	0.14441648	-1.112669858	0.128639674
Gps1|209318	-1.122366285	0.214792411	-1.240349063	0.059564012
Kirrel|170643	-1.122413197	0.363517456	-1.122413197	0.363517456
Sirt6|50721	-1.122471411	0.3260399	-1.122471411	0.3260399
Zfp937|245174	-1.122558018	0.392333158	-1.337399206	0.221360425
Sltm|66660	-1.12265571	0.210408296	-1.007593303	0.470434448
Polr3d|67065	-1.122681868	0.25630204	-1.020255948	0.448104731
Ormdl3|66612	-1.122783985	0.090199469	-1.122783985	0.090199469
Odf2l|52184	-1.122823639	0.400869599	1.185489476	0.361115938
Mtrf1|211253	-1.122864442	0.263522989	-1.290848336	0.06210249
Arv1|68865	-1.122932958	0.337325636	1.051178635	0.425139543
Atxn1l|52335	-1.123101042	0.37125026	-1.099843164	0.38144427
Wdr53|68980	-1.123180513	0.287759952	-1.017357748	0.463830904
Zmym1|68310	-1.123182012	0.448325546	-2.093828795	0.230800024
Dgat2|67800	-1.123251752	0.036742491	-1.123251752	0.036742491
Sik3|70661	-1.123294896	0.215515343	1.055744049	0.284015502
Cpox|12892	-1.123329221	0.314576528	1.17050691	0.179855171
Mknk1|17346	-1.123422927	0.157818139	-1.188851554	0.055707375
Aldh1b1|72535	-1.123590546	0.271302789	-1.28234821	0.009032072
Cmtm6|67213	-1.123641299	0.277189031	1.00146929	0.496181233
Map2k3|26397	-1.123658819	0.077408054	-1.151540398	0.051130896
Smoc1|64075	-1.123698937	0.083074846	-1.022519515	0.327810163
Galns|50917	-1.123731748	0.28642357	-1.045962342	0.418980055
Sfn|55948	-1.123784775	0.313495221	-1.238936951	0.139951889
1700056E22Rik|73363	-1.123804584	0.416799005	-1.123804584	0.416799005
Qdpr|110391	-1.123832538	0.073871212	-1.026793734	0.271636516
Plk5|216166	-1.123836784	0.347453088	1.114140988	0.33253873
Gria3|53623	-1.123870601	0.352214085	1.173828244	0.216819089
Pgam1|18648	-1.123887364	0.240445556	-1.026979065	0.426711257
Tesk2|230661	-1.123914132	0.296573355	-1.25519318	0.118437698
Wdr20a|69641	-1.1239508	0.337118613	1.042488908	0.427309863
Naprt1|223646	-1.124068945	0.173965803	-1.102042999	0.181490349
2410002I01Rik|78777	-1.124118398	0.138245174	-1.072890226	0.246516663
Acpp|56318	-1.124144623	0.337717996	-1.32561556	0.166819216
Tmem201|230917	-1.124199024	0.164349834	-1.056792385	0.310655478
Smpdl3a|57319	-1.124238309	0.242425066	-1.1717565	0.1110096
Usp28|235323	-1.124268213	0.38633496	-1.114026526	0.383537011
41156|18952	-1.124395976	0.281750109	-1.437746096	0.010492798
Hgsnat|52120	-1.124434655	0.231114065	-1.124434655	0.231114065
Hspa12a|73442	-1.1244509	0.407447216	-1.1244509	0.407447216
4732418C07Rik|230648	-1.124488451	0.332846823	-1.036605113	0.41383104
Pdk2|18604	-1.124512013	0.042556946	-1.124512013	0.042556946
Ldb2|16826	-1.124544709	0.392030863	1.214013749	0.317406409
Sigmar1|18391	-1.124546607	0.065114763	-1.067245027	0.123941211
Rnpep|215615	-1.124548317	0.288523875	-1.010427342	0.475070477
Zfp9|22750	-1.124555529	0.381171211	-1.618341986	0.084473199

Ndufa9|66108	-1.124581645	0.023054505	-1.156298187	0.008761736
1700123O20Rik|58248	-1.124733189	0.203040389	-1.124733189	0.203040389
Zfp592|233410	-1.124746463	0.070905755	-1.091746311	0.133147052
Gnpda1|26384	-1.124792522	0.192608251	-1.124792522	0.192608251
Top1mt|72960	-1.124929468	0.217850178	-1.124929468	0.217850178
Itpripl1|73338	-1.125003322	0.225702221	-1.236753799	0.046299551
Dnajb4|67035	-1.125088974	0.327551046	-1.281840232	0.190248173
Cse1l|110750	-1.125107954	0.155329312	-1.125107954	0.155329312
Pmpca|66865	-1.125198444	0.067129275	-1.203751584	0.001074403
Zxdc|80292	-1.125213459	0.125373738	-1.054711518	0.25767408
Eif4g3|230861	-1.12525225	0.273089164	-1.004227368	0.490719753
Pccb|66904	-1.125259315	0.023607845	-1.129813196	0.000541698
Ttc39b|69863	-1.125282672	0.364049913	-1.020403321	0.474559715
Timd2|171284	-1.125300192	0.163485541	-1.055343428	0.306536612
Uros|22276	-1.125381256	0.212892509	-1.125381256	0.212892509
Cela1|109901	-1.125468955	0.294622782	-1.263825226	0.105675005
Flywch1|224613	-1.125488933	0.257342245	-1.077467793	0.289277978
Med24|23989	-1.125491115	0.165597179	-1.091983527	0.155129886
1110012L19Rik|68618	-1.125517743	0.299899435	-1.108637697	0.224855277
Pisd-ps3|66776	-1.125577849	0.384968935	-1.125577849	0.384968935
Pwwp2a|70802	-1.125600654	0.386651969	1.392530321	0.164976997
Glis2|83396	-1.125605107	0.399446701	1.115665165	0.414499317
Cpsf4l|52670	-1.125681895	0.32120439	-1.07139176	0.311755118
Arhgap1|228359	-1.125763773	0.209078025	-1.125763773	0.209078025
Extl3|54616	-1.12577859	0.153863163	-1.279193807	0.007252194
Zfp36l2|12193	-1.125819025	0.359654059	-1.125819025	0.359654059
6430573F11Rik|319582	-1.125856171	0.382569413	1.211482761	0.292589443
Cyp4f13|170716	-1.125873038	0.115715258	-1.074087255	0.218147672
Il15|16168	-1.125875868	0.432950309	-1.125875868	0.432950309
Nit1|27045	-1.125876408	0.098111017	-1.083338137	0.186265117
Ywhag|22628	-1.125968422	0.316901894	1.05594067	0.39998909
Sgsh|27029	-1.125970547	0.245776959	-1.030133208	0.423441428
Enpep|13809	-1.125977706	0.33186084	-1.125977706	0.33186084
Aldh3b2|621603	-1.126026437	0.444041762	-2.228922062	0.156240191
Nus1|52014	-1.126055695	0.315048459	-1.127754876	0.258897602
Phgdh|236539	-1.126075381	0.440456384	1.823071741	0.250512967
Runx2|12393	-1.126077601	0.440378648	-1.189393939	0.435388966
Ccdc47|67163	-1.126123449	0.258872635	1.010862485	0.470803909
Fgfrl1|116701	-1.126126764	0.279863732	1.004338821	0.490719026
Ube3a|22215	-1.126146512	0.283548651	-1.016684751	0.468019769
Fdxr|14149	-1.126241524	0.136554157	-1.18270796	0.054750624
Ell2|192657	-1.126283977	0.32143879	1.053080393	0.410792135
41158|56526	-1.126326186	0.373372685	-1.500607186	0.133601921
Fbxo46|243867	-1.126388829	0.22002063	-1.124187172	0.194502338
Irak1bp1|65099	-1.126416032	0.431176197	1.019949115	0.487108385
Cdkn2c|12580	-1.12643792	0.270245714	-1.410169777	0.016325183
Fam134a|227298	-1.12651328	0.03657762	-1.14867282	0.005154056
Farsa|66590	-1.126587603	0.153905131	-1.160788747	0.051279427
Dlst|78920	-1.126601608	0.150340143	-1.000226837	0.498956496
Topors|106021	-1.126622248	0.287482158	-1.089008123	0.262521427
Pcmtd1|319263	-1.126686644	0.046842232	-1.155745573	0.029570082
2210016L21Rik|72357	-1.126695527	0.223668763	-1.244269753	0.078158961
Mll2|381022	-1.126699203	0.262241653	-1.038289945	0.391697677

P2ry6|233571	-1.126700443	0.342552256	-1.345837425	0.166561399
Ivd|56357	-1.12671613	0.040366786	-1.162761661	0.001571457
Tmcc3|319880	-1.126811529	0.353393628	1.094152911	0.380857143
Cbx7|52609	-1.126843374	0.273526756	-1.126843374	0.273526756
Gm14057|100043766	-1.126864877	0.399941675	-2.054662098	0.009452415
Rrp7a|74778	-1.126871006	0.175595994	-1.048103126	0.334338868
Ap3m1|55946	-1.126944567	0.211925013	-1.093963083	0.21797025
Dusp19|68082	-1.126947809	0.326658168	-1.294344714	0.125686807
Glul|14645	-1.126956041	0.226215396	-1.066497868	0.206249765
Polr1b|20017	-1.12699484	0.23448992	-1.23012049	0.112551958
Cyfip1|20430	-1.127053667	0.245302895	-1.039856609	0.404218614
Rsbn1l|242860	-1.127159017	0.419620129	-1.127159017	0.419620129
5730419I09Rik|74741	-1.127264448	0.394440386	-1.867827307	0.061123356
Scaf8|106583	-1.127318484	0.278996746	-1.04845074	0.386904707
Gng11|66066	-1.127342473	0.316116521	1.002517909	0.496045375
Mtus2|77521	-1.127433032	0.400150048	-1.127433032	0.400150048
Nipal2|223473	-1.127461982	0.454906509	3.53972	0.173296754
Dars|226414	-1.127550093	0.288375687	1.132476057	0.206159911
1810058I24Rik|67705	-1.127598377	0.255366172	-1.240389174	0.128755357
Cfh|12628	-1.127672517	0.316403156	-1.420271729	0.043443876
Prss23|76453	-1.127676141	0.349245418	-1.299564707	0.171055652
Htra2|64704	-1.127712703	0.211943852	-1.127712703	0.211943852
Col14a1|12818	-1.127736316	0.123632467	-1.086516376	0.138004321
Iffo2|212632	-1.127804076	0.259254165	-1.226585335	0.120721515
3110052M02Rik|73229	-1.127910262	0.327950949	1.177739464	0.225237487
Iqcb1|320299	-1.128081344	0.378656038	-1.128081344	0.378656038
Nudt8|66387	-1.128085126	0.254076532	-1.128085126	0.254076532
Ski|20481	-1.128101976	0.189810251	1.031521568	0.368635938
9430020K01Rik|240185	-1.128160362	0.348681407	-1.137279303	0.32665195
Cuedc2|67116	-1.128177954	0.306465385	-1.167453968	0.174224216
Cep120|225523	-1.128306815	0.31180533	1.051256965	0.406624165
Txnrd2|26462	-1.128318073	0.131349949	-1.101840888	0.197066097
Abhd6|66082	-1.128365865	0.146869219	-1.209001155	0.04768349
Ripk4|72388	-1.128428711	0.324543843	-1.128428711	0.324543843
Pecr|111175	-1.128505933	0.1299312	-1.205986703	0.037037772
Nkap|67050	-1.128738511	0.303642123	1.035313616	0.433799624
Rnf166|68718	-1.128784369	0.185433074	-1.300619521	0.016943857
Eaf2|106389	-1.128859423	0.430027993	-1.128859423	0.430027993
Clptm1l|218335	-1.12886586	0.197107704	-1.239002972	0.059371039
Nipsnap1|18082	-1.128874078	0.047244043	-1.097555029	0.053651095
Fgfr4|14186	-1.1289507	0.170698167	-1.139634812	0.08148461
Eftud1|101592	-1.129121707	0.172129495	-1.129121707	0.172129495
Gabra3|14396	-1.12920245	0.40716763	1.126302435	0.418408492
Sharpin|106025	-1.129225701	0.116452169	-1.082482271	0.210114625
Dcaf5|320808	-1.129250124	0.096507923	-1.129250124	0.096507923
Coq10a|210582	-1.129308668	0.169026224	-1.221296418	0.026329177
Nfya|18044	-1.129545515	0.251835634	-1.129545515	0.251835634
Lrrc39|109245	-1.129557327	0.41051774	-1.650632238	0.134162752
Slc30a10|226781	-1.129628991	0.299279137	-1.303483068	0.129686319
Nudt9|74167	-1.129709503	0.101443516	-1.245354641	0.00100383
Zfp873|408062	-1.129749119	0.441537052	-1.129749119	0.441537052
Nbr1|17966	-1.129776754	0.293596255	1.125211054	0.250815401
Serinc1|56442	-1.129782912	0.33235311	-1.129782912	0.33235311

Glt1d1|319804	-1.129801618	0.365265222	-1.129801618	0.365265222
Fen1|14156	-1.129900684	0.302809548	-1.278905045	0.111578371
Alas1|11655	-1.130041562	0.222710686	-1.130041562	0.222710686
1500017E21Rik|668215	-1.130066888	0.395422724	-1.020189479	0.48121753
Lpar1|14745	-1.130143443	0.383129466	1.059119282	0.43821345
Ppp1r18|76448	-1.130178541	0.243659805	-1.130178541	0.243659805
Ruvbl2|20174	-1.130241338	0.237985391	-1.071462788	0.346371804
Mon2|67074	-1.130337815	0.251753051	1.096515409	0.215717819
Antxr1|69538	-1.130345595	0.415601497	-1.752038658	0.191487955
Lasp1|16796	-1.130480782	0.139934316	-1.287232195	0.002596342
Capn2|12334	-1.130490125	0.223904224	-1.040372246	0.398920366
Atp13a2|74772	-1.130514872	0.19814597	-1.208952855	0.052928921
Lrp4|228357	-1.130552182	0.208862533	1.04719127	0.323786483
Suds3|71954	-1.130589331	0.166091893	-1.240051446	0.030951579
Tsen34|66078	-1.130623147	0.214952143	-1.20047626	0.028825672
Dicer1|192119	-1.130713897	0.202197283	-1.350738766	0.002303581
Sumf2|67902	-1.130746292	0.223865016	-1.024011557	0.42803851
Jak2|16452	-1.130899384	0.319288304	1.093769784	0.321380968
Gls2|216456	-1.130918135	0.069900744	-1.13649974	0.036734385
Dnajc30|66114	-1.130932334	0.17047947	-1.130932334	0.17047947
Eml1|68519	-1.130986167	0.368186032	1.30872247	0.182853821
Zfyve26|211978	-1.131060466	0.331396481	-1.570035714	0.026315579
Cdkn1c|12577	-1.131210948	0.236877891	-1.131210948	0.236877891
Cdk18|18557	-1.131218402	0.066005687	-1.046601969	0.233840576
Eps8|13860	-1.131229967	0.427901796	-1.644270328	0.270433406
Lman1|70361	-1.131456962	0.044442818	-1.083157072	0.088544579
Wnt2|22413	-1.13147936	0.210724132	-1.211227132	0.095401491
Tmprss6|71753	-1.131571288	0.03268833	-1.178805421	0.005921013
B4galnt1|14421	-1.131575439	0.220041315	-1.131575439	0.220041315
Lrch4-sap25|100316903	-1.131610871	0.169214936	-1.058738541	0.318419768
Samd1|666704	-1.131637643	0.213219738	-1.149769885	0.090044964
C330006K01Rik|231855	-1.131707227	0.120907551	-1.131707227	0.120907551
Ict1|68572	-1.131848388	0.254667868	-1.388194332	0.019480607
Tial1|21843	-1.131951776	0.095305643	-1.246331324	0.006555651
Cxadr|13052	-1.131984789	0.34164743	1.230380257	0.194503668
Dmgdh|74129	-1.132020887	0.178927827	-1.031132766	0.355050903
Ttll1|319953	-1.132067005	0.177671394	-1.056500986	0.331449043
Pias2|17344	-1.132215649	0.280646704	1.128969837	0.199752741
Ankrd10|102334	-1.132271221	0.306580946	-1.265292231	0.188077619
Cabin1|104248	-1.132346499	0.168469077	-1.132346499	0.168469077
Arl6ip1|54208	-1.132400874	0.120602634	-1.270309539	0.004775009
Tbcd|108903	-1.13273972	0.180090626	-1.268095699	0.01813744
Muc6|353328	-1.132764579	0.409235304	1.007102402	0.493733054
Wdr48|67561	-1.132817562	0.261191341	-1.026156017	0.441085841
Pold4|69745	-1.132829847	0.210668731	-1.124005296	0.131947371
Herc3|73998	-1.132836703	0.365930847	-1.252004459	0.24414955
Gpc4|14735	-1.132891744	0.139985176	-1.059605294	0.276526474
Hecw2|329152	-1.132927831	0.398729011	-1.132927831	0.398729011
Drg2|13495	-1.132928995	0.162007103	-1.26593806	0.017390833
1110018J18Rik|66129	-1.132956573	0.347894294	-1.073267078	0.399659848
Mknk2|17347	-1.132981174	0.120697106	-1.034554669	0.348835999
Rnf187|108660	-1.133026941	0.022812895	-1.125015077	0.010125195
Akr1e1|56043	-1.133090505	0.102992686	-1.058586529	0.215383369

Edem2|108687	-1.133129162	0.232852816	-1.040165504	0.382494085
Zadh2|225791	-1.133235519	0.15269846	-1.052086533	0.312089591
Cbx4|12418	-1.133239343	0.28530537	-1.329331267	0.087897154
Ndel1|83431	-1.133388397	0.174259895	-1.174898071	0.136871923
Chd8|67772	-1.133388887	0.049750547	-1.184657505	0.012368138
Mettl3|56335	-1.133404913	0.259434802	-1.260827223	0.079376338
P2rx3|228139	-1.133530261	0.352604036	-1.665530874	0.040568207
Satb1|20230	-1.133804209	0.415712326	-1.133804209	0.415712326
Ift140|106633	-1.133833197	0.321026498	-1.105935377	0.326258901
Zzef1|195018	-1.13383445	0.188664434	-1.028670513	0.400253453
Dhx38|64340	-1.133845093	0.149721806	-1.065401843	0.214685875
Rbm34|52202	-1.133845728	0.279307274	-1.129661636	0.316815431
Robo4|74144	-1.133857089	0.058058133	-1.231665106	0.001786416
Pdcd5|56330	-1.133864656	0.335821537	-1.22850766	0.221966621
Rab11fip5|52055	-1.133932651	0.354784095	-1.178328877	0.236343899
Lypla2|26394	-1.134014952	0.158642212	-1.292138796	0.014282199
Marcksl1|17357	-1.134034798	0.228439274	-1.155911082	0.115653086
Gopc|94221	-1.13403967	0.334122835	1.111648637	0.324407348
Ucp2|22228	-1.134041556	0.25618736	-1.046814205	0.3930023
Arid4a|238247	-1.134076018	0.382385738	1.315153646	0.17988282
Klhl26|234378	-1.134087957	0.175994304	-1.222455321	0.072223854
Ppp2r2a|71978	-1.134106939	0.115665685	-1.063282711	0.236358331
9930021J03Rik|240613	-1.134199039	0.331656342	-1.165743064	0.282376115
Pth1r|19228	-1.134343991	0.300989835	-1.430711876	0.026677631
Ufsp1|70240	-1.134416947	0.346578075	-1.450486524	0.11633724
Zfp39|22698	-1.134436482	0.250258803	-1.114721453	0.170786184
Ddx56|52513	-1.134446893	0.154539733	-1.134446893	0.154539733
Cald1|109624	-1.134453455	0.263693071	1.065567391	0.193706179
Sod2|20656	-1.134455384	0.088347799	-1.187564764	0.038168919
BC013529|215751	-1.134522463	0.25196419	-1.134522463	0.25196419
Tagln|21345	-1.134567987	0.393103596	1.411834307	0.13754153
Lamp1|16783	-1.134576682	0.062135531	-1.234075732	0.002463852
Eif3m|98221	-1.134651232	0.159317055	-1.119644885	0.117312518
Tfg|21787	-1.134658575	0.179585919	-1.034061695	0.372547064
Kcnk13|217826	-1.134700727	0.442200703	-2.496202829	0.207457753
Tfdp2|211586	-1.134701381	0.275741749	-1.167143032	0.18380289
Mapk14|26416	-1.134762495	0.232813641	1.085569863	0.204252517
Brd8|78656	-1.134813166	0.333549781	-1.355632656	0.160976795
Ints12|71793	-1.134857469	0.217269675	-1.244394625	0.054475778
Bbs9|319845	-1.134942183	0.338442441	-1.134942183	0.338442441
Pip4k2c|117150	-1.134951122	0.133948692	-1.13668734	0.114959675
2810407C02Rik|69227	-1.134993435	0.291270311	-1.088930128	0.330290738
E130311K13Rik|329659	-1.135012697	0.273947508	-1.201811947	0.199900593
Cdc5l|71702	-1.135061149	0.317842351	1.181796143	0.205550982
Ndst2|17423	-1.135086548	0.233550711	-1.135086548	0.233550711
Ext2|14043	-1.135149886	0.180104057	-1.135149886	0.180104057
Zfp11|22648	-1.135152291	0.416112499	-1.135152291	0.416112499
Snx30|209131	-1.135282204	0.376661892	-1.589116332	0.122662349
Csde1|229663	-1.135357328	0.027881508	-1.214998647	0.000524543
1700081L11Rik|76719	-1.135374459	0.287652886	-1.135374459	0.287652886
Rnf146|68031	-1.135410794	0.24740996	-1.135410794	0.24740996
Plec|18810	-1.135411049	0.077294351	-1.220003009	0.003784441
Ppp1r13l|333654	-1.135465388	0.298338402	1.007138274	0.485922963

Tbl3|213773	-1.135595993	0.142860807	-1.135595993	0.142860807
Mtx3|382793	-1.135671118	0.368599817	1.257777487	0.184938428
Clic4|29876	-1.135783283	0.321278796	-1.162836788	0.268909974
Actn4|60595	-1.135797823	0.018855934	-1.214593534	2.30107E-05
2610019F03Rik|72148	-1.135832839	0.139687043	-1.135832839	0.139687043
C4a|625018	-1.135845454	0.326526085	-1.359778348	0.145729952
Ramp1|51801	-1.135860868	0.292716833	-1.135860868	0.292716833
Clec4f|51811	-1.135972968	0.221955618	-1.135972968	0.221955618
Ctage5|217615	-1.13598336	0.204133724	-1.039880344	0.382224779
Dgcr8|94223	-1.136004942	0.338451015	-1.203766156	0.245837124
Zfp213|449521	-1.136013073	0.278507212	1.127311041	0.197296125
Alkbh4|72041	-1.136014392	0.203962351	-1.108755922	0.216000747
Trip4|56404	-1.136139703	0.147567084	-1.318411841	0.001289949
Acvr2a|11480	-1.13619607	0.348586936	1.263451001	0.183831868
Amotl2|56332	-1.136391865	0.305742077	-1.312231623	0.150575008
Os9|216440	-1.13646632	0.046684819	-1.100268224	0.094214127
Lace1|215951	-1.136504701	0.186621635	-1.136504701	0.186621635
Tmem175|72392	-1.136515943	0.182054503	-1.320335468	0.006694627
Cd47|16423	-1.136564639	0.24176416	-1.256388617	0.113305126
Fbp1|14121	-1.136578773	0.154473216	-1.136578773	0.154473216
Mprip|26936	-1.136697182	0.163408163	-1.230984955	0.025855795
Caprin1|53872	-1.136731466	0.26586932	1.049807569	0.343544053
N6amt1|67768	-1.136752801	0.265344682	1.106439808	0.24323116
Rnf5|54197	-1.136771281	0.03074469	-1.136771281	0.03074469
Ube3c|100763	-1.136783184	0.188719667	-1.346338388	0.006334173
Dnttip2|99480	-1.13678519	0.1728709	-1.334893702	0.001425654
Pcsk5|18552	-1.136819893	0.271293872	-1.01089869	0.475815053
Rnf169|108937	-1.13682626	0.160413894	-1.13682626	0.160413894
Rint1|72772	-1.13693711	0.221004285	-1.39921739	0.002459546
Setd5|72895	-1.136949665	0.262038377	-1.083249257	0.310666388
Srp72|66661	-1.136983619	0.217626623	-1.227359358	0.120246846
2500003M10Rik|66511	-1.137077856	0.085509541	-1.027550144	0.331851539
Fkbp15|338355	-1.137098494	0.129058564	-1.067204218	0.259274221
Cd151|12476	-1.137141601	0.117243566	-1.137141601	0.117243566
Dhx34|71723	-1.13721451	0.201669412	-1.327683762	0.021208375
BC089597|216454	-1.137218912	0.108979007	-1.286271223	0.001248067
Bmp1|12153	-1.137238064	0.057094132	-1.14071815	0.015515601
C130039O16Rik|238317	-1.137248371	0.384226268	1.035228152	0.431137784
Plscr3|70310	-1.137537784	0.174806103	-1.137537784	0.174806103
Herc4|67345	-1.137565625	0.300803871	-1.167315669	0.204743749
Cst3|13010	-1.137571622	0.174194932	-1.350184009	0.000998549
Cyp2j6|13110	-1.137719803	0.168259618	-1.137719803	0.168259618
C330007P06Rik|77644	-1.137886397	0.369379614	-1.247662714	0.236442985
Hbxip|68576	-1.137955079	0.231935362	-1.137955079	0.231935362
Cdh5|12562	-1.137967664	0.174840879	-1.253438053	0.040897929
1810049H13Rik|66431	-1.138037118	0.21183949	-1.241595292	0.062348279
Mgst1|56615	-1.138282212	0.26965	-1.138282212	0.26965
Nars|70223	-1.138329394	0.222475839	-1.138329394	0.222475839
Faf2|76577	-1.13841892	0.071239373	-1.141479757	0.036816688
Ces1c|13884	-1.138443306	0.184058995	-1.338958534	0.005307786
Snx2|67804	-1.138560941	0.230954597	-1.138560941	0.230954597
Prr13|66151	-1.138626994	0.077329067	-1.076464273	0.157970374
Spna2|20740	-1.138686237	0.180734699	-1.20338617	0.055571186

Creg1|433375	-1.138699504	0.058497032	-1.160213469	0.003809097
Snx15|69024	-1.138753953	0.171167369	-1.197507558	0.095241481
Mnd1|76915	-1.138773058	0.389966684	1.041609332	0.460637096
Plekha7|233765	-1.138872749	0.332723898	1.051513129	0.415366679
Scap|235623	-1.138936319	0.141076862	-1.176408607	0.039828773
Tmem108|81907	-1.138951612	0.397517401	1.206106086	0.360232862
Dgcr2|13356	-1.138972115	0.040957329	-1.154258796	0.006724435
Slc26a6|171429	-1.138994658	0.364268792	-1.138994658	0.364268792
Unk|217331	-1.139016564	0.18893093	1.022761231	0.422264164
Mrps15|66407	-1.139030439	0.186826937	-1.051334367	0.347004821
Lrrc56|70552	-1.139080883	0.286235627	-1.159133865	0.181514106
Nipa1|233280	-1.139122715	0.449250775	1	#DIV/0!
Chn2|69993	-1.139156463	0.110172277	-1.217995702	0.011102694
Gpr19|14760	-1.139331363	0.336674517	1.028612008	0.464411275
Rad23b|19359	-1.139349768	0.270835945	-1.265772955	0.149848559
Tymp|72962	-1.139359998	0.118288414	-1.112948743	0.081479642
Pacsin3|80708	-1.139409999	0.120396599	-1.139409999	0.120396599
Add3|27360	-1.13946068	0.330871943	1.051589876	0.413918428
Clk3|102414	-1.13955587	0.028013517	-1.126017594	0.004455933
Derl3|70377	-1.139639724	0.412234677	1.540420252	0.205546211
Fbxl12|30843	-1.139761104	0.259561324	-1.146601891	0.208542528
Cdv3|321022	-1.139781128	0.032323367	-1.143695355	0.010437247
Gramd1b|235283	-1.139872053	0.356717025	1.048286062	0.438896362
Fam38a|234839	-1.139907233	0.314705659	-1.139907233	0.314705659
Fut8|53618	-1.13992178	0.326246048	-1.09385036	0.284946527
Plagl2|54711	-1.139940726	0.291000666	-1.385071792	0.063855255
Lysmd3|80289	-1.140210788	0.396383829	1.566306725	0.136377964
Kat2a|14534	-1.140385264	0.155588335	-1.210378458	0.081839968
Cdk20|105278	-1.140417439	0.213205444	1.000904151	0.496603461
Zfp748|212276	-1.140505828	0.376119548	-1.775545616	0.048946904
Ehd1|13660	-1.140552534	0.13277586	-1.227470992	0.016291718
Zfp617|170938	-1.140568581	0.312097831	1.051010115	0.417171428
Ednra|13617	-1.140626544	0.315256149	-1.309420458	0.125081933
Nmt1|18107	-1.140680744	0.125872418	-1.025289167	0.38664493
Cobll1|319876	-1.140713966	0.365933938	1.227555901	0.242718978
Neo1|18007	-1.140750078	0.240752249	-1.140750078	0.240752249
Zfp12|231866	-1.140794759	0.299879454	-1.318240157	0.145665051
Ccnk|12454	-1.14082213	0.160353379	-1.067674932	0.30090779
Creb3l2|208647	-1.140913711	0.293117826	1.153042304	0.197781944
Snx4|69150	-1.140920587	0.230211707	-1.140920587	0.230211707
Ascc3|77987	-1.141003448	0.370346986	-1.471901734	0.183331372
Mob3c|100465	-1.141018611	0.206321021	-1.008072812	0.471016644
Zbtb44|235132	-1.141114066	0.239707803	-1.080099274	0.343242268
Dab2ip|69601	-1.141296449	0.230729621	-1.243302741	0.093033739
Parp6|67287	-1.141393929	0.219523713	-1.191263034	0.178016689
Prrc2b|227723	-1.141472975	0.184230462	-1.014996824	0.435373717
Daam1|208846	-1.141553059	0.290636539	1.021979492	0.430452412
Mib2|76580	-1.141554356	0.135188963	-1.239229829	0.029473841
Harbi1|241547	-1.141557987	0.187351622	-1.086259438	0.29798126
Sdc2|15529	-1.141564122	0.054619655	-1.141564122	0.054619655
Sh3bp4|98402	-1.141568873	0.381726151	-1.710439162	0.102883955
Pmepa1|65112	-1.141587214	0.346818747	-1.617651277	0.032637354
Tstd1|226654	-1.141623219	0.335910819	-1.530611218	0.054929859

Mup3|17842	-1.141654677	0.290551453	-1.016486525	0.469909517
Rxrb|20182	-1.141718617	0.102648269	-1.263815556	0.000607395
Eif4g1|208643	-1.142025136	0.107225448	-1.219907386	0.01212509
Cryab|12955	-1.142463382	0.243029613	-1.300765861	0.078616841
Rhbdd2|215160	-1.142574976	0.143723157	-1.228837359	0.050998899
Cdk8|264064	-1.142630116	0.178937195	1.026987645	0.395632546
Flot2|14252	-1.142685233	0.033624406	-1.149294944	0.001583992
Ptcd2|68927	-1.142747416	0.192757352	-1.250372309	0.075204349
Tlk1|228012	-1.142841931	0.274365503	-1.319011337	0.107513527
Stk11ip|71728	-1.142885738	0.24671177	-1.142885738	0.24671177
Lama5|16776	-1.142890685	0.339606624	1.086698897	0.390104068
Arl4c|320982	-1.142892769	0.31976724	-1.315738595	0.132380239
Trip10|106628	-1.142903005	0.155782167	-1.142903005	0.155782167
Ap3b1|11774	-1.142947672	0.229491283	1.058978882	0.339951412
Mup4|17843	-1.142993497	0.335173381	-1.142993497	0.335173381
Fxyd5|18301	-1.143004711	0.33343068	-1.208470295	0.135523872
Usp21|30941	-1.143224492	0.251200073	1.065722486	0.342274469
Ercc5|22592	-1.143232436	0.239356033	-1.092277851	0.277407316
Trub1|72133	-1.143400717	0.22079009	-1.26556991	0.049698401
Zcwpw1|381678	-1.14344808	0.242482606	1.075230772	0.308714907
Gldc|104174	-1.143463079	0.087179321	-1.210788187	0.026445302
Stau1|20853	-1.143477913	0.128352312	-1.111699134	0.111728869
Cox8a|12868	-1.143501496	0.160641724	-1.155467148	0.023322134
Edem1|192193	-1.143638407	0.130346612	-1.214204026	0.05891254
Reep2|225362	-1.143658515	0.423179243	-1.388175758	0.348733614
Zfp362|230761	-1.143789153	0.227718972	1.088858898	0.19298302
Cops7a|26894	-1.143871356	0.06999339	-1.198166329	0.027261198
Zfp719|210105	-1.143955518	0.305903155	1.043258344	0.411875818
Ahnak|66395	-1.143958384	0.296065052	1.02518847	0.448162695
3110001D03Rik|66928	-1.144035943	0.23735536	-1.097382393	0.153065078
Spns2|216892	-1.144054399	0.197551291	-1.069175644	0.328930045
Dhx15|13204	-1.144059018	0.372788889	1.218797108	0.29202777
Tmem126b|68472	-1.144100593	0.251300083	-1.281697775	0.067207804
Eci1|13177	-1.144148932	0.110125136	-1.193258177	0.067812473
Mdh2|17448	-1.144155083	0.120681014	-1.19746712	0.072325862
Hat1|107435	-1.144192351	0.207015357	-1.182200401	0.088317863
Ddx20|53975	-1.144222915	0.289099703	-1.282910264	0.12610188
Rnf181|66510	-1.144341223	0.074966186	-1.13762249	0.041353942
Man2b1|17159	-1.144356982	0.101480992	-1.144356982	0.101480992
Secisbp2l|70354	-1.144447257	0.387821351	1.354448678	0.209879511
Btla|208154	-1.144506226	0.40952252	-1.744207205	0.204556877
Smg6|103677	-1.144509748	0.061667771	-1.152912406	0.025950902
Dnajb12|56709	-1.14456848	0.230409748	-1.14456848	0.230409748
Anapc2|99152	-1.144686246	0.092410776	-1.073827767	0.190377298
Galnt2|108148	-1.144759868	0.107653707	-1.091177086	0.199637125
Mpi|110119	-1.144777931	0.135447941	-1.187096385	0.090640808
Prr5|109270	-1.144819067	0.129205382	-1.217967285	0.055503714
Gm5801|545056	-1.144827366	0.216339239	-1.144827366	0.216339239
Csf2ra|12982	-1.144852634	0.183380067	1.028282128	0.392904514
Bmper|73230	-1.144865632	0.364954544	1.147114806	0.355397339
Zfp597|71063	-1.144873458	0.377187305	1.37991754	0.196734113
Gucy1b3|54195	-1.144890383	0.345397087	-1.144890383	0.345397087
Wrnip1|78903	-1.144944016	0.305636627	-1.239920753	0.171477914

LOC100048885|100048885	-1.145049105	0.308750643	1.028810918	0.447064484
Wdr73|71968	-1.145139514	0.188216301	-1.045663913	0.370544019
Brpf1|78783	-1.145177851	0.287692563	-1.145177851	0.287692563
Agphd1|235386	-1.145202691	0.14774681	-1.209772931	0.017186641
Sec14l1|74136	-1.145273336	0.139210735	-1.317692475	0.005200871
Slc25a42|73095	-1.145306871	0.179663175	-1.219015416	0.106133929
Arhgef15|442801	-1.145429696	0.110213413	-1.090114463	0.109995281
Lrrc4c|241568	-1.145492174	0.38160925	1.037918822	0.464623131
Triobp|110253	-1.145503714	0.140511326	-1.004119465	0.482316056
Dgka|13139	-1.145546634	0.247674085	-1.040273236	0.413873265
Zfp507|668501	-1.145884764	0.373993639	-1.000287516	0.49960416
Akap10|56697	-1.14589903	0.228114245	1.057886726	0.34439983
Jak1|16451	-1.145908976	0.184119564	-1.145908976	0.184119564
Mfsd1|66868	-1.145970412	0.192854234	-1.145970412	0.192854234
Elavl1|15568	-1.146003141	0.18094289	-1.226380314	0.074793123
Rbck1|24105	-1.14603018	0.079534762	-1.029601669	0.315653904
Zcchc7|319885	-1.146087134	0.077051889	-1.103308073	0.145262518
Ccdc17|622665	-1.146204844	0.330758665	1.286104704	0.080442928
8430427H17Rik|329540	-1.146487924	0.363928704	-1.369979024	0.177288338
Pcgf3|69587	-1.146548297	0.33627721	1.257463855	0.173022365
1700034H15Rik|98736	-1.146708542	0.41153122	-1.119758182	0.433120414
Atad3a|108888	-1.146714778	0.044320466	-1.132128124	0.021137268
Prmt6|99890	-1.146719367	0.269224257	-1.482388997	0.015656614
Ssh1|231637	-1.146735065	0.261476003	-1.242569371	0.133274251
Zfp524|66056	-1.146792524	0.233748104	-1.40664698	0.017966376
Plekhj1|78670	-1.146922707	0.212229521	-1.277862593	0.075376998
Ppib|19035	-1.146955288	0.284014477	1.027940229	0.446549547
Tbck|271981	-1.147036728	0.410030439	1.33785939	0.324957439
Kcnk5|16529	-1.147297427	0.221067818	-1.210730256	0.105790385
Rasa3|19414	-1.14737974	0.330483185	-1.461681265	0.107014632
Tpt1p|497210	-1.147429297	0.385506004	-1.657770148	0.156327277
Fam122b|78755	-1.147535211	0.425886284	1.58181987	0.137083841
Mrps7|50529	-1.147571427	0.063690111	-1.166955444	0.008849942
Spry4|24066	-1.147585922	0.363193167	1.379181862	0.136127649
Pus10|74467	-1.147821591	0.209233263	-1.17340694	0.145183981
Abhd11|68758	-1.147904438	0.215869262	-1.260249417	0.102581289
Wtap|60532	-1.147973178	0.300417332	1.048418759	0.416809051
Tmx3|67988	-1.148045003	0.324338743	-1.148045003	0.324338743
Csnk1a1|93687	-1.148062149	0.32251052	1.076264604	0.389635015
Katnb1|74187	-1.148063547	0.371098573	1.041995671	0.456854218
Coq6|217707	-1.148066968	0.216776687	-1.052946311	0.381851722
9530082P21Rik|638247	-1.148089596	0.331458462	-1.503802735	0.082092828
Rbm5|83486	-1.14821891	0.178841764	-1.118295049	0.169462906
Dlg3|53310	-1.148227632	0.141172977	-1.148227632	0.141172977
Tgfbr1|21812	-1.148376851	0.302530432	-1.510545982	0.02392281
Zfp598|213753	-1.14839441	0.039532492	-1.188992013	0.01880901
Aspg|104816	-1.148431978	0.059290595	-1.197471253	0.026192839
5430437P03Rik|68251	-1.148447604	0.066403367	-1.148447604	0.066403367
Wbp2|22378	-1.148461016	0.031260269	-1.070325974	0.105625463
Mecp2|17257	-1.148500503	0.106628554	-1.196391691	0.068220576
Zfp346|26919	-1.148554767	0.308164918	-1.077344851	0.343082257
Atrx|22589	-1.148567992	0.366292972	1.151955722	0.286911578
Cd164|53599	-1.14863849	0.278827098	-1.131609241	0.224913139

Crlf3|54394	-1.148684142	0.346241112	1.100234491	0.385798037
Man2b2|17160	-1.148845153	0.06665946	-1.276320374	0.000877188
Setd2|235626	-1.148854233	0.265113199	-1.148854233	0.265113199
Ltbp4|108075	-1.148886506	0.120878183	-1.309528162	0.00498277
Ahdc1|230793	-1.148890113	0.211615044	-1.032353572	0.416587824
Drosha|14000	-1.149022245	0.158338794	1.037908715	0.199533928
Ikbkg|16151	-1.149056042	0.267932666	1.035849867	0.41554003
Dclre1a|55947	-1.149108179	0.250665419	-1.005380537	0.486419555
1810014B01Rik|66263	-1.149155354	0.293355391	-1.296633228	0.066715438
AW146020|330361	-1.149219239	0.415262063	-1.885842894	0.201181342
Adh4|26876	-1.149221319	0.244466649	-1.291637149	0.107577684
Rap1gds1|229877	-1.149246367	0.204137495	1.03969265	0.371489325
2810428I15Rik|66462	-1.149333352	0.283223097	-1.383163296	0.078700781
Selenbp1|20341	-1.149369108	0.13215758	-1.11843064	0.120608643
Telo2|71718	-1.149401141	0.278510783	-1.149401141	0.278510783
Ctcf|13018	-1.149412836	0.273382554	-1.170335837	0.229013589
Agtr1a|11607	-1.149413176	0.286199305	-1.340800218	0.122554291
Gps2|56310	-1.149460585	0.229338385	-1.252792226	0.131230851
Dpp9|224897	-1.149478356	0.019686978	-1.194705793	0.001552019
Nfe2l2|18024	-1.149610195	0.191528054	-1.278096308	0.057343859
Sort1|20661	-1.14962437	0.323906138	-1.14962437	0.323906138
4930455C21Rik|76916	-1.149628668	0.208506957	-1.2632029	0.094096176
Mup9|100038948	-1.14965703	0.387753062	-1.14965703	0.387753062
Ghdc|80860	-1.149698889	0.11179714	-1.149698889	0.11179714
Gcsh|68133	-1.149741379	0.053731559	-1.119295834	0.100135838
Frk|14302	-1.149754461	0.308880418	-1.149754461	0.308880418
Tmem143|70209	-1.149783691	0.133846529	-1.067743449	0.273148702
Dkk3|50781	-1.14978411	0.358203135	-1.285947424	0.246566367
Taf2|319944	-1.149797312	0.259736004	-1.019067715	0.463358302
Spint1|20732	-1.149823433	0.362841143	-1.149823433	0.362841143
Mmgt1|236792	-1.14988955	0.155434303	-1.14988955	0.155434303
Dopey2|70028	-1.149901327	0.124361634	-1.23507885	0.040953128
Pkp4|227937	-1.149916917	0.059351838	-1.149916917	0.059351838
Fam135a|68187	-1.149993649	0.353924658	1.097739454	0.399435267
Cadm3|94332	-1.150018035	0.21204751	-1.150018035	0.21204751
Dusp14|56405	-1.150062762	0.389395094	1.218096211	0.348431201
Agbl5|231093	-1.150153124	0.299611528	-1.087792983	0.29487494
C1s|50908	-1.150182813	0.107209311	-1.150182813	0.107209311
Tmem184b|223693	-1.150208155	0.112561588	-1.081219606	0.22606109
Pi4k2a|84095	-1.15021615	0.09284927	-1.15021615	0.09284927
Vapb|56491	-1.150253521	0.004897886	-1.209467798	0.00017856
41167|93684	-1.150315321	0.098326263	-1.158487869	0.025637274
Slc5a1|20537	-1.150386258	0.402282081	-1.900662917	0.146212747
Cd79b|15985	-1.150397222	0.306410896	1.039921733	0.438316835
Jup|16480	-1.150419772	0.103359746	-1.103262744	0.184256567
Polr3k|67005	-1.150419914	0.179453716	-1.060058307	0.33184477
Akna|100182	-1.150430117	0.334321318	-1.163492475	0.316885893
Wdr67|210544	-1.150502429	0.33268739	-1.150502429	0.33268739
Fundc1|72018	-1.150582346	0.301523931	-1.171429784	0.196543705
Dram2|67171	-1.150678347	0.159671926	-1.06502352	0.311408578
Sec31a|69162	-1.150715722	0.066692578	-1.197964708	0.034609586
Cdc34|216150	-1.150758297	0.181887912	-1.26842636	0.060245905
Hiat1|15247	-1.150760989	0.306764126	1.213560973	0.132185143

Rabggta|56187	-1.150781359	0.077259359	-1.150781359	0.077259359
Thap11|59016	-1.150786864	0.180699614	-1.107605002	0.268462147
Whrn|73750	-1.150892248	0.386689887	-1.690303969	0.15651159
Slc25a27|74011	-1.150906331	0.355395721	-1.50936067	0.147740833
Irs2|384783	-1.150910357	0.396671394	-1.422814123	0.285911479
Kbtbd10|228003	-1.151057271	0.389850522	-1.171381302	0.374052799
Rab18|19330	-1.151193892	0.33337235	1.094967272	0.37757938
Unc93a|381058	-1.151194528	0.307051885	-1.151194528	0.307051885
Dgcr14|27886	-1.151418452	0.219816797	-1.069252986	0.351803439
Clcn3|12725	-1.151436497	0.313680442	-1.151436497	0.313680442
Rhbdd1|76867	-1.151494507	0.305723109	-1.062763785	0.399372141
Stk25|59041	-1.151658658	0.123472941	-1.151658658	0.123472941
Atl3|109168	-1.151676963	0.33176992	1.064605293	0.332012213
Thap2|66816	-1.151707494	0.317231276	-1.151707494	0.317231276
Mbtps1|56453	-1.15172631	0.083229891	-1.228906737	0.019079866
1700052N19Rik|73419	-1.151819338	0.271819237	-1.234959288	0.051277233
Atf5|107503	-1.151901488	0.041231471	-1.056402161	0.13112791
Cdk5rap1|66971	-1.151958295	0.342233555	-1.217230767	0.199081259
Slc27a3|26568	-1.151959663	0.388317052	-1.151959663	0.388317052
Hp1bp3|15441	-1.151987459	0.189349704	-1.151987459	0.189349704
Socs2|216233	-1.152021495	0.253251778	1.099619841	0.264742955
Smn1|20595	-1.152084917	0.225030251	-1.033612164	0.423433986
Arhgdib|11857	-1.152209652	0.206987602	-1.221248005	0.069185417
Pex10|668173	-1.152271972	0.282805963	-1.507194248	0.03444451
Avl9|78937	-1.15235039	0.373866968	-1.15235039	0.373866968
1700088E04Rik|27660	-1.152362425	0.434776335	2.057220559	0.233247321
Stard8|236920	-1.152387308	0.237687063	-1.080379261	0.349600214
Apol9a|223672	-1.15239813	0.318555646	-1.342565522	0.04711098
Agt|11606	-1.152413358	0.188457832	-1.152413358	0.188457832
Usp45|77593	-1.152531908	0.359040437	1.088475023	0.41623425
Mus81|71711	-1.152612899	0.222678324	-1.297970689	0.079750569
Mrc1|17533	-1.152696012	0.322874964	-1.093665351	0.375518123
Dyrk1b|13549	-1.152842903	0.165555322	-1.279193606	0.036768298
Serpinb9|20723	-1.152945451	0.379516421	-1.140071799	0.37638299
Lrrc14b|432779	-1.153002925	0.377471067	-1.562477748	0.143283389
Fbxl8|50788	-1.153009593	0.292238956	-1.358307991	0.082787208
Ppfibp1|67533	-1.153041831	0.340673405	1.128250554	0.342066797
Cldn5|12741	-1.153046895	0.169491619	-1.082145295	0.229962103
Fbxl3|50789	-1.153066402	0.357840272	1.16422311	0.331617921
Fntb|110606	-1.153147535	0.270333433	1.005206343	0.488423955
Ets1|23871	-1.153185913	0.338495527	-1.096931088	0.387272839
Gng5|14707	-1.153190304	0.182230693	-1.063042415	0.339858528
Fgfr2|14183	-1.15319062	0.29160593	1.056350758	0.394006749
Abhd3|106861	-1.153206114	0.251359894	1.093299548	0.28161473
Gal3st4|330217	-1.153208457	0.381579194	-1.364037215	0.240311046
Ar|11835	-1.153327842	0.428102672	-1.189393939	0.435388966
Dnpep|13437	-1.153448789	0.19244842	1.00971019	0.470737235
Tet2|214133	-1.153480849	0.336136294	1.022313039	0.457477749
Hk2|15277	-1.153494442	0.301304945	-1.283604224	0.171978873
Dnajc22|72778	-1.153536707	0.182388884	-1.170346291	0.100155869
Ndufv3|78330	-1.153655261	0.261597225	-1.163265252	0.160643208
Ift57|73916	-1.153747837	0.368372057	1.031484346	0.468331887
Sirt3|64384	-1.153914723	0.046537817	-1.061626441	0.163857255

Sdha|66945	-1.153944311	0.100533321	-1.153944311	0.100533321
Ireb2|64602	-1.153976412	0.308535755	-1.153976412	0.308535755
Ndufa10|67273	-1.153988254	0.069006991	-1.153988254	0.069006991
Sh3pxd2a|14218	-1.154001035	0.282545241	-1.152996237	0.279768153
Ropn1l|252967	-1.15400662	0.307853495	-1.505819838	0.044426904
Bzrap1|207777	-1.154013868	0.441341423	-2.019707773	0.301117079
Luc7l3|67684	-1.154052116	0.107757902	-1.154052116	0.107757902
Necap1|67602	-1.154069772	0.175399981	-1.258748868	0.03885567
Snf8|27681	-1.154090198	0.22956807	-1.154090198	0.22956807
Flot1|14251	-1.154110322	0.175949241	-1.061802667	0.336331189
Dedd|21945	-1.154134975	0.164413406	-1.292327787	0.025906494
Pcdh12|53601	-1.154211354	0.204083036	-1.154211354	0.204083036
Aggf1|66549	-1.154272522	0.24090962	-1.154272522	0.24090962
B2m|12010	-1.154324232	0.045830851	-1.154324232	0.045830851
Hps6|20170	-1.154340639	0.180625045	-1.278957248	0.021400426
Zdhhc18|503610	-1.154524287	0.106686066	-1.071597769	0.223634509
Actr1a|54130	-1.154548761	0.007382571	-1.154548761	0.007382571
Fmnl3|22379	-1.154549738	0.213000424	-1.25133017	0.093432568
Pcsk4|18551	-1.154550167	0.118294789	-1.068745944	0.148691555
Camta2|216874	-1.154582237	0.173356415	-1.253220846	0.046094068
Exd2|97827	-1.154607072	0.325485074	-1.214334731	0.241237746
Thap1|73754	-1.154854219	0.147349403	-1.213296142	0.098008094
Zfp512|269639	-1.154896193	0.162198037	-1.347427386	0.011727475
Enpp1|18605	-1.154907893	0.055017282	-1.198853969	0.015410468
Flii|14248	-1.154936791	0.10038775	-1.154936791	0.10038775
Myd88|17874	-1.154953812	0.169131474	-1.154953812	0.169131474
Tm9sf1|74140	-1.155062637	0.101963897	-1.100668978	0.188472594
Rbm43|71684	-1.155227084	0.288725621	-1.143265429	0.258167098
Tmem14c|66154	-1.155250611	0.076277397	-1.195712905	0.051682336
Ergic1|67458	-1.155292699	0.070739495	-1.04349732	0.22095551
Usp48|170707	-1.155314069	0.104975137	-1.098284189	0.195194948
Dcun1d2|102323	-1.155336242	0.236734684	-1.067010737	0.377815348
Carm1|59035	-1.155573994	0.097071889	-1.216153709	0.033553322
Gm5512|433224	-1.155618211	0.201834671	-1.025112354	0.428055632
Cpn1|93721	-1.155659732	0.141327476	-1.126787965	0.132269294
Gm17762|100034739	-1.155818633	0.41765293	-2.020751923	0.197627689
Ccdc61|232933	-1.156088849	0.231764048	-1.1991222	0.084683117
Zfp697|242109	-1.156198406	0.285574897	1.168744833	0.171580818
Map3k11|26403	-1.156208424	0.083597074	-1.156208424	0.083597074
Ufsp2|192169	-1.156233248	0.067786057	-1.178494911	0.018104208
Mthfs|107885	-1.156342983	0.192805055	-1.251202479	0.103919817
Ppme1|72590	-1.156358863	0.111054587	-1.252005988	0.024596266
Golga5|27277	-1.156465213	0.269862157	-1.268343374	0.148813051
Gtf2a1|83602	-1.15654792	0.27610466	-1.067163075	0.370345492
Gba2|230101	-1.156559519	0.197281888	-1.253147284	0.077002329
Dixdc1|330938	-1.156578675	0.287845695	-1.557913559	0.012907056
Rnf19a|30945	-1.156611936	0.255513101	-1.207515346	0.157861709
Hsd17b12|56348	-1.156639466	0.162416715	-1.156639466	0.162416715
Tatdn2|381801	-1.156758951	0.187101013	-1.059335379	0.352702144
Ehd4|98878	-1.156807048	0.255146063	-1.225680359	0.12721337
Mon1a|72825	-1.156811833	0.15657399	-1.288012155	0.028151199
Recql4|79456	-1.156964545	0.346144377	-1.698733919	0.038804519
Cyb5|109672	-1.156989268	0.16738572	-1.035927257	0.372212381

Ccdc30|73332	-1.156989838	0.300916966	-1.584407703	0.030780199
Adra1b|11548	-1.157013838	0.289378137	-1.466330898	0.044241442
Ugt3a1|105887	-1.157018483	0.073817829	-1.157018483	0.073817829
Mbl1|17194	-1.157055027	0.151285215	-1.15197541	0.122913968
Nr2c2ap|75692	-1.157081787	0.244275859	-1.447670135	0.02156677
Arsk|77041	-1.157125944	0.350859413	1.168807535	0.314147864
Rdh11|17252	-1.157152732	0.225176611	1.06822327	0.311702208
D930016D06Rik|100662	-1.157211457	0.293068189	-1.258227714	0.175325502
Ezh2|14056	-1.157261371	0.400302532	-2.302802941	0.060851966
Ppp1r2|66849	-1.157282198	0.212763651	-1.435589697	0.007820195
Lrrc28|67867	-1.157296115	0.088234102	-1.118382707	0.158113301
Ifih1|71586	-1.157386631	0.259834085	-1.024217375	0.456434623
Pramef8|242736	-1.157402776	0.351723087	-1.585391943	0.11310171
Tbxas1|21391	-1.157537265	0.30540383	1.030736631	0.455410479
Vps16|80743	-1.157540181	0.200993209	-1.053779754	0.365518623
Ccnl2|56036	-1.157548651	0.160224533	-1.000534329	0.498183129
Ralgps2|78255	-1.157613995	0.232037961	-1.447185555	0.017676718
Hdac6|15185	-1.157671871	0.125034306	-1.077559282	0.246692424
Micu1|216001	-1.157709755	0.083132114	-1.243069799	0.015559906
Ankib1|70797	-1.157753449	0.310957041	-1.157753449	0.310957041
Sirt7|209011	-1.157774575	0.100223452	-1.104215431	0.189495135
Pla2g12b|69836	-1.157863253	0.141324812	-1.256454794	0.02107216
Frmd8|67457	-1.157879241	0.115674126	-1.172380704	0.04704641
Ddo|70503	-1.15789615	0.151848991	-1.06075591	0.312326823
Abcf2|27407	-1.158211406	0.076874893	-1.205936448	0.03511356
Gpr182|11536	-1.158267202	0.087877834	-1.172615127	0.018300789
Nmnat1|66454	-1.15830615	0.164861088	-1.272517585	0.021000442
Pskh1|244631	-1.158398739	0.156049159	1.006397025	0.475944697
Cnot2|72068	-1.158438298	0.233633774	-1.031649304	0.43383436
Vwa5a|67776	-1.158453501	0.361307058	1.183911062	0.325193585
Polr3c|74414	-1.158472271	0.109705271	-1.067941959	0.231580983
Mup19|100189605	-1.15849334	0.392159015	1.259537922	0.337430724
Ttyh2|117160	-1.158544542	0.120739621	-1.201446214	0.017743354
Mccc2|78038	-1.158607503	0.063035678	-1.104864404	0.127021835
Wnk2|75607	-1.158645939	0.36898372	-1.158645939	0.36898372
Bzw1|66882	-1.158658786	0.304377692	-1.158658786	0.304377692
D2Wsu81e|227695	-1.158668232	0.143533506	-1.007103423	0.472614229
D9Ertd402e|382117	-1.158734238	0.196479075	-1.329595433	0.031804499
Tmx2|66958	-1.158777736	0.14878023	-1.086480266	0.265806647
Was|22376	-1.158783296	0.335955025	-1.510121087	0.118813162
Sirt2|64383	-1.158850323	0.016306624	-1.182099038	0.013293461
Txndc9|98258	-1.159251369	0.076439728	-1.196355515	0.004958134
Cc2d1a|212139	-1.159259527	0.13691959	-1.063497971	0.287528397
Dmd|13405	-1.159416099	0.339478435	1.140283798	0.330318536
Naif1|71254	-1.159426837	0.381121265	-1.182022492	0.376173231
Gorasp2|70231	-1.159497581	0.124440573	-1.1391568	0.058204293
D2hgdh|98314	-1.159563724	0.24149401	-1.021200854	0.456272653
Gbp4|17472	-1.159578942	0.362360465	1.16622302	0.348505043
Stat6|20852	-1.159672194	0.171318441	-1.159672194	0.171318441
Pter|19212	-1.159736104	0.185967672	-1.153312723	0.141254124
Rpn2|20014	-1.15984994	0.068676771	-1.050871426	0.258095903
Gtpbp3|70359	-1.159896869	0.098178656	-1.090795731	0.194715911
Fbxl17|50758	-1.160225849	0.262136342	1.031815718	0.428249405

Rapgef1|107746	-1.160241892	0.109848292	-1.11497409	0.064260714
Cd81|12520	-1.160261032	0.03189257	-1.22390988	0.004007345
Dhrs11|192970	-1.160305418	0.241205297	-1.008966668	0.477475069
Tram1|72265	-1.160311231	0.080761176	-1.239565507	0.020022416
Ttc36|192653	-1.160363473	0.229034143	-1.471221704	0.004601434
Nono|53610	-1.160400635	0.157699761	-1.25848824	0.037113827
9330151L19Rik|414085	-1.160498307	0.281247236	-1.018694852	0.47116695
Tcf4|21413	-1.160530002	0.329715414	1.084788043	0.329718338
Cadps2|320405	-1.160556225	0.121089798	-1.327238502	0.008319559
Slc15a3|65221	-1.160582435	0.205274671	-1.296251354	0.07795741
Mcat|223722	-1.160605552	0.119726173	-1.225148537	0.029415073
Zxda|668171	-1.160692669	0.38880657	-1.916416447	0.048242189
Cyp2a4|13086	-1.160694948	0.233972999	-1.160694948	0.233972999
Ktn1|16709	-1.160804131	0.311708055	1.036146052	0.45115082
Aldh1l1|107747	-1.160856343	0.003251731	-1.151226352	0.000875218
Oaz2-ps|18247	-1.160901373	0.069741471	-1.09759132	0.142620115
Pqlc1|66943	-1.160937861	0.058265169	-1.041511571	0.179125373
Slc23a2|54338	-1.161055632	0.301744175	-1.288792141	0.213971809
AW554918|225289	-1.161060909	0.369812995	-1.694687877	0.125366495
Crtc2|74343	-1.161066461	0.064560267	-1.291012507	0.000155399
Bcl2l13|94044	-1.161195249	0.224312902	-1.126166568	0.198876432
Ttc7|225049	-1.161276548	0.061845723	-1.05760204	0.230979596
D4Wsu53e|27981	-1.16136155	0.30042237	-1.400085309	0.125990384
Upp2|76654	-1.161395111	0.248614574	-1.002242882	0.495223403
Naa25|231713	-1.161404552	0.179538897	-1.208588867	0.087968827
Apoa1bp|246703	-1.161411324	0.07097116	-1.161411324	0.07097116
Ptprcap|19265	-1.161597443	0.24379325	-1.052195803	0.407390071
Dse|212898	-1.161715791	0.304584978	1.023259174	0.467436168
Bicd2|76895	-1.161719416	0.254078428	-1.095856696	0.301337145
Sfrp1|20377	-1.161797137	0.379382084	-1.656314914	0.17186137
Mlec|109154	-1.161846894	0.085740115	-1.268335751	0.005986173
Zfp248|72720	-1.16189407	0.403007224	1.927201325	0.068141101
Tpx2|72119	-1.161971376	0.426260985	-2.696597471	0.113604984
Ugp2|216558	-1.162157651	0.102510097	-1.090184164	0.207661134
Dido1|23856	-1.162286607	0.267497673	-1.022082771	0.454955471
Tmbim6|110213	-1.162306316	0.010533181	-1.149171286	0.002020212
Mina|67014	-1.162310347	0.130042557	-1.078552221	0.261822572
Dlc1|50768	-1.162329074	0.257605334	-1.308574957	0.137284062
Rnf128|66889	-1.162339504	0.19490296	-1.162339504	0.19490296
Itih3|16426	-1.162342647	0.122322542	-1.222567946	0.059301293
Fam49a|76820	-1.162382313	0.35868956	-1.480926399	0.195746513
4931414P19Rik|74359	-1.16249708	0.233169659	-1.295542597	0.073379645
Anapc7|56317	-1.162517132	0.201106258	-1.140685092	0.197100677
Mycbp2|105689	-1.162745186	0.229985029	-1.071170905	0.363336552
F8|14069	-1.162765341	0.354017002	-1.877681147	0.0400995
Zdhhc3|69035	-1.162827927	0.09165224	-1.308523541	0.002889854
Fam179b|328108	-1.162840048	0.378309808	-1.605288519	0.190484197
6330578E17Rik|76178	-1.162964266	0.235039168	-1.132541323	0.211469056
Aatf|56321	-1.162976318	0.116040769	-1.162976318	0.116040769
Hnrpll|72692	-1.162985074	0.326638488	-1.219833859	0.253503801
Niacr1|80885	-1.162990048	0.43126574	1.150701957	0.450338304
Tmem47|192216	-1.163077087	0.393515424	1.166381036	0.357980049
Zfp281|226442	-1.163077567	0.301012529	-1.384093357	0.142942317

5730494N06Rik|70612	-1.163133068	0.206090222	-1.163133068	0.206090222
Prpf3|70767	-1.163253831	0.047317297	-1.214283838	0.012522068
Flt4|14257	-1.163291369	0.127950497	-1.241002337	0.0405646
Scyl3|240880	-1.163306622	0.175407806	1.027388328	0.403867172
1500015A07Rik|68982	-1.163393931	0.345101419	1.29439236	0.20184601
Scn7a|20272	-1.163532469	0.4240673	-2.078368687	0.229588298
Hsbp1|68196	-1.163577461	0.104064793	-1.327161669	0.001733929
Cyp2r1|244209	-1.163606704	0.317476226	1.263699355	0.122378987
AA960436|101985	-1.163607021	0.236279601	-1.27674326	0.142232535
Nup35|69482	-1.163646588	0.409306379	-1.400433478	0.284360007
Cuedc1|103841	-1.163652535	0.303451708	1.078223102	0.373852026
4930555I21Rik|78806	-1.163689676	0.433899348	-1.001617989	0.49931754
Wdr81|192652	-1.164025109	0.088893263	-1.082994503	0.183319295
Eif3c|56347	-1.16419198	0.02064898	-1.189841851	0.00049419
Atp9b|50771	-1.16427026	0.041856658	-1.275607439	0.000820145
Hapln4|330790	-1.164335293	0.271423629	-1.455127611	0.040525389
2010204K13Rik|68355	-1.16437436	0.424292817	2.377509955	0.104925156
Serpina1d|20703	-1.164443809	0.120779177	-1.279256285	0.001903829
Srrt|83701	-1.164467418	0.075959248	-1.098529017	0.153936705
Rcsd1|226594	-1.164505202	0.244754738	1.000870442	0.498078024
Slc44a1|100434	-1.164624878	0.060855587	-1.221263979	0.026358556
Gas7|14457	-1.164784348	0.32869941	1.058179827	0.432856825
3000002C10Rik|378954	-1.16482281	0.356482978	-1.610825836	0.132478067
Mcm7|17220	-1.164939049	0.206189935	-1.051670027	0.385684752
Polr3b|70428	-1.164954115	0.138441752	-1.233419629	0.064108554
Nrd1|230598	-1.165102338	0.021034941	-1.257986281	9.38512E-05
Sptlc1|268656	-1.165187187	0.099529347	-1.079292693	0.141990974
1700001C19Rik|75462	-1.165198176	0.270132294	-1.165198176	0.270132294
Thnsl2|232078	-1.165281292	0.050669373	-1.1906408	0.045356313
Zfp191|59057	-1.16528713	0.090473411	-1.237576519	0.019783551
Ptgr2|77219	-1.165402641	0.100623945	-1.054074339	0.163344816
Eif2ak3|13666	-1.16547893	0.276724862	-1.299292488	0.177869062
Pcbp4|59092	-1.165629154	0.216910678	-1.392943529	0.01810093
Tmem158|72309	-1.165850394	0.437453649	-3.221509091	0.127882662
Gimap5|317757	-1.165927181	0.366771773	-1.165927181	0.366771773
Ager|11596	-1.166031552	0.389396952	-1.987541548	0.074579046
2310028H24Rik|71901	-1.166042318	0.263192671	-1.166042318	0.263192671
Sorbs3|20410	-1.166043563	0.079517026	-1.106004064	0.160015961
Chd7|320790	-1.16606934	0.194430256	1.037178359	0.358684339
Nsg1|18196	-1.166103972	0.264914733	-1.549526155	0.014219149
Sidt2|214597	-1.166192053	0.051024652	-1.166192053	0.051024652
Nudcd3|209586	-1.166223566	0.194133313	-1.166223566	0.194133313
Coro2b|235431	-1.166287483	0.238822748	-1.200614923	0.134969585
l7Rn6|67669	-1.166333747	0.192102752	-1.273952347	0.063891853
Bcl2l12|75736	-1.166346897	0.247294968	-1.151886073	0.193485153
Zkscan5|22757	-1.16640908	0.123330647	-1.266609621	0.036777142
Vps33a|77573	-1.166483875	0.167795374	-1.287234358	0.034325592
Chaf1a|27221	-1.166513146	0.29782108	-1.38522985	0.143729739
Gbf1|107338	-1.166518887	0.046235127	-1.166518887	0.046235127
Iqcd|75732	-1.166604495	0.346384504	1.021275564	0.475960388
Ociad2|433904	-1.166638354	0.287686146	-1.317136451	0.116056975
Ache|11423	-1.166669055	0.411319661	-3.485201345	0.01373478
Slc25a24|229731	-1.166754581	0.421383765	-4.416830303	0.002861384

BC026585|226527	-1.166870128	0.124816903	-1.28406249	0.023514116
Scamp3|24045	-1.167008614	0.044635156	-1.15280702	0.016478064
Dctn1|13191	-1.167011729	0.03803293	-1.055648005	0.106683486
Akr1c13|27384	-1.16702596	0.01404547	-1.168724465	0.003144258
D10Jhu81e|28295	-1.167037959	0.068978593	-1.112477338	0.138865545
Scarf2|224024	-1.167089436	0.345846382	1.113564778	0.385821642
Coasy|71743	-1.167115687	0.031311712	-1.109476296	0.053080291
Igsf11|207683	-1.167270477	0.205240513	-1.167270477	0.205240513
Pvt1|19296	-1.167325582	0.398715476	-1.167325582	0.398715476
1190007F08Rik|68859	-1.167395111	0.342782459	-1.467850276	0.177681063
Zfp828|101994	-1.167550272	0.296975974	-1.167550272	0.296975974
Ube2k|53323	-1.167734595	0.185904913	-1.28862	0.078114596
Tti2|234138	-1.16774554	0.062554027	-1.054849615	0.226125368
Lclat1|225010	-1.167775758	0.211077958	-1.167775758	0.211077958
1110008L16Rik|66132	-1.167831193	0.163214588	-1.184671434	0.093110371
Clasp1|76707	-1.167882296	0.219355902	1.066039665	0.324499276
AI837181|107242	-1.167909966	0.072237632	-1.20801744	0.052205356
5031425F14Rik|319684	-1.167976153	0.412508893	-1.305244483	0.378399987
Wdr18|216156	-1.168024388	0.031285479	-1.104645253	0.043069157
Zkscan6|52712	-1.16826177	0.307054315	-1.00276196	0.496509938
Fbxo4|106052	-1.168287243	0.22949561	-1.481405388	0.016725757
Zfp263|74120	-1.168326806	0.185614783	-1.157919394	0.158176535
Vmac|106639	-1.168327377	0.108219807	-1.316302602	0.007327138
Atf6|226641	-1.168339108	0.169941946	-1.168339108	0.169941946
Slc6a9|14664	-1.168341661	0.155204525	-1.168341661	0.155204525
Ncoa1|17977	-1.168342496	0.243648959	1.030114084	0.418127229
Myo9b|17925	-1.168391532	0.158037899	1.002074076	0.493220685
Egln1|112405	-1.168410661	0.07627851	-1.321867868	0.001311579
Sgce|20392	-1.168463272	0.26080608	-1.302019769	0.118505185
Itgb5|16419	-1.168587654	0.116960488	-1.25253339	0.027127803
Sh2b1|20399	-1.168617689	0.090374754	-1.317644329	0.00619576
Psmb9|16912	-1.168624918	0.254099623	-1.203077549	0.15633352
Bcat2|12036	-1.168638084	0.155574135	-1.168638084	0.155574135
Golga1|76899	-1.168642236	0.222757548	-1.099547742	0.332211542
Urgcp|72046	-1.168843689	0.077797471	-1.09320895	0.159086675
Aqp11|66333	-1.16884723	0.082853284	-1.08899096	0.169827149
Slc46a1|52466	-1.169136433	0.039484869	-1.176398682	0.017318945
Mga|29808	-1.169142914	0.313471258	-1.146146121	0.222887578
Luc7l2|192196	-1.169198725	0.248259412	1.042793289	0.415780973
Gigyf1|57330	-1.169228166	0.14735798	-1.269060417	0.060897662
Ak3|56248	-1.169277363	0.007442411	-1.127192229	0.008521427
Mtmr7|54384	-1.169284889	0.352533128	-1.617758411	0.12916833
Cdadc1|71891	-1.16931938	0.151819615	-1.002889247	0.489868472
Pisd-ps2|328734	-1.169320009	0.348760645	-1.025477069	0.474216428
Tmem115|56395	-1.169405943	0.132021595	-1.23897211	0.077863519
Usp47|74996	-1.169500505	0.109027514	-1.098510952	0.208658026
Cachd1|320508	-1.169502914	0.370386032	-1.479100514	0.233992227
Sh3bgrl|56726	-1.169559585	0.312016522	-1.120856773	0.325983009
Baz2a|116848	-1.16973348	0.298104717	1.177768206	0.218173178
Rtn4|68585	-1.169862533	0.186962182	-1.17126968	0.171197628
Ubxn8|108159	-1.169917229	0.080586555	-1.311852852	0.005511502
Cdt1|67177	-1.17000961	0.295700876	-1.428454933	0.115851023
Mpp5|56217	-1.170030102	0.266386615	-1.371958361	0.110483959

Hnf1a|21405	-1.17005446	0.119110177	-1.127196288	0.113807802
Cyp7a1|13122	-1.170107851	0.341398706	-1.170107851	0.341398706
Pcnx|54604	-1.170116141	0.035954871	-1.162496446	0.022024818
Fitm2|228859	-1.170135995	0.215009597	-1.459761244	0.012844802
Tcn2|21452	-1.170205629	0.116099348	-1.170205629	0.116099348
Ythdf3|229096	-1.170228179	0.321534753	-1.050311188	0.438433218
1110018G07Rik|68497	-1.170271183	0.207094203	-1.05814238	0.378915669
Uba2|50995	-1.170295031	0.264768021	-1.003906862	0.493201708
Tceb1|67923	-1.170306324	0.089790381	-1.038640198	0.331125822
Macrod2|72899	-1.170309256	0.220126326	-1.339348377	0.074776199
Mterf|545725	-1.170339789	0.274824718	-1.167297565	0.144855092
Arfgef2|99371	-1.170432491	0.203425032	-1.034509379	0.415669253
Dlg1|13383	-1.170436258	0.300284777	1.057107214	0.415098059
Ergic3|66366	-1.170449583	0.144172451	-1.376469102	0.006716292
Cops3|26572	-1.170521361	0.245379177	-1.11772579	0.275214489
Carhsp1|52502	-1.170561463	0.00401634	-1.108739668	0.002542993
Gtf2f1|98053	-1.170620555	0.206114089	-1.170620555	0.206114089
Ss18l1|269397	-1.170725749	0.318554809	-1.164278078	0.284018604
Fam203a|59053	-1.170797922	0.253193914	-1.170797922	0.253193914
Hspb7|29818	-1.170826329	0.412908888	-1.557757649	0.233183213
Chi3l1|12654	-1.170859674	0.447086006	1	#DIV/0!
Crnkl1|66877	-1.170860848	0.228990242	1.073476644	0.325705142
Brdt|114642	-1.170927559	0.326764795	1.019716241	0.474250015
Cbr1|12408	-1.171090277	0.089811548	-1.265908619	0.004073898
Pisd|320951	-1.171118004	0.069922403	-1.104774968	0.142029884
Prdm2|110593	-1.171149991	0.2618847	-1.20067761	0.125850378
Slc23a1|20522	-1.171279349	0.070755566	-1.171279349	0.070755566
Slc12a6|107723	-1.171427338	0.274965471	1.028915149	0.448853908
Sfmbt1|54650	-1.171466598	0.233992891	-1.068743584	0.335042911
Pebp1|23980	-1.171485896	0.07578682	-1.194676007	0.01847383
Tacc3|21335	-1.171494561	0.325693484	-1.767951503	0.019223771
Slc43a1|72401	-1.171637961	0.23829579	-1.34180119	0.040899079
Rnf25|57751	-1.171642771	0.181760389	-1.088145734	0.305730299
Pdgfrb|18596	-1.171671225	0.22283704	-1.332668995	0.053000034
Lrrc40|67144	-1.171705633	0.214778945	-1.171705633	0.214778945
AW011738|100382	-1.171719695	0.331538769	-1.555243365	0.113962539
Fahd2a|68126	-1.171759379	0.178652482	-1.306167646	0.020837265
Zfyve27|319740	-1.171762473	0.108369075	-1.159023208	0.092275709
Acat1|110446	-1.171766583	0.096516733	-1.096483231	0.192500763
Fiz1|23877	-1.171807545	0.072088865	-1.133118632	0.132492158
Sp140|434484	-1.1718336	0.2654422	-1.563342944	0.020920812
Sf1|22668	-1.171847737	0.072922663	-1.219681826	0.046552095
Tmc4|353499	-1.171868327	0.364100721	-1.823724121	0.004280278
Nupl2|231042	-1.171971381	0.322833465	-1.171971381	0.322833465
Atp5sl|66349	-1.172205403	0.116060138	-1.172205403	0.116060138
Rabgap1|227800	-1.172733933	0.23033913	-1.232882068	0.152180237
2210016F16Rik|70153	-1.172761834	0.096698667	-1.181306734	0.044922405
Sh3glb1|54673	-1.17276987	0.027082571	-1.17276987	0.027082571
S1pr3|13610	-1.172852542	0.286766694	-1.340942707	0.103015291
Arhgap12|75415	-1.172920958	0.317359914	-1.456333878	0.144009401
Fam89a|69627	-1.173015199	0.274665735	-1.122987922	0.26939769
Cnot3|232791	-1.173046619	0.098041167	-1.098493277	0.193979014
Zscan22|232878	-1.173120796	0.250141276	-1.164467238	0.171919339

2810006K23Rik|72650	-1.173125173	0.123998746	-1.257599525	0.056900495
Ppa1|67895	-1.173218747	0.052157318	-1.179692967	0.010263433
Csda|56449	-1.173223336	0.011259808	-1.20245424	0.00542881
Sipa1|20469	-1.173478659	0.130980008	-1.308761094	0.018949788
Adamts2|216725	-1.173496649	0.18246115	-1.29519801	0.078616795
Smap2|69780	-1.17349701	0.012829548	-1.226843666	0.001739371
C8a|230558	-1.173528578	0.133427425	-1.173528578	0.133427425
Stx6|58244	-1.173529525	0.198665063	-1.051530104	0.384306682
Abcb11|27413	-1.173651097	0.176415752	-1.044341252	0.375547475
Rdh13|108841	-1.173716427	0.203661763	-1.173716427	0.203661763
Slc16a7|20503	-1.173725205	0.100722899	-1.298904992	0.008094888
D11Wsu47e|276852	-1.173742072	0.244583002	-1.200696063	0.181623043
Nae1|234664	-1.173766593	0.287085081	-1.221991535	0.21150668
Oxr1|170719	-1.173800535	0.332529443	-1.000771042	0.49876224
Irf9|16391	-1.173858694	0.102436767	-1.040871329	0.34179513
Farsb|23874	-1.173902326	0.125288477	-1.015430327	0.441118397
Rae1|66679	-1.174010065	0.189080581	-1.174010065	0.189080581
Aff1|17355	-1.174119547	0.216695327	-1.16054074	0.128740839
Acot7|70025	-1.17416414	0.065223936	-1.247071581	0.008517644
6820431F20Rik|547150	-1.174185497	0.201007609	-1.139082434	0.175942294
Pafah2|100163	-1.174196812	0.070331096	-1.086503175	0.138446317
Pde7a|18583	-1.174224164	0.248784329	1.08308308	0.339599417
Scfd2|212986	-1.174536293	0.017321995	-1.131079068	0.035615931
Mfap3l|71306	-1.17462162	0.419551604	-2.098176768	0.227724758
Tns3|319939	-1.174666126	0.349894802	-1.058728218	0.421808428
Clcn4-2|12727	-1.174674991	0.155340111	-1.292219236	0.025647314
Bach2|12014	-1.174701647	0.317977753	-1.174701647	0.317977753
Acox1|11430	-1.174733058	0.139426796	-1.261762407	0.045747032
Acss2|60525	-1.174765519	0.155069669	-1.174765519	0.155069669
Rab5c|19345	-1.174800231	0.010753241	-1.154090855	0.00251564
Ppip5k1|327655	-1.174830593	0.212839947	-1.338927894	0.075265534
Tnfaip2|21928	-1.174865894	0.23866013	-1.067376091	0.26168176
C77080|97130	-1.174867075	0.012380055	-1.100484986	0.040570375
Ebf4|228598	-1.174981695	0.413929317	-1.074966612	0.46340475
Pno1|66249	-1.174992472	0.263330197	-1.174992472	0.263330197
Ubn2|320538	-1.175012266	0.230789062	1.011132388	0.470939363
Mcoln1|94178	-1.175123716	0.107997931	-1.238126236	0.048965966
Thoc6|386612	-1.175127984	0.235825687	-1.057138914	0.393208744
Lrig1|16206	-1.1751601	0.274283017	-1.1751601	0.274283017
Nfkbib|18036	-1.175160407	0.069729727	-1.253795573	0.007058771
Uchl5|56207	-1.175199337	0.263939174	-1.175199337	0.263939174
Lonp1|74142	-1.175432349	0.026483126	-1.142179281	0.05462071
Zfp142|77264	-1.175537676	0.238750895	-1.203492939	0.159702888
Cdc23|52563	-1.175558332	0.196404601	-1.175558332	0.196404601
Fam46a|212943	-1.175575143	0.213420557	-1.266072535	0.145896881
Otud5|54644	-1.175648491	0.070849222	-1.175648491	0.070849222
AI507597|100165	-1.175653875	0.164204263	-1.07925492	0.311915211
Arhgap29|214137	-1.175777759	0.278224996	-1.175777759	0.278224996
Akap1|11640	-1.175805538	0.139600558	-1.175805538	0.139600558
Hnrnph2|56258	-1.17595665	0.264961868	1.032023199	0.437717069
Fam185a|330050	-1.176353554	0.298022942	-1.176353554	0.298022942
Fkbp8|14232	-1.176431776	0.198894123	-1.363417682	0.041256542
Plekhf2|71801	-1.176461613	0.179787177	-1.235058073	0.126604854

C2cd2|207781	-1.176462276	0.009087929	-1.097233068	0.011381347
Kmo|98256	-1.1765459	0.143945153	-1.1765459	0.143945153
Rnf24|51902	-1.176547198	0.272908313	-1.390846888	0.119014938
Fxyd6|59095	-1.176572857	0.343030679	-1.019944789	0.477744197
Hdac8|70315	-1.17661704	0.312578709	1.013006072	0.484716818
Zfp770|228491	-1.176677814	0.344278735	-2.045230263	0.006441542
Osmr|18414	-1.176727845	0.385497767	-1.727213243	0.195612428
Nagpa|27426	-1.17681135	0.183279377	-1.154969902	0.173037991
Pkn1|320795	-1.176844317	0.136139761	-1.380645493	0.008278303
Aarsd1|69684	-1.176922015	0.138124309	-1.263974619	0.045932059
Gphn|268566	-1.176945692	0.195252233	-1.287657393	0.072564857
Mynn|80732	-1.177092268	0.266346571	-1.022216721	0.465560038
Tnrc6a|233833	-1.177122587	0.295761735	1.096612041	0.332633702
Capn7|12339	-1.17717913	0.292187259	-1.597660291	0.037279394
Lhfpl2|218454	-1.177226004	0.318102943	-1.177226004	0.318102943
Rcan1|54720	-1.177290065	0.146178325	-1.217109694	0.040652616
Ssh2|237860	-1.17734785	0.393287128	1.440650232	0.261351483
Dvl1|13542	-1.177386702	0.064563318	-1.256192523	0.017014596
Nubp1|26425	-1.17742964	0.123991015	-1.17742964	0.123991015
Zfp866|330788	-1.177517946	0.323397346	1.273758954	0.18119326
Mcm2|17216	-1.177596194	0.356677619	-1.128767193	0.394689542
Kcnip3|56461	-1.177667391	0.282251355	-1.177667391	0.282251355
Gpr108|78308	-1.177729807	0.16397326	-1.268585314	0.092668425
Pde8a|18584	-1.177731004	0.198017403	-1.094660316	0.27963337
Wrn|22427	-1.177763417	0.083505322	-1.114044359	0.166514402
Aldh7a1|110695	-1.177914252	0.016487169	-1.160170969	0.032993158
Tkt|21881	-1.177945823	0.00243629	-1.142717418	0.000272089
Cir1|66935	-1.178107369	0.034283579	-1.260355089	0.002114212
1700025G04Rik|69399	-1.17817687	0.270496251	1.152934766	0.214719489
Hpgds|54486	-1.178198199	0.4034044	1.153994792	0.427016401
Zfp628|232816	-1.178358665	0.154675161	-1.178358665	0.154675161
Twf2|23999	-1.178360878	0.287410159	-1.023257733	0.464928236
Hmg20a|66867	-1.17838582	0.19781789	-1.17838582	0.19781789
Edaradd|171211	-1.178571429	0.434978868	1	#DIV/0!
Slco4a1|108115	-1.178571429	0.434978868	1	#DIV/0!
Cldn10|58187	-1.178571429	0.434978868	1	#DIV/0!
Cyp3a57|622127	-1.178571429	0.434978868	1	#DIV/0!
Aspm|12316	-1.178571429	0.434978868	1	#DIV/0!
Slc7a6|330836	-1.178571429	0.434978868	1	#DIV/0!
Wdr63|242253	-1.178571429	0.434978868	1	#DIV/0!
Tnfaip8l3|244882	-1.178571429	0.434978868	1	#DIV/0!
Slc5a11|233836	-1.178571429	0.434978868	1	#DIV/0!
Slc39a2|214922	-1.178583941	0.380771592	-1.178583941	0.380771592
Zfp612|234725	-1.178647985	0.201143614	1.052524773	0.352974604
Pdcd6ip|18571	-1.178717921	0.109579223	-1.178717921	0.109579223
Ksr1|16706	-1.178766399	0.35869223	-1.468185871	0.228617239
Ano6|105722	-1.17882012	0.280002513	1.046470024	0.4208153
Akap8|56399	-1.178886402	0.270989018	1.047961208	0.407948095
Sh2d4a|72281	-1.178932121	0.216760373	-1.223095226	0.149902667
Strada|72149	-1.178964807	0.259803084	-1.293762504	0.08401263
U2af1l4|233073	-1.179047411	0.14240331	-1.313621261	0.032201795
4930444A02Rik|74653	-1.179245017	0.249822611	-1.31892715	0.148478237
4933404O12Rik|66752	-1.179245536	0.26106994	-1.12150734	0.255830794

Wbp11|60321	-1.179347758	0.07761563	-1.077969532	0.146320344
Tgfb1i1|21804	-1.17938343	0.335244734	-1.362913162	0.072886331
Acot8|170789	-1.179445404	0.152927248	-1.062729884	0.321650334
Csf1r|12978	-1.179461311	0.143250532	-1.179461311	0.143250532
Eno3|13808	-1.179461434	0.290281907	-1.460506716	0.104388928
Ccdc64|75665	-1.179498328	0.416069701	1.280421331	0.368728465
Cep290|216274	-1.179600808	0.418903896	-1.864887618	0.261052046
Ndufs8|225887	-1.179770809	0.16108728	-1.141069508	0.175735954
Masp2|17175	-1.179798841	0.0023876	-1.192798418	0.002759016
BC037034|231807	-1.179810282	0.082237223	-1.099420641	0.169102017
Itpripl2|319622	-1.179817231	0.322794249	1.020985875	0.477608915
Hs6st1|50785	-1.179842817	0.035897466	-1.181262703	0.029214997
Gtf2ird2|114674	-1.179921323	0.278256226	-1.653838854	0.015047724
Ppp1cc|19047	-1.179975238	0.140983645	-1.253154313	0.050702688
Amz2|13929	-1.179994566	0.150857904	-1.068364698	0.312073919
Ift46|76568	-1.180045617	0.209989582	-1.180045617	0.209989582
Tnfsf10|22035	-1.180052493	0.348163853	1.043357547	0.462623034
1110004E09Rik|68001	-1.180079468	0.206223991	-1.092134183	0.327232462
Rcor1|217864	-1.180079664	0.444498788	2.023658599	0.304156948
Wdfy4|545030	-1.180294702	0.247940702	-1.392719375	0.087539088
Slc25a37|67712	-1.180536214	0.106393485	-1.032028223	0.365049566
Abr|109934	-1.180628271	0.343155099	1.28975408	0.237738089
Bcdin3d|75284	-1.180878064	0.310006218	-1.180878064	0.310006218
Pip5k1c|18717	-1.180920661	0.041856401	-1.228011277	0.016167292
Snap29|67474	-1.180975148	0.125412786	-1.299736092	0.032282349
Odz3|23965	-1.180983377	0.241507481	1.04910357	0.407993746
Dock3|208869	-1.181004543	0.328505951	-1.535911791	0.137738471
Hnf1b|21410	-1.181010451	0.015079492	-1.181010451	0.015079492
Apoc1|11812	-1.181223405	0.306402576	-1.233790023	0.18908148
Cfl2|12632	-1.181254063	0.187562969	-1.304682434	0.050927328
Trmt2b|215201	-1.181266362	0.086309358	-1.116868491	0.166124481
A830007P12Rik|227612	-1.181267257	0.219952458	-1.080752454	0.362806124
Atxn2|20239	-1.181315508	0.106353001	-1.177102624	0.046915596
Ccr7|12775	-1.181338568	0.40127872	-2.192782516	0.152660237
Il6st|16195	-1.181378255	0.172002663	-1.066851585	0.340354113
Ston1|77057	-1.181398122	0.386586029	-1.061243569	0.437578471
Tmem51|214359	-1.181546858	0.182413871	-1.312991148	0.076175682
Plekhh3|217198	-1.181632417	0.088451247	-1.181632417	0.088451247
Lrwd1|71735	-1.181662689	0.210727284	-1.33265202	0.042449156
Hoxa2|15399	-1.181710067	0.417022793	-1.047938656	0.479862512
0610040J01Rik|76261	-1.181739622	0.019647036	-1.151213157	0.041876547
Lrp3|435965	-1.181773899	0.139960663	-1.002034332	0.492384746
Tmem180|75146	-1.18187193	0.238738414	-1.282751502	0.142356289
4732471J01Rik|654804	-1.181932446	0.406525725	-1.526084848	0.310510956
Blzf1|66352	-1.18200175	0.324221195	-1.648375365	0.073087389
Nme2|18103	-1.182031114	0.193642932	-1.117144587	0.231344586
Lonrf1|244421	-1.182043806	0.275021566	-1.028656674	0.454564615
Adhfe1|76187	-1.182097759	0.221082915	-1.031740819	0.435113169
Pigg|433931	-1.182153956	0.35016718	1.123016731	0.281889663
Ttc38|239570	-1.182200518	0.046855344	-1.223537548	0.00478893
Elmo2|140579	-1.182244735	0.153101259	-1.079817106	0.302120126
Shmt1|20425	-1.182289893	0.020166559	-1.188276367	0.004586364
Naglu|27419	-1.182302937	0.127139396	-1.099683704	0.246290621

Mrpl34|94065	-1.182463504	0.197380201	-1.067054239	0.354423999
1200011I18Rik|67467	-1.182639014	0.191683859	-1.335517814	0.068264706
Cdon|57810	-1.182686751	0.35660964	-1.182686751	0.35660964
Zbtb7b|22724	-1.182736608	0.048119468	-1.210994551	0.010416444
Tbc1d23|67581	-1.182962797	0.338947979	-1.570478272	0.147375224
Lmf2|105847	-1.183039036	0.037579482	-1.152394431	0.071661572
Rps6ka4|56613	-1.183064306	0.104247335	-1.190227267	0.013194608
Rwdd4a|192174	-1.18308902	0.119669969	-1.287737854	0.040497262
Nufip1|27275	-1.183160399	0.241890317	-1.192992271	0.160429752
Srsf3|20383	-1.183297529	0.223296704	-1.041259743	0.412762324
Phf3|213109	-1.183303586	0.169339862	-1.055422787	0.354467499
Copz2|56358	-1.183306886	0.183061301	-1.062905825	0.358097588
Ints3|229543	-1.183325557	0.124440722	-1.297870188	0.036414059
Nipsnap3b|66536	-1.183502695	0.104499141	-1.183502695	0.104499141
Myh14|71960	-1.183518471	0.072742354	-1.258997843	0.027167428
Trim26|22670	-1.183532262	0.122864674	-1.07616304	0.260327599
Slc10a7|76775	-1.183556547	0.246233654	-1.151142618	0.222051461
Btaf1|107182	-1.183622642	0.285716975	-1.068219209	0.41084246
Tbc1d16|207592	-1.183682481	0.143043247	-1.193699127	0.054767808
1190002N15Rik|68861	-1.183777717	0.280314684	1.004707832	0.493153752
Gabrd|14403	-1.183874881	0.408098998	-2.380122345	0.157676859
Srsf7|225027	-1.183879948	0.311762867	-1.183879948	0.311762867
Pex7|18634	-1.183926578	0.118389275	-1.304931805	0.026980562
Plxnc1|54712	-1.183991153	0.325969912	1.051561834	0.446384571
Ttll12|223723	-1.184062361	0.160844359	-1.310291496	0.024520985
Nup62-il4i1|100328588	-1.184067473	0.401409977	-1.179215768	0.419092101
Slc28a1|434203	-1.184167091	0.417313686	2.166774358	0.161185597
Spata2l|78779	-1.184174535	0.179455781	-1.29837249	0.090932252
Ncf1|17969	-1.184292296	0.2680813	-1.65422384	0.012127996
Igsf3|78908	-1.184496858	0.376603391	-1.951633713	0.119335212
Exph5|320051	-1.18457728	0.29793411	1.008782606	0.488930826
Macf1|11426	-1.184583873	0.212412923	-1.033940398	0.410673182
40976|71779	-1.184589451	0.208689478	-1.062294929	0.340267131
Scand1|19018	-1.184598263	0.376455525	-1.804446466	0.160795832
Ppp2r5d|21770	-1.184612703	0.002195332	-1.151051683	0.002750946
Astn2|56079	-1.184621583	0.263957912	-1.389112782	0.05202758
Tbc1d12|209478	-1.184634694	0.332625847	-1.184634694	0.332625847
Nfyb|18045	-1.184716159	0.180232034	1.025853819	0.420029302
Cdc42ep1|104445	-1.18479094	0.121327731	-1.283364225	0.048174309
Zkscan17|268417	-1.184803166	0.10156674	-1.118345946	0.188416633
Aqp9|64008	-1.184836409	0.023203479	-1.156520373	0.048181491
Commd1|17846	-1.184878179	0.112810769	-1.009335953	0.455697684
1600016N20Rik|72000	-1.18502187	0.377282582	-1.81241281	0.161792722
Prpf39|328110	-1.18506915	0.257163773	-1.571682804	0.030586501
Usp32|237898	-1.185199989	0.332116745	-1.095676881	0.392710739
Gdf6|242316	-1.18525056	0.397496392	1.315138262	0.351077048
5031439G07Rik|223739	-1.18529244	0.007249592	-1.230988983	0.001618544
Madd|228355	-1.185331452	0.046987853	-1.125280302	0.095537706
Eif2c1|236511	-1.185466132	0.217174736	-1.370892531	0.073871498
Aco1|11428	-1.185510195	0.05404611	-1.125474269	0.109759573
Anapc4|52206	-1.185557116	0.102996204	-1.185557116	0.102996204
Foxj3|230700	-1.185620921	0.299616776	-1.007570916	0.487057093
Twf1|19230	-1.185645132	0.203023768	1.075095069	0.276774895

Commd4|66199	-1.185743705	0.18743837	1.034111837	0.391772336
Smc3|13006	-1.185753016	0.202851941	-1.331104726	0.090529816
Eri2|71151	-1.185776828	0.29104238	-1.185776828	0.29104238
Fbxl14|101358	-1.185840954	0.267889482	1.037532897	0.432223944
Luc7l|66978	-1.185903933	0.051248053	-1.221068398	0.041158619
Hdac7|56233	-1.185991711	0.155201873	-1.390394083	0.005146795
BC030336|233812	-1.186023289	0.25961273	1.14174269	0.230782895
Tgm2|21817	-1.186147446	0.039118027	-1.14206395	0.079037924
Rpa2|19891	-1.186252808	0.193469682	-1.186252808	0.193469682
Vars|22321	-1.186279908	0.048651663	-1.186279908	0.048651663
Copa|12847	-1.186333961	0.112011722	-1.112665646	0.208274215
Hipk1|15257	-1.186352642	0.212251223	-1.015970396	0.461448201
1700047M11Rik|67330	-1.186365023	0.381507521	-1.621424424	0.232180346
Man1b1|227619	-1.186548235	0.118811624	-1.313264566	0.024772404
1110037F02Rik|66185	-1.186554203	0.137701052	-1.002749851	0.489906204
Plekhg6|213522	-1.186636119	0.170585015	-1.31076703	0.037377748
Snx33|235406	-1.186740647	0.106201232	-1.098544903	0.217241969
Hdgfrp2|15193	-1.187322281	0.114699278	-1.273741303	0.0543694
Inpp5f|101490	-1.187418731	0.176427874	-1.051524001	0.369969343
Cmah|12763	-1.187610807	0.173430323	-1.061701672	0.352081287
Pspc1|66645	-1.187628651	0.294808609	-1.155816692	0.221420862
Atp8b5|320571	-1.187667298	0.367581033	-1.39864107	0.187975053
Arrdc1|215705	-1.187710707	0.263897658	-1.187710707	0.263897658
Btbd1|83962	-1.187892391	0.10343297	-1.025762486	0.390714398
Psme4|103554	-1.187901599	0.138545737	-1.082653764	0.273986298
Xrcc6|14375	-1.187924832	0.227460308	-1.187924832	0.227460308
Rab20|19332	-1.187961851	0.288906759	-1.187961851	0.288906759
Mblac1|330216	-1.187965817	0.356436261	-2.278992298	0.010111503
Sympk|68188	-1.188070046	0.085396215	-1.188070046	0.085396215
Pigy|66459	-1.18808105	0.051690297	-1.167888348	0.016209467
Khnyn|219094	-1.188139776	0.229828108	-1.052905473	0.409836055
1700113I22Rik|73635	-1.18818898	0.348978125	-1.18818898	0.348978125
Srrm1|51796	-1.188318688	0.182621165	-1.052901631	0.374639544
5830417I10Rik|100302730	-1.188349865	0.227586851	-1.002493485	0.494377276
1110057K04Rik|68832	-1.188508617	0.117833829	-1.122307697	0.081320084
Stt3a|16430	-1.188529826	0.06846116	-1.255912693	0.018894287
Mpv17|17527	-1.188531759	0.07854828	-1.274803738	0.02551084
Nos3|18127	-1.188576752	0.182678887	-1.1772446	0.155489754
Nat15|74763	-1.188601518	0.01334655	-1.153012327	0.028366507
Serhl|68607	-1.18863617	0.176099962	-1.185056102	0.156308764
Aco2|11429	-1.188706883	0.039836052	-1.27170375	0.005271656
Dtx2|74198	-1.188725373	0.258767358	-1.089097626	0.383169603
Tm9sf4|99237	-1.188782606	0.008144265	-1.228132989	0.001693566
Sema6c|20360	-1.188798572	0.3221308	-1.569931691	0.122318932
Tctn3|67590	-1.188909865	0.3806193	1.403650437	0.251424635
Fmo1|14261	-1.188925254	0.035368928	-1.07443589	0.119407509
Fam20b|215015	-1.188971076	0.143321251	-1.188971076	0.143321251
Whsc1l1|234135	-1.188983085	0.176901873	1.040630663	0.347763928
Pla2g15|192654	-1.189139103	0.143949309	-1.056233152	0.310841777
Atf7ip|54343	-1.189289235	0.150719384	-1.189289235	0.150719384
Ppih|66101	-1.189569892	0.238785002	-1.263173496	0.179390186
Fam198a|245050	-1.189602734	0.221395816	-1.356764427	0.043772165
Xiap|11798	-1.189687474	0.336928684	1.183206025	0.308082511

Mpst|246221	-1.189746597	0.101540847	-1.274596536	0.045339778
Btbd2|208198	-1.18996488	0.128039446	-1.250151095	0.092658356
Pycrl|66194	-1.189979299	0.129157262	-1.283314091	0.062903567
Gm10778|100233208	-1.190266316	0.355977118	-1.881392253	0.086276412
Marcksl1-ps4|111002	-1.190361939	0.138611315	-1.30164118	0.026626172
Nfe2l1|18023	-1.190401455	0.055425549	-1.102084521	0.090381491
Bloc1s1|14533	-1.190470199	0.15865867	-1.455474156	0.004997838
Ptpn11|19247	-1.190482186	0.200418854	-1.026473178	0.435010238
Chmp2b|68942	-1.190493478	0.206225552	-1.500351152	0.00986593
Mthfd1l|270685	-1.190538202	0.324060563	1.310912687	0.161369813
Snrpa|53607	-1.190756648	0.144860141	-1.205857347	0.081254837
Map3k2|26405	-1.19077692	0.285750344	-1.146361529	0.290202708
Marveld2|218518	-1.190858314	0.27062995	-1.158294314	0.256532477
Pcm1|18536	-1.190933255	0.274938298	1.058725433	0.396563009
Ubash3b|72828	-1.190939197	0.335968732	1.037180287	0.46643771
Tor1aip2|240832	-1.190945057	0.137364406	-1.076521669	0.287877729
Rufy1|216724	-1.190969824	0.10328693	-1.190969824	0.10328693
Pank4|269614	-1.191072795	0.215817307	-1.262883597	0.104075947
Hdac3|15183	-1.19112338	0.100124952	-1.238152816	0.067505265
Fbln2|14115	-1.191449385	0.386354681	-2.393278761	0.015938416
Heatr6|217026	-1.191456489	0.028332531	-1.28248408	0.000814465
2510012J08Rik|70312	-1.191466352	0.070993248	-1.299742581	0.009197971
Fam169b|434197	-1.191498236	0.057097201	-1.054527972	0.186175801
Klhl28|66689	-1.191630268	0.404464512	-2.460909091	0.146965028
Mlh1|17350	-1.191631895	0.188877369	-1.357941972	0.062192833
Gm6548|625054	-1.191703375	0.135367391	-1.193563657	0.172584357
1700037H04Rik|67326	-1.19178294	0.157606183	-1.229173366	0.05612166
Evc2|68525	-1.191837102	0.331879566	-1.414122693	0.224242655
Thsd1|56229	-1.191868193	0.132750629	-1.074416542	0.275358613
Fbxo34|78938	-1.192017734	0.133486112	-1.097378392	0.262376202
Ppil1|68816	-1.192126202	0.137646546	-1.451505324	0.001473098
Usp40|227334	-1.192281653	0.048584923	-1.192207998	0.016814561
Usp34|17847	-1.192437971	0.283866315	-1.504943863	0.041350996
Ttbk1|106763	-1.192595796	0.400700834	-2.999295613	0.068065451
1110014N23Rik|68505	-1.192616223	0.014473108	-1.218523077	0.001227859
Slc3a2|17254	-1.192638395	0.028682214	-1.192638395	0.028682214
Sec16b|89867	-1.192709686	0.186968968	-1.192709686	0.186968968
Cd200r4|239849	-1.192742857	0.430599145	1	#DIV/0!
Crb2|241324	-1.192742857	0.430599145	1	#DIV/0!
Elane|50701	-1.192742857	0.430599145	1	#DIV/0!
Shisa9|72555	-1.192742857	0.430599145	1	#DIV/0!
Cyp4a30b-ps|435802	-1.192742857	0.430599145	1	#DIV/0!
Neto2|74513	-1.192742857	0.430599145	1	#DIV/0!
Trak2|70827	-1.192820078	0.240138348	-1.130551373	0.270635153
Zfp358|140482	-1.19283488	0.22587418	-1.236149541	0.149570614
Tgfb1|21803	-1.192871526	0.135931013	-1.089084272	0.265900179
Rhobtb2|246710	-1.192888774	0.134306948	-1.143794805	0.061074589
Dusp28|67446	-1.192909353	0.193018864	-1.067214428	0.365745316
Mup8|100041687	-1.192939758	0.360574896	-1.192939758	0.360574896
Krt8|16691	-1.193265312	0.048947494	-1.13521847	0.099439529
Ccdc66|320234	-1.1933491	0.30115201	1.095827192	0.366198229
Wrap53|216853	-1.193398383	0.131348977	-1.076587489	0.278537921
A530054K11Rik|212281	-1.193494036	0.414041309	-2.097875187	0.183399184

Rbfox2|93686	-1.193569137	0.096512568	-1.093165963	0.200674903
Dock8|76088	-1.193631402	0.158052262	-1.119641375	0.152848068
Cox18|231430	-1.193664328	0.161812596	-1.109292808	0.283497484
Gm2058|100039133	-1.193707926	0.208716875	1.066713535	0.337769265
Ralgapa2|241694	-1.193789927	0.287056611	-1.19503946	0.278311605
Ubqln4|94232	-1.193838744	0.175968734	-1.02815997	0.410555473
Cyp3a13|13113	-1.193935977	0.057048514	-1.35329182	0.00100754
Zfp661|72180	-1.193989233	0.303801339	1.060079938	0.42589396
Zfp667|384763	-1.194132443	0.29575558	1.075692204	0.393896883
Med13l|76199	-1.194137903	0.272356272	1.036216976	0.441358089
Slc25a38|208638	-1.194182626	0.044278201	-1.194182626	0.044278201
Zfp90|22751	-1.194240485	0.352815327	1.132042399	0.396739622
Ppie|56031	-1.194265842	0.159003347	-1.004623978	0.487245331
Uhrf1bp1|224648	-1.194401567	0.162009228	1.008553299	0.475279562
Tut1|70044	-1.194600369	0.045302485	-1.062349724	0.1564885
Mib1|225164	-1.194602204	0.163538443	1.023503514	0.424651408
Dhrs9|241452	-1.194664337	0.416250627	-1.098041369	0.467419217
Zgpat|229007	-1.194681565	0.082699104	-1.212157415	0.007472175
Gpi1|14751	-1.194696723	0.069242468	-1.169262994	0.040611335
9130019O22Rik|78921	-1.194815666	0.348433287	1.127818724	0.395537699
Akr1a1|58810	-1.194989215	0.030155145	-1.247384043	0.014010061
Nup188|227699	-1.195045466	0.175874983	-1.278130311	0.099649315
Ormdl2|66844	-1.195131319	0.088597131	-1.095283056	0.182930798
Fads2|56473	-1.195191337	0.102516713	-1.276730448	0.032070558
Rab23|19335	-1.19521021	0.325948719	-1.461063298	0.195340754
Klhdc8b|78267	-1.195346217	0.237005434	-1.195346217	0.237005434
Fam111a|107373	-1.19538846	0.355460589	-1.276583848	0.321544893
Mgmt|17314	-1.195396988	0.099189251	-1.195396988	0.099189251
Cnot1|234594	-1.195468044	0.152201891	-1.04140944	0.350028703
Ube2v1|66589	-1.195479576	0.084821673	-1.259828361	0.05015806
Lrba|80877	-1.195494828	0.147436954	-1.424277938	0.005855252
Gns|75612	-1.19562535	0.045981841	-1.19562535	0.045981841
Fabp2|14079	-1.195854435	0.123325194	-1.331074215	0.027856971
Pcx|18563	-1.195892016	0.066422358	-1.195892016	0.066422358
Anapc5|59008	-1.195899829	0.042230354	-1.269001523	0.011937135
C8g|69379	-1.195917863	0.169645219	-1.346959668	0.057611515
Ece1|230857	-1.19605451	0.019575069	-1.15141083	0.041558016
Ppfibp2|19024	-1.19608443	0.236008847	-1.569682922	0.023213359
Vac14|234729	-1.196110288	0.098960466	-1.126239685	0.183995857
Dhx40|67487	-1.196129069	0.03565019	-1.286988713	0.002832624
Homer3|26558	-1.196136188	0.209887096	-1.37194044	0.083115019
Taf5|226182	-1.196181843	0.275885386	-1.073217867	0.386015501
Ppp1r37|232947	-1.196272383	0.059480456	-1.196272383	0.059480456
BC031361|414072	-1.196284039	0.38805376	1.290499932	0.353081854
Cds2|110911	-1.196291637	0.145390707	-1.154701441	0.124008289
Kdm3b|277250	-1.19647653	0.268292994	-1.23345484	0.143246426
Fzd8|14370	-1.196588044	0.260407008	-1.032901006	0.453170018
Pcif1|228866	-1.196643984	0.007119188	-1.214274281	0.000877244
Hagh|14651	-1.1966753	0.017355946	-1.253101981	0.001591343
Mtif2|76784	-1.196796125	0.152501554	-1.073438839	0.315787363
Gripap1|54645	-1.196895701	0.122414636	-1.022645378	0.423753021
Ifi27l2a|76933	-1.196896571	0.3089922	1.251117691	0.205634827
Abcg5|27409	-1.196911418	0.127129338	-1.196911418	0.127129338

Mfi2|30060	-1.196948511	0.419650593	-6.462983333	0.044795474
Gsta4|14860	-1.196973832	0.23293473	-1.637411141	0.004161129
Usp12|22217	-1.197153529	0.093851172	-1.168600685	0.083333983
Eif2d|16865	-1.197324896	0.065755478	-1.14594096	0.123598983
Gm129|229599	-1.197432156	0.308157205	-1.404048116	0.142179457
Ric8|101489	-1.197492089	0.059066896	-1.156777032	0.027892204
Gzf1|74533	-1.197702137	0.221914574	-1.045847213	0.418593413
Cldn25|224250	-1.197924945	0.197087146	-1.359641894	0.030861641
Fam102a|98952	-1.197999509	0.257758375	1.061455313	0.362405805
Arhgef7|54126	-1.198086727	0.098877727	-1.413373329	0.001452021
Dchs1|233651	-1.198092305	0.11215093	-1.411519114	0.003954513
Zfp259|22687	-1.198119049	0.097073164	-1.198119049	0.097073164
Stk10|20868	-1.198120681	0.281455724	-1.198120681	0.281455724
Prmt7|214572	-1.198298655	0.126886947	-1.410998622	0.007408349
Actr1b|226977	-1.198310487	0.051298242	-1.263769199	0.012504614
Tbcc|72726	-1.198345608	0.074509238	-1.209351945	0.035903424
Aph1b|208117	-1.198407714	0.365257595	-1.908279758	0.124545333
Clasrp|53609	-1.19857534	0.15750981	1.015325722	0.452232598
Ngef|53972	-1.19857791	0.143735667	-1.467267263	0.00318287
Rnf157|217340	-1.198580712	0.248788559	-1.353216157	0.104386133
Rab28|100972	-1.198622332	0.269799763	-1.198622332	0.269799763
Atmin|234776	-1.198785853	0.301644766	1.256683971	0.168963115
Inpp5k|19062	-1.199198885	0.17536971	-1.363783837	0.055617287
1700034H14Rik|67105	-1.199204582	0.223920995	-1.1895407	0.130694279
Setd6|66083	-1.199239726	0.150011974	-1.110404687	0.27299533
Cep57|74360	-1.199243054	0.275976688	1.046117138	0.427980467
Glo1|109801	-1.199248627	0.117477098	-1.189054921	0.058373691
Mras|17532	-1.199306841	0.240025116	-1.663946403	0.004359485
Cyb561d1|72023	-1.199309814	0.126369652	-1.106777811	0.247266345
Elac2|68626	-1.199549706	0.023468428	-1.231175375	0.004549637
Safb2|224902	-1.199550057	0.063206487	-1.199550057	0.063206487
Rnf19b|75234	-1.199649026	0.015804137	-1.199649026	0.015804137
Ssr1|107513	-1.199662444	0.012922763	-1.106235125	0.032400336
Zcchc4|78796	-1.20003173	0.229872807	-1.20003173	0.229872807
Acads|11409	-1.20012453	0.105054711	-1.090341447	0.220461164
Ddr1|12305	-1.200161222	0.277107466	-1.11261791	0.376948722
Cldn2|12738	-1.200206076	0.177839571	-1.109046395	0.293685822
Adam17|11491	-1.200234125	0.083945742	-1.197900001	0.022840959
Tnk2|51789	-1.200345461	0.093351003	-1.136577812	0.117840383
Thoc5|107829	-1.200418829	0.183977431	-1.450016149	0.012149238
Ankrd55|77318	-1.200504172	0.386574664	1.285963437	0.356199084
Ppp1r16a|73062	-1.200505867	0.049491354	-1.200505867	0.049491354
Ccdc137|67291	-1.200517962	0.303227639	-1.41704079	0.157759493
Arl2|56327	-1.20054345	0.216969124	-1.293736703	0.160940308
Itpr3|16440	-1.20057285	0.341463793	-1.309647944	0.265121696
Zfp324|243834	-1.200595141	0.16932067	-1.267961656	0.073639736
F5|14067	-1.200652666	0.074039887	-1.17650333	0.061449783
Il18|16173	-1.200736554	0.291869226	1.035355234	0.454979377
Cd82|12521	-1.20078153	0.007026065	-1.20078153	0.007026065
Smu1|74255	-1.200825419	0.094335843	-1.200825419	0.094335843
Ccnc|51813	-1.200880986	0.180068059	-1.052937168	0.377054039
Tdrkh|72634	-1.200962826	0.347962857	-1.432225987	0.252744652
Upp1|22271	-1.201042196	0.313241125	-1.606892048	0.109628312

Pydc3|100033459	-1.201051956	0.414099221	1.266333228	0.381642687
Ugt2b1|71773	-1.201174127	0.188145484	-1.12743402	0.213809206
Cyp7b1|13123	-1.201186724	0.213695104	-1.467935231	0.030368451
Rab7|19349	-1.201214997	0.293162129	-1.193864286	0.295146114
Qk|19317	-1.201217139	0.32152903	1.184565721	0.27184952
Supt7l|72195	-1.201249271	0.15253735	-1.327204791	0.003820814
Mbtps2|270669	-1.201414584	0.31605982	-1.157374094	0.343895246
Wnt9a|216795	-1.201437288	0.400980188	-1.201437288	0.400980188
Zfp397|69256	-1.201527187	0.255256468	-1.082319528	0.384887871
St3gal6|54613	-1.201711576	0.322612434	-1.668311527	0.103313308
Pigs|276846	-1.201720953	0.100054933	-1.15086783	0.174236261
Zfr2|103406	-1.201901175	0.326086046	1.034606158	0.467904927
Clstn1|65945	-1.202054162	0.274347242	-1.202054162	0.274347242
Raver1|71766	-1.202067216	0.147719862	-1.202067216	0.147719862
Cox15|226139	-1.202144591	0.07799902	-1.046327188	0.301792544
Dact2|240025	-1.202162757	0.168651239	-1.195062965	0.098597186
Slc30a6|210148	-1.202207142	0.114639889	-1.202207142	0.114639889
Lactb2|212442	-1.202244916	0.265823333	1.012517221	0.480867
Bag6|224727	-1.202247367	0.014643009	-1.304044482	0.000680675
Palmd|114301	-1.202353041	0.155057162	-1.300118184	0.064109431
Dtx3l|209200	-1.202510353	0.079602557	-1.036481462	0.290615835
Mtfr1|67472	-1.202514105	0.198207772	-1.036041713	0.419871909
Txndc5|105245	-1.202624324	0.041793875	-1.356028281	0.000410427
Tgif2|228839	-1.20265012	0.352121254	-1.20265012	0.352121254
Phc1|13619	-1.202789204	0.259302634	-1.202789204	0.259302634
Aldh5a1|214579	-1.202800229	0.04130625	-1.215228562	0.006666024
Sirpa|19261	-1.202825776	0.142731921	-1.103939527	0.273298852
Fbrs|14123	-1.202961928	0.081920261	-1.202961928	0.081920261
Tlr12|384059	-1.202965084	0.228351293	1.044369319	0.343077954
Apool|68117	-1.203005172	0.163931481	-1.075242596	0.330803981
1700001L05Rik|69291	-1.20307156	0.312927701	-1.719950087	0.062676191
Siae|22619	-1.203151677	0.20877755	-1.015473688	0.463847839
Paqr4|76498	-1.203154686	0.204953343	-1.06551548	0.382287319
Rap2c|72065	-1.203163604	0.193915982	-1.101775172	0.250947593
Aldh9a1|56752	-1.203219194	0.049499164	-1.125039713	0.095133127
Myo6|17920	-1.203285917	0.254639918	-1.039682481	0.362196282
Zfp652|268469	-1.203483512	0.174010747	1.026747453	0.42514123
Lrrc61|243371	-1.203555912	0.019327682	-1.156240254	0.039352667
R3hcc1|71843	-1.203743726	0.133034089	-1.305069364	0.066998231
BC006779|229003	-1.203752245	0.162927916	-1.056278041	0.279483636
Nufip2|68564	-1.203815044	0.254855669	1.038582265	0.426216276
Gpatch2|67769	-1.203847629	0.24471579	-1.070296952	0.392809486
Cops7b|26895	-1.203873875	0.063043674	-1.203873875	0.063043674
Papola|18789	-1.203882935	0.145117529	-1.001717228	0.494712548
Gm6710|626832	-1.203922289	0.400721857	-3.583784352	0.049621606
Ntn1|18208	-1.203978221	0.089874381	-1.203978221	0.089874381
BC032203|210982	-1.204052118	0.290948229	1.052895811	0.430676087
Osgepl1|72085	-1.204239008	0.243839382	-1.139511469	0.329147486
Atf2|11909	-1.20436561	0.28447243	-1.113968636	0.347415829
Syngr1|20972	-1.20438185	0.365256196	-1.20438185	0.365256196
Zkscan3|72739	-1.204426544	0.16135261	-1.081026365	0.31099272
Dok1|13448	-1.204532575	0.148146183	-1.00268344	0.4908978
Gins4|109145	-1.20457146	0.209761312	-1.20457146	0.209761312

Mtmr10|233315	-1.204719919	0.086746081	-1.157368673	0.083018866
Acaa2|52538	-1.204758372	0.024462895	-1.108638705	0.070716409
Immt|76614	-1.204913053	0.040635264	-1.126700877	0.079272661
Sod3|20657	-1.205079673	0.104277654	-1.289907324	0.021314336
Cpped1|223978	-1.205111774	0.098535422	-1.104471308	0.204424219
Ptrh1|329384	-1.205172117	0.378774538	-1.655553952	0.197951841
Tmem198b|73827	-1.205180207	0.273636984	-1.278846691	0.112168595
Ctnnbl1|66642	-1.205234351	0.101924736	-1.285156245	0.037421695
Trap1|68015	-1.205267393	0.009264693	-1.11937119	0.023704454
Clec14a|66864	-1.205320431	0.230839425	-1.426657623	0.085189865
Bcr|110279	-1.205349387	0.030392995	-1.277744506	0.007473038
Zc3h12a|230738	-1.205352701	0.307156519	-1.840786998	0.014408614
Zfpl1|81909	-1.205424272	0.145880065	-1.178914949	0.146847956
Mtm1|17772	-1.205465035	0.264393934	-1.155382378	0.200799067
Eif2ak1|15467	-1.205618039	0.009440749	-1.258284002	0.000686923
Gk5|235533	-1.205646412	0.428701337	1	#DIV/0!
Adam4|11498	-1.205646412	0.428701337	1	#DIV/0!
Fat3|270120	-1.205646412	0.428701337	1	#DIV/0!
Alox5ap|11690	-1.205682819	0.329190502	-2.149619668	0.011402638
Rbm19|74111	-1.205836642	0.039604323	-1.140876066	0.021174933
Utp14a|72554	-1.205983292	0.185404336	-1.097110989	0.317155635
Rad50|19360	-1.206102417	0.171195067	-1.419252778	0.024781306
Commd8|27784	-1.206166831	0.185358327	-1.520839403	0.011828274
Mospd1|70380	-1.206249051	0.351130815	1.171957295	0.369950151
Foxk1|17425	-1.206350996	0.195769693	-1.206350996	0.195769693
Synpo|104027	-1.20639552	0.318676914	1.336553194	0.139326727
Gnai2|14678	-1.206449239	0.029362517	-1.146018277	0.060419899
Atp6v1g2|66237	-1.206470215	0.289454376	-1.286436303	0.168733536
Zbtb4|75580	-1.206536119	0.274773276	-1.241466536	0.199316993
Rnf113a1|69942	-1.206551178	0.369057293	-1.84378515	0.168005515
Mbp|17196	-1.206734035	0.383413742	1.059485161	0.460585299
Bdp1|544971	-1.206755472	0.249548607	1.051387231	0.385105757
Pdpk1|18607	-1.206981002	0.320472353	1.171805981	0.301017732
Acd|497652	-1.207018183	0.1168505	-1.39857924	0.009958294
Zfp664|269704	-1.207106673	0.201070814	-1.350968812	0.106274311
Tubgcp2|74237	-1.207156718	0.139532642	-1.207156718	0.139532642
Rxra|20181	-1.207159944	0.020361796	-1.275960633	0.00072001
Gcdh|270076	-1.207285155	0.006317421	-1.236830901	0.003498664
Mcmbp|210711	-1.207423175	0.105918298	-1.412936278	0.006680545
Tecr|106529	-1.207473388	0.074407175	-1.232484087	0.018255482
Dennd4c|329877	-1.207691506	0.246561124	-1.207691506	0.246561124
Gna12|14673	-1.20775445	0.261194248	-1.033531326	0.454937564
Rfx1|19724	-1.20787071	0.203086805	-1.04352612	0.413270426
Erc2|238988	-1.207874294	0.214727575	-1.310709807	0.15424042
Camsap2|67886	-1.207968152	0.295852083	-1.207968152	0.295852083
Pla2g4b|211429	-1.207986724	0.250886347	1.171827683	0.141622355
Sgsm2|97761	-1.208017895	0.156556243	-1.341877931	0.015589908
Tagap1|380608	-1.208047913	0.222538168	-1.208047913	0.222538168
Slc38a2|67760	-1.208248288	0.277844536	1.014378765	0.471657538
Sdc1|20969	-1.208275419	0.106797309	-1.209751112	0.066926756
Crk|12928	-1.208450413	0.192529792	-1.064420819	0.375877648
Fam63a|75007	-1.208527603	0.0003813	-1.181784927	0.000524295
Mtmr12|268783	-1.208551244	0.203521009	-1.208551244	0.203521009

Angptl2|26360	-1.20864378	0.175402543	-1.20864378	0.175402543
Cbs|12411	-1.208723959	0.102615389	-1.184552799	0.046465949
Atpaf2|246782	-1.208781495	0.058458172	-1.127263007	0.063495389
Lin54|231506	-1.208832191	0.251912256	-1.043444479	0.439684559
Gab2|14389	-1.208887789	0.20311939	-1.009842058	0.474568887
Nab1|17936	-1.209069588	0.037365553	-1.209069588	0.037365553
Fam171a1|269233	-1.209079122	0.125969008	-1.107489006	0.25056291
Pppde2|28075	-1.209129104	0.022354774	-1.166018547	0.047188092
Echdc2|52430	-1.209159783	0.004841697	-1.162253174	0.008082736
Tmem63a|208795	-1.209167694	0.100630463	-1.26767363	0.060340617
Prtn3|19152	-1.209186004	0.431203253	3.53972	0.173296754
Wdr6|83669	-1.209189976	0.128558199	-1.11504191	0.247609819
Nucb1|18220	-1.209201563	0.038259813	-1.165560301	0.075680503
Vps45|22365	-1.209283884	0.18395394	-1.209283884	0.18395394
Gp5|14729	-1.209306875	0.385047983	-2.497414141	0.097650894
Synj2bp|24071	-1.209467086	0.209613368	-1.098930252	0.23037566
Spnb2|20742	-1.209527736	0.291261726	-1.046223335	0.44059931
Xk|22439	-1.209557179	0.228790494	-1.485929168	0.051647396
Pggt1b|225467	-1.209671264	0.293223496	1.045094715	0.444992357
Commd3|12238	-1.209737565	0.045094753	-1.29529545	0.010975828
AI661453|224833	-1.209754635	0.096351677	-1.030903704	0.376009147
Slco2b1|101488	-1.210034764	0.012955422	-1.211407302	0.001858194
Tob1|22057	-1.210100162	0.248483217	-1.414403345	0.124931619
Cdk5|12568	-1.210115383	0.083503238	-1.237803182	0.082583618
Tmem81|74626	-1.210195706	0.277815404	-1.031062267	0.445784309
Pim1|18712	-1.210215556	0.29899093	-1.010072561	0.487216288
Epb4.1l5|226352	-1.210232241	0.096137102	-1.274069018	0.065525425
Foxa2|15376	-1.210237217	0.190201279	-1.394635718	0.066092991
Acot11|329910	-1.210474969	0.299940126	1.043636756	0.450484418
Lmbr1l|74775	-1.210487662	0.180158837	-1.49074474	0.018273065
Fndc4|64339	-1.210633805	0.0663147	-1.210633805	0.0663147
Cln5|211286	-1.210640349	0.173122475	-1.076959282	0.331065578
Rfng|19719	-1.210766989	0.108177541	-1.367447766	0.013347167
Kif2a|16563	-1.21082552	0.296748297	-1.198998743	0.263397276
Ubox5|140629	-1.210931367	0.199551892	-1.538716821	0.017746653
Pou2f2|18987	-1.211012735	0.345794347	-1.211012735	0.345794347
Spice1|212514	-1.211052888	0.348167456	-1.046237458	0.454637711
Mthfd1|108156	-1.211255365	0.006647062	-1.158448327	0.010433223
Srsf5|20384	-1.211270815	0.305587276	-1.195035922	0.301653816
Ndufa12|66414	-1.211353354	0.046419364	-1.209128316	0.008648542
Tug1|544752	-1.211370969	0.192418398	1.051849374	0.365389802
Wars2|70560	-1.211379071	0.079175338	-1.32431397	0.010009651
Nr6a1|14536	-1.21152842	0.126274063	-1.21152842	0.126274063
Vti1b|53612	-1.211584353	0.038847146	-1.341811639	0.001049009
Hdac10|170787	-1.211608642	0.060688347	-1.3952213	3.35929E-06
Car11|12348	-1.21161033	0.308657706	-1.466619557	0.159072296
Pacsin2|23970	-1.21162072	0.0023583	-1.170500639	0.002442873
Rab3d|19340	-1.211628318	0.131795599	-1.211628318	0.131795599
Rasgrp2|19395	-1.211708562	0.09251229	-1.190325133	0.083411676
Ikbkb|16150	-1.211866839	0.052705642	-1.211866839	0.052705642
Nol7|70078	-1.211898143	0.11820757	-1.211898143	0.11820757
Psmd14|59029	-1.2119204	0.046830045	-1.162218829	0.094077201
Cdc26|66440	-1.212023963	0.09513547	-1.212023963	0.09513547

Rell1|100532	-1.212166845	0.076389875	-1.354230169	0.008234538
Uimc1|20184	-1.212167696	0.091695407	-1.212167696	0.091695407
Six5|20475	-1.212202257	0.128896922	-1.367852758	0.02865872
Hfe|15216	-1.212373773	0.108013296	-1.274248688	0.064493043
Zfp93|22755	-1.21245766	0.310354103	-1.21245766	0.310354103
Adcy6|11512	-1.21246184	0.233507056	1.005140722	0.489905361
Ccny|67974	-1.212496655	0.176883442	-1.347965869	0.089273246
Pnn|18949	-1.212503939	0.26759611	-1.044173519	0.405977257
Supt5h|20924	-1.212618311	0.00920864	-1.173963067	0.020352683
Trpm7|58800	-1.212656911	0.261464778	-1.106813177	0.328097822
Prkca|18750	-1.212684491	0.282592911	1.187088124	0.193746731
Eng|13805	-1.212733961	0.018880941	-1.271593505	0.006495318
Dus3l|224907	-1.212834731	0.107186328	-1.319072938	0.0200047
Rbm10|236732	-1.212883457	0.076619038	-1.212883457	0.076619038
Gtpbp8|66067	-1.213048875	0.332532595	-1.213048875	0.332532595
Angptl3|30924	-1.213120553	0.129442909	-1.482167595	0.001227506
Nsun4|72181	-1.21313319	0.196896137	-1.090318976	0.349926491
Ttc4|72354	-1.213171816	0.03049696	-1.162735786	0.063428879
Pisd-ps1|236604	-1.213189615	0.04491182	-1.085294027	0.170644466
St3gal1|20442	-1.213295241	0.011872224	-1.125752437	0.03627403
Ltb|16994	-1.21330913	0.314447679	-2.110368957	0.006499867
Tjp2|21873	-1.213413814	0.223885604	-1.042618113	0.428954156
Aig1|66253	-1.213453335	0.148579059	-1.074658383	0.315261561
Msl3|17692	-1.213563598	0.069708761	-1.129153602	0.14253222
Crat|12908	-1.213617763	0.033451214	-1.213617763	0.033451214
Ints9|210925	-1.213648015	0.076117882	-1.345131408	0.008833204
Eral1|57837	-1.213653855	0.043815817	-1.284975037	0.000428756
Ap3d1|11776	-1.213697679	0.08680942	-1.201402702	0.0287065
Zfp692|103836	-1.213716556	0.276379877	-1.014476387	0.482192794
Clec4g|75863	-1.213826576	0.0565253	-1.161436827	0.107753985
Trappc6b|78232	-1.213834552	0.13904926	-1.189458034	0.076359371
Gcat|26912	-1.213883386	0.031449657	-1.354455407	0.000869519
Nfat5|54446	-1.213883687	0.259137526	1.086311857	0.291805968
9030624J02Rik|71517	-1.213888583	0.07249982	-1.287218231	0.006413499
Kdr|16542	-1.213932748	0.273824637	-1.00186628	0.497578858
Hpd|15445	-1.21398026	0.12596515	-1.272975833	0.099407972
Ttc28|209683	-1.214002751	0.280317241	-1.01565341	0.477909284
Zfp654|72020	-1.214016565	0.256161451	1.124931518	0.296056356
Uba1|22201	-1.214114945	0.002302856	-1.138252999	0.001565539
Wwp1|107568	-1.214187721	0.246169955	-1.050535963	0.430059381
Arrdc3|105171	-1.214221846	0.296751047	-1.214221846	0.296751047
Crebzf|233490	-1.214338479	0.335449862	-1.187016581	0.313958493
Vps37d|194309	-1.21436319	0.340043235	-1.396366265	0.232822414
Gm16861|100503704	-1.214366268	0.07662164	-1.214366268	0.07662164
Pde3b|18576	-1.214416509	0.271855951	-1.422122968	0.158870438
Taok3|330177	-1.214451361	0.097377742	-1.082740163	0.188077447
Ikbke|56489	-1.214524067	0.23652506	-1.1780625	0.250243975
Eif2b2|217715	-1.214539053	0.070110166	-1.214539053	0.070110166
Nradd|67169	-1.2145467	0.398291533	-1.256171314	0.404473651
Poli|26447	-1.21460017	0.260759587	-1.015390153	0.474842722
Fbxl15|68431	-1.214671668	0.253268906	1.09405178	0.356937718
Cd1d1|12479	-1.214728944	0.102290512	-1.295066748	0.040952438
Tmem19|67226	-1.214741219	0.01100407	-1.214741219	0.01100407

Timeless|21853	-1.21483876	0.22352654	-1.057098461	0.410191178
Lamb2|16779	-1.214889441	0.157002467	-1.115769993	0.284123071
Stim1|20866	-1.214921096	0.16006607	1.019036361	0.443836208
Mettl17|52535	-1.214954551	0.192897756	1.061722295	0.32919405
Entpd5|12499	-1.215058269	0.090830203	-1.215058269	0.090830203
Txlng|353170	-1.215150818	0.276299971	-1.380347631	0.190664693
Rprd1b|70470	-1.21518862	0.124453258	-1.325165847	0.030255693
Ep400|75560	-1.215411029	0.184317722	-1.137281215	0.206135639
2010107G23Rik|69894	-1.215527444	0.295662852	-1.215527444	0.295662852
Acp2|11432	-1.215581957	0.003880081	-1.16590204	0.005489821
Cyp1a2|13077	-1.215640993	0.081539631	-1.317980845	0.027474126
Mrps30|59054	-1.21566223	0.136225271	-1.483318527	0.006627897
Dera|232449	-1.215889619	0.008204346	-1.116289015	0.00980253
Irf3|54131	-1.216011889	0.043933748	-1.130800613	0.077090482
1300017J02Rik|71775	-1.216016237	0.027386853	-1.348970845	0.000636484
Rnpepl1|108657	-1.216187214	0.011567771	-1.276080497	0.000629623
Coq9|67914	-1.216237005	0.006984176	-1.201398733	0.001709679
1100001G20Rik|66107	-1.216424678	0.172322505	-1.389031608	0.013536814
Eci2|23986	-1.2165022	0.040628476	-1.297798415	0.003266945
1110021J02Rik|68597	-1.2165059	0.227877187	-1.102021738	0.330379779
Zfp334|228876	-1.216520972	0.343493297	-2.259092194	0.029287641
Cstf1|67337	-1.216524314	0.096750567	-1.414524805	0.004145206
Cbx5|12419	-1.216528347	0.113647992	-1.028267448	0.39577872
Rnf135|71956	-1.216594024	0.09657677	-1.30756431	0.013797404
Zcchc11|230594	-1.216621361	0.20536792	-1.10535092	0.330532226
Dhrs3|20148	-1.216873916	0.098306161	-1.21850132	0.025829645
Dnmbp|71972	-1.216949204	0.274643011	1.000203617	0.499739763
Eefsec|65967	-1.216965493	0.093270733	-1.10960878	0.193368102
Bola1|69168	-1.216997515	0.147051708	-1.216997515	0.147051708
Ppp1r9b|217124	-1.217304445	0.148979658	-1.09021056	0.301870639
Hdc|15186	-1.217333938	0.221314723	-1.017445636	0.466816345
Tecpr2|104859	-1.217366916	0.128888666	-1.343535153	0.051227366
Ddx18|66942	-1.217579275	0.11915836	-1.200450795	0.055293007
Mllt1|64144	-1.217591104	0.093671261	-1.256750367	0.076341416
Mkrn2|67027	-1.217595565	0.217787728	-1.031975216	0.442142969
Il6ra|16194	-1.217681282	0.30315848	1.108617468	0.370664978
Prkag2|108099	-1.217759467	0.15600458	-1.34007732	0.046638575
Pofut2|80294	-1.217842514	0.06276877	-1.217842514	0.06276877
Zfp276|57247	-1.217913216	0.089238939	-1.280231712	0.048748493
Usp19|71472	-1.217966275	0.112585653	-1.327420185	0.023147803
Myh9|17886	-1.218036357	0.097549609	-1.101977991	0.202881516
Pck2|74551	-1.218179676	0.111872623	-1.377251189	0.017843302
Epb4.1|269587	-1.218427686	0.211794524	1.093589896	0.269317404
B630019K06Rik|102941	-1.218644764	0.419490267	-6.580133333	0.095073527
Trappc1|245828	-1.218693789	0.093465146	-1.12878399	0.188703654
Dock6|319899	-1.218789621	0.080416184	-1.050636522	0.289940027
Iqce|74239	-1.218806222	0.261048071	1.11374154	0.33471909
Med16|216154	-1.21888457	0.01274458	-1.116015194	0.033722574
Nf1|18015	-1.219105274	0.228552242	-1.460261917	0.082621991
Nmi|64685	-1.219190262	0.149187539	-1.010225256	0.471745909
Fam83h|105732	-1.219191527	0.043196782	-1.219191527	0.043196782
Zfyve21|68520	-1.21919413	0.158696237	-1.093953564	0.299719468
Gpr4|319197	-1.219463256	0.289271595	-2.006499212	0.001835595

Dgkz|104418	-1.219469125	0.023175533	-1.262399668	0.01593673
2810474O19Rik|67246	-1.219540816	0.077394479	-1.39858245	0.006130199
Ptges|64292	-1.219668276	0.384879513	-2.099930691	0.17960658
Bcar3|29815	-1.219783426	0.101470343	-1.031677193	0.383220668
Gga1|106039	-1.219804684	0.004311434	-1.219804684	0.004311434
Fadd|14082	-1.219867493	0.073924565	-1.334205978	0.017169719
Prrt1|260297	-1.21989608	0.387191095	-2.15097535	0.178555935
Pdxdc1|94184	-1.219993764	0.036854706	-1.174317385	0.075056865
Ube2q2|109161	-1.219999547	0.166933602	-1.219999547	0.166933602
Cyp27a1|104086	-1.220041474	0.062672854	-1.230279067	0.019023305
Ubr4|69116	-1.220095406	0.016055469	-1.254485303	0.00182893
Lmo7|380928	-1.220173738	0.243055206	-1.183523132	0.21553436
Acap1|216859	-1.220181392	0.301838337	-1.637522765	0.107525861
Hist2h2aa1|15267	-1.220200161	0.203985722	-1.351600864	0.089718483
Cutc|66388	-1.220230122	0.128539243	-1.384240137	0.028030292
Acox3|80911	-1.220388473	0.156595949	-1.11470963	0.276770064
Pyroxd2|74580	-1.220390277	0.214680203	-1.212216528	0.175996803
Znrd1as|76416	-1.220429785	0.114638078	-1.305934167	0.069373807
Pdlim2|213019	-1.220831666	0.286619931	-1.5405671	0.126581665
Neurl3|214854	-1.220905304	0.122675549	-1.350865526	0.044915644
Gabpb2|213054	-1.220962279	0.250095736	1.131899942	0.274551023
Inadl|12695	-1.221012688	0.244834243	-1.332650987	0.088543402
Arhgap17|70497	-1.221268356	0.184273325	-1.082639502	0.351375127
Atxn7l2|72522	-1.22128215	0.193377037	-1.22128215	0.193377037
Cyp2c37|13096	-1.221334236	0.148253961	-1.260285186	0.062107414
Prelp|116847	-1.221478805	0.074184501	-1.415576999	0.003659502
Syne2|319565	-1.221755249	0.198295735	-1.223885703	0.169553484
Syk|20963	-1.221761189	0.159698667	-1.39497739	0.050593515
Sbf2|319934	-1.221768673	0.175701381	-1.221768673	0.175701381
Tor1aip1|208263	-1.2219039	0.120221502	-1.110645272	0.244420039
Tmem188|382030	-1.222118468	0.22476367	-1.364907434	0.104097114
Midn|59090	-1.222302487	0.113436062	-1.245948806	0.026268787
Sugp2|234373	-1.222372882	0.182302239	-1.510825672	0.011078741
BC024479|235184	-1.222427966	0.305200449	-1.316385067	0.221736797
Stox2|71069	-1.222851561	0.361497103	-1.222851561	0.361497103
Smad2|17126	-1.222874488	0.12309947	-1.222874488	0.12309947
Pygl|110095	-1.223139344	0.051636384	-1.064251696	0.186943411
Tmem106c|380967	-1.223181032	0.181717839	-1.507163671	0.012793293
Chst11|58250	-1.223206359	0.350994719	-2.656775654	0.009368805
Stard10|56018	-1.223332681	0.149146597	-1.179534963	0.163270334
Cd36|12491	-1.223386422	0.141662669	-1.087422654	0.296349902
Dguok|27369	-1.223525491	0.135665433	1.008420789	0.470035388
Clpb|20480	-1.223955038	0.057806117	-1.163180851	0.112136973
Zfp777|72306	-1.224004802	0.067694049	-1.321960003	0.022810555
2310061I04Rik|69662	-1.224233206	0.005557349	-1.19099655	0.012257325
9430015G10Rik|230996	-1.224299176	0.142034061	-1.093897089	0.281476556
Slc13a2|20500	-1.224354742	0.265191272	-1.408954092	0.08534996
Ccdc91|67015	-1.224446009	0.177877423	-1.224446009	0.177877423
Qprt|67375	-1.224454232	0.021277975	-1.182590736	0.04479508
Ncoa7|211329	-1.224628607	0.404983886	1.707719016	0.288410395
Evi2a|14017	-1.224667681	0.293501811	-1.224667681	0.293501811
Ppp1r26|241289	-1.224866667	0.433068168	1	#DIV/0!
Smad3|17127	-1.224871152	0.161540358	-1.083541494	0.32774823

Ccbl1|70266	-1.224918545	0.035877413	-1.217857173	0.015698764
Wdr93|626359	-1.22492331	0.302150715	-1.854794213	0.040140729
Rnf216|108086	-1.224925045	0.151944305	1.017766674	0.425914903
Mvd|192156	-1.225013012	0.263605135	-1.482336773	0.127384144
Slfn8|276950	-1.225099274	0.380032667	-3.972802444	0.00311299
Dhrs13|70451	-1.225163614	0.178704403	-1.607348621	0.001384208
Hsd17b2|15486	-1.225218086	0.002300702	-1.191340601	0.005165222
Gtf3c6|67371	-1.225263005	0.148851559	-1.356533158	0.035463955
Etv3|27049	-1.225295158	0.327012139	-1.24189808	0.281039705
Pard3|93742	-1.225304283	0.027070483	-1.165488885	0.056529234
Slc35e2|320541	-1.225370427	0.187704336	1.055422498	0.363403772
H2-Ke6|14979	-1.225421633	0.111890979	-1.369804917	0.029284457
N4bp1|80750	-1.225444825	0.053791933	-1.287918875	0.032525974
Piga|18700	-1.225515784	0.31319329	1.111122148	0.388279679
1110008F13Rik|67388	-1.225667967	0.046919598	-1.323337783	0.001531381
Gca|227960	-1.225681217	0.347940711	1.440670469	0.07828063
Wdr59|319481	-1.225903998	0.13572511	-1.225903998	0.13572511
Mrpl35|66223	-1.225922568	0.056814898	-1.061416628	0.213973107
Beta-s|100503605	-1.225924622	0.292859308	-1.225924622	0.292859308
Blk|12143	-1.225926987	0.414148685	3.216934969	0.094488886
Trib3|228775	-1.226057381	0.236269824	-1.565437781	0.045324374
Sh3bgr|50795	-1.226166243	0.427473343	-4.58098	0.070596339
Setd1a|233904	-1.226221041	0.188142065	-1.221729916	0.166386223
Stk24|223255	-1.226249196	0.227603601	-1.030080102	0.451093582
Crym|12971	-1.226330137	0.379489652	-3.34685095	0.02323403
Adap1|231821	-1.2263943	0.347002706	-2.839175958	0.000727097
2200002D01Rik|72275	-1.226416029	0.204621545	-1.034731428	0.432613312
Adipor2|68465	-1.2264757	0.036385936	-1.150486711	0.068894934
Fam193a|231128	-1.22655835	0.154521871	-1.348435303	0.050681228
Emilin1|100952	-1.22658244	0.071185228	-1.419353553	0.002920361
Fubp3|320267	-1.226674852	0.268573206	-1.065625575	0.38854002
Gata2|14461	-1.226776828	0.272540474	-1.074771835	0.38114776
Sec23b|27054	-1.226858048	0.179244513	-1.226858048	0.179244513
Tcof1|21453	-1.226913704	0.073312899	-1.12466512	0.15010731
Pik3c2b|240752	-1.226937277	0.094716149	-1.299200453	0.046505235
Bckdk|12041	-1.226944568	0.190242324	-1.534530579	0.01286921
Apoe|11816	-1.226975618	0.187828948	-1.432347021	0.062800419
Irak1|16179	-1.227015829	0.114412154	-1.102942382	0.240005178
Als2cr4|381259	-1.227089708	0.256063515	-1.489669122	0.115360279
Gm15760|100042948	-1.22723382	0.272934537	-1.22723382	0.272934537
Taf5l|102162	-1.227297415	0.138821236	-1.227297415	0.138821236
N6amt2|68043	-1.227318455	0.297982744	-1.227318455	0.297982744
Zkscan14|67235	-1.227348399	0.20563074	-1.41474716	0.095073605
Pdilt|71830	-1.227420426	0.108897582	-1.309675594	0.025946763
Tmppe|100504715	-1.227429181	0.174308784	-1.227429181	0.174308784
Mag|17136	-1.227592811	0.380506968	-1.227592811	0.380506968
Nfic|18029	-1.22768007	0.069955385	-1.218548234	0.003519932
F11|109821	-1.227729505	0.129804002	-1.227729505	0.129804002
Nktr|18087	-1.227764996	0.24884636	-1.220578895	0.284726212
Galnt1|14423	-1.227785094	0.001861149	-1.26425066	0.000400677
2810047C21Rik1|72716	-1.227792455	0.4209036	-1.08282	0.173296754
Clock|12753	-1.227895488	0.30891272	1.21918599	0.114931781
Me2|107029	-1.228000342	0.306236315	-1.573100557	0.155445584

Nav2|78286	-1.228152215	0.113979523	-1.087845283	0.242155379
Wdr62|233064	-1.228184772	0.276790487	-1.683470047	0.055854192
Prox1|19130	-1.22823914	0.295670678	-1.042744305	0.428476614
Eya3|14050	-1.228244912	0.052210196	-1.228244912	0.052210196
B330016D10Rik|320456	-1.228311139	0.267257657	-1.228311139	0.267257657
Aldh2|11669	-1.228386962	0.053896184	-1.192084478	0.00091429
Tmem8b|242409	-1.228388681	0.199672671	-1.228388681	0.199672671
Rgs16|19734	-1.228433818	0.261305289	-1.009148892	0.487933492
4930523C07Rik|67647	-1.228484351	0.143514135	-1.311246832	0.022993528
Stard13|243362	-1.228612426	0.249466648	1.06716432	0.352366241
Lrrc59|98238	-1.22862008	0.030777199	-1.147083212	0.058348273
Gcnt2|14538	-1.228630199	0.18940503	-1.049057004	0.400804745
Dusp16|70686	-1.228674804	0.169695372	-1.151566487	0.259416664
Hmg20b|15353	-1.228681112	0.104520068	-1.228681112	0.104520068
Slc5a6|330064	-1.228691438	0.182362883	-1.439568524	0.05480554
Col18a1|12822	-1.228699508	0.004848466	-1.269159114	0.001321103
Specc1|432572	-1.228703739	0.234034214	-1.479681788	0.092752752
Adamtsl1|77739	-1.228851305	0.404306894	-4.537055556	0.078490172
Gsdmd|69146	-1.228859179	0.017587278	-1.17067947	0.036409867
Rfx5|53970	-1.22889611	0.319162982	-1.22889611	0.319162982
Sh3glb2|227700	-1.22903067	0.074968647	-1.239222902	0.034916819
Isoc2b|67441	-1.229054675	0.123958515	-1.170954617	0.141284246
L2hgdh|217666	-1.229112254	0.212189595	-1.363375199	0.139193226
Zfp385a|29813	-1.229234396	0.115895453	-1.229234396	0.115895453
Upf2|326622	-1.229468595	0.214580179	-1.089098461	0.360494642
Ambp|11699	-1.229516161	0.097551973	-1.311797622	0.058971357
Lgals3bp|19039	-1.229544222	0.008270465	-1.339747881	0.000134051
Ppox|19044	-1.229591817	0.154334448	-1.057415012	0.34628906
B230208H17Rik|227624	-1.229709045	0.048341105	-1.308479518	0.020411228
Zbtb8a|73680	-1.229935353	0.151053895	-1.229935353	0.151053895
Twsg1|65960	-1.230069558	0.242645353	-1.230069558	0.242645353
Gsk3b|56637	-1.230088363	0.249490551	1.013108762	0.468307955
Pik3cg|30955	-1.230120243	0.362168543	1.178060395	0.396752683
B3galt4|54218	-1.230227668	0.305301696	1.061564844	0.438569312
Zfp148|22661	-1.23032275	0.165097801	-1.135529645	0.270432959
Epha4|13838	-1.230366683	0.377641563	1.318577386	0.348231523
Rfx4|71137	-1.230409066	0.324272556	1.110261349	0.405051306
Tmx4|52837	-1.230456117	0.283631384	-1.329091184	0.190335722
Olfml1|244198	-1.230498606	0.173771175	1.038372299	0.401776435
Rptor|74370	-1.230525389	0.084412446	-1.116594047	0.173701805
Nudt7|67528	-1.230624657	0.04309955	-1.167351487	0.088547933
Ebag9|55960	-1.230624984	0.118138192	-1.334796335	0.037530283
Galt|14430	-1.230739371	0.07753969	-1.115287041	0.152354287
Rg9mtd3|69934	-1.230895722	0.265219565	-1.230895722	0.265219565
Slc19a2|116914	-1.230947755	0.23322913	-1.040314461	0.440838506
Prps2|110639	-1.23097527	0.212498978	-1.446220642	0.088451143
Zfp780b|338354	-1.230999586	0.227446024	-1.495472884	0.078359819
Hivep1|110521	-1.231020238	0.248669543	1.129456463	0.298392806
3110062M04Rik|78412	-1.231442035	0.2882876	-1.32988524	0.211689951
Abcg2|26357	-1.231544592	0.246519798	-1.281757195	0.16542273
Iyd|70337	-1.231560558	0.072914876	-1.231560558	0.072914876
Gnai3|14679	-1.231565271	0.26322752	-1.076132361	0.397900682
Olig1|50914	-1.231642438	0.337986132	-1.189259879	0.367199444

Plekha4|69217	-1.231661499	0.373539609	1.586432029	0.185997352
Prr12|233210	-1.231693704	0.030409953	-1.231693704	0.030409953
Tnks1bp1|228140	-1.23190513	0.014293532	-1.297084411	0.001061267
Itgb3bp|67733	-1.231979683	0.407398993	1.282144901	0.403677687
Prkdc|19090	-1.232068761	0.244108271	1.159484452	0.211683066
Galm|319625	-1.232069185	0.000888012	-1.155623922	0.000168982
Trp53i13|216964	-1.232147951	0.212906632	-1.172739812	0.248854487
Rab8a|17274	-1.23217037	0.003515009	-1.249795779	0.004532928
Gmfg|63986	-1.232170541	0.258141182	-1.884073956	0.005352505
Dmtf1|23857	-1.232235717	0.097442933	-1.323678307	0.025267772
Cyb5rl|230582	-1.232370465	0.137130216	-1.175516719	0.158633765
Slc38a1|105727	-1.232560776	0.229922142	-1.425267824	0.125094982
Slc16a13|69309	-1.232714147	0.227747409	-1.585605941	0.03524665
Magi3|99470	-1.232733441	0.302309297	1.322902887	0.142241819
Slc16a5|217316	-1.23275569	0.006006008	-1.335037099	0.000163371
Ift27|67042	-1.232759943	0.294230708	1.294101001	0.052635624
Idh1|15926	-1.232866364	0.064793208	-1.133486141	0.129002809
Dnajc13|235567	-1.232868733	0.079600571	-1.130441149	0.163394821
Nxt2|237082	-1.232927218	0.246876255	-1.201761141	0.227119975
4930429B21Rik|67576	-1.232990753	0.409410548	-2.76078	0.173296754
4930578N16Rik|75051	-1.232990753	0.409410548	-2.76078	0.173296754
1700024P16Rik|242594	-1.233214066	0.298941865	-1.415044725	0.138435679
Gigyf2|227331	-1.233259453	0.187894441	-1.034491434	0.422143615
Map2k6|26399	-1.233308973	0.26840616	-1.807360969	0.02664825
Pla2r1|18779	-1.233319511	0.368616021	-2.156249	0.141354078
Sf3a1|67465	-1.233524074	0.075337807	-1.117114925	0.153373966
Srsf4|57317	-1.233587228	0.042962523	-1.16043144	0.088255475
Kcnn1|84036	-1.233588296	0.111526043	-1.363143104	0.041647344
Pyhin1|236312	-1.233686769	0.325082022	1.091711219	0.424141403
Cited2|17684	-1.233885271	0.215963579	-1.288752819	0.156416898
Capn5|12337	-1.233916358	0.237069963	-1.233916358	0.237069963
Myst1|67773	-1.233957751	0.116885723	-1.31042036	0.064529452
Tnip1|57783	-1.234032672	0.089765155	-1.342861055	0.036517085
Dyrk1a|13548	-1.234045933	0.131787987	-1.36203267	0.060992174
2310015A10Rik|69548	-1.234087674	0.240328602	-1.106159198	0.363711256
Cyp4v3|102294	-1.234232425	0.073743538	-1.250983204	0.036993172
Txnrd3|232223	-1.234246409	0.236344263	-1.496246279	0.026983799
Syde1|71709	-1.234275575	0.134529969	-1.234275575	0.134529969
Psd3|234353	-1.234315328	0.266630214	1.068589792	0.392600693
Ppp1ca|19045	-1.234315858	0.020256838	-1.282904538	0.012774795
Ap1g1|11765	-1.23454036	0.169408181	1.036281068	0.404307486
Ttc37|218343	-1.234584024	0.070905273	-1.237307747	0.028272343
Zfhx3|11906	-1.234739542	0.095558449	-1.033681688	0.374885705
Nat6|56441	-1.234879395	0.060120019	-1.130856574	0.113084569
Ybx1|22608	-1.234923243	0.007057006	-1.253704671	0.001965477
Laptm5|16792	-1.235148349	0.047929481	-1.281668837	0.00321758
Tbc1d20|67231	-1.235222979	0.222603699	-1.077264054	0.381184704
Sntb2|20650	-1.235304633	0.283874925	1.223684853	0.2320602
Mink1|50932	-1.23537809	0.123886787	-1.182699669	0.145343312
Sall2|50524	-1.235522558	0.301011423	-1.235522558	0.301011423
Snapc5|330959	-1.235635274	0.093449459	-1.235635274	0.093449459
Gm8994|668137	-1.235675617	0.26039119	1.182841442	0.226476493
Emp3|13732	-1.235696106	0.336173916	-1.131276815	0.359715189

Cnot10|78893	-1.235797595	0.058731517	-1.363812574	0.007952671
Aldob|230163	-1.235855884	0.145380403	-1.235855884	0.145380404
Trappc8|75964	-1.236074844	0.309383289	1.346323377	0.146998955
Paip2|67869	-1.236115335	0.04628243	-1.151693797	0.090923569
Lmf1|76483	-1.236229618	0.124036432	-1.236229618	0.124036432
Cap1|12331	-1.236304055	0.036493121	-1.320171455	0.011636249
Dcxr|67880	-1.236628237	0.069851038	-1.348011017	0.021167871
Itpr2|16439	-1.236702204	0.076552752	-1.062580934	0.289453457
Agxt2|268782	-1.236805375	0.187038556	-1.070849084	0.373710029
Adamtsl5|66548	-1.23680843	0.294314628	-1.23680843	0.294314628
Msn|17698	-1.236826727	0.268090524	1.000912688	0.498869518
Pde7b|29863	-1.237066394	0.368708421	-1.548979625	0.242155324
Myo18a|360013	-1.237083025	0.023473418	-1.391312484	0.00026879
Hyal3|109685	-1.237336321	0.379340985	1.230602636	0.395387796
Tmem63b|224807	-1.237386313	0.008017329	-1.233389695	0.000709022
Zfp526|210172	-1.237408176	0.171039008	-1.133419381	0.293374088
Lrp11|237253	-1.237424164	0.333604971	-1.237424164	0.333604971
Hist1h4c|319155	-1.237431459	0.400922831	1	#DIV/0!
Hist1h4j|319159	-1.237431459	0.400922831	1	#DIV/0!
Coq2|71883	-1.237488479	0.098349513	-1.404340732	0.014363421
Wasf2|242687	-1.237541953	0.221260331	1.098349942	0.280548761
Tlr5|53791	-1.237542507	0.338306476	-1.705426404	0.167446261
Heatr7a|223658	-1.237649348	0.090485586	-1.128815747	0.18662626
Stk16|20872	-1.237656364	0.086163804	-1.10731633	0.168369245
Fbxw8|231672	-1.237940405	0.01479329	-1.176377983	0.029757516
Ppil4|67418	-1.237990356	0.123605926	-1.399078402	0.035221789
Rps6kb2|58988	-1.238074547	0.086108073	-1.155005895	0.169709058
Pfkfb2|18640	-1.23808583	0.322583973	-1.044624193	0.440413763
Ccl5|20304	-1.238106804	0.231472639	-1.433283017	0.070091878
Ift88|21821	-1.238148451	0.239159914	-1.242052544	0.203704165
Ugt1a2|22236	-1.238229607	0.279529383	-1.0414596	0.42639597
Stxbp4|20913	-1.238235718	0.282278968	-2.009497975	0.004463408
Dnm2|13430	-1.238365457	0.005440931	-1.29964043	0.001002626
Gmcl1|23885	-1.238438666	0.243012215	-1.14969036	0.329893223
Wdr36|225348	-1.238444393	0.143058034	-1.141846039	0.245638351
Pusl1|433813	-1.238701782	0.165312131	-1.238701782	0.165312131
Tcirg1|27060	-1.238704502	0.018088776	-1.238704502	0.018088776
Izumo4|71564	-1.238737879	0.221838052	-1.499150494	0.080156681
Tmem128|66309	-1.238738329	0.045932871	-1.238738329	0.045932871
Oxsm|71147	-1.238794502	0.144660588	-1.267209583	0.096956141
Gm3219|100041231	-1.238846032	0.229389565	-1.164384946	0.202916625
Med1|19014	-1.238950714	0.156664149	-1.256773042	0.098334876
Rqcd1|58184	-1.239004677	0.098543128	-1.157618704	0.187282299
Pcdhgc3|93706	-1.239087672	0.364708619	1.358855336	0.302417671
Mfsd6|98682	-1.239253323	0.294639124	-1.614673615	0.133414628
Pla2g6|53357	-1.239256701	0.166368234	-1.236373763	0.116365355
Habp2|226243	-1.239285621	0.031915124	-1.337005947	0.005509696
Zdhhc8|27801	-1.239342712	0.008683106	-1.296159347	0.003016463
1810048J11Rik|67708	-1.239382106	0.259477788	-1.548280291	0.107326784
Neu1|18010	-1.239417683	0.001843632	-1.217846954	0.004732295
B4galt7|218271	-1.239424196	0.103206437	-1.192234995	0.057559599
Epn1|13854	-1.239425861	0.129920352	-1.239425861	0.129920352
Ell|13716	-1.239484416	0.122362202	-1.093567211	0.261711455

Parp2|11546	-1.239515238	0.206637844	-1.239515238	0.206637844
Pdlim7|67399	-1.239588499	0.188006664	-1.507828956	0.037540369
Gpr114|382045	-1.239685012	0.36444659	1.42769032	0.258414798
Zc3h18|76014	-1.239717644	0.080622276	-1.150333903	0.162870489
Siglece|83382	-1.239756248	0.278084789	-1.598881667	0.022553885
Cpne2|234577	-1.23989071	0.14452816	-1.461282178	0.022131317
Bckdha|12039	-1.239940543	0.045531566	-1.378793958	0.003925296
Plbd1|66857	-1.240048261	0.170314106	-1.240048261	0.170314106
Bgn|12111	-1.240138899	0.080476101	-1.37090349	0.020110368
Sh2b3|16923	-1.240192501	0.126337933	-1.103843047	0.264406083
Hn1|15374	-1.240250937	0.182577005	-1.303699446	0.145764654
Camk2n1|66259	-1.240265409	0.093829621	-1.407230517	0.01281191
Rab10|19325	-1.240463904	0.142548599	-1.378946201	0.06746274
Ttc9c|70387	-1.240483076	0.186764048	-1.008389141	0.473975532
Tbc1d1|57915	-1.240569579	0.263091328	-1.082869596	0.4128573
Parp12|243771	-1.240620805	0.122343537	-1.015867535	0.451429573
Cpeb1|12877	-1.240768618	0.403386724	2.034446561	0.230763487
Dap|223453	-1.240862029	0.003277154	-1.204539214	0.007552201
Pde4dip|83679	-1.240982636	0.140372604	1.018886282	0.426702093
Zfp946|74149	-1.241048826	0.21756221	-1.347353928	0.142801557
Scaf1|233208	-1.241107888	0.002226979	-1.241107888	0.002226979
Acsl5|433256	-1.241178252	0.018078661	-1.262046085	0.002077577
BC064078|408064	-1.241206809	0.406475112	-2.8	0.173296754
Mgl2|216864	-1.241206809	0.406475112	-2.8	0.173296754
Gbp5|229898	-1.241206809	0.406475112	-2.8	0.173296754
Trmt5|76357	-1.241229117	0.286884774	-1.241229117	0.286884774
Pds5a|71521	-1.241268968	0.24171185	-1.245222018	0.188974949
Ccdc109a|215999	-1.241329047	0.337875743	-2.197578242	0.058438409
Prelid1|66494	-1.241338161	0.023343452	-1.271789296	0.001598047
Aplf|72103	-1.241518746	0.322241537	-1.484426795	0.175661402
Ppard|19015	-1.241530785	0.025632366	-1.162150337	0.022526175
Cfdp1|23837	-1.241541183	0.068428248	-1.241541183	0.068428248
C1qtnf2|69183	-1.241552896	0.345498322	-1.964746675	0.131301799
Nrap|18175	-1.241808287	0.309413638	-1.464879894	0.130455137
Rasa1|218397	-1.241875682	0.182961421	-1.078014981	0.35062697
Ccdc90a|76137	-1.241941835	0.118947547	-1.030129468	0.414884445
Map3k14|53859	-1.242276011	0.259064567	-1.053008681	0.439904938
Pnrc2|52830	-1.242325789	0.174135942	-1.242325789	0.174135942
Nck2|17974	-1.24246534	0.253067037	-1.023544422	0.469997565
Ccnh|66671	-1.242499732	0.177431461	-1.46069046	0.055379822
4933411K20Rik|66756	-1.242660707	0.204689872	1.104621467	0.253362382
Ang|11727	-1.242676978	0.127485526	-1.511881811	0.007111402
Atp5s|68055	-1.242827942	0.264248438	-1.363190802	0.187424873
Hmbs|15288	-1.242829687	0.065017226	-1.242829687	0.065017226
Tctn2|67978	-1.242879437	0.285668435	-1.242879437	0.285668435
Dnajc4|57431	-1.242917531	0.147299415	-1.242917531	0.147299415
Pcsk6|18553	-1.242945281	0.052889706	-1.242945281	0.052889706
Nup107|103468	-1.242991582	0.289952817	-1.658731751	0.093869103
Mrpl39|27393	-1.242993656	0.017495931	-1.139002641	0.056419554
Psme1|19186	-1.243059532	0.143630858	-1.256328425	0.082621815
Sbk1|104175	-1.243246014	0.147967047	1.029231811	0.398857923
Bhlha15|17341	-1.243381278	0.405985266	-2.76078	0.173296754
Itpk1|217837	-1.243464723	0.007932311	-1.138160253	0.010496245

Hsd17b6|27400	-1.243476099	0.182949427	-1.398770691	0.100006627
Kdm1b|218214	-1.243633999	0.36525879	1.950250532	0.01965004
Armc1|74252	-1.243857576	0.003623733	-1.243857576	0.003623733
Prkab1|19079	-1.243901588	0.00937004	-1.356993649	0.000196571
Inhbc|16325	-1.243913283	0.087368297	-1.505526578	0.001209814
Rexo4|227656	-1.243963381	0.066160276	-1.200998958	0.018517086
Pxn|19303	-1.244005626	0.055589792	-1.186977886	0.108640138
Hibadh|58875	-1.244121367	0.034688598	-1.324283434	0.013056885
Mfap4|76293	-1.244151422	0.283213273	-1.598422312	0.127834244
Cpt2|12896	-1.244167132	0.001107035	-1.222248706	0.002829027
Ptpn13|19249	-1.244190322	0.298757075	-1.244190322	0.298757075
Dmap1|66233	-1.244362238	0.064634558	-1.394952573	0.00580463
Lass2|76893	-1.244370867	0.002407478	-1.235890871	0.000341166
Sall1|58198	-1.244389745	0.203021984	-1.244389745	0.203021984
Lgr5|14160	-1.244418753	0.324754909	-1.445849723	0.204866197
Upb1|103149	-1.24445721	0.002289704	-1.197198763	0.002360796
Zc3h12d|237256	-1.244466828	0.208666159	-1.422294356	0.087174688
Map2k4|26398	-1.244473087	0.213797728	1.064287845	0.38664499
Ppm1l|242083	-1.244571325	0.131824229	-1.08327704	0.280104744
Akr1c12|622402	-1.244763047	0.052967515	-1.35622334	0.012694397
Kctd21|622320	-1.244989539	0.156358131	-1.406373742	0.026149361
Tfip11|54723	-1.245033824	0.021772429	-1.389142608	0.000898288
Zbtb49|75079	-1.245086557	0.222170186	-1.040423252	0.428719804
Rspo3|72780	-1.245116254	0.248792195	1.009498011	0.486109431
Klhl13|67455	-1.245165307	0.333713391	-1.768942156	0.161409843
Zfp944|319615	-1.245358802	0.242314513	-1.857806429	0.008597623
Rsph9|75564	-1.245438434	0.40497268	-2.8202	0.173296754
Zfyve9|230597	-1.245542305	0.191980868	-1.080359456	0.357362028
Cyp2d22|56448	-1.24556855	0.014171407	-1.318866339	0.000589429
Secisbp2|75420	-1.245607425	0.118458328	-1.141503803	0.229083965
Mterfd2|69821	-1.245742056	0.086885836	-1.224181718	0.043689917
Abcb8|74610	-1.245840107	0.021679489	-1.193010005	0.045528861
B230219D22Rik|78521	-1.245996442	0.355533551	1.127888835	0.361440648
Med12|59024	-1.246048321	0.013913302	-1.130713455	0.027270574
Als2|74018	-1.246101106	0.011687106	-1.186616216	0.019884254
Fam78a|241303	-1.246107568	0.232619658	-1.08341517	0.396955508
Stx1b|56216	-1.246217509	0.365272158	-1.713821207	0.204246189
Polg|18975	-1.246381726	0.075881338	-1.246381726	0.075881338
Noc4l|100608	-1.246568042	0.052042877	-1.147250729	0.105289003
Pdlim5|56376	-1.246604288	0.289747109	1.297687839	0.112907731
Cr1l|12946	-1.246789931	0.059958053	-1.144942553	0.118350054
Nr1h3|22259	-1.246888417	0.043732736	-1.154059117	0.081050324
Lrrc1|214345	-1.246936683	0.371683001	-1.246936683	0.371683001
Vegfa|22339	-1.24695973	0.130758842	-1.34080284	0.088151495
Irf5|27056	-1.247069006	0.154243473	1.008915363	0.477747122
Acbd5|74159	-1.247098436	0.265290956	-1.137335329	0.330734538
Nit2|52633	-1.247275903	0.033708794	-1.161955317	0.059824599
Fam177a|73385	-1.247336846	0.193084801	-1.205843392	0.123638566
Pamr1|210622	-1.247476281	0.126640786	-1.089585848	0.267452607
Osbp2|74309	-1.247551784	0.249194645	-1.803898599	0.024727516
Phyhd1|227696	-1.247562924	0.072483355	-1.389273178	0.012993852
Ftl1|14325	-1.247606452	0.161288609	-1.294589329	0.075628353
Inhbe|16326	-1.247610511	0.132127202	-1.247610511	0.132127202

Strbp|20744	-1.247869646	0.150101161	-1.110828672	0.29638285
Gstz1|14874	-1.247975454	0.000194952	-1.274364093	7.19717E-05
Fam193b|212483	-1.248098415	0.061732141	-1.248098415	0.061732141
Ptgr1|67103	-1.248103876	0.282285543	-1.886161381	0.031848755
Src|20779	-1.248411916	0.068085712	-1.072902029	0.247355827
Snrnp200|320632	-1.248474576	0.106427007	-1.114770234	0.223153538
Hps5|246694	-1.248480568	0.197742429	-1.248480568	0.197742429
2310068J16Rik|70281	-1.248537997	0.401212986	-1.555558606	0.363361927
Fos|14281	-1.248598274	0.257965717	-1.494316702	0.011059258
Cat|12359	-1.24865314	0.04173515	-1.343110253	0.013065862
Lpp|210126	-1.248688023	0.074125388	-1.114417927	0.126289531
Pdcd7|50996	-1.248740274	0.221039419	-1.572040376	0.055768317
Rad52|19365	-1.248843867	0.220571916	-1.629354597	0.028336023
2410002F23Rik|668661	-1.248868289	0.184815486	-1.299254857	0.116135395
Aldh4a1|212647	-1.248907741	0.028404951	-1.314785971	0.014476682
Hsd11b1|15483	-1.249039151	0.064159713	-1.134217839	0.1207841
Tmem106a|217203	-1.249051177	0.101318001	-1.402033469	0.027149779
Pgrmc1|53328	-1.249062915	0.102751445	-1.226317028	0.083349859
Wdr11|207425	-1.249067727	0.116330304	-1.127032999	0.227909529
Slc38a10|72055	-1.249142602	0.043962862	-1.296792559	0.035664782
Sar1b|66397	-1.249296098	0.0104231	-1.213517893	0.006077793
AW112010|107350	-1.249356958	0.075945741	-1.318429811	0.039253339
Mrpl46|67308	-1.249540937	0.164881263	-1.205463604	0.222173017
Fibin|67606	-1.249563636	0.414087512	-5.7452	0.078237796
B4galt1|14595	-1.249670744	0.029878294	-1.116044868	0.107132898
Dnm3|103967	-1.249786634	0.335524091	-1.126641657	0.415701522
Baz2b|407823	-1.249813616	0.305868175	-1.309789728	0.262637496
Cpsf1|94230	-1.249841954	0.018805496	-1.206151324	0.002763202
Slc11a1|18173	-1.250225862	0.090281829	-1.277634294	0.050099371
Aldh6a1|104776	-1.250270976	0.141731664	-1.155523351	0.238373931
B4galt6|56386	-1.25037815	0.186230034	-1.229521022	0.074698993
Pkd2l2|53871	-1.250547371	0.352541338	-1.250547371	0.352541338
Ppp1r7|66385	-1.250552948	0.049284689	-1.158863928	0.100764664
Tspan17|74257	-1.250658884	0.202239789	-1.083374935	0.37750056
Cxx1a|66158	-1.250706218	0.061120364	-1.231926438	0.046488202
Myl9|98932	-1.250890802	0.366242534	1.1662312	0.348543862
Dcbld1|66686	-1.250904962	0.205105165	-1.460368832	0.097103173
Ifitm2|80876	-1.251093861	0.086311298	-1.21018055	0.06831173
Rock1|19877	-1.251228751	0.201323717	-1.173320276	0.228114243
2310047M10Rik|71923	-1.251269285	0.118062578	-1.120017361	0.235615103
Hrct1|100039781	-1.251271519	0.270803035	-1.251271519	0.270803035
Fam20c|80752	-1.25131196	0.00679253	-1.307573371	0.00079541
Cml1|66116	-1.251356072	0.201274796	-1.798072241	0.000935398
Pomc|18976	-1.251374587	0.344281191	1.1044618	0.4341432
Mlf1ip|71876	-1.251428714	0.393034783	1	#DIV/0!
Dnajc2|22791	-1.251481309	0.116614738	-1.098150245	0.248744866
Hist1h4a|326619	-1.251498342	0.394960838	-1.0353	0.173296754
Hist1h4k|319160	-1.251498342	0.394960838	-1.0353	0.173296754
Cacybp|12301	-1.251535712	0.06668855	-1.354401248	0.01038859
Mup6|620807	-1.251591997	0.370335334	-2.327468391	0.146043794
Mr1|15064	-1.251592495	0.300097469	-1.559744779	0.179748894
Ccnd1|12443	-1.251644248	0.174943386	-1.088639536	0.347621107
Snx14|244962	-1.251747399	0.217852901	-1.051803686	0.423817373

2810405K02Rik|66469	-1.251815386	0.202261213	-1.251815386	0.202261213
Pon3|269823	-1.251831101	0.071999679	-1.139934902	0.147309001
Gapdh|14433	-1.251942371	0.091501984	-1.315087282	0.071868139
Solh|50817	-1.252140583	0.137670849	-1.252140583	0.137670849
Zfp58|238693	-1.252193875	0.270391818	-1.252193875	0.270391818
A230050P20Rik|319278	-1.252227967	0.046726321	-1.45069473	0.000982268
Ptk2|14083	-1.25228452	0.090919884	-1.036796277	0.363959445
Nckap5|210356	-1.252303122	0.368399993	1.369889374	0.321727643
Stx7|53331	-1.252339346	0.218657872	-1.042167474	0.436100817
Vcpip1|70675	-1.252349782	0.250025798	1.061222874	0.39168966
Tnfrsf11b|18383	-1.252379513	0.393522657	-5.179251995	0.051053893
AI848100|226551	-1.252571003	0.270593227	-1.953493867	0.005793574
Tmem129|68366	-1.25281057	0.074360521	-1.283644504	0.018969584
Cops4|26891	-1.252961359	0.053460693	-1.391680372	0.006169262
Gm6251|621697	-1.25305474	0.141489289	-1.095609735	0.298667599
Lgals9|16859	-1.253121062	0.021641168	-1.316839124	0.010725574
Snx9|66616	-1.253178942	0.141679766	-1.107476272	0.27859283
Tiam1|21844	-1.253203981	0.328294879	-1.584610843	0.217334142
Usp22|216825	-1.253220663	0.192406706	-1.637367034	0.017283115
Tra2b|20462	-1.253251612	0.088101469	-1.263802738	0.029956829
Atxn7|246103	-1.253298904	0.23847025	1.03608996	0.434515756
Gabbr1|54393	-1.253386945	0.2316725	-1.253386945	0.2316725
Lphn1|330814	-1.253601675	0.096164071	-1.41789817	0.020124166
Tmem134|66990	-1.253617046	0.011072195	-1.335262781	0.001545242
Mfsd7a|243197	-1.25364117	0.143594826	-1.25364117	0.143594826
Prkrir|72981	-1.253664382	0.009620073	-1.210092474	0.020701908
S100a8|20201	-1.253797875	0.401267692	1.255130842	0.411829119
Acsm3|20216	-1.253853108	0.137692984	-1.139135828	0.2483706
Hoxb5|15413	-1.253949938	0.279822397	-1.253949938	0.279822397
Ift172|67661	-1.254047091	0.06075068	-1.254047091	0.06075068
Fuca1|71665	-1.254063036	0.049698037	-1.254063036	0.049698037
Zfx|22764	-1.254173617	0.194836723	-1.385858863	0.058770378
Zfp790|233056	-1.254185489	0.249114682	-1.254185489	0.249114682
Dpys|64705	-1.254232165	0.002663032	-1.220176656	0.006389456
Stx8|55943	-1.254307151	0.19219353	-1.465498246	0.083364124
Idh3g|15929	-1.254354663	0.004725741	-1.191438128	0.006644886
Pcyox1|66881	-1.254421978	0.07939839	-1.324478572	0.05666905
Zbed3|72114	-1.254549677	0.060120791	-1.148135245	0.122582558
Hmha1|70719	-1.254562255	0.052078634	-1.1654319	0.105431836
Ankrd16|320816	-1.254579933	0.160618549	-1.254579933	0.160618549
Gsta3|14859	-1.254820689	0.062354538	-1.434925479	0.004129247
Mtor|56717	-1.254899113	0.061220241	-1.254899113	0.061220241
Slc25a32|69906	-1.254938257	0.315817189	1.094973071	0.421742541
Ralgapb|228850	-1.254984627	0.158413923	-1.342435416	0.041503798
Slc37a3|72144	-1.255018834	0.026712689	-1.255018834	0.026712689
Mylk|107589	-1.255109459	0.127014611	-1.141942857	0.197340284
Ric8b|237422	-1.255133531	0.068672427	-1.310914948	0.044727563
Mapk1ip1|69546	-1.25514679	0.087600607	-1.065612085	0.310940147
Cdr2l|237988	-1.255306591	0.108497193	-1.155448506	0.200636173
Dhx32|101437	-1.255359243	0.064613692	-1.255359243	0.064613692
Abcc9|20928	-1.255517947	0.201378238	1.059361794	0.391811258
Entpd6|12497	-1.255542433	0.135837093	-1.081623339	0.297744075
Dna2|327762	-1.255753253	0.093107929	-1.263853122	0.070373493

Stab1|192187	-1.255804213	0.006429307	-1.271847221	0.000682181
Bmp8b|12164	-1.255828959	0.401589961	-2.8202	0.173296754
Vcl|22330	-1.256035918	0.208184691	-1.168944813	0.271045544
Apob|238055	-1.256086277	0.015836083	-1.33867608	0.000353899
2610005L07Rik|381598	-1.256235151	0.203158201	-1.468249931	0.096772936
Hadh|15107	-1.256326416	0.007455118	-1.256326416	0.007455118
Serpina3n|20716	-1.256328979	0.07918091	-1.371621323	0.011301036
Rab1b|76308	-1.256365512	0.017810604	-1.256365512	0.017810604
Sfswap|231769	-1.25637092	0.017055395	-1.408357549	0.000272458
1810043G02Rik|67884	-1.256403413	0.070942733	-1.338019817	0.027359399
Fgd5|232237	-1.256442127	0.170314029	-1.182677387	0.19839728
Gbe1|74185	-1.256459913	0.081882428	-1.181918724	0.051613396
Rab43|69834	-1.256523402	0.035020682	-1.105982844	0.128688268
Fancc|14088	-1.256615525	0.149653125	-1.137760314	0.27634445
Cmbl|69574	-1.256716323	0.001632867	-1.217820449	0.001308136
Cgn|70737	-1.256743904	0.10350568	-1.366475435	0.055494212
Epb4.1l2|13822	-1.256942535	0.214581896	1.106066857	0.278368805
Got2|14719	-1.257058221	0.001985889	-1.257058221	0.001985889
4930404N11Rik|432479	-1.257123332	0.302790538	-1.95955702	0.059229023
Ctdp1|67655	-1.257261704	0.056075354	-1.239731756	0.020586939
Rgs3|50780	-1.257296376	0.101886556	-1.135483821	0.209646708
Tln2|70549	-1.25732012	0.159888089	-1.25732012	0.159888089
Acbd4|67131	-1.257363563	0.092014879	-1.257363563	0.092014879
Alkbh3|69113	-1.257533808	0.070981658	-1.378679226	0.023311065
Ccdc132|73288	-1.257560679	0.19707389	-1.257560679	0.19707389
Uroc1|243537	-1.257843129	0.068395203	-1.438856144	0.003127522
Fam123a|72125	-1.25787034	0.335112066	1.077347807	0.448394543
Apom|55938	-1.257888616	0.189469601	-1.275335668	0.132798585
2410002O22Rik|66975	-1.257909387	0.061561087	-1.379828451	0.016836749
Slc47a1|67473	-1.258348091	0.112110028	-1.119754262	0.234611548
Zbtb9|474156	-1.258604389	0.073367877	-1.373046578	0.009393522
3110040N11Rik|67290	-1.258687559	0.155966169	-1.096608797	0.320318248
Nr2c1|22025	-1.258688418	0.069492608	-1.258688418	0.069492608
Gls|14660	-1.258731185	0.154438888	-1.258731185	0.154438888
Mrps11|67994	-1.258814934	0.145358958	-1.014984811	0.463663154
Uggt2|66435	-1.258829595	0.336638718	-1.459026591	0.237981721
Lims2|225341	-1.25884687	0.054616698	-1.33244538	0.033539919
Zfp187|432731	-1.258896158	0.166657592	-1.258896158	0.166657592
Parp10|671535	-1.258939927	0.025408521	-1.364239825	0.003508805
Rassf2|215653	-1.25902942	0.204771438	-1.776450628	0.006040696
Gpatch8|237943	-1.25903031	0.199057989	-1.048977576	0.41682773
Gas8|104346	-1.259084141	0.038708375	-1.090776568	0.115453251
Bcorl1|320376	-1.2591007	0.177513022	-1.101101699	0.256921602
Zbtb8os|67106	-1.259119431	0.161546766	1.047376123	0.347311155
Pcbd2|72562	-1.259241431	0.01631412	-1.135624221	0.054393601
Tmem85|68032	-1.259452914	0.0467557	-1.208990103	0.087307358
Mgrn1|17237	-1.259639036	0.063480683	-1.331736673	0.029080344
Pdzrn3|55983	-1.259673786	0.164000374	-1.35649129	0.033116462
Ndst1|15531	-1.259709725	0.308872894	1.172380143	0.333020814
Zfp410|52708	-1.259794984	0.208510216	-1.590530546	0.04679554
Cry2|12953	-1.259815738	0.030733001	-1.180608274	0.059115827
Aes|14797	-1.25990568	0.204361308	-1.282908442	0.121918766
Fli1|14247	-1.260174524	0.235302242	-1.053251937	0.422384832

Wdr75|73674	-1.26019271	0.144447866	-1.101991184	0.288850309
Tsen15|66637	-1.260315253	0.205920418	-1.112299416	0.355450575
Tmem205|235043	-1.260351608	0.166870526	-1.232261829	0.144310576
D8Ertd82e|244418	-1.260579602	0.197419625	-1.466308125	0.096191656
Stk3|56274	-1.260673144	0.273975489	-1.260673144	0.273975489
Sfxn2|94279	-1.260747627	0.01453225	-1.118415302	0.013250086
Fhad1|329977	-1.260773907	0.194541599	-1.260773907	0.194541599
Sowahb|78088	-1.260905231	0.22557763	-1.260905231	0.22557763
Syt11|229521	-1.261119164	0.160301151	-1.087808137	0.321889754
Spsb2|14794	-1.261183386	0.07156581	-1.19249269	0.047045234
Mgp|17313	-1.261242829	0.336413522	-1.63487368	0.222811858
Cbll1|104836	-1.26131186	0.130369359	-1.25689236	0.090981337
Tigd5|105734	-1.261330687	0.187988638	-1.245587903	0.156379961
Acvr2b|11481	-1.26141913	0.181236183	-1.274752096	0.098404299
Tmem186|66690	-1.26146894	0.118100662	-1.26146894	0.118100662
Zfp128|243833	-1.26173228	0.337328191	1.639240756	0.052190564
Mtss1|211401	-1.261851269	0.202312096	-1.516998408	0.076732253
Lad1|16763	-1.261870987	0.150956095	-1.261870987	0.150956095
Man2c1|73744	-1.261933294	0.012383233	-1.235981993	0.007148972
Mettl22|239706	-1.262175383	0.192316518	-1.440770849	0.056037316
Pcca|110821	-1.26219614	0.106358417	-1.380866076	0.054679489
Aak1|269774	-1.262317885	0.143070273	-1.101486495	0.299365061
Stk11|20869	-1.262588222	0.005715	-1.260175061	0.010050031
Rcbtb2|105670	-1.262609525	0.065097511	-1.432306019	0.005091474
Tspyl1|22110	-1.26263361	0.191119715	-1.190846561	0.228869034
Mon1b|270096	-1.262724137	0.108722362	-1.123338063	0.227445946
5430416N02Rik|100041797	-1.262841373	0.196306559	-1.057457425	0.405319708
Zmym6|100177	-1.262888146	0.337803226	-1.262888146	0.337803226
Nub1|53312	-1.262962789	0.000511103	-1.255620164	0.000307594
Dnmt1|13433	-1.263077806	0.271917389	-1.00738949	0.487941222
Cul7|66515	-1.263108189	0.106517446	-1.601084085	0.000563997
Eid2|386655	-1.263167257	0.370650152	-2.057583119	0.184774269
Trf|22041	-1.263193153	0.080559199	-1.246135635	0.046843821
Pcyt1a|13026	-1.263197654	0.204380426	1.082875248	0.335309074
A630007B06Rik|213993	-1.263345047	0.382407463	1.397748466	0.352468975
Zc3h14|75553	-1.263362344	0.12442	-1.359947108	0.085229864
Zfp119b|240120	-1.263416139	0.324820318	-2.761147608	0.004142902
Tufm|233870	-1.263470422	0.017535017	-1.206512329	0.037631102
Csf3r|12986	-1.263500159	0.254828044	-1.687196477	0.072869477
Ddx58|230073	-1.263514558	0.129163251	-1.540238524	0.005743622
Itga4|16401	-1.263543392	0.111035476	-1.098737512	0.233299561
Adamts4|240913	-1.26356978	0.406838122	-7.725	0.072984714
Slc25a17|20524	-1.263625768	0.055712999	-1.147129635	0.111022612
2810453I06Rik|67238	-1.263727985	0.079626278	-1.372036376	0.017350359
Prdm15|114604	-1.263917862	0.347457245	-1.034412002	0.470320406
Adar|56417	-1.263945465	0.017017165	-1.12594805	0.028943096
5730409E04Rik|230757	-1.264059633	0.131975075	-1.264059633	0.131975075
Capns1|12336	-1.264092826	0.123487599	-1.253770632	0.072346203
Hsd3b7|101502	-1.264390058	0.012819204	-1.260256591	0.000470792
BC049762|193286	-1.264547479	0.321753856	-1.264547479	0.321753856
Crtam|54698	-1.264669856	0.402322572	-6.194816667	0.093788939
Fjx1|14221	-1.264669856	0.402322572	-6.194816667	0.093788939
Zfp574|232976	-1.264860325	0.089766837	-1.434094415	0.017957084

Ero1l|50527	-1.264882418	0.125144385	-1.12306123	0.257549797
Ssfa2|70599	-1.264888337	0.280353874	-1.193990527	0.298159671
Agpat1|55979	-1.265029344	0.044749698	-1.377373894	0.011670501
Hmga1|15361	-1.265347607	0.036365888	-1.162584011	0.055746378
AI467606|101602	-1.265363106	0.365222852	-2.178604522	0.172235104
Slc22a15|242126	-1.265456962	0.060078091	-1.355939252	0.004638087
Heatr7b1|100040766	-1.265561645	0.240978047	-1.511007903	0.059506486
Abcb10|56199	-1.265674486	0.133655594	-1.265674486	0.133655594
Ash1l|192195	-1.265950381	0.23182888	-1.211595158	0.251202491
Tom1l1|71943	-1.265968993	0.172263952	-1.265968993	0.172263952
Nacc1|66830	-1.266017268	0.007168449	-1.266017268	0.007168449
Rps6ka2|20112	-1.266066379	0.064297133	-1.379026003	0.024345651
Ccl9|20308	-1.26607538	0.044750106	-1.268953696	0.026734919
Bmp4|12159	-1.266153874	0.144786643	-1.102271017	0.302464334
Pik3r5|320207	-1.266204441	0.13531734	-1.121310451	0.274683274
Apobec3|80287	-1.266246532	0.189113809	-1.641866645	0.0158237
Lrrc51|69358	-1.266489367	0.224522201	-1.125007683	0.347323496
Zdhhc13|243983	-1.266497454	0.295054002	-1.266497454	0.295054002
Golm1|105348	-1.266508372	0.190560573	-1.266508372	0.190560573
1810024B03Rik|329509	-1.266582675	0.200684109	-1.238644805	0.014842791
Nelf|56876	-1.266841209	0.004299652	-1.20785532	0.004546937
Nav1|215690	-1.266873234	0.101260835	-1.110881185	0.208941878
Cyp3a25|56388	-1.266995919	0.129099714	-1.243076848	0.069697701
Mup21|381531	-1.267044316	0.111063423	-1.267044316	0.111063423
D330012F22Rik|269952	-1.267053093	0.120839339	-1.021934924	0.434970962
Banp|53325	-1.267055984	0.050544918	-1.158068483	0.101748423
Mgat3|17309	-1.26713579	0.396662316	1.282144901	0.403677687
Aars2|224805	-1.26719805	0.055898663	-1.149512745	0.111697311
Ston2|108800	-1.267265786	0.214126915	-1.267265786	0.214126915
Papss1|23971	-1.267276853	0.234899431	-1.267276853	0.234899431
Trfr2|50765	-1.267301903	0.000707998	-1.357899714	5.71681E-06
Selp|20344	-1.267372387	0.298989432	-1.060145974	0.444180875
Plxnb2|140570	-1.267382676	0.000456444	-1.304481823	7.38477E-05
Dhrs1|52585	-1.267419945	0.00670953	-1.200691542	0.006744758
Rdh9|103142	-1.267460498	0.079928752	-1.267460498	0.079928752
Nf2|18016	-1.267464235	0.126931959	-1.110277757	0.268272212
Mamdc2|71738	-1.267727407	0.232076047	-1.43136457	0.120870554
Wdr70|545085	-1.267736858	0.10651962	-1.358423186	0.019911964
Bid|12122	-1.26776951	0.092766011	-1.21784509	0.07189963
Nmnat3|74080	-1.268072875	0.072844277	-1.059206624	0.283759078
Khdrbs1|20218	-1.268107769	0.092167782	-1.371029394	0.025610684
Lyn|17096	-1.268145678	0.079022965	-1.555726589	0.000252829
Mtif3|76366	-1.268228102	0.059735768	-1.268228102	0.059735768
Mtrf1l|108853	-1.268294067	0.140493181	-1.427997309	0.065658104
Fig4|103199	-1.268330838	0.093115238	-1.406335788	0.009635118
Kank1|107351	-1.268394606	0.212842309	-1.056459382	0.419442895
Tubgcp3|259279	-1.268420234	0.166520774	-1.397024454	0.10995791
Shroom1|71774	-1.268467087	0.01095724	-1.213265729	0.023793039
Pcnt|18541	-1.268528281	0.116623426	-1.196254985	0.197696415
Znfx1|98999	-1.26867391	0.077925811	-1.135353562	0.154294628
Btbd9|224671	-1.268882282	0.027820174	-1.449844608	0.000563021
Irf6|54139	-1.269024375	0.085170631	-1.130498148	0.171984335
Hif3a|53417	-1.269071298	0.346091174	1.74399645	0.057734153

Apoa2|11807	-1.269207608	0.190976195	-1.185611607	0.230979505
Spc24|67629	-1.269235153	0.034610507	-1.407738921	0.002244271
Tjap1|74094	-1.269243254	0.060379555	-1.399451268	0.015663031
Batf|53314	-1.269317497	0.306748093	-1.836985521	0.119060806
Lipc|15450	-1.269433574	0.044472218	-1.269433574	0.044472218
Tesk1|21754	-1.269434327	0.017791089	-1.269434327	0.017791089
Bag1|12017	-1.269575516	0.053950256	-1.221478756	0.095844204
Tspan15|70423	-1.269685884	0.192724811	-1.766959714	0.005370535
Tbkbp1|73174	-1.269695146	0.05489682	-1.396811026	0.01357301
Sema4a|20351	-1.269813384	0.000211403	-1.228576818	0.000235273
Slc39a3|106947	-1.269897889	0.013876273	-1.245608671	0.003540098
Zfp260|26466	-1.270126031	0.106707807	-1.144355732	0.21733556
Pcf11|74737	-1.270189729	0.148634083	-1.473730008	0.051269534
Zdhhc9|208884	-1.27026834	0.000625039	-1.245375261	0.001703771
Tgfbrap1|73122	-1.270470932	0.033969355	-1.109114402	0.11860454
Lipg|16891	-1.270682197	0.181598878	-1.080099979	0.3694313
Sf3a2|20222	-1.270736619	0.054866046	-1.163367115	0.107405114
Fcgr3|14131	-1.270768878	0.08483026	-1.398735477	0.011080928
Mtss1l|244654	-1.270966168	0.067491447	-1.270966168	0.067491447
Rnf26|213211	-1.271074237	0.034046041	-1.118042856	0.125650899
Xpa|22590	-1.271150096	0.136135177	-1.271150096	0.136135177
Fech|14151	-1.271386707	0.066180651	-1.271386707	0.066180651
Dcaf15|212123	-1.271604254	0.173327013	-1.431820223	0.057259156
Egln3|112407	-1.271831258	0.1961888	-1.314334407	0.142757405
Slc25a13|50799	-1.271897606	0.082654708	-1.27685545	0.05817163
Pofut1|140484	-1.271948017	0.028600987	-1.216793354	0.012217562
Zfp948|381066	-1.271973423	0.37816165	-3.956991387	0.065139467
Phf7|71838	-1.272034312	0.173006657	-1.435878891	0.098169507
Tyw1|100929	-1.272145292	0.138436683	-1.459278381	0.050965837
Dus2l|66369	-1.27217242	0.18652277	-1.440978052	0.110147176
St7l|229681	-1.272219789	0.189249306	-1.092663995	0.364552186
Dand5|23863	-1.272230453	0.116472456	-1.272230453	0.116472456
Smarca2|67155	-1.27223733	0.126438617	-1.239193742	0.121986949
Igsf21|230868	-1.272277489	0.312964352	-1.081156729	0.431489825
Il17rc|171095	-1.272321997	0.152280856	-1.524413926	0.033230578
Srr|27364	-1.272461958	0.05221149	-1.107316885	0.190395374
2810422J05Rik|75620	-1.272479822	0.009352818	-1.357697414	0.001369676
Ankrd42|73845	-1.27248592	0.395615311	1.11373758	0.461395715
Tnrc6c|217351	-1.272491364	0.076577136	-1.196538715	0.082999412
Srrm2|75956	-1.272523166	0.010692424	-1.226704982	0.023781344
Ciz1|68379	-1.272599892	0.016299728	-1.272599892	0.016299728
1110020G09Rik|68646	-1.272681393	0.151509636	-1.272681393	0.151509636
Bahd1|228536	-1.272756345	0.150532915	-1.451453097	0.067221701
Ppp2r5e|26932	-1.272900356	0.016469574	-1.272900356	0.016469574
Kcnt2|240776	-1.273031136	0.384865273	-3.967316667	0.09324139
Susd3|66329	-1.273031136	0.384865273	-3.967316667	0.09324139
Lyve1|114332	-1.273084483	0.189577952	-1.182979163	0.199842033
Vars2|68915	-1.273183469	0.059280568	-1.148005107	0.106024275
Arhgef12|69632	-1.273339855	0.060537689	-1.225164769	0.05635818
Trim11|94091	-1.273391648	0.142872851	-1.064591818	0.323260962
Fgl1|234199	-1.2734215	0.130604778	-1.27842047	0.090606876
BC005561|100042165	-1.27357244	0.262841598	-1.596188536	0.133527739
Slc39a8|67547	-1.273642165	0.074276886	-1.155593224	0.151673394

Snx19|102607	-1.273735625	0.038342638	-1.202364981	0.079076573
Gtpbp5|52856	-1.273736145	0.033792365	-1.199170543	0.069238815
Nars2|244141	-1.273770745	0.19387608	-1.273770745	0.19387608
Gas6|14456	-1.273907931	0.075877769	-1.571287524	0.000334594
Spag4|245865	-1.273946958	0.293382401	-1.846496639	0.097111319
Alcam|11658	-1.273947392	0.025486947	-1.283763958	0.011943745
Ap1m1|11767	-1.274000164	0.008123961	-1.336998111	0.003246033
Tk1|21877	-1.274123404	0.242692556	-1.708176939	0.061268476
Speg|11790	-1.274202412	0.259097172	1.040628045	0.448194069
Clk4|12750	-1.274248353	0.213530751	-1.06322863	0.413893619
2610507B11Rik|72503	-1.274338384	0.005916726	-1.206352777	0.005249945
Lhx2|16870	-1.274417962	0.206084033	-1.112845041	0.346112796
Stag1|20842	-1.274491736	0.170629249	-1.378277252	0.061917776
Bcl9l|80288	-1.274495096	0.059672854	-1.155743041	0.06435119
Sipa1l1|217692	-1.274507154	0.039567653	-1.195368536	0.081257015
Kif7|16576	-1.274535621	0.240605824	-1.390844258	0.173813491
Agfg2|231801	-1.274840814	0.016725086	-1.221377303	0.036047201
Rgmb|68799	-1.274916091	0.3020346	-1.274916091	0.3020346
Vezf1|22344	-1.275049786	0.218161773	-1.275049786	0.218161773
Nup214|227720	-1.275110809	0.026226735	-1.275110809	0.026226735
Cask|12361	-1.275291912	0.145186832	-1.158820724	0.26260667
Utp6|216987	-1.275299772	0.023035478	-1.205471499	0.048516877
Cwf19l1|72502	-1.275458962	0.055918655	-1.177402947	0.11370702
Zfp746|69228	-1.275565883	0.140305378	-1.346297784	0.064152567
Axl|26362	-1.275585318	0.169775402	-1.275585318	0.169775402
Cic|71722	-1.275589495	0.153996651	-1.379982285	0.113626511
Scly|50880	-1.275715911	0.008378515	-1.32158569	0.006054415
Lrrc24|378937	-1.275742138	0.397652912	-1.305244483	0.378399987
Hsd3b3|15494	-1.27579571	0.053424961	-1.449551685	0.002857332
Cacng7|81904	-1.27588365	0.354772041	-1.27588365	0.354772041
Pmaip1|58801	-1.275899985	0.256772726	-1.512880075	0.097462385
Col15a1|12819	-1.275932048	0.26903713	-1.728853382	0.095728567
Insr|16337	-1.275961965	0.158773686	-1.142559431	0.228640815
Slc7a7|20540	-1.275981712	0.164442671	-1.524408809	0.048174033
Zscan2|22691	-1.275989197	0.206588438	-1.800605173	0.002591507
Rsad1|237926	-1.276047407	0.138837553	-1.287001376	0.047086798
Rfc3|69263	-1.276062612	0.181338712	-1.095050759	0.356136391
Mocs1|56738	-1.27608826	0.051646882	-1.27608826	0.051646882
Exoc6|107371	-1.276144956	0.213027859	-1.035452931	0.446056856
Crtc3|70461	-1.276340413	0.118830869	-1.134022238	0.24416124
Zfp516|329003	-1.27647531	0.149427864	-1.283901804	0.092833023
Gm10768|100038628	-1.276679459	0.017213267	-1.340232203	0.004681094
Rfx7|319758	-1.276681527	0.238199451	-1.276681527	0.238199451
4930524L23Rik|78185	-1.277012407	0.320424379	-1.277012407	0.320424379
Ddx41|72935	-1.277094968	0.118829749	-1.277094968	0.118829749
E130309F12Rik|272031	-1.277325724	0.346405787	-2.22811962	0.130894336
Cml2|93673	-1.277732232	0.004287246	-1.326658128	0.001092808
Tpm3|59069	-1.277743865	0.030578996	-1.204858694	0.062721036
Fbxw7|50754	-1.277779887	0.104196804	-1.028022855	0.411357076
Coro1b|23789	-1.277915876	0.059784601	-1.377950919	0.02916021
Adamtsl4|229595	-1.277985542	0.119037147	-1.133316098	0.245261746
Nsun6|74455	-1.27866606	0.251031952	-1.073190919	0.413701181
Ccdc51|66658	-1.278749546	0.145993902	-1.408678162	0.059281656

Fkrp|243853	-1.278887136	0.114646871	-1.622431703	0.003946985
5730455O13Rik|70567	-1.278909344	0.156428018	-1.278909344	0.156428018
Prkar2a|19087	-1.27922528	0.244139754	1.017529169	0.475870442
Pnkd|56695	-1.279329365	0.004052956	-1.279329365	0.004052956
Wdr76|241627	-1.279388292	0.248053715	-1.202410955	0.287681895
Agtrap|11610	-1.279520775	0.101115432	-1.225112982	0.164662367
Sos1|20662	-1.279715592	0.214916625	-1.253693818	0.186897519
Ppp1r12a|17931	-1.279818502	0.309385239	1.433096181	0.132045177
Cxxc5|67393	-1.280098948	0.001027937	-1.19553247	0.00109971
Helq|191578	-1.280120078	0.240426492	-1.280120078	0.240426492
Celf1|13046	-1.280138745	0.229051329	-1.280138745	0.229051329
Ippk|75678	-1.280141468	0.116891678	-1.609355825	0.006202164
A230072C01Rik|320742	-1.280144167	0.260135228	-1.603890736	0.085475572
Hlf|217082	-1.28028117	0.08635491	-1.139760941	0.176636187
Ube3b|117146	-1.280388498	0.004081003	-1.352424383	0.000708622
Mapt|17762	-1.280474074	0.250115957	-1.178255363	0.249228049
Cdh4|12561	-1.28051818	0.335212368	-1.28051818	0.335212368
Mex3b|108797	-1.280963333	0.386755678	-6.404816667	0.044430303
Bbx|70508	-1.281017004	0.296158387	1.23144556	0.203431354
Flna|192176	-1.281189655	0.206663341	-1.403812535	0.032936759
Rnf44|105239	-1.281259865	0.247293576	-1.195279855	0.295605654
Zfp386|56220	-1.281371137	0.178794588	-1.160518131	0.29891688
Rev3l|19714	-1.281376415	0.230419495	-1.599845373	0.096057342
Cyp20a1|77951	-1.281433236	0.257828253	-1.467944489	0.143295151
Fuca2|66848	-1.281437083	0.218959223	1.082872039	0.372836543
Ndor1|78797	-1.281532627	0.025493324	-1.451810117	0.000364237
Gatc|384281	-1.281551645	0.014058695	-1.375142317	0.002566809
Dyrk3|226419	-1.281735163	0.220921576	-1.129074958	0.237170275
Zfp467|68910	-1.281742981	0.016602642	-1.437238186	0.000828059
Sh2d3c|27387	-1.281763069	0.037954477	-1.382160796	0.002635358
Kcnj10|16513	-1.281979689	0.343708965	-1.410184994	0.208275831
Nisch|64652	-1.282072764	0.009196361	-1.331193008	0.006423891
Col13a1|12817	-1.282122381	0.052974243	-1.206315816	0.106894029
Fam118a|73225	-1.282222278	0.292225796	-1.282222278	0.292225796
Cux2|13048	-1.282309673	0.068694721	-1.106997038	0.224688501
Myo5b|17919	-1.282370464	0.07689518	-1.262610675	0.023420248
Pdf|68023	-1.282514436	0.043409824	-1.161749964	0.081784054
Fam107b|66540	-1.282725487	0.141311552	-1.126636504	0.284679157
Chst7|60322	-1.282742683	0.347515529	-1.282742683	0.347515529
Enpp5|83965	-1.282775524	0.075373776	-1.282775524	0.075373776
Syap1|67043	-1.282984059	0.199166035	-1.609514546	0.055335681
Dcaf7|71833	-1.283069955	0.246395911	1.014174242	0.481448745
Slc6a12|14411	-1.283081653	0.005552656	-1.320236572	0.005003686
Zfp446|269870	-1.283126076	0.17888657	-1.138323722	0.318042065
Irf1|16362	-1.283250687	0.076540392	-1.267104028	0.034463641
H2-DMa|14998	-1.283268066	0.216732566	-1.588497315	0.025400018
Slc12a5|57138	-1.283272763	0.124209846	-1.009255364	0.464224008
Parp1|11545	-1.28332907	0.068746412	-1.28332907	0.068746412
Vsig4|278180	-1.283392935	0.148425948	-1.283392935	0.148425948
Rsph4a|212892	-1.283396357	0.268804416	1.061367014	0.429000526
Ces2e|234673	-1.283543666	0.009116732	-1.341482128	0.002262869
Ppp1r12b|329251	-1.283606591	0.390191718	1.170129778	0.437059136
Nr4a1|15370	-1.283617073	0.30558231	-1.134913908	0.377417309

D730040F13Rik|242474	-1.28362798	0.20349999	-1.107330194	0.366651204
Col11a2|12815	-1.28388744	0.359034401	1.239630944	0.388132903
Ifi44|99899	-1.283904082	0.111584321	-1.283904082	0.111584321
Unc13d|70450	-1.284033779	0.273213046	-1.309086592	0.21920762
Jmjd8|72106	-1.284040832	0.055074582	-1.305941625	0.004514336
Pars2|230577	-1.28408739	0.221983631	-1.28408739	0.221983631
40974|223455	-1.284126316	0.172223917	1.030494105	0.43610832
Fam65c|69553	-1.28421403	0.233827996	-1.065504205	0.425475803
Ntan1|18203	-1.284278771	0.044348726	-1.257953341	0.035914771
Necap2|66147	-1.284479161	0.069332926	-1.548564196	0.001051574
Fbxw5|30839	-1.284729112	0.041490134	-1.232416689	0.078063233
Iqsec2|245666	-1.28475209	0.024723176	-1.200104401	0.050573954
Thoc7|66231	-1.284939001	0.113548466	-1.532661752	0.01282073
Bcap29|12033	-1.284965763	0.273291527	1.001790113	0.498235268
Slc39a4|72027	-1.28496956	0.010377196	-1.314224528	0.001290018
Pard3b|72823	-1.285207962	0.307483473	-1.663529187	0.189019128
Bcor|71458	-1.285307278	0.063839792	-1.274658006	0.015978646
Hectd3|76608	-1.285397555	0.0031522	-1.34379422	0.000683071
Rab14|68365	-1.285415604	0.11954773	-1.121089419	0.252874512
Rrbp1|81910	-1.285536434	0.105402741	-1.432615258	0.046755671
H2afy|26914	-1.285537	0.013288875	-1.285537	0.013288875
Fkbp14|231997	-1.285598574	0.185922295	-1.545313858	0.071069139
Slc39a6|106957	-1.285622667	0.31243265	-1.951485754	0.120564913
Rnf10|50849	-1.285667525	0.022356247	-1.285667525	0.022356247
Shisa2|219134	-1.285679259	0.36403843	-3.06356435	0.047515593
2810008D09Rik|76972	-1.285707657	0.196902346	-1.073507093	0.379802768
Rreb1|68750	-1.285711302	0.206670372	-1.285711302	0.206670372
Ugt1a10|394430	-1.285862891	0.388691436	-7.669380845	0.043082451
Hmgn5|50887	-1.286003492	0.035928837	-1.195025289	0.070274562
Ggcx|56316	-1.2860236	0.03813641	-1.194701268	0.075514718
Acyp1|66204	-1.286396068	0.161112235	-1.546796782	0.045563462
Gpam|14732	-1.286474592	0.302734788	1.158996727	0.357902916
Otub2|68149	-1.286500887	0.232833261	-1.607877572	0.101552798
Sgpl1|20397	-1.286542736	0.159089512	-1.286542736	0.159089512
Irgm1|15944	-1.286712022	0.070971532	-1.286712022	0.070971532
Nfs1|18041	-1.286763413	0.068233746	-1.286763413	0.068233746
Notch2|18129	-1.28683722	0.20246921	-1.020091283	0.460064144
Stk19|54402	-1.286851876	0.124636568	-1.286851876	0.124636568
Btnl9|237754	-1.286872687	0.30939876	-1.011219449	0.490588665
Mapk8ip3|30957	-1.286922646	0.064522551	-1.286922646	0.064522551
Idh3b|170718	-1.286933598	0.000532352	-1.286933598	0.000532352
5730437N04Rik|70544	-1.286972686	0.021179212	-1.194151508	0.029528081
Kdm6a|22289	-1.287051612	0.314889315	-1.246197282	0.349321169
Dcaf11|28199	-1.287081336	0.000153261	-1.32157623	2.64455E-05
Son|20658	-1.287101817	0.143398851	-1.159493813	0.209863012
Lrrc29|234684	-1.287105469	0.102933537	-1.162413256	0.207216276
Nudt14|66174	-1.287143919	0.103248445	-1.506001351	0.016334326
Vti1a|53611	-1.287188028	0.014002392	-1.379431546	0.00300289
Wipf3|330319	-1.287198392	0.044916803	-1.198992268	0.091769543
Dnajc5|13002	-1.287245936	0.19782931	-1.065243305	0.403886945
Gpsm3|106512	-1.287273293	0.164179671	-1.684386089	0.005878046
Itgb7|16421	-1.28731284	0.216213461	-1.526164123	0.116839611
Ankrd17|81702	-1.287384487	0.234741053	1.020520888	0.468955449

Sardh|192166	-1.287743881	0.005691633	-1.427972068	5.61912E-05
Nrn1|68404	-1.28776054	0.041539801	-1.358251511	0.028035751
Adam10|11487	-1.287941131	0.116664944	-1.152636708	0.234036674
Slc16a3|80879	-1.287969754	0.352269115	-1.810348122	0.188260339
Pctp|18559	-1.288042041	0.057960005	-1.474353749	0.003830142
Cd55|13136	-1.288061321	0.166237702	-1.047277837	0.389877147
Smtn|29856	-1.288073691	0.060232527	-1.158518176	0.111650708
Cetn3|12626	-1.288213854	0.023895888	-1.288213854	0.023895888
Aurkb|20877	-1.288222857	0.403696353	1	#DIV/0!
Tnfrsf25|85030	-1.288222857	0.403696353	1	#DIV/0!
Chd5|269610	-1.288222857	0.403696353	1	#DIV/0!
Cap2|67252	-1.288222857	0.403696353	1	#DIV/0!
Cdh26|381409	-1.288222857	0.403696353	1	#DIV/0!
Reln|19699	-1.288228466	0.141751522	-1.013995849	0.469370247
Ube2w|66799	-1.288341363	0.137105271	-1.23709624	0.091098351
Mmaa|109136	-1.288434871	0.039706102	-1.183149736	0.072166016
Gm5523|433273	-1.288511061	0.148617354	-1.73156143	0.002423733
Hmgcs2|15360	-1.288702694	0.028605441	-1.288702694	0.028605441
Thyn1|77862	-1.288751951	0.13307746	-1.288751951	0.13307746
Npr3|18162	-1.288777957	0.277824604	-1.041793967	0.463760206
2010011I20Rik|67017	-1.288914526	0.005841035	-1.176521365	0.016338477
Apitd1|69928	-1.288920026	0.379257424	-4.016833333	0.093227909
Gm711|279029	-1.288920026	0.379257424	-4.016833333	0.093227909
Slc25a16|73132	-1.288938048	0.306206334	-1.131906807	0.380228195
Cnp|12799	-1.288964164	0.074228899	-1.570708846	0.001406237
Fam47e|384198	-1.289084212	0.192407683	-1.675112871	0.030341063
Vwa1|246228	-1.289127729	0.261547553	-2.057651375	0.017585678
Prdm9|213389	-1.289143113	0.180124791	-1.11378613	0.342331868
BC018507|218333	-1.289227127	0.252880126	-1.007136113	0.483152771
Klhl3|627648	-1.289337918	0.307204467	-2.423001018	0.031124376
Usp18|24110	-1.289433644	0.167124304	-1.436276155	0.070559816
Shmt2|108037	-1.289715868	0.000976927	-1.340364557	6.00929E-05
Cbr4|234309	-1.289748476	0.027968432	-1.289748476	0.027968432
Agl|77559	-1.289771206	0.096414167	-1.400921751	0.057819941
Kank3|80880	-1.289833095	0.099566033	-1.030913514	0.391290756
N4bp2l2|381695	-1.289877868	0.123784622	-1.030918483	0.428170102
Mapkbp1|26390	-1.289899745	0.1790068	-1.448323596	0.114848213
Aldh3a2|11671	-1.290013212	0.101703662	-1.148498772	0.21050933
Mug-ps1|17835	-1.290061819	0.152244448	-1.290061819	0.152244448
Aasdh|231326	-1.29013306	0.171878357	-1.29013306	0.171878357
Slc16a11|216867	-1.290168587	0.056130361	-1.24230949	0.096946554
Suox|211389	-1.290237418	0.017216742	-1.231740341	0.006524208
0610009O20Rik|66839	-1.290292437	0.0053904	-1.353713337	0.000549224
Parp3|235587	-1.290430344	0.065096107	-1.163679848	0.13298124
Magi1|14924	-1.290662628	0.107325223	-1.412801481	0.06245985
Lrrc45|217366	-1.290882084	0.196251845	-1.290882084	0.196251845
Nucb2|53322	-1.290986773	0.234365841	-1.155510718	0.324649379
Rcor3|214742	-1.291174086	0.314664867	1.098435614	0.42957084
Oaf|102644	-1.291331472	0.046972402	-1.405024793	0.004163112
Ranbp3|71810	-1.291331695	0.002762792	-1.407682089	8.88661E-05
Tmem170b|621976	-1.291461638	0.254501954	-1.328361266	0.144238545
St3gal2|20444	-1.291545076	0.16495238	1.020271606	0.458277338
Slc30a5|69048	-1.291569275	0.150536077	-1.505633719	0.058598461

Rufy3|52822	-1.291639035	0.026972811	-1.183244454	0.031350354
Tead3|21678	-1.291694443	0.096251562	-1.333693242	0.029295327
Armcx1|78248	-1.291737736	0.340731572	-1.812670781	0.149215492
Prosapip1|241638	-1.292014251	0.053350593	-1.392328863	0.026658384
Gas2l1|78926	-1.292108382	0.0119477	-1.163856988	0.038442017
Ap4e1|108011	-1.292224937	0.038859758	-1.292224937	0.038859758
H6pd|100198	-1.292236711	0.006716544	-1.343100444	0.002352757
Ercc1|13870	-1.292567718	0.111635514	-1.130658627	0.234990332
Slc16a2|20502	-1.292792702	0.117390453	-1.468438669	0.046312326
Sh3bp2|24055	-1.292919187	0.089285902	-1.129916349	0.184294288
Fads1|76267	-1.293600657	0.029478785	-1.142211276	0.105975515
Narfl|67563	-1.293889331	0.014773344	-1.293889331	0.014773344
Fam114a1|68303	-1.294209876	0.071795963	-1.060205296	0.281459762
Camkk2|207565	-1.294569641	0.104562469	-1.137417279	0.218953116
Iqgap2|544963	-1.294586176	0.126009944	-1.481496151	0.049488316
Cenpv|73139	-1.294890418	0.077579352	-1.294890418	0.077579352
Pnldc1|240023	-1.295039914	0.281292145	1.014081713	0.48730269
Akap7|432442	-1.295056637	0.24562846	-1.429171332	0.173111149
Mast2|17776	-1.295061853	0.011740925	-1.218369975	0.01741078
Spag9|70834	-1.295123107	0.183067842	-1.295123107	0.183067842
2810417H13Rik|68026	-1.295144696	0.371596936	1.525170402	0.307810225
Hgfac|54426	-1.295307235	0.017784585	-1.392572256	0.000446046
Gorasp1|74498	-1.295408352	0.044319335	-1.15812147	0.076277403
Tmem43|74122	-1.295433231	0.062362465	-1.066755292	0.229771882
Zc3h4|330474	-1.295453043	0.197929685	-1.140279174	0.262332334
Tnxb|81877	-1.295543178	0.20738664	-1.060850559	0.417304067
Tmpo|21917	-1.295836984	0.160764412	-1.341957674	0.105211694
Zfp236|329002	-1.2959394	0.167115909	1.034447784	0.42498728
Golgb1|224139	-1.296008369	0.104133769	-1.149266599	0.215776323
Pawr|114774	-1.296042003	0.299546684	1.04739355	0.462720466
Sp6|83395	-1.29609618	0.387020615	-2.8	0.173296754
Fam71f2|245884	-1.29609618	0.387020615	-2.8	0.173296754
Tulp4|68842	-1.296128992	0.013325465	-1.369000425	0.000405313
Tspan18|241556	-1.296344411	0.006500205	-1.376048806	0.001473394
Mtap4|17758	-1.296494147	0.073280561	-1.223607123	0.130525136
Cdc42bpb|217866	-1.296680766	0.024394972	-1.201372638	5.82413E-05
Vezt|215008	-1.296900902	0.101780064	-1.414363037	0.036011726
Pdp2|382051	-1.296961907	0.228640621	-1.562182922	0.125744522
Psd|73728	-1.29704201	0.318205793	-1.202794778	0.362818418
Fgd2|26382	-1.297048146	0.309831181	-2.93946282	0.002632129
Prkx|19108	-1.297294417	0.319195577	-1.810938157	0.179641039
Emr1|13733	-1.297451242	0.200958723	-1.759204033	0.021764279
Ntn3|18209	-1.297679048	0.160913998	-1.297679048	0.160913998
Rin3|217835	-1.297707895	0.014044854	-1.297707895	0.014044854
Tmem42|66079	-1.297771942	0.080165156	-1.175243509	0.164239834
Rgs12|71729	-1.297902841	0.133952726	-1.537397869	0.035508868
Atn1|13498	-1.297986095	0.010984181	-1.28306035	0.003561104
Ces3a|382053	-1.29808818	0.042863836	-1.29808818	0.042863836
Fermt3|108101	-1.298146918	0.089811989	-1.164785034	0.179691919
Slc22a4|30805	-1.29817336	0.203320087	-1.673731248	0.051789717
Amt|434437	-1.298195394	0.141957229	-1.151149805	0.273489459
Fastkd1|320720	-1.298228874	0.141709158	-1.123031915	0.292283559
Wdr37|207615	-1.298304168	0.168258965	-1.109264953	0.336835

Igsf5|72058	-1.298332503	0.043026579	-1.099122329	0.119211836
Ptpn6|15170	-1.298377839	0.012983323	-1.298377839	0.012983323
Gm14378|100044509	-1.298416031	0.095689896	-1.512036985	0.017040247
Fzd5|14367	-1.29843466	0.222476261	-1.070400909	0.417792176
Gas1|14451	-1.298757863	0.163156869	-1.298757863	0.163156869
A4galt|239559	-1.298834574	0.393066013	2.519565727	0.185597322
Hcfc1|15161	-1.298872458	0.171545677	-1.059531942	0.386644388
Leng8|232798	-1.298919942	0.049484918	-1.409214535	0.007032881
Crispld2|78892	-1.298937921	0.333030723	-1.782833453	0.139483127
Slc35a3|229782	-1.298948043	0.256112771	-1.120033773	0.265070731
Khdrbs3|13992	-1.299039874	0.053089126	-1.372455149	0.026662922
Morn4|226123	-1.299159564	0.368681317	1.282318471	0.390467519
Itch|16396	-1.29926892	0.128422314	-1.244549619	0.101902276
Nes|18008	-1.299425099	0.220384009	-1.934088868	0.010762149
Pxk|218699	-1.299427151	0.102278415	-1.284848015	0.03695481
Tmem136|235300	-1.299446989	0.302470924	-1.299446989	0.302470924
Mtcp1|17763	-1.299559803	0.197998638	-1.309978448	0.151545805
Hdhd3|72748	-1.299779204	0.051206493	-1.199774585	0.104138162
Nckipsd|80987	-1.299860538	0.041823039	-1.363703863	0.023124746
Tti1|75425	-1.300062788	0.036838226	-1.234868198	0.013548533
Coq3|230027	-1.300094888	0.078602501	-1.40235975	0.028916163
Tmem86b|68255	-1.300110554	0.004340278	-1.306703142	0.001235523
Pdha1|18597	-1.300342035	0.178613703	-1.181051282	0.277653933
Igsf6|80719	-1.300412527	0.132688391	-1.176540257	0.19500847
Gtf3c5|70239	-1.300449013	0.092392272	-1.44018084	0.016389193
Slc50a1|19729	-1.300534824	0.075569025	-1.418634017	0.017679521
Fam98a|72722	-1.300570923	0.102777434	-1.300570923	0.102777434
Naip6|17952	-1.300704063	0.286436963	-1.300704063	0.286436963
Sox6|20679	-1.300871664	0.233433739	-1.882976437	0.027477692
Tmem214|68796	-1.30094609	0.030177097	-1.30094609	0.030177097
Zyx|22793	-1.301068025	0.011760242	-1.278410065	0.003700632
Malat1|72289	-1.301126835	0.040634472	-1.270490757	0.022786373
Fbxo45|268882	-1.301752955	0.159625125	-1.431950576	0.081214992
Gm12070|654472	-1.30176062	0.052234263	-1.519241561	0.001958216
Pprc1|226169	-1.301840933	0.059921211	-1.301840933	0.059921211
Lpin3|64899	-1.301873424	0.193327782	-1.606133058	0.069932014
Rpusd2|271842	-1.302017322	0.122212234	-1.085879602	0.263378787
Arhgap22|239027	-1.3020638	0.305380249	-1.3020638	0.305380249
Glb1|12091	-1.302133132	0.037285992	-1.506873775	0.001330264
Trappc9|76510	-1.302157704	0.047648806	-1.302157704	0.047648806
St6galnac4|20448	-1.302218235	0.253563206	-1.899568551	0.051177499
Impad1|242291	-1.302244708	0.272316043	1.015622143	0.485109989
2410004B18Rik|66421	-1.30233908	0.203626031	-1.574280954	0.095435235
Ep300|328572	-1.302399299	0.260546239	-1.090894033	0.405006561
Golga4|54214	-1.302416244	0.050578228	-1.116173694	0.144363277
Asb13|142688	-1.30254436	0.081396321	-1.492726348	0.016766913
Abcc5|27416	-1.302578927	0.208502691	-1.644310281	0.073390816
Zfp407|240476	-1.302683215	0.162581105	-1.302683215	0.162581105
Usp54|78787	-1.302754174	0.273736059	-2.113363075	0.033792786
Cgnl1|68178	-1.302801285	0.184844262	1.003120723	0.489257758
Ubtd1|226122	-1.302845623	0.103672966	-1.64967142	0.003372997
Copg2|54160	-1.302861356	0.114867594	-1.536178691	0.024902414
Mapkap1|227743	-1.303081385	0.015860723	-1.410506579	0.00276574

Rara|19401	-1.303300915	0.155709118	-1.279850464	0.132731808
Tsc2|22084	-1.303307636	0.021826227	-1.303307636	0.021826227
Hipk3|15259	-1.30334123	0.195427099	-1.115637093	0.300701507
Sh2b2|23921	-1.30336992	0.226597998	-1.30336992	0.226597998
Igfbp5|16011	-1.303487002	0.201930174	-1.140039419	0.344985192
1600014C10Rik|72244	-1.303530558	0.000322513	-1.225063919	0.000455732
BC021891|234878	-1.303844422	0.115713358	-1.505200267	0.037961535
1810037I17Rik|67704	-1.303991091	0.035471718	-1.454902671	0.004041716
Slc38a7|234595	-1.30415098	0.010964538	-1.30415098	0.010964538
Sdc3|20970	-1.304481965	0.060572367	-1.070427949	0.218498375
Uck1|22245	-1.304582028	0.033244918	-1.188938179	0.063366944
Acad12|338350	-1.304664451	0.169775052	-1.402917865	0.118327852
1810012P15Rik|66274	-1.304681948	0.177311834	-1.292196353	0.126559069
Usp8|84092	-1.30484534	0.231132852	-1.383893861	0.100217448
Fndc3a|319448	-1.304964318	0.250853036	-1.19917475	0.305615277
Gimap9|317758	-1.305151633	0.147896925	-1.166744194	0.2722524
Prdx6|11758	-1.305186428	0.000045104	-1.27240578	3.57738E-05
Hn1l|52009	-1.305248322	0.12379952	-1.214310951	0.148458354
Fam175b|109359	-1.305809929	0.082713796	-1.439493316	0.039470254
Exoc7|53413	-1.305916293	0.056649347	-1.093663777	0.18511658
Scx|20289	-1.306007307	0.337265607	-2.697116162	0.079573611
Cntd1|68107	-1.306315061	0.4068475	1	#DIV/0!
Skiv2l2|72198	-1.306397902	0.200436955	-1.100007872	0.377728372
Rfk|54391	-1.306501952	0.088848626	-1.444846858	0.012314149
Zfp62|22720	-1.306529507	0.24629791	-1.306529507	0.24629791
Kctd1|106931	-1.306592672	0.283862383	-1.306592672	0.283862383
Phf20|228829	-1.306687812	0.173019652	-1.306687812	0.173019652
Dennd2a|209773	-1.306783274	0.203549864	-1.196295187	0.30542669
Jakmip1|76071	-1.306857348	0.348445103	-2.334383838	0.154268948
Stambp|70527	-1.306857561	0.094998295	-1.147815588	0.197114879
Aldh3b1|67689	-1.306873054	0.257698173	1.013435437	0.485617696
Heg1|77446	-1.306981061	0.245790433	-1.040551032	0.445263447
Tab1|66513	-1.307066717	0.107768151	-1.187843573	0.208007413
Alg14|66789	-1.307334476	0.081000366	-1.631926658	0.001994607
Raph1|77300	-1.307347294	0.053400728	-1.193675294	0.106973003
BC065397|436230	-1.307395982	0.273399285	-1.546413196	0.14054864
Tubb6|67951	-1.307457031	0.089967359	-1.307457031	0.089967359
Slc9a1|20544	-1.307479567	0.014340637	-1.484833899	6.45233E-05
Gp1ba|14723	-1.307544191	0.212285004	-1.983971066	0.01169902
Sh2d2a|27371	-1.307616752	0.131637474	-1.42938014	0.090574014
2610528E23Rik|66497	-1.307732137	0.132207594	-1.012446008	0.471388975
Mup7|100041658	-1.307750569	0.307535811	-1.969919324	0.13183592
Armc9|78795	-1.307845463	0.096848692	-1.243978493	0.161982127
Rassf5|54354	-1.307853421	0.12473306	-1.55962021	0.027808456
1110034A24Rik|109065	-1.307900063	0.282621425	-2.272544879	0.026134045
Slc6a7|240332	-1.308151617	0.363999082	-2.114794391	0.165608225
4632428C04Rik|100043102	-1.308200826	0.299723657	-1.832456006	0.096192173
Onecut2|225631	-1.308353217	0.085832281	-1.224816204	0.018385788
Def6|23853	-1.308528456	0.220348217	-1.154907584	0.327170965
Matr3|17184	-1.308705164	0.10525333	-1.167522203	0.213884313
Gclm|14630	-1.30874859	0.03535939	-1.403244544	0.017419906
Dak|225913	-1.309048759	0.031965008	-1.226460092	0.008272014
Pola2|18969	-1.309049487	0.137438384	-1.309049487	0.137438384

Sars2|71984	-1.309058437	0.100152904	-1.249446306	0.102728317
1110031I02Rik|66179	-1.309183725	0.11657007	-1.446643369	0.068786843
Magohb|66441	-1.309490667	0.387136555	2.800934524	0.110689841
Exoc1|69940	-1.309525612	0.160825914	-1.460592319	0.106172991
Bmp6|12161	-1.309584334	0.254982113	-1.032284995	0.468415712
Optn|71648	-1.309598986	0.056094966	-1.216566858	0.114071597
Pnpla6|50767	-1.309883272	0.012498917	-1.239139744	0.026665569
Rab3il1|74760	-1.309962517	0.129768605	-1.291322424	0.044178861
Lpgat1|226856	-1.310022799	0.021936307	-1.142591751	0.038531801
Pgm2l1|70974	-1.310254315	0.294528519	1.13347832	0.385399825
Lims1|110829	-1.310360088	0.194739622	-1.310360088	0.194739622
Gbp11|634650	-1.31059586	0.290701317	1.369223895	0.169323377
Acad9|229211	-1.310599489	0.057729361	-1.10916905	0.207761005
Hamp2|66438	-1.310671605	0.291165332	-2.915726784	0.000580835
Tcf7|21414	-1.31068716	0.086628336	-1.062574693	0.326074589
Spp1|20750	-1.310784583	0.043366103	-1.108360439	0.11508405
Tsc1|64930	-1.310945117	0.114655311	-1.636544154	0.0028514
G630016D24Rik|619301	-1.310977809	0.375459435	-6.42165	0.04426109
Fbxl5|242960	-1.311005654	0.12453411	-1.511156046	0.048713932
Crim1|50766	-1.311093043	0.162572861	-1.11181505	0.33191547
Ubp1|22221	-1.311112627	0.125374079	-1.246766668	0.121165368
Ptpre|19267	-1.311136112	0.286353663	-1.000852445	0.499310665
Topbp1|235559	-1.31117326	0.144196764	-1.128516511	0.295766973
Fubp1|51886	-1.311192493	0.209207283	-1.311192493	0.209207283
Agk|69923	-1.311261738	0.148596186	-1.659621294	0.017404803
Gpr18|110168	-1.311364959	0.382214599	-2.8202	0.173296754
Taf3|209361	-1.311424961	0.020299432	-1.310140077	0.001307936
Wdr19|213081	-1.311539514	0.317198745	-2.131170548	0.122194279
Rfx2|19725	-1.311576752	0.24933893	-2.088144357	0.015541816
Rap1gap|110351	-1.311629231	0.109586325	-1.321212634	0.082245881
Gna11|14672	-1.311991695	0.00188263	-1.258536915	0.000404442
Slk|20874	-1.312116034	0.100367623	-1.171128804	0.204826545
BC023829|236848	-1.31232758	0.038141792	-1.144131761	0.11777293
Aifm1|26926	-1.312438114	0.013326813	-1.245609411	0.026778961
Trim41|211007	-1.312441973	0.033222859	-1.28715113	0.013777641
Mll5|69188	-1.312477127	0.216818607	-1.185336836	0.273559797
Soat2|223920	-1.312540171	0.040740502	-1.427019763	0.015914306
Lphn2|99633	-1.312555292	0.138507939	1.007686313	0.472012011
Zmiz2|52915	-1.312565508	0.003132474	-1.379055865	0.001074174
Tmem125|230678	-1.312851949	0.035548517	-1.384706745	0.025102144
Nr1h5|381463	-1.313017779	0.20841633	-1.449577544	0.102137561
Vpreb3|22364	-1.31315117	0.372374696	-2.942539542	0.131712047
4933427D14Rik|74477	-1.313421604	0.253950347	-1.313421604	0.253950347
Ppm1f|68606	-1.313503858	0.11424858	-1.037114991	0.412860034
Afp|11576	-1.313595679	0.318844312	1.218606246	0.353137859
Ddx17|67040	-1.313626255	0.127521386	-1.040027867	0.35535759
Tm7sf2|73166	-1.313652051	0.041152186	-1.456329598	0.000241616
Gapvd1|66691	-1.313899132	0.222241235	-1.194831199	0.293610554
Hcn3|15168	-1.313939614	0.135843154	-1.488465624	0.071507855
Ccdc157|216516	-1.314084215	0.058102909	-1.314084215	0.058102909
2900026A02Rik|243219	-1.314175823	0.085173278	-1.150970451	0.171322303
Npr1|18160	-1.314216434	0.082402477	-1.169567714	0.169260151
Nr1h2|22260	-1.314309847	0.023553724	-1.314309847	0.023553724

D730039F16Rik|77996	-1.314375572	0.001578579	-1.375279193	0.000524056
2610015P09Rik|212153	-1.314659423	0.22614742	1.132162252	0.322541384
2810055F11Rik|67217	-1.314817605	0.038680422	-1.13328963	0.134939413
2310042D19Rik|74183	-1.314986529	0.297923016	-1.314986529	0.297923016
Abl1|11350	-1.315116932	0.08867089	-1.231394224	0.161696413
C3|12266	-1.315160498	0.023604426	-1.3767233	0.003562921
Osbpl2|228983	-1.315414297	0.004464033	-1.315414297	0.004464033
Pld4|104759	-1.315455559	0.137928909	-1.48059525	0.038302405
Phkb|102093	-1.3157904	0.151115811	-1.592699615	0.04458858
Fam43a|224093	-1.315891418	0.116931885	-1.538173481	0.035319849
Pldn|18457	-1.315963939	0.140862923	-1.007011774	0.485148919
Ceacam18|72431	-1.315979476	0.373894521	-6.44615	0.04497135
Bdnf|12064	-1.31602366	0.39920484	1	#DIV/0!
Immp2l|93757	-1.316349878	0.087980104	-1.30103008	0.07626039
Cept1|99712	-1.316399589	0.040070915	-1.3096622	0.010671412
Zbed4|223773	-1.316511437	0.345347049	1.284432703	0.311544108
Nek4|23955	-1.316699113	0.269678944	-1.531102273	0.140266256
Rsph3a|66832	-1.316791834	0.133342872	-1.731282252	0.010967572
Gimap4|107526	-1.316982862	0.109850724	-1.210032277	0.202227924
Rab11fip1|75767	-1.317018524	0.014640405	-1.244025695	0.031362082
Pias3|229615	-1.317035489	0.155461902	-1.104118676	0.330049567
2410076I21Rik|73673	-1.317048596	0.294589062	1.022181134	0.483313319
Cstf2t|83410	-1.317097709	0.122853913	-1.020350854	0.449765107
Amot|27494	-1.31714343	0.25623615	-1.732569535	0.120185515
Mup15|100039150	-1.317309738	0.277488925	-1.098173927	0.414607627
Gsta1|14857	-1.317346061	0.35984882	-1.317346061	0.35984882
Gpr146|80290	-1.317382493	0.002994015	-1.202642197	0.003823197
Pbld1|68371	-1.317507199	0.000288409	-1.237218098	0.000251031
Slc25a19|67283	-1.317646886	0.007406115	-1.351328972	0.008158154
Ptger2|19217	-1.317844946	0.280333344	-2.073848937	0.070345111
2210417A02Rik|70138	-1.318091179	0.371956267	-1.117471429	0.447113135
Phf20l1|239510	-1.318187816	0.276540898	1.151945641	0.326913403
Efcab2|68226	-1.318236518	0.297872061	-1.183374847	0.36069788
Arl5b|75869	-1.318451573	0.317671918	1.28704839	0.295280851
Yaf2|67057	-1.318550427	0.098078868	-1.456524047	0.054072487
Timm17b|21855	-1.318558157	0.029726044	-1.318558157	0.029726044
Zcchc14|142682	-1.31868255	0.139533774	-1.097916635	0.307152306
Abcc6|27421	-1.318703296	0.00630505	-1.189291641	0.016701648
Pom121|107939	-1.31891148	0.259396197	-1.211924439	0.309970143
Pdk1|228026	-1.318968334	0.123638541	-1.158940033	0.237814303
Paox|212503	-1.319028824	0.02662753	-1.150644111	0.098576666
Sec22c|215474	-1.319034201	0.010928072	-1.498335914	0.000141799
Ftl2|14337	-1.319079939	0.083136141	-1.47096178	0.035781471
Kif5a|16572	-1.319214725	0.147875255	-1.104063473	0.305835527
Ccdc45|320162	-1.319391354	0.066799329	-1.319391354	0.066799329
E2f7|52679	-1.319415996	0.372645112	-6.462983333	0.044795474
Zbtb5|230119	-1.31984101	0.005138303	-1.385948957	0.002408339
Klhl12|240756	-1.319971935	0.17495225	-1.483048105	0.078250761
Pdap1|231887	-1.320065538	0.015217803	-1.320065538	0.015217803
Zfp429|72807	-1.320213411	0.344840544	-2.344773177	0.157931685
Kifc5b|16580	-1.320245596	0.376538721	-7.662133333	0.044268711
Camk1d|227541	-1.320492905	0.208782007	-1.62264234	0.099218391
Ddx23|74351	-1.320590638	0.015050451	-1.320590638	0.015050451

Apol9b|71898	-1.320682791	0.165770042	-1.594762941	0.002006612
E2f6|50496	-1.320713681	0.040563013	-1.593449504	0.000177822
Gm5918|546143	-1.320841744	0.086650918	-1.43776906	0.030463476
Wiz|22404	-1.32097398	0.010785823	-1.32097398	0.010785823
Tex2|21763	-1.320990791	0.135738534	-1.320990791	0.135738534
Ugt1a1|394436	-1.321044557	0.029042668	-1.194976616	0.050481436
Pot1b|72836	-1.321125352	0.200476114	-1.461760061	0.123907526
Zfp518a|72672	-1.321137394	0.270887033	1.047712117	0.454726263
Zfp623|78834	-1.321319296	0.008811293	-1.381414097	0.005809153
Morn2|378462	-1.321419624	0.189007164	-1.321419624	0.189007164
Nsg2|18197	-1.321603493	0.381581988	-2.8	0.173296754
Prr11|270906	-1.321603493	0.381581988	-2.8	0.173296754
Tmem40|94346	-1.321603493	0.381581988	-2.8	0.173296754
Pcdhb20|93891	-1.321603493	0.381581988	-2.8	0.173296754
Abhd14b|76491	-1.321813726	0.029571257	-1.418414896	0.01405959
Ap1g2|11766	-1.322319395	0.133173145	-1.783222978	0.00398412
Bbs4|102774	-1.322325543	0.240780936	1.12204292	0.352864241
Gnas|14683	-1.32232857	0.001227014	-1.32232857	0.001227014
Parp11|101187	-1.322473311	0.154728108	-1.133235465	0.309690858
Unc13c|208898	-1.322528919	0.170447665	-1.582101652	0.073706634
Plxna4|243743	-1.322588535	0.257754241	-2.415676301	0.005248411
Cacnb3|12297	-1.322730347	0.302755016	1.10859724	0.42157837
Cenpl|70454	-1.322856648	0.267838908	-1.027634775	0.476224776
Fam126a|84652	-1.322907896	0.223365888	-1.536743424	0.078033122
Rhbg|58176	-1.323084727	0.103910142	-1.238440935	0.045805364
Smyd2|226830	-1.323122195	0.03366996	-1.222513603	0.069620902
Crebl2|232430	-1.323166496	0.229240361	1.118332682	0.356090691
Rabep2|70314	-1.323214309	0.09781138	-1.323214309	0.09781138
3110070M22Rik|67304	-1.323289955	0.250507445	-1.652146514	0.100586598
Ubr2|224826	-1.323300448	0.040234575	-1.323300448	0.040234575
6430598A04Rik|243300	-1.323479792	0.218602778	-1.567061796	0.033223292
Pik3r6|104709	-1.323591814	0.330142473	-2.859502781	0.06641704
Lix1l|280411	-1.323868535	0.195053267	-1.352575892	0.164331455
Smc1a|24061	-1.32409219	0.041443012	-1.129607876	0.150220272
BC057079|230393	-1.324127892	0.20173885	-1.324127892	0.20173885
Klhl24|75785	-1.324456749	0.27149353	-1.136840738	0.374982418
Ubl7|69459	-1.32490404	0.016521056	-1.363454735	0.017269752
Coil|12812	-1.32491097	0.261181692	-1.32491097	0.261181692
Degs2|70059	-1.324964935	0.301180797	-1.324964935	0.301180797
Necab1|69352	-1.325035261	0.079565116	-1.178156625	0.163020371
Lgr4|107515	-1.325157638	0.146500017	-1.325157638	0.146500017
Cd200|17470	-1.325219034	0.313124173	-1.325219034	0.313124173
Klk8|259277	-1.325385609	0.340255191	-3.089363256	0.074093315
Nkd1|93960	-1.325659107	0.034418804	-1.549906182	0.002059732
Psma8|73677	-1.325835117	0.38018765	-2.8202	0.173296754
Prkd1|18760	-1.325835117	0.38018765	-2.8202	0.173296754
Spg7|234847	-1.325967001	0.002034781	-1.368720668	0.00173085
Hist1h2bc|68024	-1.325973278	0.02002992	-1.325973278	0.02002992
Map2k5|23938	-1.326178906	0.026731445	-1.468363327	0.004080703
Sema3g|218877	-1.326374118	0.376936656	-9.39515	0.038412663
Dr1|13486	-1.326727538	0.289016874	-1.208033174	0.363297945
Mgll|23945	-1.327017527	0.010567552	-1.244148988	0.021026542
Gnat2|14686	-1.327048274	0.359385975	1.266316107	0.39603646

Eno2|13807	-1.327048274	0.359385975	1.266316107	0.39603646
Ebi3|50498	-1.327071081	0.292408598	-1.459512442	0.2321273
Scfd1|76983	-1.327089617	0.073779465	-1.620341535	0.006357144
Pigr|18703	-1.327200988	0.014712387	-1.52314159	0.000124621
Mocos|68591	-1.327291997	0.046042534	-1.213115658	0.091377618
Nthl1|18207	-1.327363781	0.150008073	-1.795593086	0.009123087
Nlk|18099	-1.327378121	0.244490424	-1.248951504	0.138727518
Lpcat1|210992	-1.327453214	0.375381462	-4.56078	0.070574133
Ank1|11733	-1.327566667	0.383751051	-3.925	0.173296754
Suclg2|20917	-1.327766937	0.010957733	-1.264544706	0.024205395
Ces1g|12623	-1.327770065	0.044754732	-1.118837878	0.148729962
Maob|109731	-1.327976806	0.051421877	-1.377590738	0.049592776
Fam82a2|67809	-1.328239392	0.061914169	-1.097512215	0.237900785
H2-Oa|15001	-1.328587642	0.340680717	-2.287459648	0.164128923
Zdhhc5|228136	-1.32861983	0.046648562	-1.388103075	0.013940786
Crebbp|12914	-1.328691076	0.258666804	-1.204247011	0.29243425
Trak1|67095	-1.328771209	0.062511868	-1.3268566	0.034877855
Eepd1|67484	-1.328821204	0.03313564	-1.361853301	0.009351714
Smarca4|20586	-1.328932059	0.006207172	-1.179734862	0.005961482
Dgke|56077	-1.328940719	0.353955329	1.523109041	0.284366488
Fam76a|230789	-1.32899241	0.064676535	-1.301047472	0.008548424
Ccar1|67500	-1.32907348	0.075286502	-1.388407071	0.02452775
Susd1|634731	-1.329297505	0.169561393	-1.718171172	0.031692902
Pptc7|320717	-1.32936791	0.269892673	1.092435633	0.406071498
Sap30bp|57230	-1.32949714	0.042973549	-1.217883645	0.085049487
Etl4|208618	-1.329506379	0.223345218	1.10875104	0.364932735
Tbc1d14|100855	-1.329511228	0.090197093	-1.187145026	0.184654914
Parp4|328417	-1.329553188	0.130097754	-1.084180985	0.28119422
Gstm1|14862	-1.329719871	0.122414607	-1.372907235	0.03728544
Mark4|232944	-1.33014244	0.099757634	-1.596048201	0.01450168
Il15ra|16169	-1.330154367	0.050775302	-1.438793487	0.012344311
Samd8|67630	-1.330593249	0.126219958	-1.112062004	0.273859473
Frmd4b|232288	-1.330622603	0.121967136	-1.336180288	0.083432379
Bcl3|12051	-1.330696066	0.07633792	-1.168427185	0.150692846
Hdhd2|76987	-1.330744718	0.025533387	-1.409978259	0.005260259
Prkcb|18751	-1.330760793	0.167176812	-1.397267097	0.086576375
Hc|15139	-1.330773514	0.004998491	-1.330773514	0.004998491
Myo1d|338367	-1.330925821	0.010363138	-1.413819907	0.001039782
Ppapdc2|74411	-1.331017873	0.08563455	-1.668998335	0.003916811
Gcgr|14527	-1.331218472	0.001390434	-1.434993047	6.54951E-05
Slc25a30|67554	-1.33133916	0.274402195	1.105780525	0.396674352
Adck2|57869	-1.331345399	0.045244709	-1.331345399	0.045244709
Myst3|244349	-1.331351996	0.208669439	1.026016111	0.445183777
Car2|12349	-1.331571092	0.101081732	-1.166012989	0.203421392
Dnm1l|74006	-1.331717531	0.070491475	-1.084045224	0.250763374
R3hdm2|71750	-1.332010053	0.00850282	-1.418281536	0.002915319
Dennd4b|229541	-1.33215899	0.242446982	-1.528539075	0.138817309
Snx18|170625	-1.332189989	0.063942165	-1.332189989	0.063942165
Ino80c|225280	-1.332376935	0.09986168	-1.161005302	0.208185589
Pgm5|226041	-1.332747121	0.268154581	-1.244866503	0.30241805
Stxbp6|217517	-1.332832099	0.064728896	-1.488270106	0.023633113
Hcls1|15163	-1.333230574	0.090074618	-1.333230574	0.090074618
Mx1|17857	-1.333432453	0.342706022	-3.943418182	0.013288051

Tle3|21887	-1.33347032	0.06353116	-1.07367455	0.237246147
Nbas|71169	-1.333514823	0.035905522	-1.333514823	0.035905522
Tmem220|338369	-1.333645761	0.211194061	-1.333645761	0.211194061
Cd300lg|52685	-1.333896044	0.017807324	-1.377349315	0.01401513
Pxmp2|19301	-1.333947135	0.004635243	-1.407297681	0.000366519
Fbxo21|231670	-1.334224355	0.060323208	-1.334224355	0.060323208
Zfp963|620419	-1.334388852	0.113462561	-1.492052511	0.029704813
8030462N17Rik|212163	-1.334421912	0.29931595	-2.065116859	0.1236989
Cphx|105594	-1.334585635	0.357217834	1.469092247	0.321394913
Vgll4|232334	-1.334957486	0.284696954	-1.522916469	0.157754153
Xrcc6bp1|68876	-1.33508279	0.209100753	-1.336592672	0.092336591
Nek9|217718	-1.335203997	0.005114041	-1.255692005	0.009029527
Mmp19|58223	-1.335276828	0.075580411	-1.160434891	0.14014469
Aoc3|11754	-1.335355238	0.384009973	-1.906558976	0.209895125
Tef|21685	-1.335475113	0.022106837	-1.418138115	0.005884095
2810403A07Rik|74200	-1.335573567	0.129798709	-1.335573567	0.129798709
Bahcc1|268515	-1.335846429	0.183696801	-1.051710481	0.415956888
Clcn7|26373	-1.335943244	0.106283486	-1.335943244	0.106283486
Ints6|18130	-1.336089657	0.148261308	-1.139461229	0.300952037
Hk3|212032	-1.336108028	0.231476967	-1.022829383	0.474131376
Kdm2a|225876	-1.336159255	0.096371608	-1.522237374	0.038288339
Lgals3|16854	-1.336176299	0.132478998	-1.191984544	0.234654743
Kalrn|545156	-1.336188404	0.119683997	-1.590435962	0.033773166
Glud1|14661	-1.336302404	0.003400112	-1.290932059	0.000564967
Pstpip2|19201	-1.336325085	0.17084267	-1.634612536	0.065945999
Rnf31|268749	-1.336331321	0.016936865	-1.521817373	6.53592E-05
Ssx2ip|99167	-1.33639393	0.154342395	-1.75855581	0.014771034
Flt1|14254	-1.336482297	0.181032996	-1.083239238	0.382768715
Wnk4|69847	-1.336813789	0.134255903	-1.336813789	0.134255903
Klhl38|268807	-1.336998492	0.394181862	1	#DIV/0!
Gm11992|626870	-1.336998492	0.394181862	1	#DIV/0!
Akap6|238161	-1.336998492	0.394181862	1	#DIV/0!
Atxn2l|233871	-1.337038585	0.008669799	-1.253822684	0.017000996
P4hb|18453	-1.337077772	0.002120248	-1.297847348	0.004952255
Hist1h1e|50709	-1.337091956	0.366630241	-6.549566667	0.045145192
Gmfb|63985	-1.337435428	0.19012039	-1.158471867	0.310467534
Ppt1|19063	-1.337452156	0.059213454	-1.302331869	0.002030634
Chrnb2|11444	-1.3374547	0.185100075	-1.3374547	0.185100075
Hes6|55927	-1.33747836	0.109339227	-1.319705876	0.084225281
Smug1|71726	-1.337590268	0.243286774	1.13705417	0.358239726
Uba3|22200	-1.33776753	0.109997415	-1.33776753	0.109997415
She|214547	-1.338038583	0.301169082	-1.593624653	0.143195321
Exosc1|66583	-1.338176197	0.02147379	-1.158854828	0.070847325
Upf3b|68134	-1.338272843	0.159267445	-1.123930774	0.326180969
Spen|56381	-1.338501081	0.130994525	-1.573884884	0.050897272
Ctbp2|13017	-1.338738827	0.323385391	1.253162873	0.353490254
Fbxo28|67948	-1.338792618	0.131565105	-1.591378121	0.045363097
Vwce|71768	-1.339018685	0.005798974	-1.259418034	0.006452525
Cep68|216543	-1.339118868	0.114544809	-1.205842837	0.218313743
Zfp672|319475	-1.339257486	0.090620411	-1.244119797	0.157464842
Wwc2|52357	-1.339386058	0.160333038	-1.065780667	0.370847115
Mcm3ap|54387	-1.339800736	0.041954335	-1.239255843	0.085840705
Mboat7|77582	-1.339828193	0.015384697	-1.339828193	0.015384697

Gja5|14613	-1.339888993	0.216315192	-1.635874279	0.055989877
Ccm2|216527	-1.339913283	0.039263086	-1.392719756	0.004877383
Suv39h1|20937	-1.340203924	0.133941603	-1.340203924	0.133941603
Cpsf6|432508	-1.340253692	0.109416692	-1.340253692	0.109416692
Agpat2|67512	-1.340397905	0.023607701	-1.340397905	0.023607701
2410018M08Rik|71970	-1.340438985	0.18795568	-1.065061106	0.407095348
Cryz|12972	-1.340649147	0.021949636	-1.252696718	0.043235125
Nr5a2|26424	-1.340727944	0.233854001	1.039906944	0.445212708
Cherp|27967	-1.340800521	0.037447826	-1.121216056	0.127981699
Eif4ebp2|13688	-1.340874332	0.209895486	-1.564581875	0.138993006
Cradd|12905	-1.341696706	0.007923981	-1.282924005	0.016782507
Dennd1c|70785	-1.341927444	0.167517676	-1.213134838	0.276021277
Acsl1|14081	-1.342078269	0.101303052	-1.171100405	0.210519624
Fnbp4|55935	-1.342353607	0.208307561	-1.033853749	0.454570613
Acr|11434	-1.342422709	0.376959751	1.264185714	0.40907038
Plxnd1|67784	-1.342456234	0.0080049	-1.38867251	0.000201976
Etnk1|75320	-1.342707436	0.118845355	-1.221166708	0.165350616
Gda|14544	-1.342711274	0.201319083	-1.017711315	0.471696111
Gm6788|627788	-1.342760622	0.204865465	-1.475933003	0.090025341
Fam133b|68152	-1.342854019	0.177586981	-1.723285139	0.049047652
Zfp280b|64453	-1.342927439	0.109070489	-1.153607231	0.22292114
Hoga1|67432	-1.342943499	0.001107108	-1.407316863	0.000081076
Zfp292|30046	-1.343082609	0.177043323	-1.120424413	0.350107265
Lrpap1|16976	-1.343273047	0.001252103	-1.296928622	0.003016666
Khk|16548	-1.34345156	0.056794383	-1.304230543	0.043143579
Zfp422|67255	-1.343602494	0.077131589	-1.343602494	0.077131589
Smpd4|77626	-1.343625507	0.064390695	-1.198292138	0.131467343
Hipk2|15258	-1.344169483	0.074671953	-1.735543683	0.00047452
Pomt1|99011	-1.344291767	0.007939054	-1.411854583	0.002067242
Pkd2|18764	-1.344331712	0.245529103	1.08938637	0.374043628
9630033F20Rik|319801	-1.344424956	0.110022251	-1.344424956	0.110022251
Slc35d1|242585	-1.344558694	0.159347344	-1.093808728	0.349362469
Caskin2|140721	-1.344751725	0.057680409	-1.488685401	0.02421375
Adat3|100113398	-1.344755135	0.267160703	1.273735929	0.243989024
Entpd8|72090	-1.344837481	0.002606586	-1.344837481	0.002606586
AA986860|212439	-1.344984168	0.104852972	-1.174505864	0.215646249
B230206F22Rik|78878	-1.345025684	0.192890209	-1.340754799	0.069009306
Plcb1|18795	-1.345073603	0.259389107	-1.729054581	0.149635913
Zfp185|22673	-1.345305628	0.331957862	-2.334383838	0.154268948
Fcer2a|14128	-1.345316498	0.369516808	-1.051233705	0.480889446
H2-DMb2|15000	-1.345463362	0.374343142	-1.379436792	0.355043416
Mettl15|76894	-1.345475007	0.268539601	-1.345475007	0.268539601
Ifit3|15959	-1.345906715	0.037515181	-1.29432553	0.07192693
Antxr2|71914	-1.346021247	0.090728075	-1.473885185	0.032593974
Agmat|75986	-1.346130913	0.017490504	-1.31790127	0.012133234
Ube2d1|216080	-1.346186789	0.133293188	-1.346186789	0.133293188
Serpind1|15160	-1.346416139	0.005128103	-1.270941711	0.010180795
Ndufaf1|69702	-1.346441747	0.049838979	-1.217590645	0.102023949
Rnf115|67845	-1.346542962	0.162307242	-1.124357171	0.331733448
Orc5|26429	-1.346690428	0.040767141	-1.197917368	0.07648288
Med28|66999	-1.346757622	0.013814019	-1.23870566	0.01243618
Zfp319|79233	-1.346835424	0.10800052	-1.123734444	0.227579614
Dut|110074	-1.346887114	0.071455615	-1.210309314	0.146218672

Cebpa|12606	-1.346976006	0.02635659	-1.225506668	0.037014197
Ptprc|19264	-1.346979373	0.198300628	-1.126963952	0.147920313
Csnk1g2|103236	-1.34726091	0.003332371	-1.34726091	0.003332371
C230035I16Rik|320842	-1.34780144	0.391661509	1	#DIV/0!
4930519G04Rik|67593	-1.34780144	0.391661509	1	#DIV/0!
Klrb1b|80782	-1.34780144	0.391661509	1	#DIV/0!
Cpn2|71756	-1.347883479	0.001746789	-1.290500826	0.002531694
Golph3l|229593	-1.348016597	0.191701864	-1.635952563	0.097622336
Zfp687|78266	-1.348123422	0.029848575	-1.234209365	0.060943441
Elac1|114615	-1.348164757	0.020036831	-1.238157008	0.028225682
Edc3|353190	-1.348420303	0.048227736	-1.477132732	0.022204248
Adipoq|11450	-1.348555544	0.409447926	1	#DIV/0!
Fam178a|226151	-1.348658833	0.136332838	-1.02645089	0.439285908
Cdc45|12544	-1.348702716	0.200233393	-1.501180715	0.123793287
1810010H24Rik|69066	-1.348736424	0.221179198	-2.114819753	0.013218768
Wdr25|212198	-1.348741611	0.053070944	-1.090186534	0.172388361
Sphk2|56632	-1.348856953	0.001244283	-1.300222942	0.002947643
Atpaf1|230649	-1.348911999	0.13797448	-1.892530254	0.004146658
Ptpn23|104831	-1.348974548	0.000451019	-1.372806686	0.000526848
Irx1|16371	-1.349019632	0.163860993	-1.349019632	0.163860993
Cd300ld|217305	-1.349070352	0.045230256	-1.364283035	0.016474665
Arhgap26|71302	-1.349300894	0.104969615	-1.126946392	0.21034102
Hlx|15284	-1.349553618	0.017385966	-1.431356272	0.004468666
Zfp426|235028	-1.349801075	0.21434975	1.156628207	0.267821489
Prss36|77613	-1.349830488	0.100830166	-1.507227102	0.028148996
E230029C05Rik|319711	-1.349911456	0.388243206	-3.9746	0.173296754
Nr1i3|12355	-1.350055558	0.006435138	-1.478899237	0.000290984
Med25|75613	-1.350180131	0.016511101	-1.265617648	0.031862831
Fam48a|56790	-1.350205866	0.043295048	-1.211169058	0.086908095
Fbf1|217335	-1.350356162	0.080642738	-1.196393662	0.165672432
Cobra1|58202	-1.350398608	0.011362586	-1.291248544	0.004189617
Ftsj2|68017	-1.350610196	0.095858248	-1.173508391	0.199192278
Ipp|16351	-1.350676056	0.178690678	-1.350676056	0.178690678
Hfe2|69585	-1.350822138	0.014157242	-1.49575268	0.001183003
Akap9|100986	-1.350846503	0.146480908	-1.000728771	0.498268677
Fgfr3|14184	-1.35085553	0.064463413	-1.224066056	0.131708463
Fn3krp|238024	-1.350924946	0.134873179	-1.536862254	0.034808562
Ddx26b|236790	-1.350925335	0.287955466	1.061922605	0.454450904
Stim2|116873	-1.350931469	0.197793018	-1.030806705	0.453518338
Pvr|52118	-1.350978781	0.131534211	-1.522044253	0.04113575
Zfp189|230162	-1.351050521	0.306274506	-1.868402147	0.188726082
BC017158|233913	-1.351338592	0.01687486	-1.269737113	0.036198531
Brpf3|268936	-1.351352532	0.069550235	-1.274592759	0.120884546
Gm7694|665574	-1.351474642	0.253200348	-1.464717511	0.19287666
Ehbp1|216565	-1.351510805	0.160739023	-1.866241731	0.009858682
Plcd1|18799	-1.351671391	0.233885768	-1.351671391	0.233885768
Fchsd1|319262	-1.351930117	0.213452833	-2.16580548	0.015492818
Tmem209|72649	-1.352240322	0.1693354	-1.352240322	0.1693354
Ick|56542	-1.352471862	0.091308435	-1.394024016	0.002347593
Ccdc111|408022	-1.352490219	0.247545134	-2.239852819	0.025496855
Tmem131|56030	-1.352774532	0.059058228	-1.416964469	0.025696171
Crtc1|382056	-1.352795217	0.008966867	-1.434459282	0.001214146
Fam188b|330323	-1.352858329	0.185298438	-1.722981362	0.044657856

Foxj1|15223	-1.352858382	0.352298073	1.230375659	0.37752335
Lemd3|380664	-1.352945127	0.150105214	-1.828390172	0.008903277
Bzw2|66912	-1.353159759	0.018662125	-1.413421582	0.003598916
Snx22|382083	-1.353443429	0.090676035	-1.235976011	0.164937371
Gstt2|14872	-1.353524283	0.064624261	-1.498528341	0.030698248
Atg7|74244	-1.353719131	0.015630086	-1.36827828	0.005383422
Ift81|12589	-1.353784957	0.228630478	-1.132709931	0.385615835
BC016423|105203	-1.35385673	0.275766949	1.098707743	0.414293843
Rgl3|71746	-1.35391588	0.000469541	-1.380408223	0.000489225
Mast3|546071	-1.353926644	0.089394671	-1.223603618	0.168191607
Cdr2|12585	-1.35398064	0.285540033	-1.35398064	0.285540033
Racgap1|26934	-1.353992812	0.343682474	-2.508442186	0.166458789
Tjp3|27375	-1.354012199	0.015792587	-1.270386516	0.033827114
Gm12942|100039968	-1.354068564	0.07675085	-1.354068564	0.07675085
Rhoq|104215	-1.354112948	0.267751258	-1.339642396	0.225313806
Ell3|269344	-1.354225644	0.262858912	-2.303446792	0.038448787
Apaf1|11783	-1.354406892	0.132999828	-1.92953544	0.000644602
Gm4926|237749	-1.354443828	0.29025544	-1.354443828	0.29025544
Tapbpl|213233	-1.35454958	0.02514321	-1.13848528	0.057031545
Vipar|104799	-1.354557992	0.056814095	-1.503428459	0.023774299
Hook3|320191	-1.354651048	0.178732306	-1.164912279	0.252258076
Wbp1|22377	-1.354805744	0.011669287	-1.354805744	0.011669287
Lmtk2|231876	-1.354825564	0.078528037	-1.232130952	0.149490845
Polr2a|20020	-1.354923436	0.011097767	-1.380111949	0.000141044
Shpk|74637	-1.355109201	0.012807557	-1.284641218	0.00072607
Gulo|268756	-1.355117254	0.006829872	-1.349616095	0.004335941
Acot6|217700	-1.355171573	0.386914243	-2.76078	0.173296754
Mcph1|244329	-1.355235317	0.142301636	-1.132375479	0.286426842
1700027D21Rik|76573	-1.355490213	0.287659882	1.034232827	0.475569282
Dpy19l4|381510	-1.355559808	0.253783749	-1.001784054	0.498341388
Abcg1|11307	-1.355561083	0.03744324	-1.468429607	0.006856434
Mustn1|66175	-1.355743511	0.069678314	-1.542861238	0.02245349
Sqrdl|59010	-1.355752182	0.001512866	-1.483676774	9.39701E-05
Gm8369|666926	-1.355943524	0.307794225	-1.355943524	0.307794225
Mks1|380718	-1.356279524	0.228176968	-1.356279524	0.228176968
Tnrc18|231861	-1.356869542	0.021435915	-1.367550206	0.008813541
Ftsjd1|234728	-1.357009557	0.348005399	1.67712829	0.222597488
Slc25a21|217593	-1.357040919	0.042033691	-1.134008215	0.104464007
Ntm|235106	-1.357149171	0.306827708	-1.357149171	0.306827708
Ceacam2|26367	-1.357166634	0.230387007	-2.462930579	0.003499089
Fbrsl1|381668	-1.357208561	0.120467445	-1.601962211	0.044724871
Gc|14473	-1.357341828	0.029974818	-1.566305141	0.000734528
Pnp2|667034	-1.35744614	0.030238418	-1.242659823	0.055226126
Naa15|74838	-1.357450466	0.026902558	-1.427552562	0.000536357
Ttr|22139	-1.357712487	0.140131155	-1.188744542	0.135277413
Cnot4|53621	-1.357904564	0.049730031	-1.357904564	0.049730031
Hp|15439	-1.358056591	0.108983424	-1.358056591	0.108983424
Gak|231580	-1.358225329	0.003743152	-1.322993221	0.009024383
Cd2ap|12488	-1.358494988	0.18891759	-1.009071399	0.481253534
Smad4|17128	-1.358586155	0.096878269	-1.396128548	0.029738718
Hyi|68180	-1.358648994	0.10793397	-1.717908918	0.007885661
Ptprb|19263	-1.358800919	0.118801104	-1.2921243	0.030548034
Ccdc41|77048	-1.359029207	0.109645877	-1.4569585	0.036772395

Inpp5b|16330	-1.359189249	0.03494717	-1.129561371	0.11180282
Rtn4rl2|269295	-1.359226097	0.171442156	1.017579567	0.473078734
Slc22a26|236149	-1.359331454	0.058561148	-1.43905623	0.020896037
Gm4144|100042980	-1.35965	0.170446566	1	#DIV/0!
Gm8453|667094	-1.35965	0.170446566	1	#DIV/0!
Slc25a45|107375	-1.359674725	0.027061577	-1.211215188	0.041847437
AA415398|433752	-1.35974668	0.072866563	-1.477305797	0.027240578
Lrrc46|69297	-1.359755453	0.257236997	-1.437788618	0.170137979
Uchl4|93841	-1.35989871	0.103669178	-1.545173812	0.015195097
Ggh|14590	-1.360090134	0.12463482	-1.597449699	0.051812096
Rragb|245670	-1.360222857	0.351764186	-3.967316667	0.09324139
Cyp3a44|337924	-1.360333784	0.23081477	-1.637200477	0.091007512
Sftpa1|20387	-1.36053594	0.273616688	-1.038255141	0.472092654
Rbms2|56516	-1.360691735	0.009977377	-1.399389928	0.011137678
Kri1|215194	-1.360716788	0.04765346	-1.533663994	0.01271131
Wdr24|268933	-1.360769327	0.003249779	-1.443772897	8.07141E-05
Dnajc14|74330	-1.361023981	0.010909849	-1.353752217	0.003049797
Prr16|71373	-1.361030535	0.189869849	-1.124142654	0.364192226
Cks1b|54124	-1.361057538	0.060208019	-1.21821474	0.122658738
Srebf2|20788	-1.361091965	0.0801247	-1.079917686	0.291898496
Ankrd12|106585	-1.361884933	0.115251451	-1.361884933	0.115251451
Cep250|16328	-1.362118163	0.057623194	-1.208682797	0.11757834
Hao2|56185	-1.362225	0.32369376	-4.234332783	0.01414089
Zfp192|93681	-1.362619614	0.171098346	-1.313278793	0.105459378
Exog|208194	-1.362841906	0.180207378	-1.362841906	0.180207378
Dock5|68813	-1.362947099	0.028187547	-1.240982353	0.046346443
1300010F03Rik|219189	-1.363569531	0.044846092	-1.363569531	0.044846092
Klhl25|207952	-1.363693305	0.019799636	-1.319687487	0.008461778
Slc25a29|214663	-1.363752862	0.149141563	1.033758702	0.426379199
Batf3|381319	-1.363919663	0.237626509	1.255084747	0.15148536
Mab21l2|23937	-1.363920042	0.17485789	-1.137603476	0.3409462
Dok2|13449	-1.363931927	0.17567716	-1.699126233	0.071168731
Plch1|269437	-1.363981872	0.400533235	1	#DIV/0!
Tbc1d5|72238	-1.364093725	0.001214459	-1.364093725	0.001214459
Itgb4|192897	-1.364503043	0.178247717	-1.364503043	0.178247717
Dhcr24|74754	-1.365303118	0.005735577	-1.42560345	0.00434391
Fblim1|74202	-1.365443945	0.127416386	-1.365443945	0.127416386
Daam2|76441	-1.365456668	0.148897683	-1.365456668	0.148897683
Slc1a5|20514	-1.36582096	0.080586239	-1.300100129	0.136640923
Zdhhc16|74168	-1.365898231	0.014970877	-1.415786632	0.010897557
Kl|16591	-1.365994286	0.34992363	-3.98415	0.093264411
Acy1|109652	-1.366139071	0.010448511	-1.366139071	0.010448511
Crhr2|12922	-1.366357574	0.325198799	-2.287459648	0.164128923
Sacm1l|83493	-1.366423267	0.170345477	1.053212231	0.408398892
Zscan20|269585	-1.366484569	0.251112733	-1.662240025	0.156620493
Ptprf|19268	-1.366819886	0.009683023	-1.450698452	0.001535417
Dnase1l3|13421	-1.366821433	0.013247293	-1.319157091	0.011367569
Noxo1|71893	-1.366848078	0.129176875	-1.366848078	0.129176875
Cyp2d13|68444	-1.366919496	0.035429045	-1.293807057	0.069048645
Slc4a4|54403	-1.367030259	0.22246785	-1.223149946	0.22512822
Ocel1|77090	-1.367257715	0.266048113	-2.001607601	0.110569796
Prss53|330657	-1.367273951	0.132583512	-1.278925444	0.073883476
Srpk2|20817	-1.367431613	0.080483645	-1.517245604	0.018362506

Ino80d|227195	-1.367483995	0.081792616	-1.696254046	0.006815997
Farp2|227377	-1.367500287	0.121367117	-1.154644785	0.256790551
Lamb1|16777	-1.367627989	0.098433408	-1.367627989	0.098433408
Esyt1|23943	-1.367668177	0.013839087	-1.367668177	0.013839087
Nckap5l|380969	-1.367726317	0.246785198	-1.414730654	0.133070055
Ccdc64b|212733	-1.367738506	0.325720761	-2.289771507	0.165889172
Aspa|11484	-1.368037193	0.195979893	-1.077705491	0.407397778
Exoc4|20336	-1.36813263	0.164044486	-1.144472865	0.307562581
Alad|17025	-1.36819759	0.015974891	-1.311572256	0.010347948
Lypla1|18777	-1.36864174	0.137233297	-1.261640564	0.126642024
Tmem104|320534	-1.368651056	0.008158954	-1.452744859	0.004044425
Abcc2|12780	-1.368857029	0.089987546	-1.204624068	0.185050868
A730011L01Rik|338371	-1.368878773	0.020234649	-1.35931623	0.009803814
Tada2b|231151	-1.369042063	0.125252076	-1.543522624	0.038943372
Ttc14|67120	-1.369129473	0.138661226	-1.342349642	0.137234493
Dlg5|71228	-1.369157736	0.18085997	-1.1936258	0.293753962
Eif2c2|239528	-1.369214479	0.271922774	-2.013251401	0.118869322
Dsg2|13511	-1.369707525	0.045251509	-1.369707525	0.045251509
Ugdh|22235	-1.369952706	0.032030463	-1.626077109	0.001118807
Zfp646|233905	-1.370008306	0.019566713	-1.284759113	0.040049175
Slc30a7|66500	-1.370033381	0.023795248	-1.456242066	0.007983001
Sepx1|27361	-1.370060393	0.049456969	-1.354383063	0.020952244
Lrp1|16971	-1.370331359	0.003154292	-1.439130954	0.00174441
Ccdc37|243538	-1.370621322	0.370686858	-7.725	0.072984714
Kif3c|16570	-1.370680117	0.088160758	-1.207067629	0.174946714
Aars|234734	-1.370689085	0.011602659	-1.349068073	0.006222731
Fam57b|68952	-1.370695837	0.240445758	-2.345509408	0.018096045
Ppap2b|67916	-1.37082388	0.13255223	-1.21884052	0.229892141
Mllt3|70122	-1.370847377	0.166283797	-1.372666733	0.021663419
Mlst8|56716	-1.370923446	0.035917464	-1.602167559	0.001404432
Neil1|72774	-1.370986952	0.181791007	-1.318458353	0.167099588
Mettl9|59052	-1.371239501	0.037022975	-1.507427719	0.015196515
Myo18b|74376	-1.371468822	0.365548643	-2.76078	0.173296754
Larp6|67557	-1.371468822	0.365548643	-2.76078	0.173296754
Zfp820|75424	-1.371468822	0.365548643	-2.76078	0.173296754
Ifitm3|66141	-1.371630126	0.034358364	-1.539697337	0.007719524
Clec1a|243653	-1.371676929	0.297558623	1.029360642	0.481678818
Cyp2c29|13095	-1.371839433	0.041943324	-1.196713363	0.030096724
Ino80b|70020	-1.371921632	0.046365524	-1.371921632	0.046365524
Polr3g|67486	-1.372344805	0.210589744	-1.184565558	0.25915258
Atrn|11990	-1.372425956	0.142762446	-1.095989391	0.320857335
Timd4|276891	-1.372433331	0.086246895	-1.728194322	0.004431072
Il2rg|16186	-1.37243927	0.104054728	-1.608733896	0.0381154
Neu2|23956	-1.372606798	0.203553081	-1.638049745	0.061439593
Fam82a1|381110	-1.372633413	0.150166478	-1.317541194	0.143284698
Nbn|27354	-1.372748962	0.030863666	-1.372748962	0.030863666
Golim4|73124	-1.37282893	0.208525486	-1.085967229	0.410604126
Pax1|18503	-1.37283176	0.355805489	-4.56078	0.070574133
Wfdc1|67866	-1.372931143	0.214769833	-1.075099504	0.423329787
Kctd12|239217	-1.373110937	0.090851235	-1.524936827	0.054333965
Klf12|16597	-1.373152972	0.18894986	-1.079360156	0.400999481
Atp11c|320940	-1.373206103	0.235581695	-1.141186572	0.297240828
Haao|107766	-1.373316041	0.016541094	-1.372872023	0.005802739

Arhgef11|213498	-1.373484362	0.024731256	-1.507379146	0.008064605
Fcho1|74015	-1.373821277	0.188537467	-2.10593426	0.005231011
Zc3h13|67302	-1.373965271	0.0747422	-1.409910673	0.029294088
Fam63b|235461	-1.374124508	0.231890485	-2.413903026	0.012572329
2010003K15Rik|75606	-1.374127227	0.014372685	-1.36011306	0.008334385
Eif2ak2|19106	-1.374330386	0.052411328	-1.459944777	0.004385075
Pdcd10|56426	-1.37433759	0.11823498	-1.047927753	0.408104341
Sult1b1|56362	-1.374339955	0.118075272	-1.684687948	0.004449345
Sema4g|26456	-1.374396002	0.04036705	-1.225329552	0.064791735
Smyd3|69726	-1.374522229	0.159182166	-1.374522229	0.159182166
Cenpt|320394	-1.374525457	0.159482782	-1.153225218	0.298339705
Aldh8a1|237320	-1.374813137	0.001951073	-1.447572783	0.000834493
Slit1|20562	-1.37500106	0.3243873	-2.456106061	0.148633746
Phlpp1|98432	-1.375134699	0.119368529	-1.375134699	0.119368529
Crot|74114	-1.375231964	0.099610864	-1.429648123	0.063731584
Zscan12|22758	-1.375316728	0.119205284	-1.252624676	0.212145694
BC017647|216971	-1.375505471	0.004956877	-1.328577475	0.011478424
Bod1l|665775	-1.376123758	0.137019466	-1.13497338	0.294454237
Fbln5|23876	-1.376196442	0.16930265	-1.219152137	0.228148132
Serpina3g|20715	-1.376308033	0.027086867	-1.293575354	0.002937639
Fam101b|76566	-1.376472892	0.260540935	-2.55009207	0.023662449
Mir686|751525	-1.376565313	0.356750222	-8.263164218	0.038200272
Arhgef5|54324	-1.376693266	0.024121989	-1.394281272	0.0081283
Ikzf1|22778	-1.376751578	0.279172331	-1.244661992	0.336049189
Epb4.1l3|13823	-1.376767551	0.271687252	-1.376767551	0.271687252
Lonp2|66887	-1.376787795	0.00580144	-1.310609179	0.011553562
Mapk4|225724	-1.376907849	0.262228954	-1.376907849	0.262228954
2310003H01Rik|71885	-1.37713444	0.075022103	-1.205810111	0.15199518
B230118H07Rik|68170	-1.377191807	0.036566825	-1.234338276	0.059241915
Gbp7|229900	-1.377264395	0.132253397	-1.098989152	0.290328467
Thumpd3|14911	-1.377388431	0.084708987	-1.177075523	0.174440926
Gm3414|100041576	-1.377689939	0.322765172	-2.460909091	0.146965028
Vav2|22325	-1.377750989	0.211561484	1.124796275	0.341776157
Gm13308|621580	-1.377877103	0.345406301	1.298566723	0.385674711
Dio1|13370	-1.377885862	0.007641207	-1.445686895	0.005534754
Gnao1|14681	-1.378009589	0.262146083	-1.517362477	0.193892634
41160|20362	-1.378037872	0.090526693	-1.069932851	0.343539825
Cyp2f2|13107	-1.378092154	0.017802252	-1.510038291	0.004876936
Pibf1|52023	-1.378144715	0.309109916	-2.492723342	0.081213626
Sik2|235344	-1.378287074	0.230663695	-1.448296524	0.192518363
Gtf3a|66596	-1.378433077	0.146135253	-1.378433077	0.146135253
Atg3|67841	-1.37845722	0.004993745	-1.458645486	0.000567676
Yars2|70120	-1.378721373	0.080438385	-1.46879629	0.051246532
Nr2f2|11819	-1.378748817	0.067476549	-1.32869998	0.076977578
Dynlt1a|100310872	-1.378965642	0.318380277	-1.397166728	0.337866371
Rbm20|73713	-1.379111652	0.228659709	-1.704742563	0.141481094
Tubg1|103733	-1.379161438	0.009409663	-1.458450088	0.005767648
Aaas|223921	-1.379237503	0.146531564	-1.84072627	0.018259453
Psip1|101739	-1.379451967	0.05293051	-1.379451967	0.05293051
Gba|14466	-1.379566846	0.01627601	-1.379566846	0.01627601
Tpcn1|252972	-1.379687592	0.000276515	-1.379687592	0.000276515
Bcl2|12043	-1.379878053	0.285220517	-1.834027078	0.171077072
Srsf11|69207	-1.379919415	0.190984655	1.068641153	0.406717095

Car9|230099	-1.380048213	0.362834977	-2.8	0.173296754
Prodh|19125	-1.380091954	0.003295134	-1.345513877	0.000715713
Odf2|18286	-1.380268176	0.148562695	-1.74214934	0.043210081
Eda|13607	-1.38042249	0.194935966	-1.669980239	0.113370679
Sdccag3|68112	-1.380578979	0.00455344	-1.42667383	0.001603199
Gmppa|69080	-1.38075742	0.058830824	-1.136961065	0.212972903
Fam188a|66960	-1.380769044	0.19274176	-1.075620797	0.408782705
Clcn2|12724	-1.380796216	0.001078411	-1.380796216	0.001078411
Elmo3|234683	-1.380964057	0.009021394	-1.539673917	0.000354513
Aim1l|230806	-1.3810004	0.194075377	-1.718475033	0.097951795
Pold2|18972	-1.381481303	0.046101881	-1.381481303	0.046101881
Ccrn4l|12457	-1.381582378	0.102283491	-1.373219294	0.054161493
Rbl2|19651	-1.381728712	0.219433443	-1.606817199	0.087484785
Ispd|75847	-1.381975385	0.27162601	-1.612452991	0.182349547
Rom1|19881	-1.38220252	0.074630213	-1.180347872	0.151582095
Baz1a|217578	-1.382271076	0.114112312	-1.256434961	0.206225732
Trim44|80985	-1.382340488	0.129399691	-1.581349814	0.07586868
Nod2|257632	-1.382378653	0.341144726	-1.382378653	0.341144726
Tnfrsf21|94185	-1.382401241	0.130170034	-1.696585706	0.040641712
Cnih4|98417	-1.382480124	0.002098227	-1.45484347	0.000181572
Acly|104112	-1.382519471	0.086740959	-1.19126724	0.177724908
Zfp799|240064	-1.382542426	0.174937073	-2.217059735	0.00209878
Cdkn2aip|70925	-1.382803012	0.149969622	-1.382803012	0.149969622
Tram2|170829	-1.382850482	0.142327196	-1.113388742	0.314541002
Arid1a|93760	-1.382873553	0.117937065	-1.113237768	0.24074699
Zfc3h1|216345	-1.382931286	0.155271549	-1.066013813	0.364890319
Pkp3|56460	-1.382997547	0.095073759	-1.163956027	0.192015432
Snrnp25|78372	-1.383003557	0.198747425	-1.383003557	0.198747425
Plcxd1|403178	-1.383215676	0.109333066	-1.217264147	0.204764569
Naaa|67111	-1.383479856	0.150560821	-1.142206583	0.313624281
St3gal3|20441	-1.383577558	0.004338734	-1.32440716	0.01020353
Mme|17380	-1.384098766	0.207274372	1.173122546	0.223982008
Thnsl1|208967	-1.384191483	0.234362869	1.194728796	0.288001375
Cd4|12504	-1.384388638	0.05988025	-1.384549973	0.060509026
2410091C18Rik|73694	-1.384421546	0.045940671	-1.218109993	0.070040812
Ttc15|217449	-1.38452783	0.049543858	-1.511475417	0.028437143
Gpr39|71111	-1.384635554	0.097251678	-1.601113837	0.005790899
Rdh16|19683	-1.384888276	0.079670658	-1.51544462	0.016299959
Klhl17|231003	-1.385075579	0.026068959	-1.498962386	0.013241755
Mmp9|17395	-1.385150748	0.235527833	-1.089293569	0.425973545
Crkl|12929	-1.385193796	0.274763364	-1.385193796	0.274763364
Postn|50706	-1.385228535	0.189138251	-1.537224751	0.12557546
Kif11|16551	-1.385313689	0.315371574	-1.385313689	0.315371574
Klb|83379	-1.386007476	0.039487781	-1.298046008	0.030418116
Ccnl1|56706	-1.386043081	0.12653697	-1.186531669	0.256718888
Hvcn1|74096	-1.386153923	0.156397706	-1.155794774	0.31397169
Cyp2c50|107141	-1.386525958	0.126993994	-1.115032291	0.275718862
Huwe1|59026	-1.386628779	0.047758473	-1.297411812	0.045304074
Evi5|14020	-1.38679163	0.16075036	1.015914331	0.468835704
Rad54l|19366	-1.386799788	0.30480756	-3.356105893	0.038371173
Vldlr|22359	-1.386960215	0.201270543	-1.270852999	0.214720014
Zfp763|73451	-1.387952655	0.317155729	-1.903582746	0.19072579
Rtn1|104001	-1.388110974	0.260484873	-1.842196518	0.152968183

Pde1b|18574	-1.388471312	0.200211628	-1.180343835	0.342890587
Camk2a|12322	-1.388521756	0.297796367	-2.391230356	0.116468777
Arhgap32|330914	-1.388564699	0.167885339	-1.376547679	0.158314968
Gse1|382034	-1.388667786	0.197801024	-1.638785125	0.131695203
Gm5465|432879	-1.388883333	0.170446566	1	#DIV/0!
6230400D17Rik|76133	-1.388902728	0.231855875	-1.165483644	0.357431858
Sc5d|235293	-1.389275205	0.053835562	-1.273732513	0.023762986
C1qtnf7|109323	-1.389545849	0.279829777	-3.010586893	0.023511985
Zfand1|66361	-1.389815682	0.165590607	-1.389815682	0.165590607
Noa1|56412	-1.390264982	0.029227972	-1.540755162	0.009423792
Trpm4|68667	-1.390560948	0.077666283	-1.552015943	0.015741031
Zc3h11a|70579	-1.390592353	0.132174864	-1.390592353	0.132174864
Inf2|70435	-1.390718507	0.000426567	-1.407281491	0.000841315
Dcun1d4|100737	-1.390899117	0.121369066	-1.060279308	0.395816383
Vps41|218035	-1.390970243	0.028676185	-1.37086041	0.00740971
Atm|11920	-1.391002034	0.222220319	-1.938700635	0.081856295
Zfp180|210135	-1.391143009	0.119674046	-1.492384976	0.044479407
Gjb2|14619	-1.391193476	0.042443507	-1.135955433	0.146632485
Ido2|209176	-1.391398638	0.016612676	-1.391398638	0.016612676
Osbpl5|79196	-1.39146991	0.109670302	-1.408156722	0.089242275
Mgam|232714	-1.391580247	0.020583374	-1.506286999	0.009653938
Ncoa6|56406	-1.391721138	0.196877321	-1.278717648	0.245947453
Metap1d|66559	-1.391795808	0.015977309	-1.483564869	0.003792987
Gpr110|77596	-1.391928303	0.278993051	-1.391928303	0.278993051
Ubap2|68926	-1.392022777	0.116252826	-1.595079071	0.064426589
Rgl1|19731	-1.392122216	0.053126673	-1.392122216	0.053126673
BC005537|79555	-1.392319256	0.162467093	-1.307599354	0.19279403
Cmpk2|22169	-1.392346524	0.169410614	-1.226486976	0.288287299
Bcl7a|77045	-1.392677348	0.027120386	-1.163116866	0.087621202
S1pr1|13609	-1.392838823	0.08754642	-1.180234429	0.174527771
Trim46|360213	-1.392862431	0.303053729	-3.088025253	0.059099193
Vangl1|229658	-1.392959969	0.097607403	-1.366251841	0.065099975
Fzd1|14362	-1.393095078	0.153460669	-1.20125434	0.268131068
Cyp2e1|13106	-1.39325762	0.038138293	-1.261337684	0.073428732
Mafb|16658	-1.393524677	0.047690005	-1.272314597	0.097594958
Gm5069|277333	-1.393922029	0.091836311	-1.276186884	0.16057112
Clec16a|74374	-1.394194668	0.038525958	-1.30430756	0.078924482
Masp1|17174	-1.394372375	0.088370854	-1.375354556	0.065218534
Cyp2b9|13094	-1.39440033	0.02530359	-1.39440033	0.02530359
Lrch1|380916	-1.394498864	0.062938894	-1.394498864	0.062938894
Aqr|11834	-1.394819505	0.038679656	-1.551430928	0.014675793
Ing5|66262	-1.394963033	0.109310717	-1.662112837	0.039777711
Rapgef2|76089	-1.395038682	0.186026539	-1.032543746	0.447650408
Snai2|20583	-1.395054898	0.197535227	-1.247063581	0.181364675
Loxl1|16949	-1.395670553	0.186122766	-2.24976513	0.007408039
Slc46a3|71706	-1.395858563	0.012379634	-1.317732051	0.025716046
Nfix|18032	-1.396133801	0.234748644	-1.179973133	0.292289683
Adck1|72113	-1.396543439	0.04193251	-1.624253327	0.005416471
Igtp|16145	-1.396565322	0.0010129	-1.342090453	0.001752293
Rdx|19684	-1.396663459	0.144780234	-1.189687249	0.192364698
Fam189b|68521	-1.396717296	0.258872616	-1.036661912	0.472620163
Rbms3|207181	-1.396830066	0.289216007	-2.370158645	0.110597757
Ddb2|107986	-1.396908202	0.266470036	1.119222435	0.395387717

Abcc10|224814	-1.397336525	0.055167668	-1.757414999	0.001130136
Tpk1|29807	-1.397402766	0.086703459	-1.825288334	0.006802134
1190007I07Rik|544717	-1.397416249	0.124652571	-1.148529968	0.268043013
Ncf2|17970	-1.39767543	0.191379085	-1.39767543	0.191379085
Med15|94112	-1.397694534	0.050421427	-1.131490954	0.192059388
Mapkapk3|102626	-1.397936532	0.199282005	1.073114991	0.40938713
Gpd1|14555	-1.398168875	0.004131419	-1.347850643	0.009543132
Pdgfa|18590	-1.398219388	0.244183524	-1.063214385	0.449805873
1300018I17Rik|72325	-1.398293107	0.028466097	-1.161497761	0.082701263
Grlf1|232906	-1.398328048	0.046196404	-1.235089506	0.079503591
Pipox|19193	-1.398776285	4.04544E-05	-1.441602628	3.02541E-05
Mdm4|17248	-1.398818884	0.277935505	-1.749027877	0.159597467
Wasf1|83767	-1.398881142	0.168150962	-1.398881142	0.168150962
Adamts7|108153	-1.398886439	0.16353285	-1.170672938	0.205504817
Nqo2|18105	-1.399153412	0.0544023	-1.254927357	0.110000972
Tctex1d2|66061	-1.399198323	0.292822829	1.261638371	0.310538711
Rabif|98710	-1.399225809	0.040711047	-1.399225809	0.040711047
Mtap1s|270058	-1.399388446	0.059522741	-1.398559327	0.053224869
Ubiad1|71707	-1.399487494	0.12773291	-1.399487494	0.12773291
Evc|59056	-1.399614702	0.121369993	-1.717418482	0.037781344
Acad10|71985	-1.399714728	0.003250499	-1.544995845	5.85779E-05
Ifi47|15953	-1.399886492	0.101486161	-1.189879008	0.211981759
Memo1|76890	-1.400067583	0.025404259	-1.342245296	0.016114482
Epn2|13855	-1.400160256	0.026868099	-1.466271088	0.024698763
Ccdc68|381175	-1.400308529	0.212769544	1.141221796	0.328795646
Fes|14159	-1.400334461	0.051335208	-1.255172746	0.10271895
Trpm2|28240	-1.400468943	0.156365616	-1.891593714	0.028230363
9030617O03Rik|217830	-1.400510987	0.001499539	-1.400510987	0.001499539
Fam122a|68034	-1.400887126	0.039361736	-1.400887126	0.039361736
Clip2|269713	-1.401230547	0.104806492	-1.011446674	0.46403691
Gpx6|75512	-1.401304222	0.333146906	1.52036734	0.280467625
Saa4|20211	-1.401395602	0.049233417	-1.251074693	0.096070989
Spata7|104871	-1.401434453	0.315381505	-1.401434453	0.315381505
Bco2|170752	-1.401594578	0.03058228	-1.534527963	0.003129076
Hck|15162	-1.401717193	0.089729336	-1.591665576	0.013647685
Frzb|20378	-1.402133561	0.268924107	1.023331014	0.482819656
Srbd1|78586	-1.402644168	0.157556522	-1.402644168	0.157556522
Nup43|69912	-1.402713733	0.18479868	-1.612141871	0.086431447
Cyb5r3|109754	-1.40278312	0.009951249	-1.40278312	0.009951249
Dusp12|80915	-1.402839273	0.064024259	-1.371849997	0.03014364
Cxcr3|12766	-1.4028895	0.298789489	1.190041347	0.384964438
Gvin1|74558	-1.403101033	0.267324374	-1.025776776	0.481932633
Pacs1|107975	-1.403143396	0.076197213	-1.562224772	0.017807812
Nt5e|23959	-1.403344388	0.092602344	-1.268725357	0.16654517
Cdc42ep3|260409	-1.403381438	0.173719881	-1.59328595	0.086403434
Rabgap1l|29809	-1.403415996	0.057130078	-1.321524022	0.10132577
Parp14|547253	-1.403474022	0.025002673	-1.599281644	0.003034164
4933403F05Rik|108654	-1.403555754	0.03234927	-1.256327483	0.050622205
Cd19|12478	-1.40360422	0.280111284	-2.071762477	0.043677989
Fgd3|30938	-1.403877997	0.380314069	1.264185714	0.40907038
Ophn1|94190	-1.403898225	0.066098939	-1.534755373	0.043946904
R3hdm1|226412	-1.403948107	0.132356612	-1.202330299	0.142205943
Cd97|26364	-1.404154892	0.034099183	-1.404154892	0.034099183

BC029214|227622	-1.404707792	0.150384308	-1.420022616	0.113223191
Clec11a|20256	-1.404743262	0.150899133	-1.959801993	0.014742001
Oasl2|23962	-1.404809191	0.093631936	-1.619347537	0.043680835
Zmym2|76007	-1.405535725	0.121704985	-1.405535725	0.121704985
Tmc6|217353	-1.40566478	0.069332035	-1.226218686	0.138799428
Npepl1|228961	-1.405767187	0.000643752	-1.458382423	0.000530498
D6Ertd527e|52372	-1.40616	0.375963643	1	#DIV/0!
Zfp788|67607	-1.406208007	0.27759175	-1.406208007	0.27759175
Sh3yl1|24057	-1.406427249	0.03927316	-1.239661095	0.031373244
Apobec1|11810	-1.406560556	0.057283137	-1.253023527	0.115501782
Tm6sf2|107770	-1.406671323	0.005383758	-1.406671323	0.005383758
0610007P08Rik|76251	-1.406855895	0.104903251	-1.235822548	0.196086947
Acot4|171282	-1.407165002	0.010809877	-1.528112661	0.003584439
Acrbp|54137	-1.407204667	0.01871488	-1.325859856	0.039679899
Gtse1|29870	-1.407346838	0.251479021	-1.653455991	0.15800477
Slc25a36|192287	-1.407837697	0.193752387	-1.21624931	0.321824738
Nodal|18119	-1.407848561	0.320727913	-2.699824713	0.141377162
Ing3|71777	-1.408281164	0.078292316	-1.629962263	0.03013198
2210009G21Rik|74243	-1.408450704	0.376110186	1	#DIV/0!
Ccbe1|320924	-1.40857499	0.198211437	-1.105299181	0.395067858
Hyls1|76832	-1.409122588	0.200107895	-1.675877995	0.071822305
Atp11a|50770	-1.409285541	0.108683266	-1.045522533	0.407104272
Myl4|17896	-1.409424705	0.216035884	-1.065315382	0.437142415
Stxbp5l|207227	-1.409574731	0.310604314	-2.44590404	0.149033783
Cldn3|12739	-1.409848681	0.035976292	-1.558294525	0.002787465
Ptgis|19223	-1.410102794	0.197371742	1.143837416	0.281559745
A830010M20Rik|231570	-1.410350966	0.296325252	-2.704623563	0.095200291
Lactb|80907	-1.410617041	0.021552819	-1.267830555	0.022825139
Gpd2|14571	-1.410646816	0.042382876	-1.145666575	0.154838858
Itfg1|71927	-1.410663953	0.039146942	-1.317563624	0.024445473
2410127L17Rik|67383	-1.410908837	0.106011217	-1.550521042	0.051675711
Phf12|268448	-1.410931058	0.066579681	-1.475825654	0.030743921
Prlr|19116	-1.411181855	0.023890403	-1.536911231	0.011494546
1700018L02Rik|67329	-1.411649739	0.154815327	-1.371474752	0.117680419
Dhrs7|66375	-1.411787062	0.002498953	-1.276217807	0.006941464
Acsf2|264895	-1.411919407	0.003855852	-1.446699544	0.000816466
Dnajc28|246738	-1.412209803	0.075966111	-1.13700508	0.214017071
Acss3|380660	-1.412600775	0.293832544	-1.559277338	0.195837
Slc17a3|105355	-1.412801345	0.008063261	-1.30685468	0.015582352
Gga3|260302	-1.412839786	0.128002722	-1.863363476	0.018721771
Ecscr|68545	-1.412996723	0.089892027	-1.230776394	0.176860363
Cby1|73739	-1.413106992	0.054634283	-1.388585698	0.020951934
Ankrd49|56503	-1.413504215	0.154946117	-1.364895688	0.096917227
Hirip3|233876	-1.413521382	0.074846854	-1.588289568	0.040524447
Anpep|16790	-1.41362881	3.34942E-05	-1.45062851	1.20536E-05
Mmp15|17388	-1.41367882	0.001868105	-1.41367882	0.001868105
Pdxk|216134	-1.413732439	0.003848413	-1.572966343	9.18979E-05
Kbtbd11|74901	-1.413814902	0.272771105	-1.80018926	0.145920046
Brd4|57261	-1.413960717	0.159794969	-1.210884738	0.205818038
Chrnb1|11443	-1.413969466	0.287928252	-2.420672074	0.114888232
Pygo1|72135	-1.414025049	0.30814708	-2.453626263	0.146761448
Ipo7|233726	-1.414041046	0.186005104	-1.414041046	0.186005104
Ltbp1|268977	-1.414339315	0.28138026	-3.170216909	0.03206401

Afap1l2|226250	-1.41436273	0.275988159	-1.035522573	0.477609645
Cyp2c54|404195	-1.414389628	0.128593206	1.00537599	0.484911039
Alg13|67574	-1.414714235	0.163560971	-1.414714235	0.163560971
2610528J11Rik|66451	-1.414977942	0.005571439	-1.428686755	0.001966722
Ipo8|320727	-1.415417977	0.100369617	-1.047482485	0.390211865
Fam171a2|217219	-1.415471234	0.121527105	-1.031229894	0.441747739
Ccdc159|67119	-1.415483367	0.101771049	-1.064174548	0.372158673
Hars2|70791	-1.415603876	0.060912347	-1.43334227	0.035001552
Phf6|70998	-1.415915493	0.049332544	-1.135138399	0.120333184
Sdccag8|76816	-1.416189228	0.115175905	-1.52767969	0.038681624
Pclo|26875	-1.416291337	0.356657582	-2.76078	0.173296754
Dfna5|54722	-1.416317681	0.074050663	-1.269946816	0.149604302
Cdc25b|12531	-1.41646556	0.176859855	-1.693589215	0.043408505
Arhgef40|268739	-1.416593693	0.03231154	-1.458104334	0.004855699
Tiam2|24001	-1.417031251	0.19850059	-2.198513623	0.025170376
Zfp942|73233	-1.417194586	0.239235462	-1.187512232	0.356355769
Fam131a|78408	-1.41727279	0.225856265	-2.498591419	0.016722945
Rhoj|80837	-1.417533676	0.07985509	-1.204064809	0.157083228
Esrp2|77411	-1.417944438	0.030623474	-1.417944438	0.030623474
D14Ertd668e|219132	-1.418120739	0.087328045	-1.217517434	0.180065862
Ugt2b34|100727	-1.418268508	0.059664463	-1.558092349	0.037643108
Snapin|20615	-1.418300856	0.094047623	-1.589327276	0.027426518
Gnpnat1|54342	-1.418500646	0.009874293	-1.418500646	0.009874293
St6gal1|20440	-1.418521845	0.007538767	-1.418521845	0.007538767
Tenc1|209039	-1.418583842	0.008293733	-1.418583842	0.008293733
Gsto2|68214	-1.418732965	0.284414672	-1.418732965	0.284414672
Mir135a-1|387153	-1.419459524	0.146733581	-1.184110487	0.275247336
Ylpm1|56531	-1.419511097	0.162054554	-1.047050378	0.396716938
Socs6|54607	-1.419585041	0.078706044	-1.626468011	0.014810649
Ankrd23|78321	-1.419830056	0.234351954	-2.063826636	0.091888324
Slc26a1|231583	-1.420024539	0.052049001	-1.132222473	0.198536506
Tia1|21841	-1.420109931	0.123824387	-1.149744272	0.267251144
Depdc5|277854	-1.420328972	0.058568243	-1.217969747	0.116973552
Ncor2|20602	-1.420761215	0.042753081	-1.453715295	0.016468355
Car8|12319	-1.420802686	0.00965154	-1.366314876	0.021171535
Tomm40l|641376	-1.421160482	0.00873611	-1.506951109	0.005761983
Zfp64|22722	-1.421661414	0.012029204	-1.381401471	0.025643021
Fnip1|216742	-1.422132145	0.231876193	-1.220076656	0.335571855
Plcl1|227120	-1.422617218	0.327887737	1.527583413	0.276310115
Tbc1d10c|108995	-1.422715398	0.277198657	-1.231308931	0.296562833
Ptpra|19262	-1.422809921	0.035616182	-1.14106711	0.075137911
Meg3|17263	-1.422911612	0.258409597	1.04937929	0.460618226
Zfp229|381067	-1.423168575	0.229815127	-1.423168575	0.229815127
Mbd6|110962	-1.42344178	0.061624965	-1.13206525	0.198890229
Zfp579|68490	-1.423688741	0.008864064	-1.423688741	0.008864064
Sfpi1|20375	-1.423764074	0.025596093	-1.291613917	0.04455389
Pank3|211347	-1.42410865	0.145604673	-1.084851618	0.333451451
Spn|20737	-1.424365949	0.169305655	-1.424365949	0.169305655
Mllt10|17354	-1.424656684	0.150460793	-1.664873945	0.094181156
Inhbb|16324	-1.424696719	0.211669317	-1.512123214	0.14609309
Tbce|70430	-1.424753242	0.104739075	-1.549560409	0.060902093
Tmem173|72512	-1.424851982	0.192378275	-2.0373991	0.049953457
Frmd4a|209630	-1.424961015	0.104482816	-1.48156468	0.031634099

Jmjd1c|108829	-1.425125432	0.191959107	-1.084263209	0.408256126
Cc2d2a|231214	-1.425136202	0.15692297	-2.059627633	0.007487411
Abhd2|54608	-1.425422497	0.223829466	-1.17300254	0.322473431
Glt8d1|76485	-1.425573857	0.135022333	-1.425573857	0.135022333
Srp54c|100101806	-1.42558307	0.164639066	-1.191182978	0.292467213
Cpne1|266692	-1.425583461	0.132024801	-1.425583461	0.132024801
Angel1|68737	-1.425679882	0.071166646	-1.157908555	0.232516559
Nubpl|76826	-1.425685452	0.04027966	-1.790737659	0.000776513
1110032A03Rik|68721	-1.425798775	0.006397287	-1.355342513	0.013518839
Treml4|224840	-1.426032852	0.192627574	-1.20016334	0.335077934
Eif4e3|66892	-1.426307757	0.081195062	-1.23829695	0.166862079
Tmem165|21982	-1.42640871	0.154485948	1.028910789	0.452743269
Smarcc2|68094	-1.426430091	0.019479208	-1.416119635	0.005151076
Tnrc6b|213988	-1.426613682	0.220190993	-1.121471088	0.340053522
Nacc2|67991	-1.426634498	0.068137009	-1.401498044	0.037987534
Plekhm3|241075	-1.426983731	0.287841483	1.117819616	0.428080422
Dpp8|74388	-1.426996464	0.145314904	-1.1215145	0.322437951
Prepl|213760	-1.427051362	0.020543193	-1.709882238	0.000744308
Slc35a5|74102	-1.427095133	0.151690778	-1.842430864	0.050320354
Fam82b|66302	-1.427140498	0.032681686	-1.563866856	0.004628997
Slc16a12|240638	-1.427375613	0.133072826	-1.099796861	0.288596126
Kank2|235041	-1.427406508	0.022846274	-1.297794196	0.038838688
Gatad2b|229542	-1.427740646	0.321574037	-1.724175539	0.251994748
Inpp5e|64436	-1.42791305	0.108000739	-1.42791305	0.108000739
Ceacam1|26365	-1.428038119	0.081338069	-1.447033455	0.05611244
Letm1|56384	-1.428525892	0.008441055	-1.428525892	0.008441055
Ptafr|19204	-1.428571429	0.37128647	1	#DIV/0!
Gm13718|100270744	-1.428571429	0.37128647	1	#DIV/0!
Ptprt|19281	-1.428571429	0.37128647	1	#DIV/0!
Rab27b|80718	-1.428739018	0.35290708	-2.8202	0.173296754
Prr14|233895	-1.429098272	0.00263999	-1.429098272	0.00263999
Asap3|230837	-1.429129028	0.06895035	-1.573594391	0.022681265
Gm12824|666048	-1.429236008	0.295131839	-3.010586893	0.023511985
Gm16039|100036521	-1.42937124	0.062458585	-1.828414896	0.005296941
Tatdn3|68972	-1.429525125	0.253048038	-2.224778781	0.029464894
Gm10069|791299	-1.430019884	0.020865976	-1.442239707	0.003000908
Naip5|17951	-1.430040294	0.209821769	-1.096069766	0.41360369
Slc25a10|27376	-1.430064353	0.000897871	-1.484733215	0.000819037
Arhgap39|223666	-1.43017911	0.100458316	-1.462476779	0.026017378
Smad6|17130	-1.43035428	0.064005722	-1.743780253	0.008084606
Ftsjd2|74157	-1.430400314	0.043262408	-1.306731758	0.088797628
Chac1|69065	-1.430776931	0.264342869	-1.236319846	0.318474282
F8a|14070	-1.43082557	0.051208636	-1.547186312	0.021209963
Adnp|11538	-1.430953605	0.222669633	-1.430953605	0.222669633
Nckap1l|105855	-1.431470435	0.129230395	-1.236306557	0.232018151
Batf2|74481	-1.431884415	0.116205616	-2.00077619	0.011510981
Mgst3|66447	-1.432291424	0.160981507	-1.972719678	0.03528883
2310021P13Rik|268721	-1.432445271	0.002107989	-1.579474223	0.000142126
Acat3|224530	-1.432633631	0.00237515	-1.517243923	0.001209515
Klhl18|270201	-1.432877598	0.031568385	-1.561407948	0.018322919
4833418N02Rik|74597	-1.43293744	0.193115615	-1.43293744	0.193115615
Plce1|74055	-1.433401935	0.158674354	-1.433401935	0.158674354
Rapgef5|217944	-1.43353	0.33978158	-4.56078	0.070574133

Klhdc9|68874	-1.433694028	0.10441395	-1.712016054	0.042578971
Wasl|73178	-1.433696297	0.1678347	-1.039843017	0.418214104
Bcl6b|12029	-1.433870023	0.217401948	-2.355590157	0.034728497
Prrc2c|226562	-1.433922684	0.200283181	1.000596096	0.499112145
Tmem8|60455	-1.433962247	0.007204721	-1.433962247	0.007204721
Atxn1|20238	-1.434084123	0.25565431	-1.434084123	0.25565431
Svil|225115	-1.434192839	0.128494819	-1.378872437	0.094839796
1700008J07Rik|629159	-1.434499281	0.056783684	-1.531676405	0.033507039
Nnt|18115	-1.434963029	0.07655749	-1.296083202	0.141876348
Samd9l|209086	-1.43502139	0.129558858	-1.200385188	0.248798071
Cspp1|211660	-1.435266156	0.193697118	-1.042325139	0.44684692
Pilra|231805	-1.435296594	0.150432081	-1.000724093	0.498626105
Zfpm1|22761	-1.435467476	0.014022953	-1.306558597	0.027341203
Gtf2h2|23894	-1.435749027	0.150103042	-1.400378688	0.077321888
Ggta1|14594	-1.436589905	0.229300142	-1.204745698	0.344068972
Bclaf1|72567	-1.436595338	0.041989044	-1.436595338	0.041989044
2610044O15Rik|72139	-1.436771961	0.131408998	-2.069521973	0.001229066
Afmid|71562	-1.436836328	0.002888371	-1.514338797	0.000526542
Pknox2|208076	-1.437100977	0.274996054	1.012805512	0.49185709
Dlg4|13385	-1.437366772	0.219114039	-1.768716624	0.082271489
Tpp2|22019	-1.437374743	0.065448487	-1.117217406	0.251977695
Cnpy3|72029	-1.437550763	0.031845571	-1.278474572	0.049477538
Adamts5|23794	-1.437606185	0.15337067	-1.209002447	0.293637875
1700052K11Rik|73431	-1.437886038	0.151955185	-1.439268133	0.037800324
Nrxn1|18189	-1.437963687	0.088563516	-1.219578275	0.182330686
Fam110c|104943	-1.438044073	0.302112836	-2.713213277	0.093707629
E130303B06Rik|102124	-1.438264928	0.205143898	-1.438264928	0.205143898
Med14|26896	-1.438311834	0.055906661	-1.796364739	0.003022855
A930018P22Rik|68243	-1.438410057	0.258104673	-1.529102287	0.192900919
Papss2|23972	-1.438470618	0.187190108	-1.364395776	0.178534543
Zranb2|53861	-1.439472292	0.14690154	1.001705649	0.496534216
Xdh|22436	-1.43958225	0.006665273	-1.43958225	0.006665273
4933421O10Rik|71073	-1.439754416	0.238233041	-1.999076555	0.029512668
Ankrd1|107765	-1.440114286	0.368944409	1	#DIV/0!
Ndrg4|234593	-1.440114286	0.368944409	1	#DIV/0!
Nfkb1|18033	-1.440159594	0.04175386	-1.748174371	0.002287905
Lum|17022	-1.440195785	0.105733096	-1.94256971	0.006463193
Qrsl1|76563	-1.440338303	0.157025025	-1.635736253	0.117501577
Gm4532|100043580	-1.440952732	0.295921904	-1.440952732	0.295921904
Dusp7|235584	-1.441292973	0.096967517	-1.717035527	0.038717566
Cep350|74081	-1.441375078	0.112705135	-1.16935236	0.238713921
Card10|105844	-1.442082823	0.008669328	-1.473710305	0.00077232
Cenpq|83815	-1.442125418	0.273118558	-1.151062214	0.418507868
Zscan21|22697	-1.442159609	0.075594472	-1.934268631	0.003592951
Polh|80905	-1.442373372	0.198265328	-1.335404223	0.153686989
D330041H03Rik|654822	-1.442927896	0.090425467	-1.346051559	0.120361229
Isg15|100038882	-1.443049456	0.112194827	-1.237474831	0.226156539
Atg14|100504663	-1.44327192	0.051782966	-1.44327192	0.051782966
Dtwd1|69185	-1.44371834	0.224056428	-1.987550202	0.10942287
2610002M06Rik|67028	-1.443858199	0.066450953	-1.301079881	0.127102899
Hrg|94175	-1.44386135	0.017875484	-1.580211678	0.000997944
Otud3|73162	-1.444217656	0.065771805	-1.444217656	0.065771805
41153|54204	-1.444745095	0.08737516	-1.080274445	0.333757392

Slc6a13|14412	-1.445219272	0.001539039	-1.432590358	0.000580814
Fam149b|105428	-1.445219309	0.002008277	-1.344325885	0.002957975
Acad8|66948	-1.445266257	0.002743567	-1.445266257	0.002743567
Rabl5|67286	-1.44562565	0.181390292	-2.084650138	0.042919347
Lmnb2|16907	-1.445638273	0.015518036	-1.206059058	0.035863733
Bcl6|12053	-1.445665752	0.228533321	-1.000862447	0.499173167
Tbc1d9b|76795	-1.446317836	0.00224171	-1.502297333	0.001196548
Limd2|67803	-1.446484155	0.025144624	-1.322130484	0.048693433
Acy3|71670	-1.446490956	0.003928957	-1.344399185	0.003169794
Arid3a|13496	-1.446698534	0.154336409	-1.449293921	0.05132666
Rcn2|26611	-1.44690301	0.165891244	-1.350918607	0.182424991
Card6|239319	-1.447670088	0.043039876	-1.154706439	0.157452714
Tnfaip3|21929	-1.447793249	0.095802329	-1.426127191	0.077460866
Gm4788|214403	-1.448026671	0.124033956	-1.782320354	0.049906014
Otc|18416	-1.44829233	0.000216898	-1.38218248	0.000382114
Thtpa|105663	-1.448354057	0.000355908	-1.435164813	0.000025943
Herc2|15204	-1.448483846	0.073240502	-1.448483846	0.073240502
Erbb2|13866	-1.44879728	0.185564848	-2.571790198	0.003788873
Serpina12|68054	-1.449652661	0.196103798	-1.902643787	0.096852251
Sipa1l2|244668	-1.449886089	0.202379935	-1.43872661	0.228970451
Zbtb48|100090	-1.449917269	0.020959843	-1.567202218	0.004270266
4632411B12Rik|226976	-1.450070409	0.006257024	-1.528277058	0.004914297
Chst12|59031	-1.450153009	0.166188199	-1.401118061	0.141003461
Zfp940|233057	-1.450317158	0.259570295	-1.450317158	0.259570295
Zbtb45|232879	-1.450611916	0.045753385	-1.588161541	0.012565331
Myo7a|17921	-1.450648088	0.071675326	-1.266973242	0.146581074
Traf7|224619	-1.450657283	0.022391662	-1.316980626	0.038890241
Zswim6|67263	-1.450718447	0.22498762	-1.066071587	0.392916051
Adam9|11502	-1.451237307	0.165441119	-1.128068947	0.354575388
Porcn|53627	-1.451716888	0.032809502	-1.366590074	0.038706097
Rere|68703	-1.451962512	0.162521852	1.015465975	0.466818736
Chchd6|66098	-1.451984092	0.026720656	-1.363057088	0.055944953
Tgm1|21816	-1.452228062	0.039761528	-1.266617979	0.07890805
Fryl|72313	-1.452439276	0.081674063	-1.222833287	0.163182793
Slc22a27|171405	-1.45254537	0.158401415	-1.282248059	0.167187685
Pira2|18725	-1.452587939	0.2160171	-1.452587939	0.2160171
Tmem170|66817	-1.452660466	0.181219587	-1.141008438	0.364777232
Cables1|63955	-1.452882931	0.208022344	-2.066353988	0.061560444
Epor|13857	-1.453057542	0.114129757	-1.79155886	0.041640091
Asb7|117589	-1.453437974	0.18125177	-1.420528884	0.116920138
Fdft1|14137	-1.454349738	0.081446632	-1.626464326	0.053203518
Tiparp|99929	-1.4544265	0.127158018	-1.154123812	0.27615525
Kif1b|16561	-1.454455719	0.21914645	-1.107657161	0.354787948
Rdh10|98711	-1.45480647	0.052358809	-1.418951672	0.042110308
Akr1b10|67861	-1.455407743	0.23714079	-1.455407743	0.23714079
Map3k13|71751	-1.455572987	0.303955644	1.02207559	0.488357997
Stard3|59045	-1.455810174	0.004423458	-1.48695465	0.005310995
Pinx1|72400	-1.456156729	0.100498243	-1.347084562	0.160572861
AI464131|329828	-1.456197715	0.000354269	-1.517761033	5.56774E-05
Onecut1|15379	-1.456309479	0.199554082	-1.305695676	0.216284419
Lass4|67260	-1.456340274	0.166276885	-1.489023237	0.154820476
Coro7|78885	-1.456403667	0.00143299	-1.530916086	0.000225746
Npcd|504193	-1.456781695	0.212782511	-1.089391799	0.423871745

Mipep|70478	-1.457238227	0.013024255	-1.457238227	0.013024255
Ranbp10|74334	-1.457291237	0.02806625	-1.288577731	0.035860758
Zfyve16|218441	-1.457831262	0.177892799	-1.618724076	0.151968462
B3galt1|26877	-1.458052181	0.119322267	-1.214232911	0.247359367
8430429K09Rik|71523	-1.458476633	0.220877809	-1.102054753	0.420646142
Anks4b|72074	-1.458510839	0.009353491	-1.721215273	0.000200864
Hlcs|110948	-1.458678341	0.100373366	-1.771235935	0.037144593
5730494M16Rik|66648	-1.458811607	0.039566406	-1.134362797	0.088261593
Stx11|74732	-1.45882769	0.31429881	-1.72578057	0.278974436
Pms1|227099	-1.458864122	0.253461464	-1.00889755	0.493651787
Stra13|20892	-1.459123974	0.037410448	-1.340915904	0.004354522
Ttll5|320244	-1.459391788	0.012345278	-1.503594787	0.000856636
Abl2|11352	-1.459529232	0.087389052	-1.723881169	0.03716573
Taco1|70207	-1.459625924	0.002204961	-1.633155076	0.000189098
Gpr133|243277	-1.459629636	0.259124954	-3.822654545	0.005502342
Nacad|192950	-1.459718661	0.293278866	-2.532914141	0.141518419
Acat2|110460	-1.460919167	0.028250156	-1.460919167	0.028250156
Cyp46a1|13116	-1.46175316	0.037700166	-1.30246994	0.071746003
Zcchc6|214290	-1.461761982	0.104833009	-1.220448093	0.219017652
Prim2|19076	-1.461975458	0.145347616	-1.802663406	0.012520431
Per2|18627	-1.462139737	0.167824301	-1.308257241	0.249080515
Clic5|224796	-1.462198652	0.325661435	1.778618873	0.184006478
A430107O13Rik|214642	-1.462227274	0.253060209	1.109126815	0.407007099
Gyltl1b|228366	-1.462721788	0.211169425	-1.736549835	0.099493143
Gm13375|433408	-1.463042255	0.06766784	-1.463042255	0.06766784
Ntn4|57764	-1.463084175	0.2009768	-2.005515889	0.089251163
1700106N22Rik|73582	-1.46335636	0.185482229	-1.539247025	0.097576844
1600012H06Rik|67912	-1.463452866	0.008193733	-1.344420529	0.009137575
Slc35b4|58246	-1.463512724	0.006143966	-1.393133594	0.004040276
Aqp3|11828	-1.464023683	0.356831873	1.264185714	0.40907038
Odf3b|70113	-1.464068601	0.005975765	-1.516139184	0.000243142
Map4k3|225028	-1.464090262	0.100784841	-1.297421725	0.193376277
Kifc3|16582	-1.464330134	0.015614575	-1.735026644	0.000526762
Cyp2d37-ps|627860	-1.464619024	0.022621782	-1.553333716	0.011450394
Ces1f|234564	-1.464710522	0.022993932	-1.466663316	0.002710341
Polk|27015	-1.465002439	0.202625066	-2.71922527	0.005834538
Tssc1|380752	-1.465195766	0.051058323	-1.465195766	0.051058323
Iqsec1|232227	-1.465263481	0.025049502	-1.388055254	0.004657442
Slc9a9|331004	-1.46530725	0.155582201	-1.721040552	0.072867354
Pcnxl3|104401	-1.465662292	0.002371993	-1.562114507	0.000107965
Zeb2|24136	-1.465756	0.184939337	-1.654448888	0.152770469
Ttc19|72795	-1.466297244	0.003805498	-1.391358166	0.008989329
5830418K08Rik|319675	-1.466339652	0.095754369	-1.466339652	0.095754369
Klhl15|236904	-1.46642219	0.270471222	1.048505508	0.469355521
Dpy19l3|233115	-1.466674886	0.159710116	-1.750989158	0.039450828
Art3|109979	-1.466734238	0.26315125	-2.342264667	0.12014837
Ccdc84|382073	-1.466804141	0.02375946	-1.786577206	0.000672554
2310067B10Rik|71947	-1.466876921	0.015241026	-1.251067579	0.052013647
Slamf6|30925	-1.467018339	0.281672	-1.467018339	0.281672
Atp10d|231287	-1.467046419	0.142613613	-1.232010951	0.276412718
Shq1|72171	-1.467486765	0.071138529	-1.298569027	0.144798725
Ada|11486	-1.467979167	0.024643797	-1.378829386	0.049985481
Sult2a2|100043194	-1.468064937	0.120084449	-1.542883851	0.091169911

Htt|15194	-1.468091425	0.027422282	-1.439289734	0.003718956
Cyp2d26|76279	-1.468944096	0.018656262	-1.686938189	0.001485459
Ppp6r2|71474	-1.46991178	0.005789731	-1.603227482	0.001764872
Glmn|170823	-1.469923258	0.222558473	-2.707837662	0.026031116
Tmtc4|70551	-1.469938002	0.287936823	-1.469938002	0.287936823
Numb|18222	-1.470523844	0.169972799	-1.156455507	0.347316326
Azin1|54375	-1.471245312	0.138020495	-1.510138105	0.085835296
Trim21|20821	-1.471398993	0.122873071	-1.225487429	0.25175912
Ralbp1|19765	-1.471668753	0.036813468	-1.723171326	0.007345556
Aqp4|11829	-1.471984283	0.29538682	-2.170942675	0.173435293
Rnf123|84585	-1.472081369	0.001473667	-1.338806035	0.000421372
Slc38a3|76257	-1.472268026	0.000519579	-1.427117294	0.001456888
Phka2|110094	-1.472378766	0.008929548	-1.508265598	0.000298625
Sdpr|20324	-1.472388896	0.03758829	-1.37819866	0.000240519
Heca|380629	-1.472654899	0.251976168	-1.284468772	0.312401878
Grik5|14809	-1.472791787	0.003419432	-1.332682706	0.00270429
Abca1|11303	-1.472834969	0.05648616	-1.35478709	0.022505062
H2-T24|15042	-1.472878227	0.246889298	-1.037245264	0.474057162
Ccdc43|52715	-1.47315803	0.106136284	-1.308573748	0.184372527
Cxcl12|20315	-1.473161508	0.029017723	-1.473161508	0.029017723
Acp6|66659	-1.473283456	0.000705058	-1.371292614	0.000670214
Tubg2|103768	-1.473698636	0.307694868	-1.473698636	0.307694868
Recql|19691	-1.474117261	0.216533265	-1.640816781	0.136128918
Rab11fip4|268451	-1.474215435	0.218307709	1.052676819	0.432842182
Wnk1|232341	-1.474328901	0.096013152	-1.332732589	0.063320271
Zbtb12|193736	-1.474451511	0.073281605	-1.474451511	0.073281605
Synj2|20975	-1.474748755	0.092840159	-1.474748755	0.092840159
Dmpk|13400	-1.475538908	0.005688678	-1.711187398	0.000233829
Fam125b|72543	-1.476044049	0.171810232	-1.662695703	0.044910888
Grid1|14803	-1.476059925	0.119469193	-1.476059925	0.119469193
Gstm2|14863	-1.476087137	0.052605287	-1.350590199	0.021303141
Arhgap21|71435	-1.476172754	0.127622251	-1.018486348	0.456899951
Cnpy4|66455	-1.476269268	0.117102037	-1.794883861	0.054135446
Smyd4|319822	-1.476955147	0.026664031	-1.476955147	0.026664031
BC068281|238037	-1.47815767	0.018270029	-1.578563292	0.006762852
2610306M01Rik|67170	-1.478337524	0.106937274	-1.478337524	0.106937274
Gbp9|236573	-1.478471734	0.153772574	-1.240380228	0.267347524
Thsd4|207596	-1.478611787	0.188903348	-1.168695951	0.361065484
Slfn1|20555	-1.478770805	0.190930906	-2.687240445	0.006001337
Tjp1|21872	-1.478786278	0.200983086	-1.154885254	0.333039919
Ppcdc|66812	-1.478802537	0.012586383	-1.401355736	0.027526211
Naa38|76522	-1.47919432	0.188400201	-1.769807268	0.069477116
Fhdc1|229474	-1.479478122	0.250216199	1.014209271	0.489677372
Sec16a|227648	-1.479495151	0.024468799	-1.364774341	0.051240227
Yeats2|208146	-1.479495605	0.126304421	-1.757580452	0.072693763
Zfp318|57908	-1.479553577	0.083974176	-1.804885345	0.026309126
Frs3|107971	-1.479710277	0.099136148	-1.478912103	0.038983274
Rbm12|75710	-1.479748573	0.241869957	1.103090603	0.400756507
Sec14l2|67815	-1.479966582	0.002121778	-1.375641514	0.000935836
Rbbp9|26450	-1.480126635	0.045040901	-1.297004954	0.092352212
Dalrd3|67789	-1.480258848	0.000365913	-1.480258848	0.000365913
Nckap1|50884	-1.480413395	0.214149137	-1.107890595	0.416031159
Klhdc8a|213417	-1.480725605	0.091430347	-1.336510164	0.158333283

3110057O12Rik|269423	-1.480844637	0.121362884	-1.360414587	0.185586248
Atp6v0a2|21871	-1.481272724	0.156706988	-1.383552166	0.148272044
Ust|338362	-1.48129156	0.270775399	-3.284075758	0.055632218
Foxa1|15375	-1.481332116	0.126703371	-2.096283027	0.013971042
Plcb3|18797	-1.481478871	0.001267219	-1.306944102	0.000694581
Dhdh|71755	-1.481960355	0.107974843	-1.463900777	0.032744862
Pet112l|229487	-1.482412146	0.006429373	-1.356087794	0.004620046
Sec14l4|103655	-1.482555457	0.003450617	-1.409610247	0.007129477
Cxcl10|15945	-1.482769011	0.215876034	-1.658721777	0.064867621
Slco3a1|108116	-1.482875621	0.037492958	-1.315680429	0.077334148
Trappc10|216131	-1.483748626	0.048921064	-1.331756774	0.100037651
Asah2|54447	-1.483823637	0.11985984	-1.873122394	0.04378966
Atp13a3|224088	-1.484693592	0.131542272	-1.052082502	0.405083768
Spg11|214585	-1.485168761	0.106309842	-1.691011461	0.072229072
Rnf43|207742	-1.485191284	0.047448308	-1.615586645	0.018764037
Strn|268980	-1.485681471	0.300017266	1.289727604	0.359609713
Macrod1|107227	-1.485996662	0.019867334	-1.851471571	0.00010547
Zkscan1|74570	-1.486312984	0.047812861	-1.486312984	0.047812861
Tnfrsf19|29820	-1.486674084	0.079983701	-1.281185064	0.164155923
Xylb|102448	-1.486843642	0.000970242	-1.48938423	0.000132647
Tmem25|71687	-1.486846333	0.057928967	-1.301081012	0.083582828
Hrsp12|15473	-1.487071228	0.00093288	-1.487071228	0.00093288
Zfp1|22640	-1.48724769	0.009934578	-1.508692908	0.00293963
Dnahc5|110082	-1.487269513	0.331084142	-5.916333333	0.093233821
Rora|19883	-1.487760538	0.2767688	-1.036536884	0.477174813
2310044G17Rik|217732	-1.487763119	0.029471283	-1.207305157	0.105620549
Actr3b|242894	-1.487944268	0.307832802	-1.29416451	0.382135102
Qtrtd1|106248	-1.488135666	0.246705379	1.056446913	0.45568495
A430084P05Rik|327957	-1.4883822	0.20201189	-2.02464181	0.100561805
Ghr|14600	-1.48875941	0.016153418	-1.488375312	0.00043116
Hspb6|243912	-1.488852311	0.007409224	-1.724098884	0.000485027
Rnf111|93836	-1.48923041	0.187502816	-1.444516733	0.191782791
2310009B15Rik|69549	-1.489502684	0.105330594	-1.489502684	0.105330594
Gbas|14467	-1.489646978	0.021615464	-1.817966783	0.000600345
9930021D14Rik|319259	-1.4899	0.170446566	1	#DIV/0!
Pip4k2b|108083	-1.490129689	0.009184539	-1.478757148	0.001029317
Col12a1|12816	-1.490684462	0.255553357	-1.490684462	0.255553357
Ccl4|20303	-1.491799871	0.310527873	-1.288457844	0.384067592
Itgam|16409	-1.491867768	0.128262088	-2.201483243	0.001476175
C4b|12268	-1.491996836	0.002626049	-1.579457601	0.000705507
Echdc3|67856	-1.492159849	0.011161289	-1.758971308	0.000136468
Ccnyl1|227210	-1.492442444	0.095029546	-1.569165089	0.050522227
Gltscr1|243842	-1.492479016	0.140779083	-1.434829087	0.148049679
Paqr9|75552	-1.492858769	0.032950463	-1.323561481	0.067970841
Zfp955b|100043468	-1.492925105	0.090864662	-1.447061648	0.13259446
Cyp2u1|71519	-1.49336458	0.048463112	-1.825927933	0.006582828
Zfp341|228807	-1.493466407	0.038468802	-1.695368647	0.016465867
D630037F22Rik|544696	-1.493516348	0.127643889	-1.277861764	0.243924402
D3Ertd751e|73852	-1.493540587	0.084337512	-1.799421629	0.032594958
Pbld2|67307	-1.493708237	0.004489929	-1.705321746	8.49155E-05
Laptm4b|114128	-1.493845885	0.01582581	-1.607663945	0.00395722
Ces2g|72361	-1.493901673	0.003166108	-1.647229375	0.000449878
Ankrd2|56642	-1.494155052	0.22024145	-1.494155052	0.22024145

Ptcd1|71799	-1.494598587	0.000655038	-1.562177439	0.000149886
Atxn7l1|380753	-1.495051263	0.047666674	-1.171650097	0.120857842
Nlrp1a|195046	-1.495197775	0.185677045	-1.186119908	0.352075572
Usp39|28035	-1.495235738	0.00761252	-1.601754626	0.001277926
Ano8|382014	-1.495802614	0.050902947	-1.358881265	0.098210184
Sctr|319229	-1.495904722	0.27732681	-2.575533281	0.131921431
C630043F03Rik|68285	-1.496052595	0.034004855	-1.341497438	0.070450263
Pls3|102866	-1.496138849	0.063028492	-1.262225485	0.114077317
Ednrb|13618	-1.496638993	0.154222065	-1.598382651	0.043135543
Glb1l|74577	-1.49765386	0.114330459	-1.020594564	0.449986599
Fzd4|14366	-1.497856099	0.116581304	-1.497856099	0.116581304
Mup17|100039206	-1.497870127	0.17797592	-1.497870127	0.17797592
Stom|13830	-1.498005728	0.02386786	-1.498005728	0.02386786
Fmr1|14265	-1.498087327	0.231493937	-1.164200793	0.368559082
Mettl21d|207965	-1.498136764	0.081150829	-1.498136764	0.081150829
Tipin|66131	-1.498335093	0.116879937	-1.395737228	0.185156043
Abcd2|26874	-1.498805481	0.02540013	-1.473982162	0.014367842
Muted|17828	-1.498846175	0.036042742	-1.498846175	0.036042742
Slc2a9|117591	-1.499602531	0.014083564	-1.499602531	0.014083564
Purb|19291	-1.499885699	0.14436561	-1.499885699	0.14436561
Asph|65973	-1.500194132	0.053553301	-1.32134992	0.108309796
Abat|268860	-1.500454026	0.117310147	-1.05161417	0.409955809
Alg6|320438	-1.501084545	0.222711956	-1.257006204	0.327331301
Dcdc2a|195208	-1.501338159	0.134333505	-1.214377311	0.278311413
Sgcb|24051	-1.501567139	0.191299579	-2.64465281	0.01343738
Armc7|276905	-1.502012397	0.024614331	-1.502012397	0.024614331
Bmi1|12151	-1.502162921	0.151410065	-1.145800653	0.332392828
Dars2|226539	-1.502668867	0.051845038	-1.634413056	0.007550642
Bex1|19716	-1.502869103	0.194106566	-1.502869103	0.194106566
Dennd1a|227801	-1.503454357	0.001648407	-1.69902448	2.57662E-05
Il10ra|16154	-1.503827978	0.11531653	-1.969232712	0.031628277
Trpv2|22368	-1.504237893	0.150134248	-1.666499341	0.060709199
Rap2b|74012	-1.504243467	0.071909583	-1.437509614	0.072077624
Zfp862|58894	-1.504567657	0.137137271	-1.523761927	0.109981107
Extl2|58193	-1.504776557	0.102285621	-1.868113815	0.038579031
Folr2|14276	-1.506401345	0.008472258	-1.280857165	0.020966654
Hdac11|232232	-1.506552524	0.002995598	-1.574204353	0.000205238
Ifi204|15951	-1.506939074	0.080931036	-1.417785605	0.022400437
Xrra1|446101	-1.507369804	0.311946822	-7.38365	0.04272972
Iglon5|210094	-1.507484968	0.109380968	-1.214251917	0.230911359
Fam116b|69440	-1.507533145	0.000739806	-1.507533145	0.000739806
Tube1|71924	-1.508333333	0.349254813	-2.8	0.173296754
Lsp1|16985	-1.508471008	0.121705329	-1.791024904	0.017598794
Serpinb1a|66222	-1.508474339	0.019223356	-1.610125566	0.008231104
Siglecg|243958	-1.508635178	0.253880384	-1.737539682	0.158096304
Dhfr|13361	-1.508660926	0.013656682	-1.508660926	0.013656682
Akr1c19|432720	-1.508695853	0.031276637	-1.755321986	0.000811767
Rnd2|11858	-1.508797324	0.042796524	-1.508797324	0.042796524
Epb4.1l4b|54357	-1.508811348	0.041715667	-1.51407453	0.031371756
P2ry4|57385	-1.50885227	0.215300604	1.227376656	0.239691143
Nphp3|74025	-1.509040762	0.134205862	-1.196308214	0.285174772
Cstf2|108062	-1.509091912	0.035243591	-1.75247578	4.10506E-05
Gpc3|14734	-1.50951857	0.273002689	-1.50951857	0.273002689

Mrpl3|94062	-1.510092432	0.00547365	-1.392538714	0.011324991
Bcar1|12927	-1.510105512	0.006866557	-1.491521531	0.00025248
Pcmtd2|245867	-1.510430901	0.175229159	-1.483691849	0.176804164
Foxn3|71375	-1.510487405	0.152226665	-1.206984257	0.308242133
Pde12|211948	-1.510539164	0.051525854	-2.006570878	0.002558986
Lipt1|623661	-1.510635023	0.047218781	-1.510635023	0.047218781
Pin4|69713	-1.510755134	0.154294329	-1.390510301	0.163820519
Prkar1b|19085	-1.511303092	0.224961389	-1.511303092	0.224961389
Ank3|11735	-1.511727827	0.020822015	-1.610403447	0.009981094
S1pr5|94226	-1.511822398	0.024643093	-1.918999013	0.00051879
Arhgap4|171207	-1.512811499	0.0788684	-2.231098612	0.000913616
Gm8883|667931	-1.513437105	0.070203952	-1.284755374	0.140689411
Elk1|13712	-1.51360668	0.077192836	-1.834280496	0.027937938
Zfp607|545938	-1.514316768	0.208484421	-1.514316768	0.208484421
Ccdc57|71276	-1.514578871	0.099893302	-1.670286811	0.082113403
Lnp|69605	-1.514739211	0.096207927	-1.514739211	0.096207927
Nup88|19069	-1.515592794	0.011244082	-1.429915806	0.000765751
Cit|12704	-1.515719056	0.314578126	-1.430775758	0.37678302
Taf6l|225895	-1.515952379	0.065113613	-1.98626904	0.004416832
Sepsecs|211006	-1.516056307	0.184878177	-2.112407517	0.079947724
Stx3|20908	-1.516136287	0.060377943	-1.157172467	0.221114938
Cd79a|12518	-1.516365423	0.134219118	-1.750236937	0.045774162
Vps13d|230895	-1.516400279	0.149602831	-1.439741963	0.155211556
Zfp493|72958	-1.516463251	0.277624168	-2.782858531	0.107709595
Extl1|56219	-1.516760788	0.147211329	-1.509129036	0.115822404
Cd247|12503	-1.51721331	0.251236866	-1.51721331	0.251236866
Tchh|99681	-1.517224199	0.066374605	-1.122542846	0.258517608
Olfml2b|320078	-1.517478615	0.308724264	-7.433166667	0.042082278
Pgcp|54381	-1.517563405	0.001338026	-1.569652474	0.000992861
Mertk|17289	-1.517877329	0.187631147	1.079007183	0.404026603
Ap3m2|64933	-1.518154241	0.256844682	-2.990696787	0.07655088
Amhr2|110542	-1.518182962	0.327267251	1.784660662	0.257682973
Sp100|20684	-1.51857604	0.002857774	-1.750535948	1.89256E-05
Zfp317|244713	-1.518800815	0.123840478	-1.405848325	0.103561095
Ddx11|320209	-1.518908275	0.049818344	-1.370629081	0.101770888
Ly75|17076	-1.51956209	0.246751643	-4.682726317	0.001521082
Apcs|20219	-1.52012661	0.018525843	-1.666033872	0.002203162
Mdm1|17245	-1.520574676	0.294512838	-3.345078327	0.117211989
Tifab|212937	-1.520772321	0.018912848	-1.414957297	0.039975045
Fn1|14268	-1.520923236	0.025879952	-1.383770419	0.054231374
Ggn|243897	-1.521029333	0.072920134	-1.638535756	0.047100323
Cyp2s1|74134	-1.52114526	0.157292601	-1.807174404	0.050826696
Acnat1|230161	-1.5212281	0.02012973	-1.432852203	0.013011333
Proz|66901	-1.521342572	0.030163387	-1.644929118	0.023478746
Aifm3|72168	-1.521511351	0.116055264	-1.521511351	0.116055264
Pex3|56535	-1.521902656	0.05798975	-1.519087918	0.044404097
Atp2b4|381290	-1.522298155	0.367365318	3.53972	0.173296754
Zfp772|232855	-1.52280885	0.25944609	-3.253782729	0.065154741
Ccdc141|545428	-1.52311854	0.223737659	-1.295483838	0.237008367
Syce2|71846	-1.523155238	0.084079441	-1.711349559	0.029428578
Tmem123|71929	-1.523202839	0.083283823	-1.287788006	0.171369264
Trp53i11|277414	-1.523248107	0.010580934	-1.523248107	0.010580934
Il13ra1|16164	-1.523970239	0.134164119	-1.432250229	0.133137108

Gpr124|78560	-1.524887583	0.056914496	-1.654181888	0.029539312
Slc8a1|20541	-1.524944473	0.245305307	-4.699512956	0.001660581
Thrsp|21835	-1.52497231	0.047804397	-1.308985716	0.0976852
Abcg8|67470	-1.525492471	0.005376215	-1.57935835	0.000179347
Mum1|68114	-1.525710681	0.013714333	-1.454782242	0.002707889
Ankdd1b|271144	-1.525786287	0.2290074	-1.047919505	0.465399325
Ncald|52589	-1.525836439	0.00665159	-1.298609668	0.009077553
Cnnm4|94220	-1.526500322	0.040235303	-1.399972	0.078296406
Mup1|17840	-1.526531324	0.253091567	1.105734307	0.424729409
Stat2|20847	-1.526764771	0.006465743	-1.800433948	0.000391474
Acaca|107476	-1.527250832	0.003405626	-1.429814864	0.005268757
Slc12a7|20499	-1.527956739	0.001857037	-1.455828781	0.004651474
BC046331|230967	-1.528309922	0.049330865	-1.676281971	0.037198336
Kifc2|16581	-1.528321833	0.019838868	-1.779453161	0.003390758
AU040320|100317	-1.52882344	0.001749188	-1.609781615	0.000442614
Tbc1d2|381605	-1.529184686	0.146123417	1.019264622	0.471109609
Vmo1|327956	-1.529382637	0.145264406	-1.949712663	0.072618092
Lyplal1|226791	-1.53005767	0.056645677	-1.53005767	0.056645677
Map3k4|26407	-1.530314435	0.009883361	-1.405751439	0.016239913
Ctsw|13041	-1.531321838	0.319318683	-4.970412121	0.06319026
Tmem132e|270893	-1.531531478	0.013556138	-1.659423984	0.002429597
Fgf1|14164	-1.531544573	0.002566067	-1.41832199	0.005034712
Ogg1|18294	-1.532184714	0.04296306	-1.335836458	0.088277846
Ctps2|55936	-1.532364303	0.051907837	-1.389014526	0.105019839
Abcb9|56325	-1.532390563	0.047808718	-1.732254448	0.005725322
Slc35a4|67843	-1.532401427	0.013905211	-1.532401427	0.013905211
Kcp|333088	-1.532556357	0.05606118	-1.433490985	0.096409827
Tmem65|74868	-1.532876631	0.186629758	-1.372437871	0.218920694
Cd160|54215	-1.533091694	0.299879933	-10.91965	0.013608087
Gnal|14680	-1.533633203	0.068688468	-1.653769052	0.043443131
Mex3c|240396	-1.533736793	0.243732193	1.006369106	0.495613547
Wdr13|73447	-1.533840257	0.063090331	-1.711153228	0.001987832
Adh1|11522	-1.533938395	0.019177809	-1.472619625	0.017722598
Fgf11|14166	-1.534145781	0.271652919	-2.662060606	0.134533887
Specc1l|74392	-1.534320346	0.0471975	-1.871310328	0.009451216
Herc6|67138	-1.534988017	0.134470283	-1.348267492	0.187274235
Glis1|230587	-1.535223956	0.210876767	-1.813516454	0.116357841
Bmf|171543	-1.535608951	0.123480661	-1.080000998	0.39929512
E130203B14Rik|320736	-1.535793846	0.206727669	-1.007012387	0.492731216
Dmxl1|240283	-1.53617833	0.230531877	-1.285509496	0.283907371
Intu|380614	-1.536228289	0.222883798	-1.314626311	0.315907644
Ankrd50|99696	-1.536249603	0.110513292	-1.9106809	0.049907153
Irf7|54123	-1.536378216	0.059406537	-1.929056466	0.010470071
Hjurp|381280	-1.536548627	0.004180591	-1.454125073	0.009963771
Dcbld2|73379	-1.536752295	0.21198205	-1.128656882	0.410237719
Rbm12b|72397	-1.537221017	0.190480637	-1.02327861	0.471039394
Spry2|24064	-1.537350854	0.180367613	-1.537350854	0.180367613
Bphl|68021	-1.537432683	0.000245044	-1.493680782	0.000739557
0610010B08Rik|100039060	-1.537479416	0.147803695	-1.537479416	0.147803695
Hnrnpul1|232989	-1.538354991	0.041270362	-1.193317948	0.152360177
Abi1|11308	-1.539367778	0.151950787	1.014260946	0.479572523
Tlr11|239081	-1.539557313	0.362390887	1	#DIV/0!
Tceal5|331532	-1.539557313	0.362390887	1	#DIV/0!

Paqr8|74229	-1.539628856	0.342115672	-2.8	0.173296754
Cxcr7|12778	-1.539919236	0.282745318	-1.689048841	0.291071893
Gpr77|319430	-1.540341574	0.031427692	-1.231331259	0.017331506
Cib2|56506	-1.540426542	0.151613931	-1.28749279	0.256794588
Serpina3c|16625	-1.540912438	0.156788928	-1.289938604	0.261381144
Gypc|71683	-1.541046976	0.015712942	-1.367099222	0.030579951
Nfatc1|18018	-1.542135058	0.019164574	-1.22647523	0.03583598
Rb1|19645	-1.543032526	0.113301307	-2.287092014	0.002459936
F2rl1|14063	-1.543280038	0.224382481	-1.133889134	0.41596626
Il2rb|16185	-1.54359295	0.062978951	-1.601687327	0.024779171
4930572J05Rik|223626	-1.544172249	0.015995753	-1.425681358	0.034554712
Arid5b|71371	-1.54424292	0.159056876	1.037108292	0.441171607
Ankrd11|77087	-1.544279603	0.071039435	-1.586961286	0.025852154
A1cf|69865	-1.545201196	0.114866881	-1.312061395	0.164311015
Usp46|69727	-1.54534025	0.075142281	-1.348870607	0.142669398
Camkk1|55984	-1.545763408	0.315832467	-1.122857175	0.457104283
Colec10|239447	-1.546011466	0.132218884	-1.199282575	0.284564014
Sh3bp1|20401	-1.546061097	0.006527105	-1.48890926	0.015065161
Ace2|70008	-1.546228662	0.166707417	-1.830508982	0.066972281
Sp4|20688	-1.546898416	0.272699172	1.076102787	0.459919448
Btk|12229	-1.546903636	0.302737371	-14.17995	0.015325435
Abi3|66610	-1.546974437	0.039237629	-1.434651207	0.038771179
Syne1|64009	-1.547957115	0.049233429	-1.751866847	0.006610097
Nme4|56520	-1.548095851	0.143639814	-1.860483983	0.024064779
Tceanc|245695	-1.549694262	0.163774668	-1.482507586	0.18404372
Gatm|67092	-1.550151248	0.054521303	-1.986896129	0.006051227
Fbxl2|72179	-1.550530375	0.331854274	1	#DIV/0!
Dagla|269060	-1.550561435	0.082774646	-1.754495919	0.026453473
Chst3|53374	-1.550914141	0.037644271	-1.677830215	0.031418045
Dag1|13138	-1.55113971	0.15022694	-1.982523362	0.080569493
Prkd3|75292	-1.551670904	0.037767787	-1.22086756	0.104870306
Dmrta1|242523	-1.552081021	0.291013488	-3.617771345	0.113887469
Snx16|74718	-1.55208659	0.103273243	-1.55208659	0.103273243
Ccr5|12774	-1.552198458	0.089102087	-1.300542876	0.174614347
Dennd2d|72121	-1.552746625	0.115716178	-1.988226557	0.047585971
Slc10a1|20493	-1.552925337	0.002995821	-1.330018668	0.004158503
Ccdc76|229780	-1.553313607	0.223945921	-1.137163103	0.415313841
Birc3|11796	-1.553383926	0.101073897	-1.553383926	0.101073897
Galnt7|108150	-1.553926582	0.225922661	-3.188621513	0.039634992
Tnfrsf4|22163	-1.554061514	0.262476572	-1.554061514	0.262476572
Ptpn14|19250	-1.55436246	0.149130489	-1.858823969	0.043065603
Ppp1r13b|21981	-1.554976536	0.006562489	-1.420398268	0.013645139
Plekha2|83436	-1.555223688	0.043207639	-1.377268958	0.087283579
Il12rb2|16162	-1.555477331	0.253190296	-3.4218599	0.063814866
Epas1|13819	-1.555639074	0.11208802	-1.417586456	0.09515356
Pcgf1|69837	-1.555702505	0.210870334	-1.775696054	0.139631926
Dtx1|14357	-1.556090388	0.093477639	-2.016836725	0.026639682
Trp53bp1|27223	-1.556273292	0.028607234	-1.359528879	0.058484334
Evi2b|216984	-1.556433618	0.156672324	-1.47891201	0.148792405
Asb10|117590	-1.55650523	0.338984102	-2.8	0.173296754
Eif2ak4|27103	-1.556705952	0.177449468	-1.130054459	0.38310223
Dnahc2|327954	-1.557275672	0.314234734	-6.194816667	0.093788939
Retnla|57262	-1.557275672	0.314234734	-6.194816667	0.093788939

Pm20d1|212933	-1.557305397	0.006025764	-1.501308667	0.013495647
Klhl20|226541	-1.558042137	0.126016381	-2.310722562	0.017741065
Slc22a1|20517	-1.558595984	0.001095457	-1.452766073	0.000533831
Mir425|723864	-1.55896843	0.297835998	-2.283448485	0.141958073
Bcl2l11|12125	-1.559192274	0.048413105	-1.596867965	0.01335335
Fnbp1l|214459	-1.559560132	0.146142457	-1.224699442	0.300870114
Rnase4|58809	-1.560106934	0.027657104	-1.722772698	0.018004709
Ctcfl|664799	-1.560538386	0.198798634	-3.656357477	0.004133968
Prkcdbp|109042	-1.560583447	0.25532231	1.187473196	0.364326203
Ino80|68142	-1.560708842	0.039527286	-1.747208002	0.006334326
Jak3|16453	-1.561009675	0.00556943	-1.436819304	0.00732906
Slc26a10|216441	-1.56190716	0.077445912	-1.467652978	0.121017091
Gclc|14629	-1.561960846	0.151484842	-1.38543239	0.219444859
Lilrb3|18733	-1.562039624	0.064971738	-1.658415695	0.009069945
Alkbh8|67667	-1.562932543	0.248695232	-3.308206487	0.035640116
Cd1d2|12480	-1.563008714	0.242443375	-3.208217213	0.034403315
Krt7|110310	-1.563714783	0.14221993	-1.747006612	0.055263716
Dntt|21673	-1.563822097	0.198843721	-1.066646823	0.441770604
Rab11fip2|74998	-1.563982825	0.126488707	-1.232244404	0.267532215
Cyp2c44|226143	-1.564090586	0.008526893	-1.836446386	3.07219E-06
Plcxd2|433022	-1.56414291	0.194146542	-1.064439044	0.440141993
Saa3|20210	-1.565067281	0.168213539	-2.833616052	0.011648592
Elovl6|170439	-1.565209541	0.046376743	-1.211402148	0.174741134
Reck|53614	-1.565453361	0.203050189	-1.813406579	0.153955159
Pion|212167	-1.565776387	0.016611764	-1.791163955	0.004884137
Rttn|246102	-1.566244922	0.208300982	-2.28030796	0.072602753
Ptpru|19273	-1.566285346	0.16644851	-1.245768609	0.318402683
Tprn|97031	-1.566297379	0.030766071	-1.451223225	0.063761831
Zfp384|269800	-1.56635453	0.061543931	-1.56635453	0.061543931
Ints1|68510	-1.566543591	0.001067908	-1.731142159	0.000104317
Wls|68151	-1.566866546	0.151699333	-1.566866546	0.151699333
Rwdd3|66568	-1.567096365	0.058597882	-1.602897003	0.011611658
Cyth4|72318	-1.567247431	0.152257241	-2.924262337	0.00201497
Slc6a17|229706	-1.567666196	0.311763001	-6.23615	0.093645599
Cilp|214425	-1.567666196	0.311763001	-6.23615	0.093645599
Igj|16069	-1.567666196	0.311763001	-6.23615	0.093645599
Ncor1|20185	-1.567815852	0.062942358	-1.163717895	0.179193152
Nfatc2|18019	-1.568987676	0.287456513	1.238734538	0.392280275
Diap2|54004	-1.569185673	0.194028966	-1.225974571	0.26054405
Ddi2|68817	-1.569845165	0.152969036	-1.569845165	0.152969036
Lcp2|16822	-1.569937325	0.151550102	-2.888316914	0.002883122
Cdca8|52276	-1.570199787	0.247533163	-2.267464698	0.139822829
Usp3|235441	-1.571399183	0.000573822	-1.673211273	0.000310782
4931406C07Rik|70984	-1.57149936	0.023385543	-1.458956176	0.047633177
Sh3bgrl2|212531	-1.572078412	0.112175845	-1.269252286	0.234074021
Galnt4|14426	-1.572161332	0.150102821	-1.199179146	0.317891669
Atoh8|71093	-1.572369502	0.034916158	-1.714631852	0.027693195
Megf9|230316	-1.572435837	0.283106416	1.025747745	0.472973442
Fign|60344	-1.572452599	0.058329299	-1.353616681	0.117598797
Gpr125|70693	-1.572662866	0.000378931	-1.506556793	0.000109425
Gstm5|14866	-1.573417194	0.076883171	-1.887444903	0.037398498
Ccdc151|77609	-1.573592048	0.044248656	-1.788047916	0.0256689
Oas1c|114643	-1.575329309	0.158573102	-1.210390234	0.325467108

Rassf8|71323	-1.575523509	0.224649525	-1.007915804	0.482975275
Ccdc34|68201	-1.575791103	0.163668065	-1.723982197	0.138750165
Nup160|59015	-1.576662975	0.190471665	-3.232031681	0.009217895
Kif21b|16565	-1.576690535	0.081270601	-1.31705013	0.161102552
Ggt6|71522	-1.576786677	0.000573074	-1.576786677	0.000573074
Trim23|81003	-1.576882422	0.267584621	1.090316231	0.452439847
9430008C03Rik|68108	-1.577083277	0.066728194	-1.29168521	0.118536748
Mndal|100040462	-1.577635105	0.019426403	-1.370227576	0.036628176
9130409I23Rik|619326	-1.577715255	0.140743766	-2.197820113	0.049151217
5033411D12Rik|192136	-1.578439521	0.010683043	-1.578439521	0.010683043
Apbb1|11785	-1.578542446	0.09643818	-2.198856559	0.015220117
2210404O07Rik|72273	-1.578640504	0.240504669	-4.107081102	0.028677687
Acvrl1|11482	-1.579701192	0.026089688	-1.23395639	0.071260462
Ola1|67059	-1.579948672	0.011754106	-1.860343488	0.000238838
Fam113a|319513	-1.580073478	0.00019613	-1.580073478	0.00019613
Casp12|12364	-1.580135052	0.273237139	1.02527114	0.487743454
Gm10516|100038712	-1.581886266	0.010852052	-1.393064285	0.018881386
Taf1d|75316	-1.582048877	0.141991553	-2.008958014	0.079546392
40975|57438	-1.582110592	0.234129594	1.012862175	0.490946164
Spred2|114716	-1.582140399	0.233458877	-1.510815282	0.274581911
Trex1|22040	-1.58250949	0.033534937	-1.566935402	0.009339279
Crygn|214301	-1.582712354	0.251887384	1.119528457	0.422542708
1810029B16Rik|66282	-1.582815493	0.038530843	-1.322555508	0.074101905
Camsap3|69697	-1.583209823	0.005532922	-1.567087977	0.000518612
Il27ra|50931	-1.583463064	0.129113721	-1.3220787	0.23266195
Rai14|75646	-1.583648545	0.077624609	-1.328300371	0.159054097
Rapgef6|192786	-1.58388196	0.150558546	-1.58388196	0.150558546
Hmgxb4|70823	-1.584164385	0.186108392	-1.693590956	0.151363868
Pgap2|233575	-1.584262065	0.082007572	-2.235697491	0.004139097
1700028J19Rik|70004	-1.584343213	0.299197086	-2.8	0.173296754
2700029M09Rik|72612	-1.584752165	0.024985367	-2.018152653	0.001507516
Tpcn2|233979	-1.585587808	0.041939432	-1.585587808	0.041939432
Setbp1|240427	-1.585594755	0.277293131	-4.343232323	0.079035869
Alg10b|380959	-1.585639549	0.246129189	1.048000444	0.436850153
9330020H09Rik|100048895	-1.585894853	0.130526848	-1.030491862	0.458912256
Kcns3|238076	-1.585940474	0.353523693	1	#DIV/0!
Zfp935|71508	-1.586437222	0.161089254	-1.290707975	0.298255924
Insig1|231070	-1.58677896	0.070207322	-1.558178959	0.049390727
Zdhhc24|70605	-1.587267951	0.03704918	-1.372648455	0.064465422
Pvrl3|58998	-1.587363094	0.012632247	-1.696692159	0.005143478
4833422F24Rik|74614	-1.587378851	0.160296973	-1.230304356	0.321357002
Xkr8|381560	-1.588057431	0.01804917	-1.261048225	0.027093718
Mid1ip1|68041	-1.588248879	0.079805227	-1.156966176	0.206010451
Slc9a3r1|26941	-1.588398709	0.00095291	-1.499063652	0.002297965
Il33|77125	-1.588916087	0.261312272	-2.838589444	0.129383442
Ctso|229445	-1.589092008	0.003275623	-1.484506229	0.005401749
Hexim2|71059	-1.589731553	0.140672848	-2.455375598	0.025631666
Myoz3|170947	-1.589744687	0.297810565	-2.8202	0.173296754
Phldb2|208177	-1.589927734	0.031288211	-1.382409003	0.051233173
Fam123b|72345	-1.590179181	0.158706029	-2.868501068	0.006925345
Txndc16|70561	-1.591221906	0.143922109	-1.007048633	0.491058321
Scara3|219151	-1.591455113	0.135393467	-1.496103475	0.164141594
BC020402|407824	-1.591730968	0.120606103	-1.573862376	0.144898997

Abi3bp|320712	-1.592097235	0.137861964	-1.592097235	0.137861964
Dnajc16|214063	-1.592286937	0.041684871	-1.96751655	0.008394212
Mblac2|72852	-1.592689311	0.146211849	-1.592689311	0.146211849
Nr3c1|14815	-1.59334996	0.095376901	-1.532377656	0.070463265
Abcd1|11666	-1.593375591	0.000682676	-1.593375591	0.000682676
Loxl3|16950	-1.594047941	0.10109697	-2.225364137	0.019770006
Mll3|231051	-1.594889282	0.21688583	-1.173715417	0.366116169
P2ry13|74191	-1.596058647	0.094637108	-1.302469298	0.196335649
Gnptab|432486	-1.596981551	0.06430132	-1.695889875	0.0431622
Tmem184a|231832	-1.597388062	0.01165997	-1.597388062	0.01165997
Sult5a1|57429	-1.59739801	0.037313021	-1.853070255	0.017076296
Zfp583|213011	-1.597606238	0.296561254	-2.8	0.173296754
Angptl1|72713	-1.597606238	0.296561254	-2.8	0.173296754
Ccdc78|381077	-1.597798717	0.317446086	-2.8202	0.173296754
Ccdc152|100039139	-1.599299906	0.247007752	-2.753551128	0.120180084
Myl6b|216459	-1.599568176	0.198995047	-1.077313758	0.435909646
Asb3|65257	-1.600680045	0.100566965	-1.914208325	0.012363994
Siglec1|20612	-1.601055184	0.186770571	-1.794245103	0.150294882
Sipa1l3|74206	-1.602222035	0.02378125	-1.433781478	0.045166297
Rpgrip1l|244585	-1.602267462	0.256403677	-4.183444187	0.058973421
Smg1|233789	-1.602363637	0.104484263	-1.468566314	0.098274668
Dock2|94176	-1.602983316	0.093567904	-1.950569294	0.0504774
Cdk3-ps|69681	-1.603007712	0.295191603	-2.8202	0.173296754
Gm14308|100043381	-1.604712685	0.199127463	-2.835007566	0.055122727
Sord|20322	-1.604801779	0.005818919	-1.564435148	0.000792504
Fbxo5|67141	-1.605098455	0.229824007	-1.023197358	0.484630668
Bicc1|83675	-1.60532342	0.235710905	-2.068929547	0.183148971
Arid4b|94246	-1.605340717	0.152807412	-1.213869927	0.320646688
Xist|213742	-1.605484827	0.08682832	-1.242973834	0.14850238
Abca2|11305	-1.605550103	0.001991723	-1.477399521	0.000997897
Xlr3a|22445	-1.60577638	0.18241962	-1.60577638	0.18241962
Zfp865|319748	-1.605808057	0.025868078	-2.027117134	0.001320744
4930412C18Rik|320152	-1.605988331	0.1480522	-1.278922803	0.290967519
Cyb5r4|266690	-1.606032761	0.111790391	-1.664424334	0.070263358
Zbtb38|245007	-1.606506029	0.186260142	-1.322969275	0.282680438
Cpt1c|78070	-1.606613599	0.186140156	-1.606613599	0.186140156
Car15|80733	-1.60662548	0.253003047	-2.020938106	0.219451422
Slc28a2|269346	-1.606807507	0.258054881	-4.627698349	0.050034393
Phip|83946	-1.607075249	0.142358957	-1.873021199	0.062413192
Car13|71934	-1.607640514	0.350174696	1	#DIV/0!
Nkd2|72293	-1.607640514	0.350174696	1	#DIV/0!
Zfp7|223669	-1.607716728	0.028615563	-1.470776146	0.004649246
Erbb3|13867	-1.607808997	0.190322354	-1.12423708	0.40485186
Ikbip|67454	-1.607825458	0.102975081	-1.346793396	0.189860869
4933407K13Rik|74396	-1.608110114	0.293443847	-1.454545455	0.37214076
6720401G13Rik|103012	-1.608396755	0.080528125	-1.453199717	0.072791897
Ppp1r9a|243725	-1.608453959	0.235170179	-1.02931305	0.463159745
Cdc40|71713	-1.608686171	0.249954545	-3.398344211	0.051109741
Selplg|20345	-1.608799224	0.040689258	-1.608799224	0.040689258
Cenpc1|12617	-1.608948056	0.177496276	-1.608948056	0.177496276
Gstm6|14867	-1.609527409	0.015350337	-1.486017978	0.004260455
Lect2|16841	-1.609946078	0.011292485	-1.609946078	0.011292485
Syde2|214804	-1.609948149	0.124486078	-2.123796245	0.055342287

Hao1|15112	-1.610301958	0.001729455	-1.654655661	9.40692E-05
Slc2a2|20526	-1.611415528	0.047561519	-1.226845521	0.16636865
Ccnt1|12455	-1.611489724	0.224350974	-1.078904146	0.451018
Pkd1|18763	-1.611926992	0.030291207	-1.611926992	0.030291207
Grin3b|170483	-1.612074938	0.201310347	-1.612074938	0.201310347
Arhgef9|236915	-1.612867491	0.079194972	-1.642375884	0.043377631
Zfp758|224598	-1.613180905	0.182214255	-1.190080651	0.366167061
Myef2|17876	-1.613511034	0.167879211	-2.133325372	0.060550937
B930003M22Rik|100526796	-1.614297864	0.274113798	-6.42165	0.04426109
Pls1|102502	-1.614305727	0.254026732	-2.511332479	0.158381868
Numbl|18223	-1.615375928	0.039611248	-1.234805345	0.147526917
Ly6g6e|70274	-1.616026667	0.285301413	-8.080133333	0.046315002
Shisa5|66940	-1.616223989	8.91539E-06	-1.761838275	1.22053E-07
Znhit3|448850	-1.616533669	0.128844444	-1.656004257	0.072259519
Tusc3|80286	-1.616901573	0.133672777	-2.458370216	0.028668526
Spock2|94214	-1.618231609	0.247870828	-1.637938004	0.215939844
Myrip|245049	-1.619002205	0.31126629	1.823071741	0.250512967
Npr2|230103	-1.619130519	0.003148973	-1.421943694	0.006863266
Cpsf7|269061	-1.619175447	0.047628627	-1.254342679	0.091115778
Ppapdc1b|71910	-1.619298816	0.001842607	-1.378268124	0.001332744
Arsb|11881	-1.619623499	0.001363903	-1.619623499	0.001363903
Scaper|244891	-1.619632501	0.11859522	-2.832202974	0.00359722
Cabp2|29866	-1.620456764	0.272972998	-6.44615	0.04497135
Nudt12|67993	-1.620652802	0.204779744	-2.444157559	0.103671955
Large|16795	-1.621364969	0.09497313	-2.368311853	0.011457857
Larp4b|217980	-1.622030347	0.162117107	-1.281970475	0.190925481
Glcci1|170772	-1.622143198	0.199467428	-1.047433818	0.459494493
A430110N23Rik|269855	-1.622460676	0.09083142	-1.284983229	0.093524385
Ccdc89|70054	-1.62251392	0.2717845	-6.454333333	0.043911777
Cdk5r1|12569	-1.622563333	0.284264933	-8.112816667	0.047356131
Cav2|12390	-1.62319493	0.15215857	-2.209583687	0.074992497
Hhip|15245	-1.623768002	0.134440853	-1.460592413	0.170095797
Il1b|16176	-1.623929196	0.192095453	-1.782149709	0.133382615
Ltb4r1|16995	-1.624538456	0.027808575	-1.271838585	0.096780712
Fkbp1b|14226	-1.624688389	0.271784804	-6.462983333	0.044795474
Nsun3|106338	-1.624832109	0.115680412	-1.624832109	0.115680412
Prickle2|243548	-1.624842868	0.107712667	-2.031931907	0.044971069
Ddah1|69219	-1.625440616	0.013131479	-1.547323704	0.026970996
Mapk12|29857	-1.625834319	0.095305676	-1.625834319	0.095305676
Appbp2|66884	-1.62748941	0.084068519	-1.953092216	0.005921471
Ica1|15893	-1.627911341	0.102823563	-1.362594106	0.205536844
Pacsin1|23969	-1.62808083	0.212708546	-2.908171717	0.075151509
Lrit1|239037	-1.628142449	0.123067643	-2.559747366	0.007642996
Phc3|241915	-1.628509648	0.063399717	-2.074612736	0.018071976
Gng7|14708	-1.628536858	0.109967806	-1.945856217	0.020854713
Lrch3|70144	-1.629054461	0.262860453	1.210611987	0.384563535
Naa30|70646	-1.629522478	0.0594977	-1.424042006	0.074597628
5330417C22Rik|229722	-1.629993496	0.19585666	-1.82274941	0.17333311
Tcf12|21406	-1.630285214	0.102356831	-1.630285214	0.102356831
Sfrs18|66625	-1.630940582	0.117145	-1.182497077	0.233776538
Nrp1|18186	-1.631108769	0.019518308	-1.682042165	0.007617073
Vamp1|22317	-1.631159012	0.072533165	-1.807490381	0.061462579
G6pdx|14381	-1.631228551	0.066506323	-1.631228551	0.066506323

Rgs5|19737	-1.632196258	0.18771404	-1.993502383	0.145855161
Itih4|16427	-1.632375743	0.025295859	-1.44972895	0.04770136
Tarsl2|272396	-1.63267133	0.043386648	-1.781245239	0.020964843
Rrad|56437	-1.632904445	0.269476835	-6.495666667	0.044433779
Clec2h|94071	-1.632904445	0.269476835	-6.495666667	0.044433779
Snord93|100217436	-1.632904445	0.269476835	-6.495666667	0.044433779
Lass1|93898	-1.632904445	0.269476835	-6.495666667	0.044433779
Rtel1|269400	-1.632980793	0.049533021	-1.424600986	0.100871593
Dok3|27261	-1.633353868	0.037445446	-2.063967505	0.005749858
Gpr12|14738	-1.634120753	0.19207759	-1.634120753	0.19207759
Cyp2j9|74519	-1.63424655	0.065615146	-1.135246198	0.247652736
B230217C12Rik|68127	-1.634362759	0.255029243	-13.76916	2.35691E-05
I830012O16Rik|667370	-1.634676243	0.070361761	-1.386187036	0.143936113
Orm3|18407	-1.634963816	0.077880214	-1.636219163	0.023821232
Scnn1a|20276	-1.635003634	0.002323471	-1.736852002	0.002103379
Lrit2|239038	-1.635133867	0.117350692	-2.097900437	0.039394864
Pip4k2a|18718	-1.635214119	0.027472804	-1.793956034	0.007386502
Srrd|70118	-1.635214449	0.037406883	-1.681656109	0.02287575
Adamts10|224697	-1.635535693	0.045347646	-2.032984921	0.000624214
Klf1|16596	-1.635737403	0.008185294	-1.370620793	0.018333029
Lgals4|16855	-1.636113995	0.001853017	-1.503209752	0.00091684
Tbl1x|21372	-1.636134746	0.183354483	-1.767484564	0.083374674
Endod1|71946	-1.636675691	0.081294888	-1.862142448	0.028410415
Mdk|17242	-1.637959816	0.195901697	-2.081950954	0.044897724
Cyb5b|66427	-1.638113553	0.000511412	-1.531381791	0.000232689
Dgkq|110524	-1.638394691	0.072170787	-1.638394691	0.072170787
Prkd2|101540	-1.638398829	0.026367702	-1.291836643	0.062095083
Nfam1|74039	-1.638757528	0.047373772	-1.638757528	0.047373772
Adora2a|11540	-1.638883825	0.063005492	-2.412988768	0.001225
Chchd8|68185	-1.639252525	0.01359365	-1.338164261	0.0427362
Rpap1|68925	-1.639899787	0.02235921	-1.578653155	0.001716884
Faim3|69169	-1.639978841	0.170118073	-1.788640801	0.155634446
Rad21|19357	-1.641253737	0.166556833	-1.086201725	0.400102845
Gli1|14632	-1.641900542	0.329677389	1.621721864	0.33676256
Rasal2|226525	-1.642401147	0.113198512	-1.370979133	0.18136733
Cramp1l|57354	-1.642735043	0.212893245	-1.21077619	0.345315059
Slc39a5|72002	-1.642814631	0.034238881	-1.752130171	0.009431005
Papolg|216578	-1.642904021	0.144825611	-1.256830723	0.299424643
Nfkbie|18037	-1.643262124	0.100627488	-2.124531625	0.007885477
Ankra2|68558	-1.64333539	0.026045703	-1.512260153	0.054684224
Zfp606|67370	-1.643519059	0.075063497	-1.182205812	0.249209277
Leap2|259301	-1.643582931	0.030210708	-1.862290744	0.018268937
Zfp956|101197	-1.643717108	0.066101169	-1.427422856	0.134202797
Tnfsf14|50930	-1.643830208	0.234907367	-1.906558976	0.209895125
Myo1g|246177	-1.643987065	0.069536326	-2.693347977	1.18394E-05
Pcolce2|76477	-1.644198633	0.062303198	-1.945291742	0.010044334
Serpina6|12401	-1.644225267	0.088336707	-2.599971481	0.002468242
Xrcc4|108138	-1.644439638	0.028398744	-1.450624736	0.053819734
Tyk2|54721	-1.645122966	0.012413625	-1.666694069	0.00715312
2700094K13Rik|72657	-1.645622937	0.039741059	-1.598558068	0.013738932
Rdm1|66599	-1.645728289	0.038619136	-1.870257322	0.004659062
Adam19|11492	-1.646225397	0.158558999	-1.903768073	0.098509017
Cep78|208518	-1.646481101	0.092331762	-2.287550754	0.021684298

Cstad|78617	-1.646971955	0.260467465	-1.637078639	0.293631698
Neat1|66961	-1.64762945	0.076020943	-1.182932461	0.264929275
Rundc1|217201	-1.64798201	0.004625089	-1.823874468	0.001257236
Agrn|11603	-1.648871648	0.060439478	-1.369207618	0.116611081
Mast1|56527	-1.64975439	0.284679928	-8.96592	0.022859172
Slc9a6|236794	-1.650093248	0.017680498	-1.613249791	0.004358599
Cd84|12523	-1.650736664	0.067999225	-1.404855536	0.139018086
Gm17296|212728	-1.650874603	0.165083139	-1.945969931	0.123409541
Pml|18854	-1.651119128	4.91955E-05	-1.59584458	9.32477E-05
Zfp174|385674	-1.651421139	0.127429645	-3.123575722	0.002547196
Slc4a9|240215	-1.651864552	0.198770972	-1.014013088	0.486720651
Gckr|231103	-1.652657226	0.001837315	-1.652657226	0.001837315
Naalad2|72560	-1.652993175	0.275238286	-8.096966667	0.046064383
Car14|23831	-1.653632512	0.000139617	-1.751691073	1.99656E-06
Iqgap3|404710	-1.654137984	0.293749357	-6.580133333	0.095073527
Tnfrsf9|21942	-1.654137984	0.293749357	-6.580133333	0.095073527
Gstm3|14864	-1.654318694	0.053617597	-1.345747007	0.079587522
0610040B10Rik|67672	-1.65584106	0.149616652	-1.258466693	0.307267274
Irgm2|54396	-1.656170054	0.017200273	-1.476060579	0.02912842
Ccdc3|74186	-1.656814853	0.062110265	-1.501332878	0.108758185
Snapc3|77634	-1.657461981	0.242022018	1.107576357	0.432071815
Zfp710|209225	-1.657658748	4.62669E-05	-1.522489837	4.69083E-05
Aldoc|11676	-1.657741482	0.113098969	-1.243650871	0.239010043
Atat1|73242	-1.658139728	0.000863425	-1.552860655	0.00088946
Runx3|12399	-1.658583124	0.191866325	-3.726504649	0.020988845
Gm13698|668096	-1.658638303	0.124054589	-2.367267604	0.04191262
Isoc1|66307	-1.659234514	0.08305695	-1.519054972	0.065906178
Tfcp2l1|81879	-1.65941296	0.087169911	-1.428793812	0.148573421
Rac2|19354	-1.659424395	0.002085866	-1.630289375	0.005126785
E330016A19Rik|214763	-1.659842094	0.226658992	-1.659842094	0.226658992
Trip13|69716	-1.660179572	0.292881026	-3.925	0.173296754
Mybl2|17865	-1.660399188	0.184765033	-2.539471071	0.085388228
Cux1|13047	-1.660474775	0.003996866	-1.625634095	0.000424867
Sepn1|74777	-1.660723208	0.060735946	-1.660723208	0.060735946
Rbm33|381626	-1.660784016	0.092332312	-1.254741909	0.165323539
Tecpr1|70381	-1.661784763	0.012481974	-1.479662947	0.016710515
Arhgef6|73341	-1.661972752	0.099661378	-2.483511189	0.015212152
Zfhx2|239102	-1.662099075	0.036654131	-1.279387248	0.08867245
C78339|97863	-1.662211032	0.26013642	-1.134450896	0.422062756
1700025K23Rik|66337	-1.663534103	0.321565824	-2.76078	0.173296754
BC024386|212965	-1.663698165	0.006434047	-1.558879349	0.014853862
Gpr160|71862	-1.663976591	0.195257619	-2.864906452	0.071212126
Plekha6|240753	-1.664117677	0.007608015	-1.664117677	0.007608015
Pfkfb3|170768	-1.66438917	0.169440089	-1.403455999	0.08952357
C1galt1|94192	-1.665388026	0.175061037	-2.116168968	0.050684142
Gemin8|237221	-1.666032837	0.087405362	-1.989941165	0.010527848
Bend4|666938	-1.666318835	0.22124899	1.176242295	0.384301458
Nkx2-1|21869	-1.666337759	0.272375901	-8.162333333	0.046595088
Nr1d2|353187	-1.666344298	0.094260316	-1.235941003	0.150107928
Glg1|20340	-1.666540613	0.022977094	-1.514353905	0.047791085
Tceal6|66104	-1.666666667	0.170446566	1	#DIV/0!
Igf2|16002	-1.667120053	0.115879404	-1.667120053	0.115879404
Frs2|327826	-1.667496281	0.165698577	-1.151543146	0.370371736

Zfp395|380912	-1.668176212	0.005181306	-1.834240315	0.002987509
Gabrq|57249	-1.668642822	0.291292711	-3.925	0.173296754
Mef2a|17258	-1.668728027	0.276959175	1.705693277	0.014134966
Nudt13|67725	-1.670256782	0.004052161	-1.562089295	0.008120882
Mnda|381308	-1.670333466	0.122496434	-1.357129652	0.242305176
Dnajc9|108671	-1.67060973	0.019854035	-1.326979511	0.055362401
Chd3|216848	-1.67281074	0.005453755	-1.530681801	0.008021484
Jam3|83964	-1.672995736	0.20416446	-2.077146768	0.161022001
Gprc5b|64297	-1.673107757	0.190023623	-2.275047768	0.097089671
Cyp4f17|208285	-1.673684276	0.028063236	-1.673684276	0.028063236
Tmem199|195040	-1.673929489	0.03682826	-1.450802673	0.070740456
Akr1c14|105387	-1.674078226	0.069213461	-1.454558066	0.069278023
Rbmx2|209003	-1.6741979	0.15324935	-1.020617533	0.479596899
Zfp518b|100515	-1.674251429	0.166598536	-3.460873102	0.010490817
Rarb|218772	-1.674491474	0.14083798	-1.218209669	0.307236577
Mlxipl|58805	-1.676091496	0.014280816	-1.578401984	0.029512733
Ildr2|100039795	-1.676187126	0.010646664	-1.676187126	0.010646664
Gbp8|76074	-1.676343733	0.063347622	-2.172696156	0.018725395
Treml1|71326	-1.676478453	0.283006472	-2.76078	0.173296754
Serpinb1c|380839	-1.676478453	0.283006472	-2.76078	0.173296754
Ripk3|56532	-1.676569969	0.140365872	-1.264225056	0.294498843
Cxcl14|57266	-1.676778915	0.120502822	-1.395523875	0.230427877
1810064F22Rik|69862	-1.677061188	0.228367159	-1.677061188	0.228367159
Spats2|72572	-1.677587726	0.018600661	-1.817389655	0.007271777
Mir546|723882	-1.67789458	0.263734308	-2.3037	0.15892251
Slc22a23|73102	-1.678293376	0.016534187	-1.341705074	0.035285634
Thbs2|21826	-1.678757227	0.141521236	-1.678757227	0.141521236
Gla|11605	-1.678874319	0.022519256	-1.620296005	0.004328827
Rasal3|320484	-1.679136581	0.077080623	-1.451564148	0.153488784
Zfp160|224585	-1.679227806	0.197937755	-1.171968448	0.396178324
Mfsd6l|215723	-1.679565441	0.341175328	1	#DIV/0!
Pgc|109820	-1.679565441	0.341175328	1	#DIV/0!
Cyp4f14|64385	-1.679746683	0.000239333	-1.78791945	4.10795E-06
Mars2|212679	-1.680412889	0.207595328	-1.074948834	0.449911652
Mdc1|240087	-1.680575348	0.076706127	-1.391301218	0.157248663
Traf1|22029	-1.680821735	0.161901704	-2.225794019	0.077446582
Slc41a2|338365	-1.68084442	0.227724913	1.389051461	0.144313583
Ppp1r3e|105651	-1.680848028	0.007673158	-2.111402436	0.000254409
Emb|13723	-1.681267527	0.255241667	-6.404816667	0.044430303
Lst1|16988	-1.681351162	0.21228564	-2.577069326	0.079542024
Mical1|171580	-1.681464202	0.02414458	-1.681464202	0.02414458
Lyst|17101	-1.682081398	0.119125472	-1.256995996	0.255155356
Mir1949|100316700	-1.682297636	0.077400348	-2.620684414	0.00457064
Ano1|101772	-1.682674715	0.081546374	-1.95494224	0.022586413
Fabp1|14080	-1.682754208	0.046971055	-1.427868151	0.09035385
Ppwd1|238831	-1.68294513	0.07010921	-2.099976177	0.031831111
Hsd3b2|15493	-1.684659721	0.013125423	-1.684659721	0.013125423
Dopey1|320615	-1.685093922	0.061690651	-1.990949993	0.037553166
Ascl3|56787	-1.685686286	0.25407837	-6.42165	0.04426109
Fam55c|385658	-1.686247212	0.246141559	-1.72578057	0.278974436
5730559C18Rik|67313	-1.686965869	0.280519474	-2.8	0.173296754
Mdfic|16543	-1.687604588	0.065749031	-1.37741626	0.127736568
Lrg1|76905	-1.687952922	0.019577966	-1.883261072	0.002364582

6230427J02Rik|68176	-1.688144453	0.183045694	-4.430131319	0.00377609
Ddit4l|73284	-1.689392652	0.083437475	-1.346787572	0.16945781
Pmp22|18858	-1.690271227	0.0597296	-1.952665874	0.002985716
Fastkd3|69577	-1.691882624	0.064012732	-1.691882624	0.064012732
Ankrd24|70615	-1.691942632	0.005034794	-1.691942632	0.005034794
Slc22a13b|109280	-1.692117548	0.253065206	-6.44615	0.04497135
Nrp2|18187	-1.692260986	0.023916083	-2.164632246	0.002071187
4930513N10Rik|319960	-1.692367343	0.279253887	-2.8202	0.173296754
4930432K21Rik|74666	-1.692367343	0.279253887	-2.8202	0.173296754
1700067K01Rik|73453	-1.692815864	0.111088303	-1.692815864	0.111088303
Nxnl1|234404	-1.692857143	0.272492918	-2.8	0.173296754
Adamts3|330119	-1.692857143	0.272492918	-2.8	0.173296754
Spns3|77577	-1.692949721	0.290590697	-4.6202	0.07056113
Gmip|78816	-1.6929673	0.001103473	-1.6929673	0.001103473
1110028C15Rik|68691	-1.693097091	0.111425824	-1.080303666	0.396566232
Arhgef37|328967	-1.694022649	0.12387789	-1.285177103	0.262743865
Gm10638|666945	-1.694116199	0.289329356	-1.217265772	0.429152681
Pcbp3|59093	-1.694420337	0.11601919	-2.776875309	0.01515967
Snord123|66835	-1.694462629	0.197688348	-2.016429422	0.128863339
Cxcl9|17329	-1.694643742	0.004650859	-1.835921561	0.000629346
Chdh|218865	-1.695050447	0.021736002	-1.50442317	0.040781958
Zc3hav1|78781	-1.695146128	0.049980578	-1.866196689	0.013022672
Asns|27053	-1.695201945	0.279868539	-1.717057615	0.248962855
Plek2|27260	-1.695282989	0.209838265	-3.665132774	0.054624765
Efnb3|13643	-1.696536306	0.251912966	-6.462983333	0.044795474
Rps6ka1|20111	-1.696635705	0.056232287	-1.214537324	0.067544819
Ephb6|13848	-1.697853054	0.172488405	-2.268304799	0.094675925
4933411K16Rik|66765	-1.697986844	0.205449342	-2.733259285	0.071305896
Sufu|24069	-1.698496332	0.09656172	-2.266232002	0.039486462
Smarcd3|66993	-1.698605798	0.260803399	1.32887796	0.318962999
Cd200r1|57781	-1.698628571	0.271179325	-2.8202	0.173296754
Pou6f1|19009	-1.699245358	0.043839176	-1.685150973	0.027570063
Cpne3|70568	-1.699575718	0.095252243	-1.61376596	0.054642774
Lime1|72699	-1.699614116	0.003660654	-1.699614116	0.003660654
Fan1|330554	-1.700035036	0.111082196	-1.078668386	0.396820522
Nmt2|18108	-1.700169116	0.12521227	-1.281006811	0.266430708
Il12rb1|16161	-1.700304149	0.142051309	-3.044906276	0.020499896
Slc13a3|114644	-1.701917046	0.023000941	-1.829393675	0.012743083
AB124611|382062	-1.702129165	0.02732242	-1.702129165	0.02732242
Pyroxd1|232491	-1.702593675	0.000477183	-1.499016507	0.001168728
1810011O10Rik|69068	-1.703130867	0.021206549	-1.444358714	0.015145517
Plxdc1|72324	-1.703163912	0.160542737	-1.703163912	0.160542737
Vps13c|320528	-1.703653108	0.058979495	-1.576850163	0.058527471
Ptk7|71461	-1.704050886	0.218710349	1.026920947	0.480583447
Gstt3|103140	-1.704784252	0.002469838	-1.985792974	0.000162619
Pcdh7|54216	-1.705115697	0.249673789	-6.495666667	0.044433779
Acsm1|117147	-1.705233872	0.000197845	-1.628679295	0.000567933
Pgbd1|319207	-1.705639273	0.243342833	-2.629015854	0.067030285
Arhgap30|226652	-1.705676215	0.048024585	-1.705676215	0.048024585
Zfp791|244556	-1.705759401	0.263909036	-7.88976	0.029636667
Rpusd4|71989	-1.70587985	0.008618859	-1.818323758	0.004229495
9930012K11Rik|268759	-1.706003278	0.242290604	-9.21165	0.014618853
Ahrr|11624	-1.707028571	0.27016815	-2.8	0.173296754

Hhipl1|214305	-1.707028571	0.27016815	-2.8	0.173296754
Ttll8|239591	-1.707028571	0.27016815	-2.8	0.173296754
A930004D18Rik|77940	-1.708568057	0.187984678	-3.341597838	0.049363341
Xpo4|57258	-1.708593	0.29809535	-4.72156	0.173296754
Fhod3|225288	-1.70910297	0.125287711	-1.70910297	0.125287711
Gm3893|100042539	-1.709403299	0.070261985	-2.930095829	0.000351319
Coro2a|107684	-1.70968552	0.068464315	-1.406436809	0.138169071
Ptger1|19216	-1.71099352	0.060937737	-1.71099352	0.060937737
Rnf152|320311	-1.711450733	0.174924896	-1.470144008	0.160892583
Ube2cbp|70348	-1.711508961	0.024522112	-1.711508961	0.024522112
4931440F15Rik|216622	-1.711548172	0.21681309	-2.563656748	0.109738328
Zfp781|331188	-1.711573102	0.128498695	-3.086198958	0.006804607
Phtf2|68770	-1.712072458	0.065887033	-1.163442675	0.241233708
Nefh|380684	-1.712216699	0.194861962	-1.712216699	0.194861962
Spata5|57815	-1.712377294	0.175723037	-1.260883033	0.344849915
Gpc2|71951	-1.7128	0.26887049	-2.8202	0.173296754
Plcd3|72469	-1.713476514	0.146032874	-2.042980886	0.060331101
Zfp958|233987	-1.713560716	0.224903489	-1.651933176	0.232208156
Apol7a|75761	-1.714076446	0.001150119	-1.714076446	0.001150119
Myo19|66196	-1.714240271	0.047988717	-1.714240271	0.047988717
Olfm3|229759	-1.714924863	0.240084264	-1.84810098	0.2616155
Mpp2|50997	-1.715117898	0.233787796	-1.55095307	0.306183755
Ltb4r2|57260	-1.715506599	0.203984634	-1.141511102	0.417541925
Pklr|18770	-1.715592909	0.008919402	-1.578604988	0.017990658
Mpdz|17475	-1.71574472	0.176104317	-1.546700174	0.209610546
Cpeb4|67579	-1.717450638	0.24031264	-1.178880102	0.375964502
Igfals|16005	-1.717539968	2.86355E-05	-1.597617626	1.86187E-05
Fyco1|17281	-1.717549508	0.11996895	-1.570091976	0.072471932
Naf1|234344	-1.71887885	0.105540091	-1.970778971	0.048892658
6430571L13Rik|235599	-1.719195138	0.170797878	-1.292059827	0.329771645
Spats2l|67198	-1.719447509	0.200632522	-1.055876767	0.459473236
Adrb1|11554	-1.720456332	0.166799925	-3.140974485	0.016189949
Tdrd3|219249	-1.720750851	0.002889897	-1.720750851	0.002889897
Cenpj|219103	-1.720792842	0.191316928	-2.719100932	0.096306777
Fam129a|63913	-1.720952106	0.102197434	-1.741820709	0.052847403
Gsta2|14858	-1.721852257	0.085710357	-3.380983938	2.34565E-05
Tubgcp5|233276	-1.721923746	0.213207188	-3.330312925	0.083399312
Gpx8|69590	-1.722807466	0.227181455	-13.82858	1.93905E-05
Tra2a|101214	-1.723079154	0.069796084	-1.593050577	0.02841413
Akr1c6|83702	-1.725384144	0.019344402	-1.640416079	0.015250931
Arhgap27|544817	-1.725718003	0.057157444	-1.725718003	0.057157444
Lamc3|23928	-1.72830013	0.061444652	-1.72830013	0.061444652
Fam65b|193385	-1.729431459	0.099099412	-2.227265096	0.052971872
Akr1d1|208665	-1.73161085	0.014671283	-1.618159117	0.019072998
Sema6d|214968	-1.733391587	0.166293529	-1.374910106	0.235804615
Zc3h12c|244871	-1.734399745	0.276647844	1.535969564	0.275519807
C130050O18Rik|319772	-1.734908569	0.237949802	1.14606924	0.409744528
Abca3|27410	-1.736156601	0.015882812	-1.597559917	0.006295349
BC024139|271278	-1.736650368	0.012889473	-1.952377473	0.008001652
Actn1|109711	-1.737365978	0.026105076	-1.737365978	0.026105076
Eid2b|434156	-1.737659131	0.017569964	-1.737659131	0.017569964
Ptpn18|19253	-1.73788693	0.070582804	-1.48759903	0.142564231
Clec4a3|73149	-1.737913762	0.103467985	-1.383073788	0.213234617

Ankrd26|232339	-1.738221829	0.180942259	-1.460013226	0.262406953
4930430F08Rik|68281	-1.73831551	0.125734449	-2.561840353	0.047631559
Kdelc2|68304	-1.739455499	0.05318945	-1.463680732	0.106158092
Pik3ap1|83490	-1.740004305	0.117257412	-1.915513539	0.066605295
Rem1|19700	-1.74013784	0.119056117	-1.74013784	0.119056117
Cdan1|68968	-1.740420645	0.072170496	-2.098512426	0.045428982
Maoa|17161	-1.741498805	0.018612496	-1.741498805	0.018612496
Podxl2|319655	-1.741903928	0.287669356	-1.430775758	0.37678302
Pdpr|319518	-1.742638592	0.211164201	1.029063296	0.47748618
Tas1r3|83771	-1.743084174	0.208884425	-1.062358995	0.460737989
Tmie|20776	-1.743084275	0.009907419	-2.063328581	0.00241533
Nynrin|277154	-1.744630546	0.081742396	-1.333987584	0.151531093
BC030307|103220	-1.745492613	0.081398678	-1.745492613	0.081398678
Spty2d1|101685	-1.746792095	0.085761918	-1.373556401	0.175244778
Paxip1|55982	-1.747077727	0.077245671	-1.196698234	0.271502791
Lin7a|108030	-1.748256861	0.023884706	-1.897539297	0.022728792
Cd22|12483	-1.750359337	0.110888724	-1.328099769	0.233937128
2810007J24Rik|76971	-1.7506163	0.009647348	-1.538737522	0.0084537
Krit1|79264	-1.750765464	0.10834922	-1.481397862	0.099546361
Cd37|12493	-1.751773868	0.010698193	-1.601962947	0.021662221
Stat1|20846	-1.751837868	0.004728563	-1.58024103	0.004802197
Tle6|114606	-1.752781066	0.08898503	-1.844341215	0.06879483
Kif12|16552	-1.753806915	0.129965018	-3.316960282	0.007449951
Icam2|15896	-1.753914407	0.089309677	-1.368807924	0.183459367
Neurl1b|240055	-1.754128148	0.116593752	-2.108495289	0.088695485
Inha|16322	-1.755482859	0.230794647	-1.020204885	0.489224964
1200009I06Rik|74190	-1.755850976	0.046479727	-1.755850976	0.046479727
Fem1c|240263	-1.756903701	0.15676915	-1.174457979	0.355178258
Slpi|20568	-1.756913572	0.12958707	-1.540915589	0.097109963
Synpo2l|68760	-1.75696	0.25338571	-8.7848	0.047326766
Wwc1|211652	-1.757462367	0.010480433	-1.4084321	0.019302918
Igfbp6|16012	-1.757623209	0.153122711	-2.631471977	0.072947586
AI597479|98404	-1.75874351	0.109470477	-1.294501515	0.229967673
Tert|21752	-1.759544783	0.198105819	-1.759544783	0.198105819
O3far1|107221	-1.759752067	0.217844801	-5.48415	0.036928558
Cdc14b|218294	-1.759767407	0.04812941	-1.357167708	0.004135054
Caprin2|232560	-1.759940278	0.180724806	-1.913728518	0.154463118
Sema6a|20358	-1.760038628	0.000606933	-1.666743529	0.001102135
Fasn|14104	-1.760545318	0.008780127	-1.760545318	0.008780127
Ptpn7|320139	-1.760641429	0.179520222	-3.23060157	0.061792298
Gbp3|55932	-1.761071755	0.128105436	-1.162212251	0.242912755
Saa1|20208	-1.762355647	0.163308525	-3.751538053	0.02465028
Anxa9|71790	-1.762734838	0.16409575	-2.013977045	0.135283857
Tmem26|327766	-1.76464803	0.264256115	1.168103268	0.432025983
Lect1|16840	-1.766751454	0.199776926	-5.631641297	0.018448563
Wdpcp|216560	-1.768353086	0.135212242	-3.882710768	0.002318131
Il1f9|215257	-1.768684641	0.257898942	1.0485625	0.479655978
Aldh18a1|56454	-1.769106202	0.249677554	-10.16853004	0.038438126
Ids|15931	-1.771038492	0.076465443	-1.375264616	0.145808866
Ngfr|18053	-1.771597234	0.012859792	-1.771597234	0.012859792
BC005764|216152	-1.771763755	0.160702222	-3.167452019	0.049026479
Nlgn3|245537	-1.772169845	0.168803574	-1.772169845	0.168803574
D630039A03Rik|242484	-1.773553634	0.010154678	-1.42617495	0.005074572

Slc36a1|215335	-1.773746392	0.024912345	-1.376016168	0.069855468
Gm6981|629557	-1.774758159	0.122480107	-1.361343299	0.252904244
1700039E15Rik|76713	-1.77537585	0.150907079	-1.295196733	0.311944506
Susd4|96935	-1.776694004	0.219467046	1.131568473	0.388974061
Gale|74246	-1.777808618	0.025508107	-1.47994979	0.022045905
BC025920|268319	-1.778266832	0.189449736	-2.486249929	0.059958096
BC018242|235044	-1.77909193	0.007135426	-2.070642267	0.002302264
Kcnn4|16534	-1.779094089	0.145510499	-1.503228565	0.175719424
Car5a|12352	-1.78005237	8.38919E-05	-1.883161299	8.18144E-05
Gpat2|215456	-1.780208399	0.035978243	-1.491932034	0.07439302
Cdh13|12554	-1.780405662	0.189896141	-4.627291613	0.029720189
Nr4a2|18227	-1.780617361	0.217915002	-3.677262626	0.091207367
Gpr155|68526	-1.780692891	0.025921744	-1.591864552	0.053575028
Ncs1|14299	-1.783027624	0.21016136	-1.783027624	0.21016136
1810046K07Rik|69809	-1.784068219	0.02328419	-1.782590024	0.00577815
Casz1|69743	-1.784425604	0.069760203	-1.517147695	0.141175205
Fdx1l|68165	-1.785307802	0.118258462	-2.455395438	0.054147816
Apba1|319924	-1.785416269	0.155405531	-2.260298314	0.099496502
Sult2a5|434264	-1.785729767	0.188653721	-2.491834775	0.079896404
Fam26f|215900	-1.786120975	0.231195524	-2.985012121	0.094640659
Adamdec1|58860	-1.786837004	0.071442825	-2.130585869	0.009190051
Eps8l1|67425	-1.786957419	0.111068199	-1.314946465	0.234929331
Sec61g|20335	-1.787097513	0.060066958	-1.518219694	0.122695623
Ttll4|67534	-1.787116331	0.06302931	-1.787116331	0.06302931
Lrtm1|319476	-1.787256287	0.002466475	-1.736227276	0.006138725
Samhd1|56045	-1.788638075	0.005584573	-1.59366214	0.004636347
Olfml3|99543	-1.789770248	0.011060672	-1.969231529	0.002086595
Neb|17996	-1.791051041	0.179076836	-1.895694497	0.181653608
BC052040|399568	-1.791539876	0.037637195	-2.199141021	0.016866593
Kif1a|16560	-1.791715806	0.123191515	-2.201129506	0.07889046
Iqcj-schip1|100505386	-1.791866768	0.265913293	-1.61274826	0.354514631
1700019G17Rik|75541	-1.792424533	0.065153774	-1.792424533	0.065153774
Srek1ip1|67288	-1.792678304	0.024661008	-2.621149395	7.48605E-05
Rpap2|231571	-1.792689775	0.021087817	-1.353638436	0.047311291
Pds5b|100710	-1.792939085	0.033278237	-1.505368463	0.054081083
Tlr3|142980	-1.795142222	0.125956585	-4.358395581	0.000478307
Amigo3|320844	-1.795280245	0.039511556	-1.666626295	0.076251112
Homez|239099	-1.795943498	0.00813635	-2.349595572	0.000127976
Pdpn|14726	-1.797598448	0.239870069	-4.58098	0.070596339
Mpped2|77015	-1.797598448	0.239870069	-4.58098	0.070596339
Dab2|13132	-1.798285619	0.050986808	-1.446041379	0.084135171
Satb2|212712	-1.798461317	0.268243122	-1.430775758	0.37678302
Cluap1|76779	-1.798476472	0.008679465	-2.149192874	0.002069805
Orm2|18406	-1.799939365	0.02697974	-1.529839391	0.042455821
Dennd3|105841	-1.800680309	0.11948791	-1.298550064	0.256140286
Meis1|17268	-1.801396015	0.06159594	-1.509651788	0.125738964
Hook1|77963	-1.801914474	0.155123802	-1.681630954	0.190077218
Arhgap33|233071	-1.802331418	0.055979293	-1.515929971	0.109433913
Snora30|100217442	-1.802357914	0.179856773	-4.061967816	0.040170907
Wnt7b|22422	-1.802508571	0.256175854	-2.8	0.173296754
2900006K08Rik|72873	-1.80356067	0.191140862	-3.871375758	0.023866915
4933407H18Rik|71101	-1.80490409	0.027963012	-2.176636362	0.012859592
Wnt9b|22412	-1.806371492	0.098907422	-3.251136338	0.002767779

Orm1|18405	-1.806923654	0.021314191	-2.268375539	0.001743138
Itpkb|320404	-1.80749729	0.175347838	-1.80749729	0.175347838
Hivep3|16656	-1.808809969	0.109718861	-1.407429699	0.226110948
Slc10a5|241877	-1.809324447	0.205358072	-1.408484491	0.285847742
Asap1|13196	-1.809705872	0.029392236	-1.518447808	0.043189585
Alox12|11684	-1.809892288	0.171933905	-1.225154778	0.364078421
Dcp1a|75901	-1.811188585	0.049663242	-2.268107142	0.023559599
Nos1ap|70729	-1.812406165	0.295357991	1.170129778	0.437059136
1700028K03Rik|76421	-1.813086369	0.057166167	-1.54860358	0.116754605
Adcy7|11513	-1.813769934	0.205267889	1.053670469	0.453967044
Orai2|269717	-1.814148272	0.151280701	-1.814148272	0.151280701
Ldlr|16835	-1.814929015	0.023967452	-1.590802185	0.018414308
Tmc8|217356	-1.815162932	0.004264936	-2.130676874	0.000951787
Sntb1|20649	-1.815984541	0.166844726	-2.514374605	0.110918024
4930441O14Rik|68271	-1.816891905	0.188349461	-2.555049552	0.120507484
Atp8a1|11980	-1.818224585	0.06120423	-2.171913251	0.005451997
Wdr90|106618	-1.819054919	0.02253112	-1.3370474	0.072253745
Zfhx4|80892	-1.819553779	0.12651166	-1.226824527	0.271376896
Lair1|52855	-1.820558028	0.129732432	-2.683045165	0.061346791
Zfp532|328977	-1.821098179	0.153399516	-1.275302041	0.325536689
Gbp2|14469	-1.821316942	0.011634642	-1.522840748	0.020141173
Angpt1|11600	-1.821551101	0.18693396	-2.566129524	0.119075489
Thbs1|21825	-1.822039251	0.073514296	-2.050854951	0.036971362
Slc14a1|108052	-1.823719423	0.260442814	-2.8202	0.173296754
Cys1|12879	-1.824853931	0.045752102	-2.133493644	0.015962787
Ank2|109676	-1.825816307	0.170756375	-1.825816307	0.170756375
4930528F23Rik|75178	-1.827216516	0.246949986	-3.034138293	0.085514775
Tnk1|83813	-1.827230926	0.221091992	-6.960866667	0.041230736
Lyl1|17095	-1.827679911	0.074649995	-2.200769692	0.052947244
4922501C03Rik|382090	-1.828541321	0.099245706	-1.412532591	0.206417113
Chtf18|214901	-1.829120897	0.260231664	-2.8	0.173296754
Gm216|241112	-1.829120897	0.260231664	-2.8	0.173296754
Tpm2|22004	-1.829374559	0.030792843	-1.517277902	0.041820772
Slc25a23|66972	-1.829839811	0.000570653	-1.815371176	0.000176783
Traf3ip2|103213	-1.831898005	0.011109707	-1.635086226	0.007434584
Plekhg2|101497	-1.832023823	0.000173906	-1.73173963	0.000488063
Tnnt1|21955	-1.832503123	0.021627045	-1.597413893	0.004630179
Reep6|70335	-1.832544371	0.000478985	-1.907466058	4.17706E-05
Pltp|18830	-1.832572352	0.056498999	-1.437721911	0.089476575
Atp2b2|11941	-1.835370832	0.00868041	-1.437978841	0.02255789
Ninl|78177	-1.835676892	0.084941169	-1.835676892	0.084941169
Map3k8|26410	-1.835792849	0.210605694	1.076475079	0.439904845
L3mbtl3|237339	-1.83611879	0.229613791	-3.574776786	0.124634646
Rnf186|66825	-1.837389231	0.145998984	-2.687832786	0.035898787
Rabepk|227746	-1.83798843	0.035654931	-1.877159616	0.015195491
Tcerg1|56070	-1.839723868	0.071416039	-1.683790078	0.055224753
E030010A14Rik|226040	-1.84005013	0.098206264	-1.371020224	0.203095497
Gm11517|629750	-1.84007918	0.134845996	-1.255988274	0.297047748
BC046404|192976	-1.842110588	0.247630967	-8.6404	0.070560672
Sult2a1|20859	-1.842360379	0.073791938	-2.762415626	0.016210463
Sap130|269003	-1.843564165	0.001146382	-1.694110413	0.000930468
Trim12a|76681	-1.843602834	0.008194094	-1.865645711	0.00469604
Foxp2|114142	-1.843723	0.034177607	-1.946822772	0.011633102

Rab25|53868	-1.844067643	0.156287994	-3.293979798	0.05623339
Slc7a11|26570	-1.845010453	0.116821267	-1.845010453	0.116821267
Bcs1l|66821	-1.846552863	0.002300338	-1.504172687	0.00380195
Dnm3os|474332	-1.84771178	0.280957704	-2.8202	0.173296754
Ccdc88b|78317	-1.848257557	0.008034241	-1.848257557	0.008034241
Pik3r3|18710	-1.850368723	0.189900945	-1.223054295	0.386779131
Zfp105|22646	-1.850596667	0.20440672	-9.252983333	0.014711231
Osbpl3|71720	-1.850887671	0.179849574	-6.989187469	0.008832059
Rev1|56210	-1.851211861	0.096543895	-1.800969012	0.073250162
2810002D19Rik|66457	-1.851767119	0.039272415	-2.22885046	0.023274768
P4htm|74443	-1.851786003	0.180261229	-2.752539394	0.103543379
Acvr1b|11479	-1.852088098	0.113400974	-1.786948262	0.091938875
Fam149a|212326	-1.853384446	0.000377673	-1.943371822	0.000185052
Abca8a|217258	-1.854142071	0.023609401	-1.395212591	0.066316428
Cyyr1|224405	-1.855620432	0.095991455	-2.799211487	0.031793882
Sult1c2|69083	-1.855679978	0.088552872	-2.154256339	0.040798994
Kcnt1|227632	-1.856128943	0.219190212	-7.38365	0.04272972
Myo5c|208943	-1.856128943	0.219190212	-7.38365	0.04272972
Dhx58|80861	-1.857331327	0.005563773	-2.345040091	0.000243221
Hddc3|68695	-1.858440088	0.116577332	-2.324727716	0.042602132
1700029G01Rik|66938	-1.858761134	0.037369625	-1.79278341	0.009299252
Acsl3|74205	-1.858978354	0.076665951	-1.560918642	0.153850793
Ces2c|234671	-1.85916985	0.044224611	-1.518980191	0.029730475
Ms4a6d|68774	-1.859443999	0.135792921	-1.859443999	0.135792921
Mtap1a|17754	-1.860575911	0.254099771	-13.2522	0.061310843
Cyp26a1|13082	-1.862263184	0.112821641	-2.883828602	0.041884096
Fbxl21|213311	-1.86234949	0.049211106	-1.608438725	0.100735226
Esrrg|26381	-1.862793093	0.189523858	-1.534574665	0.29227622
Kcnk10|72258	-1.862893345	0.212484052	-2.100621395	0.237974883
Papd4|100715	-1.863814276	0.005873401	-1.742006247	0.0134775
Ldhd|52815	-1.863869893	4.03251E-05	-2.047448046	6.83593E-06
Rgs10|67865	-1.864981831	0.186000037	1.189154721	0.297267973
Mmab|77697	-1.865598773	0.018755272	-1.575206212	0.01918897
1700054N08Rik|73420	-1.867484357	0.243835528	-3.335808081	0.159331054
3300002I08Rik|69277	-1.86816	0.248985221	-2.76078	0.173296754
Csnk1g1|214897	-1.868416514	0.112967584	-2.164287385	0.074945611
Tlcd2|380712	-1.868645758	0.00138008	-2.038759478	4.35865E-05
Rgs14|51791	-1.869800872	0.061096291	-2.22696766	0.007600498
Fcrl1|229499	-1.870446611	0.199436708	-9.162133333	0.014939816
Fndc1|68655	-1.870874437	0.201091539	-2.192054545	0.202100697
Prl8a1|73244	-1.871846364	0.032037233	-1.565193139	0.066553055
Oasl1|231655	-1.872893739	0.005871036	-1.58305932	0.013157002
Zmiz1|328365	-1.873599994	0.141980762	-2.207384787	0.125806184
Fzd6|14368	-1.874400784	0.056226331	-1.874400784	0.056226331
Kif16b|16558	-1.875518038	0.080121564	-2.002991804	0.035286946
Csgalnact1|234356	-1.877092895	0.222788753	-4.58098	0.070596339
Oas1b|23961	-1.877888167	0.068486625	-1.574824995	0.126515023
Lynx1|23936	-1.878993253	0.08778359	-2.371926695	0.026024097
Gpr17|574402	-1.879068661	0.091150257	-1.879068661	0.091150257
Cpne7|102278	-1.88028	0.188260531	-5.48415	0.036928558
Xkr9|381246	-1.880307284	0.060772617	-1.694033207	0.020792153
Ear12|503845	-1.880464457	0.247325655	-4.112363095	0.139484904
Slc24a5|317750	-1.882911392	0.310017691	1	#DIV/0!

Kif23|71819	-1.884973952	0.181378769	-2.128806798	0.164353183
Lama3|16774	-1.885394867	0.145682001	-3.271550505	0.056060025
Ttbk2|140810	-1.885532523	0.032692283	-1.640499683	0.066560517
Gm4172|100043016	-1.885966667	0.170446566	1	#DIV/0!
Msi1|17690	-1.887635695	0.277658334	-2.76078	0.173296754
Hoxa3|15400	-1.888180595	0.15770525	-1.888180595	0.15770525
Kifap3|16579	-1.888371978	0.085705613	-1.888371978	0.085705613
Rcan2|53901	-1.890155398	0.008906396	-2.167448248	0.00535914
Vtcn1|242122	-1.891384455	0.107444097	-3.658060998	0.013210415
Dhtkd1|209692	-1.896508565	0.14353425	-1.194109919	0.3263038
Zfp558|72230	-1.900083376	0.275082594	-2.8202	0.173296754
Cyp3a41b|100041375	-1.900319956	0.115954972	-3.171261283	0.037124439
Ppapdc3|227721	-1.903170246	0.169685854	-4.062896753	0.05384268
Guca1a|14913	-1.903576112	0.097504658	-2.154517175	0.079555671
Zfp608|269023	-1.903588454	0.131447934	-1.903588454	0.131447934
Nedd9|18003	-1.903617713	0.124222449	-1.350416437	0.26588373
Ttll11|74410	-1.903757692	0.028737933	-2.080522318	0.015430475
Gins1|69270	-1.905976634	0.085455316	-1.454936561	0.174620242
Gstt1|14871	-1.906011132	0.000574614	-1.906011132	0.000574614
Mid1|17318	-1.906280546	0.057063295	-1.596166245	0.046908876
Hmgb1-rs17|628431	-1.907875443	0.086917389	-2.440175866	0.045983368
Mpz|17528	-1.909574755	0.040504288	-1.916744772	0.001456971
Fam102b|329739	-1.911957411	0.080791576	-3.235045214	0.004477426
Pde6g|18588	-1.912892293	0.110916254	-1.912892293	0.110916254
Gm13157|100041677	-1.913072102	0.120263227	-5.029012121	0.000977813
Nlrp6|101613	-1.91539941	0.000185298	-1.777207537	4.79773E-05
Cd248|70445	-1.916323028	0.003776931	-1.693859469	0.001401338
Sox4|20677	-1.916424218	0.27096672	-2.76078	0.173296754
Wfikkn2|278507	-1.916608717	0.141453136	-1.916608717	0.141453136
Cmklr1|14747	-1.916896654	0.000557681	-1.916896654	0.000557681
Itgbl1|223272	-1.917029266	0.228503045	1.103561233	0.446958035
Tmem185b|226351	-1.917172453	0.008942391	-1.917172453	0.008942391
Cyp2d40|71754	-1.917694917	0.005670131	-1.691347849	0.004873614
Rasl12|70784	-1.917799396	0.210568613	-1.870260925	0.258949734
Ap1s3|252903	-1.917964997	0.109027827	-1.917964997	0.109027827
Rhoh|74734	-1.918706376	0.14869179	-1.918706376	0.14869179
Klrk1|27007	-1.919594365	0.118722426	-1.919594365	0.118722426
Hebp2|56016	-1.920502198	0.188309218	-3.215169561	0.110411701
Ncf4|17972	-1.921113931	0.064819936	-2.067078482	0.042165435
Gm13697|668115	-1.922392518	0.090687625	-2.00076489	0.084959835
Akr1c20|116852	-1.923680465	0.004149908	-1.85976096	0.00261705
Serpina3i|628900	-1.926135102	0.207858507	-7.662133333	0.044268711
Btbd19|78611	-1.929833488	0.056094076	-1.876640071	0.060628458
Gm4951|240327	-1.929907314	0.055503849	-1.462921261	0.067295027
Tcea3|21401	-1.931334964	0.011593213	-2.365123822	0.003863126
Fam115c|232748	-1.931651429	0.242141558	-2.76078	0.173296754
Osbpl10|74486	-1.931651429	0.242141558	-2.76078	0.173296754
Slc25a48|328258	-1.931686875	0.090257524	-1.931686875	0.090257524
Tle2|21886	-1.933190255	0.003986198	-1.865548328	0.00332922
Serac1|321007	-1.93353404	0.031831581	-2.30716527	0.020824065
Nlrc5|434341	-1.935898513	0.029407147	-1.935898513	0.029407147
Sult3a1|57430	-1.936284575	0.053899237	-2.317330161	0.010481406
Aox1|11761	-1.93729537	1.95195E-05	-1.993387625	5.42843E-06

Epha7|13841	-1.9382541	0.060326482	-1.532888167	0.116087487
Slc35e4|103710	-1.939226528	0.03505609	-2.232408978	0.006282127
Gm11711|100043125	-1.939318674	0.17829403	-1.108309306	0.425090047
Fads3|60527	-1.941320592	0.051677676	-1.941320592	0.051677676
Aox3|71724	-1.94209543	0.006947521	-2.14219353	0.006391864
Slc22a30|319800	-1.943353285	0.012519889	-1.735641795	0.027397847
Dclk3|245038	-1.943481033	0.011405821	-2.172171976	0.001837479
Hnmt|140483	-1.94445762	0.005270897	-1.94445762	0.005270897
Folr1|14275	-1.944741682	0.204334673	-7.73615	0.043865772
Clca1|12722	-1.945608757	0.085416324	-1.458398111	0.172628657
Rgs19|56470	-1.949021757	0.078836343	-3.348453291	0.001159828
Sec31b|240667	-1.955405907	0.082402455	-2.215190186	0.055779378
Cdca7|66953	-1.955477398	0.085635064	-1.47586734	0.17472525
Ralgps1|241308	-1.955559217	0.050904321	-1.646698649	0.097500653
Tmem107|66910	-1.9594491	0.141660828	-1.9594491	0.141660828
E530011L22Rik|320301	-1.961283333	0.170446566	1	#DIV/0!
Cyp2b13|13089	-1.961779337	0.010622332	-1.676018567	0.007938546
Col20a1|73368	-1.961828742	0.038103933	-2.251790217	0.008809095
Zfp112|57745	-1.961881667	0.177852185	-3.378637012	0.099347744
Tdp2|56196	-1.962364866	0.005180086	-2.614853779	0.000167031
Robo1|19876	-1.962415389	0.109482867	-1.394052033	0.231072164
Qrfp|227717	-1.962703401	0.168876246	-16.57554	1.22771E-05
Peg3|18616	-1.963518127	0.251523633	-1.189602278	0.402044751
Xrcc3|74335	-1.96492335	0.013348971	-1.96492335	0.013348971
Slc4a5|232156	-1.965173863	0.299667008	1	#DIV/0!
Wdr38|76646	-1.966200228	0.073733645	-1.966200228	0.073733645
Inmt|21743	-1.966721607	0.005519719	-2.738249114	1.01641E-05
1700012D14Rik|75479	-1.968120319	0.196600626	-1.183107182	0.414958471
Hist1h4b|326620	-1.96878985	0.192508438	-4.117133333	0.090539541
Btbd3|228662	-1.969291979	0.083209168	-2.473441692	0.059688336
Pdlim3|53318	-1.969669457	0.301680421	-2.8	0.173296754
Lss|16987	-1.971111983	0.009472095	-1.673752472	0.0039813
Rgma|244058	-1.971401071	0.088005991	-1.212149433	0.291142831
Mmp23|26561	-1.973429346	0.088221507	-1.973429346	0.088221507
Mthfd2|17768	-1.97509	0.182398485	-9.87545	0.017130116
S1pr4|13611	-1.976528171	0.051334967	-1.660055799	0.104884137
Epb4.1l4a|13824	-1.978898648	0.185498662	-3.003528484	0.128782899
Zfp871|208292	-1.978923341	0.108941014	-1.706340518	0.057021707
Gm8989|668128	-1.979166667	0.170446566	1	#DIV/0!
Ttc30a1|78802	-1.979417202	0.131795938	-1.511053308	0.257287526
Spopl|76857	-1.979678572	0.113084143	-1.421556615	0.239062196
Usp53|99526	-1.985167572	0.188632987	-2.959471574	0.148386268
Spib|272382	-1.985503631	0.031925078	-1.985503631	0.031925078
Ngb|64242	-1.986472724	0.212243368	-2.024513774	0.263278926
Spic|20728	-1.986515767	0.143173298	-2.758874541	0.092509394
Lrfn4|225875	-1.987138839	0.130700223	-1.934315561	0.066302254
Pcp4l1|66425	-1.988561066	0.001965512	-1.988561066	0.001965512
Kctd18|51960	-1.988912379	0.021134536	-2.285608541	0.002039176
1700112E06Rik|76633	-1.989886291	0.213433769	-1.714700598	0.281517273
Met|17295	-1.990124393	0.018018379	-1.990124393	0.018018379
Ccdc102a|234582	-1.990333019	0.068318823	-3.101004576	0.018122458
Snca|20617	-1.997377461	0.098017793	-1.449965718	0.203310794
AI480653|268880	-1.997884105	0.070172398	-3.236555486	0.015861588

Lgals1|16852	-1.998289322	0.036164014	-2.199159318	0.000635477
Mfsd7b|226844	-1.999057169	0.181457279	-1.310987214	0.365074041
Zfp81|224694	-2	0.293589278	1	#DIV/0!
Ckap2l|70466	-2	0.293589278	1	#DIV/0!
Slc29a2|13340	-2.000787317	0.056326076	-1.627865486	0.113646452
Gm8221|666661	-2.001053555	0.223204954	-6.23615	0.093645599
Spin2|278240	-2.001053555	0.223204954	-6.23615	0.093645599
Nkapl|66707	-2.001053555	0.223204954	-6.23615	0.093645599
Zfp169|67911	-2.002286104	0.058246171	-4.048670762	0.000959555
Vnn1|22361	-2.008079108	0.001146249	-2.008079108	0.001146249
Myh11|17880	-2.009174895	0.181226041	-1.171690729	0.264743498
Trim68|101700	-2.009378258	0.162622104	-3.680625097	0.079834633
1700037C18Rik|73261	-2.011255126	0.087303242	-1.494317989	0.178226543
Lsamp|268890	-2.011313771	0.191665382	-7.662133333	0.044268711
Cenpo|52504	-2.011623963	0.013237346	-2.011623963	0.013237346
Bex4|406217	-2.012102784	0.112912358	-2.012102784	0.112912358
Ugt1a5|394433	-2.014064327	0.004578147	-2.396213952	0.001021307
Syt1|20979	-2.014198718	0.115157798	-2.200400113	0.093994577
Ttll3|101100	-2.018480767	0.112449862	-1.852159739	0.148833313
Mad2l1|56150	-2.01916291	0.156530084	-4.476555556	0.052460665
Nkain4|58237	-2.020822946	0.194485374	-8.0388	0.046362285
Pcdh17|219228	-2.021583677	0.100974076	-4.249323052	0.013888815
Ltbp3|16998	-2.02340284	0.025329882	-2.242701981	0.003267607
1700010I14Rik|66931	-2.023936709	0.212274449	-1.935026828	0.275417362
C1rb|667277	-2.024719533	0.16508133	-3.410627532	0.095393078
Slc26a2|13521	-2.025054571	0.193616745	-8.055633333	0.046112137
Tgfb3|21809	-2.026450382	0.071545744	-1.648573331	0.145855148
Olfm1|56177	-2.030548711	0.040911657	-2.030548711	0.040911657
Rif1|51869	-2.03191842	0.196625011	-1.684453115	0.235002086
S100a6|20200	-2.03352718	0.183640662	-3.684652689	0.031325192
Pag1|94212	-2.035909972	0.132017906	-1.378781325	0.284084379
Srebf1|20787	-2.03682026	0.016377001	-2.056418538	0.00947129
L1cam|16728	-2.039616574	0.115699646	-1.520561372	0.236769574
Tsc22d1|21807	-2.042525523	0.015363949	-1.836039932	0.032179597
Zfp955a|77652	-2.044476742	0.077959351	-1.718607334	0.133587628
Cd53|12508	-2.046491201	0.019525816	-2.370732748	0.001243303
Gstcd|67553	-2.048269349	0.111456195	-1.505571555	0.231562551
Rnls|67795	-2.04835811	0.119269418	-4.65400259	0.021961238
3010026O09Rik|68067	-2.04887478	0.003769115	-2.25135025	0.000555185
Pcyt1b|236899	-2.049118414	0.216296019	-5.7948	0.078760145
Prpsap2|212627	-2.050845031	0.017526669	-2.338380968	0.008697004
Fam84a|105005	-2.051522111	0.120673337	-2.051522111	0.120673337
Cenpn|72155	-2.055175254	0.172940789	-6.404816667	0.044430303
Tbc1d19|67249	-2.055194718	0.183033632	-4.673019335	0.050653644
Ttn|22138	-2.056108832	0.19000698	-8.179166667	0.047131331
Flrt1|396184	-2.056474071	0.045820433	-2.449285622	0.005291819
Fam70b|272465	-2.056640928	0.260634069	-2.8	0.173296754
Gm12250|631323	-2.056739856	0.07654734	-3.752668649	0.012987395
ORF63|224419	-2.060872553	0.259850238	-2.8202	0.173296754
Trerf1|224829	-2.062826416	0.115926538	-3.182952324	0.045975943
Prkar2b|19088	-2.068438279	0.171659036	-6.44615	0.04497135
Ypel1|106369	-2.068885773	0.189790204	-1.151738686	0.425983244
Kdm4c|76804	-2.070343092	0.061898637	-1.283944599	0.17736405

Pcdhb22|93893	-2.071064144	0.170222954	-6.454333333	0.043911777
Tmem163|72160	-2.071449969	0.164004728	-2.071449969	0.164004728
Ttc39a|230603	-2.07169007	0.103797613	-2.07169007	0.103797613
Muc1|17829	-2.071895575	0.096327184	-2.752179715	0.067447689
Plscr2|18828	-2.074604712	0.000858678	-2.074604712	0.000858678
Zfp354a|21408	-2.075368581	0.032701521	-2.075368581	0.032701521
Lax1|240754	-2.07672958	0.126778717	-14.79178333	1.41502E-05
Syt7|54525	-2.079430312	0.034655335	-2.079430312	0.034655335
Auts2|319974	-2.080084616	0.126317417	-2.080084616	0.126317417
Trim30a|20128	-2.083036579	0.092618584	-1.444691243	0.178694254
Paqr6|68957	-2.084327169	0.168965258	-6.495666667	0.044433779
Kdm3a|104263	-2.084581251	0.034339365	-2.404310652	0.008793082
Raet1d|56554	-2.085802437	0.029168041	-1.695898315	0.042700423
G630025P09Rik|74341	-2.090524193	0.249174852	-21.44690607	0.093588468
2010001M06Rik|69812	-2.09431784	0.015741681	-2.335115428	0.004596932
Kazn|71529	-2.095624333	0.035878602	-2.095624333	0.035878602
Mkx|210719	-2.096343378	0.125877407	-4.329287879	0.039981992
Oas2|246728	-2.099276916	0.013752827	-2.099276916	0.013752827
Mia2|338320	-2.101707552	0.094854468	-2.176878891	0.066598478
Gamt|14431	-2.10292548	0.005125387	-2.47133404	0.002960401
Ccdc14|239839	-2.105362353	0.182612366	-14.91298333	0.025273478
Spag1|26942	-2.106373293	0.187648848	-2.090787879	0.212529809
Itgal|16408	-2.106568255	0.066908872	-2.106568255	0.066908872
Kcnab3|16499	-2.108265924	0.136081663	-1.304556138	0.302249255
Ankrd37|654824	-2.109369309	0.105947395	-2.109369309	0.105947395
Mfsd4|213006	-2.109381504	0.166326609	-1.632541037	0.217964699
Mtap1b|17755	-2.109435686	0.182789127	-8.3913	0.047323967
4930452B06Rik|74430	-2.109789858	0.188762108	-1.137469949	0.432079999
BC013712|230787	-2.111102036	0.051373229	-4.589794721	8.08424E-05
Rprl1|19783	-2.111690895	0.046109325	-2.111690895	0.046109325
Trim14|74735	-2.118633609	0.020142815	-2.566413	0.01328841
Hdac9|79221	-2.11914261	0.211056266	-3.925	0.173296754
Ttc12|235330	-2.119797951	0.101973574	-3.548816479	0.043809509
Cd5|12507	-2.121883366	0.180385446	-8.440816667	0.046554152
Prg4|96875	-2.122054491	0.000738794	-2.562681868	9.52412E-05
Tmprss9|432478	-2.123937143	0.204963277	-6.194816667	0.093788939
Rps6kl1|238323	-2.124455432	0.233050069	-1.29416451	0.382135102
Ccdc126|57895	-2.125380149	0.117443208	-4.211948026	0.039415209
A930015D03Rik|77810	-2.126211544	0.017054522	-2.126211544	0.017054522
C530005A16Rik|654318	-2.127449592	0.185714947	-10.76386667	0.042008874
H2-Q1|15006	-2.127710849	0.065367474	-2.127710849	0.065367474
Ccdc67|234964	-2.129077962	0.217007669	-1.151755433	0.445938962
Fgd1|14163	-2.12958694	0.123752242	-1.33425067	0.2645575
Dimt1|66254	-2.130326203	0.125645262	-3.794636269	0.058410186
Matn2|17181	-2.130439108	0.156937792	-2.392902078	0.098232087
Spag8|433700	-2.131711453	0.052958369	-2.535591761	0.010005017
Fam33a|66140	-2.132012994	0.093481736	-4.213442893	0.021945248
Ift122|81896	-2.133268831	0.045319142	-2.133268831	0.045319142
Zfp882|382019	-2.133537499	0.010513446	-1.556657416	0.026382135
Zfp641|239652	-2.135885189	0.074601683	-1.572050156	0.144275213
Bfsp1|12075	-2.138108571	0.203349305	-6.23615	0.093645599
Pcdh18|73173	-2.138108571	0.203349305	-6.23615	0.093645599
Cpxm2|55987	-2.138650578	0.226980942	1.287350529	0.356134013

Saa2|20209	-2.140480969	0.123389169	-6.094008494	0.01856575
Fgl2|14190	-2.1428024	0.149982927	-1.218889544	0.347562276
Map3k15|270672	-2.142966048	0.127044585	-2.695412121	0.103958819
Gm5901|545989	-2.143887778	0.284786919	1	#DIV/0!
Tmem221|434325	-2.145023279	0.101128193	-2.145023279	0.101128193
Dct|13190	-2.14543621	0.072780038	-3.60068269	0.005941273
Pdzd2|68070	-2.145761219	0.192743788	-2.528222571	0.213970332
Ms4a8a|64381	-2.148161588	0.203769197	-3.245448485	0.128662
Fcgr1|14129	-2.149051975	0.022208125	-3.230967981	0.0015659
Pvrl4|71740	-2.151133523	0.026763451	-1.799882127	0.048905899
Trip11|109181	-2.154039425	0.189410248	-1.081750489	0.455876868
Dsg1c|211924	-2.157538549	0.086961662	-3.231232245	0.046535676
Fam55b|78252	-2.157615528	0.015993626	-2.157615528	0.015993626
Bspry|192120	-2.15779791	0.164202615	-4.56078	0.070574133
Plscr4|235527	-2.158349577	0.059126306	-1.697466591	0.118072188
Atp7b|11979	-2.158909876	0.07792219	-1.632178033	0.1590466
Nek1|18004	-2.159487014	0.151040841	-5.381832196	0.052347267
Cyp2d11|545123	-2.159876561	0.02155677	-1.505090398	0.059304487
Vmn2r29|76229	-2.159933081	0.239044833	-4.6202	0.07056113
Rspo1|192199	-2.160284926	0.084767199	-3.443237573	0.0375981
Snhg11|319317	-2.162029369	0.120578338	-3.750113236	0.053068718
Cyp4f41-ps|77875	-2.163199384	0.163339522	-4.58098	0.070596339
Slc44a3|213603	-2.165217796	0.047649818	-3.189564283	0.017879057
Slc24a3|94249	-2.165726745	0.136893432	-4.354087329	0.058838492
Zfp78|330463	-2.169777899	0.092045824	-3.046232117	0.052673028
Col27a1|373864	-2.169968924	0.029790369	-1.739693855	0.041724487
Golt1b|66964	-2.170022613	0.007621502	-2.143668492	0.00705986
Akap17b|338351	-2.17307714	0.13639866	-1.421311974	0.293279282
Apol11b|328563	-2.175857684	0.204988494	-1.72578057	0.278974436
Cnih2|12794	-2.176769117	0.10753859	-3.194032296	0.046780431
Sned1|208777	-2.177176195	0.054408035	-1.686454286	0.102388555
Xpnpep3|321003	-2.178364004	0.032212946	-2.499142486	0.017295365
Kif26a|668303	-2.17965385	0.097660755	-2.17965385	0.097660755
Abcc4|239273	-2.182880837	0.027511359	-2.182880837	0.027511359
Mvk|17855	-2.186118721	0.005084855	-2.186118721	0.005084855
5830403L16Rik|240817	-2.186303598	0.194725898	-6.60118	0.089542423
9530091C08Rik|320440	-2.191158574	0.155337017	-3.772567698	0.096195335
Pgap1|241062	-2.194541037	0.184609057	-6.8996	0.070566833
Mcf2l|17207	-2.196306932	0.194332322	-6.56078	0.089099836
Dbndd1|72185	-2.201708571	0.149853351	-6.42165	0.04426109
Rundc2a|385605	-2.201708571	0.149853351	-6.42165	0.04426109
Lck|16818	-2.202222352	0.071127357	-2.997788186	0.046481411
Traf3ip3|215243	-2.203647852	0.082646632	-4.800038482	0.005589043
Fdps|110196	-2.205405452	0.03107761	-1.648206617	0.052114915
Osr1|23967	-2.205623086	0.266859735	-3.925	0.173296754
Ccdc112|240261	-2.206415744	0.192366027	-6.6202	0.087728953
1500031L02Rik|66994	-2.209785183	0.048063549	-2.209785183	0.048063549
Trpc3|22065	-2.210108571	0.149695126	-6.44615	0.04497135
Shank2|210274	-2.210962063	0.030942987	-2.354073878	0.005347169
Fbxl7|448987	-2.212914286	0.148235538	-6.454333333	0.043911777
Mefv|54483	-2.212914286	0.148235538	-6.454333333	0.043911777
Agtpbp1|67269	-2.216974635	0.022794178	-3.869968394	0.000433549
Gm5113|330503	-2.220127798	0.21795853	-10.875	0.093289779

Hoxb2|103889	-2.22126845	0.056960865	-1.726179098	0.111569177
Sbsn|282619	-2.222366394	0.043898134	-1.859152332	0.089917904
Lrrcc1|71710	-2.222805202	0.120304071	-4.96812931	0.039290251
Trdmt1|13434	-2.222831424	0.00557498	-2.457412942	0.000201519
Cytip|227929	-2.225197518	0.080495503	-1.717169224	0.16500186
I830077J02Rik|433638	-2.227085714	0.147268402	-6.495666667	0.044433779
Ptch2|19207	-2.23042676	0.083024142	-1.612564007	0.167745938
Csdc2|105859	-2.232032051	0.110114588	-2.629015854	0.067030285
Grhl2|252973	-2.232568214	0.198201397	-6.95765	0.094047919
Dok4|114255	-2.235081162	0.00864061	-1.924495238	0.025862351
Ern1|78943	-2.236054103	0.03825844	-1.473007062	0.095278674
Abcb1a|18671	-2.236545052	0.035130182	-1.539606843	0.094669528
Zswim3|67538	-2.23683097	0.131381789	-2.178440208	0.179699967
Bard1|12021	-2.241874286	0.194723882	-3.925	0.173296754
Acta2|11475	-2.242194257	0.239088122	-1.058653504	0.437113281
Mterfd3|74238	-2.247011366	0.106793656	-1.495670894	0.223101858
Cd274|60533	-2.249948892	0.082636699	-2.536606624	0.06782142
Arap2|212285	-2.250182863	0.032373611	-2.480900876	0.035830014
Lrrc8e|72267	-2.250939227	0.093002716	-1.667235359	0.192078613
Ace|11421	-2.253230542	0.063772244	-2.207376425	0.078825309
Cyp2a22|233005	-2.254935741	0.002294043	-2.093057153	0.005736526
Gm14446|667373	-2.264285714	0.193051427	-3.925	0.173296754
Cyp51|13121	-2.267493286	0.071704615	-1.486463048	0.012858179
Casq1|12372	-2.274313339	0.15693707	-16.1991	0.023548285
Bbs5|72569	-2.274766101	0.100028865	-4.063318182	0.045249537
Sspo|243369	-2.278171787	0.143955391	-3.221509091	0.127882662
41155|24050	-2.278457143	0.191540287	-3.9746	0.173296754
Zfp867|237775	-2.280943652	0.033978584	-2.280943652	0.033978584
Zranb3|226409	-2.283243483	0.071929572	-4.446816333	0.017615005
Socs1|12703	-2.283504809	0.207861329	1.060234708	0.471507944
Fbln7|70370	-2.287522151	0.052558468	-1.657367521	0.063478851
Gpr65|14744	-2.28800522	0.126583012	-2.264525909	0.158102501
Wdr27|71682	-2.291317238	0.1242064	-1.560752707	0.260403166
Sox10|20665	-2.292313107	0.221828508	-3.925	0.173296754
Atf7|223922	-2.294592172	0.072279564	-1.716996305	0.145885919
Slain1|105439	-2.297939911	0.193498082	-4.6202	0.07056113
Vps13a|271564	-2.298047956	0.069493624	-2.736379933	0.022880753
B4galnt3|330406	-2.298762119	0.111316826	-2.862593939	0.100767863
6030446N20Rik|338363	-2.299689913	0.010252773	-2.582901086	0.001539078
Gmpr|66355	-2.303529838	0.037745975	-3.567566147	0.01203082
Setmar|74729	-2.305286428	0.020058625	-1.954270207	0.037299787
Bmpr2|12168	-2.305337918	0.101938088	-3.083674272	0.078319454
AU021092|239691	-2.305483375	0.057350455	-3.172195126	0.036628887
Ifi203|15950	-2.306026725	0.04570083	-2.34961222	0.00248706
Plekhn1|231002	-2.307429899	0.104565907	-4.547924695	0.029978483
Nsdhl|18194	-2.30950207	0.007003017	-2.040982448	0.016026504
Slc2a4|20528	-2.309737456	0.210498672	-4.30878	0.173296754
Lrrc49|102747	-2.313601113	0.118077465	-9.203466667	0.015035698
Adam32|353188	-2.313954577	0.14700288	-16.48145	0.019808349
Pram1|378460	-2.315024568	0.078638192	-1.639973789	0.152831797
Gm13695|545432	-2.316412378	0.102767807	-1.998898774	0.136819957
Akap12|83397	-2.322773165	0.054065207	-3.401457717	0.028422563
Zfp931|353208	-2.323431231	0.187460159	-4.56078	0.070574133

Magee1|107528	-2.326533015	0.206301278	1.085043178	0.458319617
Aoc2|237940	-2.32929831	0.071807351	-2.336813633	0.075249344
BC068157|73072	-2.330602604	0.05302311	-4.716460667	0.001683677
Atp6v0e2|76252	-2.33114795	0.087076513	-2.33114795	0.087076513
Prr5l|72446	-2.331723794	0.016698214	-2.331723794	0.016698214
Acsbg1|94180	-2.332142513	0.188677129	-7.267966667	0.093239512
Kcnb1|16500	-2.332860712	0.139146574	-2.332860712	0.139146574
Pwwp2b|101631	-2.333082812	0.003198591	-2.868934676	0.000410342
Epb4.9|13829	-2.334786788	0.047115714	-1.795753715	0.084104014
Praf2|54637	-2.335358464	0.002319478	-1.9518596	0.004556606
Htra1|56213	-2.335927869	0.09785167	-3.541561269	0.063400858
Glrp1|14659	-2.336891809	0.146206415	-9.296116667	0.038064488
Bmp2k|140780	-2.339989054	0.039105669	-2.863598716	0.013143338
Nr1d1|217166	-2.340078178	0.002919911	-3.200631657	6.64799E-05
Gna14|14675	-2.340912973	0.04840399	-3.744955172	0.009079915
Pilrb1|170741	-2.341334425	0.104809068	-1.635532836	0.219157359
Cyp2a5|13087	-2.341902722	0.010276344	-2.94543875	0.0058087
Tmem150b|330460	-2.34237074	0.061529096	-4.975806489	0.003162203
Lrp2bp|67620	-2.343530739	0.140521033	-1.359327497	0.314310072
Reep1|52250	-2.344121674	0.018943107	-2.616726404	0.007791565
Mtr|238505	-2.34449241	0.007606112	-1.682788568	0.012534845
Olfr1393|258463	-2.344960647	0.109557264	-8.933166667	0.013413549
Adap2|216991	-2.348915085	0.033034252	-1.88824872	0.058725951
A930005I04Rik|403174	-2.349957029	0.201173836	1.02215228	0.489259865
Xaf1|327959	-2.350170288	0.00301564	-2.069710316	0.002940241
Cdc14a|229776	-2.35105154	0.090543249	-4.101087113	0.043493317
Trim30d|209387	-2.351239807	0.121285403	-4.429154902	0.063880629
Nbeal1|269198	-2.355192665	0.099723943	-1.624263637	0.207971345
Irs3|16369	-2.356170096	0.088661358	-1.641023522	0.180096118
Csf1|12977	-2.356562165	0.005205422	-2.122203276	0.012120914
Gpr64|237175	-2.357703267	0.025337065	-3.99730858	0.003228156
2010002N04Rik|106878	-2.362514433	0.0775566	-1.664763609	0.149692992
A130022J15Rik|101351	-2.364690476	0.058930027	-2.547954885	0.012114491
Kcnq1ot1|63830	-2.36901595	0.0640468	-2.613756821	0.027203788
Tmem91|320208	-2.369262939	0.135524133	-7.38365	0.04272972
Bank1|242248	-2.371930395	0.074313325	-4.337411179	0.02766758
2810422O20Rik|69962	-2.374070307	0.004490514	-2.039998079	0.002968709
Ogn|18295	-2.379630401	0.152766303	-8.40118	0.035583449
Cenpm|66570	-2.38506934	0.000119107	-2.569822217	4.24492E-05
1700029J11Rik|76426	-2.38548	0.181585296	-6.95765	0.094047919
Serpine1|18787	-2.387113871	0.152626978	-1.279998358	0.35286604
Ttf2|74044	-2.393359629	0.055924349	-3.909889821	0.010532407
Ddx60|234311	-2.396313184	0.050310908	-2.886180831	0.01258836
Zfp709|236193	-2.398352187	0.078259377	-2.398352187	0.078259377
Efemp2|58859	-2.402004797	0.014259857	-1.979583226	0.01599175
Psat1|107272	-2.402130058	0.111106304	-9.555633333	0.016606094
Rab19|19331	-2.405064509	0.105933962	-9.162133333	0.014939816
Armcx6|278097	-2.408661177	0.04900158	-2.408661177	0.04900158
Calml4|75600	-2.408744957	0.137001188	-3.218533333	0.075309191
Prrt3|210673	-2.412520582	0.110120093	-9.596966667	0.016642218
Dapp1|26377	-2.414528582	0.036843468	-2.414528582	0.036843468
H2-T22|15039	-2.417270465	0.112836013	-1.874584596	0.116901717
Igsf10|242050	-2.41871422	0.068111909	-4.233000208	0.028188468

Zfp418|232854	-2.419048857	0.036910115	-4.542719147	0.005528153
Adc|242669	-2.41927444	0.047564015	-1.83391025	0.082981207
Rsph1|22092	-2.420333288	0.104403332	-9.2203	0.014927457
Tbc1d4|210789	-2.428263101	0.025789195	-2.428263101	0.025789195
Sncaip|67847	-2.430207431	0.169529955	-1.784568438	0.237848562
Awat2|245532	-2.431125758	0.017135631	-2.431125758	0.017135631
Lbx2|16815	-2.432135971	0.118898654	-4.18746398	0.074433504
Mfsd2b|432628	-2.434748567	0.11368475	-4.224792929	0.068876081
Stap1|56792	-2.436005904	0.21603094	-2.76078	0.173296754
Mphosph6|68533	-2.441450831	0.036320897	-4.333676353	0.00219841
Naip1|17940	-2.442153333	0.147460321	-7.63538	0.025880344
Ehf|13661	-2.444290267	0.128208988	-17.40978333	0.017225558
Itpka|228550	-2.444621122	0.075681408	-1.306117158	0.083688436
Chmp4c|66371	-2.446414369	0.15887028	-2.230407828	0.191047562
Hs3st6|328779	-2.447205208	0.031366957	-1.970747526	0.056708232
Gm14137|623781	-2.453769305	0.120620667	-3.41653147	0.056204377
Acot3|171281	-2.460725044	0.000123639	-2.460725044	0.000123639
Cyb5d1|327951	-2.464340245	0.039804388	-2.464340245	0.039804388
Hemgn|93966	-2.467316667	0.170446566	1	#DIV/0!
Il9r|16199	-2.467316667	0.170446566	1	#DIV/0!
Gsg1|14840	-2.467316667	0.170446566	1	#DIV/0!
Phyhip|105653	-2.467316667	0.170446566	1	#DIV/0!
4930503E24Rik|75030	-2.467316667	0.170446566	1	#DIV/0!
Wbscr17|212996	-2.467316667	0.170446566	1	#DIV/0!
P2rx5|94045	-2.467316667	0.170446566	1	#DIV/0!
Pla2g3|237625	-2.467316667	0.170446566	1	#DIV/0!
Aox3l1|213043	-2.467316667	0.170446566	1	#DIV/0!
2900060B14Rik|68204	-2.467316667	0.170446566	1	#DIV/0!
AY358078|278676	-2.467316667	0.170446566	1	#DIV/0!
Gpr81|243270	-2.467316667	0.170446566	1	#DIV/0!
Cnpy1|269637	-2.467316667	0.170446566	1	#DIV/0!
E030011O05Rik|319859	-2.467316667	0.170446566	1	#DIV/0!
Cryaa|12954	-2.467316667	0.170446566	1	#DIV/0!
Kcnh4|380728	-2.467316667	0.170446566	1	#DIV/0!
Gm5741|436049	-2.467316667	0.170446566	1	#DIV/0!
Clic6|209195	-2.467316667	0.170446566	1	#DIV/0!
Upk1a|109637	-2.467316667	0.170446566	1	#DIV/0!
Chst10|98388	-2.467316667	0.170446566	1	#DIV/0!
4931408C20Rik|210940	-2.467316667	0.170446566	1	#DIV/0!
Got1l1|76615	-2.467316667	0.170446566	1	#DIV/0!
E330020D12Rik|626058	-2.467316667	0.170446566	1	#DIV/0!
Kcnh3|16512	-2.467316667	0.170446566	1	#DIV/0!
Asb14|142687	-2.467316667	0.170446566	1	#DIV/0!
Krtap3-3|66380	-2.467316667	0.170446566	1	#DIV/0!
Glra2|237213	-2.467316667	0.170446566	1	#DIV/0!
1600029I14Rik|69797	-2.467316667	0.170446566	1	#DIV/0!
Asf1b|66929	-2.467316667	0.170446566	1	#DIV/0!
Rhebl1|69159	-2.467316667	0.170446566	1	#DIV/0!
Tmc3|233424	-2.467316667	0.170446566	1	#DIV/0!
Gpr115|78249	-2.467316667	0.170446566	1	#DIV/0!
9930013L23Rik|80982	-2.467316667	0.170446566	1	#DIV/0!
9030612E09Rik|74530	-2.467316667	0.170446566	1	#DIV/0!
Bbs12|241950	-2.467316667	0.170446566	1	#DIV/0!

Alox12e|11685	-2.467316667	0.170446566	1	#DIV/0!
Pak7|241656	-2.467316667	0.170446566	1	#DIV/0!
Cd69|12515	-2.467316667	0.170446566	1	#DIV/0!
Esyt3|272636	-2.467316667	0.170446566	1	#DIV/0!
Ebf3|13593	-2.467316667	0.170446566	1	#DIV/0!
Dkk2|56811	-2.467316667	0.170446566	1	#DIV/0!
Wfdc12|192200	-2.467316667	0.170446566	1	#DIV/0!
Klk10|69540	-2.467316667	0.170446566	1	#DIV/0!
Nova1|664883	-2.467316667	0.170446566	1	#DIV/0!
AI427809|381524	-2.467316667	0.170446566	1	#DIV/0!
1700019D03Rik|67080	-2.467316667	0.170446566	1	#DIV/0!
Mdga2|320772	-2.467316667	0.170446566	1	#DIV/0!
Ankrd53|75305	-2.467316667	0.170446566	1	#DIV/0!
Casc1|320662	-2.467316667	0.170446566	1	#DIV/0!
9130008F23Rik|71583	-2.467316667	0.170446566	1	#DIV/0!
BY080835|434436	-2.467316667	0.170446566	1	#DIV/0!
Hrh2|15466	-2.467316667	0.170446566	1	#DIV/0!
Aqp7|11832	-2.467316667	0.170446566	1	#DIV/0!
Bend5|67621	-2.467316667	0.170446566	1	#DIV/0!
Txlnb|378431	-2.467316667	0.170446566	1	#DIV/0!
Butr1|192194	-2.467316667	0.170446566	1	#DIV/0!
9930111J21Rik2|245240	-2.467316667	0.170446566	1	#DIV/0!
Ercc6l|236930	-2.467316667	0.170446566	1	#DIV/0!
C030034L19Rik|320088	-2.467316667	0.170446566	1	#DIV/0!
Klre1|243655	-2.467316667	0.170446566	1	#DIV/0!
Nxph3|104079	-2.467316667	0.170446566	1	#DIV/0!
Klhl30|70788	-2.467316667	0.170446566	1	#DIV/0!
Ffar2|233079	-2.467316667	0.170446566	1	#DIV/0!
Rnf151|67504	-2.467316667	0.170446566	1	#DIV/0!
2900011O08Rik|67254	-2.467316667	0.170446566	1	#DIV/0!
Gm5434|432649	-2.467316667	0.170446566	1	#DIV/0!
Kcnrg|328424	-2.467316667	0.170446566	1	#DIV/0!
Asxl3|211961	-2.467316667	0.170446566	1	#DIV/0!
Tmod4|50874	-2.467316667	0.170446566	1	#DIV/0!
Akap4|11643	-2.467316667	0.170446566	1	#DIV/0!
4930519F09Rik|114676	-2.467316667	0.170446566	1	#DIV/0!
Ankrd34b|218440	-2.467316667	0.170446566	1	#DIV/0!
Nkpd1|69547	-2.467316667	0.170446566	1	#DIV/0!
4930415O20Rik|73863	-2.467316667	0.170446566	1	#DIV/0!
Prss12|19142	-2.467316667	0.170446566	1	#DIV/0!
Xlr3c|22446	-2.467316667	0.170446566	1	#DIV/0!
Vwde|232585	-2.467316667	0.170446566	1	#DIV/0!
Mia1|12587	-2.467316667	0.170446566	1	#DIV/0!
Asb15|78910	-2.467316667	0.170446566	1	#DIV/0!
Mpl|17480	-2.467316667	0.170446566	1	#DIV/0!
Krt17|16667	-2.467316667	0.170446566	1	#DIV/0!
Asprv1|67855	-2.467316667	0.170446566	1	#DIV/0!
Slc1a7|242607	-2.467316667	0.170446566	1	#DIV/0!
Adora3|11542	-2.467316667	0.170446566	1	#DIV/0!
Stk32b|64293	-2.467316667	0.170446566	1	#DIV/0!
Fam181a|544888	-2.467316667	0.170446566	1	#DIV/0!
Dnahc10|56087	-2.467316667	0.170446566	1	#DIV/0!
Rims4|241770	-2.467316667	0.170446566	1	#DIV/0!

Ltk|17005	-2.467316667	0.170446566	1	#DIV/0!
Zdhhc2|70546	-2.469109987	0.128219642	-7.694816667	0.043805315
Tmem132a|98170	-2.47305614	0.004034079	-2.47305614	0.004034079
Ifit2|15958	-2.475630438	0.040072871	-2.475630438	0.040072871
Rest|19712	-2.479656116	0.168970511	-2.089837133	0.111438353
Sema3b|20347	-2.481874951	0.116927513	-1.56427143	0.249669356
Nol3|78688	-2.48611173	0.006129612	-2.262502597	0.013511875
Slc4a3|20536	-2.487720157	0.048737542	-1.97204547	0.098007258
BC048644|407789	-2.492585253	0.033972385	-1.614372315	0.09438735
Skap1|78473	-2.499185393	0.089617817	-1.794873935	0.185152809
Serpina3f|238393	-2.499977956	0.004690186	-2.846016578	0.0002449
Prkag3|241113	-2.5	0.170446566	1	#DIV/0!
Als2cr12|108812	-2.5	0.170446566	1	#DIV/0!
Rapsn|19400	-2.5	0.170446566	1	#DIV/0!
Zfp389|100038371	-2.5	0.170446566	1	#DIV/0!
Wasf3|245880	-2.5	0.170446566	1	#DIV/0!
Gm13152|195531	-2.5	0.170446566	1	#DIV/0!
Slc1a6|20513	-2.5	0.170446566	1	#DIV/0!
Kcnq4|60613	-2.5	0.170446566	1	#DIV/0!
A930013F10Rik|68074	-2.5	0.170446566	1	#DIV/0!
Zfp677|210503	-2.5	0.170446566	1	#DIV/0!
9130024F11Rik|329160	-2.5	0.170446566	1	#DIV/0!
Lrch2|210297	-2.5	0.170446566	1	#DIV/0!
Sult4a1|29859	-2.5	0.170446566	1	#DIV/0!
Ankrd61|66729	-2.5	0.170446566	1	#DIV/0!
Diras2|68203	-2.5	0.170446566	1	#DIV/0!
Cdh6|12563	-2.5	0.170446566	1	#DIV/0!
4933427E11Rik|66769	-2.5	0.170446566	1	#DIV/0!
Syn2|20965	-2.5	0.170446566	1	#DIV/0!
Gm410|242037	-2.5	0.170446566	1	#DIV/0!
Vmn2r57|269902	-2.5	0.170446566	1	#DIV/0!
9230116N13Rik|320054	-2.5	0.170446566	1	#DIV/0!
Begain|380785	-2.5	0.170446566	1	#DIV/0!
1700019N12Rik|67077	-2.5	0.170446566	1	#DIV/0!
Zfp202|80902	-2.5	0.170446566	1	#DIV/0!
Mmp25|240047	-2.5	0.170446566	1	#DIV/0!
Pdx1|18609	-2.5	0.170446566	1	#DIV/0!
Zfp811|240063	-2.5	0.170446566	1	#DIV/0!
Gm1564|268491	-2.5	0.170446566	1	#DIV/0!
Gnb3|14695	-2.5	0.170446566	1	#DIV/0!
Tmeff2|56363	-2.5	0.170446566	1	#DIV/0!
Dnahc7b|227058	-2.5	0.170446566	1	#DIV/0!
Phf16|382207	-2.5	0.170446566	1	#DIV/0!
Cabp1|29867	-2.5	0.170446566	1	#DIV/0!
Accn1|11418	-2.5	0.170446566	1	#DIV/0!
Nrcam|319504	-2.5	0.170446566	1	#DIV/0!
Gbgt1|227671	-2.5	0.170446566	1	#DIV/0!
D5Ertd605e|100039805	-2.5	0.170446566	1	#DIV/0!
Tecta|21683	-2.5	0.170446566	1	#DIV/0!
Zfp72|238722	-2.5	0.170446566	1	#DIV/0!
Ptprq|237523	-2.5	0.170446566	1	#DIV/0!
Myadml2|68515	-2.5	0.170446566	1	#DIV/0!
Zdhhc15|108672	-2.5	0.170446566	1	#DIV/0!

Olfr1030|258581	-2.5	0.170446566	1	#DIV/0!
Smok3b|622474	-2.5	0.170446566	1	#DIV/0!
Trank1|320429	-2.5	0.170446566	1	#DIV/0!
6330406I15Rik|70717	-2.5	0.170446566	1	#DIV/0!
Drp2|13497	-2.5	0.170446566	1	#DIV/0!
LOC100038947|100038947	-2.5	0.170446566	1	#DIV/0!
Clvs1|74438	-2.5	0.170446566	1	#DIV/0!
Oit1|18300	-2.5	0.170446566	1	#DIV/0!
Bcas1|76960	-2.5	0.170446566	1	#DIV/0!
Aff2|14266	-2.5	0.170446566	1	#DIV/0!
Tpsab1|17230	-2.5	0.170446566	1	#DIV/0!
Gm13051|626316	-2.5	0.170446566	1	#DIV/0!
Syce3|75459	-2.5	0.170446566	1	#DIV/0!
9330182L06Rik|231014	-2.5	0.170446566	1	#DIV/0!
Cenpi|102920	-2.5	0.170446566	1	#DIV/0!
Nrg2|100042150	-2.5	0.170446566	1	#DIV/0!
Pcdhb7|93878	-2.5	0.170446566	1	#DIV/0!
Umodl1|52020	-2.5	0.170446566	1	#DIV/0!
Col24a1|71355	-2.5	0.170446566	1	#DIV/0!
Ism1|319909	-2.5	0.170446566	1	#DIV/0!
Trpm8|171382	-2.5	0.170446566	1	#DIV/0!
Gm14393|664987	-2.5	0.170446566	1	#DIV/0!
Foxh1|14106	-2.5	0.170446566	1	#DIV/0!
Rad51ap1|19362	-2.5	0.170446566	1	#DIV/0!
Cnga4|233649	-2.5	0.170446566	1	#DIV/0!
Olfml2a|241327	-2.5	0.170446566	1	#DIV/0!
Gpr174|213439	-2.5	0.170446566	1	#DIV/0!
Glis3|226075	-2.5	0.170446566	1	#DIV/0!
Oxct2b|353371	-2.5	0.170446566	1	#DIV/0!
Krt222|268481	-2.5	0.170446566	1	#DIV/0!
Gfi1b|14582	-2.5	0.170446566	1	#DIV/0!
Mall|228576	-2.5	0.170446566	1	#DIV/0!
Trem3|58218	-2.5	0.170446566	1	#DIV/0!
Atp6v0a4|140494	-2.5	0.170446566	1	#DIV/0!
Slc29a4|243328	-2.5	0.170446566	1	#DIV/0!
Kcnq5|226922	-2.5	0.170446566	1	#DIV/0!
Nhlrc4|621239	-2.5	0.170446566	1	#DIV/0!
Cml3|93674	-2.5	0.170446566	1	#DIV/0!
Slamf1|27218	-2.5	0.170446566	1	#DIV/0!
D3Bwg0562e|229791	-2.5	0.170446566	1	#DIV/0!
Nlrc3|268857	-2.5	0.170446566	1	#DIV/0!
Gpr44|14764	-2.5	0.170446566	1	#DIV/0!
Gm973|381260	-2.5	0.170446566	1	#DIV/0!
Cd101|630146	-2.5	0.170446566	1	#DIV/0!
Scg2|20254	-2.5	0.170446566	1	#DIV/0!
Grifin|77998	-2.5	0.170446566	1	#DIV/0!
Ly6i|57248	-2.5	0.170446566	1	#DIV/0!
Cdh16|12556	-2.5	0.170446566	1	#DIV/0!
Tmem52|69671	-2.5	0.170446566	1	#DIV/0!
Trem1|58217	-2.5	0.170446566	1	#DIV/0!
Zim1|22776	-2.5	0.170446566	1	#DIV/0!
Clec2i|93675	-2.5	0.170446566	1	#DIV/0!
Abca15|320631	-2.5	0.170446566	1	#DIV/0!

Cd164l2|69655	-2.5	0.170446566	1	#DIV/0!
Mybl1|17864	-2.5	0.170446566	1	#DIV/0!
Trpv3|246788	-2.5	0.170446566	1	#DIV/0!
Rnase13|497071	-2.5	0.170446566	1	#DIV/0!
Efna3|13638	-2.5	0.170446566	1	#DIV/0!
Zfp541|666528	-2.5	0.170446566	1	#DIV/0!
Itgb2l|16415	-2.5	0.170446566	1	#DIV/0!
Raver2|242570	-2.5	0.170446566	1	#DIV/0!
Efcab9|69306	-2.5	0.170446566	1	#DIV/0!
Il5ra|16192	-2.5	0.170446566	1	#DIV/0!
Slfn9|237886	-2.5	0.170446566	1	#DIV/0!
Spin4|270624	-2.5	0.170446566	1	#DIV/0!
Gm8234|666680	-2.5	0.170446566	1	#DIV/0!
Cecr6|94047	-2.5	0.170446566	1	#DIV/0!
Egfem1|75740	-2.502443493	0.131364174	-4.308514044	0.089804908
Notch3|18131	-2.505776625	0.09049602	-2.24281773	0.100958579
6030408B16Rik|77717	-2.516833333	0.170446566	1	#DIV/0!
Gm12185|620913	-2.516833333	0.170446566	1	#DIV/0!
Gjb6|14623	-2.516833333	0.170446566	1	#DIV/0!
Rbm24|666794	-2.516833333	0.170446566	1	#DIV/0!
Gm14047|329514	-2.516833333	0.170446566	1	#DIV/0!
Pkdrej|18766	-2.516833333	0.170446566	1	#DIV/0!
Smtnl1|68678	-2.516833333	0.170446566	1	#DIV/0!
Myoz2|59006	-2.516833333	0.170446566	1	#DIV/0!
Mas1|17171	-2.516833333	0.170446566	1	#DIV/0!
Cngb3|30952	-2.516833333	0.170446566	1	#DIV/0!
Trim6|94088	-2.516833333	0.170446566	1	#DIV/0!
Nobox|18291	-2.516833333	0.170446566	1	#DIV/0!
Nos2|18126	-2.516833333	0.170446566	1	#DIV/0!
Slc38a11|320106	-2.516833333	0.170446566	1	#DIV/0!
Pla2g4c|232889	-2.516833333	0.170446566	1	#DIV/0!
Gm7325|653016	-2.516833333	0.170446566	1	#DIV/0!
Pcdhb15|93886	-2.516833333	0.170446566	1	#DIV/0!
Ccr10|12777	-2.516833333	0.170446566	1	#DIV/0!
Zfp114|232966	-2.516833333	0.170446566	1	#DIV/0!
Shd|20420	-2.516833333	0.170446566	1	#DIV/0!
Vaultrc5|378472	-2.516833333	0.170446566	1	#DIV/0!
Dusp26|66959	-2.516833333	0.170446566	1	#DIV/0!
Gm11978|100041311	-2.516833333	0.170446566	1	#DIV/0!
Klhl29|208439	-2.516833333	0.170446566	1	#DIV/0!
Clspn|269582	-2.516833333	0.170446566	1	#DIV/0!
Kcnq2|16536	-2.516833333	0.170446566	1	#DIV/0!
G6pd2|14380	-2.516833333	0.170446566	1	#DIV/0!
D1Pas1|110957	-2.516833333	0.170446566	1	#DIV/0!
R3hdml|100043899	-2.516833333	0.170446566	1	#DIV/0!
Sftpc|20389	-2.516833333	0.170446566	1	#DIV/0!
Galr3|14429	-2.516833333	0.170446566	1	#DIV/0!
Bend6|320705	-2.516833333	0.170446566	1	#DIV/0!
40979|211147	-2.516833333	0.170446566	1	#DIV/0!
Rasef|242505	-2.516833333	0.170446566	1	#DIV/0!
Gm5151|381582	-2.516833333	0.170446566	1	#DIV/0!
Gm5544|433632	-2.516833333	0.170446566	1	#DIV/0!
Lrrc4b|272381	-2.516833333	0.170446566	1	#DIV/0!

2010002M12Rik|112419	-2.516833333	0.170446566	1	#DIV/0!
Zfp599|235048	-2.516833333	0.170446566	1	#DIV/0!
Trim31|224762	-2.516833333	0.170446566	1	#DIV/0!
Kcnc4|99738	-2.516833333	0.170446566	1	#DIV/0!
Kcnv2|240595	-2.516833333	0.170446566	1	#DIV/0!
Trim60|234329	-2.516833333	0.170446566	1	#DIV/0!
9130230L23Rik|231253	-2.516833333	0.170446566	1	#DIV/0!
Macc1|238455	-2.516833333	0.170446566	1	#DIV/0!
Zfp934|77117	-2.516833333	0.170446566	1	#DIV/0!
Kcnj13|100040591	-2.516833333	0.170446566	1	#DIV/0!
Kcnma1|16531	-2.516833333	0.170446566	1	#DIV/0!
Olfr1442|258692	-2.516833333	0.170446566	1	#DIV/0!
Kif4|16571	-2.516833333	0.170446566	1	#DIV/0!
Vil1|22349	-2.516833333	0.170446566	1	#DIV/0!
Gm11110|100169874	-2.516833333	0.170446566	1	#DIV/0!
1700025E21Rik|75647	-2.516833333	0.170446566	1	#DIV/0!
Ptx3|19288	-2.516833333	0.170446566	1	#DIV/0!
Samd3|268288	-2.516833333	0.170446566	1	#DIV/0!
Npas1|18142	-2.516833333	0.170446566	1	#DIV/0!
Gm600|239151	-2.516833333	0.170446566	1	#DIV/0!
Serpinb12|71869	-2.516833333	0.170446566	1	#DIV/0!
Kcnk2|16526	-2.516833333	0.170446566	1	#DIV/0!
B3gnt7|227327	-2.516833333	0.170446566	1	#DIV/0!
Gucy2g|73707	-2.516833333	0.170446566	1	#DIV/0!
Gxylt2|232313	-2.516833333	0.170446566	1	#DIV/0!
Rgr|57811	-2.516833333	0.170446566	1	#DIV/0!
Murc|68016	-2.516833333	0.170446566	1	#DIV/0!
Hist1h1t|107970	-2.516833333	0.170446566	1	#DIV/0!
Prdm5|70779	-2.516833333	0.170446566	1	#DIV/0!
Cnrip1|380686	-2.527181018	0.03696948	-2.124495507	0.07625478
Gbp10|626578	-2.528836719	0.03578146	-3.909115828	0.011583368
Slc15a5|277898	-2.529380465	0.15994295	-5.725	0.078582895
Lamc2|16782	-2.531218987	0.137940916	-2.539690909	0.13105537
Fancg|60534	-2.531836058	0.008496618	-2.531836058	0.008496618
Pcdhgb6|93703	-2.536217719	0.210730115	-5.83124	0.173296754
Myst4|54169	-2.536781208	0.078102959	-2.016032707	0.022410628
Sucnr1|84112	-2.539619336	0.00095107	-2.761010386	0.000216073
G530011O06Rik|654820	-2.542815371	0.047943582	-2.542815371	0.047943582
Mfap1b|100034361	-2.543796463	0.006015395	-2.292727349	0.012750733
Bsn|12217	-2.545087754	0.126459335	-10.12431667	0.037434332
Upk3b|100647	-2.545087754	0.126459335	-10.12431667	0.037434332
0610008F07Rik|68314	-2.546862257	0.00020714	-2.958138477	3.31062E-05
Xirp1|22437	-2.546865962	0.158109741	-12.47548333	0.059683025
Pcp2|18545	-2.55	0.170446566	1	#DIV/0!
Pdzd9|67983	-2.5505	0.170446566	1	#DIV/0!
Mpp7|75739	-2.553549245	0.046124068	-3.045861348	0.036328653
Hectd2|226098	-2.554213629	0.08844341	-4.866235003	0.025395598
6430550D23Rik|320095	-2.554728317	0.230351658	-2.8202	0.173296754
Zfp280d|235469	-2.558460373	0.037356945	-2.558460373	0.037356945
Rgn|19733	-2.560471537	0.001508836	-2.42152425	0.000426909
Phf21b|271305	-2.561926717	0.096005858	-2.561926717	0.096005858
Cldn6|54419	-2.565907029	0.081574194	-2.565907029	0.081574194
Sez6l2|233878	-2.567250327	0.091557526	-17.42063333	0.005587537

1700096K18Rik|73571	-2.568770183	0.010680099	-2.568770183	0.010680099
Per3|18628	-2.570449129	0.038249521	-2.570449129	0.038249521
Tlr9|81897	-2.571428571	0.257082261	1	#DIV/0!
Gm1943|384864	-2.576055411	0.025693353	-2.923340273	0.013602722
9030619P08Rik|105892	-2.577277635	0.002598479	-3.119355837	0.001854358
Prdm1|12142	-2.577793333	0.112456322	-9.46676	0.027509545
Bcl2a1d|12047	-2.587841208	0.248181938	1	#DIV/0!
5730507C01Rik|236366	-2.588996099	0.151556385	-6.10878	0.08277987
Pole2|18974	-2.591485714	0.20181887	-2.8202	0.173296754
Esco1|77805	-2.593247862	0.057127015	-4.631172579	0.024524315
Efnb2|13642	-2.594310543	0.061594535	-2.594310543	0.061594535
Sema5b|20357	-2.597906637	0.004364805	-1.879038695	0.008105304
Cyfip2|76884	-2.608781903	0.014882919	-2.608781903	0.014882919
Pdk3|236900	-2.614511541	0.056927323	-12.28058	2.26551E-05
Zfp37|22696	-2.618173373	0.103527824	-1.737055864	0.216907909
Emr4|52614	-2.618296995	0.05105169	-3.906689157	0.015112091
A230056P14Rik|320845	-2.618363103	0.058661092	-2.618363103	0.058661092
Eya2|14049	-2.619116051	0.143606318	-6.6202	0.087728953
B230206H07Rik|320871	-2.621003302	0.064746709	-1.831394606	0.114357007
Zfp354b|27274	-2.625658233	0.026654261	-2.059120485	0.038400059
2010110P09Rik|70261	-2.627566667	0.152504417	-7.7654	0.073181307
Samd10|229011	-2.635895248	0.013629555	-4.233935731	0.002958045
Lama4|16775	-2.636537198	0.045468671	-2.636537198	0.045468671
Cd46|17221	-2.638222857	0.112593519	-7.694816667	0.043805315
Rap1gap2|380711	-2.639155458	0.061627826	-1.925207129	0.119072822
Cep192|70799	-2.640479326	0.117410165	-10.05895	0.03738237
Bcl11a|14025	-2.641188571	0.113022404	-7.703466667	0.044331703
Itga6|16403	-2.642440423	0.122313867	-5.858383838	0.067890355
Msh4|55993	-2.643994286	0.111962198	-7.71165	0.043574427
Rnf208|68846	-2.650796667	0.167585833	-6.38098	0.020002315
Ribc1|66611	-2.650977817	0.00080618	-2.387067591	0.000789877
Utp14b|195434	-2.655639413	0.019365364	-5.246993939	0.000559844
Tmem67|329795	-2.655715333	0.008034698	-2.655715333	0.008034698
Trim35|66854	-2.660153733	0.0003041	-2.192286472	0.000468306
Rgs9|19739	-2.661868719	0.028824897	-2.661868719	0.028824897
Egf|13645	-2.662008741	0.079963396	-10.14096667	0.012545915
Plk4|20873	-2.662008741	0.079963396	-10.14096667	0.012545915
Cog5|238123	-2.665294745	0.053395091	-3.718251689	0.020983218
Rdh19|216453	-2.666666667	0.170446566	1	#DIV/0!
Sync|68828	-2.66835	0.170446566	1	#DIV/0!
Abca4|11304	-2.670588132	0.078976029	-10.17365	0.012340756
Chrd|12667	-2.678960877	2.93338E-05	-2.770812985	1.31945E-05
Ifit1|15957	-2.680997029	0.010865034	-2.19230787	0.009733054
N4bp2|333789	-2.687976019	0.115153677	-3.09281768	0.040766669
Nfkbiz|80859	-2.692271913	0.052923582	-2.692271913	0.052923582
Kctd12b|207474	-2.698586495	0.029741639	-4.127846389	0.010766957
Il17re|57890	-2.712120312	0.121612504	-2.752539394	0.103543379
Ildr1|106347	-2.712537773	0.120548221	-9.57578	0.028800441
Syt3|20981	-2.714306666	0.011498167	-2.714306666	0.011498167
BC021767|545551	-2.714478119	0.131854361	-7.47616	0.020098075
AI662270|100043636	-2.724556519	0.051482869	-1.930621719	0.077234095
C230091D08Rik|330544	-2.72695547	0.067245687	-1.612892807	0.195406671
Eci3|69123	-2.728294341	0.029186277	-3.248451516	0.004742063

Gm4925|237433	-2.730165714	0.065356157	-7.962983333	0.01361011
Trim56|384309	-2.734803505	0.11055963	-8.345151594	4.25795E-05
Cd300e|217306	-2.740450435	0.009947877	-2.306316875	0.022039297
Zbtb1|268564	-2.748500538	0.092956891	-7.166811181	0.037803466
Aqp8|11833	-2.752256813	0.002192635	-3.423380903	0.001474361
Hist1h2bp|319188	-2.752533333	0.170446566	1	#DIV/0!
Robo3|19649	-2.753859245	0.11953938	-1.515644735	0.247307335
Mob1a|232157	-2.757831791	0.131852354	-2.709600084	0.143484141
Evpl|14027	-2.7672069	0.038537203	-2.7672069	0.038537203
Doc2g|60425	-2.771116357	0.000783402	-2.771116357	0.000783402
Fgd4|224014	-2.772389393	0.017962647	-3.308384008	0.001167527
Gbp6|100702	-2.78008262	0.002422072	-2.78008262	0.002422072
4930473A06Rik|320226	-2.780558644	0.007043617	-2.700120714	0.001296318
Agbl3|76223	-2.781080499	0.039725995	-17.77034	4.84841E-08
Rhof|23912	-2.78941923	0.061747114	-3.21443927	0.045393606
Gpr97|54672	-2.791210316	0.000218643	-3.055010715	9.38918E-06
4930563E22Rik|75304	-2.793805714	0.197880848	-2.76078	0.173296754
Acn9|71238	-2.7978382	0.095410268	-1.744950503	0.190023771
Bbs7|71492	-2.801981742	0.019135737	-5.122379282	0.000222342
Tssk1|22114	-2.802184904	0.05342064	-5.553198181	0.022031674
Hey2|15214	-2.805011429	0.196537171	-2.8	0.173296754
Mmgt2|216829	-2.822192114	0.07848064	-5.828282828	0.040150201
Tnnt2|21956	-2.822249642	0.06595673	-1.644041308	0.191781906
Panx1|55991	-2.823281478	0.099024577	-7.5948	0.026105266
Sorcs2|81840	-2.825645714	0.064479698	-8.241466667	0.016004313
Phactr1|218194	-2.830405271	0.09861442	-2.830405271	0.09861442
3110001I22Rik|66598	-2.836736806	0.072921648	-3.548325926	0.064555885
Zfp715|69930	-2.844455721	0.034220375	-6.719374673	0.005872542
Nap1l3|54561	-2.849885714	0.167543931	-3.9746	0.173296754
Fndc5|384061	-2.856920802	0.137490632	-6.6202	0.087728953
Ccdc62|208908	-2.861298497	0.000884845	-2.861298497	0.000884845
Tnfsf13|69583	-2.862369223	0.006317543	-3.252363091	0.001005421
1700009P17Rik|75472	-2.865449172	0.013811933	-2.787485769	0.012911592
Fitm1|68680	-2.866000982	0.000352809	-2.866000982	0.000352809
Synm|233335	-2.86694191	0.162020882	-4.72156	0.173296754
Ovol2|107586	-2.8672	0.167172108	-3.9746	0.173296754
Snn|20621	-2.8674335	0.035011336	-2.217731256	0.062819965
Peli3|240518	-2.878731844	0.049803609	-3.793878788	0.02520964
Zfp647|239546	-2.882158031	0.123717515	-15.37578	0.009220078
Adam1a|280668	-2.887895458	0.115440739	-7.725	0.072984714
Etv4|18612	-2.890799311	0.143439081	-4.56078	0.070574133
Scgb1c1|338417	-2.913811407	0.164828986	-6.60118	0.089542423
Gm7120|633640	-2.919213471	0.089373788	-2.512088614	0.062882096
Bcl9|77578	-2.935144932	0.106440103	-5.148996941	0.079002833
Pmel|20431	-2.941622706	0.04032891	-3.935468495	0.028426471
Esr1|13982	-2.94256999	0.069904551	-2.463921898	0.109253095
Apc2|23805	-2.95959	0.083999635	-10.74	0.021597467
Scd1|20249	-2.960754856	0.01154218	-2.396171756	0.011909639
Gm6787|627782	-2.973822857	0.087331737	-8.67365	0.037277465
Alpk1|71481	-3.002433267	0.104420224	-3.221509091	0.127882662
Stambpl1|76630	-3.003644019	0.014521658	-3.584015173	0.000861442
Card11|108723	-3.011931429	0.097726719	-8.7848	0.047326766
Emid1|140703	-3.015605654	0.107626729	-7.725	0.072984714

Zfp960|449000	-3.021066127	0.092970709	-6.516672795	0.056561398
Klhdc7a|242721	-3.023212152	0.006046629	-3.378725066	0.003784669
Ces4a|234677	-3.02339	0.084122769	-15.11695	0.022871287
Thbs3|21827	-3.024368748	0.007240765	-5.539806891	0.000908083
Bhlhb9|70237	-3.041049426	0.061088595	-2.097710659	0.110687616
Ppbp|57349	-3.042479648	0.101056723	-7.50598	0.025961531
Lat2|56743	-3.053568818	0.016409234	-4.702485691	0.002184739
Relt|320100	-3.05821102	0.029864917	-3.05821102	0.029864917
Stard4|170459	-3.088166692	0.060434643	-2.746903283	0.090545155
Haus6|230376	-3.088794286	0.13254585	-4.56078	0.070574133
Tnfrsf13c|72049	-3.090178564	0.04395869	-2.317833786	0.082974413
Espl1|105988	-3.09176	0.084088104	-9.017633333	0.038462883
Pbx4|80720	-3.10144075	0.142255205	-4.58098	0.070596339
Scd2|20250	-3.111908385	0.047036312	-2.190211442	0.072259439
Zfp951|626391	-3.113880987	0.033701259	-14.66436	7.47236E-05
D830015G02Rik|791403	-3.114033333	0.170446566	1	#DIV/0!
Tmem71|213068	-3.114286631	0.085842262	-9.705466667	0.038460173
Spred3|101809	-3.115729235	0.045748446	-3.837684848	0.005488291
Pabpc4l|241989	-3.127549656	0.085108819	-9.7468	0.038269251
AU041133|216177	-3.138189786	0.125558494	-6.8996	0.070566833
Smpdl3b|100340	-3.139031799	0.156389575	-4.72156	0.173296754
Rdh18-ps|380674	-3.147265034	0.082845795	-10.21234137	0.035831364
Prph|19132	-3.155474286	0.049773787	-9.203466667	0.015035698
Gpr137c|70713	-3.155904696	0.122673308	-5.813836158	0.097228399
Cyp2g1|13108	-3.158479679	0.013364058	-9.593463858	0.000281329
Mup14|100039116	-3.158520742	0.07536837	-6.071364267	0.049963663
Limch1|77569	-3.159327436	0.084145838	-9.845833333	0.038439314
Tmem194|210035	-3.159399096	0.139696169	-2.513733224	0.155741267
Palld|72333	-3.169824869	0.053193431	-2.248474625	0.096856049
Ppp1r3f|54646	-3.173861127	0.100273722	-12.62556667	0.045659527
Zkscan4|544922	-3.179381047	0.043384447	-31.68491667	0.00178628
Sgsm1|52850	-3.192394279	0.0219653	-6.44726988	0.001026835
Pilrb2|545812	-3.195205632	0.001615307	-3.195205632	0.001615307
Zfhx2as|432855	-3.20175	0.170446566	1	#DIV/0!
Zfp101|22643	-3.216769744	0.086978391	-5.38528942	0.0695168
Cdca2|108912	-3.223822857	0.079082075	-9.402816667	0.038132641
Tmprss4|214523	-3.227712235	0.080462464	-10.05895	0.03738237
Arhgap44|216831	-3.231255985	0.055529045	-3.231255985	0.055529045
Gprasp1|67298	-3.23840862	0.016158771	-5.627286767	0.007118792
Ap1s2|108012	-3.254033063	0.035159938	-5.602542951	0.021803184
Ldb3|24131	-3.254088541	0.107719325	-6.10878	0.08277987
Fzd2|57265	-3.266596559	0.143453667	-6.06956	0.083596805
Sc4mol|66234	-3.270762326	0.016188812	-2.456795672	0.005770179
Car1|12346	-3.273271337	0.012776123	-2.561359987	0.009531733
Icos|54167	-3.292534398	0.03510613	-6.031012176	0.020023345
Ankle1|234396	-3.293091577	0.037546818	-4.970946855	0.017686137
Idi1|319554	-3.310759015	0.040798916	-2.456391624	0.00829248
2810432L12Rik|67063	-3.316165236	0.087242887	-11.48696	0.021283245
Lrrc50|68270	-3.333333333	0.170446566	1	#DIV/0!
Elf3|13710	-3.33584469	0.018662417	-5.242375103	0.003093408
Sectm1a|209588	-3.339131429	0.076030926	-9.739133333	0.038536342
Col4a4|12829	-3.342682012	0.095567558	-13.29713333	0.046995664
Esrrb|26380	-3.348171299	0.042844032	-4.626419566	0.038446498

Kitl|17311	-3.352965714	0.105022556	-5.73538	0.079831528
Fam132b|227358	-3.359366667	0.170446566	1	#DIV/0!
Gfra2|14586	-3.362799178	0.002617829	-3.714502745	0.000361244
Tmem14a|75712	-3.378960809	0.000759383	-3.761368131	7.07692E-05
Aim2|383619	-3.390688766	0.013050217	-4.039360589	0.001076418
Lcn2|16819	-3.412952112	0.026462407	-2.215178381	0.039449461
Npff|54615	-3.424629252	0.06102326	-8.459633333	0.033016045
Zfp398|272347	-3.426304649	0.019447565	-2.685832395	0.029152791
Pde6c|110855	-3.42915974	0.006605334	-3.42915974	0.006605334
D17H6S56E-3|27762	-3.430855183	0.06333734	-9.33468	0.026770599
Pde1c|18575	-3.4375	0.170446566	1	#DIV/0!
Gm13034|627585	-3.4375	0.170446566	1	#DIV/0!
Nxnl2|75124	-3.4375	0.170446566	1	#DIV/0!
Zbtb8b|215627	-3.4375	0.170446566	1	#DIV/0!
2700099C18Rik|77022	-3.4375	0.170446566	1	#DIV/0!
E330034G19Rik|105418	-3.4375	0.170446566	1	#DIV/0!
Cyct|13067	-3.4375	0.170446566	1	#DIV/0!
Olfr1034|258216	-3.4375	0.170446566	1	#DIV/0!
Cckbr|12426	-3.4375	0.170446566	1	#DIV/0!
Tbrg3|21378	-3.4375	0.170446566	1	#DIV/0!
Pnpla1|433091	-3.4375	0.170446566	1	#DIV/0!
1500009C09Rik|76505	-3.4375	0.170446566	1	#DIV/0!
A630075F10Rik|100043910	-3.4375	0.170446566	1	#DIV/0!
Trim42|78911	-3.4375	0.170446566	1	#DIV/0!
Zfp442|668923	-3.4375	0.170446566	1	#DIV/0!
Miat|330166	-3.4375	0.170446566	1	#DIV/0!
Pspn|19197	-3.4375	0.170446566	1	#DIV/0!
Trpc1|22063	-3.4375	0.170446566	1	#DIV/0!
Akr1c21|77337	-3.4375	0.170446566	1	#DIV/0!
Nkx1-2|20231	-3.4375	0.170446566	1	#DIV/0!
Tex14|83560	-3.4375	0.170446566	1	#DIV/0!
Pcdh8|18530	-3.4375	0.170446566	1	#DIV/0!
Klra5|16636	-3.4375	0.170446566	1	#DIV/0!
Chst4|26887	-3.4375	0.170446566	1	#DIV/0!
Nov|18133	-3.4375	0.170446566	1	#DIV/0!
4930404I05Rik|67394	-3.4375	0.170446566	1	#DIV/0!
Lrrc63|70859	-3.4375	0.170446566	1	#DIV/0!
Pcnxl2|270109	-3.4375	0.170446566	1	#DIV/0!
Prdm6|225518	-3.4375	0.170446566	1	#DIV/0!
Prr19|623131	-3.4375	0.170446566	1	#DIV/0!
Sirpb1b|668101	-3.4375	0.170446566	1	#DIV/0!
Csmd3|239420	-3.4375	0.170446566	1	#DIV/0!
Hoxb1|15407	-3.4375	0.170446566	1	#DIV/0!
F930015N05Rik|654805	-3.4375	0.170446566	1	#DIV/0!
Wdr72|546144	-3.4375	0.170446566	1	#DIV/0!
Kif17|16559	-3.4375	0.170446566	1	#DIV/0!
Gm15348|100038554	-3.4375	0.170446566	1	#DIV/0!
Tox3|244579	-3.4375	0.170446566	1	#DIV/0!
Eif5a2|208691	-3.4375	0.170446566	1	#DIV/0!
Mcf2|109904	-3.4375	0.170446566	1	#DIV/0!
BC094916|545384	-3.4375	0.170446566	1	#DIV/0!
Mtap2|17756	-3.4375	0.170446566	1	#DIV/0!
Tekt2|24084	-3.4375	0.170446566	1	#DIV/0!

Drd5|13492	-3.4375	0.170446566	1	#DIV/0!
A930017M01Rik|239410	-3.4375	0.170446566	1	#DIV/0!
Olfr1443|258693	-3.4375	0.170446566	1	#DIV/0!
Clnk|27278	-3.4375	0.170446566	1	#DIV/0!
Pqlc3|217430	-3.4375	0.170446566	1	#DIV/0!
Ccdc46|76380	-3.4375	0.170446566	1	#DIV/0!
Usp29|57775	-3.4375	0.170446566	1	#DIV/0!
Gm5779|544707	-3.4375	0.170446566	1	#DIV/0!
Rgs17|56533	-3.4375	0.170446566	1	#DIV/0!
Duox2|214593	-3.4375	0.170446566	1	#DIV/0!
Erbb4|13869	-3.4375	0.170446566	1	#DIV/0!
Lrtm2|211187	-3.4375	0.170446566	1	#DIV/0!
Timm8a2|223262	-3.4375	0.170446566	1	#DIV/0!
Rem2|140743	-3.4375	0.170446566	1	#DIV/0!
Tekt5|70426	-3.4375	0.170446566	1	#DIV/0!
Mylk4|238564	-3.4375	0.170446566	1	#DIV/0!
Snora64|104366	-3.4375	0.170446566	1	#DIV/0!
Ryr3|20192	-3.4375	0.170446566	1	#DIV/0!
Olfr92|258448	-3.4375	0.170446566	1	#DIV/0!
3830408C21Rik|100040322	-3.4375	0.170446566	1	#DIV/0!
Ncan|13004	-3.4375	0.170446566	1	#DIV/0!
Rtn4r|65079	-3.4375	0.170446566	1	#DIV/0!
Gpr88|64378	-3.4375	0.170446566	1	#DIV/0!
Xlr4b|27083	-3.4375	0.170446566	1	#DIV/0!
Meis3|17537	-3.437607249	0.031473106	-21.69192	0.000829565
9930014A18Rik|320469	-3.439360494	0.033096024	-2.632254343	0.061036807
Zfp182|319535	-3.46942635	0.012908321	-6.453128919	0.005238345
Fsip1|71313	-3.478833333	0.170446566	1	#DIV/0!
Pxt1|69307	-3.478833333	0.170446566	1	#DIV/0!
Trim63|433766	-3.478833333	0.170446566	1	#DIV/0!
Fcamr|64435	-3.478833333	0.170446566	1	#DIV/0!
Tcf15|21407	-3.478833333	0.170446566	1	#DIV/0!
4922505G16Rik|629499	-3.478833333	0.170446566	1	#DIV/0!
Gm10190|791338	-3.478833333	0.170446566	1	#DIV/0!
Fbn2|14119	-3.478833333	0.170446566	1	#DIV/0!
Mir670|735259	-3.478833333	0.170446566	1	#DIV/0!
Slc30a8|239436	-3.478833333	0.170446566	1	#DIV/0!
Mir155|387173	-3.478833333	0.170446566	1	#DIV/0!
Trpm1|17364	-3.478833333	0.170446566	1	#DIV/0!
Alpk3|116904	-3.478833333	0.170446566	1	#DIV/0!
Foxd2|17301	-3.478833333	0.170446566	1	#DIV/0!
BC048671|243535	-3.478833333	0.170446566	1	#DIV/0!
Neto1|246317	-3.478833333	0.170446566	1	#DIV/0!
Tm7sf4|75766	-3.478833333	0.170446566	1	#DIV/0!
Grk1|24013	-3.478833333	0.170446566	1	#DIV/0!
Ptk6|20459	-3.478833333	0.170446566	1	#DIV/0!
Ttll9|74711	-3.478833333	0.170446566	1	#DIV/0!
Cenpe|229841	-3.478833333	0.170446566	1	#DIV/0!
S100a14|66166	-3.478833333	0.170446566	1	#DIV/0!
1700019L03Rik|227736	-3.478833333	0.170446566	1	#DIV/0!
Matn4|17183	-3.478833333	0.170446566	1	#DIV/0!
Akap3|11642	-3.478833333	0.170446566	1	#DIV/0!
Mgst2|211666	-3.478833333	0.170446566	1	#DIV/0!

Six2|20472	-3.478833333	0.170446566	1	#DIV/0!
Nrxn3|18191	-3.478833333	0.170446566	1	#DIV/0!
Mpo|17523	-3.478833333	0.170446566	1	#DIV/0!
Ntsr2|18217	-3.478833333	0.170446566	1	#DIV/0!
Ulk4|209012	-3.478833333	0.170446566	1	#DIV/0!
Mfap5|50530	-3.478833333	0.170446566	1	#DIV/0!
Dux|664783	-3.478833333	0.170446566	1	#DIV/0!
Cdkl1|71091	-3.478833333	0.170446566	1	#DIV/0!
Isl2|104360	-3.478833333	0.170446566	1	#DIV/0!
Pcdhb4|93875	-3.478833333	0.170446566	1	#DIV/0!
Klhdc1|271005	-3.478833333	0.170446566	1	#DIV/0!
Tcf23|69852	-3.478833333	0.170446566	1	#DIV/0!
4930511M06Rik|75084	-3.478833333	0.170446566	1	#DIV/0!
Alox15|11687	-3.478833333	0.170446566	1	#DIV/0!
Ppm1n|232941	-3.478833333	0.170446566	1	#DIV/0!
A530099J19Rik|319293	-3.478833333	0.170446566	1	#DIV/0!
Nt5c1b|70881	-3.478833333	0.170446566	1	#DIV/0!
Nudt10|102954	-3.478833333	0.170446566	1	#DIV/0!
Fgf18|14172	-3.478833333	0.170446566	1	#DIV/0!
Pax9|18511	-3.478833333	0.170446566	1	#DIV/0!
Gm7092|632778	-3.478833333	0.170446566	1	#DIV/0!
Gpr55|227326	-3.478833333	0.170446566	1	#DIV/0!
1700066B19Rik|73449	-3.478833333	0.170446566	1	#DIV/0!
Gm1574|380842	-3.478833333	0.170446566	1	#DIV/0!
Wnt11|22411	-3.478833333	0.170446566	1	#DIV/0!
Kif20b|240641	-3.478833333	0.170446566	1	#DIV/0!
Edar|13608	-3.478833333	0.170446566	1	#DIV/0!
Kbtbd13|74492	-3.478833333	0.170446566	1	#DIV/0!
Nwd1|319555	-3.478833333	0.170446566	1	#DIV/0!
Car10|72605	-3.478833333	0.170446566	1	#DIV/0!
Tpte|234129	-3.478833333	0.170446566	1	#DIV/0!
Ppp1r3a|140491	-3.478833333	0.170446566	1	#DIV/0!
Polq|77782	-3.478833333	0.170446566	1	#DIV/0!
Slc2a13|239606	-3.478833333	0.170446566	1	#DIV/0!
Epha3|13837	-3.478833333	0.170446566	1	#DIV/0!
Mms22l|212377	-3.485259467	0.01658467	-2.755260703	0.02386326
Pou2af1|18985	-3.49430375	0.037540757	-7.746994949	0.018765086
S100pbp|74648	-3.494966667	0.083451178	-11.5948	0.042761804
Pla2g4a|18783	-3.496528811	0.058200863	-13.90913333	0.02136273
Slc22a29|236293	-3.515633333	0.170446566	1	#DIV/0!
Dlgap3|242667	-3.525026931	0.004920397	-6.296606061	0.001429184
Mir1839|100316717	-3.525982596	0.083332883	-14.0263	0.041490024
Hsd3b6|15497	-3.541666667	0.170446566	1	#DIV/0!
Zbed6|667118	-3.561268482	0.233897864	1	#DIV/0!
Hist1h2bb|319178	-3.58465	0.170446566	1	#DIV/0!
Cnn1|12797	-3.610683116	0.189641251	1.206415778	0.394652548
Dzip1|66573	-3.629628078	0.147551708	-6.8	0.173296754
Dnase1l2|66705	-3.629628078	0.147551708	-6.8	0.173296754
Rnase6|78416	-3.633518017	0.116351852	-5.7452	0.078237796
Etv5|104156	-3.638575288	0.000327788	-3.638575288	0.000327788
Arhgap6|11856	-3.647334106	0.070692703	-2.152003708	0.089741351
Sybu|319613	-3.654550735	0.016276344	-6.103573588	0.010609883
Cct6b|12467	-3.671109067	0.165999474	-3.925	0.173296754

Fbxw17|109082	-3.675531429	0.014274216	-12.66436	5.78335E-05
Gzmb|14939	-3.683131429	0.093445767	-6.54176	0.086882566
Robo2|268902	-3.690363169	0.103598997	-14.0585	0.064345641
1110012J17Rik|68617	-3.69350062	0.083221725	-3.832670381	0.114337623
Pfn4|382562	-3.705542857	0.092547859	-6.6202	0.087728953
Zbp1|58203	-3.707987339	0.000965312	-3.106727154	0.002597225
Cyp1b1|13078	-3.714117634	0.059519908	-7.88976	0.029636667
Cyp4f39|320997	-3.714801051	0.100684576	-7.92898	0.029263299
Mmp24|17391	-3.743383333	0.170446566	1	#DIV/0!
Ccdc96|66717	-3.74388	0.033923263	-10.91965	0.013608087
Ppm1e|320472	-3.757316667	0.170446566	1	#DIV/0!
Pak3|18481	-3.757316667	0.170446566	1	#DIV/0!
Grm3|108069	-3.757316667	0.170446566	1	#DIV/0!
Slc36a2|246049	-3.757316667	0.170446566	1	#DIV/0!
Gm5095|328953	-3.757316667	0.170446566	1	#DIV/0!
1700029M20Rik|73937	-3.757316667	0.170446566	1	#DIV/0!
Dpep3|71854	-3.757316667	0.170446566	1	#DIV/0!
Expi|14038	-3.757316667	0.170446566	1	#DIV/0!
Tnfrsf17|21935	-3.757316667	0.170446566	1	#DIV/0!
Gjb3|14620	-3.757316667	0.170446566	1	#DIV/0!
Olfr518|258303	-3.757316667	0.170446566	1	#DIV/0!
Fancd2|211651	-3.757316667	0.170446566	1	#DIV/0!
A530058N18Rik|320846	-3.757316667	0.170446566	1	#DIV/0!
Vmn1r61|636731	-3.757316667	0.170446566	1	#DIV/0!
Tnnt3|21957	-3.757316667	0.170446566	1	#DIV/0!
Gm3985|100042715	-3.757316667	0.170446566	1	#DIV/0!
4933432I09Rik|66779	-3.757316667	0.170446566	1	#DIV/0!
B430319G15Rik|78575	-3.757316667	0.170446566	1	#DIV/0!
Tyrp1|22178	-3.757316667	0.170446566	1	#DIV/0!
Olfr699|258180	-3.757316667	0.170446566	1	#DIV/0!
Irs4|16370	-3.757316667	0.170446566	1	#DIV/0!
Ryr1|20190	-3.757316667	0.170446566	1	#DIV/0!
Gpr156|239845	-3.757316667	0.170446566	1	#DIV/0!
Crb1|170788	-3.757316667	0.170446566	1	#DIV/0!
F830045P16Rik|228592	-3.757316667	0.170446566	1	#DIV/0!
Olfr912|258806	-3.757316667	0.170446566	1	#DIV/0!
Tcp10a|21460	-3.757316667	0.170446566	1	#DIV/0!
4933427G17Rik|74466	-3.757316667	0.170446566	1	#DIV/0!
Olfr114|258284	-3.757316667	0.170446566	1	#DIV/0!
Gabrg1|14405	-3.757316667	0.170446566	1	#DIV/0!
Tmem232|381107	-3.757316667	0.170446566	1	#DIV/0!
Osr2|107587	-3.757316667	0.170446566	1	#DIV/0!
Col6a6|245026	-3.757316667	0.170446566	1	#DIV/0!
Vmn2r77|546983	-3.757316667	0.170446566	1	#DIV/0!
Olfr574|258357	-3.757316667	0.170446566	1	#DIV/0!
Sncg|20618	-3.757316667	0.170446566	1	#DIV/0!
Rtl1|353326	-3.757316667	0.170446566	1	#DIV/0!
Ccl17|20295	-3.757316667	0.170446566	1	#DIV/0!
Tfap2e|332937	-3.757316667	0.170446566	1	#DIV/0!
Pax3|18505	-3.757316667	0.170446566	1	#DIV/0!
1700057K13Rik|73435	-3.757316667	0.170446566	1	#DIV/0!
Celsr3|107934	-3.757316667	0.170446566	1	#DIV/0!
Gm10825|100038752	-3.757316667	0.170446566	1	#DIV/0!

Cd96|84544	-3.757316667	0.170446566	1	#DIV/0!
Ppy|19064	-3.757316667	0.170446566	1	#DIV/0!
Abca12|74591	-3.757316667	0.170446566	1	#DIV/0!
Olfr763|258861	-3.757316667	0.170446566	1	#DIV/0!
Shh|20423	-3.757316667	0.170446566	1	#DIV/0!
Rasd2|75141	-3.757316667	0.170446566	1	#DIV/0!
Mcpt4|17227	-3.757316667	0.170446566	1	#DIV/0!
Il11|16156	-3.757316667	0.170446566	1	#DIV/0!
Olfr684|244187	-3.757316667	0.170446566	1	#DIV/0!
Exo1|26909	-3.757316667	0.170446566	1	#DIV/0!
4933404M02Rik|66748	-3.757316667	0.170446566	1	#DIV/0!
Olfr353|258943	-3.757316667	0.170446566	1	#DIV/0!
Tspan1|66805	-3.757316667	0.170446566	1	#DIV/0!
Gm10125|791318	-3.757316667	0.170446566	1	#DIV/0!
BC125332|213765	-3.757316667	0.170446566	1	#DIV/0!
Radil|231858	-3.757316667	0.170446566	1	#DIV/0!
Svopl|320590	-3.757316667	0.170446566	1	#DIV/0!
Pla2g2f|26971	-3.757316667	0.170446566	1	#DIV/0!
Fam47a-ps|70864	-3.757316667	0.170446566	1	#DIV/0!
Sobp|109205	-3.757316667	0.170446566	1	#DIV/0!
Gm628|268816	-3.757316667	0.170446566	1	#DIV/0!
Apcdd1|494504	-3.757316667	0.170446566	1	#DIV/0!
Arpm1|76652	-3.757316667	0.170446566	1	#DIV/0!
Morn5|75495	-3.757316667	0.170446566	1	#DIV/0!
Ucp3|22229	-3.757316667	0.170446566	1	#DIV/0!
Ankrd34c|330998	-3.757316667	0.170446566	1	#DIV/0!
Olfr910|258807	-3.757316667	0.170446566	1	#DIV/0!
Esr2|13983	-3.757316667	0.170446566	1	#DIV/0!
Fam166b|329831	-3.757316667	0.170446566	1	#DIV/0!
Adig|246747	-3.757316667	0.170446566	1	#DIV/0!
Olfr610|259085	-3.757316667	0.170446566	1	#DIV/0!
Gm10790|100038577	-3.757316667	0.170446566	1	#DIV/0!
Syna|214292	-3.757316667	0.170446566	1	#DIV/0!
Flrt3|71436	-3.757316667	0.170446566	1	#DIV/0!
Grin2b|14812	-3.757316667	0.170446566	1	#DIV/0!
Cma1|17228	-3.757316667	0.170446566	1	#DIV/0!
Gria1|14799	-3.757316667	0.170446566	1	#DIV/0!
Aqp5|11830	-3.757316667	0.170446566	1	#DIV/0!
D7Ertd715e|52480	-3.757316667	0.170446566	1	#DIV/0!
Crnn|381457	-3.757316667	0.170446566	1	#DIV/0!
Ppp1r14c|76142	-3.757316667	0.170446566	1	#DIV/0!
Olfr1506|257665	-3.757316667	0.170446566	1	#DIV/0!
Stil|20460	-3.757316667	0.170446566	1	#DIV/0!
Gm3002|100040852	-3.757316667	0.170446566	1	#DIV/0!
Slc24a4|238384	-3.757316667	0.170446566	1	#DIV/0!
6330545A04Rik|76166	-3.757316667	0.170446566	1	#DIV/0!
Flg2|229574	-3.757316667	0.170446566	1	#DIV/0!
Trim43c|666731	-3.757316667	0.170446566	1	#DIV/0!
Sacs|50720	-3.757316667	0.170446566	1	#DIV/0!
Aspn|66695	-3.757316667	0.170446566	1	#DIV/0!
Hmgcll1|208982	-3.757316667	0.170446566	1	#DIV/0!
Ppfia3|76787	-3.757316667	0.170446566	1	#DIV/0!
Kcnj12|16515	-3.757316667	0.170446566	1	#DIV/0!

Lrrc4|192198	-3.757316667	0.170446566	1	#DIV/0!
Entpd3|215446	-3.757316667	0.170446566	1	#DIV/0!
3110035E14Rik|76982	-3.757316667	0.170446566	1	#DIV/0!
Rgnef|110596	-3.757316667	0.170446566	1	#DIV/0!
Ano2|243634	-3.757316667	0.170446566	1	#DIV/0!
Magi2|50791	-3.757316667	0.170446566	1	#DIV/0!
C1ql4|239659	-3.757316667	0.170446566	1	#DIV/0!
Pdzd7|212684	-3.757316667	0.170446566	1	#DIV/0!
Nipsnap3a|73398	-3.757316667	0.170446566	1	#DIV/0!
Cst9|13013	-3.757316667	0.170446566	1	#DIV/0!
Gm14164|791417	-3.757316667	0.170446566	1	#DIV/0!
Gm13154|433804	-3.757316667	0.170446566	1	#DIV/0!
5031434O11Rik|100039684	-3.757316667	0.170446566	1	#DIV/0!
Tas2r126|387353	-3.757316667	0.170446566	1	#DIV/0!
Ccdc7|74703	-3.757316667	0.170446566	1	#DIV/0!
Hcrtr1|230777	-3.757316667	0.170446566	1	#DIV/0!
Rph3a|19894	-3.757316667	0.170446566	1	#DIV/0!
Ankrd32|105377	-3.763436835	0.079830056	-7.725	0.072984714
Kif18a|228421	-3.783486464	0.074070559	-11.79098333	0.044340227
Nr3c2|110784	-3.786453319	0.059532397	-2.436615709	0.096117552
Serpina7|331535	-3.78662814	0.036592561	-2.780152612	0.06421625
Spag17|74362	-3.802083333	0.170446566	1	#DIV/0!
Car3|12350	-3.805007256	0.042683101	-3.957832631	0.000806178
Tnc|21923	-3.828148571	0.080801914	-6.38098	0.020002315
Ccdc92|215707	-3.831116667	0.058469763	-12.46038	0.00415976
Prdx6b|320769	-3.842759522	0.035764925	-11.32618	0.006284669
Ccdc85c|668158	-3.84780617	0.007468245	-5.973314477	0.005337981
Pcdhga7|93715	-3.866666667	0.170446566	1	#DIV/0!
Cpe|12876	-3.867731421	0.120927163	-8.6996	0.024337076
Mthfd2l|665563	-3.868771629	0.145255652	-5.68578	0.079282153
Tff2|21785	-3.884686127	0.227397646	1	#DIV/0!
Cxcr5|12145	-3.898949654	0.144309579	-4.58098	0.070596339
Kcng2|240444	-3.899017143	0.073294895	-7.64656	0.072618354
Pou2f1|18986	-3.909903987	0.01383886	-3.909903987	0.01383886
Gpr135|238252	-3.910222857	0.110885741	-5.68578	0.079282153
Tssk4|71099	-3.913834286	0.220458896	1	#DIV/0!
Pcsk9|100102	-3.926378226	0.02320206	-2.402670307	0.003424212
Zfp800|627049	-3.934884706	0.066180567	-27.8721	0.028446391
Ankrd13d|68423	-3.93868	0.058090855	-7.53538	0.026553614
Mylpf|17907	-3.950974939	0.012374846	-14.57554	5.88971E-05
Tas1r2|83770	-3.967316667	0.072480209	-3.967316667	0.072480209
Ly6g5b|266614	-3.967316667	0.072480209	-3.967316667	0.072480209
Gpr27|14761	-3.967316667	0.072480209	-3.967316667	0.072480209
Serpini1|20713	-3.967316667	0.072480209	-3.967316667	0.072480209
Ccr3|12771	-3.967316667	0.072480209	-3.967316667	0.072480209
BC030476|239368	-3.967316667	0.072480209	-3.967316667	0.072480209
Inpp5j|170835	-3.967316667	0.072480209	-3.967316667	0.072480209
Cacna1i|239556	-3.967316667	0.072480209	-3.967316667	0.072480209
3110021A11Rik|67289	-3.967316667	0.072480209	-3.967316667	0.072480209
Fam154b|330577	-3.967316667	0.072480209	-3.967316667	0.072480209
Tro|56191	-3.967316667	0.072480209	-3.967316667	0.072480209
DXBay18|574405	-3.967316667	0.072480209	-3.967316667	0.072480209
Ccr9|12769	-3.968502857	0.053196162	-7.88976	0.029636667

Gjc1|14615	-3.969828571	0.058185722	-7.5452	0.025671227
Kif13b|16554	-3.973451163	0.080569928	-2.168712143	0.103339441
Cd177|68891	-3.98415	0.072501035	-3.98415	0.072501035
Lox|16948	-3.98415	0.072501035	-3.98415	0.072501035
Tbx4|21387	-3.98415	0.072501035	-3.98415	0.072501035
Pcdhb17|93888	-3.98415	0.072501035	-3.98415	0.072501035
B3gnt6|272411	-3.98415	0.072501035	-3.98415	0.072501035
Zfp459|328274	-3.98415	0.072501035	-3.98415	0.072501035
Sit1|54390	-3.98415	0.072501035	-3.98415	0.072501035
Fmod|14264	-3.984164185	0.001858819	-3.984164185	0.001858819
Vav3|57257	-4.000996667	0.131062407	-8.38098	0.03489567
Ptpn22|19260	-4.007028571	0.072378198	-7.7746	0.072687134
Tbx20|57246	-4.007028571	0.072378198	-7.7746	0.072687134
Tlr7|170743	-4.01231643	0.025882697	-12.50411667	0.010000955
Caps2|353025	-4.016833333	0.072468013	-4.016833333	0.072468013
4732415M23Rik|320869	-4.016833333	0.072468013	-4.016833333	0.072468013
Clic3|69454	-4.016833333	0.072468013	-4.016833333	0.072468013
2900002K06Rik|70226	-4.016833333	0.072468013	-4.016833333	0.072468013
BB123696|105404	-4.016833333	0.072468013	-4.016833333	0.072468013
Shank1|243961	-4.016833333	0.072468013	-4.016833333	0.072468013
Peg12|27412	-4.016833333	0.072468013	-4.016833333	0.072468013
Chrna4|11438	-4.016833333	0.072468013	-4.016833333	0.072468013
4933403G14Rik|74393	-4.016833333	0.072468013	-4.016833333	0.072468013
9430060I03Rik|100037260	-4.017333333	0.072500935	-4.017333333	0.072500935
Fam57a|116972	-4.027877354	0.033211771	-2.926135751	0.053529524
Hace1|209462	-4.028654921	0.036356264	-6.990550505	0.02980384
Fam151a|230579	-4.031371283	0.04138522	-18.08462821	0.016396663
B3gnt9-ps|97440	-4.045849493	0.056054929	-4.990597974	0.062871287
Mtbp|105837	-4.100059906	0.032041164	-6.864031618	0.027260213
Abca17|381072	-4.1013	0.170446566	1	#DIV/0!
Wscd2|320916	-4.1013	0.170446566	1	#DIV/0!
Padi4|18602	-4.1013	0.170446566	1	#DIV/0!
Gm10046|100043229	-4.1013	0.170446566	1	#DIV/0!
Zfp712|78251	-4.1013	0.170446566	1	#DIV/0!
Ikzf3|22780	-4.1013	0.170446566	1	#DIV/0!
Prrg1|546336	-4.1013	0.170446566	1	#DIV/0!
3110079O15Rik|73234	-4.1013	0.170446566	1	#DIV/0!
Tagap|72536	-4.105815747	0.059219726	-9.6606	0.02250222
Chml|12663	-4.106547394	0.011374079	-5.819498074	0.008325994
Ggt7|207182	-4.132142138	0.024778656	-29.43173333	0.00493151
Aff3|16764	-4.132575874	0.051944503	-15.74311667	0.027477208
Tyro3|22174	-4.14312	0.157863084	-3.9746	0.173296754
Hdgfrp3|29877	-4.158125202	0.04781766	-5.40412236	0.027479211
4933437F05Rik|71275	-4.166666667	0.170446566	1	#DIV/0!
Gal3st2|381334	-4.166666667	0.170446566	1	#DIV/0!
1700026L06Rik|69987	-4.166666667	0.170446566	1	#DIV/0!
Rab15|104886	-4.166666667	0.170446566	1	#DIV/0!
Gm14492|677289	-4.166666667	0.170446566	1	#DIV/0!
Cdc6|23834	-4.166666667	0.170446566	1	#DIV/0!
Lca5|75782	-4.166666667	0.170446566	1	#DIV/0!
Lrrc8b|433926	-4.166666667	0.170446566	1	#DIV/0!
9330175E14Rik|320377	-4.166666667	0.170446566	1	#DIV/0!
Meox1|17285	-4.166666667	0.170446566	1	#DIV/0!

Vmn1r90|627280	-4.166666667	0.170446566	1	#DIV/0!
Gen1|209334	-4.166666667	0.170446566	1	#DIV/0!
Zfa|22639	-4.166666667	0.170446566	1	#DIV/0!
Olfr920|258783	-4.166666667	0.170446566	1	#DIV/0!
Hepacam|72927	-4.166666667	0.170446566	1	#DIV/0!
Osm|18413	-4.166666667	0.170446566	1	#DIV/0!
Zfp808|630579	-4.166666667	0.170446566	1	#DIV/0!
Gprasp2|245607	-4.166666667	0.170446566	1	#DIV/0!
Gja10|14610	-4.166666667	0.170446566	1	#DIV/0!
Scube2|56788	-4.166666667	0.170446566	1	#DIV/0!
Slc39a10|227059	-4.17042407	0.060649645	-9.68976	0.005314071
Crybb3|12962	-4.185571416	0.026568015	-16.65013333	0.009431887
Atp10a|11982	-4.189742857	0.07576674	-7.64656	0.072618354
6530402F18Rik|76220	-4.190315477	0.031462601	-7.01513114	0.027246689
Ms4a4c|64380	-4.194345334	0.002219971	-4.788455993	0.000233968
Dgkg|110197	-4.200333333	0.170446566	1	#DIV/0!
Rpl39l|68172	-4.200333333	0.170446566	1	#DIV/0!
St8sia2|20450	-4.200333333	0.170446566	1	#DIV/0!
Igsf1|209268	-4.200333333	0.170446566	1	#DIV/0!
Casq2|12373	-4.200333333	0.170446566	1	#DIV/0!
Caly|68566	-4.200333333	0.170446566	1	#DIV/0!
Dnahc17|69926	-4.200333333	0.170446566	1	#DIV/0!
Trim69|70928	-4.200333333	0.170446566	1	#DIV/0!
Ms4a1|12482	-4.200333333	0.170446566	1	#DIV/0!
Pcdhb5|93876	-4.200333333	0.170446566	1	#DIV/0!
Gnaz|14687	-4.200333333	0.170446566	1	#DIV/0!
Eif2c3|214150	-4.211428571	0.023316371	-12.28333333	0.010495633
Pitpnm3|327958	-4.236894741	0.041320968	-9.5948	0.027916032
Tssk6|83984	-4.259687891	0.084422417	-13.27503333	0.061241107
Prr22|224908	-4.264899556	0.021023564	-28.9404	0.00426534
Mybpc3|17868	-4.311814788	0.10764473	-6.60118	0.089542423
Atp8b4|241633	-4.315142462	0.04712525	-21.13715	0.022968078
Moap1|64113	-4.343321901	0.221721953	2.44286	0.173296754
Efha2|78506	-4.406902121	0.019363604	-10.48058	0.003988962
Gm3417|100041586	-4.413441822	0.076654802	-2.76078	0.173296754
Cep170|545389	-4.429162435	0.033163075	-31.5473	0.01175898
Tnni3|21954	-4.441534446	0.218797203	1	#DIV/0!
Sult2a7|638251	-4.508294439	0.003632886	-4.837219759	0.003954989
6430562O15Rik|320893	-4.516666667	0.170446566	1	#DIV/0!
Ccdc48|58229	-4.534497143	0.01932204	-13.22561667	0.009603687
Prss50|235631	-4.585345473	0.06717417	-11.43774	0.066710478
Sqle|20775	-4.600024978	0.035396829	-2.584234184	0.016843922
1500004A13Rik|319830	-4.623339693	0.054638186	-5.129565848	0.001788852
Mzf1|109889	-4.709622547	0.010129409	-22.28176	9.71651E-05
Zfp69|381549	-4.736897674	0.031167344	-18.8433	0.01666352
Grk4|14772	-4.737557892	0.032989512	-10.6996	0.021466055
40971|320253	-4.801838452	0.023445101	-19.10163333	0.011400496
Matk|17179	-4.81141617	0.040162457	-12.44136	0.003844518
Cacna1a|12286	-4.830657143	0.100023492	-6.38098	0.020002315
D630029K05Rik|103175	-4.904816667	0.080654503	-2.76078	0.148332518
Pla2g4f|271844	-4.904816667	0.080654503	-2.76078	0.148332518
Ephx4|384214	-4.904816667	0.080654503	-2.76078	0.148332518
Kcnd1|16506	-4.9375	0.079997504	-2.8	0.148332518

5730577I03Rik|66662	-4.9375	0.079997504	-2.8	0.148332518
Parp8|52552	-4.9375	0.079997504	-2.8	0.148332518
Gal3st3|545276	-4.9375	0.079997504	-2.8	0.148332518
Trpv1|193034	-4.9375	0.079997504	-2.8	0.148332518
Cd209a|170786	-4.9375	0.079997504	-2.8	0.148332518
Mageb3|17147	-4.9375	0.079997504	-2.8	0.148332518
Ptgir|19222	-4.94615	0.08117076	-2.76078	0.148332518
Fndc8|78919	-4.94615	0.08117076	-2.76078	0.148332518
Sh3d20|70559	-4.94615	0.08117076	-2.76078	0.148332518
BC006965|217294	-4.94615	0.08117076	-2.76078	0.148332518
Alox5|11689	-4.94615	0.08117076	-2.76078	0.148332518
Fam171b|241520	-4.94615	0.08117076	-2.76078	0.148332518
AA474331|213332	-4.94615	0.08117076	-2.76078	0.148332518
Rmrp|19782	-4.94615	0.08117076	-2.76078	0.148332518
B230319C09Rik|320775	-4.94615	0.08117076	-2.76078	0.148332518
Hrasls|27281	-4.94615	0.08117076	-2.76078	0.148332518
Ect2l|100039660	-4.94615	0.08117076	-2.76078	0.148332518
Rbl1|19650	-4.952504466	0.010835139	-12.46038	0.00415976
Gprc5a|232431	-4.954333333	0.079673302	-2.8202	0.148332518
Ano5|233246	-4.954333333	0.079673302	-2.8202	0.148332518
Necab3|56846	-4.954333333	0.079673302	-2.8202	0.148332518
Panx2|406218	-4.954333333	0.079673302	-2.8202	0.148332518
Rell2|225392	-4.960316667	0.170446566	1	#DIV/0!
1700001J03Rik|69282	-4.978833333	0.080496976	-2.8	0.148332518
Ccl22|20299	-4.978833333	0.080496976	-2.8	0.148332518
Ect2|13605	-4.978833333	0.080496976	-2.8	0.148332518
Slc2a12|353169	-4.978833333	0.080496976	-2.8	0.148332518
Sidt1|320007	-4.995666667	0.080164031	-2.8202	0.148332518
Fam13c|71721	-4.995666667	0.080164031	-2.8202	0.148332518
Barx2|12023	-4.995666667	0.080164031	-2.8202	0.148332518
Zmynd10|114602	-4.995666667	0.080164031	-2.8202	0.148332518
Ipcef1|320495	-4.995666667	0.080164031	-2.8202	0.148332518
2310010M20Rik|69576	-4.995666667	0.080164031	-2.8202	0.148332518
Cdkn2b|12579	-4.995666667	0.080164031	-2.8202	0.148332518
Rccd1|269955	-5.048451727	0.010992564	-11.19259596	0.007584192
Pde5a|242202	-5.075705142	0.018679422	-19.33598333	0.009621034
Dpf1|29861	-5.100686682	0.01002458	-12.82514	0.003882869
Pcdhga2|93710	-5.104166667	0.170446566	1	#DIV/0!
Nkx6-2|14912	-5.112474817	0.06949652	-2.775680432	0.078579103
Wee1|22390	-5.139616667	0.058644094	-16.8377	0.054336293
Clstn2|64085	-5.224633333	0.084711076	-2.76078	0.148332518
Cited1|12705	-5.224633333	0.084711076	-2.76078	0.148332518
Fzd9|14371	-5.224633333	0.084711076	-2.76078	0.148332518
Tspo2|70026	-5.224633333	0.084711076	-2.76078	0.148332518
Scn10a|20264	-5.224633333	0.084711076	-2.76078	0.148332518
Efna4|13639	-5.224633333	0.084711076	-2.76078	0.148332518
Zfp521|225207	-5.224633333	0.084711076	-2.76078	0.148332518
Gm13242|100041379	-5.224633333	0.084711076	-2.76078	0.148332518
Adamts12|239337	-5.224633333	0.084711076	-2.76078	0.148332518
Gipc3|209047	-5.224633333	0.084711076	-2.76078	0.148332518
Uhrf1|18140	-5.224633333	0.084711076	-2.76078	0.148332518
Podnl1|244550	-5.232446089	0.033607249	-14.45118	0.015946801
Rnf32|56874	-5.257316667	0.08394264	-2.8	0.148332518

Zbtb3|75291	-5.257316667	0.08394264	-2.8	0.148332518
Slc6a4|15567	-5.257316667	0.08394264	-2.8	0.148332518
Pask|269224	-5.257316667	0.08394264	-2.8	0.148332518
Fam196a|627214	-5.257316667	0.08394264	-2.8	0.148332518
Slc25a2|83885	-5.257316667	0.08394264	-2.8	0.148332518
Melk|17279	-5.257316667	0.08394264	-2.8	0.148332518
Hmgn3|94353	-5.257316667	0.08394264	-2.8	0.148332518
Garnl3|99326	-5.257316667	0.08394264	-2.8	0.148332518
Cd209c|170776	-5.257316667	0.08394264	-2.8	0.148332518
Kcnk4|16528	-5.27415	0.083560145	-2.8202	0.148332518
Ttll2|100216474	-5.27415	0.083560145	-2.8202	0.148332518
Me3|109264	-5.27415	0.083560145	-2.8202	0.148332518
Akr1cl|70861	-5.27415	0.083560145	-2.8202	0.148332518
Tmem59l|67937	-5.27415	0.083560145	-2.8202	0.148332518
Prss30|30943	-5.27415	0.083560145	-2.8202	0.148332518
Myom2|17930	-5.27415	0.083560145	-2.8202	0.148332518
Ly6g6c|68468	-5.27415	0.083560145	-2.8202	0.148332518
Tubb2b|73710	-5.31165	0.072540194	-5.31165	0.072540194
1700029I01Rik|70005	-5.341202015	0.162567817	-3.9746	0.173296754
Gabbr2|242425	-5.433939815	0.025372958	-6.184243646	0.006359085
Blnk|17060	-5.450616667	0.003911042	-27.25308333	0.000855779
D6Mm5e|110958	-5.6013	0.088228602	-2.8	0.148332518
Zmynd17|74843	-5.633983333	0.089892176	-2.76078	0.148332518
Boc|117606	-5.633983333	0.089892176	-2.76078	0.148332518
Fktn|246179	-5.662454594	0.040372478	-14.53018	0.016384346
Rasl10a|75668	-5.66765	0.090309247	-2.76078	0.148332518
Gm12839|631037	-5.66765	0.090309247	-2.76078	0.148332518
Cdx4|12592	-5.66765	0.090309247	-2.76078	0.148332518
Cd209f|69142	-5.676888205	0.028110088	-16.57276	0.023349923
Lrrc17|74511	-5.6835	0.08860069	-2.8202	0.148332518
Cd8a|12525	-5.700333333	0.089443396	-2.8	0.148332518
Gpr3|14748	-5.7353	0.170446566	1	#DIV/0!
Meig1|104362	-5.7353	0.170446566	1	#DIV/0!
Il27|246779	-5.7353	0.170446566	1	#DIV/0!
Rhox8|434768	-5.7353	0.170446566	1	#DIV/0!
Tmem150c|231503	-5.7353	0.170446566	1	#DIV/0!
4933431E20Rik|329735	-5.782027982	0.001213507	-11.62448348	0.000814706
Sult2a3|629203	-5.786906996	0.05022558	-24.90022	0.012979003
Trpm6|225997	-5.826863696	0.12925353	-7.815	0.072872131
Uprt|331487	-5.833333333	0.170446566	1	#DIV/0!
F830002L21Rik|414125	-5.833333333	0.170446566	1	#DIV/0!
Fut4|14345	-5.833333333	0.170446566	1	#DIV/0!
Gli2|14633	-5.833333333	0.170446566	1	#DIV/0!
Fv1|14349	-5.833333333	0.170446566	1	#DIV/0!
A430093F15Rik|403202	-5.833333333	0.170446566	1	#DIV/0!
Flnc|68794	-5.861702857	0.112981056	-7.81754	0.173296754
D630003M21Rik|228846	-5.863159219	0.011675226	-4.247235302	0.003274015
Ckmt2|76722	-5.883833333	0.170446566	1	#DIV/0!
Myh3|17883	-5.916333333	0.072473362	-5.916333333	0.072473362
Fgf9|14180	-5.916333333	0.072473362	-5.916333333	0.072473362
1700019B03Rik|76406	-5.916333333	0.072473362	-5.916333333	0.072473362
Far2|330450	-6.005062912	0.042650456	-29.415	0.032516007
Sell|20343	-6.027504733	0.014722808	-14.46038	0.010822609

Gm5640|434797	-6.041666667	0.170446566	1	#DIV/0!
Dnahc11|13411	-6.041666667	0.170446566	1	#DIV/0!
Xlr3b|574437	-6.119783333	0.170446566	1	#DIV/0!
Tmem154|320782	-6.124333333	0.170446566	1	#DIV/0!
Dpep2|319446	-6.124333333	0.170446566	1	#DIV/0!
Cckar|12425	-6.124333333	0.170446566	1	#DIV/0!
Foxf1a|15227	-6.165848748	0.04891509	-6.165848748	0.04891509
0610039K10Rik|68386	-6.194816667	0.072975795	-6.194816667	0.072975795
Tbx19|83993	-6.194816667	0.072975795	-6.194816667	0.072975795
Sost|74499	-6.194816667	0.072975795	-6.194816667	0.072975795
Cspg5|29873	-6.194816667	0.072975795	-6.194816667	0.072975795
Creb5|231991	-6.194816667	0.072975795	-6.194816667	0.072975795
Col4a6|94216	-6.194816667	0.072975795	-6.194816667	0.072975795
Gabre|14404	-6.23615	0.072846013	-6.23615	0.072846013
C920009B18Rik|606736	-6.23615	0.072846013	-6.23615	0.072846013
1810062O18Rik|75602	-6.23615	0.072846013	-6.23615	0.072846013
Foxp3|20371	-6.266933333	0.072599614	-6.266933333	0.072599614
Hgf|15234	-6.348331429	0.019871512	-12.84416	0.004174475
Npl|74091	-6.376766175	0.025283065	-14.32352	0.023723069
Gpr183|321019	-6.404816667	0.03059946	-6.404816667	0.03059946
2010001M09Rik|69816	-6.404816667	0.03059946	-6.404816667	0.03059946
Zfp831|100043757	-6.404816667	0.03059946	-6.404816667	0.03059946
Zfp456|408065	-6.404816667	0.03059946	-6.404816667	0.03059946
Mmp28|118453	-6.404816667	0.03059946	-6.404816667	0.03059946
Sh2d5|230863	-6.44615	0.031032931	-6.44615	0.031032931
Nmnat2|226518	-6.44615	0.031032931	-6.44615	0.031032931
Srpx|51795	-6.44615	0.031032931	-6.44615	0.031032931
Sgip1|73094	-6.454333333	0.030184854	-6.454333333	0.030184854
Gm5409|386551	-6.47715	0.097710279	-2.8202	0.148332518
E130306D19Rik|230098	-6.495666667	0.030602242	-6.495666667	0.030602242
Fam109b|338368	-6.495666667	0.030602242	-6.495666667	0.030602242
Dbp|13170	-6.521435582	0.002826266	-5.365739851	0.002478306
4833424O15Rik|75769	-6.5388	0.074397793	-3.925	0.148332518
Lca5l|385668	-6.5388	0.074397793	-3.925	0.148332518
1700011H14Rik|67082	-6.5388	0.074397793	-3.925	0.148332518
Anxa8|11752	-6.580133333	0.074140319	-6.580133333	0.074140319
Fpr1|14293	-6.596058671	0.000136764	-6.596058671	0.000136764
Cacna1c|12288	-6.604166667	0.074741536	-3.925	0.148332518
D830031N03Rik|442834	-6.604166667	0.074741536	-3.925	0.148332518
Tmem35|67564	-6.6455	0.074462325	-3.9746	0.148332518
Snai3|30927	-6.679166667	0.0746359	-3.9746	0.148332518
Atp10b|319767	-6.681283333	0.170446566	1	#DIV/0!
5730522E02Rik|70626	-6.681283333	0.170446566	1	#DIV/0!
Thsd7b|210417	-6.681283333	0.170446566	1	#DIV/0!
BC055324|381306	-6.681283333	0.170446566	1	#DIV/0!
Ctnna2|12386	-6.681283333	0.170446566	1	#DIV/0!
Il1rl1|17082	-6.681283333	0.170446566	1	#DIV/0!
Onecut3|246086	-6.681283333	0.170446566	1	#DIV/0!
4921536K21Rik|67430	-6.681283333	0.170446566	1	#DIV/0!
Tpsb2|17229	-6.681283333	0.170446566	1	#DIV/0!
Olr1|108078	-6.681283333	0.170446566	1	#DIV/0!
Pcdh15|11994	-6.681283333	0.170446566	1	#DIV/0!
1600014K23Rik|71996	-6.681283333	0.170446566	1	#DIV/0!

Gm12359|100037262	-6.724633333	0.034159916	-4.56078	0.051959117
Pax5|18507	-6.741466667	0.033983246	-4.58098	0.051978224
Asb4|65255	-6.741466667	0.033983246	-4.58098	0.051978224
5730408K05Rik|67531	-6.741466667	0.033983246	-4.58098	0.051978224
Klrg2|74253	-6.858616667	0.07292953	-6.858616667	0.07292953
Xkrx|331524	-6.858616667	0.07292953	-6.858616667	0.07292953
Gm11346|76024	-6.923983333	0.073108617	-6.923983333	0.073108617
6030419C18Rik|319477	-6.923983333	0.073108617	-6.923983333	0.073108617
Krt80|74127	-7.082420322	0.009347013	-21.3085	0.002423147
Mb|17189	-7.1122456	0.169575856	-2.777151865	0.10836643
Slc22a28|434674	-7.181866667	0.090606899	-3.32	0.148332518
Gm88|224813	-7.2353	0.105817953	-2.8	0.148332518
Cdkl4|381113	-7.267966667	0.07247851	-7.267966667	0.07247851
Kctd14|233529	-7.267966667	0.07247851	-7.267966667	0.07247851
Kcna2|16490	-7.350166667	0.10621631	-2.8202	0.148332518
Kif19a|286942	-7.35115	0.108071376	-2.76078	0.148332518
Fank1|66930	-7.38365	0.029242748	-7.38365	0.029242748
Slc35f2|72022	-7.433166667	0.028728557	-7.433166667	0.028728557
4921517L17Rik|70873	-7.43435	0.072497133	-7.43435	0.072497133
Cdhr2|268663	-7.5	0.170446566	1	#DIV/0!
Prrg4|228413	-7.5	0.170446566	1	#DIV/0!
Rundc3b|242819	-7.541666667	0.108485978	-2.8	0.148332518
2610203C22Rik|72481	-7.541666667	0.108485978	-2.8	0.148332518
Hrc|15464	-7.567333333	0.170446566	1	#DIV/0!
Tceal3|594844	-7.624316667	0.048963139	-7.624316667	0.048963139
Gm10565|100038693	-7.624333333	0.109174211	-2.8	0.148332518
Barx1|12022	-7.641166667	0.108702169	-2.8202	0.148332518
Ccdc160|434778	-7.703466667	0.030520559	-7.703466667	0.030520559
Stac2|217154	-7.703466667	0.030520559	-7.703466667	0.030520559
Cytl1|231162	-7.73615	0.030148108	-7.73615	0.030148108
Nphp4|260305	-7.73615	0.030148108	-7.73615	0.030148108
Fgf13|14168	-7.73615	0.030148108	-7.73615	0.030148108
Dok7|231134	-7.752983333	0.029962697	-7.752983333	0.029962697
Mylk3|213435	-7.752983333	0.029962697	-7.752983333	0.029962697
Gnai1|14677	-7.897097143	0.092023903	-23.0332	0.093667967
Obscn|380698	-8.0388	0.03215127	-8.0388	0.03215127
Mst1r|19882	-8.080133333	0.032113161	-8.080133333	0.032113161
Trappc2|66226	-8.112816667	0.032953773	-8.112816667	0.032953773
Plekhh1|211945	-8.135866667	0.095562058	-3.46	0.148332518
Pcdhga9|93717	-8.1371	0.082303856	-4.11372	0.148332518
Dlk2|106565	-8.1486	0.114390887	-2.76078	0.148332518
Nyx|236690	-8.1486	0.114390887	-2.76078	0.148332518
Tet1|52463	-8.162333333	0.032338998	-8.162333333	0.032338998
Has3|15118	-8.181283333	0.113485384	-2.8	0.148332518
Eef1a2|13628	-8.198116667	0.113024445	-2.8202	0.148332518
Mir1941|100316694	-8.198116667	0.113024445	-2.8202	0.148332518
Egr2|13654	-8.270833333	0.08665517	-3.925	0.148332518
Leprel1|210530	-8.3913	0.032927758	-8.3913	0.032927758
Gli3|14634	-8.440816667	0.032305977	-8.440816667	0.032305977
Sfxn4|94281	-8.440816667	0.032305977	-8.440816667	0.032305977
Parm1|231440	-8.440816667	0.032305977	-8.440816667	0.032305977
Hnf4g|30942	-8.561833333	0.088869733	-3.925	0.148332518
Mecom|14013	-8.59065	0.08234736	-4.30878	0.148332518

Gypa|14934	-8.67365	0.024954816	-8.67365	0.024954816
9530009M10Rik|320856	-8.67365	0.024954816	-8.67365	0.024954816
Gm6644|626009	-8.689147434	0.144845475	-2.64314	0.173296754
Rab3c|67295	-8.702283333	0.073152949	-8.702283333	0.073152949
Sp7|170574	-8.769833333	0.170446566	1	#DIV/0!
Angpt4|11602	-8.798983333	0.08375992	-4.30878	0.148332518
Dpysl4|26757	-8.801633333	0.033258491	-8.801633333	0.033258491
Sstr2|20606	-8.900483333	0.007764865	-8.900483333	0.007764865
Cldn4|12740	-8.900483333	0.007764865	-8.900483333	0.007764865
Gm6524|624710	-9.017633333	0.025878741	-9.017633333	0.025878741
Pln|18821	-9.033666667	0.077981353	-4.8404	0.148332518
Atl1|73991	-9.0585	0.061817996	-4.6202	0.051947928
Actn2|11472	-9.067333333	0.119319775	-2.8	0.148332518
Bdkrb2|12062	-9.083	0.026114379	-9.083	0.026114379
Ttc26|264134	-9.083	0.026114379	-9.083	0.026114379
Mpp3|13384	-9.09165	0.063731618	-4.56078	0.051959117
Hist1h2be|319179	-9.09885	0.084656217	-4.37894	0.148332518
Ghrhr|14602	-9.178966667	0.008580774	-9.178966667	0.008580774
Ak1|11636	-9.203466667	0.008716537	-9.203466667	0.008716537
Frem1|329872	-9.21165	0.008437944	-9.21165	0.008437944
Ndn|17984	-9.21165	0.008437944	-9.21165	0.008437944
Dennd2c|329727	-9.2203	0.008644074	-9.2203	0.008644074
4921507P07Rik|70821	-9.361483333	0.025742591	-9.361483333	0.025742591
Lrrn4|320974	-9.361483333	0.025742591	-9.361483333	0.025742591
Gm14391|665001	-9.362020513	0.038380913	-9.14482	0.035522074
Cdca7l|217946	-9.402816667	0.025620866	-9.402816667	0.025620866
Zfp385c|278304	-9.494766667	0.077438686	-5.11372	0.148332518
Gm4956|241041	-9.60515	0.010493449	-9.60515	0.010493449
Sst|20604	-9.605266667	0.170446566	1	#DIV/0!
Nr2c2|22026	-9.637950515	0.122650282	-11.66406	0.117632635
Tmem229a|319832	-9.730966667	0.045494217	-5.7948	0.059069839
4921506M07Rik|70846	-9.73815	0.047552281	-5.68578	0.059527684
D130017N08Rik|320064	-9.908133333	0.010556556	-9.908133333	0.010556556
A230072E10Rik|331547	-9.918783333	0.091274433	-4.30878	0.148332518
Nfkbid|243910	-9.987816667	0.049134567	-5.73538	0.060010083
Nusap1|108907	-10.06183333	0.049761807	-5.725	0.058914492
Zc3hav1l|209032	-10.14115	0.045029246	-6.10878	0.062608491
Rftn2|74013	-10.16143333	0.031931373	-6.8996	0.051952835
Cd8b1|12526	-10.50098333	0.04290035	-6.60118	0.068626797
Fabp3|14077	-10.53465	0.078560485	-4.56078	0.051959117
Zfp879|214779	-10.61878333	0.054943378	-5.725	0.058914492
Abp1|76507	-10.7203	0.001087301	-12.66436	1.46142E-05
Zfp455|218311	-10.742	0.043882614	-6.6404	0.067804405
Olfr1372-ps1|257871	-10.742	0.043882614	-6.6404	0.067804405
Gbp1|14468	-10.74966667	0.036080215	-6.8996	0.051952835
Pcdhgb2|93700	-10.95208333	0.077077642	-5.8625	0.148332518
Popdc2|64082	-11.16543333	0.037346851	-6.38098	0.011940725
Sulf1|240725	-11.31915	0.036339011	-7.23378	0.052414049
Plcb4|18798	-11.31915	0.036339011	-7.23378	0.052414049
Gm514|208080	-11.40315	0.100270059	-4.30878	0.148332518
A730008H23Rik|212427	-11.5748	0.020092838	-7.88976	0.018871593
Lrrc19|100061	-11.64096667	0.000842659	-13.76916	5.27897E-06
Col23a1|237759	-11.64096667	0.000842659	-13.76916	5.27897E-06

Acss1|68738	-12.18448333	0.042466883	-12.18448333	0.042466883
D330028D13Rik|231946	-12.31295	0.001093475	-14.57554	1.49198E-05
Ccdc158|320696	-12.50816667	0.042679982	-12.50816667	0.042679982
Gm2518|100039957	-12.71621667	0.028161145	-12.71621667	0.028161145
Gabrb3|14402	-13.00465	0.007873374	-9.35558	0.001847968
Ckm|12715	-13.24861667	0.073170373	-13.24861667	0.073170373
Plat|18791	-13.72008333	0.018187122	-9.44656	0.017457321
Scai|320271	-13.75065	0.000811755	-16.30078	4.47165E-06
Btc|12223	-13.88365	0.02663959	-8.66038	0.015258085
Lef1|16842	-13.93316667	0.026152107	-8.7198	0.014875607
Myh7|140781	-13.99831667	0.170446566	1	#DIV/0!
Wnt2b|22414	-14.76428333	0.012568228	-10.6996	0.012963072
)TTMUSG00000016609|66803	-14.85891667	0.122797642	-3.8707	0.148332518
P4ha3|320452	-15.01008333	0.052796595	-7.48578	0.016276578
Sema3d|108151	-15.4188	0.055611868	-8.50256	0.083078807
Dclk2|70762	-15.71263333	0.002211314	-15.71263333	0.002211314
Lypd1|72585	-16.91820917	0.097732253	-14.35212	0.151447022
Sssca1|56390	-17.79908085	0.076432971	-80.87961667	0.088053685
Cand2|67088	-18.02148333	0.002582566	-18.02148333	0.002582566
Cox6a2|12862	-18.19798333	0.075056505	-7.69616	0.053485125
Actc1|11464	-19.15151667	0.122312941	-4.8	0.148332518
Cyp2c55|72082	-19.94473333	0.019176112	-13.40736	0.017382437
Prrt2|69017	-20.3375	0.027087757	-20.3375	0.027087757
Myl3|17897	-21.66613333	0.098140694	-7.81754	0.148332518
Rnaset2a|100037283	-22.11666667	0.108584867	-6.8	0.148332518
Actg2|11468	-24.22515	0.170446566	1	#DIV/0!
Spna1|20739	-24.87213333	0.011000808	-24.87213333	0.011000808
Barhl1|54422	-27.67323333	0.001556458	-33.00788	4.79011E-05
Aard|239435	-27.97458333	0.000616649	-27.97458333	0.000616649
F830016B08Rik|240328	-37.69991667	0.016287056	-23.2399	6.55023E-05
Myl2|17906	-55.50071667	0.157784995	-3.9746	0.148332518
Myh6|17888	-55.97699429	0.154502989	-13.73766	0.026725499

Supplementary Table 2.

Category	Term	Count	%	PValue	Genes	List Total	Pop Hits	Pop Total	Fold Enrichment	Bonferroni	Benjamini	FDR
SP_PIR_KEYWORDS	lipid synthesis	14	2.36	5.51E-06	SCD1, SCD2, ACACA, FDPS, LSS, SC4MOL, AWAT2, FAR2, AKR1C6, FASN, MVK, ELOVL6, IDI1, NSDHL	518	99	17854	4.87414687	0.0019	0.001934	0.0075
SP_PIR_KEYWORDS	alternative splicing	174	29.3	9.58E-06	[illegible] ZC3HAV1, LTBP3, INO80, GPAT2, BC018242, MAP3K4, ANK3, SBSN, SLC4A3, 1700029G01RIK, MATK, SLC12A7, POU2AF1, MYLK3, SLC22A23, RBL1, MLXIPL, OLA1, WDR90, BCL2L11, LAT2, SSTR2, TRIM35, TNNT1, SGSM1, PITPNM3, ZFP280D, 6530402F18RIK, BMP2K, MST1R, PGCP, TDRD3, AB124611, BBS7, A730008H23RIK, ASAP1, BDKRB2, DENND2C, SEMA5B, PVRL4, TRP53BP1, MEIS3, PDE6C, TTBK2, GMIP, SPATS2, PVRL3, CEP170, RCCD1, STAMBPL1, D330028D13RIK, OLFM1, 6030446N20RIK, FGD4, FN1, SGIP1, SREBF1, REEP6, OBSCN, AGBL3, ABCB9, CSTF2, AK1, TNFRSF13C, PPAPDC1B, HACE1, HGF, SHANK2, CCDC158, MMAB, FOXP2, AWAT2, ABCG8, SEMA6A, GGT6, SAP130, FREM1, CCDC151, MYO19, RGS9, COL20A1, ARSB, NRP2, PRPH, POU6F1, ELF3, AGTPBP1, CDC14B, BCAR1, PION, BC052040, ACOT3, TTLL11, FAR2, SRPX, SERAC1, MFAP1B, HOMEZ, MTBP, SPIB, ZC3HAV1L, RTEL1, CCDC88B, SP100, FBXL21, PRG4, NUP88, ACNAT1, GPRASP1, SCAI,	518	4481	17854	1.33838196	0.0034	0.001679	0.0131
KEGG_PATHWAY	mmu00982: Drug metabolism	11	1.85	5.12E-05	CYP2A22, CYP2C55, CYP2C44, ADH1, UGT1A5, MAOA, AOX1, CYP2A5, GSTT1, CYP2B13, GSTM6	167	75	5738	5.03936128	0.0068	0.006787	0.0599
GOTERM_BP_FAT	GO:0055114 ~oxidation reduction	38	6.41	6.37E-05	[illegible] PYROXD1, CYP2D11, LDHD, SC4MOL, FAR2, ADH1, AKR1C6, FASN, MICAL1, KDM3A, NSDHL, CYP2G1, SCD1, CHDH, CYP2C55, SCD2, CYP2D40, MAOA, CYP4F14, CYP2B13, CYB5B, GMPR, AKR1C20, TET1, HAO1, ABP1, CYP2A22, DHFR, SQLE, AKR1C19, AOX1, CYP2A5, AOX3, LEPREL1, AKR1D1	381	672	13588	2.01671666	0.1087	0.108696	0.1082

INTERPRO	IPR011993:Pleckstrin homology-type	22	3.71	1.05E-04	ASAP1, E130306D19RIK, NUMBL, PRKD2, PLEKHA6, PLEKHG2, ADAP2, DOK3, DOK4, DAPP1, SH2D5, FAM109B, MFAP1B, DOK7, TBC1D4, SPRED3, EPB4.1L4B, PHLDB2, ARAP2, PRKD3, PLEKHA2, FGD4	513	289	17763	2.6358688	0.0912	0.091179	0.1637
GOTERM_BP_FAT	GO:0006694~steroid biosynthetic process	10	1.69	1.53E-04	HSD3B2, AKR1C6, FDPS, MVK, LSS, IDI1, AKR1D1, AKR1C20, SC4MOL, NSDHL	381	71	13588	5.02310451	0.2416	0.129158	0.26
KEGG_PATHWAY	mmu02010:ABC transporters	8	1.35	2.68E-04	ABCG8, ABCB9, ABCB1A, ABCD1, ABCA8A, ABCC4, ABCA2, ABCA3	167	45	5738	6.1083167	0.0351	0.017687	0.3133
SP_PIR_KEYWORDS	oxidoreductase	33	5.56	3.10E-04	HSD3B2, SORD, CYP2C44, CYP2D11, PYROXD1, LDHD, SC4MOL, FAR2, ADH1, AKR1C6, FASN, KDM3A, NSDHL, CYP2G1, SCD1, CHDH, CYP2C55, SCD2, CYP2D40, MAOA, CYP4F14, CYP2B13, GMPR, TET1, HAO1, ABP1, CYP2A22, DHFR, SQLE, AOX1, CYP2A5, LEPREL1, AKR1D1	518	572	17854	1.98849124	0.1032	0.035649	0.4236

GOTERM_MF_FAT	GO:0046872~metal ion binding	137	23.1	3.75E-04	[illegible] SYT3, CAR5A, SYT7, ATP2B2, MAP3K4, SLC25A23, RGN, ZFP831, ZFP882, DDAH1, SLC12A7, CYP2C55, NUDT13, NCALD, MYLK3, ACTN1, CYP2B13, TRIM35, PITPNM3, ZFP280D, PDE5A, PGCP, CYP2C44, SFXN4, ASAP1, ZFP456, ZFP455, GMIP, STAMBPL1, DHX58, FGD4, SGIP1, SCD1, AGBL3, SCD2, CYP2D40, ESRRB, EFEMP2, ACACA, TREX1, MYLPF, TET1, FOXP2, PRPSAP2, ADAP2, FREM1, MTR, MZF1, SLC13A3, ZFHX2, IDI1, KLF1, ARSB, PILRB2, ACVRL1, LDLR, AGTPBP1, MMP28, GLI3, SC4MOL, ZKSCAN4, GM14391, PLCB4, STAC2, PROZ, SETMAR, ITIH4, ZFP354A, MICAL1, ZC3HAV1L, RTEL1, ZFP354B, ATP8B4, APCS, CAR14, CYB5B, GMPR, PRKD2, ZFP867, PKLR, CYP2A5, ERN1, SPNA1, LEPREL1, PRKD3, ZFP418, ZFP710, ZFP395, SORD, ZFP715, USP3, ZFP69, CD248, ZFP398, TRIM14, PML, TPCN2, DPF1, ZDHHC24, NR1D1, TCEA3, ADH1, FASN, ADAMTS10, PCSK9, KDM3A, SLC39A5, XAF1, SCNN1A, CAR1, THBS3, CHD3, CAR3, CYP2G1, PM20D1,	367	3850	13288	1.28840794	0.204	0.204016	0.5537

GOTERM_MF_FAT	GO:0043169~cation binding	138	23.3	3.80E-04	[illegible] SYT3, CAR5A, SYT7, ATP2B2, MAP3K4, SLC25A23, RGN, ZFP831, ZFP882, DDAH1, SLC12A7, CYP2C55, NUDT13, NCALD, MYLK3, ACTN1, CYP2B13, TRIM35, PITPNM3, ZFP280D, PDE5A, PGCP, CYP2C44, SFXN4, ASAP1, ZFP456, ZFP455, GMIP, STAMBPL1, DHX58, FGD4, SGIP1, SCD1, AGBL3, SCD2, CYP2D40, ESRRB, EFEMP2, ACACA, TREX1, MYLPF, TET1, FOXP2, PRPSAP2, ADAP2, FREM1, MTR, MZF1, SLC13A3, ZFHX2, IDI1, KLF1, ARSB, PILRB2, ACVRL1, LDLR, AGTPBP1, MMP28, GLI3, SC4MOL, ZKSCAN4, GM14391, PLCB4, STAC2, PROZ, SETMAR, ITIH4, ZFP354A, MICAL1, ZC3HAV1L, RTEL1, ZFP354B, ATP8B4, APCS, CAR14, CYB5B, GMPR, PRKD2, ZFP867, PKLR, CYP2A5, ERN1, SPNA1, LEPREL1, PRKD3, ZFP418, ZFP710, ZFP395, SORD, ZFP715, USP3, ZFP69, CD248, ZFP398, TRIM14, PML, TPCN2, DPF1, ZDHHC24, NR1D1, TCEA3, ADH1, FASN, ADAMTS10, PCSK9, KDM3A, SLC39A5, XAF1, SCNN1A, CAR1, THBS3, CHD3, CAR3, CYP2G1, PM20D1,	367	3885	13288	1.28612037	0.2064	0.109173	0.5611
INTERPRO	IPR008067:Cytochrome P450, E-class, group I, CYP2A-like	4	0.67	4.46E-04	CYP2G1, CYP2A22, CYP2C44, CYP2A5	513	6	17763	23.0838207	0.3341	0.183964	0.6944

GOTERM_MF_FAT	GO:0043167~ion binding	139	23.4	4.54E-04	CYP2D11, SYT3, CAR5A, SYT7, ATP2B2, MAP3K4, SLC25A23, RGN, ZFP831, ZFP882, DDAH1, SLC12A7, CYP2C55, NUDT13, NCALD, MYLK3, ACTN1, CYP2B13, TRIM35, PITPNM3, ZFP280D, PDE5A, PGCP, CYP2C44, SFXN4, ASAP1, ZFP456, ZFP455, GMIP, STAMBPL1, DHX58, FGD4, SGIP1, SCD1, AGBL3, SCD2, CYP2D40, ESRRB, EFEMP2, ACACA, TREX1, MYLPF, TET1, FOXP2, PRPSAP2, ADAP2, FREM1, MTR, MZF1, SLC13A3, ZFHX2, IDI1, KLF1, ARSB, PILRB2, ACVRL1, LDLR, AGTPBP1, MMP28, GLI3, SC4MOL, ZKSCAN4, GM14391, PLCB4, STAC2, PROZ, SETMAR, ITIH4, ZFP354A, MICAL1, ZC3HAV1L, RTEL1, ZFP354B, ATP8B4, APCS, CAR14, CYB5B, GMPR, PRKD2, ZFP867, PKLR, CYP2A5, ERN1, SPNA1, LEPREL1, PRKD3, ZFP418, ZFP710, ZFP395, SORD, ZFP715, USP3, ZFP69, CD248, ZFP398, TRIM14, PML, TPCN2, DPF1, ZDHHC24, NR1D1, TCEA3, ADH1, FASN, ADAMTS10, PCSK9, KDM3A, SLC39A5, XAF1, SCNN1A, CAR1, THBS3, CHD3, CAR3, CYP2G1, PM20D1,	367	3934	13288	1.27930471	0.2414	0.087982	0.6701
INTERPRO	IPR017871:ABC transporter, conserved site	9	1.52	4.84E-04	ABCG8, ABP1, ABCB9, ABCB1A, ABCD1, ABCA8A, ABCC4, ABCA2, ABCA3	513	64	17763	4.86924342	0.3564	0.136623	0.7524
GOTERM_BP_FAT	GO:0008202~steroid metabolic process	14	2.36	5.94E-04	SREBF1, HSD3B2, LDLR, FDPS, ABCA2, LSS, AKR1C20, SC4MOL, AKR1C6, PCSK9, MVK, IDI1, AKR1D1, NSDHL	381	161	13588	3.10122104	0.6576	0.300385	1.0035
INTERPRO	IPR003439:ABC transporter-like	8	1.35	7.76E-04	ABCG8, ABCB9, ABCB1A, ABCD1, ABCA8A, ABCC4, ABCA2, ABCA3	513	53	17763	5.22652543	0.5069	0.162027	1.2044
PIR_SUPERFAMILY	PIRSF005552:guanine nucleotide-binding protein 1	4	0.67	8.63E-04	GBP6, GBP10, GBP2, GBP1	211	8	8136	19.2796209	0.1779	0.177896	1.0979
INTERPRO	IPR002401:Cytochrome P450, E-class, group I	9	1.52	8.87E-04	CYP2G1, CYP2A22, CYP2C55, CYP2D40, CYP2C44, CYP2D11, CYP2A5, CYP4F14, CYP2B13	513	70	17763	4.4518797	0.5546	0.149362	1.3766

SP_PIR_KEYWORDS	Steroid biosynthesis	7	1.18	9.52E-04	AKR1C6, FDPS, MVK, LSS, IDI1, SC4MOL, NSDHL	518	40	17854	6.03175676	0.2841	0.080173	1.2946
GOTERM_BP_FAT	GO:0008610~lipid biosynthetic process	19	3.2	0.001125	SCD1, HSD3B2, SCD2, ACACA, FDPS, LSS, GPAT2, AKR1C20, SC4MOL, AWAT2, FAR2, ACSM1, AKR1C6, FASN, MVK, ELOVL6, IDI1, AKR1D1, NSDHL	381	285	13588	2.3776028	0.869	0.398389	1.8947
SP_PIR_KEYWORDS	iron	21	3.54	0.001143	CYP2G1, SCD1, CYP2C55, SCD2, CYP2D40, CYP2C44, CYP2D11, CYP2B13, SFXN4, CYP4F14, CYB5B, TET1, SC4MOL, CYP2A22, AOX1, CYP2A5, AOX3, KDM3A, LEPREL1, CYB5D1, RTEL1	518	321	17854	2.25486234	0.3307	0.077155	1.5528
GOTERM_MF_FAT	GO:0016712~oxidoreductase activity, acting on paired donors, with incorporation or reduction of molecular oxygen, reduced flavin or flavoprotein as one donor, and incorporation of one atom of oxygen	7	1.18	0.001221	CYP2G1, CYP2A22, CYP2C55, CYP2C44, CYP2D11, CYP2A5, CYP2B13	367	44	13288	5.76021798	0.5244	0.16954	1.7921
GOTERM_MF_FAT	GO:0005506~iron ion binding	21	3.54	0.001352	CYP2G1, SCD1, CYP2C55, SCD2, CYP2D40, CYP2C44, CYP2D11, CYP2B13, SFXN4, CYP4F14, CYB5B, TET1, SC4MOL, CYP2A22, AOX1, CYP2A5, AOX3, KDM3A, LEPREL1, CYB5D1, RTEL1	367	343	13288	2.21676027	0.5608	0.151714	1.9821
GOTERM_BP_FAT	GO:0016125~sterol metabolic process	9	1.52	0.001359	SREBF1, LDLR, FDPS, PCSK9, ABCA2, MVK, IDI1, SC4MOL, NSDHL	381	77	13588	4.16852439	0.9141	0.387912	2.2834

SP_PIR_KEYWORDS	glycoprotein	133	22.4	0.001563	[illegible] SLC44A3, AU040320, LTBP3, AQP8, LRTM1, BTC, TNFSF13, CD53, TLR7, KLHDC7A, GHRHR, OLFML3, BC018242, SLC2A2, S1PR5, VNN1, SLC4A3, SLC12A7, SLC22A23, TMEM132A, TMEM132E, SSTR2, CD37, RELT, ABCB1A, 6530402F18RIK, MST1R, PGCP, GLG1, AB124611, ABCA2, BDKRB2, SEMA5B, PVRL4, FOLR2, PVRL3, ICOS, OLFM1, FN1, PLAT, FKTN, CD8B1, EFEMP2, MET, CD300E, TNFRSF13C, HGF, FCGR1, SHANK2, LCN2, ABCG8, SEMA6A, GGT6, LAMA4, GGT7, LTB4R1, FREM1, SLC13A3, CHRD, COL20A1, ARSB, NRP2, PILRB2, NRP1, PODNL1, ACVRL1, LDLR, GYPA, LRRC19, SERPINA3F, PAX5, GABBR2, RAET1D, DNAJC16, SERPINA7, PROZ, SLC22A1, GPR97, PARM1, APCS, CAR14, CMKLR1, PRG4, IGFALS, CHST3, NFAM1, TMEM67, AMIGO3, CTSO, ERN1, NGFR, LEPREL1, FMOD, GPR183, HS3ST6, CSF1, CD248, ABCD1, GPR64, FPR1, CXCL9, COL27A1, UGT1A5, ADAMTS10, PCSK9, PKD1, SUCNR1, POPDC2, SLC39A5, SELPLG, SCNN1A, THBS3,	518	3600	17854	1.27337087	0.4225	0.087447	2.1176
INTERPRO	IPR001849:Pleckstrin homology	18	3.04	0.001573	OBSCN, ASAP1, TECPR1, E130306D19RIK, PRKD2, PLEKHA6, PLEKHG2, ADAP2, DOK3, DOK4, DAPP1, FAM109B, DOK7, PHLDB2, ARAP2, PRKD3, PLEKHA2, FGD4	513	261	17763	2.38798145	0.7617	0.212618	2.4279
INTERPRO	IPR003191:Guanylate-binding protein, C-terminal	4	0.67	0.001756	GBP6, GBP10, GBP2, GBP1	513	9	17763	15.3892138	0.7984	0.20451	2.7073
GOTERM_BP_FAT	GO:0006633~fatty acid biosynthetic process	9	1.52	0.001886	SCD1, AWAT2, FAR2, ACSM1, SCD2, FASN, ACACA, ELOVL6, SC4MOL	381	81	13588	3.96267133	0.9669	0.433292	3.156
GOTERM_BP_FAT	GO:0016053~organic acid biosynthetic process	12	2.02	0.002026	SCD1, AWAT2, FAR2, ACSM1, SCD2, DHFR, MTR, FASN, ACACA, ELOVL6, AKR1D1, SC4MOL	381	141	13588	3.03523762	0.9743	0.407207	3.3862
GOTERM_BP_FAT	GO:0046394~carboxylic acid biosynthetic process	12	2.02	0.002026	SCD1, AWAT2, FAR2, ACSM1, SCD2, DHFR, MTR, FASN, ACACA, ELOVL6, AKR1D1, SC4MOL	381	141	13588	3.03523762	0.9743	0.407207	3.3862

GOTERM_CC_FAT	GO:0005626~insoluble fraction	27	4.55	0.002079	CYP2D11, BCAR1, PML, GHRHR, ATP2B2, PLCB4, FOLR2, RAC2, ANK3, UGT1A5, SCNN1A, CYP2C55, ANKRA2, MET, IGFALS, CYP4F14, CYB5B, BCL2L11, SLC10A1, LIN7A, CYP2A22, SSTR2, SQLE, CYP2A5, CYFIP2, ABCC4, SLC13A3	336	528	12504	1.90300325	0.437	0.436994	2.7086
PIR_SUPERFAMILY	PIRSF000045:cytochrome P450 CYP2D6	6	1.01	0.002146	CYP2G1, CYP2A22, CYP2C55, CYP2D11, CYP2A5, CYP2B13	211	36	8136	6.42654028	0.3859	0.216338	2.7104
SMART	SM00233:PH	18	3.04	0.002199	OBSCN, ASAP1, TECPR1, E130306D19RIK, PRKD2, PLEKHA6, PLEKHG2, ADAP2, DOK3, DOK4, DAPP1, FAM109B, DOK7, PHLDB2, ARAP2, PRKD3, PLEKHA2, FGD4	275	261	9131	2.28990596	0.3389	0.338852	2.6937
INTERPRO	IPR017973:Cytochrome P450, C-terminal region	9	1.52	0.002297	CYP2G1, CYP2A22, CYP2C55, CYP2D40, CYP2C44, CYP2D11, CYP2A5, CYP4F14, CYP2B13	513	81	17763	3.84730344	0.8769	0.230381	3.5264
UP_SEQ_FEATURE	splice variant	168	28.3	0.002595	[illegible] ZC3HAV1, LTBP3, INO80, GPAT2, BC018242, MAP3K4, SBSN, SLC4A3, 1700029G01RIK, MATK, SLC12A7, MYLK3, SLC22A23, RBL1, MLXIPL, OLA1, WDR90, BCL2L11, LAT2, SSTR2, TRIM35, TNNT1, SGSM1, PITPNM3, ZFP280D, 6530402F18RIK, BMP2K, PGCP, TDRD3, AB124611, BBS7, A730008H23RIK, ASAP1, BDKRB2, DENND2C, SEMA5B, PVRL4, TRP53BP1, MEIS3, PDE6C, TTBK2, GMIP, SPATS2, PVRL3, CEP170, RCCD1, STAMBPL1, D330028D13RIK, OLFM1, 6030446N20RIK, FGD4, SGIP1, SREBF1, REEP6, OBSCN, AGBL3, ABCB9, CSTF2, AK1, TNFRSF13C, PPAPDC1B, ACACA, HACE1, HGF, SHANK2, CCDC158, MMAB, FOXP2, AWAT2, ABCG8, SEMA6A, GGT6, SAP130, FREM1, CCDC151, MYO19, RGS9, COL20A1, ARSB, NRP2, PRPH, POU6F1, ELF3, AGTPBP1, CDC14B, BCAR1, PION, BC052040, ACOT3, TTLL11, FAR2, SRPX, SERAC1, MFAP1B, HOMEZ, MTBP, SPIB, ZC3HAV1L, RTEL1, CCDC88B, SP100, FBXL21, PRG4, NUP88, ACNAT1, GPRASP1, SCAI, NUSAP1, LEF1, MPP7,	500	4448	16021	1.21021942	0.9826	0.982628	4.2445

GOTERM_CC_FAT	GO:0005624~membrane fraction	26	4.38	0.002631	CYP2D11, BCAR1, GHRHR, ATP2B2, PLCB4, FOLR2, RAC2, ANK3, UGT1A5, SCNN1A, CYP2C55, ANKRA2, MET, IGFALS, CYP4F14, CYB5B, BCL2L11, SLC10A1, LIN7A, CYP2A22, SSTR2, SQLE, CYP2A5, CYFIP2, ABCC4, SLC13A3	336	510	12504	1.89719888	0.5167	0.304789	3.4158
INTERPRO	IPR000483:Cysteine-rich flanking region, C-terminal	9	1.52	0.00268	FLRT1, LRRN4, GPR125, LRG1, LRTM1, LRRC19, IGFALS, PKD1, TLR7	513	83	17763	3.75459734	0.9132	0.237849	4.1026
GOTERM_BP_FAT	GO:0030879~mammary gland development	9	1.52	0.002758	ATP2B2, ELF3, CSF1, MET, PML, LEF1, GLI3, BCL2L11, GHRHR	381	86	13588	3.73228346	0.9932	0.463772	4.5836
GOTERM_MF_FAT	GO:0046906~tetrapyrrole binding	12	2.02	0.003075	CYP2G1, CYP2A22, CYP2C55, CYP2D40, CYP2C44, CYP2D11, MTR, CYP2A5, CYP4F14, CYP2B13, CYB5B, CYB5D1	367	151	13288	2.8773842	0.8463	0.268071	4.4544
GOTERM_MF_FAT	GO:0016765~transferase activity, transferring alkyl or aryl (other than methyl) groups	7	1.18	0.003229	CHML, MTR, FDPS, GSTT1, GSTT3, GSTM6, MMAB	367	53	13288	4.78206776	0.86	0.244902	4.6725
KEGG_PATHWAY	mmu00830:Retinol metabolism	8	1.35	0.003284	AWAT2, CYP2A22, CYP2C55, CYP2C44, ADH1, UGT1A5, CYP2A5, CYP2B13	167	68	5738	4.0422684	0.3543	0.135694	3.7732
GOTERM_BP_FAT	GO:0008203~cholesterol metabolic process	8	1.35	0.00334	SREBF1, LDLR, FDPS, PCSK9, ABCA2, MVK, IDI1, NSDHL	381	70	13588	4.07589051	0.9976	0.488773	5.5245
SMART	SM00082:LRRCT	9	1.52	0.003341	FLRT1, LRRN4, GPR125, LRG1, LRTM1, LRRC19, IGFALS, PKD1, TLR7	275	83	9131	3.6003943	0.467	0.26992	4.0673
INTERPRO	IPR001128:Cytochrome P450	9	1.52	0.003594	CYP2G1, CYP2A22, CYP2C55, CYP2D40, CYP2C44, CYP2D11, CYP2A5, CYP4F14, CYP2B13	513	87	17763	3.58197217	0.9624	0.279612	5.4654

GOTERM_MF_FAT	GO:0046914~transition metal ion binding	94	15.9	0.003633	[illegible] AGTPBP1, CYP2D11, CAR5A, MMP28, GLI3, ZKSCAN4, SC4MOL, GM14391, STAC2, SETMAR, ZFP354A, ZFP831, MICAL1, ZC3HAV1L, ZFP882, DDAH1, ZFP354B, RTEL1, CYP2C55, CAR14, CYP2B13, CYB5B, PRKD2, TRIM35, ZFP867, ZFP280D, PDE5A, CYP2A5, PGCP, LEPREL1, PRKD3, ZFP710, ZFP418, ZFP395, SORD, USP3, ZFP715, CYP2C44, ZFP69, ZFP398, TRIM14, PML, ASAP1, SFXN4, ZFP456, ZFP455, DPF1, ZDHHC24, GMIP, NR1D1, TCEA3, ADH1, FASN, STAMBPL1, ADAMTS10, KDM3A, SLC39A5, XAF1, CAR1, DHX58, CAR3, FGD4, CHD3, SGIP1, CYP2G1, SCD1, AGBL3, SCD2, CYP2D40, ESRRB, PM20D1, 4931406C07RIK, ACACA, CYP4F14, XPNPEP3, TET1, FOXP2, CYP2A22, ABP1, ADAP2, ZFP182, MTR, AOX1, ZFP7, MZF1, AOX3, LIME1, ZFHX2, CYB5D1, KLF1, ARAP2	367	2608	13288	1.30500995	0.8906	0.241678	5.2434

SP_PIR_KEYWORDS	membrane	189	31.9	0.003725	[illegible] AU040320, GPR125, SLC44A3, AQP8, LRTM1, CYP2D11, BTC, SYT3, CD209F, INTS1, TNFSF13, SYT7, CD53, GPAT2, TLR7, KLHDC7A, GHRHR, SLC35A4, ATP2B2, BC018242, SLC25A23, SLC2A2, S1PR5, SPRED3, VNN1, ELOVL6, TMEM185B, SLC4A3, TMEM14A, SLC12A7, CYP2C55, GOLT1B, TMIE, SLC22A23, TMEM132A, TMEM132E, BCL2L11, RND2, LAT2, SSTR2, CD37, PITPNM3, RELT, ABCB1A, 6530402F18RIK, TMEM184A, MST1R, GBP2, GBP1, GLG1, AB124611, TMEM199, BBS7, RABEPK, SFXN4, ASAP1, ABCA2, BDKRB2, ABCA3, SEMA5B, PVRL4, PDE6C, FOLR2, TTBK2, RAC2, PVRL3, ICOS, SLC35F2, 6030446N20RIK, BLNK, SREBF1, SCD1, REEP6, ABCB9, PRAF2, FKTN, SCD2, CD8B1, MAOA, MET, DENND1A, CD300E, TNFRSF13C, PPAPDC1B, REEP1, SHANK2, FCGR1, LIN7A, AWAT2, ABCG8, SEMA6A, GGT6, GGT7, ADAP2, FAM151A, LTB4R1, SLC13A3, RGS9, PLEKHA2, NRP2, GYPC, PILRB2, NRP1, ACVRL1, CLDN4, LDLR, GYPA, LRRC19, GABBR2,	518	5507	17854	1.18291217	0.7301	0.170649	4.9776
GOTERM_MF_FAT	GO:0001883~purine nucleoside binding	61	10.3	0.003807	KIFC2, XRCC3, ACVRL1, LDHD, INO80, ATP2B2, ACSS1, MAP3K4, DDX11, OASL1, TARSL2, RTEL1, ATP8B4, MATK, NLRP6, NMNAT2, MYLK3, OLA1, TYK2, PRKD2, PAPD4, ABCB1A, SQLE, PDE5A, PKLR, ERN1, GM6524, BMP2K, MVK, MST1R, PRKD3, GBP1, ABCD1, ABCA2, OAS2, ABCA3, TTBK2, DCLK3, DCLK2, DHX58, CHD3, OBSCN, CHDH, ABCB9, AK1, MET, ABCA8A, ACACA, TREX1, BCS1L, NPR2, MMAB, ABCG8, ACSM1, AOX1, ABCC4, AOX3, GRK4, MYO19, JAK3, PIP4K2A	367	1548	13288	1.42676496	0.9016	0.227143	5.487
SP_PIR_KEYWORDS	liver	5	0.84	0.003972	AKR1C6, LTBP3, FABP1, CAR5A, ORM2	518	23	17854	7.49286554	0.7527	0.160238	5.3005

INTERPRO	IPR017972:Cytochrome P450, conserved site	9	1.52	0.004133	CYP2G1, CYP2A22, CYP2C55, CYP2D40, CYP2C44, CYP2D11, CYP2A5, CYP4F14, CYP2B13	513	89	17763	3.50147842	0.977	0.290339	6.2607
INTERPRO	IPR003591:Leucine-rich repeat, typical subtype	11	1.85	0.004185	FMOD, AMIGO3, FLRT1, PODNL1, LRRN4, GPR125, LRG1, LRTM1, IGFALS, PKD1, TLR7	513	129	17763	2.95258171	0.9781	0.272693	6.3381
GOTERM_MF_FAT	GO:0001882~nucleoside binding	61	10.3	0.00432	KIFC2, XRCC3, ACVRL1, LDHD, INO80, ATP2B2, ACSS1, MAP3K4, DDX11, OASL1, TARSL2, RTEL1, ATP8B4, MATK, NLRP6, NMNAT2, MYLK3, OLA1, TYK2, PRKD2, PAPD4, ABCB1A, SQLE, PDE5A, PKLR, ERN1, GM6524, BMP2K, MVK, MST1R, PRKD3, GBP1, ABCD1, ABCA2, OAS2, ABCA3, TTBK2, DCLK3, DCLK2, DHX58, CHD3, OBSCN, CHDH, ABCB9, AK1, MET, ABCA8A, ACACA, TREX1, BCS1L, NPR2, MMAB, ABCG8, ACSM1, AOX1, ABCC4, AOX3, GRK4, MYO19, JAK3, PIP4K2A	367	1558	13288	1.41760729	0.9281	0.231444	6.2053
SP_PIR_KEYWORDS	sterol biosynthesis	5	0.84	0.004659	FDPS, MVK, IDI1, SC4MOL, NSDHL	518	24	17854	7.18066281	0.8059	0.166514	6.1902
SMART	SM00369:LRR_TYP	11	1.85	0.005356	FMOD, AMIGO3, FLRT1, PODNL1, LRRN4, GPR125, LRG1, LRTM1, IGFALS, PKD1, TLR7	275	129	9131	2.83131783	0.6356	0.285749	6.4454
GOTERM_CC_FAT	GO:0000267~cell fraction	28	4.72	0.005404	SORD, CYP2D11, BCAR1, PML, GHRHR, ATP2B2, PLCB4, RAC2, FOLR2, ANK3, UGT1A5, SCNN1A, CYP2C55, ANKRA2, MET, IGFALS, CYP4F14, CYB5B, BCL2L11, SLC10A1, LIN7A, CYP2A22, SSTR2, SQLE, CYP2A5, CYFIP2, ABCC4, SLC13A3	336	596	12504	1.74832215	0.7759	0.392556	6.899
INTERPRO	IPR015894:Guanylate-binding protein, N-terminal	4	0.67	0.005488	GBP6, GBP10, GBP2, GBP1	513	13	17763	10.6540711	0.9934	0.319977	8.2325

GOTERM_CC_FAT	GO:0005886~plasma membrane	99	16.7	0.005852	[illegible] GABRB3, AQP8, BTC, SYT3, TNFSF13, SYT7, GHRHR, ATP2B2, SLC2A2, S1PR5, VNN1, SPRED3, ACTN1, RND2, SSTR2, LAT2, ABCB1A, RELT, 6530402F18RIK, MST1R, GBP2, GBP1, GLG1, BBS7, BDKRB2, ABCA3, PVRL4, PDE6C, FOLR2, ICOS, PVRL3, FN1, BLNK, CD8B1, AK1, MAOA, MET, TNFRSF13C, CD300E, DENND1A, FCGR1, SHANK2, LIN7A, ABCG8, ADAP2, LTB4R1, CYFIP2, KCTD18, RGS9, PLEKHA2, NRP2, GYPC, NRP1, CLDN4, BCAR1, GYPA, GABBR2, FAM57A, RAET1D, AP1S2, ITIH4, SLC22A1, GPR97, COL23A1, CMKLR1, MPP7, RFTN2, NFAM1, SLC9A3R1, TMEM67, DOK3, EVPL, DOK7, GM6524, KCTD12B, NGFR, GPR183, CD248, CSF1, GPR64, FPR1, UGT1A5, PKD1, SUCNR1, SLC39A5, SELPLG, SCNN1A, CNNM4, DLGAP3, SELL, ABCA8A, NPR2, GPR77, ABCC4, LIME1, GFRA2	336	2906	12504	1.26779569	0.8021	0.332998	7.4508
GOTERM_MF_FAT	GO:0020037~heme binding	11	1.85	0.00652	CYP2G1, CYP2A22, CYP2C55, CYP2D40, CYP2C44, CYP2D11, CYP2A5, CYP4F14, CYP2B13, CYB5B, CYB5D1	367	144	13288	2.76581895	0.9813	0.303398	9.2246
PIR_SUPERFAMILY	PIRSF001392:carbonate dehydratase	4	0.67	0.007405	CAR14, CAR5A, CAR1, CAR3	211	16	8136	9.63981043	0.815	0.430166	9.071
GOTERM_MF_FAT	GO:0030554~adenyl nucleotide binding	59	9.95	0.007428	LDHD, INO80, ATP2B2, ACSS1, MAP3K4, DDX11, OASL1, TARSL2, RTEL1, ATP8B4, MATK, NLRP6, NMNAT2, MYLK3, OLA1, TYK2, PRKD2, PAPD4, ABCB1A, SQLE, PKLR, ERN1, GM6524, BMP2K, MVK, MST1R, PRKD3, ABCD1, ABCA2, OAS2, ABCA3, TTBK2, DCLK3, DCLK2, DHX58, CHD3, OBSCN, CHDH, ABCB9, AK1, MET, ABCA8A, ACACA, TREX1, BCS1L, NPR2, MMAB, ABCG8, ACSM1, AOX1, ABCC4, AOX3, GRK4, MYO19, JAK3, PIP4K2A	367	1535	13288	1.39167295	0.9893	0.3146	10.445

SP_PIR_KEYWORDS	metal-binding	99	16.7	0.007876	ZC3HAV1, CYP2D11, SYT3, CAR5A, SYT7, ATP2B2, MAP3K4, ZFP882, DDAH1, CYP2C55, CYP2B13, TRIM35, PITPNM3, ZFP280D, PDE5A, PGCP, CYP2C44, ASAP1, ZFP456, ZFP455, PDE6C, GMIP, STAMBPL1, DHX58, FGD4, SGIP1, AGBL3, CYP2D40, ESRRB, ACACA, FDPS, TET1, FOXP2, ADAP2, FREM1, MTR, MZF1, IDI1, KLF1, ARSB, PILRB2, ACVRL1, AGTPBP1, MMP28, GLI3, ZKSCAN4, STAC2, GM14391, ZFP354A, MICAL1, ZC3HAV1L, ZFP354B, RTEL1, APCS, CAR14, CYB5B, GMPR, PRKD2, ZFP867, PKLR, ERN1, CYP2A5, LEPREL1, PRKD3, ZFP710, ZFP418, SORD, USP3, ZFP715, ZFP69, ZFP398, TRIM14, PML, DPF1, ZDHHC24, NR1D1, TCEA3, ADH1, ADAMTS10, KDM3A, XAF1, CAR1, CHD3, CAR3, CYP2G1, PM20D1, 4931406C07RIK, CYP4F14, CYP2A22, ABP1, ACSM1, ZFP182, AOX1, ZFP7, SULF1, LIME1, AOX3, CYB5D1, ARAP2	518	2682	17854	1.27227851	0.9377	0.242352	10.254
GOTERM_CC_FAT	GO:0019898~extrinsic to membrane	23	3.88	0.00829	GNA14, CYP2C55, DLGAP3, ANKRA2, CYP2D11, DENND1A, CYP4F14, MPP7, SLC9A3R1, BCL2L11, LIN7A, CYP2A22, PITPNM3, DAPP1, MFAP1B, DOK7, CYP2A5, SPRED3, EPB4.1L4B, RGS9, JAK3, PHLDB2, PLEKHA2	336	472	12504	1.81340799	0.8995	0.368401	10.401
INTERPRO	IPR012675:Beta-grasp fold, ferredoxin-type	4	0.67	0.008365	AOX1, OLA1, AOX3, TARSL2	513	15	17763	9.23352827	0.9995	0.42109	12.291
SP_PIR_KEYWORDS	heme	11	1.85	0.008394	CYP2G1, CYP2A22, CYP2C55, CYP2D40, CYP2C44, CYP2D11, CYP2A5, CYP4F14, CYP2B13, CYB5B, CYB5D1	518	142	17854	2.66999293	0.9481	0.235839	10.893
GOTERM_BP_FAT	GO:0016126~sterol biosynthetic process	5	0.84	0.009284	FDPS, MVK, IDI1, SC4MOL, NSDHL	381	30	13588	5.944007	1	0.814306	14.654
GOTERM_BP_FAT	GO:0007167~enzyme linked receptor protein signaling pathway	16	2.7	0.010038	PLAT, ACVRL1, NRP1, NDN, LTBP3, BCAR1, MET, PML, HGF, DOK4, DCP1A, DOK7, MST1R, JAK3, FGF1, GFRA2	381	273	13588	2.09020026	1	0.809002	15.75

INTERPRO	IPR002345:Lipocalin	6	1.01	0.01011	LCN2, ORM1, MEIS3, GPRASP1, ABCA2, ORM2	513	46	17763	4.51639969	0.9999	0.460507	14.669
GOTERM_MF_FAT	GO:0009055~electron carrier activity	13	2.19	0.010304	CYP2G1, HAO1, CYP2A22, CYP2C55, CYP2D40, CYP2C44, CYP2D11, MAOA, AOX1, CYP2A5, CYP4F14, AOX3, CYP2B13	367	202	13288	2.3301589	0.9982	0.38395	14.209
GOTERM_MF_FAT	GO:0005158~insulin receptor binding	4	0.67	0.010576	DOK3, DOK4, SLC2A2, DOK7	367	17	13288	8.51931399	0.9984	0.369827	14.557
UP_SEQ_FEATURE	domain:PH	14	2.36	0.010819	OBSCN, ASAP1, TECPR1, E130306D19RIK, PRKD2, PLEKHG2, PLEKHA6, DOK3, DAPP1, DOK4, FAM109B, DOK7, PHLDB2, PRKD3	500	202	16021	2.22073267	1	0.999793	16.606
KEGG_PATHWAY	mmu00980:Metabolism of xenobiotics by cytochrome P450	7	1.18	0.011606	CYP2C55, CYP2C44, ADH1, UGT1A5, GSTT1, CYP2B13, GSTM6	167	66	5738	3.64416621	0.7883	0.321691	12.759
GOTERM_CC_FAT	GO:0042995~cell projection	26	4.38	0.011628	NRP2, PRPH, NRP1, BBS7, GABRB3, BCAR1, ABI3, GABBR2, GLI3, TTLL11, ATP2B2, NPHP4, IFT122, PLCB4, SRPX, ANK3, SLC2A2, PKD1, CYS1, FGD4, MET, DENND1A, SHANK2, SEMA6A, GM6524, GAMT	336	575	12504	1.68273292	0.9604	0.416099	14.299
INTERPRO	IPR018338:Carbonic anhydrase, alpha-class, conserved site	4	0.67	0.011979	CAR14, CAR5A, CAR1, CAR3	513	17	17763	8.14723082	1	0.496512	17.151
KEGG_PATHWAY	mmu00100:Steroid biosynthesis	4	0.67	0.011996	SQLE, LSS, SC4MOL, NSDHL	167	17	5738	8.08453681	0.7991	0.274595	13.161
GOTERM_MF_FAT	GO:0004089~carbonate dehydratase activity	4	0.67	0.012437	CAR14, CAR5A, CAR1, CAR3	367	18	13288	8.04601877	0.9995	0.397854	16.903
COG_ONTOLOGY	Secondary metabolites biosynthesis, transport, and catabolism	10	1.69	0.012737	CYP2G1, CYP2A22, CYP2C55, CYP2D40, CYP2C44, CYP4F17, CYP2D11, CYP2A5, CYP4F14, CYP2B13	73	109	2040	2.56378032	0.2162	0.216162	9.1598
INTERPRO	IPR018159:Spectrin/alpha-actinin	5	0.84	0.012945	BC024139, SYNE1, EVPL, SPNA1, ACTN1	513	32	17763	5.41027047	1	0.502544	18.407

GOTERM_MF_FAT	GO:0017076~purine nucleotide binding	68	11.5	0.013111	KIFC2, GNA14, XRCC3, ACVRL1, LDHD, INO80, ATP2B2, ACSS1, MAP3K4, DDX11, OASL1, GBP10, TARSL2, RTEL1, ATP8B4, MATK, NLRP6, NMNAT2, GBP6, MYLK3, OLA1, TYK2, RND2, PRKD2, PAPD4, ABCB1A, SQLE, PDE5A, PKLR, GM6524, ERN1, BMP2K, MVK, MST1R, PRKD3, GBP2, GBP1, ABCD1, ABCA2, OAS2, ABCA3, TTBK2, RAC2, DCLK3, DCLK2, DHX58, CHD3, OBSCN, CHDH, ABCB9, IRGM2, AK1, MET, ABCA8A, ACACA, TREX1, BCS1L, NPR2, MMAB, ABCG8, ACSM1, AOX1, ABCC4, AOX3, GRK4, MYO19, JAK3, PIP4K2A	367	1871	13288	1.31591755	0.9997	0.394397	17.74
SP_PIR_KEYWORDS	Monooxygenase	9	1.52	0.013204	CYP2G1, CYP2A22, CYP2C55, CYP2D40, CYP2C44, CYP2D11, CYP2A5, CYP4F14, CYP2B13	518	108	17854	2.87226512	0.9906	0.322122	16.629
GOTERM_BP_FAT	GO:0006955~immune response	23	3.88	0.013205	GPR183, XRCC4, GBP6, CD8B1, PRG4, CXCL9, CD300E, TNFRSF13C, SAMHD1, TNFSF13, OAS2, TLR7, FCGR1, TRAF3IP2, RAET1D, LAT2, OASL1, GBP10, VNN1, LIME1, DHX58, GBP2, GBP1	381	471	13588	1.74155619	1	0.864585	20.212
SP_PIR_KEYWORDS	Cholesterol biosynthesis	4	0.67	0.014248	FDPS, MVK, IDI1, NSDHL	518	18	17854	7.65937366	0.9935	0.321221	17.828
GOTERM_BP_FAT	GO:0055088~lipid homeostasis	5	0.84	0.014399	ABCG8, LDLR, ACACA, PCSK9, ABCA2	381	34	13588	5.24471206	1	0.866523	21.838
INTERPRO	IPR001611:Leucine-rich repeat	13	2.19	0.014534	FMOD, NLRC5, AMIGO3, FLRT1, PODNL1, LRRN4, GPR125, LRG1, LRTM1, LRRC19, IGFALS, PKD1, TLR7	513	202	17763	2.22838863	1	0.523354	20.433
SMART	SM00150:SPEC	5	0.84	0.014726	BC024139, SYNE1, EVPL, SPNA1, ACTN1	275	32	9131	5.18806818	0.9385	0.502064	16.812
GOTERM_CC_FAT	GO:0044421~extracellular region part	32	5.4	0.016222	FMOD, PODNL1, LDLR, LTBP3, CSF1, BTC, CXCL9, TNFSF13, MMP28, SERAC1, COL27A1, MFAP1B, PCSK9, VNN1, ADAMTS10, FGF1, OLFM1, FN1, PLAT, LGALS1, EFEMP2, IGFALS, WNT2B, ORM1, ABP1, LAMA4, FREM1, 6530402F18RIK, SULF1, CHRD, COL20A1, ORM2	336	774	12504	1.53857512	0.989	0.475258	19.408
INTERPRO	IPR018170:Aldo/keto reductase, conserved site	4	0.67	0.01636	AKR1C6, AKR1C19, AKR1D1, AKR1C20	513	19	17763	7.28962758	1	0.546566	22.704

INTERPRO	IPR001148:Carbonic anhydrase, alpha-class, catalytic domain	4	0.67	0.01636	CAR14, CAR5A, CAR1, CAR3	513	19	17763	7.28962758	1	0.546566	22.704
INTERPRO	IPR000372:Leucine-rich repeat, cysteine-rich flanking region, N-terminal	8	1.35	0.017902	FMOD, AMIGO3, FLRT1, PODNL1, LRTM1, IGFALS, PKD1, TLR7	513	93	17763	2.9785575	1	0.560816	24.574
UP_SEQ_FEATURE	glycosylation site:N-linked (GlcNAc...)	128	21.6	0.018267	SLC44A3, AU040320, LTBP3, AQP8, LRTM1, BTC, TNFSF13, CD53, TLR7, KLHDC7A, GHRHR, OLFML3, BC018242, SLC2A2, S1PR5, VNN1, SLC4A3, SLC12A7, SLC22A23, TMEM132A, TMEM132E, SSTR2, CD37, RELT, ABCB1A, 6530402F18RIK, MST1R, PGCP, GLG1, AB124611, ABCA2, BDKRB2, PVRL4, SEMA5B, FOLR2, PVRL3, ICOS, OLFM1, FN1, PLAT, FKTN, CD8B1, EFEMP2, MET, TNFRSF13C, CD300E, HGF, FCGR1, LCN2, ABCG8, SEMA6A, GGT6, LAMA4, GGT7, LTB4R1, FREM1, SLC13A3, CHRD, COL20A1, NRP2, ARSB, PILRB2, NRP1, PODNL1, ACVRL1, LDLR, LRRC19, SERPINA3F, GABBR2, RAET1D, DNAJC16, SERPINA7, PROZ, SLC22A1, GPR97, PARM1, APCS, CAR14, CMKLR1, PRG4, IGFALS, CHST3, NFAM1, TMEM67, AMIGO3, CTSO, ERN1, NGFR, LEPREL1, FMOD, GPR183, HS3ST6, CSF1, ABCD1, GPR64, FPR1, CXCL9, COL27A1, UGT1A5, ADAMTS10, PCSK9, PKD1, SUCNR1, POPDC2, SLC39A5, SELPLG, SCNN1A, THBS3, CNNM4, TMC8, LRRN4, SELL, ABCA8A, PM20D1, NPR2,	500	3444	16021	1.19087573	1	0.999931	26.492
KEGG_PATHWAY	mmu05340: Primary immunodeficiency	5	0.84	0.019357	CD8B1, ICOS, TNFRSF13C, JAK3, BLNK	167	36	5738	4.77212242	0.9257	0.35162	20.431
GOTERM_CC_FAT	GO:0031012~extracellular matrix	16	2.7	0.019398	EFEMP2, LGALS1, MMP28, WNT2B, LAMA4, SERAC1, FREM1, COL27A1, MFAP1B, 6530402F18RIK, ADAMTS10, FGF1, FN1	336	309	12504	1.92695331	0.9955	0.491258	22.774

GOTERM_CC_FAT	GO:0005792~microsome	11	1.85	0.020192	ATP2B2, CYP2A22, CYP2C55, PLCB4, SQLE, CYP2D11, UGT1A5, CYP2A5, CYP4F14, IGFALS, CYB5B	336	176	12504	2.32589286	0.9964	0.465039	23.595
INTERPRO	IPR016167:FAD-binding, type 2, subdomain 1	3	0.51	0.020691	LDHD, AOX1, AOX3	513	8	17763	12.9846491	1	0.596271	27.849
GOTERM_MF_FAT	GO:0008047~enzyme activator activity	14	2.36	0.020871	ASAP1, FGF13, CYB5B, ARHGAP30, ADAP2, SGSM1, GMIP, CHML, TBC1D4, RGS9, SH3BP1, ARAP2, ADC, FN1	367	249	13288	2.03573969	1	0.529687	26.808
SP_PIR_KEYWORDS	hydro-lyase	3	0.51	0.020872	CAR5A, CAR1, CAR3	518	8	17854	12.9251931	0.9994	0.410701	25.069
GOTERM_BP_FAT	GO:0007050~cell cycle arrest	6	1.01	0.021249	BC024139, AK1, PML, MTBP, ERN1, PKD1	381	57	13588	3.75410968	1	0.93728	30.57
SMART	SM00013:LRRNT	8	1.35	0.02147	FMOD, AMIGO3, FLRT1, PODNL1, LRTM1, IGFALS, PKD1, TLR7	275	93	9131	2.85622678	0.9831	0.557824	23.606
GOTERM_MF_FAT	GO:0016887~ATPase activity	15	2.53	0.023773	XRCC3, ABCB9, ABCD1, ABCA8A, INO80, ABCA2, ABCA3, ABCG8, ATP2B2, DDX11, ABCB1A, GM6524, ABCC4, RTEL1, ATP8B4	367	281	13288	1.93276252	1	0.556341	29.952
INTERPRO	IPR001395:Aldo/keto reductase	4	0.67	0.0244	AKR1C6, AKR1C19, AKR1D1, AKR1C20	513	22	17763	6.29558745	1	0.640453	31.999
GOTERM_CC_FAT	GO:0042598~vesicular fraction	11	1.85	0.024799	ATP2B2, CYP2A22, CYP2C55, PLCB4, SQLE, CYP2D11, UGT1A5, CYP2A5, CYP4F14, IGFALS, CYB5B	336	182	12504	2.24921507	0.999	0.499961	28.2
GOTERM_BP_FAT	GO:0006695~cholesterol biosynthetic process	4	0.67	0.025382	FDPS, MVK, IDI1, NSDHL	381	23	13588	6.20244209	1	0.954668	35.386
GOTERM_MF_FAT	GO:0005524~ATP binding	53	8.94	0.025893	INO80, ATP2B2, ACSS1, MAP3K4, DDX11, OASL1, TARSL2, RTEL1, MATK, ATP8B4, NLRP6, NMNAT2, MYLK3, OLA1, TYK2, PRKD2, PAPD4, ABCB1A, PKLR, ERN1, GM6524, BMP2K, MVK, MST1R, PRKD3, ABCD1, ABCA2, OAS2, ABCA3, TTBK2, DCLK3, DCLK2, DHX58, CHD3, OBSCN, ABCB9, AK1, ABCA8A, MET, ACACA, BCS1L, NPR2, MMAB, ABCG8, ACSM1, ABCC4, GRK4, JAK3, MYO19, PIP4K2A	367	1443	13288	1.32985134	1	0.568066	32.169
GOTERM_CC_FAT	GO:0030863~cortical cytoskeleton	5	0.84	0.027497	GYPC, SPNA1, ACTN1, SCNN1A, EPB4.9	336	43	12504	4.32724252	0.9995	0.503212	30.778

KEGG_PATHWAY	mmu00910:Nitrogen metabolism	4	0.67	0.027535	CAR14, CAR5A, CAR1, CAR3	167	23	5738	5.97552721	0.9756	0.411696	27.854
GOTERM_MF_FAT	GO:0016836~hydro-lyase activity	5	0.84	0.027836	CAR14, FASN, CAR5A, CAR1, CAR3	367	42	13288	4.3103672	1	0.576086	34.144
GOTERM_BP_FAT	GO:0006749~glutathione metabolic process	4	0.67	0.028422	GGT6, GGT7, GSTT1, GSTT3	381	24	13588	5.944007	1	0.961333	38.725
GOTERM_CC_FAT	GO:0005578~proteinaceous extracellular matrix	15	2.53	0.029411	FMOD, PODNL1, LTBP3, EFEMP2, LGALS1, MMP28, WNT2B, LAMA4, FREM1, COL27A1, MFAP1B, 6530402F18RIK, ADAMTS10, FGF1, FN1	336	297	12504	1.87950938	0.9997	0.49671	32.553
GOTERM_MF_FAT	GO:0030246~carbohydrate binding	16	2.7	0.030003	GLG1, APCS, PRG4, LGALS4, SELL, LGALS1, CD248, CD209F, ABP1, FREM1, ITIH4, PKD1, FGF1, CHRD, SELPLG, FN1	367	317	13288	1.82748691	1	0.586026	36.283
SP_PIR_KEYWORDS	SH2 domain	8	1.35	0.030047	TYK2, DAPP1, SH2D5, JAK3, STAT1, BLNK, MATK, STAT2	518	103	17854	2.67706264	1	0.510265	34.126
SP_PIR_KEYWORDS	Flavoprotein	8	1.35	0.030047	HAO1, CHDH, SQLE, PYROXD1, MAOA, LDHD, AOX1, MICAL1	518	103	17854	2.67706264	1	0.510265	34.126
BIOCARTA	m_ifnaPathway:IFN alpha signaling pathway	3	0.51	0.030667	TYK2, STAT1, STAT2	43	8	1171	10.2122093	0.9033	0.903289	27.884
GOTERM_BP_FAT	GO:0046578~regulation of Ras protein signal transduction	11	1.85	0.031056	OBSCN, PLEKHG2, SGSM1, ADAP2, CSF1, SCAI, TBC1D4, ASAP1, E130306D19RIK, ARAP2, FGD4	381	181	13588	2.16742797	1	0.964906	41.487
GOTERM_MF_FAT	GO:0032559~adenyl ribonucleotide binding	53	8.94	0.031358	INO80, ATP2B2, ACSS1, MAP3K4, DDX11, OASL1, TARSL2, RTEL1, MATK, ATP8B4, NLRP6, NMNAT2, MYLK3, OLA1, TYK2, PRKD2, PAPD4, ABCB1A, PKLR, ERN1, GM6524, BMP2K, MVK, MST1R, PRKD3, ABCD1, ABCA2, OAS2, ABCA3, TTBK2, DCLK3, DCLK2, DHX58, CHD3, OBSCN, ABCB9, AK1, ABCA8A, MET, ACACA, BCS1L, NPR2, MMAB, ABCG8, ACSM1, ABCC4, GRK4, JAK3, MYO19, PIP4K2A	367	1460	13288	1.31436676	1	0.585427	37.588
UP_SEQ_FEATURE	domain:SH3	11	1.85	0.0316	OBSCN, STAC2, BCAR1, ABI3, SPNA1, ASAP1, PPP1R13B, MPP7, SHANK2, 1700029G01RIK, MATK	500	163	16021	2.16234356	1	0.999996	41.494
INTERPRO	IPR016166:FAD-binding, type 2	3	0.51	0.032012	LDHD, AOX1, AOX3	513	10	17763	10.3877193	1	0.72437	39.827

GOTERM_BP_FAT	GO:0051186~cofactor metabolic process	11	1.85	0.03208	NMNAT2, ACSS1, GGT6, GGT7, ACNAT1, MTR, GSTT1, SPNA1, GSTT3, MMAB, ACOT3	381	182	13588	2.15551902	1	0.961982	42.529
SP_PIR_KEYWORDS	carbon-oxygen lyase	3	0.51	0.032286	CAR5A, CAR1, CAR3	518	10	17854	10.3401544	1	0.513229	36.176
SP_PIR_KEYWORDS	Pyrrolidone carboxylic acid	5	0.84	0.032686	FMOD, RNASE4, HGF, SELPLG, FN1	518	42	17854	4.10323589	1	0.496485	36.536
GOTERM_BP_FAT	GO:0006631~fatty acid metabolic process	11	1.85	0.0342	SCD1, AWAT2, FAR2, ACSM1, SCD2, ACNAT1, FASN, ACACA, ELOVL6, SC4MOL, ACOT3	381	184	13588	2.13208947	1	0.963331	44.629
GOTERM_BP_FAT	GO:0055092~sterol homeostasis	4	0.67	0.035052	ABCG8, LDLR, PCSK9, ABCA2	381	26	13588	5.48677569	1	0.960053	45.454
GOTERM_BP_FAT	GO:0042632~cholesterol homeostasis	4	0.67	0.035052	ABCG8, LDLR, PCSK9, ABCA2	381	26	13588	5.48677569	1	0.960053	45.454
GOTERM_CC_FAT	GO:0009986~cell surface	15	2.53	0.035292	COL23A1, CD8B1, ACVRL1, GPR125, SELL, GYPA, TNFRSF13C, TNFSF13, NFAM1, FCGR1, GHRHR, RAET1D, ICOS, SULF1, SCNN1A	336	305	12504	1.83021077	1	0.533648	37.75
GOTERM_BP_FAT	GO:0000904~cell morphogenesis involved in differentiation	12	2.02	0.036291	SEMA6A, ATP2B2, NRP1, NDN, ESRRB, ANK3, 6530402F18RIK, LEF1, NGFR, GLI3, NFATC1, NUMBL	381	212	13588	2.01871936	1	0.958302	46.631
UP_SEQ_FEATURE	short sequence motif:Histidine box-2	3	0.51	0.036919	SCD1, SCD2, SC4MOL	500	10	16021	9.6126	1	0.999992	46.634
UP_SEQ_FEATURE	short sequence motif:Histidine box-1	3	0.51	0.036919	SCD1, SCD2, SC4MOL	500	10	16021	9.6126	1	0.999992	46.634
UP_SEQ_FEATURE	short sequence motif:Histidine box-3	3	0.51	0.036919	SCD1, SCD2, SC4MOL	500	10	16021	9.6126	1	0.999992	46.634
INTERPRO	IPR000980:SH2 motif	8	1.35	0.041592	TYK2, DAPP1, SH2D5, JAK3, STAT1, BLNK, MATK, STAT2	513	111	17763	2.49554818	1	0.800618	48.481
INTERPRO	IPR017940:ABC transporter integral membrane type 1	4	0.67	0.041716	ABCB9, ABCB1A, ABCD1, ABCC4	513	27	17763	5.12973793	1	0.788337	48.585
KEGG_PATHWAY	mmu01040:Biosynthesis of unsaturated fatty acids	4	0.67	0.041831	SCD1, SCD2, ELOVL6, ACOT3	167	27	5738	5.09026392	0.9966	0.508558	39.326
SP_PIR_KEYWORDS	immune response	11	1.85	0.042011	GPR183, LAT2, CD8B1, CD300E, TNFRSF13C, SAMHD1, LIME1, TNFSF13, TLR7, FCGR1, DHX58	518	184	17854	2.06053802	1	0.566959	44.414

GOTERM_BP_FAT	GO:0051188~cofactor biosynthetic process	7	1.18	0.042104	NMNAT2, ACSS1, GGT6, GGT7, MTR, SPNA1, MMAB	381	91	13588	2.74338785	1	0.970674	51.844
SP_PIR_KEYWORDS	electron transfer	4	0.67	0.042199	CYP2A22, CYP2D11, CYP2A5, CYP2B13	518	27	17854	5.10624911	1	0.549094	44.563
SP_PIR_KEYWORDS	atp-binding	49	8.26	0.042485	[illegible] ABCD1, INO80, ABCA2, ABCA3, ATP2B2, ACSS1, MAP3K4, TTBK2, DDX11, DCLK3, DCLK2, TARSL2, DHX58, RTEL1, MATK, ATP8B4, OBSCN, NLRP6, NMNAT2, ABCB9, MYLK3, AK1, MET, ABCA8A, ACACA, OLA1, BCS1L, MMAB, TYK2, ABCG8, PRKD2, ACSM1, PAPD4, ABCB1A, PKLR, BMP2K, ERN1, GM6524, ABCC4, GRK4, MVK, MST1R, MYO19, JAK3, PIP4K2A, PRKD3	518	1287	17854	1.31227031	1	0.533228	44.789
SP_PIR_KEYWORDS	Fatty acid biosynthesis	5	0.84	0.043587	SCD1, SCD2, FASN, ACACA, ELOVL6	518	46	17854	3.74643277	1	0.525211	45.653
GOTERM_BP_FAT	GO:0007169~transmembrane receptor protein tyrosine kinase signaling pathway	11	1.85	0.043666	PLAT, NRP1, DOK4, NDN, BCAR1, MET, DOK7, HGF, MST1R, FGF1, GFRA2	381	192	13588	2.04325241	1	0.96992	53.16
GOTERM_BP_FAT	GO:0006575~cellular amino acid derivative metabolic process	9	1.52	0.044477	CHDH, ATP2B2, GGT6, GGT7, MAOA, GSTT1, GAMT, GSTT3, ADC	381	141	13588	2.27642821	1	0.967344	53.831
GOTERM_MF_FAT	GO:0016229~steroid dehydrogenase activity	4	0.67	0.044634	HSD3B2, AKR1C6, AKR1D1, NSDHL	367	29	13288	4.99408062	1	0.700913	49.114
GOTERM_BP_FAT	GO:0042398~cellular amino acid derivative biosynthetic process	5	0.84	0.044656	CHDH, GGT6, GGT7, GAMT, ADC	381	48	13588	3.71500437	1	0.963057	53.978

UP_SEQ_FEATURE	transmembrane region	146	24.6	0.045244	SLC44A3, AU040320, AQP8, LRTM1, BTC, SYT3, INTS1, TNFSF13, SYT7, CD53, GPAT2, TLR7, KLHDC7A, GHRHR, SLC35A4, ATP2B2, BC018242, SLC25A23, SLC2A2, S1PR5, ELOVL6, TMEM185B, SLC4A3, TMEM14A, SLC12A7, GOLT1B, TMIE, SLC22A23, TMEM132A, TMEM132E, LAT2, SSTR2, CD37, RELT, ABCB1A, TMEM184A, MST1R, GLG1, AB124611, TMEM199, SFXN4, ABCA2, BDKRB2, ABCA3, SEMA5B, PVRL4, TTBK2, PVRL3, ICOS, SLC35F2, 6030446N20RIK, SREBF1, SCD1, REEP6, ABCB9, FKTN, PRAF2, SCD2, CD8B1, MAOA, MET, CD300E, TNFRSF13C, PPAPDC1B, REEP1, FCGR1, AWAT2, ABCG8, SEMA6A, GGT6, GGT7, LTB4R1, FAM151A, SLC13A3, NRP2, GYPC, PILRB2, NRP1, ACVRL1, CLDN4, LDLR, GYPA, LRRC19, GABBR2, FAM57A, SC4MOL, FAR2, DNAJC16, FITM1, SERAC1, SLC22A1, GPR97, PARM1, COL23A1, CAR14, CMKLR1, SCAI, CHST3, NFAM1, CYB5B, TMEM67, AMIGO3, SLC35E4, TLCD2, SQLE, ERN1, NGFR, CUX1, HSD3B2, GPR183, HS3ST6, CSF1,	500	4113	16021	1.13740141	1	0.999994	53.834
SP_PIR_KEYWORDS	sialic acid	3	0.51	0.045586	GLG1, GYPA, SELPLG	518	12	17854	8.61679537	1	0.524983	47.186
INTERPRO	IPR003593:ATPase, AAA+ type, core	9	1.52	0.046569	ABCB9, ABCB1A, ABCD1, ABCA8A, GM6524, ABCC4, ABCA2, BCS1L, ABCA3	513	138	17763	2.25819985	1	0.811923	52.503
GOTERM_MF_FAT	GO:0032555~purine ribonucleotide binding	62	10.5	0.046574	ACVRL1, INO80, ATP2B2, ACSS1, MAP3K4, DDX11, OASL1, GBP10, TARSL2, RTEL1, MATK, ATP8B4, NLRP6, NMNAT2, GBP6, MYLK3, OLA1, RND2, TYK2, PRKD2, PAPD4, ABCB1A, PDE5A, PKLR, ERN1, GM6524, BMP2K, MVK, MST1R, PRKD3, GBP2, GBP1, ABCD1, ABCA2, OAS2, ABCA3, TTBK2, RAC2, DCLK3, DCLK2, DHX58, CHD3, OBSCN, ABCB9, IRGM2, AK1, MET, ABCA8A, ACACA, BCS1L, NPR2, MMAB, ABCG8, ACSM1, ABCC4, GRK4, MYO19, JAK3, PIP4K2A	367	1796	13288	1.24991049	1	0.701275	50.622

GOTERM_MF_FAT	GO:0032553~ribonucleotide binding	62	10.5	0.046574	ACVRL1, INO80, ATP2B2, ACSS1, MAP3K4, DDX11, OASL1, GBP10, TARSL2, RTEL1, MATK, ATP8B4, NLRP6, NMNAT2, GBP6, MYLK3, OLA1, RND2, TYK2, PRKD2, PAPD4, ABCB1A, PDE5A, PKLR, ERN1, GM6524, BMP2K, MVK, MST1R, PRKD3, GBP2, GBP1, ABCD1, ABCA2, OAS2, ABCA3, TTBK2, RAC2, DCLK3, DCLK2, DHX58, CHD3, OBSCN, ABCB9, IRGM2, AK1, MET, ABCA8A, ACACA, BCS1L, NPR2, MMAB, ABCG8, ACSM1, ABCC4, GRK4, MYO19, JAK3, PIP4K2A	367	1796	13288	1.24991049	1	0.701275	50.622
GOTERM_BP_FAT	GO:0006732~coenzyme metabolic process	9	1.52	0.047636	NMNAT2, ACSS1, GGT6, GGT7, ACNAT1, MTR, GSTT1, GSTT3, ACOT3	381	143	13588	2.24459006	1	0.966238	56.357
GOTERM_BP_FAT	GO:0006790~sulfur metabolic process	7	1.18	0.04804	GGT6, GGT7, MTR, SULF1, CYTL1, GSTT1, GSTT3	381	94	13588	2.65583291	1	0.962794	56.67
SMART	SM00252:SH2	8	1.35	0.0492	TYK2, DAPP1, SH2D5, JAK3, STAT1, BLNK, MATK, STAT2	275	111	9131	2.39305487	0.9999	0.794194	46.525
GOTERM_MF_FAT	GO:0008270~zinc ion binding	71	12	0.049754	PILRB2, ZC3HAV1, AGTPBP1, CAR5A, MMP28, GLI3, ZKSCAN4, GM14391, STAC2, SETMAR, ZFP831, ZFP354A, MICAL1, ZC3HAV1L, ZFP882, DDAH1, ZFP354B, CAR14, PRKD2, TRIM35, ZFP867, ZFP280D, PDE5A, PGCP, PRKD3, ZFP710, ZFP418, ZFP395, SORD, USP3, ZFP715, ZFP69, ZFP398, TRIM14, PML, ASAP1, ZFP456, DPF1, ZFP455, ZDHHC24, NR1D1, GMIP, TCEA3, ADH1, STAMBPL1, FASN, ADAMTS10, KDM3A, XAF1, SLC39A5, CAR1, DHX58, CAR3, FGD4, CHD3, SGIP1, AGBL3, ESRRB, PM20D1, 4931406C07RIK, TET1, FOXP2, ADAP2, ZFP182, MTR, ZFP7, MZF1, LIME1, ZFHX2, ARAP2, KLF1	367	2105	13288	1.22123658	1	0.710948	53.004
GOTERM_CC_FAT	GO:0009897~external side of plasma membrane	11	1.85	0.050518	RAET1D, COL23A1, CD8B1, GPR125, SELL, ICOS, GYPA, TNFRSF13C, TNFSF13, SCNN1A, FCGR1	336	206	12504	1.9871706	1	0.640116	49.536
PIR_SUPERFAMILY	PIRSF000617:tyrosine-protein kinase, HGF/MSP receptor type	2	0.34	0.050959	MET, MST1R	211	2	8136	38.5592417	1	0.948606	48.786

PIR_SUPERFAMILY	PIRSF000617:TyrPK_HGF-R	2	0.34	0.050959	MET, MST1R	211	2	8136	38.5592417	1	0.948606	48.786
UP_SEQ_FEATURE	domain:IRS-type PTB	3	0.51	0.05198	DOK3, DOK4, DOK7	500	12	16021	8.0105	1	0.999993	58.981
INTERPRO	IPR002404:Insulin receptor substrate-1, PTB	3	0.51	0.052429	DOK3, DOK4, DOK7	513	13	17763	7.99055331	1	0.837496	56.862
GOTERM_MF_FAT	GO:0000166~nucleotide binding	73	12.3	0.054313	KIFC2, GNA14, XRCC3, ACVRL1, LDHD, INO80, ATP2B2, ACSS1, MAP3K4, DDX11, OASL1, GBP10, TARSL2, RTEL1, RDM1, ATP8B4, MATK, NLRP6, NMNAT2, GBP6, MYLK3, OLA1, TYK2, RND2, HAO1, PRKD2, ABCB1A, PAPD4, SQLE, PDE5A, PKLR, GM6524, ERN1, BMP2K, CPSF7, MVK, MST1R, PRKD3, GBP2, GBP1, ABCD1, ABCA2, OAS2, ABCA3, TTBK2, RAC2, DCLK3, DCLK2, DHX58, CHD3, OBSCN, CHDH, ABCB9, IRGM2, CSTF2, AK1, MET, ABCA8A, ACACA, TREX1, BCS1L, NPR2, MMAB, ABCG8, ACSM1, DHFR, AOX1, ABCC4, AOX3, GRK4, MYO19, JAK3, PIP4K2A	367	2183	13288	1.21077287	1	0.729063	56.231
SP_PIR_KEYWORDS	Lectin	10	1.69	0.054316	GLG1, APCS, LGALS4, SELL, CD248, LGALS1, FREM1, CD209F, PKD1, SELPLG	518	167	17854	2.06390308	1	0.573554	53.426
INTERPRO	IPR002165:Plexin	4	0.67	0.054384	SEMA5B, SEMA6A, MET, MST1R	513	30	17763	4.61676413	1	0.837872	58.231
GOTERM_BP_FAT	GO:0042482~positive regulation of odontogenesis	2	0.34	0.055152	CSF1, NGFR	381	2	13588	35.664042	1	0.974193	61.852
GOTERM_BP_FAT	GO:0042488~positive regulation of odontogenesis of dentine-containing tooth	2	0.34	0.055152	CSF1, NGFR	381	2	13588	35.664042	1	0.974193	61.852
UP_SEQ_FEATURE	domain:Sema	4	0.67	0.05532	SEMA5B, SEMA6A, MET, MST1R	500	28	16021	4.57742857	1	0.999985	61.328
UP_SEQ_FEATURE	domain:ABC transporter 2	4	0.67	0.05532	ABCB1A, ABCA8A, ABCA2, ABCA3	500	28	16021	4.57742857	1	0.999985	61.328
UP_SEQ_FEATURE	domain:ABC transporter 1	4	0.67	0.05532	ABCB1A, ABCA8A, ABCA2, ABCA3	500	28	16021	4.57742857	1	0.999985	61.328
SMART	SM00382:AAA	9	1.52	0.055708	ABCB9, ABCB1A, ABCD1, ABCA8A, GM6524, ABCC4, ABCA2, BCS1L, ABCA3	275	138	9131	2.16545455	1	0.785503	50.893

SMART	SM00310:PTBI	3	0.51	0.056283	DOK3, DOK4, DOK7	275	13	9131	7.66237762	1	0.743677	51.262
INTERPRO	IPR016244:Tyrosine-protein kinase, HGF/MSP receptor	2	0.34	0.056819	MET, MST1R	513	2	17763	34.625731	1	0.840802	59.878
GOTERM_BP_FAT	GO:0050853~B cell receptor signaling pathway	3	0.51	0.056869	LAT2, BCAR1, NFAM1	381	14	13588	7.64229471	1	0.973859	63.013
GOTERM_BP_FAT	GO:0015893~drug transport	3	0.51	0.056869	ABP1, ABCB1A, ABCC4	381	14	13588	7.64229471	1	0.973859	63.013
SP_PIR_KEYWORDS	leucine-rich repeat	14	2.36	0.057728	FMOD, NLRP6, FLRT1, PODNL1, LRRN4, FBXL21, GPR125, LRTM1, LRRC19, IGFALS, TLR7, AMIGO3, LRG1, PKD1	518	275	17854	1.75469287	1	0.580887	55.673
SP_PIR_KEYWORDS	neurogenesis	9	1.52	0.058347	NRP2, SEMA5B, SEMA6A, NRP1, 6530402F18RIK, PAX5, ATOH8, NGFR, DPF1	518	144	17854	2.15419884	1	0.570041	56.07
INTERPRO	IPR000863:Sulfotransferase	4	0.67	0.05897	HS3ST6, SULT5A1, CHST3, 2810007J24RIK	513	31	17763	4.46783626	1	0.842084	61.283
INTERPRO	IPR001627:Semaphorin/CD100 antigen	4	0.67	0.05897	SEMA5B, SEMA6A, MET, MST1R	513	31	17763	4.46783626	1	0.842084	61.283
SP_PIR_KEYWORDS	peroxisome	7	1.18	0.060062	HAO1, FAR2, ABCD1, ACNAT1, MVK, IDI1, ACOT3	518	96	17854	2.51323198	1	0.566647	57.152
KEGG_PATHWAY	mmu00900:Terpenoid backbone biosynthesis	3	0.51	0.060283	FDPS, MVK, IDI1	167	14	5738	7.36270317	0.9997	0.601013	51.665
GOTERM_CC_FAT	GO:0005777~peroxisome	7	1.18	0.060539	HAO1, FAR2, ABCD1, ACNAT1, MVK, IDI1, ACOT3	336	104	12504	2.50480769	1	0.683068	56.128
GOTERM_CC_FAT	GO:0042579~microbody	7	1.18	0.060539	HAO1, FAR2, ABCD1, ACNAT1, MVK, IDI1, ACOT3	336	104	12504	2.50480769	1	0.683068	56.128
UP_SEQ_FEATURE	domain:PKD 4	2	0.34	0.061325	AU040320, PKD1	500	2	16021	32.042	1	0.999983	65.233
UP_SEQ_FEATURE	domain:PKD 3	2	0.34	0.061325	AU040320, PKD1	500	2	16021	32.042	1	0.999983	65.233
GOTERM_BP_FAT	GO:0042592~homeostatic process	24	4.05	0.061639	SLC12A7, SCD2, SLC9A6, LDLR, NDN, CSF1, MLXIPL, TNFRSF13C, ACACA, ABCA2, ILDR2, SLC9A3R1, GHRHR, BCL2L11, ABCG8, ATP2B2, GCKR, DNAJC16, PCSK9, RGN, SLC39A5, 2700094K13RIK, KLF1, RTEL1	381	584	13588	1.46564556	1	0.978243	66.065

UP_SEQ_FEATURE	topological domain:Extracellular	81	13.7	0.061913	NRP2, GYPC, SLC36A1, PILRB2, ACVRL1, NRP1, GABRB3, AU040320, GPR125, LDLR, CLDN4, AQP8, LRTM1, BTC, GYPA, LRRC19, TNFSF13, CD53, GABBR2, TLR7, GHRHR, ATP2B2, DNAJC16, FITM1, SLC2A2, S1PR5, SLC22A1, GPR97, PARM1, COL23A1, CAR14, CMKLR1, GOLT1B, TMIE, TMEM132A, NFAM1, TMEM132E, CD37, SSTR2, LAT2, AMIGO3, RELT, ABCB1A, NGFR, MST1R, GLG1, GPR183, AB124611, CD248, CSF1, GPR64, FPR1, BDKRB2, TPCN2, SEMA5B, PVRL4, ICOS, PVRL3, SHISA5, SUCNR1, SLC39A5, SELPLG, SCNN1A, PRAF2, CD8B1, MPZ, LRRN4, SELL, MET, TNFRSF13C, CD300E, NPR2, GPR77, FCGR1, ABCG8, SEMA6A, GGT6, GGT7, LTB4R1, PLSCR2, LIME1	500	2174	16021	1.19383717	1	0.999953	65.595
SP_PIR_KEYWORDS	transferase	51	8.6	0.062274	GPAT2, GSTM6, ZDHHC24, MAP3K4, HNMT, TTBK2, UGT1A5, TRDMT1, SETMAR, OASL1, DCLK3, FASN, DCLK2, LIPT1, MATK, OBSCN, NMNAT2, FKTN, MYLK3, AK1, MET, ACNAT1, FDPS, GSTT1, CHST3, GSTT3, MMAB, INMT, TYK2, AWAT2, PRKD2, GGT6, RPAP1, GGT7, PAPD4, MTR, PKLR, ERN1, BMP2K, 5033411D12RIK, GRK4, GAMT, MVK, 2810007J24RIK, MST1R, JAK3, PIP4K2A, PRKD3	518	1385	17854	1.26918863	1	0.5665	58.511
GOTERM_MF_FAT	GO:0005529~sugar binding	10	1.69	0.063106	GLG1, APCS, LGALS4, SELL, CD248, LGALS1, FREM1, CD209F, PKD1, SELPLG	367	181	13288	2.00039141	1	0.769583	61.882
INTERPRO	IPR000203:GPS	4	0.67	0.063731	GPR97, GPR125, GPR64, PKD1	513	32	17763	4.32821637	1	0.855607	64.231
GOTERM_MF_FAT	GO:0032403~protein complex binding	6	1.01	0.063789	DOK3, DOK4, SLC2A2, DOK7, TREX1, FCGR1	367	78	13288	2.78516034	1	0.760998	62.291
SP_PIR_KEYWORDS	lyase	8	1.35	0.064324	CAR14, FASN, NPL, NPR2, CAR5A, OGG1, CAR1, CAR3	518	122	17854	2.26014305	1	0.565454	59.736
SMART	SM00630:Sema	4	0.67	0.064802	SEMA5B, SEMA6A, MET, MST1R	275	31	9131	4.28434018	1	0.753278	56.448
GOTERM_MF_FAT	GO:0070330~aromatase activity	4	0.67	0.066182	CYP2A22, CYP2C55, CYP2D11, CYP2A5	367	34	13288	4.259657	1	0.762022	63.692
GOTERM_MF_FAT	GO:0017124~SH3 domain binding	6	1.01	0.066639	PLSCR2, BCAR1, DENND1A, MICAL1, SH3BP1, SHANK2	367	79	13288	2.74990515	1	0.752822	63.954

GOTERM_CC_FAT	GO:0030424~axon	7	1.18	0.06758	NRP2, PRPH, SEMA6A, NRP1, GABRB3, ANK3, GM6524	336	107	12504	2.43457944	1	0.700913	60.273
KEGG_PATHWAY	mmu04520:Adherens junction	6	1.01	0.068194	PVRL4, RAC2, PVRL3, MET, ACTN1, LEF1	167	76	5738	2.71257485	0.9999	0.609131	56.214
SP_PIR_KEYWORDS	cell membrane	61	10.3	0.068929	GYPC, SLC36A1, GPR125, CLDN4, GABRB3, BTC, TNFSF13, GABBR2, FAM57A, GHRHR, ATP2B2, RAET1D, S1PR5, VNN1, SLC22A1, GPR97, PARM1, COL23A1, CMKLR1, NFAM1, RFTN2, TMEM67, SSTR2, LAT2, DOK3, RELT, ABCB1A, 6530402F18RIK, DOK7, GBP2, GBP1, GLG1, GPR183, BBS7, CSF1, GPR64, FPR1, BDKRB2, PVRL4, PDE6C, FOLR2, PVRL3, SUCNR1, SLC39A5, SELPLG, SCNN1A, BLNK, CNNM4, DLGAP3, ABCA8A, CD300E, DENND1A, GPR77, FCGR1, SHANK2, LIN7A, ADAP2, LTB4R1, LIME1, PLEKHA2,	518	1713	17854	1.22737774	1	0.578709	62.364
SMART	SM00303:GPS	4	0.67	0.069982	GPR97, GPR125, GPR64, PKD1	275	32	9131	4.15045455	1	0.744356	59.349
SP_PIR_KEYWORDS	microsome	7	1.18	0.070316	CYP2A22, CYP2C55, SQLE, CYP2D11, UGT1A5, CYP2A5, CYP4F14	518	100	17854	2.4127027	1	0.573889	63.124
GOTERM_CC_FAT	GO:0005929~cilium	8	1.35	0.070894	ATP2B2, NPHP4, SRPX, BBS7, PKD1, CYS1, GLI3, TTLL11	336	135	12504	2.20529101	1	0.696938	62.096
UP_SEQ_FEATURE	nucleotide phosphate-binding region:ATP 1	4	0.67	0.070953	ABCB1A, ABCA8A, ABCA2, ABCA3	500	31	16021	4.13445161	1	0.999971	70.731
UP_SEQ_FEATURE	nucleotide phosphate-binding region:ATP 2	4	0.67	0.070953	ABCB1A, ABCA8A, ABCA2, ABCA3	500	31	16021	4.13445161	1	0.999971	70.731
UP_SEQ_FEATURE	domain:GPS	4	0.67	0.070953	GPR97, GPR125, GPR64, PKD1	500	31	16021	4.13445161	1	0.999971	70.731
INTERPRO	IPR013320:Concanavalin A-like lectin/glucanase, subgroup	6	1.01	0.071321	LAMA4, APCS, LGALS4, LGALS1, COL20A1, THBS3	513	77	17763	2.69810891	1	0.878332	68.499
GOTERM_BP_FAT	GO:0046068~cGMP metabolic process	3	0.51	0.072317	ATP2B2, PDE5A, NPR2	381	16	13588	6.68700787	1	0.987358	72.061
PIR_SUPERFAMILY	PIRSF800007:secretin receptor-like G protein-coupled receptors	4	0.67	0.073944	GPR97, GPR125, GPR64, GHRHR	211	38	8136	4.05886755	1	0.969428	62.574

SP_PIR_KEYWORDS	sh3 domain	11	1.85	0.074203	OBSCN, STAC2, BCAR1, ABI3, SPNA1, ASAP1, PPP1R13B, MPP7, SHANK2, 1700029G01RIK, MATK	518	204	17854	1.85852449	1	0.582291	65.178
PIR_SUPERFAMILY	PIRSF000636:TyrPK_Jak	2	0.34	0.075461	TYK2, JAK3	211	3	8136	25.7061611	1	0.948615	63.351
PIR_SUPERFAMILY	PIRSF005680:interferon-induced 56K protein	2	0.34	0.075461	IFIT2, IFIT1	211	3	8136	25.7061611	1	0.948615	63.351
PIR_SUPERFAMILY	PIRSF036899:alpha(1)-acid glycoprotein	2	0.34	0.075461	ORM1, ORM2	211	3	8136	25.7061611	1	0.948615	63.351
PIR_SUPERFAMILY	PIRSF000344:acyl-CoA desaturase	2	0.34	0.075461	SCD1, SCD2	211	3	8136	25.7061611	1	0.948615	63.351
PIR_SUPERFAMILY	PIRSF036899:AGP	2	0.34	0.075461	ORM1, ORM2	211	3	8136	25.7061611	1	0.948615	63.351
PIR_SUPERFAMILY	PIRSF036960:Neuropilin	2	0.34	0.075461	NRP2, NRP1	211	3	8136	25.7061611	1	0.948615	63.351
PIR_SUPERFAMILY	PIRSF000636:tyrosine-protein kinase, Jak/Tyk2 type	2	0.34	0.075461	TYK2, JAK3	211	3	8136	25.7061611	1	0.948615	63.351
UP_SEQ_FEATURE	domain:PH 2	4	0.67	0.076559	ADAP2, ARAP2, PLEKHA2, FGD4	500	32	16021	4.00525	1	0.999968	73.544
INTERPRO	IPR006209:EGF	8	1.35	0.077194	PLAT, LDLR, LTBP3, SELL, CD248, 6530402F18RIK, BTC, PROZ	513	128	17763	2.16410819	1	0.891148	71.47
UP_SEQ_FEATURE	domain:Protein kinase 2	3	0.51	0.077813	TYK2, OBSCN, JAK3	500	15	16021	6.4084	1	0.99994	74.137
UP_SEQ_FEATURE	domain:Protein kinase 1	3	0.51	0.077813	TYK2, OBSCN, JAK3	500	15	16021	6.4084	1	0.99994	74.137
GOTERM_BP_FAT	GO:0007242~intracellular signaling cascade	34	5.73	0.079519	PML, FGF13, GHRHR, PLEKHG2, PLCB4, STAC2, RAC2, GMIP, DCLK2, PKD1, SPRED3, FGD4, NFATC1, OBSCN, GPR155, MET, NPR2, HGF, NFAM1, SLC9A3R1, E130306D19RIK, RCAN2, RND2, TYK2, PRKD2, LAT2, SSTR2, DOK3, ADAP2, DOK4, RGS9, JAK3, ASB4, PRKD3	381	915	13588	1.32522123	1	0.990663	75.526
GOTERM_MF_FAT	GO:0004031~aldehyde oxidase activity	2	0.34	0.080382	AOX1, AOX3	367	3	13288	24.1380563	1	0.806697	71.057

GOTERM_MF_FAT	GO:0016623~oxidoreductase activity, acting on the aldehyde or oxo group of donors, oxygen as acceptor	2	0.34	0.080382	AOX1, AOX3	367	3	13288	24.1380563	1	0.806697	71.057
GOTERM_BP_FAT	GO:0010166~wax metabolic process	2	0.34	0.081579	AWAT2, FAR2	381	3	13588	23.776028	1	0.990483	76.44
GOTERM_BP_FAT	GO:0051450~myoblast proliferation	2	0.34	0.081579	MET, HGF	381	3	13588	23.776028	1	0.990483	76.44
GOTERM_BP_FAT	GO:0048012~hepatocyte growth factor receptor signaling pathway	2	0.34	0.081579	MET, HGF	381	3	13588	23.776028	1	0.990483	76.44
GOTERM_BP_FAT	GO:0010025~wax biosynthetic process	2	0.34	0.081579	AWAT2, FAR2	381	3	13588	23.776028	1	0.990483	76.44
UP_SEQ_FEATURE	domain:PH 1	4	0.67	0.082353	ADAP2, ARAP2, PLEKHA2, FGD4	500	33	16021	3.88387879	1	0.999931	76.182
INTERPRO	IPR001500:Alpha-1-acid glycoprotein	2	0.34	0.084008	ORM1, ORM2	513	3	17763	23.0838207	1	0.904738	74.587
INTERPRO	IPR016251:Tyrosine-protein kinase, Jak/Tyk2	2	0.34	0.084008	TYK2, JAK3	513	3	17763	23.0838207	1	0.904738	74.587
INTERPRO	IPR006554:Helicase-like, DEXD box c2 type	2	0.34	0.084008	DDX11, RTEL1	513	3	17763	23.0838207	1	0.904738	74.587
INTERPRO	IPR014648:Neuropilin	2	0.34	0.084008	NRP2, NRP1	513	3	17763	23.0838207	1	0.904738	74.587

GOTERM_BP_FAT	GO:0006350~transcription	60	10.1	0.084013	ZKSCAN4, GM14391, HOMEZ, ZFP354A, SPIB, ATOH8, ZFP882, ZFP354B, POU2AF1, BARHL1, RBL1, MLXIPL, SCAI, LEF1, HDAC11, TLE2, ZFP867, ZFP280D, ERN1, CAND2, CDCA7L, CUX1, TRAPPC2, ZFP710, ZFP418, ZFP715, NDN, ZFP69, ZFP398, PML, ZFP456, DPF1, ZFP455, TSC22D1, TRP53BP1, TCEA3, NR1D1, POU2F1, KDM3A, PER3, NFATC1, SREBF1, ESRRB, STAT1, FOXP2, STAT2, FOXF1A, RPAP1, SAP130, HNRNPUL1, ZFP182, DBP, ZFP7, MZF1, LIME1, KLF1	381	1772	13588	1.20758607	1	0.990521	77.478
INTERPRO	IPR001565:Synaptotagmin	3	0.51	0.084816	SYT3, DOC2G, SYT7	513	17	17763	6.11042312	1	0.900432	74.935
GOTERM_MF_FAT	GO:0019904~protein domain specific binding	10	1.69	0.084834	ATP2B2, LAT2, PLSCR2, BCAR1, DENND1A, MICAL1, SH3BP1, SHANK2, KLF1, SCNN1A	367	192	13288	1.88578565	1	0.814435	73.063
GOTERM_MF_FAT	GO:0005096~GTPase activator activity	10	1.69	0.084834	ARHGAP30, SGSM1, ADAP2, CHML, GMIP, TBC1D4, ASAP1, RGS9, SH3BP1, ARAP2	367	192	13288	1.88578565	1	0.814435	73.063
PIR_SUPERFAMILY	PIRSF000503:glutathione transferase	3	0.51	0.085077	GSTT1, GSTT3, GSTM6	211	19	8136	6.08830132	1	0.944056	67.939
GOTERM_MF_FAT	GO:0031406~carboxylic acid binding	6	1.01	0.085193	FOLR2, FASN, ACACA, BC046331, FABP1, LEPREL1	367	85	13288	2.5557942	1	0.806124	73.219
SP_PIR_KEYWORDS	multifunctional enzyme	5	0.84	0.08695	HSD3B2, FASN, ACACA, ERN1, OGG1	518	58	17854	2.97130875	1	0.631297	71.195
UP_SEQ_FEATURE	domain:ABC transporter	3	0.51	0.087153	ABCG8, ABCB9, ABCD1	500	16	16021	6.007875	1	0.999924	78.179
SMART	SM00488:DEXDc2	2	0.34	0.087358	DDX11, RTEL1	275	3	9131	22.1357576	1	0.790348	67.83

SP_PIR_KEYWORDS	signal	99	16.7	0.087533	GPR125, GABRB3, LTBP3, LRTM1, BTC, BPHL, TLR7, GHRHR, OLFML3, SBSN, VNN1, TMIE, TMEM132A, TMEM132E, RELT, 6530402F18RIK, PGCP, MST1R, GLG1, AB124611, LEAP2, PVRL4, SEMA5B, FOLR2, ICOS, PVRL3, OLFM1, FN1, PLAT, ABCB9, CD8B1, EFEMP2, MET, CD300E, HGF, FCGR1, LCN2, SEMA6A, LAMA4, FREM1, CHRD, NRP2, ARSB, PILRB2, PODNL1, ACVRL1, NRP1, LDLR, LRRC19, GABBR2, MMP28, 2010001M09RIK, RAET1D, DNAJC16, SRPX, SERPINA7, PROZ, GPR97, PARM1, APCS, CAR14, PRG4, IGFALS, NFAM1, AMIGO3, CTSO, ERN1, NGFR, LEPREL1, FMOD, CD248, CSF1, GPR64, CXCL9, LRG1, UGT1A5, COL27A1, SHISA5, PKD1, PCSK9, ADAMTS10, SLC39A5, LECT2, SELPLG, THBS3, MPZ, LRRN4, SELL, RNASE4, PM20D1, NPR2, 4930572J05RIK, WNT2B, ABP1, ORM1, GLA, SULF1, ORM2, GFRA2	518	2970	17854	1.14890605	1	0.622553	71.446
UP_SEQ_FEATURE	metal ion-binding site:Iron (heme axial ligand)	7	1.18	0.088697	CYP2A22, CYP2C55, CYP2D11, CYP2A5, CYP4F14, CYB5B, CYB5D1	500	99	16021	2.26559596	1	0.999883	78.787
GOTERM_BP_FAT	GO:0006518~peptide metabolic process	4	0.67	0.089301	GGT6, GGT7, GSTT1, GSTT3	381	38	13588	3.75410968	1	0.991966	79.588
UP_SEQ_FEATURE	binding site:Substrate	14	2.36	0.09003	HAO1, ARSB, NMNAT2, DHFR, HNMT, AKR1C6, RNASE4, PKLR, GAMT, GALE, IDI1, AKR1D1, LIPT1, DDAH1	500	275	16021	1.63122909	1	0.999826	79.299
UP_SEQ_FEATURE	domain:PKD 1	2	0.34	0.090565	AU040320, PKD1	500	3	16021	21.3613333	1	0.999733	79.501
UP_SEQ_FEATURE	repeat:1; approximate	2	0.34	0.090565	CSTF2, PRG4	500	3	16021	21.3613333	1	0.999733	79.501
UP_SEQ_FEATURE	repeat:12; approximate	2	0.34	0.090565	CSTF2, PRG4	500	3	16021	21.3613333	1	0.999733	79.501
UP_SEQ_FEATURE	domain:PKD 2	2	0.34	0.090565	AU040320, PKD1	500	3	16021	21.3613333	1	0.999733	79.501
KEGG_PATHWAY	mmu00590:Arachidonic acid metabolism	6	1.01	0.091578	CYP2C55, GGT6, GGT7, CYP2C44, CYP4F14, CYP2B13	167	83	5738	2.48380348	1	0.686919	67.472
INTERPRO	IPR013149:Alcohol dehydrogenase, zinc-binding	3	0.51	0.093659	SORD, ADH1, FASN	513	18	17763	5.77095517	1	0.916968	78.46

GOTERM_BP_FAT	GO:0000902~cell morphogenesis	14	2.36	0.094261	NRP1, NDN, ESRRB, LEF1, HGF, GLI3, NUMBL, SEMA6A, ATP2B2, FOXF1A, ANK3, 6530402F18RIK, NGFR, NFATC1	381	309	13588	1.61584656	1	0.993015	81.396
SP_PIR_KEYWORDS	FAD	7	1.18	0.096834	CHDH, SQLE, PYROXD1, MAOA, LDHD, AOX1, MICAL1	518	109	17854	2.21348872	1	0.650565	75.181
GOTERM_MF_FAT	GO:0003677~DNA binding	59	9.95	0.097013	POU6F1, XRCC4, XRCC3, ELF3, PAX5, INO80, GLI3, ZKSCAN4, DDX11, HOMEZ, ZFP354A, SPIB, ATOH8, ZFP354B, RTEL1, RDM1, POU2AF1, SP100, BARHL1, MLXIPL, NUSAP1, LEF1, ZFP280D, CUX1, ZFP710, ZFP395, NDN, A730008H23RIK, PML, TSC22D1, MEIS3, TRP53BP1, TCEA3, NR1D1, HJURP, TRDMT1, POU2F1, ETV5, DHX58, NFATC1, ZBP1, SGIP1, CHD3, SREBF1, PDS5B, ESRRB, TREX1, STAT1, TET1, FOXP2, STAT2, FOXF1A, RPAP1, ZFP182, DBP, MZF1, LIME1, ZFHX2, OGG1, KLF1	367	1781	13288	1.19944862	1	0.838758	77.907
KEGG_PATHWAY	mmu04810:Regulation of actin cytoskeleton	11	1.85	0.09711	RAC2, MYLK3, BCAR1, CYFIP2, ACTN1, MYLPF, FGF13, BDKRB2, PIP4K2A, FGF1, FN1	167	217	5738	1.74171473	1	0.677683	69.714
GOTERM_BP_FAT	GO:0051336~regulation of hydrolase activity	10	1.69	0.099539	SGSM1, ADAP2, CHML, PML, ERN1, RGN, TBC1D4, ASAP1, INTS1, ARAP2	381	196	13588	1.81959398	1	0.993994	83.155

Supplementary Table 3.

© 2000-2015 QIAGEN. All rights reserved.

**Ingenuity Canonical Pathways**	**-log(p-value)**	**Ratio**	**Overlap**	**Molecules**
Superpathway of Cholesterol Biosynthes	4.28E00	2.14E-01	6/28	FDPS,SQLE,NSDHL,IDI1,MVK,LSS
LXR/RXR Activation	2.5E00	7.44E-02	9/121	ABCG8,SCD,MLXIPL,LDLR,SREBF1,ITIH4,NGFR,FASN,ACACA
LPS/IL-1 Mediated Inhibition of RXR Fu	2.49E00	5.94E-02	13/219	ABCG8,ABCB1,SLC10A1,ABCB9,Cyp2a12/Cyp2a22,SREBF1,NGFR,CHST3,FABP1,CYP2A6 (includes others),HS3ST6,ABCC4,MAOA
Cholesterol Biosynthesis I	2.45E00	2.31E-01	3/13	SQLE,NSDHL,LSS
Cholesterol Biosynthesis II (via 24,25-di	2.45E00	2.31E-01	3/13	SQLE,NSDHL,LSS
Cholesterol Biosynthesis III (via Desmos	2.45E00	2.31E-01	3/13	SQLE,NSDHL,LSS
IL-15 Production	2.39E00	1.48E-01	4/27	TYK2,MST1R,STAT1,JAK3
Nicotine Degradation II	2.34E00	9.52E-02	6/63	CYP2C18,CYP2A6 (includes others),AOX1,UGT1A4,INMT,Aox3
Trans, trans-farnesyl Diphosphate Biosy	2.24E00	4E-01	2/5	FDPS,IDI1
Superpathway of Geranylgeranyldiphos	2.1E00	1.76E-01	3/17	FDPS,IDI1,MVK
Hepatic Fibrosis / Hepatic Stellate Cell A	1.98E00	5.56E-02	11/198	MET,FN1,CSF1,COL23A1,TNFSF13,NGFR,HGF,COL20A1,STAT1,COL27A1,FGF1
Nicotine Degradation III	1.98E00	9.26E-02	5/54	CYP2C18,CYP2A6 (includes others),AOX1,UGT1A4,Aox3
Maturity Onset Diabetes of Young (MOD	1.79E00	1.36E-01	3/22	PKLR,SLC2A2,FABP1
TR/RXR Activation	1.73E00	7.06E-02	6/85	LDLR,SREBF1,FASN,ACACA,RCAN2,THRSP
Actin Cytoskeleton Signaling	1.71E00	5.07E-02	11/217	RAC2,FN1,CYFIP2,MYLPF,MYLK3,PIP4K2A,BCAR1,ACTN1,FGF13,FGF1,MATK
Role of JAK1, JAK2 and TYK2 in Interfe	1.69E00	1.25E-01	3/24	TYK2,STAT2,STAT1
Activation of IRF by Cytosolic Pattern R	1.68E00	7.81E-02	5/64	DHX58,ZBP1,STAT2,STAT1,IFIT2
Sorbitol Degradation I	1.61E00	1E00	1/1	SORD
Lipoate Salvage and Modification	1.61E00	1E00	1/1	LIPT1
Methylglyoxal Degradation VI	1.61E00	1E00	1/1	LDHD
Lanosterol Biosynthesis	1.61E00	1E00		LSS
UDP-N-acetyl-D-galactosamine Biosynt	1.61E00	1E00		GALE
FXR/RXR Activation	1.42E00	5.51E-02		ABCG8,MLXIPL,SLC10A1,PKLR,SREBF1,ITIH4,FASN
Primary Immunodeficiency Signaling	1.41E00	7.69E-02		BLNK,ICOS,JAK3,TNFRSF13C
Oleate Biosynthesis II (Animals)	1.4E00	1.54E-01		SCD,Scd2
Mevalonate Pathway I	1.4E00	1.54E-01		IDI1,MVK
Bile Acid Biosynthesis, Neutral Pathway	1.4E00	1.54E-01		AKR1D1,AKR1C4
Semaphorin Signaling in Neurons	1.38E00	7.55E-02		RND2,MET,DPYSL4,NRP1
Role of BRCA1 in DNA Damage Respor	1.36E00	6.41E-02		POU2F1,DPF1,FANCG,STAT1,RBL1
Epoxysqualene Biosynthesis	1.31E00	5E-01		SQLE
Lipoate Biosynthesis and Incorporation	1.31E00	5E-01		LIPT1
Choline Degradation I	1.31E00	5E-01		CHDH
Palmitate Biosynthesis I (Animals)	1.31E00	5E-01		FASN
Fatty Acid Biosynthesis Initiation II	1.31E00	5E-01		FASN
Glycine Degradation (Creatine Biosynthe	1.31E00	5E-01		GAMT
Oncostatin M Signaling	1.29E00	8.82E-02		TYK2,STAT1,JAK3
Interferon Signaling	1.29E00	8.82E-02		TYK2,STAT2,STAT1
Androgen Biosynthesis	1.29E00	1.33E-01		HSD3B1,AKR1C4
Leukotriene Biosynthesis	1.29E00	1.33E-01		GGT6,GGT7
γ-glutamyl Cycle	1.29E00	1.33E-01		GGT6,GGT7
IL-17A Signaling in Fibroblasts	1.26E00	8.57E-02		NFKBID,TRAF3IP2,LCN2
tRNA Splicing	1.26E00	8.57E-02		PDE6C,TDP2,PDE5A
Triacylglycerol Biosynthesis	1.26E00	8.57E-02		PPAPDC1B,GPAT2,ELOVL6
Stearate Biosynthesis I (Animals)	1.26E00	8.57E-02		FASN,ACOT1,ELOVL6
phagosome formation	1.24E00	5.41E-02		RND2,PLCB4,FN1,TLR7,PRKD3,FCGR1A
Estrogen Biosynthesis	1.2E00	8.11E-02		CYP2C18,CYP2A6 (includes others),AKR1C4
Superpathway of Melatonin Degradation	1.17E00	6.45E-02		CYP2C18,CYP2A6 (includes others),UGT1A4,MAOA
Methionine Salvage II (Mammalian)	1.14E00	3.33E-01		MTR
Axonal Guidance Signaling	1.14E00	3.7E-02		RAC2,NRP2,MYLPF,WNT2B,SEMA6A,GNA14,BCAR1,NFATC1,MET,MICAL1,PLCB4,GLI3,NGFR,SHANK2,PRKD3,NRP1
Granulocyte Adhesion and Diapedesis	1.14E00	4.52E-02		SELL,MMP28,CLDN4,NGFR,Cxcl9,GLG1,SELPLG,FPR1
PXR/RXR Activation	1.08E00	5.97E-02		ABCB1,SCD,CYP2A6 (includes others),ABCB9
DNA Methylation and Transcriptional Re	1.06E00	1E-01		CHD3,SAP130
Regulation of the Epithelial-Mesenchym	1.06E00	4.35E-02		MET,HGF,WNT2B,TYK2,LEF1,JAK3,FGF13,FGF1
GDNF Family Ligand-Receptor Interacti	1.06E00	5.88E-02		DOK4,DOK7,GFRA2,DOK3
Macropinocytosis Signaling	1.06E00	5.88E-02		MET,CSF1,HGF,PRKD3
iNOS Signaling	1.02E00	6.82E-02		TYK2,STAT1,JAK3
α-tocopherol Degradation	1.02E00	2.5E-01		CYP4F12

Geranylgeranyldiphosphate Biosynthesi	1.02E00	2.5E-01		FDPS
Biotin-carboxyl Carrier Protein Assembly	1.02E00	2.5E-01		ACACA
Melatonin Degradation II	1.02E00	2.5E-01		MAOA
Tec Kinase Signaling	1.01E00	4.43E-02		RND2,TYK2,STAT2,GNA14,STAT1,JAK3,PRKD3
JAK/Stat Signaling	9.89E-01	5.56E-02		TYK2,STAT2,STAT1,JAK3
Hepatic Cholestasis	9.69E-01	4.32E-02		ABCG8,ABCB1,SLC10A1,SREBF1,NGFR,MAP3K4,PRKD3
TREM1 Signaling	9.41E-01	5.33E-02		NLRC5,NLRP6,TLR7,LAT2
Galactose Degradation I (Leloir Pathway	9.3E-01	2E-01		GALE
dTMP De Novo Biosynthesis	9.3E-01	2E-01		DHFR
Acetate Conversion to Acetyl-CoA	9.3E-01	2E-01		ACSS1
IL-22 Signaling	9.29E-01	8.33E-02		TYK2,STAT1
Leukocyte Extravasation Signaling	9.26E-01	4.04E-02		RAC2,MMP28,CLDN4,MAP3K4,PRKD3,BCAR1,ACTN1,SELPLG
HGF Signaling	9.26E-01	4.76E-02		MET,ELF3,HGF,MAP3K4,PRKD3
Role of Macrophages, Fibroblasts and E	9.14E-01	3.69E-02		PLCB4,FN1,TRAF3IP2,CSF1,NGFR,WNT2B,TLR7,LEF1,PRKD3,FCGR1A,NFATC1
Role of JAK family kinases in IL-6-type (	9E-01	8E-02		TYK2,STAT1
Bupropion Degradation	9E-01	8E-02		CYP2C18,CYP2A6 (includes others)
Acetone Degradation I (to Methylglyoxal	8.72E-01	7.69E-02		CYP2C18,CYP2A6 (includes others)
Zymosterol Biosynthesis	8.56E-01	1.67E-01		NSDHL
NAD Biosynthesis III	8.56E-01	1.67E-01		NMNAT2
D-myo-inositol (1,4,5)-Trisphosphate Bio	8.45E-01	7.41E-02		PLCB4,PIP4K2A
Epithelial Adherens Junction Signaling	8.13E-01	4.11E-02		MET,HGF,PVRL3,LEF1,ACTN1,FGF1
FGF Signaling	7.99E-01	4.71E-02		MET,HGF,FGF13,FGF1
Production of Nitric Oxide and Reactive	7.99E-01	3.89E-02		RND2,NGFR,TYK2,MAP3K4,STAT1,JAK3,PRKD3
Wnt/Ca+ pathway	7.95E-01	5.36E-02		PLCB4,PDE6C,NFATC1
NAD Biosynthesis from 2-amino-3-carbo	7.94E-01	1.43E-01		NMNAT2
NAD Salvage Pathway III	7.94E-01	1.43E-01		NMNAT2
Pregnenolone Biosynthesis	7.94E-01	1.43E-01		MICAL1
Melatonin Degradation I	7.79E-01	5.26E-02		CYP2C18,CYP2A6 (includes others),UGT1A4
Glutathione-mediated Detoxification	7.72E-01	6.67E-02		Gstt3,Gstt1
Altered T Cell and B Cell Signaling in Rh	7.62E-01	4.55E-02		CSF1,TNFSF13,TLR7,TNFRSF13C
CXCR4 Signaling	7.57E-01	3.95E-02		RND2,PLCB4,MYLPF,GNA14,PRKD3,BCAR1
Aldosterone Signaling in Epithelial Cells	7.57E-01	3.95E-02		SCNN1A,PLCB4,DNAJC9,PIP4K2A,PRKD3,DNAJC16
Glucose and Glucose-1-phosphate Degr	7.41E-01	1.25E-01		RGN
G-Protein Coupled Receptor Signaling	7.34E-01	3.52E-02		GABBR2,GRK4,PLCB4,LTB4R,PDE6C,TDP2,PDE5A,GNA14,FPR1
Regulation of Actin-based Motility by Rh	7.26E-01	4.4E-02		RND2,RAC2,MYLPF,PIP4K2A
Communication between Innate and Ada	7.26E-01	4.4E-02		TNFSF13,TLR7,TNFRSF13C,CD8B
Agranulocyte Adhesion and Diapedesis	7.26E-01	3.7E-02		SELL,FN1,MMP28,CLDN4,Cxcl9,GLG1,SELPLG
Circadian Rhythm Signaling	7.08E-01	6.06E-02		PER3,NR1D1
Retinoate Biosynthesis I	7.08E-01	6.06E-02		ADH1C,AKR1C4
Ethanol Degradation II	7.08E-01	6.06E-02		ADH1C,ACSS1
Calcium Transport I	6.95E-01	1.11E-01		ATP2B2
UDP-N-acetyl-D-galactosamine Biosynth	6.95E-01	1.11E-01		GALE
Folate Transformations I	6.95E-01	1.11E-01		MTR
Role of JAK1 and JAK3 in γc Cytokine S	6.91E-01	4.76E-02		BLNK,STAT1,JAK3
IL-9 Signaling	6.89E-01	5.88E-02		STAT1,JAK3
Germ Cell-Sertoli Cell Junction Signaling	6.89E-01	3.75E-02		RND2,RAC2,PVRL3,MAP3K4,BCAR1,ACTN1
IL-17A Signaling in Airway Cells	6.77E-01	4.69E-02		TRAF3IP2,TYK2,JAK3
Serotonin Degradation	6.77E-01	4.69E-02		ADH1C,UGT1A4,MAOA
PI3K Signaling in B Lymphocytes	6.77E-01	3.91E-02		BLNK,PLCB4,DAPP1,PLEKHA2,NFATC1
Coagulation System	6.7E-01	5.71E-02		BDKRB2,PLAT
Role of JAK2 in Hormone-like Cytokine	6.7E-01	5.71E-02		TYK2,STAT1
Noradrenaline and Adrenaline Degradat	6.7E-01	5.71E-02		ADH1C,MAOA
Histidine Degradation VI	6.55E-01	1E-01		MICAL1
Colorectal Cancer Metastasis Signaling	6.38E-01	3.39E-02		RND2,MMP28,WNT2B,TYK2,TLR7,LEF1,STAT1,JAK3
Remodeling of Epithelial Adherens Junc	6.27E-01	4.41E-02		MET,HGF,ACTN1
Cholecystokinin/Gastrin-mediated Signa	6.2E-01	3.96E-02		RND2,PLCB4,PRKD3,BCAR1
April Mediated Signaling	6.17E-01	5.26E-02		TNFSF13,NFATC1
Relaxin Signaling	6.16E-01	3.7E-02		PDE6C,TDP2,PDE5A,GNA14,NPR2
Growth Hormone Signaling	6.15E-01	4.35E-02		IGFALS,STAT1,PRKD3
Protein Kinase A Signaling	6.1E-01	3.12E-02		PLCB4,GLI3,MYLPF,NGFR,CDC14B,PDE6C,MYLK3,TDP2,PDE5A,LEF1,PRKD3,NFATC1
Role of NFAT in Regulation of the Immu	6.04E-01	3.51E-02		BLNK,PLCB4,GNA14,FCGR1A,RCAN2,NFATC1
Netrin Signaling	6.01E-01	5.13E-02		RAC2,NFATC1
T Helper Cell Differentiation	5.92E-01	4.23E-02		NGFR,ICOS,STAT1
Cleavage and Polyadenylation of Pre-m	5.86E-01	8.33E-02		CSTF2

Hematopoiesis from Multipotent Stem C	5.86E-01	8.33E-02		CSF1
Acyl-CoA Hydrolysis	5.86E-01	8.33E-02		ACOT1
Mineralocorticoid Biosynthesis	5.86E-01	8.33E-02		HSD3B1
BER pathway	5.86E-01	8.33E-02		OGG1
Role of PKR in Interferon Induction and	5.85E-01	5E-02		STAT1,FCGR1A
B Cell Activating Factor Signaling	5.85E-01	5E-02		TNFRSF13C,NFATC1
Basal Cell Carcinoma Signaling	5.8E-01	4.17E-02		GLI3,WNT2B,LEF1
B Cell Receptor Signaling	5.69E-01	3.41E-02		BLNK,PAX5,RAC2,DAPP1,MAP3K4,NFATC1
Ubiquinol-10 Biosynthesis (Eukaryotic)	5.56E-01	7.69E-02		MICAL1
Histamine Degradation	5.56E-01	7.69E-02		HNMT
Guanosine Nucleotides Degradation III	5.56E-01	7.69E-02		AOX1
Glucocorticoid Biosynthesis	5.56E-01	7.69E-02		HSD3B1
fMLP Signaling in Neutrophils	5.56E-01	3.7E-02		PLCB4,PRKD3,NFATC1,FPR1
Calcium Signaling	5.56E-01	3.37E-02		TNNT1,HDAC11,RCAN2,Tpm2,NFATC1,ATP2B2
VEGF Family Ligand-Receptor Interactio	5.38E-01	3.95E-02		NRP2,PRKD3,NRP1
IL-4 Signaling	5.38E-01	3.95E-02		TYK2,JAK3,NFATC1
Molecular Mechanisms of Cancer	5.32E-01	3.01E-02		RND2,RAC2,PLCB4,WNT2B,TYK2,LEF1,GNA14,RBL1,JAK3,PRKD3,BCL2L11
DNA Double-Strand Break Repair by No	5.29E-01	7.14E-02		XRCC4
Urate Biosynthesis/Inosine 5'-phosphate	5.29E-01	7.14E-02		AOX1
Phenylalanine Degradation IV (Mammal	5.29E-01	7.14E-02		MAOA
Colanic Acid Building Blocks Biosynthes	5.29E-01	7.14E-02		GALE
PDGF Signaling	5.28E-01	3.9E-02		TYK2,STAT1,JAK3
Dermatan Sulfate Biosynthesis (Late Sta	5.28E-01	4.55E-02		CHST3,HS3ST6
cAMP-mediated signaling	5.25E-01	3.2E-02		GABBR2,GRK4,LTB4R,PDE6C,TDP2,PDE5A,FPR1
Gαq Signaling	5.25E-01	3.4E-02		RND2,PLCB4,GNA14,PRKD3,NFATC1
NAD biosynthesis II (from tryptophan)	5.05E-01	6.67E-02		NMNAT2
Chondroitin Sulfate Degradation (Metazo	5.05E-01	6.67E-02		ARSB
MSP-RON Signaling Pathway	5.02E-01	4.35E-02		CSF1,MST1R
Chondroitin Sulfate Biosynthesis (Late S	5.02E-01	4.35E-02		CHST3,HS3ST6
Type II Diabetes Mellitus Signaling	4.84E-01	3.42E-02		PKLR,NGFR,SLC2A2,PRKD3
CDP-diacylglycerol Biosynthesis I	4.82E-01	6.25E-02		GPAT2
Oxidative Ethanol Degradation III	4.82E-01	6.25E-02		ACSS1
Ephrin A Signaling	4.77E-01	4.17E-02		NGFR,BCAR1
PKCθ Signaling in T Lymphocytes	4.77E-01	3.39E-02		RAC2,POU2F1,MAP3K4,NFATC1
PTEN Signaling	4.77E-01	3.39E-02		RAC2,NGFR,BCAR1,BCL2L11
GPCR-Mediated Nutrient Sensing in Ent	4.64E-01	3.57E-02		PLCB4,GNA14,PRKD3
Adenosine Nucleotides Degradation II	4.6E-01	5.88E-02		AOX1
Putrescine Degradation III	4.6E-01	5.88E-02		MAOA
Gustation Pathway	4.56E-01	3.31E-02		SCNN1A,PDE6C,TDP2,PDE5A
Heparan Sulfate Biosynthesis (Late Stag	4.54E-01	4E-02		CHST3,HS3ST6
Xenobiotic Metabolism Signaling	4.5E-01	2.95E-02		ABCB1,ANKRA2,CHST3,HS3ST6,MAP3K4,UGT1A4,PRKD3,MAOA
RhoA Signaling	4.49E-01	3.28E-02		NRP2,MYLPF,MYLK3,PIP4K2A
Hematopoiesis from Pluripotent Stem C	4.43E-01	3.92E-02		CSF1,CD8B
Tryptophan Degradation X (Mammalian,	4.4E-01	5.56E-02		MAOA
Phosphatidylglycerol Biosynthesis II (No	4.4E-01	5.56E-02		GPAT2
Bladder Cancer Signaling	4.39E-01	3.45E-02		MMP28,FGF13,FGF1
CNTF Signaling	4.33E-01	3.85E-02		TYK2,STAT1
Neuregulin Signaling	4.31E-01	3.41E-02		BTC,PRKD3,MATK
UVA-Induced MAPK Signaling	4.31E-01	3.41E-02		PLCB4,ZC3HAV1,STAT1
Integrin Signaling	4.23E-01	2.99E-02		RND2,RAC2,ASAP1,MYLK3,BCAR1,ACTN1
Phototransduction Pathway	4.23E-01	3.77E-02		RGS9,PDE6C
Phospholipase C Signaling	4.22E-01	2.93E-02		RND2,BLNK,PLCB4,HDAC11,MYLPF,PRKD3,NFATC1
GADD45 Signaling	4.22E-01	5.26E-02		MAP3K4
Glutathione Redox Reactions I	4.22E-01	5.26E-02		Gstt1
Ethanol Degradation IV	4.22E-01	5.26E-02		ACSS1
Adipogenesis pathway	4.16E-01	3.15E-02		SREBF1,HDAC11,SAP130,FGF1
Chondroitin Sulfate Biosynthesis	4.13E-01	3.7E-02		CHST3,HS3ST6
Unfolded protein response	4.13E-01	3.7E-02		SREBF1,ERN1
Purine Nucleotides Degradation II (Aero	4.05E-01	5E-02		AOX1
GNRH Signaling	4.03E-01	3.1E-02		PLCB4,GNA14,MAP3K4,PRKD3
Thrombopoietin Signaling	4.03E-01	3.64E-02		STAT1,PRKD3
Fcγ Receptor-mediated Phagocytosis in	3.93E-01	3.23E-02		RAC2,PRKD3,FCGR1A
Endoplasmic Reticulum Stress Pathway	3.89E-01	4.76E-02		ERN1
SAPK/JNK Signaling	3.86E-01	3.19E-02		RAC2,MAP3K4,NFATC1
ErbB2-ErbB3 Signaling	3.84E-01	3.51E-02		TYK2,JAK3
Heparan Sulfate Biosynthesis	3.84E-01	3.51E-02		CHST3,HS3ST6

Dermatan Sulfate Biosynthesis	3.84E-01	3.51E-02		CHST3,HS3ST6
Mouse Embryonic Stem Cell Pluripotenc	3.79E-01	3.16E-02		TYK2,LEF1,JAK3
Pyrimidine Deoxyribonucleotides De No	3.73E-01	4.55E-02		AK1
Differential Regulation of Cytokine Produ	3.59E-01	4.35E-02		LCN2
Sertoli Cell-Sertoli Cell Junction Signalin	3.47E-01	2.81E-02		CLDN4,PVRL3,MAP3K4,BCAR1,ACTN1
Nitric Oxide Signaling in the Cardiovasc	3.46E-01	3E-02		BDKRB2,PDE5A,PRKD3
Estrogen-mediated S-phase Entry	3.45E-01	4.17E-02		RBL1
Dopamine Degradation	3.45E-01	4.17E-02		MAOA
PPARα/RXRα Activation	3.43E-01	2.79E-02		PLCB4,FASN,CYP2C18,Cyp2c44,GNA14
Dendritic Cell Maturation	3.43E-01	2.79E-02		PLCB4,NGFR,STAT2,STAT1,FCGR1A
NRF2-mediated Oxidative Stress Respo	3.38E-01	2.78E-02		DNAJC9,AOX1,PRKD3,DNAJC16,ABCC4
Antiproliferative Role of Somatostatin R	3.34E-01	3.17E-02		SSTR2,NPR2
Role of Lipids/Lipid Rafts in the Pathoge	3.32E-01	4E-02		FDPS
Glycolysis I	3.32E-01	4E-02		PKLR
Synaptic Long Term Depression	3.31E-01	2.82E-02		PLCB4,GNA14,PRKD3,NPR2
Cell Cycle: G1/S Checkpoint Regulation	3.26E-01	3.12E-02		HDAC11,RBL1
NAD Salvage Pathway II	3.2E-01	3.85E-02		NMNAT2
IL-15 Signaling	3.12E-01	3.03E-02		TYK2,JAK3
Glioblastoma Multiforme Signaling	3.11E-01	2.74E-02		RND2,PLCB4,WNT2B,LEF1
Pancreatic Adenocarcinoma Signaling	3.11E-01	2.83E-02		TYK2,STAT1,JAK3
ERK/MAPK Signaling	3.08E-01	2.67E-02		RAC2,ELF3,STAT1,BCAR1,NFATC1
GABA Receptor Signaling	3.05E-01	2.99E-02		GABBR2,GABRB3
AMPK Signaling	3.02E-01	2.7E-02		AK1,DPF1,FASN,ACACA
iCOS-iCOSL Signaling in T Helper Cells	3E-01	2.78E-02		ICOS,PLEKHA2,NFATC1
Sphingosine-1-phosphate Signaling	2.95E-01	2.75E-02		RND2,PLCB4,S1PR5
Thrombin Signaling	2.91E-01	2.62E-02		RND2,PLCB4,MYLPF,GNA14,PRKD3
Agrin Interactions at Neuromuscular Jur	2.91E-01	2.9E-02		RAC2,PKLR
Pyrimidine Ribonucleotides Interconvers	2.87E-01	3.45E-02		AK1
Melatonin Signaling	2.85E-01	2.86E-02		PLCB4,PRKD3
Corticotropin Releasing Hormone Signa	2.85E-01	2.7E-02		GLI3,PRKD3,NPR2
Role of NANOG in Mammalian Embryon	2.85E-01	2.7E-02		WNT2B,TYK2,JAK3
IL-3 Signaling	2.79E-01	2.82E-02		STAT1,PRKD3
Renal Cell Carcinoma Signaling	2.79E-01	2.82E-02		MET,HGF
GPCR-Mediated Integration of Enteroen	2.79E-01	2.82E-02		PLCB4,GNA14
Sonic Hedgehog Signaling	2.77E-01	3.33E-02		GLI3
Pyrimidine Ribonucleotides De Novo Bio	2.68E-01	3.23E-02		AK1
Prolactin Signaling	2.66E-01	2.74E-02		STAT1,PRKD3
Ephrin B Signaling	2.66E-01	2.74E-02		RAC2,GNA14
STAT3 Pathway	2.66E-01	2.74E-02		NGFR,TYK2
FLT3 Signaling in Hematopoietic Proger	2.6E-01	2.7E-02		STAT2,STAT1
Superpathway of Methionine Degradatio	2.59E-01	3.12E-02		MTR
G Protein Signaling Mediated by Tubby	2.5E-01	3.03E-02		PLCB4
Synaptic Long Term Potentiation	2.47E-01	2.52E-02		PLCB4,GNA14,PRKD3
Acute Myeloid Leukemia Signaling	2.44E-01	2.6E-02		LEF1,PML
HMGB1 Signaling	2.42E-01	2.5E-02		RND2,NGFR,PLAT
Gαi Signaling	2.42E-01	2.5E-02		GABBR2,LTB4R,FPR1
VDR/RXR Activation	2.38E-01	2.56E-02		SERPINB1,PRKD3
Sperm Motility	2.38E-01	2.48E-02		PLCB4,MST1R,PRKD3
Thyroid Hormone Metabolism II (via Cor	2.34E-01	2.86E-02		UGT1A4
Role of Wnt/GSK-3β Signaling in the Pa	2.33E-01	2.53E-02		WNT2B,LEF1
Ceramide Signaling	2.28E-01	2.5E-02		S1PR5,NGFR
Antigen Presentation Pathway	2.19E-01	2.7E-02		NLRC5
Notch Signaling	2.12E-01	2.63E-02		NUMBL
tRNA Charging	2.05E-01	2.56E-02		TARSL2
Inhibition of Matrix Metalloproteases	2.05E-01	2.56E-02		MMP28
Thyroid Cancer Signaling	1.99E-01	2.5E-02		LEF1

Supplementary Table 4.

Category	Term	Count	%	PValue	Genes	List Total	Pop Hits	Pop Total	Fold Enrichment	Bonferroni	Benjamini	FDR
SP_PIR_KEYWORDS	extracellular matrix	15	5.682	7.50E-07	COL4A2, PXDN, FBN1, ELN, COL3A1, ADAMTS15, NID1, SPARC, MMP2, COL5A1, SMOC2, LEPRE1, COL1A2, COL6A1,	232	213	17854	5.419	2.08E-04	2.08E-04	9.90E-04
GOTERM_MF_FAT	GO:0005201~extracellular matrix structural constituent	7	2.652	2.05E-06	COL4A2, FBN1, ELN, COL3A1, COL1A2, COL1A1, COL5A1	174	30	13288	17.82	7.12E-04	7.12E-04	0.0028
SP_PIR_KEYWORDS	Secreted	41	15.53	2.31E-06	PXDN, NRG4, TNF, LEPR, HSD17B13, ADAMTS15, COL3A1, FST, ELN, PF4, FSTL1, CX3CL1, MMP2, MFRP, AHSG, SMOC2, C1QTNF6, C1QTNF4, COL6A1, DEFB1, MUP11, COL4A2, SVEP1, NTF3, GZMA, FBN1, IL1RN, NID1, SERPINA1E, FGF21, SPARC, COL5A1, CHRDL2, LEPRE1, COL1A2, HBEGF, EGFL8, COL1A1, GDF15, IGFBP2, TREM2	232	1420	17854	2.222	6.39E-04	3.19E-04	0.00304
SP_PIR_KEYWORDS	triple helix	5	1.894	3.27E-06	COL4A2, COL3A1, COL1A2, COL6A1, COL1A1	232	9	17854	42.75	9.05E-04	3.02E-04	0.00432
GOTERM_MF_FAT	GO:0048407~platelet-derived growth factor binding	5	1.894	3.32E-06	COL3A1, COL1A2, COL6A1, COL1A1, COL5A1	174	9	13288	42.43	0.001156	5.78E-04	0.00454
GOTERM_CC_FAT	GO:0005576~extracellular region	45	17.05	3.50E-06	PXDN, NRG4, TNF, LEPR, HSD17B13, ADAMTS15, COL3A1, FST, ELN, PF4, FSTL1, CX3CL1, MMP2, MFRP, AHSG, SMOC2, C1QTNF6, C1QTNF4, GLIPR1, COL6A1, CASP1, DEFB1, MUP11, COL4A2, SVEP1, NGP, NTF3, GZMA, FBN1, IL1RN, NID1, SERPINA1E, FGF21, SPARC, COL5A1, CHRDL2, LEPRE1, COL1A2, HBEGF, EGFL8, COL1A1, GDF15, ADAM12, IGFBP2,	165	1680	12504	2.03	6.83E-04	6.83E-04	0.00437
GOTERM_CC_FAT	GO:0031012~extracellular matrix	16	6.061	1.34E-05	COL4A2, FBN1, ELN, ADAMTS15, COL3A1, NID1, SPARC, MMP2, COL5A1, AHSG, SMOC2, LEPRE1, COL1A2, COL6A1, COL1A1, ADAM12	165	309	12504	3.924	0.002601	0.001301	0.01667
GOTERM_CC_FAT	GO:0044420~extracellular matrix part	9	3.409	2.70E-05	SMOC2, COL4A2, FBN1, COL3A1, COL1A2, NID1, SPARC, COL1A1, COL5A1	165	92	12504	7.413	0.005248	0.001752	0.03368
GOTERM_CC_FAT	GO:0005578~proteinaceous extracellular matrix	15	5.682	3.62E-05	COL4A2, FBN1, ELN, COL3A1, ADAMTS15, NID1, SPARC, MMP2, COL5A1, SMOC2, LEPRE1, COL1A2, COL6A1,	165	297	12504	3.827	0.007036	0.001764	0.04519
GOTERM_MF_FAT	GO:0070330~aromatase activity	6	2.273	7.23E-05	CYP2D9, CYP4A12A, CYP1A1, CYP3A11, CYP2C67, CYP2C40, CYP2B10	174	34	13288	13.48	0.024862	0.008357	0.09881
INTERPRO	IPR008160:Collagen triple helix repeat	8	3.03	7.48E-05	COL4A2, C1QTNF6, COL3A1, COL1A2, COL6A1, COL1A1, COL5A1, MFRP	226	81	17763	7.763	0.035928	0.035928	0.10736
UP_SEQ_FEATURE	domain:VWFC	5	1.894	8.50E-05	PXDN, COL3A1, FSTL1, COL1A1, AMN	231	17	16021	20.4	0.06314	0.06314	0.12972
GOTERM_CC_FAT	GO:0005581~collagen	5	1.894	9.48E-05	COL4A2, COL3A1, COL1A2, COL1A1, COL5A1	165	19	12504	19.94	0.018321	0.003691	0.11829

SP_PIR_KEYWORDS	signal	62	23.48	1.05E-04	LY9, LRRC15, AMN, C530028O21RIK, MMP2, CD93, DSEL, TREH, MUP11, GZMA, ERP29, DLL1, FGF21, CHRDL2, COL1A2, CD300LF, EGFL8, COL1A1, ADAM12, TREM2, CTSF, EMCN, HSD17B13, ADAMTS15, COL3A1, ELN, PF4, CX3CL1, SERPINH1, MFRP, AHSG, VCAM1, LY6A, SMOC2, C1QTNF6, C1QTNF4, GLIPR1, NUP210, COL6A1, GPNMB, DEFB1, LYZ2, COL4A2, LYZ1, SVEP1, NTF3, IL1RN, FBN1, NID1, SERPINA1E, SPARC, COL5A1, LAMP3, LEPRE1, HBEGF, GDF15, IGFBP2	232	2970	17854	1.607	0.028785	0.007275	0.13909
SP_PIR_KEYWORDS	collagen	8	3.03	1.08E-04	COL4A2, C1QTNF6, COL3A1, COL1A2, COL6A1, COL1A1, COL5A1, MFRP	232	84	17854	7.329	0.029411	0.005953	0.14216
UP_SEQ_FEATURE	region of interest:Triple-helical region	5	1.894	1.08E-04	COL4A2, COL3A1, COL6A1, COL1A1, COL5A1	231	18	16021	19.27	0.079572	0.04061	0.16489
SP_PIR_KEYWORDS	hydroxylation	7	2.652	1.76E-04	COL4A2, ELN, COL3A1, COL1A2, COL6A1, COL1A1, COL5A1	232	64	17854	8.417	0.047631	0.008101	0.2323
INTERPRO	IPR001007:von Willebrand factor, type C	6	2.273	1.81E-04	CHRDL2, PXDN, COL3A1, FSTL1, COL1A1, AMN	226	42	17763	11.23	0.084758	0.043317	0.25967
GOTERM_CC_FAT	GO:0044421~extracellular region part	24	9.091	1.90E-04	COL4A2, TNF, LEPR, FBN1, ELN, ADAMTS15, IL1RN, COL3A1, NID1, PF4, CX3CL1, SPARC, MMP2, COL5A1, AHSG, SMOC2, LEPRE1, COL1A2, COL6A1, HBEGF, COL1A1, IGFBP2, GDF15,	165	774	12504	2.35	0.036411	0.006163	0.23714
GOTERM_MF_FAT	GO:0016712~oxidoreductase activity, acting on paired donors, with incorporation or reduction of molecular oxygen, reduced flavin or flavoprotein as one donor, and incorporation of one atom of oxygen	6	2.273	2.54E-04	CYP2D9, CYP4A12A, CYP1A1, CYP3A11, CYP2C67, CYP2C40, CYP2B10	174	44	13288	10.41	0.084669	0.021875	0.34679
SMART	SM00214:VWC	6	2.273	2.91E-04	CHRDL2, PXDN, COL3A1, FSTL1, COL1A1, AMN	130	42	9131	10.03	0.03492	0.03492	0.33489
INTERPRO	IPR000885:Fibrillar collagen, C-terminal	4	1.515	3.07E-04	COL3A1, COL1A2, COL1A1, COL5A1	226	11	17763	28.58	0.139402	0.048811	0.43975
UP_SEQ_FEATURE	domain:Fibrillar collagen NC1	4	1.515	3.25E-04	COL3A1, COL1A2, COL1A1, COL5A1	231	10	16021	27.74	0.22084	0.079814	0.49541
GOTERM_BP_FAT	GO:0006259~DNA metabolic process	16	6.061	3.38E-04	DNMT3A, FHIT, ING4, HMGB2, APEX2, NASP, EME1, POT1A, MCM6, RFC5, DCLRE1C, EPC2, RFC4, EEF1E1, TDG, DNMT3B	175	421	13588	2.951	0.313056	0.313056	0.54013
GOTERM_MF_FAT	GO:0019838~growth factor binding	7	2.652	3.43E-04	NTF3, COL3A1, COL1A2, COL6A1, COL1A1, IGFBP2, COL5A1	174	72	13288	7.425	0.112438	0.023573	0.46728

SMART	SM00038:COLFI	4	1.515	4.18E-04	COL3A1, COL1A2, COL1A1, COL5A1	130	11	9131	25.54	0.049775	0.025205	0.48068
SP_PIR_KEYWORDS	collagen binding	3	1.136	9.83E-04	NID1, SPARC, SERPINH1	232	4	17854	57.72	0.238486	0.038173	1.28995
GOTERM_BP_FAT	GO:0044036~cell wall macromolecule metabolic process	4	1.515	0.001022	LYZ2, LYZ1, LYSMD1, LYSMD4	175	16	13588	19.41	0.678683	0.433152	1.62418
GOTERM_BP_FAT	GO:0016998~cell wall macromolecule catabolic process	4	1.515	0.001022	LYZ2, LYZ1, LYSMD1, LYSMD4	175	16	13588	19.41	0.678683	0.433152	1.62418
INTERPRO	IPR006209:EGF	8	3.03	0.001233	NRG4, SVEP1, CD93, FBN1, HBEGF, DLL1, NID1, EGFL8	226	128	17763	4.912	0.452973	0.139992	1.75537
SP_PIR_KEYWORDS	disulfide bond	50	18.94	0.001305	[illegible] LEPR, FST, FSTL1, LY9, MMP2, CD93, CXCR4, TREH, MUP11, GZMA, DLL1, SAG, CCR2, CD300LF, EGFL8, TREM2, ADAM12, CTSF, TNF, ADAMTS15, ELN, COL3A1, PF4, CX3CL1, MFRP, AHSG, VCAM1, LY6A, SMOC2, ADRA2A, GALNT11, DEFB1, LYZ2, COL4A2, LYZ1, SVEP1, NTF3, FBN1, IL1RN, NID1, SPARC, TFRC, HBEGF, IGFBP2,	232	2469	17854	1.558	0.303564	0.044215	1.70928
INTERPRO	IPR015369:Follistatin/Osteonectin-like EGF domain	3	1.136	0.001558	FST, FSTL1, SPARC	226	5	17763	47.16	0.533411	0.141408	2.21301
UP_SEQ_FEATURE	signal peptide	62	23.48	0.001586	LY9, LRRC15, AMN, C530028O21RIK, MMP2, CD93, DSEL, TREH, MUP11, GZMA, ERP29, DLL1, FGF21, CHRDL2, COL1A2, CD300LF, EGFL8, COL1A1, ADAM12, TREM2, CTSF, EMCN, HSD17B13, ADAMTS15, COL3A1, ELN, PF4, CX3CL1, SERPINH1, MFRP, AHSG, VCAM1, LY6A, SMOC2, C1QTNF6, C1QTNF4, GLIPR1, NUP210, COL6A1, GPNMB, DEFB1, LYZ2, COL4A2, LYZ1, SVEP1, NTF3, IL1RN, FBN1, NID1, SERPINA1E, SPARC, COL5A1, LAMP3, LEPRE1, HBEGF, GDF15, IGFBP2	231	2963	16021	1.451	0.704093	0.262454	2.39439
GOTERM_BP_FAT	GO:0030198~extracellular matrix organization	7	2.652	0.001878	SMOC2, TNF, ELN, COL3A1, NID1, SERPINH1, COL5A1	175	101	13588	5.381	0.875935	0.501249	2.9652
PIR_SUPERFAMILY	PIRSF002255:collagen alpha 1(I) chain	3	1.136	0.001893	COL3A1, COL1A2, COL1A1	115	5	8136	42.45	0.206418	0.206418	2.15828
SP_PIR_KEYWORDS	microsome	7	2.652	0.001906	CYP2D9, CYP4A12A, CYP1A1, CYP3A11, CYP2C67, FMO3, CYP2C40, CYP2B10	232	100	17854	5.387	0.410415	0.057014	2.48635
KEGG_PATHWAY	mmu00830:Retinol metabolism	6	2.273	0.002306	CYP4A12A, CYP1A1, CYP3A11, CYP2C67, CYP2C40, ADH7, CYP2B10	80	68	5738	6.329	0.218874	0.218874	2.56161
SP_PIR_KEYWORDS	Monooxygenase	7	2.652	0.002809	CYP2D9, CYP4A12A, CYP1A1, CYP3A11, CYP2C67, FMO3, CYP2C40, CYP2B10	232	108	17854	4.988	0.541241	0.074964	3.6453

UP_SEQ_FEATURE	propeptide:N-terminal propeptide	3	1.136	0.002963	COL3A1, COL1A2, COL1A1	231	6	16021	34.68	0.897325	0.365703	4.42906
UP_SEQ_FEATURE	metal ion-binding site:Iron (heme axial ligand)	7	2.652	0.003029	CYP2D9, PXDN, CYP4A12A, CYP1A1, CYP3A11, CYP2C67, CYP2C40, CYP2B10	231	99	16021	4.904	0.902385	0.321443	4.52513
GOTERM_CC_FAT	GO:0005583~fibrillar collagen	3	1.136	0.003439	COL3A1, COL1A2, COL1A1	165	7	12504	32.48	0.489156	0.091496	4.20838
KEGG_PATHWAY	mmu00982:Drug metabolism	6	2.273	0.003543	CYP2D9, CYP3A11, CYP2C67, FMO3, CYP2C40, ADH7, CYP2B10	80	75	5738	5.738	0.315984	0.172947	3.91107
INTERPRO	IPR017973:Cytochrome P450, C-terminal region	6	2.273	0.003662	CYP2D9, CYP4A12A, CYP1A1, CYP3A11, CYP2C67, CYP2C40, CYP2B10	226	81	17763	5.822	0.833678	0.258419	5.12981
INTERPRO	IPR000742:EGF-like, type 3	9	3.409	0.003923	NRG4, SVEP1, CD93, FBN1, HBEGF, DLL1, NID1, EGFL8,	226	199	17763	3.555	0.853719	0.240127	5.48673
GOTERM_BP_FAT	GO:0060348~bone development	7	2.652	0.004089	CHRDL2, INSIG2, COL1A1, GPNMB, WWTR1, MMP2, AHSG	175	118	13588	4.606	0.98941	0.679206	6.34894
UP_SEQ_FEATURE	propeptide:C-terminal propeptide	3	1.136	0.004109	COL3A1, COL1A2, COL1A1	231	7	16021	29.72	0.957506	0.363135	6.09242
GOTERM_BP_FAT	GO:0001568~blood vessel development	10	3.788	0.004211	EMCN, HEY1, CXCR4, LEPR, COL3A1, HBEGF, COL1A1, CX3CL1, MMP2, COL5A1	175	244	13588	3.182	0.990761	0.608148	6.53318
GOTERM_MF_FAT	GO:0008201~heparin binding	6	2.273	0.004524	SMOC2, HBEGF, PF4, FSTL1, GPNMB, COL5A1	174	83	13288	5.521	0.793597	0.231248	6.00808
UP_SEQ_FEATURE	short sequence motif:Cell attachment site	6	2.273	0.004719	TFRC, COL6A1, NID1, COL1A1, GPNMB, IGFBP2	231	76	16021	5.475	0.973429	0.364594	6.9659
GOTERM_BP_FAT	GO:0001944~vasculature development	10	3.788	0.004943	EMCN, HEY1, CXCR4, LEPR, COL3A1, HBEGF, COL1A1, CX3CL1, MMP2, COL5A1	175	250	13588	3.106	0.995916	0.6002	7.62728
INTERPRO	IPR001128:Cytochrome P450	6	2.273	0.004969	CYP2D9, CYP4A12A, CYP1A1, CYP3A11, CYP2C67, CYP2C40, CYP2B10	226	87	17763	5.421	0.912463	0.26248	6.90072
SP_PIR_KEYWORDS	electron transfer	4	1.515	0.004972	CYP2D9, CYP1A1, CYP3A11, CYP2B10	232	27	17854	11.4	0.748607	0.117963	6.36807
SP_PIR_KEYWORDS	glycoprotein	64	24.24	0.005423	B3GALT6, LEPR, FST, FSTL1, LY9, LRRC15, AMN, MMP2, CD93, CXCR4, DSEL, TREH, SLC43A2, B3GALTL, GZMA, DLL1, SAG, CHRDL2, COL1A2, EGFL8, COL1A1, ADAM12, TREM2, CTSF, EMCN, TNF, ADAMTS15, COL3A1, PF4, SERPINH1, MFRP, AHSG, VCAM1, LY6A, SMOC2, C1QTNF6, NUP210, PTPLA, ADRA2A, COL6A1, GALNT11, GPNMB, LYSMD4, COL4A2, SVEP1, NTF3, IL1RN, FBN1, SLC10A6, NID1, SERPINA1E, SPARC, CD63, LAMP3, LEPRE1, TFRC, HBEGF, GDF15, IGFBP2,	232	3600	17854	1.368	0.778253	0.11796	6.9263
INTERPRO	IPR017972:Cytochrome P450, conserved site	6	2.273	0.005469	CYP2D9, CYP4A12A, CYP1A1, CYP3A11, CYP2C67, CYP2C40, CYP2B10	226	89	17763	5.299	0.931567	0.257691	7.57119
KEGG_PATHWAY	mmu04512:ECM-receptor interaction	6	2.273	0.005473	COL4A2, COL3A1, COL1A2, COL6A1, COL1A1, COL5A1	80	83	5738	5.185	0.444129	0.177774	5.98246

GOTERM_BP_FAT	GO:0033554~cellular response to stress	13	4.924	0.005847	ING4, PXDN, HMGB2, APEX2, TNF, EME1, DCLRE1C, EPC2, CDKN1A, EEF1E1, TDG, TRPV4, PHLDA3	175	404	13588	2.499	0.998511	0.605426	8.96173
SP_PIR_KEYWORDS	metalloprotein	5	1.894	0.005926	CYP2D9, CYP1A1, CYP3A11, ADH7, CYP2B10	232	56	17854	6.871	0.807249	0.118952	7.54581
INTERPRO	IPR001881:EGF-like calcium-binding	6	2.273	0.006005	SVEP1, CD93, FBN1, DLL1, NID1, EGFL8	226	91	17763	5.182	0.947413	0.255117	8.28309
GOTERM_MF_FAT	GO:0001871~pattern binding	7	2.652	0.006554	SMOC2, FBN1, HBEGF, PF4, FSTL1, GPNMB, COL5A1	174	128	13288	4.176	0.898578	0.278861	8.59432
GOTERM_MF_FAT	GO:0030247~polysaccharide binding	7	2.652	0.006554	SMOC2, FBN1, HBEGF, PF4, FSTL1, GPNMB, COL5A1	174	128	13288	4.176	0.898578	0.278861	8.59432
INTERPRO	IPR003645:Follistatin-like, N-terminal	3	1.136	0.006723	FST, FSTL1, SPARC	226	10	17763	23.58	0.963059	0.259076	9.22904
INTERPRO	IPR000152:EGF-type aspartate/asparagine hydroxylation conserved site	6	2.273	0.006876	SVEP1, CD93, FBN1, DLL1, NID1, EGFL8	226	94	17763	5.017	0.965745	0.245093	9.42998
INTERPRO	IPR018097:EGF-like calcium-binding, conserved site	6	2.273	0.006876	SVEP1, CD93, FBN1, DLL1, NID1, EGFL8	226	94	17763	5.017	0.965745	0.245093	9.42998
SP_PIR_KEYWORDS	cell binding	3	1.136	0.007004	COL4A2, COL6A1, NID1	232	10	17854	23.09	0.857308	0.129838	8.86123
GOTERM_BP_FAT	GO:0030155~regulation of cell adhesion	6	2.273	0.007134	SMOC2, EMCN, PIK3CB, NID1, COL1A1, CYTH3	175	94	13588	4.956	0.999646	0.629702	10.8304
UP_SEQ_FEATURE	disulfide bond	49	18.56	0.008207	MPZL1, PXDN, NRG4, CORIN, LEPR, FST, FSTL1, LY9, MMP2, CD93, CXCR4, MUP11, GZMA, DLL1, SAG, CCR2, CD300LF, EGFL8, TREM2, ADAM12, CTSF, TNF, ADAMTS15, ELN, COL3A1, PF4, CX3CL1, MFRP, AHSG, VCAM1, LY6A, SMOC2, ADRA2A, GALNT11, DEFB1, LYZ2, COL4A2, SVEP1, LYZ1, NTF3, FBN1, IL1RN, NID1, SPARC, TFRC, HBEGF,	231	2379	16021	1.428	0.998202	0.504574	11.8212
SMART	SM00274:FOLN	3	1.136	0.008275	FST, FSTL1, SPARC	130	10	9131	21.07	0.63715	0.286749	9.12392
SP_PIR_KEYWORDS	egf-like domain	9	3.409	0.00845	NRG4, SVEP1, CD93, FBN1, HBEGF, DLL1, NID1, EGFL8,	232	222	17854	3.12	0.904695	0.14505	10.5975
GOTERM_MF_FAT	GO:0030246~carbohydrate binding	11	4.167	0.008612	SMOC2, EMCN, CD93, FBN1, HBEGF, PF4, FSTL1, GPNMB, GALNT11, GPCPD1, COL5A1	174	317	13288	2.65	0.950712	0.313576	11.148
UP_SEQ_FEATURE	domain:EGF-like 5; calcium-binding	4	1.515	0.00891	SVEP1, CD93, FBN1, NID1	231	30	16021	9.247	0.998956	0.496627	12.7693
SMART	SM00179:EGF_CA	6	2.273	0.009197	SVEP1, CD93, FBN1, DLL1, NID1, EGFL8	130	91	9131	4.631	0.676086	0.24559	10.0923
SP_PIR_KEYWORDS	basement membrane	4	1.515	0.00952	SMOC2, COL4A2, NID1, SPARC	232	34	17854	9.054	0.929329	0.152621	11.8625
SP_PIR_KEYWORDS	heme	7	2.652	0.010478	CYP2D9, PXDN, CYP4A12A, CYP1A1, CYP3A11, CYP2C67, CYP2C40, CYP2B10	232	142	17854	3.794	0.945942	0.157709	12.9809
COG_ONTOLOGY	Secondary metabolites biosynthesis, transport, and catabolism	6	2.273	0.01061	CYP2D9, CYP4A12A, CYP1A1, CYP3A11, CYP2C67, CYP2C40, CYP2B10	27	109	2040	4.159	0.129479	0.129479	6.81827

UP_SEQ_FEATURE	domain:EGF-like 4; calcium-binding	4	1.515	0.010659	SVEP1, CD93, FBN1, DLL1	231	32	16021	8.669	0.999731	0.526307	15.0902
GOTERM_MF_FAT	GO:0020037~heme binding	7	2.652	0.011374	CYP2D9, PXDN, CYP4A12A, CYP1A1, CYP3A11, CYP2C67, CYP2C40, CYP2B10	174	144	13288	3.712	0.981331	0.357456	14.4714
INTERPRO	IPR013091:EGF calcium-binding	5	1.894	0.011404	SVEP1, CD93, FBN1, NID1, EGFL8	226	69	17763	5.695	0.996334	0.350427	15.1812
GOTERM_BP_FAT	GO:0001503~ossification	6	2.273	0.011661	CHRDL2, COL1A1, GPNMB, WWTR1, MMP2, AHSG	175	106	13588	4.395	0.999998	0.764635	17.1206
GOTERM_BP_FAT	GO:0006974~response to DNA damage stimulus	10	3.788	0.011679	DCLRE1C, ING4, CDKN1A, EPC2, HMGB2, APEX2, EEF1E1, EME1, TDG, PHLDA3	175	287	13588	2.705	0.999998	0.728551	17.1449
INTERPRO	IPR002401:Cytochrome P450, E-class, group I	5	1.894	0.011978	CYP2D9, CYP4A12A, CYP1A1, CYP2C67, CYP2C40, CYP2B10	226	70	17763	5.614	0.99724	0.343548	15.8853
GOTERM_BP_FAT	GO:0043062~extracellular structure organization	7	2.652	0.012309	SMOC2, TNF, ELN, COL3A1, NID1, SERPINH1, COL5A1	175	149	13588	3.648	0.999999	0.713446	17.987
KEGG_PATHWAY	mmu00980:Metabolism of xenobiotics by cytochrome P450	5	1.894	0.012582	CYP1A1, CYP3A11, CYP2C67, CYP2C40, ADH7, CYP2B10	80	66	5738	5.434	0.742016	0.287314	13.2666
GOTERM_BP_FAT	GO:0006260~DNA replication	7	2.652	0.013476	RFC5, FHIT, ING4, RFC4, NASP, POT1A, MCM6	175	152	13588	3.576	1	0.714919	19.524
PIR_SUPERFAMILY	PIRSF000045:cytochrome P450 CYP2D6	4	1.515	0.013672	CYP2D9, CYP1A1, CYP2C67, CYP2C40, CYP2B10	115	36	8136	7.861	0.813522	0.568169	14.6574
GOTERM_MF_FAT	GO:0046906~tetrapyrrole binding	7	2.652	0.014144	CYP2D9, PXDN, CYP4A12A, CYP1A1, CYP3A11, CYP2C67, CYP2C40, CYP2B10	174	151	13288	3.54	0.992969	0.390881	17.689
GOTERM_BP_FAT	GO:0010810~regulation of cell-substrate adhesion	4	1.515	0.014412	SMOC2, PIK3CB, NID1, COL1A1	175	40	13588	7.765	1	0.71048	20.7382
GOTERM_BP_FAT	GO:0051258~protein polymerization	4	1.515	0.014412	TUBA8, TUBB2A, TUBD1, TES	175	40	13588	7.765	1	0.71048	20.7382
SP_PIR_KEYWORDS	chromoprotein	4	1.515	0.014854	CYP2D9, CYP1A1, CYP3A11, CYP2B10	232	40	17854	7.696	0.984165	0.205711	17.9265
INTERPRO	IPR008983:Tumour necrosis factor-like	4	1.515	0.015008	TNF, C1QTNF6, C1QTNF4, MFRP	226	41	17763	7.668	0.999385	0.389184	19.5129
INTERPRO	IPR006210:EGF-like	8	3.03	0.015026	NRG4, SVEP1, CD93, FBN1, HBEGF, DLL1, NID1, EGFL8	226	203	17763	3.097	0.999391	0.370423	19.534
GOTERM_CC_FAT	GO:0005604~basement membrane	5	1.894	0.015412	SMOC2, COL4A2, NID1, SPARC, COL5A1	165	73	12504	5.191	0.951622	0.315173	17.6234
SP_PIR_KEYWORDS	glycosidase	5	1.894	0.015515	LYZ2, LYZ1, TDG, TREH, NEU3	232	74	17854	5.2	0.98685	0.203849	18.65
GOTERM_MF_FAT	GO:0005539~glycosaminoglycan binding	6	2.273	0.01661	SMOC2, HBEGF, PF4, FSTL1, GPNMB, COL5A1	174	114	13288	4.019	0.997059	0.411341	20.4585
INTERPRO	IPR000716:Thyroglobulin type-1	3	1.136	0.017054	SMOC2, NID1, IGFBP2	226	16	17763	14.74	0.999778	0.390305	21.8808
INTERPRO	IPR000886:Endoplasmic reticulum, targeting sequence	4	1.515	0.017058	LEPRE1, ERP29, SERPINH1, B3GALTL	226	43	17763	7.311	0.999778	0.373369	21.8845

INTERPRO	IPR013032:EGF-like region, conserved site	10	3.788	0.017601	NRG4, SVEP1, CD93, FBN1, HBEGF, DLL1, NID1, EGFL8, ADAM12, ZFP61	226	310	17763	2.535	0.999831	0.366837	22.502
UP_SEQ_FEATURE	domain:EGF-like 1	6	2.273	0.018361	SVEP1, CD93, FBN1, DLL1, NID1, EGFL8	231	106	16021	3.926	0.999999	0.694102	24.6399
GOTERM_BP_FAT	GO:0045765~regulation of angiogenesis	4	1.515	0.019763	COL4A2, PF4, CX3CL1, RUNX1	175	45	13588	6.902	1	0.794557	27.3535
SMART	SM00211:TY	3	1.136	0.020881	SMOC2, NID1, IGFBP2	130	16	9131	13.17	0.923802	0.40243	21.5696
INTERPRO	IPR002350:Proteinase inhibitor I1, Kazal	4	1.515	0.022835	SMOC2, FST, FSTL1, SPARC	226	48	17763	6.55	0.999988	0.431514	28.2225
UP_SEQ_FEATURE	glycosylation site:O-linked (GalNAc...)	4	1.515	0.023645	TFRC, HBEGF, PF4, NID1	231	43	16021	6.452	1	0.756304	30.6
SMART	SM00181:EGF	8	3.03	0.024812	NRG4, SVEP1, CD93, FBN1, HBEGF, DLL1, NID1, EGFL8	130	203	9131	2.768	0.953356	0.400026	25.1195
INTERPRO	IPR009428:Beta-catenin-interacting ICAT	2	0.758	0.025174	CTNNBIP1, LZIC	226	2	17763	78.6	0.999996	0.447717	30.6501
GOTERM_BP_FAT	GO:0060346~bone trabecula formation	2	0.758	0.025448	COL1A1, MMP2	175	2	13588	77.65	1	0.851551	33.8131
SP_PIR_KEYWORDS	laminin binding	2	0.758	0.02571	COL4A2, NID1	232	2	17854	76.96	0.999264	0.302839	29.0943
SP_PIR_KEYWORDS	bacteriolytic enzyme	2	0.758	0.02571	LYZ2, LYZ1	232	2	17854	76.96	0.999264	0.302839	29.0943
GOTERM_CC_FAT	GO:0005584~collagen type I	2	0.758	0.026061	COL1A2, COL1A1	165	2	12504	75.78	0.994196	0.43568	28.079
INTERPRO	IPR000217:Tubulin	3	1.136	0.026124	TUBA8, TUBB2A, TUBD1	226	20	17763	11.79	0.999998	0.444777	31.6143
INTERPRO	IPR003008:Tubulin/FtsZ, GTPase domain	3	1.136	0.026124	TUBA8, TUBB2A, TUBD1	226	20	17763	11.79	0.999998	0.444777	31.6143
PIR_SUPERFAMILY	PIRSF038428:DNA (cytosine-5)-methyltransferase 3B, Dnmt3b type	2	0.758	0.027829	DNMT3A, DNMT3B	115	2	8136	70.75	0.96804	0.682652	27.7441
GOTERM_CC_FAT	GO:0005792~microsome	7	2.652	0.028453	CYP2D9, CYP4A12A, CYP1A1, CYP3A11, CYP2C67, FMO3, CYP2C40, CYP2B10	165	176	12504	3.014	0.996407	0.430433	30.2533
UP_SEQ_FEATURE	region of interest:Nonhelical region (N-terminal)	2	0.758	0.028507	COL3A1, COL1A1	231	2	16021	69.35	1	0.794945	35.692
GOTERM_BP_FAT	GO:0030199~collagen fibril organization	3	1.136	0.02919	COL3A1, SERPINH1, COL5A1	175	21	13588	11.09	1	0.871937	37.7673
SMART	SM00280:KAZAL	4	1.515	0.030035	SMOC2, FST, FSTL1, SPARC	130	48	9131	5.853	0.975775	0.412267	29.6093
GOTERM_BP_FAT	GO:0001501~skeletal system development	9	3.409	0.03044	CHRDL2, INSIG2, HOXB8, COL1A1, RUNX1, GPNMB, WWTR1, MMP2, AHSG	175	285	13588	2.452	1	0.867134	39.0371
SP_PIR_KEYWORDS	heparin-binding	4	1.515	0.031184	HBEGF, PF4, FSTL1, COL5A1	232	53	17854	5.808	0.999846	0.341564	34.1771
PIR_SUPERFAMILY	PIRSF002306:tubulin	3	1.136	0.031372	TUBA8, TUBB2A, TUBD1	115	20	8136	10.61	0.979528	0.621741	30.7186
GOTERM_CC_FAT	GO:0042598~vesicular fraction	7	2.652	0.032767	CYP2D9, CYP4A12A, CYP1A1, CYP3A11, CYP2C67, FMO3, CYP2C40, CYP2B10	165	182	12504	2.915	0.998491	0.446004	34.0219
GOTERM_BP_FAT	GO:0019835~cytolysis	3	1.136	0.034588	LYZ2, LYZ1, GZMA	175	23	13588	10.13	1	0.885903	43.0818

GOTERM_BP_FAT	GO:0019221~cytokine-mediated signaling pathway	4	1.515	0.034886	TNF, CCR2, PF4, CX3CL1	175	56	13588	5.546	1	0.874378	43.3622
INTERPRO	IPR017975:Tubulin, conserved site	3	1.136	0.036719	TUBA8, TUBB2A, TUBD1	226	24	17763	9.825	1	0.548585	41.552
INTERPRO	IPR013748:Replication factor C	2	0.758	0.037523	RFC5, RFC4	226	3	17763	52.4	1	0.541248	42.2487
GOTERM_BP_FAT	GO:0043045~DNA methylation during embryonic development	2	0.758	0.037929	DNMT3A, DNMT3B	175	3	13588	51.76	1	0.883052	46.1547
SP_PIR_KEYWORDS	heterotrimer	3	1.136	0.038164	COL4A2, COL6A1, COL1A1	232	24	17854	9.62	0.999979	0.387328	40.1687
GOTERM_MF_FAT	GO:0003886~DNA (cytosine-5-)-methyltransferase activity	2	0.758	0.038554	DNMT3A, DNMT3B	174	3	13288	50.91	0.999999	0.680245	41.5662
GOTERM_MF_FAT	GO:0009008~DNA-methyltransferase activity	2	0.758	0.038554	DNMT3A, DNMT3B	174	3	13288	50.91	0.999999	0.680245	41.5662
GOTERM_CC_FAT	GO:0031982~vesicle	13	4.924	0.040507	TNF, NTF3, NGP, IL1RN, ERP29, DLL1, WIPI1, TFRC, CD93, SLC30A3, DOC2B, GPNMB, TES	165	519	12504	1.898	0.999685	0.489281	40.3173
GOTERM_BP_FAT	GO:0048545~response to steroid hormone stimulus	4	1.515	0.041516	HMGB2, LEPR, COL1A1, TES	175	60	13588	5.176	1	0.893678	49.2804
GOTERM_MF_FAT	GO:0050840~extracellular matrix binding	3	1.136	0.041564	ELN, NID1, SPARC	174	25	13288	9.164	1	0.679037	44.0171
UP_SEQ_FEATURE	zinc finger region:ADD-type	2	0.758	0.042456	DNMT3A, DNMT3B	231	3	16021	46.24	1	0.89121	48.431
UP_SEQ_FEATURE	domain:Follistatin-like	2	0.758	0.042456	FSTL1, SPARC	231	3	16021	46.24	1	0.89121	48.431
UP_SEQ_FEATURE	region of interest:Interaction with the PRC2/EED-EZH2 complex	2	0.758	0.042456	DNMT3A, DNMT3B	231	3	16021	46.24	1	0.89121	48.431
GOTERM_CC_FAT	GO:0019898~extrinsic to membrane	12	4.545	0.046477	CYP2D9, SVEP1, CYP1A1, CYP3A11, CYP2C67, CYP2C40, LANCL1, CYP2B10, CYP4A12A, CERK, NEU3, DOC2B, PHLDA3	165	472	12504	1.927	0.999907	0.510259	44.7909
GOTERM_MF_FAT	GO:0009055~electron carrier activity	7	2.652	0.048687	CYP2D9, CYB5R1, CYP4A12A, CYP1A1, CYP3A11, CYP2C67, CYP2C40, CYP2B10	174	202	13288	2.646	1	0.710814	49.4427
UP_SEQ_FEATURE	domain:EGF-like 7; calcium-binding	3	1.136	0.049583	SVEP1, FBN1, DLL1	231	25	16021	8.323	1	0.912648	53.989
GOTERM_BP_FAT	GO:0042742~defense response to bacterium	5	1.894	0.049962	LYZ2, TNF, LYZ1, NGP, DEFB1	175	108	13588	3.595	1	0.924674	55.9809
GOTERM_BP_FAT	GO:0032835~glomerulus development	2	0.758	0.050252	NID1, WWTR1	175	4	13588	38.82	1	0.916944	56.1956

SP_PIR_KEYWORDS	stress-induced protein	2	0.758	0.050762	HSPB1, SERPINH1	232	4	17854	38.48	0.999999	0.466026	49.7257
SP_PIR_KEYWORDS	heat shock	2	0.758	0.050762	HSPB1, SERPINH1	232	4	17854	38.48	0.999999	0.466026	49.7257
GOTERM_MF_FAT	GO:0005547~phosphatidylinositol-3,4,5-trisphosphate binding	2	0.758	0.051075	CYTH3, PHLDA3	174	4	13288	38.18	1	0.703664	51.149
GOTERM_MF_FAT	GO:0033170~protein-DNA loading ATPase activity	2	0.758	0.051075	RFC5, RFC4	174	4	13288	38.18	1	0.703664	51.149
GOTERM_MF_FAT	GO:0003689~DNA clamp loader activity	2	0.758	0.051075	RFC5, RFC4	174	4	13288	38.18	1	0.703664	51.149
GOTERM_CC_FAT	GO:0005663~DNA replication factor C complex	2	0.758	0.051446	RFC5, RFC4	165	4	12504	37.89	0.999966	0.520811	48.2765
SP_PIR_KEYWORDS	transmembrane protein	10	3.788	0.051576	CYP2D9, VCAM1, CYP4A12A, TFRC, CYP1A1, LEPR, CYP3A11, HBEGF, CYP2B10, CD63	232	370	17854	2.08	1	0.457285	50.2921
INTERPRO	IPR001073:Complement C1q protein	3	1.136	0.051845	C1QTNF6, C1QTNF4, MFRP	226	29	17763	8.131	1	0.647014	53.4314
UP_SEQ_FEATURE	short sequence motif:Prevents secretion from ER	4	1.515	0.052804	LEPRE1, ERP29, SERPINH1, B3GALTL	231	59	16021	4.702	1	0.913499	56.313
KEGG_PATHWAY	mmu04510:Focal adhesion	7	2.652	0.054299	COL4A2, PIK3CB, COL3A1, COL1A2, COL6A1, COL1A1, COL5A1	80	198	5738	2.536	0.997455	0.697216	46.6101
PIR_SUPERFAMILY	PIRSF004274:phage T4 DNA polymerase accessory protein 44	2	0.758	0.05489	RFC5, RFC4	115	4	8136	35.37	0.998979	0.747786	47.7951
SP_PIR_KEYWORDS	iron	9	3.409	0.057866	CYP2D9, TET3, PXDN, CYP4A12A, LEPRE1, CYP1A1, CYP3A11, CYP2C67, CYP2C40, CYP2B10	232	321	17854	2.158	1	0.483386	54.4724
GOTERM_BP_FAT	GO:0009725~response to hormone stimulus	6	2.273	0.060785	HMGB2, LEPR, SERPINA1E, COL1A1, AHSG, TES	175	165	13588	2.823	1	0.944997	63.3584
INTERPRO	IPR001525:C-5 cytosine-specific DNA methylase	2	0.758	0.061756	DNMT3A, DNMT3B	226	5	17763	31.44	1	0.698477	59.952
GOTERM_BP_FAT	GO:0051798~positive regulation of hair follicle development	2	0.758	0.062417	TNF, FST	175	5	13588	31.06	1	0.942822	64.3647
GOTERM_BP_FAT	GO:0006346~methylation-dependent chromatin silencing	2	0.758	0.062417	DNMT3A, DNMT3B	175	5	13588	31.06	1	0.942822	64.3647
GOTERM_BP_FAT	GO:0001957~intramembranous ossification	2	0.758	0.062417	COL1A1, MMP2	175	5	13588	31.06	1	0.942822	64.3647
SMART	SM00110:C1Q	3	1.136	0.062757	C1QTNF6, C1QTNF4, MFRP	130	29	9131	7.266	0.999632	0.627825	52.5849
SP_PIR_KEYWORDS	trimer	2	0.758	0.063046	COL3A1, COL1A2	232	5	17854	30.78	1	0.50032	57.6677

SP_PIR_KEYWORDS	polysaccharide degradation	2	0.758	0.063046	LYZ2, LYZ1	232	5	17854	30.78	1	0.50032	57.6677
INTERPRO	IPR018111:K Homology, type 1, subgroup	3	1.136	0.06526	EIF4EBP3, KRR1, IGF2BP3	226	33	17763	7.145	1	0.705438	62.0463
GOTERM_BP_FAT	GO:0006281~DNA repair	7	2.652	0.066	DCLRE1C, EPC2, HMGB2, APEX2, EEF1E1, EME1, TDG	175	222	13588	2.448	1	0.945795	66.4834
SP_PIR_KEYWORDS	oxidoreductase	13	4.924	0.068997	CYP2D9, PXDN, CYB5R1, CYP1A1, CYP3A11, HSD17B13, CYP2C67, CYP2C40, ADH7, CYP2B10, TET3, CYP4A12A, LEPRE1, FMO3	232	572	17854	1.749	1	0.519757	61.0827
GOTERM_MF_FAT	GO:0005198~structural molecule activity	11	4.167	0.069782	COL4A2, TUBA8, TUBB2A, KRT79, FBN1, ELN, COL3A1, COL1A2, TUBD1, COL1A1, COL5A1	174	450	13288	1.867	1	0.792643	62.786
GOTERM_CC_FAT	GO:0031410~cytoplasmic vesicle	12	4.545	0.070813	TNF, TFRC, NGP, NTF3, CD93, ERP29, DLL1, SLC30A3, GPNMB, DOC2B, WIPI1, TES	165	508	12504	1.79	0.999999	0.615105	60.018
SP_PIR_KEYWORDS	sulfation	3	1.136	0.071361	CXCR4, NID1, COL5A1	232	34	17854	6.79	1	0.519255	62.367
INTERPRO	IPR002482:Peptidoglycan-binding Lysin subgroup	2	0.758	0.073644	LYSMD1, LYSMD4	226	6	17763	26.2	1	0.737096	66.651
INTERPRO	IPR018117:C-5 cytosine-specific DNA methylase, active site	2	0.758	0.073644	DNMT3A, DNMT3B	226	6	17763	26.2	1	0.737096	66.651
INTERPRO	IPR018392:Peptidoglycan-binding lysin domain	2	0.758	0.073644	LYSMD1, LYSMD4	226	6	17763	26.2	1	0.737096	66.651
SP_PIR_KEYWORDS	Cyclosporin	2	0.758	0.075172	PPID, PPIC	232	6	17854	25.65	1	0.525949	64.3554
GOTERM_BP_FAT	GO:0008544~epidermis development	5	1.894	0.076764	FOXQ1, NTF3, FST, COL1A1, COL5A1	175	125	13588	3.106	1	0.962506	72.1605
GOTERM_MF_FAT	GO:0005506~iron ion binding	9	3.409	0.079764	CYP2D9, TET3, PXDN, CYP4A12A, LEPRE1, CYP1A1, CYP3A11, CYP2C67, CYP2C40, CYP2B10	174	343	13288	2.004	1	0.817612	67.8873
PIR_SUPERFAMILY	PIRSF001064:lysozyme c	2	0.758	0.081204	LYZ2, LYZ1	115	6	8136	23.58	0.999967	0.821303	62.2849
SMART	SM00257:LysM	2	0.758	0.08185	LYSMD1, LYSMD4	130	6	9131	23.41	0.99997	0.68575	62.5888
SP_PIR_KEYWORDS	growth factor	5	1.894	0.082911	NRG4, NTF3, HBEGF, FGF21, GDF15	232	127	17854	3.03	1	0.550288	68.0978
UP_SEQ_FEATURE	repeat:LysM	2	0.758	0.083117	LYSMD1, LYSMD4	231	6	16021	23.12	1	0.975216	73.4097
KEGG_PATHWAY	mmu03030:DNA replication	3	1.136	0.083192	RFC5, RFC4, MCM6	80	35	5738	6.148	0.999908	0.787529	62.3307
INTERPRO	IPR004129:Glycerophosphoryl diester phosphodiesterase	2	0.758	0.085382	GPCPD1, GDPD1	226	7	17763	22.46	1	0.777965	72.2297
GOTERM_MF_FAT	GO:0003924~GTPase activity	5	1.894	0.086014	TUBA8, TUBB2A, TUBD1, AGAP2, ARL4A	174	128	13288	2.983	1	0.824275	70.7426
GOTERM_BP_FAT	GO:0051797~regulation of hair follicle development	2	0.758	0.086285	TNF, FST	175	7	13588	22.18	1	0.972047	76.4177
GOTERM_BP_FAT	GO:0045684~positive regulation of epidermis development	2	0.758	0.086285	TNF, FST	175	7	13588	22.18	1	0.972047	76.4177

GOTERM_BP_FAT	GO:0042634~regulation of hair cycle	2	0.758	0.086285	TNF, FST	175	7	13588	22.18	1	0.972047	76.4177
SP_PIR_KEYWORDS	phosphoprotein	93	35.23	0.087076	CYP2D9, FHIT, PXDN, MPZL1, 5730508B09RIK, TUBB2A, LEPR, MED23, GPCPD1, STK30, MMP2, EIF4EBP3, FAM83A, HEY1, CD93, CXCR4, TRPV4, FAM181B, AGAP2, FAM83G, SLC43A2, CCDC28B, KRR1, PURG, CYP1A1, ERP29, CCDC138, PIM2, CYP2B10, CDKL5, MCM6, DCLRE1C, TOMM20, HSPB1, RIN1, CD300LF, NEU3, ADAM12, THEM4, GADD45B, CTSF, ING4, HMGB2, TNF, DBNDD2, HSD17B13, SNX8, COL3A1, 2810021B07RIK, ANLN, IGF2BP3, 5830433M19RIK, TAGLN3, AHSG, LY6A, VCAM1, NCAPH, NPAS2, HSPA2, C1QTNF4, NUP210, FMO3, NUDT21, ADRA2A, SLC30A3, EIF3J, UCK2, GPNMB, RUNX1, LYSMD1, TES, AATK, DNMT3A, NASP, FBN1, EME1, TAF7, PHF10, SPARC, WWTR1, TAB3, E130012A19RIK, TUBA8,	232	6311	17854	1.134	1	0.556937	69.9583
INTERPRO	IPR004087:K Homology	3	1.136	0.087237	EIF4EBP3, KRR1, IGF2BP3	226	39	17763	6.046	1	0.774141	73.0272
INTERPRO	IPR001818:Peptidase M10A and M12B, matrixin and adamalysin	3	1.136	0.087237	ADAMTS15, ADAM12, MMP2	226	39	17763	6.046	1	0.774141	73.0272
GOTERM_BP_FAT	GO:0009719~response to endogenous stimulus	6	2.273	0.087798	HMGB2, LEPR, SERPINA1E, COL1A1, AHSG, TES	175	184	13588	2.532	1	0.970321	77.035
UP_SEQ_FEATURE	domain:EGF-like 3; calcium-binding	3	1.136	0.089675	SVEP1, CD93, NID1	231	35	16021	5.945	1	0.977467	76.1697
SP_PIR_KEYWORDS	notch signaling pathway	3	1.136	0.090437	HEY1, APH1C, DLL1	232	39	17854	5.92	1	0.559804	71.3861
GOTERM_BP_FAT	GO:0007398~ectoderm development	5	1.894	0.091435	FOXQ1, NTF3, FST, COL1A1, COL5A1	175	133	13588	2.919	1	0.971215	78.458
GOTERM_BP_FAT	GO:0001525~angiogenesis	5	1.894	0.091435	EMCN, CXCR4, LEPR, HBEGF, CX3CL1	175	133	13588	2.919	1	0.971215	78.458
UP_SEQ_FEATURE	domain:GDPD	2	0.758	0.096284	GPCPD1, GDPD1	231	7	16021	19.82	1	0.979402	78.6783
UP_SEQ_FEATURE	glycosylation site:O-linked (Gal...)	2	0.758	0.096284	COL3A1, COL1A1	231	7	16021	19.82	1	0.979402	78.6783
INTERPRO	IPR017878:TGF-beta binding	2	0.758	0.096972	FBN1, FST	226	8	17763	19.65	1	0.799911	76.8754
INTERPRO	IPR000974:Glycoside hydrolase, family 22, lysozyme	2	0.758	0.096972	LYZ2, LYZ1	226	8	17763	19.65	1	0.799911	76.8754

INTERPRO	IPR012461:Protein of unknown function DUF1669	2	0.758	0.096972	FAM83A, FAM83G	226	8	17763	19.65	1	0.799911	76.8754
SP_PIR_KEYWORDS	dna replication	4	1.515	0.097909	RFC5, RFC4, NASP, MCM6	232	85	17854	3.622	1	0.578909	74.338
INTERPRO	IPR006025:Peptidase M, neutral zinc metallopeptidases, zinc-binding site	4	1.515	0.098377	AMZ1, ADAMTS15, ADAM12, MMP2	226	87	17763	3.614	1	0.794541	77.3865
BIOCARTA	m_eea1Pathway:The role of FYVE-finger proteins in vesicle transport	2	0.758	0.098401	TFRC, 4921523A10RIK	21	6	1171	18.59	0.996278	0.996278	63.7516
UP_SEQ_FEATURE	domain:EGF-like 6	3	1.136	0.098555	FBN1, DLL1, NID1	231	37	16021	5.623	1	0.977395	79.4817
SP_PIR_KEYWORDS	sulfoprotein	2	0.758	0.098957	COL3A1, NID1	232	8	17854	19.24	1	0.572132	74.7289
SP_PIR_KEYWORDS	blocked carboxyl end	2	0.758	0.098957	LY6A, VCAM1	232	8	17854	19.24	1	0.572132	74.7289
GOTERM_MF_FAT	GO:0008889~glycerophosphodiester phosphodiesterase activity	2	0.758	0.099555	GPCPD1, GDPD1	174	8	13288	19.09	1	0.853496	76.141

Supplementary Table 5.

© 2000-2015 QIAGEN. All rights reserved.

**Ingenuity Canonical Pathways**	**-log(p-valu**	**Ratio**	**Overlap**	**Molecules**
Atherosclerosis Signaling	5.98E00	8.06E-02	10/124	COL1A2,COL1A1,LYZ,VCAM1,CXCR4,IL1RN,SERPINA1,CCR2,TNF,COL3A1
Estrogen Biosynthesis	4.32E00	1.35E-01	5/37	CYP1A1,HSD17B13,Cyp2c40 (includes others),CYP3A5,CYP2B6
Nicotine Degradation II	4.2E00	9.52E-02	6/63	FMO3,ADH7,CYP1A1,Cyp2c40 (includes others),CYP3A5,CYP2B6
Hepatic Fibrosis / Hepatic Stellate Cell Activation	4.19E00	5.05E-02	10/198	COL5A1,COL1A2,COL1A1,VCAM1,COL6A1,LEPR,MMP2,COL4A2,TNF,COL3A1
Bupropion Degradation	3.84E00	1.6E-01	4/25	CYP1A1,Cyp2c40 (includes others),CYP3A5,CYP2B6
Acetone Degradation I (to Methylglyoxal)	3.77E00	1.54E-01	4/26	CYP1A1,Cyp2c40 (includes others),CYP3A5,CYP2B6
Nicotine Degradation III	3.53E00	9.26E-02	5/54	ADH7,CYP1A1,Cyp2c40 (includes others),CYP3A5,CYP2B6
Melatonin Degradation I	3.42E00	8.77E-02	5/57	CYP1A1,Cyp2c40 (includes others),CYP3A5,CYP2B6,Sult1d1
Superpathway of Melatonin Degradation	3.25E00	8.06E-02	5/62	CYP1A1,Cyp2c40 (includes others),CYP3A5,CYP2B6,Sult1d1
Granulocyte Adhesion and Diapedesis	3.14E00	4.52E-02	8/177	VCAM1,CXCR4,IL1RN,PF4,MMP2,CX3CL1,TNF,HSPB1
Dendritic Cell Maturation	3.11E00	4.47E-02	8/179	COL1A2,COL1A1,IL1RN,LEPR,TREM2,PIK3CB,TNF,COL3A1
Granzyme A Signaling	2.9E00	1.5E-01	3/20	GZMA,H1FX,HMGB2
Role of BRCA1 in DNA Damage Response	2.79E00	6.41E-02	5/78	RFC4,CDKN1A,PLK1,RFC5,PHF10
Hereditary Breast Cancer Signaling	2.54E00	4.65E-02	6/129	GADD45B,RFC4,CDKN1A,PIK3CB,RFC5,PHF10
Role of CHK Proteins in Cell Cycle Checkpoint	2.52E00	7.27E-02	4/55	RFC4,CDKN1A,PLK1,RFC5
Airway Pathology in Chronic Obstructive Pulmonary	2.5E00	2.5E-01	2/8	MMP2,TNF
Intrinsic Prothrombin Activation Pathway	2.42E00	1.03E-01	3/29	COL1A2,COL1A1,COL3A1
Agranulocyte Adhesion and Diapedesis	2.33E00	3.7E-02	7/189	VCAM1,CXCR4,IL1RN,PF4,MMP2,CX3CL1,TNF
IL-6 Signaling	2.06E00	4.31E-02	5/116	COL1A1,IL1RN,PIK3CB,TNF,HSPB1
LPS/IL-1 Mediated Inhibition of RXR Function	1.99E00	3.2E-02	7/219	FMO3,IL1RN,CYP3A5,FABP7,CYP2B6,Sult1d1,TNF
LXR/RXR Activation	1.98E00	4.13E-02		LYZ,IL1RN,AHSG,SERPINA1,TNF
Glucocorticoid Receptor Signaling	1.97E00	2.91E-02		VCAM1,IL1RN,CDKN1A,PIK3CB,TAF7,TNF,HSPA2,PHF10
Mismatch Repair in	1.89E00	1.25E-01		RFC4,RFC5
MSP-RON Signaling	1.86E00	6.52E-02		PIK3CB,CCR2,TNF
Bladder Cancer Signaling	1.82E00	4.6E-02		FGF21,CDKN1A,MMP2,RASSF1
GADD45 Signaling	1.75E00	1.05E-01		GADD45B,CDKN1A
DNA Methylation and Transcriptional Repression Signaling	1.71E00	1E-01		DNMT3B,DNMT3A
Taurine Biosynthesis	1.66E00	5E-01		CSAD
Dermatan Sulfate	1.62E00	5.26E-02		B3GALT6,DSEL,Sult1d1
Xenobiotic Metabolism Signaling	1.54E00	2.58E-02		FMO3,CYP1A1,CYP3A5,PIK3CB,CYP2B6,Sult1d1,TNF
Trehalose Degradation II (Trehalase)	1.49E00	3.33E-01		TREH
L-serine Degradation	1.49E00	3.33E-01		SDSL
Non-Small Cell Lung Cancer Signaling	1.47E00	4.62E-02		FHIT,PIK3CB,RASSF1
IL-15 Signaling	1.45E00	4.55E-02		VCAM1,PIK3CB,TNF
PXR/RXR Activation	1.44E00	4.48E-02		CYP3A5,CYP2B6,TNF
Acute Phase Response Signaling	1.42E00	2.96E-02		IL1RN,AHSG,SERPINA1,PIK3CB,TNF
14-3-3-mediated Signaling	1.41E00	3.42E-02		TUBA8,TUBB2A,PIK3CB,TNF
Airway Inflammation in	1.37E00	2.5E-01		TNF
PI3K/AKT Signaling	1.34E00	3.25E-02		GDF15,CDKN1A,PIK3CB,THEM4

HER-2 Signaling in Breast Cancer	1.3E00	3.95E-02		CDKN1A,MMP2,PIK3CB
FXR/RXR Activation	1.3E00	3.15E-02		IL1RN,AHSG,SERPINA1,TNF
Acute Myeloid Leukemia Signaling	1.29E00	3.9E-02		RUNX1,PIK3CB,PIM2
Clathrin-mediated Endocytosis Signaling	1.28E00	2.7E-02		FGF21,LYZ,TFRC,SERPINA1,PIK3CB
IL-9 Signaling	1.28E00	5.88E-02		PIK3CB,TNF
CMP-N-acetylneuraminate Biosynthesis I (Eukaryotes)	1.27E00	2E-01		NANP
Ceramide Signaling	1.25E00	3.75E-02		PIK3CB,CERK,TNF
IL-12 Signaling and Production in Macrophages	1.22E00	2.96E-02		LYZ,SERPINA1,PIK3CB,TNF
UDP-N-acetyl-D-glucosamine Biosynthesis II	1.2E00	1.67E-01		FMO3
Notch Signaling	1.19E00	5.26E-02		DLL1,HEY1
Aryl Hydrocarbon Receptor Signaling	1.18E00	2.86E-02		CYP1A1,CDKN1A,TNF,HSPB1
Inhibition of Matrix Metalloproteases	1.17E00	5.13E-02		ADAM12,MMP2
ErbB Signaling	1.17E00	3.49E-02		NRG4,HBEGF,PIK3CB
Glycoaminoglycan-protein Linkage Region	1.13E00	1.43E-01		B3GALT6
Melanoma Signaling	1.12E00	4.76E-02		CDKN1A,PIK3CB
Death Receptor Signaling	1.1E00	3.26E-02		PARP16,TNF,HSPB1
Dermatan Sulfate Biosynthesis (Late Stages)	1.08E00	4.55E-02		DSEL,Sult1d1
Role of Hypercytokinemia/hyperchemokinemia in the Pathogenesis of Influenza	1.07E00	4.44E-02		IL1RN,TNF
p53 Signaling	1.04E00	3.06E-02		GADD45B,CDKN1A,PIK3CB
Telomerase Signaling	1.03E00	3.03E-02		CDKN1A,PIK3CB,POT1
Graft-versus-Host Disease Signaling	1.02E00	4.17E-02		IL1RN,TNF
Germ Cell-Sertoli Cell Junction Signaling	1.01E00	2.5E-02		TUBA8,TUBB2A,PIK3CB,TNF
Cell Cycle: G2/M DNA Damage Checkpoint	1E00	4.08E-02		CDKN1A,PLK1
Glycine Betaine	9.85E-01	1E-01		SDSL
Pancreatic Adenocarcinoma Signaling	9.63E-01	2.83E-02		CDKN1A,HBEGF,PIK3CB
Lymphotoxin β Receptor Signaling	9.33E-01	3.7E-02		VCAM1,PIK3CB
Chondroitin Sulfate Biosynthesis	9.33E-01	3.7E-02		B3GALT6,Sult1d1
NF-κB Signaling	9.2E-01	2.31E-02		IL1RN,PIK3CB,TNF,TAB3
Cleavage and Polyadenylation of Pre-	9.1E-01	8.33E-02		NUDT21
Acyl-CoA Hydrolysis	9.1E-01	8.33E-02		THEM4
Role of Cytokines in Mediating Communication between Immune Cells	9.07E-01	3.57E-02		IL1RN,TNF
Glioma Invasiveness	8.94E-01	3.51E-02		MMP2,PIK3CB
ErbB2-ErbB3 Signaling	8.94E-01	3.51E-02		NRG4,PIK3CB
Heparan Sulfate	8.94E-01	3.51E-02		B3GALT6,Sult1d1
Production of Nitric Oxide and Reactive Oxygen Species in Macrophages	8.75E-01	2.22E-02		LYZ,SERPINA1,PIK3CB,TNF
ATM Signaling	8.7E-01	3.39E-02		GADD45B,CDKN1A
p38 MAPK Signaling	8.69E-01	2.56E-02		IL1RN,TNF,HSPB1
PTEN Signaling	8.61E-01	2.54E-02		CDKN1A,PREX2,PIK3CB
Induction of Apoptosis by	8.58E-01	3.33E-02		CXCR4,TNF
ErbB4 Signaling	8.58E-01	3.33E-02		NRG4,PIK3CB
IL-8 Signaling	8.5E-01	2.17E-02		VCAM1,HBEGF,MMP2,PIK3CB

DNA Double-Strand Break Repair by Non-Homologous End Joining	8.48E-01	7.14E-02		DCLRE1C
HMGB1 Signaling	8.46E-01	2.5E-02		VCAM1,PIK3CB,TNF
Estrogen-Dependent Breast Cancer Signaling	8.35E-01	3.23E-02		HSD17B13,PIK3CB
GM-CSF Signaling	8.35E-01	3.23E-02		RUNX1,PIK3CB
Antiproliferative Role of Somatostatin Receptor 2	8.24E-01	3.17E-02		CDKN1A,PIK3CB
Telomere Extension by Telomerase	8.2E-01	6.67E-02		POT1
RAR Activation	8.15E-01	2.11E-02		ADH7,PNRC1,PIK3CB,PHF10
Serotonin Degradation	8.13E-01	3.12E-02		ADH7,Sult1d1
Breast Cancer Regulation by Stathmin1	8.09E-01	2.09E-02		TUBA8,CDKN1A,TUBB2A,PIK3CB
Role of Pattern Recognition Receptors in Recognition of Bacteria and Viruses	7.94E-01	2.36E-02		CASP1,PIK3CB,TNF
Neurotrophin/TRK Signaling	7.82E-01	2.99E-02		NTF3,PIK3CB
IL-10 Signaling	7.72E-01	2.94E-02		IL1RN,TNF
Remodeling of Epithelial Adherens Junctions	7.72E-01	2.94E-02		TUBA8,TUBB2A
Leukocyte Extravasation Signaling	7.7E-01	2.02E-02		VCAM1,CXCR4,MMP2,PIK3CB
Agrin Interactions at Neuromuscular Junction	7.62E-01	2.9E-02		GABPB1,NRG4
Differential Regulation of Cytokine Production in Macrophages and T Helper Cells by IL-17A and IL-17F	7.48E-01	5.56E-02		TNF
Small Cell Lung Cancer Signaling	7.43E-01	2.82E-02		FHIT,PIK3CB
JAK/Stat Signaling	7.34E-01	2.78E-02		CDKN1A,PIK3CB
Leptin Signaling in Obesity	7.16E-01	2.7E-02		LEPR,PIK3CB
Toll-like Receptor Signaling	7.16E-01	2.7E-02		IL1RN,TNF
Axonal Guidance Signaling	7.13E-01	1.62E-02		NTF3,TUBA8,ADAM12,CXCR4,TUBB2A,MMP2,PIK3CB
TREM1 Signaling	7.07E-01	2.67E-02		CASP1,TNF
Epithelial Adherens Junction Signaling	6.73E-01	2.05E-02		DLL1,TUBA8,TUBB2A
Role of Osteoblasts, Osteoclasts and Chondrocytes in	6.65E-01	1.83E-02		COL1A1,IL1RN,PIK3CB,TNF
AMPK Signaling	6.61E-01	2.03E-02		ADRA2A,PIK3CB,PHF10
Systemic Lupus Erythematosus Signaling	6.61E-01	1.82E-02		IL1RN,PIK3CB,TNF,PIM2
Differential Regulation of Cytokine Production in Intestinal Epithelial Cells by IL-17A and IL-17F	6.53E-01	4.35E-02		TNF
Prostate Cancer Signaling	6.49E-01	2.44E-02		CDKN1A,PIK3CB
Aldosterone Signaling in Epithelial Cells	6.39E-01	1.97E-02		PIK3CB,HSPA2,HSPB1
Estrogen-mediated S-phase Entry	6.36E-01	4.17E-02		CDKN1A
Dopamine Degradation	6.36E-01	4.17E-02		Sult1d1
FGF Signaling	6.27E-01	2.35E-02		FGF21,PIK3CB
Gap Junction Signaling	6.23E-01	1.94E-02		TUBA8,TUBB2A,PIK3CB
IL-17A Signaling in Gastric Cells	6.21E-01	4E-02		TNF
HIPPO signaling	6.19E-01	2.33E-02		WWTR1,RASSF1
Neuregulin Signaling	6.05E-01	2.27E-02		NRG4,HBEGF
Altered T Cell and B Cell Signaling in Rheumatoid Arthritis	6.05E-01	2.27E-02		IL1RN,TNF
UVA-Induced MAPK	6.05E-01	2.27E-02		PARP16,PIK3CB
Virus Entry via Endocytic Pathways	5.98E-01	2.25E-02		TFRC,PIK3CB

Crosstalk between Dendritic Cells and Natural Killer	5.98E-01	2.25E-02		TREM2,TNF
PAK Signaling	5.98E-01	2.25E-02		PIK3CB,TNF
Cell Cycle Control of Chromosomal Replication	5.92E-01	3.7E-02		MCM6
Communication between Innate and Adaptive	5.84E-01	2.2E-02		IL1RN,TNF
Chronic Myeloid Leukemia Signaling	5.71E-01	2.15E-02		CDKN1A,PIK3CB
Salvage Pathways of Pyrimidine Ribonucleotides	5.71E-01	2.15E-02		PLK1,UCK2
TNFR2 Signaling	5.66E-01	3.45E-02		TNF
PPAR Signaling	5.65E-01	2.13E-02		IL1RN,TNF
Glioma Signaling	5.58E-01	2.11E-02		CDKN1A,PIK3CB
Role of p14/p19ARF in Tumor Suppression	5.53E-01	3.33E-02		PIK3CB
IGF-1 Signaling	5.46E-01	2.06E-02		PIK3CB,IGFBP2
Amyotrophic Lateral Sclerosis Signaling	5.4E-01	2.04E-02		CASP1,PIK3CB
Cholecystokinin/Gastrin-mediated Signaling	5.22E-01	1.98E-02		IL1RN,TNF
Retinoate Biosynthesis I	5.18E-01	3.03E-02		ADH7
Ethanol Degradation II	5.18E-01	3.03E-02		ADH7
HIF1α Signaling	5.16E-01	1.96E-02		MMP2,PIK3CB
Sertoli Cell-Sertoli Cell Junction Signaling	5.15E-01	1.69E-02		TUBA8,TUBB2A,TNF
HGF Signaling	4.99E-01	1.9E-02		CDKN1A,PIK3CB
Coagulation System	4.97E-01	2.86E-02		SERPINA1
Thyroid Hormone Metabolism II (via Conjugation and/or	4.97E-01	2.86E-02		Sult1d1
Stearate Biosynthesis I (Animals)	4.97E-01	2.86E-02		THEM4
Noradrenaline and Adrenaline Degradation	4.97E-01	2.86E-02		ADH7
Regulation of the Epithelial-Mesenchymal Transition Pathway	4.9E-01	1.63E-02		FGF21,MMP2,PIK3CB
autophagy	4.87E-01	2.78E-02		WIPI1
ERK/MAPK Signaling	4.78E-01	1.6E-02		LAMTOR3,PIK3CB,HSPB1
Fc Epsilon RI Signaling	4.78E-01	1.83E-02		PIK3CB,TNF
Renin-Angiotensin	4.78E-01	1.83E-02		PIK3CB,TNF
Sphingosine-1-phosphate Signaling	4.78E-01	1.83E-02		CASP1,PIK3CB
Role of Tissue Factor in	4.73E-01	1.82E-02		HBEGF,PIK3CB
Docosahexaenoic Acid (DHA) Signaling	4.59E-01	2.56E-02		PIK3CB
Role of PKR in Interferon Induction and Antiviral Response	4.5E-01	2.5E-02		TNF
Thyroid Cancer Signaling	4.5E-01	2.5E-02		NTF3
FcγRIIB Signaling in B Lymphocytes	4.42E-01	2.44E-02		PIK3CB
Type II Diabetes Mellitus Signaling	4.39E-01	1.71E-02		PIK3CB,TNF
phagosome maturation	4.25E-01	1.67E-02		TUBA8,TUBB2A
Chondroitin Sulfate Biosynthesis (Late Stages)	4.03E-01	2.17E-02		Sult1d1
Adipogenesis pathway	3.95E-01	1.57E-02		CTNNBIP1,TNF
Estrogen Receptor	3.95E-01	1.57E-02		MED23,TAF7
Role of Macrophages, Fibroblasts and Endothelial Cells in Rheumatoid	3.9E-01	1.34E-02		VCAM1,IL1RN,PIK3CB,TNF
Ephrin A Signaling	3.89E-01	2.08E-02		PIK3CB
TNFR1 Signaling	3.82E-01	2.04E-02		TNF
Ovarian Cancer Signaling	3.79E-01	1.53E-02		MMP2,PIK3CB

Assembly of RNA Polymerase II Complex	3.75E-01	2E-02		TAF7
Heparan Sulfate Biosynthesis (Late Stages)	3.75E-01	2E-02		Sult1d1
Human Embryonic Stem Cell Pluripotency	3.67E-01	1.49E-02		NTF3,PIK3CB
CNTF Signaling	3.62E-01	1.92E-02		PIK3CB
Endometrial Cancer	3.62E-01	1.92E-02		PIK3CB
Primary Immunodeficiency Signaling	3.62E-01	1.92E-02		DCLRE1C
Cardiac Hypertrophy	3.58E-01	1.35E-02		ADRA2A,PIK3CB,HSPB1
UVB-Induced MAPK	3.56E-01	1.89E-02		PIK3CB
IL-2 Signaling	3.56E-01	1.89E-02		PIK3CB
Phototransduction Pathway	3.56E-01	1.89E-02		SAG
Role of IL-17A in Arthritis	3.5E-01	1.85E-02		PIK3CB
Unfolded protein response	3.5E-01	1.85E-02		HSPA2
Thrombopoietin Signaling	3.44E-01	1.82E-02		PIK3CB
Huntington's Disease	3.39E-01	1.3E-02		CASP1,PIK3CB,HSPA2
EGF Signaling	3.39E-01	1.79E-02		PIK3CB
eNOS Signaling	3.39E-01	1.41E-02		PIK3CB,HSPA2
Myc Mediated Apoptosis Signaling	3.28E-01	1.72E-02		PIK3CB
Regulation of eIF4 and p70S6K Signaling	3.25E-01	1.37E-02		EIF4EBP3,PIK3CB
Glioblastoma Multiforme Signaling	3.25E-01	1.37E-02		CDKN1A,PIK3CB
Colorectal Cancer Metastasis Signaling	3.23E-01	1.27E-02		MMP2,PIK3CB,TNF
CXCR4 Signaling	3.06E-01	1.32E-02		CXCR4,PIK3CB
Role of JAK1 and JAK3 in γc Cytokine Signaling	3.02E-01	1.59E-02		PIK3CB
PCP pathway	3.02E-01	1.59E-02		HSPB1
Activation of IRF by Cytosolic Pattern	2.98E-01	1.56E-02		TNF
Retinoic acid Mediated Apoptosis Signaling	2.98E-01	1.56E-02		PARP16
IL-17A Signaling in Airway	2.98E-01	1.56E-02		PIK3CB
Pyridoxal 5'-phosphate Salvage Pathway	2.98E-01	1.56E-02		PLK1
Cell Cycle: G1/S Checkpoint Regulation	2.98E-01	1.56E-02		CDKN1A
3-phosphoinositide Biosynthesis	2.94E-01	1.28E-02		PIK3CB,STYXL1
CD40 Signaling	2.93E-01	1.54E-02		PIK3CB
Tec Kinase Signaling	2.89E-01	1.27E-02		PIK3CB,TNF
Angiopoietin Signaling	2.88E-01	1.52E-02		PIK3CB
Mitotic Roles of Polo-Like Kinase	2.88E-01	1.52E-02		PLK1
Role of PI3K/AKT Signaling in the Pathogenesis of	2.88E-01	1.52E-02		PIK3CB
Erythropoietin Signaling	2.84E-01	1.49E-02		PIK3CB
GDNF Family Ligand-Receptor Interactions	2.8E-01	1.47E-02		PIK3CB
Macropinocytosis Signaling	2.8E-01	1.47E-02		PIK3CB
Hepatic Cholestasis	2.77E-01	1.23E-02		IL1RN,TNF
G-Protein Coupled Receptor Signaling	2.77E-01	1.17E-02		LAMTOR3,ADRA2A,PIK3CB
Growth Hormone Signaling	2.75E-01	1.45E-02		PIK3CB
Role of MAPK Signaling in the Pathogenesis of	2.75E-01	1.45E-02		TNF
IL-3 Signaling	2.67E-01	1.41E-02		PIK3CB
T Helper Cell Differentiation	2.67E-01	1.41E-02		TNF
Renal Cell Carcinoma	2.67E-01	1.41E-02		PIK3CB
PEDF Signaling	2.67E-01	1.41E-02		PIK3CB
Chemokine Signaling	2.67E-01	1.41E-02		CXCR4
Tight Junction Signaling	2.64E-01	1.2E-02		NUDT21,TNF
IL-17 Signaling	2.63E-01	1.39E-02		PIK3CB

LPS-stimulated MAPK Signaling	2.59E-01	1.37E-02		PIK3CB
NF-κB Activation by Viruses	2.59E-01	1.37E-02		PIK3CB
Prolactin Signaling	2.59E-01	1.37E-02		PIK3CB
Ephrin B Signaling	2.59E-01	1.37E-02		CXCR4
STAT3 Pathway	2.59E-01	1.37E-02		CDKN1A
FLT3 Signaling in Hematopoietic Progenitor	2.55E-01	1.35E-02		PIK3CB
Endothelin-1 Signaling	2.52E-01	1.16E-02		CASP1,PIK3CB
Molecular Mechanisms of Cancer	2.49E-01	1.1E-02		LAMTOR3,CDKN1A,PIK3CB,Aph1c
VEGF Family Ligand-Receptor Interactions	2.48E-01	1.32E-02		PIK3CB
IL-4 Signaling	2.48E-01	1.32E-02		PIK3CB
BMP signaling pathway	2.48E-01	1.32E-02		FST
PDGF Signaling	2.44E-01	1.3E-02		PIK3CB
VDR/RXR Activation	2.41E-01	1.28E-02		CDKN1A
Cyclins and Cell Cycle Regulation	2.41E-01	1.28E-02		CDKN1A
Reelin Signaling in Neurons	2.37E-01	1.27E-02		PIK3CB
PPARα/RXRα Activation	2.35E-01	1.12E-02		MED23,Cyp2c40 (includes others)
NRF2-mediated Oxidative Stress Response	2.33E-01	1.11E-02		ERP29,PIK3CB
Melanocyte Development and Pigmentation Signaling	2.21E-01	1.19E-02		PIK3CB
TR/RXR Activation	2.18E-01	1.18E-02		PIK3CB
Allograft Rejection Signaling	2.14E-01	1.16E-02		TNF
α-Adrenergic Signaling	2.11E-01	1.15E-02		ADRA2A
FAK Signaling	2.11E-01	1.15E-02		PIK3CB
TGF-β Signaling	2.11E-01	1.15E-02		TGIF1
CTLA4 Signaling in Cytotoxic T Lymphocytes	2.08E-01	1.14E-02		PIK3CB
G Beta Gamma Signaling	2.08E-01	1.14E-02		HBEGF
RANK Signaling in	2.08E-01	1.14E-02		PIK3CB
Apoptosis Signaling	2.06E-01	1.12E-02		TNF
